# Supplementary material for: Trialkoxysilane-Induced Iridium-Catalyzed para-Selective C–H Bond Borylation of Arenes
Source: Nat Commun. 2024 Apr 2;15:2847. doi: 10.1038/s41467-024-47205-8 (PMC10987550; doi:10.1038/s41467-024-47205-8)
Supplement: Supplementary file 1 — Supplementary Information [file 41467_2024_47205_MOESM1_ESM.pdf]

Supplementary Information

Trialkoxysilane-Induced Iridium-Catalyzed *para*-Selective C–H Bond Borylation of Arenes

Guodong Ju, [a] Zhibin Huang, [a] Yingsheng Zhao*[a][b]

[a] Key Laboratory of Organic Synthesis of Jiangsu Province, College of Chemistry, Chemical Engineering and Materials Science, Soochow University, Suzhou 215123, P. R. China.

[b] School of Chemistry and Chemical Engineering, Henan Normal University, Xinxiang 453000, P. R. China.

Email: yszhao@suda.edu.cn

Table of Contents

1 General information 2

1.1 Reagents and solvents 2

1.2 Data analysis 2

1.3 Naming of compounds 2

2. Optimization of reaction conditions 3

3. Preparation of starting compounds 18

4. Trialkoxysilane-induced Ir-catalyzed *para*-selective C–H borylation of arenes 51

4.1 *para* C−H borylation of benzaldehyde and acetophenones derivatives 51

4.2 *para* C−H borylation of benzoic acid, benzyl alcohol and phenol derivatives 73

4.3 Substrate scope of *para* C-H borylation of aryl and benzyl silicon compounds 82

5. Switchable site-selective C-H borylation 104

6. Late-stage C−H borylation of important molecules 108

7. Silylarenes as useful building blocks for the construction of 4-acetoxybiphenyl derivatives 119

8. Derivatization of the products from C–H borylation 128

9. Computational method 136

10. NMR Spectra 137

11. Supplementary reference 462

1 General information

1.1 Reagents and solvents

All reagents, unless otherwise stated, were used as supplied by commercial sources without further purification. $[Ir(cod)OMe]_2$, Me_4phen was purchased from Adamas and 4,4,4',4',6,6,6',6'-octamethyl-2,2'-bi(1,3,2-dioxaborinane) (B_2dmg_2) was purchased from Bide. Cyclohexane was purchased from commercial sources and not anhydrous treated. Column chromatography purifications performed using 200–300 and 300-400 mesh silica gel. Thin layer chromatography was performed on 0.2±0.03 mm thick aluminum-backed silica gel plates and visualized with ultraviolet light (λ = 254 nm).

1.2 Data analysis

NMR spectra were recorded on Varian Inova–400 MHz, Inova–300 MHz, Bruker DRX–400 or Bruker DRX–500 instruments and calibrated using residual solvent peaks as internal reference. The boron bearing carbon atom was not observed due to quadrupolar relaxation. All coupling constants are apparent *J* values measured at the indicated field strengths in Hertz (s = singlet, d = doublet, t = triplet, q = quartet, dd = doublet of doublets, bs = broad singlet, dt = doublet of triplet, tt = triplet of triplet, td = triplet of doublet, sept = septet). ^{13}C signals are singlets unless otherwise stated, coupling constants *J* in Hz. HRMS analysis were carried out using a Bruker microTOF–Q instrument or a TOF–MS instrument.

1.3 Naming of compounds

Compound names were generated by ChemDraw 19.0 software (PerkinElmer), following the IUPAC.

2. Optimization of reaction conditions

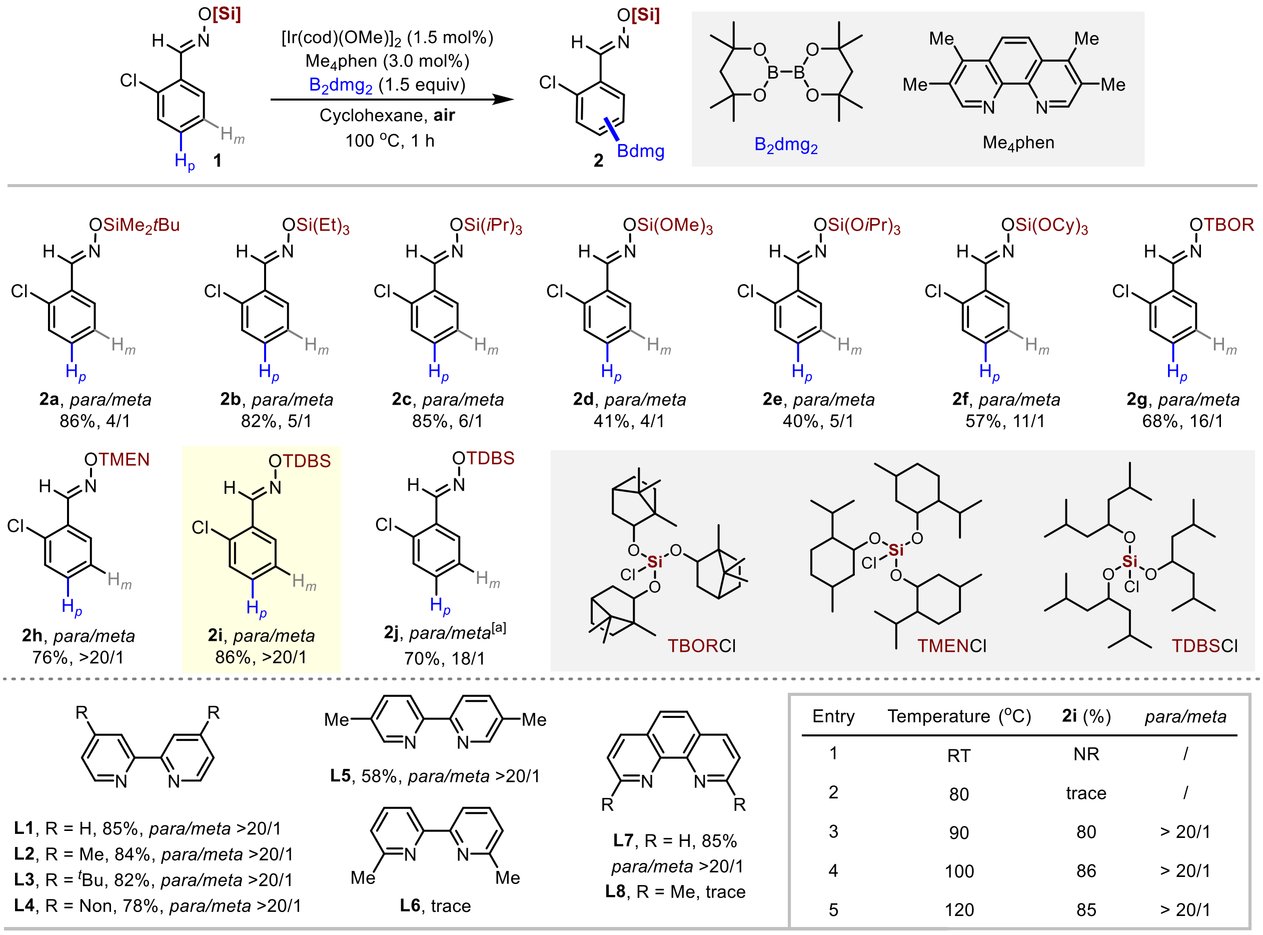

Entry	Temperature (°C)	2i (%)	para/meta
1	RT	NR	/
2	80	trace	/
3	90	80	> 20/1
4	100	86	> 20/1
5	120	85	> 20/1

Supplementary Fig. 1: Effects of various silicon functional groups, ligand and temperature for *para* C-H borylation. Reaction conditions: substrate **1** (0.2 mmol), B_2dmg_2 (1.5 equiv), $[Ir(cod)(OMe)]_2$ (1.5 mol%), Me_4phen (3.0 mol%), cyclohexane (1 mL), 100 °C, 1 h, isolated yield. Ratios of *meta* to *para* were determined from the crude ^{1}H-NMR spectra after borylation. [a] B_2pin_2 used instead of B_2dmg_2. B_2dmg_2 = 4,4,4',4',6,6,6',6'-Octamethyl-2,2'-bi(1,3,2-dioxaborinane). Me_4phen = 3,4,7,8-Tetramethyl-1,10-phenanthroline.

Typical procedure for the borylation of 2-chlorobenzaldehyde O-trialkyl(oxy)ylsilyl oxime:

2-Chlorobenzaldehyde O-trialkyl(oxy)ylsilyl oxime **1** (0.2 mmol, 1.0 equiv), B_2dmg_2 (84.6 mg, 0.3 mmol, 1.5 equiv), $[Ir(cod)OMe]_2$ (2.0 mg, 0.003 mmol), Me_4phen (1.5 mg, 0.006 mmol), and Cyclohexane (1.0 mL) were added to a 15 mL glass vial under air atmosphere. The glass vial was capped with a teflon pressure cap and placed into an aluminum block pre-heated to 100 ºC for 1 h. After completion, cyclohexane was removed under reduced pressure and chromatographic separation with silica gel (20% ethyl acetate in hexane as eluent) gave the borylated product **2** as a colorless liquid. The *para:meta* ratio of products was reported from the analysis of ^{1}H NMR.

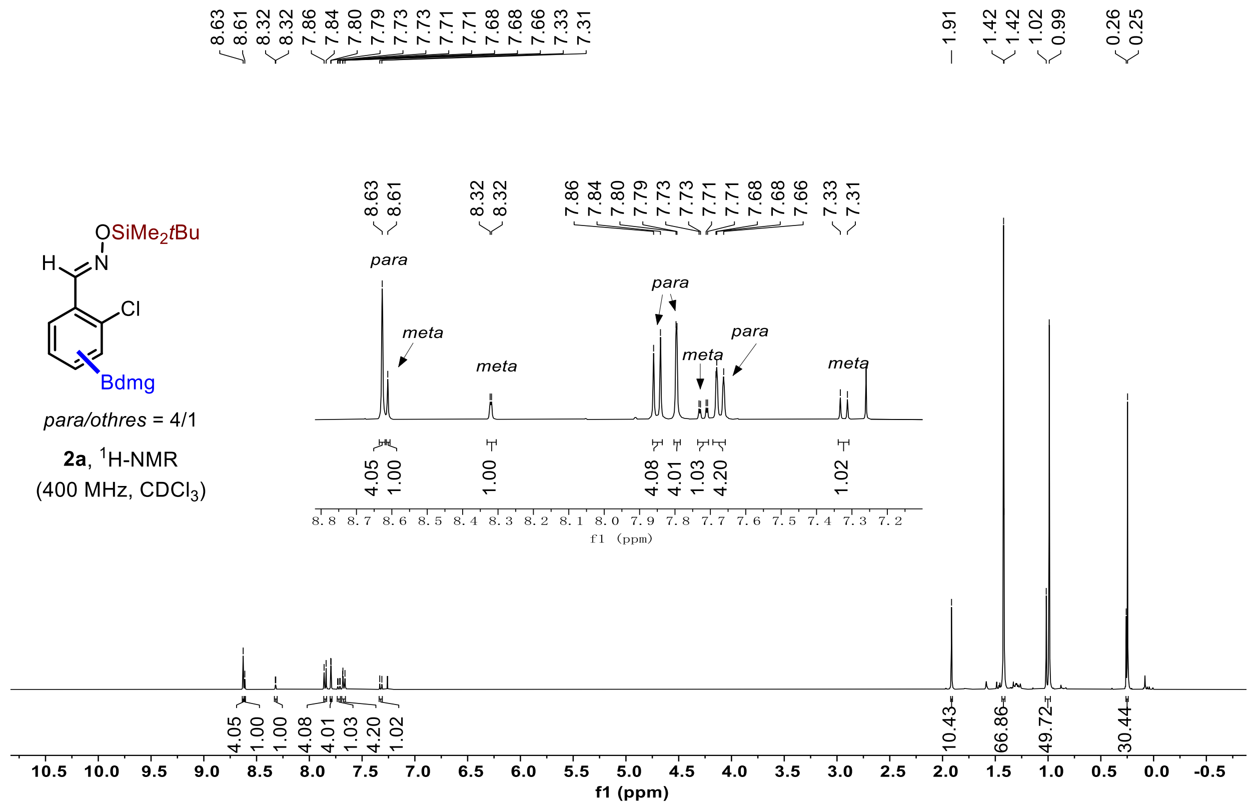

Supplementary Fig. 2: ^{1}**H-NMR** spectra of crude **2a** (25 °C, 400 MHz, $CDCl_3$)

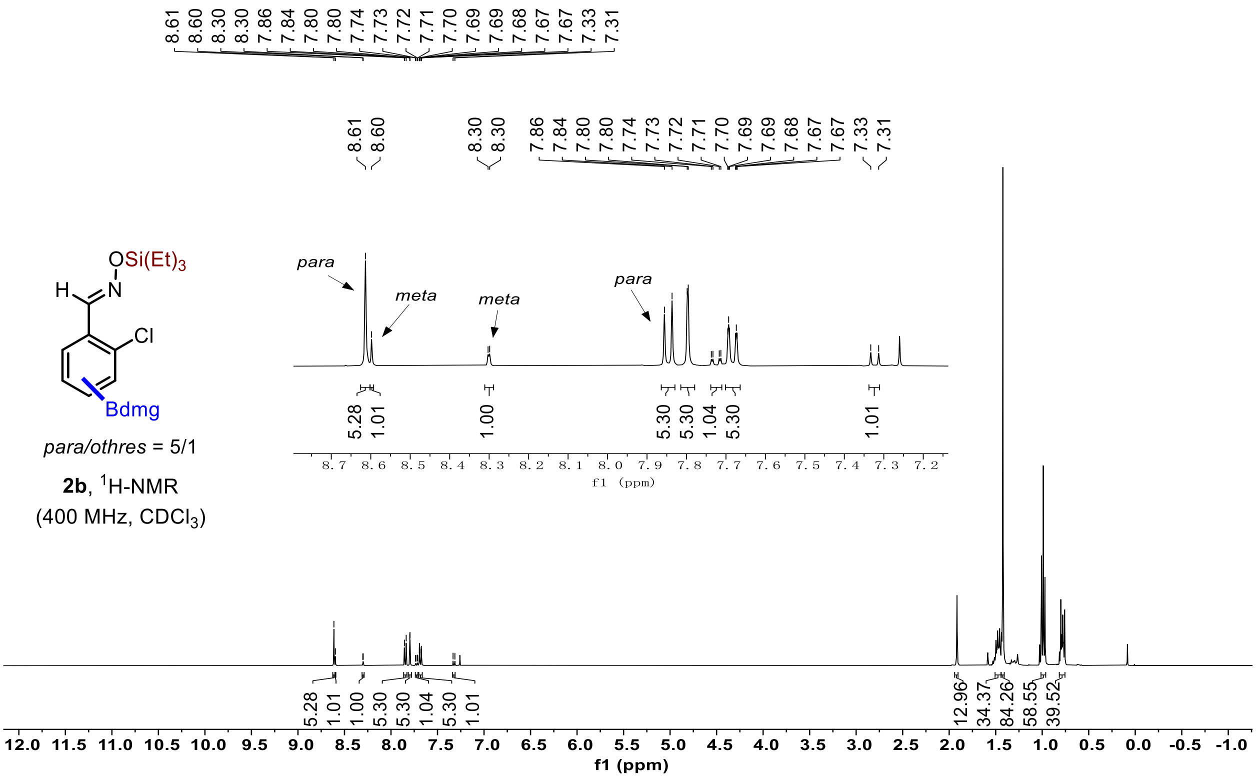

Supplementary Fig. 3: ^{1}**H-NMR** spectra of crude **2b** (25 °C, 400 MHz, $CDCl_3$)

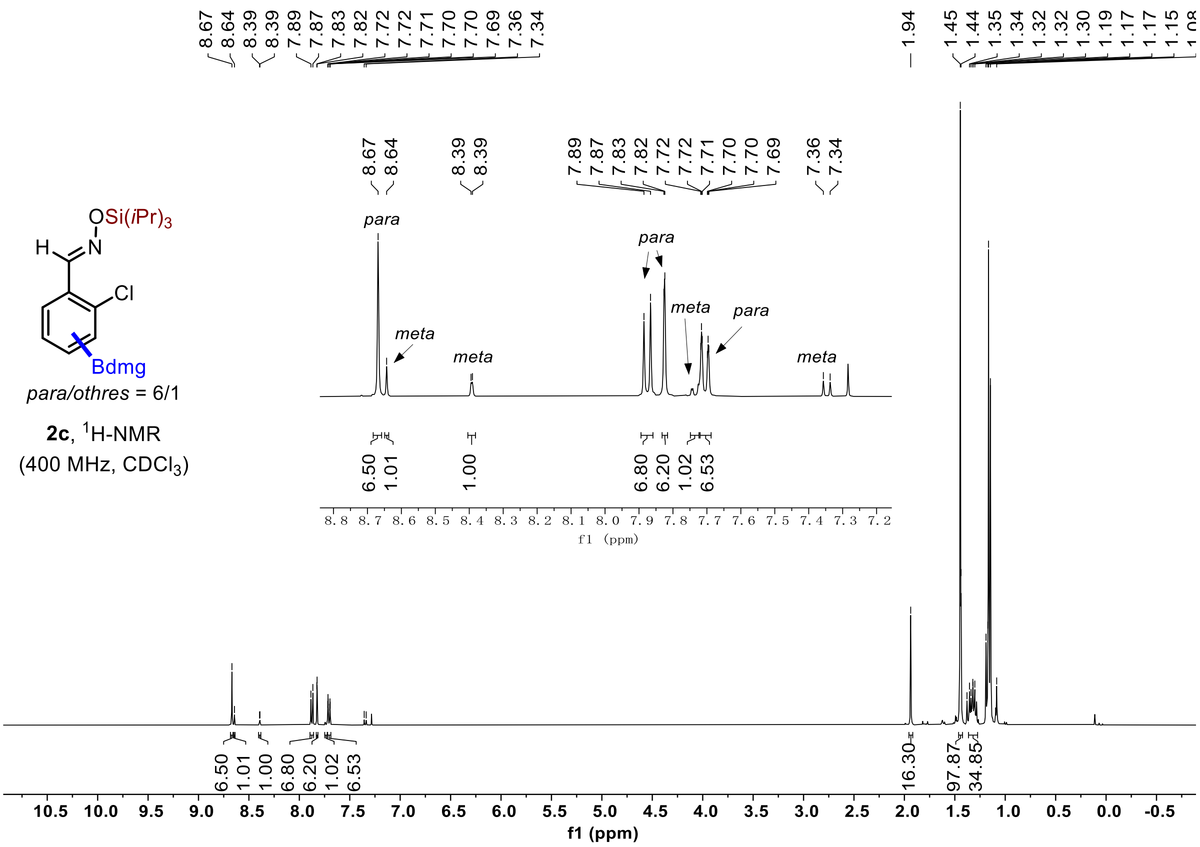

Supplementary Fig. 4: ^{1}H-NMR spectra of crude **2c** (25 °C, 400 MHz, $CDCl_3$)

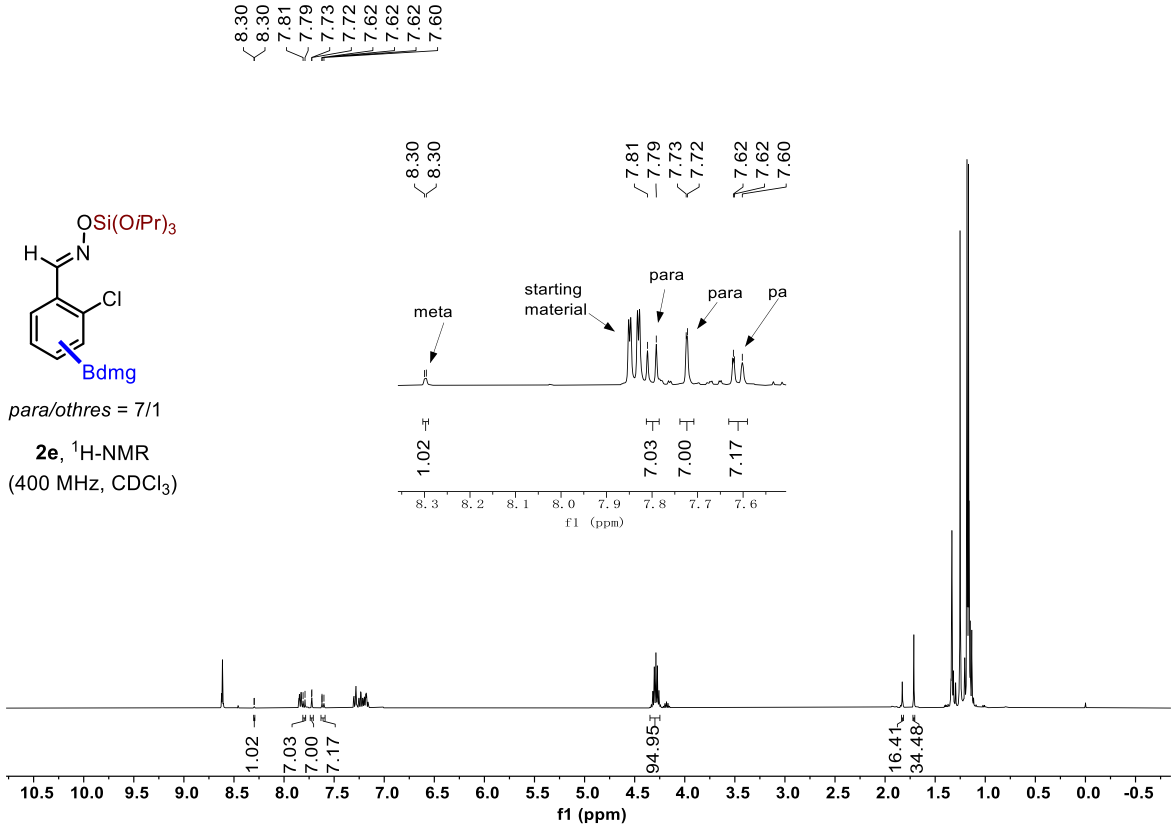

Supplementary Fig. 5: ^{1}H-NMR spectra of crude **2e** (25 °C, 400 MHz, $CDCl_3$)

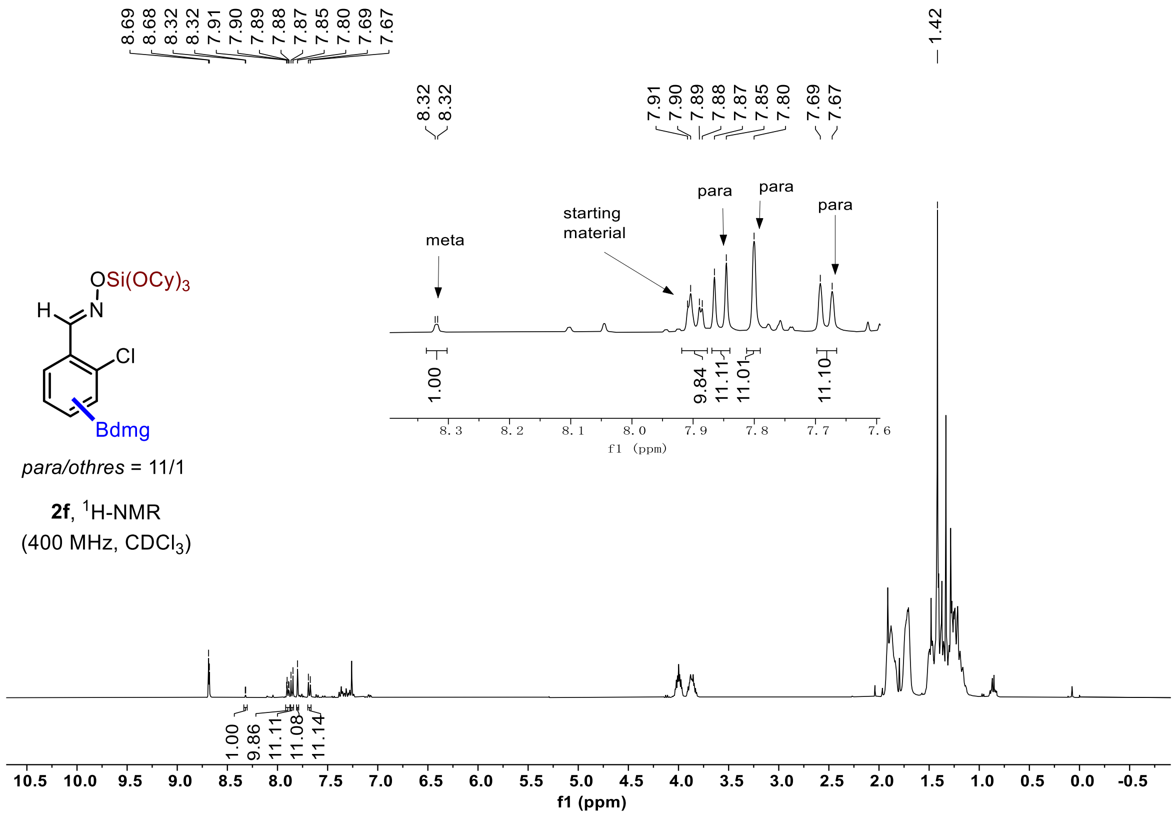

Supplementary Fig. 6: ^{1}H-NMR spectra of crude **2f** (25 °C, 400 MHz, CDCl3)

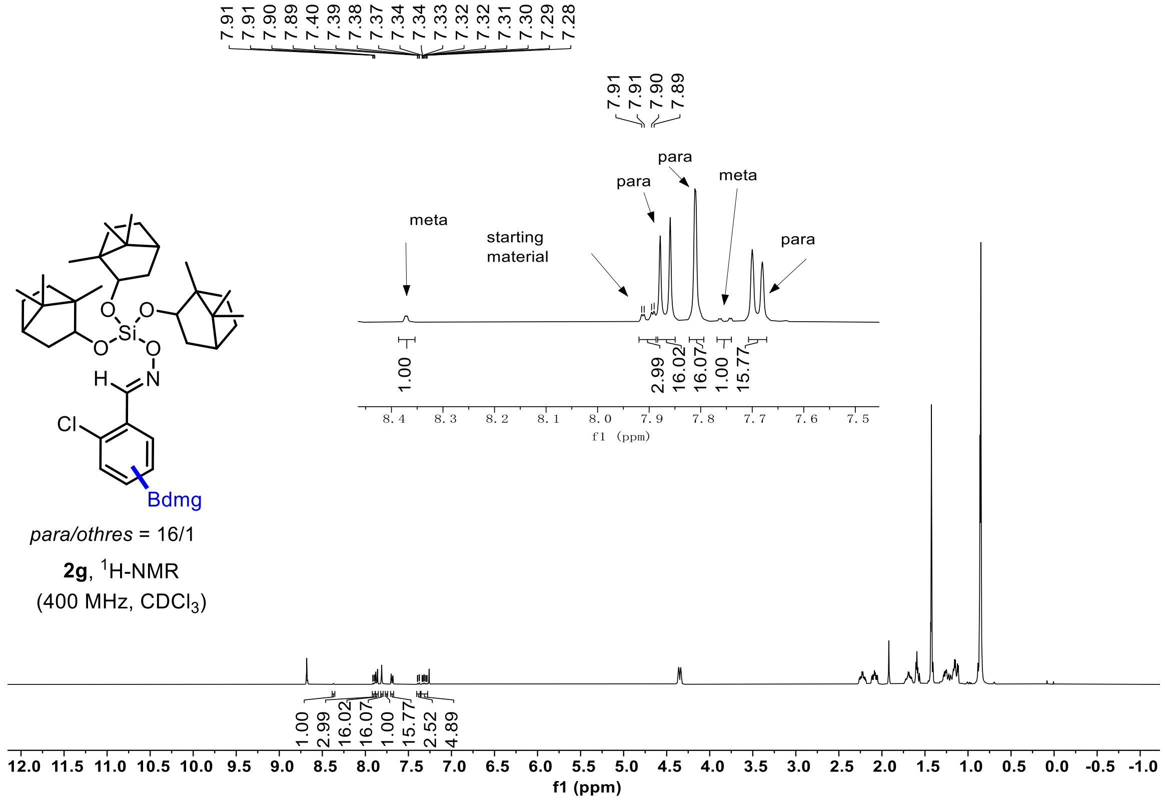

Supplementary Fig. 7: ^{1}H-NMR spectra of crude **2g** (25 °C, 400 MHz, CDCl3)

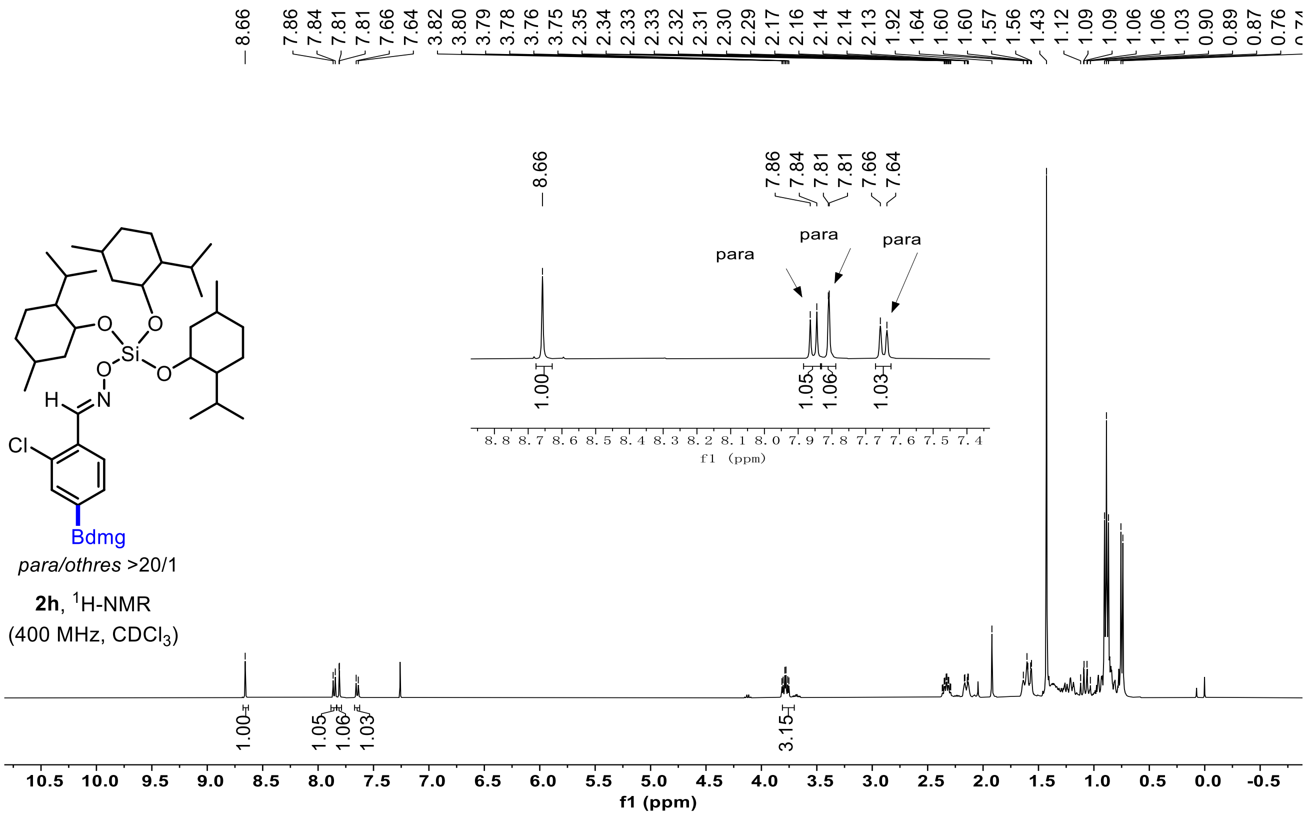

Supplementary Fig. 8: ^{1}H-NMR spectra of crude **2h** (25 °C, 400 MHz, $CDCl_3$)

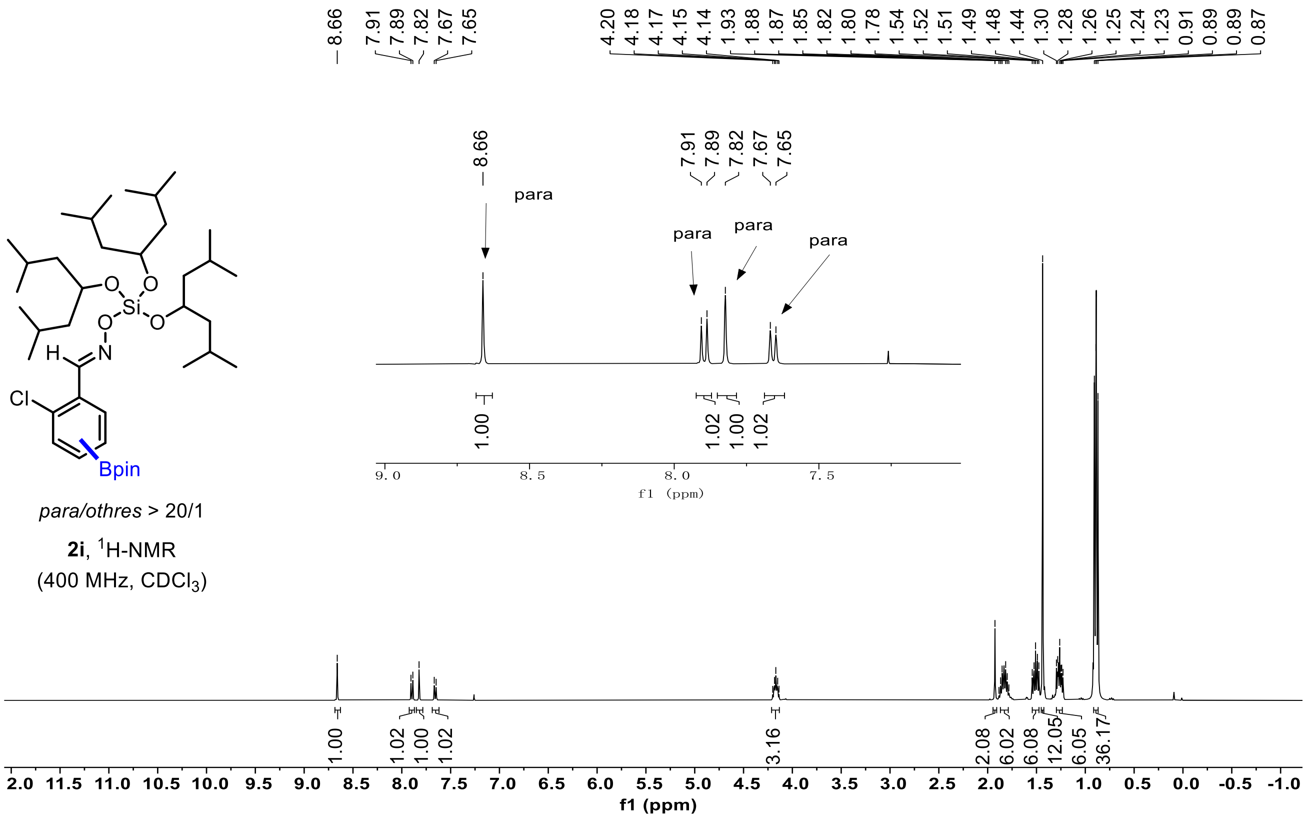

Supplementary Fig. 9: ^{1}H-NMR spectra of crude **2i** (25 °C, 400 MHz, $CDCl_3$)

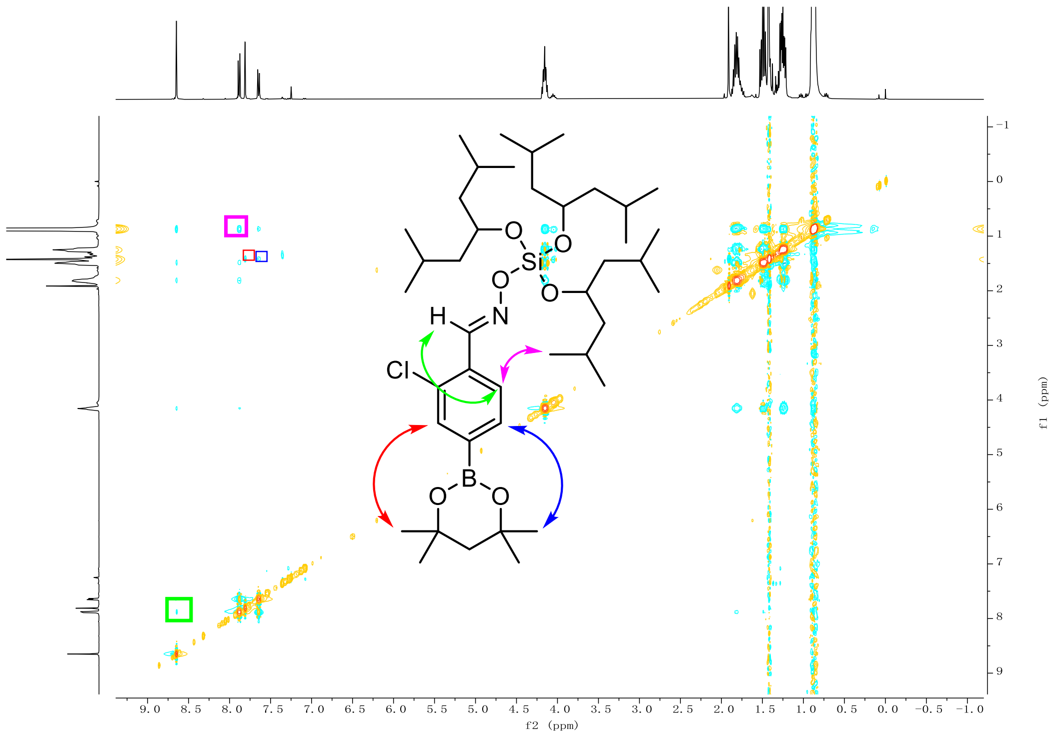

Supplementary Fig. 10: NOESY spectrum of **2i** (25 °C, 400 MHz, $CDCl_3$)

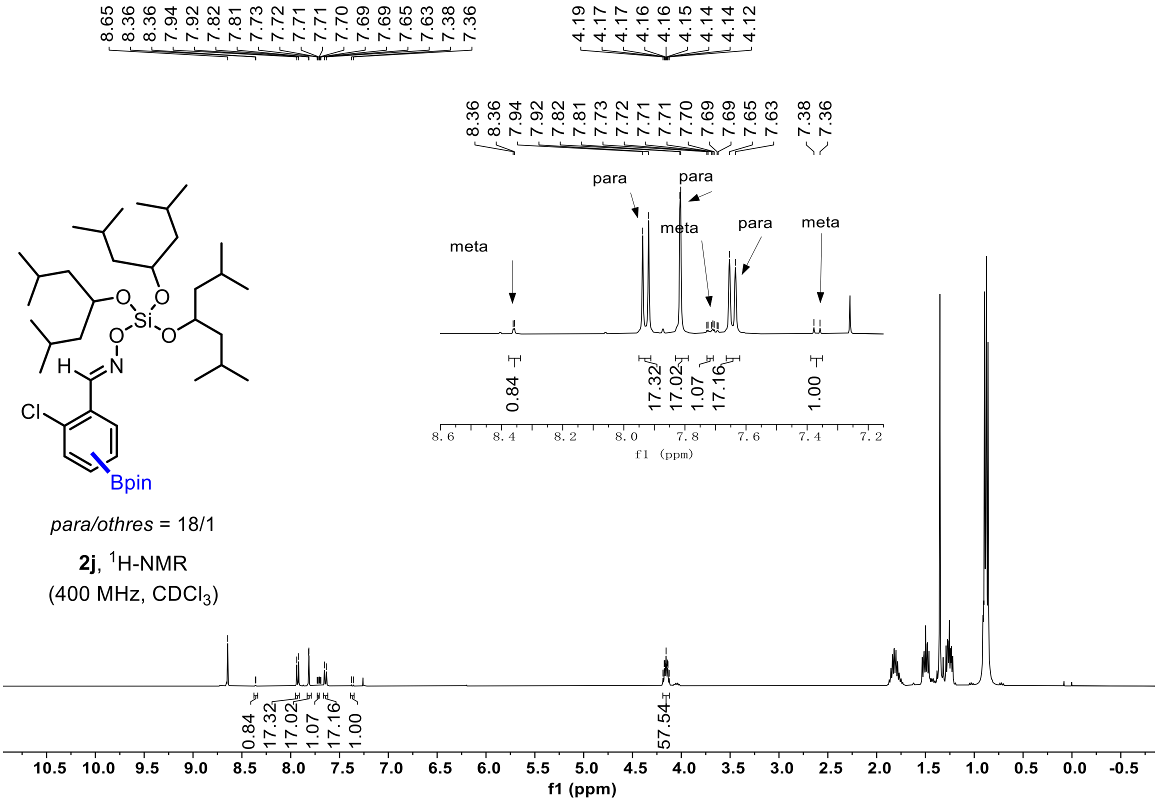

Supplementary Fig. 11: ^{1}H-NMR spectra of crude **2j** (25 °C, 400 MHz, $CDCl_3$)

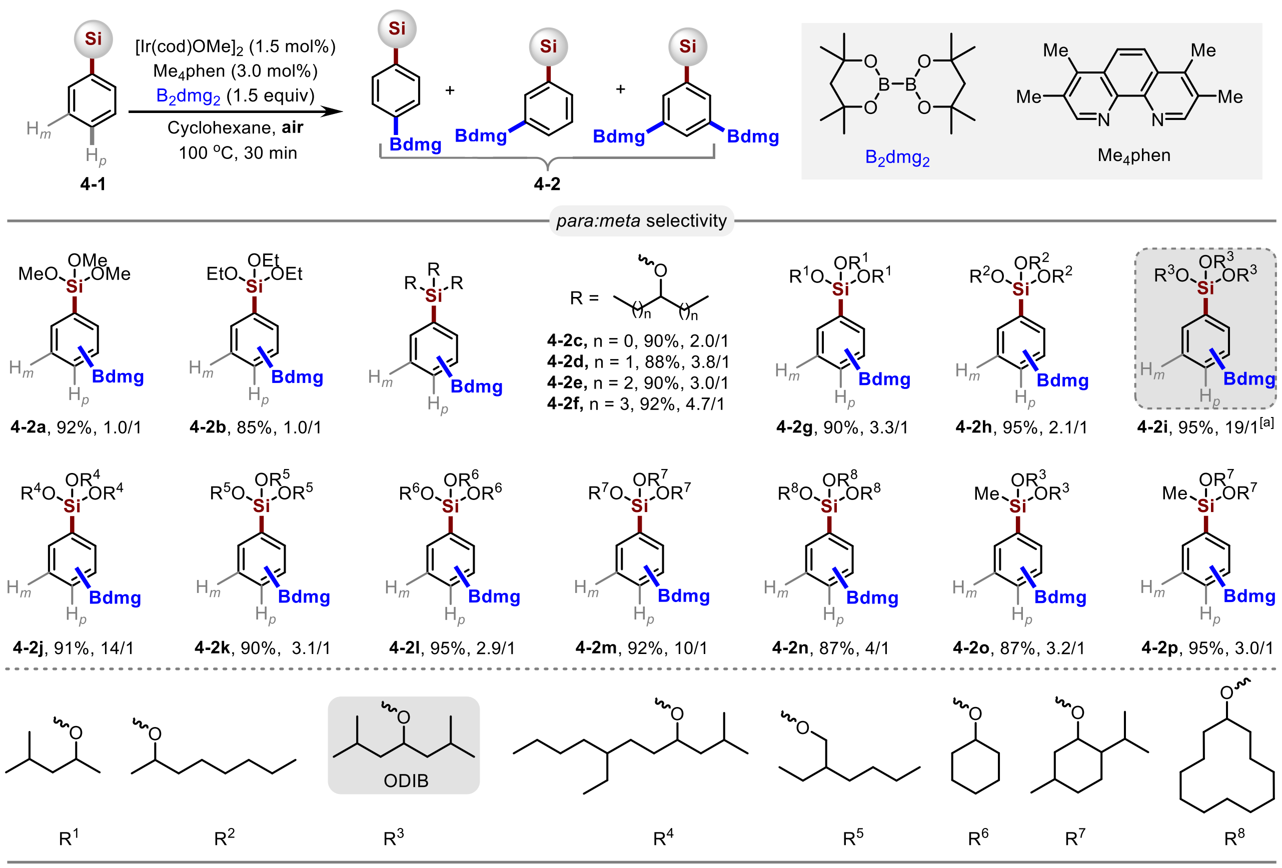

Supplementary Fig. 12: Effects of various silicon functional groups for *para*- selective C-H borylation. Substrate **4-1**, 0.2 mmol, B_2dmg_2 (1.5 equiv), $[Ir(OMe)cod]_2$ (1.5 mol%), and Me_4phen (3.0 mol%), cyclohexane (0.2 M), 100 °C, 30 min, isolated yield. [a] Reaction scale 3.0 mmol. Ratios of *meta* to *para* were determined from the crude ^{1}H-NMR spectra after borylation.

Typical procedure for the silylation of arenes:

Arylsilane **4-1** (0.2 mmol, 1.0 equiv), B_2dmg_2 (84.6 mg, 0.3 mmol, 1.5 equiv), $[Ir(cod)OMe]_2$ (2.0 mg, 0.003 mmol), Me_4phen (1.5 mg, 0.006 mmol), and Cyclohexane (1.0 mL) were added to a 15 mL glass vial under air atmosphere. The glass vial was capped with a teflon pressure cap and placed into an aluminum block pre-heated to 100 ºC for 30 minutes. After completion, cyclohexane was removed under reduced pressure and chromatographic separation with silica gel (20% ethylacetate in hexane as eluent) gave the borylated product **4-2** as colorless liquid. The *para:meta* ratio of products was reported from analysis of ^{1}H NMR.

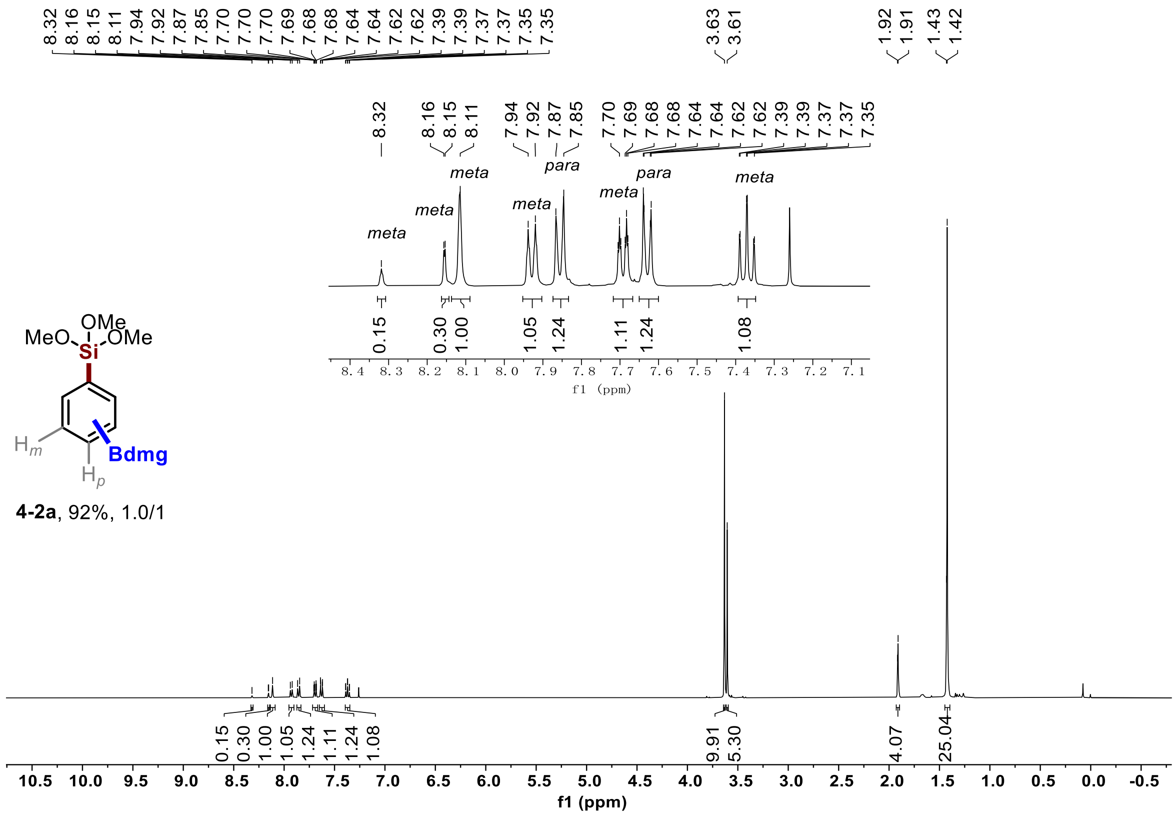

Supplementary Fig. 13: ^{1}H-NMR spectra of crude **4-2a** (25 °C, 400 MHz, $CDCl_3$)

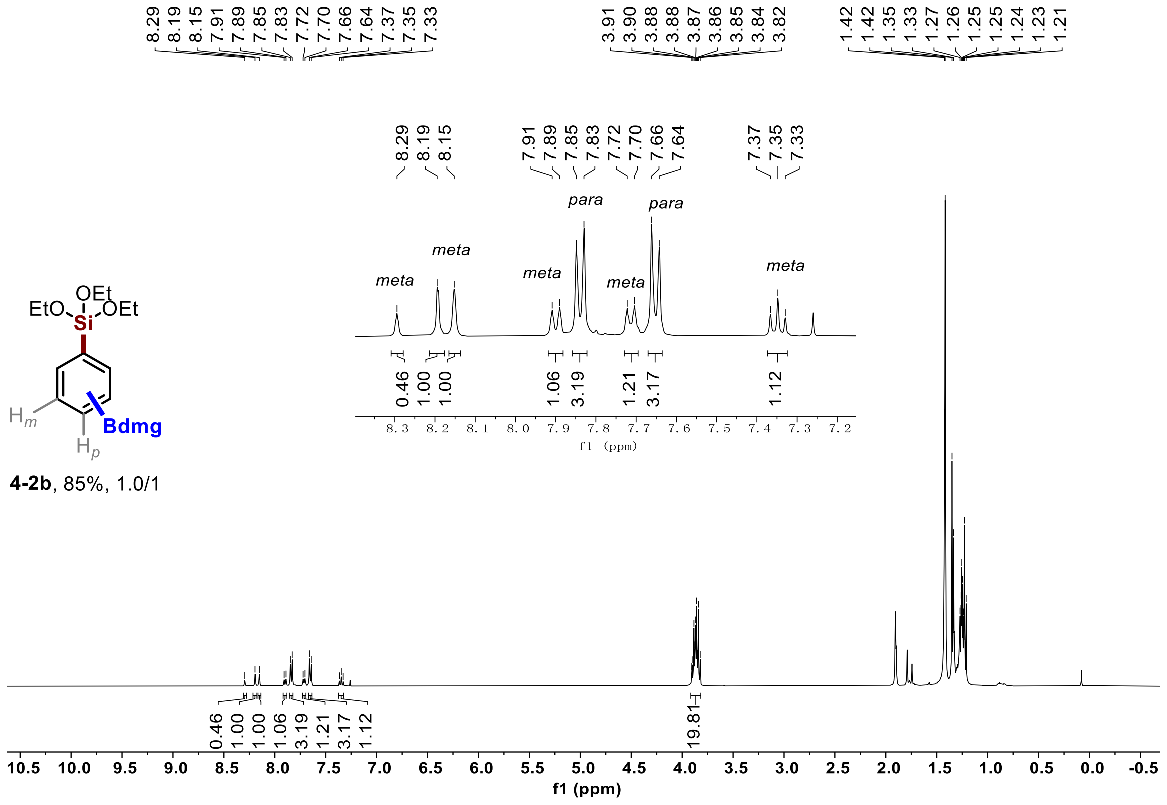

Supplementary Fig. 14: ^{1}H-NMR spectra of crude **4-2b** (25 °C, 400 MHz, $CDCl_3$)

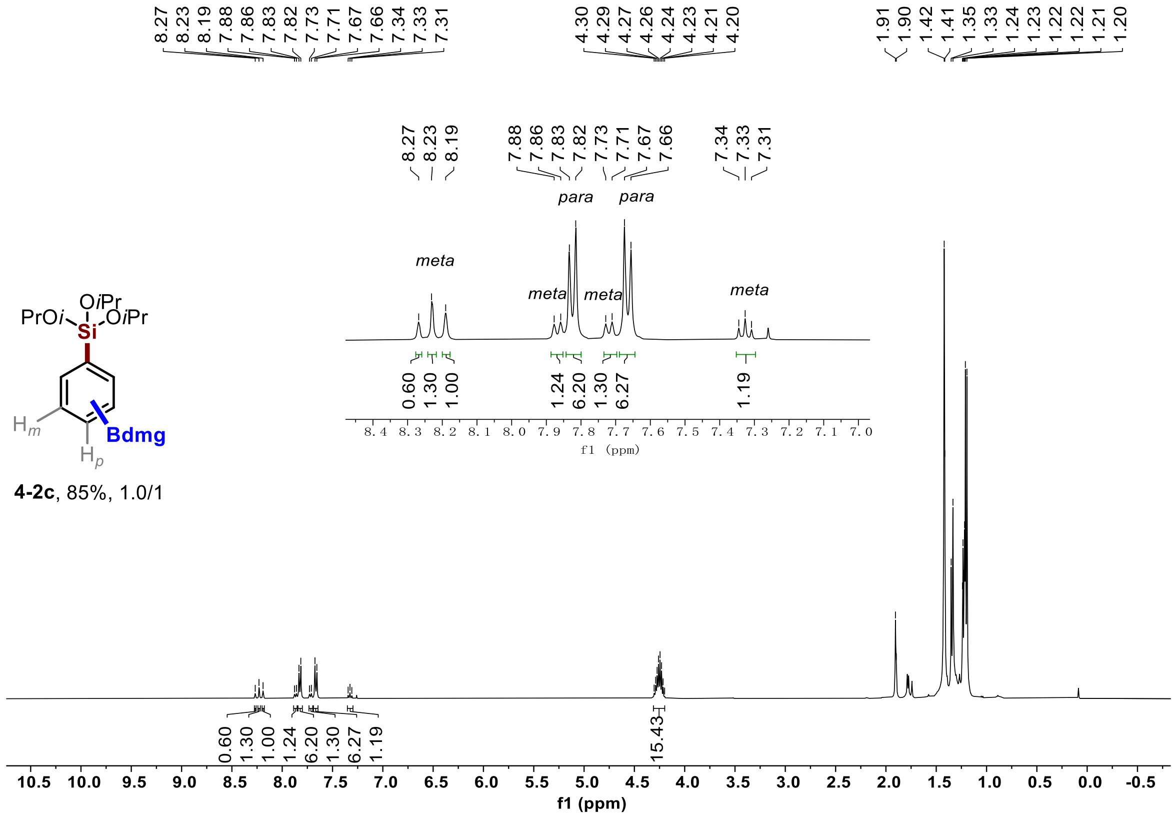

Supplementary Fig. 15: ^{1}H-NMR spectra of crude **4-2c** (25 °C, 400 MHz, $CDCl_3$)

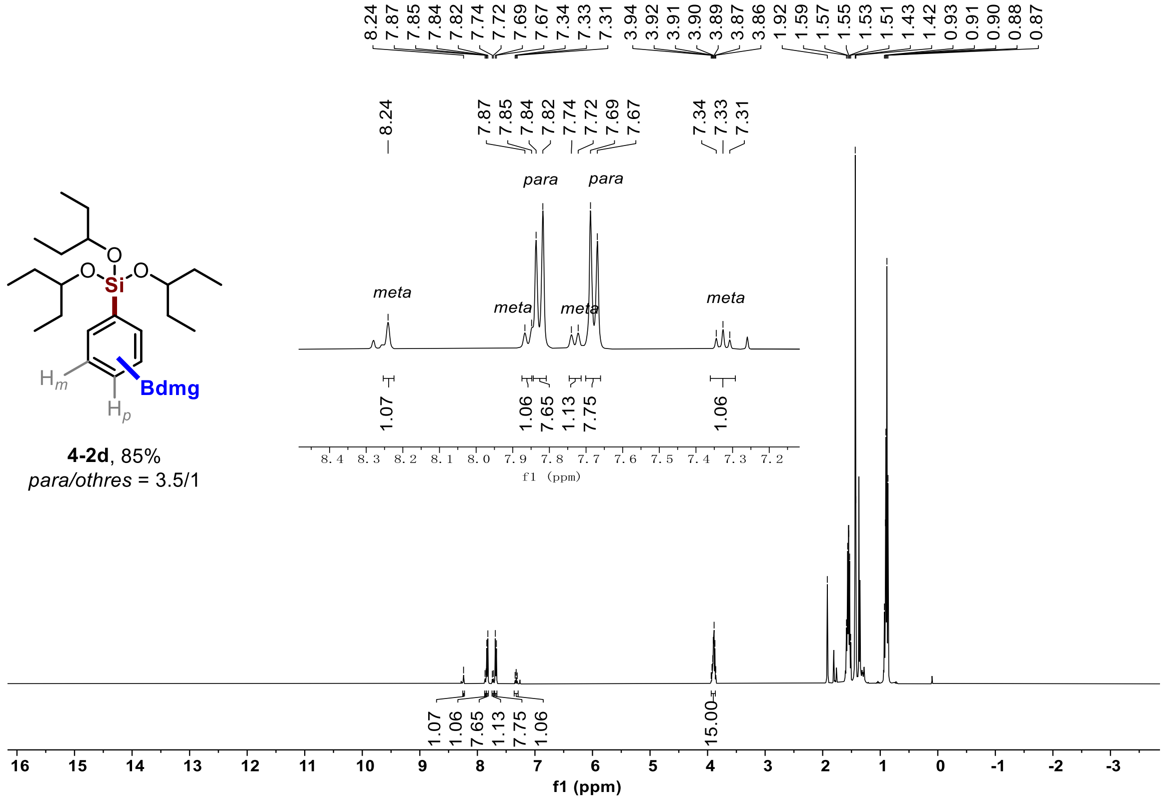

Supplementary Fig. 16: ^{1}H-NMR spectra of crude **4-2d** (25 °C, 400 MHz, $CDCl_3$)

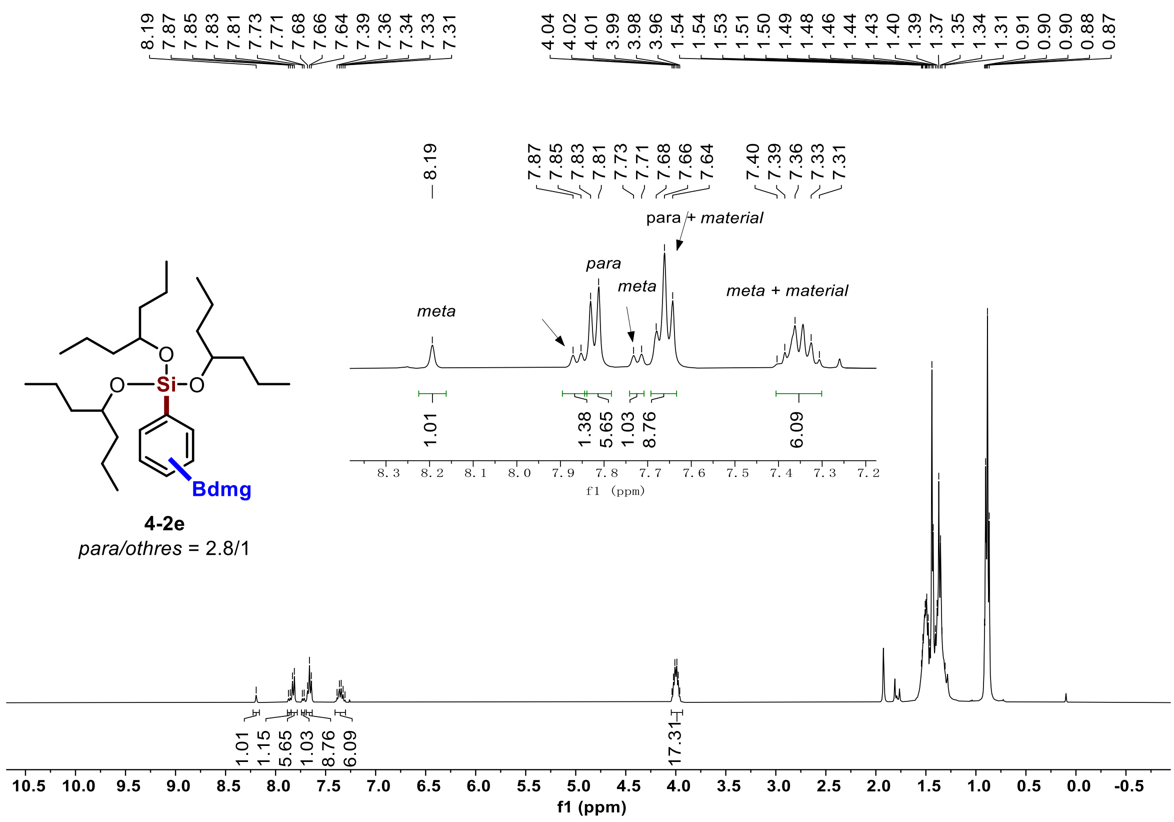

Supplementary Fig. 17: ^{1}H-NMR spectra of crude **4-2e** (25 °C, 400 MHz, $CDCl_3$)

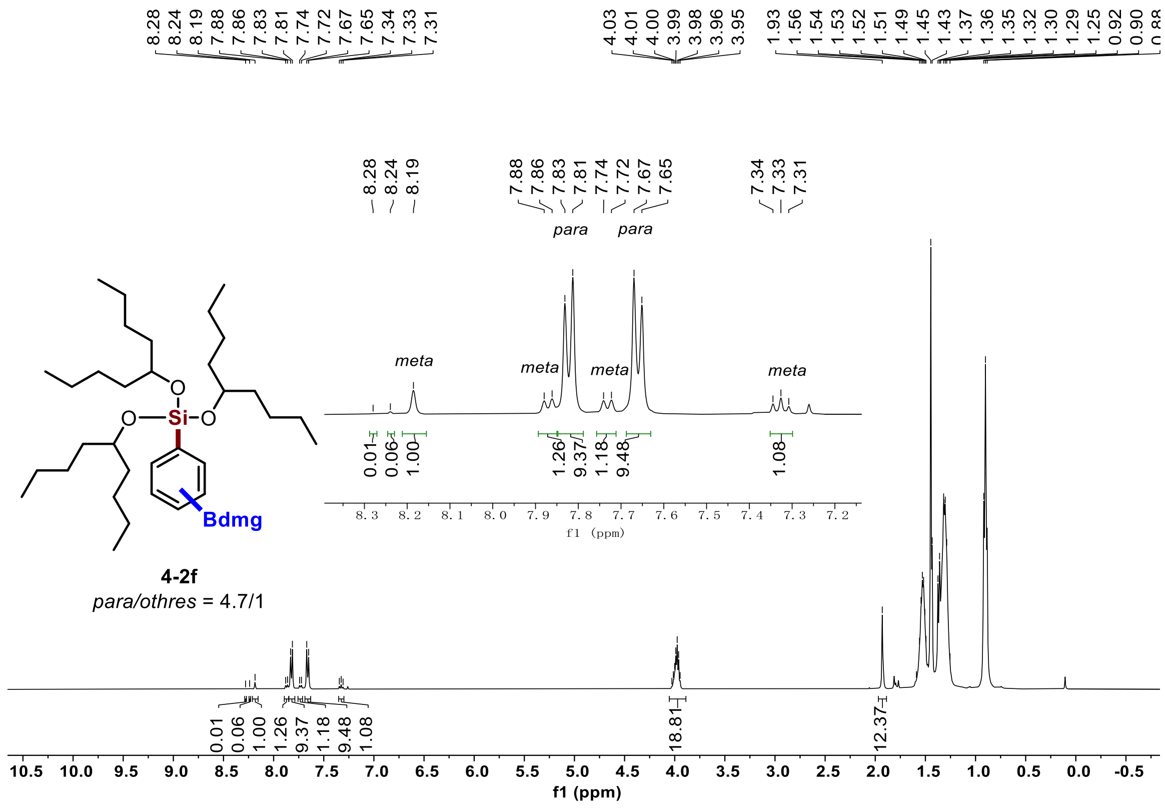

Supplementary Fig. 18: ^{1}H-NMR spectra of crude **4-2f** (25 °C, 400 MHz, $CDCl_3$)

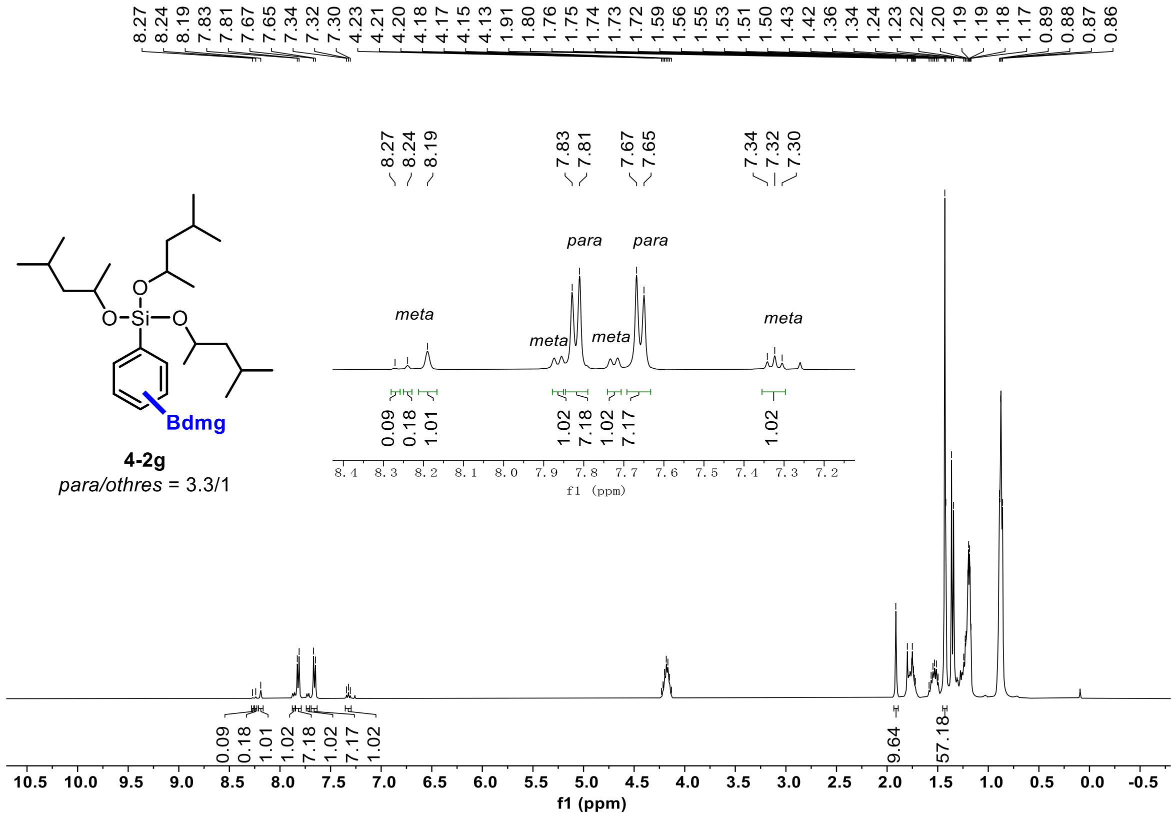

Supplementary Fig. 19: ¹H-NMR spectra of crude **4-2g** (25 °C, 400 MHz, $CDCl_3$)

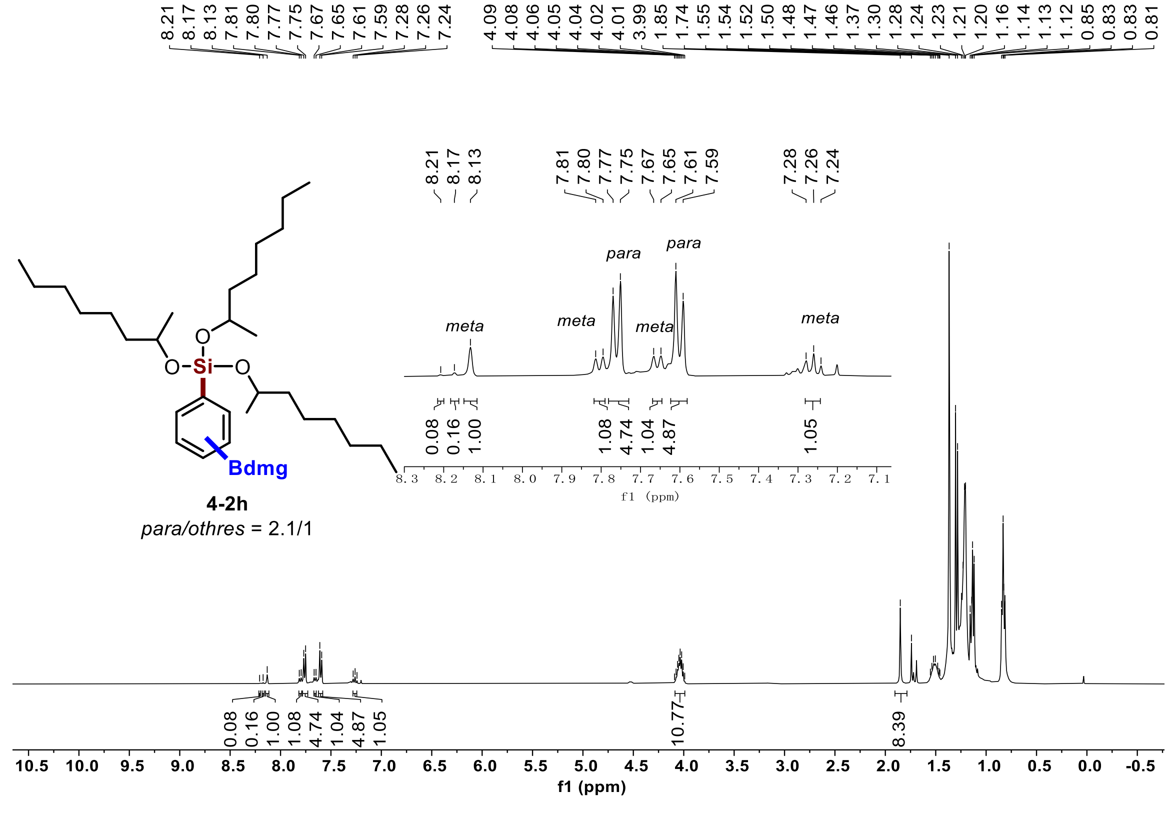

Supplementary Fig. 20: ¹H-NMR spectra of crude **4-2h** (25 °C, 400 MHz, $CDCl_3$)

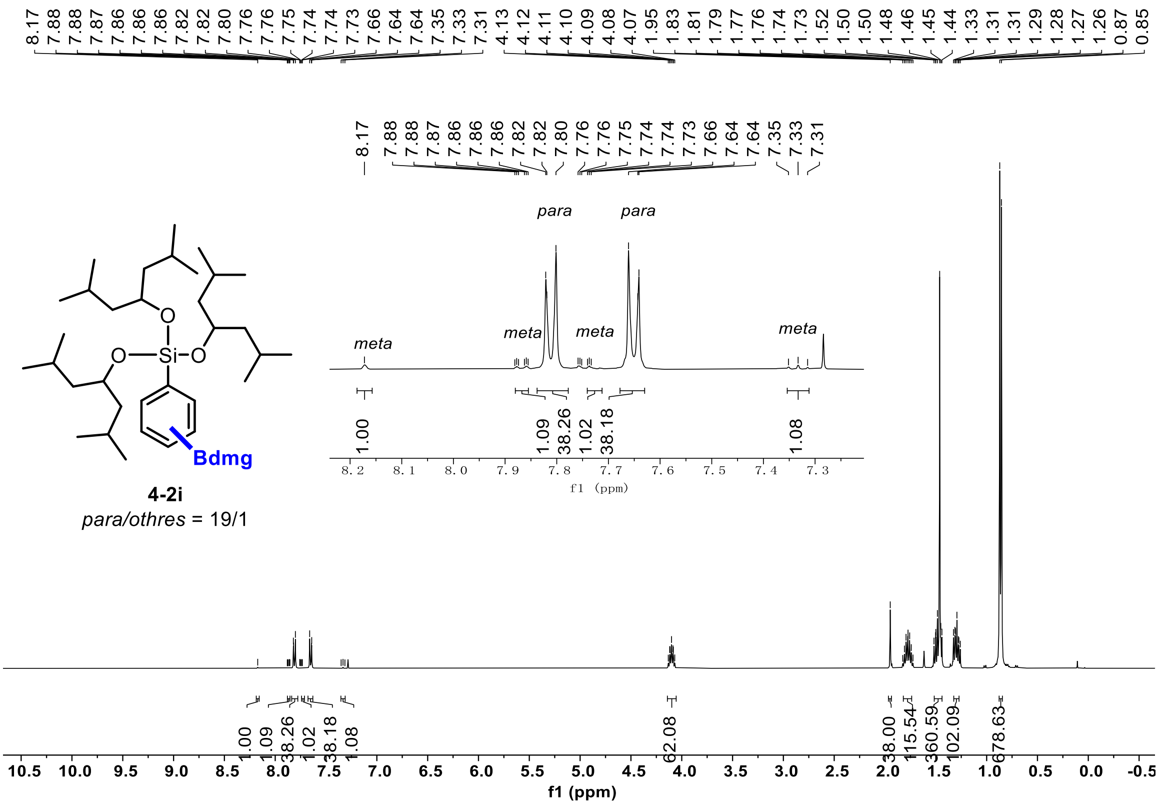

Supplementary Fig. 21: ^{1}H-NMR spectra of crude **4-2i** (25 ºC, 400 MHz, $CDCl_3$)

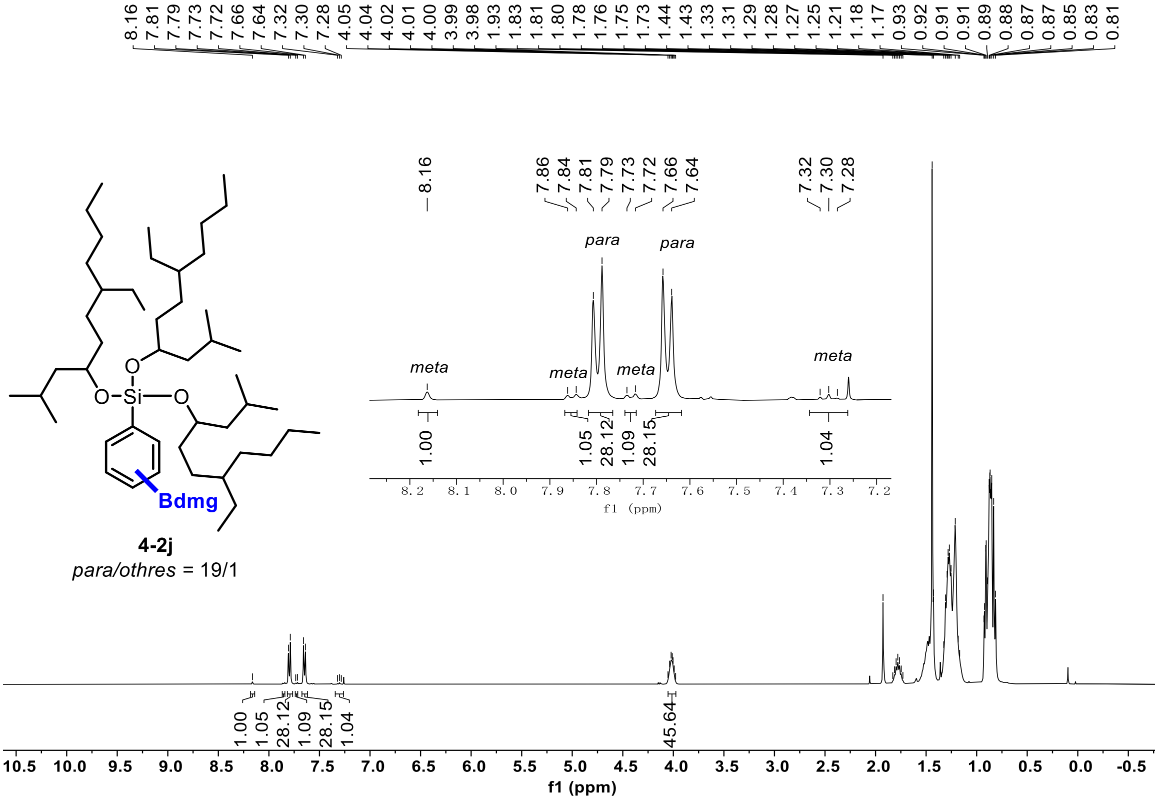

Supplementary Fig. 22: ^{1}H-NMR spectra of crude **4-2j** (25 ºC, 400 MHz, $CDCl_3$)

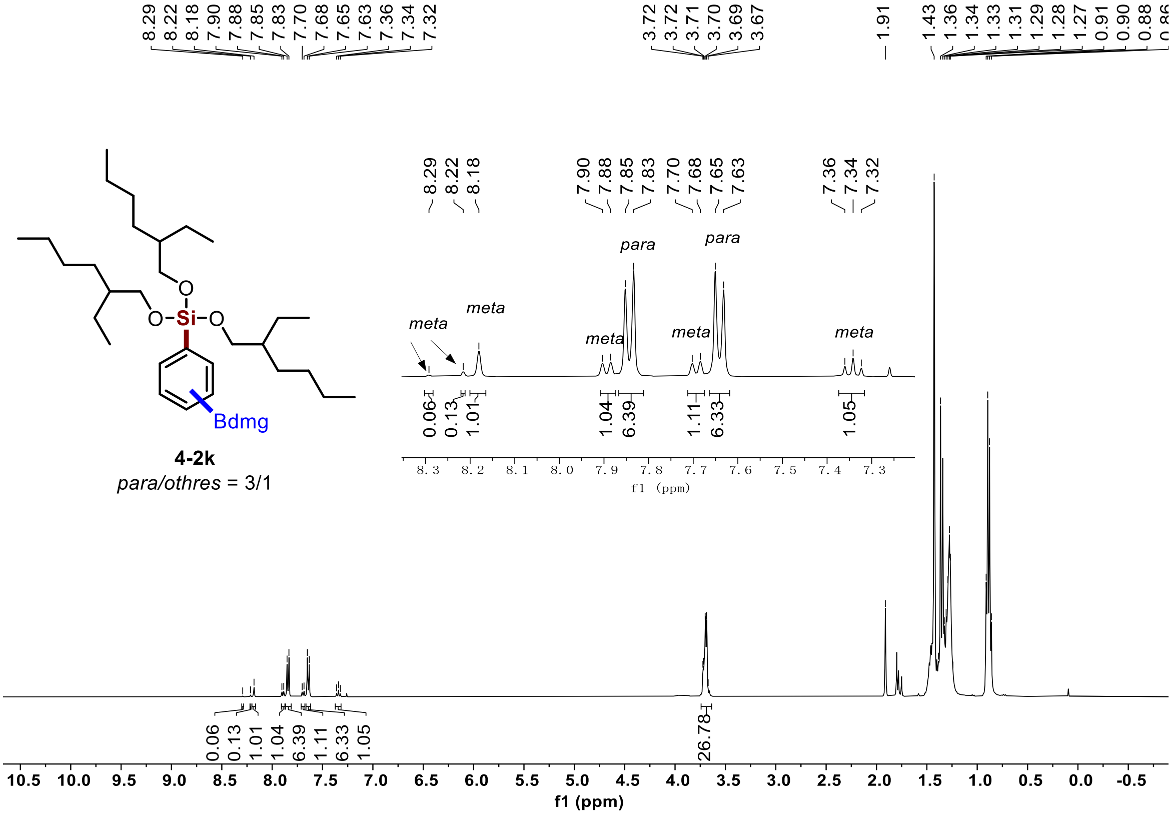

Supplementary Fig. 23: ^{1}H-NMR spectra of crude **4-2k** (25 °C, 400 MHz, $CDCl_3$)

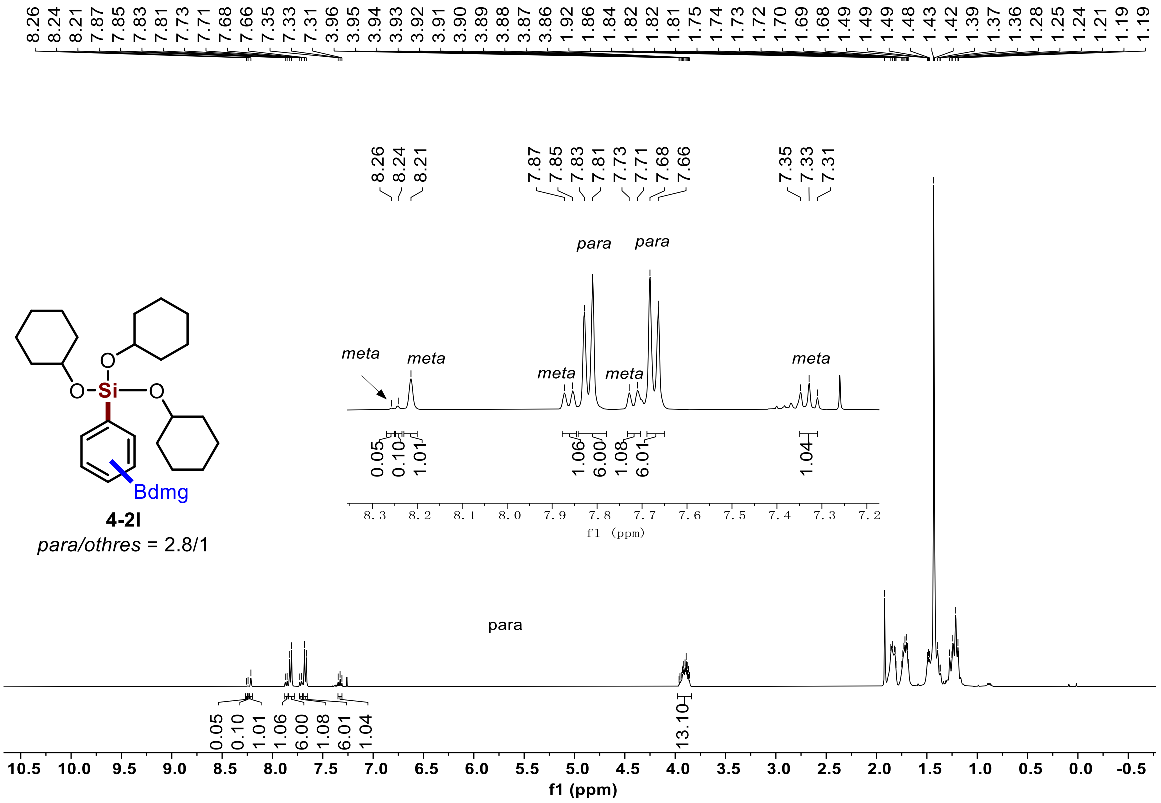

Supplementary Fig. 24: ^{1}H-NMR spectra of crude **4-2l** (25 °C, 400 MHz, $CDCl_3$)

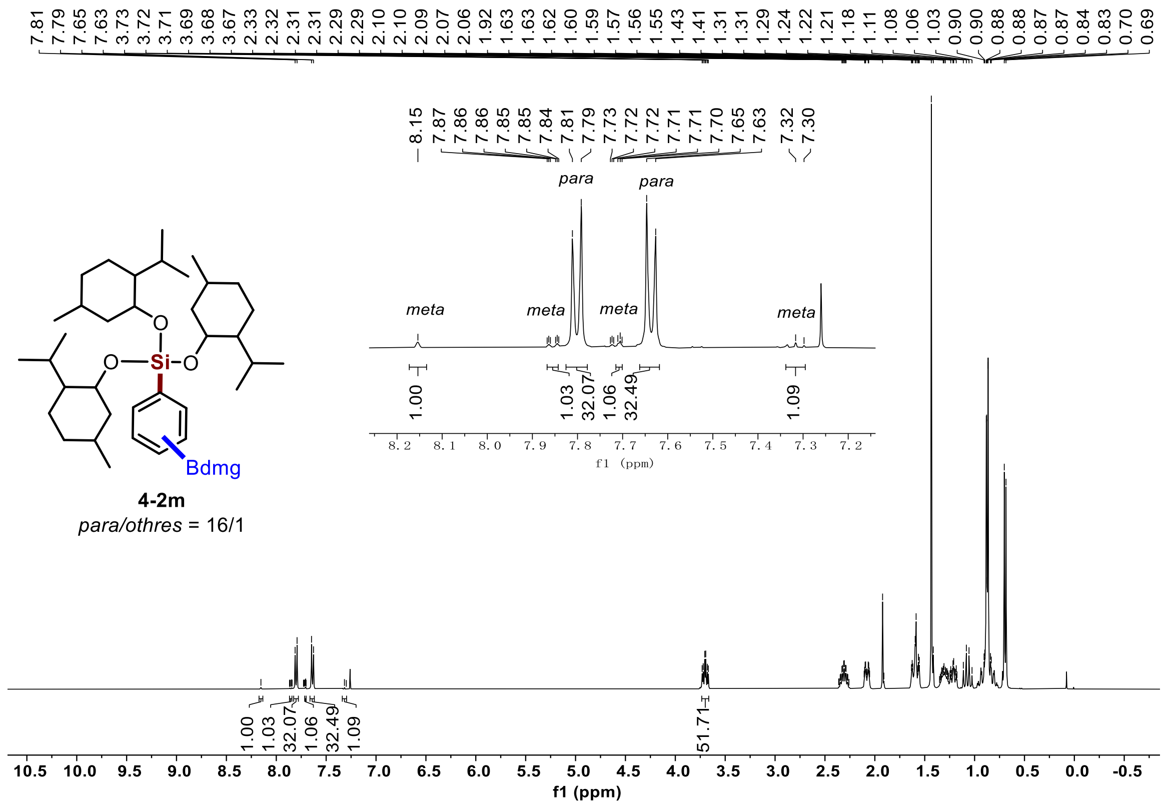

Supplementary Fig. 25: ^{1}H-NMR spectra of crude **4-2m** (25 °C, 400 MHz, $CDCl_3$)

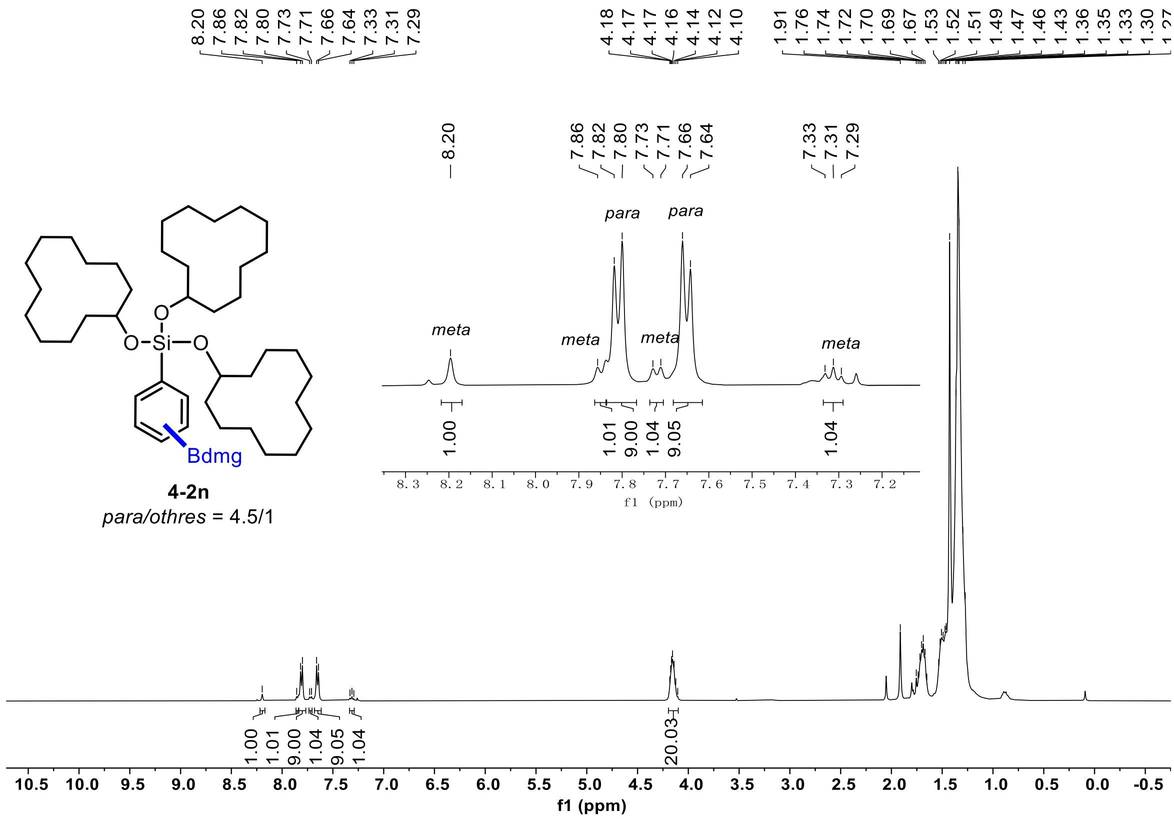

Supplementary Fig. 26: ^{1}H-NMR spectra of crude **4-2n** (25 °C, 400 MHz, $CDCl_3$)

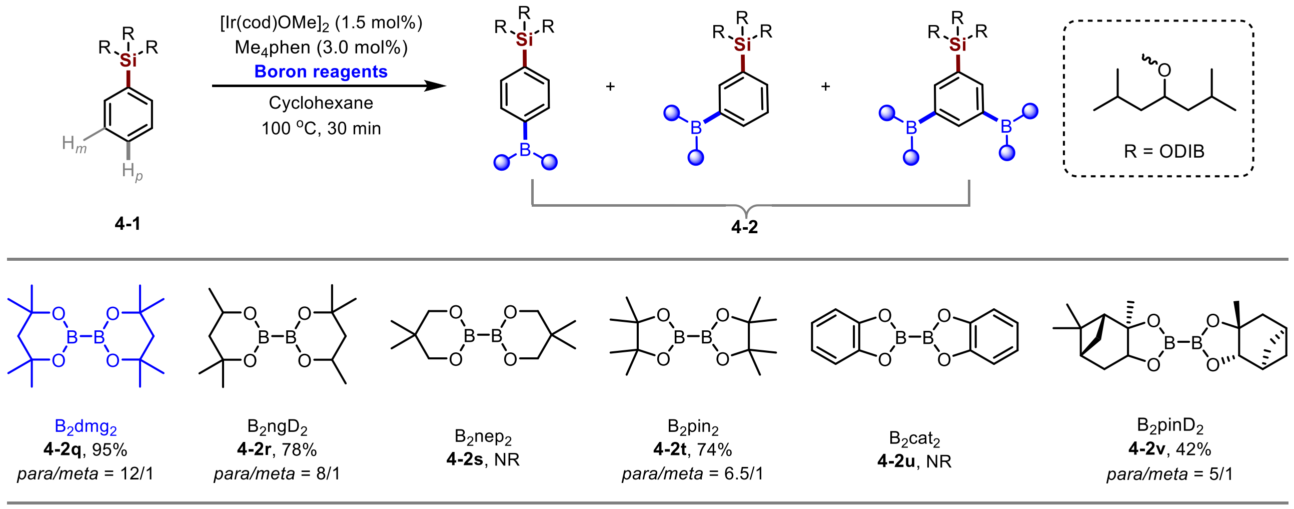

Supplementary Fig. 27: Effects of various boron reagents for *para*- selective C-H borylation. Substrate **4-1**, 0.2 mmol, boron reagent (1.5 equiv), $[Ir(OMe)cod]_2$ (1.5 mol%), and Me_4phen (3.0 mol%), cyclohexane (0.2 M), 100 °C, 30 min, isolated yield. [b]Reaction scale 3.0 mmol. Ratios of *meta* to *para* were determined from the crude ^{1}H-NMR spectra after borylation.

Typical procedure for the silylation of arenes:

Arylsilane **4-1** (0.2 mmol, 1.0 equiv), boron reagent (0.3 mmol, 1.5 equiv), $[Ir(cod)OMe]_2$ (2.0 mg, 0.003 mmol), Me_4phen (1.5 mg, 0.006 mmol), and Cyclohexane (1.0 mL) were added to a 15 mL glass vial under air atmosphere. The glass vial was capped with a teflon pressure cap and placed into an aluminum block pre-heated to 100 ºC for 30 minutes. After completion, cyclohexane was removed under reduced pressure and chromatographic separation with silica gel (20% ethylacetate in hexane as eluent) gave the borylated product **4-2** as colorless liquid. The *para:meta* ratio of products was reported from analysis of ^{1}H NMR.

3. Preparation of starting compounds

All derivatives of benzaldehyde and acetophenone oxime were synthesized according to previous reports.[1]

3.1 General procedure for the preparation of chlorotris((2,6-dimethylheptan-4-yl)oxy)silane (GP1)

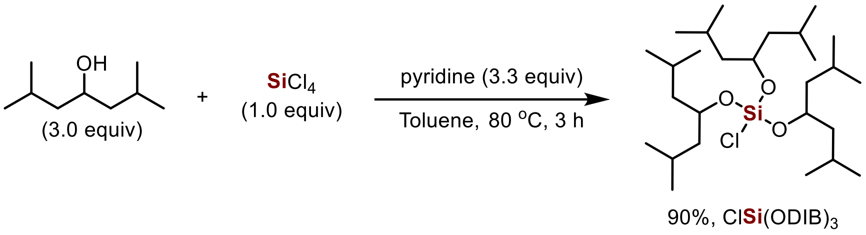

A 1000 mL, oven-dried double-necked round-bottomed flask equipped with a septum, nitrogen inlet, and a magnetic stir bar was charged with diisobutylcarbinol (522 mmol, 75.3 g, 3.0 equiv), pyridine (574 mmol, 45.4 g, 3.3 equiv) and toluene (400 mL). trichlorophenylsilane (23.5 g, 174 mmol) and toluene (400 mL). After the mixture was stirred at 0 °C for 5 min, $SiCl_4$ (174 mmol, 29.6 g, 1.0 equiv) was added via a syringe. The resulting mixture was heated at 80 °C for 3 h. After cooling to room temperature, the colorless precipitate of pyridinium chloride was filtered and the solvent was removed under reduced pressure. The residue consists of chlorotris((2,6-dimethylheptan-4-yl)oxy)silane in 90% yield (77.3 g). Chlorotris((2,6-dimethylhept-4-yl)oxy)silane was used directly without further purification for the next step.

3.2 General procedure B for the preparation of trialkylsilane derivatives of benzaldoxime (GP2)

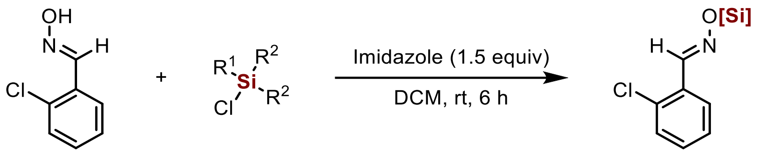

To a stirred solution of (E)-2-chlorobenzaldehyde oxime (1.5 g, 10 mmol) and trialkylchlorosilane (15 mmol, 1.5 equiv) in CH_2Cl_2 (20 mL) was added dropwise a solution of imidazole (15 mmol, 1.5 equiv) in CH_2Cl_2 (10 mL) at room temperature. The mixture was stirred at room temperature for 6 h. The white precipitate was removed by filtration under nitrogen. Water 20 mL was added and the product was extracted into CH_2Cl_2, washed with brine, dried over Na_2SO_4, and concentrated. The crude product was purified (5% ethyl acetate in hexane as eluent) by silica gel chromatography.[2]

2-chlorobenzaldehyde O-(tert-butyldimethylsilyl) oxime (1a)

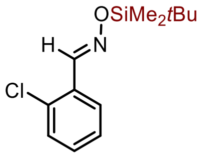

The product was obtained as a colorless oil.

^{1}H NMR (400 MHz, Chloroform-*d*) δ 8.63 (s, 1H), 7.91 (dd, *J* = 7.7, 1.8 Hz, 1H), 7.37 (dd, *J* = 7.9, 1.5 Hz, 1H), 7.32 – 7.23 (m, 2H), 1.00 (s, 9H), 0.26 (s, 6H). **^{13}C NMR** (101 MHz, $CDCl_3$) δ 150.4, 133.9, 130.8, 130.6, 130.0, 127.3, 127.0, 26.3, 18.4, -5.1. **HRMS (ESI)** *m/z* Calcd for $C_{13}H_{20}ClNNaOSi$ $[M+Na]^+$ 276.0946, Found: 276.0952.

2-chlorobenzaldehyde O-triethylsilyl oxime (1b)

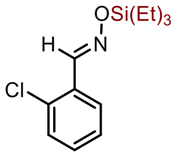

The product was obtained as a colorless oil. **^{1}H NMR** (400 MHz, $CDCl_3$) δ 8.61 (s, 1H), 7.90 (dd, *J* = 7.6, 2.0 Hz, 1H), 7.43 – 7.35 (m, 1H), 7.34 – 7.22 (m, 2H), 1.54 – 1.40 (m, 6H), 1.00 (t, *J* = 7.3 Hz, 9H), 0.83 – 0.73 (m, 6H). **^{13}C NMR** (101 MHz, $CDCl_3$) δ 150.3, 133.9, 130.8, 130.7, 123.0, 127.3, 127.0, 18.4, 16.8, 16.3. **HRMS (ESI)** *m/z* Calcd for $C_{13}H_{20}ClNNaOSi$ $[M+Na]^+$ 276.0946, Found: 276.0955.

3.3 General procedure C for the preparation of trialkoxysilane derivatives of 2-chlorobenzaldoxime (GP3)

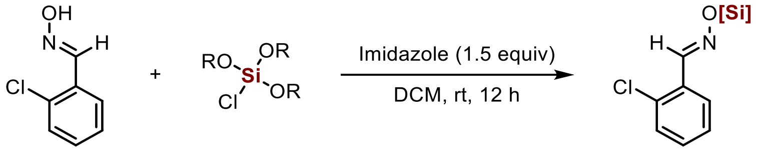

To a stirred solution of (E)-2-chlorobenzaldehyde oxime (1.5 g, 10 mmol) and trialkoxychlorosilane (15 mmol, 1.5 equiv) in CH_2Cl_2 (20 mL) was added dropwise a solution of imidazole (15 mmol, 1.5 equiv) in CH_2Cl_2 (10 mL) at room temperature. The mixture was stirred at room temperature for 12 h. The white precipitate was removed by filtration under nitrogen. Water 20 mL was added and the product was extracted into CH_2Cl_2, washed with brine, dried over Na_2SO_4, and concentrated. The crude product was purified (5% ethyl acetate in hexane as eluent) by silica gel chromatography.[3]

2-chlorobenzaldehyde O-triisopropoxysilyl oxime (1e)

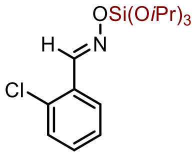

The product was obtained as a colorless oil. **^{1}H NMR** (400 MHz, $CDCl_3$) δ 8.62 (s, 1H), 7.84 (dd, *J* = 7.7, 1.9 Hz, 1H), 7.30 (dd, *J* = 8.0, 1.5 Hz, 1H), 7.28 – 7.14 (m, 2H), 4.29 (hept, *J* = 6.1 Hz, 3H), 1.18 (d, *J* = 6.2 Hz, 18H). **^{13}C NMR** (101 MHz, $CDCl_3$) δ 151.9, 134.2, 131.2, 130.1, 130.0, 127.4, 127.1, 66.8, 25.4. **HRMS (ESI)** *m/z* Calcd for $C_{16}H_{26}ClNNaO_4Si$ $[M+Na]^+$ 382.1212, Found: 382.1219.

2-chlorobenzaldehyde O-(tris((1,7,7-trimethylbicyclo[2.2.1]heptan-2-yl)oxy)silyl) oxime (1g)

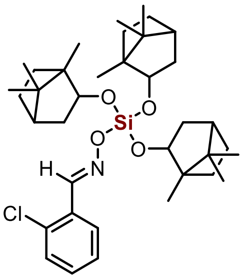

The product was obtained as a white solid. **^{1}H NMR** (400 MHz, $CDCl_3$) δ 8.67 (s, 1H), 7.90 (dd, *J* = 7.7, 1.9 Hz, 1H), 7.37 (dd, *J* = 7.9, 1.5 Hz, 1H), 7.35 – 7.22 (m, 2H), 4.38 – 4.31 (m, 3H), 2.28 – 2.17 (m, 3H), 2.13 – 2.03 (m, 3H), 1.76 – 1.63 (m, 3H), 1.62 – 1.57 (m, 3H), 1.30 – 1.10 (m, 9H), 0.85 (d, *J* = 5.2 Hz, 27H). **^{13}C NMR** (101 MHz, $CDCl_3$) δ 151.5, 134.2, 131.1, 130.2, 130.0, 127.3, 127.1, 78.8, 49.9, 47.6, 45.3, 39.0, 28.4, 26.3, 20.4, 18.9, 13.7. **HRMS (ESI)** *m/z* Calcd for $C_{37}H_{56}ClNNaO_4Si$ $[M+Na]^+$ 664.3559, Found: 664.3580.

2-chlorobenzaldehyde O-(tris((2-isopropyl-5-methylcyclohexyl)oxy)silyl) oxime (1h)

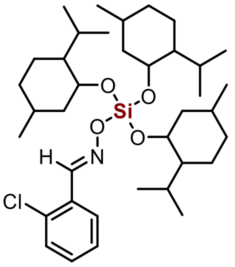

The product was obtained as a colorless oil. **^{1}H NMR** ((400 MHz, $CDCl_3$) δ 8.65 (s, 1H), 7.90 (dd, *J* = 7.8, 1.8 Hz, 1H), 7.37 (dd, *J* = 8.0, 1.4 Hz, 1H), 7.30 (td, *J* = 7.6, 1.8 Hz, 1H), 7.24 – 7.21 (m, 1H), 3.79 (td, *J* = 10.5, 4.3 Hz, 3H), 2.39 – 2.27 (m, 3H), 2.20 – 2.11 (m, 3H), 1.68 – 1.53 (m, 6H), 1.45 – 1.29 (m, 3H), 1.27 – 1.18 (m, 3H), 1.13 – 1.03 (m, 3H), 0.95 – 0.83 (m, 24H), 0.75 (d, *J* = 6.9 Hz, 9H). **^{13}C NMR** (101 MHz, $CDCl_3$) δ 151.4, 134.1, 131.1, 130.3, 130.0, 127.4, 126.9, 74.0, 49.9, 44.8, 34.7, 31.8, 25.3, 22.9, 22.4, 21.4, 15.9. **HRMS (ESI)** *m/z* Calcd for $C_{37}H_{63}ClNO_4Si$ $[M+H]^+$ 648.4029, Found: 648.4048.

2-chlorobenzaldehyde O-(tris((2,6-dimethylheptan-4-yl)oxy)silyl) oxime (1i)

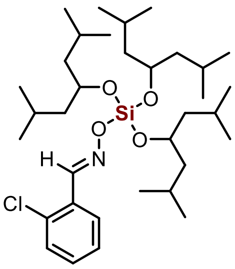

The product was obtained as a colorless oil. **^{1}H NMR** (400 MHz, $CDCl_3$) δ 8.64 (s, 1H), 7.93 (dd, *J* = 7.8, 1.8 Hz, 1H), 7.38 (dd, *J* = 8.0, 1.4 Hz, 1H), 7.31 (td, *J* = 7.6, 1.8 Hz, 1H), 7.24 (td, *J* = 7.5, 1.4 Hz, 1H), 4.26 – 4.11 (m, 3H), 1.92 – 1.71 (m, 6H), 1.56 – 1.45 (m, 6H), 1.32 – 1.21 (m, 6H), 0.88 (dd, *J* = 8.6, 6.6 Hz, 36H). **^{13}C NMR** (101 MHz, $CDCl_3$) δ 151.3, 134.1, 131.1, 130.3, 130.0, 127.5, 126.8, 70.8, 46.9, 24.4, 23.3, 22.7. **HRMS (ESI)** *m/z* Calcd for $C_{34}H_{62}ClNNaO_4Si$ $[M+Na]^+$ 634.4029, Found: 634.4042.

3.4 General procedure C for the preparation of trialkoxysilane derivatives of benzaldehyde and acetophenone oxime (GP4)

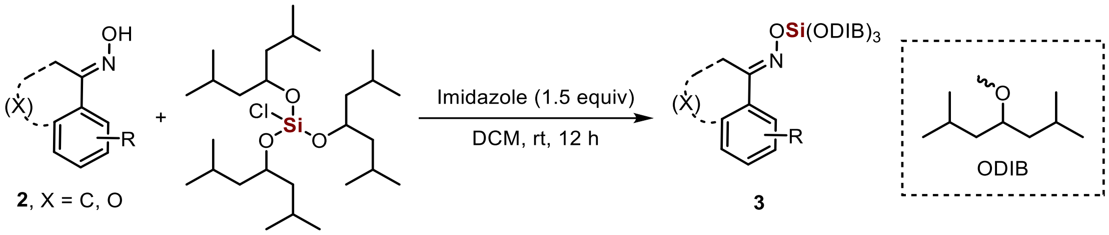

2, X = C, O

3

To a stirred solution of the corresponding benzaldehyde hydroxime or acetophenone hydroxime (5 mmol, 1.0 equiv) and chlorotris((2,6-dimethylheptan-4-yl)oxy)silane (7.5 mmol, 1.5 equiv) in CH_2Cl_2 (10 mL) was added dropwise a solution of imidazole (7.5 mmol, 1.5 equiv) in CH_2Cl_2 (10 mL) at room temperature. The mixture was stirred at room temperature for 12 h. The white precipitate was removed by filtration under nitrogen. Water 10 mL was added and the product was extracted into CH_2Cl_2, washed with brine, dried over Na_2SO_4 and concentrated. The crude product was purified (1% ethyl acetate in hexane as eluent) by silica gel chromatography as a colorless oil.[3]

2-methoxybenzaldehyde O-(tris((2,6-dimethylheptan-4-yl)oxy)silyl) oxime (3a)

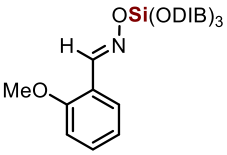

The product was obtained as colorless oil. **^{1}H NMR** (400 MHz, $CDCl_3$) δ 8.60 (t, J = 2.9 Hz, 1H), 7.89 – 7.79 (m, 1H), 7.39 – 7.30 (m, 1H), 6.97 – 6.86 (m, 2H), 4.21 – 4.13 (m, 3H), 3.85 (s, 3H), 1.89 – 1.79 (m, 6H), 1.58 – 1.44 (m, 6H), 1.38 – 1.20 (m, 6H), 0.94 – 0.84 (m, 36H). **^{13}C NMR** (101 MHz, $CDCl_3$) δ 157.7, 150.0, 131.3, 126.7, 121.1, 120.7, 111.1, 70.6, 55.7, 46.9, 24.4, 23.3, 22.7. **HRMS (ESI)** *m/z* Calcd for $C_{35}H_{65}ClNNaO_5Si$ $[M+Na]^+$ 630.4524, Found: 630.4511.

2-ethoxybenzaldehyde O-(tris((2,6-dimethylheptan-4-yl)oxy)silyl) oxime (4a)

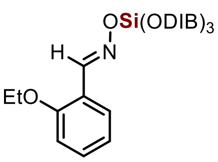

The product was obtained as colorless oil. **^{1}H NMR** (400 MHz, $CDCl_3$) δ 8.63 (s, 1H), 7.85 (d, J = 7.7 Hz, 1H), 7.36 – 7.27 (m, 1H), 6.99 – 6.78 (m, 2H), 4.22 – 4.11 (m, 3H), 4.06 (q, J = 7.0 Hz, 2H), 1.91 – 1.76 (m, 6H), 1.56 – 1.45 (m, 6H), 1.42 (t, J = 7.0 Hz, 6H), 1.31 – 1.20 (m, 3H), 0.88 (dd, J = 8.7, 6.8 Hz, 36H). **^{13}C NMR** (101 MHz, $CDCl_3$) δ 157.2, 150.3, 131.2, 126.6, 121.2, 120.5, 112.2, 70.6, 64.1, 46.9, 24.4, 23.3, 22.7, 14.9. **HRMS (ESI)** *m/z* Calcd for $C_{36}H_{67}ClNNaO_5Si$ $[M+Na]^+$ 644.4681, Found: 644.4699.

2-(2-methoxyethoxy)benzaldehyde O-(tris((2,6-dimethylheptan-4-yl)oxy)silyl) oxime (5a)

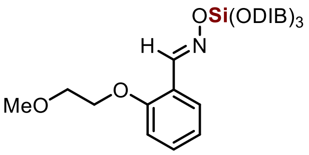

^{1}**H NMR** (400 MHz, $CDCl_3$) δ 8.64 (s, 1H), 7.86 (dd, *J* = 7.8, 1.7 Hz, 1H), 7.32 (ddd, *J* = 8.9, 7.4, 1.8 Hz, 1H), 7.07 – 6.84 (m, 2H), 4.22 – 4.11 (m, 5H), 3.81 – 3.74 (m, 2H), 3.46 (s, 3H), 1.99 – 1.71 (m, 6H), 1.51 (ddd, *J* = 13.6, 7.6, 6.0 Hz, 6H), 1.25 (ddd, *J* = 13.4, 8.0, 5.2 Hz, 6H), 0.88 (dd, *J* = 8.9, 6.6 Hz, 36H). ^{13}**C NMR** (101 MHz, $CDCl_3$) δ 157.0, 150.1, 131.3, 126.7, 121.5, 121.0, 112.5, 71.1, 70.6, 68.4, 59.5, 46.9, 24.4, 23.3, 22.7. **HRMS (ESI)** *m/z* Calcd for $C_{37}H_{69}ClNNaO_6Si$ $[M+Na]^+$ 674.4786, Found: 674.4811.

2-(difluoromethoxy)benzaldehyde O-(tris((2,6-dimethylheptan-4-yl)oxy)silyl) oxime (6a)

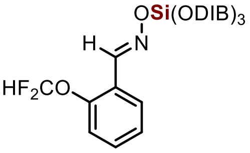

^{1}**H NMR** (400 MHz, $CDCl_3$) δ 8.53 (s, 1H), 7.96 (dd, *J* = 7.9, 1.8 Hz, 1H), 7.48 – 7.34 (m, 1H), 7.24 – 7.19 (m, 1H), 7.15 (dd, *J* = 8.3, 1.1 Hz, 1H), 6.52 (t, *J* = 73.4 Hz, 1H), 4.21 – 4.12 (m, 3H), 1.90 – 1.75 (m, 6H), 1.51 (ddd, *J* = 13.5, 7.6, 5.9 Hz, 6H), 1.27 (ddd, *J* = 13.4, 8.1, 5.2 Hz, 6H), 0.89 (dd, *J* = 8.4, 6.5 Hz, 36H). ^{13}**C NMR** (101 MHz, $CDCl_3$) δ 149.3 (t, *J* = 2.5 Hz), 149.0, 131.3, 127.2, 125.7, 124.8, 119.8, 116.1 (t, *J* = 261.2 Hz), 70.8, 47.0, 24.4, 23.3, 22.7. ^{19}**F NMR** (376 MHz, $CDCl_3$) δ -80.7. **HRMS (ESI)** *m/z* Calcd for $C_{35}H_{63}F_2NNaO_5Si$ $[M+Na]^+$ 666.4336, Found: 666.4359.

2-(trifluoromethoxy)benzaldehyde O-(tris((2,6-dimethylheptan-4-yl)oxy)silyl) oxime (7a)

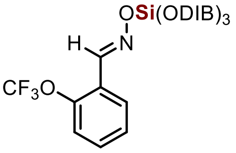

^{1}**H NMR** (400 MHz, $CDCl_3$) δ 8.49 (s, 1H), 7.99 (dd, *J* = 7.8, 1.7 Hz, 1H), 7.42 (td, *J* = 7.7, 1.7 Hz, 1H), 7.34 – 7.24 (m, 2H), 4.22 – 4.11 (m, 3H), 1.90 – 1.74 (m, 6H), 1.56 – 1.45 (m, 6H), 1.26 (ddd, *J* = 13.5, 8.2, 5.1 Hz, 6H), 0.88 (dd, *J* = 8.2, 6.6 Hz, 36H). ^{13}**C NMR** (101 MHz, $CDCl_3$) δ 148.5, 147.4, 131.3, 127.2, 127.0, 125.8, 120.5 (q = 257.3), 70.8, 46.9, 24.4, 23.3, 22.7. ^{19}**F NMR** (376 MHz, $CDCl_3$) δ -57.7. **HRMS (ESI)** *m/z* Calcd for $C_{35}H_{62}F_3NNaO_5Si$ $[M+Na]^+$ 684.4242, Found: 684.4230.

2-(trifluoromethyl)benzaldehyde O-(tris((2,6-dimethylheptan-4-yl)oxy)silyl) oxime (8a)

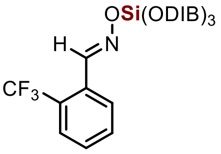

^{1}**H NMR** (400 MHz, $CDCl_3$) δ 8.59 (s, 1H), 8.13 (d, *J* = 7.7 Hz, 1H), 7.69 (dd, *J* = 7.7, 1.5 Hz, 1H), 7.61 – 7.43 (m, 2H), 4.22 – 4.11 (m, 3H), 2.06 – 1.74 (m, 6H), 1.57 – 1.42 (m, 6H), 1.32 – 1.21 (m, 6H), 0.88 (dd, *J* = 8.0, 6.5 Hz, 36H). ^{13}**C NMR** (101 MHz, $CDCl_3$) δ 151.0 (d, *J* = 1.8 Hz), 131.8, 130.7 (d, *J* = 1.3 Hz), 129.7, 129.0 – 128.1 (m), 127.5, 125.9 (q, *J* = 5.6 Hz), 122.7, 70.9, 46.9, 24.4, 23.3, 22.7. **HRMS (ESI)** *m/z* Calcd for $C_{35}H_{62}F_3NNaO_4Si$ $[M+Na]^+$ 668.4292, Found: 668.4310.

2-chlorobenzaldehyde O-(tris((2,6-dimethylheptan-4-yl)oxy)silyl) oxime (9a)

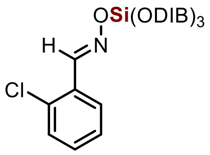

^{1}H NMR (400 MHz, $CDCl_3$) δ 8.64 (s, 1H), 7.93 (dd, J = 7.8, 1.8 Hz, 1H), 7.38 (dd, J = 8.0, 1.4 Hz, 1H), 7.31 (td, J = 7.6, 1.8 Hz, 1H), 7.24 (td, J = 7.5, 1.4 Hz, 1H), 4.26 – 4.11 (m, 3H), 1.92 – 1.71 (m, 6H), 1.56 – 1.45 (m, 6H), 1.32 – 1.21 (m, 6H), 0.88 (dd, J = 8.6, 6.6 Hz, 36H). **^{13}C NMR** (101 MHz, $CDCl_3$) δ 151.3, 134.1, 131.1, 130.3, 130.0, 127.5, 126.8, 70.8, 46.9, 24.4, 23.3, 22.7. **HRMS (ESI)** *m/z* Calcd for $C_{34}H_{62}ClNNaO_4Si$ $[M+Na]^+$ 634.4029, Found: 634.4043.

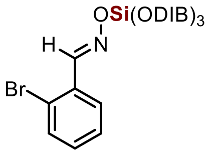

2-bromobenzaldehyde O-(tris((2,6-dimethylheptan-4-yl)oxy)silyl) oxime (10a)

^{1}H NMR (400 MHz, $CDCl_3$) δ 8.61 (s, 1H), 7.92 (dd, J = 7.6, 1.8 Hz, 1H), 7.57 (dd, J = 7.8, 1.4 Hz, 1H), 7.47 – 7.15 (m, 2H), 4.22 – 4.10 (m, 3H), 1.91 – 1.75 (m, 6H), 1.50 (ddd, J = 13.6, 7.6, 6.0 Hz, 6H), 1.26 (ddd, J = 13.4, 8.1, 5.2 Hz, 6H), 0.88 (dd, J = 8.7, 6.6 Hz, 36H). **^{13}C NMR** (101 MHz, $CDCl_3$) δ 153.6, 133.2, 131.8, 131.3, 127.8, 127.4, 124.1, 70.8, 46.9, 24.4, 23.3, 22.7. **HRMS (ESI)** *m/z* Calcd for $C_{34}H_{62}BrNNaO_4Si$ $[M+Na]^+$ 678.3524, Found: 678.3529.

2-(piperidin-1-yl)benzaldehyde O-(tris((2,6-dimethylheptan-4-yl)oxy)silyl) oxime (11a)

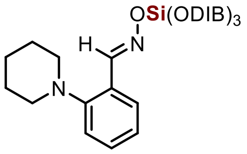

^{1}H NMR (400 MHz, $CDCl_3$) δ 8.52 (s, 1H), 7.83 (dd, J = 7.8, 1.7 Hz, 1H), 7.51 – 7.28 (m, 1H), 7.10 – 6.96 (m, 2H), 4.24 – 4.13 (m, 3H), 2.98 – 2.78 (m, 4H), 1.92 – 1.78 (m, 6H), 1.72 (p, J = 5.7 Hz, 4H), 1.62 – 1.57 (m, 2H), 1.52 (ddd, J = 13.6, 7.6, 5.9 Hz, 6H), 1.26 (ddd, J = 13.4, 8.1, 5.2 Hz, 6H), 0.89 (dd, J = 9.1, 6.6 Hz, 36H). **HRMS (ESI)** *m/z* Calcd for $C_{39}H_{72}N_2NaO_4Si$ $[M+Na]^+$ 683.5154, Found: 683.5169.

2-morpholinobenzaldehyde O-(tris((2,6-dimethylheptan-4-yl)oxy)silyl) oxime (12a)

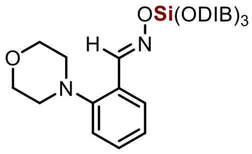

^{1}H NMR (400 MHz, $CDCl_3$) δ 8.55 (s, 1H), 7.83 (dd, J = 8.1, 1.6 Hz, 1H), 7.45 – 7.31 (m, 1H), 7.12 – 7.01 (m, 2H), 4.21 – 4.12 (m, 3H), 3.90 – 3.81 (m, 4H), 3.00 – 2.91 (m, 4H), 1.94 – 1.76 (m, 6H), 1.50 (ddd, J = 13.4, 7.5, 5.9 Hz, 6H), 1.25 (ddd, J = 13.4, 8.0, 5.0 Hz, 6H), 0.88 (dd, J = 8.2, 6.6 Hz, 36H). **^{13}C NMR** (101 MHz, $CDCl_3$) δ 151.9, 130.9, 127.6, 126.4, 123.6, 119.1, 70.6, 67.3, 53.5, 46.9, 24.4, 23.3, 22.7. **HRMS (ESI)** *m/z* Calcd for $C_{38}H_{70}N_2NaO_5Si$ $[M+Na]^+$ 685.4946, Found: 685.4923.

Ethyl-2-(4,4-bis((2,6-dimethylheptan-4-yl)oxy)-6-isobutyl-8-methyl-3,5-dioxa-2-aza-4-silanon-1-en-1-yl)benzoate (13a)

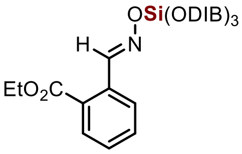

^{1}**H NMR** (400 MHz, $CDCl_3$) δ 9.02 (s, 1H), 7.98 (ddd, J = 19.9, 7.7, 1.5 Hz, 2H), 7.53 – 7.39 (m, 2H), 4.38 (q, J = 7.1 Hz, 2H), 4.21 – 4.12 (m, 3H), 1.90 – 1.75 (m, 6H), 1.50 (ddd, J = 13.5, 7.6, 5.9 Hz, 6H), 1.40 (t, J = 7.1 Hz, 3H), 1.25 (ddd, J = 13.4, 8.1, 5.2 Hz, 6H), 0.87 (dd, J = 9.2, 6.6 Hz, 36H). ^{13}**C NMR** (101 MHz, $CDCl_3$) δ 166.8, 153.5, 133.2, 131.9, 130.7, 129.9, 129.5, 127.5, 70.7, 61.5, 46.9, 24.4, 23.3, 22.7, 14.4. **HRMS (ESI)** *m/z* Calcd for $C_{37}H_{67}NNaO_6Si$ $[M+Na]^+$ 672.4630, Found: 672.4649.

2-bromo-5-fluorobenzaldehyde O-(tris((2,6-dimethylheptan-4-yl)oxy)silyl) oxime (14a)

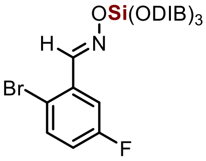

^{1}**H NMR** (400 MHz, $CDCl_3$) δ 8.54 (d, J = 1.8 Hz, 1H), 7.63 (dd, J = 9.5, 3.1 Hz, 1H), 7.53 (dd, J = 8.9, 5.2 Hz, 1H), 7.03 – 6.91 (m, 1H), 4.56 – 3.86 (m, 3H), 1.89 – 1.74 (m, 6H), 1.59 – 1.40 (m, 6H), 1.39 – 1.17 (m, 6H), 0.88 (dd, J = 8.2, 6.6 Hz, 36H). ^{13}**C NMR** (101 MHz, $CDCl_3$) δ 161.9 (d, J = 247.1 Hz), 152.8 (d, J = 2.4 Hz), 134.5 (d, J = 7.8 Hz), 133.6 (d, J = 8.4 Hz), 118.8 (d, J = 23.1 Hz), 118.1 (d, J = 3.2 Hz), 114.3 (d, J = 24.6 Hz), 70.9, 46.9, 24.4, 23.3, 22.7. **HRMS (ESI)** *m/z* Calcd for $C_{34}H_{61}BrFNNaO_4Si$ $[M+Na]^+$ 696.3429, Found: 696.3421.

5-fluoro-2-methylbenzaldehyde O-(tris((2,6-dimethylheptan-4-yl)oxy)silyl) oxime (15a)

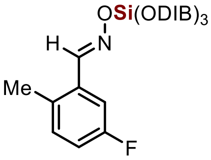

^{1}**H NMR** (400 MHz, $CDCl_3$) δ 8.44 (d, J = 1.7 Hz, 1H), 7.52 (dd, J = 10.0, 2.8 Hz, 1H), 7.13 (dd, J = 8.5, 5.6 Hz, 1H), 6.97 (td, J = 8.2, 2.8 Hz, 1H), 4.23 – 4.12 (m, 3H), 2.38 (s, 3H), 1.91 – 1.76 (m, 6H), 1.51 (ddd, J = 13.6, 7.6, 5.9 Hz, 6H), 1.27 (ddd, J = 13.5, 8.1, 5.2 Hz, 6H), 0.89 (dd, J = 8.7, 6.6 Hz, 36H). ^{13}**C NMR** (101 MHz, $CDCl_3$) δ 161.3 (d, J = 243.5 Hz), 151.9 (d, J = 2.8 Hz), 132.4 (d, J = 3.1 Hz), 132.2 (t, J = 8.1 Hz), 116.9 (d, J = 21.4 Hz), 112.8 (d, J = 23.0 Hz), 70.8, 46.9, 24.4, 23.3, 22.7, 18.8. ^{19}**F NMR** (376 MHz, $CDCl_3$) δ -117.3 (d, J = 2.3 Hz). **HRMS (ESI)** *m/z* Calcd for $C_{35}H_{64}FNNaO_4Si$ $[M+Na]^+$ 632.3935, Found: 632.3954.

2,3-difluorobenzaldehyde O-(tris((2,6-dimethylheptan-4-yl)oxy)silyl) oxime (16a)

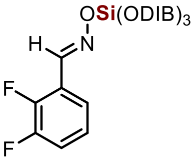

^{1}**H NMR** (400 MHz, $CDCl_3$) δ 8.44 (s, 1H), 7.64 – 7.58 (m, 1H), 7.23 – 7.13 (m, 1H), 7.10 – 7.03 (m, 1H), 4.24 – 4.09 (m, 3H), 1.91 – 1.73 (m, 6H), 1.49 (ddd, J = 13.6, 7.7, 5.9 Hz, 6H), 1.26 (ddd, J = 13.5, 8.1, 5.2 Hz, 6H), 0.88 (dd, J = 8.3, 6.6 Hz, 36H). ^{13}**C NMR** (101 MHz, $CDCl_3$) δ 151.2 (dd, J = 163.8, 13.0 Hz), 148.7 (dd, J = 170.0, 13.1 Hz), 146.9 (t, J = 4.0 Hz), 124.2 (dd, J = 6.8, 4.7 Hz), 122.6 (d, J = 7.5 Hz), 121.7 (dd, J = 9.0, 4.9 Hz), 118.5 (d, J = 16.8 Hz), 70.8, 46.9, 24.4, 23.3, 22.7. ^{19}**F NMR**

(376 MHz, $CDCl_3$) δ -138.0 (d, *J* = 20.1 Hz), -144.5 (d, *J* = 20.1 Hz). **HRMS (ESI)** *m/z* Calcd for $C_{34}H_{62}F_2NO_4Si$ $[M+H]^+$ 644.4411, Found: 614.4402.

2-fluoro-6-methoxybenzaldehyde O-(tris((2,6-dimethylheptan-4-yl)oxy)silyl) oxime (17a)

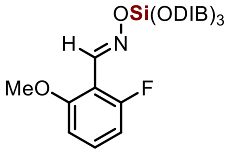

^{1}H NMR (400 MHz, $CDCl_3$) δ 8.45 (s, 1H), 7.25 – 7.20 (m, 1H), 6.68 (dd, *J* = 20.1, 8.7 Hz, 2H), 4.19 – 4.08 (m, 3H), 3.82 (s, 3H), 1.86 – 1.71 (m, 6H), 1.47 (ddd, *J* = 13.5, 7.6, 6.0 Hz, 6H), 1.21 (ddd, *J* = 13.4, 8.0, 5.3 Hz, 6H), 0.84 (dd, *J* = 9.2, 6.6 Hz, 36H). **^{13}C NMR** (101 MHz, $CDCl_3$) δ 161.4 (d, *J* = 257.3 Hz), 159.3 (d, *J* = 7.0 Hz), 147.8 (d, *J* = 4.0 Hz), 131.0 (d, *J* = 10.6 Hz), 109.8 (d, *J* = 14.2 Hz), 108.8 (d, *J* = 22.2 Hz), 106.4 (d, *J* = 3.3 Hz), 70.6, 56.2, 46.8, 24.4, 23.2, 22.7. **^{19}F NMR** (376 MHz, $CDCl_3$) δ -107.7. **HRMS (ESI)** *m/z* Calcd for $C_{35}H_{64}FNNaO_5Si$ $[M+Na]^+$ 648.4430, Found: 648.4455.

2-fluoro-6-(trifluoromethyl)benzaldehyde O-(tris((2,6-dimethylheptan-4-yl)oxy)silyl) oxime (19a)

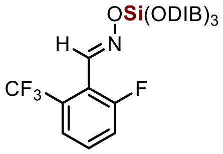

^{1}H NMR (400 MHz, $CDCl_3$) δ 8.45 (d, *J* = 2.4 Hz, 1H), 7.51 (d, *J* = 1.3 Hz, 1H), 7.49 – 7.42 (m, 1H), 7.36 – 7.31 (m, 1H), 4.20 – 4.10 (m, 3H), 1.89 – 1.73 (m, 6H), 1.49 (ddd, *J* = 13.5, 7.6, 6.0 Hz, 6H), 1.25 (ddd, *J* = 13.5, 8.1, 5.2 Hz, 6H), 0.87 (dd, *J* = 8.3, 6.6 Hz, 36H). **^{13}C NMR** (101 MHz, $CDCl_3$) δ 162.2, 159.6, 147.3 (d, *J* = 3.0 Hz), 130.9 (dd, *J* = 31.4, 2.8 Hz), 130.6 (d, *J* = 9.0 Hz), 122.1 – 121.8 (m), 120.4 (d, *J* = 23.2 Hz), 119.0 (dd, *J* = 13.9, 4.3 Hz), 70.8, 46.8, 24.4, 23.2, 22.6. **^{19}F NMR** (376 MHz, $CDCl_3$) δ -58.7, -106.8. **HRMS (ESI)** *m/z* Calcd for $C_{35}H_{61}F_4NNaO_4Si$ $[M+Na]^+$ 686.4198, Found: 686.4201.

2-bromo-6-fluorobenzaldehyde O-(tris((2,6-dimethylheptan-4-yl)oxy)silyl) oxime (20a)

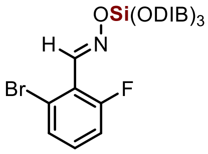

^{1}H NMR (400 MHz, $CDCl_3$) δ 8.47 (s, 1H), 7.41 (dd, *J* = 8.0, 1.2 Hz, 1H), 7.19 (td, *J* = 8.2, 5.5 Hz, 1H), 7.13 – 7.04 (m, 1H), 4.21 – 4.12 (m, 3H), 1.89 – 1.73 (m, 6H), 1.50 (ddd, *J* = 13.5, 7.6, 6.0 Hz, 6H), 1.25 (ddd, *J* = 13.4, 8.1, 5.3 Hz, 6H), 0.87 (dd, *J* = 8.7, 6.6 Hz, 36H). **^{13}C NMR** (101 MHz, $CDCl_3$) δ 161.0 (d, *J* = 261.1 Hz), 150.6 (d, *J* = 4.8 Hz), 131.2 (d, *J* = 9.2 Hz), 129.0 (d, *J* = 3.7 Hz), 124.6 (d, *J* = 4.0 Hz), 120.9 (d, *J* = 14.7 Hz), 115.7 (d, *J* = 22.4 Hz), 70.8, 46.8, 24.4, 23.2, 22.7. **^{19}F NMR** (376 MHz, $CDCl_3$) δ -105.7. **HRMS (ESI)** *m/z* Calcd for $C_{34}H_{61}BrFNNaO_4Si$ $[M+Na]^+$ 696.3429, Found: 696.3437.

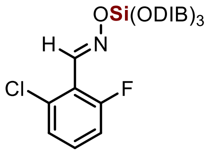

2-chloro-6-fluorobenzaldehyde O-(tris((2,6-dimethylheptan-4-yl)oxy)silyl) oxime (21a)

^{1}H NMR (400 MHz, $CDCl_3$) δ 8.49 (s, 1H), 7.25 – 7.17 (m, 2H), 7.01 (ddd, *J* = 9.7, 7.9, 1.6 Hz, 1H), 4.22 – 4.11 (m, 3H), 1.88 – 1.70 (m, 6H), 1.48 (ddd, *J* = 13.5, 7.6, 6.0 Hz, 6H), 1.23 (ddd, *J* = 13.4, 8.0, 5.2 Hz, 6H), 0.85 (dd, *J* = 8.8, 6.6 Hz, 36H). **^{13}C NMR** (101 MHz, $CDCl_3$) δ 161.2 (d, *J* = 260.1 Hz), 148.6 (d, *J* = 4.1 Hz), 135.2 (d, *J* = 4.6 Hz), 130.8 (d, *J* = 9.5 Hz), 125.8 (d, *J* = 3.7 Hz), 119.4 (d, *J* = 14.8 Hz), 115.1

(d, J = 22.3 Hz), 70.8, 46.9, 24.4, 23.3, 22.7. **^{19}F NMR** (376 MHz, $CDCl_3$) δ -106.7. **HRMS (ESI)** *m/z* Calcd for $C_{34}H_{61}ClFNNaO_4Si$ $[M+Na]^+$ 652.3935, Found: 652.3921.

2-bromo-6-chlorobenzaldehyde O-(tris((2,6-dimethylheptan-4-yl)oxy)silyl) oxime (22a)

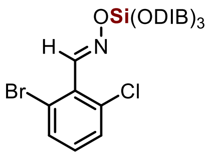

^{1}H NMR (400 MHz, $CDCl_3$) δ 8.40 (s, 1H), 7.53 (dd, J = 8.0, 1.1 Hz, 1H), 7.39 (dd, J = 8.0, 1.1 Hz, 1H), 7.13 (t, J = 8.1 Hz, 1H), 4.22 – 4.11 (m, 3H), 1.88 – 1.74 (m, 6H), 1.50 (ddd, J = 13.5, 7.6, 6.0 Hz, 6H), 1.26 (ddd, J = 13.4, 8.0, 5.3 Hz, 6H), 0.87 (dd, J = 9.8, 6.6 Hz, 36H). **^{13}C NMR** (101 MHz, $CDCl_3$) δ 152.0, 135.1, 131.8, 130.9, 130.6, 129.4, 124.5, 70.8, 46.9, 24.4, 23.3, 22.8. **HRMS (ESI)** *m/z* Calcd for $C_{34}H_{61}BrClNNaO_4Si$ $[M+Na]^+$ 712.3134, Found: 712.3146.

2,6-dibromobenzaldehyde O-(tris((2,6-dimethylheptan-4-yl)oxy)silyl) oxime (23a)

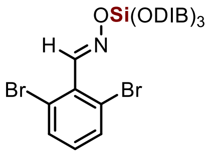

^{1}H NMR (400 MHz, $CDCl_3$) δ 8.34 (s, 1H), 7.58 (d, J = 8.1 Hz, 2H), 7.06 (t, J = 8.0 Hz, 1H), 4.31 – 4.07 (m, 3H), 1.92 – 1.73 (m, 6H), 1.62 – 1.42 (m, 6H), 1.37 – 1.18 (m, 6H), 0.87 (dd, J = 10.1, 6.6 Hz, 36H). **^{13}C NMR** (101 MHz, $CDCl_3$) δ 153.3, 132.5, 131.0, 124.2, 70.8, 46.8, 24.4, 23.3, 22.8. **HRMS (ESI)** *m/z* Calcd for $C_{34}H_{61}Br_2NNaO_4Si$ $[M+Na]^+$ 756.2629, Found: 756.2618.

2,6-dichlorobenzaldehyde O-(tris((2,6-dimethylheptan-4-yl)oxy)silyl) oxime (24a)

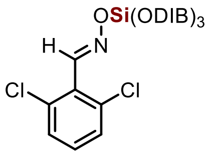

^{1}H NMR (400 MHz, $CDCl_3$) δ 8.46 (s, 1H), 7.34 (d, J = 8.1 Hz, 2H), 7.21 (dd, J = 8.7, 7.4 Hz, 1H), 4.23 – 4.05 (m, 3H), 1.94 – 1.73 (m, 6H), 1.55 – 1.44 (m, 6H), 1.40 – 1.19 (m, 6H), 0.87 (dd, J = 9.5, 6.6 Hz, 36H). **^{13}C NMR** (101 MHz, $CDCl_3$) δ 150.5, 135.3, 130.3, 129.2, 128.7, 70.8, 46.8, 24.4, 23.3, 22.7. **HRMS (ESI)** *m/z* Calcd for $C_{34}H_{62}Cl_2NO_4Si$ $[M+H]^+$ 646.3820, Found: 646.3836.

1-(2-ethoxyphenyl)ethan-1-one O-(tris((2,6-dimethylheptan-4-yl)oxy)silyl) oxime (25a)

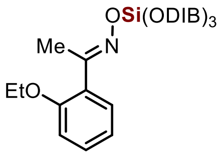

^{1}H NMR (400 MHz, $CDCl_3$) δ 7.37 (dd, J = 7.5, 1.8 Hz, 1H), 7.29 (td, J = 7.8, 1.8 Hz, 1H), 6.92 – 6.85 (m, 2H), 4.20 – 4.09 (m, 3H), 4.04 (q, J = 7.0 Hz, 2H), 2.24 (s, 3H), 1.83 (ddd, J = 12.5, 7.9, 6.3 Hz, 6H), 1.50 (ddd, J = 13.5, 7.6, 5.9 Hz, 6H), 1.40 (t, J = 7.0 Hz, 3H), 1.24 (ddd, J = 13.4, 8.0, 5.1 Hz, 6H), 0.86 (dd, J = 9.3, 6.6 Hz, 36H). **^{13}C NMR** (101 MHz, $CDCl_3$) δ 161.43, 157.12, 130.09, 130.05, 127.39, 120.17, 111.71, 70.47, 63.72, 46.96, 24.41, 23.28, 22.73, 16.18, 14.91. **HRMS (ESI)** *m/z* Calcd for $C_{37}H_{69}NNaO_5Si$ $[M+Na]^+$ 658.4837, Found: 658.4855.

1-(2-chlorophenyl)ethan-1-one O-(tris((2,6-dimethylheptan-4-yl)oxy)silyl) oxime (26a)

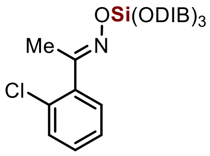

^{1}H NMR (400 MHz, $CDCl_3$) δ 7.41 – 7.31 (m, 2H), 7.33 – 7.19 (m, 2H), 4.20 – 4.09 (m, 3H), 2.27 (s, 3H), 1.93 – 1.74 (m, 6H), 1.55 – 1.43 (m, 6H), 1.30 – 1.18 (m, 6H), 0.86 (dd, J = 9.3, 6.6 Hz, 36H). **^{13}C NMR** (101 MHz, $CDCl_3$) δ 160.8, 137.2, 132.8, 130.6, 129.9, 126.6, 70.6, 46.9, 24.4, 23.3, 22.7, 16.6. **HRMS (ESI)** *m/z* Calcd for $C_{35}H_{64}ClNNaO_4Si$ $[M+Na]^+$ 648.4185, Found: 648.4202.

1-(2-bromophenyl)ethan-1-one O-(tris((2,6-dimethylheptan-4-yl)oxy)silyl) oxime (27a)

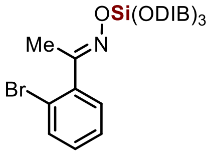

^{1}H NMR (400 MHz, $CDCl_3$) δ 7.61 – 7.50 (m, 1H), 7.34 – 7.01 (m, 3H), 4.19 – 3.97 (m, 3H), 2.23 (d, J = 31.9 Hz, 3H), 1.88 – 1.68 (m, 6H), 1.53 – 1.35 (m, 6H), 1.28 – 1.11 (m, 6H), 0.86 (dd, J = 9.5, 6.6 Hz, 36H). **^{13}C NMR** (101 MHz, $CDCl_3$) δ 161.7, 139.3, 133.1, 130.8, 130.0, 127.1, 121.9, 70.6, 46.9, 24.4, 23.3, 22.7, 16.8. **HRMS (ESI)** *m/z* Calcd for $C_{35}H_{64}BrNNaO_4Si$ $[M+Na]^+$ 692.3680, Found: 692.3692.

2,3-dihydro-1H-inden-1-one O-(tris((2,6-dimethylheptan-4-yl)oxy)silyl) oxime (29a)

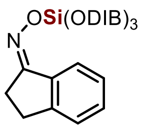

^{1}H NMR (400 MHz, $CDCl_3$) δ 7.67 (d, J = 7.7 Hz, 1H), 7.41 – 7.26 (m, 2H), 7.28 – 7.19 (m, 1H), 4.24 – 4.10 (m, 3H), 3.06 – 3.00 (m, 2H), 2.98 – 2.90 (m, 2H), 1.91 – 1.77 (m, 6H), 1.50 (ddd, J = 13.5, 7.6, 5.9 Hz, 6H), 1.24 (ddd, J = 13.4, 8.1, 5.1 Hz, 6H), 0.87 (dd, J = 9.0, 6.6 Hz, 36H). **^{13}C NMR** (101 MHz, $CDCl_3$) δ 167.4, 148.7, 136.5, 130.4, 126.9, 125.6, 122.1, 70.5, 47.0, 28.5, 26.8, 24.4, 23.3, 22.8. **HRMS (ESI)** *m/z* Calcd for $C_{36}H_{65}NNaO_4Si$ $[M+Na]^+$ 626.4575, Found: 626.4579.

3,4-dihydronaphthalen-1(2H)-one O-(tris((2,6-dimethylheptan-4-yl)oxy)silyl) oxime (30a)

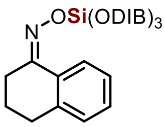

^{1}H NMR (400 MHz, $CDCl_3$) δ 8.05 (d, J = 7.9 Hz, 1H), 7.26 (td, J = 7.3, 1.4 Hz, 1H), 7.21 – 7.11 (m, 2H), 4.22 – 4.12 (m, 3H), 2.83 (t, J = 6.7 Hz, 2H), 2.76 (t, J = 6.0 Hz, 2H), 1.91 – 1.79 (m, 8H), 1.57 – 1.47 (m, 6H), 1.26 (ddd, J = 13.4, 8.1, 5.2 Hz, 8H), 0.88 (dd, J = 7.9, 6.6 Hz, 36H). **^{13}C NMR** (101 MHz, $CDCl_3$) δ 158.9, 139.8, 131.0, 129.2, 128.5, 126.1, 125.0, 70.6, 46.9, 30.1, 24.7, 24.4, 23.3, 22.8, 21.7. **HRMS (ESI)** *m/z* Calcd for $C_{37}H_{67}NNaO_4Si$ $[M+Na]^+$ 640.4732, Found: 640.4750.

4-methyl-3,4-dihydronaphthalen-1(2H)-one O-(tris((2,6-dimethylheptan-4-yl)oxy)silyl) oxime (31a)

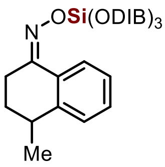

^{1}H NMR (400 MHz, $CDCl_3$) δ 8.04 (dd, J = 7.9, 1.4 Hz, 1H), 7.30 (td, J = 7.4, 1.4 Hz, 1H), 7.22 (dd, J = 7.9, 1.4 Hz, 1H), 7.16 (td, J = 7.5, 7.1, 1.5 Hz, 1H), 4.22 – 4.11 (m, 3H), 2.97 – 2.81 (m, 3H), 2.00 – 1.89 (m, 1H), 1.91 – 1.77 (m, 6H), 1.67 (dq, J = 13.0, 6.5 Hz, 1H), 1.51 (ddd, J = 13.6, 7.4, 6.2 Hz, 6H), 1.32 – 1.20 (m, 9H), 0.87 (dd, J = 8.0, 6.6 Hz, 36H). **^{13}C NMR** (101 MHz, $CDCl_3$) δ 158.8, 144.5,

130.3, 129.4, 127.0, 126.0, 125.1, 70.6, 46.9, 33.1, 28.7, 24.4, 23.3, 22.8, 21.8, 20.7. **HRMS (ESI)** *m/z* Calcd for $C_{38}H_{69}NNaO_4Si$ $[M+Na]^+$ 654.4888, Found: 654.4901.

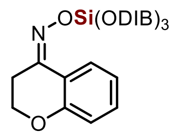

Chroman-4-one O-(tris((2,6-dimethylheptan-4-yl)oxy)silyl) oxime (32a)

^{1}H NMR (400 MHz, $CDCl_3$) δ 7.98 (dd, *J* = 7.9, 1.7 Hz, 1H), 7.28 (ddd, *J* = 8.5, 7.2, 1.7 Hz, 1H), 6.99 – 6.89 (m, 2H), 4.25 (t, *J* = 6.2 Hz, 2H), 4.23 – 4.14 (m, 1H), 3.01 (t, *J* = 6.2 Hz, 2H), 1.94 – 1.79 (m, 6H), 1.53 (ddd, *J* = 13.6, 7.6, 5.9 Hz, 6H), 1.29 (ddd, *J* = 13.4, 8.1, 5.1 Hz, 6H), 0.96 – 0.85 (m, 36H). **^{13}C NMR** (101 MHz, $CDCl_3$) δ 156.8, 153.2, 131.2, 124.9, 121.3, 118.8, 117.6, 70.7, 65.3, 46.9, 24.4, 23.3, 22.7. **HRMS (ESI)** *m/z* Calcd for $C_{36}H_{65}NNaO_5Si$ $[M+Na]^+$ 642.4254, Found: 642.4265.

6,7,8,9-tetrahydro-5H-benzo[7]annulen-5-one O-(tris((2,6-dimethylheptan-4-yl)oxy)silyl) oxime (33a)

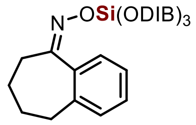

^{1}H NMR (400 MHz, $CDCl_3$) δ 7.48 (dd, *J* = 7.5, 1.5 Hz, 1H), 7.36 – 7.24 (m, 1H), 7.19 (td, *J* = 7.5, 1.4 Hz, 1H), 7.12 (dd, *J* = 7.3, 1.4 Hz, 1H), 4.21 – 4.12 (m, 3H), 2.80 – 2.71 (m, 4H), 1.93 – 1.73 (m, 8H), 1.66 (p, *J* = 6.4 Hz, 2H), 1.51 (ddd, *J* = 13.5, 7.7, 5.9 Hz, 6H), 1.25 (ddd, *J* = 13.4, 8.1, 5.1 Hz, 6H), 0.87 (dd, *J* = 8.9, 6.6 Hz, 36H). **^{13}C NMR** (101 MHz, $CDCl_3$) δ 166.4, 139.6, 136.6, 129.1, 128.8, 128.2, 126.2, 70.5, 46.9, 32.3, 26.9, 26.3, 24.4, 23.3, 22.7, 22.1. **HRMS (ESI)** *m/z* Calcd for $C_{38}H_{69}NNaO_4Si$ $[M+Na]^+$ 654.4888, Found: 654.4906.

1-(2-chloro-6-fluorophenyl)ethan-1-one O-(tris((2,6-dimethylheptan-4-yl)oxy)silyl) oxime (34a)

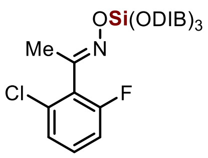

^{1}H NMR (400 MHz, $CDCl_3$) δ 7.27 – 7.13 (m, 2H), 6.98 (td, *J* = 8.4, 1.3 Hz, 1H), 4.17 – 4.06 (m, 3H), 2.19 (s, 3H), 1.85 – 1.69 (m, 6H), 1.52 – 1.39 (m, 6H), 1.27 – 1.16 (m, 6H), 0.83 (dd, *J* = 9.7, 6.6 Hz, 36H). **^{13}C NMR** (101 MHz, $CDCl_3$) δ 160.7 (d, *J* = 250.7 Hz), 154.9, 134.5 (d, *J* = 4.9 Hz), 130.2 (d, *J* = 9.4 Hz), 126.0 (d, *J* = 19.7 Hz), 125.2 (d, *J* = 3.2 Hz), 114.2 (d, *J* = 22.4 Hz), 70.7, 46.9, 24.4, 23.2, 22.7, 16.2. **^{19}F NMR** (376 MHz, $CDCl_3$) δ -111.6. **HRMS (ESI)** *m/z* Calcd for $C_{35}H_{64}ClFNO_4Si$ $[M+H]^+$ 644.4272, Found: 644.4260.

1-(2-fluoro-6-methoxyphenyl)ethan-1-one O-(tris((2,6-dimethylheptan-4-yl)oxy)silyl) oxime (35a)

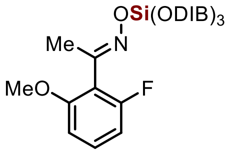

^{1}H NMR (400 MHz, $CDCl_3$) δ 7.33 – 7.27 (m, 1H), 6.88 – 6.53 (m, 2H), 4.24 – 4.13 (m, 3H), 3.83 (s, 3H), 2.21 (s, 3H), 1.93 – 1.78 (m, 6H), 1.53 (ddd, *J* = 13.5, 7.6, 5.9 Hz, 6H), 1.28 (ddd, *J* = 13.4, 8.1, 5.3 Hz, 6H), 0.90 (dd, *J* = 9.4, 6.6 Hz, 36H). **^{13}C NMR** (101 MHz, $CDCl_3$) δ 160.7 (d, *J* = 247.6 Hz), 158.9 (d, *J* = 6.9 Hz), 154.7, 129.9 (d, *J* = 10.4 Hz), 115.7 (d, *J* = 18.8 Hz), 108.2 (d, *J* =

22.5 Hz), 106.2 (d, J = 2.9 Hz), 70.5, 55.9, 46.9, 24.4, 23.2, 22.7, 16.4. **^{19}F NMR** (376 MHz, $CDCl_3$) δ -114.2. **HRMS (ESI)** *m/z* Calcd for $C_{36}H_{66}FNNaO_5Si$ $[M+Na]^+$ 662.4587, Found: 662.4609.

1-(2-bromo-5-fluorophenyl)ethan-1-one O-(tris((2,6-dimethylheptan-4-yl)oxy)silyl) oxime (36a)

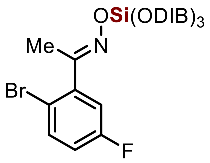

^{1}H NMR (400 MHz, $CDCl_3$) δ 7.52 (dd, J = 8.8, 5.1 Hz, 1H), 7.06 (dd, J = 8.9, 3.1 Hz, 1H), 6.95 (td, J = 8.3, 3.1 Hz, 1H), 4.21 – 4.02 (m, 3H), 2.27 (s, 3H), 1.89 – 1.72 (m, 6H), 1.49 (ddd, J = 13.6, 7.7, 5.9 Hz, 6H), 1.25 (ddd, J = 13.5, 8.1, 5.2 Hz, 6H), 0.87 (dd, J = 9.0, 6.6 Hz, 36H). **^{13}C NMR** (101 MHz, $CDCl_3$) δ 161.53 (d, J = 247.9 Hz), 160.7, 140.7 (d, J = 7.8 Hz), 134.2 (d, J = 7.9 Hz), 117.9 (d, J = 23.5 Hz), 117.0 (d, J = 22.3 Hz), 115.9 (d, J = 3.4 Hz), 70.5, 46.8, 24.3, 23.1, 22.6, 16.4. **^{19}F NMR** (376 MHz, $CDCl_3$) δ -115.2. **HRMS (ESI)** *m/z* Calcd for $C_{35}H_{63}BrFNNaO_4Si$ $[M+Na]^+$ 710.3586, Found: 710.3591.

1-(2-chloro-5-fluorophenyl)ethan-1-one O-(tris((2,6-dimethylheptan-4-yl)oxy)silyl) oxime (37a)

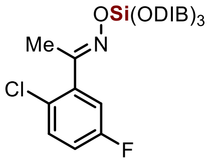

^{1}H NMR (400 MHz, $CDCl_3$) δ 7.33 (dd, J = 8.8, 5.0 Hz, 1H), 7.09 (dd, J = 8.8, 3.1 Hz, 1H), 7.01 (ddd, J = 8.8, 7.7, 3.1 Hz, 1H), 4.18 – 4.08 (m, 3H), 2.27 (s, 3H), 1.88 – 1.71 (m, 6H), 1.48 (ddd, J = 13.6, 7.6, 5.9 Hz, 6H), 1.24 (ddd, J = 13.4, 8.1, 5.2 Hz, 6H), 0.86 (dd, J = 8.5, 6.6 Hz, 36H). **^{13}C NMR** (101 MHz, $CDCl_3$) δ161.1 (d, J = 247.1 Hz), 160.0, 138.7 (d, J = 8.0 Hz), 131.2 (d, J = 8.2 Hz), 127.8 (d, J = 3.4 Hz), 117.7 (d, J = 23.9 Hz), 116.9 (d, J = 22.7 Hz), 70.7, 46.9, 24.4, 23.3, 22.7, 16.3. **^{19}F NMR** (376 MHz, $CDCl_3$) δ -115.9. **HRMS (ESI)** *m/z* Calcd for $C_{35}H_{63}ClFNNaO_4Si$ $[M+Na]^+$ 666.4091, Found: 666.4079.

3.5 General procedure D for the preparation of bulky trialkoxysilane derivatives of benzoic acid (GP5)

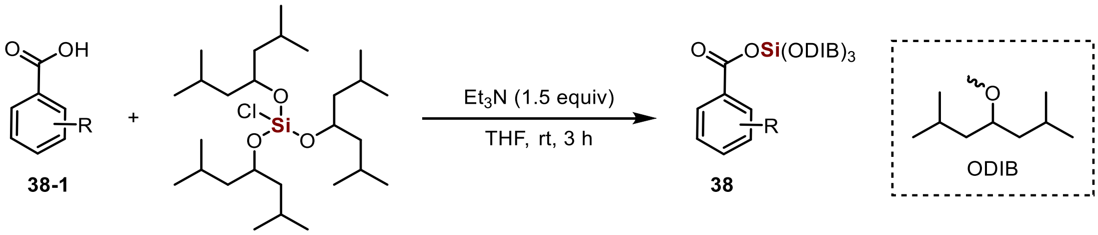

To a stirred solution of the corresponding benzoic acid (5 mmol, 1.0 equiv) and Et_3N (7.5 mmol, 1.5 equiv) was added dropwise a solution of chlorotris((2,6-dimethylheptan-4-yl)oxy)silane (7.5 mmol, 1.5 equiv) in CH_2Cl_2 (10 mL) at room temperature. The mixture was stirred at room temperature for 3 h. The white precipitate was removed by filtration under nitrogen. Water 10 mL was added and the product was extracted into CH_2Cl_2, washed with brine, dried over Na_2SO_4 and concentrated. The crude product was purified (1% ethyl acetate in hexane as eluent) by silica gel chromatography as a colorless oil.[4]

2-chlorobenzoic (tris(2,6-dimethylheptan-4-yl) silicic) anhydride (38a)

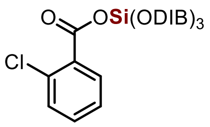

^{1}H NMR (400 MHz, $CDCl_3$) δ 7.84 (dd, *J* = 7.8, 1.6 Hz, 1H), 7.49 – 7.36 (m, 2H), 7.30 (td, *J* = 7.4, 1.5 Hz, 1H), 4.35 – 4.15 (m, 3H), 1.84 – 1.71 (m, 6H), 1.55 – 1.44 (m, 6H), 1.34 – 1.23 (m, 6H), 0.88 (t, *J* = 6.2 Hz, 36H). **^{13}C NMR** (101 MHz, $CDCl_3$) δ 163.5, 134.1, 132.6, 131.8, 131.4, 131.2, 126.5, 71.3, 46.7, 24.5, 23.2, 22.8. **HRMS (ESI)** *m/z* Calcd for $C_{34}H_{61}ClNaO_5Si$ $[M+Na]^+$ 635.3869, Found: 635.3877.

2-bromobenzoic (tris(2,6-dimethylheptan-4-yl) silicic) anhydride (38b)

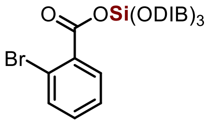

^{1}H NMR (400 MHz, $CDCl_3$) δ 7.82 (dd, *J* = 7.5, 2.1 Hz, 1H), 7.66 (dd, *J* = 7.7, 1.6 Hz, 1H), 7.36 – 7.28 (m, 2H), 4.30 – 4.19 (m, 3H), 1.86 – 1.71 (m, 6H), 1.56 – 1.44 (m, 6H), 1.41 – 1.23 (m, 6H), 0.99 – 0.77 (m, 36H). **^{13}C NMR** (101 MHz, $CDCl_3$) δ 163.8, 134.6, 133.3, 132.6, 131.7, 127.1, 122.2, 71.3, 46.7, 24.5, 23.2, 22.8. **HRMS (ESI)** *m/z* Calcd for $C_{34}H_{61}BrNaO_5Si$ $[M+Na]^+$ 679.3364, Found: 679.3382.

2-(trifluoromethoxy)benzoic (tris(2,6-dimethylheptan-4-yl) silicic) anhydride (38c)

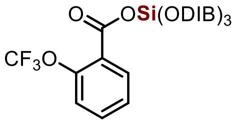

^{1}H NMR (400 MHz, $CDCl_3$) δ 7.94 (dd, *J* = 7.8, 1.8 Hz, 1H), 7.55 (td, *J* = 7.8, 1.8 Hz, 1H), 7.41 – 7.30 (m, 2H), 4.33 – 4.14 (m, 3H), 1.92 – 1.67 (m, 6H), 1.55 – 1.43 (m, 6H), 1.34 – 1.23 (m, 6H), 0.88 (dd, *J* = 6.7, 5.7 Hz, 36H). **^{13}C NMR** (101 MHz, $CDCl_3$) δ 162.0, 148.3 (d, *J* = 1.9 Hz), 133.5, 132.3, 126.8, 126.2, 122.4, 120.5 (d, *J* = 258.0 Hz), 71.3, 46.7, 24.5, 23.2, 22.7. **^{19}F NMR** (376 MHz, $CDCl_3$) δ -57.2. **HRMS (ESI)** *m/z* Calcd for $C_{35}H_{61}F_3NaO_6Si$ $[M+Na]^+$ 685.4082, Found: 685.4101.

2-(trifluoromethyl)benzoic (tris(2,6-dimethylheptan-4-yl) silicic) anhydride (38d)

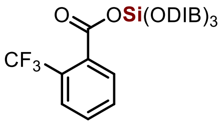

^{1}H NMR (400 MHz, $CDCl_3$) δ 7.94 (dd, *J* = 7.8, 1.8 Hz, 1H), 7.55 (td, *J* = 7.8, 1.8 Hz, 1H), 7.40 – 7.30 (m, 2H), 4.35 – 4.17 (m, 3H), 1.86 – 1.71 (m, 6H), 1.55 – 1.44 (m, 6H), 1.38 – 1.24 (m, 6H), 0.88 (dd, *J* = 6.9, 5.6 Hz, 36H). **^{13}C NMR** (101 MHz, $CDCl_3$) δ 162.00, 148.26 (d, *J* = 1.9 Hz), 133.48, 132.32, 126.76, 126.24, 122.43, 120.55 (d, *J* = 257.9 Hz), 71.35, 46.66, 24.50, 23.15, 22.74. **^{19}F NMR** (376 MHz, $CDCl_3$) δ -57.2. **HRMS (ESI)** *m/z* Calcd for $C_{35}H_{61}F_3NaO_5Si$ $[M+Na]^+$ 669.4133, Found: 669.4121.

2-methylbenzoic (tris(2,6-dimethylheptan-4-yl) silicic) anhydride (38e)

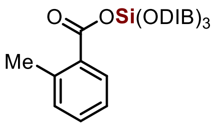

^{1}H NMR (400 MHz, $CDCl_3$) δ 7.98 (d, *J* = 8.0 Hz, 1H), 7.41 (td, *J* = 7.5, 1.5 Hz, 1H), 7.31 – 7.20 (m, 2H), 4.33 – 4.20 (m, 3H), 2.65 (s, 3H), 2.02 – 1.73 (m, 6H), 1.56 – 1.46 (m, 6H), 1.30 (ddd, *J* = 13.4, 8.0, 5.4 Hz, 6H), 1.09 – 0.75 (m, 36H). **^{13}C NMR** (101 MHz, $CDCl_3$) δ 165.3,

141.4, 132.2, 131.9, 131.5, 130.3, 125.6, 71.1, 46.8, 24.5, 23.2, 22.8, 22.0. **HRMS (ESI)** *m/z* Calcd for $C_{35}H_{64}NaO_5Si$ $[M+Na]^+$ 615.4415, Found: 615.4421.

2,3-difluorobenzoic (tris(2,6-dimethylheptan-4-yl) silicic) anhydride (38f)

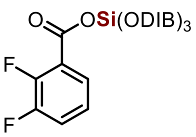

^{1}H NMR (400 MHz, $CDCl_3$) δ 7.69 (td, *J* = 7.8, 1.7 Hz, 1H), 7.42 – 7.29 (m, 1H), 7.20 – 7.07 (m, 1H), 4.31 – 4.18 (m, 3H), 1.89 – 1.70 (m, 6H), 1.49 (ddd, *J* = 13.6, 7.5, 6.0 Hz, 6H), 1.28 (ddd, *J* = 13.4, 8.0, 5.3 Hz, 6H), 0.88 (dd, *J* = 6.9, 5.5 Hz, 36H). **^{13}C NMR** (101 MHz, $CDCl_3$) δ 161.3, 152.3 (dd, *J* = 55.7, 13.3 Hz), 149.8 (dd, *J* = 69.4, 13.3 Hz), 127.2 (d, *J* = 3.6 Hz), 123.7 (dd, *J* = 6.6, 5.2 Hz), 122.2 (d, *J* = 6.6 Hz), 121.5 (d, *J* = 17.4 Hz), 71.4, 46.7, 24.5, 23.2, 22.7. **^{19}F NMR** (376 MHz, $CDCl_3$) δ -135.2, -137.0. **HRMS (ESI)** *m/z* Calcd for $C_{34}H_{60}F_2NaO_5Si$ $[M+Na]^+$ 637.4070, Found: 637.4084.

3.6 General procedure E for the preparation of bulky trialkoxysilane derivatives of benzyl alcohol (GP6)

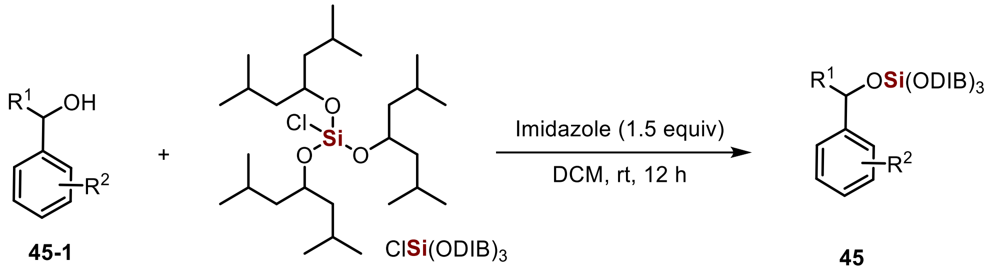

45-1 **45**

To a stirred solution of the corresponding benzyl alcohol (5 mmol, 1.0 equiv) and trialkoxychlorosilane (7.5 mmol, 1.5 equiv) in CH_2Cl_2 (10 mL) was added dropwise a solution of imidazole (7.5 mmol, 1.5 equiv) in CH_2Cl_2 (10 mL) at room temperature. The mixture was stirred at room temperature for 12 h. The white precipitate was removed by filtration under nitrogen. Water 20 mL was added and the product was extracted into CH_2Cl_2, washed with brine, dried over Na_2SO_4 and concentrated. The crude product was purified (5% ethyl acetate in hexane as eluent) by silica gel chromatography as a colorless oil.[5]

2-chlorobenzyl tris(2,6-dimethylheptan-4-yl) silicate (45a)

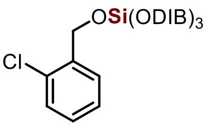

^{1}H NMR (400 MHz, $CDCl_3$) δ 7.58 (dd, *J* = 7.7, 1.7 Hz, 1H), 7.31 – 7.18 (m, 2H), 7.14 (td, *J* = 7.6, 1.8 Hz, 1H), 4.91 (s, 2H), 4.14 – 3.99 (m, 3H), 1.85 – 1.67 (m, 6H), 1.43 (ddd, *J* = 13.6, 7.5, 6.1 Hz, 6H), 1.23 (ddd, *J* = 13.4, 8.0, 5.4 Hz, 6H), 0.85 (dd, *J* = 8.3, 6.6 Hz, 36H). **^{13}C NMR** (101 MHz, $CDCl_3$) δ 138.6, 131.6, 128.9, 127.9, 127.8, 126.6, 70.4, 62.6, 47.0, 24.6, 23.2, 22.8. **HRMS (ESI)** *m/z* Calcd for $C_{34}H_{63}ClNaO_4Si$ $[M+Na]^+$ 621.4076, Found: 621.4093.

2-bromobenzyl tris(2,6-dimethylheptan-4-yl) silicate (45b)

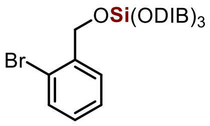

^{1}H NMR (400 MHz, $CDCl_3$) δ 7.62 (dd, *J* = 7.8, 1.6 Hz, 1H), 7.50 (dd, *J* = 8.0, 1.2 Hz, 1H), 7.32 (td, *J* = 7.5, 1.2 Hz, 1H), 7.12 (td, *J* = 7.6, 1.7 Hz, 1H), 4.90 (s, 2H), 4.30 – 4.06 (m, 3H), 1.86 – 1.73 (m, 6H), 1.47 (ddd, *J* = 13.6, 7.5, 6.0 Hz, 6H), 1.28 (ddd, *J* = 13.4, 7.9, 5.3 Hz, 6H), 0.89 (dd, *J* = 8.3, 6.6 Hz, 36H). **^{13}C NMR** (101 MHz, $CDCl_3$) δ 140.0, 132.1, 128.2, 127.9, 127.2, 121.2, 70.4, 64.9, 46.9, 24.6, 23.2, 22.8. **HRMS (ESI)** *m/z* Calcd for $C_{34}H_{63}BrNaO_4Si$ $[M+Na]^+$ 665.3571, Found: 665.3565.

Tris(2,6-dimethylheptan-4-yl) (2-(trifluoromethyl)benzyl) silicate (45c)

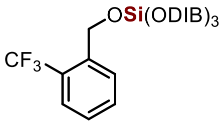

^{1}H NMR (400 MHz, $CDCl_3$) δ 7.87 (d, *J* = 7.8 Hz, 1H), 7.61 (d, *J* = 7.8 Hz, 1H), 7.55 (t, *J* = 7.6 Hz, 1H), 7.34 (t, *J* = 7.6 Hz, 1H), 5.10 (s, 2H), 4.40 – 3.87 (m, 3H), 1.88 – 1.72 (m, 6H), 1.54 – 1.42 (m, 6H), 1.28 (ddd, *J* = 18.9, 6.4, 1.8 Hz, 6H), 0.89 (dd, *J* = 10.2, 6.5 Hz, 36H). **^{13}C NMR** (101 MHz, $CDCl_3$) δ 139.8, 131.8, 127.6, 126.7, 126.2 (d, *J* = 33.0 Hz), 125.5 (q, *J* = 5.8 Hz), 123.3, 70.4, 61.3 (q, *J* = 3.6 Hz), 47.0, 24.6, 23.2, 22.7. **^{19}F NMR** (376 MHz, $CDCl_3$) δ -61.0. **HRMS (ESI)** *m/z* Calcd for $C_{35}H_{63}F_3NaO_4Si$ $[M+Na]^+$ 655.4340, Found: 655.4349.

1-(2-chlorophenyl)ethyl tris(2,6-dimethylheptan-4-yl) silicate (45d)

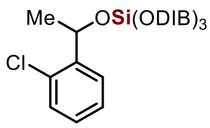

^{1}H NMR (400 MHz, $CDCl_3$) δ 7.66 (dd, *J* = 7.8, 1.8 Hz, 1H), 7.26 (ddd, *J* = 14.3, 7.7, 1.4 Hz, 2H), 7.15 (td, *J* = 7.6, 1.8 Hz, 1H), 5.49 (q, *J* = 6.2 Hz, 1H), 4.19 – 3.95 (m, 3H), 1.85 – 1.65 (m, 6H), 1.50 – 1.34 (m, 9H), 1.30 – 1.16 (m, 6H), 0.94 – 0.80 (m, 36H). **^{13}C NMR** (101 MHz, $CDCl_3$) δ 144.1, 131.0, 129.0, 127.8, 127.2, 126.9, 70.2, 68.0, 46.9, 46.8, 25.2, 24.5, 23.2, 23.1, 22.9, 22.7. **HRMS (ESI)** *m/z* Calcd for $C_{35}H_{65}ClNaO_4Si$ $[M+Na]^+$ 635.4233, Found: 635.4245.

Methyl 2-(2-bromophenyl)-2-((tris((2,6-dimethylheptan-4-yl)oxy)silyl)oxy)acetate (45e)

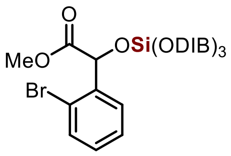

^{1}H NMR (400 MHz, $CDCl_3$) δ 7.63 (dd, *J* = 7.8, 1.7 Hz, 1H), 7.52 (dd, *J* = 8.1, 1.2 Hz, 1H), 7.30 (td, *J* = 7.6, 1.3 Hz, 1H), 7.14 (td, *J* = 7.7, 1.8 Hz, 1H), 5.89 (s, 1H), 4.13 – 4.04 (m, 3H), 3.67 (s, 3H), 1.80 – 1.62 (m, 6H), 1.40 (dddd, *J* = 24.8, 13.5, 7.4, 6.2 Hz, 6H), 1.22 (dddd, *J* = 13.5, 11.4, 7.9, 5.4 Hz, 6H), 0.93 – 0.73 (m, 36H). **^{13}C NMR** (101 MHz, $CDCl_3$) δ 171.4, 138.8, 132.7, 129.6, 129.4, 127.6, 122.9, 73.1, 70.5, 52.1, 46.7 (d, *J* = 7.3 Hz), 24.5 (d, *J* = 2.0 Hz), 23.2 (d, *J* = 2.3 Hz), 22.8 (d, *J* = 13.6 Hz). **HRMS (ESI)** *m/z* Calcd for $C_{36}H_{65}BrNaO_6Si$ $[M+Na]^+$ 723.3626, Found: 723.3653.

2,3-difluorobenzyl tris(2,6-dimethylheptan-4-yl) silicate (45f)

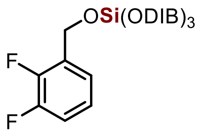

^{1}H NMR (400 MHz, $CDCl_3$) δ 7.32 – 7.24 (m, 1H), 7.11 – 6.98 (m, 2H), 4.93 (s, 2H), 4.13 – 4.02 (m, 3H), 1.82 – 1.70 (m, 6H), 1.50 – 1.39 (m, 6H), 1.26 (ddd, *J* = 13.5, 7.9, 5.4 Hz, 6H), 0.90 – 0.83 (m, 1H). **^{13}C NMR** (101 MHz, $CDCl_3$) δ 150.4 (dd, *J* = 247.0, 12.4 Hz), 146.7 (d, *J* = 12.9 Hz), 130.6 (d, *J* = 11.1 Hz), 123.8 (dd, *J* = 6.8, 4.6 Hz), 123.6 (t, *J* = 3.4 Hz), 115.7 (d, *J* = 17.1 Hz), 70.4, 58.8 (dd, *J* = 5.1, 3.1 Hz), 46.9, 24.6, 23.2, 22.7. **^{19}F NMR** (377 MHz, $CDCl_3$) δ -140.0 (d, *J* = 20.9 Hz), -145.1 (d, *J* = 21.1 Hz). **HRMS (ESI)** *m/z* Calcd for $C_{34}H_{62}F_2NaO_4Si$ $[M+Na]^+$ 623.4278, Found: 623.4283.

3.7 General procedure F for the preparation of bulky trialkoxysilane derivatives of phenol (GP7)

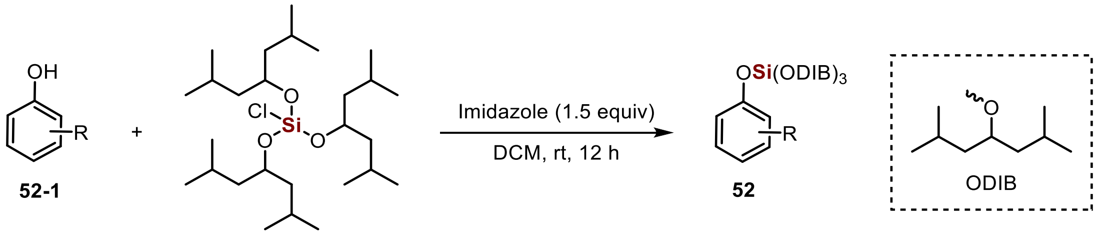

To a stirred solution of the corresponding phenol (5 mmol, 1.0 equiv) and trialkoxychlorosilane (7.5 mmol, 1.5 equiv) in CH_2Cl_2 (10 mL) was added dropwise a solution of imidazole (7.5 mmol, 1.5 equiv) in CH_2Cl_2 (10 mL) at room temperature. The mixture was stirred at room temperature for 12 h. The white precipitate was removed by filtration under nitrogen. Water 20 mL was added and the product was extracted into CH_2Cl_2, washed with brine, dried over Na_2SO_4 and concentrated. The crude product was purified (5% ethyl acetate in hexane as eluent) by silica gel chromatography as a colorless oil.[5]

2-chlorophenyl tris(2,6-dimethylheptan-4-yl) silicate (52a)

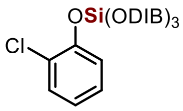

^{1}H NMR (400 MHz, $CDCl_3$) δ 7.32 (dd, *J* = 7.9, 1.6 Hz, 1H), 7.22 (dd, *J* = 8.2, 1.5 Hz, 1H), 7.10 (td, *J* = 7.8, 1.7 Hz, 1H), 6.88 (td, *J* = 7.6, 1.5 Hz, 1H), 4.27 – 4.10 (m, 3H), 1.99 – 1.63 (m, 6H), 1.59 – 1.41 (m, 6H), 1.33 – 1.20 (m, 6H), 0.86 (dd, *J* = 11.2, 6.6 Hz, 36H). **^{13}C NMR** (101 MHz, $CDCl_3$) δ 150.5, 130.2, 127.3, 124.9, 122.2, 120.6, 71.1, 46.7, 24.5, 23.1, 22.8. **HRMS (ESI)** *m/z* Calcd for $C_{33}H_{61}ClNaO_4Si$ $[M+Na]^+$ 607.3920, Found: 607.3913.

2-bromophenyl tris(2,6-dimethylheptan-4-yl) silicate (52b)

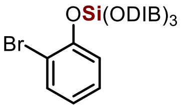

^{1}H NMR (400 MHz, $CDCl_3$) δ 7.50 (dd, *J* = 8.0, 1.6 Hz, 1H), 7.33 – 7.20 (m, 1H), 7.20 – 7.11 (m, 1H), 6.82 (td, *J* = 7.6, 1.5 Hz, 1H), 4.31 – 4.11 (m, 3H), 1.96 – 1.60 (m, 6H), 1.60 – 1.42 (m, 6H), 1.40 – 1.23 (m, 6H), 0.87 (dd, *J* = 11.5, 6.6 Hz, 36H). **^{13}C NMR** (101 MHz, $CDCl_3$) δ 151.5, 133.3, 128.1, 122.6, 120.4, 114.5, 71.2, 46.7, 24.6, 23.1, 22.9. **HRMS (ESI)** *m/z* Calcd for $C_{33}H_{61}BrNaO_4Si$ $[M+Na]^+$ 651.3415, Found: 651.3431.

Tris(2,6-dimethylheptan-4-yl) (2-(methylthio)phenyl) silicate (52c)

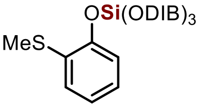

^{1}H NMR (400 MHz, $CDCl_3$) δ 7.18 – 7.06 (m, 2H), 7.05 – 6.91 (m, 2H), 4.32 – 4.11 (m, 3H), 2.39 (s, 3H), 1.83 – 1.67 (m, 6H), 1.54 – 1.43 (m, 6H), 1.34 – 1.25 (m, 6H), 0.87 (dd, *J* = 11.2, 6.6 Hz, 36H). **^{13}C NMR** (101 MHz, $CDCl_3$) δ 151.2, 129.4, 125.5, 125.0, 122.0, 118.5, 70.9, 46.5, 24.4, 23.0, 22.7, 14.6. **HRMS (ESI)** *m/z* Calcd for $C_{34}H_{64}NaO_4SSi$ $[M+Na]^+$ 619.4187, Found: 619.4196.

2,3-difluorophenyl tris(2,6-dimethylheptan-4-yl) silicate (52d)

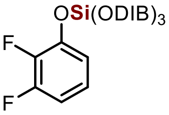

^{1}H NMR (400 MHz, $CDCl_3$) δ 7.01 – 6.83 (m, 2H), 6.82 – 6.70 (m, 1H), 4.27 – 4.05 (m, 3H), 1.81 – 1.66 (m, 6H), 1.52 – 1.38 (m, 6H), 1.34 – 1.19 (m, 6H), 0.88 (dd, *J* = 10.2, 6.7 Hz, 36H). **^{13}C NMR** (101 MHz, $CDCl_3$) δ 151.9 (dd, *J* = 246.5, 10.7 Hz), 144.2 (dd, *J* = 8.9, 3.0 Hz), 141.7 (d, *J* = 13.5 Hz), 122.6 (dd, *J* = 8.7, 5.2 Hz), 116.8 (d, *J* = 3.0 Hz), 109.8 (d, *J* = 17.4 Hz), 71.2, 46.8, 24.6, 23.1, 22.7. **^{19}F NMR** (376 MHz, $CDCl_3$) δ -137.8 (dd, *J* = 20.2, 3.2 Hz), -156.9 (d, *J* = 19.9 Hz). **HRMS (ESI)** *m/z* Calcd for $C_{33}H_{61}F_2O_4Si$ $[M+H]^+$ 587.4032, Found: 587.4043.

4-((triisopropylsilyl)oxy)-2H-chromen-2-one (52e)

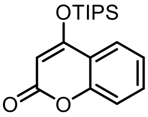

^{1}H NMR (400 MHz, $CDCl_3$) δ 7.85 (dd, *J* = 7.9, 1.7 Hz, 1H), 7.56 (ddd, *J* = 8.6, 7.2, 1.6 Hz, 1H), 7.38 – 7.25 (m, 2H), 5.73 (s, 1H), 1.43 (hept, *J* = 7.4 Hz, 3H), 1.17 (d, *J* = 7.7 Hz, 18H). **^{13}C NMR** (101 MHz, $CDCl_3$) δ 164.5, 163.4, 153.9, 132.5, 124.0, 123.5, 117.8, 116.9, 96.8, 17.9, 12.8. **HRMS (ESI)** *m/z* Calcd for $C_{18}H_{26}NaO_3Si$ $[M+Na]^+$ 341.1543, Found: 341.1551.

3.8 General procedure for the preparation of trialkoxyphenylsilane (GP8)

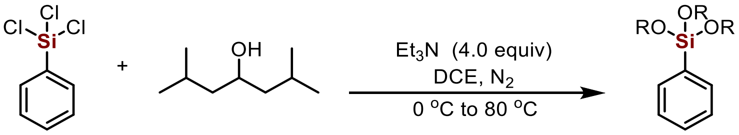

A 100-mL, oven-dried double-necked round-bottomed flask equipped with a septum, nitrogen inlet, and a magnetic stir bar was charged with trichlorophenylsilane (2.12g, 10 mmol) and 1,2-dichloroethane (30 mL). After the mixture was stirred at 0 °C for 5 min, diisobutylcarbinol (33 mmol, 3.3 equiv) was added via a syringe. Et_3N (5.6 mL, 40 mmol) was slowly added to the resulting mixture over 5 min and the mixture was warmed to 80 °C over 5 min. The resulting solution was stirred at 80 °C for 12 h, hexane (20 mL) was then added and the precipitate was filtered off by using a Büchner funnel. The filtrate was concentrated under reduced pressure, and hexane (16 mL) was then added to the residue. The reaction mixture was concentrated and purified directly by column chromatography on silica gel (hexane as eluent) to give arylsilanes as colourless liquid.[6]

Tris((2,6-dimethylheptan-4-yl)oxy)(phenyl)silane (59a)

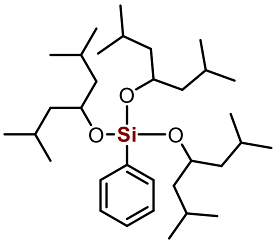

The product was obtained as colorless oil. **^{1}H NMR** (400 MHz, $CDCl_3$) δ 7.72 – 7.62 (m, 2H), 7.42 – 7.29 (m, 3H), 4.15 – 4.04 (m, 3H), 1.89 – 1.68 (m, 6H), 1.57 – 1.40 (m, 6H), 1.37 – 1.23 (m, 6H), 0.85 (dd, *J* = 6.6, 1.9 Hz, 36H). **^{13}C NMR** (101 MHz, $CDCl_3$) δ 135.1, 134.1, 129.7, 127.6, 70.0, 47.1, 24.6, 23.2, 22.9. **HRMS (ESI)** *m/z* Calcd for $C_{33}H_{62}NaO_3Si$ $[M+Na]^+$ 557.4360, Found: 557.4361.

3.9 General procedure for the preparation of various phenyltriethoxysilane (GP9)

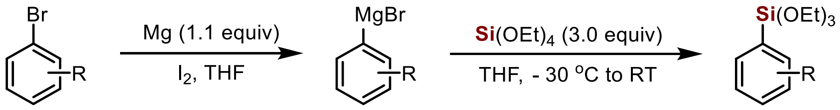

A 100 mL, three-neck, pear-shaped flask was fitted with an addition funnel, a reflux condenser, a rubber septum, and a stir bar. The flask was then charged with freshly washed magnesium turnings (0.792 mg, 33 mmol), flame-dried under vacuum, and back-filled with argon. THF (30 mL) was added to the magnesium turnings via syringe. The addition funnel was charged with the aryl halide (30 mmol) in 10 mL THF. The reaction was initiated by addition of 5-10 drops of the aryl halide solution to the magnesium turnings with stirring, followed by gentle heating. The rest of the aryl halide solution was then added at such a rate that the THF maintained a moderate reflux. Upon final addition, the solution was allowed to stir at room temperature until the complete consumption of aryl halide. The arylmagnesiumhalide solution was then transferred via cannula to a second flame-dried addition funnel, to which was fitted a 50 mL round-bottom flask containing tetraethyl orthosilicate or tetramethyl orthosilicate (90 mmol) in 20 mL of THF. The silane solution was cooled to -30 °C, and then the arylmagnesiumhalide solution was added dropwise (1 drop per second). The solution was allowed to stir at the indicated temperature for 1 h and then at room

temperature for 12 h. The crude reaction mixture was then poured into 50 mL of pentane in a 500 mL separatory funnel. The amber solution was washed with 50 mL of water, dried over $MgSO_4$, filtered, and concentrated in vacuo. Purification of the residue by short-path distillation to yield the desired arylsilanes.[7,8]

Triethoxy(2-ethylphenyl)silane (61aa)

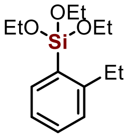

The product was obtained as colorless oil. **^{1}H NMR** (400 MHz, $CDCl_3$) δ 7.75 (dd, *J* = 7.4, 1.6 Hz, 1H), 7.39 (td, *J* = 7.5, 1.6 Hz, 1H), 7.26 (d, *J* = 7.7 Hz, 1H), 7.19 (td, *J* = 7.3, 1.2 Hz, 1H), 3.89 (q, *J* = 7.0 Hz, 6H), 2.87 (q, *J* = 7.5 Hz, 2H), 1.27 (t, *J* = 7.0 Hz, 12H). **^{13}C NMR** (101 MHz, $CDCl_3$) δ 151.2, 136.6, 130.7, 129.4, 128.2, 124.9, 58.6, 29.1, 18.2, 16.2. **HRMS (ESI)** *m/z* Calcd for $C_{14}H_{24}NaO_3Si$ $[M+Na]^+$ 291.1387, Found: 291.1392.

Triethoxy(2-isopropylphenyl)silane (62aa)

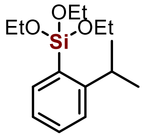

The product was obtained as colorless oil. **^{1}H NMR** (400 MHz, $CDCl_3$) δ 7.75 (d, *J* = 7.3 Hz, 1H), 7.46 – 7.37 (m, 1H), 7.35 (d, *J* = 7.8 Hz, 1H), 7.19 (td, *J* = 7.3, 1.3 Hz, 1H), 4.09 – 3.80 (m, 6H), 3.43 – 3.28 (m, 1H), 1.37 – 1.15 (m, 15H). **^{13}C NMR** (101 MHz, $CDCl_3$) δ 156.2, 136.6, 130.9, 129.0, 125.2, 125.1, 58.7, 33.8, 24.7, 18.3. **HRMS (ESI)** *m/z* Calcd for $C_{15}H_{26}NaO_3Si$ $[M+Na]^+$ 305.1543, Found: 305.1548.

Triethoxy(2-cyclopropylphenyl)silane (63aa)

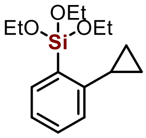

The product was obtained as colorless oil. **^{1}H NMR** (400 MHz, $CDCl_3$) δ 7.75 (d, *J* = 7.4 Hz, 1H), 7.33 (td, *J* = 7.6, 1.6 Hz, 1H), 7.21 – 7.08 (m, 1H), 6.79 (d, *J* = 7.9 Hz, 1H), 3.89 (q, *J* = 7.0 Hz, 6H), 2.54 – 2.24 (m, 1H), 1.26 (t, *J* = 7.1 Hz, 9H), 1.06 – 0.96 (m, 2H), 0.78 – 0.70 (m, 2H). **^{13}C NMR** (101 MHz, $CDCl_3$) δ 150.5, 136.7, 130.9, 129.9, 124.6, 122.0, 58.6, 18.3, 15.2, 10.2. **HRMS (ESI)** *m/z* Calcd for $C_{15}H_{24}NaO_3Si$ $[M+Na]^+$ 303.1387, Found: 303.1377.

Triethoxy(2-ethoxyphenyl)silane (65aa)

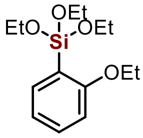

The product was obtained as colorless oil. **^{1}H NMR** (400 MHz, $CDCl_3$) δ 7.67 (dd, *J* = 7.2, 1.8 Hz, 1H), 7.47 – 7.33 (m, 1H), 6.95 (td, *J* = 7.3, 0.9 Hz, 1H), 6.83 (dd, *J* = 8.4, 0.9 Hz, 1H), 4.06 (q, *J* = 7.0 Hz, 2H), 3.90 (q, *J* = 7.0 Hz, 6H), 1.44 (t, *J* = 7.0 Hz, 3H), 1.25 (t, *J* = 7.0 Hz, 9H). **^{13}C NMR** (101 MHz, $CDCl_3$) δ 163.9, 137.7, 132.2, 120.4, 119.5, 110.4, 63.3, 58.7, 18.3, 14.9. **HRMS (ESI)** *m/z* Calcd for $C_{14}H_{25}O_4Si$ $[M+H]^+$ 285.1517, Found: 285.1514.

Trimethoxy(2-(trifluoromethyl)phenyl)silane (66aa)

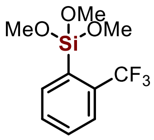

The product was obtained as yellow oil. **^{1}H NMR** (400 MHz, $CDCl_3$) δ 8.03 – 7.92 (m, 1H), 7.80 – 7.67 (m, 1H), 7.62 – 7.50 (m, 2H), 3.63 (s, 9H). **^{13}C NMR** (101 MHz, $CDCl_3$) δ 138.1, 135.9 (q, *J* = 31.5 Hz), 130.9 (d, *J* = 1.3 Hz), 130.7, 128.2 (q, *J* = 7.4 Hz), 126.2 (q, *J* = 5.2 Hz), 124.5 (d, *J* = 273.4 Hz), 51.04. **HRMS (ESI)** *m/z* Calcd for $C_{10}H_{13}F_3NaO_3Si$ $[M+Na]^+$ 289.0478, Found: 289.0484.

Triethoxy(2-(trifluoromethoxy)phenyl)silane (67aa)

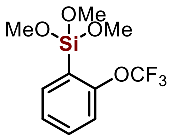

The product was obtained as colorless oil. **^{1}H NMR** (400 MHz, $CDCl_3$) δ 7.54 (dd, *J* = 7.6, 1.9 Hz, 1H), 7.42 – 7.24 (m, 1H), 7.10 (dd, *J* = 8.2, 6.2 Hz, 2H), 3.46 (s, 9H). **^{13}C NMR** (101 MHz, $CDCl_3$) δ 154.42, 137.82, 132.52, 126.12, 122.91, 120.70 (d, *J* = 257.7 Hz), 118.48 (q, *J* = 2.1 Hz), 50.92. **^{19}F NMR** (376 MHz, $CDCl_3$) δ -56.38. **HRMS (ESI)** *m/z* Calcd for $C_{10}H_{13}F_3NaO_4Si$ $[M+Na]^+$ 305.0427, Found: 305.0430.

Triethoxy(2-isopropoxyphenyl)silane (70aa)

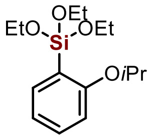

The product was obtained as colorless oil. **^{1}H NMR** (400 MHz, $CDCl_3$) δ 7.67 (dd, *J* = 7.2, 1.9 Hz, 1H), 7.40 – 7.32 (m, 1H), 6.91 (td, *J* = 7.3, 0.9 Hz, 1H), 6.83 (d, *J* = 8.4 Hz, 1H), 4.70 – 4.56 (m, 1H), 3.90 (q, *J* = 7.0 Hz, 6H), 1.37 (d, *J* = 6.1 Hz, 6H), 1.25 (t, *J* = 7.0 Hz, 9H). **^{13}C NMR** (101 MHz, $CDCl_3$) δ 162.7, 138.0, 132.0, 120.0, 111.2, 69.0, 58.7, 22.0, 18.6. **HRMS (ESI)** *m/z* Calcd for $C_{15}H_{27}O_4Si$ $[M+H]^+$ 299.1673, Found: 299.1679.

(2-((3,5-dimethoxybenzyl)oxy)phenyl)triethoxysilane (74a)

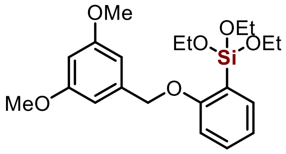

The product was obtained as colorless oil. **^{1}H NMR** (400 MHz, $CDCl_3$) δ 7.71 (dd, *J* = 7.2, 1.8 Hz, 1H), 6.42 – 6.41 (m, 1H), 7.11 – 6.85 (m, 1H), 6.89 (d, *J* = 8.2 Hz, 1H), 6.71 (d, *J* = 2.4 Hz, 2H), 7.44 – 7.34 (m, 1H), 5.07 (s, 2H), 3.89 (q, *J* = 7.1 Hz, 6H), 3.81 (s, 6H), 1.22 (t, *J* = 7.0 Hz, 9H). **^{13}C NMR** (101 MHz, $CDCl_3$) δ 163.6, 161.1, 139.8, 137.9, 132.3, 120.9, 119.8, 111.1, 104.9, 99.8, 69.8, 58.8, 55.5, 18.3. **HRMS (ESI)** *m/z* Calcd for $C_{21}H_{31}O_6Si$ $[M+H]^+$ 407.1884, Found: 407.1887.

Triethoxy(3-methylthiophen-2-yl)silane (76a)

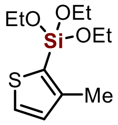

The product was obtained as colorless oil. **^{1}H NMR** (400 MHz, $CDCl_3$) δ 7.50 (d, *J* = 4.6 Hz, 1H), 7.00 (d, *J* = 4.6 Hz, 1H), 3.89 (q, *J* = 7.0 Hz, 6H), 2.42 (s, 3H), 1.26 (t, *J* = 7.0 Hz, 9H). **^{13}C NMR** (101 MHz, $CDCl_3$) δ 147.6, 131.5, 123.6, 58.9, 18.2, 16.4. **HRMS (ESI)** *m/z* Calcd for $C_{11}H_{20}NaO_3SSi$ $[M+Na]^+$ 283.0795, Found: 283.0795.

Triethoxy(5-chlorothiophen-2-yl)silane (77a)

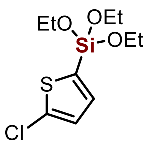

The product was obtained as colorless oil. **^{1}H NMR** (400 MHz, $CDCl_3$) δ 7.21 (d, J = 3.6 Hz, 1H), 6.98 (d, J = 3.6 Hz, 1H), 3.87 (q, J = 7.0 Hz, 6H), 1.24 (t, J = 7.0 Hz, 9H). **^{13}C NMR** (101 MHz, $CDCl_3$) δ 136.5, 136.4, 129.7, 127.7, 59.2, 18.2. **HRMS (ESI)** *m/z* Calcd for $C_{11}H_{17}ClNaO_3SSi$ $[M+Na]^+$ 303.0248, Found: 303.0241.

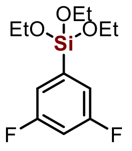

(3,5-difluorophenyl)triethoxysilane (78a)

^{1}H NMR (400 MHz, $CDCl_3$) δ 7.19 – 7.12 (m, 2H), 6.88 – 6.80 (m, 1H), 3.87 (q, J = 7.0 Hz, 6H), 1.24 (t, J = 7.0 Hz, 9H). **^{13}C NMR** (101 MHz, $CDCl_3$) δ 163.0 (dd, $J_{C\text{-}F}$ = 251.5, 10.6 Hz), 136.0, 126.70 – 113.1 (m), 105.9 (t, $J_{C\text{-}F}$ = 25.0 Hz), 59.1, 18.3. **^{19}F NMR** (376 MHz, $CDCl_3$) δ -110.2. **HRMS (ESI)** *m/z* Calcd for $C_{12}H_{18}F_2NaO_3Si$ $[M+Na]^+$ 299.0885, Found: 299.0877.

(3-chloro-5-fluorophenyl)triethoxysilane (79a)

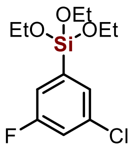

^{1}H NMR (400 MHz, $CDCl_3$) δ 7.41 (dd, J = 2.0, 0.7 Hz, 1H), 7.28 – 7.21 (m, 1H), 7.16 – 7.11 (m, 1H), 3.87 (q, J = 7.0 Hz, 6H), 1.25 (t, J = 7.0 Hz, 9H). **^{13}C NMR** (101 MHz, $CDCl_3$) δ 162.6 (d, $J_{C\text{-}F}$ = 252.1 Hz), 136.1 (d, $J_{C\text{-}F}$ = 5.3 Hz), 135.1 (d, $J_{C\text{-}F}$ = 8.7 Hz), 130.4 (d, $J_{C\text{-}F}$ = 3.2 Hz), 119.5 (d, $J_{C\text{-}F}$ = 19.1 Hz), 118.2 (d, $J_{C\text{-}F}$ = 24.5 Hz), 59.2, 18.3. **^{19}F NMR** (376 MHz, $CDCl_3$) δ -111.3. **HRMS (ESI)** *m/z* Calcd for $C_{12}H_{18}ClFNaO_3Si$ $[M+Na]^+$ 315.0590, Found: 315.0592.

(5-chloro-2-isopropoxyphenyl)triethoxysilane (80a)

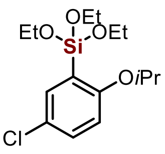

^{1}H NMR (400 MHz, $CDCl_3$) δ 7.60 (d, J = 2.8 Hz, 1H), 7.45 – 7.20 (m, 1H), 6.75 (d, J = 8.8 Hz, 1H), 4.56 (hept, J = 6.1 Hz, 1H), 3.93 – 3.86 (m, 6H), 1.35 (d, J = 6.0 Hz, 6H), 1.24 (t, J = 7.0 Hz, 9H). **^{13}C NMR** (101 MHz, $CDCl_3$) δ 161.2, 137.5, 131.6, 125.1, 122.7, 112.6, 69.8, 58.88, 22.0, 18.4. **HRMS (ESI)** *m/z* Calcd for $C_{15}H_{25}ClNaO_4Si$ $[M+Na]^+$ 355.1103, Found: 355.1109.

(5-chloro-2-methoxyphenyl)triethoxysilane (81a)

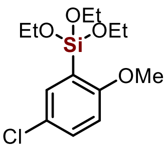

^{1}H NMR (400 MHz, $CDCl_3$) δ 7.58 (d, J = 2.7 Hz, 1H), 7.33 (dd, J = 8.7, 2.7 Hz, 1H), 6.77 (d, J = 8.8 Hz, 1H), 3.87 (q, J = 7.0 Hz, 6H), 3.81 (s, 3H), 1.23 (t, J = 7.0 Hz, 9H). **^{13}C NMR** (101 MHz, $CDCl_3$) δ 163.0, 137.2, 131.8, 125.9, 122.1, 111.2, 58.9, 55.7, 18.3. **HRMS (ESI)** *m/z* Calcd for $C_{13}H_{21}ClNaO_4Si$ $[M+Na]^+$ 327.0790, Found: 327.0779.

(5-fluoro-2-(methoxymethoxy)phenyl)triethoxysilane (82a)

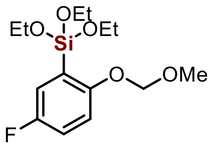

^{1}H NMR (400 MHz, $CDCl_3$) δ 7.36 (dd, J = 8.5, 3.3 Hz, 1H), 7.11 – 7.00 (m, 2H), 5.16 (s, 2H), 3.90 (q, J = 7.0 Hz, 6H), 3.51 (s, 3H), 1.25 (t, J = 7.0 Hz, 9H). **^{13}C NMR** (101 MHz, $CDCl_3$) δ 158.3 (d, J_{C-F} = 1.9 Hz), 157.8 (d, J_{C-F} = 240.8 Hz), 123.5 (d, J_{C-F} = 21.7 Hz), 122.7 (d, J_{C-F} = 4.7 Hz), 118.4 (d, J_{C-F} = 23.4 Hz), 114.9 (d, J_{C-F} = 7.2 Hz), 95.0, 59.3, 58.8, 56.0, 18.3. **^{19}F NMR** (376 MHz, $CDCl_3$) δ -123.2. **HRMS (ESI)** *m/z* Calcd for $C_{14}H_{23}FNaO_5Si$ $[M+Na]^+$ 341.1191, Found: 341.1184.

(2-((3,5-dimethoxybenzyl)oxy)-5-fluorophenyl)triethoxysilane (83a)

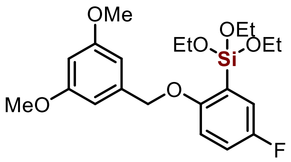

^{1}H NMR (400 MHz, $CDCl_3$) δ 7.42 – 7.35 (m, 1H), 7.09 – 6.97 (m, 1H), 6.89 – 6.76 (m, 1H), 6.67 (s, 2H), 6.41 (d, J = 2.2 Hz, 1H), 5.03 (s, 2H), 3.87 (q, J = 7.9, 7.0 Hz, 6H), 3.80 (d, J = 1.8 Hz, 6H), 1.21 (t, J = 7.9 Hz, 9H). **^{13}C NMR** (101 MHz, $CDCl_3$) δ 161.10, 159.48 (d, J_{C-F} = 1.7 Hz), 157.34 (d, J_{C-F} = 240.0 Hz), 139.55, 123.85 (d, J_{C-F} = 21.8 Hz), 122.23 (d, J_{C-F} = 4.5 Hz), 118.20 (d, J_{C-F} = 23.3 Hz), 112.30 (d, J_{C-F} = 7.2 Hz), 104.93, 99.89, 70.60, 58.92, 55.47, 18.31. **^{19}F NMR** (376 MHz, $CDCl_3$) δ -124.2. **HRMS (ESI)** *m/z* Calcd for $C_{21}H_{29}FNaO_6Si$ $[M+Na]^+$ 447.1610, Found: 447.1625.

(2-((3,5-dimethylphenoxy)methyl)-5-fluorophenyl)triethoxysilane (84a)

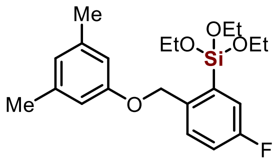

^{1}H NMR (400 MHz, $CDCl_3$) δ 7.64 – 7.53 (m, 1H), 7.55 – 7.47 (m, 1H), 7.19 – 7.08 (m, 1H), 6.68 – 6.62 (m, 3H), 5.21 (s, 2H), 3.90 (q, J = 7.0 Hz, 6H), 2.30 (s, 6H), 1.33 – 1.20 (m, 9H). **^{13}C NMR** (101 MHz, $CDCl_3$) δ 161.8 (d, J_{C-F} = 247.1 Hz), 159.0, 139.5 (d, J_{C-F} = 3.0 Hz), 139.2, 132.0 (d, J_{C-F} = 4.9 Hz), 129.6 (d, J_{C-F} = 7.0 Hz), 122.8 (d, J_{C-F} = 19.9 Hz), 122.7, 117.6 (d, J_{C-F} = 21.1 Hz), 112.7, 68.9, 59.0, 21.6, 18.3. **^{19}F NMR** (376 MHz, $CDCl_3$) δ -115.8 (d, J = 4.3 Hz). **HRMS (ESI)** *m/z* Calcd for $C_{21}H_{30}FO_4Si$ $[M+H]^+$ 393.1892, Found: 393.1901.

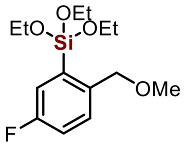

Triethoxy(5-fluoro-2-(methoxymethyl)phenyl)silane (85a)

^{1}H NMR (400 MHz, $CDCl_3$) δ 7.51 – 7.39 (m, 2H), 7.09 (td, J = 8.5, 2.9 Hz, 1H), 4.58 (s, 2H), 3.86 (q, J = 7.0 Hz, 6H), 3.42 (s, 3H), 1.25 (t, J = 7.0 Hz, 9H). **^{13}C NMR** (101 MHz, $CDCl_3$) δ 161.7 (d, J_{C-F} = 247.0 Hz), 140.6 (d, J_{C-F} = 3.0 Hz), 132.3 (d, J_{C-F} = 4.9 Hz), 129.8 (d, J_{C-F} = 7.1 Hz), 122.8 (d, J_{C-F} = 19.9 Hz), 117.4 (d, J_{C-F} = 21.2 Hz), 73.7, 58.9, 58.4, 18.2. **^{19}F NMR** (376 MHz, $CDCl_3$) δ -116.2. **HRMS (ESI)** *m/z* Calcd for $C_{14}H_{23}FNaO_4Si$ $[M+Na]^+$ 325.1242, Found: 325.1246.

(2-(1,3-dioxolan-2-yl)-5-fluorophenyl)triethoxysilane (86a)

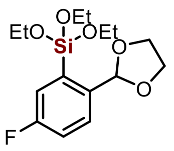

^{1}H NMR (400 MHz, $CDCl_3$) δ 7.66 (dd, *J* = 8.6, 5.4 Hz, 1H), 7.49 (dd, *J* = 9.2, 2.8 Hz, 1H), 7.14 (td, *J* = 8.5, 2.8 Hz, 1H), 6.07 (s, 1H), 4.20 – 4.10 (m, 2H), 4.07 – 3.98 (m, 2H), 3.86 (q, *J* = 7.0 Hz, 6H), 1.24 (t, *J* = 7.0 Hz, 9H). **^{13}C NMR** (101 MHz, $CDCl_3$) δ 162.8 (d, J_{C-F} = 249.2 Hz), 139.8 (d, J_{C-F} = 3.3 Hz), 133.4 (d, J_{C-F} = 5.1 Hz), 128.7 (d, J_{C-F} = 7.4 Hz), 122.7 (d, J_{C-F} = 20.2 Hz), 117.7 (d, J_{C-F} = 21.4 Hz), 102.5, 65.5, 58.9, 18.1. **^{19}F NMR** (376 MHz, $CDCl_3$) δ -113.3. **HRMS (ESI)** *m/z* Calcd for $C_{15}H_{23}FNaO_5Si$ $[M+Na]^+$ 353.1191, Found: 353.1180.

(5-fluoro-2-methylphenyl)triethoxysilane (87a)

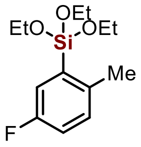

^{1}H NMR (400 MHz, $CDCl_3$) δ 7.42 (dd, *J* = 9.2, 3.0 Hz, 1H), 7.12 (dd, *J* = 8.4, 5.2 Hz, 1H), 6.98 (td, *J* = 8.5, 2.9 Hz, 1H), 3.86 (q, *J* = 7.0 Hz, 6H), 2.46 (s, 1H), 1.25 (t, *J* = 7.0 Hz, 9H). **^{13}C NMR** (101 MHz, $CDCl_3$) δ 160.7 (d, J_{C-F} = 244.7 Hz), 134.0 (d, J_{C-F} = 3.3 Hz), 132. 7 (d, J_{C-F} = 4.4 Hz), 131.4 (d, J_{C-F} = 6.6 Hz), 122.7 (d, J_{C-F} = 19.6 Hz), 117.1 (d, J_{C-F} = 21.0 Hz), 58.7, 21.5, 18.2. **^{19}F NMR** (376 MHz, $CDCl_3$) δ -119.3 (d, *J* = 5.2 Hz). **HRMS (ESI)** *m/z* Calcd for $C_{13}H_{22}FO_3Si$ $[M+H]^+$ 273.1317, Found: 273.1324.

(5-fluoro-2-methoxyphenyl)triethoxysilane (88a)

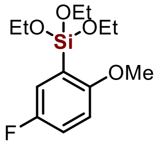

^{1}H NMR (400 MHz, $CDCl_3$) δ 7.34 (dd, *J* = 8.3, 3.2 Hz, 1H), 7.13 – 6.99 (m, 1H), 6.77 (dd, *J* = 8.9, 3.8 Hz, 1H), 3.87 (q, *J* = 7.0 Hz, 6H), 3.80 (s, 3H), 1.23 (t, *J* = 7.0 Hz, 9H). **^{13}C NMR** (101 MHz, $CDCl_3$) δ 160.3 (d, J_{C-F} = 1.7 Hz), 157.1 (d, J_{C-F} = 239.7 Hz), 123.5 (d, J_{C-F} = 21.8 Hz), 121.7 (d, J_{C-F} = 4.9 Hz), 118.0 (d, J_{C-F} = 23.4 Hz), 110.8 (d, J_{C-F} = 7.3 Hz), 58.8, 55.8, 18.2. **^{19}F NMR** (376 MHz, $CDCl_3$) δ -124.8. **HRMS (ESI)** *m/z* Calcd for $C_{13}H_{21}FNaO_4Si$ $[M+Na]^+$ 311.1085, Found: 311.1088.

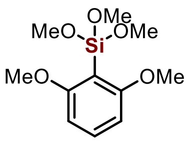

(2,6-dimethoxyphenyl)trimethoxysilane (89a)

The product was obtained as colorless oil. **^{1}H NMR** (400 MHz, $CDCl_3$) δ 7.36 – 7.32 (m, 1H), 6.53 (d, *J* = 8.3 Hz, 2H), 3.82 (s, 6H), 3.61 (s, 9H). **^{13}C NMR** (101 MHz, $CDCl_3$) δ 166.2, 133.4, 103.8, 55.9, 50.8. **HRMS (ESI)** *m/z* Calcd for $C_{11}H_{18}NaO_5Si$ $[M+Na]^+$ 281.0816, Found: 281.0809.

(2-methoxy-6-methylphenyl)trimethoxysilane (90a)

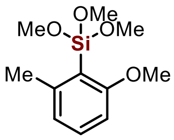

^{1}H NMR (400 MHz, $CDCl_3$) δ 7.29 – 7.25 (m, 1H), 6.80 (d, *J* = 7.6 Hz, 1H), 6.71 (d, *J* = 8.3 Hz, 1H), 3.84 (s, 3H), 3.62 (s, 9H), 2.49 (s, 3H). **^{13}C NMR** (101 MHz, $CDCl_3$) δ 165.5, 147.4, 131.8, 123.9, 117.3, 107.5, 55.8, 50.6, 23.3. **HRMS (ESI)** *m/z* Calcd for $C_{11}H_{18}NaO_4Si$ $[M+Na]^+$ 265.0867, Found: 265.0861.

(2,6-diethylphenyl)trimethoxysilane (91a)

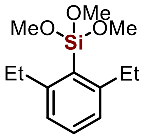

^{1}H NMR (400 MHz, $CDCl_3$) δ 7.34 – 7.30 (m, 1H), 7.11 (d, *J* = 7.6 Hz, 2H), 3.64 (s, 9H), 2.93 (q, *J* = 7.5 Hz, 4H), 1.26 (t, *J* = 7.5 Hz, 6H). **^{13}C NMR** (101 MHz, $CDCl_3$) δ 152.8, 130.7, 126.8, 126.0, 50.5, 29.8, 17.4. **HRMS (ESI)** *m/z* Calcd for $C_{13}H_{22}NaO_3Si$ $[M+Na]^+$ 277.1230, Found: 277.1224.

3.10 General procedure for the preparation of tris((2,6-dimethylheptan-4-yl)oxy)(phenyl)silane (GP10)

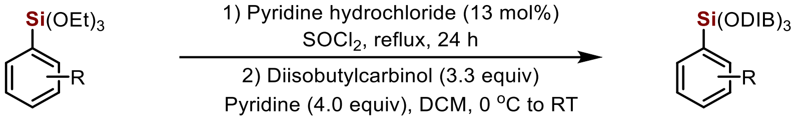

1) Phenyltriethoxysilane (5 mmol), thionylchloride (5 mL) and pyridinium hydrochloride (75.1 mg, 0.65 mmol) were refluxed and stirred for 24 h. Excess of $SOCl_2$ was distilled off and pentafluorophenyltrichlorosilane isolated by vacuum-distillation.

2) A 50 mL, oven-dried double-necked round-bottomed flask equipped with a septum, nitrogen inlet, and a magnetic stir bar was charged with trichlorophenylsilane (4 mmol, 1.0 equiv) and dichloromethane (10 mL). After the mixture was stirred at 0 °C for 5 min, diisobutylcarbinol (13.2 mmol, 3.3 equiv) was added via a syringe. Pyridine (1.26 g, 4.0 equiv) was slowly added to the resulting mixture over 5 min. The resulting solution was stirred at room temperature for 12 h, hexane (20 mL) was then added and the precipitate was filtered off by using a Büchner funnel. The filtrate was concentrated under reduced pressure, and hexane (10 mL) was then added to the residue. The reaction mixture was concentrated and purified directly by column chromatography on silica gel (hexane as eluent) to give arylsilanes as colourless liquid.[9]

Tris((2,6-dimethylheptan-4-yl)oxy)(o-tolyl)silane (60a)

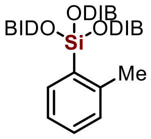

The product was obtained as colorless oil. **^{1}H NMR** (400 MHz, $CDCl_3$) δ 7.69 (dd, *J* = 7.7, 1.6 Hz, 1H), 7.24 (td, *J* = 7.4, 1.5 Hz, 1H), 7.16 – 7.08 (m, 2H), 4.11 – 4.02 (m, 3H), 2.51 (s, 3H), 1.87 – 1.63 (m, 6H), 1.60 – 1.39 (m, 6H), 1.36 – 1.23 (m, 6H), 0.82 (dd, *J* = 6.7, 4.3 Hz, 36H). **^{13}C NMR** (101 MHz, $CDCl_3$) δ 143.9, 136.5, 133.5, 129.7, 129.6, 124.5, 70.2, 47.0, 24.6, 23.1, 23.1. **HRMS (ESI)** *m/z* Calcd for $C_{34}H_{65}O_3Si$ $[M+H]^+$ 549.4697, Found: 549.4711 .

Tris((2,6-dimethylheptan-4-yl)oxy)(2-ethylphenyl)silane (61a)

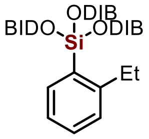

The product was obtained as colorless oil.**^{1}H NMR** (400 MHz, $CDCl_3$) δ 7.68 (dd, *J* = 7.5, 1.5 Hz, 1H), 7.31 (td, *J* = 7.5, 1.6 Hz, 1H), 7.20 (d, *J* = 9.0 Hz, 1H), 7.13 (td, *J* = 7.3, 1.3 Hz, 1H), 4.06 (p, *J* = 6.3 Hz, 3H), 2.86 (q, *J* = 7.5 Hz, 2H), 1.86 – 1.64 (m, 6H), 1.51 – 1.37 (m, 6H), 1.36 – 1.17 (m,

9H), 0.82 (dd, *J* = 6.6, 4.4 Hz, 36H). **^{13}C NMR** (101 MHz, $CDCl_3$) δ 150.4, 136.4, 132.9, 129.9, 127.7, 124.6, 70.2, 47.0, 29.9, 29.4, 24.6, 23.1, 16.2. **HRMS (ESI)** *m/z* Calcd for $C_{35}H_{66}NaO_3Si$ $[M+Na]^+$ 585.4673, Found: 585.4678.

Tris((2,6-dimethylheptan-4-yl)oxy)(2-isopropylphenyl)silane (62a)

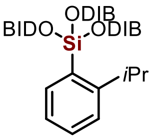

The product was obtained as colorless oil. **^{1}H NMR** (400 MHz, $CDCl_3$) δ 7.68 (dd, *J* = 7.3, 2.5 Hz, 1H), 7.40 – 7.26 (m, 2H), 7.22 – 7.03 (m, 1H), 4.18 – 4.05 (m, 3H), 3.54 – 3.42 (m, 1H), 1.82 – 1.68 (m, 6H), 1.54 – 1.44 (m, 6H), 1.38 – 1.30 (m, 6H), 1.29 – 1.21 (m, 6H), 0.89 – 0.81 (m, 36H). **^{13}C NMR** (101 MHz, $CDCl_3$) δ 155.4, 136.2, 132.6, 130.0, 124.9, 124.7, 70.3, 46.9, 33.3, 25.0, 24.6, 23.1. **HRMS (ESI)** *m/z* Calcd for $C_{36}H_{68}NaO_3Si$ $[M+Na]^+$ 599.4830, Found: 599.4842.

Tris((2,6-dimethylheptan-4-yl)oxy)(2-cyclopropylphenyl)silane (63a)

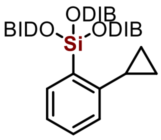

The product was obtained as colorless oil. **^{1}H NMR** (400 MHz, $CDCl_3$) δ 7.69 (d, *J* = 7.4 Hz, 1H), 7.27 – 7.21 (m, 1H), 7.07 (t, *J* = 7.3 Hz, 1H), 6.62 (d, *J* = 7.9 Hz, 1H), 4.11 (p, *J* = 6.3 Hz, 3H), 2.45 (td, *J* = 8.6, 4.1 Hz, 1H), 1.80 – 1.64 (m, 6H), 1.52 – 1.41 (m, 6H), 1.36 – 1.26 (m, 6H), 1.03 – 0.93 (m, 2H), 0.83 – 0.71 (m, 36H), 0.75 – 0.69 (m, 2H). **^{13}C NMR** (101 MHz, $CDCl_3$) δ 150.1, 136.2, 133.4, 130.0, 124.2, 120.7, 70.2, 47.0, 24.6, 23.1, 23.0, 15.5, 11.8. **HRMS (ESI)** *m/z* Calcd for $C_{36}H_{67}O_3Si$ $[M+H]^+$ 575.4854, Found: 575.4872.

Tris((2,6-dimethylheptan-4-yl)oxy)(2-methoxyphenyl)silane (64a)

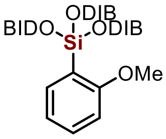

The product was obtained as colorless oil. **^{1}H NMR** (400 MHz, $CDCl_3$) δ 7.72 (d, *J* = 7.2 Hz, 1H), 7.37 – 7.33 (m, 1H), 6.96– 6.92 (m, 1H), 6.79 (d, *J* = 8.3 Hz, 1H), 4.09 (p, *J* = 6.5 Hz, 3H), 3.79 (s, 3H), 1.84 – 1.67 (m, 6H), 1.52 – 1.39 (m, 6H), 1.36 – 1.20 (m, 6H), 0.86 – 0.77 (m, 36H). **^{13}C NMR** (101 MHz, $CDCl_3$) δ 163.8, 138.2, 131.5, 122.2, 120.4, 108.7, 69.5, 54.7, 47.1, 24.5, 23.2, 23.0. **HRMS (ESI)** *m/z* Calcd for $C_{34}H_{64}NaO_4Si$ $[M+Na]^+$ 587.4466, Found: 587.4470.

Tris((2,6-dimethylheptan-4-yl)oxy)(2-ethoxyphenyl)silane (65a)

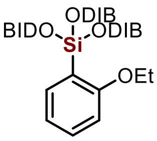

The product was obtained as colorless oil. **^{1}H NMR** (400 MHz, $CDCl_3$) δ 7.72 (dd, *J* = 7.3, 1.9 Hz, 1H), 7.38 – 7.29 (m, 1H), 6.92 (td, *J* = 7.3, 0.9 Hz, 1H), 6.78 (d, *J* = 8.3 Hz, 1H), 4.20 – 4.10 (m, 3H), 4.05 (q, *J* = 7.0 Hz, 2H), 1.88 – 1.66 (m, 6H), 1.55 – 1.41 (m, 9H), 1.36 – 1.21 (m, 6H), 0.83 (dd, *J* = 6.7, 4.2 Hz, 36H). **^{13}C NMR** (101 MHz, $CDCl_3$) δ 163.4, 138.1, 131.3, 122.8, 120.2, 109.9, 70.0, 63.2, 47.0, 24.5, 23.2, 23.1, 15.1. **HRMS (ESI)** *m/z* Calcd for $C_{35}H_{66}NaO_4Si$ $[M+Na]^+$ 601.4623, Found: 601.4634 .

Tris((2,6-dimethylheptan-4-yl)oxy)(2-(trifluoromethyl)phenyl)silane (66a)

The product was obtained as colorless oil. **^{1}H NMR** (400 MHz, $CDCl_3$) δ 8.07 (dd, *J* = 6.6, 2.3 Hz, 1H), 7.66 (dd, *J* = 6.9, 2.2 Hz, 1H), 7.53 – 7.45 (m, 2H), 4.10 (p, *J* = 6.4 Hz, 3H), 1.87 – 1.63 (m, 6H), 1.54 – 1.40 (m, 6H), 1.39 – 1.23 (m, 6H), 0.83 (dd, *J* = 6.7, 3.0 Hz, 36H). **^{13}C NMR** (101 MHz, $CDCl_3$) δ 138.1, 135.4 (q, *J* = 31.1 Hz), 133.0 (d, *J* = 2.8 Hz), 130.4, 129.7, 126.2 (q, *J* = 5.0 Hz), 70.7, 46.9, 24.6, 23.1, 23.0. **^{19}F NMR** (376 MHz, $CDCl_3$) δ -58.8. **HRMS (ESI)** *m/z* Calcd for $C_{34}H_{61}F_3NaO_3Si$ $[M+Na]^+$ 625.4234, Found: 625.4236.

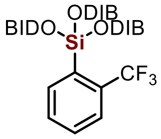

Tris((2,6-dimethylheptan-4-yl)oxy)(2-(trifluoromethoxy)phenyl)silane (67a)

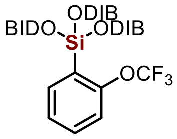

The product was obtained as colorless oil. **^{1}H NMR** (400 MHz, $CDCl_3$) δ 7.78 (dd, *J* = 7.5, 2.0 Hz, 1H), 7.40 (td, *J* = 7.8, 1.8 Hz, 1H), 7.20 – 7.24 (m, 2H), 4.21 – 4.11 (m, 3H), 1.84 – 1.55 (m, 6H), 1.52 – 1.38 (m, 6H), 1.39 – 1.15 (m, 6H), 0.84 (dd, *J* = 6.8, 3.7 Hz, 36H). **^{13}C NMR** (101 MHz, $CDCl_3$) δ 154.2, 137.8, 131.4, 126.5, 125.3, 120.8 (d, *J* = 258.0 Hz), 116.6 (d, *J* = 9.9 Hz), 70.4, 46.9, 24.6, 23.0, 23.0. **^{19}F NMR** (376 MHz, $CDCl_3$) δ -55.4. **HRMS (ESI)** *m/z* Calcd for $C_{34}H_{61}F_3NaO_4Si$ $[M+Na]^+$ 641.4183, Found: 641.4172.

Tris((2,6-dimethylheptan-4-yl)oxy)(m-tolyl)silane (68a)

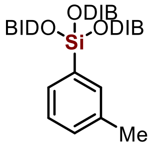

The product was obtained as colorless oil. **^{1}H NMR** (400 MHz, $CDCl_3$) δ 7.51 – 7.44 (m, 2H), 7.29 – 7.16 (m, 2H), 4.15 – 4.04 (m, 3H), 2.36 (s, 3H), 1.87 – 1.70 (m, 6H), 1.54 – 1.41 (m, 6H), 1.39 – 1.24 (m, 6H), 0.86 (dd, *J* = 6.7, 1.8 Hz, 36H). **^{13}C NMR** (101 MHz, $CDCl_3$) δ 136.6, 135.8, 133.7, 132.3, 130.4, 127.5, 69.9, 47.2, 24.6, 23.2, 22.9, 21.6. **HRMS (ESI)** *m/z* Calcd for $C_{34}H_{65}O_3Si$ $[M+H]^+$ 549.4697, Found: 549.4694.

Tris((2,6-dimethylheptan-4-yl)oxy)(3-chlorophenyl)silane (69a)

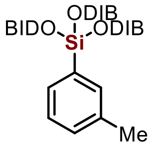

The product was obtained as colorless oil. **^{1}H NMR** (400 MHz, $CDCl_3$) δ 7.64 (d, *J* = 2.2 Hz, 1H), 7.54 (d, *J* = 7.1 Hz, 1H), 7.37 (dd, *J* = 8.1, 3.5 Hz, 1H), 7.33 – 7.27 (m, 1H), 4.15 – 4.04 (m, 3H), 1.83 – 1.68 (m, 6H), 1.53 – 1.42 (m, 6H), 1.37 – 1.25 (m, 6H), 0.87 (dd, *J* = 6.7, 1.4 Hz, 36H). **^{13}C NMR** (101 MHz, $CDCl_3$) δ 136.9, 134.9, 134.0, 133.0, 129.7, 129.1, 70.2, 47.1, 24.6, 23.2, 22.9. **HRMS (ESI)** *m/z* Calcd for $C_{33}H_{61}ClNaO_3Si$ $[M+Na]^+$ 591.3971, Found: 591.3986.

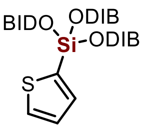

Tris((2,6-dimethylheptan-4-yl)oxy)(thiophen-2-yl)silane (75a)

The product was obtained as colorless oil. **^{1}H NMR** (400 MHz, $CDCl_3$) δ 7.59 (dd, *J* = 4.6, 0.9 Hz, 1H), 7.41 (dd, *J* = 3.3, 0.9 Hz, 1H), 7.18 (dd, *J* = 4.6, 3.4 Hz, 1H), 4.67 – 3.64 (m, 3H), 1.84 – 1.67

(m, 6H), 1.52 – 1.41 (m, 6H), 1.38 – 1.21 (m, 6H), 0.84 (dd, J = 6.6, 1.9 Hz, 36H). **^{13}C NMR** (101 MHz, $CDCl_3$) δ 136.5, 132.5, 131.2, 127.8, 70.3, 47.0, 24.6, 23.2, 22.8. **HRMS (ESI)** *m/z* Calcd for $C_{31}H_{60}NaO_3SSi$ $[M+Na]^+$ 563.3925, Found: 563.3910.

3.11 General procedure for the preparation of bis((2,6-dimethylheptan-4-yl)oxy)(ethoxy)(phenyl)silane (GP11)

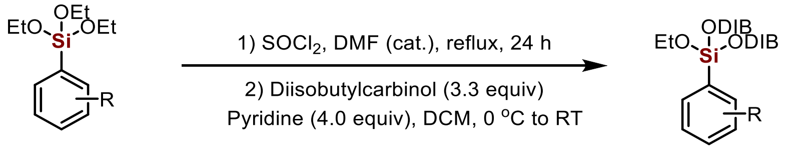

1) A Claisen flask was charged with Phenyltriethoxysilane (5 mmol), $SOCl_2$ (5 mL) and a catalytic amount (1–2 drops) of DMF. The resulting solution was stirred at 80 °C for 48 h. The pure dichloro(ethoxy)(phenyl)silane was distilled off the reaction mixture.

2) A 50 mL, oven-dried double-necked round-bottomed flask equipped with a septum, nitrogen inlet, and a magnetic stir bar was charged with dichloro(ethoxy)(phenyl)silane (4 mmol, 1.0 equiv) and dichloromethane (10 mL). After the mixture was stirred at 0 °C for 5 min, diisobutylcarbinol (13.2 mmol, 3.3 equiv) was added via a syringe. Pyridine (1.26 g, 4.0 equiv) was slowly added to the resulting mixture over 5 min. The resulting solution was stirred at room temperature for 12 h, hexane (20 mL) was then added and the precipitate was filtered off by using a Büchner funnel. The filtrate was concentrated under reduced pressure, and hexane (10 mL) was then added to the residue. The reaction mixture was concentrated and purified directly by column chromatography on silica gel (hexane as eluent) to give arylsilanes as colourless liquid.[10]

Bis((2,6-dimethylheptan-4-yl)oxy)(ethoxy)(2-isopropoxyphenyl)silane (70a)

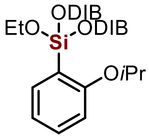

The product was obtained as colorless oil. **^{1}H NMR** (400 MHz, $CDCl_3$) δ 7.69 (dd, J = 7.3, 1.9 Hz, 1H), 7.38 – 7.28 (m, 1H), 6.89 (t, J = 7.3 Hz, 1H), 6.80 (d, J = 8.3 Hz, 1H), 4.69 – 4.54 (m, 1H), 4.26 – 4.05 (m, 2H), 3.89 (q, J = 6.9 Hz, 2H), 1.86 – 1.67 (m, 4H), 1.54 – 1.41 (m, 4H), 1.38 (d, J = 6.0 Hz, 6H), 1.32 – 1.20 (m, 7H), 0.92 – 0.78 (m, 24H). **^{13}C NMR** (101 MHz, $CDCl_3$) δ 162.6, 138.3, 131.5, 122.1, 119.8, 111.0, 69.6, 69.1, 58.7, 47.1, 47.0, 24.5, 23.3, 23.3, 23.0, 22.9, 22.3, 18.5. **HRMS (ESI)** *m/z* Calcd for $C_{29}H_{55}O_4Si$ $[M+H]^+$ 495.3864, Found: 495.3868.

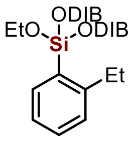

Bis((2,6-dimethylheptan-4-yl)oxy)(ethoxy)(2-ethylphenyl)silane (71a)

The product was obtained as colorless oil. **^{1}H NMR** (400 MHz, $CDCl_3$) δ 7.71 (dd, J = 7.4, 1.6 Hz, 1H), 7.34 (td, J = 7.5, 1.5 Hz, 1H), 7.23 (d, J = 7.7 Hz, 1H), 7.16 (td, J = 7.4, 1.2 Hz, 1H), 4.13 – 4.01

(m, 2H), 3.91 (q, J = 7.0 Hz, 2H), 2.87 (q, J = 7.5 Hz, 2H), 1.80 – 1.65 (m, 4H), 1.57 – 1.39 (m, 4H), 1.36 – 1.19 (m, 10H), 0.85 (dd, J = 15.3, 6.7 Hz, 24H). **^{13}C NMR** (101 MHz, $CDCl_3$) δ 150.8, 136.4, 131.6, 130.2, 128.0, 124.7, 70.1, 58.7, 47.0 (d, J = 2.9 Hz), 29.4, 24.6 (d, J = 1.7 Hz), 23.1, 22.9 (d, J = 5.5 Hz), 18.3, 16.3. **HRMS (ESI)** *m/z* Calcd for $C_{22}H_{52}NaO_3Si$ $[M+Na]^+$ 487.3578, Found: 487.3583.

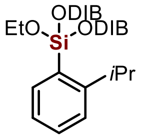

Bis((2,6-dimethylheptan-4-yl)oxy)(ethoxy)(2-isopropylphenyl)silane (72a)

The product was obtained as colorless oil. **^{1}H NMR** (400 MHz, $CDCl_3$) δ 7.69 (dd, J = 7.3, 1.9 Hz, 1H), 7.38 – 7.28 (m, 1H), 6.91 – 6.87 (m, 1H), 6.80 (d, J = 8.3 Hz, 1H), 4.69 – 4.54 (m, 1H), 4.26 – 4.05 (m, 2H), 3.89 (q, J = 6.9 Hz, 2H), 1.86 – 1.67 (m, 4H), 1.54 – 1.41 (m, 4H), 1.38 (d, J = 6.0 Hz, 6H), 1.32 – 1.20 (m, 7H), 0.92 – 0.78 (m, 24H). **^{13}C NMR** (101 MHz, $CDCl_3$) δ 162.6, 138.3, 131.5, 122.1, 119.8, 111.0, 69.6, 69.1, 58.7, 47.1, 47.0, 24.5, 23.3, 23.3, 23.0, 22.9, 22.3, 18.5. **HRMS (ESI)** *m/z* Calcd for $C_{29}H_{55}O_3Si$ $[M+H]^+$ 479.3915, Found: 479.3909.

Bis((2,6-dimethylheptan-4-yl)oxy)(2-cyclopropylphenyl)(ethoxy)silane (73a)

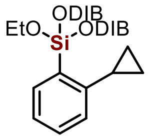

The product was obtained as colorless oil. **^{1}H NMR** (400 MHz, $CDCl_3$) δ 7.70 (dd, J = 7.5, 1.5 Hz, 1H), 7.27 (td, J = 7.4, 1.4 Hz, 1H), 7.10 (td, J = 7.4, 1.1 Hz, 1H), 6.67 (d, J = 7.9 Hz, 1H), 4.08 (p, J = 6.3, 5.7 Hz, 2H), 3.89 (q, J = 7.0 Hz, 2H), 2.61 – 2.33 (m, 1H), 1.82 – 1.63 (m, 4H), 1.55 – 1.37 (m, 4H), 1.35 – 1.20 (m, 7H), 1.03 – 0.94 (m, 2H), 0.82 (dd, J = 13.8, 7.2 Hz, 24H), 0.77 – 0.68 (m, 2H). **HRMS (ESI)** *m/z* Calcd for $C_{29}H_{52}NaO_3Si$ $[M+Na]^+$ 499.3578, Found: 499.3580.

3.12 General procedure for the preparation of benzyltris((2,6-dimethylheptan-4-yl)oxy)silane (GP12)

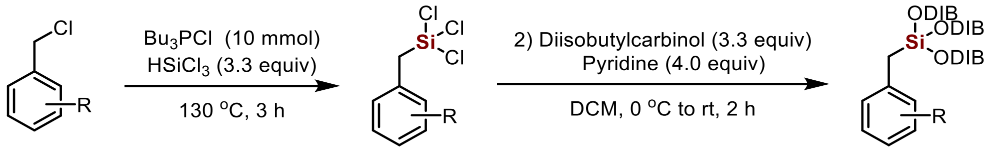

1) As a representative reaction, the reaction of benzyl chloride with Bu_3PCl is described as follows: A 25 mL dried stainless steel bomb equipped with a valve was charged with Bu_3PCl (0.22 g, 0.75 mmol), benzyl chloride (0.95 g, 7.5 mmol), and $HSiCl_3$ (3.41 g, 25.2 mmol) under a dry nitrogen atmosphere. After the valve was closed, the reactor

was kept in an oil bath at 130 °C for 4 h. The reaction mixture was fractionated to obtain colorless benzyltrichlorosilane.

2) A 50 mL, oven-dried double-necked round-bottomed flask equipped with a septum, nitrogen inlet, and a magnetic stir bar was charged with benzyltrichlorosilane (5 mmol, 1.0 equiv) and DCM (15 mL). After the mixture was stirred at 0 °C for 5 min, diisobutylcarbinol (16.5 mmol, 3.3 equiv) was added via a syringe. Pyridine (1.45 g, 4.0 equiv) was slowly added to the resulting mixture over 5 min. The resulting solution was stirred at room temperature for 12 h, hexane (20 mL) was then added and the precipitate was filtered off by using a Büchner funnel. The filtrate was concentrated under reduced pressure, and hexane (10 mL) was then added to the residue. The reaction mixture was concentrated and purified directly by column chromatography on silica gel (hexane as eluent) to give arylsilanes as colourless liquid.[11]

2-chlorobenzyl)tris((2,6-dimethylheptan-4-yl)oxy)silane (93a)

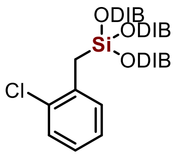

Colorless oil. **^{1}H NMR** (400 MHz, CDCl₃) δ 7.33 (dd, *J* = 7.7, 1.7 Hz, 1H), 7.27 (dd, *J* = 8.0, 1.4 Hz, 1H), 7.09 (td, *J* = 7.5, 1.5 Hz, 1H), 7.00 (td, *J* = 7.6, 1.8 Hz, 1H), 4.08 – 3.95 (m, 3H), 2.34 (s, 2H), 1.66 (dp, *J* = 13.4, 6.7 Hz, 6H), 1.35 (dt, *J* = 13.5, 6.7 Hz, 6H), 1.23 (ddd, *J* = 13.5, 7.6, 5.8 Hz, 6H), 0.84 (dd, *J* = 8.0, 6.6 Hz, 36H). **^{13}C NMR** (101 MHz, $CDCl_3$) δ 137.3, 133.9, 130.9, 129.4, 126.3, 125.8, 69.9, 46.8, 24.5, 23.1, 23.0, 19.9. **HRMS (ESI)** *m/z* Calcd for $C_{34}H_{63}ClNaO_3Si$ $[M+Na]^+$ 605.4127, Found: 605.4149.

(2-bromobenzyl)tris((2,6-dimethylheptan-4-yl)oxy)silane (94a)

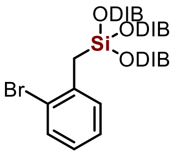

Colorless oil. **^{1}H NMR** (400 MHz, $CDCl_3$) δ 7.47 (dd, *J* = 8.1, 1.3 Hz, 1H), 7.36 (dd, *J* = 7.8, 1.7 Hz, 1H), 7.14 (td, *J* = 7.5, 1.4 Hz, 1H), 6.92 (td, *J* = 7.6, 1.7 Hz, 1H), 4.11 – 3.97 (m, 3H), 2.38 (s, 2H), 1.67 (dp, *J* = 13.4, 6.7 Hz, 6H), 1.36 (dt, *J* = 13.4, 6.7 Hz, 6H), 1.24 (ddd, *J* = 13.4, 7.6, 5.8 Hz, 6H), 0.85 (dd, *J* = 8.0, 6.6 Hz, 36H). **^{13}C NMR** (101 MHz, $CDCl_3$) δ 139.1, 132.7, 130.8, 127.0, 126.1, 125.2, 69.9, 46.8, 24.6, 23.1, 23.0, 22.9. **HRMS (ESI)** *m/z* Calcd for $C_{34}H_{63}BrNaO_3Si$ $[M+Na]^+$ 649.3622, Found: 649.3638.

2-((tris((2,6-dimethylheptan-4-yl)oxy)silyl)methyl)benzonitrile (95a)

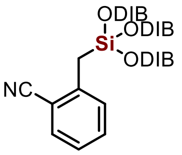

Colorless oil. **^{1}H NMR** (400 MHz, $CDCl_3$) δ 7.52 (d, *J* = 7.8 Hz, 1H), 7.45 – 7.34 (m, 2H), 7.14 (ddd, *J* = 8.2, 6.9, 1.9 Hz, 1H), 4.05 (p, *J* = 6.4 Hz, 3H), 2.42 (s, 2H), 1.64 (dp, *J* = 13.4, 6.7 Hz, 6H), 1.37 (dt, *J* = 13.5, 6.7 Hz, 6H), 1.26 (ddd, *J* = 13.5, 7.6, 5.8 Hz, 6H), 0.86 (dd, *J* = 8.1, 6.5 Hz, 36H). **^{13}C NMR** (101 MHz, $CDCl_3$) δ 143.9, 132.6, 132.0, 130.0, 124.9, 118.7, 112.9, 70.1, 46.7, 24.5, 23.0, 22.9, 22.1. **HRMS (ESI)** *m/z* Calcd for $C_{35}H_{63}NNaO_3Si$ $[M+Na]^+$ 596.4469, Found: 596.4485.

Tris((2,6-dimethylheptan-4-yl)oxy)(2-(trifluoromethyl)benzyl)silane (96a)

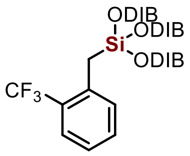

Colorless oil. **^{1}H NMR** (400 MHz, $CDCl_3$) δ 7.53 (dd, *J* = 19.7, 7.9 Hz, 1H), 7.36 (t, *J* = 7.6 Hz, 1H), 7.16 (t, *J* = 7.7 Hz, 1H), 4.04 (p, *J* = 7.2, 6.6 Hz, 3H), 2.18 (d, *J* = 1.9 Hz, 2H), 1.67 (dp, *J* = 13.3, 6.7 Hz, 6H), 1.43 – 1.31 (m, 6H), 1.32 – 1.20 (m, 6H), 0.86 (td, *J* = 6.9, 1.6 Hz, 36H). **^{13}C NMR** (101 MHz, $CDCl_3$) δ 138.5, 131.8, 131.2, 124.0 (q, *J* = 273.9 Hz), 126.0 (q, *J* = 5.7 Hz), 124.5, 70.0, 46.8, 24.5, 23.0, 23.0, 19.5. **^{19}F NMR** (376 MHz, $CDCl_3$) δ -59.8. **HRMS (ESI)** *m/z* Calcd for $C_{35}H_{63}F_3NaO_3Si$ $[M+Na]^+$ 639.4391, Found: 639.4345.

Tris((2,6-dimethylheptan-4-yl)oxy)(2-(trifluoromethoxy)benzyl)silane (97a)

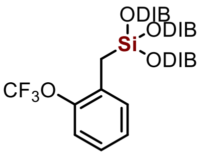

Colorless oil. **^{1}H NMR** (400 MHz, $CDCl_3$) δ 7.40 (dd, *J* = 7.3, 2.1 Hz, 1H), 7.18 – 7.03 (m, 3H), 4.02 (p, *J* = 6.4 Hz, 3H), 2.21 (s, 2H), 1.65 (dp, *J* = 13.4, 6.7 Hz, 6H), 1.42 – 1.29 (m, 6H), 1.23 (ddd, *J* = 14.8, 7.6, 5.8 Hz, 6H), 0.84 (dd, *J* = 8.6, 6.6 Hz, 36H). **^{13}C NMR** (101 MHz, $CDCl_3$) δ 147.41, 131.95, 131.62, 126.25, 125.73, 120.7 (q, *J* = 259.0 Hz), 69.9, 46.8, 24.6, 23.0, 22.9, 15.8. **^{19}F NMR** (376 MHz, $CDCl_3$) δ -56.9. **HRMS (ESI)** *m/z* Calcd for $C_{35}H_{63}F_3NaO_4Si$ $[M+Na]^+$ 655.4340, Found: 655.4365.

(2,3-difluorobenzyl)tris((2,6-dimethylheptan-4-yl)oxy)silane (98a)

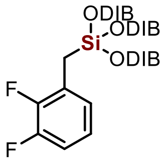

Colorless oil. **^{1}H NMR** (400 MHz, $CDCl_3$) δ 7.07 – 6.98 (m, 1H), 6.95 – 6.81 (m, 2H), 4.04 (p, *J* = 7.2, 6.6 Hz, 3H), 2.18 (d, *J* = 1.9 Hz, 2H), 1.67 (dp, *J* = 13.3, 6.7 Hz, 6H), 1.43 – 1.31 (m, 6H), 1.32 – 1.20 (m, 6H), 0.86 (td, *J* = 6.9, 1.6 Hz, 36H). **^{13}C NMR** (101 MHz, $CDCl_3$) δ 152.2 (d, *J* = 13.6 Hz), 149.9 (t, *J* = 12.9 Hz), 147.5 (d, *J* = 12.3 Hz), 128.9 (d, *J* = 13.2 Hz), 126.1 (t, *J* = 3.3 Hz), 123.1 (dd, *J* = 7.2, 4.6 Hz), 113.1 (d, *J* = 17.2 Hz), 70.0, 46.8, 24.6, 23.1, 22.9, 14.9 (t, *J* = 2.2 Hz). **^{19}F NMR** (376 MHz, $CDCl_3$) δ -139.8 (d, *J* = 21.0 Hz), -142.1 (d, *J* = 21.0 Hz). **HRMS (ESI)** *m/z* Calcd for $C_{34}H_{62}F_2NaO_3Si$ $[M+Na]^+$ 607.4328, Found: 607.4355.

(2-chloro-6-fluorobenzyl)tris((2,6-dimethylheptan-4-yl)oxy)silane (99a)

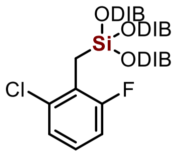

Colorless oil. **^{1}H NMR** (400 MHz $CDCl_3$) δ 7.10 (d, *J* = 8.0 Hz, 1H), 6.97 (td, *J* = 8.1, 5.9 Hz, 1H), 6.92 – 6.85 (m, 1H), 4.07 (p, *J* = 6.4 Hz, 3H), 2.33 (d, *J* = 2.9 Hz, 2H), 1.65 (dp, *J* = 13.4, 6.7 Hz, 6H), 1.35 (dt, *J* = 13.4, 6.7 Hz, 6H), 1.23 (ddd, *J* = 13.4, 7.5, 5.8 Hz, 6H), 0.88 – 0.82 (m, 38H). **^{13}C NMR** (101 MHz, $CDCl_3$) δ 161.1 (d, *J* = 246.4 Hz), 134.8 (d, *J* = 6.4 Hz), 126.7 (d, *J* = 19.8 Hz), 125.4 (d, *J* = 9.4 Hz), 124.8 (d, *J* = 3.3 Hz), 113.3 (d, *J* = 23.0 Hz), 70.0, 46.7, 24.5, 23.1, 23.0, 13.9 (d, *J* = 2.5 Hz). **^{19}F NMR** (376 MHz, $CDCl_3$) δ -110.2. **HRMS (ESI)** *m/z* Calcd for $C_{34}H_{62}ClFNaO_3Si$ $[M+Na]^+$ 623.4033, Found: 623.4058.

Tris(2,6-dimethylheptan-4-yl) (2-(2-methyl-1,3-dioxolan-2-yl)phenyl) silicate (101)

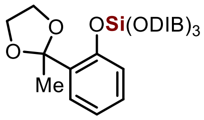

Following **general procedure 7.** **^{1}H NMR** (400 MHz, $CDCl_3$) δ 7.49 – 7.39 (m, 1H), 7.16 – 7.10 (m, 2H), 6.89 (ddd, *J* = 7.7, 5.5, 3.0 Hz, 1H), 4.27 – 4.16 (m, 3H), 4.02 – 3.91 (m, 2H), 3.80 – 3.72 (m, 2H), 1.77 – 1.66 (m, 9H), 1.46 (ddd, *J* = 13.6, 7.2, 6.3 Hz, 6H), 1.28 (ddd, *J* = 13.5, 7.7, 5.7 Hz, 6H), 0.85 (dd, *J* = 12.1, 6.6 Hz, 36H). **^{13}C NMR** (101 MHz, $CDCl_3$) δ 152.2, 131.9, 128.8, 126.9, 120.9, 120.8, 108.5, 70.8, 64.3, 46.6, 26.0, 24.5, 23.1, 22.9. **HRMS (ESI)** *m/z* Calcd for $C_{35}H_{64}NaO_6Si$ $[M+Na]^+$ 631.4364, Found: 631.4353.

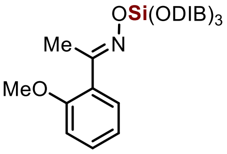

1-(2-methoxyphenyl)ethan-1-one O-(tris((2,6-dimethylheptan-4-yl)oxy)silyl) oxime (103)

Following **general procedure 1**. **^{1}H NMR** (400 MHz, $CDCl_3$) δ 7.42 – 7.28 (m, 2H), 7.00 – 6.85 (m, 2H), 4.20 – 4.02 (m, 3H), 3.83 (d, *J* = 13.4 Hz, 3H), 2.24 (d, *J* = 5.6 Hz, 3H), 1.94 – 1.70 (m, 6H), 1.50 (ddd, *J* = 13.5, 7.6, 5.9 Hz, 6H), 1.24 (ddd, *J* = 13.4, 8.0, 5.2 Hz, 6H), 0.87 (dd, *J* = 9.4, 6.6 Hz, 36H). **^{13}C NMR** (101 MHz, $CDCl_3$) δ 161.3, 157.7, 130.1, 130.0, 127.3, 120.3, 110.8, 70.5, 46.9, 24.4, 23.3, 22.7, 16.2. **HRMS (ESI)** *m/z* Calcd for $C_{36}H_{67}NNaO_5Si$ $[M+Na]^+$ 644.4681, Found: 644.4687.

2-(1,3-dioxolan-2-yl)phenyl tris(2,6-dimethylheptan-4-yl) silicate (106)

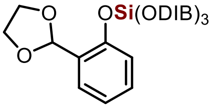

Following **general procedure 7**. **^{1}H NMR** (400 MHz, $CDCl_3$) δ 7.50 (dd, *J* = 7.7, 1.7 Hz, 1H), 7.23 – 7.15 (m, 2H), 6.97 (td, *J* = 7.3, 1.6 Hz, 1H), 6.20 (s, 1H), 4.20 – 4.08 (m, 5H), 3.99 – 3.94 (m, 2H), 1.83 – 1.66 (m, 6H), 1.46 (ddd, *J* = 13.6, 7.3, 6.2 Hz, 6H), 1.28 (ddd, *J* = 13.4, 7.7, 5.6 Hz, 6H), 0.86 (dd, *J* = 11.5, 6.6 Hz, 36H). **^{13}C NMR** (101 MHz, $CDCl_3$) δ 153.0, 129.8, 127.6, 126.9, 121.5, 119.4, 99.4, 71.0, 65.2, 46.6, 24.5, 23.1, 22.8. **HRMS (ESI)** *m/z* Calcd for $C_{36}H_{60}NaO_6Si$ $[M+Na]^+$ 645.4521, Found: 645.4535.

2-((triisopropylsilyl)oxy)benzaldehyde O-(tris((2,6-dimethylheptan-4-yl)oxy)silyl) oxime (108)

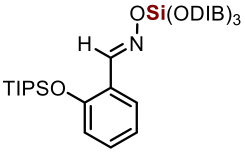

Following **general procedure 4**. **^{1}H NMR** (400 MHz, $CDCl_3$) δ 8.63 (s, 1H), 7.94 – 7.71 (m, 1H), 7.22 (td, *J* = 7.7, 1.8 Hz, 1H), 6.90 (td, *J* = 7.6, 2.2 Hz, 1H), 6.83 (dd, *J* = 7.8, 2.1 Hz, 1H), 4.24 – 4.10 (m, 3H), 1.93 – 1.73 (m, 6H), 1.57 – 1.45 (m, 6H), 1.36 – 1.27 (m, 3H), 1.31 – 1.19 (m, 6H), 1.12 (dd, *J* = 7.6, 2.7 Hz, 18H), 0.88 (dd, *J* = 8.7, 6.7 Hz, 36H). **^{13}C NMR** (101 MHz, $CDCl_3$) δ 154.7, 150.4, 131.0, 126.9, 123.1, 121.0, 119.1, 70.57, 47.0, 24.4, 23.3, 22.7, 18.1, 13.1. **HRMS (ESI)** *m/z* Calcd for $C_{43}H_{83}NNaO_5Si_2$ $[M+Na]^+$ 772.5702, Found: 772.5711.

2-(dimethylcarbamoyl)phenyl tris(2,6-dimethylheptan-4-yl) silicate (111)

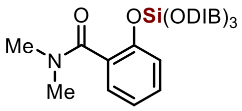

Following **general procedure 7**. **^{1}H NMR** (400 MHz, $CDCl_3$) δ 7.23 – 7.14 (m, 3H), 6.96 (td, *J* = 7.2, 1.5 Hz, 1H), 4.11 (ddd, *J* = 12.9, 7.2, 5.7 Hz, 3H), 3.07 (s, 3H), 2.85 (s, 3H), 1.77 – 1.64 (m, 6H), 1.48 – 1.35 (m, 6H), 1.27 (ddd, *J* = 14.9, 8.4, 6.4 Hz, 6H), 0.85 (dd, *J* = 11.6, 6.6 Hz, 36H). **^{13}C NMR** (101 MHz, $CDCl_3$) δ 169.4, 150.2, 129.6, 128.2, 128.2, 121.7, 119.6, 71.1, 46.5, 38.3, 35.0, 24.5, 23.1, 22.8. **HRMS (ESI)** *m/z* Calcd for $C_{36}H_{67}NNaO_5Si$ $[M+Na]^+$ 644.4681, Found: 644.4665.

2-methoxybenzoic (tris(2,6-dimethylheptan-4-yl) silicic) anhydride (113)

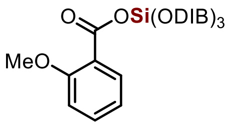

Following **general procedure 5**. **^{1}H NMR** (400 MHz, $CDCl_3$) δ 7.80 (dd, *J* = 7.9, 1.8 Hz, 1H), 7.45 (ddd, *J* = 8.5, 7.4, 1.8 Hz, 1H), 7.03 – 6.82 (m, 2H), 4.30 – 4.19 (m, 3H), 3.87 (s, 3H), 1.87 – 1.71 (m, 6H), 1.50 (ddd, *J* = 13.6, 7.5, 6.0 Hz, 6H), 1.28 (ddd, *J* = 13.5, 8.0, 5.3 Hz, 6H), 0.88 (t, *J* = 6.7 Hz, 36H). **^{13}C NMR** (101 MHz, $CDCl_3$) δ 163.9, 159.6, 133.5, 132.1, 121.5, 119.9, 111.9, 71.0, 55.8, 46.7, 24.5, 23.3, 22.8. **HRMS (ESI)** *m/z* Calcd for $C_{35}H_{64}NaO_6Si$ $[M+Na]^+$ 631.4364, Found: 631.4375.

2-((methoxyimino)methyl)benzoic (tris(2,6-dimethylheptan-4-yl) silicic) anhydride (116)

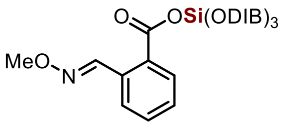

Following **general procedure 4**. **^{1}H NMR** (400 MHz, $CDCl_3$) δ 9.02 (s, 1H), 7.98 (ddd, *J* = 12.1, 7.9, 1.4 Hz, 2H), 7.53 (td, *J* = 7.6, 1.4 Hz, 1H), 7.42 (td, *J* = 7.6, 1.4 Hz, 1H), 4.28 – 4.20 (m, 3H), 3.99 (s, 3H), 1.89 – 1.69 (m, 6H), 1.50 (ddd, *J* = 13.5, 7.5, 6.0 Hz, 6H), 1.29 (ddd, *J* = 13.4, 7.9, 5.2 Hz, 6H), 0.88 (t, *J* = 6.3 Hz, 36H). **^{13}C NMR** (101 MHz, $CDCl_3$) δ 164.6, 148.3, 134.1, 132.5, 131.3, 130.0, 129.2, 127.6, 71.3, 62.1, 46.8, 24.5, 23.2, 22.8. **HRMS (ESI)** *m/z* Calcd for $C_{36}H_{65}NNaO_6Si$ $[M+Na]^+$ 658.4473, Found: 658.4483.

Methyl (E)-2-(4,4-bis((2,6-dimethylheptan-4-yl)oxy)-6-isobutyl-8-methyl-3,5-dioxa-2-aza-4-silanon-1-en-1-yl)benzoate (118)

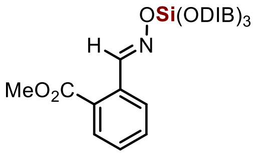

Following **general procedure 4**. **^{1}H NMR** (400 MHz, $CDCl_3$) δ 9.03 (s, 1H), 8.02 (dd, *J* = 7.9, 1.5 Hz, 1H), 7.96 (dd, *J* = 7.8, 1.5 Hz, 1H), 7.49 (td, *J* = 7.6, 1.6 Hz, 1H), 7.43 (td, *J* = 7.5, 1.5 Hz, 1H), 4.22 – 4.11 (m, 3H), 3.92 (s, 3H), 1.91 – 1.75 (m, 6H), 1.50 (ddd, *J* = 13.6, 7.6, 5.9 Hz, 6H), 1.25 (ddd, *J* = 13.4, 8.1, 5.2 Hz, 6H), 0.87 (dd, *J* = 9.2, 6.6 Hz, 36H). **^{13}C NMR** (101 MHz, $CDCl_3$) δ 167.2, 153.5, 133.3, 132.0, 130.8, 129.5, 129.4, 127.5, 70.7, 52.40, 46.9, 24.4, 23.3, 22.7. **HRMS (ESI)** *m/z* Calcd for $C_{36}H_{65}NNaO_6Si$ $[M+Na]^+$ 658.4473, Found: 658.4481.

3,3,5-trimethylcyclohexyl 2-(2-chlorophenyl)-2-((tris((2,6-dimethylheptan-4-yl)oxy)silyl)oxy)acetate (122a)

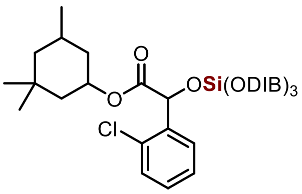

Following **general procedure 7**. **^{1}H NMR** (400 MHz, $CDCl_3$) δ 7.65 (dt, *J* = 7.6, 2.2 Hz, 1H), 7.32 (dd, *J* = 7.7, 1.6 Hz, 1H), 7.27 – 7.14 (m, 2H), 5.87 (s, 1H), 4.94 – 4.80 (m, 1H), 4.10 (dt, *J* = 11.6, 6.1 Hz, 3H), 2.05 – 1.94 (m, 0.5H), 1.83 – 1.63 (m, 8H), 1.58 – 1.47 (m, 0.5H), 1.41 (ddd, *J* = 26.8, 13.5, 6.7 Hz, 8H), 1.31 – 1.16 (m, 6H), 0.96 – 0.79 (m, 45H), 0.78 – 0.70 (m, 2H). **^{13}C NMR** (101 MHz, $CDCl_3$) δ 170.4 (d, *J* = 2.0 Hz), 137.4 (d, *J* = 2.0 Hz), 132.9, 129.3, 129.1, 129.0, 126.8, 71.9 (d, *J* = 5.1 Hz), 70.8 (d, *J* = 3.4 Hz), 70.4, 47.7 (d, *J* = 3.5 Hz), 46.7 (d, *J* = 8.7 Hz), 43.8 (d, *J* = 31.5 Hz), 40.3 (d, *J* = 35.2 Hz), 33.2 (d, *J* = 3.6 Hz), 32.3 (d, *J* = 4.4 Hz), 27.2 (d, *J* = 3.3 Hz), 25.6 (d, *J* = 2.6 Hz), 24.5 (d, *J* = 3.7 Hz), 23.2 (d, *J* = 2.4 Hz), 22.8 (d, *J* = 20.1 Hz), 22.4 (d, *J* = 5.2 Hz). **HRMS (ESI)** *m/z* Calcd for $C_{44}H_{79}ClNaO_6Si$ $[M+Na]^+$ 789.5227, Found: 789.5245.

Methyl 2-(2-chlorophenyl)-2-((tris((2,6-dimethylheptan-4-yl)oxy)silyl)oxy)acetate (147a)

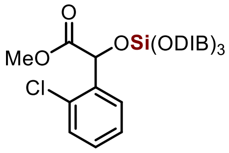

Following **general procedure 7.** **^{1}H NMR** (400 MHz, $CDCl_3$) δ 7.66 (dd, *J* = 7.5, 2.1 Hz, 1H), 7.36 (dd, *J* = 7.5, 1.8 Hz, 1H), 7.32 – 7.19 (m, 2H), 5.95 (s, 1H), 4.18 – 4.06 (m, 3H), 3.69 (s, 3H), 1.83 – 1.64 (m, 6H), 1.52 – 1.35 (m, 6H), 1.33 – 1.18 (m, 6H), 0.98 – 0.76 (m, 36H). **^{13}C NMR** (101 MHz, $CDCl_3$) δ 171.4, 137.1, 132.9, 129.4, 129.3, 129.2, 127.0, 70.8, 70.5, 52.1, 46.7 (d, *J* = 7.9 Hz), 24.5 (d, *J* = 2.2 Hz), 23.1, 22.8 (d, *J* = 14.8 Hz). **HRMS (ESI)** *m/z* Calcd for $C_{36}H_{65}ClNaO_6Si$ $[M+Na]^+$ 679.4131, Found: 679.4145.

4. Trialkoxysilane-induced Ir-catalyzed *para*-selective C–H borylation of arenes

4.1 *para* C−H borylation of benzaldehyde and acetophenones derivatives

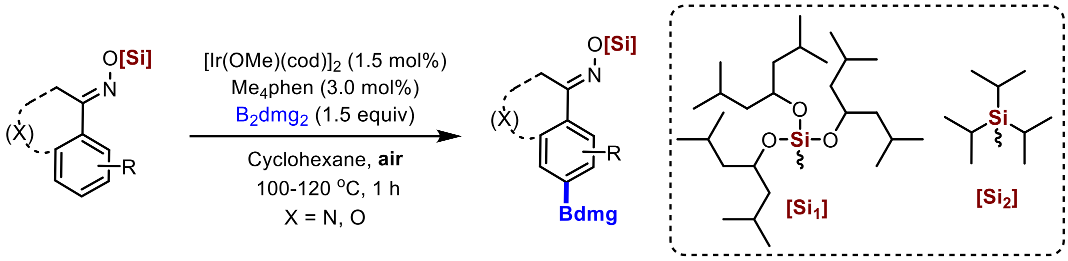

Benzaldehyde/acetophenone O-(tris((2,6-dimethylheptan-4-yl)oxy)silyl) oxime (0.2 mmol, 1.0 equiv), B_2dmg_2 (84.6 mg, 0.3 mmol, 1.5 equiv), $[Ir(cod)OMe]_2$ (2.0 mg, 0.003 mmol), Me_4phen (1.5 mg, 0.006 mmol), and cyclohexane (1.0 mL) were added to a 15 mL glass vial under air atmosphere. The glass vial was capped with a teflon pressure cap and placed into an aluminum block pre-heated to 100-120 °C for 1 h. After completion, cyclohexane was removed under reduced pressure and chromatographic separation with silica gel (15% ethyl acetate in hexane as eluent) gave the borylated product as colorless liquid. The *para:meta* ratio of products was reported from analysis of ^{1}H NMR.

2-methoxy-4-(4,4,6,6-tetramethyl-1,3,2-dioxaborinan-2-yl)benzaldehyde O-(tris((2,6-dimethylheptan-4-yl)oxy)silyl) oxime (3)

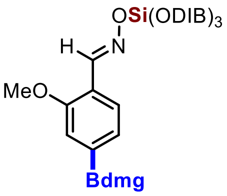

2-methoxybenzaldehyde O-(tris((2,6-dimethylheptan-4-yl)oxy)silyl) oxime **3a** (0.2 mmol, 121.6 mg), B_2dmg_2 (84.6 mg, 0.3 mmol, 1.5 equiv), $[Ir(cod)OMe]_2$ (2.0 mg, 0.003 mmol), Me_4phen (1.5 mg, 0.006 mmol), and cyclohexane (1.0 mL) were added to a 15 mL glass vial under air atmosphere. The glass vial was capped with a teflon pressure cap and placed into an aluminum block pre-heated to 100 °C for 1 h. After completion, cyclohexane was removed under reduced pressure and chromatographic separation with silica gel (15% ethyl acetate in hexane as eluent) gave the borylated product **3** (131.6 mg, 88%) as colorless liquid. The *para:meta* ratio of products was reported from analysis of ^{1}H NMR.

^{1}H NMR (400 MHz, $CDCl_3$) δ 8.61 (s, 1H), 7.82 (d, *J* = 7.7 Hz, 1H), 7.52 – 7.34 (m, 2H), 4.26 – 4.10 (m, 3H), 3.90 (s, 3H), 1.93 (s, 2H), 1.91 – 1.74 (m, 6H), 1.51 (ddd, *J* = 13.6, 7.6, 6.0 Hz, 6H), 1.44 (s, 12H), 1.26 (ddd, *J* = 13.5, 8.0, 5.2 Hz, 6H), 0.89 (dd, *J* = 8.6, 6.7 Hz, 36H). **^{13}C NMR** (101 MHz, $CDCl_3$) δ 157.1, 150.4, 126.2, 125.6, 122.8, 116.1, 71.2, 70.6, 55.8, 49.2, 46.9, 32.0, 24.4, 23.3, 22.8. **^{11}B NMR** (128 MHz, $CDCl_3$) δ 26.2. **HRMS (ESI)** *m/z* Calcd for $C_{42}H_{78}BNNaO_7Si$ $[M+Na]^+$ 770.5533, Found: 770.5541.

Same reaction was performed with the **2-methoxybenzaldehyde O-triisopropylsilyl oxime** under identical conditions. Outcomes: **p/m:** 14/1, **conv.** 85% (based on crude ^{1}H NMR analysis). 2-ethoxybenzaldehyde O-

triisopropylsilyl oxime

2-ethoxy-4-(4,4,6,6-tetramethyl-1,3,2-dioxaborinan-2-yl)benzaldehyde O-(tris((2,6-dimethylheptan-4-yl)oxy)silyl) oxime (4)

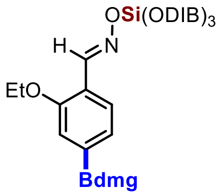

2-ethoxybenzaldehyde O-(tris((2,6-dimethylheptan-4-yl)oxy)silyl) oxime **4a** (0.2 mmol, 124.4 mg), B_2dmg_2 (84.6 mg, 0.3 mmol, 1.5 equiv), $[Ir(cod)OMe]_2$ (2.0 mg, 0.003 mmol), Me_4phen (1.5 mg, 0.006 mmol), and cyclohexane (1.0 mL) were added to a 15 mL glass vial under air atmosphere. The glass vial was capped with a teflon pressure cap and placed into an aluminum block pre-heated to 100 ºC for 1 h. After completion, cyclohexane was removed under reduced pressure and chromatographic separation with silica gel (15% ethyl acetate in hexane as eluent) gave the borylated product **4** (128.0 mg, 84%) as colorless liquid. The *para:meta* ratio of products was reported from analysis of ^{1}H NMR.

^{1}H NMR (400 MHz, $CDCl_3$) δ 8.65 (s, 1H), 7.82 (d, *J* = 7.7 Hz, 1H), 7.39 (d, *J* = 7.7 Hz, 1H), 7.34 (s, 1H), 4.28 – 4.07 (m, 5H), 1.92 (s, 2H), 1.91 – 1.77 (m, 6H), 1.57 – 1.46 (m, 6H), 1.45 – 1.41 (m, 15H), 1.31 – 1.20 (m, 6H), 0.89 (dd, *J* = 8.8, 6.5 Hz, 36H). **^{13}C NMR** (101 MHz, $CDCl_3$) δ 156.6, 150.7, 126.1, 125.5, 122.9, 117.3, 71.2, 70.6, 64.2, 49.2, 46.9, 32.0, 24.4, 23.3, 22.8, 15.0. **^{11}B NMR** (128 MHz, $CDCl_3$) δ 26.3. **HRMS (ESI)** *m/z* Calcd for $C_{43}H_{81}BNO_7Si$ $[M+H]^+$ 762.5807, Found: 762.5815.

Same reaction was performed with the **2-ethoxybenzaldehyde O-triisopropylsilyl oxime** under identical conditions. Outcomes: **p/m:** 12/1, **conv.** 90% (based on crude ^{1}H NMR analysis).

2-(2-methoxyethoxy)-4-(4,4,6,6-tetramethyl-1,3,2-dioxaborinan-2-yl)benzaldehyde O-(tris((2,6-dimethylheptan-4-yl)oxy)silyl) oxime (5)

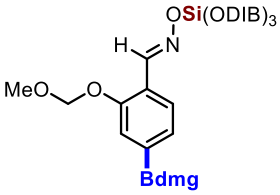

2-(2-methoxyethoxy)benzaldehyde O-(tris((2,6-dimethylheptan-4-yl)oxy)silyl) oxime **5a** (0.2 mmol, 130.4 mg), B_2dmg_2 (84.6 mg, 0.3 mmol, 1.5 equiv), $[Ir(cod)OMe]_2$ (2.0 mg, 0.003 mmol), Me_4phen (1.5 mg, 0.006 mmol), and cyclohexane (1.0 mL) were added to a 15 mL glass vial under air atmosphere. The glass vial was capped with a teflon pressure cap and placed into an aluminum block pre-heated to 100 ºC for 1 h. After completion, cyclohexane was removed under reduced pressure and chromatographic separation with silica gel (15% ethyl acetate in hexane as eluent) gave the borylated product **5** (134.6 mg, 85%) as colorless liquid. The *para:meta* ratio of products was reported from

analysis of ^{1}H NMR.

^{1}H NMR (400 MHz, $CDCl_3$) δ 8.66 (s, 1H), 7.83 (d, *J* = 7.7 Hz, 1H), 7.40 (d, *J* = 7.7 Hz, 1H), 7.35 (s, 1H), 4.25 – 4.18 (m, 2H), 4.20 – 4.11 (m, 3H), 3.83 – 3.74 (m, 2H), 3.46 (s, 3H), 1.91 (s, 2H), 1.90 – 1.76 (m, 6H), 1.50 (ddd, *J* = 13.5, 7.6, 5.9 Hz, 6H), 1.43 (s, 12H), 1.25 (ddd, *J* = 13.4, 8.1, 5.2 Hz, 6H), 0.88 (dd, *J* = 8.5, 6.6 Hz, 36H). **^{13}C NMR** (101 MHz, $CDCl_3$) δ 156.4, 150.4, 126.6, 125.5, 123.1, 117.5, 71.2, 71.2, 70.6, 68.3, 59.4, 49.1, 46.9, 31.9, 24.4, 23.3, 22.7. **^{11}B NMR** (128 MHz, $CDCl_3$) δ 25.9. **HRMS (ESI)** *m/z* Calcd for $C_{44}H_{82}BNNaO_8Si$ $[M+Na]^+$ 814.5795, Found: 814.5812.

Same reaction was performed with the **2-(2-methoxyethoxy)benzaldehyde O-triisopropylsilyl oxime** under identical conditions. Outcomes: **p/m:** 14/1, **conv.** 83% (based on crude ^{1}H NMR analysis).

2-(difluoromethoxy)-4-(4,4,6,6-tetramethyl-1,3,2-dioxaborinan-2-yl)benzaldehyde O-(tris((2,6-dimethylheptan-4-yl)oxy)silyl) oxime (6)

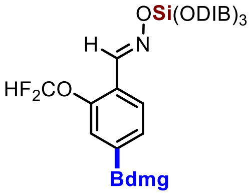

2-(difluoromethoxy)benzaldehyde O-(tris((2,6-dimethylheptan-4-yl)oxy)silyl) oxime **6a** (0.2 mmol, 128.8 mg), B_2dmg_2 (84.6 mg, 0.3 mmol, 1.5 equiv), $[Ir(cod)OMe]_2$ (2.0 mg, 0.003 mmol), Me_4phen (1.5 mg, 0.006 mmol), and cyclohexane (1.0 mL) were added to a 15 mL glass vial under air atmosphere. The glass vial was capped with a teflon pressure cap and placed into an aluminum block pre-heated to 100 ºC for 1 h. After completion, cyclohexane was removed under reduced pressure and chromatographic separation with silica gel (15% ethyl acetate in hexane as eluent) gave the borylated product **6** (138.0 mg, 88%) as colorless liquid. The *para:meta* ratio of products was reported from analysis of ^{1}H NMR.

^{1}H NMR (400 MHz, $CDCl_3$) δ 8.54 (s, 1H), 7.91 (d, *J* = 7.8 Hz, 1H), 7.64 (d, *J* = 7.7 Hz, 1H), 7.54 (s, 1H), 6.56 (t, *J* = 73.9 Hz, 1H), 4.21 – 4.10 (m, 3H), 1.93 (s, 2H), 1.90 – 1.75 (m, 6H), 1.50 (ddd, *J* = 13.6, 7.6, 5.9 Hz, 6H), 1.44 (s, 12H), 1.25 (ddd, *J* = 13.4, 8.1, 5.2 Hz, 6H), 0.88 (dd, *J* = 8.0, 6.6 Hz, 36H). **^{13}C NMR** (101 MHz, $CDCl_3$) δ 149.4, 149.2, 130.9, 126.1, 126.1, 124.3, 116.5 (t, *J* = 259.5 Hz), 71.5, 70.7, 49.1, 46.9, 31.9, 24.4, 23.3, 22.7. **^{19}F NMR** (376 MHz, $CDCl_3$) δ -80.3. **^{11}B NMR** (128 MHz, $CDCl_3$) δ 26.4. **HRMS (ESI)** *m/z* Calcd for $C_{42}H_{76}BF_2NNaO_7Si$ $[M+Na]^+$ 806.5344, Found: 806.5349.

Same reaction was performed with the **2-(difluoromethoxy)benzaldehyde O-triisopropylsilyl oxime** under identical conditions. Outcomes: **p/m:** 2/1, **conv.** 84% (based on crude ^{1}H NMR analysis).

4-(4,4,6,6-tetramethyl-1,3,2-dioxaborinan-2-yl)-2-(trifluoromethoxy)benzaldehyde O-(tris((2,6-

dimethylheptan-4-yl)oxy)silyl) oxime (7)

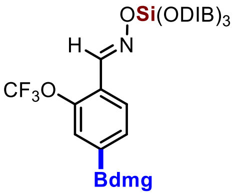

2-(trifluoromethoxy)benzaldehyde O-(tris((2,6-dimethylheptan-4-yl)oxy)silyl) oxime **7a** (0.2 mmol, 132.4 mg), B_2dmg_2 (84.6 mg, 0.3 mmol, 1.5 equiv), $[Ir(cod)OMe]_2$ (2.0 mg, 0.003 mmol), Me_4phen (1.5 mg, 0.006 mmol), and cyclohexane (1.0 mL) were added to a 15 mL glass vial under air atmosphere. The glass vial was capped with a teflon pressure cap and placed into an aluminum block pre-heated to 100 ºC for 1 h. After completion, cyclohexane was removed under reduced pressure and chromatographic separation with silica gel (15% ethyl acetate in hexane as eluent) gave the borylated product **7** (128.3 mg, 80%) as colorless liquid. The *para:meta* ratio of products was reported from analysis of ^{1}H NMR.

^{1}H NMR (400 MHz, $CDCl_3$) δ 8.50 (s, 1H), 7.95 (d, *J* = 7.8 Hz, 1H), 7.76 – 7.67 (m, 2H), 4.22 – 4.11 (m, 3H), 1.93 (s, 2H), 1.90 – 1.76 (m, 6H), 1.56 – 1.45 (m, 6H), 1.44 (s, 12H), 1.32 – 1.21 (m, 6H), 0.92 – 0.84 (m, 36H). **^{13}C NMR** (101 MHz, $CDCl_3$) δ 149.0, 147.1, 132.3, 127.2, 126.6, 126.0, 120.7 (d, *J* = 257.7 Hz), 71.5, 70.8, 49.1, 47.0, 31.9, 24.4, 23.3, 22.7. **^{19}F NMR** (376 MHz, $CDCl_3$) δ -57.5. **^{11}B NMR** (128 MHz, $CDCl_3$) δ 25.5. **HRMS (ESI)** *m/z* Calcd for $C_{42}H_{75}BF_3NNaO_7Si$ $[M+Na]^+$ 824.5250, Found: 824.5261.

Same reaction was performed with the **2-(trifluoromethoxy)benzaldehyde O-triisopropylsilyl oxime** under identical conditions. Outcomes: **p/m:** 2/1**, conv.** 83% (based on crude ^{1}H NMR analysis).

4-(4,4,6,6-tetramethyl-1,3,2-dioxaborinan-2-yl)-2-(trifluoromethyl)benzaldehyde O-(tris((2,6-dimethylheptan-4-yl)oxy)silyl) oxime (8)

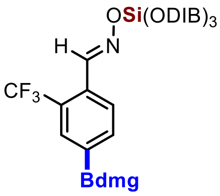

2-(trifluoromethyl)benzaldehyde O-(tris((2,6-dimethylheptan-4-yl)oxy)silyl) oxime **8a** (0.2 mmol, 129.2 mg), B_2dmg_2 (84.6 mg, 0.3 mmol, 1.5 equiv), $[Ir(cod)OMe]_2$ (2.0 mg, 0.003 mmol), dtbpy (1.6 mg, 0.006 mmol), and cyclohexane (1.0 mL) were added to a 15 mL glass vial under air atmosphere. The glass vial was capped with a teflon pressure cap and placed into an aluminum block pre-heated to 100 ºC for 1 h. After completion, cyclohexane was removed under reduced pressure and chromatographic separation with silica gel (15% ethyl acetate in hexane as eluent) gave the borylated product **8** (141.5 mg, 90%) as colorless liquid. The *para:meta* ratio of products was reported from analysis of ^{1}H NMR.

^{1}H NMR (400 MHz, $CDCl_3$) δ 8.61 (d, *J* = 2.4 Hz, 1H), 8.13 (s, 1H), 8.09 (d, *J* = 7.8 Hz, 1H), 7.96 (d, *J* = 7.9 Hz, 1H), 4.30 – 4.08 (m, 3H), 1.94 (s, 2H), 1.91 – 1.75 (m, 6H), 1.57 – 1.46 (m, 6H), 1.45 (s, 12H), 1.33 – 1.21 (m, 6H),

0.91 – 0.81 (m, 36H). **^{13}C NMR** (101 MHz, $CDCl_3$) δ 151.5 (d, J = 2.3 Hz), 137.1, 131.9 (d, J = 1.1 Hz), 131.2 (dd, J = 10.7, 5.3 Hz), 127.6 (q, J = 30.4 Hz), 126.3, 124.5 (d, J = 273.9 Hz), 71.5, 70.8, 49.2, 47.0, 31.9, 24.4, 23.3, 22.7. **^{19}F NMR** (376 MHz, $CDCl_3$) δ -57.9. **^{11}B NMR** (128 MHz, $CDCl_3$) δ 25.5. **HRMS (ESI)** *m/z* Calcd for $C_{42}H_{75}BF_3NNaO_6Si$ $[M+Na]^+$ 808.5301, Found: 808.5319.

Same reaction was performed with the **2-(trifluoromethyl)benzaldehyde O-triisopropylsilyl oxime** under identical conditions. Outcomes: **p/m:** 3/1**, conv.** 86% (based on crude ^{1}H NMR analysis).

2-chloro-4-(4,4,6,6-tetramethyl-1,3,2-dioxaborinan-2-yl)benzaldehyde O-(tris((2,6-dimethylheptan-4-yl)oxy)silyl) oxime (9)

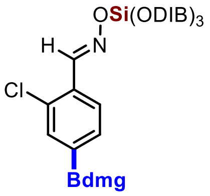

2-chlorobenzaldehyde O-(tris((2,6-dimethylheptan-4-yl)oxy)silyl) oxime **9a** (0.2 mmol, 122.5 mg), B_2dmg_2 (84.6 mg, 0.3 mmol, 1.5 equiv), $[Ir(cod)OMe]_2$ (2.0 mg, 0.003 mmol), Me_4phen (1.5 mg, 0.006 mmol), and cyclohexane (1.0 mL) were added to a 15 mL glass vial under air atmosphere. The glass vial was capped with a teflon pressure cap and placed into an aluminum block pre-heated to 100 ºC for 1 h. After completion, cyclohexane was removed under reduced pressure and chromatographic separation with silica gel (15% ethyl acetate in hexane as eluent) gave the borylated product **9** (129.4 mg, 86%) as colorless liquid. The *para:meta* ratio of products was reported from analysis of ^{1}H NMR.

^{1}H NMR (400 MHz, $CDCl_3$) δ 8.66 (s, 1H), 7.90 (d, J = 7.8 Hz, 1H), 7.82 (s, 1H), 7.66 (d, J = 7.8 Hz, 1H), 4.22 – 4.09 (m, 3H), 1.93 (s, 2H), 1.91 – 1.76 (m, 6H), 1.57 – 1.45 (m, 6H), 1.44 (s, 12H), 1.32 – 1.21 (m, 6H), 0.89 (dd, J = 8.2, 6.5 Hz, 36H). **^{13}C NMR** (101 MHz, $CDCl_3$) δ 151.8, 135.2, 133.6, 132.0, 131.4, 126.4, 71.4, 70.7, 49.1, 46.9, 31.9, 24.4, 23.3, 22.7. **^{11}B NMR** (128 MHz, $CDCl_3$) δ 26.6. **HRMS (ESI)** *m/z* Calcd for $C_{41}H_{75}BClNNaO_6Si$ $[M+Na]^+$ 774.5037, Found: 774.5034.

Same reaction was performed with the **2-chlorobenzaldehyde O-triisopropylsilyl oxime** under identical conditions. Outcomes: **p/m:** 6/1**, conv.** 82% (based on crude ^{1}H NMR analysis).

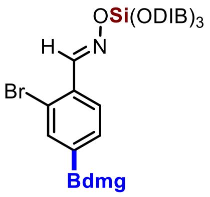

2-bromo-4-(4,4,6,6-tetramethyl-1,3,2-dioxaborinan-2-yl)benzaldehyde O-(tris((2,6-dimethylheptan-4-yl)oxy)silyl) oxime (10)

2-bromobenzaldehyde O-(tris((2,6-dimethylheptan-4-yl)oxy)silyl) oxime**10a** (0.2 mmol, 131.4 mg), B_2dmg_2 (84.6 mg, 0.3 mmol, 1.5 equiv), $[Ir(cod)OMe]_2$ (2.0 mg, 0.003 mmol), Me_4phen (1.5 mg, 0.006 mmol), and cyclohexane (1.0 mL) were added to a 15 mL glass vial under air atmosphere. The glass

vial was capped with a teflon pressure cap and placed into an aluminum block pre-heated to 120 ºC for 1 h. After completion, cyclohexane was removed under reduced pressure and chromatographic separation with silica gel (15% ethyl acetate in hexane as eluent) gave the borylated product **10** (124.3 mg, 78%) as colorless liquid. The *para:meta* ratio of products was reported from analysis of ^{1}H NMR.

^{1}H NMR (400 MHz, $CDCl_3$) δ 8.64 (s, 1H), 8.01 (s, 1H), 7.89 (d, *J* = 7.7 Hz, 1H), 7.70 (d, *J* = 7.8 Hz, 1H), 4.24 – 4.12 (m, 3H), 1.92 (s, 2H), 1.90 – 1.79 (m, 6H), 1.60 – 1.47 (m, 6H), 1.44 (s, 12H), 1.33 – 1.20 (m, 6H), 0.90 (dd, *J* = 8.5, 6.5 Hz, 36H). **^{13}C NMR** (101 MHz, $CDCl_3$) δ 154.1, 138.5, 133.0, 132.6, 126.8, 123.9, 71.4, 70.8, 49.1, 47.0, 31.9, 24.4, 23.3, 22.7. **^{11}B NMR** (128 MHz, $CDCl_3$) δ 25.8. **HRMS (ESI)** *m/z* Calcd for $C_{41}H_{75}BBrNNaO_6Si$ $[M+Na]^+$ 818.4532, Found: 818.4521.

Same reaction was performed with the **2-bromobenzaldehyde O-triisopropylsilyl oxime** under identical conditions. Outcomes: **p/m:** 5/1**, conv.** 80% (based on crude ^{1}H NMR analysis).

2-(piperidin-1-yl)-4-(4,4,6,6-tetramethyl-1,3,2-dioxaborinan-2-yl)benzaldehyde O-(tris((2,6-dimethylheptan-4-yl)oxy)silyl) oxime (11)

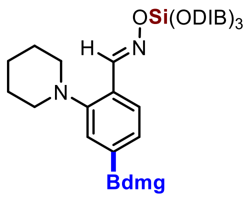

2-(piperidin-1-yl)benzaldehyde O-(tris((2,6-dimethylheptan-4-yl)oxy)silyl) oxime **11a** (0.2 mmol, 132.2 mg), B_2dmg_2 (84.6 mg, 0.3 mmol, 1.5 equiv), $[Ir(cod)OMe]_2$ (2.0 mg, 0.003 mmol), Me_4phen (1.5 mg, 0.006 mmol), and cyclohexane (1.0 mL) were added to a 15 mL glass vial under air atmosphere. The glass vial was capped with a teflon pressure cap and placed into an aluminum block pre-heated to 100 ºC for 1 h. After completion, cyclohexane was removed under reduced pressure and chromatographic separation with silica gel (15% ethyl acetate in hexane as eluent) gave the borylated product **11** (145.8 mg, 91%) as colorless liquid. The *para:meta* ratio of products was reported from analysis of ^{1}H NMR.

^{1}H NMR (400 MHz, $CDCl_3$) δ 8.57 (s, 1H), 7.81 (d, *J* = 7.7 Hz, 1H), 7.59 – 7.42 (m, 2H), 4.25 – 4.14 (m, 3H), 2.93 (t, *J* = 5.2 Hz, 4H), 1.92 (s, 2H), 1.91 – 1.80 (m, 6H), 1.73 (p, *J* = 5.7 Hz, 4H), 1.62 – 1.58 (m, 2H), 1.53 (ddd, *J* = 13.6, 7.6, 5.9 Hz, 6H), 1.44 (s, 12H), 1.26 (ddd, *J* = 13.4, 8.0, 5.2 Hz, 6H), 0.90 (dd, *J* = 8.7, 6.6 Hz, 36H). **^{13}C NMR** (101 MHz, $CDCl_3$) δ 152.7, 128.3, 128.1, 126.2, 124.4, 71.1, 70.6, 54.7, 49.2, 47.0, 32.0, 26.6, 24.4, 23.3, 22.7. **^{11}B NMR** (128 MHz, $CDCl_3$) δ 26.0. **HRMS (ESI)** *m/z* Calcd for $C_{46}H_{85}BN_2NaO_6Si$ $[M+Na]^+$ 823.6162, Found: 823.6184.

Same reaction was performed with the **2-(piperidin-1-yl)benzaldehyde O-triisopropylsilyl oxime** under identical conditions. Outcomes: **p/m:** 2/1, **conv.** 84% (based on crude ^{1}H NMR analysis).

2-morpholino-4-(4,4,6,6-tetramethyl-1,3,2-dioxaborinan-2-yl)benzaldehyde oxime (12)

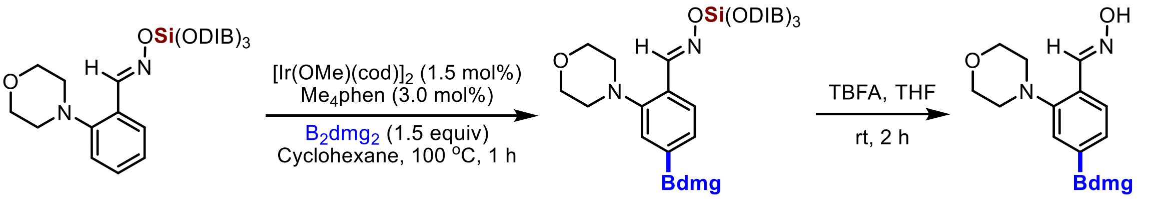

12a **12**, 81% **12aa**, 75%

2-morpholinobenzaldehyde O-(tris((2,6-dimethylheptan-4-yl)oxy)silyl) oxime **12a** (0.2 mmol, 132.2 mg), B_2dmg_2 (84.6 mg, 0.3 mmol, 1.5 equiv), $[Ir(cod)OMe]_2$ (2.0 mg, 0.003 mmol), Me_4phen (1.5 mg, 0.006 mmol), and cyclohexane (1.0 mL) were added to a 15 mL glass vial under air atmosphere. The glass vial was capped with a teflon pressure cap and placed into an aluminum block pre-heated to 100 ºC for 1 h. After completion, cyclohexane was removed under reduced pressure and chromatographic separation with silica gel (15% ethyl acetate in hexane as eluent) gave the borylated product **12** (129.7 mg, 81%) as colorless liquid. The *para:meta* ratio of products was reported from analysis of ^{1}H NMR.

2-morpholino-4-(4,4,6,6-tetramethyl-1,3,2-dioxaborinan-2-yl)benzaldehyde **12** (0.1 mmol, 80.1 mg) was dissolved in 1 mL THF and cooled to 0 ºC. The solution was treated with 200 µL TBAF (1M in THF, 2.00 equiv.) and warmed to room temperature. The mixture was stirred at room temperature for 2 h until the starting material was fully consumed monitored by TLC. The reaction was quenched with brine and extracted with diethyl ether (3 x 5 mL) and dried over $MgSO_4$. After filtration and removal of solvent, the crude was purified by silica gel chromatography to give a white solid **12aa** (25.8 mg, 75%).[6]

^{1}H NMR (400 MHz, $CDCl_3$) δ 8.53 (s, 1H), 7.71 (d, J = 7.6 Hz, 1H), 7.62 – 7.50 (m, 2H), 3.96 – 3.77 (m, 4H), 3.01 – 2.97 (m, 4H), 1.92 (s, 2H), 1.43 (s, 12H). **^{13}C NMR** (101 MHz, $CDCl_3$) δ 151.0, 148.6, 129.5, 128.0, 126.2, 124.6, 71.2, 67.4, 53.5, 49.1, 31.9. **^{11}B NMR** (128 MHz, $CDCl_3$) δ 26.0. **HRMS (ESI)** *m/z* Calcd for $C_{18}H_{27}BN_2NaO_4$ $[M+Na]^+$ 369.1956, Found: 369.1970.

Same reaction was performed with the **2-morpholinobenzaldehyde O-triisopropylsilyl oxime** under identical conditions. Outcomes: **p/m:** 5/1, **conv.** 83% (based on crude ^{1}H NMR analysis).

Ethyl-2-(4,4-bis((2,6-dimethylheptan-4-yl)oxy)-6-isobutyl-8-methyl-3,5-dioxa-2-aza-4-silanon-1-en-1-yl)-5-

(4,4,6,6-tetramethyl-1,3,2-dioxaborinan-2-yl)benzoate (13)

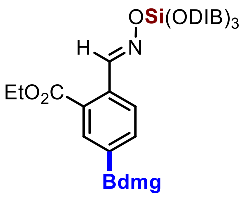

Ethyl-2-(4,4-bis((2,6-dimethylheptan-4-yl)oxy)-6-isobutyl-8-methyl-3,5-dioxa-2-aza-4-silanon-1-en-1-yl)benzoate **13a** (0.2 mmol, 130.0 mg), B_2dmg_2 (84.6 mg, 0.3 mmol, 1.5 equiv), $[Ir(cod)OMe]_2$ (2.0 mg, 0.003 mmol), Me_4phen (1.5 mg, 0.006 mmol), and cyclohexane (1.0 mL) were added to a 15 mL glass vial under air atmosphere. The glass vial was capped with a teflon pressure cap and placed into an aluminum block pre-heated to 100 ºC for 1 h. After completion, cyclohexane was removed under reduced pressure and chromatographic separation with silica gel (15% ethyl acetate in hexane as eluent) gave the borylated product **13** (131.1 mg, 83%) as colorless liquid. The *para:meta* ratio of products was reported from analysis of ^{1}H NMR.

^{1}H NMR (400 MHz, $CDCl_3$) δ 8.98 (s, 1H), 8.35 (d, *J* = 1.3 Hz, 1H), 7.97 (d, *J* = 7.8 Hz, 1H), 7.92 – 7.86 (m, 1H), 4.40 (q, *J* = 7.1 Hz, 2H), 4.22 – 4.11 (m, 3H), 1.93 (s, 2H), 1.90 – 1.74 (m, 6H), 1.50 (ddd, *J* = 13.5, 7.7, 5.9 Hz, 6H), 1.44 – 1.40 (m, 15H), 1.25 (ddd, *J* = 13.5, 8.1, 5.2 Hz, 6H), 0.88 (dd, *J* = 8.8, 6.6 Hz, 36H). **^{13}C NMR** (101 MHz, $CDCl_3$) δ 167.5, 153.8, 137.1, 136.1, 134.2, 129.3, 126.3, 71.4, 70.6, 61.4, 49.2, 46.9, 31.9, 24.4, 23.3, 22.7, 14.5. **^{11}B NMR** (128 MHz, $CDCl_3$) δ 26.2. **HRMS (ESI)** *m/z* Calcd for $C_{44}H_{80}BNNaO_8Si$ $[M+Na]^+$ 812.5638, Found: 812.5624.

Same reaction was performed with the **ethyl 2-((((triisopropylsilyl)oxy)imino)methyl)benzoate** under identical conditions. Outcomes: **p/m:** 2/1**, conv.** 85% (based on crude ^{1}H NMR analysis).

2-bromo-5-fluoro-4-(4,4,6,6-tetramethyl-1,3,2-dioxaborinan-2-yl)benzaldehyde O-(tris((2,6-dimethylheptan-4-yl)oxy)silyl) oxime (14)

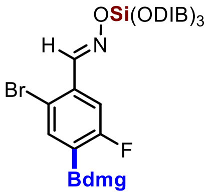

2-bromo-5-fluorobenzaldehyde O-(tris((2,6-dimethylheptan-4-yl)oxy)silyl) oxime **14a** (0.2 mmol, 135.0 mg), B_2dmg_2 (84.6 mg, 0.3 mmol, 1.5 equiv), $[Ir(cod)OMe]_2$ (2.0 mg, 0.003 mmol), Me_4phen (1.5 mg, 0.006 mmol), and cyclohexane (1.0 mL) were added to a 15 mL glass vial under air atmosphere. The glass vial was capped with a teflon pressure cap and placed into an aluminum block pre-heated to 100 ºC for 1 h. After completion, cyclohexane was removed under reduced pressure and chromatographic separation with silica gel (15% ethyl acetate in hexane as eluent) gave the borylated product **14** (132.0 mg, 81%) as colorless liquid. The *para:meta* ratio of products was reported from analysis of ^{1}H NMR.

Same reaction was performed with the **2-bromo-5-fluorobenzaldehyde O-triisopropylsilyl oxime** under identical

conditions. Outcomes: **p/m:** >20/1**, conv.** 85% (based on crude ^{1}H NMR analysis).

^{1}H NMR (400 MHz, $CDCl_3$) δ 8.53 (s, 1H), 7.87 (d, *J* = 5.5 Hz, 1H), 7.54 (d, *J* = 9.9 Hz, 1H), 4.22 – 4.09 (m, 3H), 1.94 (s, 2H), 1.89 – 1.74 (m, 6H), 1.55 – 1.44 (m, 6H), 1.44 (s, 12H), 1.33 – 1.20 (m, 6H), 0.88 (dd, *J* = 7.8, 6.6 Hz, 36H). **^{13}C NMR** (101 MHz, $CDCl_3$) δ 165.8 (d, *J* = 250.6 Hz), 153.2 (d, *J* = 2.1 Hz), 140.4 (d, *J* = 8.4 Hz), 134.8 (d, *J* = 9.1 Hz), 117.8 (d, *J* = 2.9 Hz), 114.1 (d, *J* = 28.4 Hz), 71.9, 70.8, 49.1, 46.9, 31.9, 24.4, 23.3, 22.7. **^{11}B NMR** (128 MHz, $CDCl_3$) δ 25.0. **HRMS (ESI)** *m/z* Calcd for $C_{41}H_{75}BBrFNO_6Si$ $[M+H]^+$ 814.4619, Found: 8814.4610.

5-fluoro-2-methyl-4-(4,4,6,6-tetramethyl-1,3,2-dioxaborinan-2-yl)benzaldehyde O-(tris((2,6-dimethylheptan-4-yl)oxy)silyl) oxime (15)

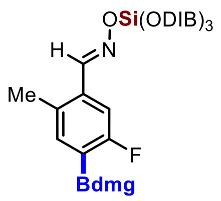

5-fluoro-2-methylbenzaldehyde O-(tris((2,6-dimethylheptan-4-yl)oxy)silyl) oxime **15a** (0.2 mmol, 122.0 mg), B_2dmg_2 (84.6 mg, 0.3 mmol, 1.5 equiv), $[Ir(cod)OMe]_2$ (2.0 mg, 0.003 mmol), Me_4phen (1.5 mg, 0.006 mmol), and cyclohexane (1.0 mL) were added to a 15 mL glass vial under air atmosphere. The glass vial was capped with a teflon pressure cap and placed into an aluminum block pre-heated to 100 ºC for 1 h. After completion, cyclohexane was removed under reduced pressure and chromatographic separation with silica gel (15% ethyl acetate in hexane as eluent) gave the borylated product **15** (124.5 mg, 83%) as colorless liquid. The *para:meta* ratio of products was reported from analysis of **^{1}H NMR**.

^{1}H NMR (400 MHz, $CDCl_3$) δ 8.42 (d, *J* = 1.6 Hz, 1H), 7.48 (d, *J* = 6.1 Hz, 1H), 7.42 (d, *J* = 10.3 Hz, 1H), 4.21 – 4.11 (m, 3H), 2.37 (s, 3H), 1.94 (s, 2H), 1.90 – 1.74 (m, 6H), 1.50 (ddd, *J* = 13.7, 7.6, 5.9 Hz, 6H), 1.45 (s, 12H), 1.26 (ddd, *J* = 13.4, 8.2, 5.0 Hz, 6H), 0.88 (dd, *J* = 8.1, 6.5 Hz, 36H). **^{13}C NMR** (101 MHz, $CDCl_3$) δ 165.3 (d, *J* = 247.3 Hz), 152.2 (d, *J* = 2.6 Hz), 138.2 (d, *J* = 8.2 Hz), 133.8 (d, *J* = 8.6 Hz), 131.4 (d, *J* = 3.2 Hz), 112.6 (d, *J* = 26.7 Hz), 71.5, 70.8, 49.1, 46.9, 31.9, 24.4, 23.3, 22.7, 18.7. **^{19}F NMR** (376 MHz, $CDCl_3$) δ -109.1. **^{11}B NMR** (128 MHz, $CDCl_3$) δ 26.0. **HRMS (ESI)** *m/z* Calcd for $C_{42}H_{77}BFNNaO_6Si$ $[M+Na]^+$ 772.5489, Found: 772.5507.

Same reaction was performed with the **5-fluoro-2-methylbenzaldehyde O-triisopropylsilyl oxime** under identical conditions. Outcomes: **p/m:** >20/1**, conv.** 88% (based on crude ^{1}H NMR analysis).

2,3-difluoro-4-(4,4,6,6-tetramethyl-1,3,2-dioxaborinan-2-yl)benzaldehyde O-(tris((2,6-dimethylheptan-4-yl)oxy)silyl) oxime (16)

2,3-difluorobenzaldehyde O-(tris((2,6-dimethylheptan-4-yl)oxy)silyl) oxime **16a** (0.2 mmol, 122.8 mg), B_2dmg_2

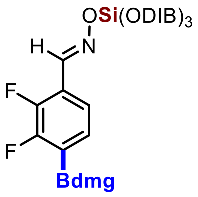

(84.6 mg, 0.3 mmol, 1.5 equiv), $[Ir(cod)OMe]_2$ (2.0 mg, 0.003 mmol), Me_4phen (1.5 mg, 0.006 mmol), and cyclohexane (1.0 mL) were added to a 15 mL glass vial under air atmosphere. The glass vial was capped with a teflon pressure cap and placed into an aluminum block pre-heated to 100 ºC for 1 h. After completion, cyclohexane was removed under reduced pressure and chromatographic separation with silica gel (15% ethyl acetate in hexane as eluent) gave the borylated product **16** (123.6 mg, 82%) as colorless liquid. The *para:meta* ratio of products was reported from analysis of ^{1}H NMR.

^{1}H NMR (400 MHz, $CDCl_3$) δ 8.44 (s, 1H), 7.61 – 7.51 (m, 1H), 7.43 – 7.35 (m, 1H), 4.21 – 4.10 (m, 3H), 1.96 (s, 2H), 1.91 – 1.75 (m, 6H), 1.50 (ddd, *J* = 13.9, 7.8, 6.1 Hz, 6H), 1.45 (s, 12H), 1.26 (td, *J* = 8.2, 4.0 Hz, 6H), 0.92 – 0.80 (m, 36H). **^{13}C NMR** (101 MHz, $CDCl_3$) δ 154.5 (dd, *J* = 251.8, 11.1 Hz), 150.3 (d, *J* = 16.1 Hz), 147.7 (d, *J* = 16.2 Hz), 147.3 (t, *J* = 3.9 Hz), 129.6 (dd, *J* = 7.3, 4.6 Hz), 123.4 (d, *J* = 7.8 Hz), 120.4 (d, *J* = 3.8 Hz), 71.7, 70.9, 49.0, 46.8, 31.7, 24.3, 23.2, 22.5. **^{19}F NMR** (376 MHz, $CDCl_3$) δ -130.8 (d, *J* = 20.1 Hz), -146.1 (d, *J* = 20.2 Hz). **^{11}B NMR** (128 MHz, $CDCl_3$) δ 25.7. **HRMS (ESI)** *m/z* Calcd for $C_{41}H_{74}BF_2NNaO_6Si$ $[M+Na]^+$ 776.5239, Found: 776.5250.

Same reaction was performed with the **2,3-difluorobenzaldehyde O-triisopropylsilyl oxime** under identical conditions. Outcomes: **p/m:** 5/1**, conv.** 86% (based on crude ^{1}H NMR analysis).

2-fluoro-6-methoxy-4-(4,4,6,6-tetramethyl-1,3,2-dioxaborinan-2-yl)benzaldehyde O-(tris((2,6-dimethylheptan-4-yl)oxy)silyl) oxime (17)

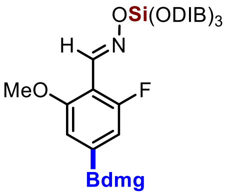

2-fluoro-6-methoxybenzaldehyde O-(tris((2,6-dimethylheptan-4-yl)oxy)silyl) oxime **17a** (0.2 mmol, 125.2 mg), B_2dmg_2 (84.6 mg, 0.3 mmol, 1.5 equiv), $[Ir(cod)OMe]_2$ (2.0 mg, 0.003 mmol), Me_4phen (1.5 mg, 0.006 mmol), and cyclohexane (1.0 mL) were added to a 15 mL glass vial under air atmosphere. The glass vial was capped with a teflon pressure cap and placed into an aluminum block pre-heated to 100 ºC for 1 h. After completion, cyclohexane was removed under reduced pressure and chromatographic separation with silica gel (15% ethyl acetate in hexane as eluent) gave the borylated product **17** (118.0 mg, 77%) as colorless liquid. The *para:meta* ratio of products was reported from analysis of ^{1}H NMR.

^{1}H NMR (400 MHz, $CDCl_3$) δ 8.52 (s, 1H), 7.17 (d, *J* = 10.3 Hz, 1H), 7.11 (s, 1H), 4.23 – 4.12 (m, 3H), 3.90 (s, 3H), 1.92 (s, 2H), 1.88 – 1.75 (m, 6H), 1.51 (ddd, *J* = 13.6, 7.6, 6.0 Hz, 6H), 1.43 (s, 12H), 1.25 (ddd, *J* = 13.4, 8.0, 5.3 Hz, 6H), 0.87 (dd, *J* = 9.0, 6.6 Hz, 36H). **^{13}C NMR** (101 MHz, $CDCl_3$) δ 160.9 (d, *J* = 257.8 Hz), 158.5 (d, *J* = 6.2 Hz), 148.3 (d, *J* = 4.2 Hz), 113.7 (d, *J* = 20.1 Hz), 111.2 (d, *J* = 14.3 Hz), 111.0 (d, *J* = 2.9 Hz), 71.5, 70.6, 56.2, 49.1,

47.0, 46.9, 31.9, 24.4, 23.3, 22.8. **^{19}F NMR** (376 MHz, $CDCl_3$) δ -109.2. **^{11}B NMR** (128 MHz, $CDCl_3$) δ 25.6. **HRMS (ESI)** *m/z* Calcd for $C_{42}H_{77}BFNNaO_7Si$ $[M+Na]^+$ 788.5439, Found: 788.5459.

Same reaction was performed with the **2-fluoro-6-methoxybenzaldehyde O-triisopropylsilyl oxime** under identical conditions. Outcomes: **p/m:** 13/1**, conv.** 85% (based on crude ^{1}H NMR analysis).

2-fluoro-4-(4,4,6,6-tetramethyl-1,3,2-dioxaborinan-2-yl)-6-((triisopropylsilyl)oxy)benzaldehyde O-(tris((2,6-dimethylheptan-4-yl)oxy)silyl) oxime (18)

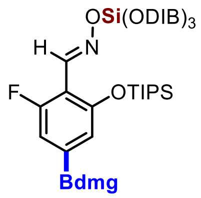

2-fluoro-6-((triisopropylsilyl)oxy)benzaldehyde O-(tris((2,6-dimethylheptan-4-yl)oxy)silyl) oxime **18a** (0.2 mmol, 153.7 mg), B_2dmg_2 (84.6 mg, 0.3 mmol, 1.5 equiv), $[Ir(cod)OMe]_2$ (2.0 mg, 0.003 mmol), Me_4phen (1.5 mg, 0.006 mmol), and cyclohexane (1.0 mL) were added to a 15 mL glass vial under air atmosphere. The glass vial was capped with a teflon pressure cap and placed into an aluminum block pre-heated to 100 ºC for 1 h. After completion, cyclohexane was removed under reduced pressure and chromatographic separation with silica gel (15% ethyl acetate in hexane as eluent) gave the borylated product **18** (145.3 mg, 80%) as colorless liquid. The *para:meta* ratio of products was reported from analysis of ^{1}H NMR.

^{1}H NMR (400 MHz, $CDCl_3$) δ 8.58 (d, *J* = 1.7 Hz, 1H), 7.13 – 7.11 (m, 2H), 4.22 – 4.10 (m, 3H), 1.91 (s, 2H), 1.90 – 1.75 (m, 6H), 1.51 (ddd, *J* = 13.6, 7.6, 6.0 Hz, 6H), 1.41 (s, 12H), 1.37 – 1.28 (m, 3H), 1.24 (ddd, *J* = 13.4, 8.0, 5.2 Hz, 6H), 1.13 (d, *J* = 7.4 Hz, 18H), 0.87 (dd, *J* = 8.8, 6.6 Hz, 36H). **^{13}C NMR** (101 MHz, $CDCl_3$) δ 161.0 (d, *J* = 259.7 Hz), 155.3 (d, *J* = 6.2 Hz), 148.8 (d, *J* = 6.5 Hz), 119.4 (d, *J* = 2.9 Hz), 113.5 (d, *J* = 19.9 Hz), 113.2 (d, *J* = 13.8 Hz), 71.3, 70.6, 48.9, 46.9, 31.9, 24.4, 23.3, 22.7, 18.1, 12.9. **^{19}F NMR** (376 MHz, $CDCl_3$) δ -106.8. **^{11}B NMR** (128 MHz, $CDCl_3$) δ 25.7. **HRMS (ESI)** *m/z* Calcd for $C_{50}H_{95}BFNNaO_7Si_2$ $[M+Na]^+$ 930.6616, Found: 930.6599.

Same reaction was performed with the **2-fluoro-6-((triisopropylsilyl)oxy)benzaldehyde O-triisopropylsilyl oxime** under identical conditions. Outcomes: **p/m:** 10/1**, conv.** 79% (based on crude ^{1}H NMR analysis).

2-fluoro-4-(4,4,6,6-tetramethyl-1,3,2-dioxaborinan-2-yl)-6-(trifluoromethyl)benzaldehyde O-(tris((2,6-dimethylheptan-4-yl)oxy)silyl) oxime (19)

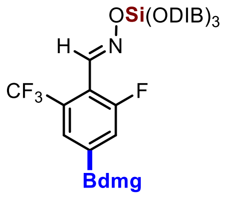

2-fluoro-6-(trifluoromethyl)benzaldehyde O-(tris((2,6-dimethylheptan-4-yl)oxy)silyl) oxime **19a** (0.2 mmol, 132.3 mg), B_2dmg_2 (84.6 mg, 0.3 mmol, 1.5 equiv), $[Ir(cod)OMe]_2$ (2.0 mg, 0.003 mmol), Me_4phen (1.5 mg, 0.006 mmol), and cyclohexane (1.0 mL) were added to a 15 mL glass vial under air atmosphere. The glass vial was capped with a teflon pressure cap and

placed into an aluminum block pre-heated to 100 ºC for 1 h. After completion, cyclohexane was removed under reduced pressure and chromatographic separation with silica gel (15% ethyl acetate in hexane as eluent) gave the borylated product **19** (127.0 mg, 79%) as colorless liquid. The *para:meta* ratio of products was reported from analysis of ^{1}H NMR.

^{1}H NMR (400 MHz, $CDCl_3$) δ 8.47 (d, *J* = 2.6 Hz, 1H), 7.93 (s, 1H), 7.74 (d, *J* = 10.5 Hz, 1H), 4.22 – 4.11 (m, 3H), 1.95 (s, 2H), 1.89 – 1.74 (m, 6H), 1.50 (ddd, *J* = 13.6, 7.6, 6.0 Hz, 6H), 1.45 (s, 12H), 1.25 (ddd, *J* = 13.5, 8.1, 5.2 Hz, 6H), 1.04 – 0.77 (m, 36H). **^{13}C NMR** (101 MHz, $CDCl_3$) δ 160.5 (d, *J* = 259.0 Hz), 148.0 (d, *J* = 4.6 Hz), 129.7 (dd, *J* = 30.5, 2.0 Hz), 126.7 (dd, *J* = 5.4, 3.1 Hz), 125.2 (d, *J* = 20.8 Hz), 119.8 (d, *J* = 13.9 Hz), 71.8, 70.8, 49.1, 46.9, 31.9, 24.4, 23.2, 22.7. **^{19}F NMR** (376 MHz, $CDCl_3$) δ -58.2, -108.6. **^{11}B NMR** (128 MHz, $CDCl_3$) δ 25.4. **HRMS (ESI)** *m/z* Calcd for $C_{42}H_{74}BF_4NNaO_6Si$ $[M+Na]^+$ 826.5207, Found: 826.5219.

Same reaction was performed with the **2-fluoro-6-(trifluoromethyl)benzaldehyde O-triisopropylsilyl oxime** under identical conditions. Outcomes: **p/m:** 4/1**, conv.** 82% (based on crude ^{1}H NMR analysis).

2-bromo-6-fluoro-4-(4,4,6,6-tetramethyl-1,3,2-dioxaborinan-2-yl)benzaldehyde O-(tris((2,6-dimethylheptan-4-yl)oxy)silyl) oxime (20)

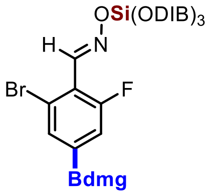

2-bromo-6-fluorobenzaldehyde O-(tris((2,6-dimethylheptan-4-yl)oxy)silyl) oxime **20a** (0.2 mmol, 135.0 mg), B_2dmg_2 (84.6 mg, 0.3 mmol, 1.5 equiv), $[Ir(cod)OMe]_2$ (2.0 mg, 0.003 mmol), Me_4phen (1.5 mg, 0.006 mmol), and cyclohexane (1.0 mL) were added to a 15 mL glass vial under air atmosphere. The glass vial was capped with a teflon pressure cap and placed into an aluminum block pre-heated to 100 ºC for 1 h. After completion, cyclohexane was removed under reduced pressure and chromatographic separation with silica gel (15% ethyl acetate in hexane as eluent) gave the borylated product **20** (122.2 mg, 75%) as colorless liquid. The *para:meta* ratio of products was reported from analysis of ^{1}H NMR.

^{1}H NMR (400 MHz, $CDCl_3$) δ 8.51 (s, 1H), 7.82 (s, 1H), 7.48 (d, *J* = 10.3 Hz, 1H), 4.22 – 4.12 (m, 3H), 1.92 (s, 2H), 1.89 – 1.74 (m, 6H), 1.50 (ddd, *J* = 13.5, 7.6, 5.9 Hz, 6H), 1.43 (s, 12H), 1.25 (ddd, *J* = 13.4, 8.0, 5.2 Hz, 6H), 0.88 (dd, *J* = 8.4, 6.6 Hz, 36H). **^{13}C NMR** (101 MHz, $CDCl_3$) δ 160.5 (d, *J* = 248.4 Hz), 151.3 (d, *J* = 5.2 Hz), 133.9, 124.0, 121.8, 120.4 (d, *J* = 20.3 Hz), 71.7, 70.8, 49.1, 46.9, 31.9, 24.4, 23.3, 22.7. **^{19}F NMR** (376 MHz, $CDCl_3$) δ -99.37 – -116.09 (m). **^{11}B NMR** (128 MHz, $CDCl_3$) δ 25.2. **HRMS (ESI)** *m/z* Calcd for $C_{41}H_{74}BFBrNNaO_6Si$ $[M+Na]^+$ 836.4438, Found: 836.4442.

Same reaction was performed with the **2-bromo-6-fluorobenzaldehyde O-triisopropylsilyl oxime** under identical

conditions. Outcomes: **p/m:** 5/1**, conv.** 80% (based on crude ^{1}H NMR analysis).

2-chloro-6-fluoro-4-(4,4,6,6-tetramethyl-1,3,2-dioxaborinan-2-yl)benzaldehyde O-(tris((2,6-dimethylheptan-4-yl)oxy)silyl) oxime (21)

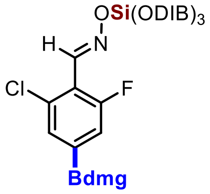

2-chloro-6-fluorobenzaldehyde O-(tris((2,6-dimethylheptan-4-yl)oxy)silyl) oxime **21a** (0.2 mmol, 126.1 mg), B_2dmg_2 (84.6 mg, 0.3 mmol, 1.5 equiv), $[Ir(cod)OMe]_2$ (2.0 mg, 0.003 mmol), Me_4phen (1.5 mg, 0.006 mmol), and cyclohexane (1.0 mL) were added to a 15 mL glass vial under air atmosphere. The glass vial was capped with a teflon pressure cap and placed into an aluminum block pre-heated to 100 ºC for 1 h. After completion, cyclohexane was removed under reduced pressure and chromatographic separation with silica gel (15% ethyl acetate in hexane as eluent) gave the borylated product **21** (123.3 mg, 80%) as colorless liquid. The *para:meta* ratio of products was reported from analysis of ^{1}H NMR.

^{1}**H NMR** (400 MHz, $CDCl_3$) δ 8.55 (s, 1H), 7.63 (s, 1H), 7.44 (d, *J* = 10.3 Hz, 1H), 4.22 – 4.12 (m, 1H), 1.93 (s, 2H), 1.89 – 1.73 (m, 6H), 1.50 (ddd, *J* = 13.6, 7.6, 5.9 Hz, 6H), 1.43 (s, 12H), 1.25 (ddd, *J* = 13.4, 8.0, 5.3 Hz, 6H), 0.87 (dd, *J* = 8.4, 6.6 Hz, 36H). ^{13}**C NMR** (101 MHz, $CDCl_3$) δ 160.7 (d, *J* = 260.7 Hz), 149.2 (d, *J* = 4.2 Hz), 134.3, 130.7 (d, *J* = 3.3 Hz), 120.2 (d, *J* = 14.7 Hz), 119.6 (d, *J* = 20.1 Hz), 71.7, 70.8, 49.1, 46.9, 31.9, 24.4, 23.3, 22.7. ^{19}**F NMR** (376 MHz, $CDCl_3$) δ -108.2. ^{11}**B NMR** (128 MHz, $CDCl_3$) δ 25.2. **HRMS (ESI)** *m/z* Calcd for $C_{41}H_{74}BFClNNaO_6Si$ $[M+Na]^+$ 792.4943, Found: 792.4963.

Same reaction was performed with the **2-chloro-6-fluorobenzaldehyde O-triisopropylsilyl oxime** under identical conditions. Outcomes: **p/m:** 5/1**, conv.** 82% (based on crude ^{1}H NMR analysis).

2-bromo-6-chloro-4-(4,4,6,6-tetramethyl-1,3,2-dioxaborinan-2-yl)benzaldehyde O-(tris((2,6-dimethylheptan-4-yl)oxy)silyl) oxime (22)

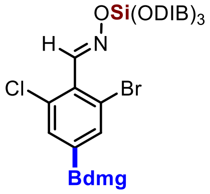

2-bromo-6-chlorobenzaldehyde O-(tris((2,6-dimethylheptan-4-yl)oxy)silyl) oxime **22a** (0.2 mmol, 138.3 mg), B_2dmg_2 (84.6 mg, 0.3 mmol, 1.5 equiv), $[Ir(cod)OMe]_2$ (2.0 mg, 0.003 mmol), Me_4phen (1.5 mg, 0.006 mmol), and cyclohexane (1.0 mL) were added to a 15 mL glass vial under air atmosphere. The glass vial was capped with a teflon pressure cap and placed into an aluminum block pre-heated to 100 ºC for 1 h. After completion, cyclohexane was removed under reduced pressure and chromatographic separation with silica gel (15% ethyl acetate in hexane as eluent) gave the borylated product **22** (124.7 mg, 75%) as colorless liquid. The *para:meta* ratio of products was reported from analysis of ^{1}H NMR.

^{1}**H NMR** (400 MHz, $CDCl_3$) δ 8.44 (s, 1H), 7.92 (s, 1H), 7.78 (s, 1H), 4.24 – 4.11 (m, 3H), 1.92 (s, 2H), 1.89 – 1.74

(m, 6H), 1.50 (ddd, J = 13.5, 7.5, 6.0 Hz, 6H), 1.43 (s, 12H), 1.26 (ddd, J = 13.4, 8.1, 5.3 Hz, 6H), 0.88 (dd, J = 9.4, 6.6 Hz, 36H). **^{13}C NMR** (101 MHz, $CDCl_3$) δ 152.5, 136.8, 134.5, 134.3, 71.7, 70.8, 49.1, 46.9, 31.9, 24.4, 23.3, 22.8. **^{11}B NMR** (128 MHz, $CDCl_3$) δ 25.7. **HRMS (ESI)** *m/z* Calcd for $C_{41}H_{74}BBrClNNaO_6Si$ $[M+Na]^+$ 852.4143, Found: 852.4172.

Same reaction was performed with the **2-bromo-6-chlorobenzaldehyde O-triisopropylsilyl oxime** under identical conditions. Outcomes: **p/m:** >20/1**, conv.** 80% (based on crude ^{1}H NMR analysis).

2,6-dibromo-4-(4,4,6,6-tetramethyl-1,3,2-dioxaborinan-2-yl)benzaldehyde O-(tris((2,6-dimethylheptan-4-yl)oxy)silyl) oxime (23)

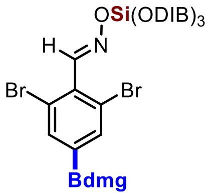

2,6-dibromobenzaldehyde O-(tris((2,6-dimethylheptan-4-yl)oxy)silyl) oxime **23a** (0.2 mmol, 147.2 mg), B_2dmg_2 (84.6 mg, 0.3 mmol, 1.5 equiv), $[Ir(cod)OMe]_2$ (2.0 mg, 0.003 mmol), Me_4phen (1.5 mg, 0.006 mmol), and cyclohexane (1.0 mL) were added to a 15 mL glass vial under air atmosphere. The glass vial was capped with a teflon pressure cap and placed into an aluminum block pre-heated to 100 ºC for 1 h. After completion, cyclohexane was removed under reduced pressure and chromatographic separation with silica gel (15% ethyl acetate in hexane as eluent) gave the borylated product **23** (126.1 mg, 72%) as colorless liquid. The *para:meta* ratio of products was reported from analysis of ^{1}H NMR.

^{1}H NMR (400 MHz, $CDCl_3$) δ 8.38 (s, 1H), 7.96 (s, 2H), 4.24 – 4.11 (m, 3H), 1.92 (s, 2H), 1.89 – 1.74 (m, 6H), 1.56 – 1.45 (m, 6H), 1.43 (s, 12H), 1.30 – 1.23 (m, 6H), 0.88 (dd, J = 9.7, 6.6 Hz, 36H). **^{13}C NMR** (101 MHz, $CDCl_3$) δ 153.7, 137.5, 133.4, 123.7, 71.7, 70.8, 49.1, 46.9, 31.9, 24.4, 23.3, 22.8. **^{11}B NMR** (128 MHz, $CDCl_3$) δ 25.0. **HRMS (ESI)** *m/z* Calcd for $C_{41}H_{74}BBr_2NNaO_6Si$ $[M+Na]^+$ 896.3637, Found: 896.3661.

Same reaction was performed with the **2,6-dibromobenzaldehyde O-triisopropylsilyl oxime** under identical conditions. Outcomes: **p/m:** >20/1**, conv.** 74% (based on crude ^{1}H NMR analysis).

2,6-dichloro-4-(4,4,6,6-tetramethyl-1,3,2-dioxaborinan-2-yl)benzaldehyde O-(tris((2,6-dimethylheptan-4-yl)oxy)silyl) oxime (24)

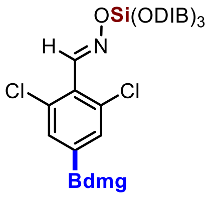

2,6-dichlorobenzaldehyde O-(tris((2,6-dimethylheptan-4-yl)oxy)silyl) oxime **24a** (0.2 mmol, 129.4 mg), B_2dmg_2 (84.6 mg, 0.3 mmol, 1.5 equiv), $[Ir(cod)OMe]_2$ (2.0 mg, 0.003 mmol), Me_4phen (1.5 mg, 0.006 mmol), and cyclohexane (1.0 mL) were added to a 15 mL glass vial under air atmosphere. The glass vial was capped with a teflon pressure cap and placed into an

aluminum block pre-heated to 100 ºC for 1 h. After completion, cyclohexane was removed under reduced pressure and chromatographic separation with silica gel (15% ethyl acetate in hexane as eluent) gave the borylated product **24** (130.6 mg, 83%) as colorless liquid. The *para:meta* ratio of products was reported from analysis of ^{1}H NMR.

^{1}H NMR (400 MHz, $CDCl_3$) δ 8.51 (s, 1H), 7.74 (s, 2H), 4.25 – 4.10 (m, 3H), 1.92 (s, 2H), 1.89 – 1.74 (m, 6H), 1.56 – 1.45 (m, 6H), 1.43 (s, 12H), 1.31 – 1.20 (m, 6H), 0.88 (dd, *J* = 9.3, 6.6 Hz, 36H). **^{13}C NMR** (101 MHz, $CDCl_3$) δ 151.1, 134.6, 133.8, 130.0, 71.7, 70.8, 49.1, 46.9, 31.9, 24.4, 23.3, 22.8. **^{11}B NMR** (128 MHz, $CDCl_3$) δ 25.2. **HRMS (ESI)** *m/z* Calcd for $C_{41}H_{74}BCl_2NNaO_6Si$ $[M+Na]^+$ 808.4648, Found: 808.4664.

Same reaction was performed with the **2,6-dichlorobenzaldehyde O-triisopropylsilyl oxime** under identical conditions. Outcomes: **p/m:** >20/1**, conv.** 82% (based on crude ^{1}H NMR analysis).

1-(2-ethoxy-4-(4,4,6,6-tetramethyl-1,3,2-dioxaborinan-2-yl)phenyl)ethan-1-one O-(tris((2,6-dimethylheptan-4-yl)oxy)silyl) oxime (25)

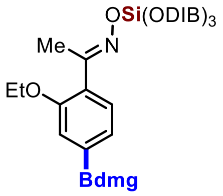

1-(2-ethoxyphenyl)ethan-1-one O-(tris((2,6-dimethylheptan-4-yl)oxy)silyl) oxime **25a** (0.2 mmol, 127.2 mg), B_2dmg_2 (84.6 mg, 0.3 mmol, 1.5 equiv), $[Ir(cod)OMe]_2$ (2.0 mg, 0.003 mmol), Me_4phen (1.5 mg, 0.006 mmol), and cyclohexane (1.0 mL) were added to a 15 mL glass vial under air atmosphere. The glass vial was capped with a teflon pressure cap and placed into an aluminum block pre-heated to 100 ºC for 1 h. After completion, cyclohexane was removed under reduced pressure and chromatographic separation with silica gel (15% ethyl acetate in hexane as eluent) gave the borylated product **25** (138.1 mg, 89%) as colorless liquid. The *para:meta* ratio of products was reported from analysis of ^{1}H NMR.

^{1}H NMR (400 MHz, $CDCl_3$) δ 7.39 – 7.29 (m, 3H), 4.20 – 4.06 (m, 5H), 2.22 (s, 3H), 1.92 (s, 2H), 1.90 – 1.75 (m, 6H), 1.49 (ddd, *J* = 13.5, 7.6, 5.9 Hz, 6H), 1.42 – 1.38 (m, 15H), 1.23 (ddd, *J* = 13.4, 8.1, 5.2 Hz, 6H), 0.86 (dd, *J* = 8.4, 6.6 Hz, 36H). **^{13}C NMR** (101 MHz, $CDCl_3$) δ 161.6, 156.4, 129.1, 129.0, 125.8, 116.6, 70.9, 70.3, 63.5, 49.0, 46.8, 31.8, 24.3, 23.2, 22.6, 16.1, 14.9. **^{11}B NMR** (128 MHz, $CDCl_3$) δ 26.1. **HRMS (ESI)** *m/z* Calcd for $C_{44}H_{82}BNNaO_7Si$ $[M+Na]^+$ 798.5846, Found: 798.5863.

Same reaction was performed with the **1-(2-ethoxyphenyl)ethan-1-one O-triisopropylsilyl oxime** under identical conditions. Outcomes: **p/m:** 10/1**, conv.** 81% (based on crude ^{1}H NMR analysis).

1-(2-chloro-4-(4,4,6,6-tetramethyl-1,3,2-dioxaborinan-2-yl)phenyl)ethan-1-one O-(tris((2,6-dimethylheptan-4-yl)oxy)silyl) oxime (26)

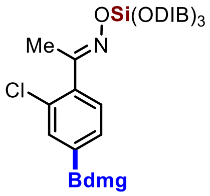

1-(2-chlorophenyl)ethan-1-one O-(tris((2,6-dimethylheptan-4-yl)oxy)silyl) oxime **26a** (0.2 mmol, 125.3 mg), B_2dmg_2 (84.6 mg, 0.3 mmol, 1.5 equiv), $[Ir(cod)OMe]_2$ (2.0 mg, 0.003 mmol), Me_4phen (1.5 mg, 0.006 mmol), and cyclohexane (1.0 mL) were added to a 15 mL glass vial under air atmosphere. The glass vial was capped with a teflon pressure cap and placed into an aluminum block pre-heated to 100 ºC for 1 h. After completion, cyclohexane was removed under reduced pressure and chromatographic separation with silica gel (15% ethyl acetate in hexane as eluent) gave the borylated product **26** (131.8 mg, 86%) as colorless liquid. The *para:meta* ratio of products was reported from analysis of ^{1}H NMR.

^{1}H NMR (400 MHz, $CDCl_3$) δ 7.82 (s, 1H), 7.67 (dd, *J* = 7.5, 1.1 Hz, 1H), 7.32 (d, *J* = 7.5 Hz, 1H), 4.21 – 4.10 (m, 3H), 2.26 (s, 3H), 1.93 (s, 2H), 1.90 – 1.75 (m, 6H), 1.56 – 1.44 (m, 6H), 1.44 (s, 12H), 1.30 – 1.19 (m, 6H), 0.88 (dd, *J* = 8.3, 6.6 Hz, 36H). **^{13}C NMR** (101 MHz, $CDCl_3$) δ 161.2, 138.6, 135.1, 132.2, 131.9, 129.7, 71.3, 70.6, 49.1, 47.0, 31.9, 24.4, 23.3, 22.8, 16.7. **^{11}B NMR** (128 MHz, $CDCl_3$) δ 25.6. **HRMS (ESI)** *m/z* Calcd for $C_{42}H_{77}BClNNaO_6Si$ $[M+Na]^+$ 788.5194, Found: 788.5201.

Same reaction was performed with the **1-(2-chlorophenyl)ethan-1-one O-triisopropylsilyl oxime** under identical conditions. Outcomes: **p/m:** 3/1**, conv.** 80% (based on crude ^{1}H NMR analysis).

1-(2-bromo-4-(4,4,6,6-tetramethyl-1,3,2-dioxaborinan-2-yl)phenyl)ethan-1-one O-(tris((2,6-dimethylheptan-4-yl)oxy)silyl) oxime (27)

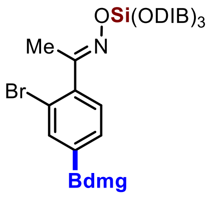

1-(2-bromophenyl)ethan-1-one O-(tris((2,6-dimethylheptan-4-yl)oxy)silyl) **27a** (0.2 mmol, 134.2 mg), B_2dmg_2 (84.6 mg, 0.3 mmol, 1.5 equiv), $[Ir(cod)OMe]_2$ (2.0 mg, 0.003 mmol), Me_4phen (1.5 mg, 0.006 mmol), and cyclohexane (1.0 mL) were added to a 15 mL glass vial under air atmosphere. The glass vial was capped with a teflon pressure cap and placed into an aluminum block pre-heated to 100 ºC for 1 h. After completion, cyclohexane was removed under reduced pressure and chromatographic separation with silica gel (15% ethyl acetate in hexane as eluent) gave the borylated product **27** (133.0 mg, 82%) as colorless liquid. The *para:meta* ratio of products was reported from analysis of ^{1}H NMR.

^{1}H NMR (400 MHz, $CDCl_3$) δ 8.03 (s, 1H), 7.74 (d, *J* = 7.5 Hz, 1H), 7.30 (dd, *J* = 7.4, 1.0 Hz, 1H), 4.26 – 4.11 (m, 3H), 2.28 (d, *J* = 1.1 Hz, 3H), 1.95 (s, 2H), 1.92 – 1.77 (m, 6H), 1.57 – 1.46 (m, 6H), 1.46 (s, 12H), 1.32 – 1.21 (m, 6H), 0.90 (dd, *J* = 8.1, 6.8 Hz, 36H). **^{13}C NMR** (101 MHz, $CDCl_3$) δ 161.9, 140.5, 138.2, 132.3, 129.7, 121.4, 71.2,

70.4, 49.0, 46.8, 31.8, 24.3, 23.2, 22.6, 16.8. **^{11}B NMR** (128 MHz, $CDCl_3$) δ 26.7. **HRMS (ESI)** *m/z* Calcd for $C_{42}H_{78}BBrNO_6Si$ $[M+H]^+$ 810.4869, Found: 810.4885.

Same reaction was performed with the **1-(2-bromophenyl)ethan-1-one O-triisopropylsilyl oxime** under identical conditions. Outcomes: **p/m:** 3/1**, conv.** 80% (based on crude ^{1}H NMR analysis).

1-(4-(4,4,6,6-tetramethyl-1,3,2-dioxaborinan-2-yl)-2-(trifluoromethoxy)phenyl)ethan-1-one O-(tris((2,6-dimethylheptan-4-yl)oxy)silyl) oxime (28)

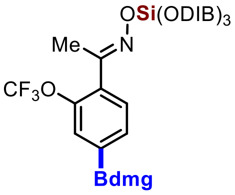

1-(2-(trifluoromethoxy)phenyl)ethan-1-one O-(tris((2,6-dimethylheptan-4-yl)oxy)silyl) oxime **28a** (0.2 mmol, 135.1 mg), B_2dmg_2 (84.6 mg, 0.3 mmol, 1.5 equiv), $[Ir(cod)OMe]_2$ (2.0 mg, 0.003 mmol), Me_4phen (1.5 mg, 0.006 mmol), and cyclohexane (1.0 mL) were added to a 15 mL glass vial under air atmosphere. The glass vial was capped with a teflon pressure cap and placed into an aluminum block pre-heated to 100 ºC for 1 h. After completion, cyclohexane was removed under reduced pressure and chromatographic separation with silica gel (15% ethyl acetate in hexane as eluent) gave the borylated product **28** (145.2 mg, 89%) as colorless liquid. The *para:meta* ratio of products was reported from analysis of ^{1}H NMR.

^{1}H NMR (400 MHz, $CDCl_3$) δ 7.93 – 7.61 (m, 2H), 7.51 – 7.16 (m, 1H), 4.26 – 3.94 (m, 3H), 2.22 (d, *J* = 26.2 Hz, 3H), 1.93 (d, *J* = 5.1 Hz, 2H), 1.88 – 1.70 (m, 6H), 1.54 – 1.37 (m, 18H), 1.30 – 1.10 (m, 6H), 1.00 – 0.73 (m, 36H). **^{13}C NMR** (101 MHz, $CDCl_3$) δ 159.3, 156.0, 146.9, 132.7, 132.0, 131.9, 131.4, 129.7, 128.1, 125.8, 125.7, 122.0, 119.5, 71.4 (d, *J* = 14.0 Hz), 70.5 (d, *J* = 20.0 Hz), 49.2, 46.9 (d, *J* = 16.2 Hz), 31.9, 24.4 (d, *J* = 7.3 Hz), 23.3 (d, *J* = 6.8 Hz), 22.7, 16.0. **^{19}F NMR** (376 MHz, $CDCl_3$) δ -56.7, -56.8. **^{11}B NMR** (128 MHz, $CDCl_3$) δ 25.6. **HRMS (ESI)** *m/z* Calcd for $C_{43}H_{77}BF_3NNaO_7Si$ $[M+Na]^+$ 838.5407, Found: 838.5400.

Same reaction was performed with the **1-(2-(trifluoromethoxy)phenyl)ethan-1-one O-triisopropylsilyl oxime** under identical conditions. Outcomes: **p/m:** 10/1**, conv.** 82% (based on crude ^{1}H NMR analysis).

5-(4,4,6,6-tetramethyl-1,3,2-dioxaborian-2-yl)-2,3-dihydro-1H-inden-1-one O-(tris((2,6-dimethylheptan-4-yl)oxy)silyl) oxime (29)

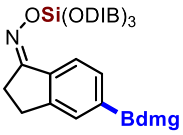

2,3-dihydro-1H-inden-1-one O-(tris((2,6-dimethylheptan-4-yl)oxy)silyl) oxime **29a** (0.2 mmol, 120.8 mg), B_2dmg_2 (84.6 mg, 0.3 mmol, 1.5 equiv), $[Ir(cod)OMe]_2$ (2.0 mg, 0.003 mmol), Me_4phen (1.5 mg, 0.006 mmol), and cyclohexane (1.0 mL) were added to a 15 mL glass vial under

air atmosphere. The glass vial was capped with a teflon pressure cap and placed into an aluminum block pre-heated to 120 ºC for 1 h. After completion, cyclohexane was removed under reduced pressure and chromatographic separation with silica gel (15% ethyl acetate in hexane as eluent) gave the borylated product **29** (119.0 mg, 80%) as colorless liquid. The *para:meta* ratio of products was reported from analysis of ^{1}H NMR.

^{1}H NMR (400 MHz, $CDCl_3$) δ 7.81 (s, 1H), 7.74 (d, *J* = 7.8 Hz, 1H), 7.67 (d, *J* = 7.7 Hz, 1H), 4.40 – 3.97 (m, 3H), 3.09 – 3.01 (m, 2H), 2.99 – 2.91 (m, 2H), 1.93 (s, 2H), 1.94 – 1.79 (m, 6H), 1.53 (ddd, *J* = 13.5, 7.6, 5.8 Hz, 6H), 1.45 (s, 12H), 1.26 (ddd, *J* = 13.4, 8.1, 5.1 Hz, 6H), 0.89 (dd, *J* = 8.7, 6.5 Hz, 36H). **^{13}C NMR** (101 MHz, $CDCl_3$) δ 167.8, 147.7, 138.3, 132.4, 131.0, 121.1, 71.1, 70.5, 49.1, 47.0, 32.0, 28.4, 26.9, 24.4, 23.3, 22.8. **^{11}B NMR** (128 MHz, $CDCl_3$) δ 26.0. **HRMS (ESI)** *m/z* Calcd for $C_{43}H_{78}BNNaO_6Si$ $[M+Na]^+$ 766.5584, Found: 766.5595.

Same reaction was performed with the **2,3-dihydro-1H-inden-1-one O-triisopropylsilyl oxime** under identical conditions. Outcomes: **p/m:** 4/1**, conv.** 85% (based on crude ^{1}H NMR analysis).

6-(4,4,6,6-tetramethyl-1,3,2-dioxaborinan-2-yl)-3,4-dihydronaphthalen-1(2*H*)-one O-(tris((2,6-dimethylheptan-4-yl)oxy)silyl) oxime (30)

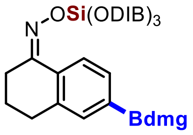

3,4-dihydronaphthalen-1(2H)-one O-(tris((2,6-dimethylheptan-4-yl)oxy)silyl) oxime **30a** (0.2 mmol, 123.6 mg), B_2dmg_2 (84.6 mg, 0.3 mmol, 1.5 equiv), $[Ir(cod)OMe]_2$ (2.0 mg, 0.003 mmol), Me_4phen (1.5 mg, 0.006 mmol), and cyclohexane (1.0 mL) were added to a 15 mL glass vial under air atmosphere. The glass vial was capped with a teflon pressure cap and placed into an aluminum block pre-heated to 120 ºC for 1 h. After completion, cyclohexane was removed under reduced pressure and chromatographic separation with silica gel (15% ethyl acetate in hexane as eluent) gave the borylated product **30** (133.4 mg, 88%) as colorless liquid. The *para:meta* ratio of products was reported from analysis of ^{1}H NMR.

^{1}H NMR (400 MHz, $CDCl_3$) δ 8.01 (d, *J* = 8.5 Hz, 1H), 7.62 – 7.60 (m, 2H), 4.23 – 4.12 (m, 3H), 2.90 – 2.72 (m, 4H), 1.92 (s, 2H), 1.91 – 1.79 (m, 6H), 1.59 – 1.47 (m, 6H), 1.44 (s, 12H), 1.26 (ddd, *J* = 13.4, 8.1, 5.1 Hz, 6H), 0.88 (dd, *J* = 7.5, 6.6 Hz, 36H). **^{13}C NMR** (101 MHz, $CDCl_3$) δ 159.3, 138.6, 134.1, 132.6, 131.4, 123.9, 71.0, 70.5, 49.2, 46.9, 32.0, 30.1, 24.9, 24.4, 23.3, 22.8, 21.8. **^{11}B NMR** (128 MHz, $CDCl_3$) δ 26.1. **HRMS (ESI)** *m/z* Calcd for $C_{44}H_{80}BNNaO_6Si$ $[M+Na]^+$ 780.5740, Found: 780.5749.

Same reaction was performed with the **3,4-dihydronaphthalen-1(2H)-one O-triisopropylsilyl oxime** under identical conditions. Outcomes: **p/m:** 5/1**, conv.** 83% (based on crude ^{1}H NMR analysis).

4-methyl-6-(4,4,6,6-tetramethyl-1,3,2-dioxaborinan-2-yl)-3,4-dihydronaphthalen-1(2*H*)-one O-(tris((2,6-dimethylheptan-4-yl)oxy)silyl) oxime (31)

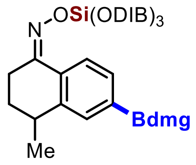

4-methyl-3,4-dihydronaphthalen-1(2H)-one O-(tris((2,6-dimethylheptan-4-yl)oxy)silyl) oxime **31a** (0.2 mmol, 126.3 mg), B_2dmg_2 (84.6 mg, 0.3 mmol, 1.5 equiv), $[Ir(cod)OMe]_2$ (2.0 mg, 0.003 mmol), Me_4phen (1.5 mg, 0.006 mmol), and cyclohexane (1.0 mL) were added to a 15 mL glass vial under air atmosphere. The glass vial was capped with a teflon pressure cap and placed into an aluminum block pre-heated to 120 ºC for 1 h. After completion, cyclohexane was removed under reduced pressure and chromatographic separation with silica gel (15% ethyl acetate in hexane as eluent) gave the borylated product **31** (128.2 mg, 83%) as colorless liquid. The *para:meta* ratio of products was reported from analysis of ^{1}H NMR.

^{1}H NMR (400 MHz, $CDCl_3$) δ 8.01 (d, *J* = 7.9 Hz, 1H), 7.68 (s, 1H), 7.62 (d, *J* = 7.9 Hz, 1H), 4.26 – 4.09 (m, 3H), 3.07 – 2.76 (m, 3H), 1.93 (s, 2H), 1.93 – 1.78 (m, 7H), 1.69 (dq, *J* = 12.2, 5.9 Hz, 1H), 1.52 (dt, *J* = 13.6, 6.8 Hz, 6H), 1.45 (d, *J* = 2.7 Hz, 12H), 1.35 – 1.20 (m, 9H), 0.88 (t, *J* = 7.1 Hz, 36H). **^{13}C NMR** (101 MHz, $CDCl_3$) δ 159.2, 143.3, 132.7, 131.8, 131.4, 124.0, 71.0, 70.5, 49.2, 47.0, 33.1, 32.0, 28.6, 24.4, 23.3, 22.8, 21.6, 20.8. **^{11}B NMR** (128 MHz, $CDCl_3$) δ 26.0. **HRMS (ESI)** *m/z* Calcd for $C_{45}H_{82}BNNaO_6Si$ $[M+Na]^+$ 794.5897, Found: 794.5903.

Same reaction was performed with the **4-methyl-3,4-dihydronaphthalen-1(2H)-one O-triisopropylsilyl oxime** under identical conditions. Outcomes: **p/m:** 8/1**, conv.** 86% (based on crude ^{1}H NMR analysis).

7-(4,4,6,6-tetramethyl-1,3,2-dioxaborinan-2-yl)chroman-4-one O-(tris((2,6-dimethylheptan-4-yl)oxy)silyl) oxime (32)

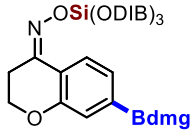

Chroman-4-one O-(tris((2,6-dimethylheptan-4-yl)oxy)silyl) oxime **32a** (0.2 mmol, 124.0 mg), B_2dmg_2 (84.6 mg, 0.3 mmol, 1.5 equiv), $[Ir(cod)OMe]_2$ (2.0 mg, 0.003 mmol), Me_4phen (1.5 mg, 0.006 mmol), and cyclohexane (1.0 mL) were added to a 15 mL glass vial under air atmosphere. The glass vial was capped with a teflon pressure cap and placed into an aluminum block pre-heated to 100 ºC for 1 h. After completion, cyclohexane was removed under reduced pressure and chromatographic separation with silica gel (15% ethyl acetate in hexane as eluent) gave the borylated product **32** (124.6 mg, 82%) as colorless liquid. The *para:meta* ratio of products was reported from analysis of ^{1}H NMR.

^{1}H NMR (400 MHz, $CDCl_3$) δ 7.91 (d, *J* = 7.9 Hz, 1H), 7.38 (s, 1H), 7.36 (dd, *J* = 7.8, 1.1 Hz, 1H), 4.24 – 4.11 (m, 5H), 2.98 (t, *J* = 6.2 Hz, 2H), 1.91 (s, 2H), 1.89 – 1.76 (m, 6H), 1.51 (ddd, *J* = 13.5, 7.6, 5.9 Hz, 6H), 1.42 (s, 12H), 1.26 (ddd, *J* = 13.4, 8.1, 5.1 Hz, 6H), 0.88 (t, *J* = 6.8 Hz, 36H). **^{13}C NMR** (101 MHz, $CDCl_3$) δ 156.2, 153.7, 126.3,

123.8, 123.0, 120.3, 71.1, 70.6, 65.1, 49.1, 46.9, 31.9, 24.6, 24.4, 23.3, 22.7. **^{11}B NMR** (128 MHz, $CDCl_3$) δ 27.1. **HRMS (ESI)** *m/z* Calcd for $C_{43}H_{78}BNNaO_7Si$ $[M+Na]^+$ 782.5533, Found: 782.5538.

Same reaction was performed with the **chroman-4-one O-triisopropylsilyl oxime** under identical conditions. Outcomes: **p/m:** 15/1**, conv.** 78% (based on crude ^{1}H NMR analysis).

2-(4,4,6,6-tetramethyl-1,3,2-dioxaborinan-2-yl)-6,7,8,9-tetrahydro-5H-benzo[7]annulen-5-one O-(tris((2,6-dimethylheptan-4-yl)oxy)silyl) oxime (33)

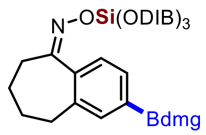

6,7,8,9-tetrahydro-5H-benzo[7]annulen-5-one O-(tris((2,6-dimethylheptan-4-yl)oxy)silyl) oxime **33a** (0.2 mmol, 126.4 mg), B_2dmg_2 (84.6 mg, 0.3 mmol, 1.5 equiv), $[Ir(cod)OMe]_2$ (2.0 mg, 0.003 mmol), Me_4phen (1.5 mg, 0.006 mmol), and cyclohexane (1.0 mL) were added to a 15 mL glass vial under air atmosphere. The glass vial was capped with a teflon pressure cap and placed into an aluminum block pre-heated to 120 ºC for 1 h. After completion, cyclohexane was removed under reduced pressure and chromatographic separation with silica gel (15% ethyl acetate in hexane as eluent) gave the borylated product **33** (140.5 mg, 91%) as colorless liquid. The *para:meta* ratio of products was reported from analysis of ^{1}H NMR.

^{1}H NMR (400 MHz, $CDCl_3$) δ 7.66 (dd, *J* = 7.6, 1.2 Hz, 1H), 7.59 (s, 1H), 7.46 (d, *J* = 7.6 Hz, 1H), 4.22 – 4.11 (m, 3H), 2.84 – 2.69 (m, 4H), 1.93 (s, 2H), 1.91 – 1.73 (m, 8H), 1.63 (q, *J* = 6.2 Hz, 2H), 1.51 (ddd, *J* = 13.5, 7.7, 5.9 Hz, 6H), 1.45 (s, 12H), 1.25 (ddd, *J* = 13.4, 8.1, 5.1 Hz, 6H), 0.88 (dd, *J* = 7.8, 6.6 Hz, 36H). **^{13}C NMR** (101 MHz, $CDCl_3$) δ 166.7, 138.3, 134.3, 131.8, 127.2, 71.0, 70.5, 49.2, 47.0, 32.2, 32.0, 27.0, 26.3, 24.4, 23.3, 22.8, 22.0. **^{11}B NMR** (128 MHz, $CDCl_3$) δ 27.7. **HRMS (ESI)** *m/z* Calcd for $C_{45}H_{82}BNNaO_6Si$ $[M+Na]^+$ 794.5897, Found: 794.5909.

Same reaction was performed with the **6,7,8,9-tetrahydro-5H-benzo[7]annulen-5-one O-triisopropylsilyl oxime** under identical conditions. Outcomes: **p/m:** 3/1**, conv.** 83% (based on crude ^{1}H NMR analysis).

1-(2-chloro-6-fluoro-4-(4,4,6,6-tetramethyl-1,3,2-dioxaborinan-2-yl)phenyl)ethan-1-one O-(tris((2,6-dimethylheptan-4-yl)oxy)silyl) oxime (34)

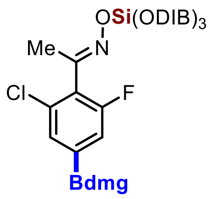

1-(2-chloro-6-fluorophenyl)ethan-1-one O-(tris((2,6-dimethylheptan-4-yl)oxy)silyl) oxime **34a** (0.2 mmol, 128.9 mg), B_2dmg_2 (84.6 mg, 0.3 mmol, 1.5 equiv), $[Ir(cod)OMe]_2$ (2.0 mg, 0.003 mmol), Me_4phen (1.5 mg, 0.006 mmol), and cyclohexane (1.0 mL) were added to a 15 mL glass vial under air atmosphere. The glass vial was capped with a teflon pressure cap and placed into an aluminum block pre-heated to 100 ºC for 1 h. After completion, cyclohexane was removed under reduced pressure and chromatographic separation with silica gel (15% ethyl acetate in hexane as eluent) gave the borylated product **34**

(133.3 mg, 85%) as colorless liquid. The *para:meta* ratio of products was reported from analysis of ^{1}H NMR.

^{1}H NMR (400 MHz, $CDCl_3$) δ 7.62 (s, 1H), 7.42 (d, *J* = 9.1 Hz, 1H), 4.22 – 4.10 (m, 3H), 2.19 (s, 3H), 1.93 (s, 2H), 1.89 – 1.74 (m, 6H), 1.55 – 1.43 (m, 6H), 1.43 (s, 12H), 1.32 – 1.18 (m, 6H), 0.87 (dd, *J* = 9.3, 6.6 Hz, 36H). **^{13}C NMR** (101 MHz, $CDCl_3$) δ 160.3 (d, *J* = 250.9 Hz), 155.3, 133.5 (d, *J* = 3.7 Hz), 130.2 (d, *J* = 3.1 Hz), 127.1 (d, *J* = 19.7 Hz), 118.9 (d, *J* = 20.0 Hz), 71.6, 70.6, 49.1, 46.9, 31.9, 24.4, 23.3, 22.7, 16.3. **^{19}F NMR** (376 MHz, $CDCl_3$) δ -113.5. **^{11}B NMR** (128 MHz, $CDCl_3$) δ 25.4. **HRMS (ESI)** *m/z* Calcd for $C_{42}H_{76}BClFNNaO_6Si$ $[M+Na]^+$ 806.5100, Found: 806.5107.

Same reaction was performed with the **1-(2-chloro-6-fluorophenyl)ethan-1-one O-triisopropylsilyl oxime** under identical conditions. Outcomes: **p/m:** 5/1**, conv.** 77% (based on crude ^{1}H NMR analysis).

1-(2-fluoro-6-methoxy-4-(4,4,6,6-tetramethyl-1,3,2-dioxaborinan-2-yl)phenyl)ethan-1-one O-(tris((2,6-dimethylheptan-4-yl)oxy)silyl) oxime (35)

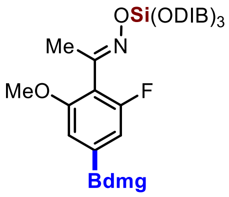

1-(2-fluoro-6-methoxyphenyl)ethan-1-one O-(tris((2,6-dimethylheptan-4-yl)oxy)silyl) oxime **35a** (0.2 mmol, 128.0 mg), B_2dmg_2 (84.6 mg, 0.3 mmol, 1.5 equiv), $[Ir(cod)OMe]_2$ (2.0 mg, 0.003 mmol), Me_4phen (1.5 mg, 0.006 mmol), and cyclohexane (1.0 mL) were added to a 15 mL glass vial under air atmosphere. The glass vial was capped with a teflon pressure cap and placed into an aluminum block pre-heated to 100 °C for 1 h. After completion, cyclohexane was removed under reduced pressure and chromatographic separation with silica gel (15% ethyl acetate in hexane as eluent) gave the borylated product **35** (135.7 mg, 87%) as colorless liquid. The *para:meta* ratio of products was reported from analysis of ^{1}H NMR.

^{1}H NMR (400 MHz, $CDCl_3$) δ 7.16 (d, *J* = 9.1 Hz, 1H), 7.10 (s, 1H), 4.22 – 4.08 (m, 3H), 3.83 (s, 3H), 2.14 (s, 3H), 1.92 (s, 2H), 1.88 – 1.74 (m, 6H), 1.48 (ddd, *J* = 13.6, 7.6, 6.0 Hz, 6H), 1.43 (s, 12H), 1.23 (ddd, *J* = 13.4, 8.0, 5.2 Hz, 6H), 0.86 (dd, *J* = 8.8, 6.6 Hz, 36H). **^{13}C NMR** (101 MHz, $CDCl_3$) δ 160.2 (d, *J* = 248.2 Hz), 158.1 (d, *J* = 6.0 Hz), 155.1, 117.4 (d, *J* = 18.7 Hz), 113.2 (d, *J* = 20.4 Hz), 110.9 (d, *J* = 2.7 Hz), 71.3, 70.5, 55.9, 49.1, 46.9, 31.9, 24.4, 23.3, 22.7, 16.5. **^{19}F NMR** (376 MHz, $CDCl_3$) δ -115.9. **^{11}B NMR** (128 MHz, $CDCl_3$) δ 25.9. **HRMS (ESI)** *m/z* Calcd for $C_{43}H_{80}BFNO_7Si$ $[M+H]^+$ 780.5776, Found: 780.5802.

Same reaction was performed with the **1-(2-fluoro-6-methoxyphenyl)ethan-1-one O-triisopropylsilyl oxime** under identical conditions. Outcomes: **p/m:** 5/1**, conv.** 80% (based on crude ^{1}H NMR analysis).

1-(2-bromo-5-fluoro-4-(4,4,6,6-tetramethyl-1,3,2-dioxaborinan-2-yl)phenyl)ethan-1-one O-(tris((2,6-dimethylheptan-4-yl)oxy)silyl) oxime (36)

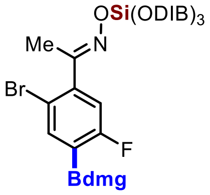

1-(2-bromo-5-fluorophenyl)ethan-1-one O-(tris((2,6-dimethylheptan-4-yl)oxy)silyl) oxime **36a** (0.2 mmol, 137.8 mg), B_2dmg_2 (84.6 mg, 0.3 mmol, 1.5 equiv), $[Ir(cod)OMe]_2$ (2.0 mg, 0.003 mmol), Me_4phen (1.5 mg, 0.006 mmol), and cyclohexane (1.0 mL) were added to a 15 mL glass vial under air atmosphere. The glass vial was capped with a teflon pressure cap and placed into an aluminum block pre-heated to 100 ºC for 1 h. After completion, cyclohexane was removed under reduced pressure and chromatographic separation with silica gel (15% ethyl acetate in hexane as eluent) gave the borylated product **36** (129.3 mg, 78%) as colorless liquid. The *para:meta* ratio of products was reported from analysis of ^{1}H NMR.

^{1}H NMR (400 MHz, $CDCl_3$) δ 7.68 (d, *J* = 5.3 Hz, 1H), 7.01 (d, *J* = 9.2 Hz, 1H), 4.19 – 4.08 (m, 3H), 2.25 (s, 3H), 1.95 (s, 2H), 1.88 – 1.74 (m, 6H), 1.57 – 1.41 (m, 18H), 1.24 (ddd, *J* = 13.4, 8.1, 5.2 Hz, 6H), 0.87 (t, *J* = 7.1 Hz, 36H). **^{13}C NMR** (101 MHz, $CDCl_3$) δ 165.7 (d, *J* = 251.7 Hz), 161.0, 142.3 (d, *J* = 8.8 Hz), 140.3 (d, *J* = 8.7 Hz), 118.0 (d, *J* = 27.4 Hz), 115.6 (d, *J* = 3.1 Hz), 71.8, 70.6, 49.1, 46.9, 31.9, 24.4, 23.3, 22.7, 16.7. **^{11}B NMR** (128 MHz, $CDCl_3$) δ 25.2. **^{19}F NMR** (376 MHz, $CDCl_3$) δ -107.52. **HRMS (ESI)** *m/z* Calcd for $C_{42}H_{76}BBrFNNaO_6Si$ $[M+Na]^+$ 850.4595, Found: 850.4611.

Same reaction was performed with the **1-(2-bromo-5-fluorophenyl)ethan-1-one O-triisopropylsilyl oxime** under identical conditions. Outcomes: **p/m:** >20/1**, conv.** 81% (based on crude ^{1}H NMR analysis).

1-(2-chloro-5-fluoro-4-(4,4,6,6-tetramethyl-1,3,2-dioxaborinan-2-yl)phenyl)ethan-1-one O-(tris((2,6-dimethylheptan-4-yl)oxy)silyl) oxime (37)

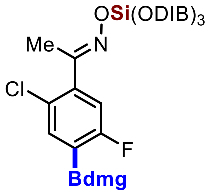

1-(2-chloro-5-fluorophenyl)ethan-1-one O-(tris((2,6-dimethylheptan-4-yl)oxy)silyl) oxime **37a** (0.2 mmol, 128.9 mg), B_2dmg_2 (84.6 mg, 0.3 mmol, 1.5 equiv), $[Ir(cod)OMe]_2$ (2.0 mg, 0.003 mmol), Me_4phen (1.5 mg, 0.006 mmol), and cyclohexane (1.0 mL) were added to a 15 mL glass vial under air atmosphere. The glass vial was capped with a teflon pressure cap and placed into an aluminum block pre-heated to 100 ºC for 1 h. After completion, cyclohexane was removed under reduced pressure and chromatographic separation with silica gel (15% ethyl acetate in hexane as eluent) gave the borylated product **37** (130.2 mg, 83%) as colorless liquid. The *para:meta* ratio of products was reported from analysis of ^{1}H NMR.

^{1}H NMR (400 MHz, $CDCl_3$) δ 7.68 (d, *J* = 5.4 Hz, 1H), 7.01 (d, *J* = 9.2 Hz, 1H), 4.18 – 4.09 (m, 3H), 2.25 (s, 3H), 1.95 (s, 2H), 1.88 – 1.74 (m, 6H), 1.54 – 1.40 (m, 18H), 1.24 (ddd, *J* = 13.4, 8.1, 5.1 Hz, 6H), 0.87 (t, *J* = 7.0 Hz,

36H). **^{13}C NMR** (101 MHz, $CDCl_3$) δ 165.09 (d, *J* = 251.1 Hz), 160.19, 140.14 (d, *J* = 8.8 Hz), 137.09 (d, *J* = 8.7 Hz), 127.05 (d, *J* = 3.2 Hz), 117.59 (d, *J* = 27.7 Hz), 71.78, 70.63, 49.08, 46.94, 31.87, 24.41, 23.30, 22.72, 16.40. **^{11}B NMR** (128 MHz, $CDCl_3$) δ 25.4. **^{19}F NMR** (376 MHz, $CDCl_3$) δ -107.42. **HRMS (ESI)** *m/z* Calcd for $C_{42}H_{76}BClFNNaO_6Si$ $[M+Na]^+$ 806.5100, Found: 806.5107.

Same reaction was performed with the **1-(2-chloro-5-fluorophenyl)ethan-1-one O-triisopropylsilyl oxime** under identical conditions. Outcomes: **p/m:** >20/1**, conv.** 84% (based on crude ^{1}H NMR analysis).

4.2 *para* C−H borylation of benzoic acid, benzyl alcohol and phenol derivatives

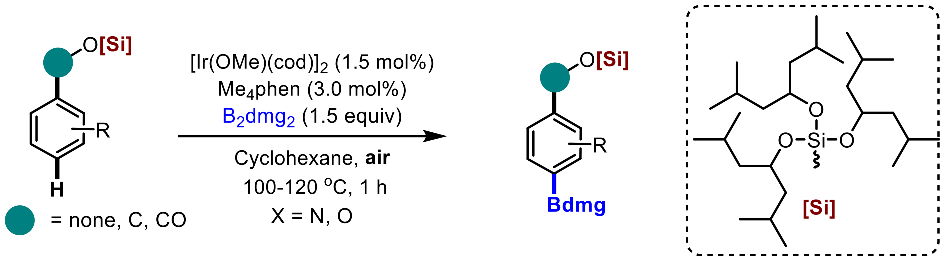

2-chloro-4-(4,4,6,6-tetramethyl-1,3,2-dioxaborinan-2-yl)benzoic (tris(2,6-dimethylheptan-4-yl) silicic) anhydride (39)

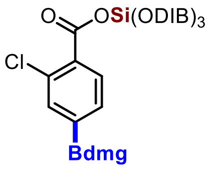

2-chlorobenzoic (tris(2,6-dimethylheptan-4-yl) silicic) anhydride **38a** (0.2 mmol, 122.7 mg), B_2dmg_2 (84.6 mg, 0.3 mmol, 1.5 equiv), $[Ir(cod)OMe]_2$ (2.0 mg, 0.003 mmol), Me_4phen (1.5 mg, 0.006 mmol), and cyclohexane (1.0 mL) were added to a 15 mL glass vial under air atmosphere. The glass vial was capped with a teflon pressure cap and placed into an aluminum block pre-heated to 100 °C for 1 h. After completion, cyclohexane was removed under reduced pressure and chromatographic separation with silica gel (15% ethyl acetate in hexane as eluent) gave the borylated product **39** (132.6 mg, 88%) as colorless liquid. The *para:meta* ratio of products was reported from analysis of ^{1}H NMR.

^{1}H NMR (400 MHz, $CDCl_3$) δ 7.88 (d, *J* = 1.0 Hz, 1H), 7.80 (d, *J* = 7.7 Hz, 1H), 7.71 (dd, *J* = 7.6, 1.1 Hz, 1H), 4.31 – 4.19 (m, 3H), 1.92 (s, 2H), 1.87 – 1.72 (m, 6H), 1.57 – 1.44 (m, 6H), 1.43 (s, 12H), 1.35 – 1.23 (m, 6H), 0.89 (dd, *J* = 6.9, 5.4 Hz, 36H). **^{13}C NMR** (101 MHz, $CDCl_3$) δ 163.7, 136.2, 133.2, 132.2, 131.5, 130.6, 71.4, 71.1, 48.9, 46.6, 31.7, 24.4, 23.1, 22.6. **^{11}B NMR** (128 MHz, $CDCl_3$) δ 25.3. **HRMS (ESI)** *m/z* Calcd for $C_{41}H_{74}BClNaO_7Si$ $[M+Na]^+$ 775.4878, Found: 775.4870.

2-bromo-4-(4,4,6,6-tetramethyl-1,3,2-dioxaborinan-2-yl)benzoic (tris(2,6-dimethylheptan-4-yl) silicic) anhydride (40)

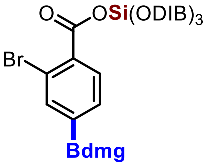

2-bromobenzaldehyde O-(tris((2,6-dimethylheptan-4-yl)oxy)silyl) oxime **38b** (0.2 mmol, 131.6 mg), B_2dmg_2 (84.6 mg, 0.3 mmol, 1.5 equiv), $[Ir(cod)OMe]_2$ (2.0 mg, 0.003 mmol), Me_4phen (1.5 mg, 0.006 mmol), and cyclohexane (1.0 mL) were added to a 15 mL glass vial under air atmosphere. The glass vial was capped with a teflon pressure cap and placed into an aluminum block pre-heated to 100 ºC for 1 h. After completion, cyclohexane was removed under reduced pressure and chromatographic separation with silica gel (15% ethyl acetate in hexane as eluent) gave the borylated product **40** (118.1 mg, 74%) as colorless liquid. The *para:meta* ratio of products was reported from analysis of ^{1}H NMR.

^{1}H NMR (400 MHz, $CDCl_3$) δ 8.08 (s, 1H), 7.78 –7.74 (m, 2H), 4.34 – 4.19 (m, 3H), 1.92 (s, 2H), 1.88 – 1.69 (m, 6H), 1.58 – 1.47 (m, 6H), 1.43 (s, 12H), 1.34 – 1.25 (m, 6H), 1.00 – 0.74 (m, 36H). **^{13}C NMR** (101 MHz, $CDCl_3$) δ 164.2, 139.7, 134.3, 132.2, 130.7, 121.5, 71.5, 71.3, 49.1, 46.7, 31.9, 24.5, 23.3, 22.8. **^{11}B NMR** (128 MHz, $CDCl_3$) δ 25.6. **HRMS (ESI)** *m/z* Calcd for $C_{41}H_{74}BBrNaO_7Si$ $[M+Na]^+$ 819.4372, Found: 819.4388.

4-(4,4,6,6-tetramethyl-1,3,2-dioxaborinan-2-yl)-2-(trifluoromethoxy)benzoic (tris(2,6-dimethylheptan-4-yl) silicic) anhydride (41)

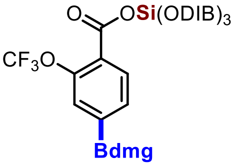

2-(trifluoromethoxy)benzoic (tris(2,6-dimethylheptan-4-yl) silicic) anhydride **38c** (0.2 mmol, 132.6 mg), B_2dmg_2 (84.6 mg, 0.3 mmol, 1.5 equiv), $[Ir(cod)OMe]_2$ (2.0 mg, 0.003 mmol), Me_4phen (1.5 mg, 0.006 mmol), and cyclohexane (1.0 mL) were added to a 15 mL glass vial under air atmosphere. The glass vial was capped with a teflon pressure cap and placed into an aluminum block pre-heated to 100 ºC for 1 h. After completion, cyclohexane was removed under reduced pressure and chromatographic separation with silica gel (15% ethyl acetate in hexane as eluent) gave the borylated product **41** (133.3 mg, 83%) as colorless liquid. The *para:meta* ratio of products was reported from analysis of ^{1}H NMR.

^{1}H NMR (400 MHz, $CDCl_3$) δ 7.79 (dd, J = 7.7, 1.2 Hz, 1H), 7.74 – 7.61 (m, 2H), 4.29 – 4.09 (m, 3H), 1.85 (s, 2H), 1.76 – 1.62 (m, 6H), 1.46 – 1.36 (m, 6H), 1.35 (s, 12H), 1.26 – 1.14 (m, 6H), 0.79 (dd, J = 6.8, 5.4 Hz, 36H). **^{13}C NMR** (101 MHz, $CDCl_3$) δ 162.4, 147.7, 132.1, 131.2, 127.5, 127.4, 124.5, 120.6 (d, J = 257.4 Hz), 71.6, 71.3, 49.1, 46.7, 31.9, 24.5, 23.2, 22.8. **^{19}F NMR** (376 MHz, $CDCl_3$) δ -57.0. **^{11}B NMR** (128 MHz, $CDCl_3$) δ 25.6. **HRMS (ESI)** *m/z* Calcd for $C_{42}H_{74}BF_3NaO_8Si$ $[M+Na]^+$ 825.5090, Found: 825.5139.

4-(4,4,6,6-tetramethyl-1,3,2-dioxaborinan-2-yl)-2-(trifluoromethyl)benzoic (tris(2,6-dimethylheptan-4-yl) silicic) anhydride (42)

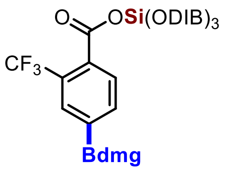

2-(trifluoromethyl)benzoic (tris(2,6-dimethylheptan-4-yl) silicic) anhydride **38d** (0.2 mmol, 129.4 mg), B_2dmg_2 (84.6 mg, 0.3 mmol, 1.5 equiv), $[Ir(cod)OMe]_2$ (2.0 mg, 0.003 mmol), Me_4phen (1.5 mg, 0.006 mmol), and cyclohexane (1.0 mL) were added to a 15 mL glass vial under air atmosphere. The glass vial was capped with a teflon pressure cap and placed into an aluminum block pre-heated to 100 ºC for 1 h. After completion, cyclohexane was removed under reduced pressure and chromatographic separation with silica gel (15% ethyl acetate in hexane as eluent) gave the borylated product **42** (141.6 mg, 90%) as colorless liquid. The *para:meta* ratio of products was reported from analysis of ^{1}**H NMR**.

^{1}**H NMR** (400 MHz, $CDCl_3$) δ 7.88 (d, *J* = 7.7 Hz, 1H), 7.84 – 7.68 (m, 2H), 4.29 – 4.18 (m, 3H), 1.94 (s, 2H), 1.85 – 1.71 (m, 6H), 1.55 – 1.44 (m, 6H), 1.44 (s, 12H), 1.34 – 1.26 (m, 6H), 0.88 (dd, *J* = 6.9, 5.3 Hz, 36H). ^{13}**C NMR** (101 MHz, $CDCl_3$) δ 162.2, 147.5, 131.9, 131.0, 127.4, 127.2, 120.5 (d, *J* = 257.4 Hz), 71.5, 71.1, 48.9, 46.5, 31.7, 24.3, 23.0, 22.6. ^{19}**F NMR** (376 MHz, $CDCl_3$) δ -57.0. ^{11}**B NMR** (128 MHz, $CDCl_3$) δ 25.2. **HRMS (ESI)** *m/z* Calcd for $C_{42}H_{74}BF_3NaO_7Si$ $[M+Na]^+$ 809.5141, Found: 809.5153.

2-methyl-4-(4,4,6,6-tetramethyl-1,3,2-dioxaborinan-2-yl)benzoic (tris(2,6-dimethylheptan-4-yl) silicic) anhydride (43)

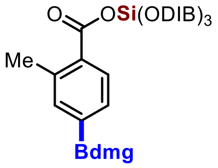

2-methylbenzoic (tris(2,6-dimethylheptan-4-yl) silicic) anhydride **38e** (0.2 mmol, 118.6 mg), B_2dmg_2 (84.6 mg, 0.3 mmol, 1.5 equiv), $[Ir(cod)OMe]_2$ (2.0 mg, 0.003 mmol), Me_4phen (1.5 mg, 0.006 mmol), and cyclohexane (1.0 mL) were added to a 15 mL glass vial under air atmosphere. The glass vial was capped with a teflon pressure cap and placed into an aluminum block pre-heated to 100 ºC for 1 h. After completion, cyclohexane was removed under reduced pressure and chromatographic separation with silica gel (15% ethyl acetate in hexane as eluent) gave the borylated product **43** (120.2 mg, 82%) as colorless liquid. The *para:meta* ratio of products was reported from analysis of ^{1}**H NMR**.

^{1}**H NMR** (400 MHz, $CDCl_3$) δ 7.93 (d, *J* = 7.7 Hz, 1H), 7.74 – 7.62 (m, 2H), 4.32 – 4.19 (m, 3H), 2.65 (s, 3H), 1.93 (s, 2H), 1.86 – 1.72 (m, 6H), 1.56 – 1.44 (m, 6H), 1.44 (s, 12H), 1.34 – 1.23 (m, 6H), 0.88 (t, *J* = 6.3 Hz, 36H). ^{13}**C NMR** (101 MHz, $CDCl_3$) δ 165.7, 139.9, 137.2, 131.6, 131.0, 130.3, 71.2, 71.1, 49.1, 46.8, 31.9, 24.5, 23.3, 22.8, 22.0. ^{11}**B NMR** (128 MHz, $CDCl_3$) δ 26.1. **HRMS (ESI)** *m/z* Calcd for $C_{42}H_{77}BNaO_7Si$ $[M+Na]^+$ 755.5424, Found: 755.5452.

2,3-difluoro-4-(4,4,6,6-tetramethyl-1,3,2-dioxaborinan-2-yl)benzoic (tris(2,6-dimethylheptan-4-yl) silicic) anhydride (44)

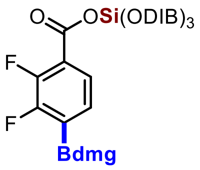

2,3-difluorobenzoic (tris(2,6-dimethylheptan-4-yl) silicic) anhydride **38f** (0.2 mmol, 123.0 mg), B_2dmg_2 (84.6 mg, 0.3 mmol, 1.5 equiv), $[Ir(cod)OMe]_2$ (2.0 mg, 0.003 mmol), Me_4phen (1.5 mg, 0.006 mmol), and cyclohexane (1.0 mL) were added to a 15 mL glass vial under air atmosphere. The glass vial was capped with a teflon pressure cap and placed into an aluminum block pre-heated to 100 ºC for 1 h. After completion, cyclohexane was removed under reduced pressure and chromatographic separation with silica gel (15% ethyl acetate in hexane as eluent) gave the borylated product **44** (119.3 mg, 79%) as colorless liquid. The *para:meta* ratio of products was reported from analysis of ^{1}H NMR.

^{1}H NMR (400 MHz, $CDCl_3$) δ 7.61 (ddd, *J* = 7.5, 5.8, 1.5 Hz, 1H), 7.43 (ddd, *J* = 7.9, 5.0, 1.6 Hz, 1H), 4.32 – 4.18 (m, 3H), 1.96 (s, 2H), 1.84 – 1.70 (m, 6H), 1.54 – 1.44 (m, 6H), 1.45 (s, 12H), 1.27 (ddd, *J* = 13.4, 8.0, 5.2 Hz, 6H), 0.87 (dd, *J* = 6.7, 5.2 Hz, 36H). **^{13}C NMR** (101 MHz, $CDCl_3$) δ 161.6 (t, *J* = 3.0 Hz), 155.0 (dd, *J* = 252.2, 12.2 Hz), 150.0 (dd, *J* = 155.4, 16.4 Hz), 132.6, 129.2 (dd, *J* = 7.3, 5.4 Hz), 126.0 (d, *J* = 3.8 Hz), 122.8 (d, *J* = 6.6 Hz), 72.0, 71.3, 49.0, 46.7, 31.8, 24.5, 23.2, 22.7. **^{11}B NMR** (128 MHz, $CDCl_3$) δ 25.5. **HRMS (ESI)** *m/z* Calcd for $C_{41}H_{73}BF_2NaO_7Si$ $[M+Na]^+$ 777.5079, Found: 777.5091.

2-chloro-4-(4,4,6,6-tetramethyl-1,3,2-dioxaborinan-2-yl)benzyl tris(2,6-dimethylheptan-4-yl) silicate (46)

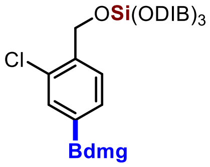

2-chlorobenzyl tris(2,6-dimethylheptan-4-yl) silicate **45a** (0.2 mmol, 119.9 mg), B_2dmg_2 (84.6 mg, 0.3 mmol, 1.5 equiv), $[Ir(cod)OMe]_2$ (2.0 mg, 0.003 mmol), Me_4phen (1.5 mg, 0.006 mmol), and cyclohexane (1.0 mL) were added to a 15 mL glass vial under air atmosphere. The glass vial was capped with a teflon pressure cap and placed into an aluminum block pre-heated to 100 ºC for 1 h. After completion, cyclohexane was removed under reduced pressure and chromatographic separation with silica gel (15% ethyl acetate in hexane as eluent) gave the borylated product **46** (125.7 mg, 85%) as colorless liquid. The *para:meta* ratio of products was reported from analysis of ^{1}H NMR.

^{1}H NMR (400 MHz, $CDCl_3$) δ 7.76 (d, *J* = 1.1 Hz, 1H), 7.70 (dd, *J* = 7.6, 1.1 Hz, 1H), 7.59 (d, *J* = 7.6 Hz, 1H), 4.95 (s, 2H), 4.23 – 4.06 (m, 3H), 1.92 (s, 2H), 1.87 – 1.72 (m, 6H), 1.53 – 1.43 (m, 6H), 1.43 (s, 12H), 1.27 (ddd, *J* = 13.5, 8.0, 5.3 Hz, 6H), 0.89 (dd, *J* = 7.9, 6.6 Hz, 36H). **^{13}C NMR** (101 MHz, $CDCl_3$) δ 140.3, 134.1, 132.0, 131.0, 126.7, 71.2, 70.3, 62.7, 49.2, 46.9, 32.0, 24.6, 23.3, 22.8. **^{11}B NMR** (128 MHz, $CDCl_3$) δ 25.7. **HRMS (ESI)** *m/z* Calcd for $C_{41}H_{76}BClNaO_6Si$ $[M+Na]^+$ 761.5085, Found: 761.5072.

2-bromo-4-(4,4,6,6-tetramethyl-1,3,2-dioxaborinan-2-yl)benzyl tris(2,6-dimethylheptan-4-yl) silicate (47)

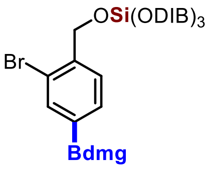

2-bromobenzyl tris(2,6-dimethylheptan-4-yl) silicate **45b** (0.2 mmol, 128.8 mg), B_2dmg_2 (84.6 mg, 0.3 mmol, 1.5 equiv), $[Ir(cod)OMe]_2$ (2.0 mg, 0.003 mmol), Me_4phen (1.5 mg, 0.006 mmol), and cyclohexane (1.0 mL) were added to a 15 mL glass vial under air atmosphere. The glass vial was capped with a teflon pressure cap and placed into an aluminum block pre-heated to 100 ºC for 1 h. After completion, cyclohexane was removed under reduced pressure and chromatographic separation with silica gel (15% ethyl acetate in hexane as eluent) gave the borylated product **47** (120.7 mg, 77%) as colorless liquid. The *para:meta* ratio of products was reported from analysis of ^{1}H NMR.

^{1}H NMR (400 MHz, $CDCl_3$) δ 7.94 (d, *J* = 1.1 Hz, 1H), 7.74 (dd, *J* = 7.6, 1.1 Hz, 1H), 7.58 (d, *J* = 7.6 Hz, 1H), 4.90 (s, 2H), 4.17 – 4.07 (m, 3H), 1.92 (s, 2H), 1.87 – 1.72 (m, 6H), 1.54 – 1.45 (m, 6H), 1.43 (s, 12H), 1.27 (ddd, *J* = 13.5, 8.0, 5.3 Hz, 6H), 0.89 (dd, *J* = 7.9, 6.6 Hz, 36H). **^{13}C NMR** (101 MHz, $CDCl_3$) δ 141.8, 137.4, 132.6, 126.8, 120.9, 71.2, 70.3, 65.1, 49.2, 46.9, 32.0, 24.6, 23.3, 22.8. **^{11}B NMR** (128 MHz, $CDCl_3$) δ 25.8. **HRMS (ESI)** *m/z* Calcd for $C_{41}H_{76}BBrNaO_6Si$ $[M+Na]^+$ 805.4580, Found: 805.4572.

Tris(2,6-dimethylheptan-4-yl) (4-(4,4,6,6-tetramethyl-1,3,2-dioxaborinan-2-yl)-2-(trifluoromethyl)benzyl) silicate (48)

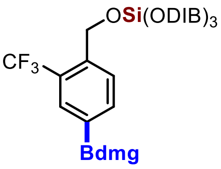

Tris(2,6-dimethylheptan-4-yl) (2-(trifluoromethyl)benzyl) silicate **45c** (0.2 mmol, 126.6 mg), B_2dmg_2 (84.6 mg, 0.3 mmol, 1.5 equiv), $[Ir(cod)OMe]_2$ (2.0 mg, 0.003 mmol), Me_4phen (1.5 mg, 0.006 mmol), and cyclohexane (1.0 mL) were added to a 15 mL glass vial under air atmosphere. The glass vial was capped with a teflon pressure cap and placed into an aluminum block pre-heated to 100 ºC for 1 h. After completion, cyclohexane was removed under reduced pressure and chromatographic separation with silica gel (15% ethyl acetate in hexane as eluent) gave the borylated product **48** (126.8 mg, 82%) as colorless liquid. The *para:meta* ratio of products was reported from analysis of ^{1}H NMR.

^{1}H NMR (400 MHz, $CDCl_3$) δ 8.05 (s, 1H), 7.98 (d, *J* = 7.8 Hz, 1H), 7.82 (d, *J* = 7.7 Hz, 1H), 5.09 (s, 2H), 4.22 – 4.06 (m, 3H), 1.93 (s, 2H), 1.86 – 1.72 (m, 6H), 1.51 – 1.45 (m, 6H), 1.45 (s, 12H), 1.35 – 1.22 (m, 6H), 0.89 (t, *J* = 7.1 Hz, 36H). **^{13}C NMR** (101 MHz, $CDCl_3$) δ 141.5, 137.3, 133.0 (d, *J* = 102.8 Hz), 130.8 (q, *J* = 5.5 Hz), 126.4, 125.4 (q, *J* = 30.4 Hz), 123.7, 71.3, 70.4, 61.4 (q, *J* = 3.5 Hz), 49.2, 46.9, 32.0, 24.6, 23.2, 22.7. **^{19}F NMR** (376 MHz, $CDCl_3$) δ -60.7. **^{11}B NMR** (128 MHz, $CDCl_3$) δ 26.0. **HRMS (ESI)** *m/z* Calcd for $C_{42}H_{77}BF_3O_6Si$ $[M+H]^+$ 773.5529, Found: 773.5546.

1-(2-chloro-4-(4,4,6,6-tetramethyl-1,3,2-dioxaborinan-2-yl)phenyl)ethyl tris(2,6-dimethylheptan-4-yl) silicate (49)

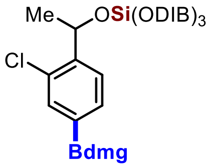

1-(2-chlorophenyl)ethyl tris(2,6-dimethylheptan-4-yl) silicate **45d** (0.2 mmol, 122.7 mg), B_2dmg_2 (84.6 mg, 0.3 mmol, 1.5 equiv), $[Ir(cod)OMe]_2$ (2.0 mg, 0.003 mmol), Me_4phen (1.5 mg, 0.006 mmol), and cyclohexane (1.0 mL) were added to a 15 mL glass vial under air atmosphere. The glass vial was capped with a teflon pressure cap and placed into an aluminum block pre-heated to 100 ºC for 1 h. After completion, cyclohexane was removed under reduced pressure and chromatographic separation with silica gel (15% ethyl acetate in hexane as eluent) gave the borylated product **49** (125.1 mg, 83%) as colorless liquid. The *para:meta* ratio of products was reported from analysis of ^{1}H NMR.

^{1}H NMR (400 MHz, $CDCl_3$) δ 7.74 – 7.65 (m, 2H), 7.62 (d, *J* = 7.6 Hz, 1H), 5.49 (q, *J* = 6.2 Hz, 1H), 4.26 – 3.91 (m, 3H), 1.91 (s, 2H), 1.84 – 1.65 (m, 6H), 1.48 – 1.35 (m, 21H), 1.30 – 1.16 (m, 6H), 1.02 – 0.74 (m, 36H). **^{13}C NMR** (101 MHz, $CDCl_3$) δ 145.7, 134.3, 132.2, 130.3, 126.1, 70.9, 70.0, 68.1, 49.0, 46.7 (d, *J* = 6.7 Hz), 31.8, 25.1, 24.4, 23.0 (d, *J* = 3.0 Hz), 22.7 (d, *J* = 12.4 Hz). **^{11}B NMR** (128 MHz, $CDCl_3$) δ 25.8. **HRMS (ESI)** *m/z* Calcd for $C_{42}H_{78}BClNaO_6Si$ $[M+Na]^+$ 775.5241, Found: 775.5249.

Methyl 2-(2-bromo-4-(4,4,6,6-tetramethyl-1,3,2-dioxaborinan-2-yl)phenyl)-2-((tris((2,6-dimethylheptan-4-yl)oxy)silyl)oxy)acetate (50)

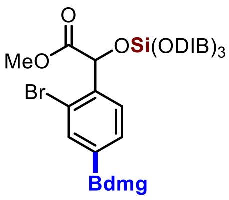

Methyl 2-(2-bromophenyl)-2-((tris((2,6-dimethylheptan-4-yl)oxy)silyl)oxy)acetate **45e** (0.2 mmol, 140.4 mg), B_2dmg_2 (84.6 mg, 0.3 mmol, 1.5 equiv), $[Ir(cod)OMe]_2$ (2.0 mg, 0.003 mmol), Me_4phen (1.5 mg, 0.006 mmol), and cyclohexane (1.0 mL) were added to a 15 mL glass vial under air atmosphere. The glass vial was capped with a teflon pressure cap and placed into an aluminum block pre-heated to 100 ºC for 1 h. After completion, cyclohexane was removed under reduced pressure and chromatographic separation with silica gel (15% ethyl acetate in hexane as eluent) gave the borylated product **50** (133.0 mg, 79%) as colorless liquid. The *para:meta* ratio of products was reported from analysis of ^{1}H NMR.

^{1}H NMR (400 MHz, $CDCl_3$) δ 7.95 (d, *J* = 1.0 Hz, 1H), 7.72 (dd, *J* = 7.7, 1.1 Hz, 1H), 7.58 (d, *J* = 7.7 Hz, 1H), 5.90 (s, 1H), 4.23 – 3.97 (m, 3H), 3.63 (s, 3H), 1.90 (s, 2H), 1.82 – 1.64 (m, 6H), 1.50 – 1.32 (m, 18H), 1.30 – 1.15 (m, 6H), 1.00 – 0.69 (m, 36H). **^{13}C NMR** (101 MHz, $CDCl_3$) δ 171.3, 140.2, 138.0, 132.9, 128.4, 122.7, 73.3, 71.2, 70.4,

52.0, 49.1, 46.7 (d, J = 7.9 Hz), 31.9, 24.5, 23.2 (d, J = 2.5 Hz), 22.8 (d, J = 11.7 Hz). **^{11}B NMR** (128 MHz, $CDCl_3$) δ 25.6. **HRMS (ESI)** *m/z* Calcd for $C_{43}H_{78}BBrNaO_8Si$ $[M+Na]^+$ 863.4635, Found: 863.4649.

2,3-difluoro-4-(4,4,6,6-tetramethyl-1,3,2-dioxaborinan-2-yl)benzyl tris(2,6-dimethylheptan-4-yl) silicate (51)

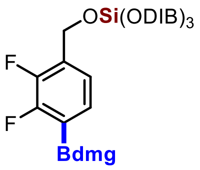

2,3-difluorobenzyl tris(2,6-dimethylheptan-4-yl) silicate **45f** (0.2 mmol, 120.2 mg), B_2dmg_2 (84.6 mg, 0.3 mmol, 1.5 equiv), $[Ir(cod)OMe]_2$ (2.0 mg, 0.003 mmol), Me_4phen (1.5 mg, 0.006 mmol), and cyclohexane (1.0 mL) were added to a 15 mL glass vial under air atmosphere. The glass vial was capped with a teflon pressure cap and placed into an aluminum block pre-heated to 100 ºC for 1 h. After completion, cyclohexane was removed under reduced pressure and chromatographic separation with silica gel (15% ethyl acetate in hexane as eluent) gave the borylated product **51** (118.6 mg, 80%) as colorless liquid. The *para:meta* ratio of products was reported from analysis of ^{1}H NMR.

^{1}H NMR (400 MHz, $CDCl_3$) δ 7.45 – 7.34 (m, 1H), 7.23 (t, J = 6.9 Hz, 1H), 4.91 (s, 2H), 4.28 – 3.96 (m, 3H), 1.95 (s, 2H), 1.86 – 1.69 (m, 6H), 1.50 – 1.39 (m, 18H), 1.25 (ddd, J = 13.5, 7.9, 5.4 Hz, 6H), 0.88 (t, J = 6.8 Hz, 36H). **^{13}C NMR** (101 MHz, $CDCl_3$) δ 154.2 (dd, J = 250.7, 11.3 Hz), 147.8 (dd, J = 247.2, 15.2 Hz), 132.2 (d, J = 11.1 Hz), 129.6 (dd, J = 7.5, 4.6 Hz), 122.4 (t, J = 3.3 Hz), 71.6, 70.3, 58.8 (dd, J = 5.2, 3.1 Hz), 49.1, 46.9, 31.9, 24.5, 23.2, 22.7. **^{19}F NMR** (376 MHz, $CDCl_3$) δ -132.87 (d, J = 20.8 Hz), -146.88 (d, J = 21.0 Hz). **^{11}B NMR** (128 MHz, $CDCl_3$) δ 25.8. **HRMS (ESI)** *m/z* Calcd for $C_{41}H_{75}BF_2NaO_6Si$ $[M+Na]^+$ 763.5286, Found: 763.5297.

2-chloro-4-(4,4,6,6-tetramethyl-1,3,2-dioxaborinan-2-yl)phenyl tris(2,6-dimethylheptan-4-yl) silicate (53)

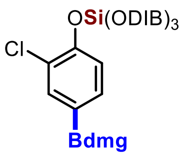

2-chlorophenyl tris(2,6-dimethylheptan-4-yl) silicate **52a** (0.2 mmol, 117.1 mg), B_2dmg_2 (84.6 mg, 0.3 mmol, 1.5 equiv), $[Ir(cod)OMe]_2$ (2.0 mg, 0.003 mmol), Me_4phen (1.5 mg, 0.006 mmol), and cyclohexane (1.0 mL) were added to a 15 mL glass vial under air atmosphere. The glass vial was capped with a teflon pressure cap and placed into an aluminum block pre-heated to 100 ºC for 1 h. After completion, cyclohexane was removed under reduced pressure and chromatographic separation with silica gel (15% ethyl acetate in hexane as eluent) gave the borylated product **53** (121.9 mg, 84%) as colorless liquid. The *para:meta* ratio of products was reported from analysis of ^{1}H NMR.

^{1}H NMR (400 MHz, $CDCl_3$) δ 7.87 (d, J = 1.6 Hz, 1H), 7.52 (dd, J = 8.1, 1.6 Hz, H), 7.12 (d, J = 8.1 Hz, H), 4.19 – 3.98 (m, 3H), 1.81 (s, 2H), 1.74 – 1.60 (m, 6H), 1.45 – 1.33 (m, 6H), 1.33 (s, 6H), 1.25 – 1.14 (m, 12H), 0.79 (dd, J = 10.5, 6.6 Hz, 36H). **^{13}C NMR** (101 MHz, $CDCl_3$) δ 153.1, 138.9, 133.9, 119.5, 114.0, 71.2, 71.0, 49.1, 46.6, 32.0,

24.6, 23.1, 22.9. **^{11}B NMR** (128 MHz, $CDCl_3$) δ 25.8. **HRMS (ESI)** *m/z* Calcd for $C_{40}H_{74}BClNaO_6Si$ $[M+Na]^+$ 747.4928, Found: 747.4907.

2-bromo-4-(4,4,6,6-tetramethyl-1,3,2-dioxaborinan-2-yl)phenyl tris(2,6-dimethylheptan-4-yl) silicate (54)

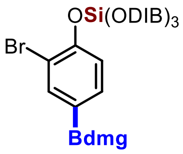

2-bromophenyl tris(2,6-dimethylheptan-4-yl) silicate **52b** (0.2 mmol, 126.0 mg), B_2dmg_2 (84.6 mg, 0.3 mmol, 1.5 equiv), $[Ir(cod)OMe]_2$ (2.0 mg, 0.003 mmol), Me_4phen (1.5 mg, 0.006 mmol), and cyclohexane (1.0 mL) were added to a 15 mL glass vial under air atmosphere. The glass vial was capped with a teflon pressure cap and placed into an aluminum block pre-heated to 100 ºC for 1 h. After completion, cyclohexane was removed under reduced pressure and chromatographic separation with silica gel (15% ethyl acetate in hexane as eluent) gave the borylated product **54** (115.5 mg, 75%) as colorless liquid. The *para:meta* ratio of products was reported from analysis of ^{1}H NMR.

^{1}H NMR (400 MHz, $CDCl_3$) δ 7.79 (s, 1H), 7.57 (d, *J* = 8.0 Hz, 1H), 7.20 (d, *J* = 8.0 Hz, 1H), 4.27 – 4.11 (m, 3H), 1.90 (s, 2H), 1.82 – 1.68 (m, 6H), 1.53 – 1.42 (m, 6H), 1.42 (s, 12H), 1.28 (dt, *J* = 13.4, 6.3 Hz, 6H), 0.87 (dd, *J* = 10.5, 6.7 Hz, 36H). **^{13}C NMR** (101 MHz, $CDCl_3$) δ 152.2, 135.8, 133.1, 124.2, 119.7, 71.1, 71.0, 49.1, 46.7, 32.0, 24.5, 23.1, 22.8. **^{11}B NMR** (128 MHz, $CDCl_3$) δ 25.6. **HRMS (ESI)** *m/z* Calcd for $C_{40}H_{74}BBrNaO_6Si$ $[M+Na]^+$ 791.4423, Found: 791.4432.

Tris(2,6-dimethylheptan-4-yl) (2-(methylthio)-4-(4,4,6,6-tetramethyl-1,3,2-dioxaborinan-2-yl)phenyl) silicate (55)

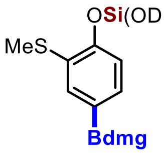

Tris(2,6-dimethylheptan-4-yl) (2-(methylthio)phenyl) silicate **52c** (0.2 mmol, 119.4 mg), B_2dmg_2 (84.6 mg, 0.3 mmol, 1.5 equiv), $[Ir(cod)OMe]_2$ (2.0 mg, 0.003 mmol), Me_4phen (1.5 mg, 0.006 mmol), and cyclohexane (1.0 mL) were added to a 15 mL glass vial under air atmosphere. The glass vial was capped with a teflon pressure cap and placed into an aluminum block pre-heated to 100 ºC for 1 h. After completion, cyclohexane was removed under reduced pressure and chromatographic separation with silica gel (15% ethyl acetate in hexane as eluent) gave the borylated product **55** (117.9 mg, 80%) as colorless liquid. The *para:meta* ratio of products was reported from analysis of ^{1}H NMR.

^{1}H NMR (400 MHz, $CDCl_3$) δ 7.62 (d, *J* = 1.6 Hz, 1H), 7.51 (dd, *J* = 8.1, 1.6 Hz, 1H), 7.11 (d, *J* = 8.0 Hz, 1H), 4.28 – 4.15 (m, 3H), 2.42 (s, 3H), 1.90 (s, 2H), 1.83 – 1.68 (m, 6H), 1.48 (ddd, *J* = 13.5, 7.2, 6.2 Hz, 6H), 1.42 (s, 12H), 1.28 (ddd, *J* = 13.4, 7.8, 5.6 Hz, 6H), 0.87 (dd, *J* = 10.2, 6.6 Hz, 36H). **^{13}C NMR** (101 MHz, $CDCl_3$) δ 153.7, 132.4,

131.8, 128.0, 117.9, 71.0, 70.8, 49.2, 46.6, 32.0, 24.6, 23.1, 22.9, 15.2. **^{11}B NMR** (128 MHz, $CDCl_3$) δ 26.1. **HRMS (ESI)** *m/z* Calcd for $C_{41}H_{77}BNaO_6SSi$ $[M+Na]^+$ 759.5195, Found: 759.5208.

2,3-difluoro-4-(4,4,5,5-tetramethyl-1,3,2-dioxaborolan-2-yl)phenyl tris(2,6-dimethylheptan-4-yl) silicate (56)

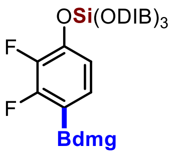

2,3-difluorophenyl tris(2,6-dimethylheptan-4-yl) silicate **52d** (0.2 mmol, 117.4 mg), B_2dmg_2 (84.6 mg, 0.3 mmol, 1.5 equiv), $[Ir(cod)OMe]_2$ (2.0 mg, 0.003 mmol), Me_4phen (1.5 mg, 0.006 mmol), and cyclohexane (1.0 mL) were added to a 15 mL glass vial under air atmosphere. The glass vial was capped with a teflon pressure cap and placed into an aluminum block pre-heated to 100 ºC for 1 h. After completion, cyclohexane was removed under reduced pressure and chromatographic separation with silica gel (15% ethyl acetate in hexane as eluent) gave the borylated product **56** (119.2 mg, 82%) as colorless liquid. The *para:meta* ratio of products was reported from analysis of ^{1}H NMR.

^{1}H NMR (400 MHz, $CDCl_3$) δ 6.47 (d, J = 8.9 Hz, 2H), 4.31 – 3.86 (m, 3H), 1.96 (s, 2H), 1.80 – 1.65 (m, 6H), 1.48 – 1.40 (m, 18H), 1.35 – 1.22 (m, 6H), 0.87 (dd, J = 8.3, 6.5 Hz, 36H). **^{13}C NMR** (101 MHz, $CDCl_3$) δ 166.45 (dd, J = 245.0, 17.7 Hz), 157.10 (t, J = 15.4 Hz),103.12 (d, J = 13.1 Hz), 103.12 (d, J = 31.1 Hz), 103.12 (d, J = 26.6 Hz),72.0, 71.1, 49.2, 46.8, 31.8, 24.5, 23.2, 22.7.**^{11}B NMR** (128 MHz, $CDCl_3$) δ 26.0. **^{19}F NMR** (376 MHz, $CDCl_3$) δ -137.1 (dd, J = 20.2, 3.2 Hz), -155.8 (d, J = 19.9 Hz). **HRMS (ESI)** *m/z* Calcd for $C_{40}H_{73}BF_2NaO_6Si$ $[M+Na]^+$ 749.5130, Found: 749.5134.

7-(4,4,6,6-tetramethyl-1,3,2-dioxaborinan-2-yl)-4-((triisopropylsilyl)oxy)-2H-chromen-2-one (57)

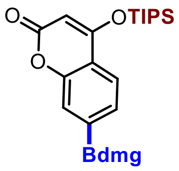

4-((triisopropylsilyl)oxy)-2H-chromen-2-one **52e** (0.2 mmol, 63.7 mg), B_2dmg_2 (84.6 mg, 0.3 mmol, 1.5 equiv), $[Ir(cod)OMe]_2$ (2.0 mg, 0.003 mmol), Me_4phen (1.5 mg, 0.006 mmol), and cyclohexane (1.0 mL) were added to a 15 mL glass vial under air atmosphere. The glass vial was capped with a teflon pressure cap and placed into an aluminum block pre-heated to 120 ºC for 1 h. After completion, cyclohexane was removed under reduced pressure and chromatographic separation with silica gel (15% ethyl acetate in hexane as eluent) gave the borylated product **57** (80.7 mg, 88%) as colorless liquid. The *para:meta* ratio of products was reported from analysis of ^{1}H NMR.

^{1}H NMR (400 MHz, $CDCl_3$) δ 7.80 – 7.71 (m, 3H), 5.71 (s, 1H), 1.93 (s, 2H), 1.42 (s, 12H), 1.41 – 1.35 (m, 3H), 1.15 (d, J = 7.5 Hz, 18H). **^{13}C NMR** (101 MHz, $CDCl_3$) δ 164.6, 163.7, 153.4, 129.1, 122.3, 122.0, 119.1, 97.4, 71.5, 49.0, 31.9, 18.0, 12.8. **^{11}B NMR** (128 MHz, $CDCl_3$) δ 20.7. **HRMS (ESI)** *m/z* Calcd for $C_{25}H_{39}BNaO_5Si$ $[M+Na]^+$ 481.2552, Found: 481.2562.

tert-butyl (5-(4,4,6,6-tetramethyl-1,3,2-dioxaborinan-2-yl)-2-((tris((2,6-dimethylheptan-4-yl)oxy)silyl)oxy)benzyl)carbamate (58)

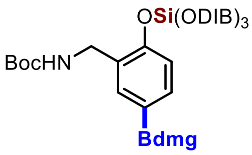

tert-butyl (2-((tris((2,6-dimethylheptan-4-yl)oxy)silyl)oxy)benzyl)carbamate **52f** (0.2 mmol, 136.0 mg), B_2dmg_2 (84.6 mg, 0.3 mmol, 1.5 equiv), $[Ir(cod)OMe]_2$ (2.0 mg, 0.003 mmol), Me_4phen (1.5 mg, 0.006 mmol), and cyclohexane (1.0 mL) were added to a 15 mL glass vial under air atmosphere. The glass vial was capped with a teflon pressure cap and placed into an aluminum block pre-heated to 120 ºC for 1 h. After completion, cyclohexane was removed under reduced pressure and chromatographic separation with silica gel (15% ethyl acetate in hexane as eluent) gave the borylated product **58** (136.1 mg, 83%) as colorless liquid. The *para:meta* ratio of products was reported from analysis of ^1H NMR.

^1H NMR (400 MHz, $CDCl_3$) δ 7.74 (d, J = 1.7 Hz, 1H), 7.62 (dd, J = 8.1, 1.7 Hz, 1H), 7.05 (d, J = 8.1 Hz, 1H), 5.41 (t, J = 5.6 Hz, 1H), 4.32 (d, J = 5.7 Hz, 2H), 4.25 – 4.06 (m, 3H), 1.88 (s, 2H), 1.75 – 1.60 (m, 6H), 1.49 – 1.42 (m, 15H), 1.40 (s, 12H), 1.28 (ddd, J = 13.6, 7.9, 5.6 Hz, 6H), 0.85 (dd, J = 12.0, 6.6 Hz, 36H). **^{13}C NMR** (101 MHz, $CDCl_3$) δ 155.9, 154.9, 136.3, 134.7, 127.9, 119.0, 78.8, 71.3, 70.8, 49.2, 46.6, 40.9, 32.0, 28.7, 24.5, 23.1, 22.8. **^{11}B NMR** (128 MHz, $CDCl_3$) δ 26.7. **HRMS (ESI)** *m/z* Calcd for $C_{46}H_{86}BNNaO_8Si$ $[M+Na]^+$ 842.6108, Found: 842.6139.

4.3 Substrate scope of *para* C-H borylation of aryl and benzyl silicon compounds

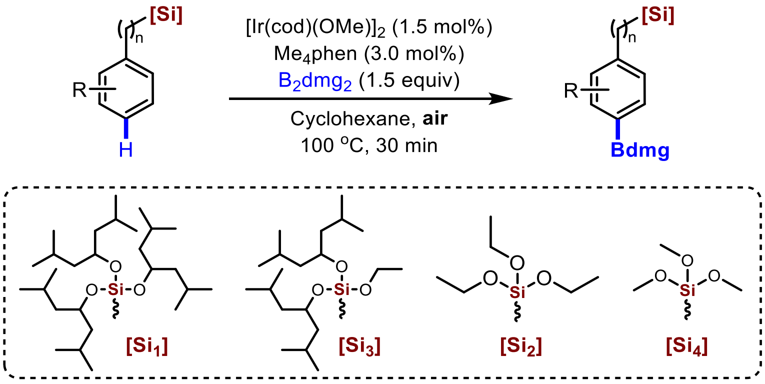

Tris((2,6-dimethylheptan-4-yl)oxy)(4-(4,4,6,6-tetramethyl-1,3,2-dioxaborinan-2-yl)phenyl)silane (59)

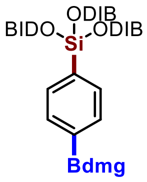

Tris((2,6-dimethylheptan-4-yl)oxy)(phenyl)silane **59a** (3 mmol, 1.0 equiv), B_2dmg_2 (1.26 g, 4.5 mmol, 1.5 equiv), $[Ir(cod)OMe]_2$ (30.0 mg, 0.045 mmol), Me_4phen (22.5 mg, 0.09 mmol), and cyclohexane (15.0 mL) were added to a 50 mL glass vial under air atmosphere. The glass vial was capped with a teflon pressure cap and placed into an aluminum block pre-heated to 100 ºC for 30

minutes. After completion, cyclohexane was removed under reduced pressure and chromatographic separation with silica gel (20% ethyl acetate in hexane as eluent) gave the borylated product **59** (2.0 g, 98%) as colorless liquid. The *para:meta* ratio of products was reported from analysis of ^{1}H NMR.

^{1}H NMR (400 MHz, $CDCl_3$) δ 7.81 (d, *J* = 7.9 Hz, 2H), 7.69 – 7.57 (m, 2H), 4.19 – 4.05 (m, 3H), 1.95 (s, 2H), 1.85 – 1.70 (m, 6H), 1.46 (s, 18H), 1.33 – 1.27 (m, 6H), 0.86 (d, *J* = 6.6 Hz, 36H). **^{13}C NMR** (101 MHz, $CDCl_3$) δ 136.0, 134.2, 132.8, 70.9, 69.9, 49.2, 47.1, 32.0, 24.6, 23.2, 22.9. **^{11}B NMR** (128 MHz, $CDCl_3$) δ 26.7. **HRMS (ESI)** *m/z* Calcd for $C_{40}H_{75}BNaO_5Si$ $[M+Na]^+$ 697.5369, Found: 697.5381.

Same reaction was performed with the **triethoxy(phenyl)silane** under identical conditions. Outcomes: **p/m:** 1/1, **conv.** 92% (based on crude ^{1}H NMR analysis).

Tris((2,6-dimethylheptan-4-yl)oxy)(2-methyl-4-(4,4,6,6-tetramethyl-1,3,2-dioxaborinan-2-yl)phenyl)silane (60)

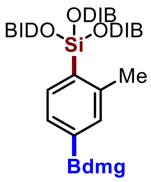

Tris((2,6-dimethylheptan-4-yl)oxy)(o-tolyl)silane **60a** (0.2 mmol, 109.8 mg), B_2dmg_2 (84.6 mg, 0.3 mmol, 1.5 equiv), $[Ir(cod)OMe]_2$ (2.0 mg, 0.003 mmol), Me_4phen (1.5 mg, 0.006 mmol), and cyclohexane (1.0 mL) were added to a 15 mL glass vial under air atmosphere. The glass vial was capped with a teflon pressure cap and placed into an aluminum block pre-heated to 100 ºC for 4h. After completion, cyclohexane was removed under reduced pressure and chromatographic separation with silica gel (20% ethyl acetate in hexane as eluent) gave the borylated product **60** (118.5 mg, 86%) as colorless liquid. The *para:meta* ratio of products was reported from analysis of ^{1}H NMR.

^{1}H NMR (400 MHz, $CDCl_3$) δ 7.70 (d, *J* = 7.6 Hz, 1H), 7.60 – 7.58 (m, 2H), 4.20 – 4.04 (m, 3H), 2.54 (s, 3H), 1.93 (s, 2H), 1.83 – 1.67 (m, 6H), 1.59 – 1.41 (m, 18H), 1.37 – 1.26 (m, 6H), 0.85 (dd, *J* = 6.7, 2.7 Hz, 36H). **^{13}C NMR** (101 MHz, $CDCl_3$) δ 142.6, 135.7, 135.5, 134.8, 129.8, 70.9, 70.1, 49.2, 47.0, 32.0, 24.6, 23.4, 23.1, 23.1. **^{11}B NMR** (128 MHz, $CDCl_3$) δ 26.8. **HRMS (ESI)** *m/z* Calcd for $C_{44}H_{77}BNaO_5Si$ $[M+Na]^+$ 711.5526, Found: 711.5531.

Same reaction was performed with the **triethoxy(o-tolyl)silane** under identical conditions. Outcomes: **p/m:** 3/1**, conv.** 83% (based on crude ^{1}H NMR analysis).

Tris((2,6-dimethylheptan-4-yl)oxy)(2-ethyl-4-(4,4,6,6-tetramethyl-1,3,2-dioxaborinan-2-yl)phenyl)silane (61)

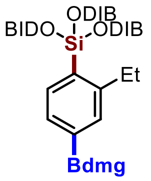

Tris((2,6-dimethylheptan-4-yl)oxy)(2-ethylphenyl)silane **61a** (0.2 mmol, 112.6 mg), B_2dmg_2 (84.6 mg, 0.3 mmol, 1.5 equiv), $[Ir(cod)OMe]_2$ (2.0 mg, 0.003 mmol), Me_4phen (1.5 mg, 0.006 mmol), and cyclohexane (1.0 mL) were added to a 15 mL glass vial under air atmosphere. The glass vial was capped with a teflon pressure cap and placed into an aluminum block pre-heated to 100 ºC for 4 h.

After completion, cyclohexane was removed under reduced pressure and chromatographic separation with silica gel (20% ethyl acetate in hexane as eluent) gave the borylated product **61** (115.3 mg, 82%) as colorless liquid. The *para:meta* ratio of products was reported from analysis of ^{1}H NMR.

^{1}H NMR (400 MHz, $CDCl_3$) δ 7.74 – 7.67 (m, 2H), 7.63 (dd, *J* = 7.3, 1.2 Hz, 1H), 4.20 – 4.02 (m, 3H), 2.91 (q, *J* = 7.5 Hz, 2H), 1.95 (s, 2H), 1.86 – 1.70 (m, 6H), 1.61 – 1.43 (m, 18H), 1.43 – 1.27 (m, 9H), 0.88 (dd, *J* = 6.7, 2.7 Hz, 36H). **^{13}C NMR** (101 MHz, $CDCl_3$) δ 149.1, 135.4, 135.0, 133.1, 129.9, 70.9, 70.2, 49.2, 47.0, 32.0, 31.7, 29.5, 24.6, 23.1, 16.6. **^{11}B NMR** (128 MHz, $CDCl_3$) δ 27.0. **HRMS (ESI)** *m/z* Calcd for $C_{42}H_{79}BNaO_5Si$ $[M+Na]^+$ 725.5682, Found: 725.5692.

Same reaction was performed with the **triethoxy(2-ethylphenyl)silane** under identical conditions. Outcomes: **p/m:** 3/1**, conv.** 85% (based on crude ^{1}H NMR analysis).

Tris((2,6-dimethylheptan-4-yl)oxy)(2-isopropyl-4-(4,4,6,6-tetramethyl-1,3,2-dioxaborinan-2-yl)phenyl)silane (62)

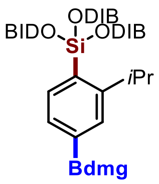

Tris((2,6-dimethylheptan-4-yl)oxy)(2-isopropylphenyl)silane **62a** (0.2 mmol, 115.4 mg), B_2dmg_2 (84.6 mg, 0.3 mmol, 1.5 equiv), $[Ir(cod)OMe]_2$ (2.0 mg, 0.003 mmol), Me_4phen (1.5 mg, 0.006 mmol), and cyclohexane (1.0 mL) were added to a 15 mL glass vial under air atmosphere. The glass vial was capped with a teflon pressure cap and placed into an aluminum block pre-heated to 100 ºC for 4 h. After completion, cyclohexane was removed under reduced pressure and chromatographic separation with silica gel (20% ethyl acetate in hexane as eluent) gave the borylated product **62** (121.9 mg, 85%) as colorless liquid. The *para:meta* ratio of products was reported from analysis of ^{1}H NMR.

^{1}H NMR (400 MHz, $CDCl_3$) δ 7.75 (s, 1H), 7.66 (d, *J* = 7.5 Hz, 1H), 7.59 (dd, *J* = 7.4, 1.2 Hz, 1H), 4.12 (p, *J* = 6.3 Hz, 3H), 3.44 (hept, *J* = 6.8 Hz, 1H), 1.92 (s, 2H), 1.82 – 1.67 (m, 6H), 1.44 (s, 18H), 1.38 – 1.28 (m, 6H), 1.28 (d, *J* = 6.8 Hz, 6H), 0.85 (dd, *J* = 6.7, 3.9 Hz, 36H). **^{13}C NMR** (101 MHz, $CDCl_3$) δ 154.0, 135.2, 134.8, 130.1, 130.1, 70.8, 70.2, 49.2, 46.9, 33.4, 32.0, 25.1, 24.6, 23.2, 23.1. **^{11}B NMR** (128 MHz, $CDCl_3$) δ 26.8. **HRMS (ESI)** *m/z* Calcd for $C_{43}H_{81}BNaO_5Si$ $[M+Na]^+$ 739.5839, Found: 739.5831.

Same reaction was performed with the **triethoxy(2-isopropylphenyl)silane** under identical conditions. Outcomes: **p/m:** 2/1**, conv.** 80% (based on crude ^{1}H NMR analysis).

Tris((2,6-dimethylheptan-4-yl)oxy)(2-cyclopropyl-4-(4,4,6,6-tetramethyl-1,3,2-dioxaborinan-2-yl)phenyl) silane (63)

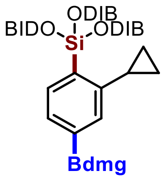

(2-cyclopropylphenyl)tris((2,6-dimethylheptan-4-yl)oxy)silane **63a** (0.2 mmol, 115.0 mg), B_2dmg_2 (84.6 mg, 0.3 mmol, 1.5 equiv), $[Ir(cod)OMe]_2$ (2.0 mg, 0.003 mmol), Me_4phen (1.5 mg, 0.006 mmol), and cyclohexane (1.0 mL) were added to a 15 mL glass vial under air atmosphere. The glass vial was capped with a teflon pressure cap and placed into an aluminum block pre-heated to 100 ºC for 4 h. After completion, cyclohexane was removed under reduced pressure and chromatographic separation with silica gel (20% ethyl acetate in hexane as eluent) gave the borylated product **63** (117.3 mg, 82%) as colorless liquid. The *para:meta* ratio of products was reported from analysis of ^{1}H NMR.

^{1}H NMR (400 MHz, $CDCl_3$) δ 7.70 (d, *J* = 7.4 Hz, 1H), 7.55 (d, *J* = 7.4 Hz, 1H), 7.11 (s, 1H), 4.14 (p, *J* = 6.3 Hz, 3H), 2.53 – 2.34 (m, 1H), 1.91 (s, 2H), 1.83 – 1.69 (m, 6H), 1.55 – 1.44 (m, 6H), 1.43 (s, 12H), 1.38 – 1.27 (m, 6H), 1.02 – 0.94 (m, 2H), 0.84 (dd, *J* = 6.7, 3.3 Hz, 38H). **^{13}C NMR** (101 MHz, $CDCl_3$) δ 148.3, 135.4, 135.2, 129.5, 125.8, 70.7,70.0, 49.1, 46.8, 31.9, 24.5, 23.0, 22.9, 15.4, 11.3. **^{11}B NMR** (128 MHz, $CDCl_3$) δ 26.3. **HRMS (ESI)** *m/z* Calcd for $C_{43}H_{79}BNaO_5Si$ $[M+Na]^+$ 737.5682, Found: 737.5685.

Same reaction was performed with the **(2-cyclopropylphenyl)triethoxysilane (3ee)** under identical conditions. Outcomes: **p/m:** 3/1**, conv.** 80% (based on crude ^{1}H NMR analysis).

Tris((2,6-dimethylheptan-4-yl)oxy)(2-methoxy-4-(4,4,6,6-tetramethyl-1,3,2-dioxaborinan-2-yl)phenyl)silane (64)

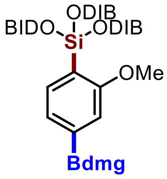

Tris((2,6-dimethylheptan-4-yl)oxy)(2-methoxyphenyl)silane **64a** (0.2 mmol, 113.0 mg), B_2dmg_2 (84.6 mg, 0.3 mmol, 1.5 equiv), $[Ir(cod)OMe]_2$ (2.0 mg, 0.003 mmol), Me_4phen (1.5 mg, 0.006 mmol), and cyclohexane (1.0 mL) were added to a 15 mL glass vial under air atmosphere. The glass vial was capped with a teflon pressure cap and placed into an aluminum block pre-heated to 100 ºC for 4 h. After completion, cyclohexane was removed under reduced pressure and chromatographic separation with silica gel (20% ethyl acetate in hexane as eluent) gave the borylated product **64** (112.8 mg, 80%) as colorless liquid. The *para:meta* ratio of products was reported from analysis of ^{1}H NMR.

^{1}H NMR (400 MHz, $CDCl_3$) δ 7.67 (d, *J* = 7.2 Hz, 1H), 7.40 (dd, *J* = 7.2, 0.8 Hz, 1H), 7.20 (s, 1H), 4.19 – 4.00 (m, 3H), 3.82 (s, 3H), 1.92 (s, 2H), 1.73 (dq, *J* = 13.2, 6.6 Hz, 6H), 1.50 – 1.38 (m, 18H), 1.28 – 1.21 (m, 6H), 0.81 (d, *J* = 6.6 Hz, 36H). **^{13}C NMR** (101 MHz, $CDCl_3$) δ 163.1, 137.2, 125.8, 124.2, 113.1, 70.9, 69.4, 54.5, 49.0, 46.9, 31.9,

24.3, 23.1, 22.9. **^{11}B NMR** (128 MHz, $CDCl_3$) δ 26.2. **HRMS (ESI)** *m/z* Calcd for $C_{41}H_{78}BO_6Si$ $[M+H]^+$ 705.5655, Found: 705.5661.

Same reaction was performed with the **triethoxy(2-methoxyphenyl)silane** under identical conditions. Outcomes: **p/m:** 10/1**, conv.** 75% (based on crude ^{1}H NMR analysis).

Tris((2,6-dimethylheptan-4-yl)oxy)(2-ethoxy-4-(4,4,6,6-tetramethyl-1,3,2-dioxaborinan-2-yl)phenyl)silane (65)

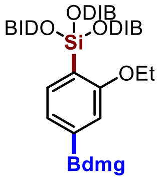

Tris((2,6-dimethylheptan-4-yl)oxy)(2-ethoxyphenyl)silane **65a** (0.2 mmol, 115.8 mg), B_2dmg_2 (84.6 mg, 0.3 mmol, 1.5 equiv), $[Ir(cod)OMe]_2$ (2.0 mg, 0.003 mmol), Me_4phen (1.5 mg, 0.006 mmol), and cyclohexane (1.0 mL) were added to a 15 mL glass vial under air atmosphere. The glass vial was capped with a teflon pressure cap and placed into an aluminum block pre-heated to 100 ºC for 4 h. After completion, cyclohexane was removed under reduced pressure and chromatographic separation with silica gel (20% ethyl acetate in hexane as eluent) gave the borylated product **65**(122.2 mg, 85%) as colorless liquid. The *para:meta* ratio of products was reported from analysis of ^{1}H NMR.

^{1}H NMR (400 MHz, $CDCl_3$) δ 7.69 (d, *J* = 7.1 Hz, 1H), 7.40 (d, *J* = 7.1 Hz, 1H), 7.22 (s, 1H), 4.23 – 4.05 (m, 5H), 1.93 (s, 2H), 1.82 – 1.66 (m, 6H), 1.51 – 1.42 (m, 21H), 1.36 – 1.19 (m, 6H), 0.83 (dd, *J* = 6.7, 2.5 Hz, 36H). **^{13}C NMR** (101 MHz, $CDCl_3$) δ 162.9, 137.1, 125.8, 125.0, 114. 6, 71.0, 69.6, 63.1, 49.2, 47.0, 32.0, 24.5, 23.3, 23.1, 15.2. **^{11}B NMR** (128 MHz, $CDCl_3$) δ 25.9. **HRMS (ESI)** *m/z* Calcd for $C_{42}H_{79}BNaO_6Si$ $[M+Na]^+$ 741.5631, Found: 741.5638.

Same reaction was performed with the **triethoxy(2-ethoxyphenyl)silane** under identical conditions. Outcomes: **p/m:** 9/1**, conv.** 86% (based on crude ^{1}H NMR analysis).

Tris((2,6-dimethylheptan-4-yl)oxy)(4-(4,4,6,6-tetramethyl-1,3,2-dioxaborinan-2-yl)-2-(trifluoromethyl)phenyl)silane (66)

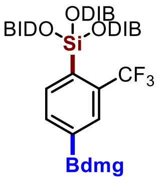

Tris((2,6-dimethylheptan-4-yl)oxy)(2-(trifluoromethyl)phenyl)silane **66a** (0.2 mmol, 115.8 mg), B_2dmg_2 (84.6 mg, 0.3 mmol, 1.5 equiv), $[Ir(cod)OMe]_2$ (2.0 mg, 0.003 mmol), Me_4phen (1.5 mg, 0.006 mmol), and cyclohexane (1.0 mL) were added to a 15 mL glass vial under air atmosphere. The glass vial was capped with a teflon pressure cap and placed into an aluminum block pre-heated to 100 ºC for 30 minutes. After completion, cyclohexane was removed under reduced pressure and chromatographic separation with silica gel (20% ethyl acetate in hexane as eluent) gave the borylated product **66** (145.6 mg, 97%) as colorless liquid. The *para:meta* ratio of products was reported from analysis of ^{1}H NMR.

^{1}**H NMR** (400 MHz, $CDCl_3$) δ 8.10 (s, 1H), 8.04 (d, *J* = 7.6 Hz, 1H), 7.93 (d, *J* = 7.5 Hz, 1H), 4.12 (p, *J* = 6.4 Hz, 3H), 1.95 (s, 2H), 1.87 – 1.65 (m, 6H), 1.51 – 1.43 (m, 18H), 1.38 – 1.26 (m, 6H), 0.85 (d, *J* = 6.8 Hz, 36H). ^{13}**C NMR** (101 MHz, $CDCl_3$) δ 137.1, 135.6, 134.6 (q, *J* = 3.5, 2.5 Hz), 134.3 (d, *J* = 30.6 Hz), 131.3 (q, *J* = 4.8 Hz), 125.2 (d, *J* = 273.9 Hz), 71.3, 70.6, 49.2, 46.8, 32.0, 24.6, 23.1, 23.1. ^{19}**F NMR** (376 MHz, $CDCl_3$) δ -58.3. ^{11}**B NMR** (128 MHz, $CDCl_3$) δ 25.4. **HRMS (ESI)** *m/z* Calcd for $C_{41}H_{74}BF_3NaO_5Si$ $[M+Na]^+$ 765.5243, Found: 765.5238.

Same reaction was performed with the **trimethoxy(2-(trifluoromethyl)phenyl)silane** under identical conditions. Outcomes: **p/m:** 1/1**, conv.** 88% (based on crude ^{1}H NMR analysis).

Tris((2,6-dimethylheptan-4-yl)oxy)(4-(4,4,6,6-tetramethyl-1,3,2-dioxaborinan-2-yl)-2-(trifluoromethoxy)phenyl)silane (67)

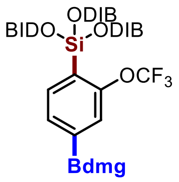

Tris((2,6-dimethylheptan-4-yl)oxy)(2-(trifluoromethyl)phenyl)silane **67a** (0.2 mmol, 115.8 mg), B_2dmg_2 (84.6 mg, 0.3 mmol, 1.5 equiv), $[Ir(cod)OMe]_2$ (2.0 mg, 0.003 mmol), Me_4phen (1.5 mg, 0.006 mmol), and cyclohexane (1.0 mL) were added to a 15 mL glass vial under air atmosphere. The glass vial was capped with a teflon pressure cap and placed into an aluminum block pre-heated to 100 ºC for 30 minutes. After completion, cyclohexane was removed under reduced pressure and chromatographic separation with silica gel (20% ethyl acetate in hexane as eluent) gave the borylated product **67** (126.2 mg, 83%) as colorless liquid. The *para:meta* ratio of products was reported from analysis of ^{1}H NMR.

^{1}**H NMR** (400 MHz, $CDCl_3$) δ 7.74 (d, *J* = 7.3 Hz, 1H), 7.67 (d, *J* = 7.2 Hz, 1H), 7.62 (s, 1H), 4.16 (p, *J* = 6.4 Hz, 3H), 1.94 (s, 2H), 1.82 – 1.65 (m, 6H), 1.52 – 1.43 (m, 18H), 1.35 – 1.24 (m, 6H), 0.85 (dd, *J* = 6.6, 2.2 Hz, 36H). ^{13}**C NMR** (101 MHz, $CDCl_3$) δ 154.0, 136.8, 130.6, 128.6, 120.8 (d, *J* = 257.3 Hz), 121.5 (q, *J* = 16.8 Hz), 71.3, 70.27, 49.2, 46.9, 31.9, 24.6, 23.1, 23.0. ^{19}**F NMR** (376 MHz, $CDCl_3$) δ -55.0. ^{11}**B NMR** (128 MHz, $CDCl_3$) δ 25.9. **HRMS (ESI)** *m/z* Calcd for C41H74BF3NaO6Si [M+Na]+ 781.5192, Found: 781.5184.

Same reaction was performed with the **trimethoxy(2-(trifluoromethoxy)phenyl)silane** under identical conditions. Outcomes: **p/m:** 4/1**, conv.** 85% (based on crude ^{1}H NMR analysis).

***Para*-selective borylation of tris((2,6-dimethylheptan-4-yl)oxy)(m-tolyl)silane (68)**

Tris((2,6-dimethylheptan-4-yl)oxy)(m-tolyl)silane **68a** (0.2 mmol, 109.8 mg), B_2dmg_2 (84.6 mg, 0.3 mmol, 1.5 equiv), $[Ir(cod)OMe]_2$ (2.0 mg, 0.003 mmol), Me_4phen (1.5 mg, 0.006 mmol), and cyclohexane (1.0 mL) were added to a 15 mL glass vial under air atmosphere. The glass vial was capped with a teflon pressure cap and placed into an aluminum block pre-heated to 100 ºC for 30 minutes. After completion, cyclohexane was removed under reduced

pressure and chromatographic separation with silica gel (20% ethyl acetate in hexane as eluent) gave the borylated product **68** (103.3 mg, 75%) as colorless liquid. The *para:meta* ratio of products was reported from analysis of ^{1}H NMR. Same reaction was performed with the **triethoxy(m-tolyl)silane** under identical conditions. Outcomes: **p/m:** < 20/1**, conv.** 80% (based on crude ^{1}H NMR analysis).

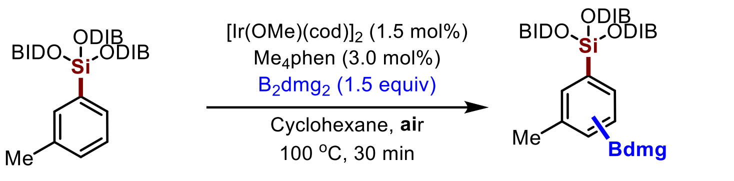

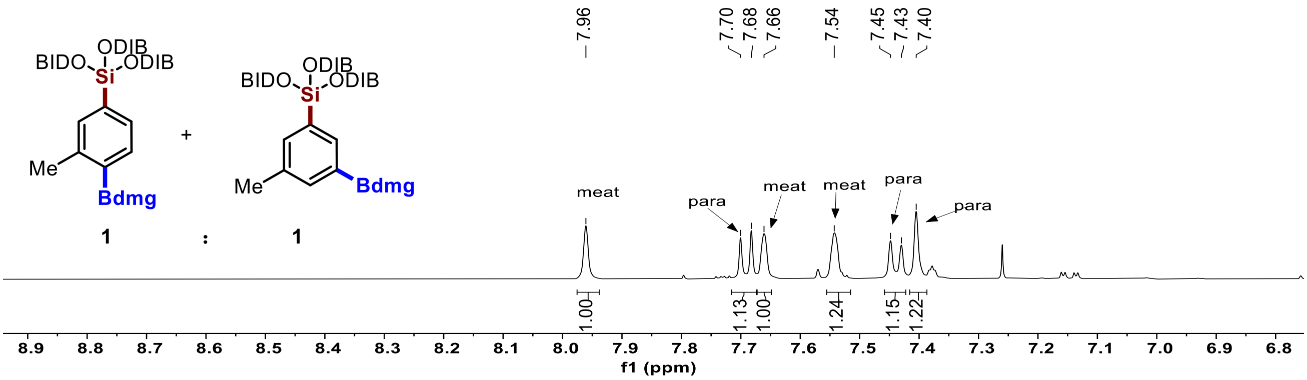

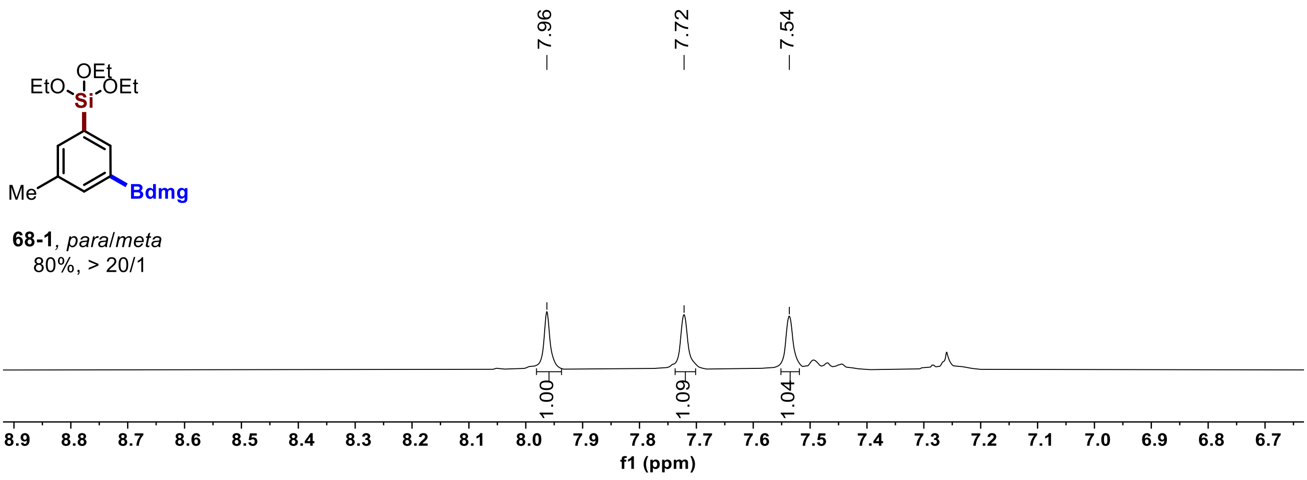

Supplementary Fig. 28: ^{1}H-NMR spectra of crude **68 and 68-1** (25 °C, 400 MHz, $CDCl_3$)

***Para*-Selective borylation of tris((2,6-dimethylheptan-4-yl)oxy)(3-chlorophenyl)silane (69)**

Tris((2,6-dimethylheptan-4-yl)oxy) (3-chlorophenyl)silane **69a** (0.2 mmol, 113.9 mg), B_2dmg_2 (84.6 mg, 0.3 mmol, 1.5 equiv), $[Ir(cod)OMe]_2$ (2.0 mg, 0.003 mmol), Me_4phen (1.5 mg, 0.006 mmol), and cyclohexane (1.0 mL) were added to a 15 mL glass vial under air atmosphere. The glass vial was capped with a teflon pressure cap and placed into an aluminum block pre-heated to 100 ºC for 30 minutes. After completion, cyclohexane was removed under

reduced pressure and chromatographic separation with silica gel (20% ethyl acetate in hexane as eluent) gave the borylated product **69** (110.7 mg, 78%) as colorless liquid. The *para:meta* ratio of products was reported from analysis of ^{1}H NMR. Same reaction was performed with the **(3-chlorophenyl)triethoxysilane** under identical conditions. Outcomes: **p/m:** 0/1**, conv.** 82% (based on crude ^{1}H NMR analysis).

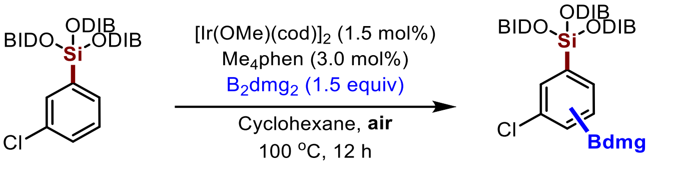

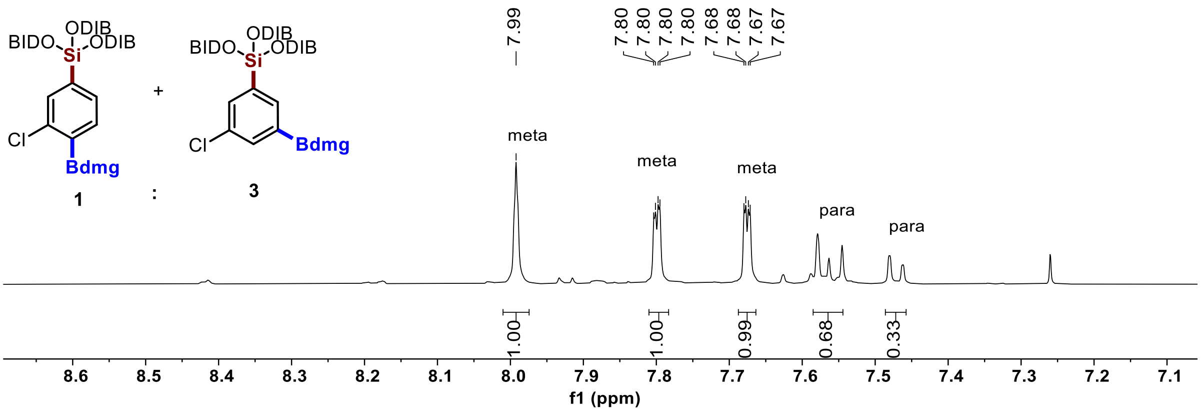

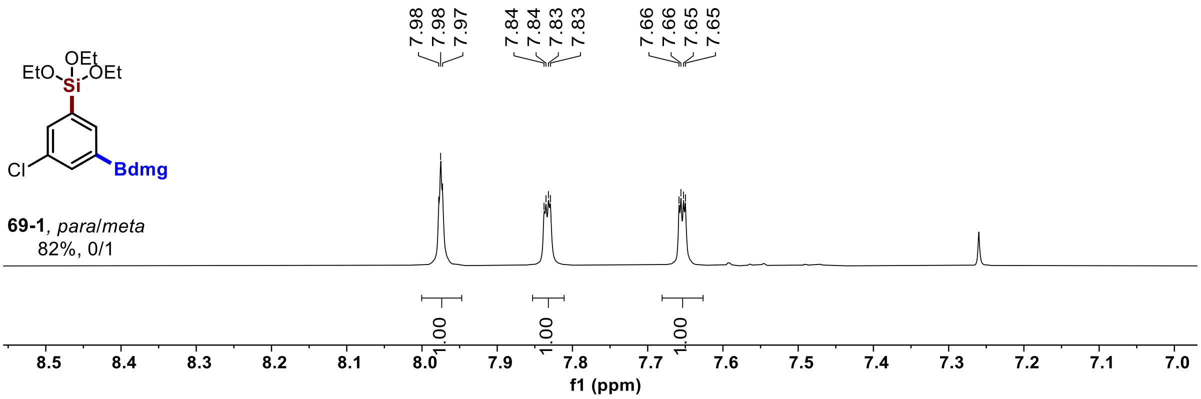

Supplementary Fig. 29: ^{1}H-NMR spectra of crude **2c** (25 °C, 400 MHz, $CDCl_3$)

Bis((2,6-dimethylheptan-4-yl)oxy)(ethoxy)(2-isopropoxy-4-(4,4,6,6-tetramethyl-1,3,2-dioxaborinan-2-yl)phenyl)silane (70)

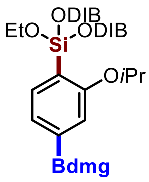

Bis((2,6-dimethylheptan-4-yl)oxy)(ethoxy)(2-isopropoxyphenyl)silane **70a** (0.2 mmol, 99.0 mg), B_2dmg_2 (84.6 mg, 0.3 mmol, 1.5 equiv), $[Ir(cod)OMe]_2$ (2.0 mg, 0.003 mmol), Me_4phen (1.5 mg, 0.006 mmol), and cyclohexane (1.0 mL) were added to a 15 mL glass vial under air atmosphere. The

glass vial was capped with a teflon pressure cap and placed into an aluminum block pre-heated to 100 ºC for 30 minutes. After completion, cyclohexane was removed under reduced pressure and chromatographic separation with silica gel (20% ethyl acetate in hexane as eluent) gave the borylated product **70** (88.8 mg, 70%) as colorless liquid. The *para:meta* ratio of products was reported from analysis of ^{1}H NMR.

^{1}H NMR (400 MHz, $CDCl_3$) δ 7.97 (d, *J* = 7.2 Hz, 1H), 7.66 (d, *J* = 7.2 Hz, 1H), 7.54 (s, 1H), 5.02 (hept, *J* = 6.0 Hz, 1H), 4.57 – 4.34 (m, 2H), 4.13 (q, *J* = 7.1 Hz, 2H), 2.21 (s, 2H), 2.14 – 2.01 (m, 4H), 1.85 – 1.76 (m, 4H), 1.73 (s, 12H), 1.68 (d, *J* = 6.0 Hz, 6H), 1.61 – 1.51 (m, 4H), 1.50 (t, *J* = 7.0 Hz, 3H), 1.20 – 1.07 (m, 24H). **^{13}C NMR** (101 MHz, $CDCl_3$) δ 162.0, 137.4, 125.4, 124.3, 115.8, 70.9, 69.6, 68.8, 58.5, 49.2, 47.0 (d, *J* = 4.4 Hz), 32.0, 24.5, 23.3, 23.0 (d, *J* = 7.9 Hz), 22.3, 18.4. **^{11}B NMR** (128 MHz, $CDCl_3$) δ 25.9. **HRMS (ESI)** *m/z* Calcd for $C_{36}H_{68}BO_6Si$ $[M+H]^+$ 635.4873, Found: 635.4878.

Bis((2,6-dimethylheptan-4-yl)oxy)(ethoxy)(2-ethyl-4-(4,4,6,6-tetramethyl-1,3,2-dioxaborinan-2-yl)phenyl)silane (71)

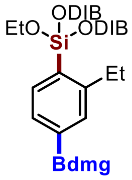

Bis((2,6-dimethylheptan-4-yl)oxy)(ethoxy)(2-ethylphenyl)silane **71a** (0.2 mmol, 93.0 mg), B_2dmg_2 (84.6 mg, 0.3 mmol, 1.5 equiv), $[Ir(cod)OMe]_2$ (2.0 mg, 0.003 mmol), Me_4phen (1.5 mg, 0.006 mmol), and cyclohexane (1.0 mL) were added to a 15 mL glass vial under air atmosphere. The glass vial was capped with a teflon pressure cap and placed into an aluminum block pre-heated to 100 ºC for 30 minutes. After completion, cyclohexane was removed under reduced pressure and chromatographic separation with silica gel (20% ethyl acetate in hexane as eluent) gave the borylated product **71** (92.0 mg, 76%) as colorless liquid. The *para:meta* ratio of products was reported from analysis of ^{1}H NMR.

^{1}H NMR (400 MHz, $CDCl_3$) δ 7.71 (d, *J* = 8.0 Hz, 2H), 7.64 (d, *J* = 7.3 Hz, 1H), 4.11 (p, *J* = 6.3 Hz, 2H), 3.88 (q, *J* = 7.0 Hz, 2H), 2.89 (q, *J* = 7.5 Hz, 2H), 1.95 (s, 2H), 1.82 – 1.69 (m, 4H), 1.56 – 1.42 (m, 16H), 1.36 – 1.21 (m, 10H), 0.99 – 0.80 (m, 24H). **^{13}C NMR** (101 MHz, $CDCl_3$) δ 149.6, 135.5, 133.6, 133.4, 130.1, 70.9, 70.0, 58.6, 49.2, 47.0 (d, *J* = 4.2 Hz), 32.0, 29.5, 24.6 (d, *J* = 2.3 Hz), 23.2 (d, *J* = 3.3 Hz), 23.0, 22.9, 18.3, 16.7. **^{11}B NMR** (128 MHz, $CDCl_3$) δ 27.3. **HRMS (ESI)** *m/z* Calcd for $C_{35}H_{65}BNaO_5Si$ $[M+Na]^+$ 627.4587, Found: 627.4598.

Bis((2,6-dimethylheptan-4-yl)oxy)(ethoxy)(2-isopropyl-4-(4,4,6,6-tetramethyl-1,3,2-dioxaborinan-2-yl)phenyl)silane (72)

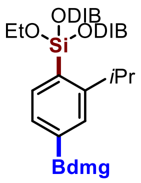

Bis((2,6-dimethylheptan-4-yl)oxy)(ethoxy)(2-isopropylphenyl)silane **72a** (0.2 mmol, 95.8 mg), B_2dmg_2 (84.6 mg, 0.3 mmol, 1.5 equiv), $[Ir(cod)OMe]_2$ (2.0 mg, 0.003 mmol), Me_4phen (1.5 mg, 0.006 mmol), and cyclohexane (1.0 mL) were added to a 15 mL glass vial under air atmosphere. The glass vial was capped with a teflon pressure cap and placed into an aluminum block pre-heated to 100 ºC for 30 minutes. After completion, cyclohexane was removed under reduced pressure and chromatographic separation with silica gel (20% ethyl acetate in hexane as eluent) gave the borylated product **72** (95.3 mg, 77%) as colorless liquid. The *para:meta* ratio of products was reported from analysis of ^{1}H NMR.

^{1}H NMR (400 MHz, $CDCl_3$) δ 7.78 (s, 1H), 7.68 (d, *J* = 7.4 Hz, 1H), 7.63 (dd, *J* = 7.4, 1.2 Hz, 1H), 4.13 (p, *J* = 6.5 Hz, 2H), 3.88 (q, *J* = 7.0 Hz, 2H), 3.47 – 3.33 (m, 1H), 1.92 (s, 2H), 1.83 – 1.67 (m, 4H), 1.51 – 1.41 (m, 16H), 1.37 – 1.22 (m, 13H), 0.94 – 0.73 (m, 24H). **^{13}C NMR** (101 MHz, $CDCl_3$) δ 154.4, 135.3, 133.6, 130.3, 130.2, 70.1, 58.7, 49.2, 46.9 (d, *J* = 5.1 Hz), 33.6, 32.0, 24.9, 24.6, 24.6, 23.2, 23.2, 23.0, 22.9, 18.3. **^{11}B NMR** (128 MHz, $CDCl_3$) δ 26.7. **HRMS (ESI)** *m/z* Calcd for $C_{36}H_{68}BO_5Si$ $[M+H]^+$ 641.4743, Found: 641.4741.

(2-cyclopropyl-4-(4,4,6,6-tetramethyl-1,3,2-dioxaborinan-2-yl)phenyl)tris((2,6-dimethylheptan-4-yl)oxy)silane (73)

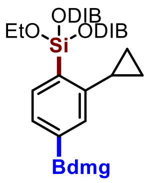

(2-cyclopropylphenyl)bis((2,6-dimethylheptan-4-yl)oxy)(ethoxy)silane **73a** (0.2 mmol, 95.4 mg), B_2dmg_2 (84.6 mg, 0.3 mmol, 1.5 equiv), $[Ir(cod)OMe]_2$ (2.0 mg, 0.003 mmol), Me_4phen (1.5 mg, 0.006 mmol), and cyclohexane (1.0 mL) were added to a 15 mL glass vial under air atmosphere. The glass vial was capped with a teflon pressure cap and placed into an aluminum block pre-heated to 100 ºC for 30 minutes. After completion, cyclohexane was removed under reduced pressure and chromatographic separation with silica gel (20% ethyl acetate in hexane as eluent) gave the borylated product **73** (93.7 mg, 76%) as colorless liquid. The *para:meta* ratio of products was reported from analysis of ^{1}H NMR.

^{1}H NMR (400 MHz, $CDCl_3$) δ 7.70 (d, *J* = 7.4 Hz, 1H), 7.55 (d, *J* = 7.4 Hz, 1H), 7.11 (s, 1H), 4.14 (p, *J* = 6.3 Hz, 3H), 2.53 – 2.34 (m, 1H), 1.91 (s, 2H), 1.83 – 1.69 (m, 4H), 1.55 – 1.44 (m, 4H), 1.43 (s, 12H), 1.38 – 1.27 (m, 5H), 1.02 – 0.94 (m, 2H), 0.84 (dd, *J* = 6.7, 3.3 Hz, 38H). **^{13}C NMR** (101 MHz, $CDCl_3$) δ 148.34, 135.41, 135.15, 129.45, 125.81, 70.69, 69.97, 49.06, 46.82, 31.85, 24.46, 23.01, 22.94, 15.43, 11.29. **^{11}B NMR** (128 MHz, $CDCl_3$) δ 26.1. **HRMS (ESI)** *m/z* Calcd for $C_{36}H_{65}BNaO_5Si$ $[M+Na]^+$ 639.4587, Found: 639.4598.

(2-((3,5-dimethoxybenzyl)oxy)-4-(4,4,6,6-tetramethyl-1,3,2-dioxaborinan-2-yl)phenyl)triethoxysilane (74)

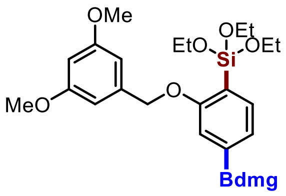

(2-((3,5-dimethoxybenzyl)oxy)phenyl)triethoxysilane **74a** (0.2 mmol, 81.3 mg), B_2dmg_2 (84.6 mg, 0.3 mmol, 1.5 equiv), $[Ir(cod)OMe]_2$ (2.0 mg, 0.003 mmol), Me_4phen (1.5 mg, 0.006 mmol), and cyclohexane (1.0 mL) were added to a 15 mL glass vial under air atmosphere. The glass vial was capped with a teflon pressure cap and placed into an aluminum block pre-heated to 100 ºC for 30 minutes. After completion, cyclohexane was removed under reduced pressure and chromatographic separation with silica gel (20% ethyl acetate in hexane as eluent) gave the borylated product **74** (89.6 mg, 82%) as colorless liquid. The *para:meta* ratio of products was reported from analysis of ^{1}H NMR.

^{1}H NMR (400 MHz, $CDCl_3$) δ 7.68 (d, *J* = 7.2 Hz, 1H), 7.47 (dd, *J* = 7.0, 0.8 Hz, 1H), 7.38 (s, 1H), 6.77 (d, *J* = 2.3 Hz, 2H), 6.41 (t, *J* = 2.3 Hz, 1H), 5.12 (s, 2H), 3.92 – 3.74 (m, 12H), 1.92 (s, 2H), 1.44 (s, 12H), 1.18 (t, *J* = 7.0 Hz, 9H). **^{13}C NMR** (101 MHz, $CDCl_3$) δ 162.92, 160.87, 139.94, 136.90, 126.36, 121.74, 115.43, 105.09, 99.87, 70.98, 69.65, 58.62, 55.39, 49.03, 31.82, 18.24. **^{11}B NMR** (128 MHz, $CDCl_3$) δ 25.1. **HRMS (ESI)** *m/z* Calcd for $C_{28}H_{43}BNaO_8Si$ $[M+Na]^+$ 569.2712, Found: 569.2701.

Tris((2,6-dimethylheptan-4-yl)oxy)(5-(4,4,6,6-tetramethyl-1,3,2-dioxaborinan-2-yl)thiophen-2-yl)silane (75)

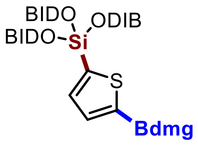

Tris((2,6-dimethylheptan-4-yl)oxy)(thiophen-2-yl)silane **75a** (0.2 mmol, 108.2 mg), B_2dmg_2 (84.6 mg, 0.3 mmol, 1.5 equiv), $[Ir(cod)OMe]_2$ (2.0 mg, 0.003 mmol), Me_4phen (1.5 mg, 0.006 mmol), and cyclohexane (1.0 mL) were added to a 15 mL glass vial under air atmosphere. The glass vial was capped with a teflon pressure cap and placed into an aluminum block pre-heated to 100 ºC for 30 minutes. After completion, cyclohexane was removed under reduced pressure and chromatographic separation with silica gel (20% ethyl acetate in hexane as eluent) gave the borylated product **75** (119.8 mg, 88%) as colorless liquid. The *para:meta* ratio of products was reported from analysis of ^{1}H NMR.

^{1}H NMR (400 MHz, $CDCl_3$) δ 7.61 (d, *J* = 3.3 Hz, 1H), 7.41 (d, *J* = 3.3 Hz, 1H), 4.53 – 3.79 (m, 3H), 1.93 (s, 2H), 1.85 – 1.70 (m, 6H), 1.56 – 1.44 (m, 6H), 1.44 (s, 12H), 1.34 – 1.26 (m, 6H), 0.86 (dd, *J* = 6.7, 2.1 Hz, 36H). **^{13}C NMR** (101 MHz, $CDCl_3$) δ 138.7, 136.9, 135.7, 71.3, 70.2, 49.2, 47.0, 31.9, 24.5, 23.2, 22.9. **^{11}B NMR** (128 MHz, $CDCl_3$) δ 25.0. **HRMS (ESI)** *m/z* Calcd for $C_{31}H_{59}BNaO_5SSi$ $[M+Na]^+$ 605.3838, Found: 605.3841.

Same reaction was performed with the **triethoxy(thiophen-2-yl)silane (3rr)** under identical conditions. Outcomes: **C5/others:** > 20/1**, conv.** 89% (based on crude ^{1}H NMR analysis).

Triethoxy(3-methyl-5-(4,4,6,6-tetramethyl-1,3,2-dioxaborinan-2-yl)thiophen-2-yl)silane (76)

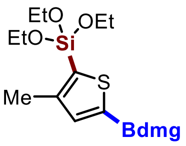

Tris((2,6-dimethylheptan-4-yl)oxy)(thiophen-2-yl)silane **76a** (0.2 mmol, 52.1 mg), B_2dmg_2 (84.6 mg, 0.3 mmol, 1.5 equiv), $[Ir(cod)OMe]_2$ (2.0 mg, 0.003 mmol), Me_4phen (1.5 mg, 0.006 mmol), and cyclohexane (1.0 mL) were added to a 15 mL glass vial under air atmosphere. The glass vial was capped with a teflon pressure cap and placed into an aluminum block pre-heated to 100 ºC for 30 minutes. After completion, cyclohexane was removed under reduced pressure and chromatographic separation with silica gel (20% ethyl acetate in hexane as eluent) gave the borylated product **76** (68.1 mg, 85%) as colorless liquid. The *para:meta* ratio of products was reported from analysis of ^{1}H NMR.

^{1}H NMR (400 MHz, $CDCl_3$) δ 7.47 (s, 1H), 3.88 (q, *J* = 7.0 Hz, 6H), 2.43 (s, 3H), 1.92 (s, 2H), 1.43 (s, 12H), 1.26 (t, *J* = 7.0 Hz, 9H). **^{13}C NMR** (101 MHz, $CDCl_3$) δ 148.2, 139.3, 129.8, 71.4, 58.8, 49.2, 31.8, 18.3, 16.2. **^{11}B NMR** (128 MHz, $CDCl_3$) δ 24.8. **HRMS (ESI)** *m/z* Calcd for $C_{17}H_{31}BNaO_5SSi$ $[M+Na]^+$ 409.1647, Found: 409.1655.

(5-chloro-4-(4,4,6,6-tetramethyl-1,3,2-dioxaborinan-2-yl)thiophen-2-yl)triethoxy silane (77)

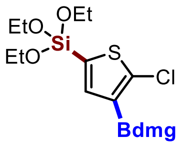

(5-chlorothiophen-2-yl)triethoxysilane **77a** (0.2 mmol, 56.2 mg), B_2dmg_2 (84.6 mg, 0.3 mmol, 1.5 equiv), $[Ir(cod)OMe]_2$ (2.0 mg, 0.003 mmol), Me_4phen (1.5 mg, 0.006 mmol), and cyclohexane (1.0 mL) were added to a 15 mL glass vial under air atmosphere. The glass vial was capped with a teflon pressure cap and placed into an aluminum block pre-heated to 100 ºC for 30 minutes. After completion, cyclohexane was removed under reduced pressure and chromatographic separation with silica gel (20% ethyl acetate in hexane as eluent) gave the borylated product **77** (69.0 mg, 82%) as colorless liquid. The *para:meta* ratio of products was reported from analysis of ^{1}H NMR.

^{1}H NMR (400 MHz, $CDCl_3$) δ 7.47 (s, 1H), 3.86 (q, *J* = 7.0 Hz, 6H), 1.91 (s, 2H), 1.41 (s, 12H), 1.24 (t, *J* = 7.0 Hz, 9H). **^{13}C NMR** (101 MHz, $CDCl_3$) δ 144.2, 142.9, 127. 5, 71.4, 59.1, 49.1, 31. 9, 18.3. **^{11}B NMR** (128 MHz, $CDCl_3$) δ 24.8. **HRMS (ESI)** *m/z* Calcd for $C_{17}H_{30}BClNaO_5SSi$ $[M+Na]^+$ 443.1257, Found: 443.1265.

(3,5-difluoro-4-(4,4,6,6-tetramethyl-1,3,2-dioxaborinan-2-yl)phenyl) triethoxy silane (78)

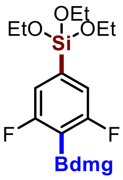

(3,5-difluorophenyl)triethoxysilane **78a** (0.2 mmol, 55.3 mg), B_2dmg_2 (84.6 mg, 0.3 mmol, 1.5 equiv), $[Ir(cod)OMe]_2$ (2.0 mg, 0.003 mmol), Me_4phen (1.5 mg, 0.006 mmol), and cyclohexane (1.0 mL) were added to a 15 mL glass vial under air atmosphere. The glass vial was capped with a teflon pressure cap and placed into an aluminum block pre-heated to 100 ºC for 30 minutes. After completion, cyclohexane was removed under reduced pressure and chromatographic separation with silica gel (20% ethyl acetate in hexane as

eluent) gave the borylated product **78** (69.9 mg, 84%) as colorless liquid. The *para:meta* ratio of products was reported from analysis of ^{1}H NMR.

^{1}H NMR (400 MHz, $CDCl_3$) δ 7.18 – 6.81 (m, 2H), 3.82 (q, *J* = 7.0 Hz, 6H), 1.98 (s, 2H), 1.45 (s, 12H), 1.22 (t, *J* = 7.0 Hz, 9H). **^{13}C NMR** (101 MHz, $CDCl_3$) δ 166.5 (d, $J_{C\text{-}F}$ = 12.3 Hz), 164.0 (d, $J_{C\text{-}F}$ = 12.6 Hz), 136.1, 122.4 – 113.4 (m), 72.4, 59.0, 49.1, 31.7, 18.3. **^{19}F NMR** (376 MHz, $CDCl_3$) δ -104.1. **^{11}B NMR** (128 MHz, $CDCl_3$) δ 25.6. **HRMS (ESI)** *m/z* Calcd for $C_{19}H_{31}BF_2NaO_5Si$ $[M+Na]^+$ 439.1894, Found: 439.1898.

(3-chloro-5-fluoro-4-(4,4,6,6-tetramethyl-1,3,2-dioxaborinan-2-yl)phenyl) triethoxysilane (79)

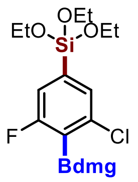

(3-chloro-5-fluorophenyl)triethoxysilane **79a** (0.2 mmol, 58.6 mg), B_2dmg_2 (84.6 mg, 0.3 mmol, 1.5 equiv), $[Ir(cod)OMe]_2$ (2.0 mg, 0.003 mmol), Me_4phen (1.5 mg, 0.006 mmol), and cyclohexane (1.0 mL) were added to a 15 mL glass vial under air atmosphere. The glass vial was capped with a teflon pressure cap and placed into an aluminum block pre-heated to 100 ºC for 30 minutes. After completion, cyclohexane was removed under reduced pressure and chromatographic separation with silica gel (20% ethyl acetate in hexane as eluent) gave the borylated product **79** (71.8 mg, 83%) as colorless liquid. The *para:meta* ratio of products was reported from analysis of ^{1}H NMR.

^{1}H NMR (400 MHz, $CDCl_3$) δ 7.36 (s, 1H), 7.16 (dd, *J* = 7.6, 0.6 Hz, 1H), 3.81 (q, *J* = 7.0 Hz, 6H), 2.00 (s, 2H), 1.46 (s, 12H), 1.22 (t, *J* = 7.0 Hz, 9H). **^{13}C NMR** (101 MHz, $CDCl_3$) δ 164.5 (d, $J_{C\text{-}F}$ = 247.1 Hz), 137.6 (d, $J_{C\text{-}F}$ = 10.9 Hz), 135.6 (d, $J_{C\text{-}F}$ = 5.3 Hz), 130.5 (d, $J_{C\text{-}F}$ = 3.0 Hz), 118.9 (d, $J_{C\text{-}F}$ = 21.9 Hz), 72.6, 59.0, 49.1, 31.6, 18.3. **^{19}F NMR** (376 MHz, $CDCl_3$) δ -104.9. **^{11}B NMR** (128 MHz, $CDCl_3$) δ 24.5. **HRMS (ESI)** *m/z* Calcd for $C_{19}H_{31}BFClNaO_5Si$ $[M+Na]^+$ 455.1599, Found: 455.1593.

(5-chloro-2-isopropoxy-4-(4,4,6,6-tetramethyl-1,3,2-dioxaborinan-2-yl)phenyl) triethoxysilane (80)

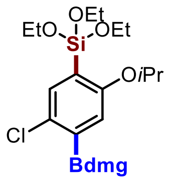

(5-chloro-2-isopropoxyphenyl)triethoxysilane **80a** (0.2 mmol, 66.6 mg), B_2dmg_2 (84.6 mg, 0.3 mmol, 1.5 equiv), $[Ir(cod)OMe]_2$ (2.0 mg, 0.003 mmol), Me_4phen (1.5 mg, 0.006 mmol), and cyclohexane (1.0 mL) were added to a 15 mL glass vial under air atmosphere. The glass vial was capped with a teflon pressure cap and placed into an aluminum block pre-heated to 100 ºC for 30 minutes. After completion, cyclohexane was removed under reduced pressure and chromatographic separation with silica gel (20% ethyl acetate in hexane as eluent) gave the borylated product **80** (63.4 mg, 67%) as colorless liquid. The *para:meta* ratio of products was reported from analysis of ^{1}H NMR.

^{1}H NMR (400 MHz, $CDCl_3$) δ 7.54 (s, 1H), 6.95 (s, 1H), 4.62 (hept, J = 6.1 Hz, 1H), 3.82 (q, J = 7.0 Hz, 6H), 1.94 (s, 2H), 1.44 (s, 12H), 1.33 (d, J = 6.1 Hz, 6H), 1.20 (t, J = 7.0 Hz, 9H). **^{13}C NMR** (101 MHz, $CDCl_3$) δ 160.6, 138.2, 129.2, 123.5, 116.8, 71.9, 69.4, 58.7, 49.0, 31.6, 22.0, 18.3. **^{11}B NMR** (128 MHz, $CDCl_3$) δ 26.4. **HRMS (ESI)** *m/z* Calcd for $C_{22}H_{38}BClNaO_6Si$ $[M+Na]^+$ 495.2111,Found: 495.2118.

(5-chloro-2-methoxy-4-(4,4,6,6-tetramethyl-1,3,2-dioxaborinan-2-yl)phenyl) triethoxysilane (81)

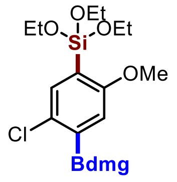

(5-chloro-2-methoxyphenyl)triethoxysilane **81a** (0.2 mmol, 60.1 mg), B_2dmg_2 (84.6 mg, 0.3 mmol, 1.5 equiv), $[Ir(cod)OMe]_2$ (2.0 mg, 0.003 mmol), Me_4phen (1.5 mg, 0.006 mmol), and cyclohexane (1.0 mL) were added to a 15 mL glass vial under air atmosphere. The glass vial was capped with a teflon pressure cap and placed into an aluminum block pre-heated to 100 ºC for 30 minutes. After completion, cyclohexane was removed under reduced pressure and chromatographic separation with silica gel (20% ethyl acetate in hexane as eluent) gave the borylated product **81** (75.6 mg, 85%) as colorless liquid. The *para:meta* ratio of products was reported from analysis of ^{1}H NMR.

^{1}H NMR (400 MHz, $CDCl_3$) δ 7.53 (s, 1H), 6.97 (s, 1H), 3.89 – 3.76 (m, 9H), 1.95 (s, 2H), 1.45 (s, 12H), 1.20 (t, J = 7.0 Hz, 9H). **^{13}C NMR** (101 MHz, $CDCl_3$) δ 162.3, 137.9, 130.0, 122.9, 115.39, 72.0, 58.8, 55.5, 49.0, 31.8, 18.3. **^{11}B NMR** (128 MHz, $CDCl_3$) δ 26.7. **HRMS (ESI)** *m/z* Calcd for $C_{20}H_{34}BClNaO_6Si$ $[M+Na]^+$ 467.1798, Found: 467.1789.

(5-fluoro-2-(methoxymethoxy)-4-(4,4,6,6-tetramethyl-1,3,2-dioxaborinan-2-yl)phenyl) triethoxysilane (82)

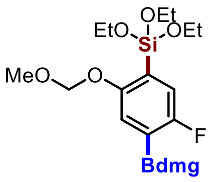

Triethoxy(5-fluoro-2-(methoxymethoxy)phenyl)silane **82a** (0.2 mmol, 63.7 mg), B_2dmg_2 (84.6 mg, 0.3 mmol, 1.5 equiv), $[Ir(cod)OMe]_2$ (2.0 mg, 0.003 mmol), Me_4phen (1.5 mg, 0.006 mmol), and cyclohexane (1.0 mL) were added to a 15 mL glass vial under air atmosphere. The glass vial was capped with a teflon pressure cap and placed into an aluminum block pre-heated to 100 ºC for 30 minutes. After completion, cyclohexane was removed under reduced pressure and chromatographic separation with silica gel (20% ethyl acetate in hexane as eluent) gave the borylated product **82** (80.7 mg, 88%) as colorless liquid. The *para:meta* ratio of products was reported from analysis of ^{1}H NMR.

^{1}H NMR (400 MHz, $CDCl_3$) δ 7.28 (d, J = 4.3 Hz, 1H), 7.25 (d, J = 8.8 Hz, 1H), 5.18 (s, 2H), 3.85 (q, J = 7.0 Hz, 6H), 3.50 (s, 3H), 1.93 (s, 2H), 1.43 (s, 12H), 1.21 (t, J = 7.0 Hz, 9H). **^{13}C NMR** (101 MHz, $CDCl_3$) δ 161.7 (d, J_{C-F} = 244.6 Hz), 157.6 (d, J_{C-F} = 1.4 Hz), 124.8 (d, J_{C-F} = 5.7 Hz), 123.6 (d, J_{C-F} = 25.5 Hz), 119.5 (d, J_{C-F} = 7.9 Hz),

94.9, 71.6, 58.8, 56.2, 49.1,31.8, 18.3. **^{19}F NMR** (376 MHz, $CDCl_3$) δ -114.9. **^{11}B NMR** (128 MHz, $CDCl_3$) δ 25.5. **HRMS (ESI)** *m/z* Calcd for $C_{21}H_{36}BFNaO_7Si$ $[M+Na]^+$ 481.2200, Found: 481.2205.

(2-((3,5-dimethoxybenzyl)oxy)-5-fluoro-4-(4,4,6,6-tetramethyl-1,3,2-dioxaborinan -2-yl)phenyl)triethoxysilane (83)

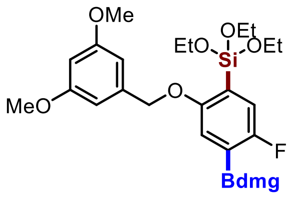

(2-((3,5-dimethoxybenzyl)oxy)-5-fluorophenyl)triethoxysilane **83a** (0.2 mmol, 84.9 mg), B_2dmg_2 (84.6 mg, 0.3 mmol, 1.5 equiv), $[Ir(cod)OMe]_2$ (2.0 mg, 0.003 mmol), Me_4phen (1.5 mg, 0.006 mmol), and cyclohexane (1.0 mL) were added to a 15 mL glass vial under air atmosphere. The glass vial was capped with a teflon pressure cap and placed into an aluminum block pre-heated to 100 ºC for 30 minutes. After completion, cyclohexane was removed under reduced pressure and chromatographic separation with silica gel (20% ethyl acetate in hexane as eluent) gave the borylated product **83** (88.1 mg, 78%) as colorless liquid. The *para:meta* ratio of products was reported from analysis of ^{1}H NMR.

^{1}H NMR (400 MHz, $CDCl_3$) δ 7.29 (d, J = 8.9 Hz, 1H), 7.16 (d, J = 4.2 Hz, 1H), 6.72 (d, J = 2.3 Hz, 2H), 6.40 (t, J = 2.3 Hz, 1H), 5.05 (s, 2H), 3.91 – 3.77 (m, 12H), 1.94 (s, 2H), 1.44 (s, 12H), 1.18 (t, J = 7.0 Hz, 9H). **^{13}C NMR** (101 MHz, $CDCl_3$) δ 161.4 (d, J_{C-F} = 242.5 Hz), 161.0, 158.9, 124.5 (d, J_{C-F} = 5.6 Hz), 124.0 (d, J_{C-F} = 25.6 Hz), 117.3 (d, J_{C-F} = 7.8 Hz), 105.2, 99.9, 71.6, 70.5, 58.8, 55.5, 49.1, 31.9, 18.3. **^{19}F NMR** (376 MHz, $CDCl_3$) δ -115.8. **^{11}B NMR** (128 MHz, $CDCl_3$) δ 26.0. **HRMS (ESI)** *m/z* Calcd for $C_{28}H_{42}BFNaO_8Si$ $[M+Na]^+$ 587.2618, Found: 587.2622.

(2-((3,5-dimethylphenoxy)methyl)-5-fluoro-4-(4,4,6,6-tetramethyl-1,3,2-dioxaborinan-2-yl)phenyl)triethoxysilane (84)

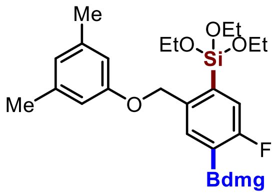

(2-((3,5-dimethylphenoxy)methyl)-5-fluorophenyl)triethoxysilane **84a** (0.2 mmol, 78.5 mg), B_2dmg_2 (84.6 mg, 0.3 mmol, 1.5 equiv), $[Ir(cod)OMe]_2$ (2.0 mg, 0.003 mmol), Me_4phen (1.5 mg, 0.006 mmol), and cyclohexane (1.0 mL) were added to a 15 mL glass vial under air atmosphere. The glass vial was capped with a teflon pressure cap and placed into an aluminum block pre-heated to 100 ºC for 30 minutes. After completion, cyclohexane was removed under reduced pressure and chromatographic separation with silica gel (20% ethyl acetate in hexane as eluent) gave the borylated product **84** (94.8 mg, 89%) as colorless liquid. The *para:meta* ratio of products was reported from analysis of ^{1}H NMR.

^{1}H NMR (400 MHz, $CDCl_3$) δ 7.87 (d, *J* = 5.7 Hz, 1H), 7.42 (d, *J* = 9.5 Hz, 1H), 6.66 (s, 2H), 6.62 (s, 1H), 5.16 (s, 2H), 3.85 (q, *J* = 7.0 Hz, 6H), 2.31 (s, 6H), 1.95 (s, 2H), 1.45 (s, 12H), 1.22 (t, *J* = 7.5 Hz, 9H). **^{13}C NMR** (101 MHz, $CDCl_3$) δ 165.9 (d, $J_{C\text{-}F}$ = 251.4 Hz), 159.2, 139.1, 138.0 (d, $J_{C\text{-}F}$ = 3.4 Hz), 136.6 (d, $J_{C\text{-}F}$ = 7.9 Hz), 134.7 (d, $J_{C\text{-}F}$ = 5.8 Hz), 122.9 (d, $J_{C\text{-}F}$ = 23.6 Hz), 122.5, 112.7, 71.5, 69.3, 58.9, 49.1, 31.9, 21.6, 18.2. **^{19}F NMR** (376 MHz, $CDCl_3$) δ -107.2. **^{11}B NMR** (128 MHz, $CDCl_3$) δ 26.1. **HRMS (ESI)** *m/z* Calcd for $C_{28}H_{42}BFNaO_6Si$ $[M+Na]^+$ 555.2720, Found: 555.2731.

(5-fluoro-2-(methoxymethyl)-4-(4,4,6,6-tetramethyl-1,3,2-dioxaborinan-2-yl) phenyl)triethoxy silane (85)

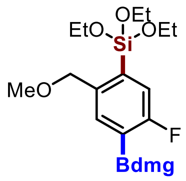

Triethoxy(5-fluoro-2-(methoxymethyl)phenyl)silane **85a** (0.2 mmol, 60.5 mg), B_2dmg_2 (84.6 mg, 0.3 mmol, 1.5 equiv), $[Ir(cod)OMe]_2$ (2.0 mg, 0.003 mmol), Me_4phen (1.5 mg, 0.006 mmol), and cyclohexane (1.0 mL) were added to a 15 mL glass vial under air atmosphere. The glass vial was capped with a teflon pressure cap and placed into an aluminum block pre-heated to 100 ºC for 30 minutes. After completion, cyclohexane was removed under reduced pressure and chromatographic separation with silica gel (20% ethyl acetate in hexane as eluent) gave the borylated product **85** (68.1 mg, 77%) as colorless liquid. The *para:meta* ratio of products was reported from analysis of ^{1}H NMR.

^{1}H NMR (400 MHz, $CDCl_3$) δ 7.74 (d, *J* = 5.8 Hz, 1H), 7.36 (d, *J* = 9.6 Hz, 1H), 4.57 (s, 2H), 3.83 (q, *J* = 7.0 Hz, 6H), 3.41 (s, 3H), 1.92 (s, 2H), 1.43 (s, 12H), 1.23 (t, *J* = 7.0 Hz, 9H). **^{13}C NMR** (101 MHz, $CDCl_3$) δ 165.7 (d, $J_{C\text{-}F}$ = 250.7 Hz), 139.3 (d, $J_{C\text{-}F}$ = 3.5 Hz), 136.0 (d, $J_{C\text{-}F}$ = 7.9 Hz), 134.5 (d, $J_{C\text{-}F}$ = 5.8 Hz), 123.0 (d, *J* = 23.8 Hz), 74.0, 71.46, 58.79, 58.31, 49.11, 31.87, 18.23. **^{19}F NMR** (376 MHz, $CDCl_3$) δ -107.7. **^{11}B NMR** (128 MHz, $CDCl_3$) δ 26.31. **HRMS (ESI)** *m/z* Calcd for $C_{21}H_{36}BFNaO_6Si$ $[M+Na]^+$ 465.2250, Found: 465.2261.

(2-(1,3-dioxolan-2-yl)-5-fluoro-4-(4,4,6,6-tetramethyl-1,3,2-dioxaborinan-2-yl) phenyl)triethoxysilane (86)

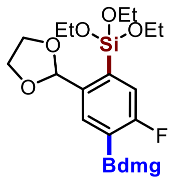

(2-(1,3-dioxolan-2-yl)-5-fluorophenyl)triethoxysilane **86a** (0.2 mmol, 66.1 mg), B_2dmg_2 (84.6 mg, 0.3 mmol, 1.5 equiv), $[Ir(cod)OMe]_2$ (2.0 mg, 0.003 mmol), Me_4phen (1.5 mg, 0.006 mmol), and cyclohexane (1.0 mL) were added to a 15 mL glass vial under air atmosphere. The glass vial was capped with a teflon pressure cap and placed into an aluminum block pre-heated to 100 ºC for 30 minutes. After completion, cyclohexane was removed under reduced pressure and chromatographic separation with silica gel (20% ethyl acetate in hexane as eluent) gave the borylated product **86** (74.3 mg, 79%) as colorless liquid. The *para:meta* ratio of products was reported from analysis of ^{1}H NMR.

^{1}H NMR (400 MHz, $CDCl_3$) δ 7.95 (d, *J* = 5.8 Hz, 1H), 7.40 (d, *J* = 9.7 Hz, 1H), 6.06 (s, 1H), 4.18 (t, *J* = 6.9 Hz, 2H), 4.00 (t, *J* = 6.8 Hz, 2H), 3.82 (q, *J* = 7.0 Hz, 6H), 1.91 (s, 2H), 1.41 (s, 12H), 1.22 (t, *J* = 7.0 Hz, 9H). **^{13}C NMR** (101 MHz, $CDCl_3$) δ 166.76 (d, $J_{C\text{-}F}$ = 252.6 Hz), 138.4 (d, $J_{C\text{-}F}$ = 3.5 Hz), 135.0 (d, $J_{C\text{-}F}$ = 6.1 Hz), 134.4 (d, $J_{C\text{-}F}$ = 8.3 Hz), 122.9 (d, $J_{C\text{-}F}$ = 23.9 Hz), 102.8, 71.4, 65.4, 58.9, 49.1, 31.9, 18.2. **^{19}F NMR** (376 MHz, $CDCl_3$) δ -105.1. **^{11}B NMR** (128 MHz, $CDCl_3$) δ 25.8. **HRMS (ESI)** *m/z* Calcd for $C_{22}H_{36}BFNaO_7Si$ $[M+Na]^+$ 493.2200, Found: 493.2196 .

(5-fluoro-2-methyl-4-(4,4,6,6-tetramethyl-1,3,2-dioxaborinan-2-yl)phenyl) triethoxysilane (87)

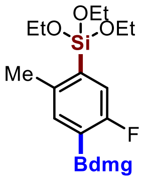

Triethoxy(5-fluoro-2-methylphenyl)silane **87a** (0.2 mmol, 54.5 mg), B_2dmg_2 (84.6 mg, 0.3 mmol, 1.5 equiv), $[Ir(cod)OMe]_2$ (2.0 mg, 0.003 mmol), Me_4phen (1.5 mg, 0.006 mmol), and cyclohexane (1.0 mL) were added to a 15 mL glass vial under air atmosphere. The glass vial was capped with a teflon pressure cap and placed into an aluminum block pre-heated to 100 ºC for 30 minutes. After completion, cyclohexane was removed under reduced pressure and chromatographic separation with silica gel (20% ethyl acetate in hexane as eluent) gave the borylated product **87** (71.8 mg, 87%) as colorless liquid. The *para:meta* ratio of products was reported from analysis of ^{1}H NMR.

^{1}H NMR (400 MHz, $CDCl_3$) δ 7.44 (d, *J* = 5.7 Hz, 1H), 7.32 (d, *J* = 9.6 Hz, 1H), 3.82 (q, *J* = 7.0 Hz, 6H), 2.45 (s, 3H), 1.93 (s, 2H), 1.43 (s, 12H), 1.23 (t, *J* = 7.0 Hz, 9H). **^{13}C NMR** (101 MHz, $CDCl_3$) δ 164.62 (d, $J_{C\text{-}F}$ = 248.2 Hz), 138.89 (d, $J_{C\text{-}F}$ = 3.6 Hz), 136.91 (d, $J_{C\text{-}F}$ = 7.2 Hz), 134.59 (d, $J_{C\text{-}F}$ = 5.8 Hz), 122.81 (d, $J_{C\text{-}F}$ = 23.3 Hz), 71.43, 58.65, 49.09, 31.88, 21.51, 18.25. **^{19}F NMR** (376 MHz, $CDCl_3$) δ -111.2. **^{11}B NMR** (128 MHz, $CDCl_3$) δ 25.8. **HRMS (ESI)** *m/z* Calcd for $C_{20}H_{34}BFNaO_5Si$ $[M+Na]^+$ 435.2145, Found: 435.2154.

(5-fluoro-2-methoxy-4-(4,4,6,6-tetramethyl-1,3,2-dioxaborinan-2-yl)phenyl) triethoxysilane (88)

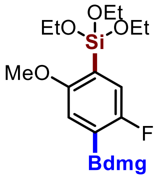

Triethoxy(5-fluoro-2-methoxyphenyl)silane **88a** (0.2 mmol, 57.7 mg), B_2dmg_2 (84.6 mg, 0.3 mmol, 1.5 equiv), $[Ir(cod)OMe]_2$ (2.0 mg, 0.003 mmol), Me_4phen (1.5 mg, 0.006 mmol), and cyclohexane (1.0 mL) were added to a 15 mL glass vial under air atmosphere. The glass vial was capped with a teflon pressure cap and placed into an aluminum block pre-heated to 100 ºC for 30 minutes. After completion, cyclohexane was removed under reduced pressure and chromatographic separation with silica gel (20% ethyl acetate in hexane as eluent) gave the borylated product **88** (72.0 mg, 84%) as colorless liquid. The *para:meta* ratio of products was reported from analysis of ^{1}H NMR.

^{1}**H NMR** (400 MHz, $CDCl_3$) δ 7.17 (d, J = 8.8 Hz, 1H), 7.01 (d, J = 4.1 Hz, 1H), 3.77 (q, J = 6.8 Hz, 9H), 1.86 (s, 2H), 1.36 (s, 12H), 1.14 (t, J = 7.0 Hz, 9H).

^{13}**C NMR** (101 MHz, $CDCl_3$) δ 161.23 (d, $J_{C\text{-}F}$ = 243.2 Hz), 159.8 (d, $J_{C\text{-}F}$ = 1.2 Hz), 124.0 (d, $J_{C\text{-}F}$ = 5.7 Hz), 123.8 (d, $J_{C\text{-}F}$ = 25.5 Hz), 115.8 (d, $J_{C\text{-}F}$ = 7.9 Hz), 71.5, 58.8, 55.9, 49.1, 31.9, 18.3. ^{19}**F NMR** (376 MHz, $CDCl_3$) δ -116.3. ^{11}**B NMR** (128 MHz, $CDCl_3$) δ 25.9. **HRMS (ESI)** *m/z* Calcd for $C_{20}H_{34}BFNaO_6Si$ $[M+Na]^+$ 451.2094, Found: 451.2097.

(2,6-dimethoxy-4-(4,4,6,6-tetramethyl-1,3,2-dioxaborinan-2-yl)phenyl) trimethoxysilane (89)

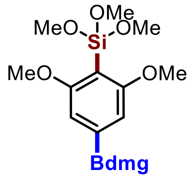

(2,6-dimethoxyphenyl)trimethoxysilane **89a** (0.2 mmol, 51.7 mg), B_2dmg_2 (84.6 mg, 0.3 mmol, 1.5 equiv), $[Ir(cod)OMe]_2$ (2.0 mg, 0.003 mmol), Me_4phen (1.5 mg, 0.006 mmol), and cyclohexane (1.0 mL) were added to a 15 mL glass vial under air atmosphere. The glass vial was capped with a teflon pressure cap and placed into an aluminum block pre-heated to 100 ºC for 30 minutes. After completion, cyclohexane was removed under reduced pressure and chromatographic separation with silica gel (20% ethyl acetate in hexane as eluent) gave the borylated product **89** (63.7 mg, 80%) as white solid. The *para:meta* ratio of products was reported from analysis of ^{1}H NMR.

^{1}**H NMR** (400 MHz, $CDCl_3$) δ 6.99 (s, 2H), 3.87 (s, 6H), 3.59 (s, 9H), 1.91 (s, 2H), 1.43 (s, 12H). ^{13}**C NMR** (101 MHz, $CDCl_3$) δ 165.6, 108.7, 107.6, 71.2, 56.0, 50.8, 49.1, 31.9. ^{11}**B NMR** (128 MHz, $CDCl_3$) δ 25.7. **HRMS (ESI)** *m/z* Calcd for $C_{18}H_{31}BNaO_7Si$ $[M+Na]^+$ 421.1824, Found: 421.1831.

Trimethoxy(2-methoxy-6-methyl-4-(4,4,6,6-tetramethyl-1,3,2-dioxaborinan-2-yl)phenyl)silane (90)

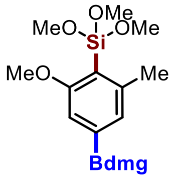

Trimethoxy(2-methoxy-6-methylphenyl)silane **90a** (0.2 mmol, 48.5 mg), B_2dmg_2 (84.6 mg, 0.3 mmol, 1.5 equiv), $[Ir(cod)OMe]_2$ (2.0 mg, 0.003 mmol), Me_4phen (1.5 mg, 0.006 mmol), and cyclohexane (1.0 mL) were added to a 15 mL glass vial under air atmosphere. The glass vial was capped with a teflon pressure cap and placed into an aluminum block pre-heated to 100 ºC for 30 minutes. After completion, cyclohexane was removed under reduced pressure and chromatographic separation with silica gel (20% ethyl acetate in hexane as eluent) gave the borylated product **90** (52.8 mg, 69%) as white solid. The *para:meta* ratio of products was reported from analysis of ^{1}H NMR.

^{1}**H NMR** (400 MHz, $CDCl_3$) δ 7.28 (s, 1H), 7.15 (s, 1H), 3.89 (s, 3H), 3.59 (s, 9H), 2.51 (s, 3H), 1.91 (s, 2H), 1.43 (s, 12H). ^{13}**C NMR** (101 MHz, $CDCl_3$) δ 165.0, 146.3, 129.3, 119.2, 112.1, 71.0, 55.8, 50.6, 49.1, 31.9, 23.2. ^{11}**B NMR** (128 MHz, $CDCl_3$) δ 25.9. **HRMS (ESI)** *m/z* Calcd for $C_{18}H_{31}BNaO_6Si$ $[M+Na]^+$ 405.1875, Found: 405.1877.

(2,6-diethyl-4-(4,4,6,6-tetramethyl-1,3,2-dioxaborinan-2-yl)phenyl) trimethoxy silane (91)

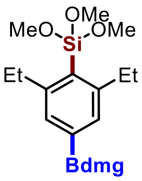

(2,6-diethylphenyl)trimethoxysilane **91a** (0.2 mmol, 50.0 mg), B_2dmg_2 (84.6 mg, 0.3 mmol, 1.5 equiv), $[Ir(cod)OMe]_2$ (2.0 mg, 0.003 mmol), Me_4phen (1.5 mg, 0.006 mmol), and cyclohexane (1.0 mL) were added to a 15 mL glass vial under air atmosphere. The glass vial was capped with a teflon pressure cap and placed into an aluminum block pre-heated to 100 ºC for 30 minutes. After completion, cyclohexane was removed under reduced pressure and chromatographic separation with silica gel (20% ethyl acetate in hexane as eluent) gave the borylated product **91** (68.6 mg, 87%) as white solid. The *para:meta* ratio of products was reported from analysis of ^{1}H NMR.

^{1}H NMR (400 MHz, $CDCl_3$) δ 7.53 (s, 2H), 3.60 (s, 9H), 2.92 (q, *J* = 7.4 Hz, 4H), 1.92 (s, 12H), 1.25 (t, *J* = 7.5 Hz, 6H). **^{13}C NMR** (101 MHz, $CDCl_3$) δ 151.7, 132.2, 127.7, 70.9, 50.3, 49.2, 31.9, 29.8, 17.7. **^{11}B NMR** (128 MHz, $CDCl_3$) δ 26.2. **HRMS (ESI)** *m/z* Calcd for $C_{20}H_{35}BNaO_5Si$ $[M+Na]^+$ 417.2239, Found: 417.2245.

(2,6-dimethyl-4-(4,4,6,6-tetramethyl-1,3,2-dioxaborinan-2-yl)phenyl) triethoxy silane (92)

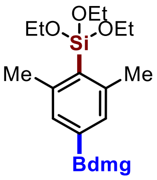

(2,6-diethylphenyl)trimethoxysilane **92a** (0.2 mmol, 53.7 mg), B_2dmg_2 (84.6 mg, 0.3 mmol, 1.5 equiv), $[Ir(cod)OMe]_2$ (2.0 mg, 0.003 mmol), Me_4phen (1.5 mg, 0.006 mmol), and cyclohexane (1.0 mL) were added to a 15 mL glass vial under air atmosphere. The glass vial was capped with a teflon pressure cap and placed into an aluminum block pre-heated to 100 ºC for 30 minutes. After completion, cyclohexane was removed under reduced pressure and chromatographic separation with silica gel (20% ethyl acetate in hexane as eluent) gave the borylated product **92** (67.1 mg, 82%) as colorless liquid. The *para:meta* ratio of products was reported from analysis of ^{1}H NMR.

^{1}H NMR (400 MHz, $CDCl_3$) δ 7.45 (s, 2H), 3.83 (q, *J* = 7.0 Hz, 6H), 2.58 (s, 6H), 1.91 (s, 2H), 1.43 (s, 12H), 1.24 (t, *J* = 7.0 Hz, 9H). **^{13}C NMR** (101 MHz, $CDCl_3$) δ 144.8, 133.1, 130.6, 70.9, 58.2, 49.2, 31.9, 23.9, 18.3. **^{11}B NMR** (128 MHz, $CDCl_3$) δ 26.3. **HRMS (ESI)** *m/z* Calcd for $C_{21}H_{37}BNaO_5Si$ $[M+Na]^+$ 431.2396, Found: 431.2389.

(2-chloro-4-(4,4,6,6-tetramethyl-1,3,2-dioxaborinan-2-yl)benzyl)tris((2,6-dimethylheptan-4-yl)oxy)silane (93)

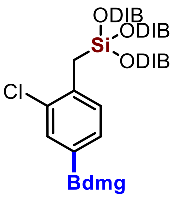

(2-chlorobenzyl)tris((2,6-dimethylheptan-4-yl)oxy)silane **93a** (0.2 mmol, 116.7 mg), B_2dmg_2 (84.6 mg, 0.3 mmol, 1.5 equiv), $[Ir(cod)OMe]_2$ (2.0 mg, 0.003 mmol), Me_4phen (1.5 mg, 0.006 mmol), and cyclohexane (1.0 mL) were added to a 15 mL glass vial under air atmosphere. The glass vial was capped with a teflon pressure cap and placed into an aluminum block pre-heated to 100 ºC for 1 hours. After completion, cyclohexane was removed under reduced pressure and chromatographic separation with

silica gel (20% ethyl acetate in hexane as eluent) gave the borylated product **93** (111.3 mg, 77%) as colorless liquid. The *para:meta* ratio of products was reported from analysis of ^{1}H NMR.

^{1}H NMR (400 MHz, $CDCl_3$) δ 7.64 (d, *J* = 1.2 Hz, 1H), 7.45 (dd, *J* = 7.6, 1.3 Hz, 1H), 7.22 – 7.15 (m, 1H), 3.99 – 3.90 (m, 3H), 2.28 (s, 2H), 1.81 (s, 2H), 1.57 (dp, *J* = 13.4, 6.7 Hz, 6H), 1.32 (s, 12H), 1.25 (dt, *J* = 13.5, 6.8 Hz, 6H), 1.14 (ddd, *J* = 13.5, 7.6, 5.8 Hz, 6H), 0.76 (dd, *J* = 8.3, 6.6 Hz, 36H). **^{13}C NMR** (101 MHz, $CDCl_3$) δ 139.3, 134.8, 133.5, 131.8, 130.0, 70.9, 69.8, 49.2, 46.8, 31.9, 24.5, 23.1, 23.0, 20.3. **^{11}B NMR** (128 MHz, $CDCl_3$) δ 25.9. **HRMS (ESI)** *m/z* Calcd for $C_{41}H_{76}BClNaO_5Si$ $[M+Na]^+$ 745.5136, Found: 745.5158.

(2-bromo-4-(4,4,6,6-tetramethyl-1,3,2-dioxaborinan-2-yl)benzyl)tris((2,6-dimethylheptan-4-yl)oxy)silane (94)

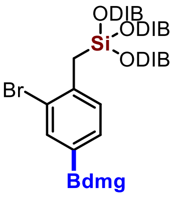

(2-bromobenzyl)tris((2,6-dimethylheptan-4-yl)oxy)silane **94a** (0.2 mmol, 125.6 mg), B_2dmg_2 (84.6 mg, 0.3 mmol, 1.5 equiv), $[Ir(cod)OMe]_2$ (2.0 mg, 0.003 mmol), Me_4phen (1.5 mg, 0.006 mmol), and cyclohexane (1.0 mL) were added to a 15 mL glass vial under air atmosphere. The glass vial was capped with a teflon pressure cap and placed into an aluminum block pre-heated to 100 ºC for 1 hours. After completion, cyclohexane was removed under reduced pressure and chromatographic separation with silica gel (20% ethyl acetate in hexane as eluent) gave the borylated product **94** (113.6 mg, 74%) as colorless liquid. The *para:meta* ratio of products was reported from analysis of ^{1}H NMR.

^{1}H NMR (400 MHz, $CDCl_3$) δ 7.92 (s, 1H), 7.58 (dd, *J* = 7.6, 1.3 Hz, 1H), 7.29 (d, *J* = 7.6 Hz, 1H), 4.12 – 3.91 (m, 3H), 2.41 (s, 2H), 1.89 (s, 2H), 1.66 (dp, *J* = 13.3, 6.7 Hz, 6H), 1.41 (s, 12H), 1.38 – 1.30 (m, 6H), 1.23 (ddd, *J* = 13.5, 7.6, 5.8 Hz, 6H), 0.85 (dd, *J* = 8.1, 6.6 Hz, 36H). **^{13}C NMR** (101 MHz, $CDCl_3$) δ 141.1, 138.2, 132.5, 129.9, 125.0, 70.9, 69.9, 49.2, 46.8, 32.0, 24.5, 23.3, 23.1, 23.0. **^{11}B NMR** (128 MHz, $CDCl_3$) δ 25.8. **HRMS (ESI)** *m/z* Calcd for $C_{41}H_{76}BBrNaO_5Si$ $[M+Na]^+$ 789.4631, Found: 789.4620.

5-(4,4,6,6-tetramethyl-1,3,2-dioxaborinan-2-yl)-2-((tris((2,6-dimethylheptan-4-yl)oxy)silyl)methyl)benzonitrile (95)

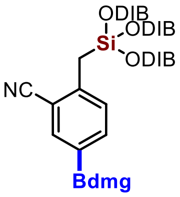

2-((tris((2,6-dimethylheptan-4-yl)oxy)silyl)methyl)benzonitrile **95a** (0.2 mmol, 114.8 mg), B_2dmg_2 (84.6 mg, 0.3 mmol, 1.5 equiv), $[Ir(cod)OMe]_2$ (2.0 mg, 0.003 mmol), Me_4phen (1.5 mg, 0.006 mmol), and cyclohexane (1.0 mL) were added to a 15 mL glass vial under air atmosphere. The glass vial was capped with a teflon pressure cap and placed into an aluminum block pre-heated to 100 ºC for 1 hours. After completion, cyclohexane was removed under reduced pressure and chromatographic

separation with silica gel (20% ethyl acetate in hexane as eluent) gave the borylated product **95** (85.7 mg, 60%) as colorless liquid. The *para:meta* ratio of products was reported from analysis of ^{1}H NMR.

^{1}H NMR (400 MHz, $CDCl_3$) δ 7.92 (d, *J* = 1.3 Hz, 1H), 7.74 (dd, *J* = 7.8, 1.4 Hz, 1H), 7.26 (d, *J* = 7.9 Hz, 1H), 4.03 – 3.91 (m, 3H), 2.35 (s, 2H), 1.83 (s, 2H), 1.54 (dq, *J* = 13.3, 6.7 Hz, 6H), 1.33 (s, 12H), 1.32 – 1.21 (m, 6H), 1.16 (ddd, *J* = 13.5, 7.6, 5.8 Hz, 6H), 0.77 (dd, *J* = 8.4, 6.5 Hz, 36H). **^{13}C NMR** (101 MHz, $CDCl_3$) δ 145.5, 138.6, 137.3, 128.9, 119.3, 112.2, 71.2, 70.1, 49.1, 46.7, 31.9, 24.5, 23.0, 22.9, 22.4. **^{11}B NMR** (128 MHz, $CDCl_3$) δ 25.9. **HRMS (ESI)** *m/z* Calcd for $C_{42}H_{76}BNNaO_5Si$ $[M+Na]^+$ 736.5478, Found: 736.5489.

Tris((2,6-dimethylheptan-4-yl)oxy)(4-(4,4,6,6-tetramethyl-1,3,2-dioxaborinan-2-yl)-2-(trifluoromethyl)benzyl)silane (96)

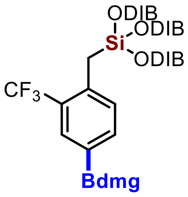

Tris((2,6-dimethylheptan-4-yl)oxy)(2-(trifluoromethyl)benzyl)silane **96a** (0.2 mmol, 123.4 mg), B_2dmg_2 (84.6 mg, 0.3 mmol, 1.5 equiv), $[Ir(cod)OMe]_2$ (2.0 mg, 0.003 mmol), Me_4phen (1.5 mg, 0.006 mmol), and cyclohexane (1.0 mL) were added to a 15 mL glass vial under air atmosphere. The glass vial was capped with a teflon pressure cap and placed into an aluminum block pre-heated to 100 ºC for 1 hours. After completion, cyclohexane was removed under reduced pressure and chromatographic separation with silica gel (20% ethyl acetate in hexane as eluent) gave the borylated product **96** (137.7 mg, 91%) as colorless liquid. The *para:meta* ratio of products was reported from analysis of ^{1}H NMR.

^{1}H NMR (400 MHz, $CDCl_3$) δ 8.01 (s, 1H), 7.81 (d, *J* = 7.7 Hz, 1H), 7.43 (d, *J* = 7.7 Hz, 1H), 4.03 (h, *J* = 6.3 Hz, 3H), 2.40 (s, 2H), 1.91 (s, 2H), 1.63 (dp, *J* = 13.6, 6.8 Hz, 6H), 1.42 (s, 12H), 1.33 (dt, *J* = 13.5, 6.8 Hz, 6H), 1.22 (ddd, *J* = 13.4, 7.6, 5.8 Hz, 6H), 0.85 (t, *J* = 7.0 Hz, 36H). **^{13}C NMR** (101 MHz, $CDCl_3$) δ 140.5, 136.7, 131.5 (q, *J* = 6.0 Hz), 127.44 (q, *J* = 35.4 Hz), 123.5 (q, J = 272.4 Hz), 126.7, 124.0, 71.0, 69.9, 49.2, 46.8, 32.0, 24.5, 23.0, 23.0, 19.9. **^{19}F NMR** (376 MHz, $CDCl_3$) δ -59.4. **^{11}B NMR** (128 MHz, $CDCl_3$) δ 25.8. **HRMS (ESI)** *m/z* Calcd for $C_{42}H_{76}BF_3NaO_5Si$ $[M+Na]^+$ 779.5339, Found: 779.5368.

Tris((2,6-dimethylheptan-4-yl)oxy)(4-(4,4,6,6-tetramethyl-1,3,2-dioxaborinan-2-yl)-2-(trifluoromethoxy)benzyl)silane (97)

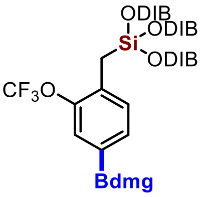

Tris((2,6-dimethylheptan-4-yl)oxy)(2-(trifluoromethoxy)benzyl)silane **97a** (0.2 mmol, 126.6 mg), B_2dmg_2 (84.6 mg, 0.3 mmol, 1.5 equiv), $[Ir(cod)OMe]_2$ (2.0 mg, 0.003 mmol), Me_4phen (1.5 mg, 0.006 mmol), and cyclohexane (1.0 mL) were added to a 15 mL glass vial under air atmosphere. The glass vial was capped with a teflon pressure cap and placed into an aluminum

block pre-heated to 100 ºC for 1 hours. After completion, cyclohexane was removed under reduced pressure and chromatographic separation with silica gel (20% ethyl acetate in hexane as eluent) gave the borylated product **97** (131.4 mg, 85%) as colorless liquid. The *para:meta* ratio of products was reported from analysis of ^{1}H NMR.

^{1}H NMR (400 MHz, $CDCl_3$) δ 7.62 – 7.55 (m, 2H), 7.34 (d, *J* = 7.8 Hz, 1H), 4.03 (p, *J* = 6.4 Hz, 3H), 2.24 (s, 2H), 1.90 (s, 2H), 1.66 (dp, *J* = 12.9, 6.4 Hz, 6H), 1.42 (s, 12H), 1.39 – 1.30 (m, 6H), 1.29 – 1.19 (m, 6H), 0.86 (dd, *J* = 9.0, 6.5 Hz, 36H). **^{13}C NMR** (101 MHz, $CDCl_3$) δ 147.2, 134.2, 131.9, 130.6, 125.9, 121.0 (q, *J* = 256.1 Hz), 71.0, 69.9, 49.2, 46.8, 31.9, 24.5, 23.0, 22.9, 16.3. **^{19}F NMR** (376 MHz, $CDCl_3$) δ -56.7. **^{11}B NMR** (128 MHz, Chloroform-*d*) δ 25.9. **HRMS (ESI)** *m/z* Calcd for $C_{42}H_{76}BF_3NaO_6Si$ $[M+Na]^+$ 795.5349, Found: 795.5370.

(2,3-difluoro-4-(4,4,6,6-tetramethyl-1,3,2-dioxaborinan-2-yl)benzyl)tris((2,6-dimethylheptan-4-yl)oxy)silane (98)

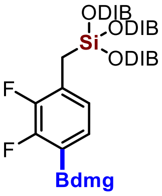

(2,3-difluorobenzyl)tris((2,6-dimethylheptan-4-yl)oxy)silane **98a** (0.2 mmol, 117.0 mg), B_2dmg_2 (84.6 mg, 0.3 mmol, 1.5 equiv), $[Ir(cod)OMe]_2$ (2.0 mg, 0.003 mmol), Me_4phen (1.5 mg, 0.006 mmol), and cyclohexane (1.0 mL) were added to a 15 mL glass vial under air atmosphere. The glass vial was capped with a teflon pressure cap and placed into an aluminum block pre-heated to 100 ºC for 1 hours. After completion, cyclohexane was removed under reduced pressure and chromatographic separation with silica gel (20% ethyl acetate in hexane as eluent) gave the borylated product **98** (137.5 mg, 90%) as colorless liquid. The *para:meta* ratio of products was reported from analysis of ^{1}H NMR.

^{1}H NMR (400 MHz, $CDCl_3$) δ 7.17 (td, *J* = 5.8, 2.8 Hz, 1H), 6.87 (ddd, *J* = 7.7, 6.2, 1.3 Hz, 1H), 4.00 – 3.89 (m, 3H), 2.09 (s, 2H), 1.83 (s, 2H), 1.57 (dp, *J* = 13.4, 6.7 Hz, 6H), 1.33 (s, 12H), 1.30 – 1.22 (m, 6H), 1.15 (ddd, *J* = 13.5, 7.6, 5.8 Hz, 6H), 0.77 (dd, *J* = 8.0, 6.5 Hz, 36H). **^{13}C NMR** (101 MHz, $CDCl_3$) δ 154.8 (dd, *J* = 250.6, 12.7 Hz), 148.5 (dd, *J* = 245.6, 14.7 Hz), 130.5 (d, *J* = 13.4 Hz), 129.0 (dd, *J* = 7.8, 4.5 Hz), 126.3 (d, *J* = 3.0 Hz), 125.0 (d, *J* = 5.8 Hz), 71.1, 69.8, 49.0, 46.6, 31.7, 24.4, 22.9, 22.8, 15.1. **^{19}F NMR** (376 MHz, $CDCl_3$) δ -132.3 (d, *J* = 20.9 Hz), -144.1 (d, *J* = 20.8 Hz). **^{11}B NMR** (128 MHz, $CDCl_3$) δ 25.8. **HRMS (ESI)** *m/z* Calcd for $C_{41}H_{75}BF_2NaO_5Si$ $[M+Na]^+$ 747.5337, Found: 747.5358.

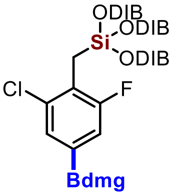

(2-chloro-6-fluoro-4-(4,4,6,6-tetramethyl-1,3,2-dioxaborinan-2-yl)benzyl)tris((2,6-dimethylheptan-4-yl)oxy)silane (99)

(2-chloro-6-fluorobenzyl)tris((2,6-dimethylheptan-4-yl)oxy)silane **99a** (0.2 mmol, 117.0 mg), B_2dmg_2 (84.6 mg, 0.3 mmol, 1.5 equiv), $[Ir(cod)OMe]_2$ (2.0 mg, 0.003 mmol), Me_4phen (1.5 mg,

0.006 mmol), and cyclohexane (1.0 mL) were added to a 15 mL glass vial under air atmosphere. The glass vial was capped with a teflon pressure cap and placed into an aluminum block pre-heated to 100 ºC for 1 hours. After completion, cyclohexane was removed under reduced pressure and chromatographic separation with silica gel (20% ethyl acetate in hexane as eluent) gave the borylated product **99** (120.2 mg, 80%) as colorless liquid. The *para:meta* ratio of products was reported from analysis of ^{1}H NMR.

^{1}H NMR (400 MHz, $CDCl_3$) δ 7.54 (s, 1H), 7.30 (d, *J* = 9.7 Hz, 1H), 4.07 (p, *J* = 6.4 Hz, 3H), 2.36 (d, *J* = 2.7 Hz, 2H), 1.89 (s, 2H), 1.63 (dp, *J* = 13.3, 6.5 Hz, 6H), 1.40 (s, 12H), 1.39 – 1.28 (m, 6H), 1.21 (ddd, *J* = 13.5, 7.7, 5.8 Hz, 6H), 0.85 (t, *J* = 7.3 Hz, 36H). **^{13}C NMR** (101 MHz, $CDCl_3$) δ 160.7 (d, *J* = 246.0 Hz), 134.1 (d, *J* = 5.6 Hz), 130.0 (d, *J* = 2.8 Hz), 128.5 (d, *J* = 19.9 Hz), 118.0 (d, *J* = 20.7 Hz), 71.1, 69.9, 49.1, 46.7, 31.9, 24.5, 23.1, 23.0, 14.2 (d, *J* = 2.5 Hz). **^{11}B NMR** (128 MHz, $CDCl_3$) δ 25.6. **^{19}F NMR** (376 MHz, $CDCl_3$) δ -112.3. **HRMS (ESI)** *m/z* Calcd for $C_{41}H_{75}BClFNaO_5Si$ $[M+Na]^+$ 763.5042, Found: 763.5066.

5. Switchable site-selective C-H borylation

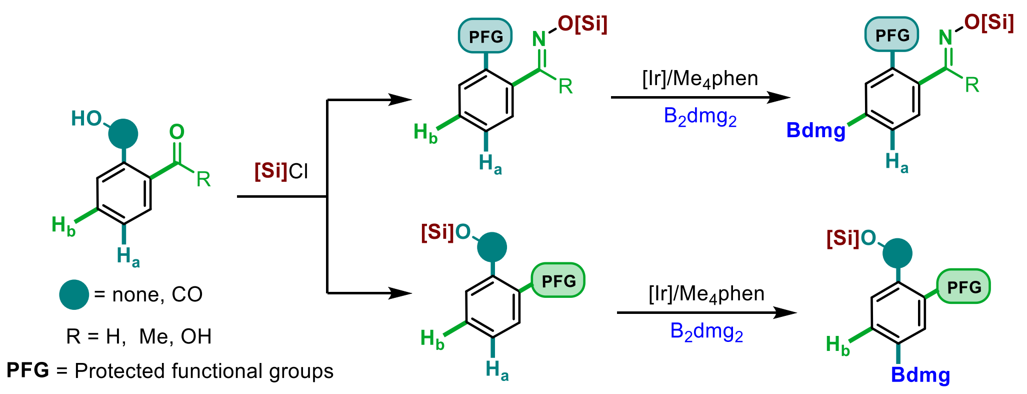

Tris(2,6-dimethylheptan-4-yl) (2-(2-methyl-1,3-dioxolan-2-yl)-4-(4,4,6,6-tetramethyl-1,3,2-dioxaborinan-2-yl)phenyl) silicate (102)

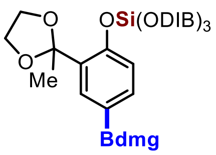

Tris(2,6-dimethylheptan-4-yl) (2-(2-methyl-1,3-dioxolan-2-yl)phenyl) silicate **101** (0.2 mmol, 127.4 mg), B_2dmg_2 (84.6 mg, 0.3 mmol, 1.5 equiv), $[Ir(cod)OMe]_2$ (2.0 mg, 0.003 mmol), Me_4phen (1.5 mg, 0.006 mmol), and cyclohexane (1.0 mL) were added to a 15 mL glass vial under air atmosphere. The glass vial was capped with a teflon pressure cap and placed into an aluminum block pre-heated to 120 ºC for 1 h. After completion, cyclohexane was removed under reduced pressure and chromatographic separation with silica gel (15% ethyl acetate in hexane as eluent) gave the borylated product **102** (124.3 mg, 80%) as colorless liquid. The *para:meta* ratio of products was reported from analysis of ^{1}H NMR.

^{1}**H NMR** (400 MHz, $CDCl_3$) δ 7.92 (d, *J* = 1.7 Hz, 1H), 7.64 (dd, *J* = 8.1, 1.8 Hz, 1H), 7.11 (d, *J* = 8.1 Hz, 1H), 4.33 – 4.17 (m, 3H), 3.98 (t, *J* = 6.8 Hz, 2H), 3.79 (t, *J* = 6.8 Hz, 2H), 1.89 (s, 2H), 1.81 – 1.67 (m, 9H), 1.47 (dt, *J* = 13.9, 6.9 Hz, 6H), 1.42 (s, 12H), 1.34 – 1.23 (m, 6H), 0.87 (dd, *J* = 11.2, 6.6 Hz, 36H). ^{13}**C NMR** (101 MHz, $CDCl_3$) δ 154.3, 135.1, 132.7, 130.5, 120.0, 108.7, 70.8, 70.7, 64.2, 49.2, 46.5, 32.0, 26.0, 24.5, 23.1, 23.0. ^{11}**B NMR** (128 MHz, $CDCl_3$) δ 20.9. **HRMS (ESI)** *m/z* Calcd for $C_{44}H_{81}BNaO_8Si$ $[M+Na]^+$ 799.5686, Found: 799.5701.

1-(2-methoxy-4-(4,4,6,6-tetramethyl-1,3,2-dioxaborinan-2-yl)phenyl)ethan-1-one O-(tris((2,6-dimethylheptan-4-yl)oxy)silyl) oxime (104)

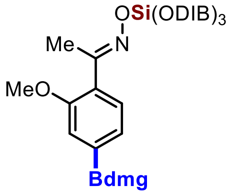

1-(2-methoxyphenyl)ethan-1-one O-(tris((2,6-dimethylheptan-4-yl)oxy)silyl) oxime **103** (0.2 mmol, 124.4 mg), B_2dmg_2 (84.6 mg, 0.3 mmol, 1.5 equiv), $[Ir(cod)OMe]_2$ (2.0 mg, 0.003 mmol), Me_4phen (1.5 mg, 0.006 mmol), and cyclohexane (1.0 mL) were added to a 15 mL glass vial under air atmosphere. The glass vial was capped with a teflon pressure cap and placed into an aluminum block pre-heated to 100 ºC for 1 h. After completion, cyclohexane was removed under reduced pressure and chromatographic separation with silica gel (15% ethyl acetate in hexane as eluent) gave the borylated product **104** (128.0 mg, 84%) as colorless liquid. The *para:meta* ratio of products was reported from analysis of ^{1}H NMR.

^{1}**H NMR** (400 MHz, $CDCl_3$) δ 7.31 (d, *J* = 7.5 Hz, 1H), 7.25 (d, *J* = 7.1 Hz, 2H), 4.12 – 4.01 (m, 3H), 3.77 (s, 3H), 2.12 (s, 3H), 1.84 (s, 2H), 1.81 – 1.67 (m, 6H), 1.46 – 1.35 (m, 6H), 1.35 (s, 12H), 1.20 – 1.09 (m, 6H), 0.78 (dd, *J* = 8.4, 6.5 Hz, 36H). ^{13}**C NMR** (101 MHz, $CDCl_3$) δ 161.5, 156.9, 129.0, 128.9, 125.9, 115.6, 71.0, 70.3, 55.2, 49.1, 46.8, 31.8, 24.3, 23.2, 22.6, 16.1. ^{11}**B NMR** (128 MHz, $CDCl_3$) δ 26.0. **HRMS (ESI)** *m/z* Calcd for $C_{43}H_{80}BNNaO_7Si$ $[M+Na]^+$ 784.5689, Found: 784.5693.

2-(1,3-dioxolan-2-yl)-4-(4,4,6,6-tetramethyl-1,3,2-dioxaborinan-2-yl)phenyl tris(2,6-dimethylheptan-4-yl) silicate (107)

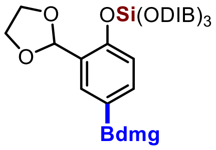

2-(1,3-dioxolan-2-yl)phenyl tris(2,6-dimethylheptan-4-yl) silicate **106** (0.2 mmol, 124.6 mg), B_2dmg_2 (84.6 mg, 0.3 mmol, 1.5 equiv), $[Ir(cod)OMe]_2$ (2.0 mg, 0.003 mmol), Me_4phen (1.5 mg, 0.006 mmol), and cyclohexane (1.0 mL) were added to a 15 mL glass vial under air atmosphere. The glass vial was capped with a teflon pressure cap and placed into an aluminum block pre-heated to 120 ºC for 1 h. After completion, cyclohexane was removed under reduced pressure and chromatographic separation

with silica gel (15% ethyl acetate in hexane as eluent) gave the borylated product **107** (129.7 mg, 85%) as colorless liquid. The *para:meta* ratio of products was reported from analysis of ^{1}H NMR.

^{1}H NMR (400 MHz, $CDCl_3$) δ 7.95 (d, *J* = 1.7 Hz, 1H), 7.68 (dd, *J* = 8.2, 1.8 Hz, 1H), 7.13 (d, *J* = 8.2 Hz, 1H), 6.19 (s, 1H), 4.24 – 4.10 (m, 5H), 4.00 – 3.94 (m, 2H), 1.88 (s, 2H), 1.81 – 1.66 (m, 6H), 1.51 – 1.39 (m, 6H), 1.40 (s, 12H), 1.27 (ddd, *J* = 13.5, 7.6, 5.4 Hz, 6H), 0.86 (dd, *J* = 10.4, 6.5 Hz, 36H). **^{13}C NMR** (101 MHz, $CDCl_3$) δ 154.9, 135.9, 132.7, 126.2, 118.4, 99.6, 71.0, 70.7, 65.2, 49.2, 46.6, 32.0, 24.5, 23.1, 22.8. **^{11}B NMR** (128 MHz, $CDCl_3$) δ 20.7. **HRMS (ESI)** *m/z* Calcd for $C_{43}H_{79}BNaO_8Si$ $[M+Na]^+$ 785.5529, Found: 785.5520.

4-(4,4,6,6-tetramethyl-1,3,2-dioxaborinan-2-yl)-2-((triisopropylsilyl)oxy)benzaldehyde O-(tris((2,6-dimethylheptan-4-yl)oxy)silyl) oxime (109)

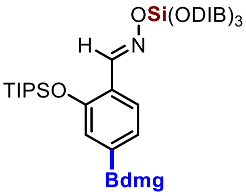

2-((triisopropylsilyl)oxy)benzaldehyde O-(tris((2,6-dimethylheptan-4-yl)oxy)silyl) oxime **108** (0.2 mmol, 150.1 mg), B_2dmg_2 (84.6 mg, 0.3 mmol, 1.5 equiv), $[Ir(cod)OMe]_2$ (2.0 mg, 0.003 mmol), Me_4phen (1.5 mg, 0.006 mmol), and cyclohexane (1.0 mL) were added to a 15 mL glass vial under air atmosphere. The glass vial was capped with a teflon pressure cap and placed into an aluminum block pre-heated to 100 °C for 1 h. After completion, cyclohexane was removed under reduced pressure and chromatographic separation with silica gel (15% ethyl acetate in hexane as eluent) gave the borylated product **109** (135.3 mg, 76%) as colorless liquid. The *para:meta* ratio of products was reported from analysis of ^{1}H NMR.

^{1}H NMR (400 MHz, $CDCl_3$) δ 8.65 (s, 1H), 7.81 (d, *J* = 7.8 Hz, 1H), 7.34 (d, *J* = 7.0 Hz, 2H), 4.25 – 4.08 (m, 3H), 1.91 (s, 2H), 1.92 – 1.77 (m, 6H), 1.57 – 1.45 (m, 6H), 1.42 (s, 12H), 1.33 – 1.19 (m, 9H), 1.13 (d, *J* = 7.4 Hz, 18H), 0.88 (dd, *J* = 8.5, 6.6 Hz, 36 H). **^{13}C NMR** (101 MHz, $CDCl_3$) δ 153.9, 150.8, 126.0, 125.7, 124.5, 124.3, 71.0, 70.6, 49.0, 47.0, 31.9, 24.4, 23.3, 22.7, 18.2, 13.0. **^{11}B NMR** (128 MHz, $CDCl_3$) δ 26.6. **HRMS (ESI)** *m/z* Calcd for $C_{50}H_{96}BNNaO_7Si_2$ $[M+Na]^+$ 912.6711, Found: 912.6745.

2-(dimethylcarbamoyl)-4-(4,4,6,6-tetramethyl-1,3,2-dioxaborinan-2-yl)phenyl tris(2,6-dimethylheptan-4-yl) silicate (112)

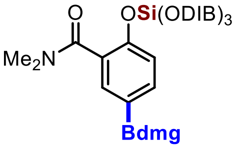

2-(dimethylcarbamoyl)phenyl tris(2,6-dimethylheptan-4-yl) silicate **111** (0.2 mmol, 124.4 mg), B_2dmg_2 (84.6 mg, 0.3 mmol, 1.5 equiv), $[Ir(cod)OMe]_2$ (2.0 mg, 0.003 mmol), Me_4phen (1.5 mg, 0.006 mmol), and cyclohexane (1.0 mL) were added to a 15 mL glass vial under air atmosphere. The glass vial was capped with a teflon pressure cap and placed into an aluminum block pre-heated to

120 °C for 1 h. After completion, cyclohexane was removed under reduced pressure and chromatographic separation with silica gel (15% ethyl acetate in hexane as eluent) gave the borylated product **112** (124.4 mg, 81%) as colorless liquid. The *para:meta* ratio of products was reported from analysis of ^{1}H NMR.

^{1}H NMR (400 MHz, $CDCl_3$) δ 7.71 (d, J = 1.7 Hz, 1H), 7.66 (dd, J = 8.2, 1.7 Hz, 1H), 7.10 (d, J = 8.3 Hz, 1H), 4.23 – 4.05 (m, 3H), 3.06 (s, 3H), 2.85 (s, 3H), 1.87 (s, 2H), 1.77 – 1.62 (m, 6H), 1.45 – 1.36 (m, 18H), 1.31 – 1.22 (m, 6H), 0.85 (dd, J = 10.7, 6.6 Hz, 36H). **^{13}C NMR** (101 MHz, $CDCl_3$) δ 170.0, 152.0, 135.6, 134.4, 127.1, 118.5, 71.1, 49.1, 46.4, 38.4, 35.0, 31.9, 24.5, 23.1, 22.8. **^{11}B NMR** (128 MHz, $CDCl_3$) δ 26.8. **HRMS (ESI)** *m/z* Calcd for $C_{43}H_{80}BNNaO_7Si$ $[M+Na]^+$ 784.5689, Found: 784.5705.

2-methoxy-4-(4,4,6,6-tetramethyl-1,3,2-dioxaborinan-2-yl)benzoic (tris(2,6-dimethylheptan-4-yl) silicic) anhydride (114)

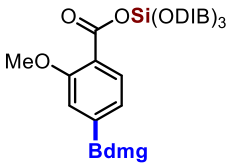

2-methoxybenzoic (tris(2,6-dimethylheptan-4-yl) silicic) anhydride **113** (0.2 mmol, 121.8 mg), B_2dmg_2 (84.6 mg, 0.3 mmol, 1.5 equiv), $[Ir(cod)OMe]_2$ (2.0 mg, 0.003 mmol), Me_4phen (1.5 mg, 0.006 mmol), and cyclohexane (1.0 mL) were added to a 15 mL glass vial under air atmosphere. The glass vial was capped with a teflon pressure cap and placed into an aluminum block pre-heated to 100 °C for 1 h. After completion, cyclohexane was removed under reduced pressure and chromatographic separation with silica gel (15% ethyl acetate in hexane as eluent) gave the borylated product **114** (119.8 mg, 80%) as colorless liquid. The *para:meta* ratio of products was reported from analysis of ^{1}H NMR.

^{1}H NMR (400 MHz, $CDCl_3$) δ 7.76 (d, J = 7.6 Hz, 1H), 7.42 – 7.40 (m, 6H), 4.29 – 4.20 (m, 3H), 3.92 (s, 3H), 1.93 (s, 2H), 1.86 – 1.71 (m, 6H), 1.50 (ddd, J = 13.6, 7.5, 6.0 Hz, 6H), 1.44 (s, 12H), 1.27 (ddd, J = 13.4, 8.0, 5.2 Hz, 6H), 0.88 (t, J = 6.4 Hz, 36H). **^{13}C NMR** (101 MHz, $CDCl_3$) δ 164.2, 158.9, 131.0, 125.3, 122.8, 116.8, 71.3, 71.0, 55.9, 49.1, 46.8, 31.9, 24.5, 23.3, 22.8. **^{11}B NMR** (128 MHz, $CDCl_3$) δ 25.4. **HRMS (ESI)** *m/z* Calcd for $C_{42}H_{77}BNaO_8Si$ $[M+Na]^+$ 771.5373, Found: 771.5352.

2-((methoxyimino)methyl)-4-(4,4,6,6-tetramethyl-1,3,2-dioxaborinan-2-yl)benzoic (tris(2,6-dimethylheptan-4-yl) silicic) anhydride (117)

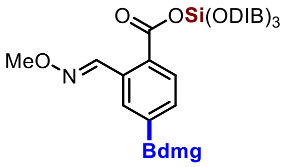

2-((methoxyimino)methyl)benzoic (tris(2,6-dimethylheptan-4-yl) silicic) anhydride **116** (0.2 mmol, 127.2 mg), B_2dmg_2 (84.6 mg, 0.3 mmol, 1.5 equiv), $[Ir(cod)OMe]_2$ (2.0 mg, 0.003 mmol), Me_4phen (1.5 mg, 0.006 mmol), and cyclohexane (1.0 mL) were added to a 15 mL glass vial under air atmosphere. The glass vial was capped with a teflon pressure cap and placed into an

aluminum block pre-heated to 100 ºC for 1 h. After completion, cyclohexane was removed under reduced pressure and chromatographic separation with silica gel (15% ethyl acetate in hexane as eluent) gave the borylated product **117** (121.1 mg, 78%) as colorless liquid. The *para:meta* ratio of products was reported from analysis of ^{1}H NMR.

^{1}H NMR (400 MHz, $CDCl_3$) δ 9.00 (s, 1H), 8.30 (s, 1H), 7.95 (d, *J* = 7.8 Hz, 1H), 7.86 (dd, *J* = 7.8, 1.3 Hz, 1H), 4.28 – 4.19 (m, 3H), 4.01 (s, 3H), 1.93 (s, 2H), 1.83 – 1.72 (m, 6H), 1.49 (ddd, *J* = 13.9, 7.7, 6.1 Hz, 6H), 1.44 (s, 12H), 1.28 (ddd, *J* = 13.3, 7.8, 5.1 Hz, 6H), 0.88 (dd, *J* = 6.7, 5.2 Hz, 36H). **^{13}C NMR** (101 MHz, $CDCl_3$) δ 164.9, 149.2, 134.6, 133.1, 132.8, 131.1, 130.1, 71.4, 71.3, 62.0, 49.2, 46.8, 31.9, 24.5, 23.3, 22.7. **^{11}B NMR** (128 MHz, $CDCl_3$) δ 26.0. **HRMS (ESI)** *m/z* Calcd for $C_{43}H_{78}BNNaO_8Si$ $[M+Na]^+$ 798.5482, Found: 798.5501.

Methyl-2-(4,4-bis((2,6-dimethylheptan-4-yl)oxy)-6-isobutyl-8-methyl-3,5-dioxa-2-aza-4-silanon-1-en-1-yl)-5-(4,4,6,6-tetramethyl-1,3,2-dioxaborinan-2-yl)benzoate (119)

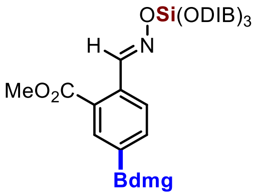

Methyl (E)-2-(4,4-bis((2,6-dimethylheptan-4-yl)oxy)-6-isobutyl-8-methyl-3,5-dioxa-2-aza-4-silanon-1-en-1-yl)benzoate **118** (0.2 mmol, 127.2 mg), B_2dmg_2 (84.6 mg, 0.3 mmol, 1.5 equiv), $[Ir(cod)OMe]_2$ (2.0 mg, 0.003 mmol), Me_4phen (1.5 mg, 0.006 mmol), and cyclohexane (1.0 mL) were added to a 15 mL glass vial under air atmosphere. The glass vial was capped with a teflon pressure cap and placed into an aluminum block pre-heated to 100 ºC for 1 h. After completion, cyclohexane was removed under reduced pressure and chromatographic separation with silica gel (15% ethyl acetate in hexane as eluent) gave the borylated product **119** (128.8 mg, 83%) as colorless liquid. The *para:meta* ratio of products was reported from analysis of ^{1}H NMR.

^{1}H NMR (400 MHz, $CDCl_3$) δ 9.00 (s, 1H), 8.35 (s, 1H), 7.98 (d, *J* = 7.9 Hz, 1H), 7.94 – 7.84 (m, 1H), 4.22 – 4.11 (m, 3H), 3.92 (s, 3H), 1.93 (s, 2H), 1.90 – 1.75 (m, 6H), 1.50 (ddd, *J* = 13.5, 7.6, 5.9 Hz, 6H), 1.44 (s, 12H), 1.25 (ddd, *J* = 13.4, 8.1, 5.1 Hz, 6H), 0.87 (dd, *J* = 8.7, 6.6 Hz, 36H). **^{13}C NMR** (101 MHz, $CDCl_3$) δ 167.8, 153.8, 137.2, 136.2, 134.4, 128.7, 126.3, 71.4, 70.7, 52.3, 49.2, 46.9, 31.9, 24.4, 23.3, 22.7. **^{11}B NMR** (128 MHz, $CDCl_3$) δ 26.8. **HRMS (ESI)** *m/z* Calcd for $C_{43}H_{78}BNNaO_8Si$ $[M+Na]^+$ 798.5482, Found: 798.5493.

6. Late-stage C−H borylation of important molecules

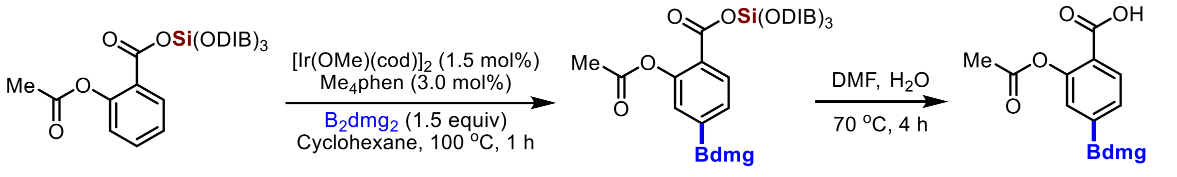

120a **120aa**, 80% **120**, 75%

2-acetoxybenzoic (tris(2,6-dimethylheptan-4-yl) silicic) anhydride **120a** (0.2 mmol, 127.4 mg), B_2dmg_2 (84.6 mg, 0.3 mmol, 1.5 equiv), $[Ir(cod)OMe]_2$ (2.0 mg, 0.003 mmol), Me_4phen (1.5 mg, 0.006 mmol), and cyclohexane (1.0 mL) were added to a 15 mL glass vial under air atmosphere. The glass vial was capped with a teflon pressure cap and placed into an aluminum block pre-heated to 100 ºC for 1 h. After completion, cyclohexane was removed under reduced pressure and chromatographic separation with silica gel (15% ethyl acetate in hexane as eluent) gave the borylated product **120aa** (124.3 mg, 80%) as colorless liquid. The *para:meta* ratio of products was reported from analysis of ^1H NMR.

A solution of the 2-acetoxy-4-(4,4,6,6-tetramethyl-1,3,2-dioxaborinan-2-yl)benzoic (tris(2,6-dimethylheptan-4-yl) silicic) anhydride **120aa** (77.7 mg, 0.1 mmol) in DMF–H_2O (20:1) (1 mL) was heated to 70 ºC, and stirred for 4 h (until all starting material has been consumed). The reaction was cooled to rt, diluted with ether (5 mL), washed successively with sat. aq. NH_4Cl and brine, dried (Na_2SO_4), and concentrated under reduced pressure. The residue was purified by silica gel flash column chromatography as a white solid **120** (24.0 mg, 75%).[12]

2-acetoxy-4-(4,4,6,6-tetramethyl-1,3,2-dioxaborinan-2-yl)benzoic acid (120)

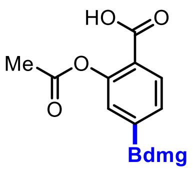

^1H NMR (400 MHz, $CDCl_3$) δ 8.05 (d, J = 7.7 Hz, 1H), 7.77 (dd, J = 7.7, 1.1 Hz, 1H), 7.55 (d, J = 1.1 Hz, 1H), 2.35 (s, 3H), 1.92 (s, 2H), 1.42 (s, 12H). **^{13}C NMR** (101 MHz, $CDCl_3$) δ 170.2, 170.2, 150.5, 131.5, 131.4, 128.9, 123.5, 71.5, 49.0, 31.8, 21.2. **^{11}B NMR** (128 MHz, $CDCl_3$) δ 25.6. **HRMS (ESI)** *m/z* Calcd for $C_{16}H_{21}BNNaO_6$ $[M+Na]^+$ 343.1323, Found: 343.1329.

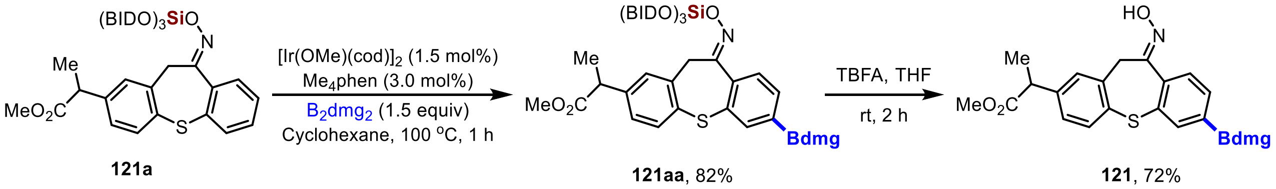

Methyl (E)-2-(10-(((tris((2,6-dimethylheptan-4-yl)oxy)silyl)oxy)imino)-10,11-dihydrodibenzo[b,f]thiepin-2-yl)propanoate **121a** (0.2 mmol, 156.8 mg), B_2dmg_2 (84.6 mg, 0.3 mmol, 1.5 equiv), $[Ir(cod)OMe]_2$ (2.0 mg, 0.003 mmol), Me_4phen (1.5 mg, 0.006 mmol), and cyclohexane (1.0 mL) were added to a 15 mL glass vial under air atmosphere. The glass vial was capped with a teflon pressure cap and placed into an aluminum block pre-heated to 100 ºC for 1 h. After completion, cyclohexane was removed under reduced pressure and chromatographic separation with silica gel (15% ethyl acetate in hexane as eluent) gave the borylated product **121aa** (151.6 mg, 82%) as colorless liquid. The *para:meta* ratio of products was reported from analysis of ^1H NMR.

Methyl (E)-2-(7-(4,4,6,6-tetramethyl-1,3,2-dioxaborinan-2-yl)-10-(((tris((2,6-dimethylheptan-4-yl)oxy)silyl)oxy)imino)-10,11-dihydrodibenzo[b,f]thiepin-2-yl)propanoate **121aa** (0.1 mmol, 92.4 mg) was dissolved in 1 mL THF and cooled to 0 ºC. The solution was treated with 200 µL TBAF (1M in THF, 2.00 equiv.) and warmed to room temperature. The mixture was stirred at room temperature for 2 h until the starting material was fully consumed monitored by TLC. The reaction was quenched with brine and extracted with diethyl ether (3 x 5 mL) and dried over $MgSO_4$. After filtration and removal of solvent, the crude was purified by silica gel chromatography to give a white solid **121** (33.6 mg, 72%).[13]

Methyl (E)-2-(10-(hydroxyimino)-7-(4,4,6,6-tetramethyl-1,3,2-dioxaborinan-2-yl)-10,11-dihydrodibenzo[b,f]thiepin-2-yl)propanoate (121)

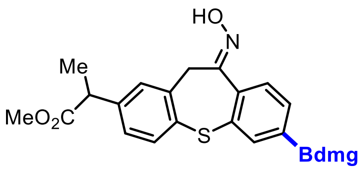

^{1}H NMR (400 MHz, $CDCl_3$) δ 9.24 (s, 1H), 7.93 (d, *J* = 1.1 Hz, 1H), 7.76 – 7.65 (m, 2H), 7.52 (d, *J* = 7.9 Hz, 1H), 7.40 (d, *J* = 2.0 Hz, 1H), 7.09 (dd, *J* = 8.0, 2.0 Hz, 1H), 4.30 (d, *J* = 1.9 Hz, 2H), 3.70 (q, *J* = 7.2 Hz, 1H), 3.61 (s, 3H), 1.92 (s, 2H), 1.47 (d, *J* = 7.2 Hz, 3H), 1.44 (s, 12H). **^{13}C NMR** (101 MHz, $CDCl_3$) δ 174.8, 154.8, 141.4, 140.1, 136.1, 135.7, 134.2, 134.1, 132.2, 131.5, 129.5, 127.7, 126.1, 71.3, 52.2, 49.1, 45.1, 33.7, 31.9, 18.6. **^{11}B NMR** (128 MHz, $CDCl_3$) δ 25.7. **HRMS (ESI)** *m/z* Calcd for $C_{25}H_{31}BNO_5S[M+Na]^+$ 468.2011, Found: 468.2023.

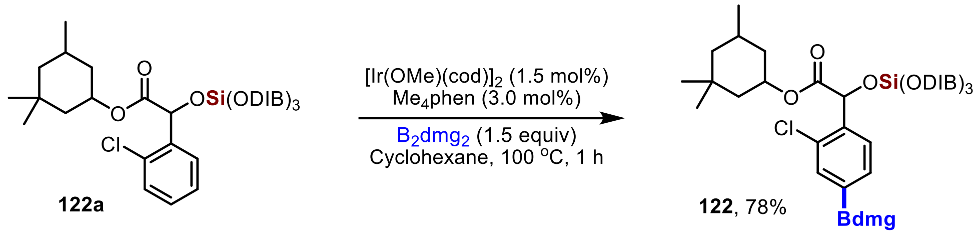

3,3,5-trimethylcyclohexyl 2-(2-chloro-4-(4,4,6,6-tetramethyl-1,3,2-dioxaborinan-2-yl)phenyl)-2-((tris((2,6-dimethylheptan-4-yl)oxy)silyl)oxy)acetate (122)

3,3,5-trimethylcyclohexyl 2-(2-chlorophenyl)-2-((tris((2,6-dimethylheptan-4-yl)oxy)silyl)oxy)acetate **122a** (0.2 mmol, 153.5 mg), B_2dmg_2 (84.6 mg, 0.3 mmol, 1.5 equiv), $[Ir(cod)OMe]_2$ (2.0 mg, 0.003 mmol), Me_4phen (1.5 mg, 0.006 mmol), and cyclohexane (1.0 mL) were added to a 15 mL glass vial under air atmosphere. The glass vial was capped with a teflon pressure cap and placed into an aluminum block pre-heated to 100 ºC for 1 h. After completion, cyclohexane was removed under reduced pressure and chromatographic separation with silica gel (15% ethyl acetate in hexane as eluent) gave the borylated product **122** (146.1 mg, 78%) as colorless liquid. The *para:meta* ratio of products was reported from analysis of ^{1}H NMR.

3,3,5-trimethylcyclohexyl 2-(2-chloro-4-(4,4,6,6-tetramethyl-1,3,2-dioxaborinan-2-yl)phenyl)-2-((tris((2,6-dimethylheptan-4-yl)oxy)silyl)oxy)acetate (122)

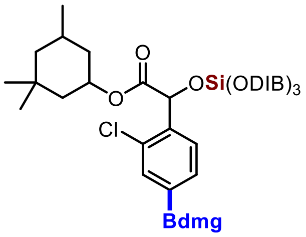

^{1}H NMR (400 MHz, $CDCl_3$) δ 7.76 (s, 1H), 7.67 (d, J = 7.7 Hz, 1H), 7.65 – 7.56 (m, 1H), 5.86 (s, 1H), 4.94 – 4.76 (m, 1H), 4.14 – 4.04 (m, 3PH), 2.02 – 1.94 (m, 0.5H), 1.91 (s, 2H), 1.72 (ddd, J = 35.4, 13.5, 6.6 Hz, 8H), 1.53 – 1.36 (m, 20.5H), 1.33 – 1.18 (m, 6H), 0.90 – 0.77 (m, 45H), 0.80 – 0.65 (m, 2H). **^{13}C NMR** (101 MHz, $CDCl_3$) δ 170.4, 138.9, 134.6, 132.4, 132.1, 128.1, 71.8 (d, J = 5.8 Hz), 71.2, 71.0 (d, J = 2.4 Hz), 70.4, 49.1, 47.7 (d, J = 4.0 Hz), 46.7 (d, J = 9.1 Hz), 43.8 (d, J = 30.8 Hz), 40.3 (d, J = 35.9 Hz), 33.2 (d, J = 3.6 Hz), 32.3 (d, J = 3.7 Hz), 31.9, 27.15 (d, J = 2.9 Hz), 25.6 (d, J = 2.9 Hz), 24.5 (d, J = 3.0 Hz), 23.2 (d, J = 2.1 Hz), 22.8 (d, J = 17.0 Hz), 22.4 (d, J = 5.4 Hz). **^{11}B NMR** (128 MHz, $CDCl_3$) δ 26.0. **HRMS (ESI)** *m/z* Calcd for $C_{51}H_{92}BClNaO_8Si[M+Na]^+$ 929.6235, Found: 929.6266.

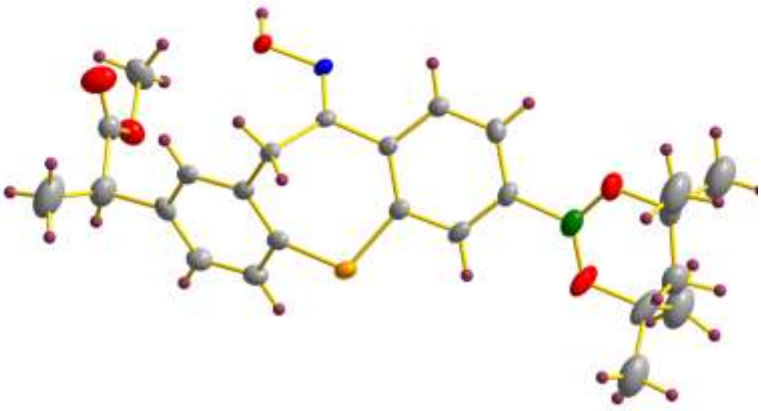

Supplementary Fig: 30. Crystal data and structure refinement for **121**

Crystals were grown from a mixture of *n-Hexane* and THF.

Supplementary Table 1. Crystal data and structure refinement for 121.

CCDC number	2266819
Empirical formula	$C_{25}H_{30}BNO_5S$
Formula weight	467.37
Temperature [K]	177.0

Crystal system	'monoclinic'
Space group (number)	$P2_1/c$ (14)
a [Å]	8.8097(6)
b [Å]	26.7906(19)
c [Å]	11.4477(8)
α [Å]	90
β [Å]	104.017(2)
γ [Å]	90
Volume [$Å^3$]	2621.4(3)
Z	4
ρ_{calc} [g/cm^3]	1.184
μ [mm^{-1}]	0.157
F(000)	992
Crystal size [mm^3]	0.04×0.02×0.02
Crystal colour	colourless
Crystal shape	needle
Radiation	MoK_α (λ=0.71073 Å)
2Θ range [°]	5.26 to 54.86 (0.77 Å)
Index ranges	-11 ≤ h ≤ 11 -34 ≤ k ≤ 34 -14 ≤ l ≤ 14
Reflections collected	43472
Independent reflections	5967 R_{int} = 0.1536 R_{sigma} = 0.0841
Completeness to θ = 25.242°	99.9 %

Data / Restraints / Parameters	5967/0/263
Goodness-of-fit on F^2	1.065
Final R indexes [$I \geq 2\sigma(I)$]	R_1 = 0.1413 wR_2 = 0.3315
Final R indexes [all data]	R_1 = 0.2246 wR_2 = 0.3857
Largest peak/hole [eÅ^3]	0.96/-0.55

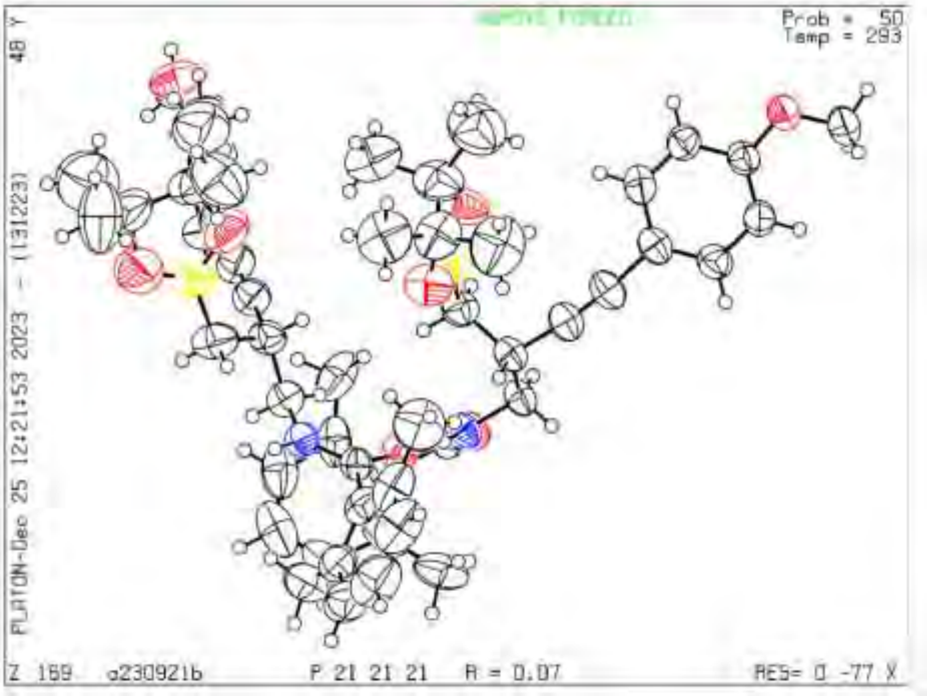

Supplementary Fig: 31. Crystal data and structure refinement for **121**

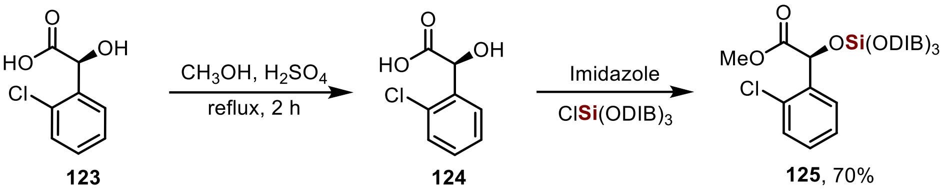

A few drops of conc. H_2SO_4 were added to a stirred solution of (S)-2-Chloromandelic acid **123** (1.86 g, 10 mmol) in methanol (30 mL). The reaction was refluxed for 2 hours, after which the solvent was removed and the residue dissolved in ethyl acetate (25 ml). The product was washed with a 10% aqueous K_2CO_3 solution (2 x 50 ml) and then with brine (2 x 50 ml). The organic layer was dried ($MgSO_4$) and reduced to give the title compound **124** as a clear oil (1.98g, 97%).[14]

To a stirred solution of methyl (R)-2-(2-chlorophenyl)-2-hydroxyacetate **124** (5 mmol, 1.0 equiv) and trialkoxychlorosilane (7.5 mmol, 1.5 equiv) in CH_2Cl_2 (20 mL) was added dropwise a solution of imidazole (7.5 mmol, 1.5 equiv) in CH_2Cl_2 (20 mL) at room temperature. The mixture was stirred at room temperature for 12 h. The white precipitate was removed by filtration under nitrogen. Water 20 mL was added and the product was extracted into CH_2Cl_2, washed with brine, dried over Na_2SO_4 and concentrated. The crude product **125** was purified (5% ethyl acetate in hexane as eluent) by silica gel chromatography as a clear oil (2.38g, 70%).

Methyl (R)-2-(2-chlorophenyl)-2-((tris((2,6-dimethylheptan-4-yl)oxy)silyl)oxy)acetate (125)

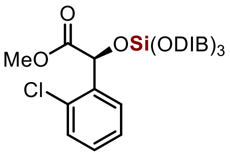

^{1}H NMR (400 MHz, $CDCl_3$) δ 7.62 (dd, J = 7.4, 2.1 Hz, 1H), 7.33 (dd, J = 7.6, 1.7 Hz, 1H), 7.30 – 7.18 (m, 2H), 5.91 (s, 1H), 4.20 – 4.11 (m, 3H), 3.67 (s, 3H), 1.85 – 1.60 (m, 6H), 1.40 (dddd, J = 24.9, 13.5, 7.4, 6.2 Hz, 6H), 1.22 (dddd, J = 13.4, 12.3, 7.9, 5.5 Hz, 6H), 0.94 – 0.73 (m, 36H). **^{13}C NMR** (101 MHz, $CDCl_3$) δ 171.4, 137.1, 132.9, 129.4, 129.3, 129.2, 127.0, 70.8, 70.5, 52.1, 46.7 (d, J = 7.9 Hz), 24.5 (d, J = 2.2 Hz), 23.1, 22.8 (d, J = 14.7 Hz). **HRMS (ESI)** *m/z* Calcd for $C_{36}H_{65}ClNaO_6Si$[M+Na]$^+$ 679.4131, Found: 679.4112.

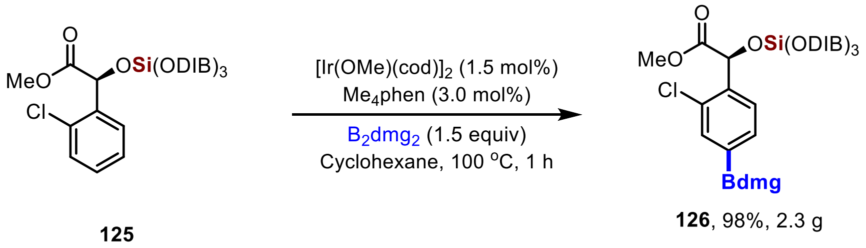

Methyl (R)-2-(2-chlorophenyl)-2-((tris((2,6-dimethylheptan-4-yl)oxy)silyl)oxy)acetate **125** (3 mmol, 1.96 g), B_2dmg_2 (1.27 g, 4.5 mmol, 1.5 equiv), $[Ir(cod)OMe]_2$ (29.4 mg, 0.045 mmol), Me_4phen (212 mg, 0.09 mmol), and cyclohexane (15 mL) were added to a 50 mL glass vial under air atmosphere. The glass vial was capped with a teflon pressure cap and placed into an aluminum block pre-heated to 100 ºC for 1 h. After completion, cyclohexane was removed under reduced pressure and chromatographic separation with silica gel (20% ethyl acetate in hexane as eluent) gave the borylated product **126** (2.3 g, 98%) as colorless liquid.

Methyl (R)-2-(2-chloro-4-(4,4,6,6-tetramethyl-1,3,2-dioxaborinan-2-yl)phenyl)-2-((tris((2,6-dimethylheptan-4-yl)oxy)silyl)oxy)acetate (126)

^{1}H NMR (400 MHz, $CDCl_3$) δ 7.69 (d, J = 1.1 Hz, 1H), 7.59 (dd, J = 7.7, 1.2 Hz, 1H), 7.50 (d, J = 7.7 Hz, 1H), 5.84 (s, 1H), 4.07 – 3.97 (m, 3H), 3.55 (s, 3H), 1.82 (s, 2H), 1.72 – 1.56 (m, 6H), 1.41 – 1.24 (m, 18H), 1.19 – 1.08 (m, 6H), 0.87 – 0.69 (m, 36H). **^{13}C NMR** (101 MHz, $CDCl_3$) δ 171.4, 138.5, 134.7, 132.4, 132.2, 128.2, 71.2, 70.9, 70.4, 52.0, 49.1, 46.7 (d, J = 8.5 Hz), 24.5 (d, J = 1.6 Hz), 23.1, 22.8, 22.7. **^{11}B NMR** (128 MHz, $CDCl_3$) δ 26.1. **HRMS (ESI)** *m/z* Calcd for $C_{43}H_{78}BClNaO_8Si[M+Na]^+$ 819.5140, Found: 819.5177.

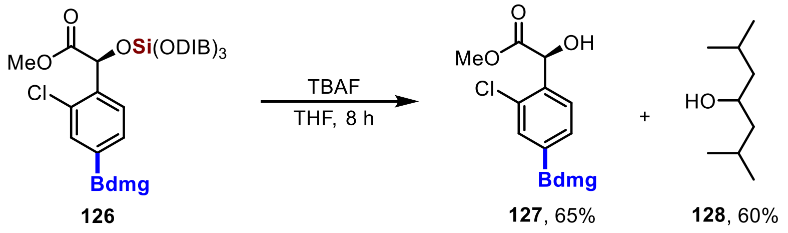

Methyl (R)-2-(2-chloro-4-(4,4,6,6-tetramethyl-1,3,2-dioxaborinan-2-yl)phenyl)-2-((tris((2,6-dimethylheptan-4-yl)oxy)silyl)oxy)acetate **126** (2 mmol, 1.6 g)was dissolved in 5 mL THF and cooled to 0 ºC. The solution was treated with 6.0 mL TBAF (1M in THF, 3.0 equiv.) and warmed to room temperature. The mixture was stirred at room temperature for 8 h until the starting material was fully con-sumed monitored by TLC. The reaction was quenched with brine and extracted with diethyl ether (3 x 10 mL) and dried over $MgSO_4$. After filtration and removal of solvent, the crude **127** was purified by silica gel chromatography to give a gum.[15]

Methyl 2-(2-chloro-4-(4,4,6,6-tetramethyl-1,3,2-dioxaborinan-2-yl)phenyl)-2-hydroxyacetate (127)

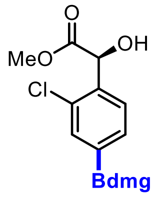

Yield: 65% (472.1 mg). Colorless liquid. Eluent ($V_{petroleum\ ether}$: V_{EtOAc} = 15:1). **^{1}H NMR** (400 MHz, $CDCl_3$) δ 7.82 (d, *J* = 1.2 Hz, 1H), 7.71 (dd, *J* = 7.6, 1.2 Hz, 1H), 7.34 (d, *J* = 7.6 Hz, 1H), 5.57 (d, *J* = 5.2 Hz, 1H), 3.74 (s, 3H), 3.54 (d, *J* = 5.3 Hz, 1H), 1.91 (s, 2H), 1.41 (s, 12H). **^{13}C NMR** (101 MHz, $CDCl_3$) δ 173.9, 137.6, 135.3, 133.1, 132.6, 128.1, 71.4, 70.6, 53.2, 49.1, 31.9. **^{11}B NMR** (128 MHz, $CDCl_3$) δ 25.9. **HRMS (ESI)** *m/z* Calcd for $C_{16}H_{22}BClNaO_5$ $[M+Na]^+$ 363.1141, Found: 363.1148.

2,6-dimethylheptan-4-ol (128)

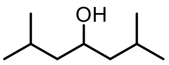

2,6-dimethylheptan-4-ol obtained by distillation under reduced pressure.

Colorless liquid, 518 mg. **^{1}H NMR** (400 MHz, $CDCl_3$) δ 3.72 (tt, *J* = 8.6, 4.1 Hz, 1H), 1.82 – 1.68 (m, 1H), 1.44 (s, 1H), 1.38 – 1.29 (m, 2H), 1.19 (ddd, *J* = 13.7, 8.8, 4.2 Hz, 2H), 0.89 (dd, *J* = 6.6, 2.1 Hz, 12H). **^{13}C NMR** (101 MHz, $CDCl_3$) δ 67.9, 47.5, 24.7, 23.5, 22.2.

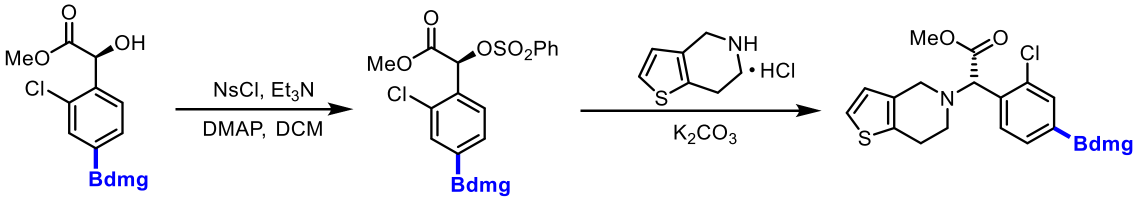

127 **129**, 78% **130**, 80%, 95% ee

To a stirred mixture of DMAP (7.3 mg, 0.06 mmol), Methyl 2-(2-chloro-4-(4,4,6,6-tetramethyl-1,3,2-dioxaborinan-2-yl)phenyl)-2-hydroxyacetate **127** (204 mg, 0.6 mmol) and Et_3N (0.078 g, 0.78 mmol) in CH_2Cl_2 (1 mL) at 0 °C was slowly added an ice-cold solution of 4-nitrobenzenesulfonyl chloride (133 mg, 0.6 mmol) in CH_2Cl_2 (1.5 mL). After stirring for 3 h at the same temperature, the mixture was quenched with water. The organic layer was separated, dried over anhydrous sodium sulfate and concentrated under reduced pressure. The residue was purified by flash column chromatography to afford the title compound **129** as a light yellow solid (246 mg, 78% yield).[16]

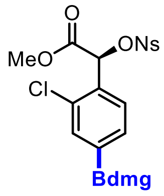

Methyl (S)-2-(2-chloro-4-(4,4,6,6-tetramethyl-1,3,2-dioxaborinan-2-yl)phenyl)-2-(((4-nitrophenyl)sulfonyl)oxy)acetate (129)

^{1}H NMR (400 MHz, $CDCl_3$) δ 8.22 (d, *J* = 8.9 Hz, 2H), 7.98 (d, *J* = 8.8 Hz, 2H), 7.68 (s, 1H), 7.58 (dd, *J* = 7.7, 1.2 Hz, 1H), 7.26 (d, *J* = 7.7 Hz, 1H), 6.36 (s, 1H), 3.72 (s, 3H), 1.90 (s, 2H), 1.39 (s, 12H). **^{13}C NMR**

(101 MHz, $CDCl_3$) δ 166.9, 150.6, 142.2, 135.1, 133.3, 132.6, 131.6, 129.3, 128.9, 124.1, 78.0, 71.5, 53.3, 48.9, 31.7.

^{13}C NMR (101 MHz, $CDCl_3$) δ 166.9, 150.6, 142.2, 135.1, 133.3, 132.6, 131.6, 129.3, 128.9, 124.1, 78.0, 71.5, 53.3, 48.9, 31.7. **^{11}B NMR** (128 MHz, $CDCl_3$) δ 25.3. **HRMS (ESI)** *m/z* Calcd for $C_{22}H_{25}BClNaO_9S$ $[M+Na]^+$ 548.0924, Found: 548.0939.

To a stirred mixture of 4,5,6,7-tetrahydrothieno [3,2-c] pyridine (50.1 mg, 0.36 mmol) and 30% aqueous solution of potassium carbonate (0.4 g) in CH_2Cl_2 (1.5 mL) was added a solution of compound **129** (157.5 mg, 0.3 mmol) in CH_2Cl_2 (0.5 mL). The two-phase mixture was refluxed for 2.5 h. The mixture was cooled to room temperature and filtered under vaccum then washed with a small amount of CH_2Cl_2 to give the methyl (S)-2-(2-chloro-4-(4,4,6,6-tetramethyl-1,3,2-dioxaborinan-2-yl)phenyl)-2-(6,7-dihydrothieno[3,2-c]pyridin-5(4H)-yl)acetate **130** (110.8 mg, 80%, 95% ee, determined by HPLC analysis).[16]

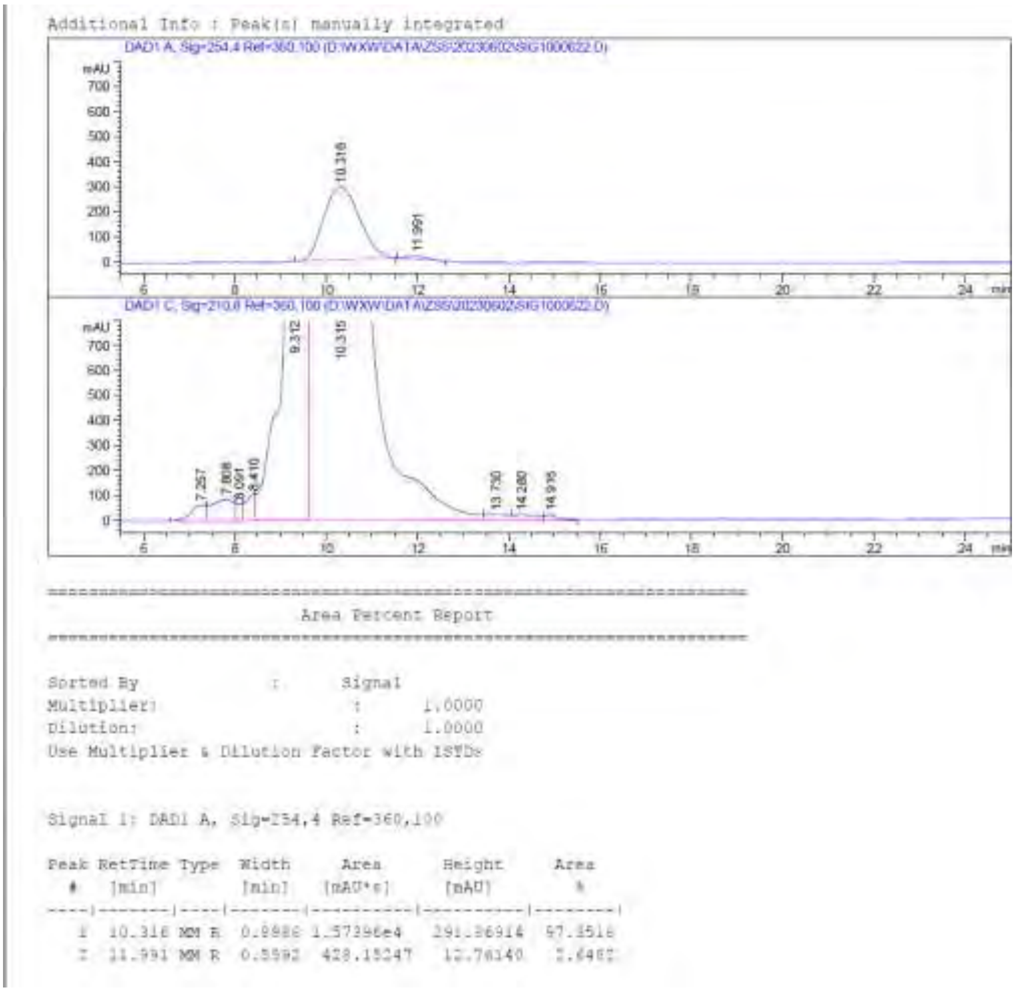

Supplementary Fig: 32. HPLC spectra of **130** (chiral)

Additional Info : Peak(s) manually integrated

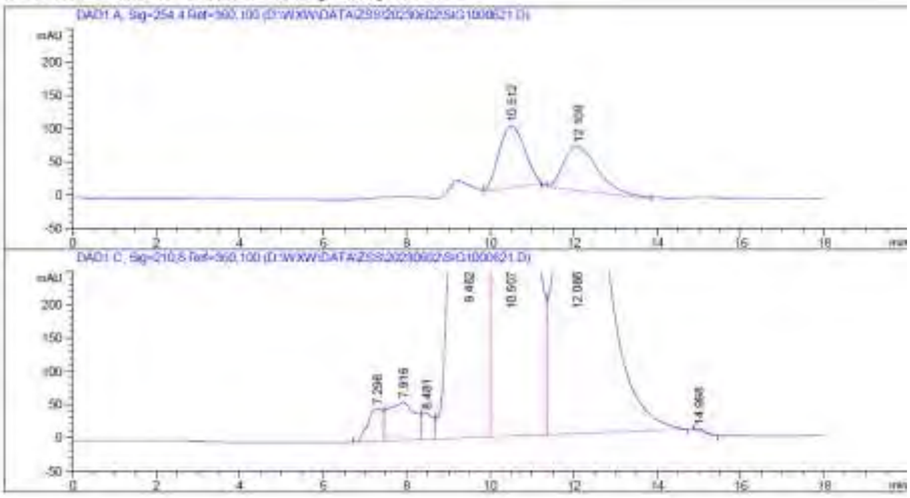

Area Percent Report

Sorted By : Signal
Multiplier: : 1.0000
Dilution: : 1.0000
Use Multiplier & Dilution Factor with ISTDs

Signal 1: DAD1 A, Sig=254,4 Ref=360,100

Peak #	RetTime [min]	Type	Width [min]	Area [mAU*s]	Height [mAU]	Area %
1	10.512	MM R	0.7120	4003.22900	93.71118	50.6871
2	12.109	MM R	0.9677	3894.68848	67.07938	49.3129

Supplementary Fig. 33: HPLC spectra of **130** (racemic)

Methyl (S)-2-(2-chloro-4-(4,4,6,6-tetramethyl-1,3,2-dioxaborinan-2-yl)phenyl)-2-(6,7-dihydrothieno[3,2-c]pyridin-5(4H)-yl)acetate (130)

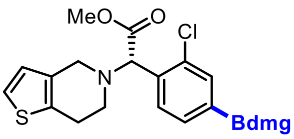

^{1}H NMR (400 MHz, $CDCl_3$) δ 7.85 (d, *J* = 1.2 Hz, 1H), 7.72 (dd, *J* = 7.6, 1.2 Hz, 1H), 7.65 (d, *J* = 7.8 Hz, 1H), 7.05 (d, *J* = 5.1 Hz, 1H), 6.65 (d, *J* = 5.1 Hz, 1H), 4.93 (s, 1H), 3.76 (d, *J* = 14.2 Hz, 1H), 3.69 (s, 3H), 3.61 (d, *J* = 14.2 Hz, 1H), 2.86 (s, 4H), 1.92 (s, 2H), 1.42 (s, 12H). **^{13}C NMR** (101 MHz, $CDCl_3$) δ 171.5, 135.5, 135.1, 134.5, 133.5, 133.4, 132.5, 129.2, 125.4, 122.8, 71.4, 68.1, 52.3, 50.9, 49.1, 48.4, 31.9, 25.6. **^{11}B NMR** (128 MHz, $CDCl_3$) δ 25.9. **HRMS (ESI)** *m/z* Calcd for $C_{23}H_{29}BClNNaO_4S$ $[M+Na]^+$ 484.1491, Found: 484.1479.

7. Silylarenes as useful building blocks for the construction of 4-acetoxybiphenyl derivatives

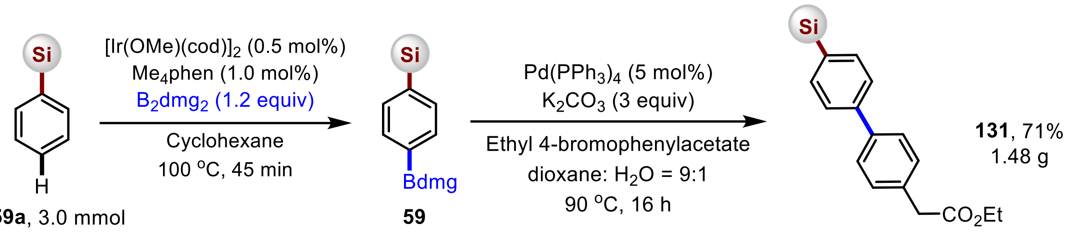

59a, 3.0 mmol **Gram scale** **59** **one pot** **131**, 71% 1.48 g

Tris((2,6-dimethylheptan-4-yl)oxy)(phenyl)silane **59a** (3 mmol, 1.6 g), B_2dmg_2 (1.01 g, 3.6 mmol, 1.2 equiv), $[Ir(cod)OMe]_2$ (10.0 mg, 0.045 mmol), Me_4phen (7.1 mg, 0.03 mmol), and cyclohexane (10.0 mL) were added to a 50 mL glass vial under air atmosphere. The glass vial was capped with a teflon pressure cap and placed into an aluminum block pre-heated to 100 °C for 45 min. After completion, cyclohexane was removed under reduced pressure. In an argon filled glove box, a 50.0 mL three-necked flask was charged with **59** (2.0 g, 3.0 mmol), $Pd(PPh_3)_4$ (87 mg, 2.5 mol%), K_2CO_3 (828 mg, 2.0 equiv.) and Ethyl 4-bromophenylacetate (875.1 mg, 1.2 equiv.). The reaction vial was taken out from glove box, DME (10 mL) and water (10 mL) was added. The three-necked flask was placed in a preheated aluminium block at 100 °C and heated for 12 h. After 12 h, the reaction mixture was cooled to room temperature, diluted with water (20 mL) and extracted with ethyl acetate (20 mL x 3). The combine organic layer was washed with brine (20 mL), dried over anhydrous Na_2SO_4 and S103 concentrated under reduced pressure. Chromatographic purification of crude mass with silica gel (2% ethyl acetate in hexane as eluent) gave 1.48 g (71%) of the product (**131**) as gummy liquid.[17]

Ethyl 2-(4'-(tris((2,6-dimethylheptan-4-yl)oxy)silyl)-[1,1'-biphenyl]-4-yl)acetate (131)

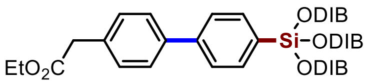

^{1}H NMR (400 MHz, Chloroform-*d*) δ 7.73 (d, *J* = 7.7 Hz, 2H), 7.60 (t, *J* = 7.5 Hz, 4H), 7.38 (d, *J* = 8.0 Hz, 2H), 4.19 (q, *J* = 7.1 Hz, 2H), 4.12 (p, *J* = 6.3 Hz, 3H), 3.67 (s, 2H), 1.83 – 1.71 (m, 6H), 1.55 – 1.42 (m, 6H), 1.35 – 1.26 (m, 6H), 0.86 (dd, *J* = 6.8, 2.7 Hz, 36H). **^{13}C NMR** (101 MHz, Chloroform-*d*) δ 171.7, 141.7, 140.1, 135.6, 133.4, 132.0, 129.8, 127.4, 126.1, 70.0, 61.1, 47.1, 41.2, 24.6, 23.2, 22.9, 14.3. **HRMS (ESI)** *m/z* Calcd for $C_{43}H_{72}NaO_5Si$ $[M+Na]^+$ 719.5041, Found: 719.5055.

Ethyl 2-(4'-iodo-[1,1'-biphenyl]-4-yl)acetate (133)

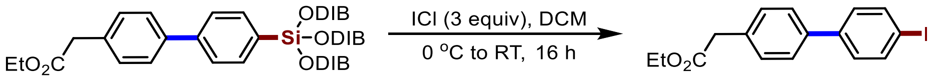

131 **133**, 80%

To a solution of **131** (69.7 mg, 0.1 mmol) in CH_2Cl_2 (1 mL) was added a solution of ICl (48.7 mg, 0.3 mmol) in CH_2Cl_2 (1 mL) at 0 °C. The reaction mixture was stirred at 23 °C for 16 h. The volatile materials were evaporated, and the residue was purified by flash column chromatography (0-20% ethyl acetate in hexanes) to give the product **133** (29.3 mg, 80%) as a colorless liquid.[18]

Data matches that previously reported.[19]

^{1}H NMR (400 MHz, Chloroform-*d*) δ 7.76 (d, *J* = 8.4 Hz, 2H), 7.51 (d, *J* = 8.3 Hz, 2H), 7.36 (d, *J* = 8.2 Hz, 2H), 7.32 (d, *J* = 8.4 Hz, 2H), 4.18 (q, *J* = 7.1 Hz, 2H), 3.66 (s, 2H), 1.28 (t, *J* = 7.1 Hz, 3H). **^{13}C NMR** (101 MHz, Chloroform-*d*) δ 171.6, 140.4, 138.9, 137.9, 133.8, 129.9, 129.0, 127.1, 93.1, 61.1, 41.1, 14.3.

Methyl 4''-(2-ethoxy-2-oxoethyl)-[1,1':4',1''-terphenyl]-3-carboxylate (134)

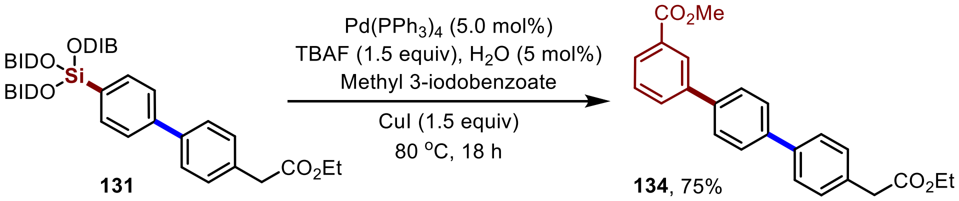

In an argon filled glove box, a 15 mL flask charged with Methyl 3-iodobenzoate (26.2 mg, 0.1 mmol), $Pd(PPh_3)_4$ (5.8 mg, 0.005 mmol), CuI (38.1 mg, 0.2 mmol). The reaction vial was taken out from glove box, THF (4 mL) were added, followed by **131** (1.5 equiv, 0.15 mmol), TBAF (1.5 equiv, 1.0 M in THF) and H_2O (0.2 mL). The flask was fitted with a condenser and the reaction mixture was stirred 7 h at 80 °C. The crude product obtained after filtration and concentration in vacuo was purified by column chromatography with ethyl acetate/hexane (1:10) as eluents to provide **134** (28.1 mg, 75%) as a white solid.[20]

^{1}H NMR (400 MHz, Chloroform-*d*) δ 8.33 (s, 1H), 8.03 (d, *J* = 9.2 Hz, 1H), 7.87 – 7.80 (m, 1H), 7.74 – 7.65 (m, 4H), 7.61 (d, *J* = 8.2 Hz, 2H), 7.53 (t, *J* = 7.8 Hz, 1H), 7.39 (d, *J* = 8.0 Hz, 2H), 4.19 (q, *J* = 7.1 Hz, 2H), 3.96 (s, 3H), 3.67 (s, 2H), 1.28 (t, *J* = 7.1 Hz, 3H). **^{13}C NMR** (101 MHz, Chloroform-*d*) δ 171.7, 167.2, 141.1, 140.3, 139.4, 139.1, 133.6, 131.5, 130.9, 129.9, 129.1, 128.6, 128.3, 127.7, 127.3, 61.1, 52.4, 41.2, 14.3. **HRMS (ESI)** *m/z* Calcd for $C_{24}H_{23}O_4$ $[M+H]^+$ 375.1591, Found: 375.1596.

Eethyl 2-(4''-methyl-[1,1':4',1''-terphenyl]-4-yl)acetate (135)

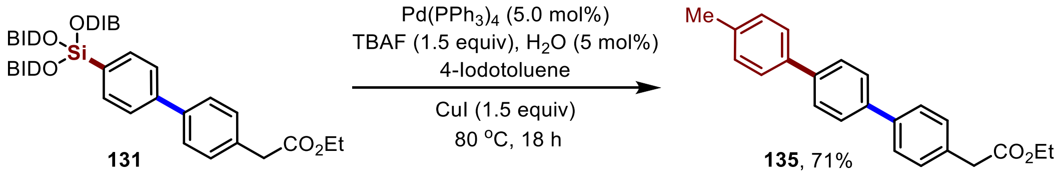

In an argon filled glove box, a 15 mL flask charged with 4-iodotoluene (21.8 mg, 0.1 mmol), $Pd(PPh_3)_4$ (5.8 mg, 0.005 mmol), CuI (38.1 mg, 0.2 mmol). The reaction vial was taken out from glove box, THF (4 mL) were added, followed by **131** (1.5 equiv, 0.15 mmol), TBAF (1.5 equiv, 1.0 M in THF) and H_2O (0.2 mL). The flask was fitted with a condenser and the reaction mixture was stirred 7 h at 80 ºC. The crude product obtained after filtration and concentration in vacuo was purified by column chromatography with ethyl acetate/hexane (1:10) as eluents to provide **135** (23.5 mg, 71%) as a white solid.[20]

^{1}H NMR (400 MHz, Chloroform-*d*) δ 7.68 (s, 4H), 7.63 (d, *J* = 8.2 Hz, 2H), 7.57 (d, *J* = 8.1 Hz, 2H), 7.40 (d, *J* = 8.2 Hz, 2H), 7.29 (d, *J* = 7.9 Hz, 2H), 4.21 (d, *J* = 7.1 Hz, 2H), 3.69 (s, 2H), 2.43 (s, 3H), 1.31 (t, *J* = 7.1 Hz, 3H). **^{13}C NMR** (101 MHz, Chloroform-*d*) δ 171.7, 140.2, 139.7, 139.5, 137.9, 137.3, 133.3, 129.8, 129.7, 127.5, 127.4, 127.3, 127.0, 61.1, 41.2, 21.2, 14.3. **HRMS** (ESI) *m/z* Calcd for $C_{23}H_{22}NaO_2$ $[M+Na]^+$ 353.1512, Found: 353.1515.

Ethyl 2-(4'-(1H-benzo[d]imidazol-1-yl)-[1,1'-biphenyl]-4-yl)acetate (136)

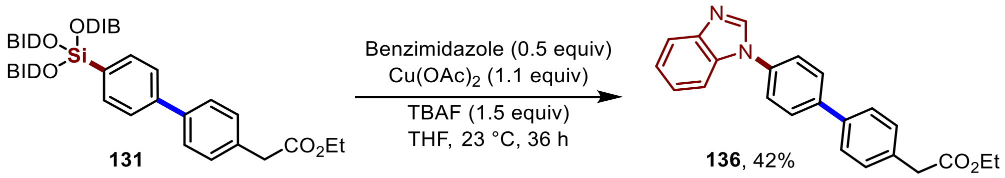

To a stirring mixture of **131** (98.0 mg, 0.2 mmol), benzimidazole (11.8 mg, 0.1 mmol), and $Cu(OAc)_2$ (22.0 mg, 0.11 mmol) in DMF (1.0 mL) was added drop wise TBAF (0.15 mL of 1.0 M THF solution) at 23 °C, and the reaction mixture was stirred at this temperature for 36 h. The mixture was then partitioned between a mixture of ethyl acetate (15 mL), hexanes (7 mL), and saturated aqueous $NaHCO_3$ (10 mL). The aqueous layer was extracted with a mixture of ethyl acetate and hexanes (2:1, 10 mL × 2), the combined organic layer washed with water (10 mL) and brine (10 mL), dried over $MgSO_4$, filtered, and the solvents were evaporated. The residue was purified by flash column chromatography (10% ethyl acetate in hexanes) to afford **136** (15.0 mg, 42%) the product as a brown liquid.[21]

^{1}H NMR (400 MHz, Chloroform-*d*) δ 8.16 (s, 1H), 7.97 – 7.84 (m, 1H), 7.78 (d, *J* = 8.5 Hz, 2H), 7.67 – 7.56 (m, 5H), 7.42 (d, *J* = 8.3 Hz, 2H), 7.39 – 7.33 (m, 2H), 4.19 (q, *J* = 7.1 Hz, 2H), 3.69 (s, 2H), 1.29 (t, *J* = 7.1 Hz, 3H). **^{13}C NMR** (101 MHz, Chloroform-*d*) δ 171.6, 142.4, 140.8, 138.7, 135.6, 134.1, 130.1, 128.7, 127.4, 124.5, 123.9, 123.0, 120.8, 110. 7, 61.2, 41.2, 14.3. **HRMS** (ESI) *m/z* Calcd for $C_{23}H_{20}N_2NaO_2$ $[M+Na]^+$ 379.1417, Found: 379.1420.

Ethyl 2-(4'-bromo-[1,1'-biphenyl]-4-yl)acetate (137)

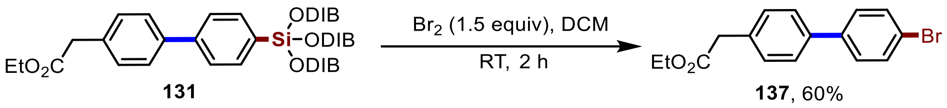

To a solution of **131** (69.7 mg, 0.1 mmol) in CH_2Cl_2 (2 mL) was added bromine (24.0 mg, 0.15 mmol) at 0 °C, and the reaction mixture was stirred at 23 °C for 2 h. The reaction was then quenched with $Na_2S_2O_3$ (30% aqueous solution, 2 mL) at 0 °C. The aqueous phase was extracted with ethyl acetate, the combined organic layer was dried over $MgSO_4$, filtered, and the solvent was evaporated. The residue was purified by flash column chromatography (20% ethyl acetate in hexanes) to give the product **137** (19.2 mg, 60%) as a colorless liquid.[18]

^{1}H NMR (400 MHz, Chloroform-*d*) δ 7.55 (d, *J* = 8.5 Hz, 2H), 7.52 (d, *J* = 8.2 Hz, 2H), 7.44 (d, *J* = 8.5 Hz, 2H), 7.36 (d, *J* = 8.3 Hz, 2H), 4.18 (q, *J* = 7.1 Hz, 2H), 3.66 (s, 2H), 1.28 (t, *J* = 7.1 Hz, 3H). **^{13}C NMR** (101 MHz, Chloroform-*d*) δ 171.6, 139.8, 138.9, 133.8, 132.0, 130.0, 128.8, 127.2, 121.6. **HRMS** (ESI) *m/z* Calcd for $C_{16}H_{15}BrNaO_2$ $[M+Na]^+$ 341.0148, Found: 341.0152.

Ethyl 2-(4'-cinnamyl-[1,1'-biphenyl]-4-yl)acetate (138)

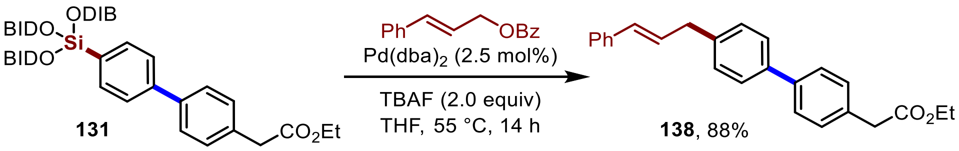

In an argon filled glove box, a 15 mL glass tube charged with trans-cinnamoyl benzoate[15] (23.8 mg, 0.1 mmol), $Pd(dba)_2$ (23.8 mg, 0.01 mmol). The reaction tube was taken out from glove box, **131** (139.4 mg, 0.2 mmol) in 1 mL of THF was added 2.0 equiv of TBAF via syringe. The reaction glass tube was placed in a preheated aluminium block at 55 °C and heated for 14 h. The reaction mixture was quenched by the addition of 5 mL of water. The aqueous layer was extracted with 4 × 5 mL of Et_2O, and the combined organic layers were dried over Na_2SO_4 and concentrated

in vacuo. The crude product obtained after filtration and concentration in vacuo was purified by column chromatography with ethyl acetate/hexane (1:20) as eluents to provide **138** (31.4 mg, 88%) as a colorless liquid.[22]

^{1}H NMR (400 MHz, Chloroform-*d*) δ 7.55 (dd, *J* = 8.2, 6.2 Hz, 4H), 7.42 – 7.26 (m, 8H), 7.26 – 7.13 (m, 1H), 6.51 (d, *J* = 15.8 Hz, 1H), 6.46 – 6.27 (m, 1H), 4.19 (q, *J* = 7.2 Hz, 2H), 3.66 (s, 2H), 3.60 (d, *J* = 6.8 Hz, 2H), 1.28 (t, *J* = 7.1 Hz, 3H). **^{13}C NMR** (101 MHz, Chloroform-*d*) δ 171.8, 140.0, 139.4, 138.9, 137.6, 133.2, 131.4, 129. 8, 129.2, 129.2, 128.7, 127.37, 127.3, 126.3, 61.1, 41.2, 39.1, 14.3. **HRMS** (ESI) *m/z* Calcd for $C_{25}H_{24}NaO_2$ $[M+Na]^+$ 379.1669, Found: 379.1677.

Butyl (E)-3-(4'-(2-ethoxy-2-oxoethyl)-[1,1'-biphenyl]-4-yl)acrylate (139)

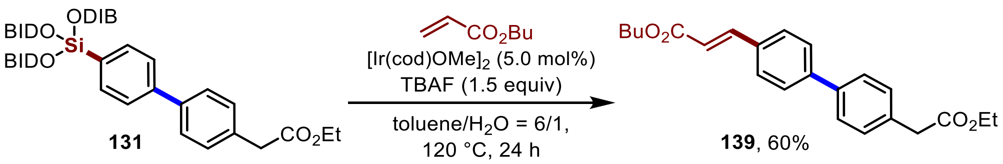

A 1 M solution of TBAF (0.2 mL) in THF was added to a mixture of **131** (69.7 mg, 0.1 mmol), butyl acrylate (38.5 mg, 0.3 mmol), and [Ir (cod)OMe] (3.4 mg, 0.005 mmol) in THF (2 mL) and H_2O (0.33 mL) under an argon atmosphere. The resulting solution was allowed to warm to 120 °C and the reaction was continued for 24 h. After cooling the solution to room temperature it was passed through a short celite pad. The crude product obtained after filtration and concentration in vacuo was purified by column chromatography with ethyl acetate/hexane (1:20) as eluents to provide **139** (22.0 mg, 60%) as a colorless liquid.[23]

^{1}H NMR (400 MHz, Chloroform-*d*) δ 7.71 (d, *J* = 16.0 Hz, 1H), 7.64 – 7.52 (m, 6H), 7.37 (d, *J* = 8.2 Hz, 2H), 6.47 (d, *J* = 16.0 Hz, 1H), 4.26 – 4.13 (m, 4H), 3.66 (s, 2H), 1.81 – 1.65 (m, 2H), 1.52 – 1.38 (m, 2H), 1.27 (t, *J* = 7.1 Hz, 3H), 0.98 (t, *J* = 7.4 Hz, 3H). **^{13}C NMR** (101 MHz, Chloroform-*d*) δ 171.6, 167.3, 144.2, 142.7, 139.1, 134.0, 133.6, 130.0, 128.7, 127.6, 127.3, 118.3, 64.6, 61.1, 41.2, 30.9, 19.4, 17.8, 13.9. **HRMS** (ESI) *m/z* Calcd for $C_{23}H_{26}NaO_4$ $[M+Na]^+$ 389.1723, Found: 389.1731.

Ethyl 2-(4'-fluoro-[1,1'-biphenyl]-4-yl)acetate (140)

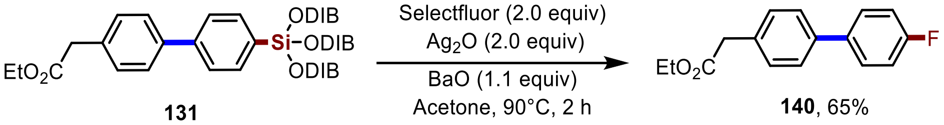

To **131** (0.1 mmol) in acetone (2.0 mL) at 25 °C was added silver oxide (69.6 mg, 0.3 mmol, 3.0 equiv), barium oxide (17.2 mg , 0.11 mmol, 1.1 equiv) and 1-chloromethy1-4-fluoro-1,4-diazoniabicyclo-[2.2.2]octane bis(tetrafluoroborate) (70.8 mg, 0.2 mmol, 2.00 equiv). The reaction mixfure was stirred for 2 h at 90 °C in a sealed vial. The reaction mixture was cooled to 23 °C, passed through a pad of Celite and concentrated under reduced pressure. To the residue was added CH_2C1_2 (20 mL) and a saturated aqueous solution of $NaHCO_3$ (20 mL). The organic phase was separated, dried over Na_2SO_4, filtered and concentratedunder reduced pressure. The crude product obtained after filtration and concentration in vacuo was purified by column chromatography with ethyl acetate/hexane (1:20) as eluents to provide **140** (16.8 mg, 65%) as a colorless liquid.[24]

^{1}H NMR (400 MHz, Chloroform-*d*) δ 7.59 – 7.47 (m, 4H), 7.36 (d, *J* = 8.3 Hz, 2H), 7.17 – 7.08 (m, 2H), 4.18 (q, *J* = 7.1 Hz, 2H), 3.66 (s, 2H), 1.28 (t, *J* = 7.1 Hz, 3H). **^{13}C NMR** (101 MHz, Chloroform-*d*) δ 171.7, 162.6 (d, *J* = 246.1 Hz), 139.2, 137.1 (d, *J* = 3.3 Hz), 133.4, 129.9, 128.7 (d, *J* = 8.0 Hz), 127.3, 115.7 (d, *J* = 21.3 Hz), 61.1, 41.1, 14.3. **^{19}F NMR** (376 MHz, Chloroform-*d*) δ -115.8 **HRMS** (ESI) *m/z* Calcd for $C_{16}H_{15}FNaO_2$ $[M+Na]^+$ 281.0948, Found: 281.0939.

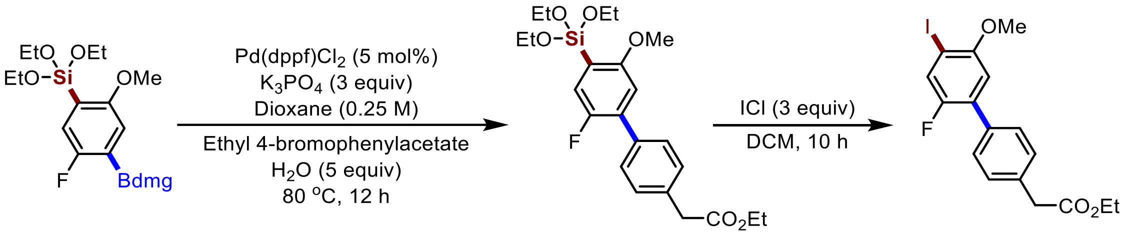

88 **141**, 65% **142**, 77%

Ethyl 2-(2'-fluoro-5'-methoxy-4'-(triethoxysilyl)-[1,1'-biphenyl]-4-yl)acetate (141)

To an oven-dried microwave vial was added Ethyl 4-bromophenylacetate (43.8 mg, 0.4 mmol, 1 equiv), **88** (33.5 mg, 0.44 mmol, 1.1 equiv), $Pd(dppf)Cl_2$ (14.5 mg, 0.02 mmol, 4 mol%), and K_3PO_4 (254.8 mg, 1.2 mmol, 3 equiv). The vial was capped and purged with N_2 before the addition of 1,4-dioxane (1.6 mL, 0.25 M), followed by H_2O (36.0 µL, 2.0 mmol, 5 equiv). The reaction mixture was heated for 4 h at 80 °C with stirring. The vial was then allowed to cool to room temperature, decapped, diluted with EtOAc (10 mL) and filtered through a plug of celite, eluting with EtOAc. The resulting solution was washed with H_2O (3 x 10 mL) followed by brine (10 mL) and the organic phases collected. The organic phase was dried over Na_2SO_4. The crude product obtained after filtration and concentration in vacuo

was purified by column chromatography with ethyl acetate/hexane (1:20) as eluents to provide **141** (117.0 mg, 65%) as a colorless liquid.

^{1}H NMR (400 MHz, Chloroform-*d*) δ 7.55 – 7.49 (m, 2H), 7.42 (d, *J* = 10.2 Hz, 1H), 7.39 – 7.35 (m, 2H), 6.85 (d, *J* = 5.6 Hz, 1H), 4.18 (q, *J* = 7.1 Hz, 2H), 3.90 (q, *J* = 7.0 Hz, 6H), 3.84 (s, 3H), 3.66 (s, 2H), 1.35 – 1.17 (m, 12H). **^{13}C NMR** (101 MHz, Chloroform-*d*) δ 171.6, 160.5, 154.2 (d, *J* = 241.8 Hz), 134.9, 134.1, 131.4 (d, *J* = 15.2 Hz), 129.5, 129.2 (d, *J* = 3.0 Hz), 124.6 (d, *J* = 23.4 Hz), 120.7 (d, *J* = 11.0 Hz), 111.7 (d, *J* = 2.8 Hz), 61.1, 59.0, 56.0, 41.3, 18.3, 14.3. **^{19}F NMR** (376 MHz, Chloroform-*d*) δ -129.6. **HRMS** (ESI) *m/z* Calcd for $C_{23}H_{31}FNaO_6$ $[M+Na]^+$ 473.1766, Found: 473.1759.

Ethyl 2-(2'-fluoro-4'-iodo-5'-methoxy-[1,1'-biphenyl]-4-yl)acetate (142)

To a solution of **88** (90.1 mg, 0.2 mmol) in CH_2Cl_2 (2.0 mL) was added a solution of ICl (97.4 mg, 0.6 mmol) in CH_2Cl_2 (1 mL) at 0 °C. The reaction mixture was stirred at 23 °C for 10 h. The volatile materials were evaporated, and the residue was purified by flash column chromatography (0-20% ethyl acetate in hexanes) to give the product **142** (63.8 mg, 77%) as a yellow liquid.

^{1}H NMR (400 MHz, Chloroform-*d*) δ 7.57 (d, *J* = 9.3 Hz, 1H), 7.53 – 7.46 (m, 2H), 7.37 (d, *J* = 8.3 Hz, 2H), 6.82 (d, *J* = 6.5 Hz, 1H), 4.18 (q, *J* = 7.1 Hz, 2H), 3.89 (s, 3H), 3.66 (s, 2H), 1.28 (t, *J* = 7.1 Hz, 3H). **^{13}C NMR** (101 MHz, Chloroform-*d*) δ 171.4, 154.9 (d, *J* = 4.4 Hz), 152.5, 134.1, 129.5, 129.2 (d, *J* = 15.0 Hz), 129.0 (d, *J* = 3.1 Hz), 126.7 (d, *J* = 26.9 Hz), 111.8 (d, *J* = 3.6 Hz), 83.8 (d, *J* = 8.6 Hz), 61.0, 57.0, 41.1, 14.2. **^{19}F NMR** (376 MHz, Chloroform-*d*) δ -126.8. **HRMS** (ESI) *m/z* Calcd for $C_{17}H_{17}FIO_3$ $[M+H]^+$ 415.0201, Found: 415.0209.

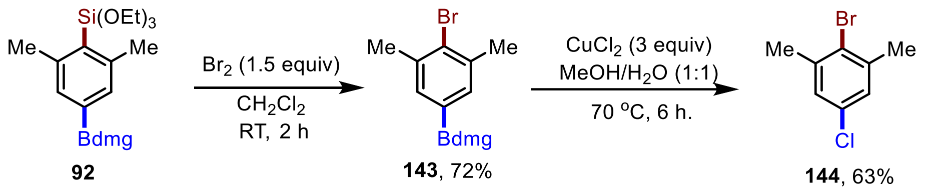

2-(4-bromo-3,5-dimethylphenyl)-4,4,6,6-tetramethyl-1,3,2-dioxaborinane (143)

To a solution of **92** (163.4 mg, 0.4 mmol) in CH_2Cl_2 (4 mL) was added bromine (48.0 mg, 0.3 mmol) at 0 °C, and the reaction mixture was stirred at 23 °C for 2 h. The reaction was then quenched with $Na_2S_2O_3$ (30% aqueous solution, 2 mL) at 0 °C. The aqueous phase was extracted with ethyl acetate, the combined organic layer was dried

over $MgSO_4$, filtered, and the solvent was evaporated. The residue was purified by flash column chromatography (20% ethyl acetate in hexanes) to give the product **143** (93.6 mg, 72%) as a colorless liquid.

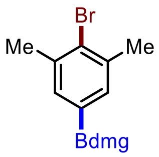

^{1}H NMR (400 MHz, Chloroform-*d*) δ 7.51 (s, 2H), 2.44 (s, 6H), 1.91 (s, 2H), 1.68 – 1.33 (m, 12H). **^{13}C NMR** (101 MHz, Chloroform-*d*) δ 137.3, 133.7, 130.5, 71.1, 49.1, 31.9, 23.9. **^{11}B NMR** (128 MHz, Chloroform-*d*) δ 26.4. **HRMS (ESI)** *m/z* Calcd for $C_{15}H_{23}BBrO_2$ $[M+H]^+$ 325.0969, Found: 325.0977.

2-bromo-5-chloro-1,3-dimethylbenzene (144)

In a 10 mL round-bottom flask, **143** (65.0 mg, 0.2 mmol), $CuCl_2$ (80.7mg, 3.0 equiv.) were dissolved in $MeOH/H_2O$ (2 mL:2 mL) under air. The reaction mixture was stirred at 70 °C for 6 h. The reaction mixture was then cooled to room temperature, solvent evaporated and extracted three times with ethyl acetate. After that, the organic layer was dried over Na_2SO_4 and concentrated under reduced pressure. The crude mass was purified by silica gel column chromatography (5% ethyl acetate in hexane as eluent) to give **144** (27.6 mg, 63%) as colorless liquid.

Data matches that previously reported[8].

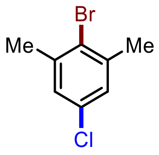

^{1}H NMR (400 MHz, Chloroform-*d*) δ 7.06 (s, 2H), 2.39 (s, 6H). **^{13}C NMR** (101 MHz, Chloroform-*d*) δ 134.0, 132.3, 128.0, 125.6, 23.9.

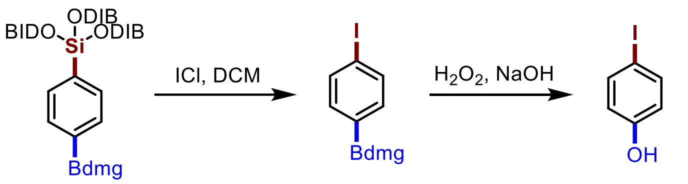

59 **145**, 84% **146**, 87%

To a solution of **59** (269.7 mg, 0.4 mmol) in CH_2Cl_2 (4 mL) was added a solution of ICl (194.8 mg, 1.2 mmol) in CH_2Cl_2 (4 mL) at 0 °C. The reaction mixture was stirred at 23 °C for 2 h. The volatile materials were evaporated, and the residue was purified by flash column chromatography (0-20% ethyl acetate in hexanes) to give the product **145** (115.6 mg, 84%) as a colorless liquid.

2-(4-bromophenyl)-4,4,6,6-tetramethyl-1,3,2-dioxaborinane (145)

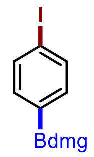

^{1}H NMR (400 MHz, Chloroform-*d*) δ 7.69 (d, *J* = 8.1 Hz, 2H), 7.55 (d, *J* = 8.1 Hz, 2H), 1.91 (s, 2H), 1.42 (s, 12H). **^{13}C NMR** (101 MHz, Chloroform-*d*) δ 136.67, 135.71, 97.75, 71.13, 49.03, 31.91. **^{11}B NMR** (128 MHz, Chloroform-*d*) δ 26.3. **HRMS (ESI)** *m/z* Calcd for $C_{13}H_{18}BINaO_2$ $[M+Na]^+$ 367.0337, Found: 367.0340.

A 10 mL round-bottom flask was charged with **145** (68.8 mg, 0.2 mmol) and THF (2 mL). The flask was cooled to 0 °C, 2.0 M NaOH solution (1.0 mL) and 30% H_2O_2 (1.0 mL) was added dropwise. The mixture was stirred for 2 h at room temperature and water (5 mL) was added to the solution. Then the solution was extracted with ethyl acetate (3 x 5 mL). The organic phase was combined, washed with brine and dried over Na_2SO_4. The crude product obtained after filtration and concentration in vacuo was purified by column chromatography with ethyl acetate/hexane (1:5) as eluents to provide **146** (38.1 mg, 88%) as a colorless liquid.[29]

4-iodophenol (146)

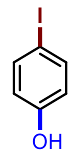

^{1}H NMR (400 MHz, Chloroform-*d*) δ 7.51 (d, *J* = 8.8 Hz, 1H), 6.63 (d, *J* = 8.9 Hz, 1H), 5.11 (s, 1H). **^{13}C NMR** (101 MHz, Chloroform-*d*) δ 155.44, 138.58, 117.94, 82.85.

8. Derivatization of the products from C–H borylation

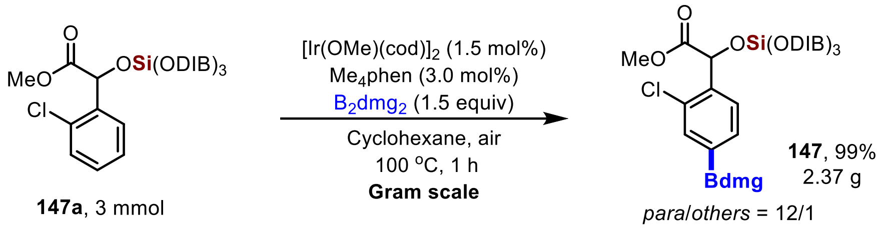

Methyl-2-(2-chlorophenyl)-2-((tris((2,6-dimethylheptan-4-yl)oxy)silyl)oxy)acetate **147a** (3 mmol, 1.96 g), B_2dmg_2 (1.27 g, 4.5 mmol, 1.5 equiv), $[Ir(cod)OMe]_2$ (29.4 mg, 0.045 mmol), Me_4phen (212 mg, 0.09 mmol), and cyclohexane (15 mL) were added to a 50 mL glass vial under air atmosphere. The glass vial was capped with a teflon pressure cap and placed into an aluminum block pre-heated to 100 °C for 1 h. After completion, cyclohexane was removed under reduced pressure and chromatographic separation with silica gel (20% ethyl acetate in hexane as eluent) gave the borylated product **147** (2.37 g, 99%) as colorless liquid. The *para:meta* ratio of products was reported from analysis of ^1H NMR.

Methyl 2-(3-chloro-3'-methoxy-[1,1'-biphenyl]-4-yl)-2-((tris((2,6-dimethylheptan-4-yl)oxy)silyl)oxy)acetate (148)

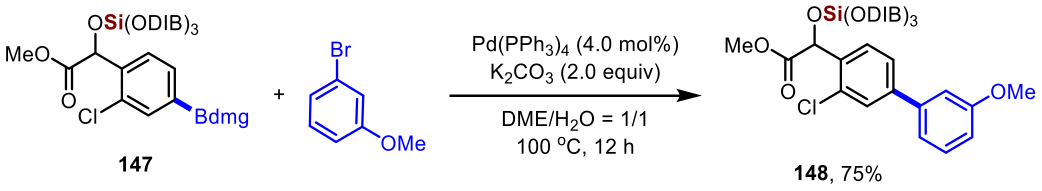

In an argon filled glove box, a 15 mL glass vial charged with **147** (159.3 mg, 0.2 mmol), $Pd(PPh_3)_4$ (9.2 mg, 0.008 mmol), K_2CO_3 (55.2 mg, 0.4 mmol) and 1-bromo-3-methoxybenzene (41.1 mg, 1.1 equiv.). The reaction vial was taken out from glove box, DME (2.0 mL) and water (2.0 mL) was added. The reaction vial was placed in a preheated aluminium block at 100 °C and heated for 12 h. After 12 h, the reaction mixture was cooled to room temperature, diluted with water (5 mL) and extracted with ethyl acetate (5 mL x 3). The combine organic layer was washed with brine (5 mL), dried over anhydrous Na_2SO_4 and concentrated under reduced pressure. Chromatographic purification of crude mass with silica gel (2% ethyl acetate in hexane as eluent) gave 114.3 mg (75%) of the product (**148**) as colorless liquid.[25]

^{1}H NMR (400 MHz, $CDCl_3$) δ 7.68 (d, *J* = 8.1 Hz, 1H), 7.56 (d, *J* = 1.8 Hz, 1H), 7.46 (dd, *J* = 8.1, 1.8 Hz, 1H), 7.35 (t, *J* = 7.9 Hz, 1H), 7.13 (dd, *J* = 7.7, 1.3 Hz, 1H), 7.07 (s, 1H), 6.91 (dd, *J* = 8.2, 2.6 Hz, 1H), 5.94 (s, 1H), 4.14 – 4.05 (m, 3H), 3.85 (s, 3H), 3.69 (s, 3H), 1.83 – 1.63 (m, 6H), 1.41 (ddd, *J* = 30.2, 13.5, 6.5 Hz, 6H), 1.32 – 1.18 (m, 6H), 1.05 – 0.75 (m, 36H). **^{13}C NMR** (101 MHz, $CDCl_3$) δ 171.4, 160.2, 142.5, 141.2, 136.0, 133.2, 130.0, 129.5, 128.0, 125.8, 119.7, 113.4, 113.0, 70.7, 70.5, 55.5, 52.2, 46.7 (d, *J* = 6.4 Hz), 24.5 (d, *J* = 1.8 Hz), 23.1 (d, *J* = 1.4 Hz), 22.8 (d, *J* = 12.3 Hz). **HRMS (ESI)** *m/z* Calcd for $C_{43}H_{72}ClO_7Si$ $[M+H]^+$ 763.4730, Found: 763.4715.

Methyl 2-(2-chloro-4-(pyridin-3-yl)phenyl)-2-((tris((2,6-dimethylheptan-4-yl)oxy)silyl)oxy)acetate (149)

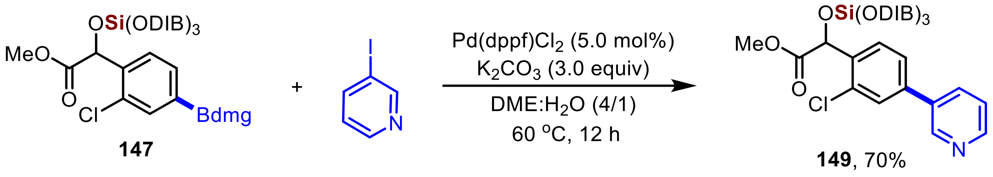

In an argon filled glove box, a 15 mL glass vial charged with **147** (159.3 mg, 0.2 mmol), $Pd(dppf)Cl_2$ (7.3 mg, 0.01 mmol), K_2CO_3 (55.2 mg, 0.4 mmol) and 3-iodopyridine (45.1 mg, 1.1 equiv.). The reaction vial was taken out from glove box, DME (2.0 mL) and water (2.0 mL) was added. The reaction vial was placed in a preheated aluminium block at 100 °C and heated for 12 h. After 12 h, the reaction mixture was cooled to room temperature, diluted with water (5 mL) and extracted with ethyl acetate (5 mL x 3). The combine organic layer was washed with brine (5 mL), dried over anhydrous Na_2SO_4 and concentrated under reduced pressure. Chromatographic purification of crude mass with silica gel (2% ethyl acetate in hexane as eluent) gave 103.4 mg (70%) of the product (**149**) as colorless liquid.[26]

^{1}H NMR (400 MHz, $CDCl_3$) δ 8.81 (d, *J* = 2.3 Hz, 1H), 8.61 (dd, *J* = 4.8, 1.6 Hz, 1H), 7.83 (dt, *J* = 8.0, 1.9 Hz, 1H), 7.73 (d, *J* = 8.1 Hz, 1H), 7.56 (d, *J* = 1.8 Hz, 1H), 7.47 (dd, *J* = 8.1, 1.9 Hz, 1H), 7.37 (ddd, *J* = 7.9, 4.8, 0.8 Hz, 1H), 5.94 (s, 1H), 4.14 – 4.04 (m, 3H), 3.69 (s, 3H), 1.81 – 1.59 (m, 6H), 1.52 – 1.29 (m, 6H), 1.30 – 1.15 (m, 6H), 1.11 – 0.72 (m, 36H). **^{13}C NMR** (101 MHz, $CDCl_3$) δ 171.2, 149.2, 148.3, 139.2, 136.9, 135.2, 134.5, 133.6, 129.8, 128.0, 125.7, 123.8, 70.7, 70.5, 52.3, 46.7 (d, *J* = 6.2 Hz), 24.5 (d, *J* = 1.9 Hz), 23.1 (d, *J* = 1.8 Hz), 22.8 (d, *J* = 11.9 Hz). **HRMS (ESI)** *m/z* Calcd for $C_{41}H_{68}ClNaO_6Si$ $[M+Na]^+$ 756.4397, Found: 756.4415.

Methyl 2-(2-chloro-4-(thiophen-3-yl)phenyl)-2-((tris((2,6-dimethylheptan-4-yl)oxy)silyl)oxy)acetate (150)

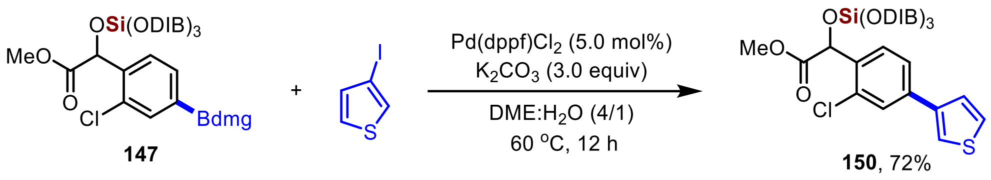

In an argon filled glove box, a 15 mL glass vial charged with **147** (159.3 mg, 0.2 mmol), Pd(dppf)Cl_2 (7.3 mg, 0.01 mmol), K_2CO_3 (55.2 mg, 0.4 mmol) and 3-lodothiophene (46.2 mg, 1.1 equiv.). The reaction vial was taken out from glove box, DME (2.0 mL) and water (2.0 mL) was added. The reaction vial was placed in a preheated aluminium block at 100 °C and heated for 12 h. After 12 h, the reaction mixture was cooled to room temperature, diluted with water (5 mL) and extracted with ethyl acetate (5 mL x 3). The combine organic layer was washed with brine (5 mL), dried over anhydrous Na_2SO_4 and concentrated under reduced pressure. Chromatographic purification of crude mass with silica gel (2% ethyl acetate in hexane as eluent) gave 106.3 mg (72%) of the product (**150**) as colorless liquid.[26]

^{1}H NMR (400 MHz, $CDCl_3$) δ 7.64 (d, J = 8.1 Hz, 1H), 7.57 (d, J = 1.8 Hz, 1H), 7.52 – 7.44 (m, 2H), 7.39 (dd, J = 5.0, 2.9 Hz, 1H), 7.35 (dd, J = 5.0, 1.4 Hz, 1H), 5.91 (s, 1H), 4.15 – 4.04 (m, 3H), 3.69 (s, 3H), 1.85 – 1.62 (m, 6H), 1.50 – 1.33 (m, 6H), 1.31 – 1.16 (m, 6H), 0.93 – 0.76 (m, 36H). **^{13}C NMR** (101 MHz, $CDCl_3$) δ 171.4, 140.8, 137.2, 135.6, 133.3, 129.5, 127.2, 126.7, 126.3, 125.1, 121.3, 70.7, 70.5, 52.2, 46.7 (d, J = 7.3 Hz), 24.5 (d, J = 2.0 Hz), 23.1 (d, J = 2.2 Hz), 22.8 (d, J = 13.4 Hz). **HRMS (ESI)** *m/z* Calcd for $C_{40}H_{67}ClNaO_6SSi$ $[M+Na]^+$ 761.4008, Found: 761.4021.

Methyl 2-(2-chloro-4-((triisopropylsilyl)ethynyl)phenyl)-2-((tris((2,6-dimethylheptan-4-yl)oxy)silyl)oxy)acetate (151)

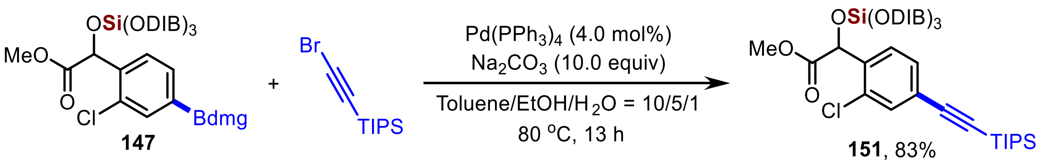

To a mixture of **147** (159.3 mg, 0.2 mmol) and $Pd(PPh_3)_4$ (9.2 mg, 0.008 mmol) under Ar were added a solution of bromo(triisopropylsilyl)acetylene (62.6 mg, 0.24 mmol) in toluene (4 mL), EtOH (2 mL), and 2 M aq. Na_2CO_3 (0.90 mL). The resulting mixture was stirred at 80 °C for 13 h under Ar in the dark. After cooling to room temperature and evaporation of solvents, the residue was partitioned between CH_2Cl_2 and H_2O. The organic layer was washed with

H_2O and brine and dried over Na_2SO_4. After evaporation of solvent, the residue was subjected to column chromatography on silica gel with ethyl acetate/hexane (1:50) as the eluent to give **151** (138.9 mg, 83% yield) as a yellow liquid.[27]

^{1}H NMR (400 MHz, $CDCl_3$) δ 7.55 (d, *J* = 8.1 Hz, 1H), 7.45 (d, *J* = 1.6 Hz, 1H), 7.34 (dd, *J* = 8.0, 1.6 Hz, 1H), 5.87 (s, 1H), 4.12 – 4.04 (m, 3H), 3.66 (s, 3H), 1.81 – 1.60 (m, 6H), 1.51 – 1.30 (m, 6H), 1.28 – 1.18 (m, 6H), 1.12 (d, *J* = 1.5 Hz, 21H), 0.93 – 0.70 (m, 36H). **^{13}C NMR** (101 MHz, $CDCl_3$) δ 171.0, 137.2, 132.7, 132.6, 130.5, 128.9, 124.9, 105.5, 92.6, 70.64, 70.5, 52.2, 46.7 (d, *J* = 6.7 Hz), 24.5 (d, *J* = 1.4 Hz), 23.1, 22.8 (d, *J* = 12.8 Hz), 18.8, 11.4. **HRMS (ESI)** *m/z* Calcd for $C_{47}H_{85}ClNaO_6Si_2$ $[M+Na]^+$ 859.5465, Found: 859.5423.

Methyl 2-(4-benzyl-2-chlorophenyl)-2-((tris((2,6-dimethylheptan-4-yl)oxy)silyl)oxy)acetate (152)

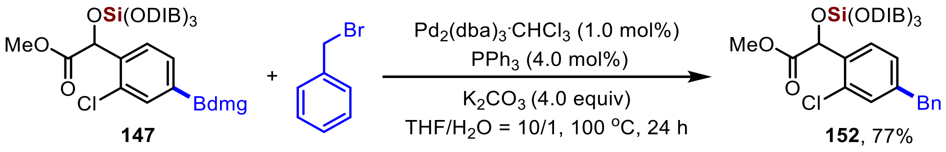

In an argon filled glove box, a 15 mL glass tube was charged with **147** (159.3 mg, 0.2 mmol), $Pd_2(dba)_3$•$CHCl_3$ (2.0 mg, 1.0 mol%), PPh_3 (2.1 mg, 4.0 mol%), K_2CO_3 (110.4 mg, 4.0 equiv) and benzyl bromide (40.2 mg, 1.2 equiv) and THF (1.0 mL). The reaction vial was taken out from glove box and water (100 µL) was added. The pressure tubewas placed in a preheated aluminium block at 100 °C and heated for 24 h. After 24 h, the reaction mixture was cooled to room temperature, diluted with water (5 mL) and extracted with ethyl acetate (5 mL x 3). The combine organic layer was washed with brine (15 mL), dried over anhydrous Na_2SO_4 and concentrated under reduced pressure. Chromatographic purification of crude mass with silica gel (5% ethyl acetate in hexane as eluent) gave **152** (115.0 mg, 77%) as colorless liquid.[28]

^{1}H NMR (400 MHz, $CDCl_3$) δ 7.54 (d, *J* = 8.0 Hz, 1H), 7.33 – 7.27 (m, 2H), 7.17 (dd, *J* = 7.8, 1.6 Hz, 1H), 7.08 (dd, *J* = 8.0, 1.8 Hz, 3H), 5.87 (s, 1H), 4.12 – 4.04 (m, 3H), 3.93 (s, 2H), 3.66 (s, 3H), 1.87 – 1.60 (m, 6H), 1.49 – 1.31 (m, 6H), 1.29 – 1.15 (m, 6H), 0.95 – 0.64 (m, 36H). **^{13}C NMR** (101 MHz, $CDCl_3$) δ 171.5, 142.8, 140.2, 134.8, 132.8, 129.7, 129.2, 129.1, 128.7, 127.6, 126.5, 70.6, 70.4, 52.1, 46.6 (d, *J* = 7.1 Hz), 41.4, 24.5 (d, *J* = 2.3 Hz), 23.1, 22.8 (d, *J* = 13.6 Hz). **HRMS (ESI)** *m/z* Calcd for $C_{43}H_{71}ClNaO_6Si$ $[M+Na]^+$ 769.4601, Found: 769.4638.

Methyl 2-(2-chloro-4-hydroxyphenyl)-2-((tris((2,6-dimethylheptan-4-yl)oxy)silyl)oxy)acetate (153)

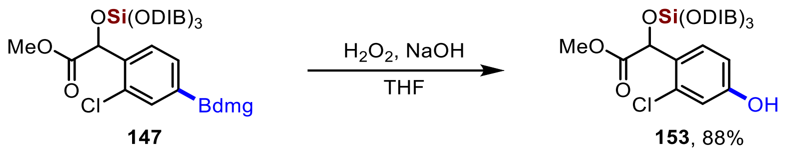

A 10 mL round-bottom flask was charged with **147** (159.3 mg, 0.2 mmol) and THF (2 mL). The flask was cooled to 0 °C, 2.0 M NaOH solution (1.0 mL) and 30% H_2O_2 (1.0 mL) was added dropwise. The mixture was stirred for 2 h at room temperature and water (5 mL) was added to the solution. Then the solution was extracted with ethyl acetate (3 x 5 mL). The organic phase was combined, washed with brine and dried over Na_2SO_4. The crude product obtained after filtration and concentration in vacuo was purified by column chromatography with ethyl acetate/hexane (1:10) as eluents to provide **153** (118.3 mg, 88%) as a colorless liquid.[29]

^{1}H NMR (400 MHz, $CDCl_3$) δ 7.41 (d, *J* = 8.5 Hz, 1H), 6.77 (d, *J* = 2.5 Hz, 1H), 6.64 (dd, *J* = 8.6, 2.5 Hz, 1H), 6.20 (d, *J* = 14.4 Hz, 1H), 4.12 – 4.04 (m, 3H), 3.69 (s, 3H), 1.82 – 1.61 (m, 6H), 1.48 – 1.33 (m, 6H), 1.30 – 1.14 (m, 6H), 0.95 – 0.75 (m, 36H). **^{13}C NMR** (101 MHz, $CDCl_3$) δ 172.7, 156.6, 133.3, 128.6, 116.3, 114.6, 70.5, 52.3, 46.6 (d, *J* = 7.4 Hz), 24.5 (d, *J* = 1.5 Hz), 23.1, 22.8 (d, *J* = 11.4 Hz). **HRMS (ESI)** *m/z* Calcd for $C_{36}H_{65}ClNaO_7Si$ $[M+Na]^+$ 695.4080, Found: 695.4108.

Methyl 2-(2-chloro-4-(phenylamino)phenyl)-2-((tris((2,6-dimethylheptan-4-yl)oxy)silyl)oxy)acetate (154)

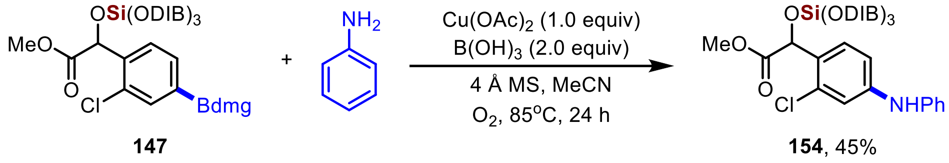

To an oven dried screw-cap 15 mL glass tube were added **147** (159.3 mg, 0.2 mmol), aniline (37.2 mg, 0.40 mmol), $Cu(OAc)_2$ (36.4 mg, 0.20 mmol), $B(OH)_3$ (24.8 mg, 0.40 mmol), and molecular sieves 4Å (powder, 80 mg), and dry MeCN (2.0 mL) under N_2. The nitrogen in the reaction tube was replaced with O_2 (1 atm, closed) and capped tightly. The mixture was stirred at 85 °C for 24 h. After cooling to room temperature, the reaction mixture was diluted with EtOAc, then filtered through a celite pad. The resulting filtrate was concentrated under reduced pressure, and the residue was purified by silica gel column chromatography (eluent: ethyl acetate/hexane = 50/1) to afford **154** (67.4 mg, 45%) as a yellow liquid.[30]

^{1}**H NMR** (400 MHz, CDCl$_3$) δ 7.39 (d, *J* = 8.5 Hz, 1H), 7.26 – 7.16 (m, 2H), 7.04 – 6.96 (m, 2H), 6.94 – 6.89 (m, 2H), 6.82 (dd, *J* = 8.5, 2.4 Hz, 1H), 5.75 (s, 1H), 5.67 (s, 1H), 4.07 – 3.92 (m, 3H), 3.60 (s, 3H), 1.73 – 1.56 (m, 6H), 1.43 – 1.26 (m, 6H), 1.22 – 1.08 (m, 6H), 0.84 – 0.71 (m, 36H). ^{13}**C NMR** (101 MHz, CDCl$_3$) δ 171.8, 144.5, 142.0, 133.7, 130.0, 129.6, 128.7, 122.3, 119.2, 116.71, 115.7, 70.5, 70.4, 52.1, 46.7 (d, *J* = 7.2 Hz), 24.5, 23.2 (d, *J* = 2.8 Hz), 22.8 (d, *J* = 10.8 Hz). **HRMS (ESI)** *m/z* Calcd for $C_{42}H_{70}ClNNaO_6Si$ $[M+Na]^+$ 770.4553, Found: 770.4568.

Methyl 2-(2-chlorophenyl-4-*d*)-2-((tris((2,6-dimethylheptan-4-yl)oxy)silyl)oxy)acetate (155)

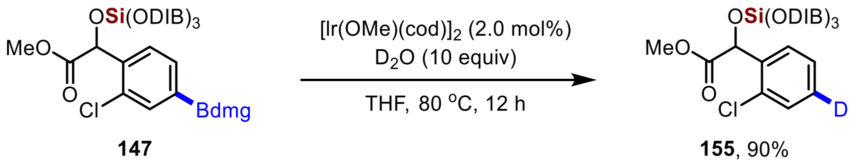

To an oven dried screw-cap 15 mL glass tube were added **147** (159.3 mg, 0.2 mmol), $[Ir(OMe)(cod)]_2$ (4.0 mg, 0.004 mmol), and D_2O (40.4 mg, 2.0 mmol) and dry THF (0.5 mL) under N_2. The mixture was stirred at 80 °C for 12 h. After cooling to room temperature, all volatiles were removed under vacuum. The residue was purified by silica gel column chromatography (eluent: ethyl acetate/hexane = 100/1) to afford **155** (118.3 mg, 90%) as a pale yellow oil.[31]

^{1}**H NMR** (400 MHz, CDCl$_3$) δ 7.66 (d, *J* = 7.8 Hz, 1H), 7.36 (d, *J* = 1.2 Hz, 1H), 7.30 – 7.24 (m, 1H), 5.94 (s, 1H), 4.17 – 4.06 (m, 3H), 3.69 (s, 3H), 1.84 – 1.63 (m, 6H), 1.52 – 1.34 (m, 6H), 1.31 – 1.18 (m, 6H), 1.01 – 0.79 (m, 36H). ^{13}**C NMR** (101 MHz, CDCl$_3$) δ 171.4, 137.1, 132.9, 129.3, 129.2, 126.8, 70.8, 70.5, 52.1, 46.7 (d, *J* = 7.9 Hz), 24.5 (d, *J* = 2.3 Hz), 23.1, 22.8 (d, *J* = 14.9 Hz). **HRMS (ESI)** *m/z* Calcd for $C_{36}H_{65}DClO_6Si$ $[M+H]^+$ 658.4374, Found: 658.4360.

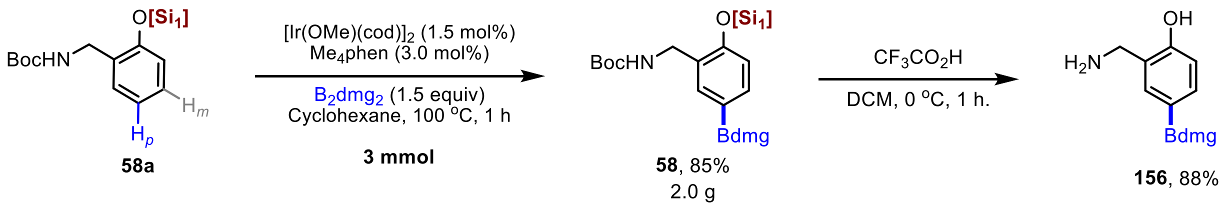

Methyl-2-(2-chlorophenyl)-2-((tris((2,6-dimethylheptan-4-yl)oxy)silyl)oxy)acetate **58a** (3 mmol, 2.0 g), B_2dmg_2 (1.27 g, 4.5 mmol, 1.5 equiv), $[Ir(cod)OMe]_2$ (29.4 mg, 0.045 mmol), Me_4phen (212 mg, 0.09 mmol), and cyclohexane (15 mL) were added to a 50 mL glass vial under air atmosphere. The glass vial was capped with a teflon pressure cap and placed into an aluminum block pre-heated to 100 ºC for 1 h. After completion, cyclohexane was removed under reduced pressure and chromatographic separation with silica gel (20% ethyl acetate in hexane as

eluent) gave the borylated product **58** (2.0 g, 85%) as colorless liquid. The *para:meta* ratio of products was reported from analysis of ^{1}H NMR.

2-(aminomethyl)-4-(4,4,6,6-tetramethyl-1,3,2-dioxaborinan-2-yl)phenol (156)

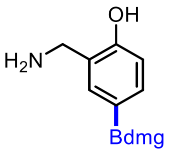

To a solution of **58** (269.7 mg, 0.2 mmol) in CH_2Cl_2 (1 mL) was added a solution of CF_3CO_2H (100 µL) in CH_2Cl_2 (1 mL) at 0 °C. The reaction mixture was stirred at 23 °C for 1 h. Add saturated Na_2CO_3 aqueous solution (3 mL) after evaporating volatile substances. Then the solution was extracted with DCM (3 x 5 mL). The resulting filtrate was concentrated under reduced pressure, and the residue was purified by silica gel column chromatography (eluent: DCM/MeOH = 1/1) to afford **156** (67.4 mg, 85%) as a yellow liquid.

^{1}H NMR (400 MHz, Chloroform-*d*) δ 7.66 (dd, *J* = 8.1, 1.6 Hz, 1H), 7.46 (s, 1H), 6.83 (d, *J* = 8.0 Hz, 1H), 4.14 (s, 2H), 1.88 (s, 2H), 1.74 (s, 2H), 1.40 (s, 12H). **^{13}C NMR** (101 MHz, Chloroform-*d*) δ 160.8, 135.3, 135.0, 133.9, 133.7, 116.2, 70.7, 49.2, 45.6, 32.0. **HRMS (ESI)** *m/z* Calcd for $C_{14}H_{22}BNNaO_3$ $[M+H]^+$ 286.1585, Found: 286.1590.

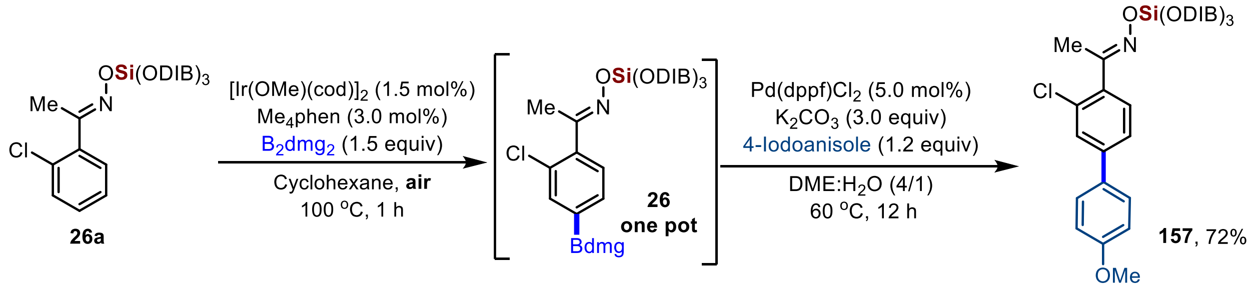

(E)-1-(2-chlorophenyl)ethan-1-one O-(tris((2,6-dimethylheptan-4-yl)oxy)silyl) oxime **26a** (1.0 mmol, 625.4 mg), B_2dmg_2 (423.0 mg, 1.5 mmol, 1.5 equiv), $[Ir(cod)OMe]_2$ (10.0 mg, 0.015 mmol), Me_4phen (6.0 mg, 0.030 mmol), and cyclohexane (5.0 mL) were added to a 30 mL glass vial under air atmosphere. The glass vial was capped with a teflon pressure cap and placed into an aluminum block pre-heated to 100 ºC for 1 h. The reaction was monitored by^{1}HNMR and after 1 hour the starting material was consumed completely. The residue was dissolved in DME (3 mL), and the solution was transferred to a 20 mL air free flask containing 36.6 mg $Pd(dppf)Cl_2$ (0.05 mmol, 5.0 mol%), 258 mg 4-iodomethoxybenzene (1.1 mmol, 1.1 equiv.) and 410 mg K_2CO_3 (3.0 mmol, 3.0 equiv.). Additional 5 mL DME and 2 mL water were added. The mixture was stirred at 60 ℃ overnight. The reaction was monitored by TLC and extracted with ethyl acetate, the organic layer washed with brine, dried over Na_2SO_4 and concentrated in

vacuo. Flash chromatography on silica gel (hexane/ethyl acetate = 20:1) gave the title compound **157** as colorless oil (527.4 mg, 72%).[32]

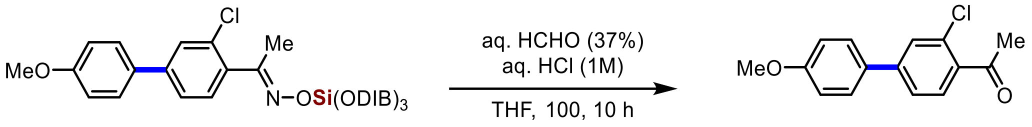

157 **158**, 70%

Compound **157** (293.0 mg, 0.4 mmol) was dissolved in 4 mL THF and 2 mL 37% aqueous formaldehyde before dropwise addition of 2 mL aq. HCl (1M). The resulting mixture was stirred vigorously at 100 ℃ for 10 hours until full consumption of **157** monitored by TLC. The reaction was then diluted with 5 mL ethyl acetate and neutralized with saturated aqueous Na_2CO_3. The aqueous phase was extracted with ethyl acetate (3 x 5 mL). The combined organic phases were washed with 10 mL water and 10 mL brine, dried over $MgSO_4$, filtered, evaporated, and purified by chromatography column on silica gel to give **158** as white solid (72.8 mg, 70%).[15]

1-(3-chloro-4'-methoxy-[1,1'-biphenyl]-4-yl)ethan-1-one (158)

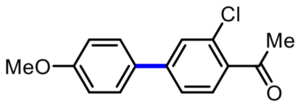

^{1}H NMR (400 MHz, $CDCl_3$) δ 7.66 (d, J = 8.1 Hz, 1H), 7.61 (d, J = 1.8 Hz, 1H), 7.57 – 7.45 (m, 3H), 7.04 – 6.95 (m, 2H), 3.86 (s, 3H), 2.68 (s, 3H). **^{13}C NMR** (101 MHz, $CDCl_3$) δ 199.8, 160.3, 145.1, 136.5, 132.4, 131.1, 130.5, 128.8, 128.4, 125.0, 114.6, 55.5, 30.9. **HRMS (ESI)** m/z Calcd for $C_{15}H_{13}ClNaO_2$ $[M+Na]^+$ 283.0496, Found: 283.0509.

9. Computational method

All the DFT calculations were carried out in ORCA 5.0.2 program package.[33] Molecular geometries were optimized using B3LYP functional,[34] combined with D3 version of Grimme's dispersion in zero-damping scheme.[35] The def2-SVP basis set[36] was used for all atoms during geometry optimization. RIJCOSX approximations[37] with the def2/J auxiliary basis sets[38] was used for accelerating the calculations. The 3D geometry structure was prepared using CYLview20 program,[39] the topographic steric maps was calculated via SambVca 2.1 tool.[40]

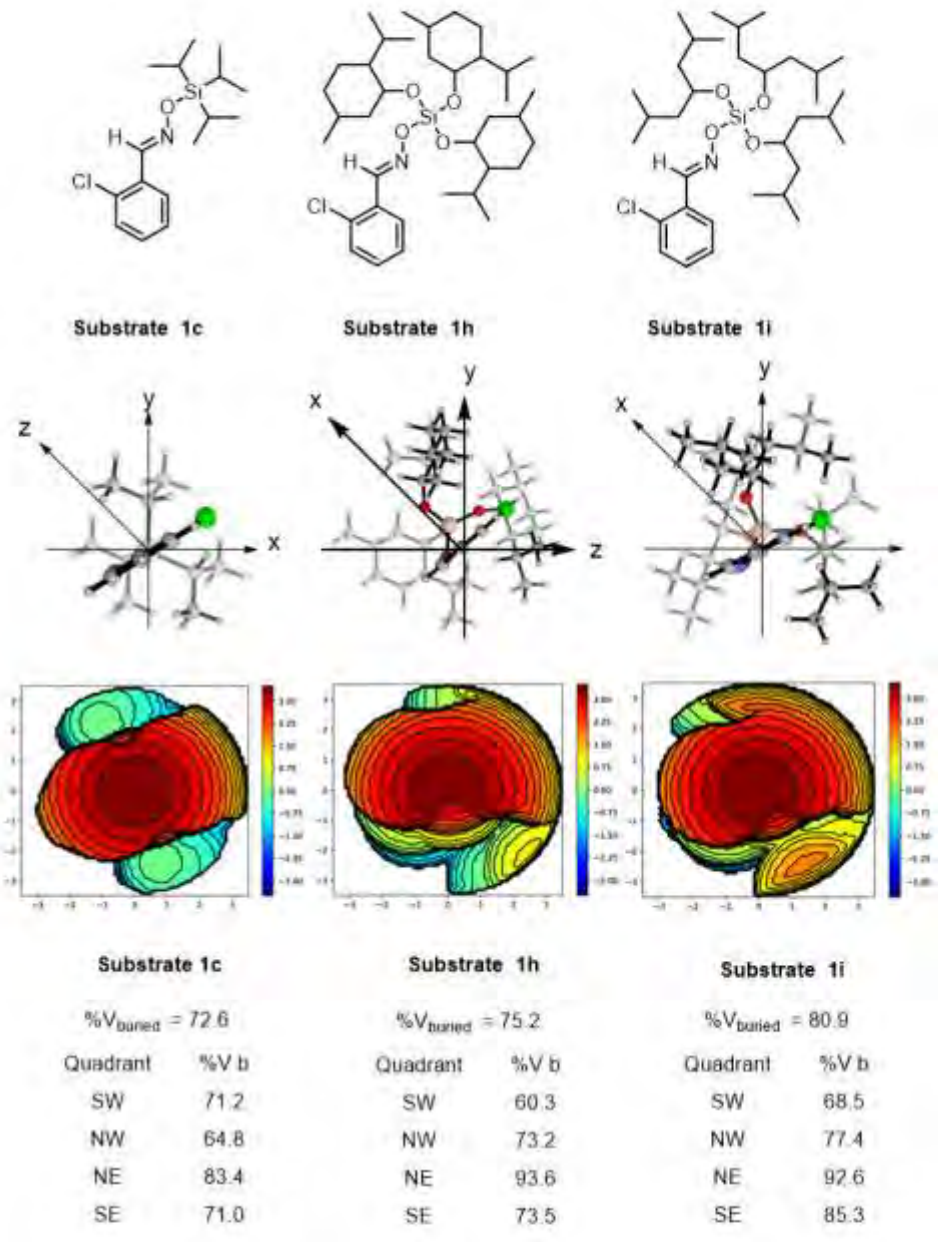

Supplementary Fig. 34: Topographic steric maps of three substrate

10. NMR spectra

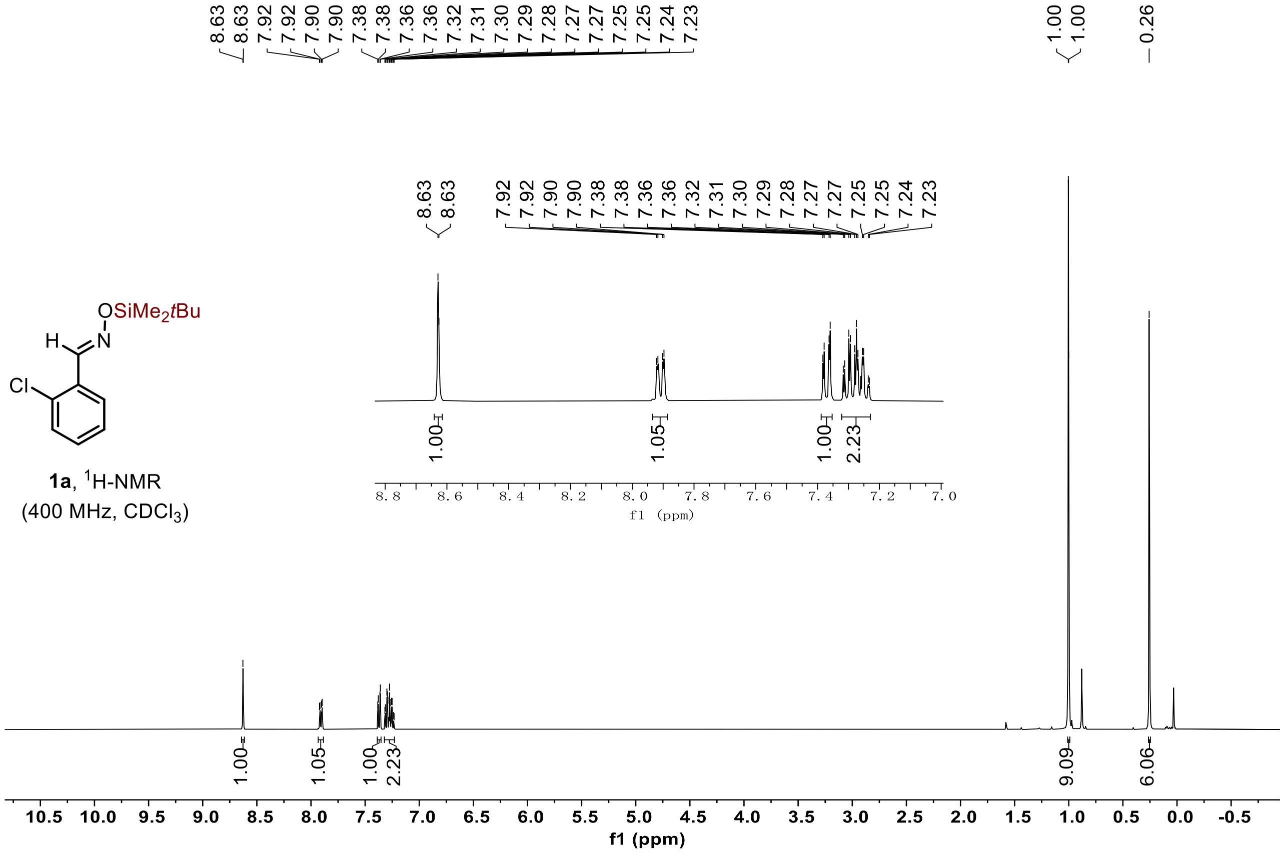

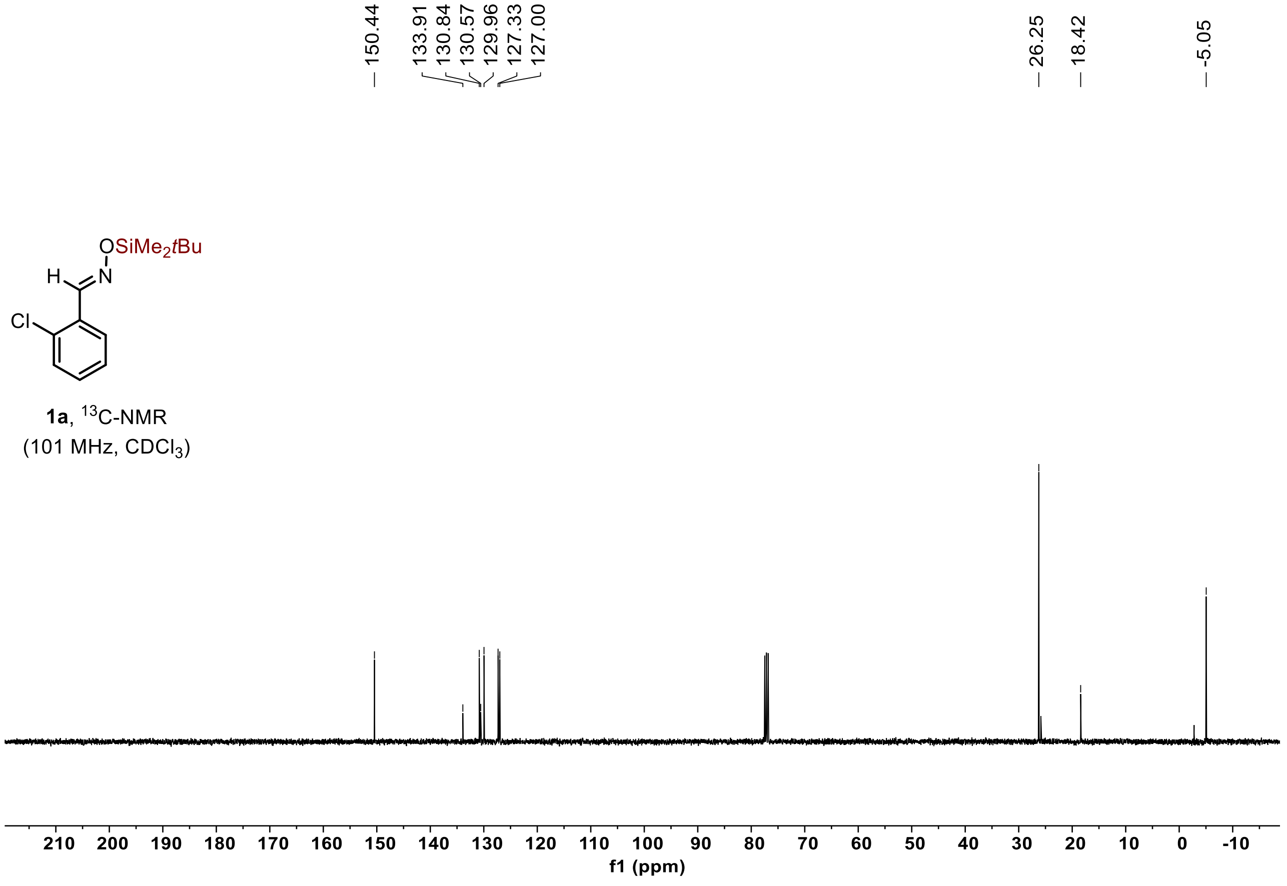

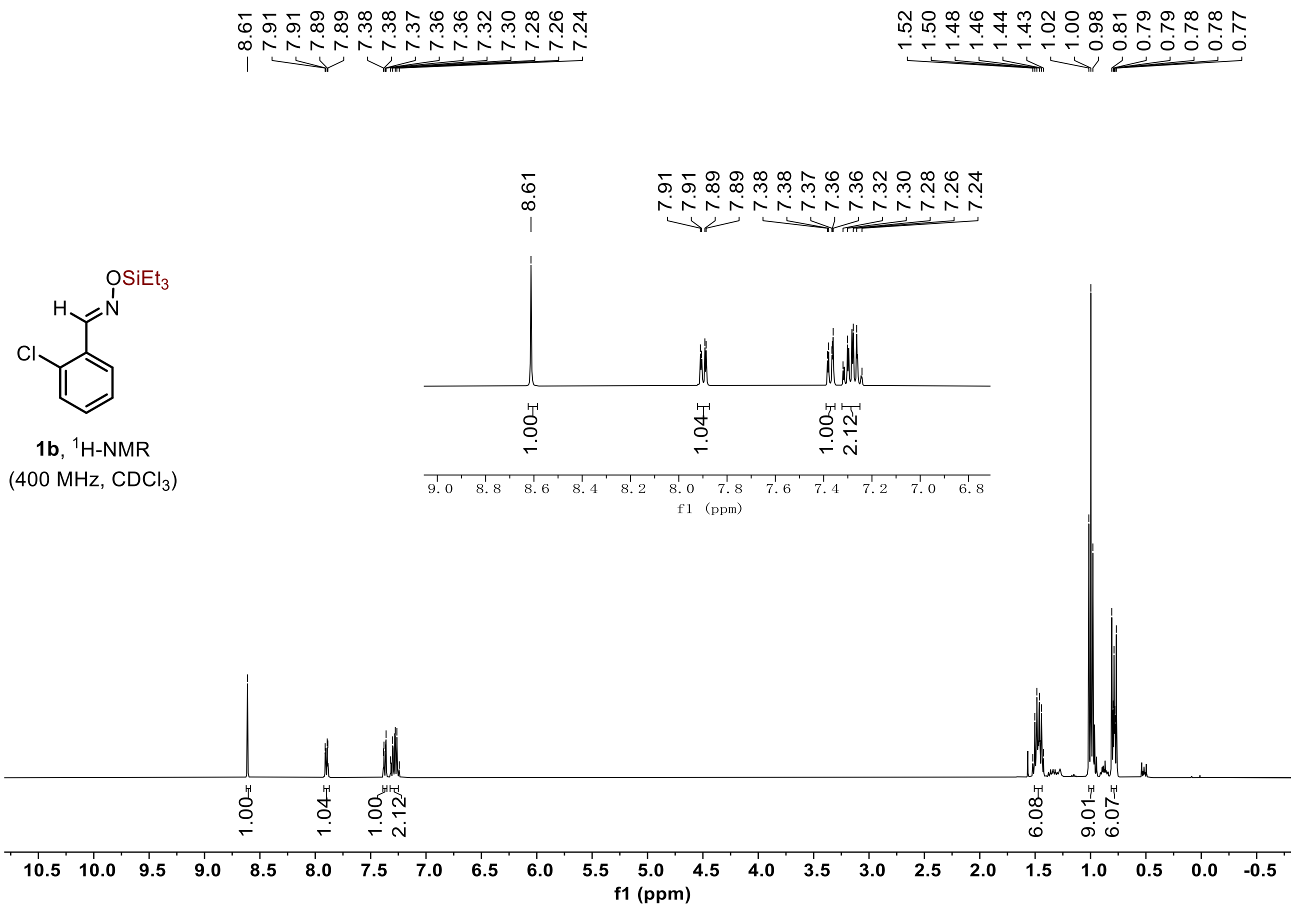

1b, ^{1}H-NMR
(400 MHz, $CDCl_3$)

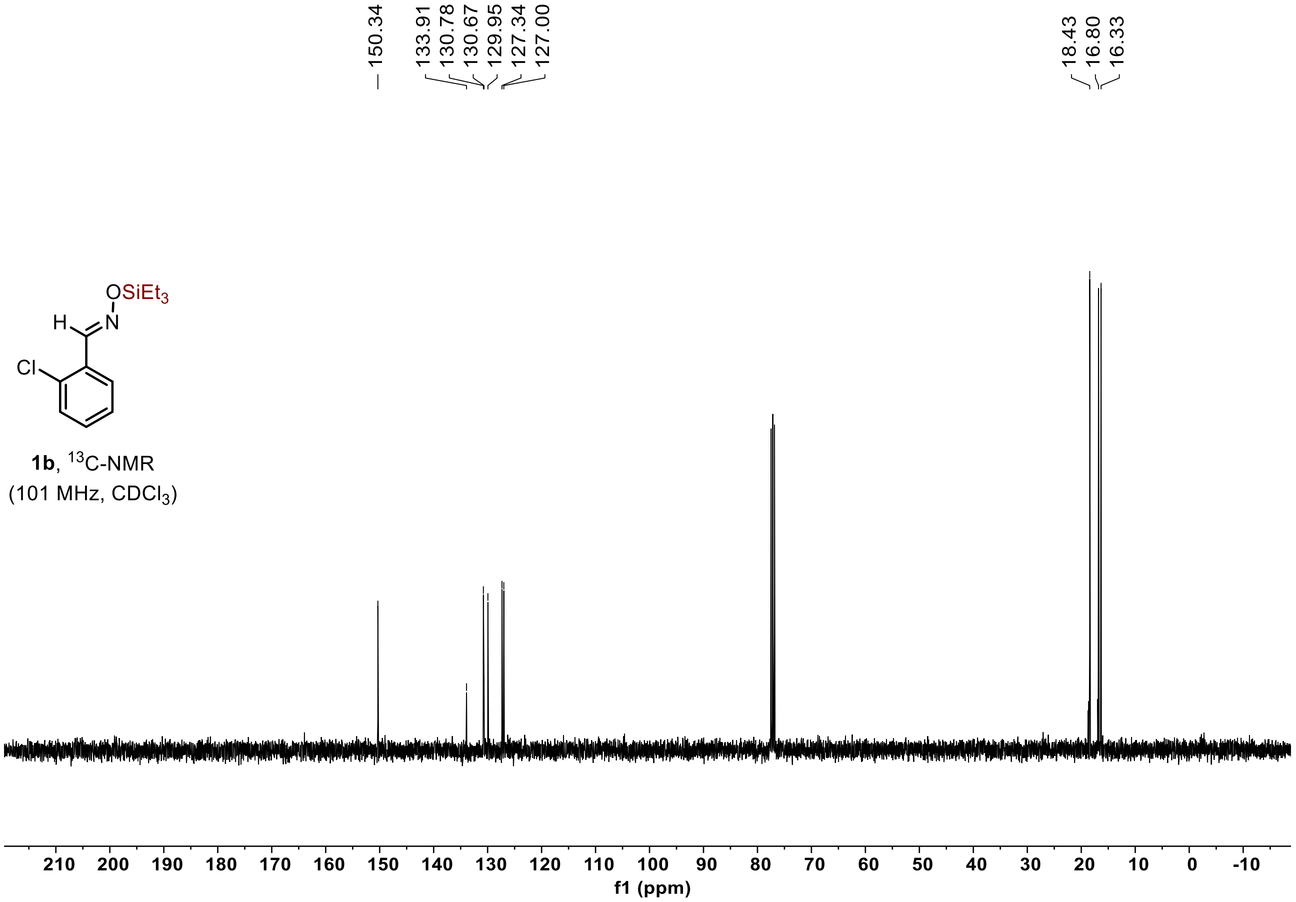

1b, ^{13}C-NMR
(101 MHz, $CDCl_3$)

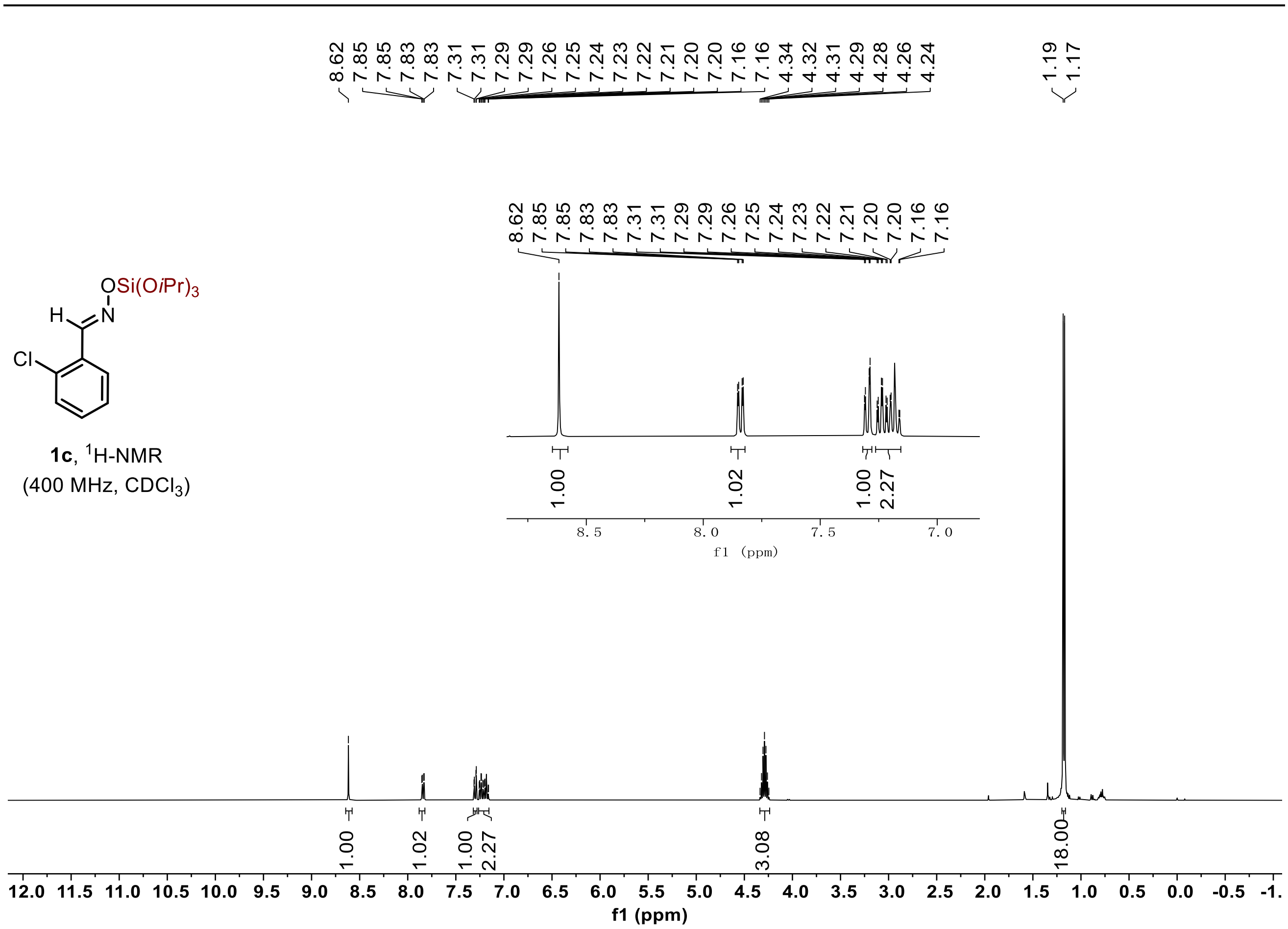

1c, ^{1}H-NMR
(400 MHz, $CDCl_3$)

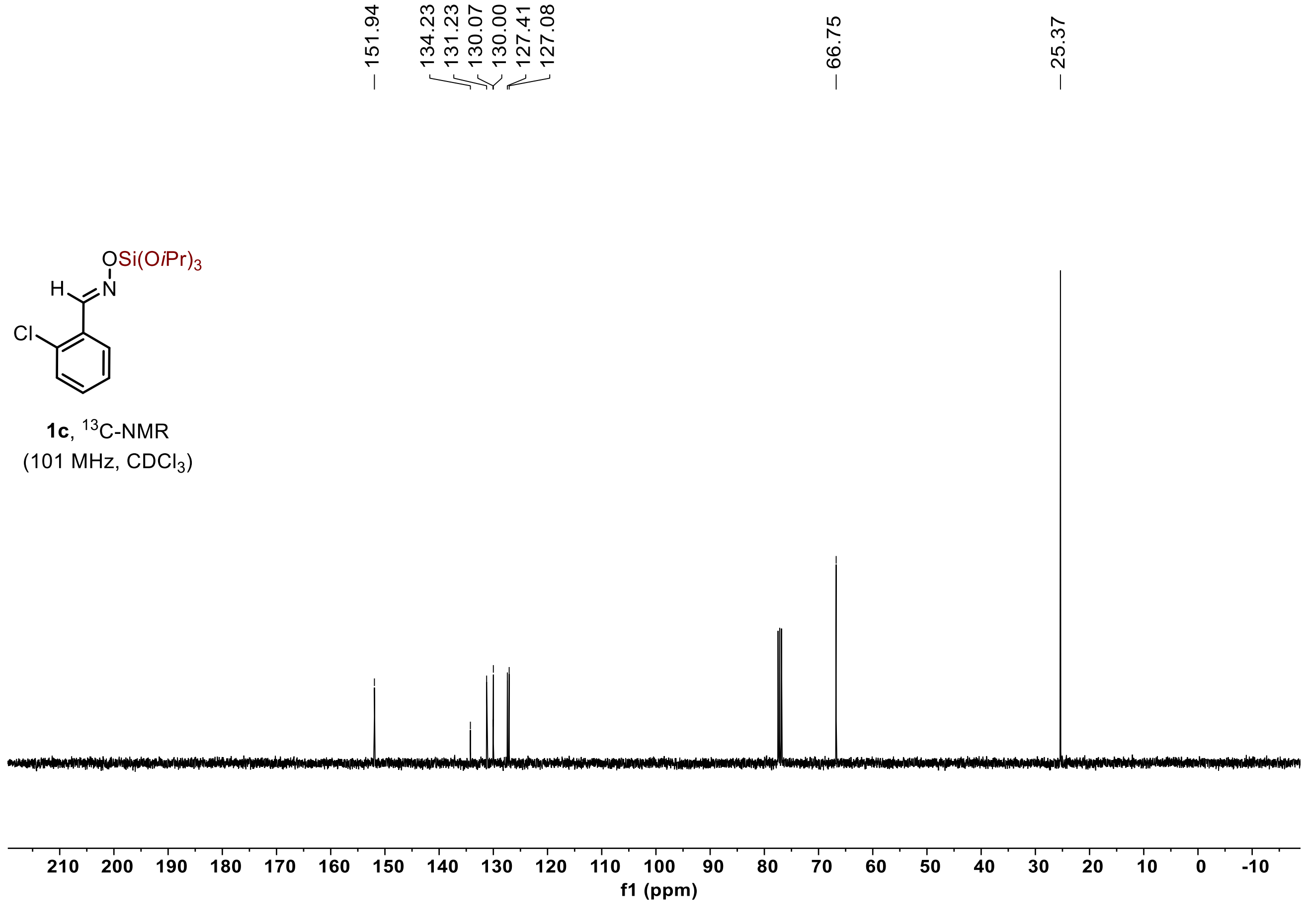

1c, ^{13}C-NMR
(101 MHz, $CDCl_3$)

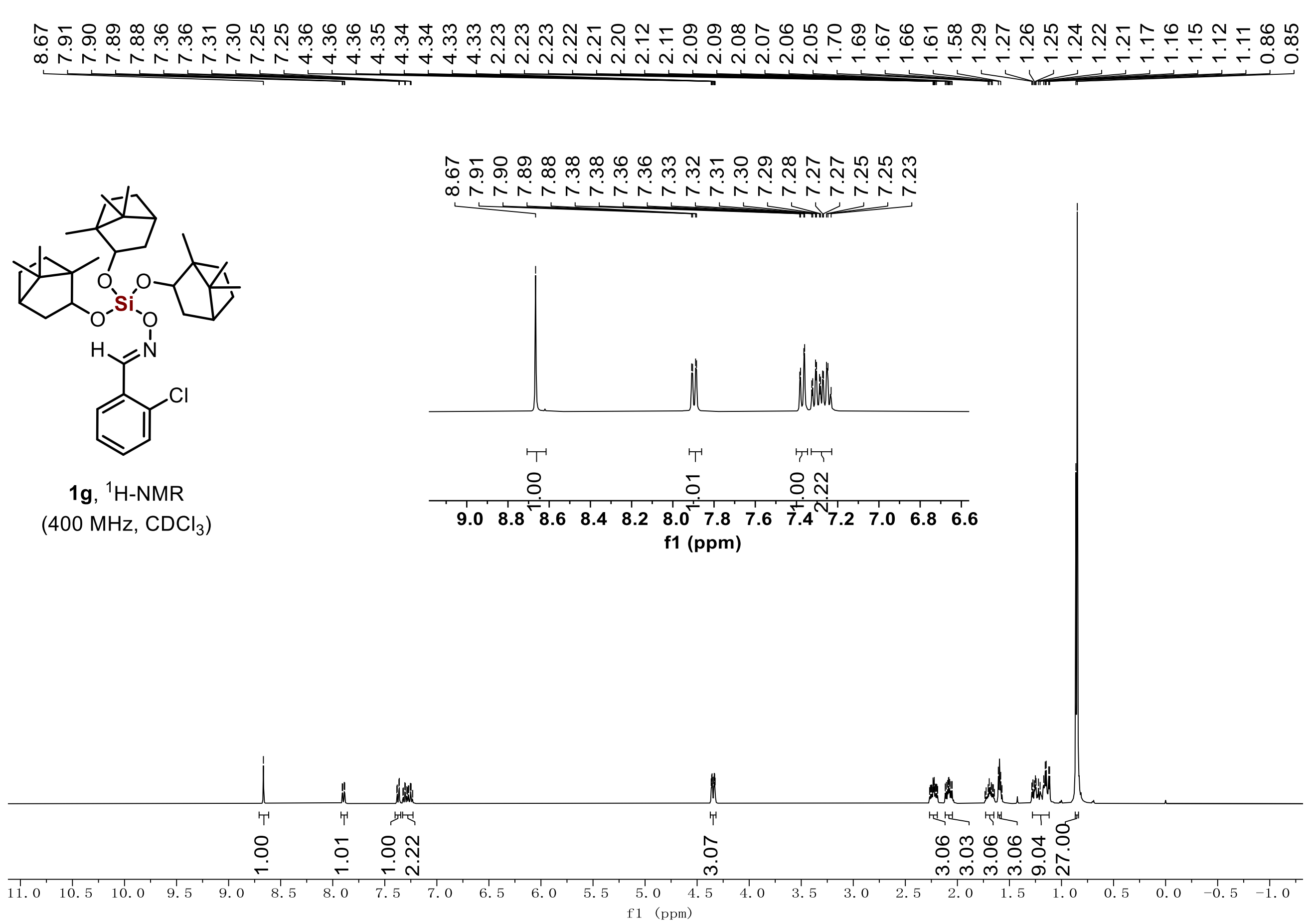

1g, ^{1}H-NMR
(400 MHz, $CDCl_3$)

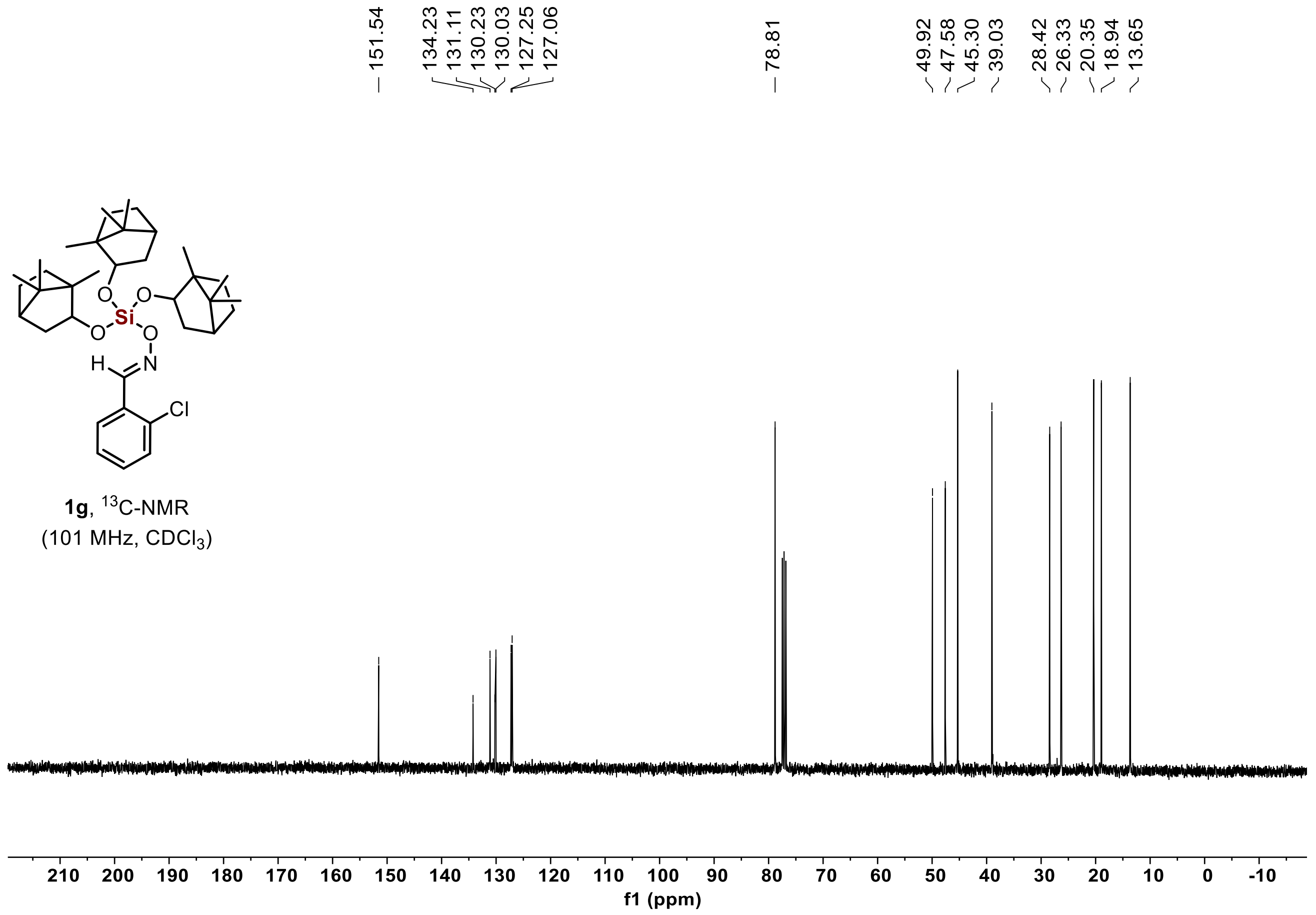

1g, ^{13}C-NMR
(101 MHz, $CDCl_3$)

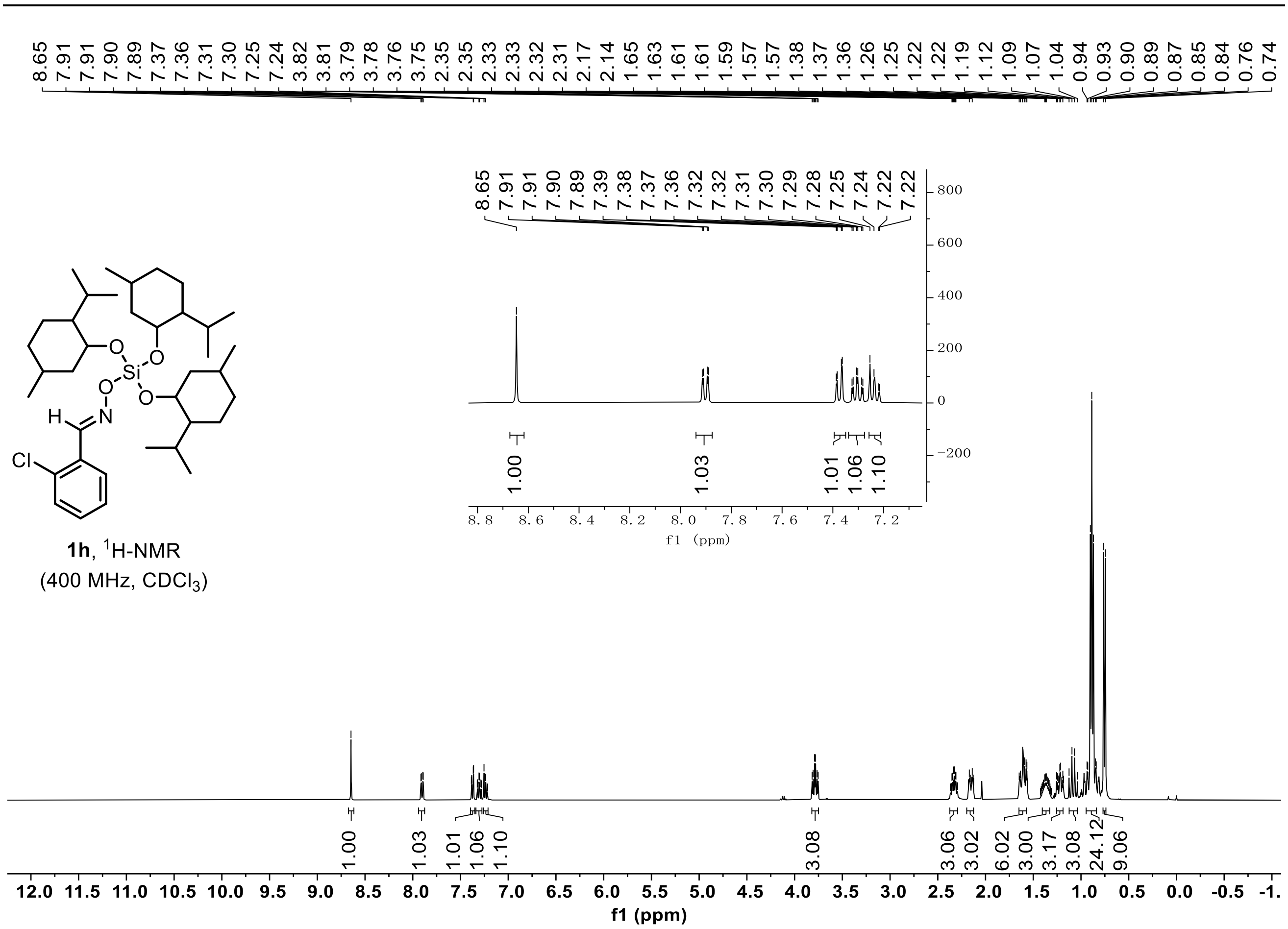

1h, ^{1}H-NMR
(400 MHz, $CDCl_3$)

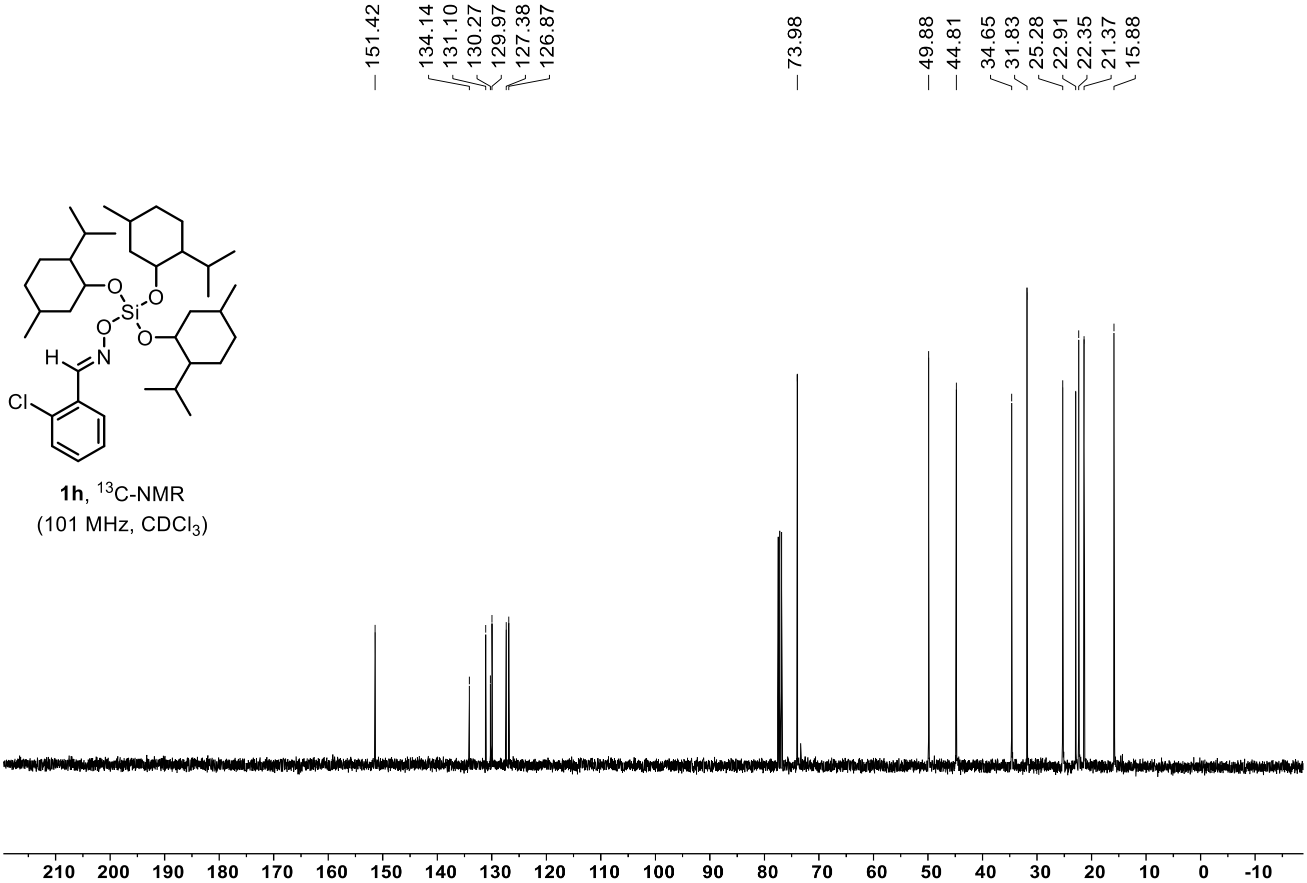

1h, ^{13}C-NMR
(101 MHz, $CDCl_3$)

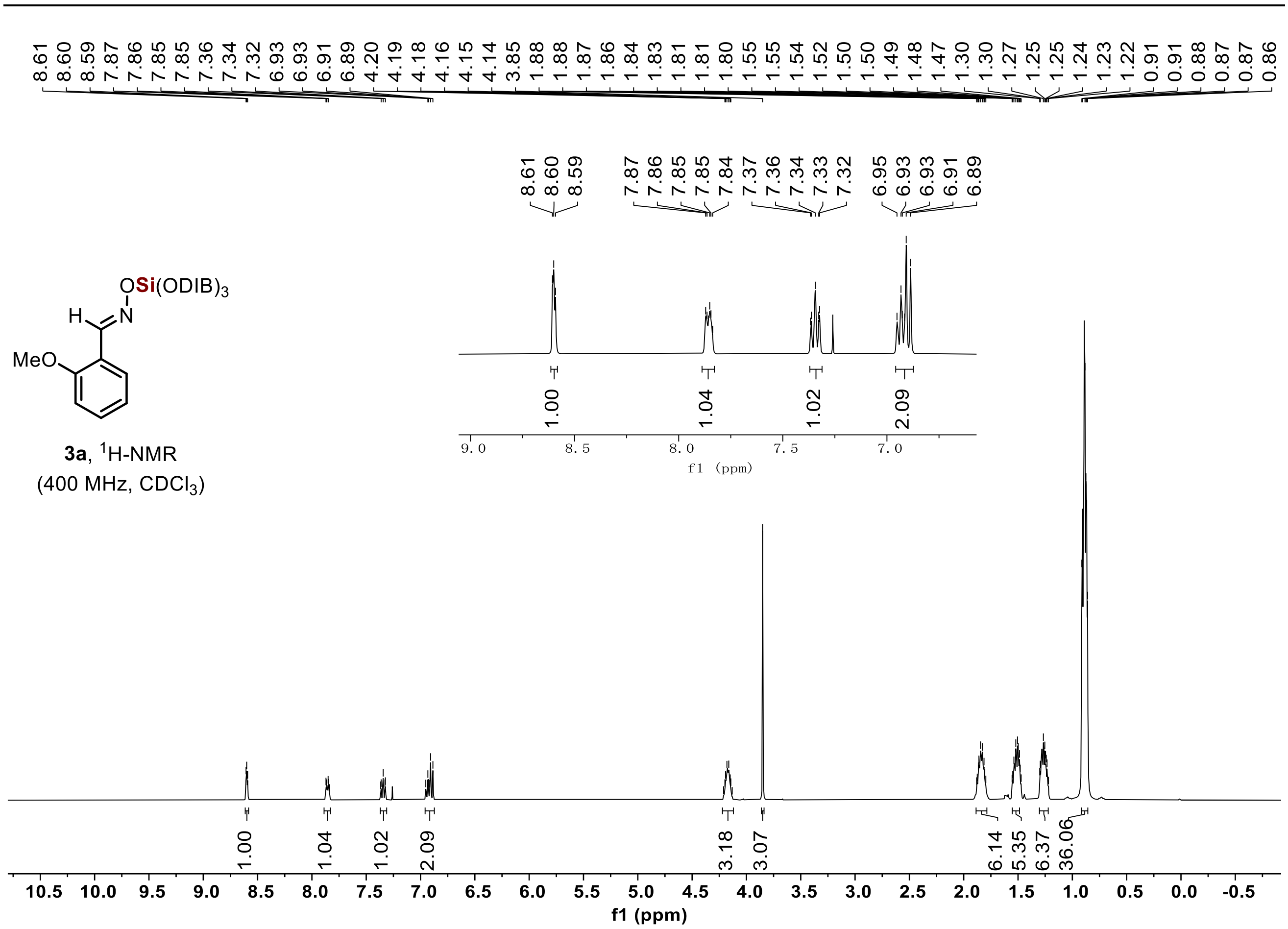

3a, ^{1}H-NMR
(400 MHz, $CDCl_3$)

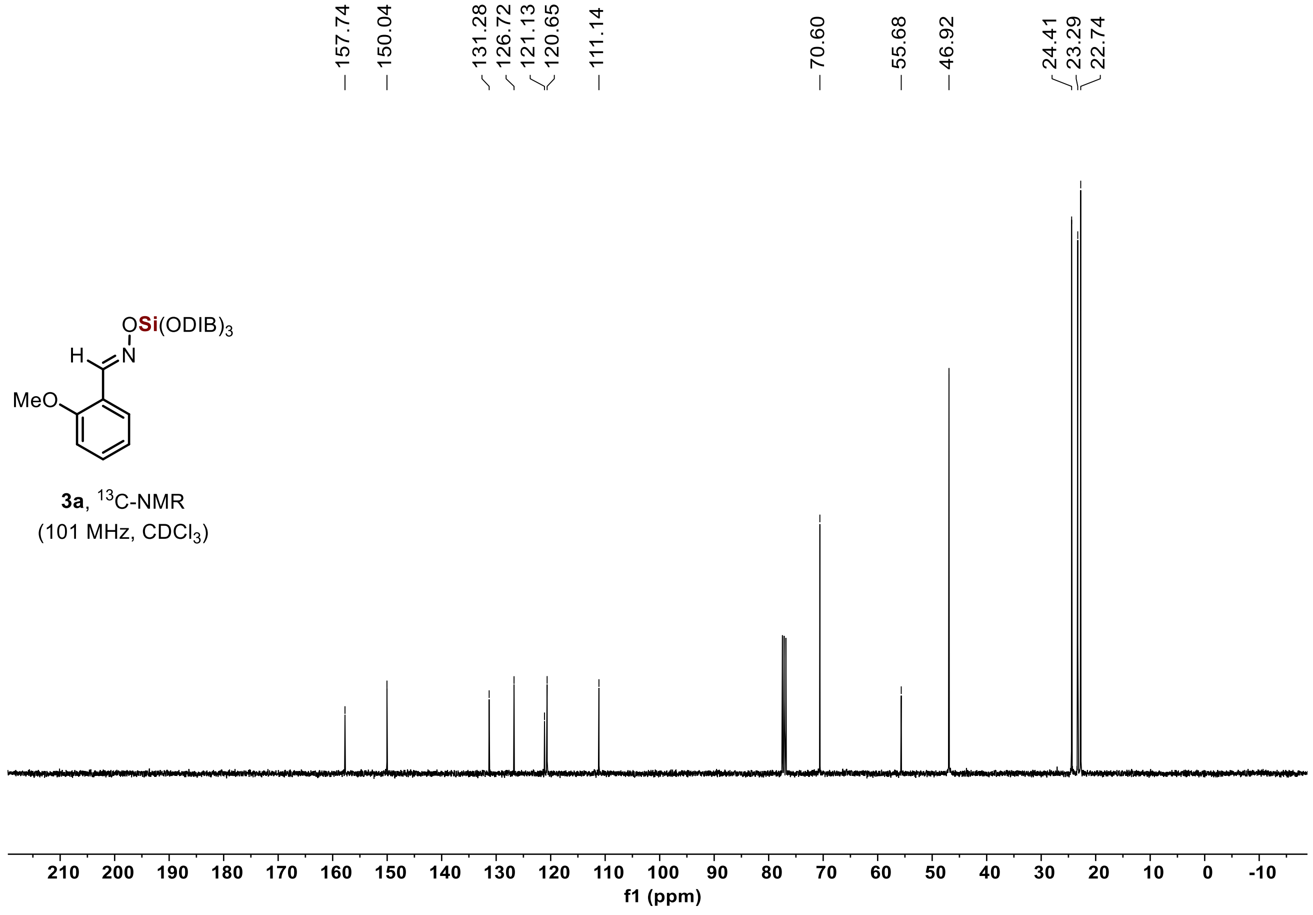

3a, ^{13}C-NMR
(101 MHz, $CDCl_3$)

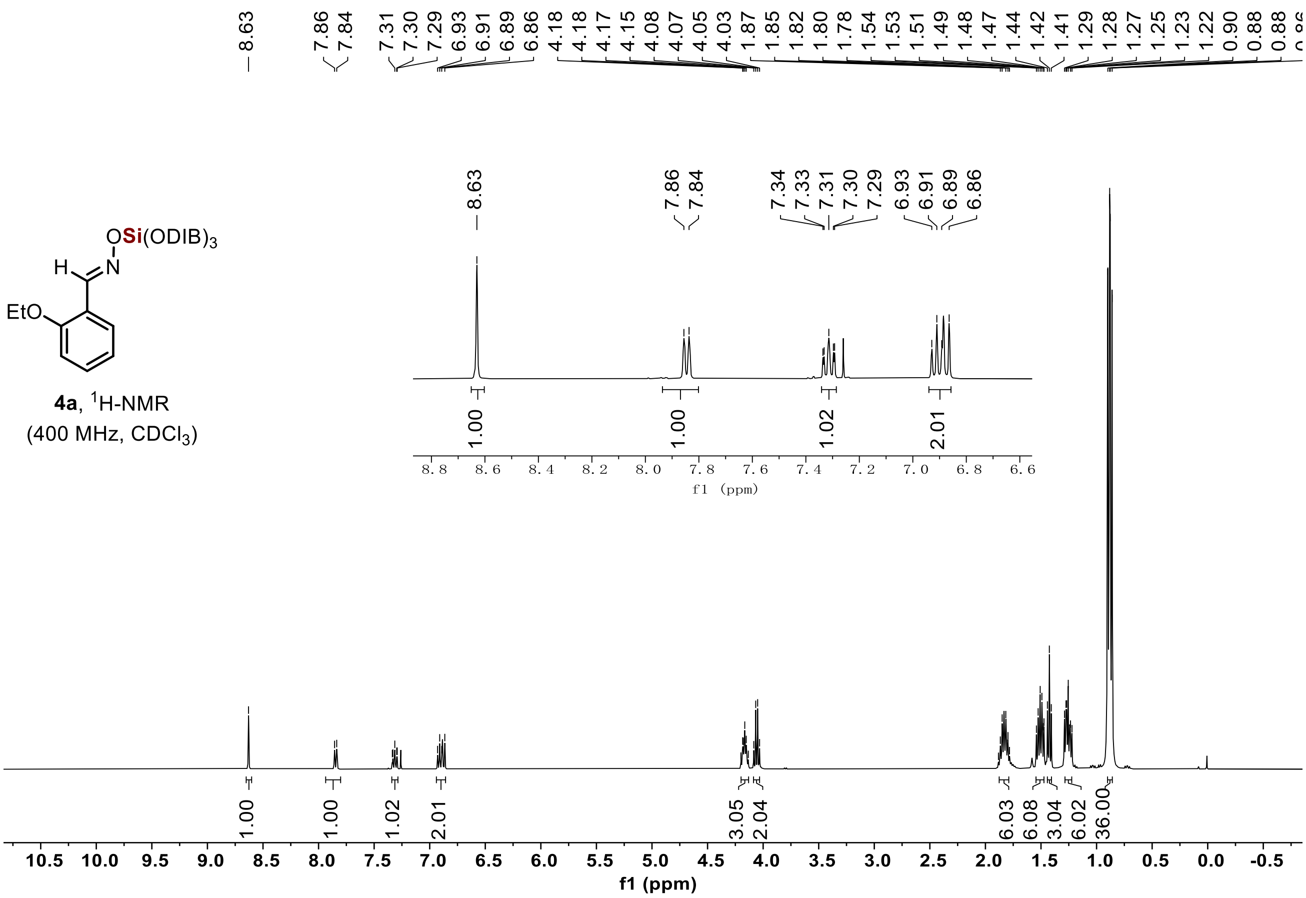
OSi(ODIB)3
H
N
EtO
4a, 1H-NMR
(400 MHz, CDCl3)
f1 (ppm)

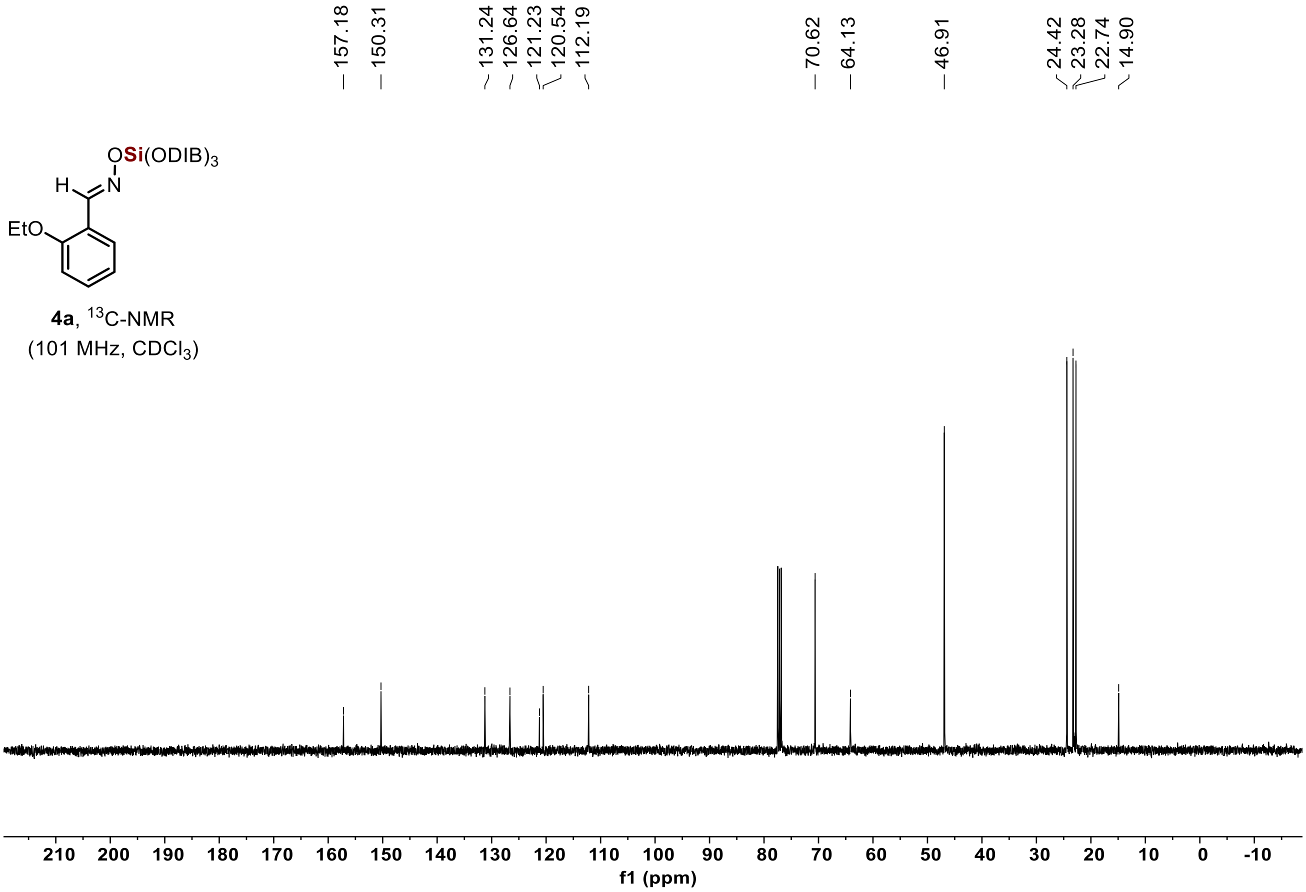
OSi(ODIB)3
H
N
EtO
4a, 13C-NMR
(101 MHz, CDCl3)
f1 (ppm)

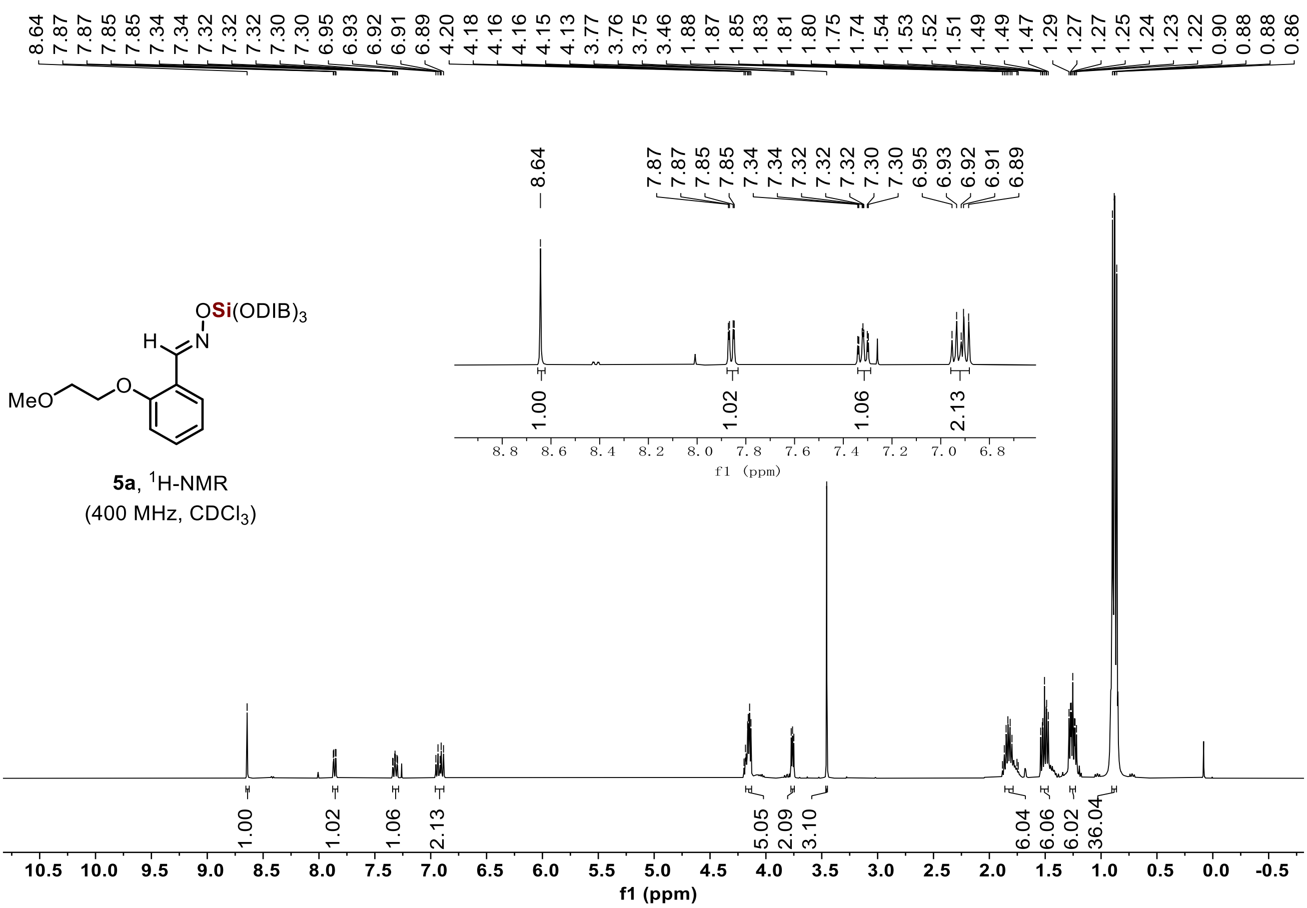

5a, ^{1}H-NMR
(400 MHz, $CDCl_3$)

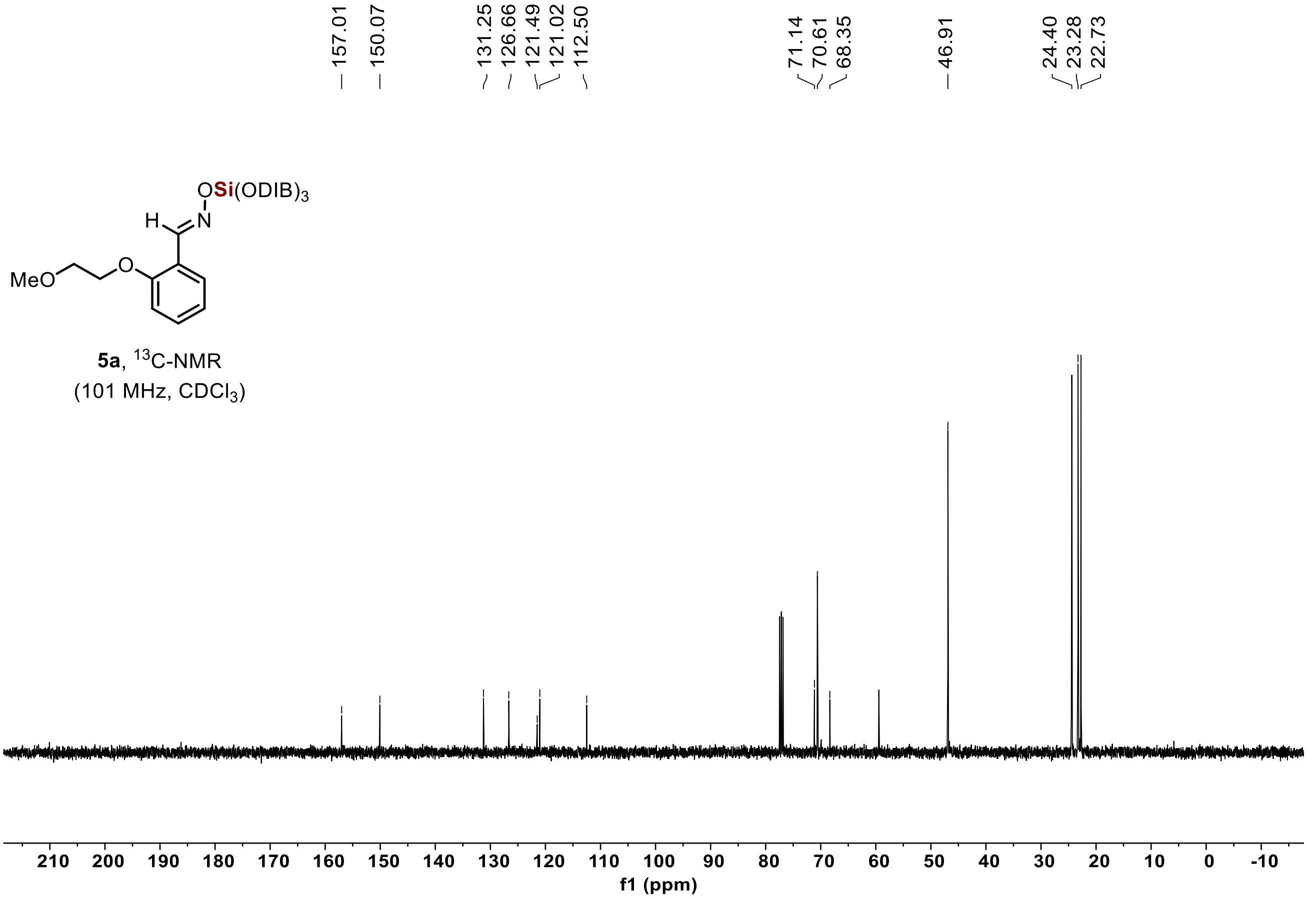

5a, ^{13}C-NMR
(101 MHz, $CDCl_3$)

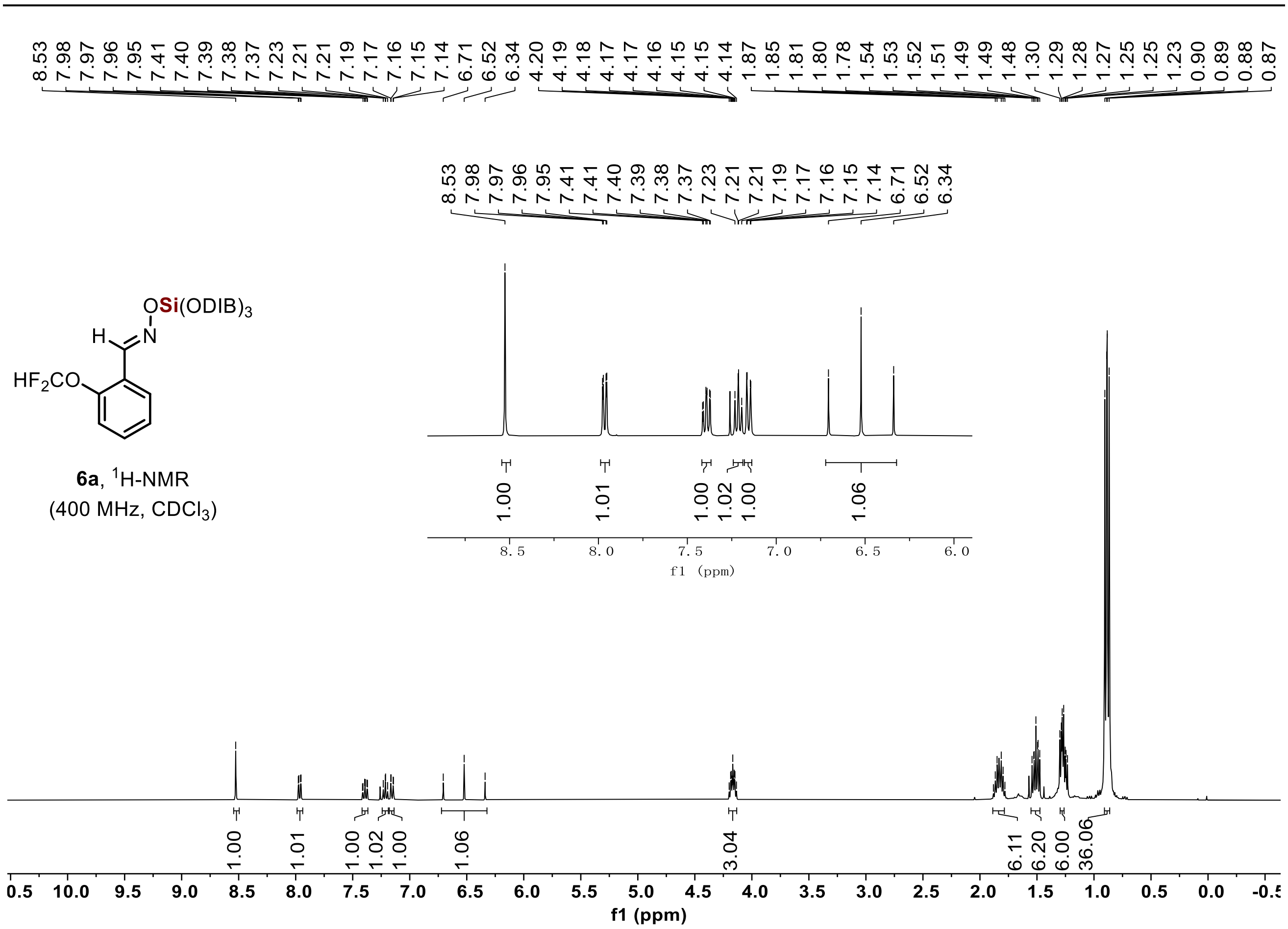

6a, ^{1}H-NMR
(400 MHz, $CDCl_3$)

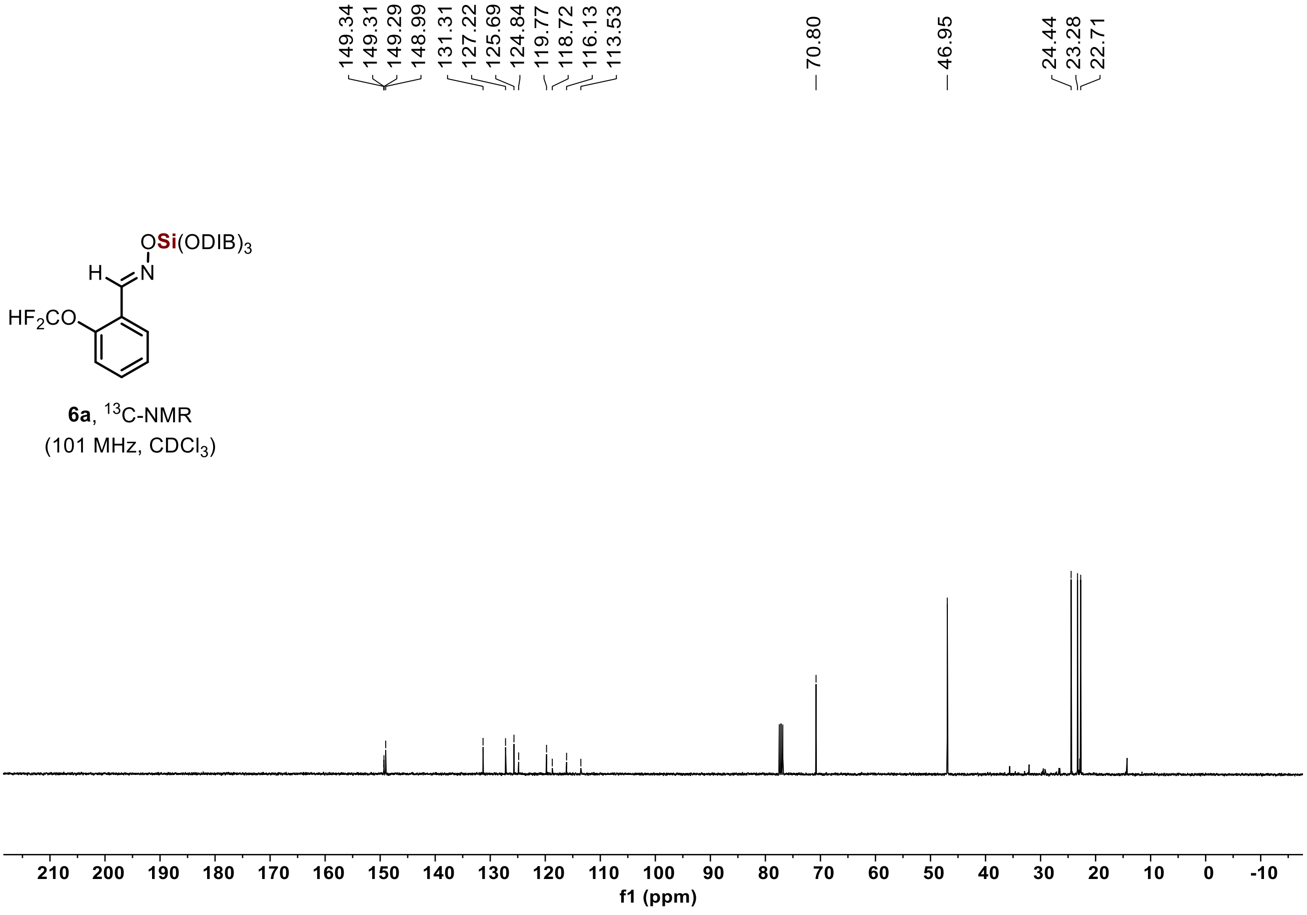

6a, ^{13}C-NMR
(101 MHz, $CDCl_3$)

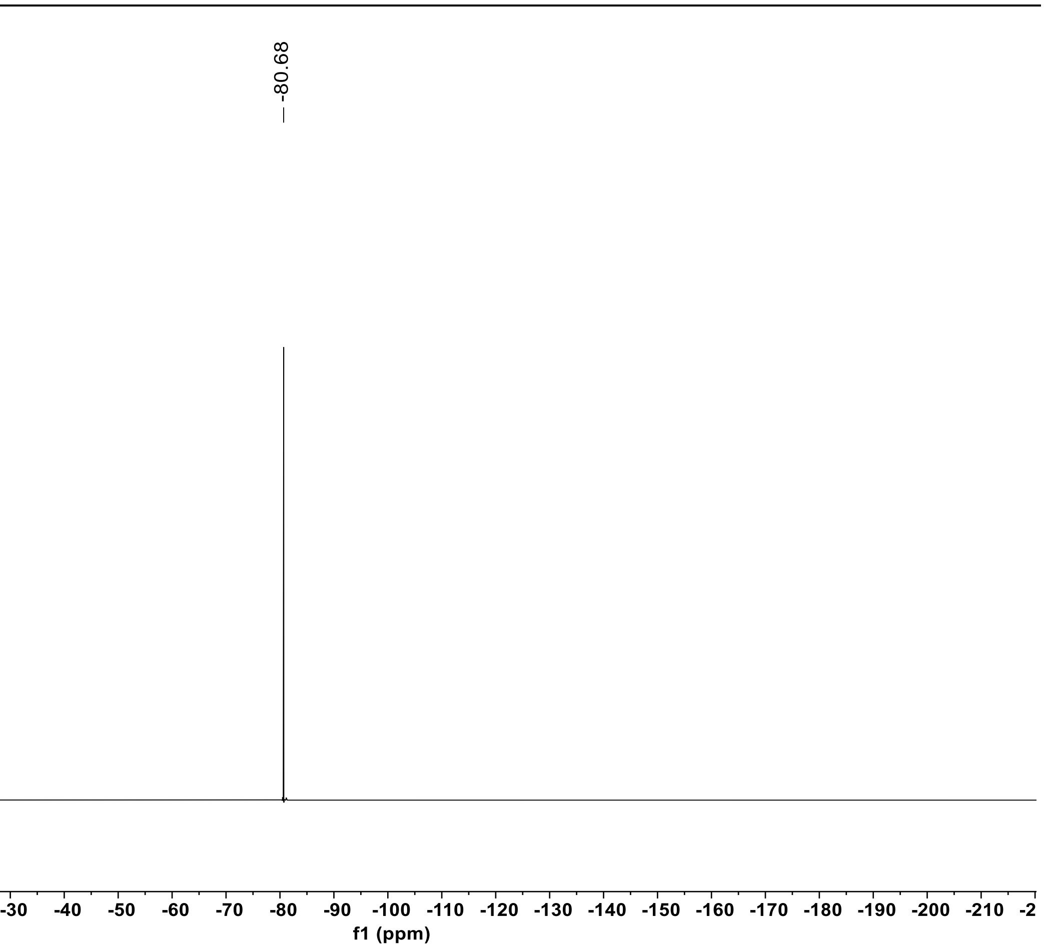

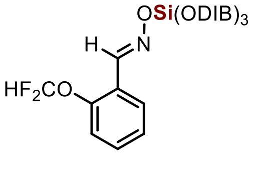

6a, ^{19}F-NMR
(376 MHz, $CDCl_3$)

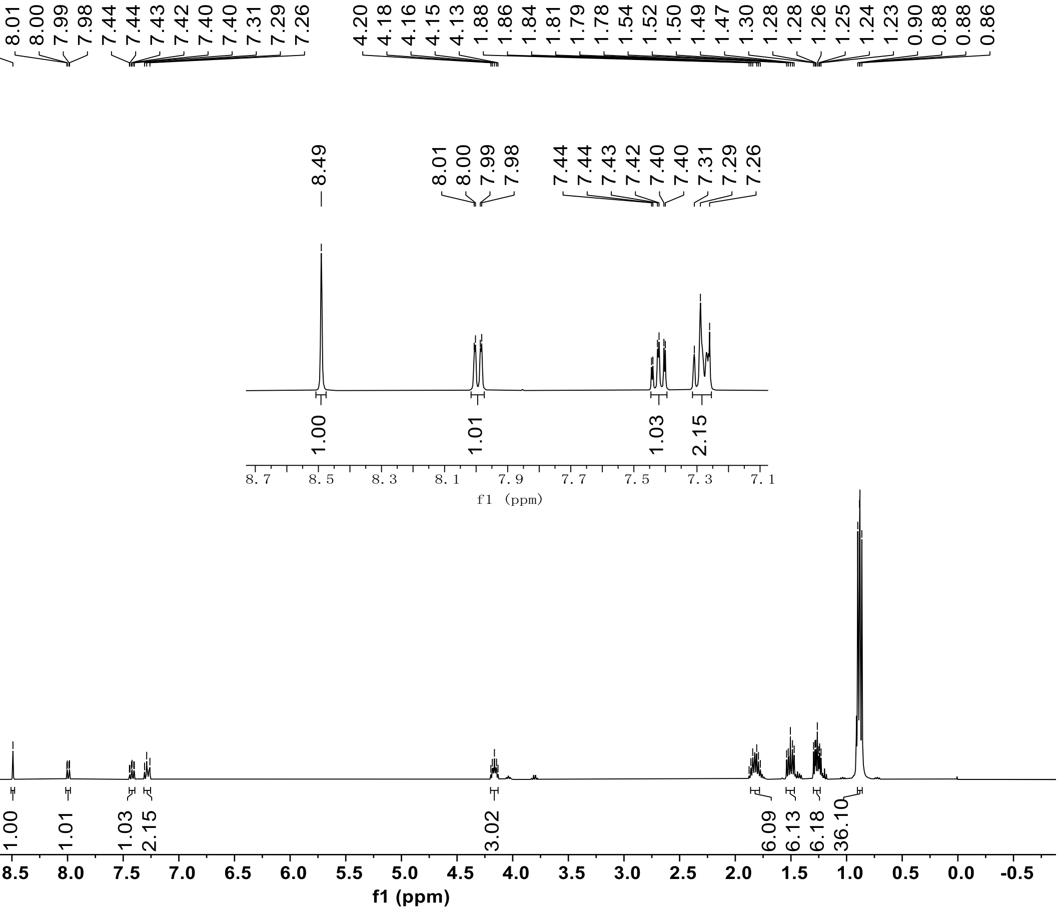

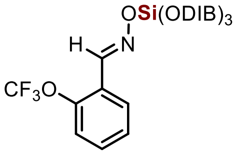

7a, ^{1}H-NMR
(400 MHz, $CDCl_3$)

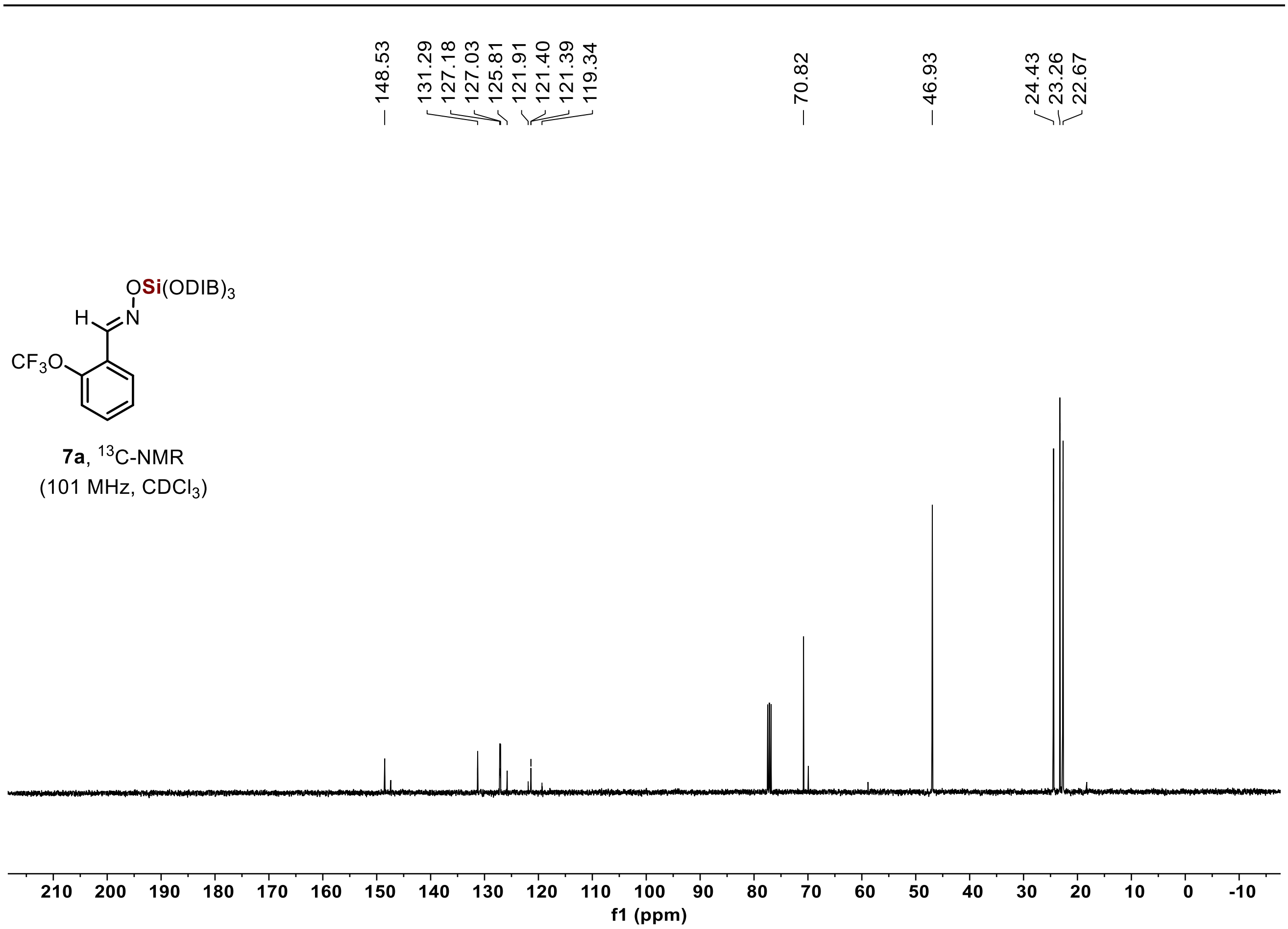

7a, ^{13}C-NMR
(101 MHz, $CDCl_3$)

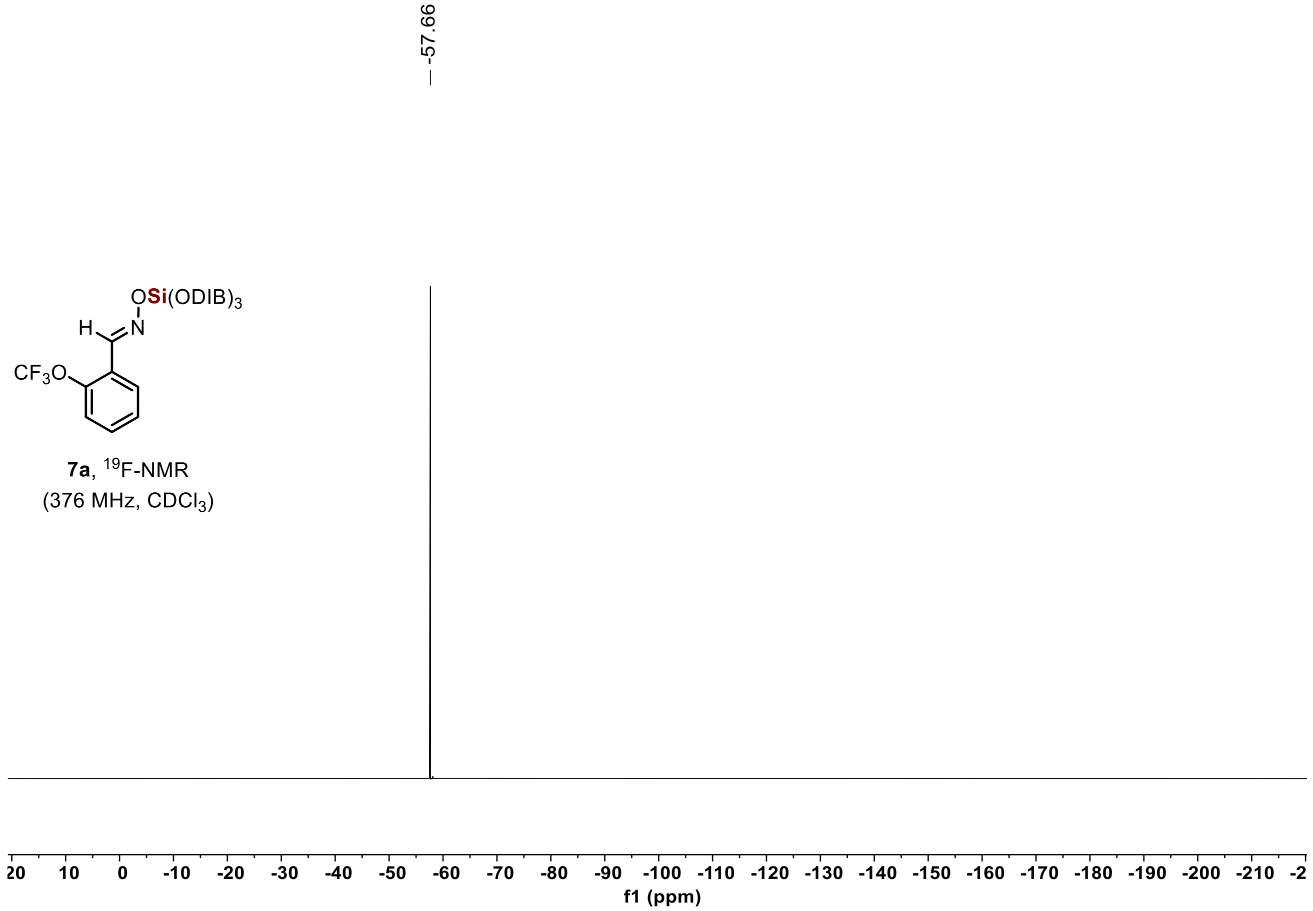

7a, ^{19}F-NMR
(376 MHz, $CDCl_3$)

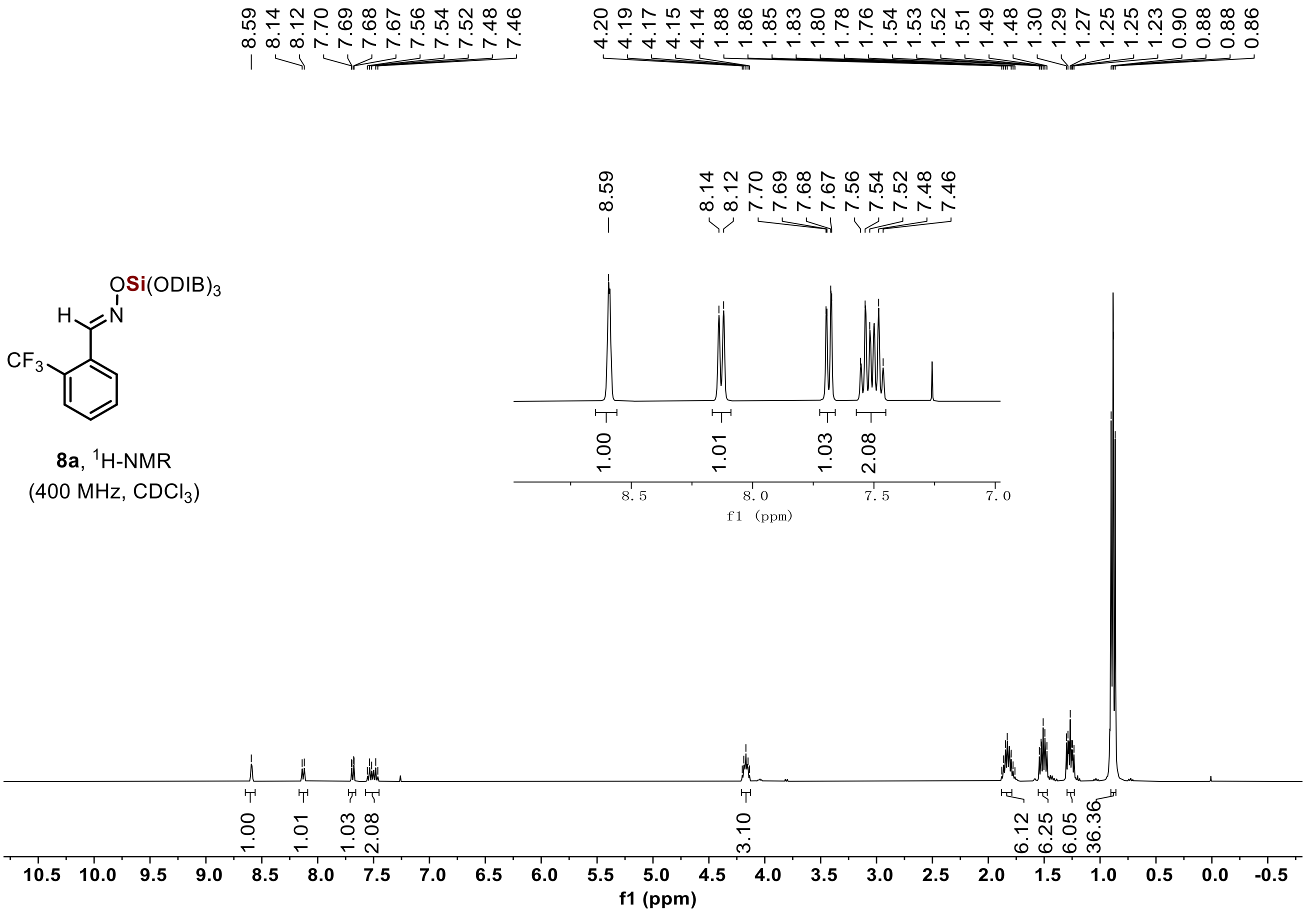
8.59 8.14 8.12 7.70 7.69 7.68 7.67 7.56 7.54 7.52 7.48 7.46
4.20 4.19 4.17 4.15 4.14 1.88 1.86 1.85 1.83 1.80 1.78 1.76 1.54 1.53 1.52 1.51 1.49 1.48 1.30 1.29 1.27 1.25 1.25 1.23 0.90 0.88 0.88 0.86
OSi(ODIB)3
H
N
CF3
8a, 1H-NMR
(400 MHz, CDCl3)
1.00 1.01 1.03 2.08 3.10 6.12 6.25 6.05 36.36
f1 (ppm)

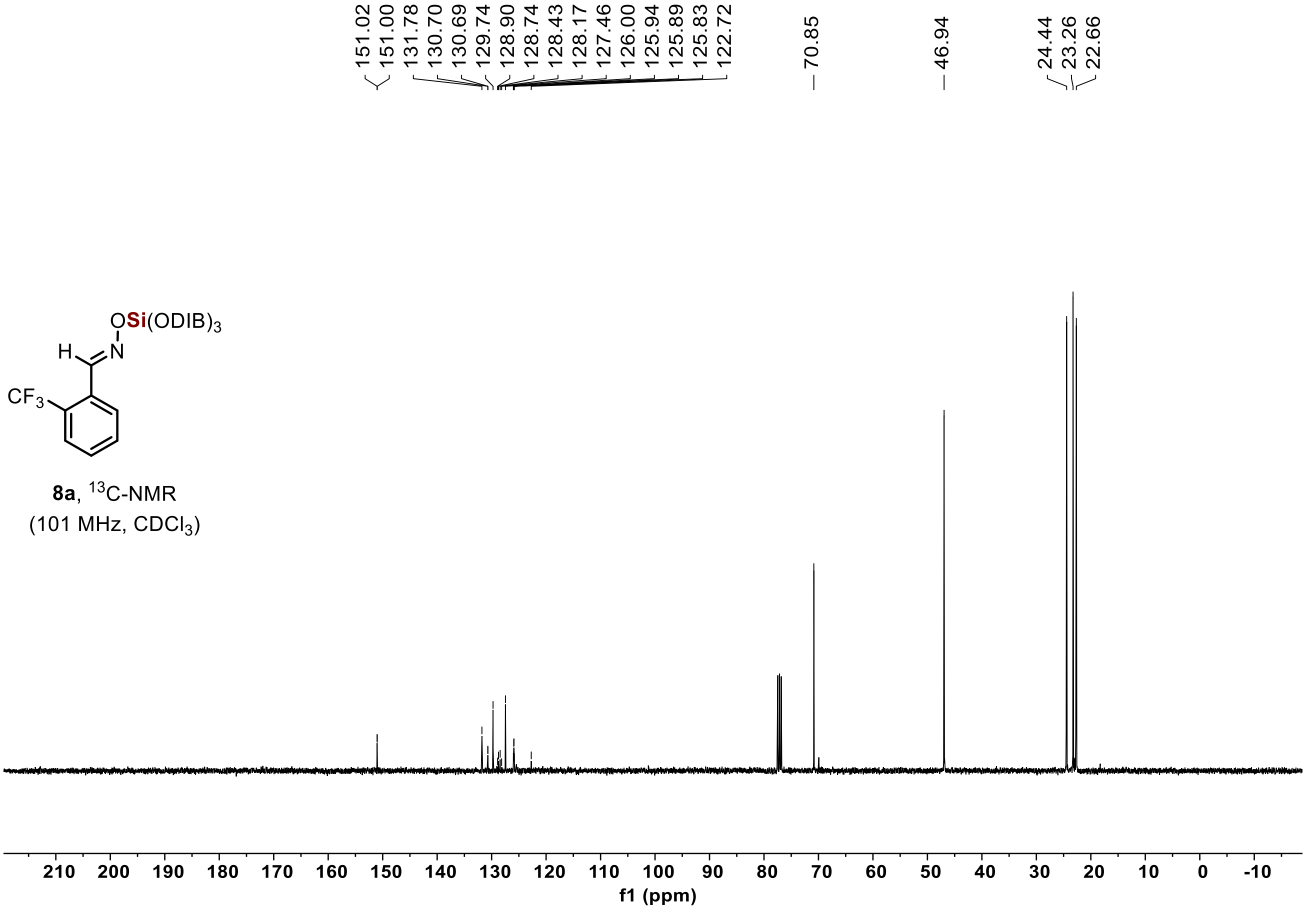
151.02 151.00 131.78 130.70 130.69 129.74 128.90 128.74 128.43 128.17 127.46 126.00 125.94 125.89 125.83 122.72
70.85
46.94
24.44 23.26 22.66
OSi(ODIB)3
H
N
CF3
8a, 13C-NMR
(101 MHz, CDCl3)
f1 (ppm)

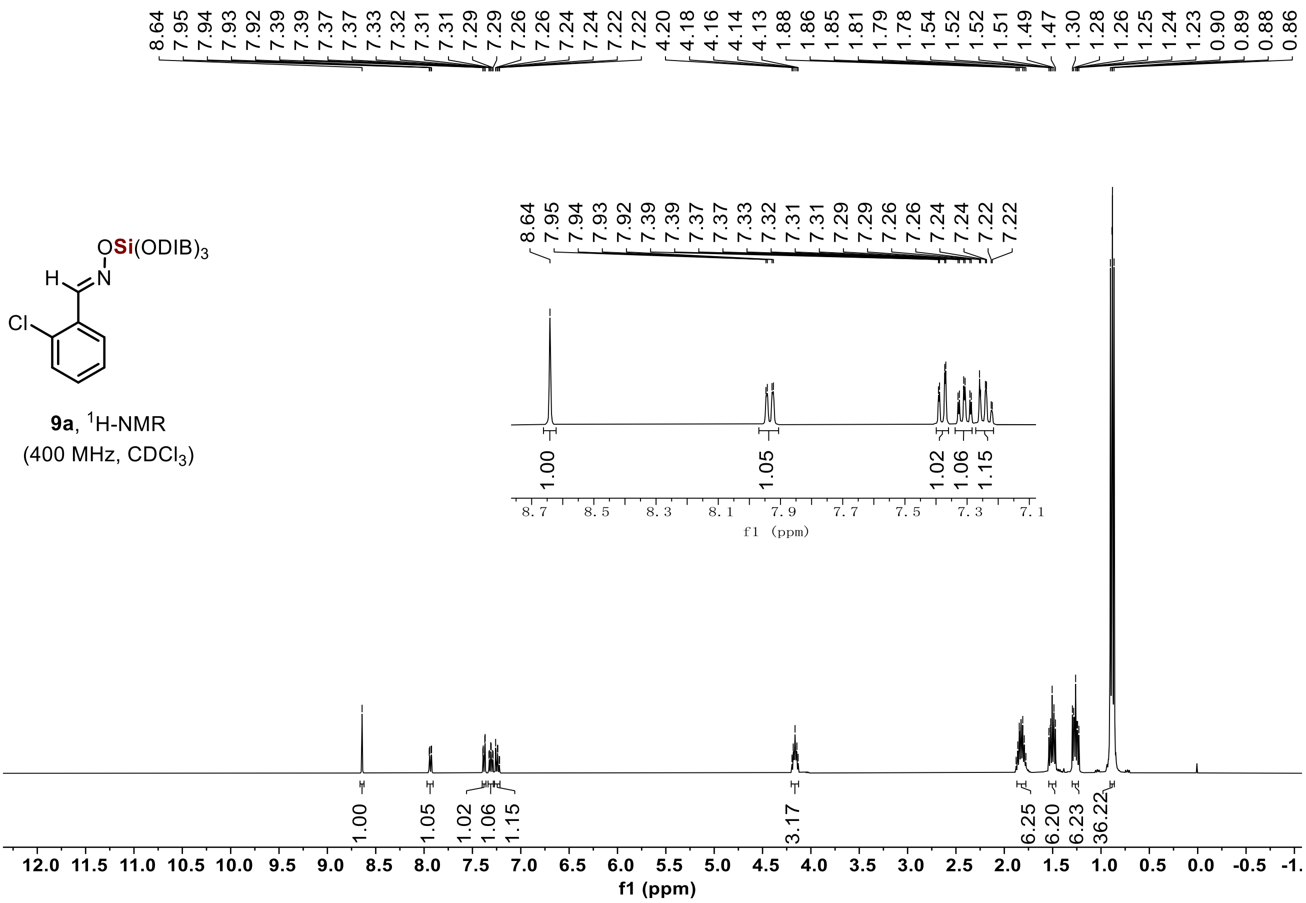

9a, ^{1}H-NMR
(400 MHz, $CDCl_3$)

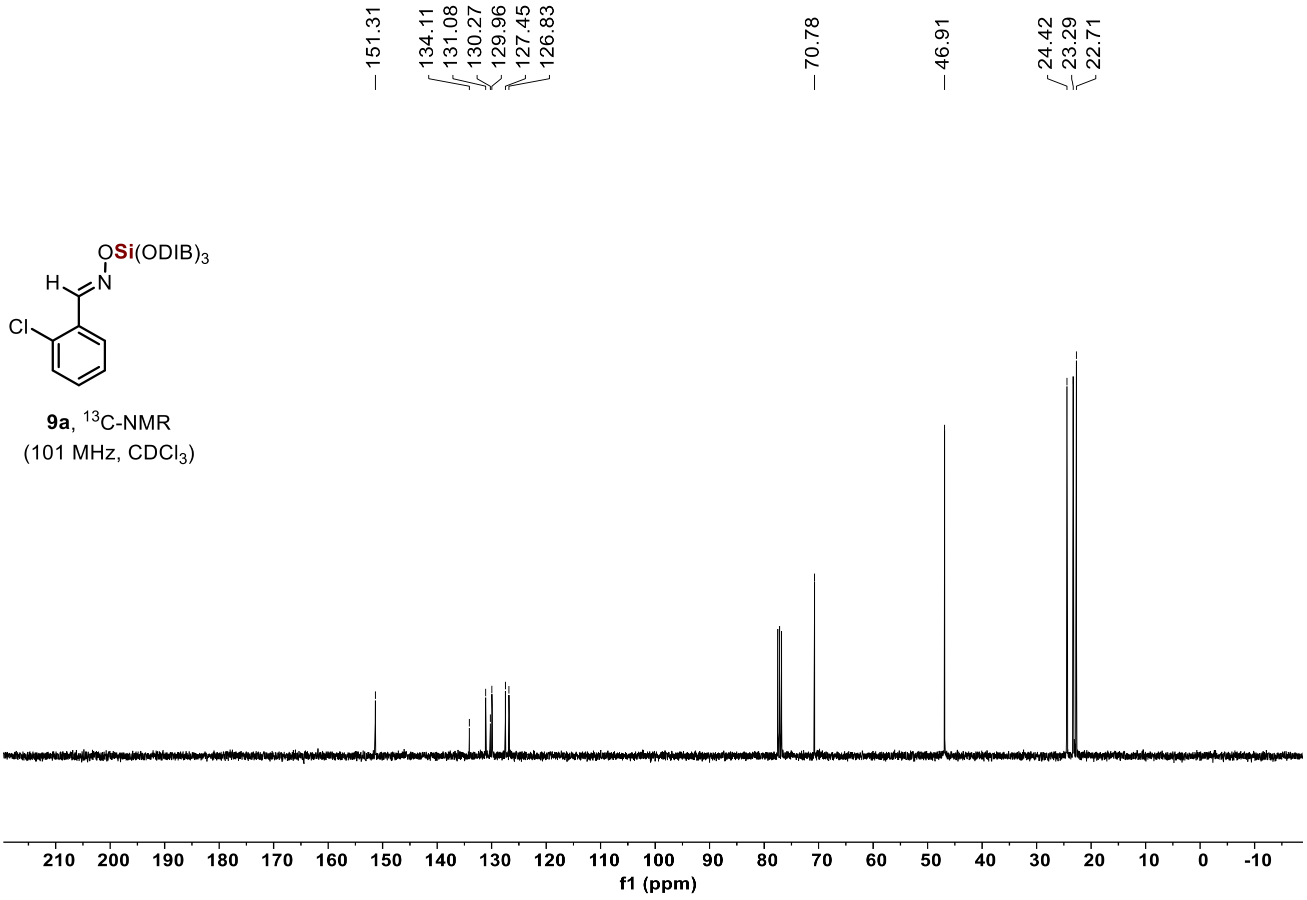

9a, ^{13}C-NMR
(101 MHz, $CDCl_3$)

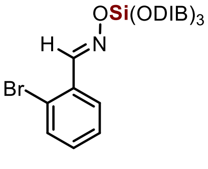

10a, ^{1}H-NMR
(400 MHz, $CDCl_3$)

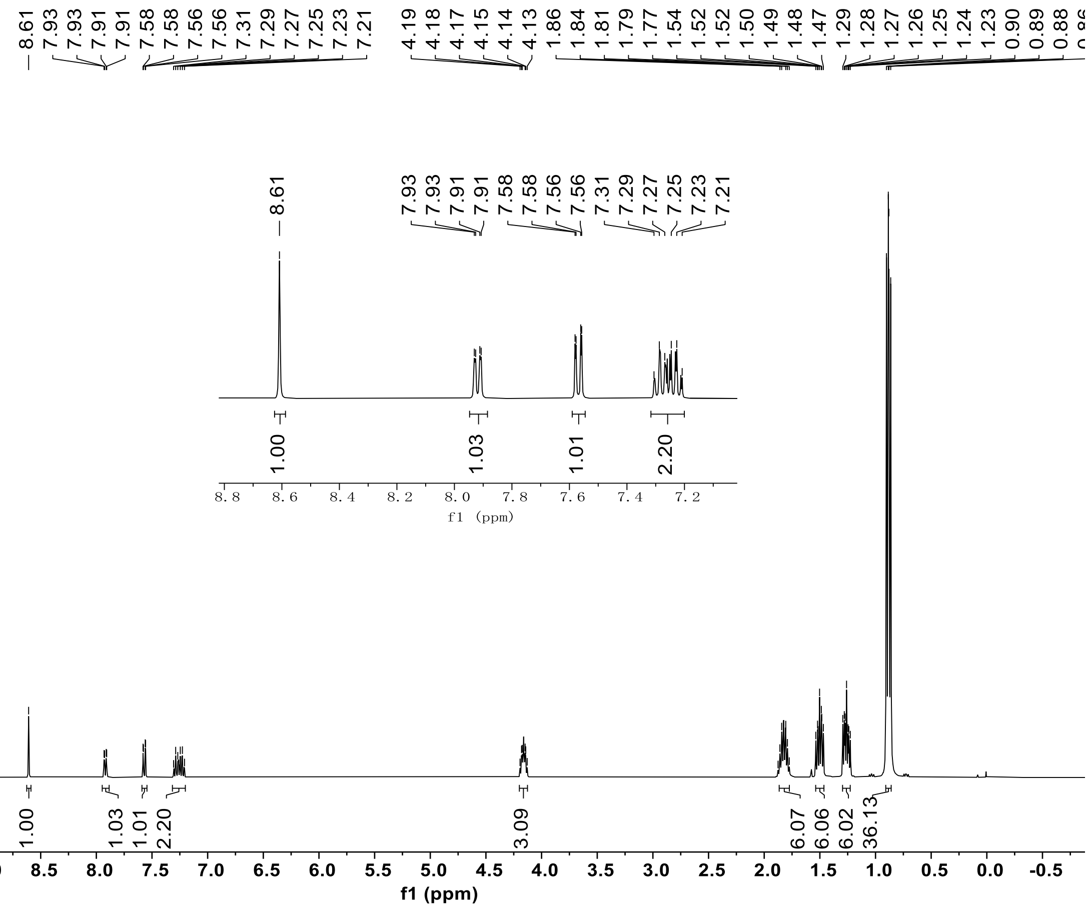

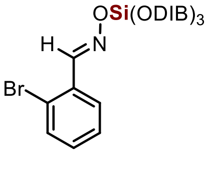

10a, ^{13}C-NMR
(101 MHz, $CDCl_3$)

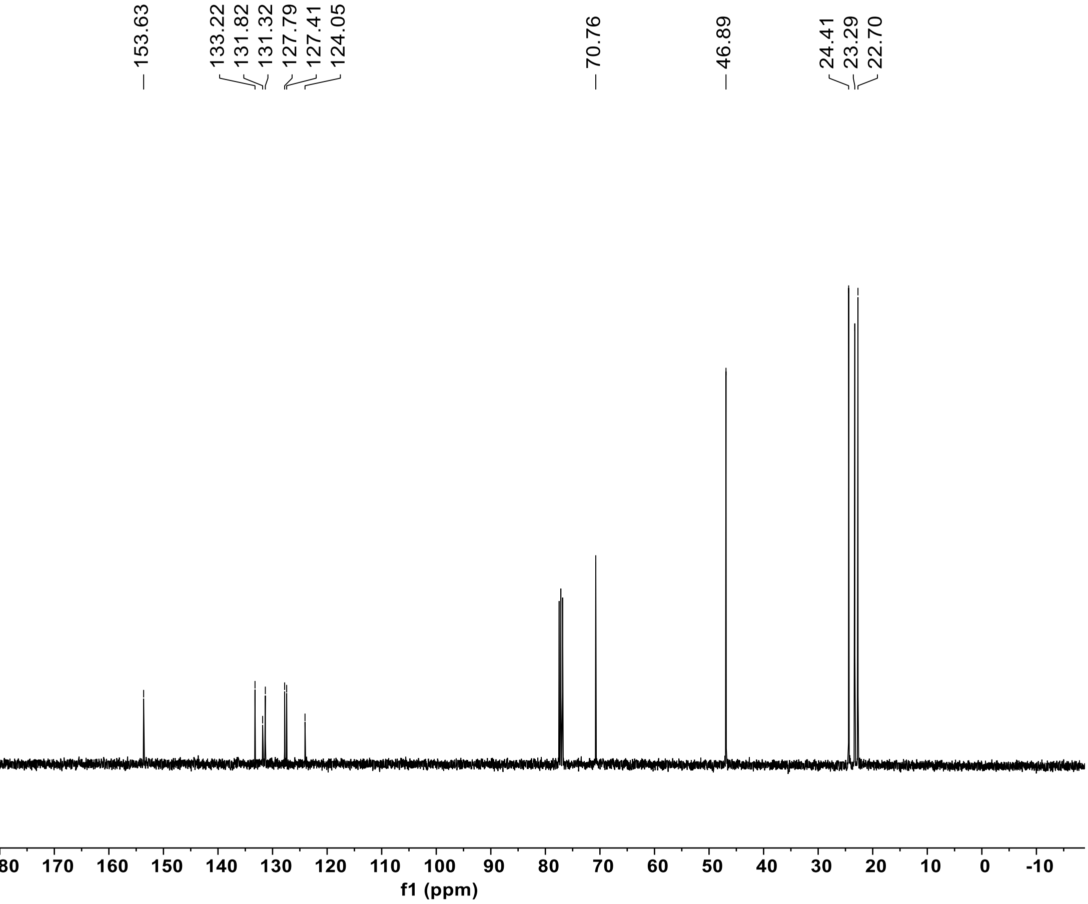

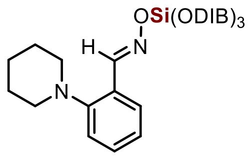

11a, ¹H-NMR
(400 MHz, $CDCl_3$)

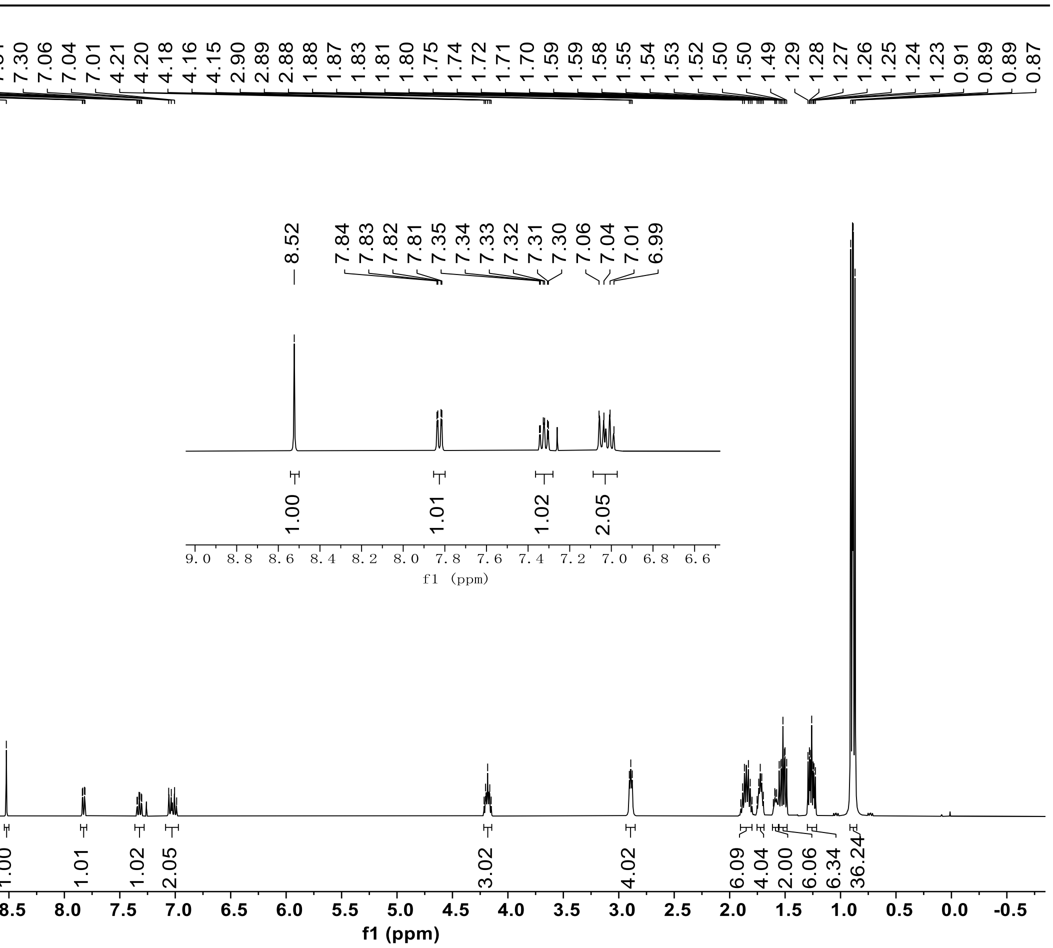

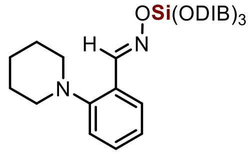

11a, ¹³C-NMR
(101 MHz, $CDCl_3$)

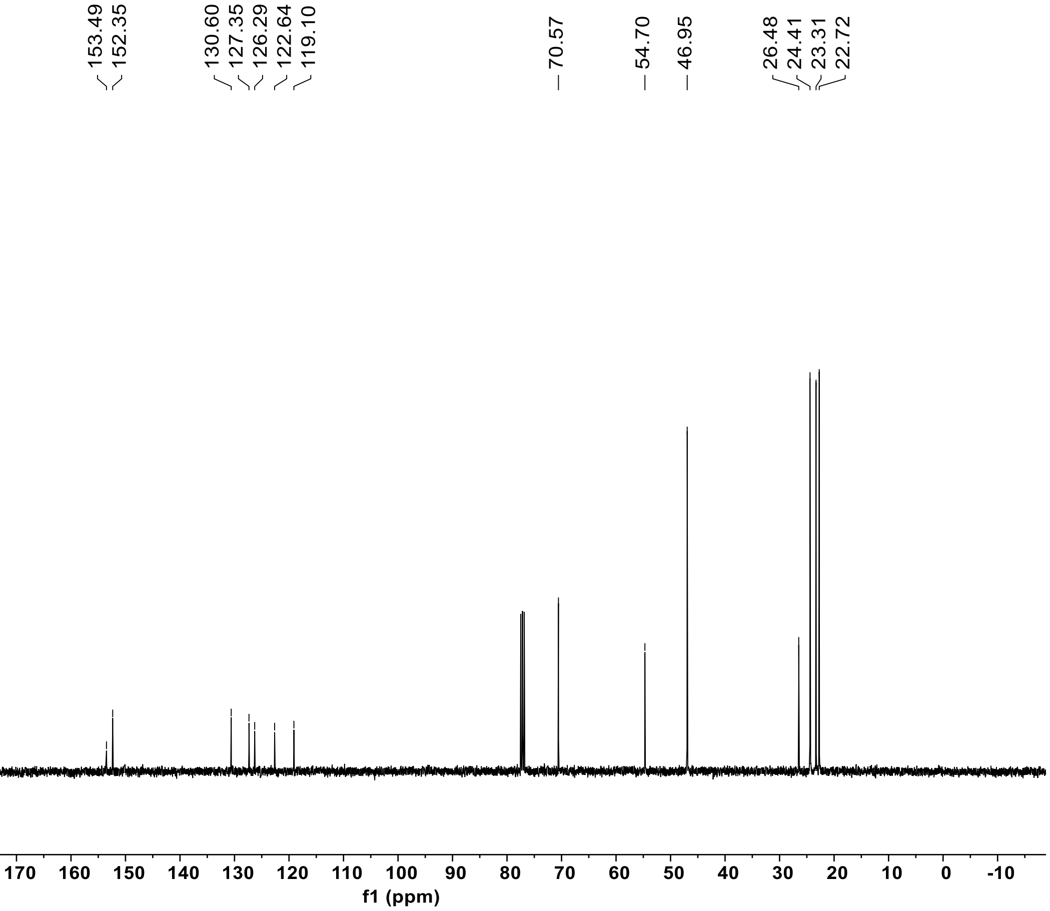

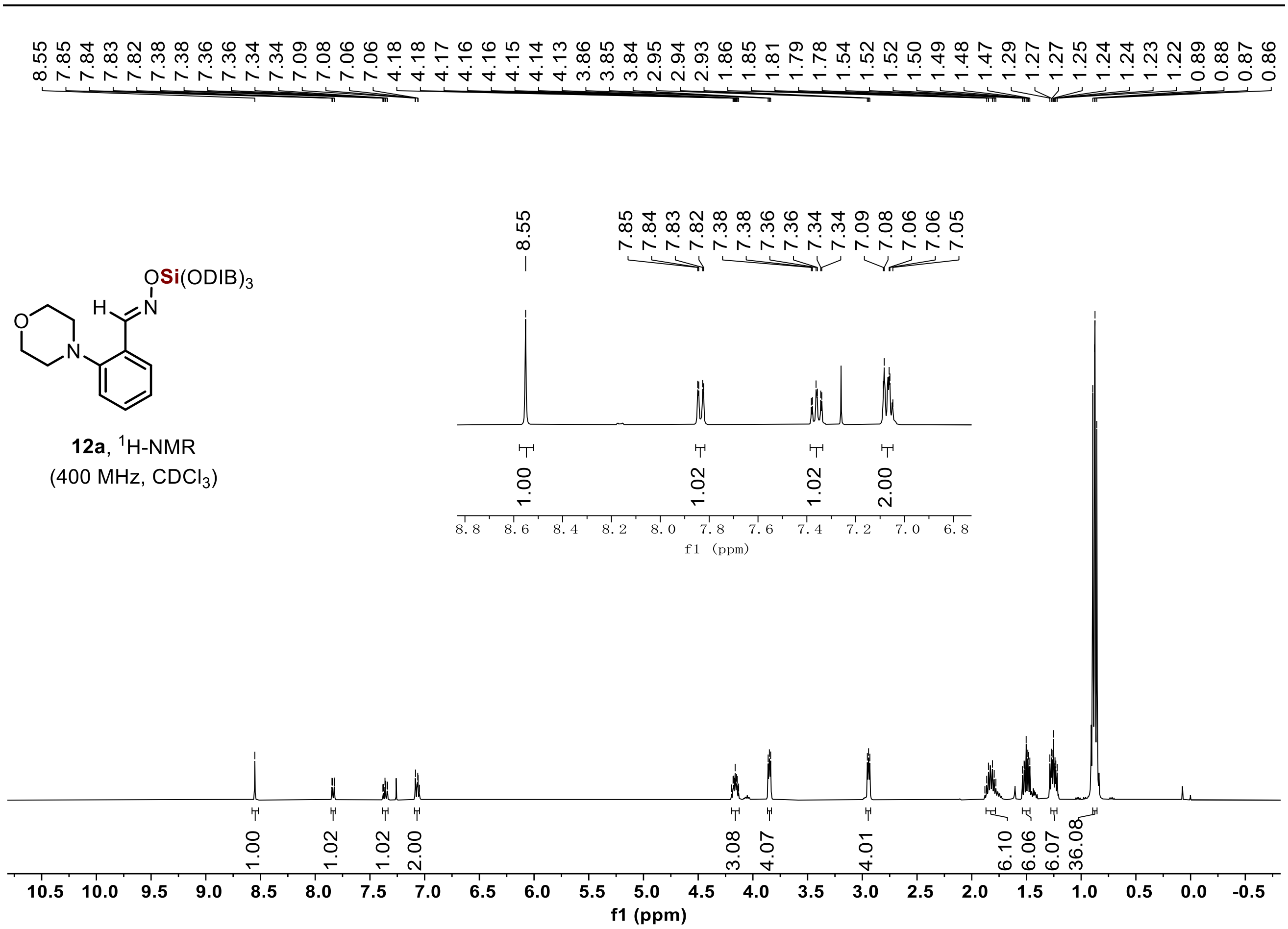

12a, ^{1}H-NMR
(400 MHz, $CDCl_3$)

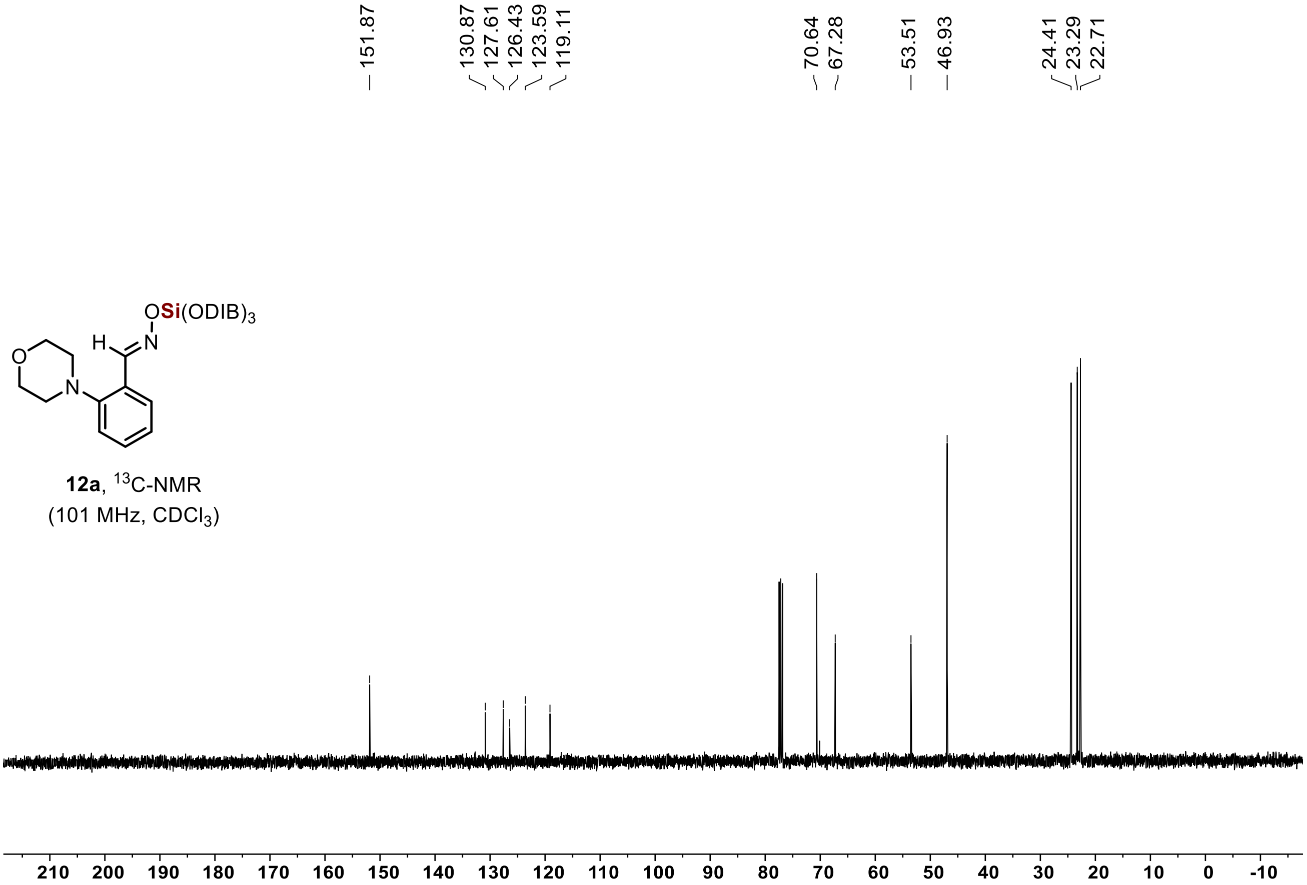

12a, ^{13}C-NMR
(101 MHz, $CDCl_3$)

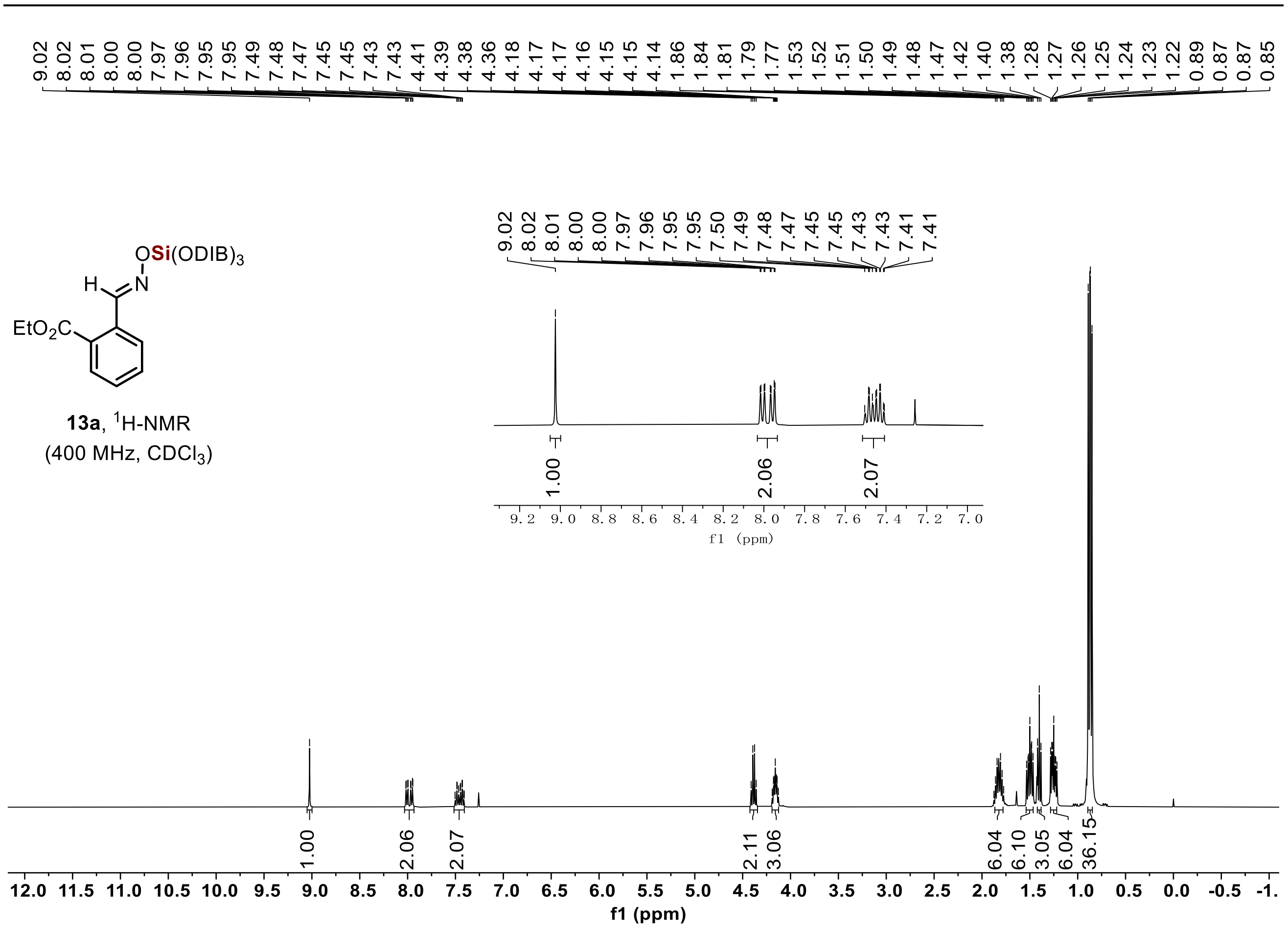

13a, ^{1}H-NMR
(400 MHz, $CDCl_3$)

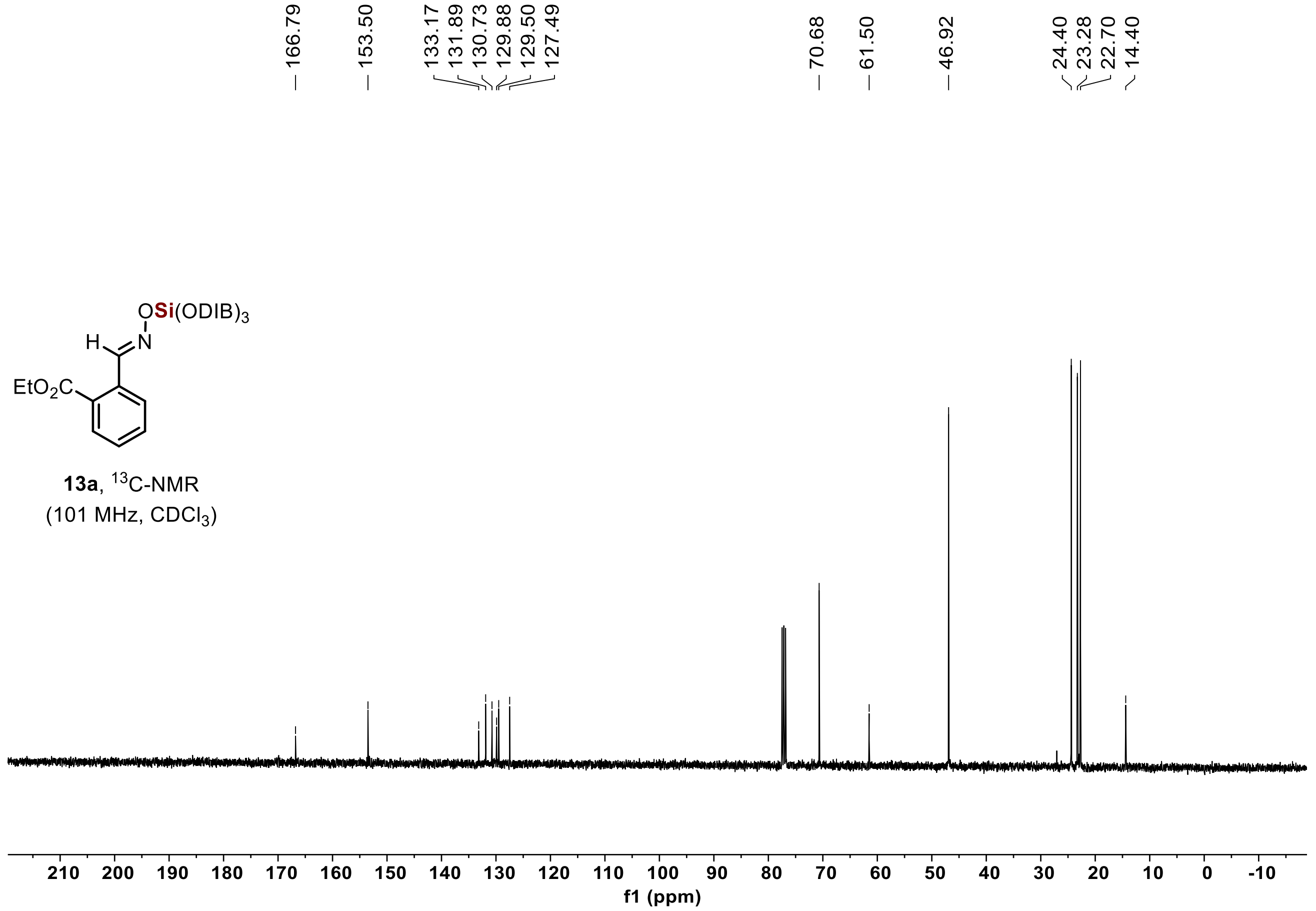

13a, ^{13}C-NMR
(101 MHz, $CDCl_3$)

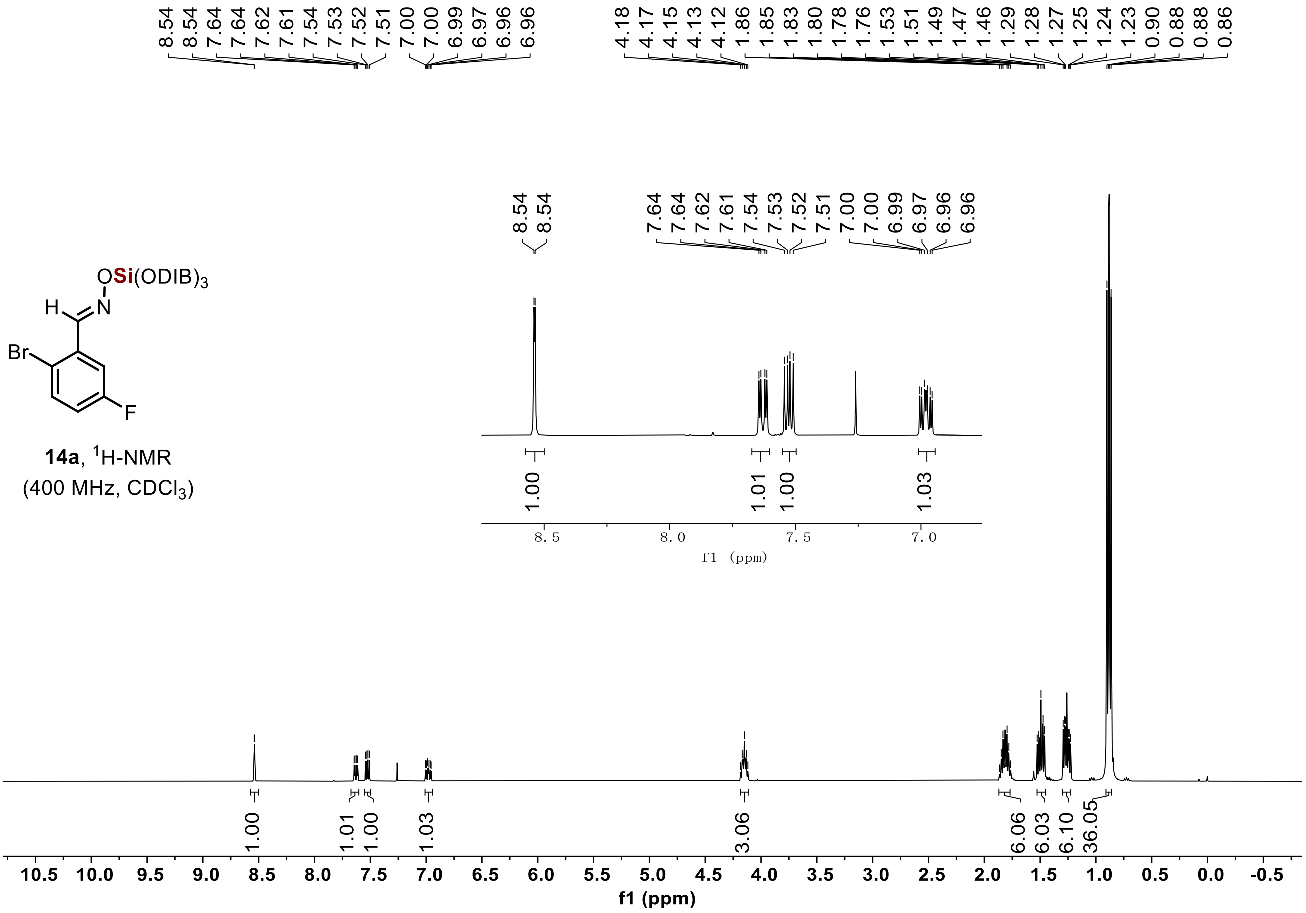

14a, ^{1}H-NMR
(400 MHz, $CDCl_3$)

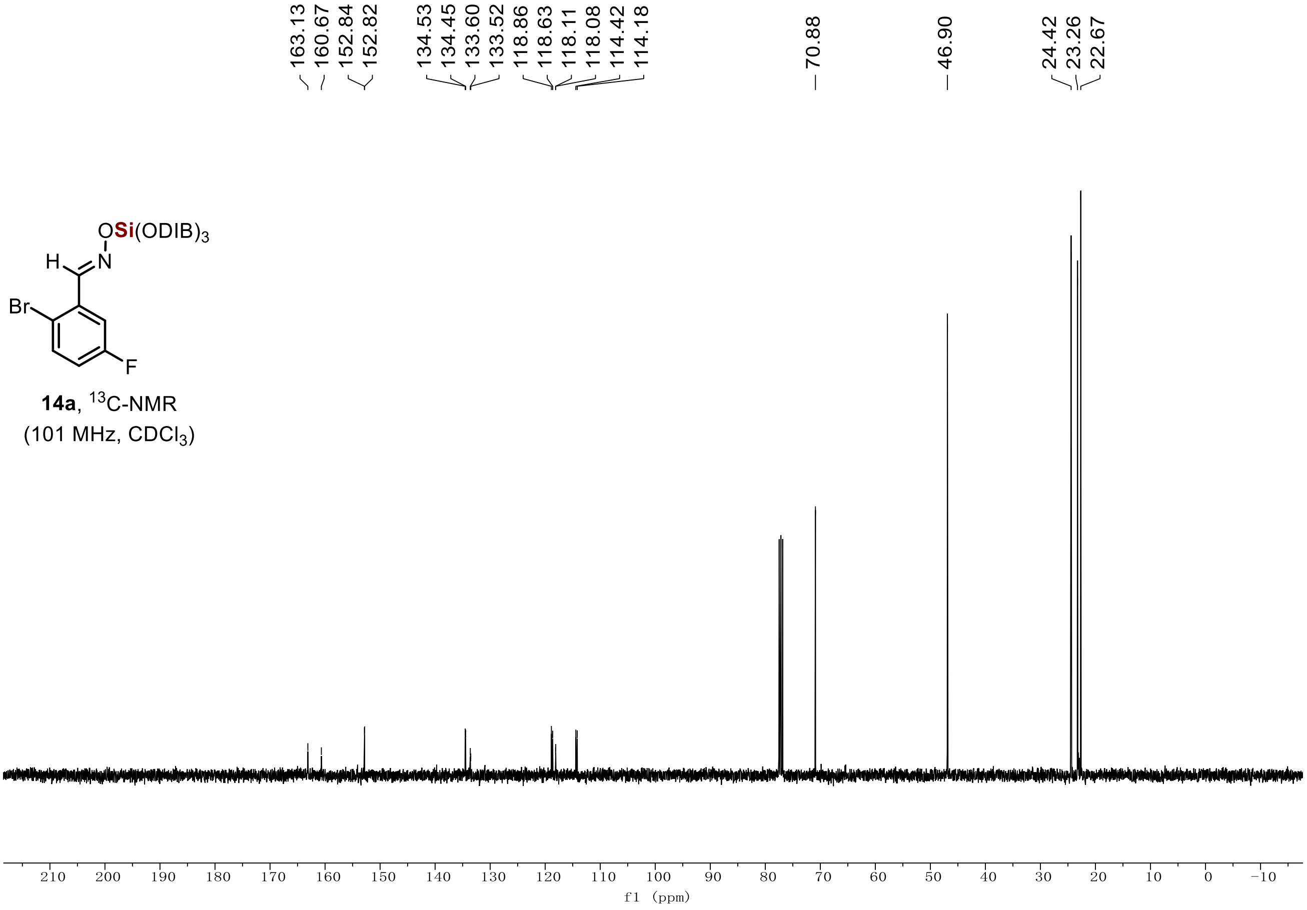

14a, ^{13}C-NMR
(101 MHz, $CDCl_3$)

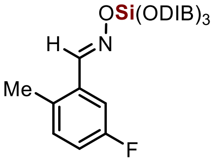

15a, ^{1}H-NMR
(400 MHz, $CDCl_3$)

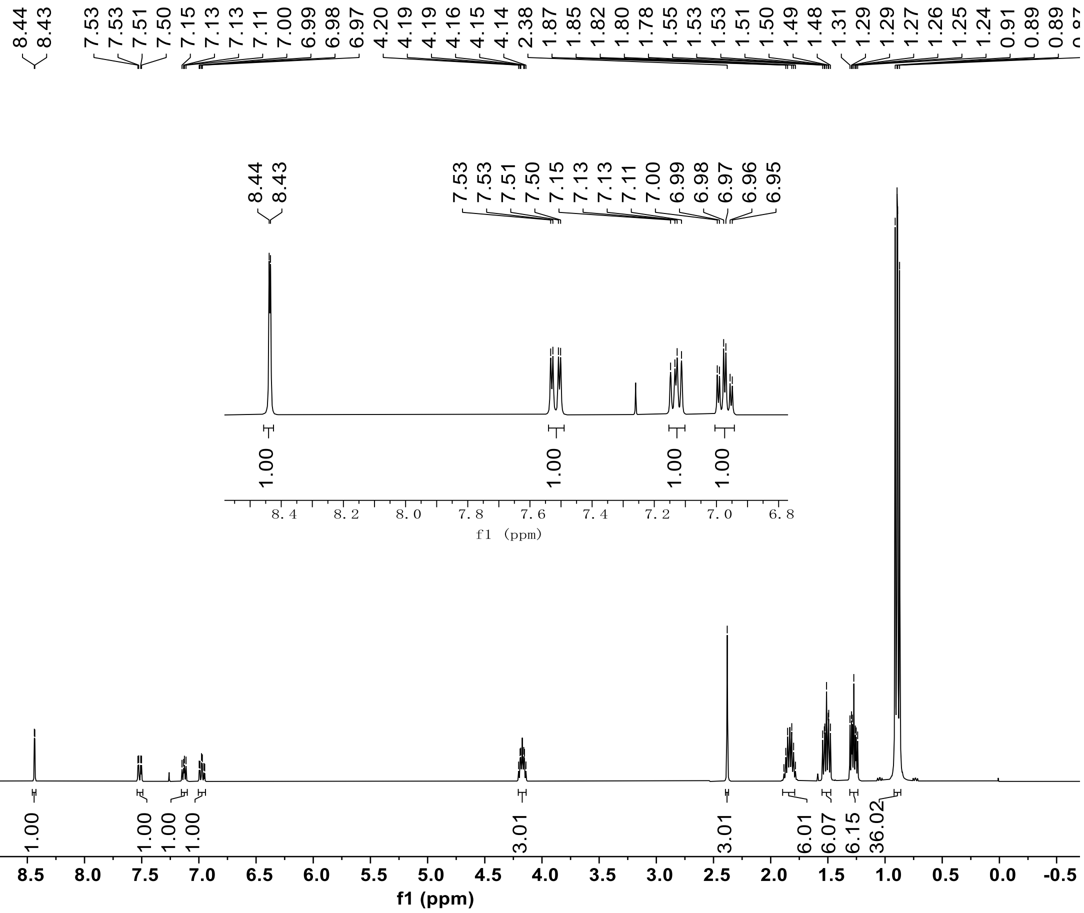

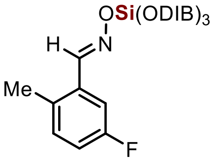

15a, ^{13}C-NMR
(101 MHz, $CDCl_3$)

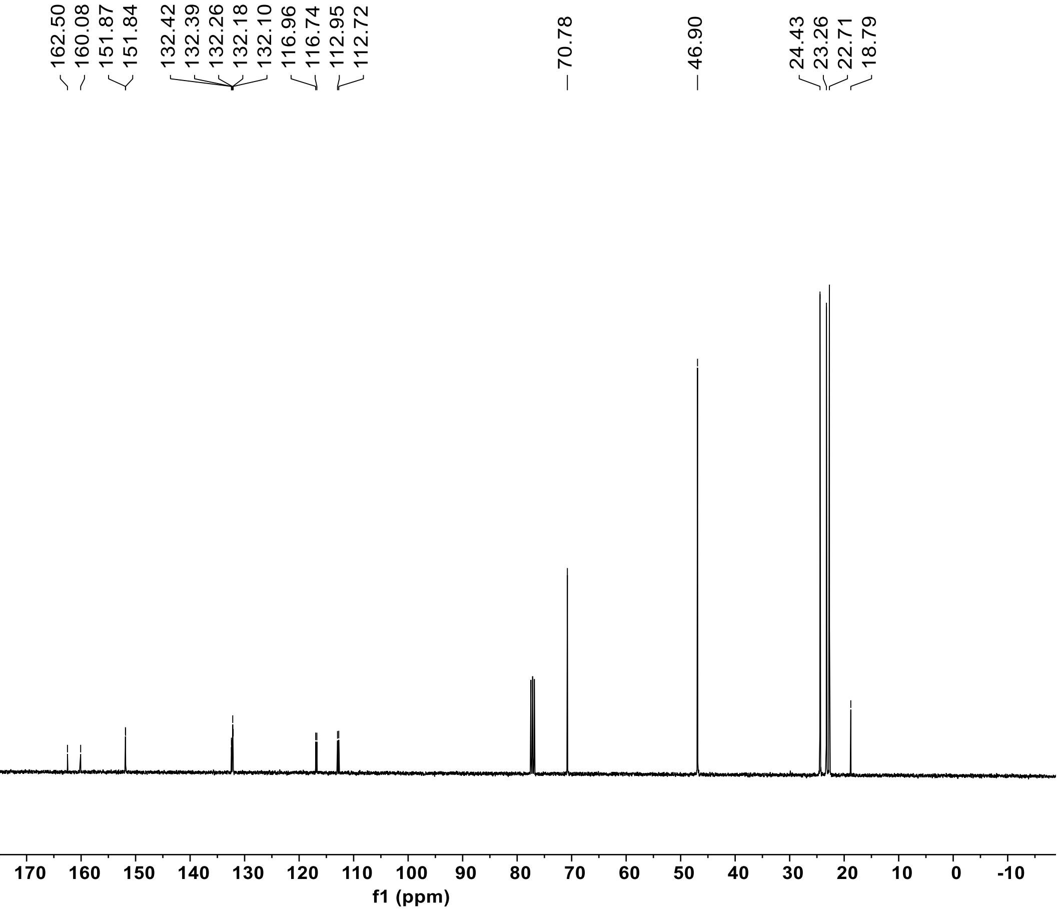

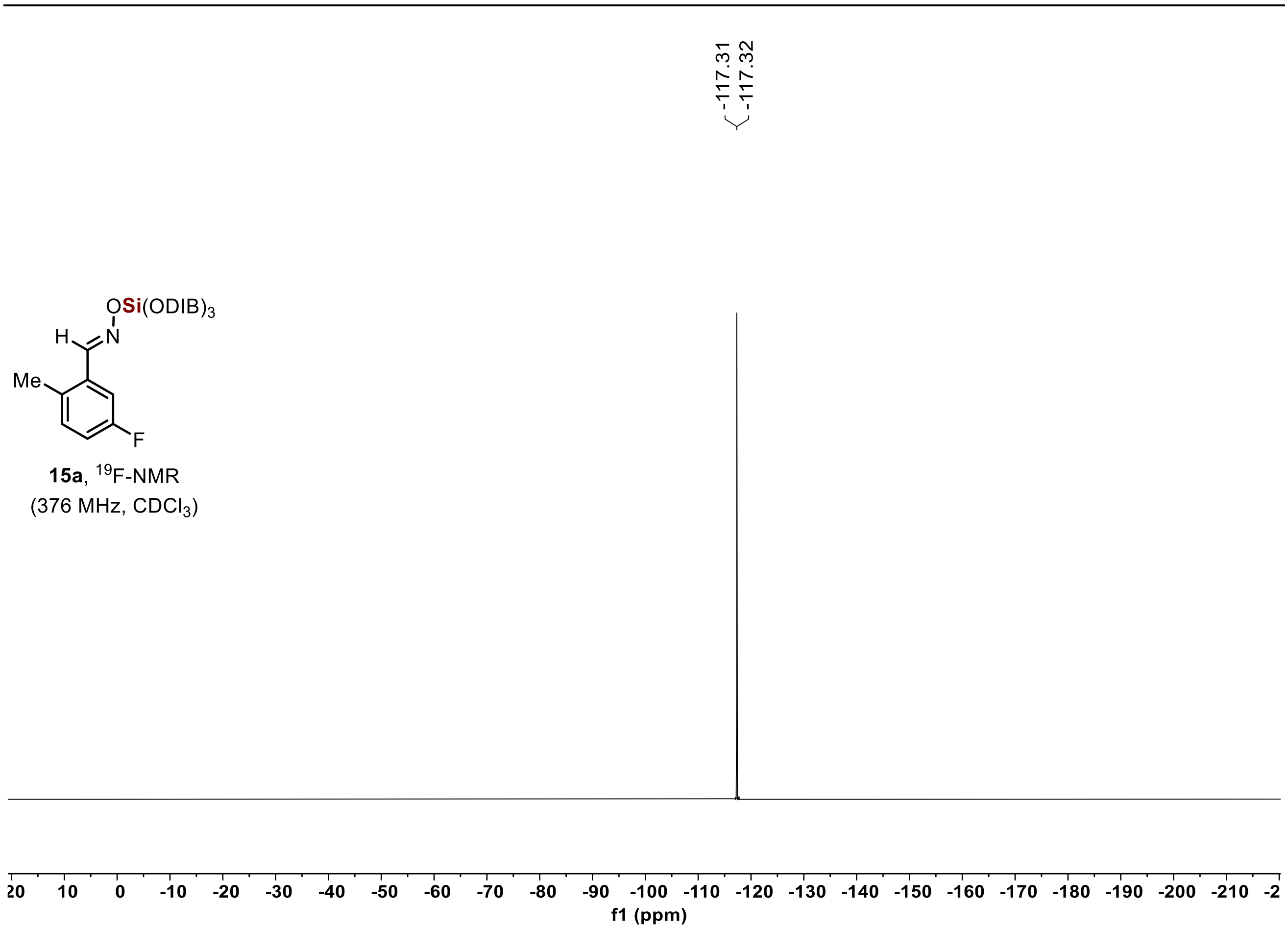

-117.31
-117.32
OSi(ODIB)3
H
N
Me
F
15a, 19F-NMR
(376 MHz, CDCl3)
20 10 0 -10 -20 -30 -40 -50 -60 -70 -80 -90 -100 -110 -120 -130 -140 -150 -160 -170 -180 -190 -200 -210 -2
f1 (ppm)

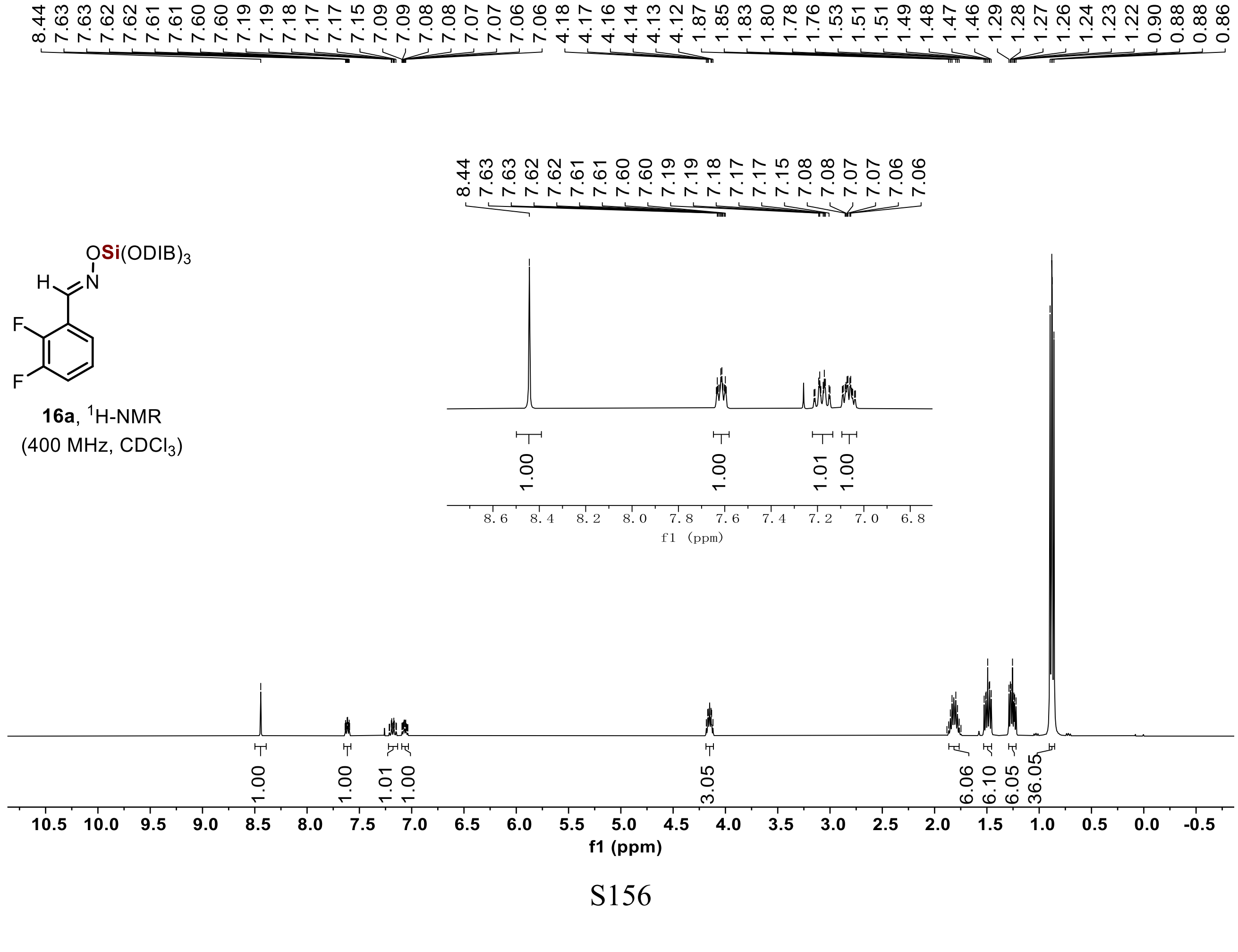

8.44 7.63 7.63 7.62 7.62 7.61 7.61 7.60 7.60 7.19 7.19 7.18 7.17 7.17 7.15 7.09 7.08 7.08 7.07 7.07 7.06 7.06 4.18 4.17 4.16 4.14 4.13 4.12 1.87 1.85 1.83 1.80 1.78 1.76 1.53 1.51 1.51 1.49 1.48 1.47 1.46 1.29 1.28 1.27 1.26 1.24 1.23 1.22 0.90 0.88 0.88 0.86
8.44 7.63 7.63 7.62 7.62 7.61 7.61 7.60 7.60 7.19 7.19 7.18 7.17 7.17 7.15 7.08 7.08 7.07 7.07 7.06 7.06
OSi(ODIB)3
H
N
F
F
16a, 1H-NMR
(400 MHz, CDCl3)
1.00
1.00
1.01
1.00
8.6 8.4 8.2 8.0 7.8 7.6 7.4 7.2 7.0 6.8
f1 (ppm)
1.00
1.00
1.01
1.00
3.05
6.06
6.10
6.05
36.05
10.5 10.0 9.5 9.0 8.5 8.0 7.5 7.0 6.5 6.0 5.5 5.0 4.5 4.0 3.5 3.0 2.5 2.0 1.5 1.0 0.5 0.0 -0.5
f1 (ppm)

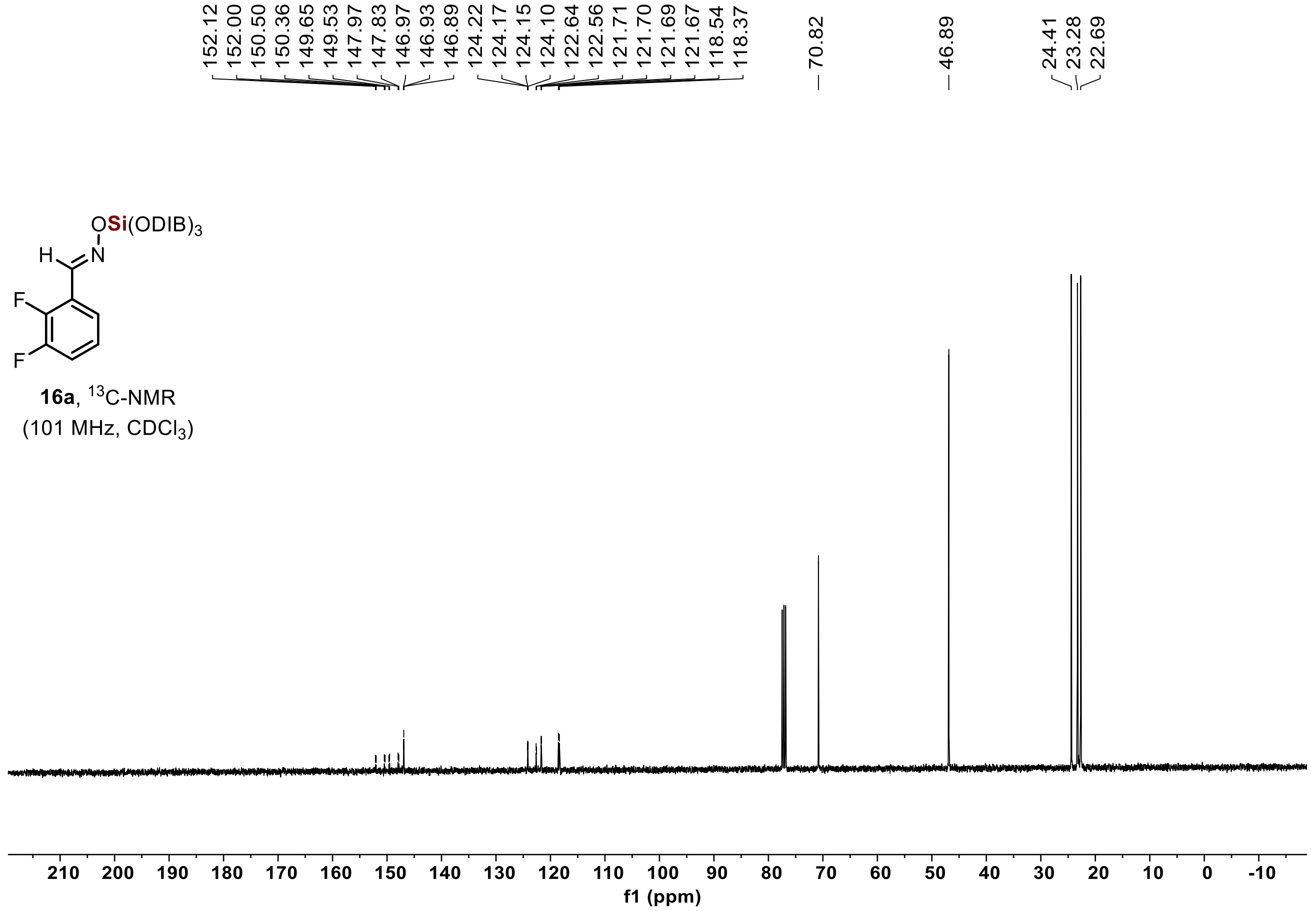

152.12
152.00
150.50
150.36
149.65
149.53
147.97
147.83
146.97
146.93
146.89
124.22
124.17
124.15
124.10
122.64
122.56
121.71
121.70
121.69
121.67
118.54
118.37
70.82
46.89
24.41
23.28
22.69
OSi(ODIB)3
H
N
F
F
16a, 13C-NMR
(101 MHz, CDCl3)
210 200 190 180 170 160 150 140 130 120 110 100 90 80 70 60 50 40 30 20 10 0 -10
f1 (ppm)

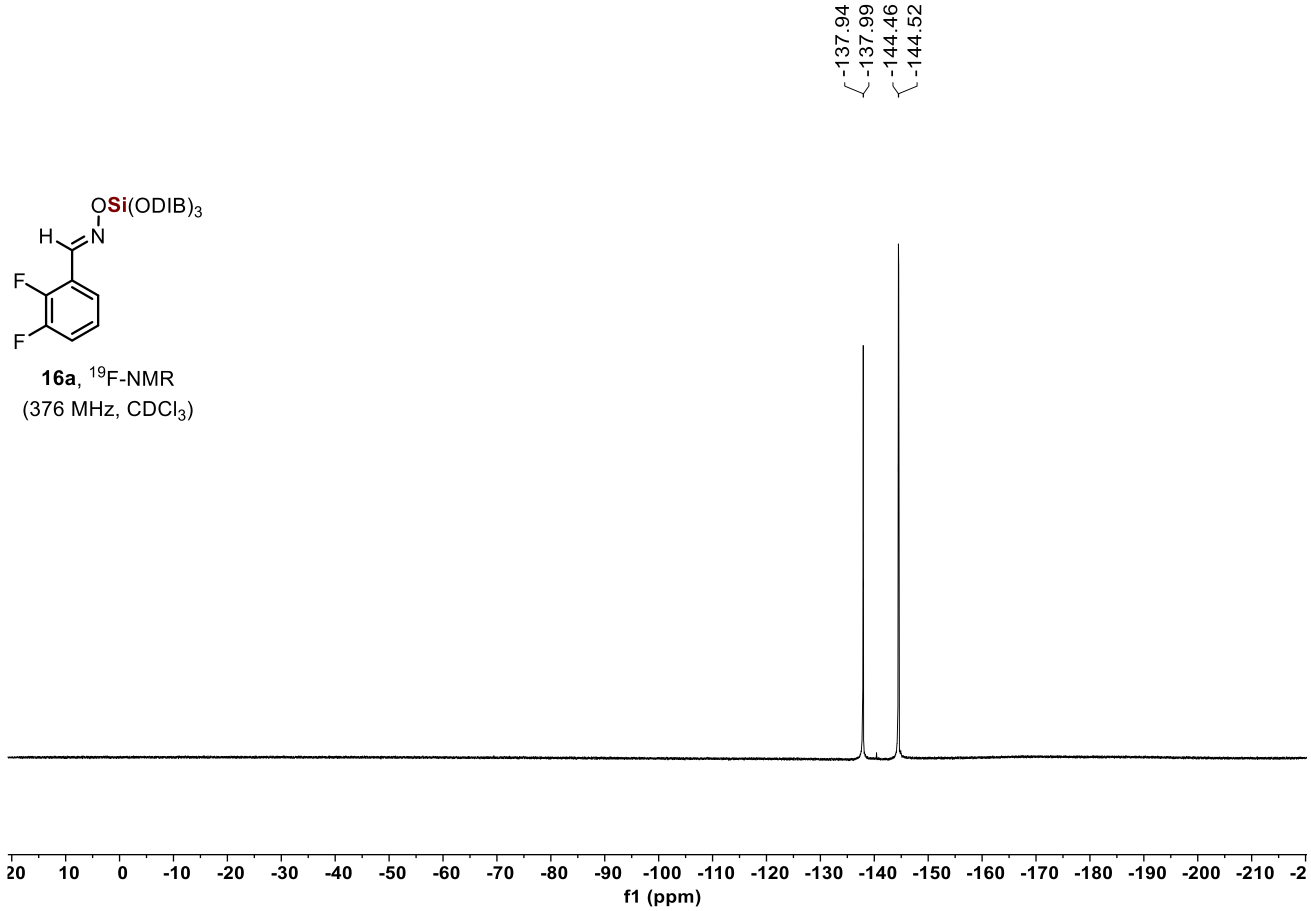

-137.94
-137.99
-144.46
-144.52
OSi(ODIB)3
H
N
F
F
16a, 19F-NMR
(376 MHz, CDCl3)
20 10 0 -10 -20 -30 -40 -50 -60 -70 -80 -90 -100 -110 -120 -130 -140 -150 -160 -170 -180 -190 -200 -210 -2
f1 (ppm)

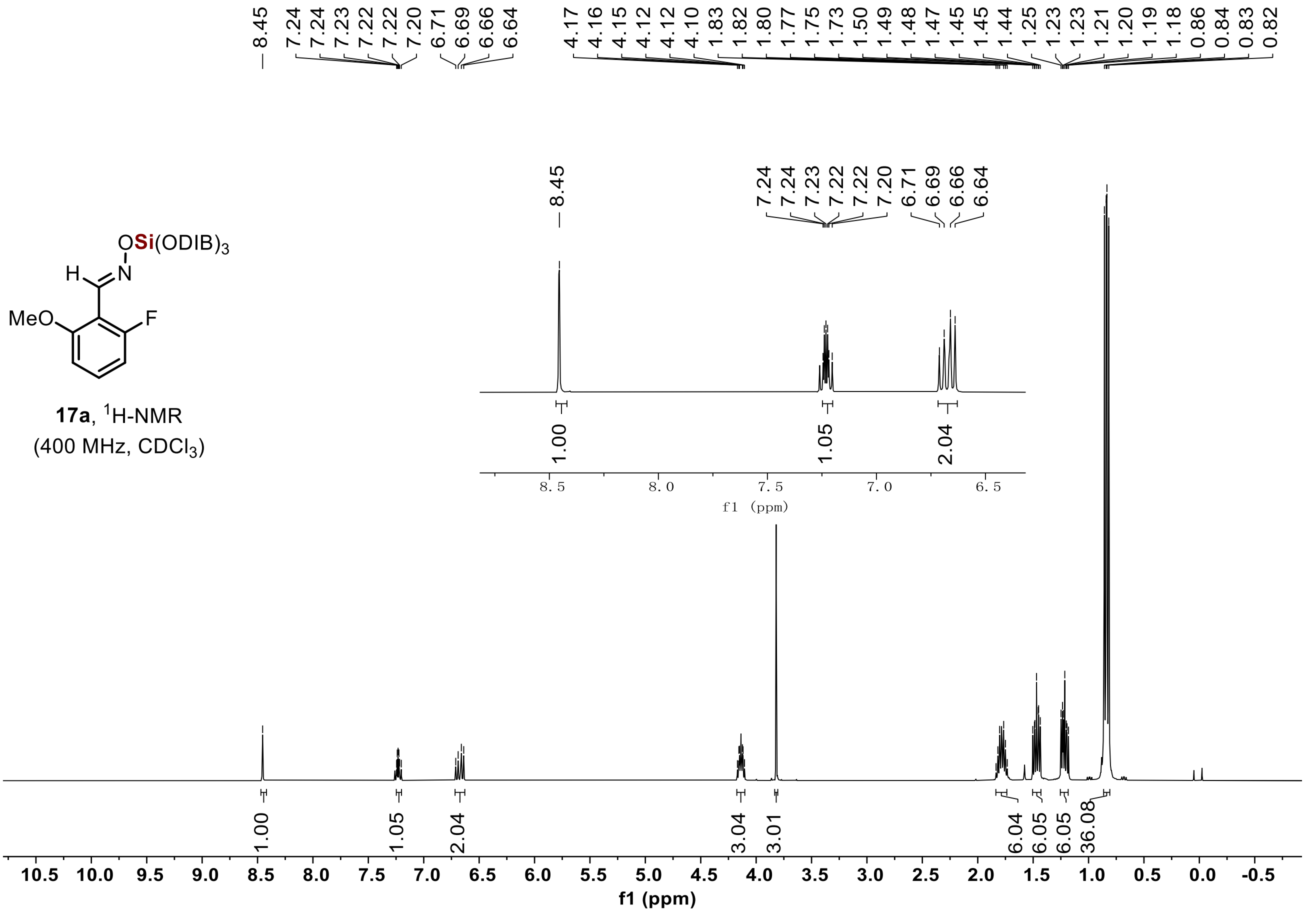

17a, ^{1}H-NMR
(400 MHz, $CDCl_3$)

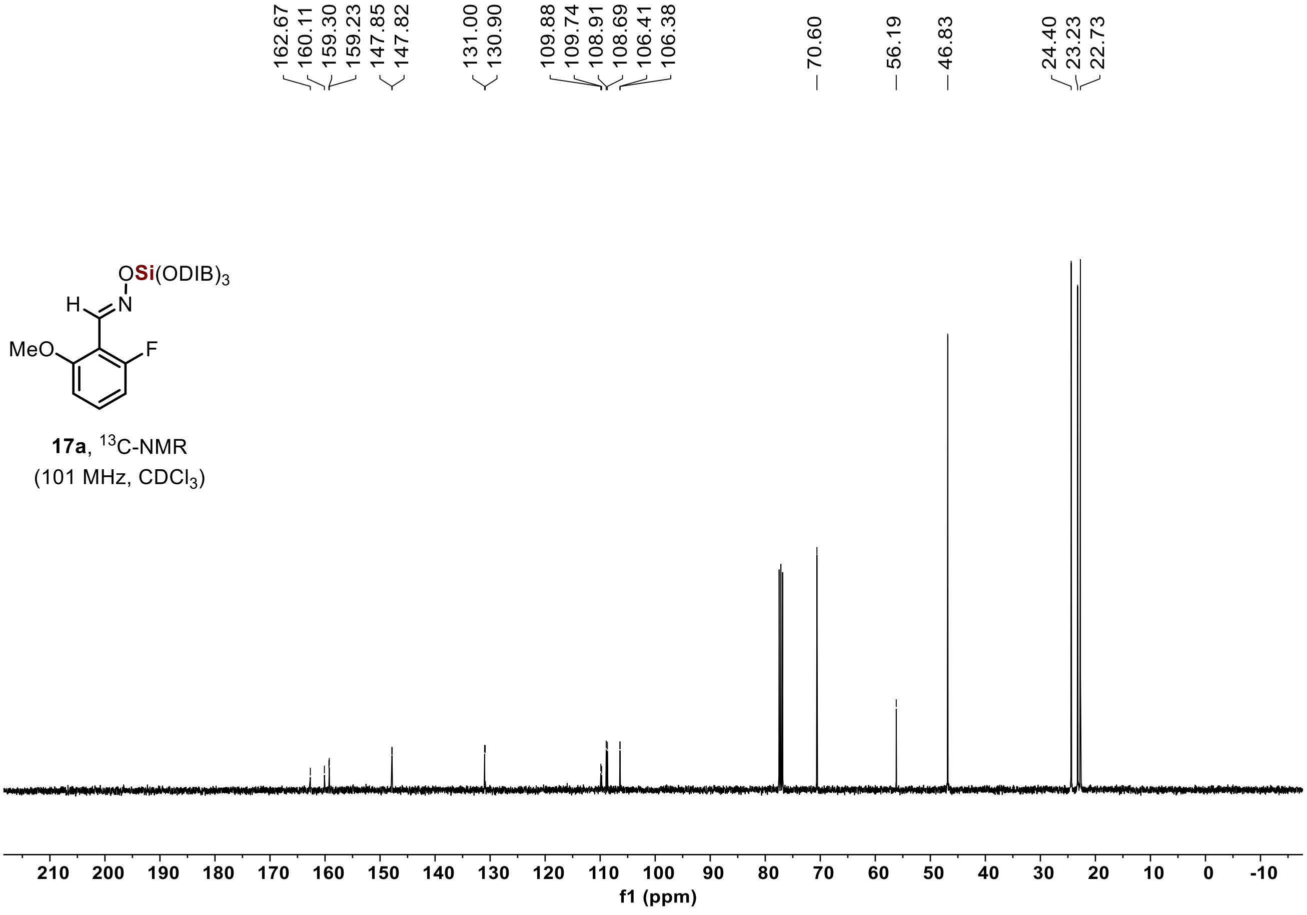

17a, ^{13}C-NMR
(101 MHz, $CDCl_3$)

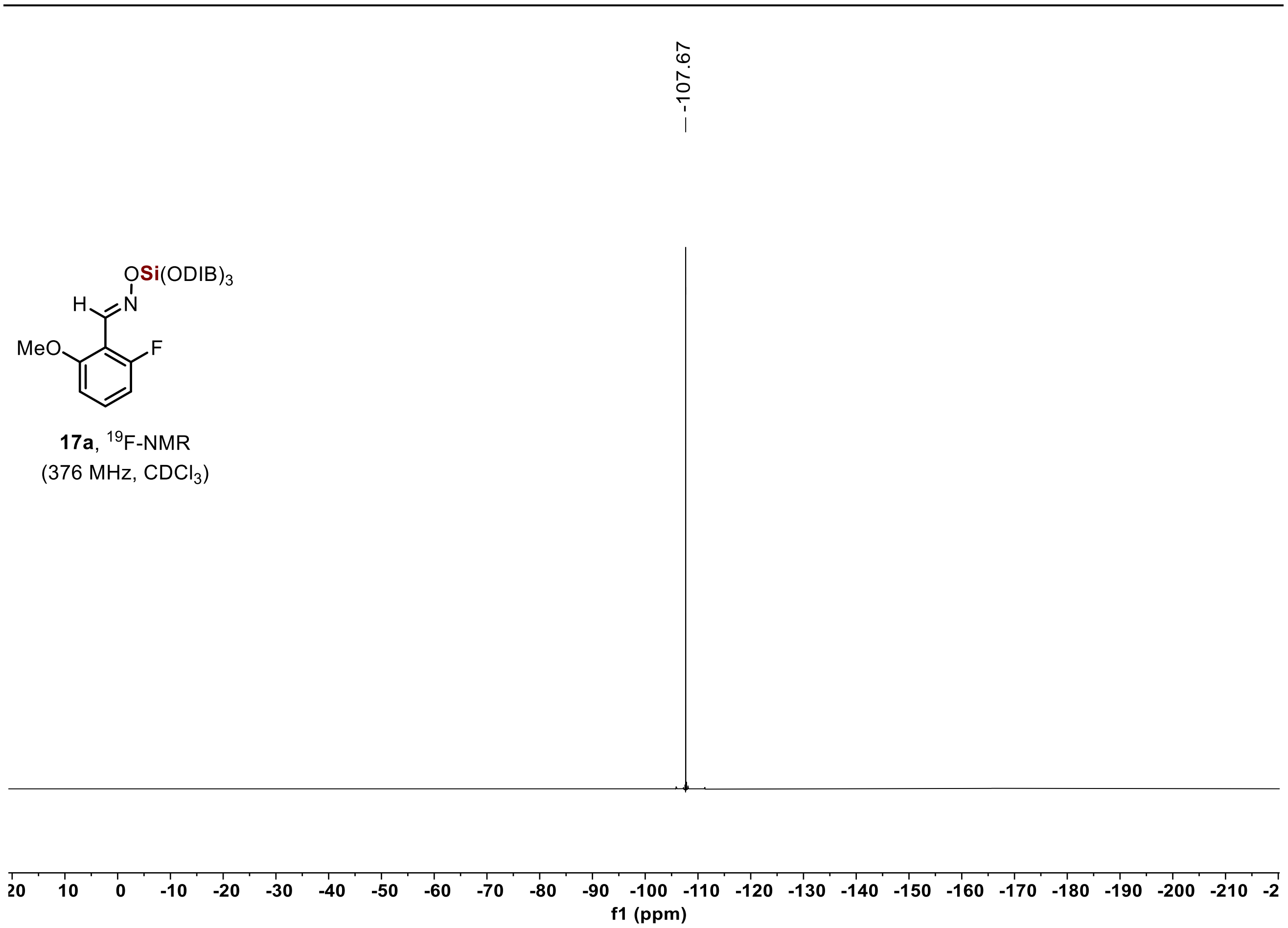

17a, ^{19}F-NMR
(376 MHz, $CDCl_3$)

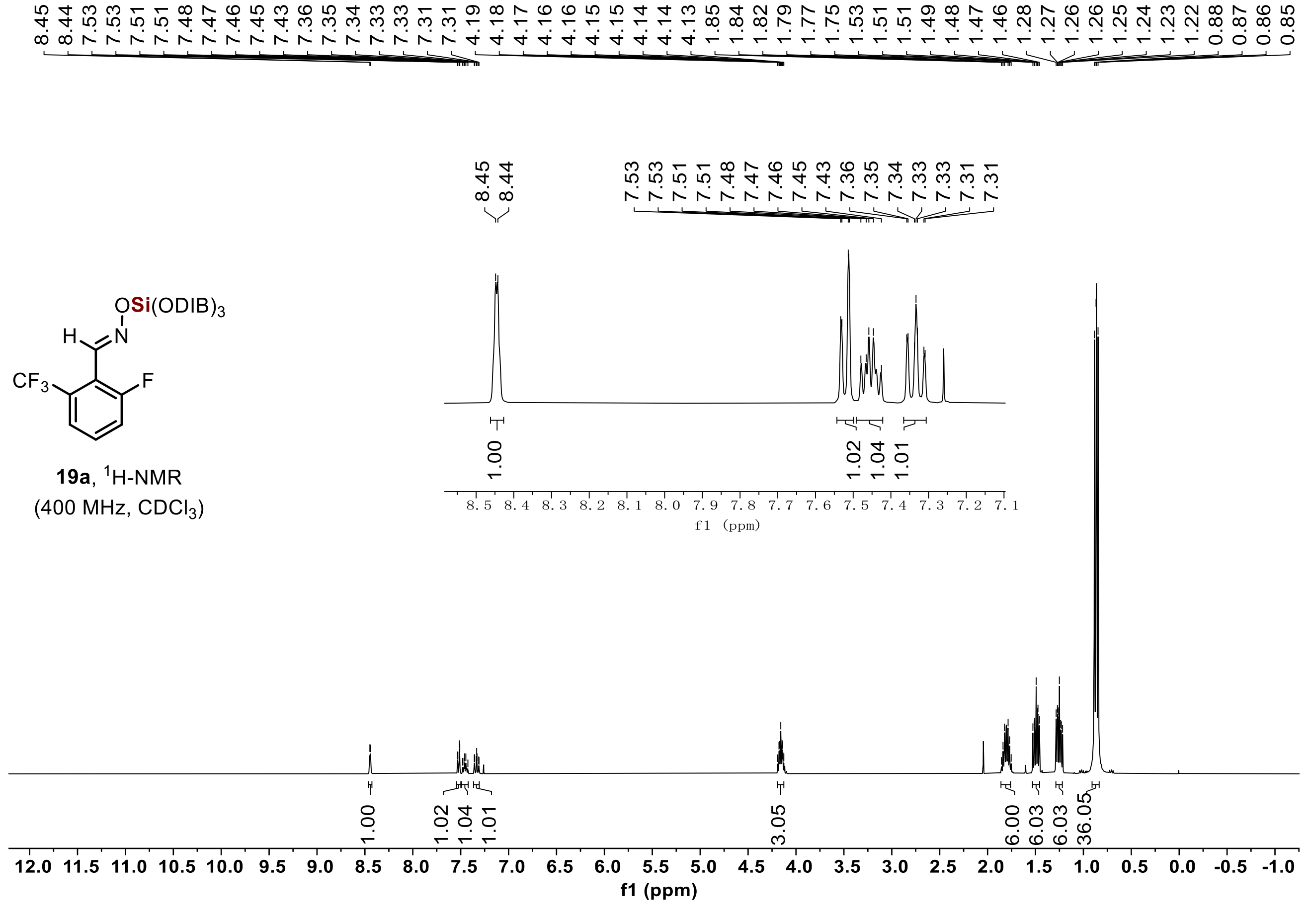

19a, ^{1}H-NMR
(400 MHz, $CDCl_3$)

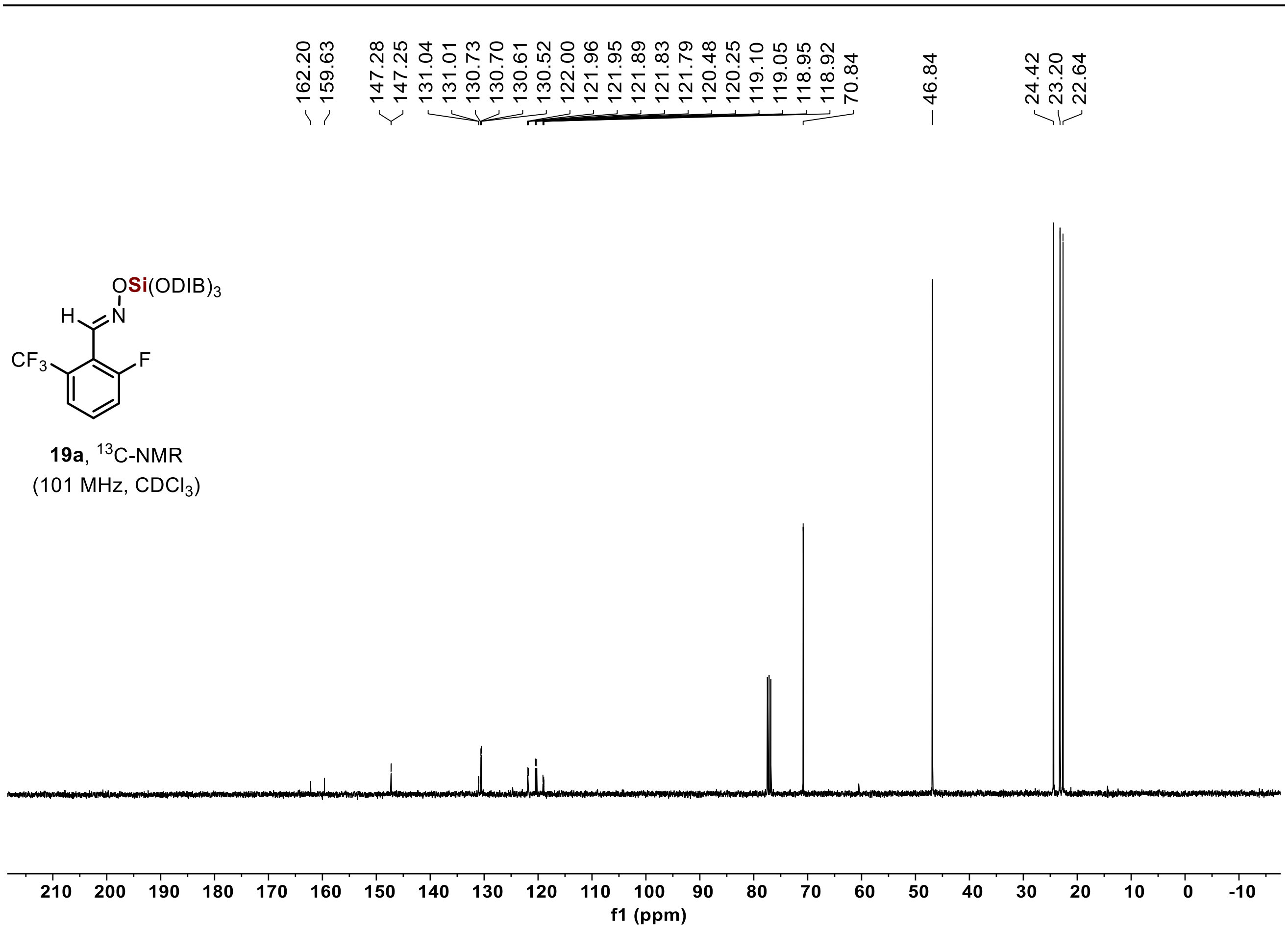
162.20
159.63
147.28
147.25
131.04
131.01
130.73
130.70
130.61
130.52
122.00
121.96
121.95
121.89
121.83
121.79
120.48
120.25
119.10
119.05
118.95
118.92
70.84
46.84
24.42
23.20
22.64
OSi(ODIB)3
H
N
CF3
F
19a, 13C-NMR
(101 MHz, CDCl3)
f1 (ppm)

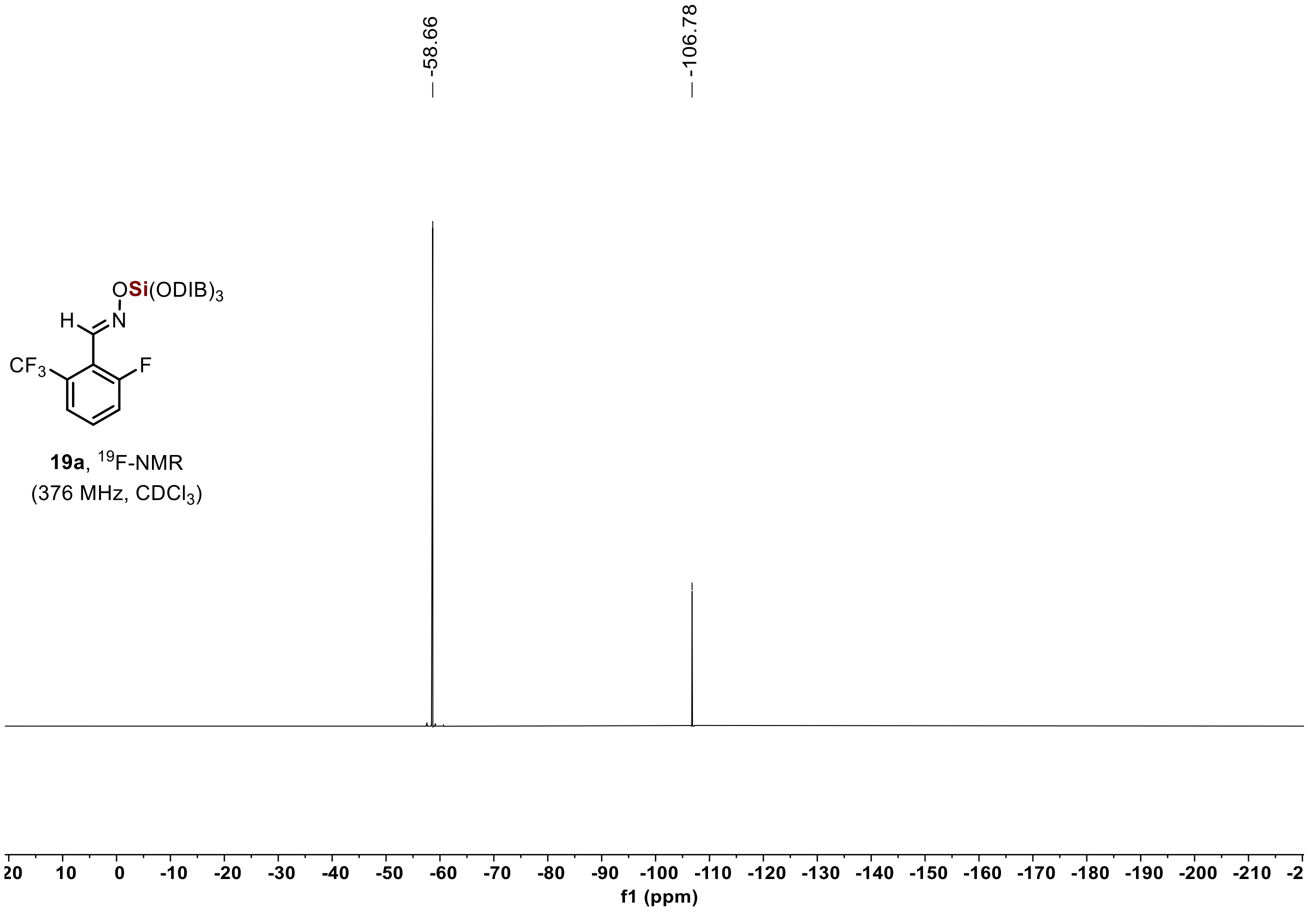
-58.66
-106.78
OSi(ODIB)3
H
N
CF3
F
19a, 19F-NMR
(376 MHz, CDCl3)
f1 (ppm)

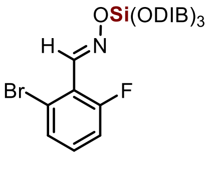

20a, ^{1}H-NMR
(400 MHz, $CDCl_3$)

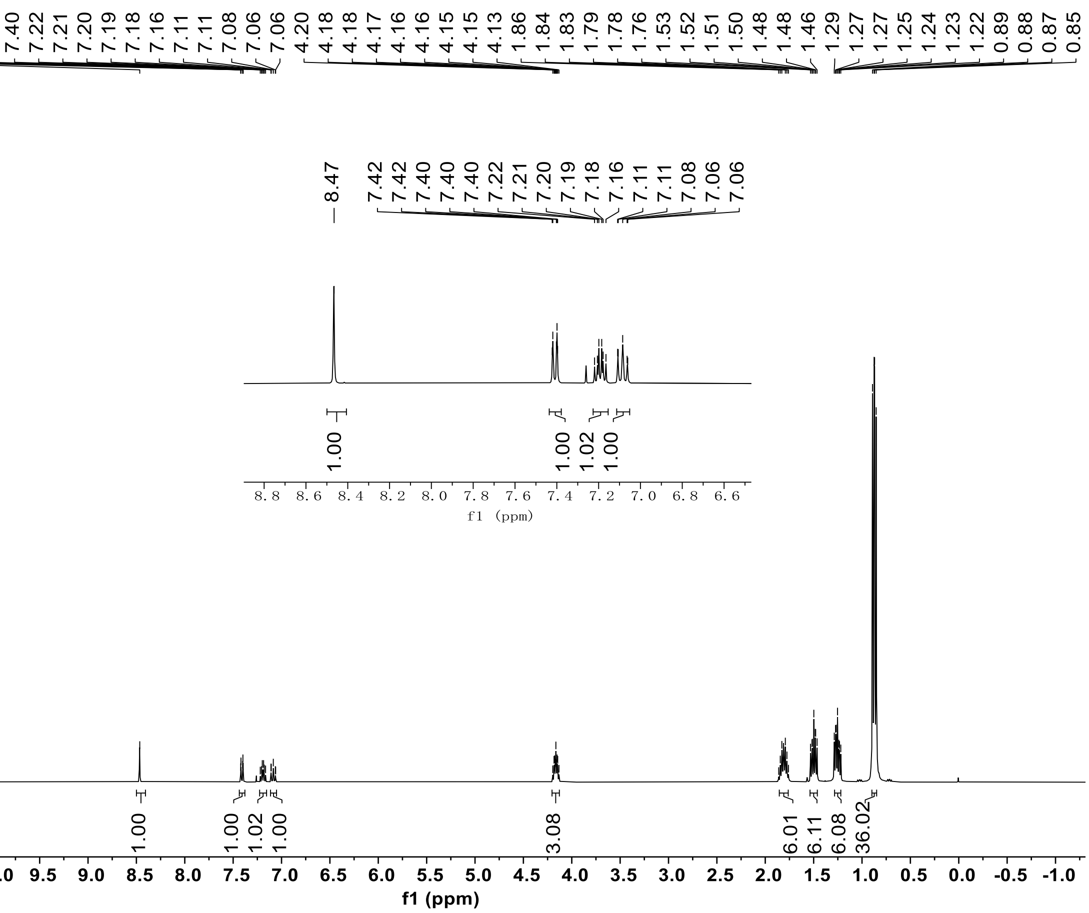

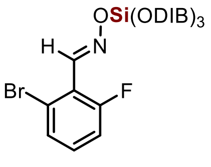

20a, ^{13}C-NMR
(101 MHz, $CDCl_3$)

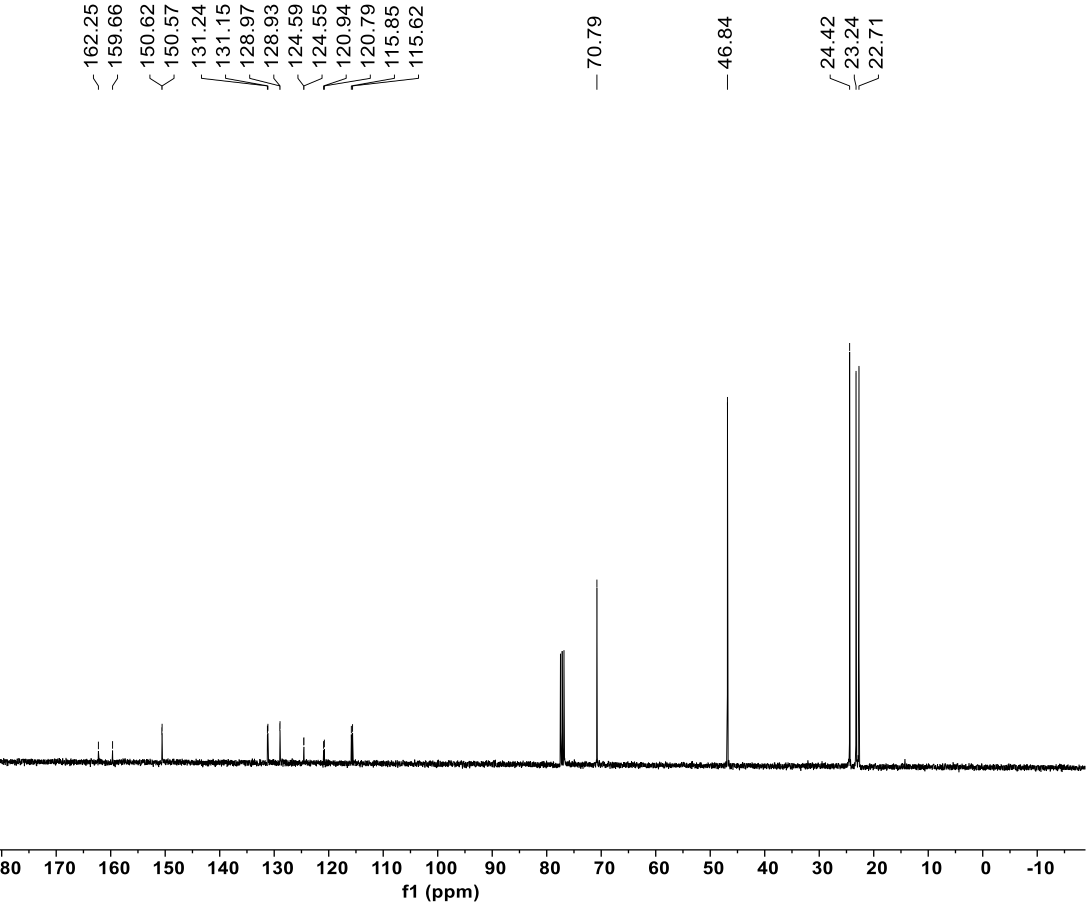

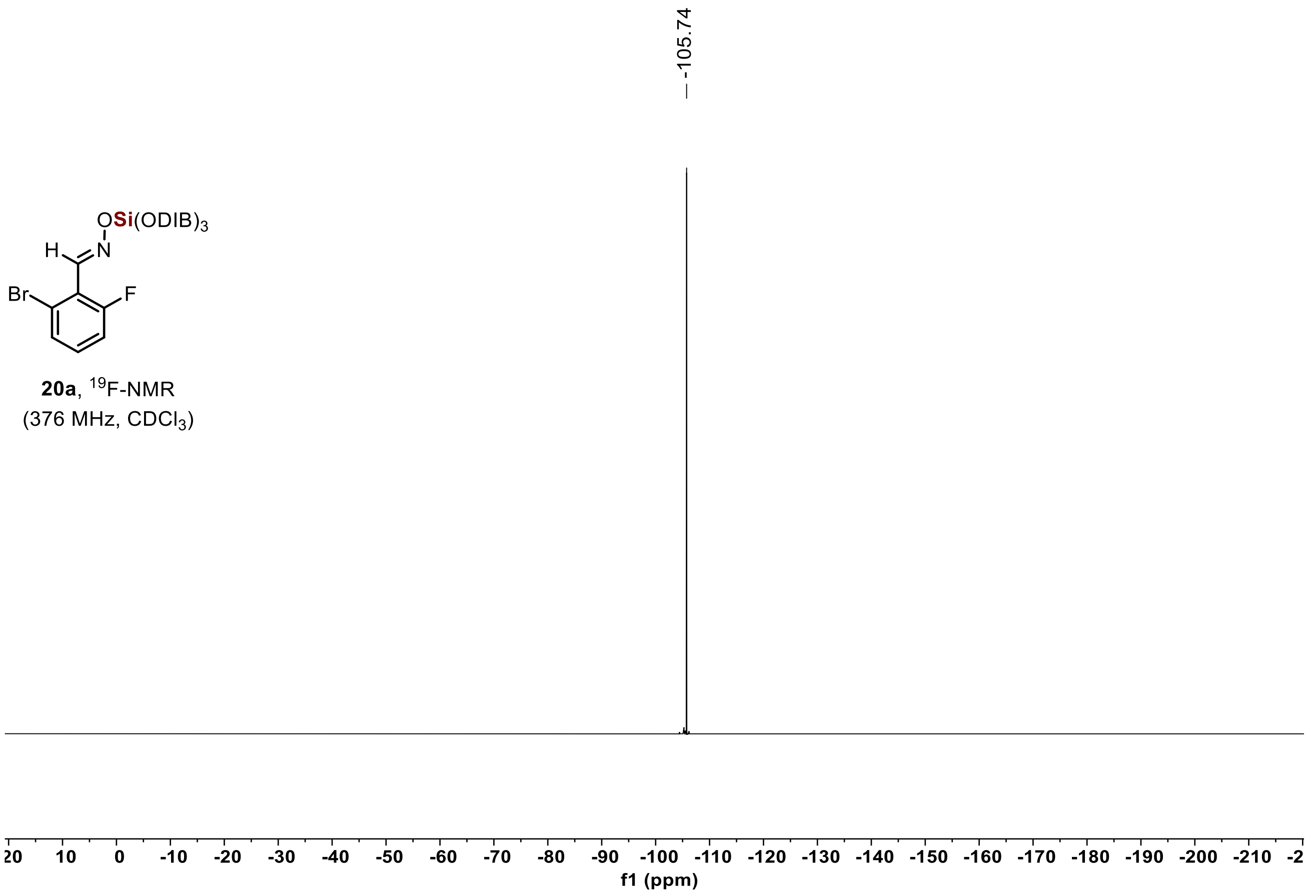

20a, ^{19}F-NMR
(376 MHz, $CDCl_3$)

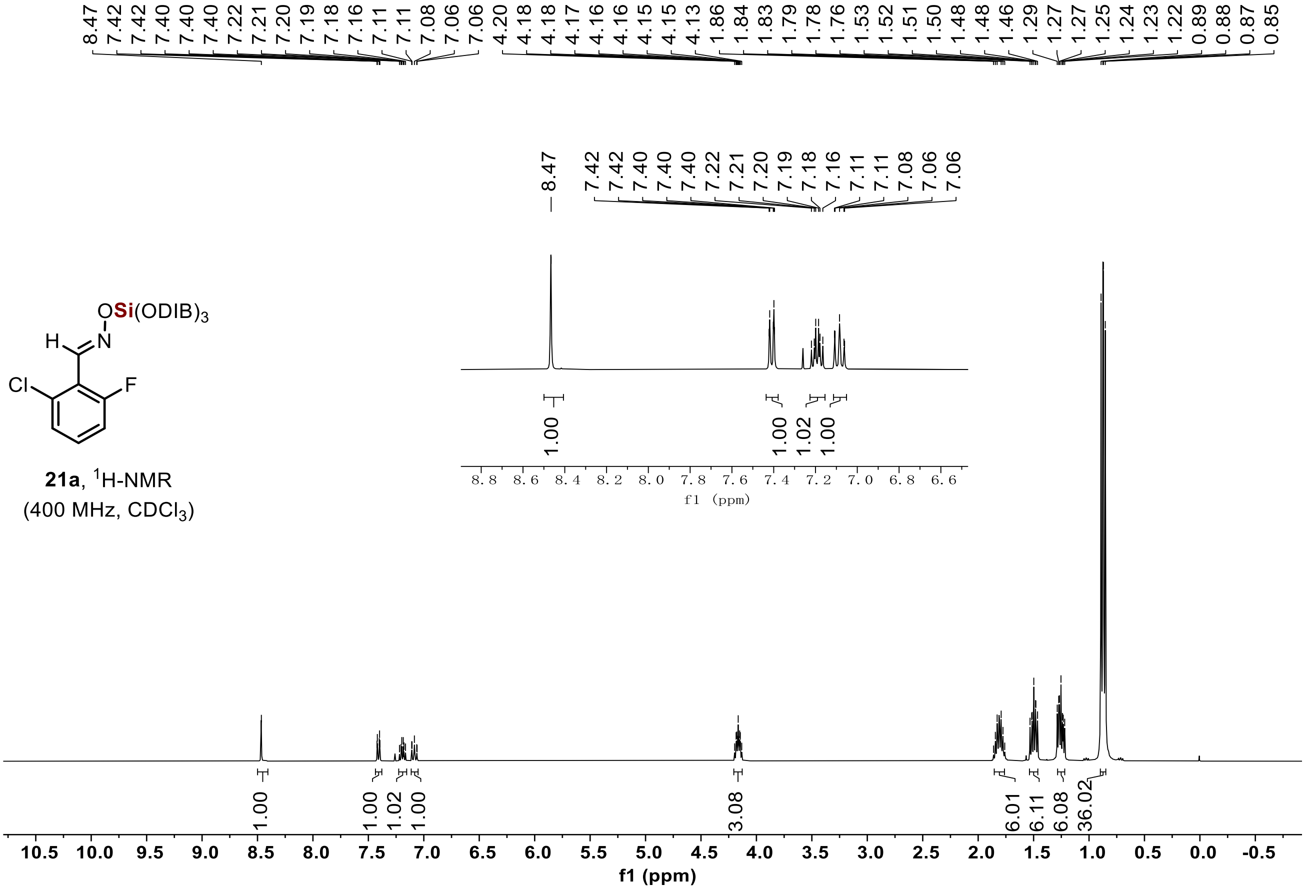

21a, ^{1}H-NMR
(400 MHz, $CDCl_3$)

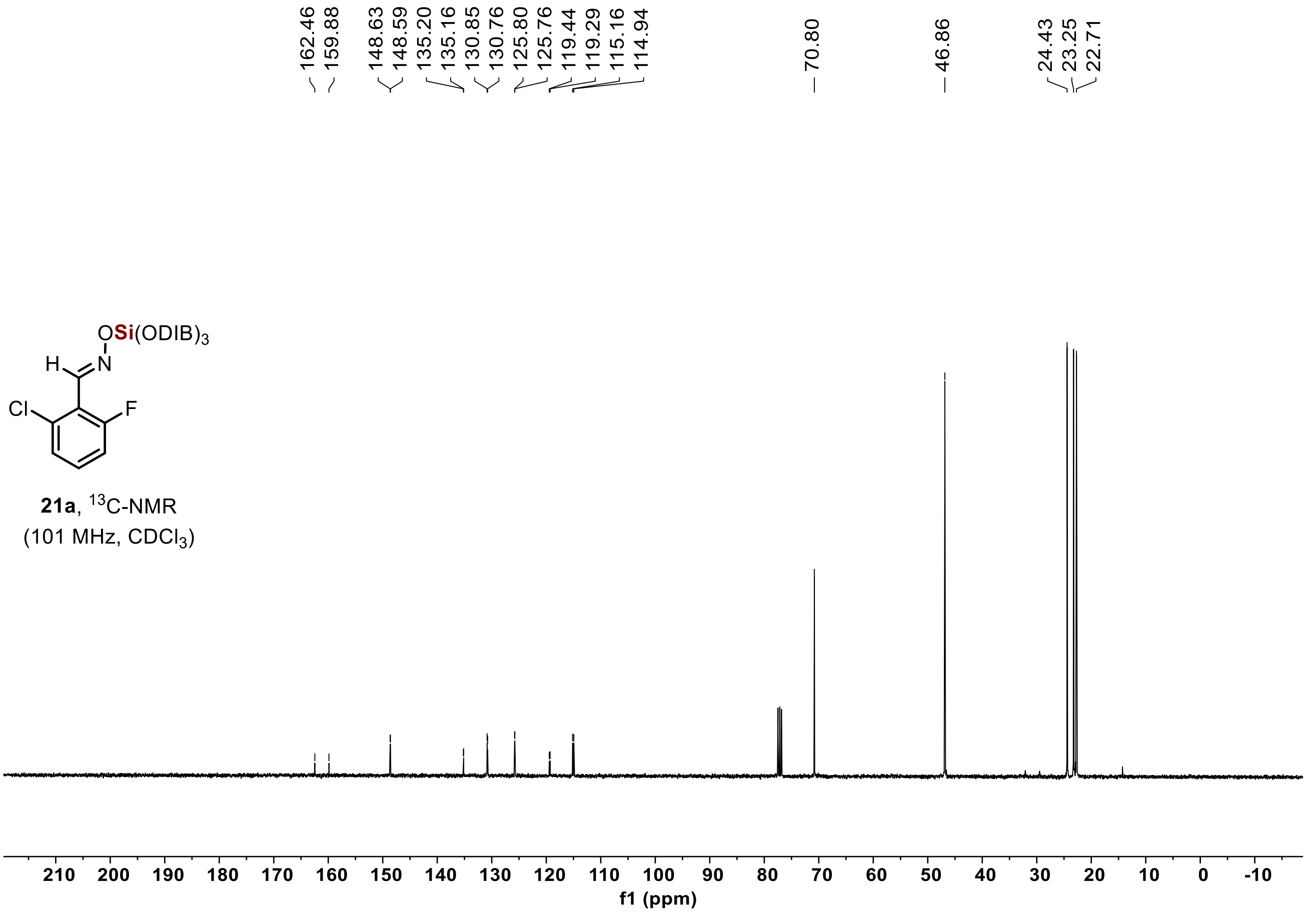

21a, ^{13}C-NMR
(101 MHz, $CDCl_3$)

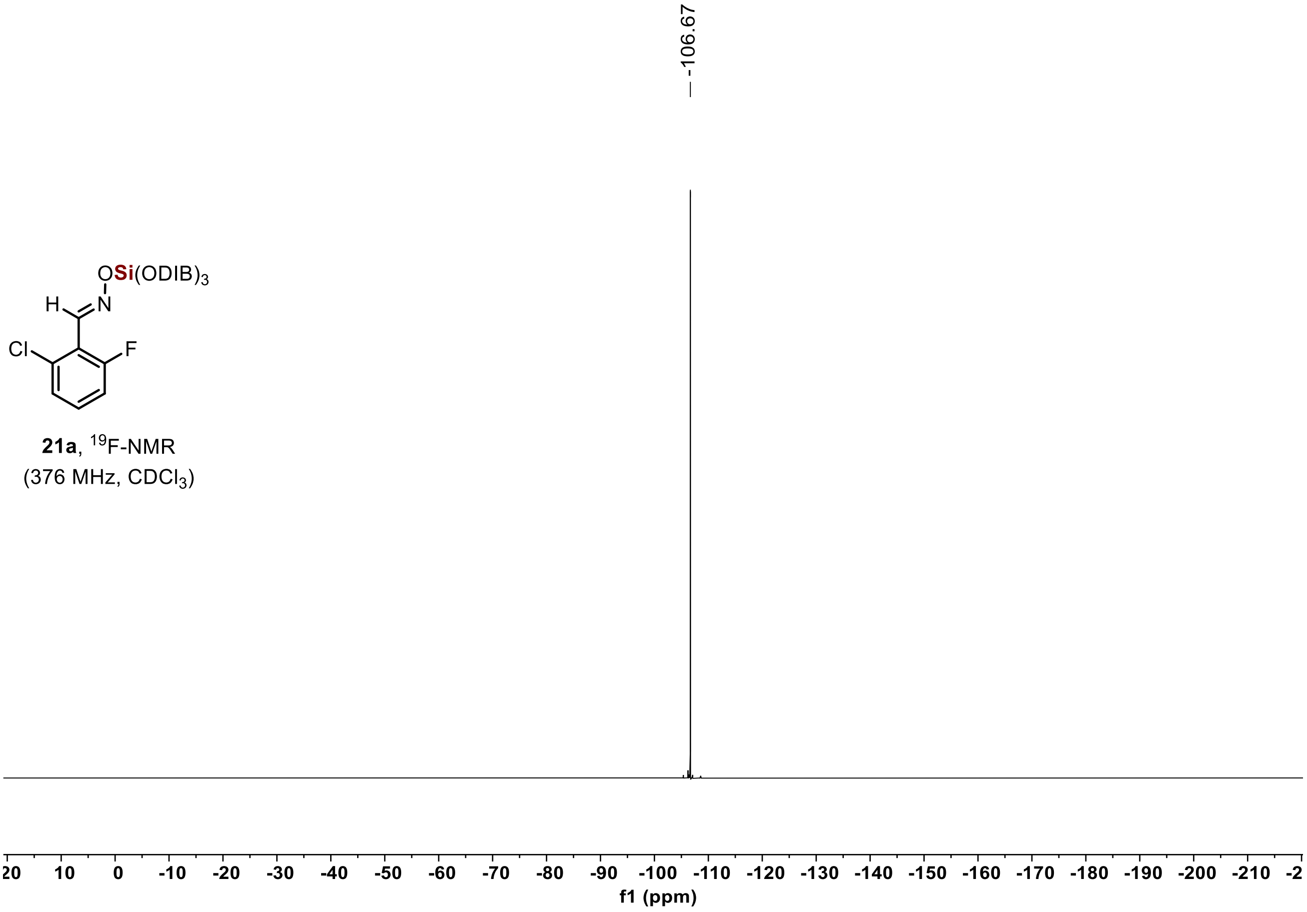

21a, ^{19}F-NMR
(376 MHz, $CDCl_3$)

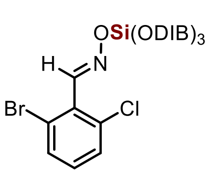

22a, ^{1}H-NMR
(400 MHz, $CDCl_3$)

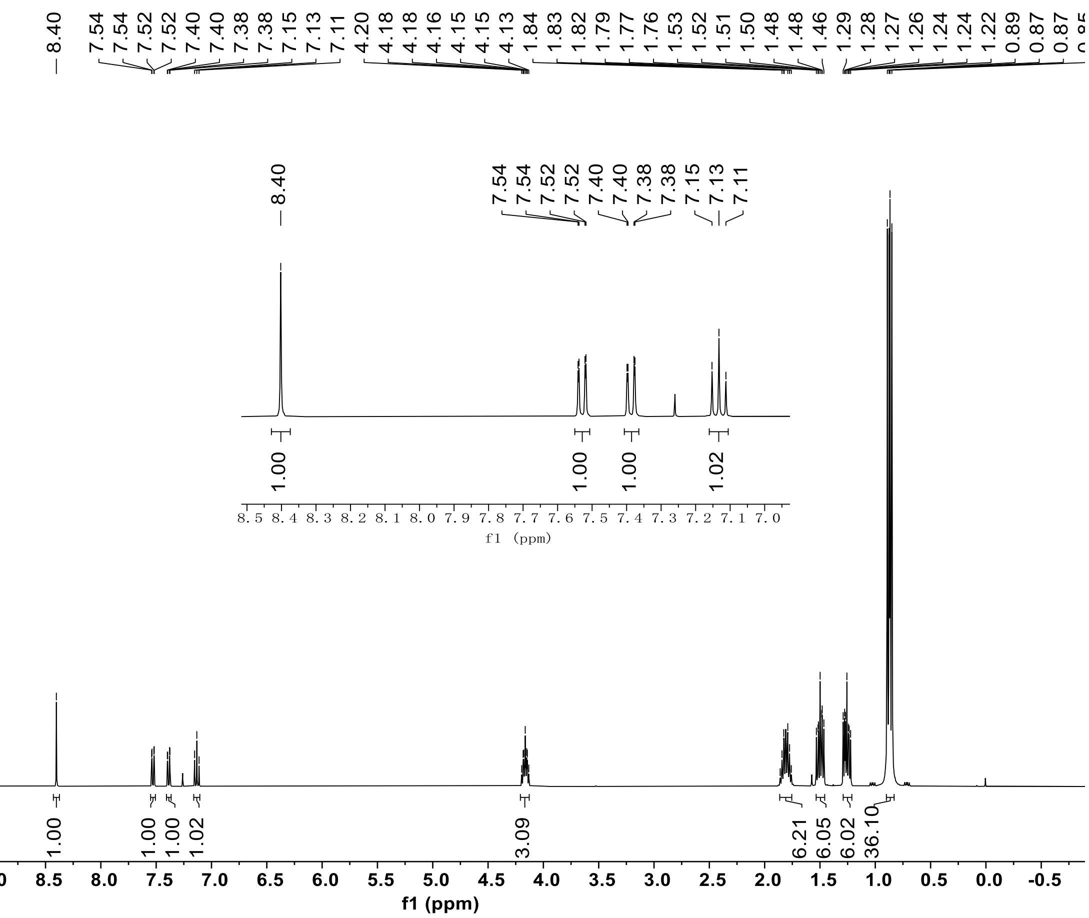

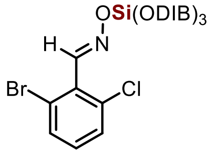

22a, ^{13}C-NMR
(101 MHz, $CDCl_3$)

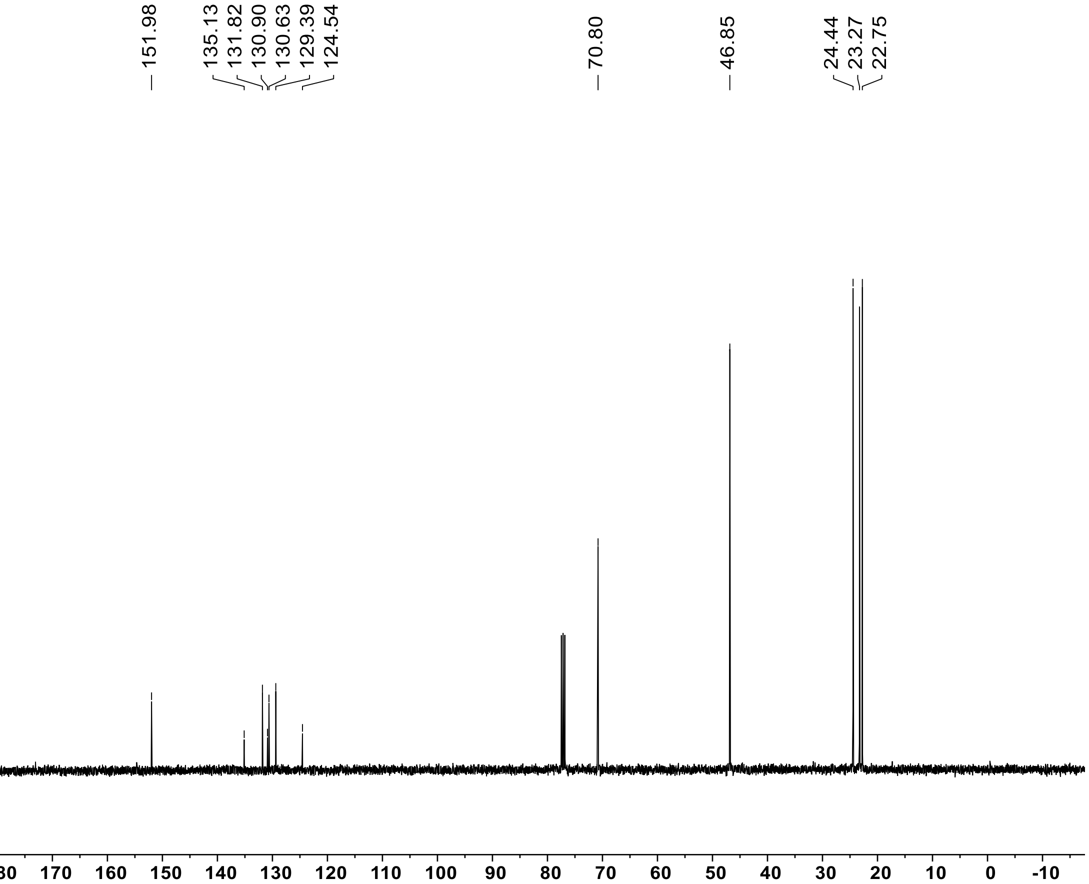

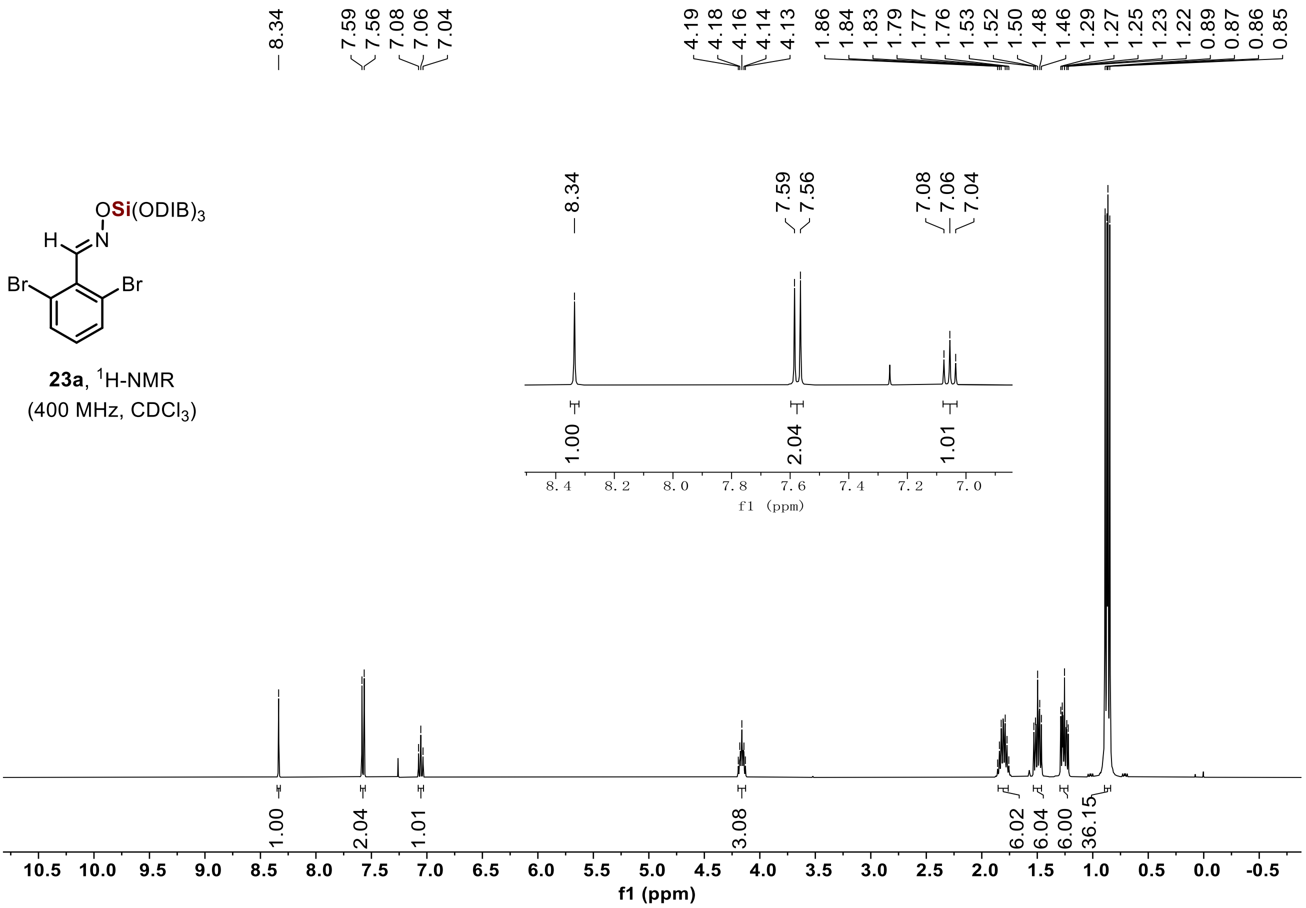
OSi(ODIB)3
H
N
Br
Br
23a, 1H-NMR
(400 MHz, CDCl3)
8.34
7.59
7.56
7.08
7.06
7.04
4.19
4.18
4.16
4.14
4.13
1.86
1.84
1.83
1.79
1.77
1.76
1.53
1.52
1.50
1.48
1.46
1.29
1.27
1.25
1.23
1.22
0.89
0.87
0.86
0.85
1.00
2.04
1.01
3.08
6.02
6.04
6.00
36.15
f1 (ppm)

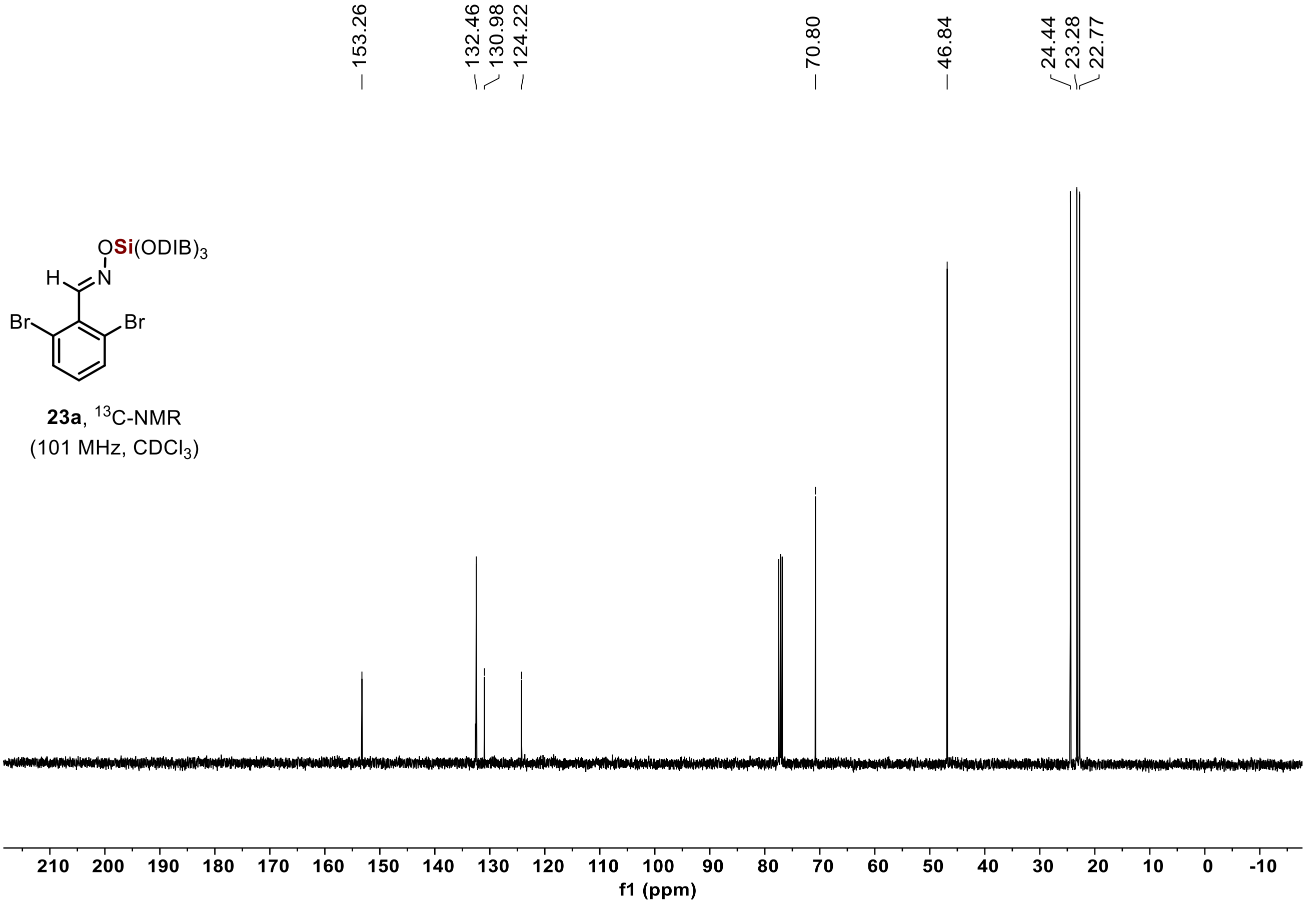
OSi(ODIB)3
H
N
Br
Br
23a, 13C-NMR
(101 MHz, CDCl3)
153.26
132.46
130.98
124.22
70.80
46.84
24.44
23.28
22.77
f1 (ppm)

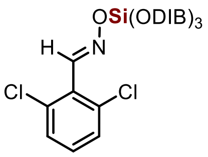

24a, ^{1}H-NMR
(400 MHz, $CDCl_3$)

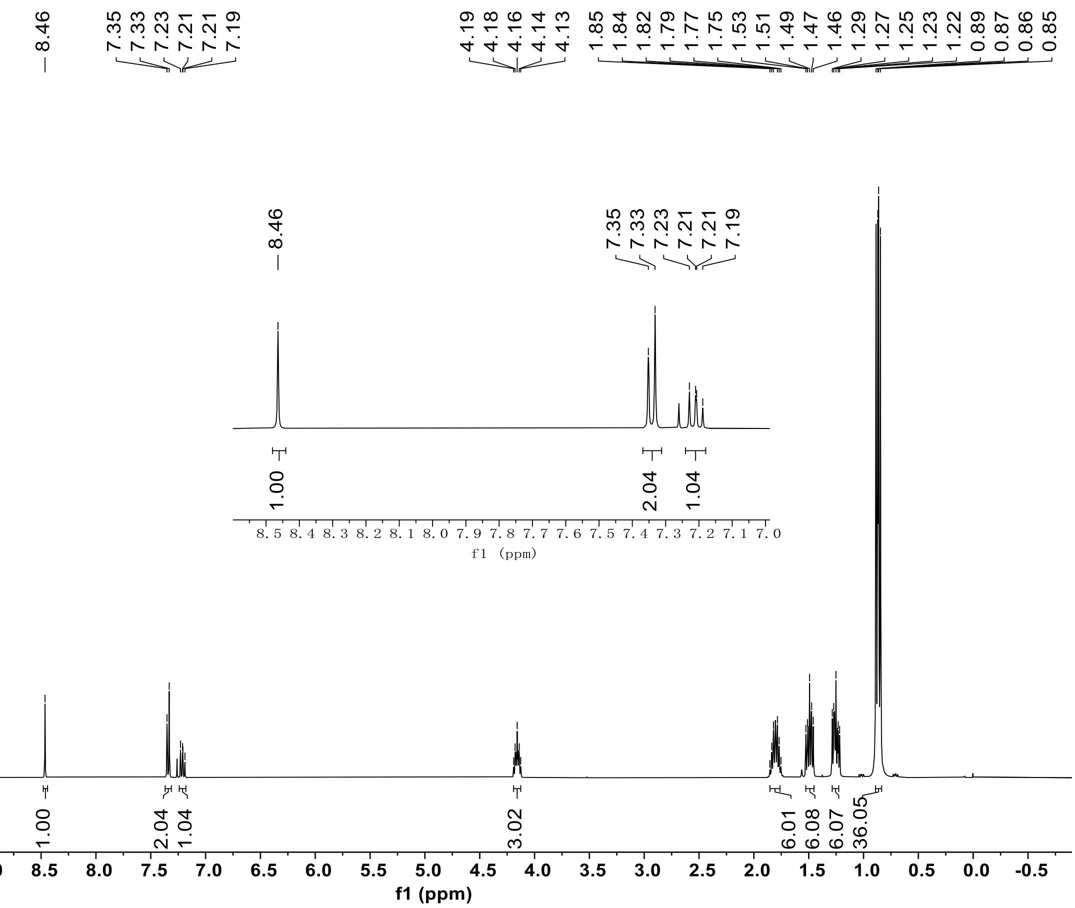

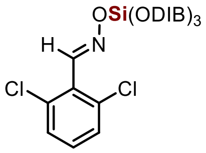

24a, ^{13}C-NMR
(101 MHz, $CDCl_3$)

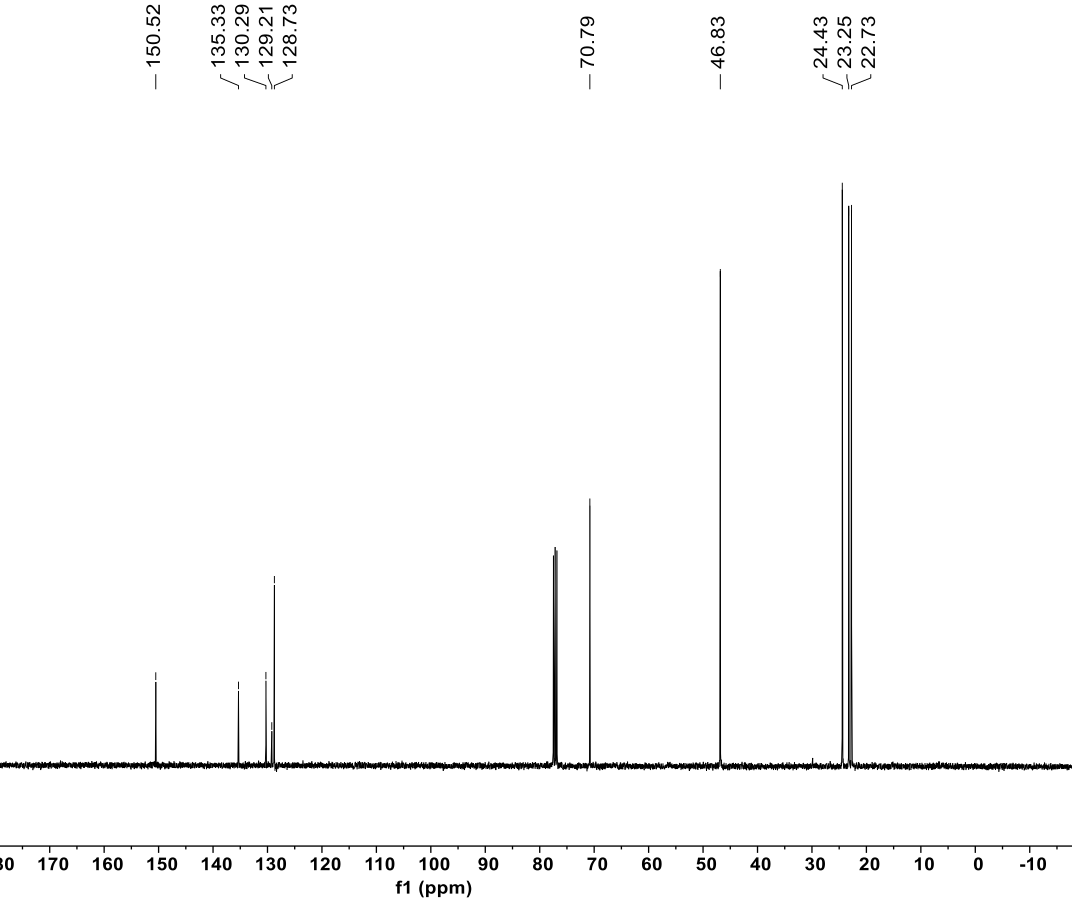

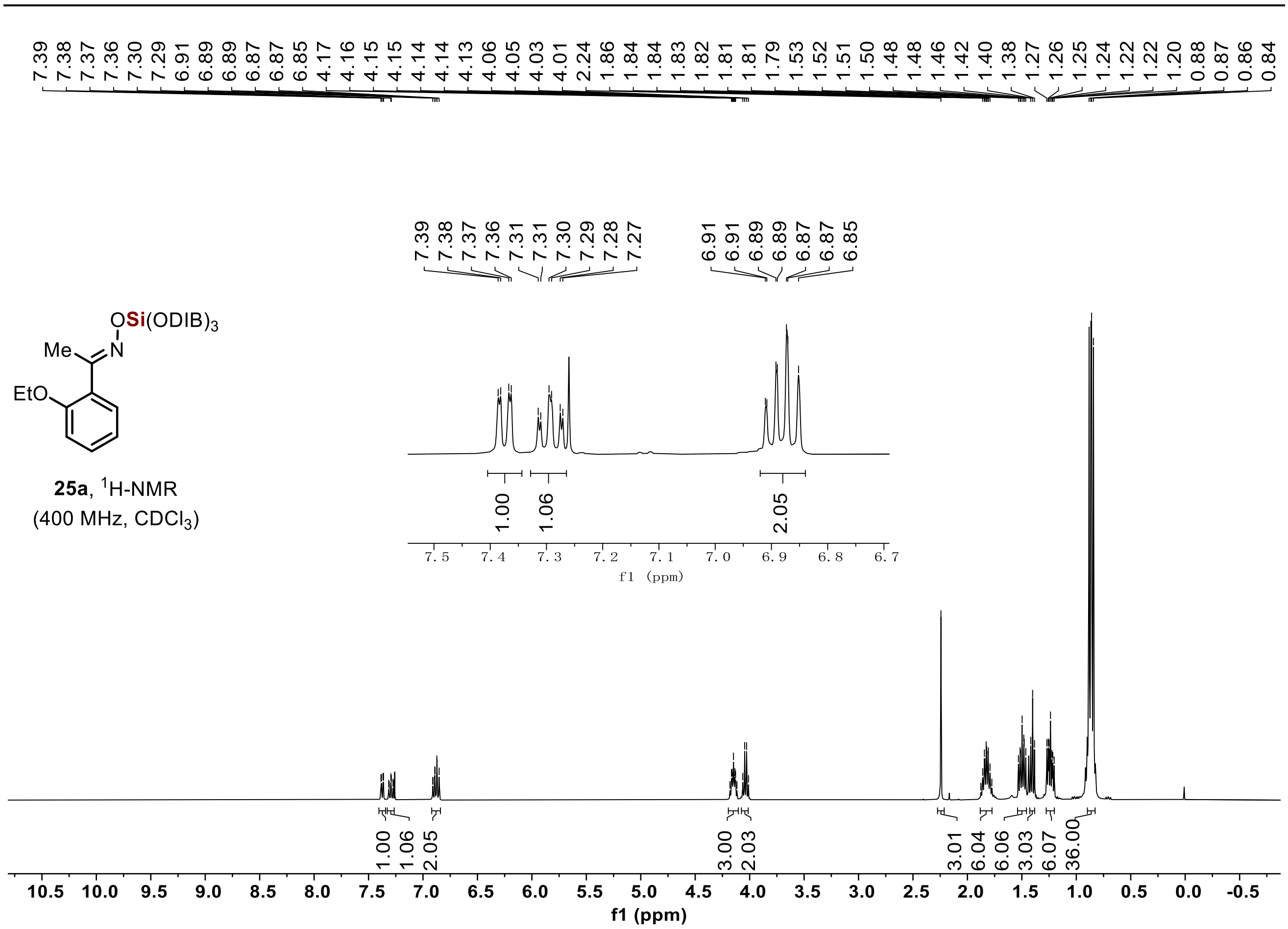
7.39 7.38 7.37 7.36 7.30 7.29 6.91 6.89 6.89 6.87 6.87 6.85 4.17 4.16 4.15 4.15 4.14 4.14 4.13 4.06 4.05 4.03 4.01 2.24 1.86 1.84 1.84 1.83 1.82 1.81 1.81 1.79 1.53 1.52 1.51 1.50 1.48 1.48 1.46 1.42 1.40 1.38 1.27 1.26 1.25 1.24 1.22 1.22 1.20 0.88 0.87 0.86 0.84
7.39 7.38 7.37 7.36 7.31 7.31 7.30 7.29 7.28 7.27
6.91 6.91 6.89 6.89 6.87 6.87 6.85
OSi(ODIB)3
Me
N
EtO
25a, 1H-NMR
(400 MHz, CDCl3)
1.00 1.06 2.05
7.5 7.4 7.3 7.2 7.1 7.0 6.9 6.8 6.7
f1 (ppm)
1.00 1.06 2.05 3.00 2.03 3.01 6.04 6.06 3.03 6.07 36.00
10.5 10.0 9.5 9.0 8.5 8.0 7.5 7.0 6.5 6.0 5.5 5.0 4.5 4.0 3.5 3.0 2.5 2.0 1.5 1.0 0.5 0.0 -0.5
f1 (ppm)

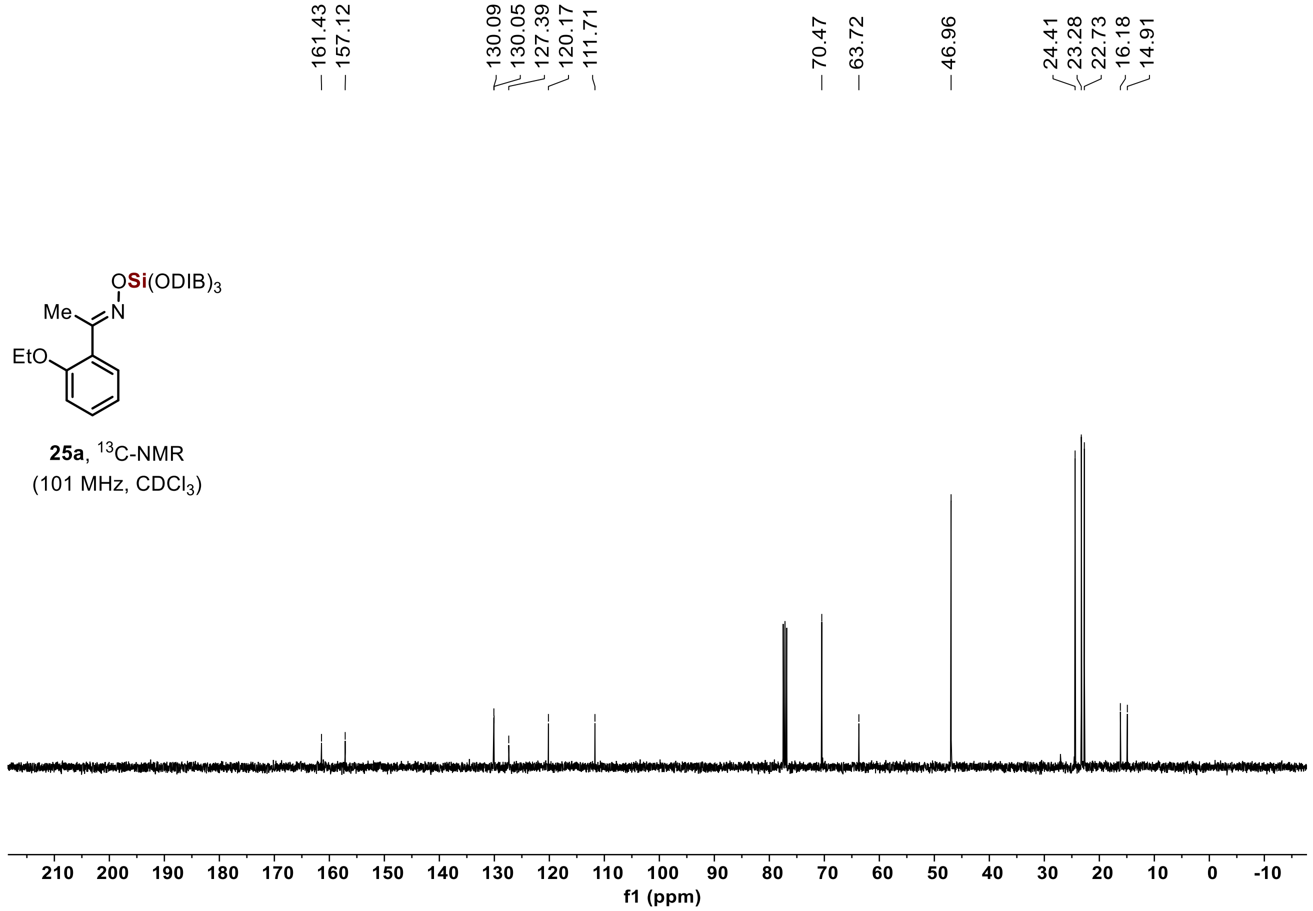
161.43 157.12 130.09 130.05 127.39 120.17 111.71 70.47 63.72 46.96 24.41 23.28 22.73 16.18 14.91
OSi(ODIB)3
Me
N
EtO
25a, 13C-NMR
(101 MHz, CDCl3)
210 200 190 180 170 160 150 140 130 120 110 100 90 80 70 60 50 40 30 20 10 0 -10
f1 (ppm)

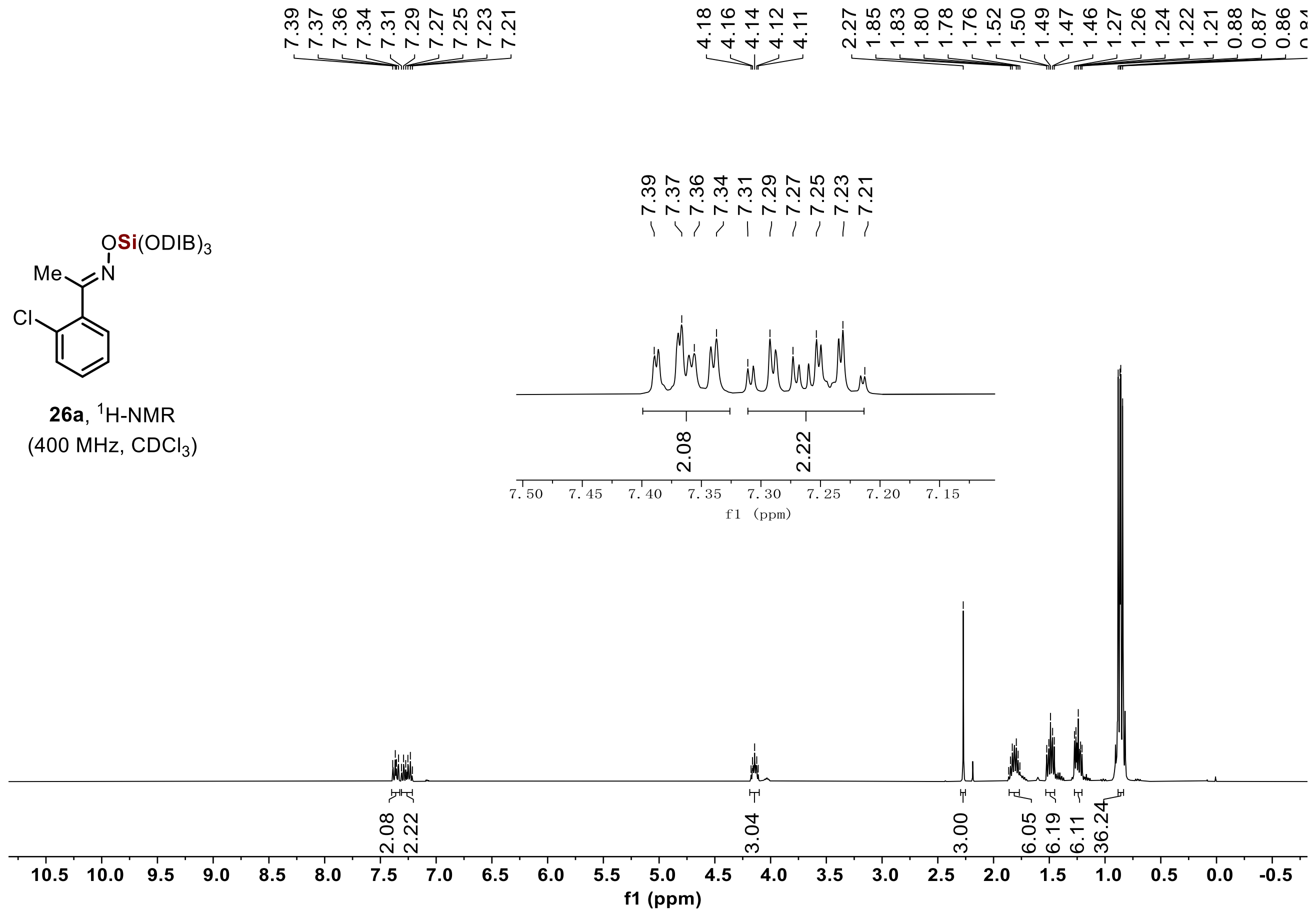

26a, ^{1}H-NMR
(400 MHz, $CDCl_3$)

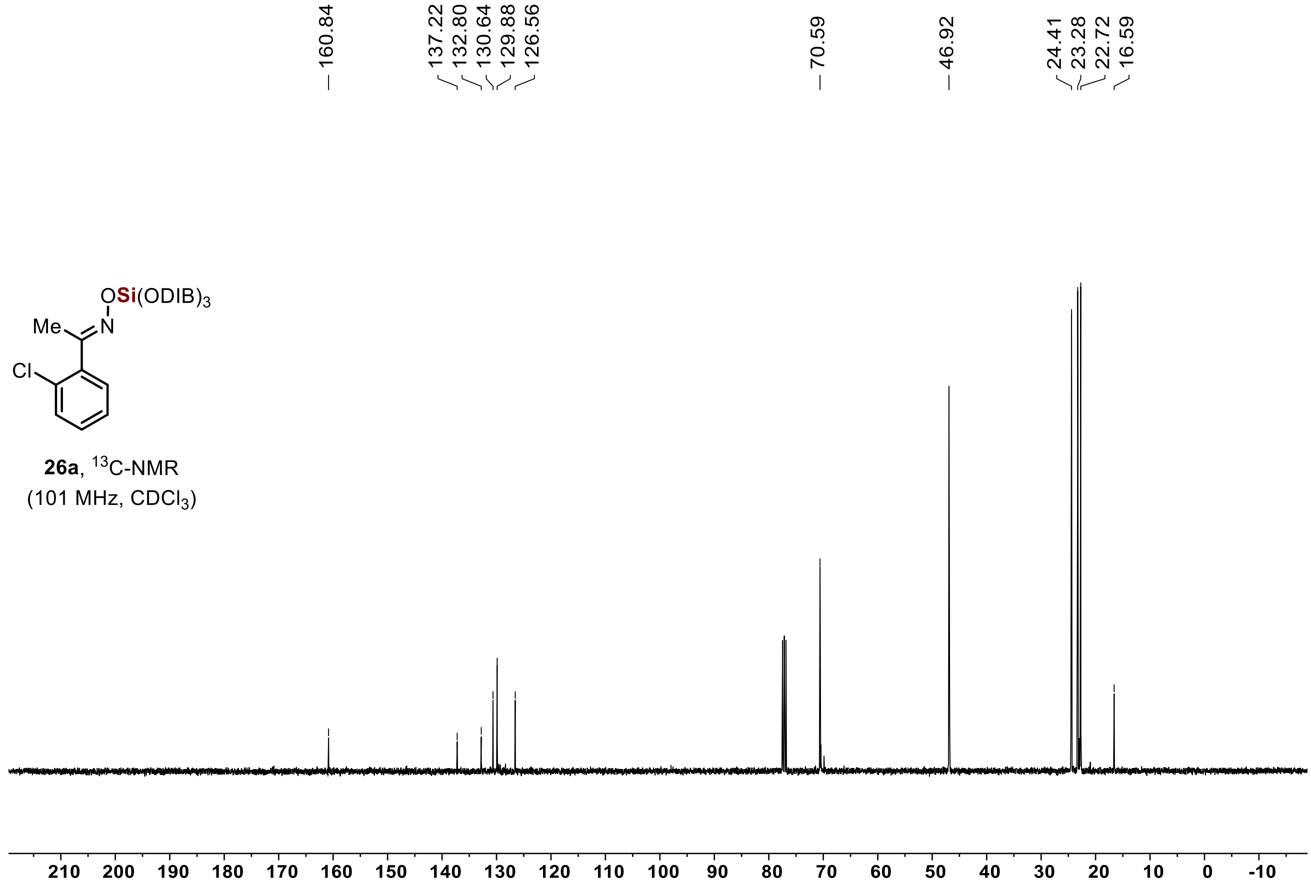

26a, ^{13}C-NMR
(101 MHz, $CDCl_3$)

27a, ^{1}H-NMR
(400 MHz, $CDCl_3$)

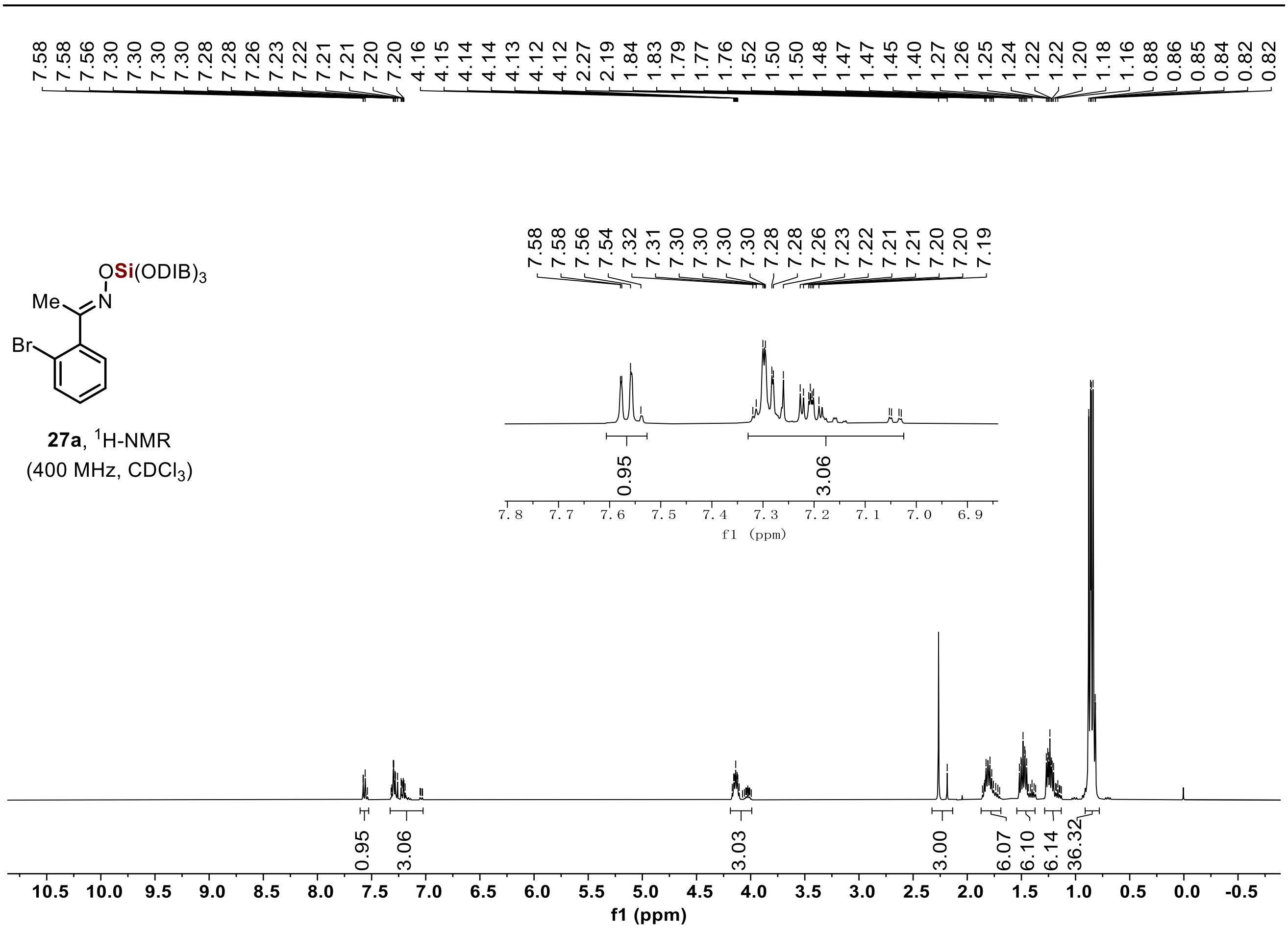

27a, ^{13}C-NMR
(101 MHz, $CDCl_3$)

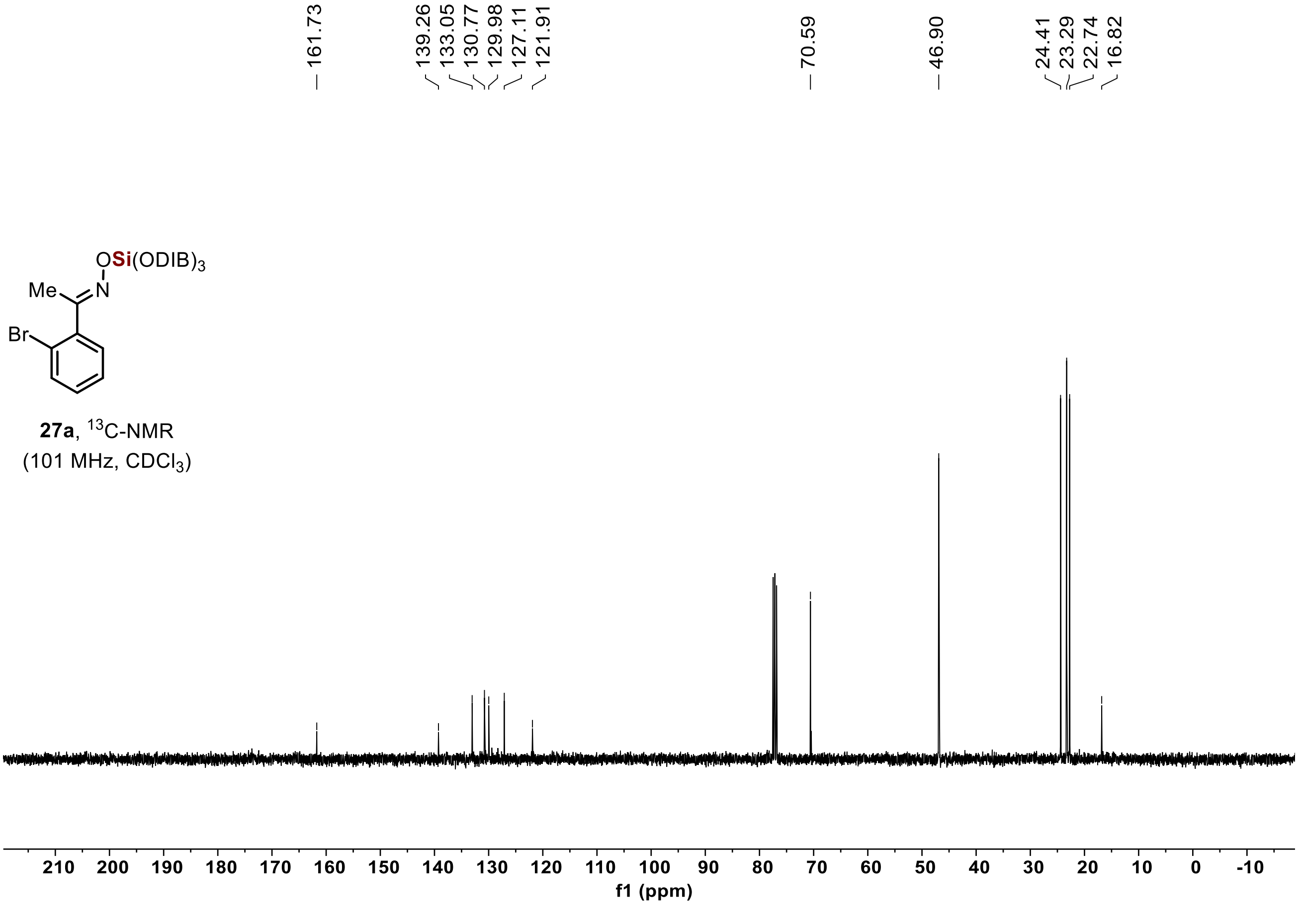

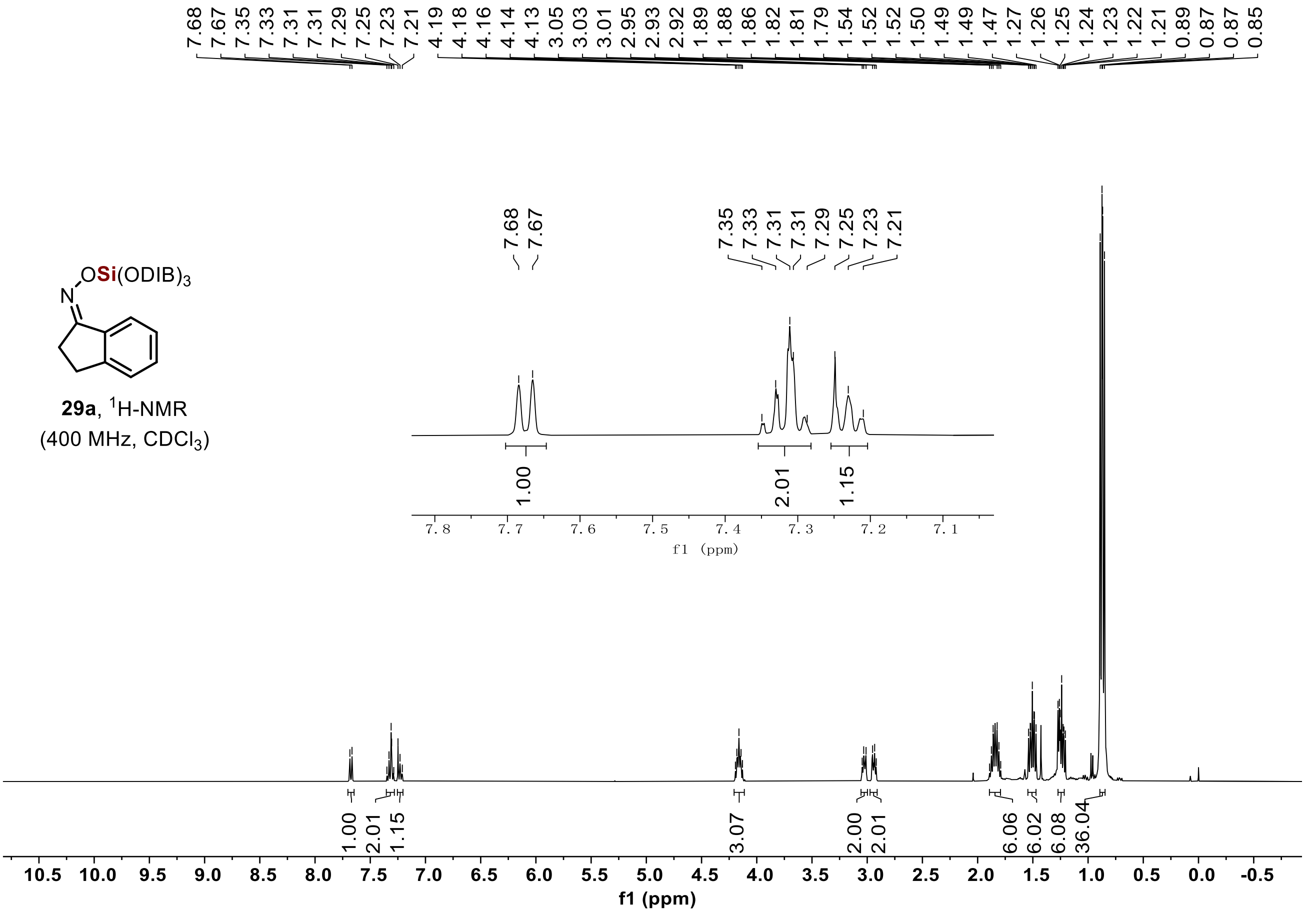
OSi(ODIB)3
N
29a, 1H-NMR
(400 MHz, CDCl3)
7.68 7.67 7.35 7.33 7.31 7.31 7.29 7.25 7.23 7.21 4.19 4.18 4.16 4.14 4.13 3.05 3.03 3.01 2.95 2.93 2.92 1.89 1.88 1.86 1.82 1.81 1.79 1.54 1.52 1.52 1.50 1.49 1.49 1.47 1.27 1.26 1.25 1.24 1.23 1.22 1.21 0.89 0.87 0.87 0.85
1.00 2.01 1.15 3.07 2.00 2.01 6.06 6.02 6.08 36.04
f1 (ppm)

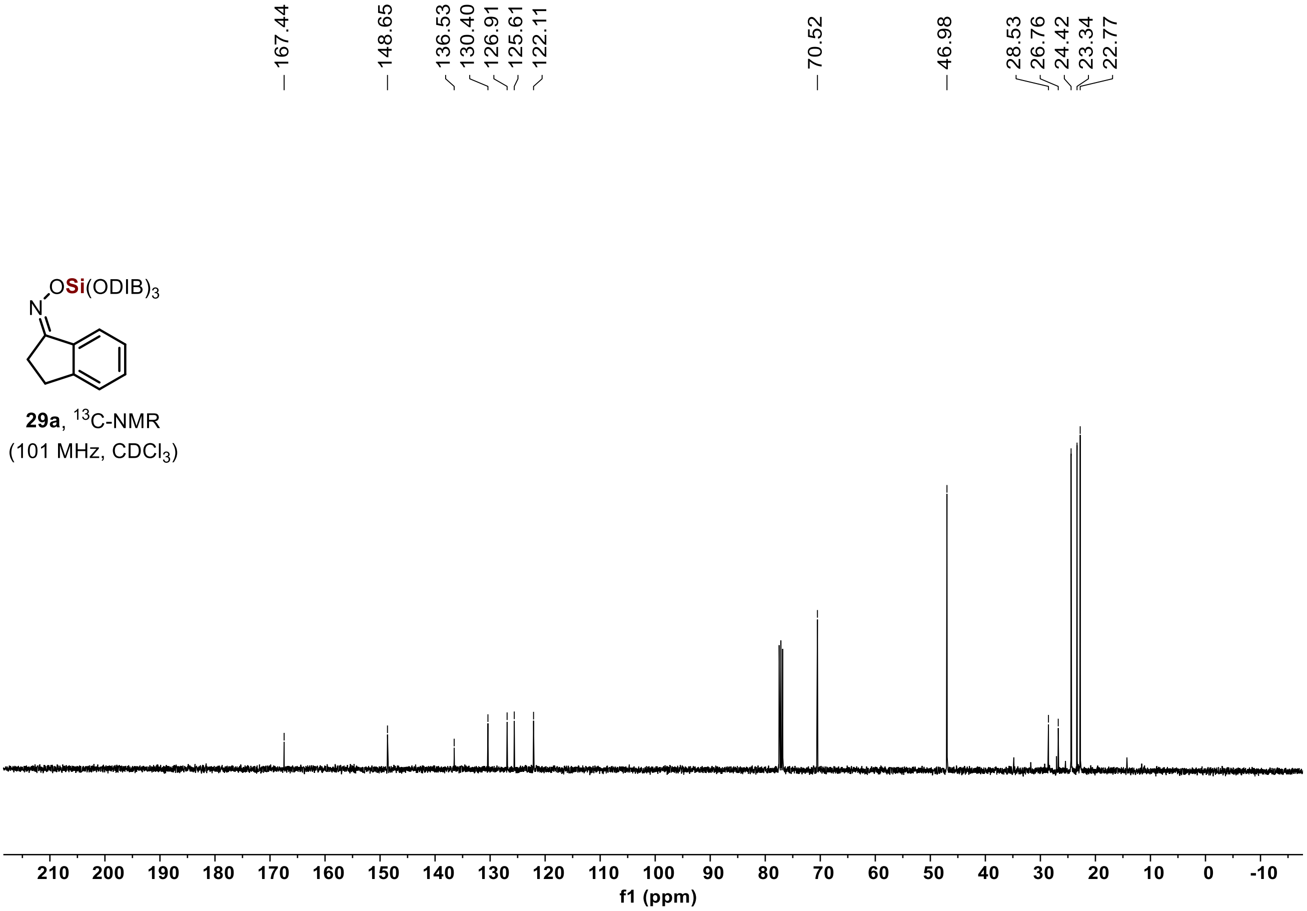
OSi(ODIB)3
N
29a, 13C-NMR
(101 MHz, CDCl3)
167.44 148.65 136.53 130.40 126.91 125.61 122.11 70.52 46.98 28.53 26.76 24.42 23.34 22.77
f1 (ppm)

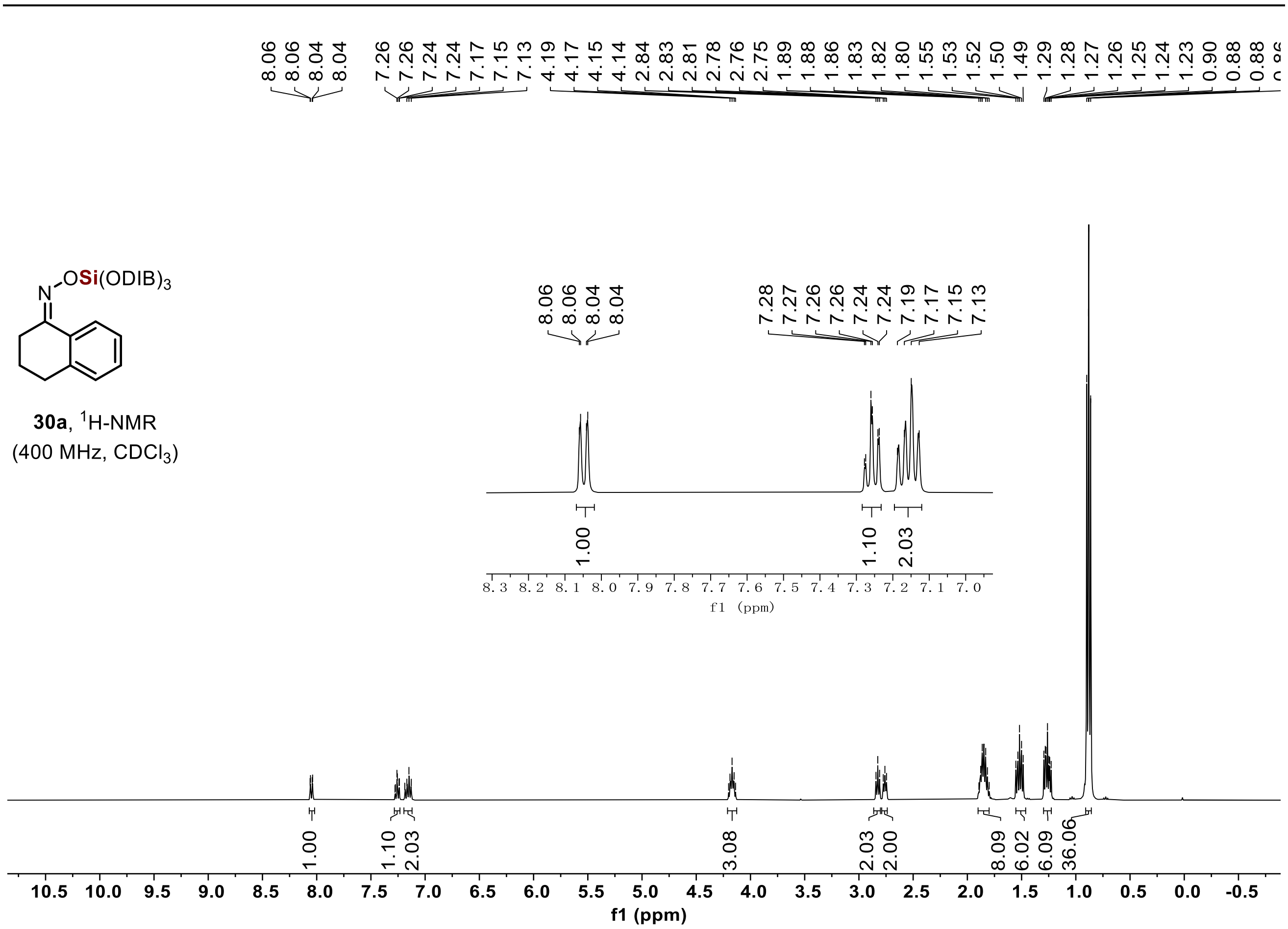
8.06 8.06 8.04 8.04 7.26 7.26 7.24 7.24 7.17 7.15 7.13 4.19 4.17 4.15 4.14 2.84 2.83 2.81 2.78 2.76 2.75 1.89 1.88 1.86 1.83 1.82 1.80 1.55 1.53 1.52 1.50 1.49 1.29 1.28 1.27 1.26 1.25 1.24 1.23 0.90 0.88 0.88
OSi(ODIB)3
30a, 1H-NMR
(400 MHz, CDCl3)
8.06 8.06 8.04 8.04
7.28 7.27 7.26 7.26 7.24 7.24 7.19 7.17 7.15 7.13
1.00 1.10 2.03
f1 (ppm)
1.00 1.10 2.03 3.08 2.03 2.00 8.09 6.02 6.09 36.06
f1 (ppm)

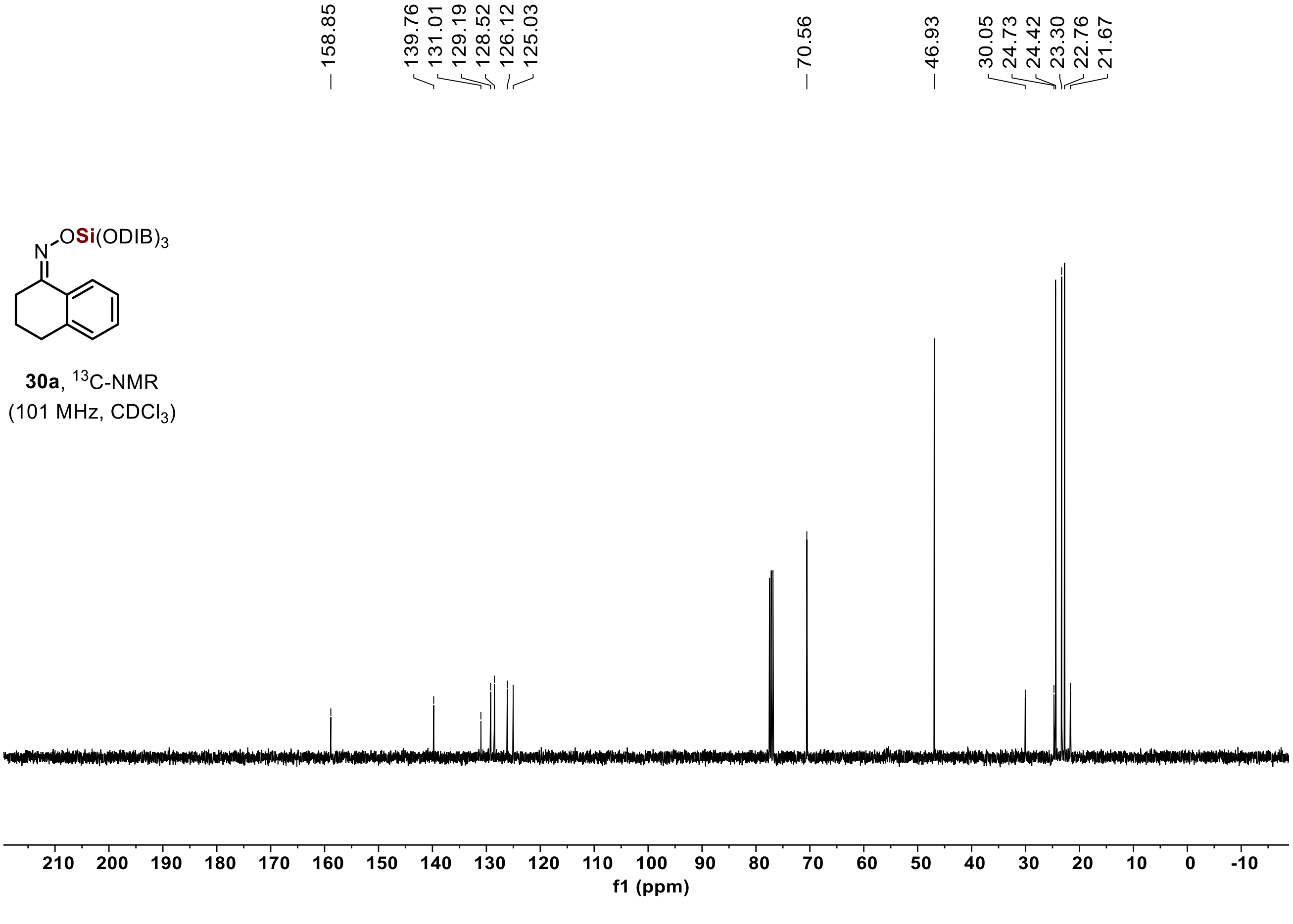
158.85 139.76 131.01 129.19 128.52 126.12 125.03 70.56 46.93 30.05 24.73 24.42 23.30 22.76 21.67
OSi(ODIB)3
30a, 13C-NMR
(101 MHz, CDCl3)
f1 (ppm)

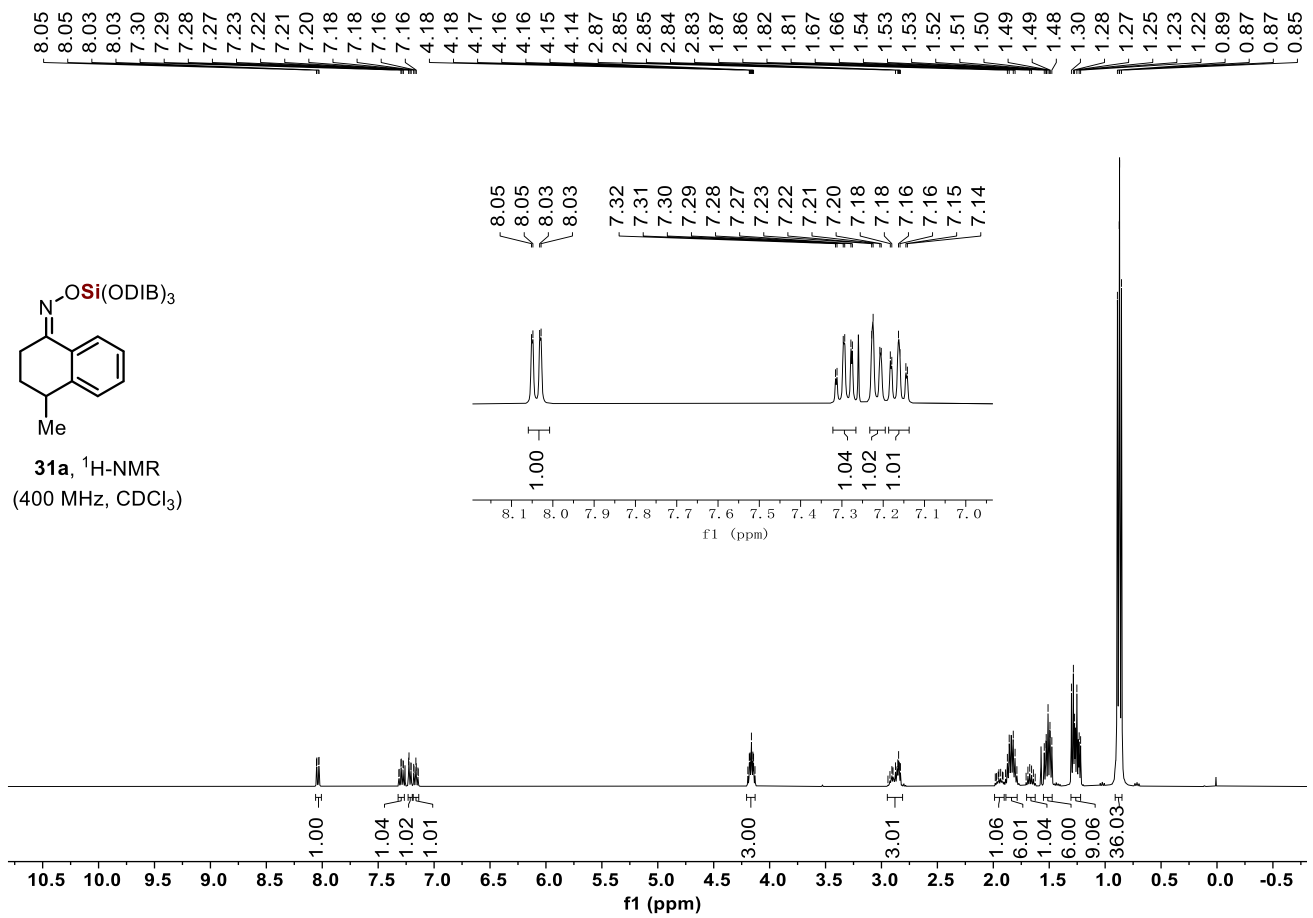

8.05 8.05 8.03 8.03 7.30 7.29 7.28 7.27 7.23 7.22 7.21 7.20 7.18 7.18 7.16 7.16 4.18 4.18 4.17 4.16 4.16 4.15 4.14 2.87 2.85 2.85 2.84 2.83 1.87 1.86 1.82 1.81 1.67 1.66 1.54 1.53 1.53 1.52 1.51 1.50 1.49 1.49 1.48 1.30 1.28 1.27 1.25 1.23 1.22 0.89 0.87 0.87 0.85
8.05 8.05 8.03 8.03 7.32 7.31 7.30 7.29 7.28 7.27 7.23 7.22 7.21 7.20 7.18 7.18 7.16 7.16 7.15 7.14
OSi(ODIB)$_3$
N
Me
31a, ^{1}H-NMR
(400 MHz, CDCl$_3$)
1.00
1.04 1.02 1.01
8.1 8.0 7.9 7.8 7.7 7.6 7.5 7.4 7.3 7.2 7.1 7.0
f1 (ppm)
1.00 1.04 1.02 1.01 3.00 3.01 1.06 6.01 1.04 6.00 9.06 36.03
10.5 10.0 9.5 9.0 8.5 8.0 7.5 7.0 6.5 6.0 5.5 5.0 4.5 4.0 3.5 3.0 2.5 2.0 1.5 1.0 0.5 0.0 -0.5
f1 (ppm)

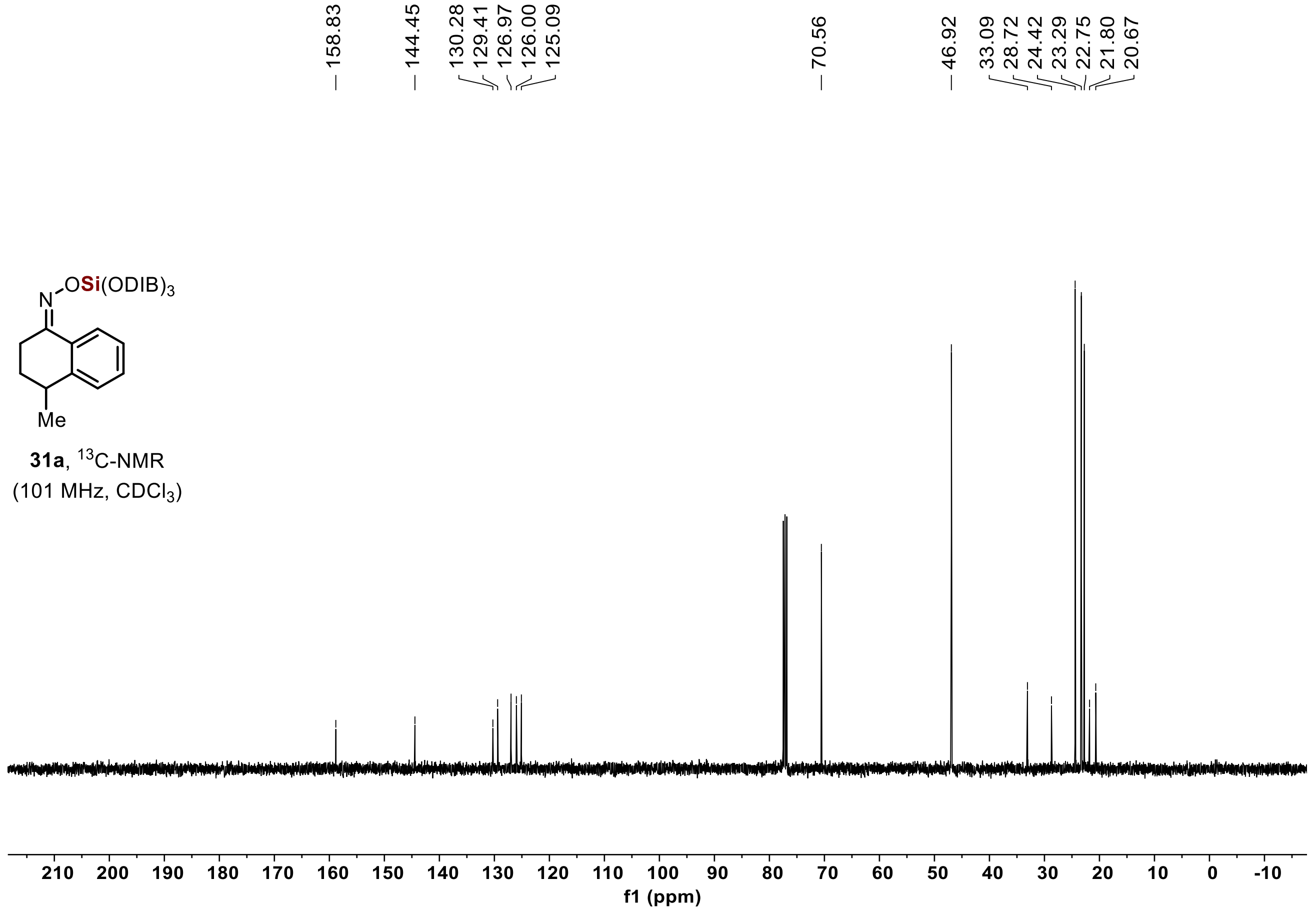

158.83 144.45 130.28 129.41 126.97 126.00 125.09 70.56 46.92 33.09 28.72 24.42 23.29 22.75 21.80 20.67
OSi(ODIB)$_3$
N
Me
31a, ^{13}C-NMR
(101 MHz, CDCl$_3$)
210 200 190 180 170 160 150 140 130 120 110 100 90 80 70 60 50 40 30 20 10 0 -10
f1 (ppm)

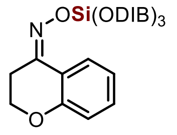

32a, ^{1}H-NMR
(400 MHz, $CDCl_3$)

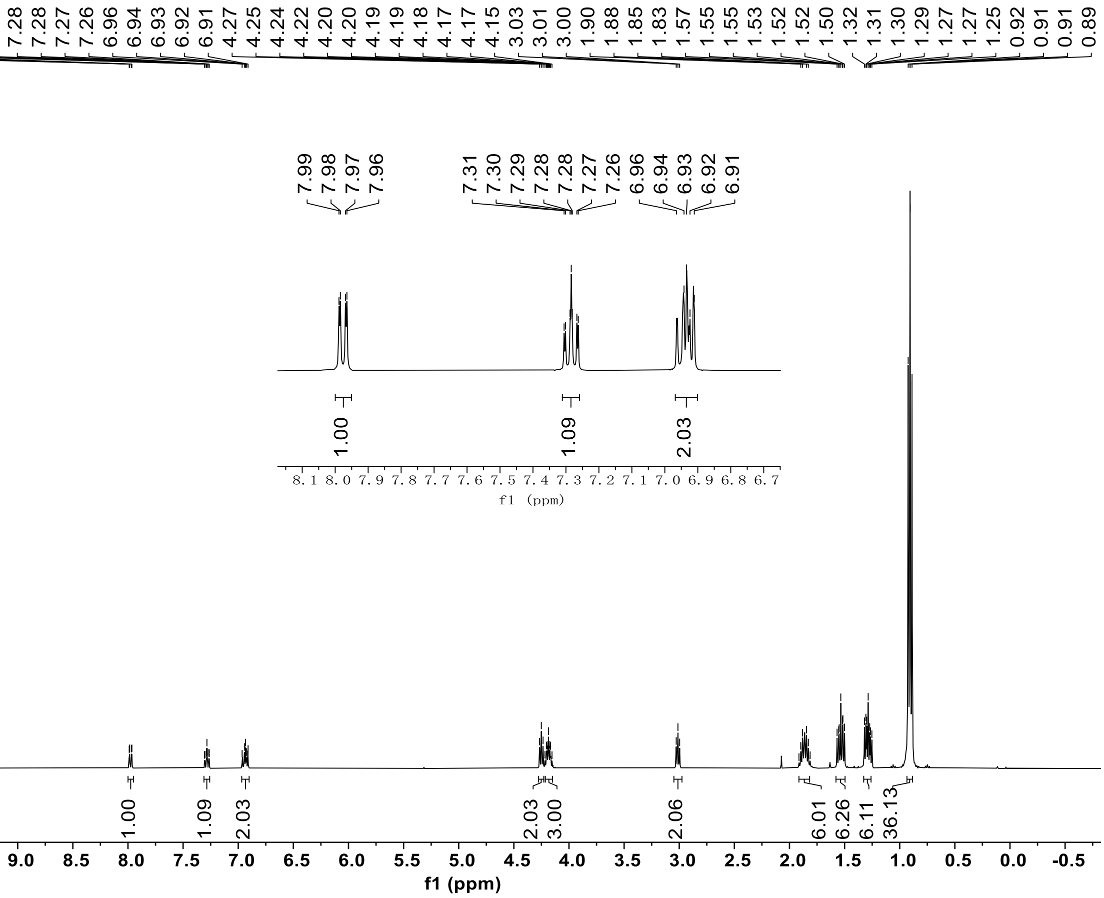

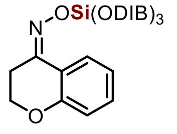

32a, ^{13}C-NMR
(101 MHz, $CDCl_3$)

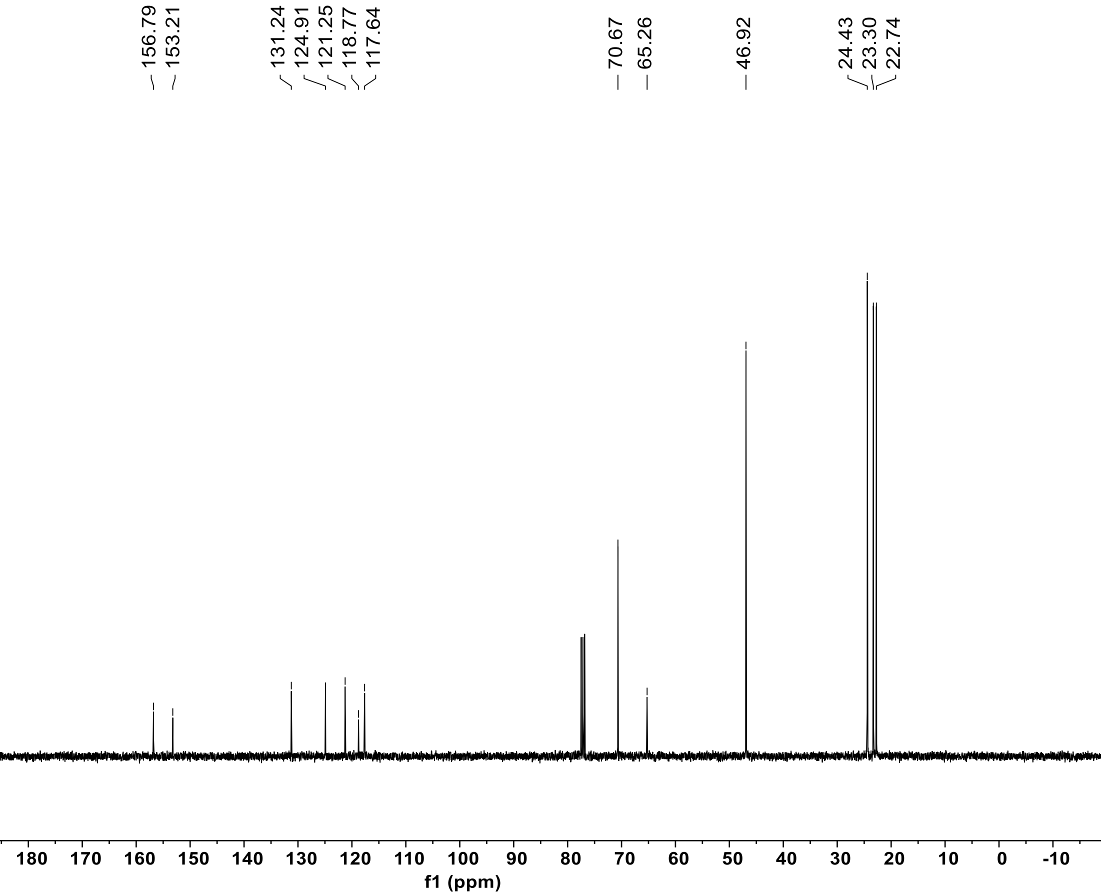

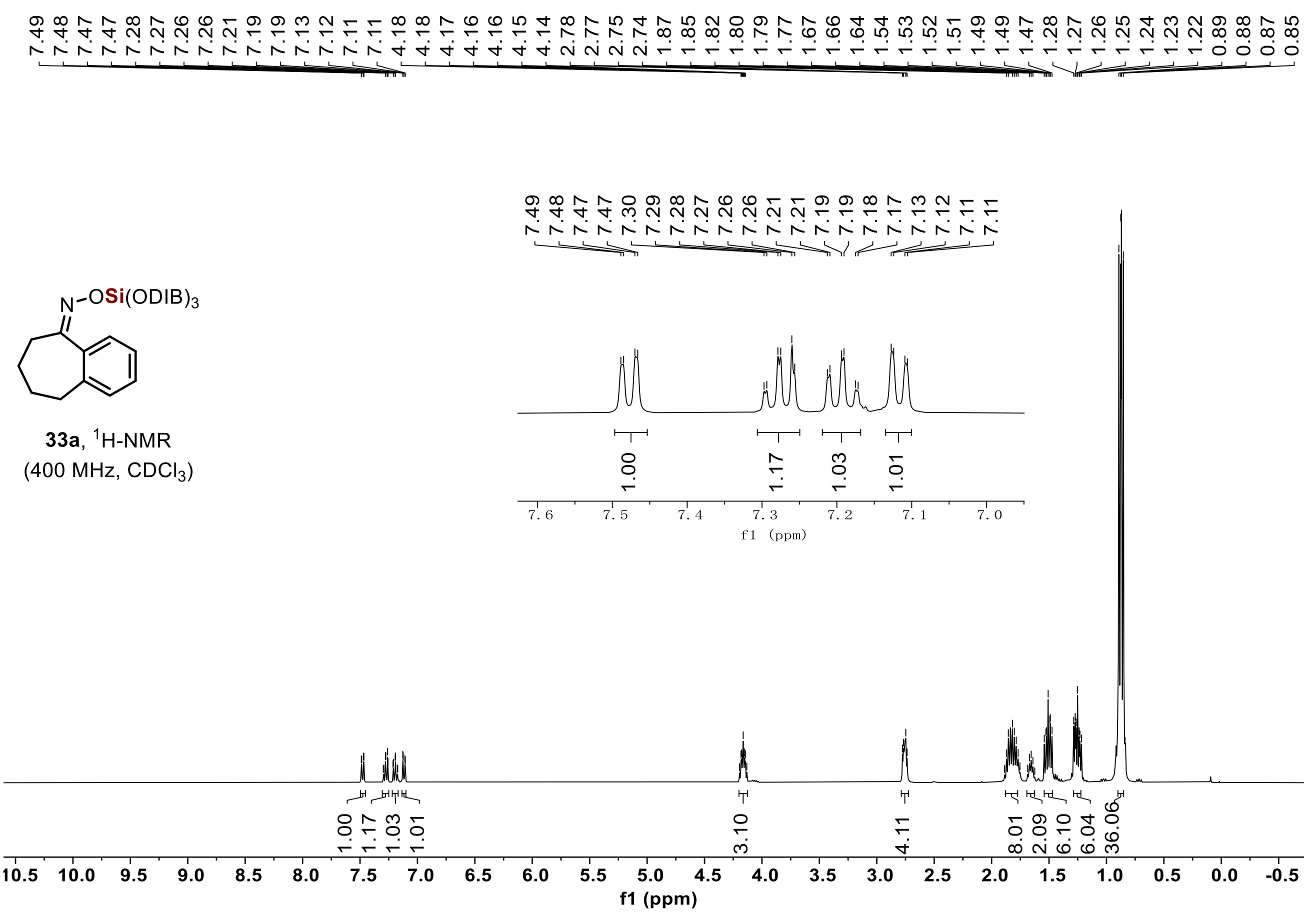
7.49 7.48 7.47 7.47 7.28 7.27 7.26 7.26 7.21 7.19 7.19 7.13 7.12 7.11 7.11 4.18 4.17 4.16 4.16 4.15 4.14 2.78 2.77 2.75 2.74 1.87 1.85 1.82 1.80 1.79 1.77 1.67 1.66 1.64 1.54 1.53 1.52 1.51 1.49 1.49 1.47 1.28 1.27 1.26 1.25 1.24 1.23 1.22 0.89 0.88 0.87 0.85
7.49 7.48 7.47 7.47 7.30 7.29 7.28 7.27 7.26 7.26 7.21 7.21 7.19 7.19 7.18 7.17 7.13 7.12 7.11 7.11
1.00 1.17 1.03 1.01
f1 (ppm)
N-OSi(ODIB)3
33a, 1H-NMR
(400 MHz, CDCl3)
1.00 1.17 1.03 1.01 3.10 4.11 8.01 2.09 6.10 6.04 36.06
f1 (ppm)

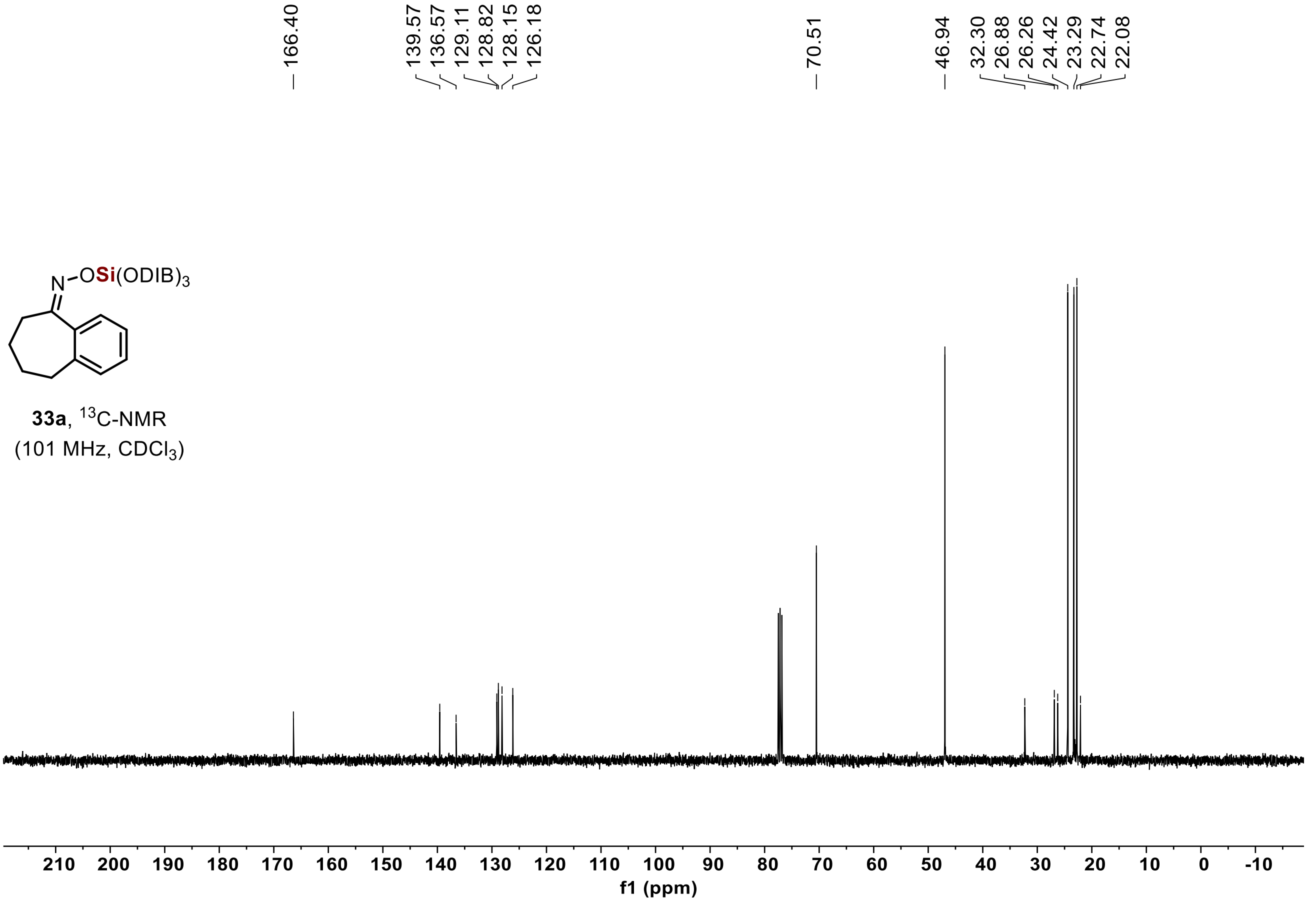
166.40
139.57 136.57 129.11 128.82 128.15 126.18
70.51
46.94 32.30 26.88 26.26 24.42 23.29 22.74 22.08
N-OSi(ODIB)3
33a, 13C-NMR
(101 MHz, CDCl3)
f1 (ppm)

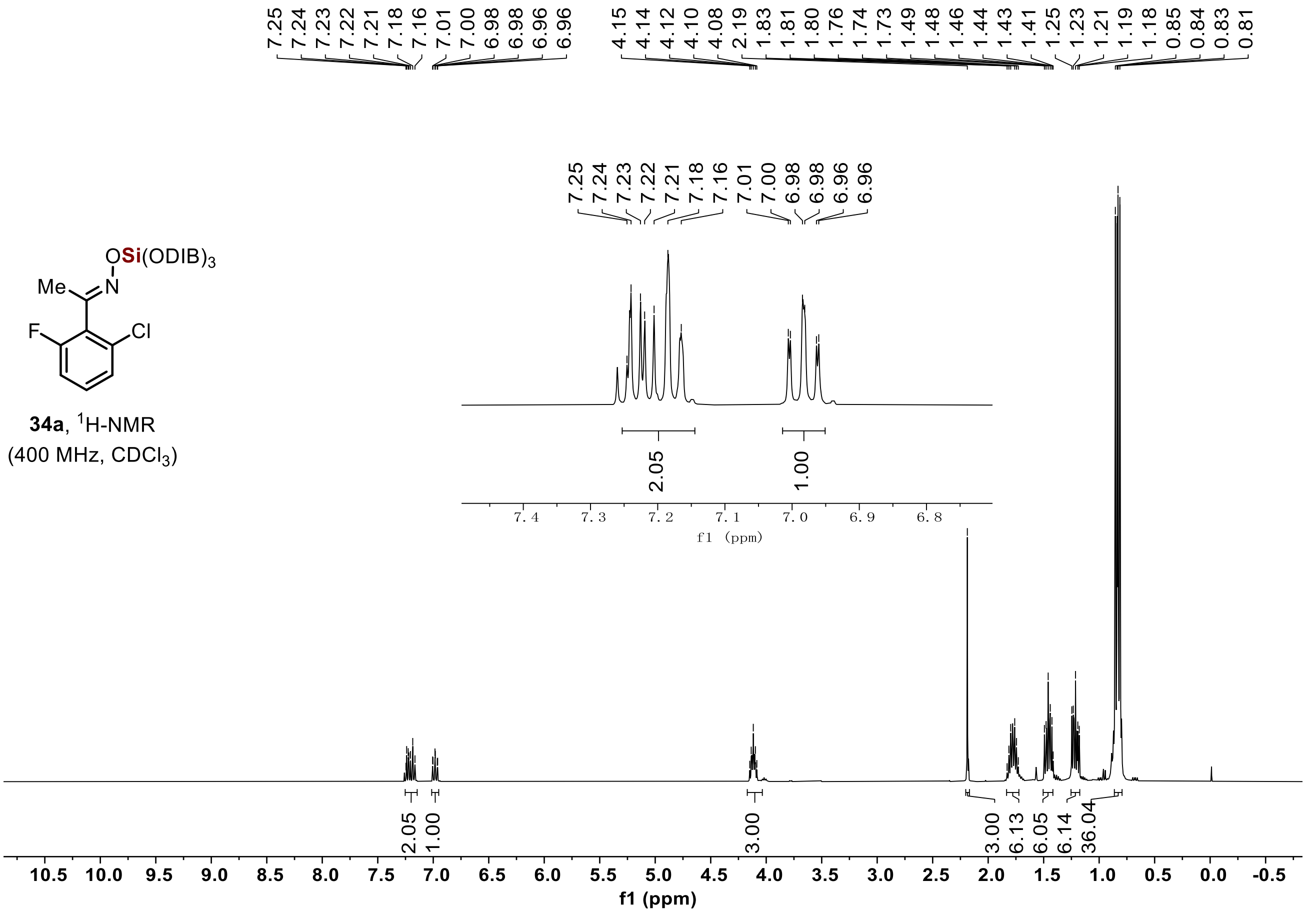

34a, ^{1}H-NMR
(400 MHz, $CDCl_3$)

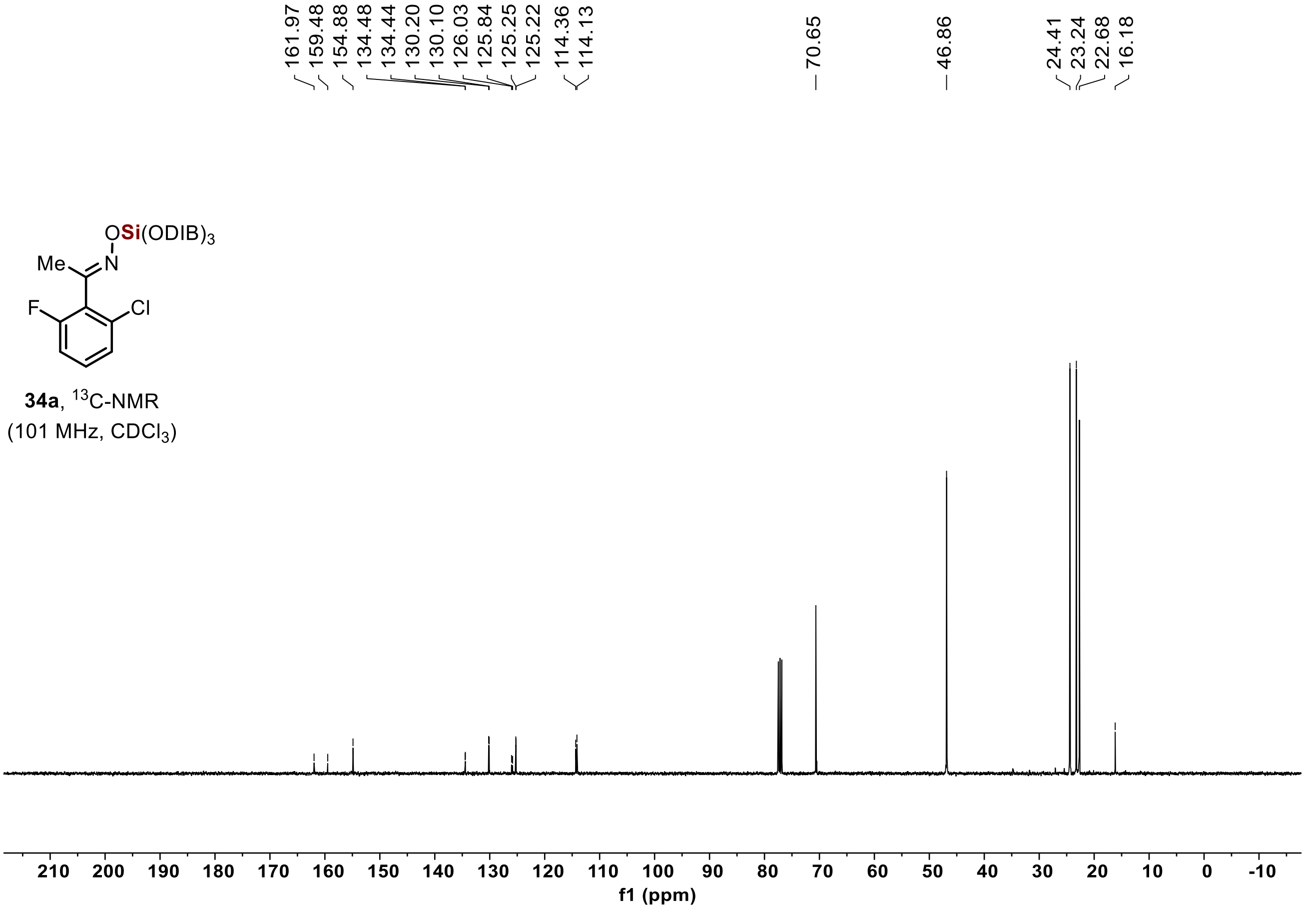

34a, ^{13}C-NMR
(101 MHz, $CDCl_3$)

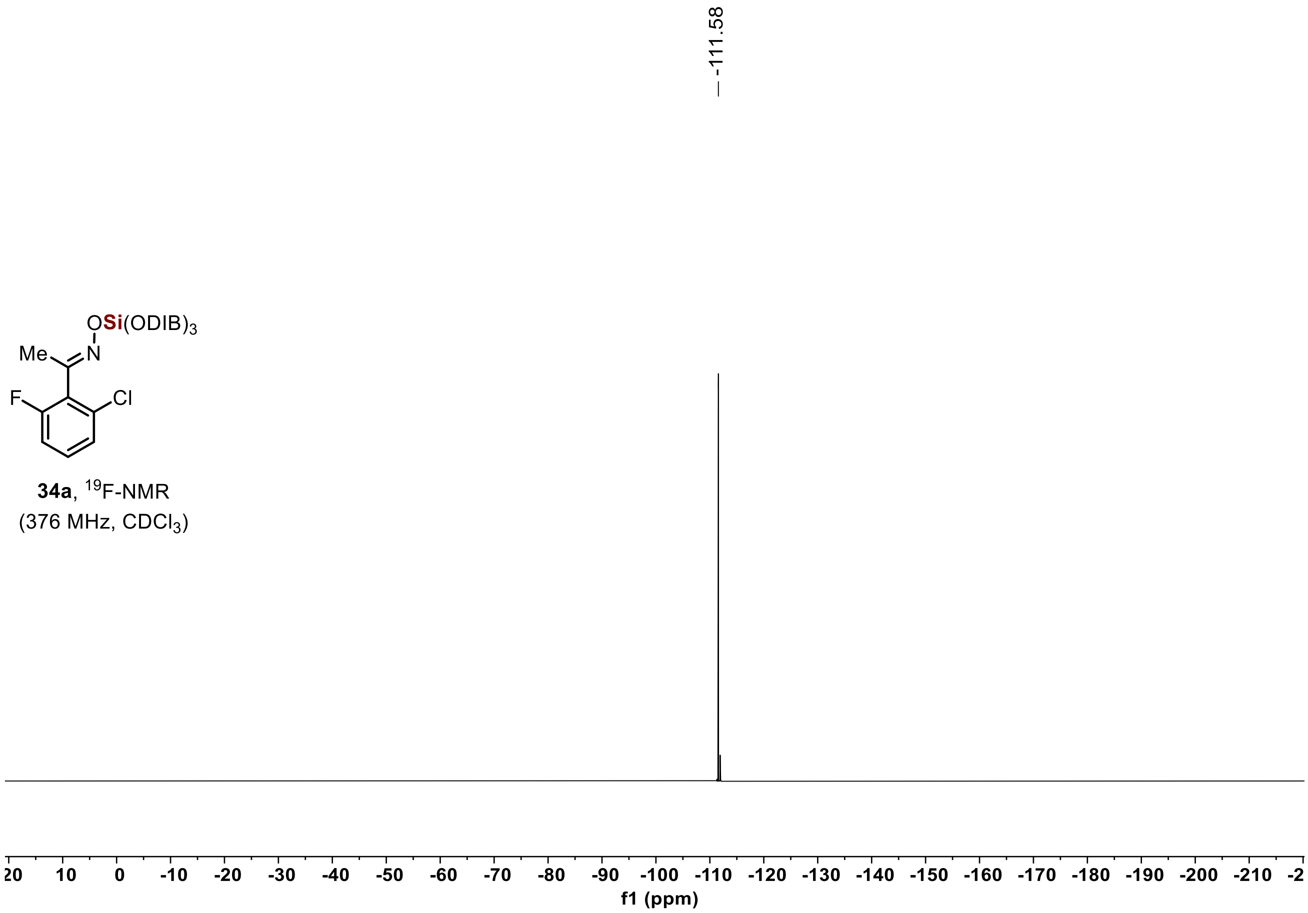

34a, ^{19}F-NMR
(376 MHz, $CDCl_3$)

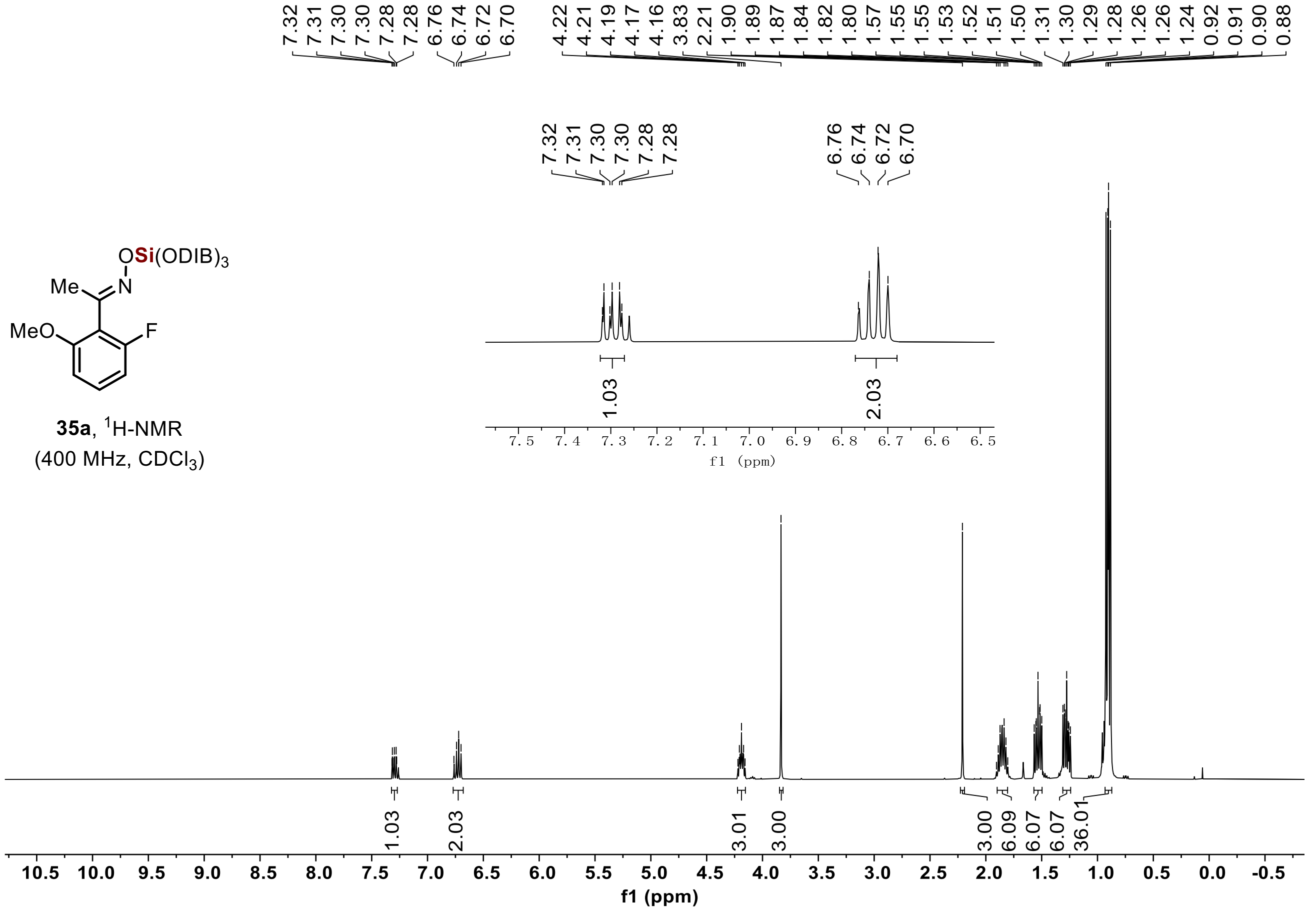

35a, ^{1}H-NMR
(400 MHz, $CDCl_3$)

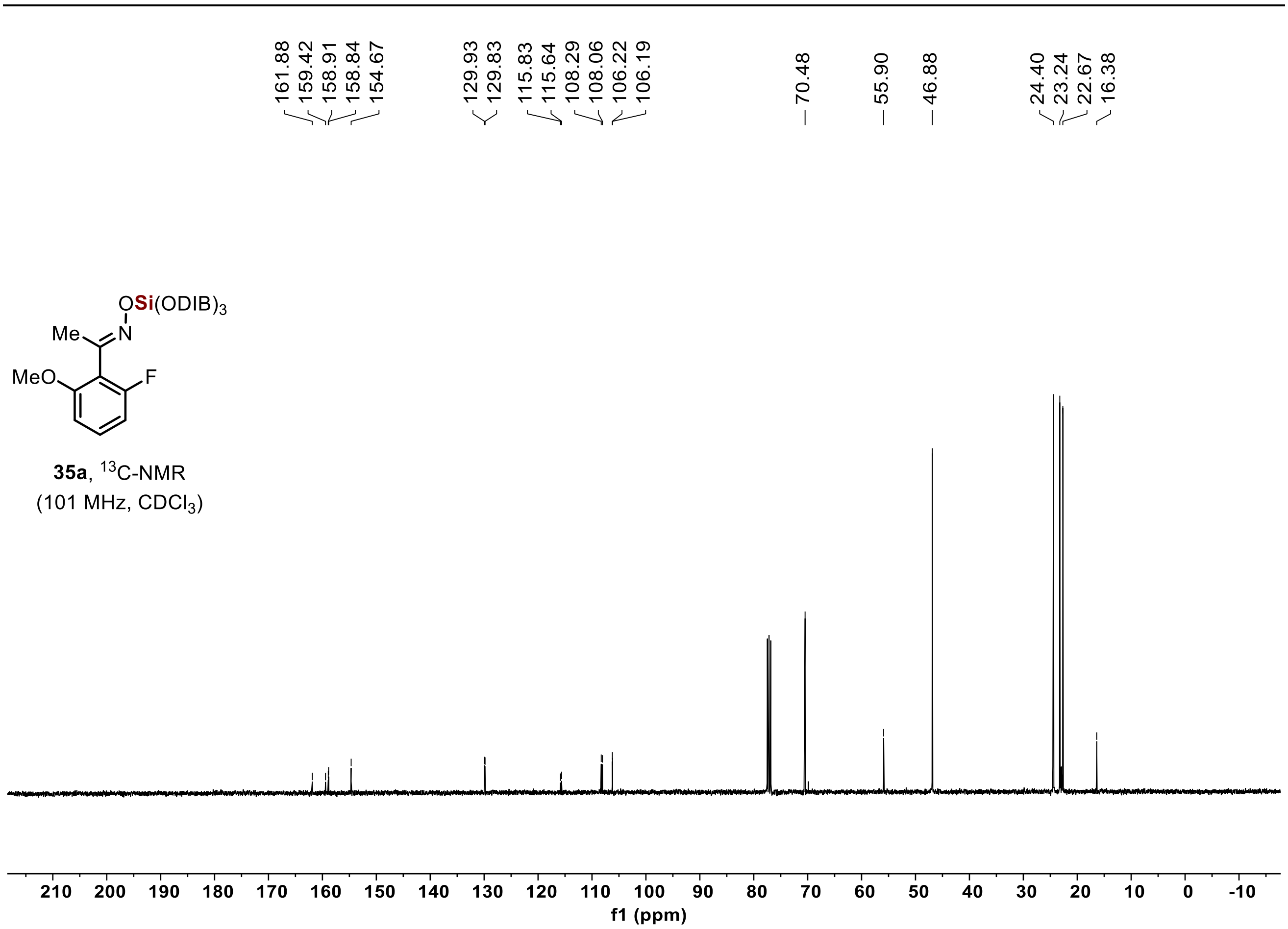
161.88
159.42
158.91
158.84
154.67
129.93
129.83
115.83
115.64
108.29
108.06
106.22
106.19
70.48
55.90
46.88
24.40
23.24
22.67
16.38
OSi(ODIB)3
Me
N
MeO
F
35a, 13C-NMR
(101 MHz, CDCl3)
210 200 190 180 170 160 150 140 130 120 110 100 90 80 70 60 50 40 30 20 10 0 -10
f1 (ppm)

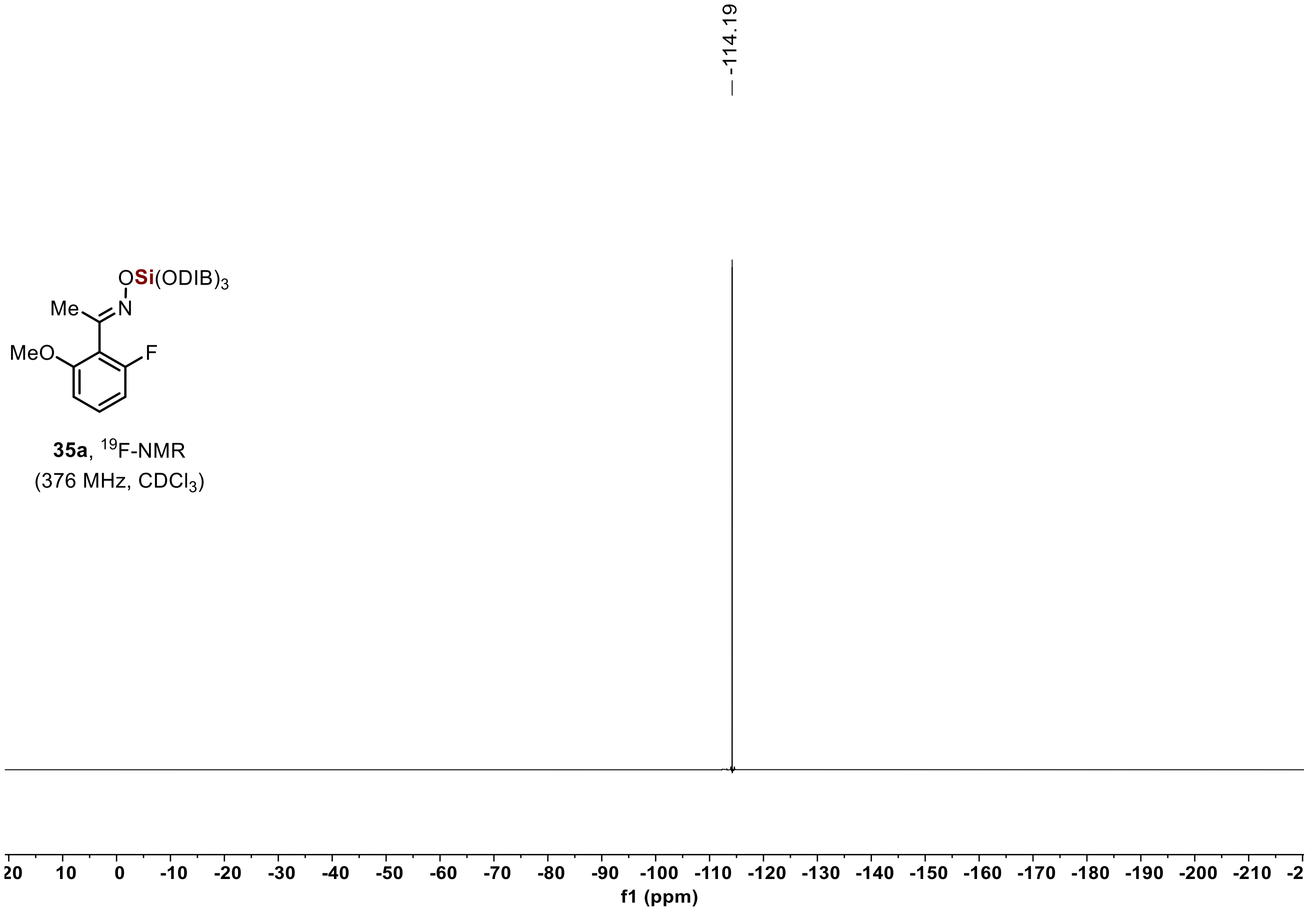
-114.19
OSi(ODIB)3
Me
N
MeO
F
35a, 19F-NMR
(376 MHz, CDCl3)
20 10 0 -10 -20 -30 -40 -50 -60 -70 -80 -90 -100 -110 -120 -130 -140 -150 -160 -170 -180 -190 -200 -210 -2
f1 (ppm)

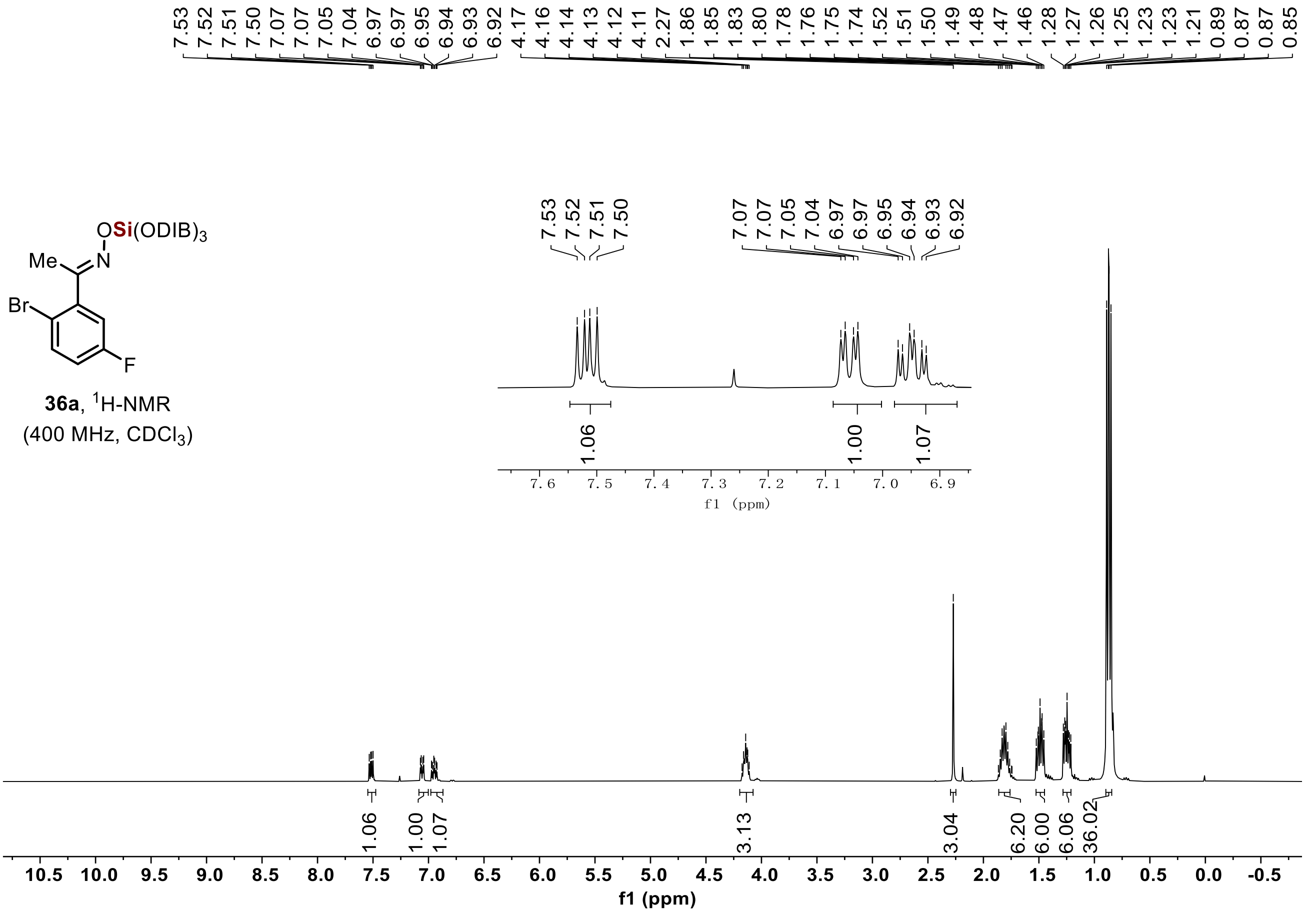

OSi(ODIB)3
Me
N
Br
F
36a, 1H-NMR
(400 MHz, CDCl3)
7.53 7.52 7.51 7.50 7.07 7.07 7.05 7.04 6.97 6.97 6.95 6.94 6.93 6.92 4.17 4.16 4.14 4.13 4.12 4.11 2.27 1.86 1.85 1.83 1.80 1.78 1.76 1.75 1.74 1.52 1.51 1.50 1.49 1.48 1.47 1.46 1.28 1.27 1.26 1.25 1.23 1.23 1.21 0.89 0.87 0.87 0.85
1.06 1.00 1.07 3.13 3.04 6.20 6.00 6.06 36.02
f1 (ppm)

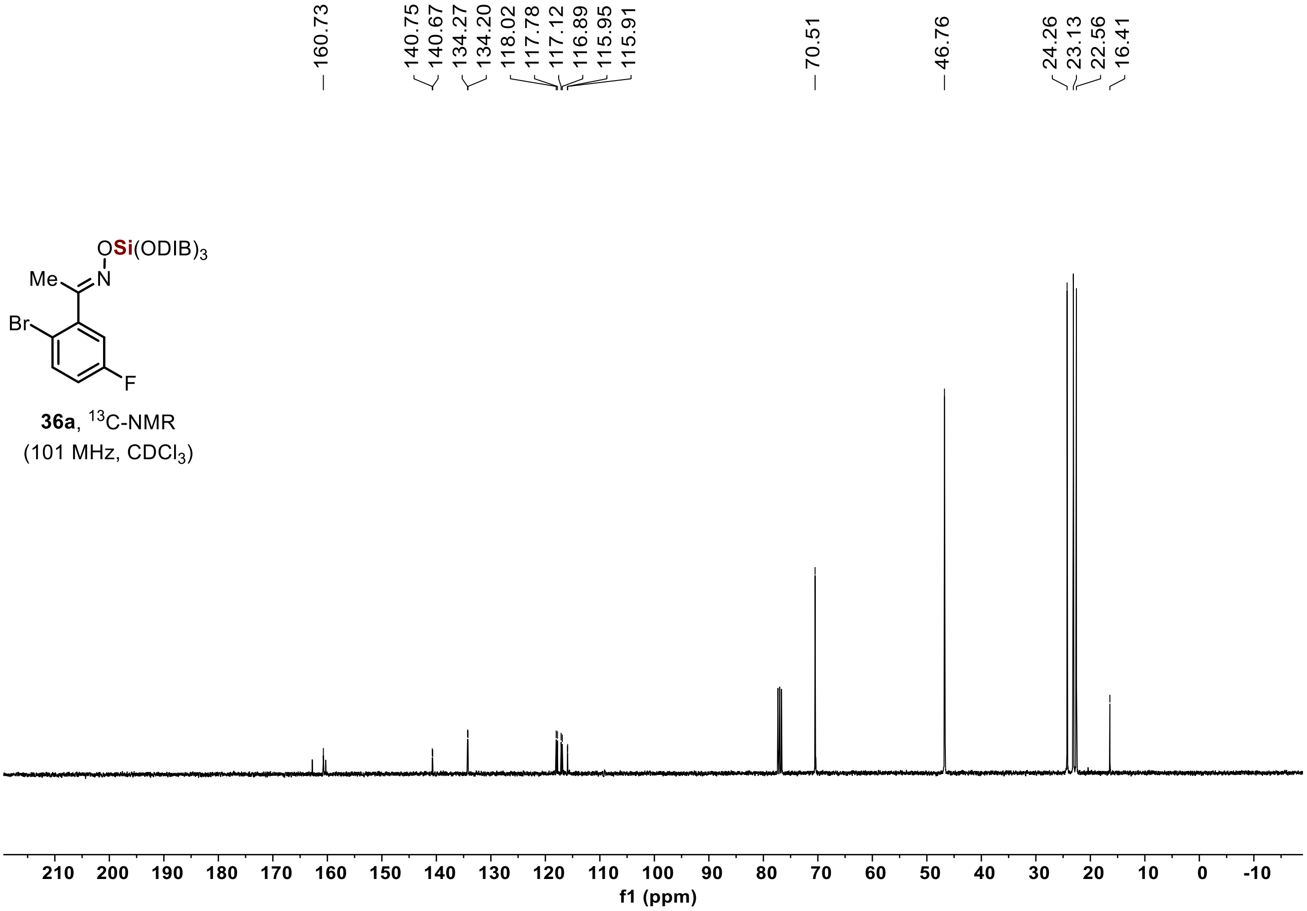

OSi(ODIB)3
Me
N
Br
F
36a, 13C-NMR
(101 MHz, CDCl3)
160.73 140.75 140.67 134.27 134.20 118.02 117.78 117.12 116.89 115.95 115.91 70.51 46.76 24.26 23.13 22.56 16.41
f1 (ppm)

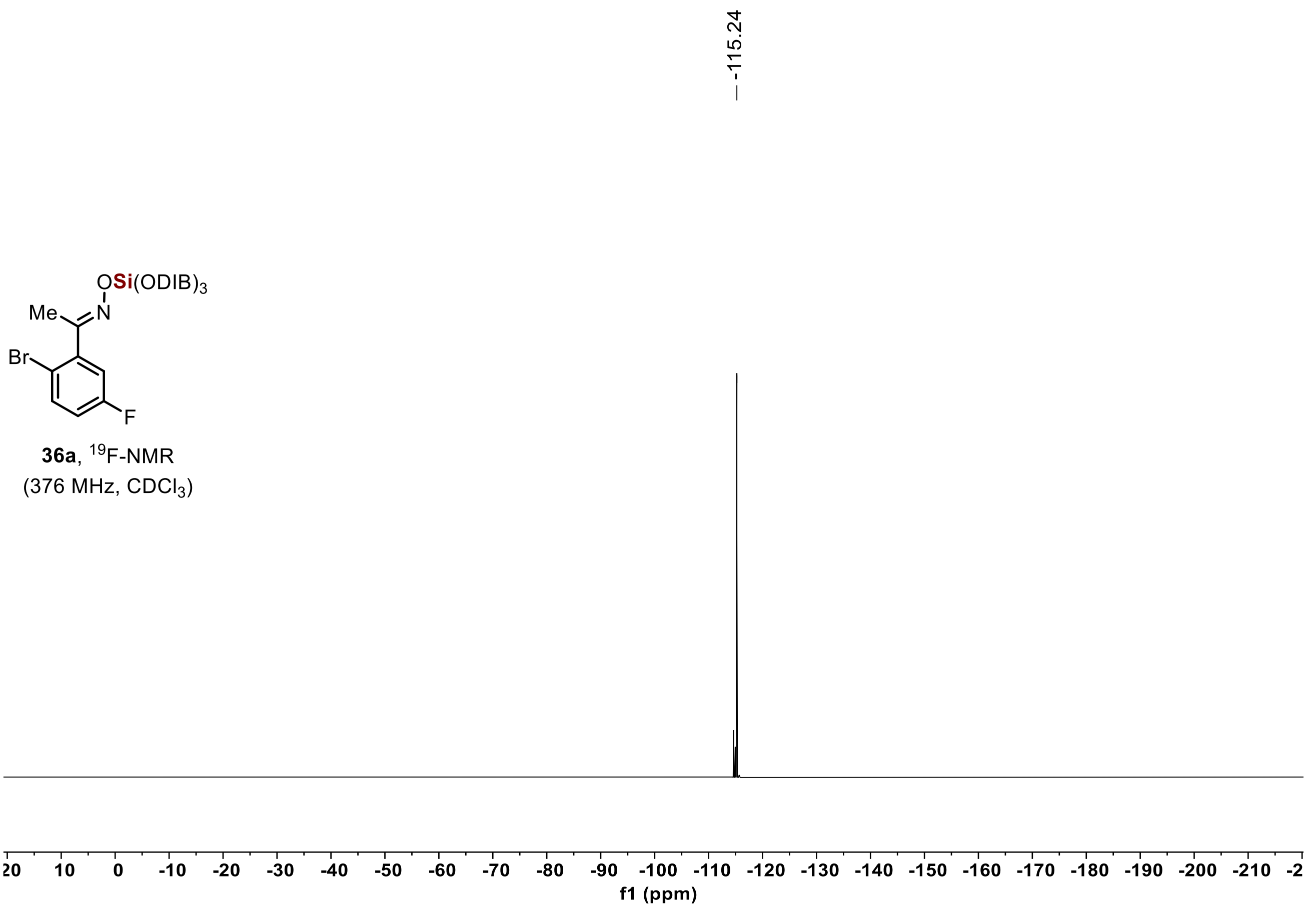

36a, ^{19}F-NMR
(376 MHz, $CDCl_3$)

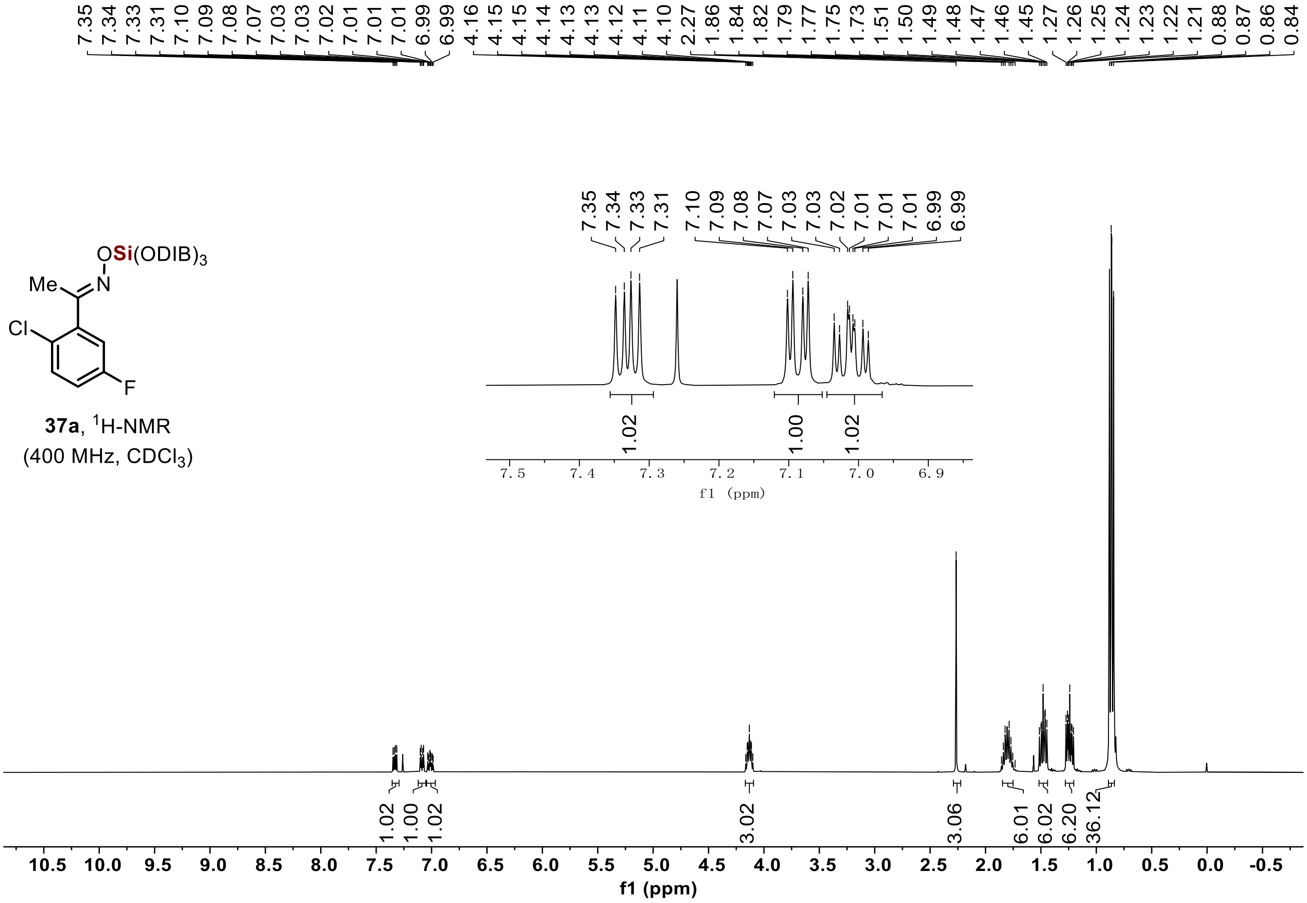

37a, ^{1}H-NMR
(400 MHz, $CDCl_3$)

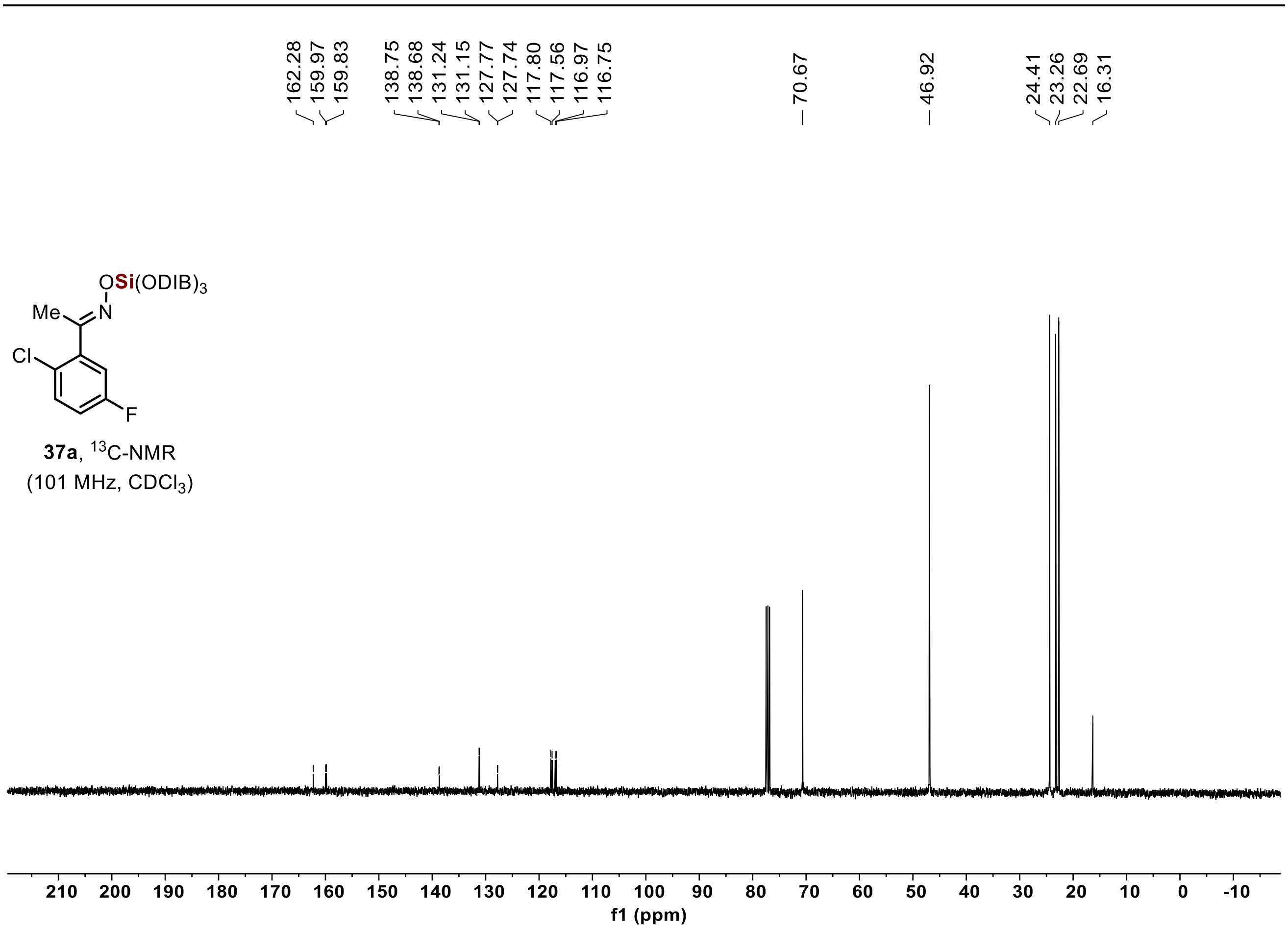

37a, ^{13}C-NMR
(101 MHz, $CDCl_3$)

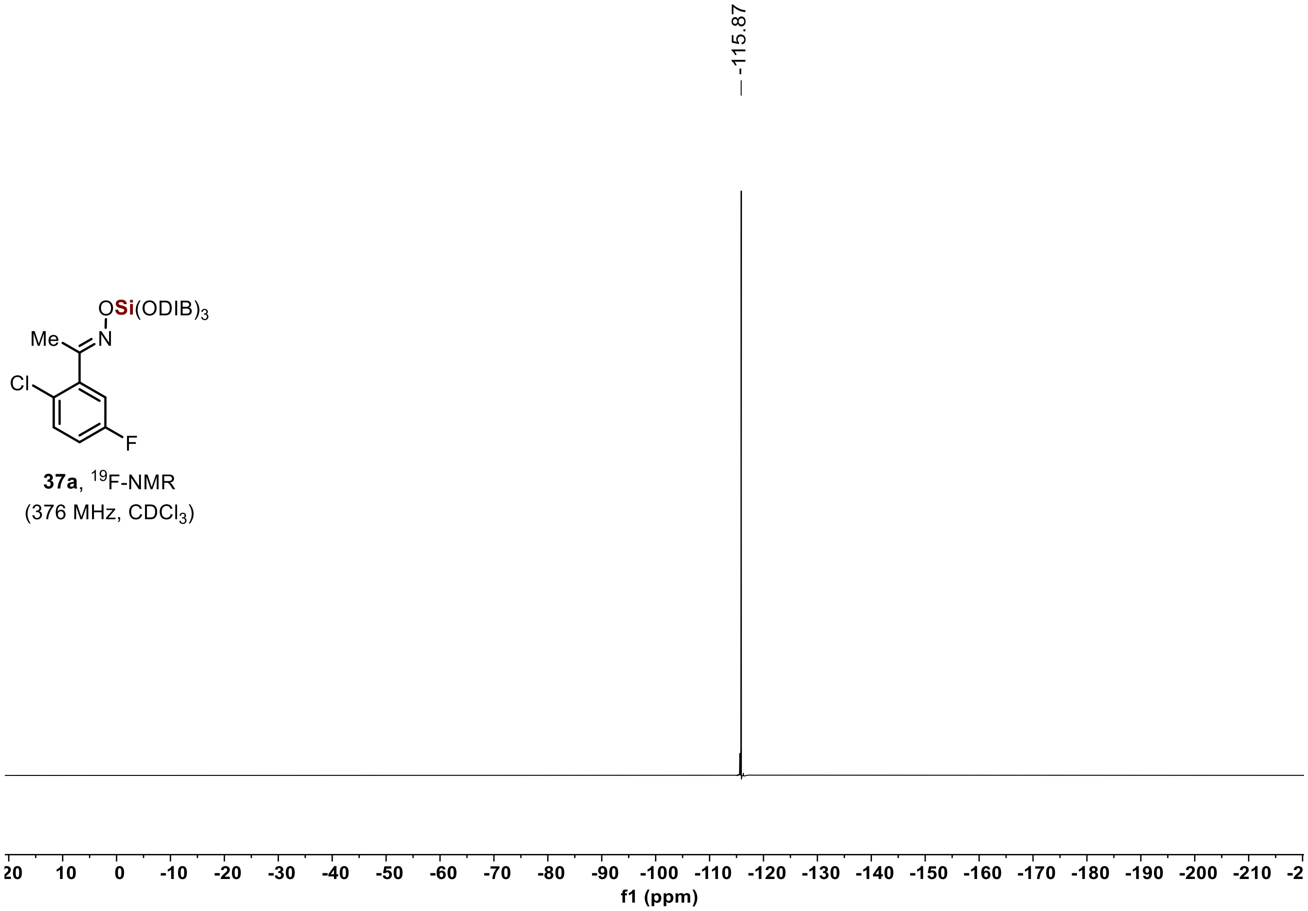

37a, ^{19}F-NMR
(376 MHz, $CDCl_3$)

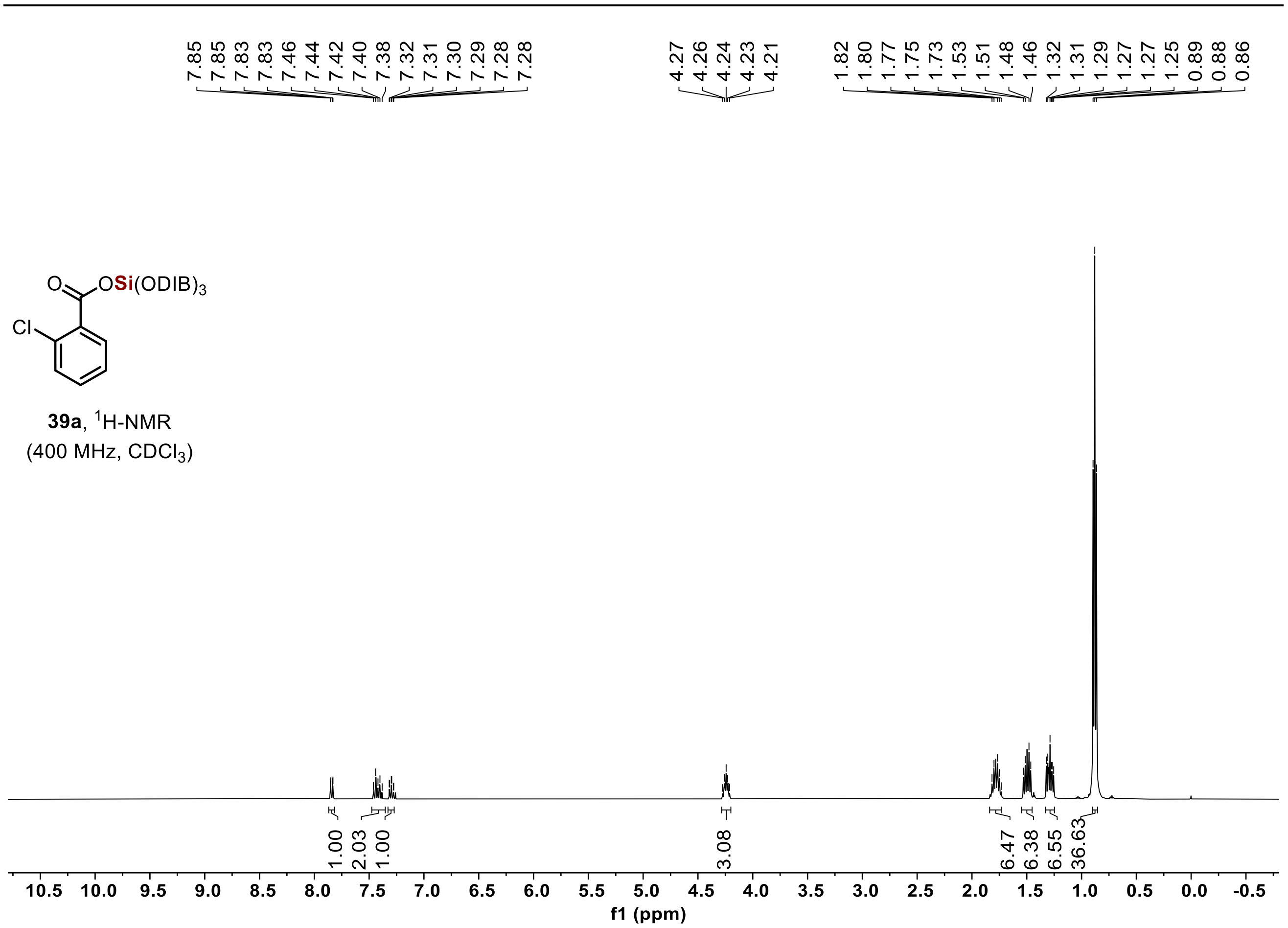
7.85 7.85 7.83 7.83 7.46 7.44 7.42 7.40 7.38 7.32 7.31 7.30 7.29 7.28 7.28
4.27 4.26 4.24 4.23 4.21
1.82 1.80 1.77 1.75 1.73 1.53 1.51 1.48 1.46 1.32 1.31 1.29 1.27 1.27 1.25 0.89 0.88 0.86
OSi(ODIB)3
Cl
O
39a, 1H-NMR
(400 MHz, CDCl3)
1.00 2.03 1.00
3.08
6.47 6.38 6.55 36.63
10.5 10.0 9.5 9.0 8.5 8.0 7.5 7.0 6.5 6.0 5.5 5.0 4.5 4.0 3.5 3.0 2.5 2.0 1.5 1.0 0.5 0.0 -0.5
f1 (ppm)

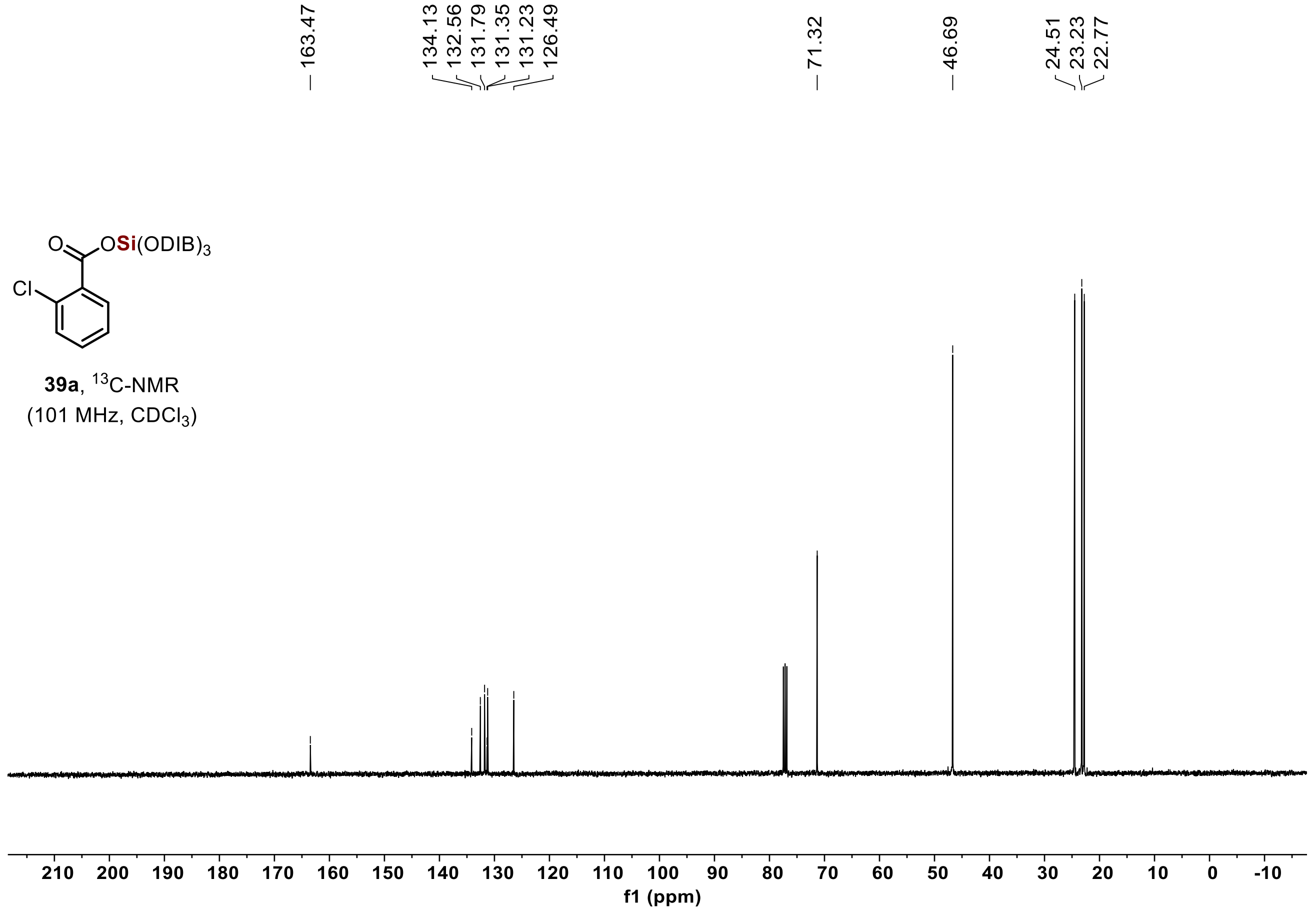
163.47
134.13 132.56 131.79 131.35 131.23 126.49
71.32
46.69
24.51 23.23 22.77
OSi(ODIB)3
Cl
O
39a, 13C-NMR
(101 MHz, CDCl3)
210 200 190 180 170 160 150 140 130 120 110 100 90 80 70 60 50 40 30 20 10 0 -10
f1 (ppm)

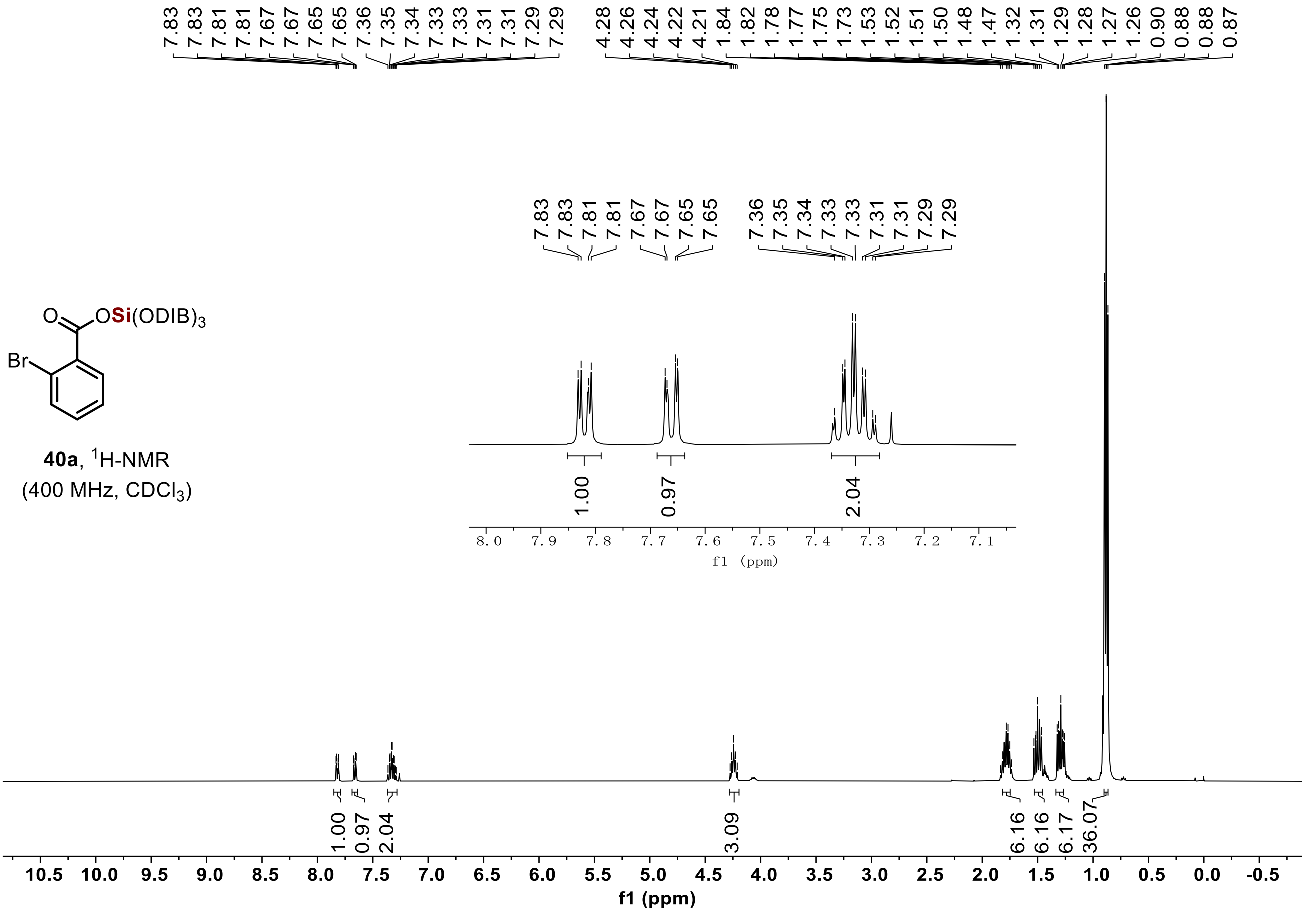

40a, ^{1}H-NMR
(400 MHz, $CDCl_3$)

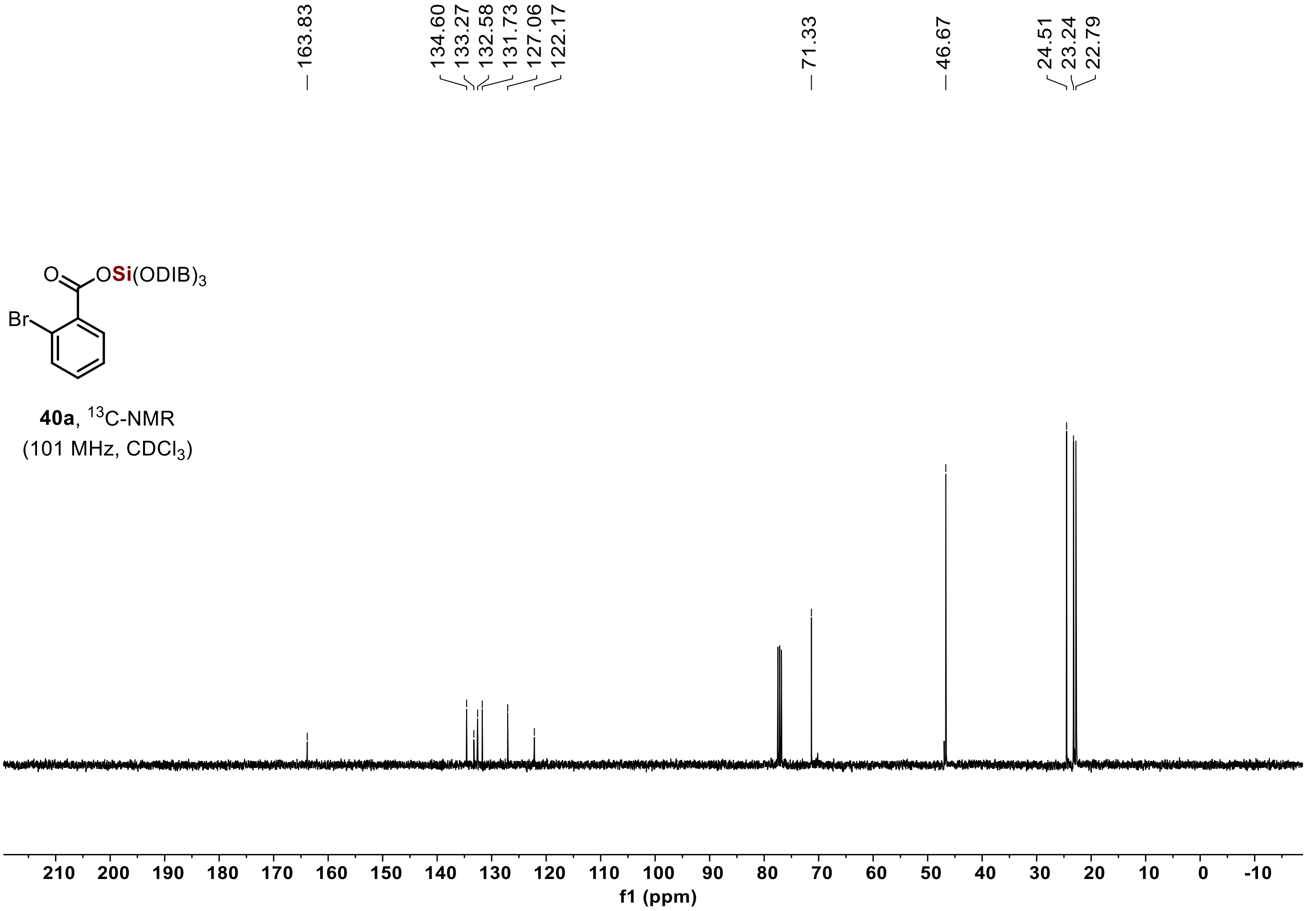

40a, ^{13}C-NMR
(101 MHz, $CDCl_3$)

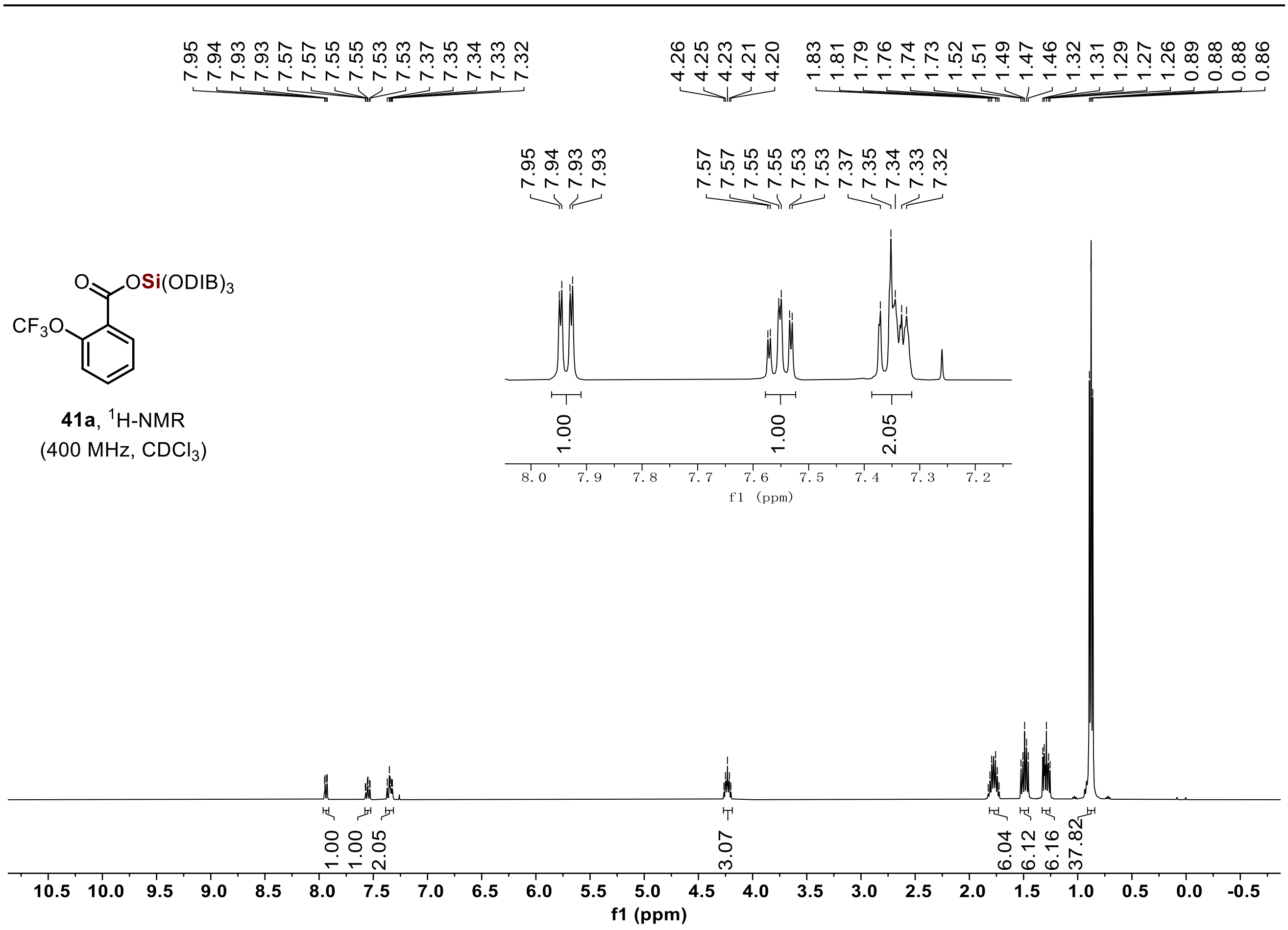

41a, 1H-NMR
(400 MHz, CDCl3)

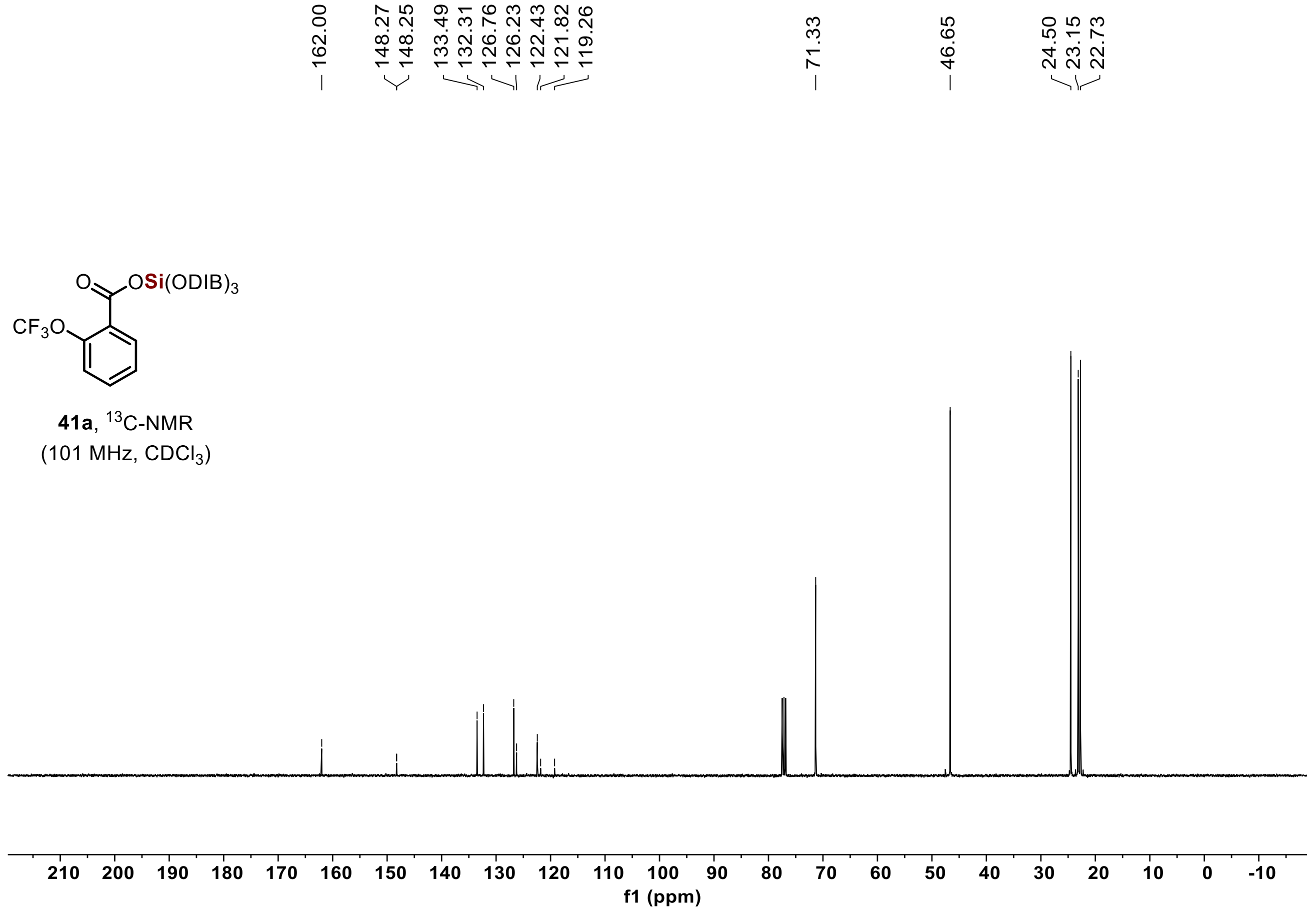

41a, 13C-NMR
(101 MHz, CDCl3)

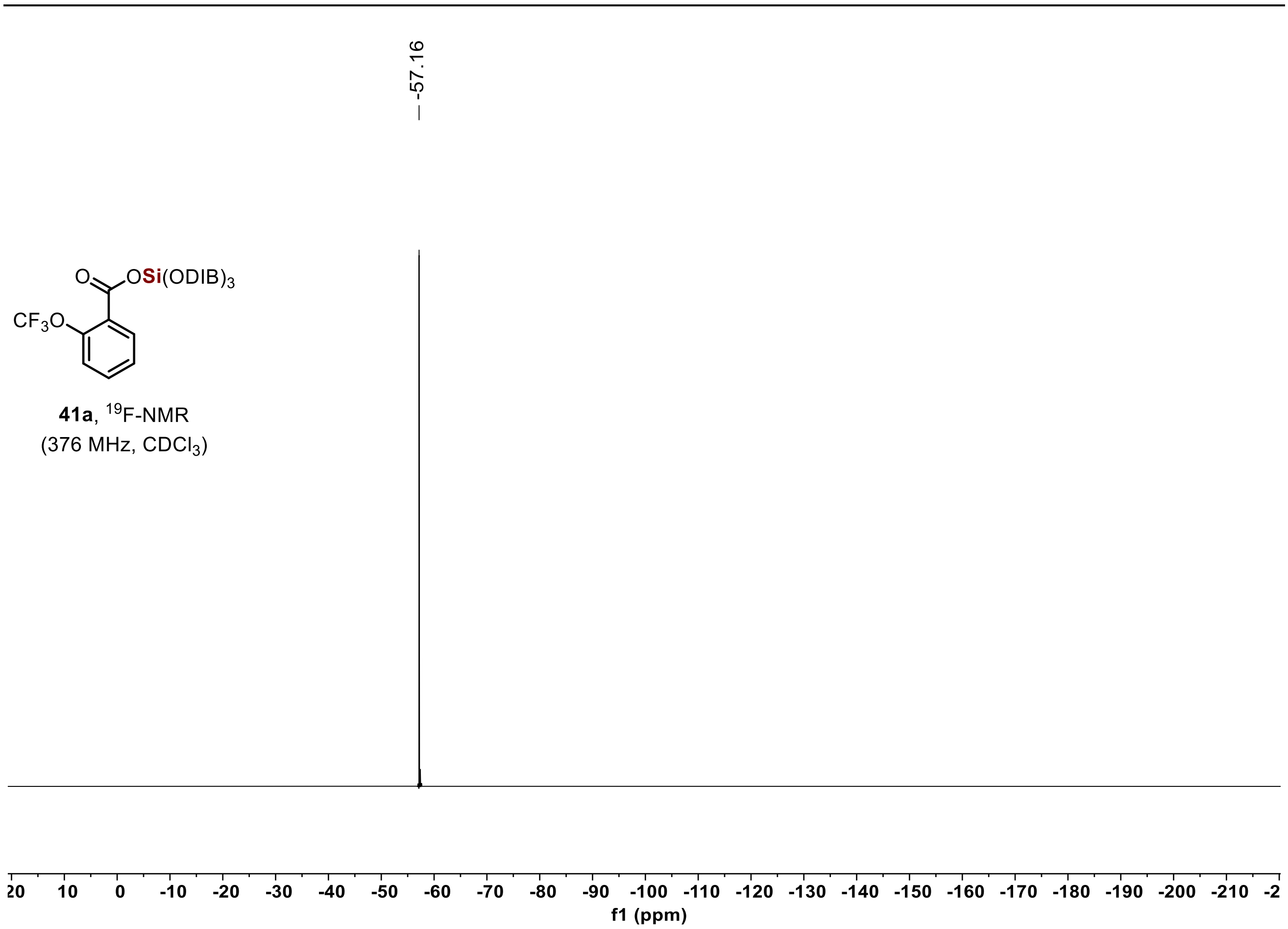

-57.16
OSi(ODIB)3
CF3O
41a, 19F-NMR
(376 MHz, CDCl3)
f1 (ppm)

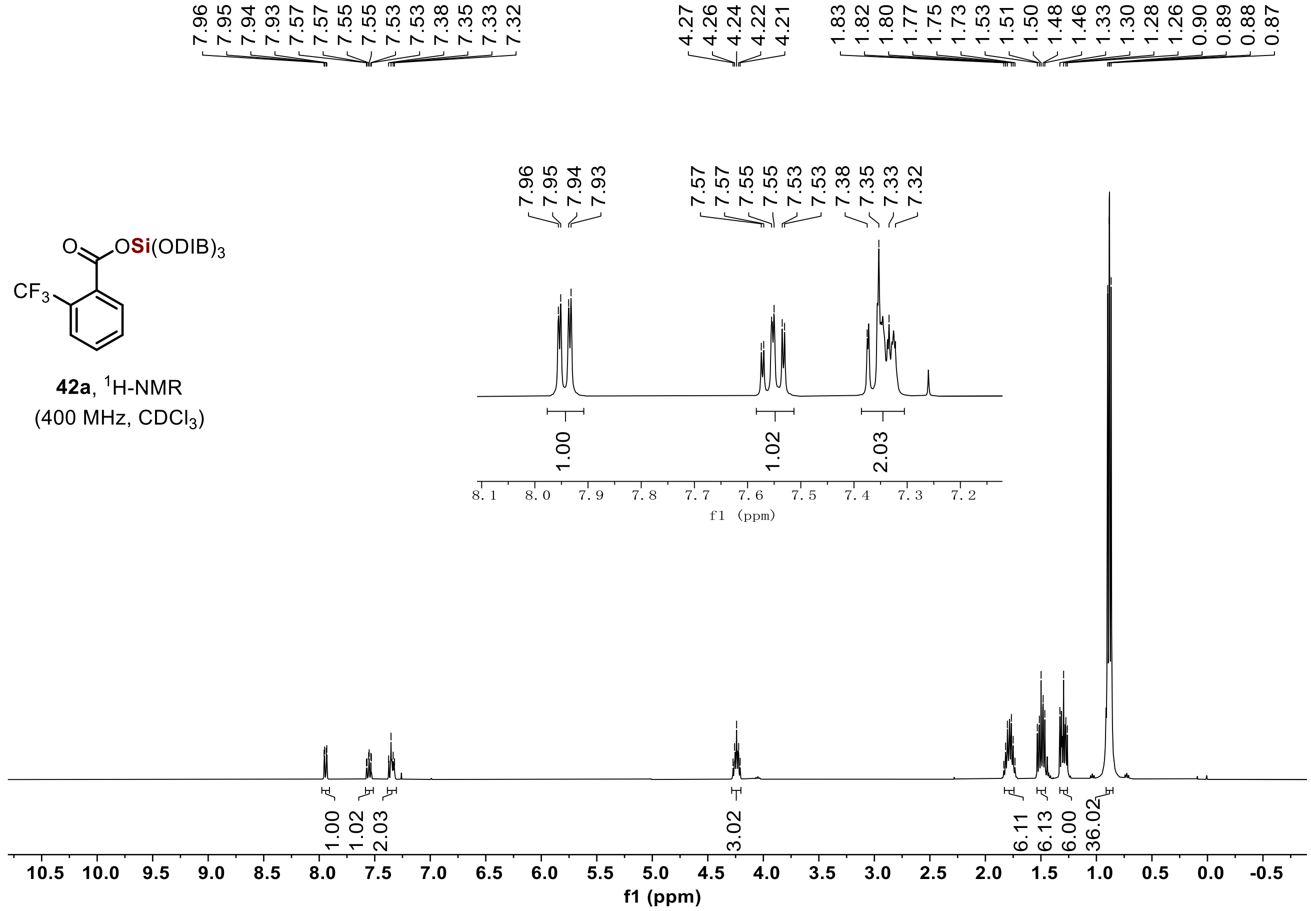

7.96 7.95 7.94 7.93 7.57 7.57 7.55 7.55 7.53 7.53 7.38 7.35 7.33 7.32
4.27 4.26 4.24 4.22 4.21
1.83 1.82 1.80 1.77 1.75 1.73 1.53 1.51 1.50 1.48 1.46 1.33 1.30 1.28 1.26 0.90 0.89 0.88 0.87
OSi(ODIB)3
CF3
42a, 1H-NMR
(400 MHz, CDCl3)
1.00 1.02 2.03 3.02 6.11 6.13 6.00 36.02
f1 (ppm)

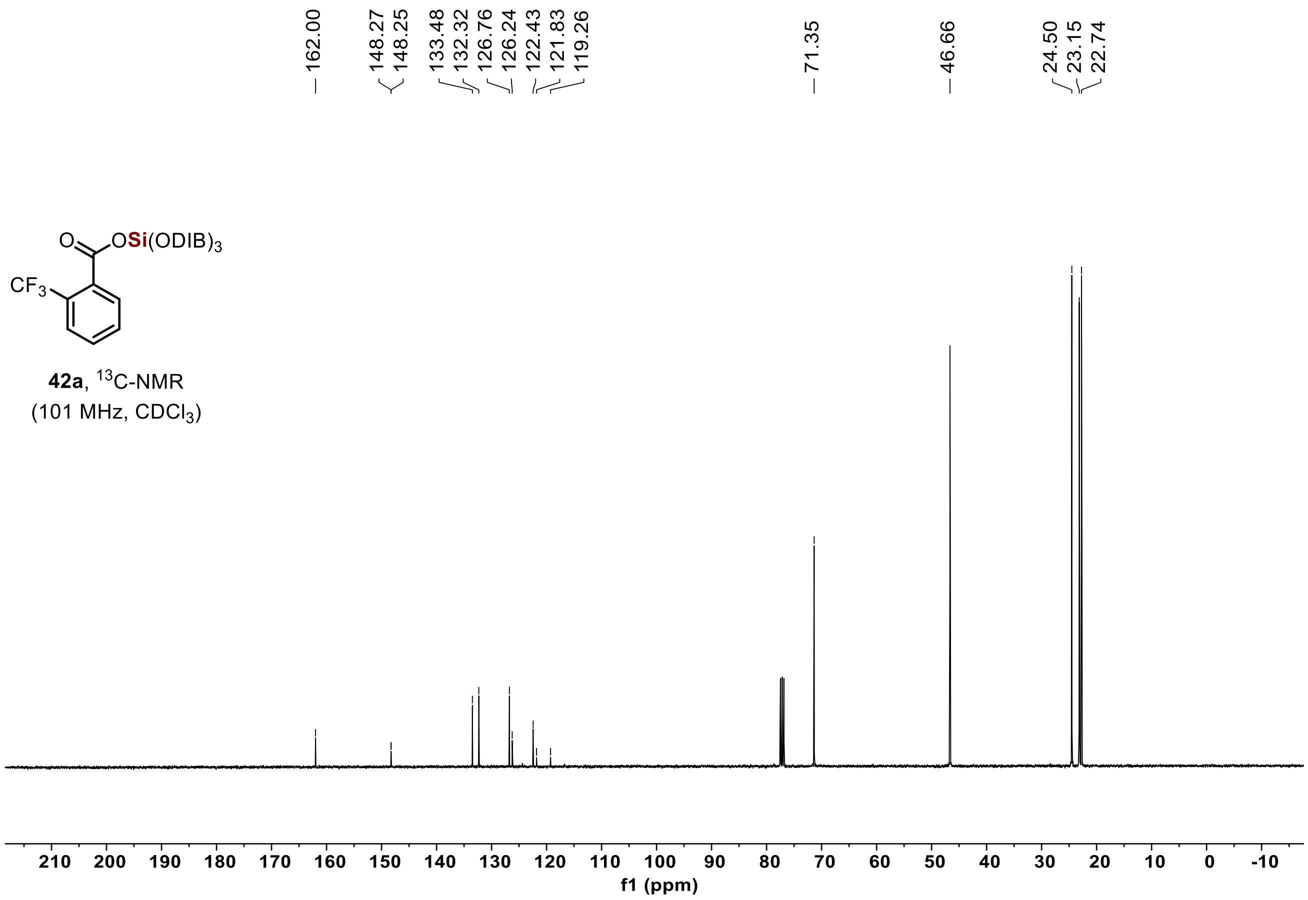
162.00
148.27
148.25
133.48
132.32
126.76
126.24
122.43
121.83
119.26
71.35
46.66
24.50
23.15
22.74
O
OSi(ODIB)3
CF3
42a, 13C-NMR
(101 MHz, CDCl3)
210 200 190 180 170 160 150 140 130 120 110 100 90 80 70 60 50 40 30 20 10 0 -10
f1 (ppm)

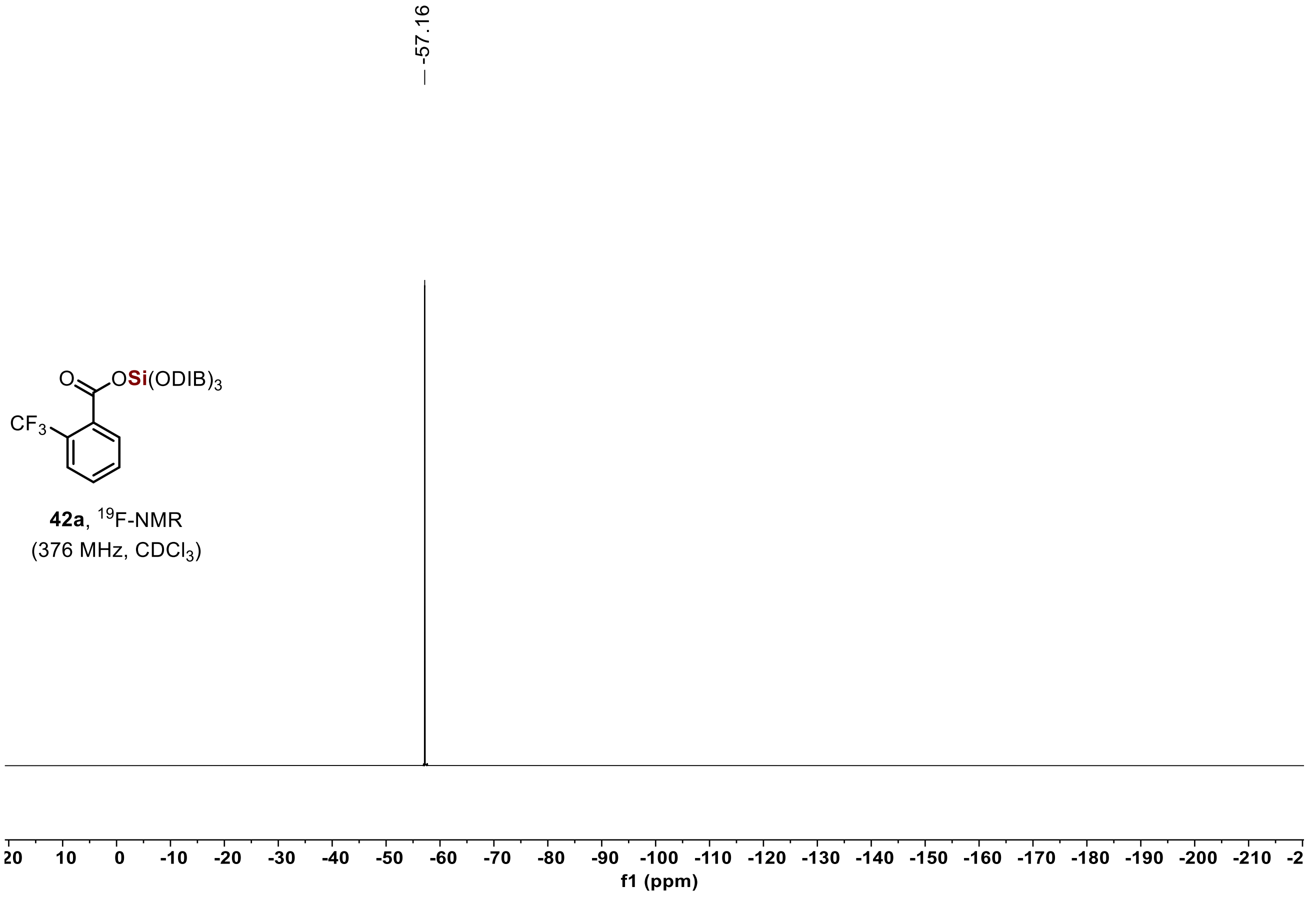
-57.16
O
OSi(ODIB)3
CF3
42a, 19F-NMR
(376 MHz, CDCl3)
20 10 0 -10 -20 -30 -40 -50 -60 -70 -80 -90 -100 -110 -120 -130 -140 -150 -160 -170 -180 -190 -200 -210 -2
f1 (ppm)

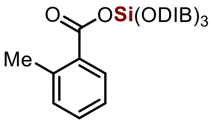

43a, ^{1}H-NMR
(400 MHz, $CDCl_3$)

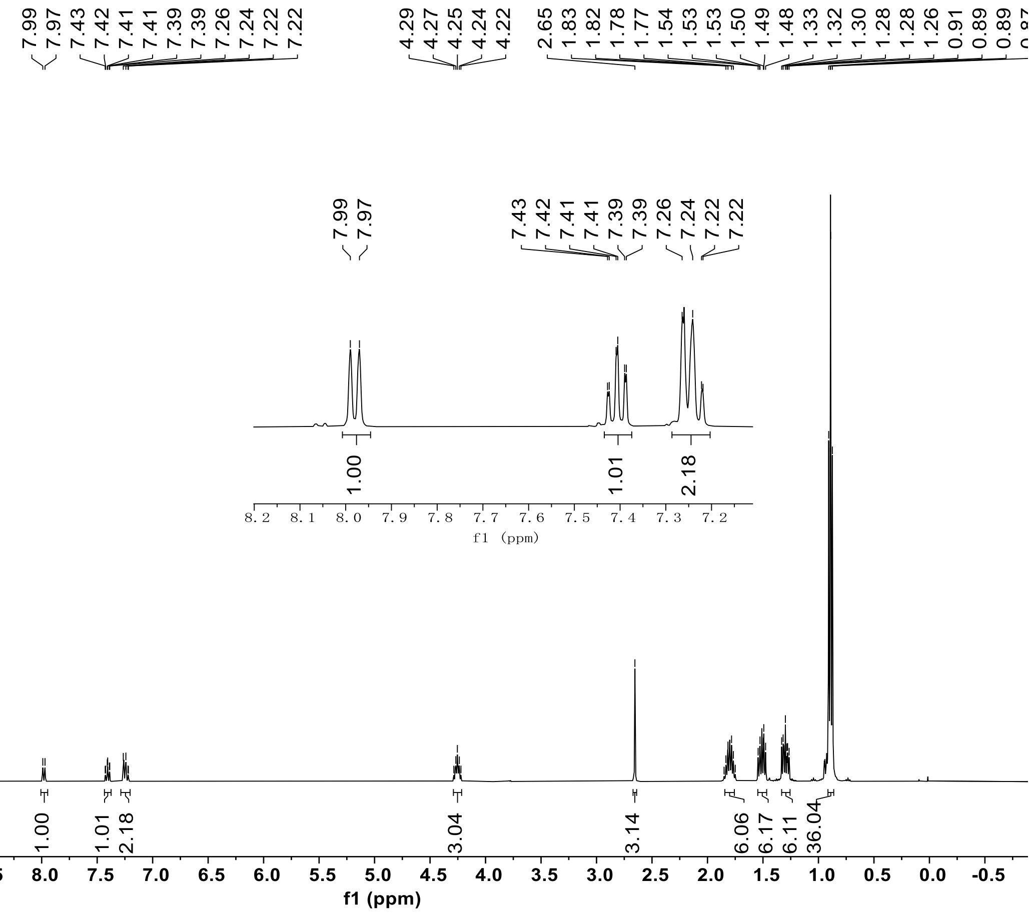

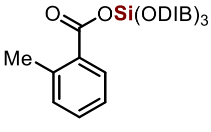

43a, ^{13}C-NMR
(101 MHz, $CDCl_3$)

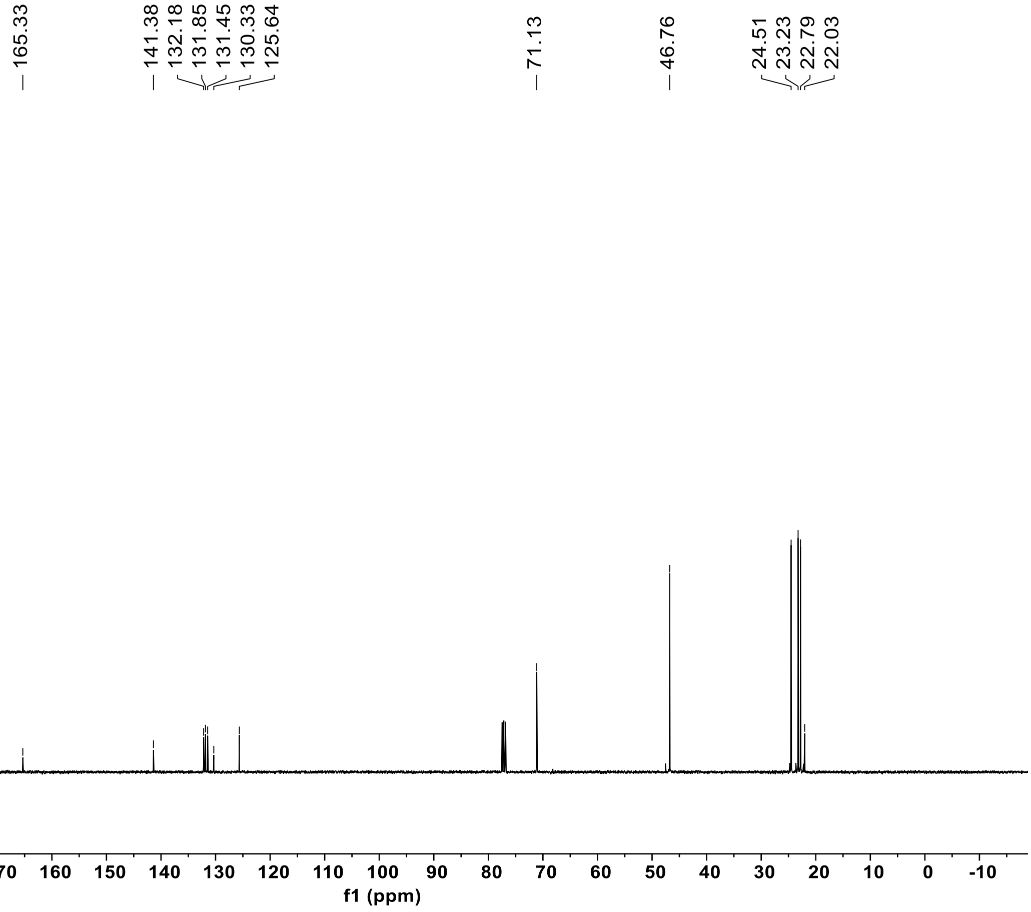

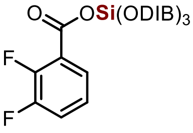

44a, ^{1}H-NMR
(400 MHz, $CDCl_3$)

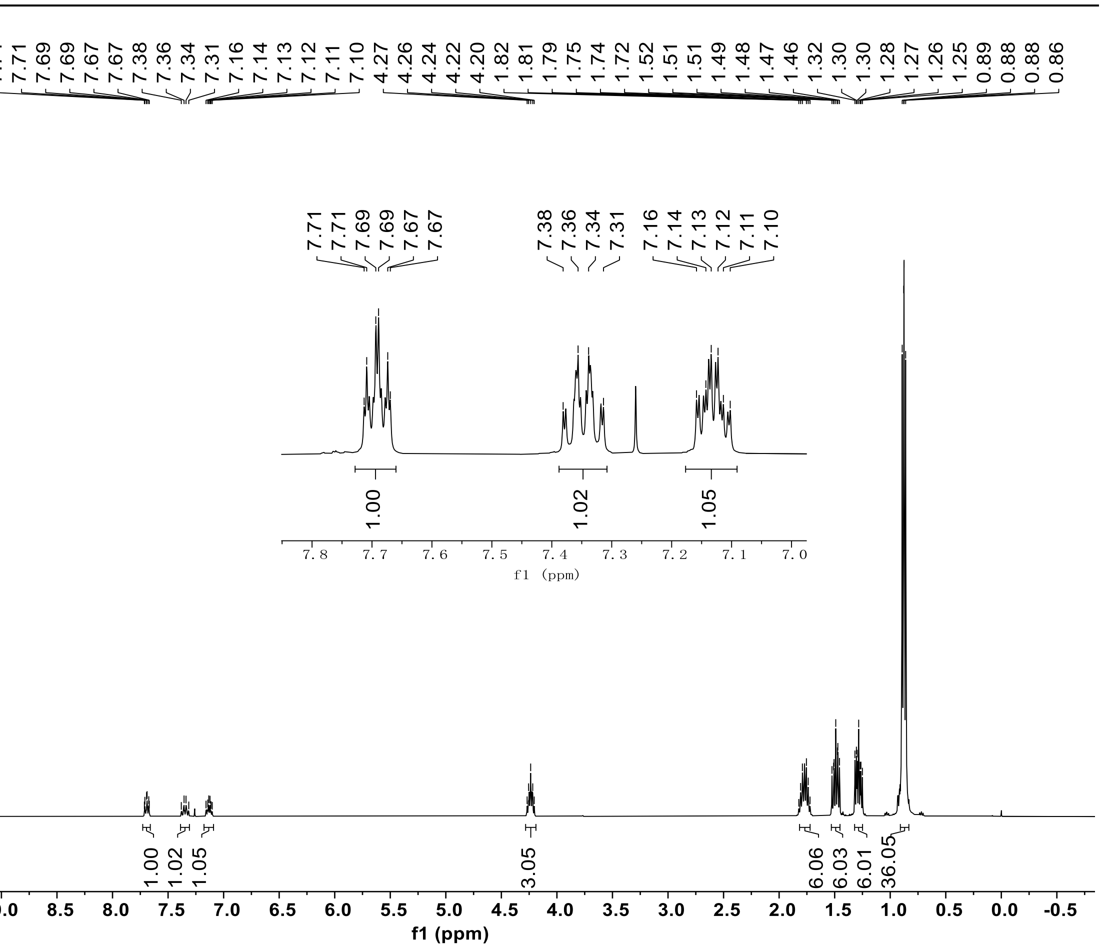

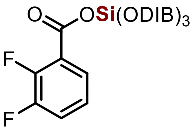

44a, ^{13}C-NMR
(101 MHz, $CDCl_3$)

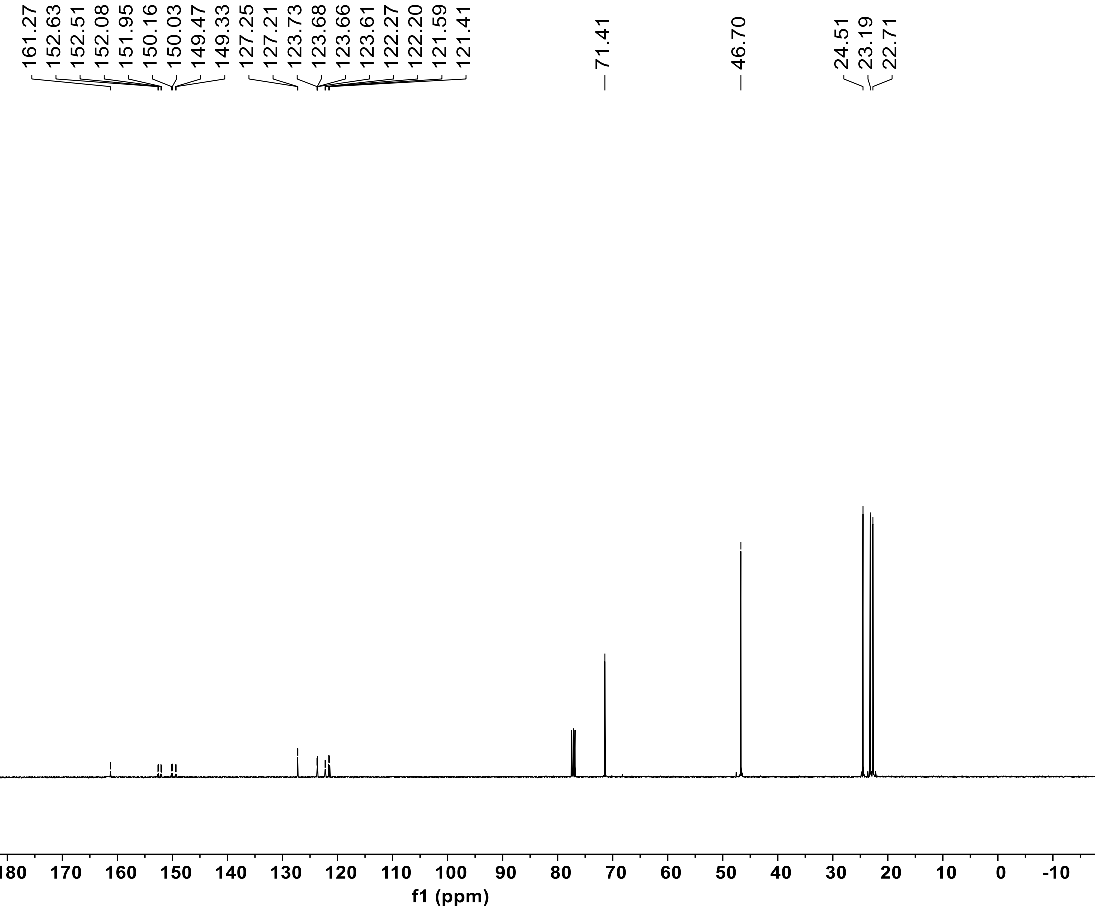

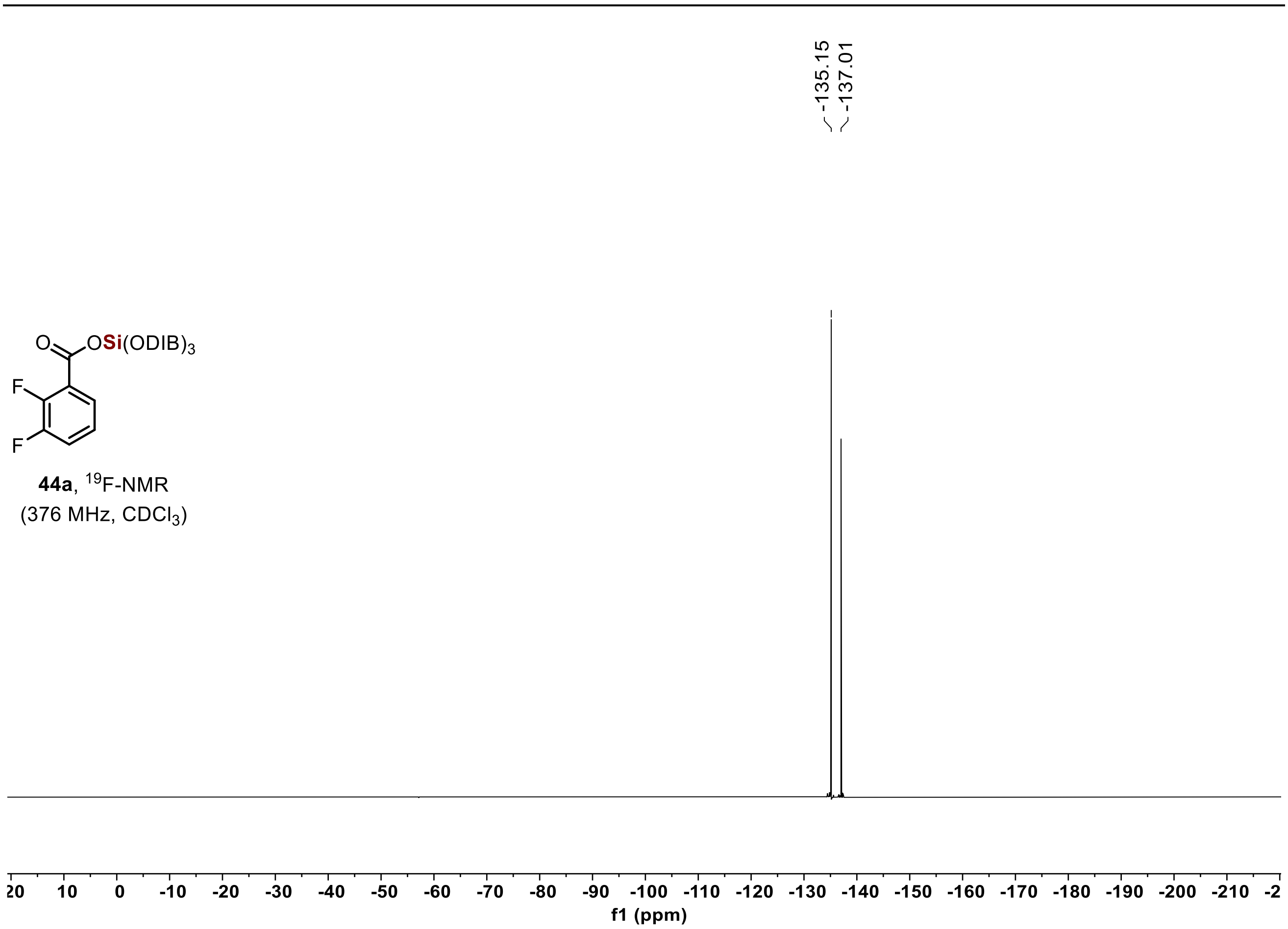

44a, ^{19}F-NMR (376 MHz, $CDCl_3$)

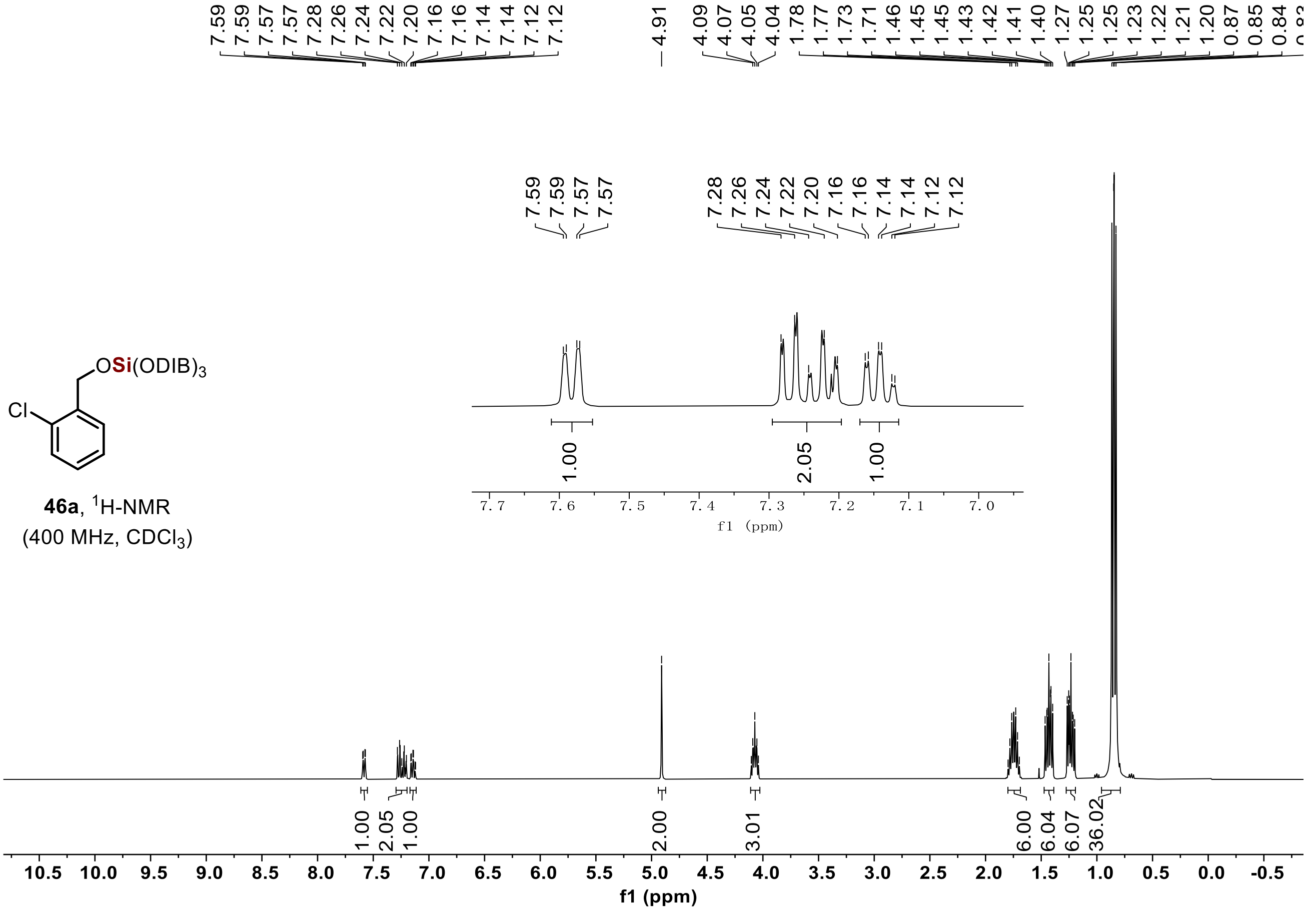

46a, ^{1}H-NMR (400 MHz, $CDCl_3$)

46a, ^{13}C-NMR
(101 MHz, $CDCl_3$)

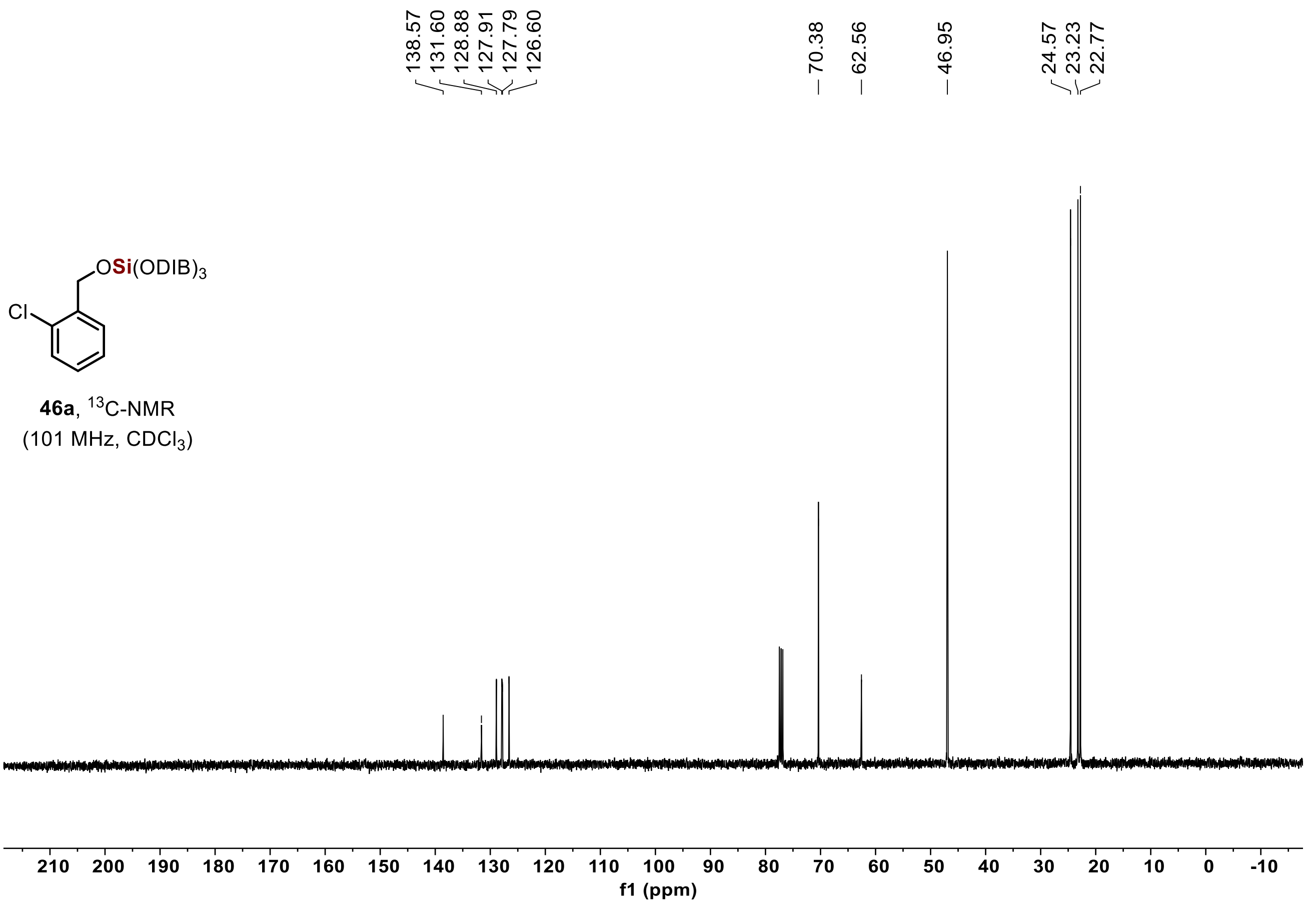

47a, ^{1}H-NMR
(400 MHz, $CDCl_3$)

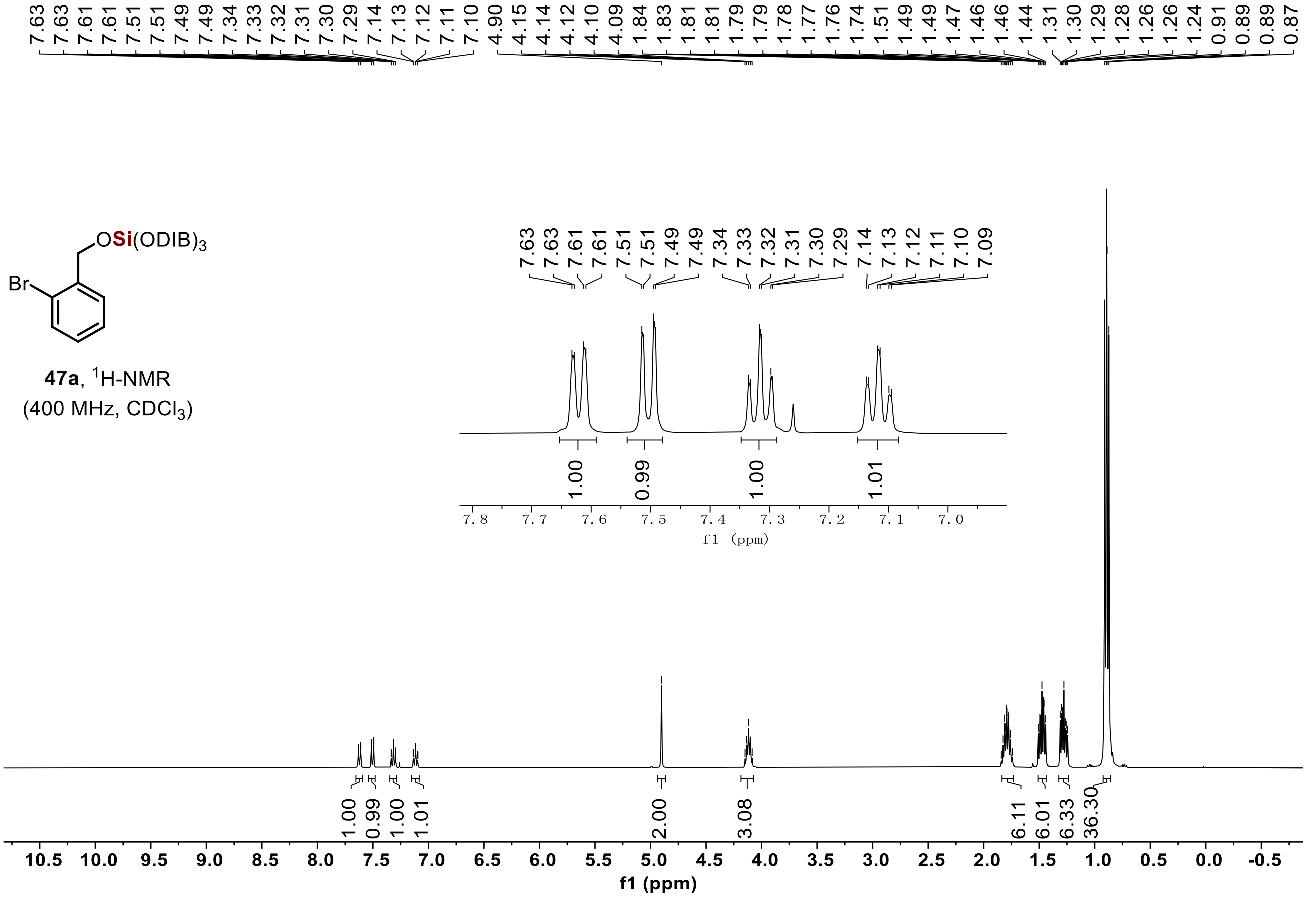

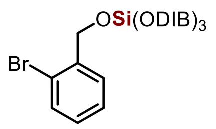

47a, ^{13}C-NMR
(101 MHz, $CDCl_3$)

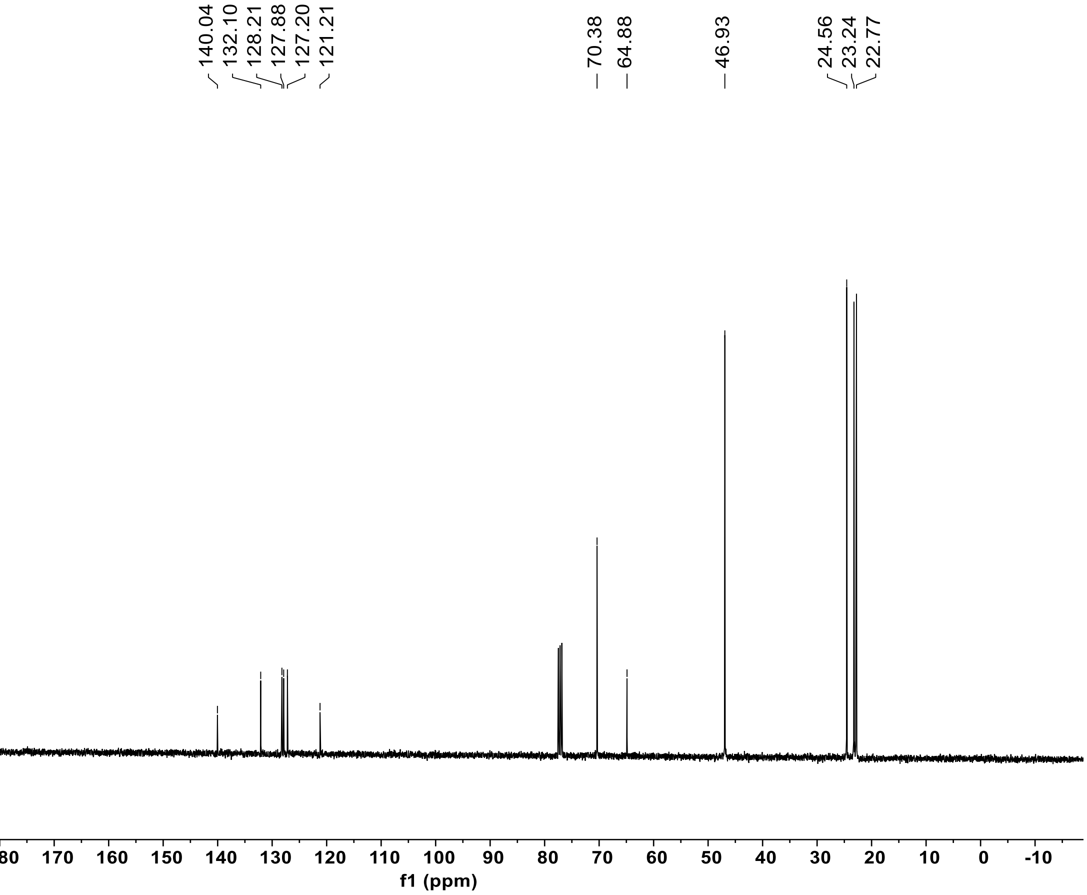

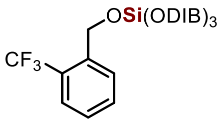

48a, ^{1}H-NMR
(400 MHz, $CDCl_3$)

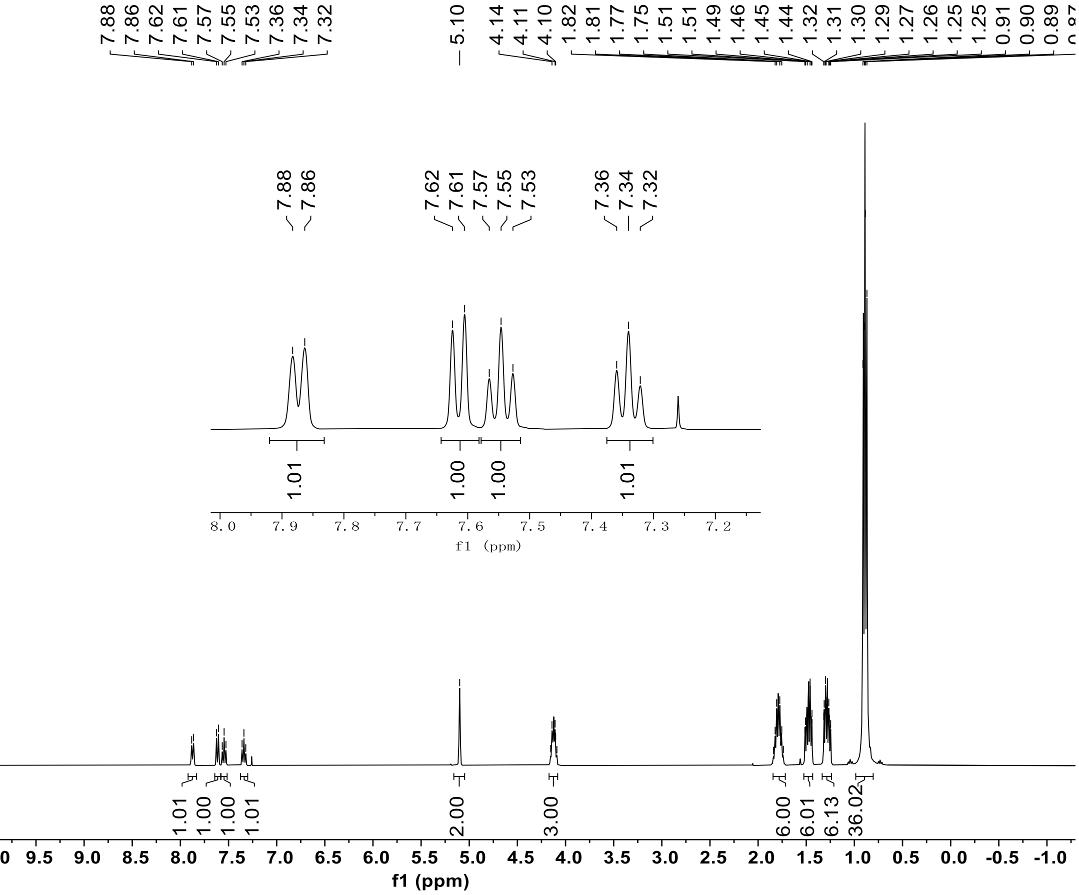

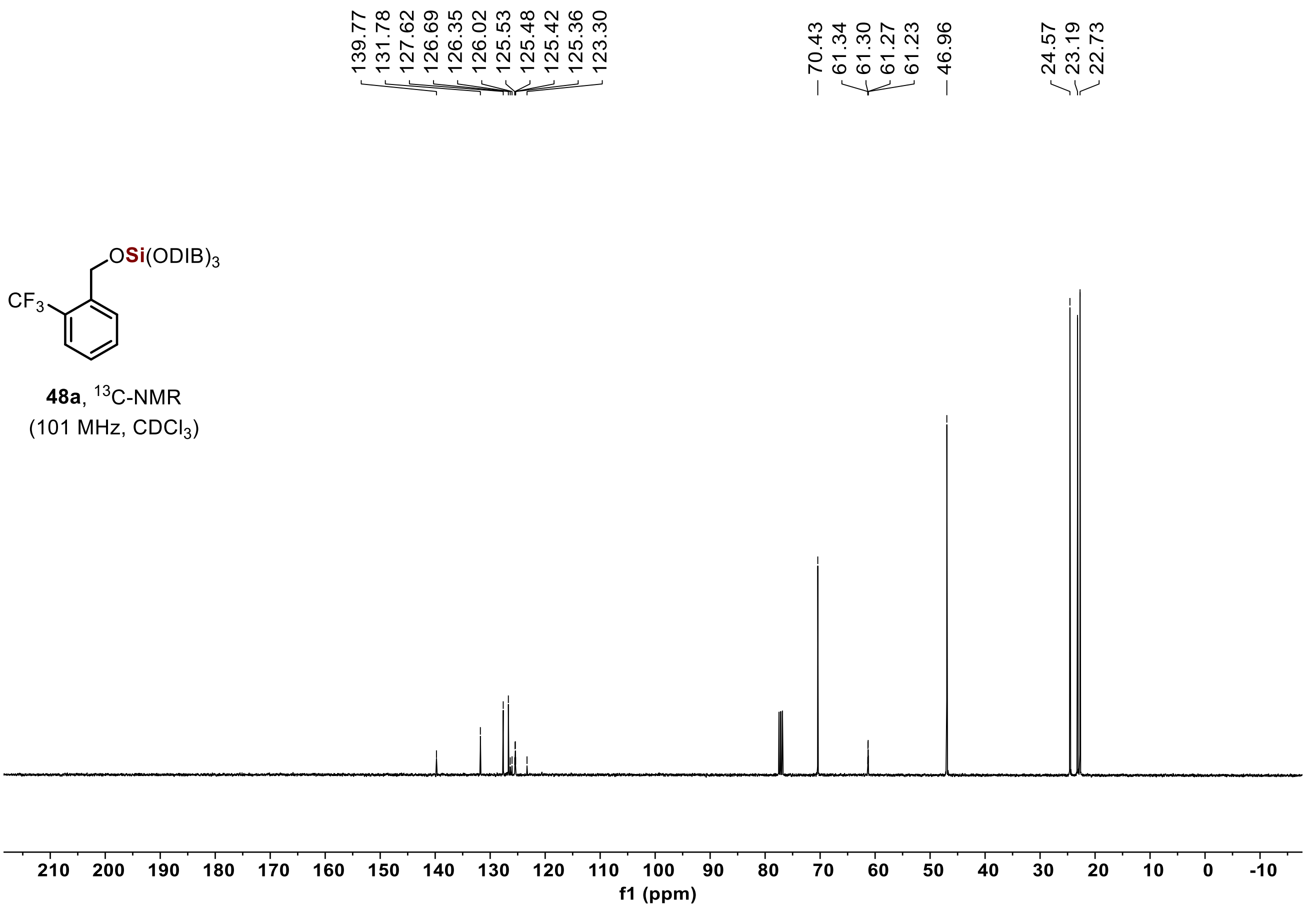

48a, ^{13}C-NMR
(101 MHz, $CDCl_3$)

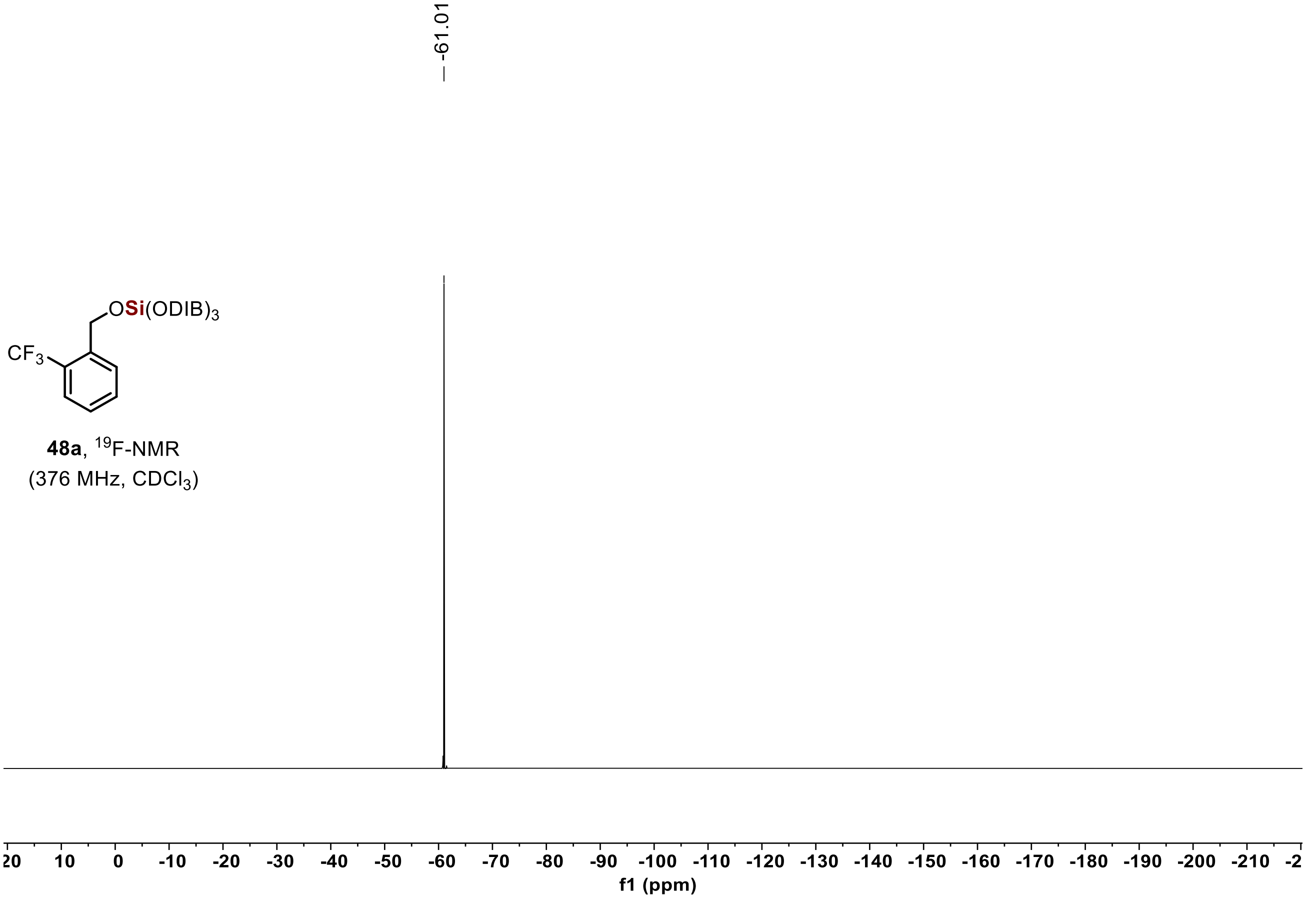

48a, ^{19}F-NMR
(376 MHz, $CDCl_3$)

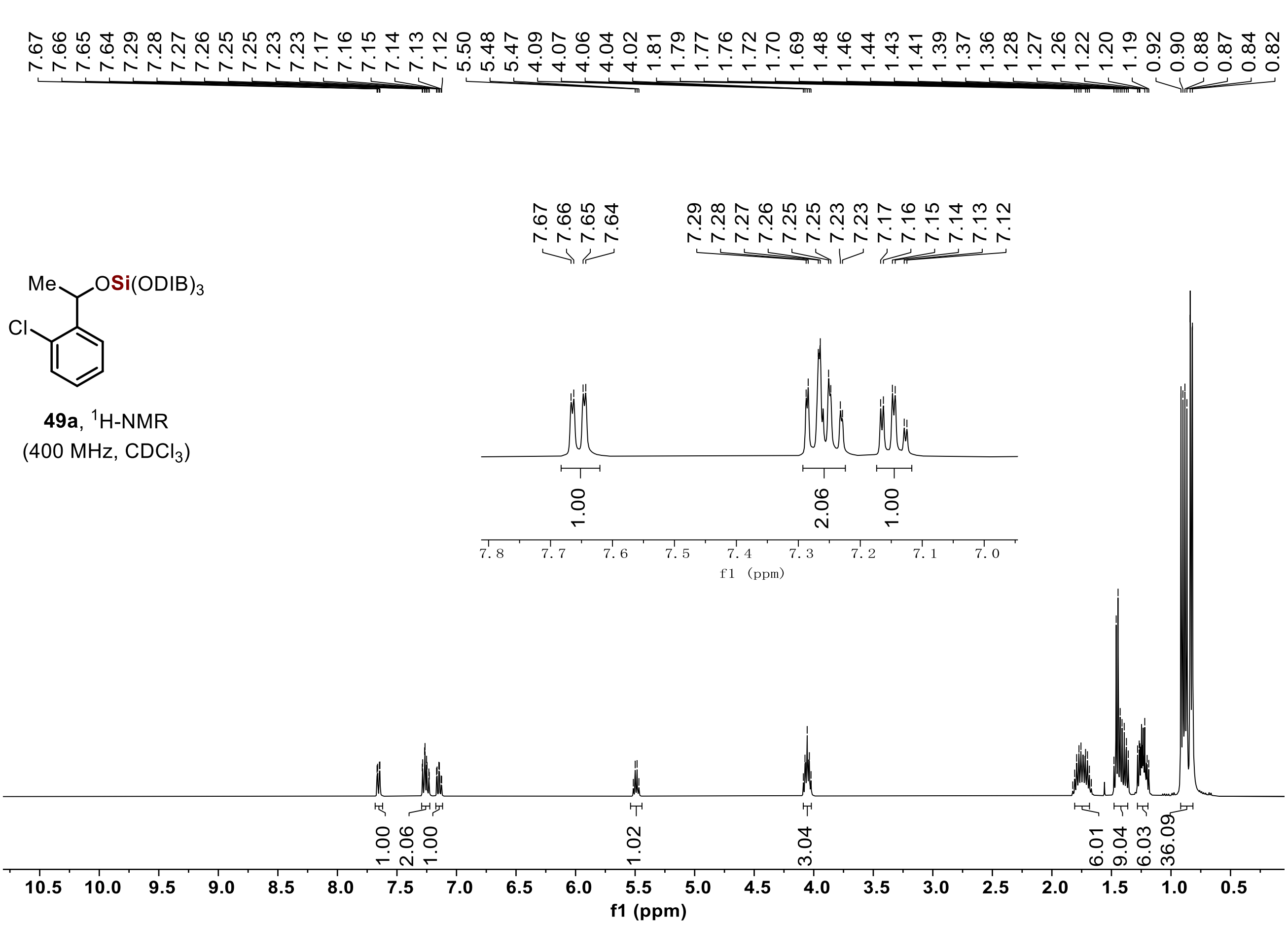
Me
OSi(ODIB)3
Cl
49a, 1H-NMR
(400 MHz, CDCl3)
f1 (ppm)

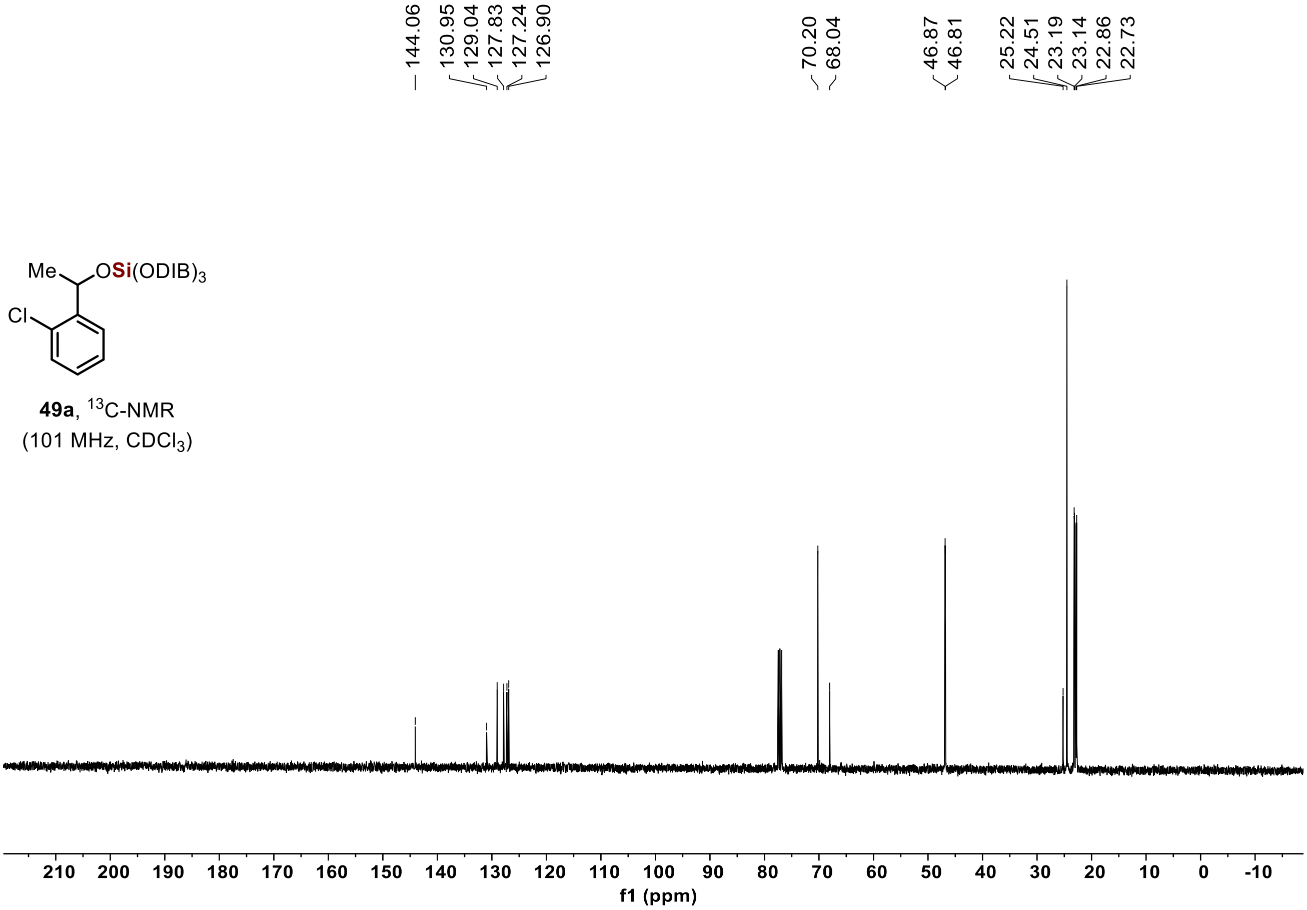
Me
OSi(ODIB)3
Cl
49a, 13C-NMR
(101 MHz, CDCl3)
f1 (ppm)

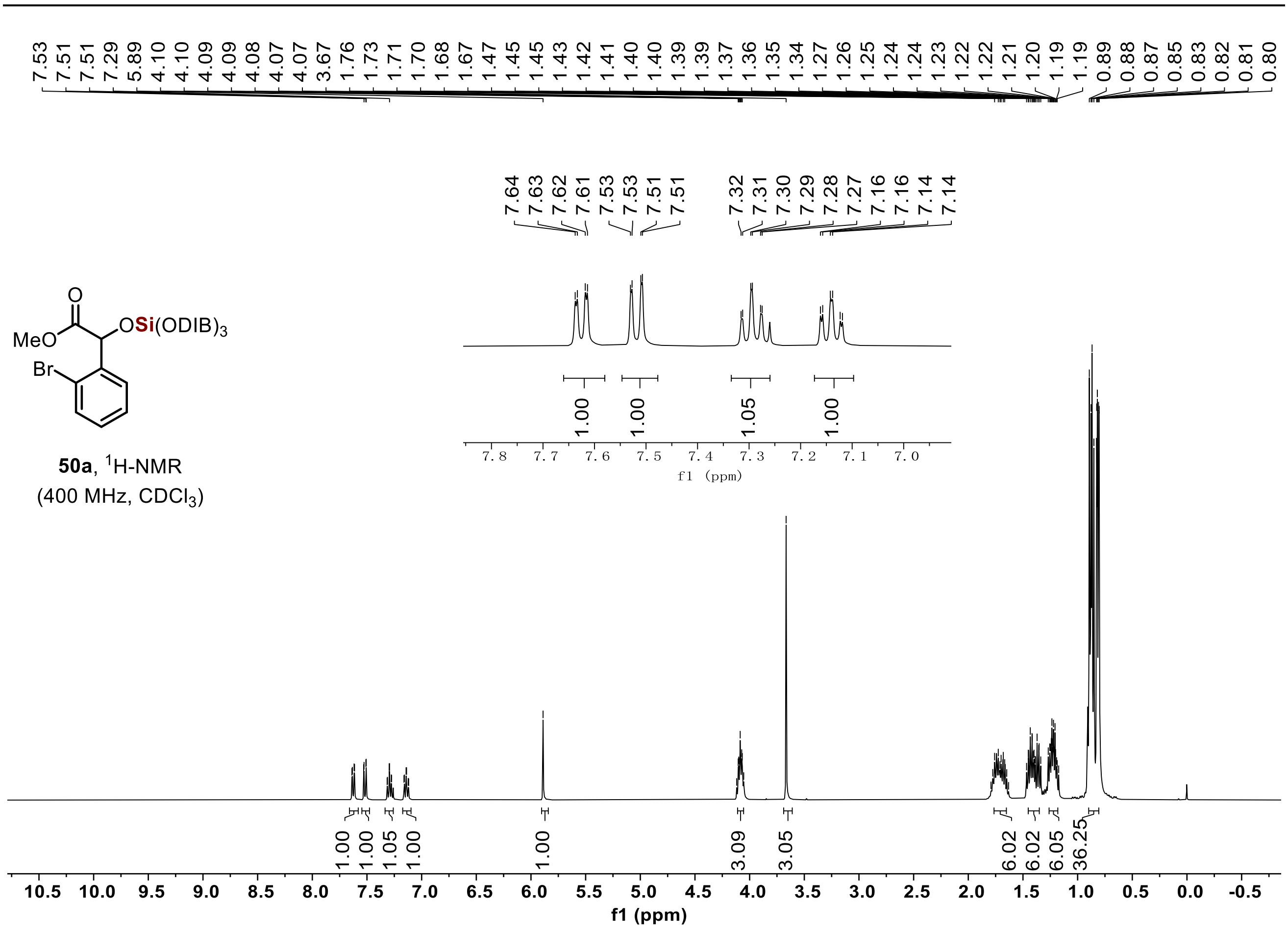

50a, ^{1}H-NMR
(400 MHz, $CDCl_3$)

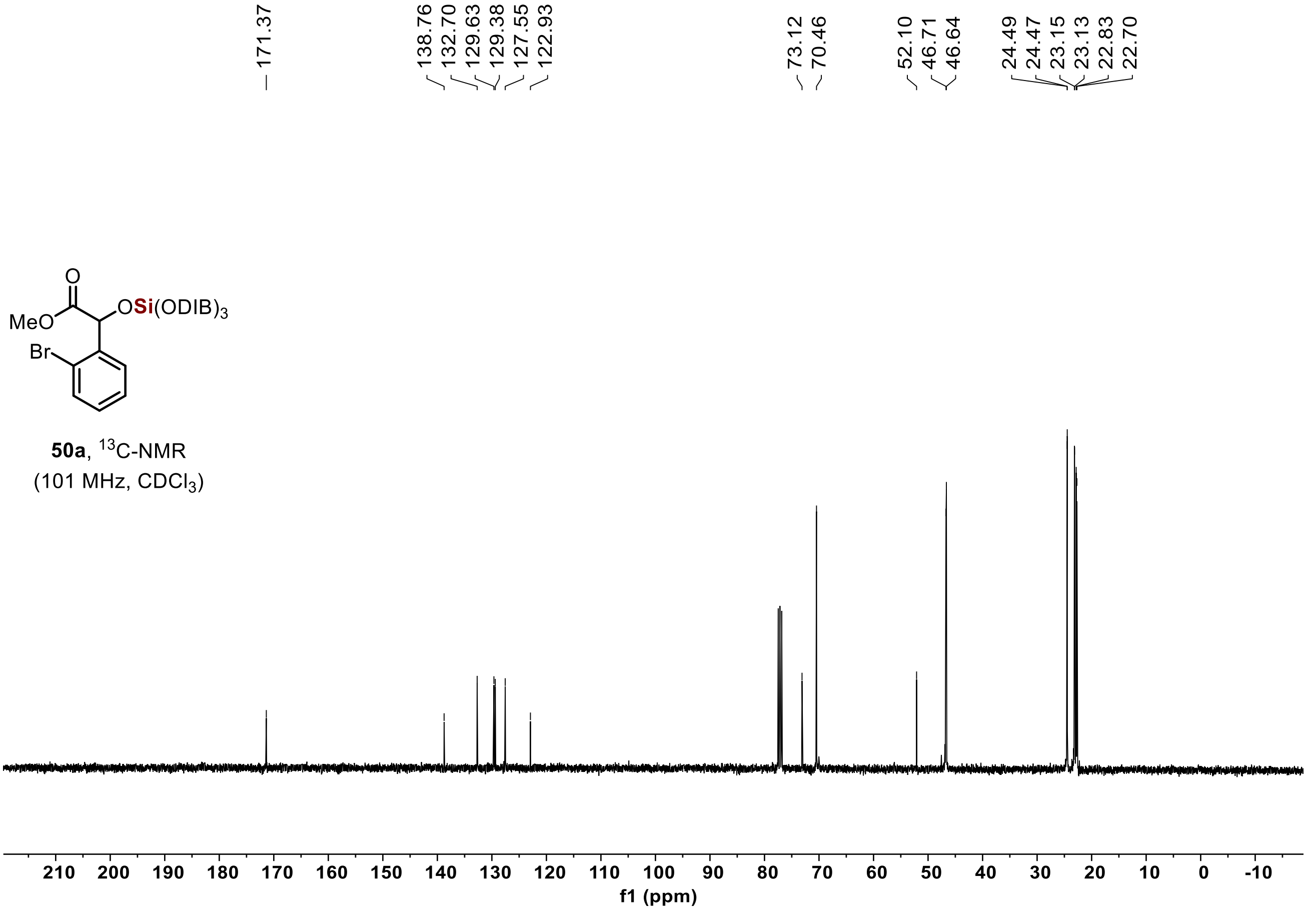

50a, ^{13}C-NMR
(101 MHz, $CDCl_3$)

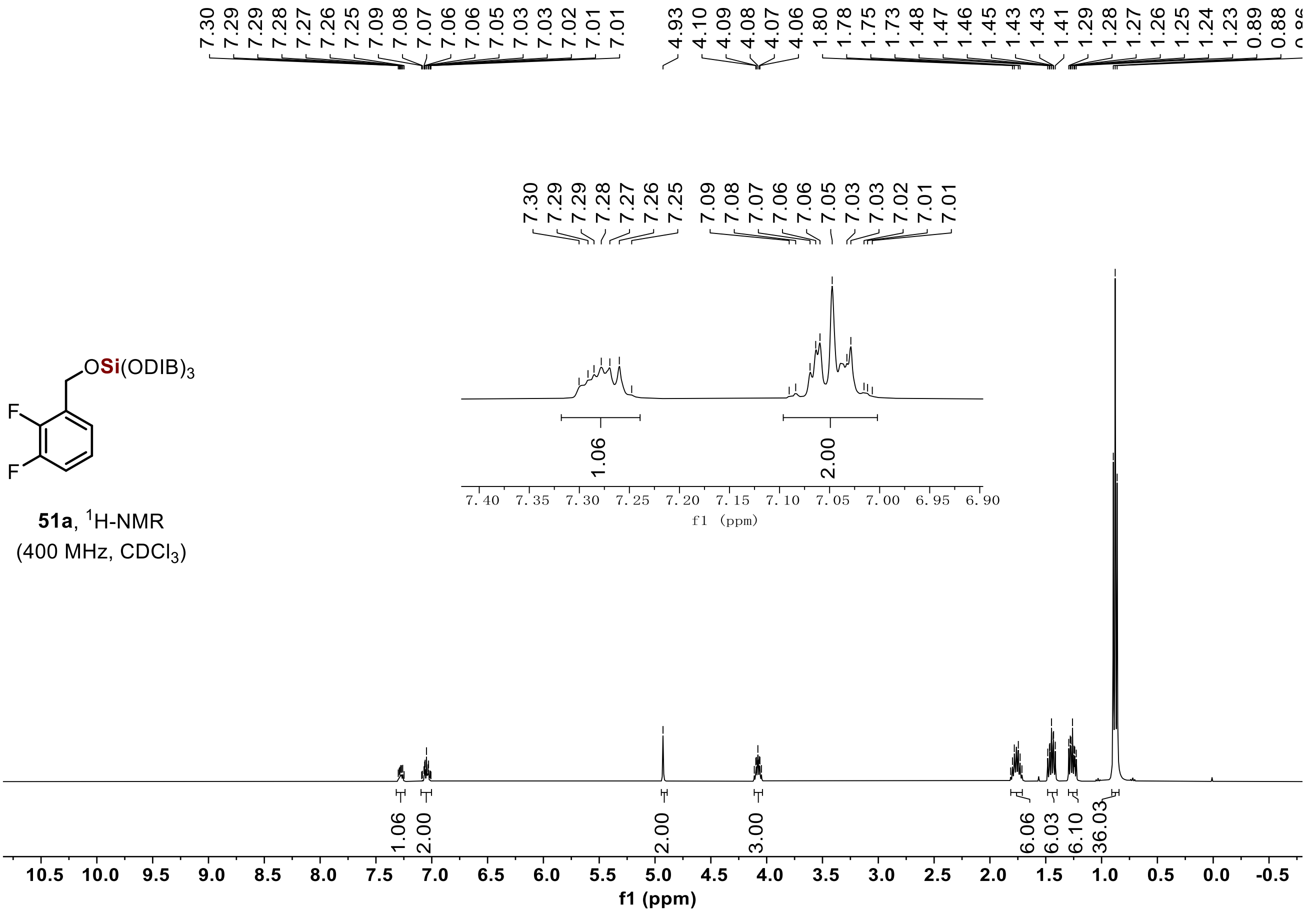

51a, ^{1}H-NMR
(400 MHz, $CDCl_3$)

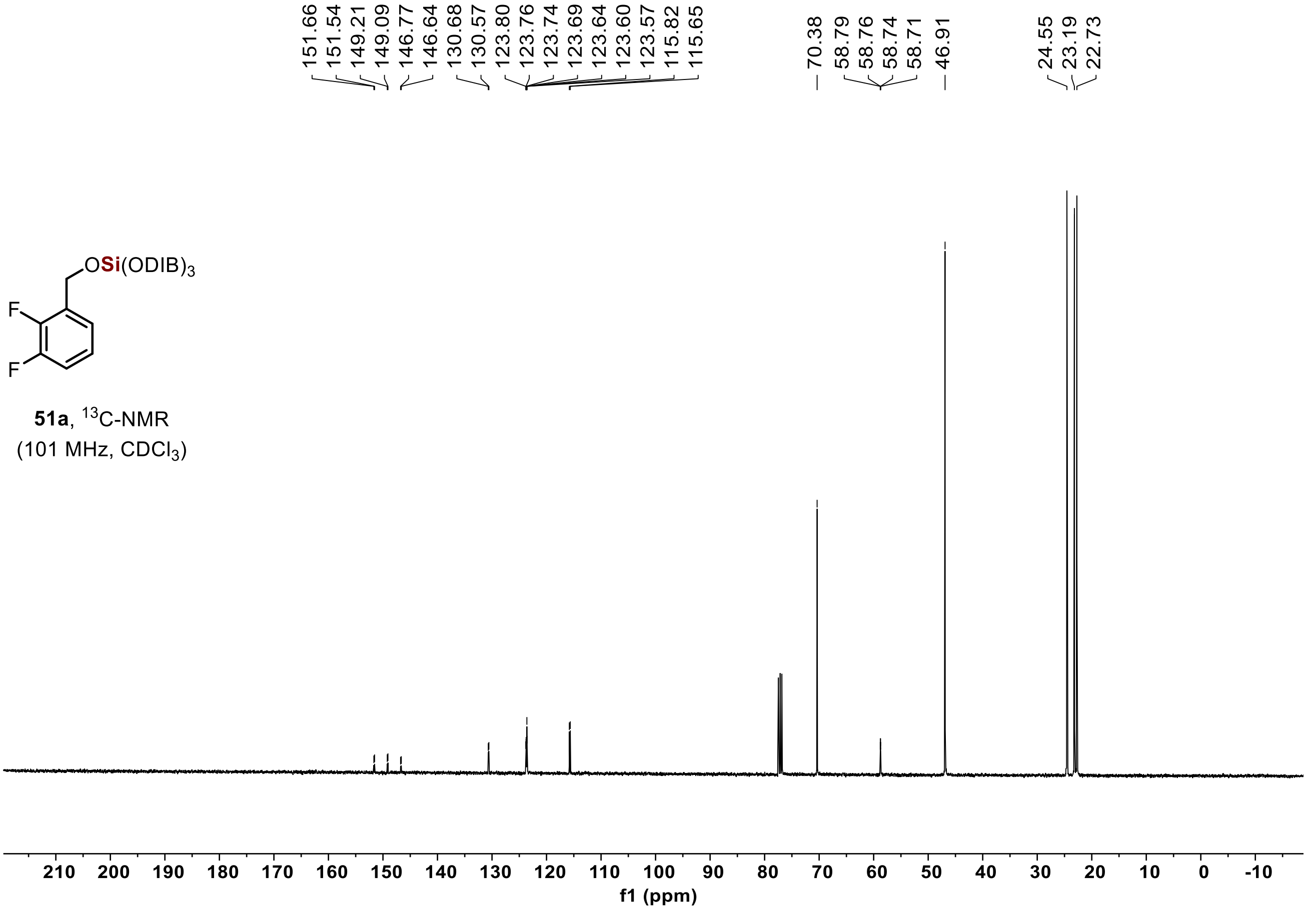

51a, ^{13}C-NMR
(101 MHz, $CDCl_3$)

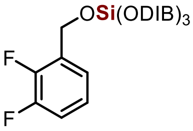

51a, ^{19}F-NMR
(376 MHz, $CDCl_3$)

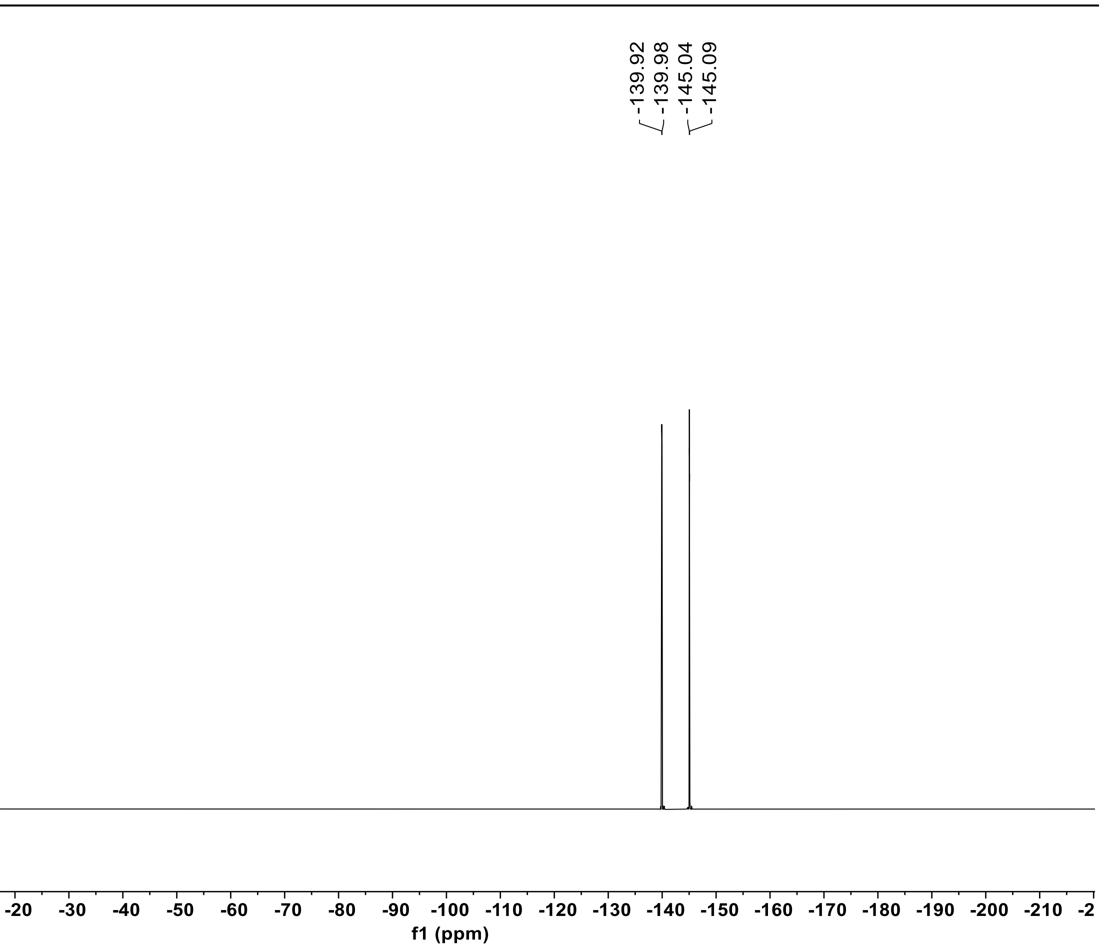

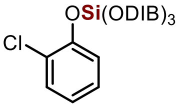

53a, ^{1}H-NMR
(400 MHz, $CDCl_3$)

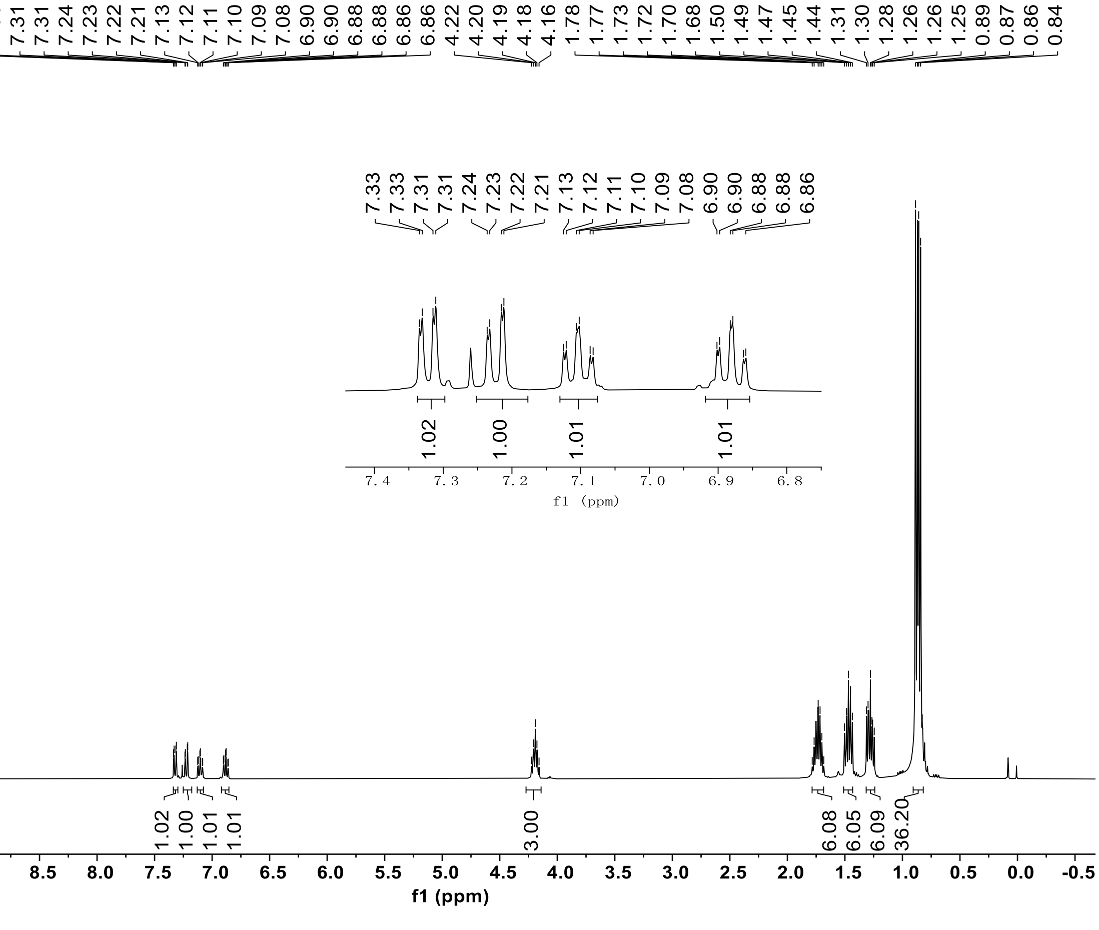

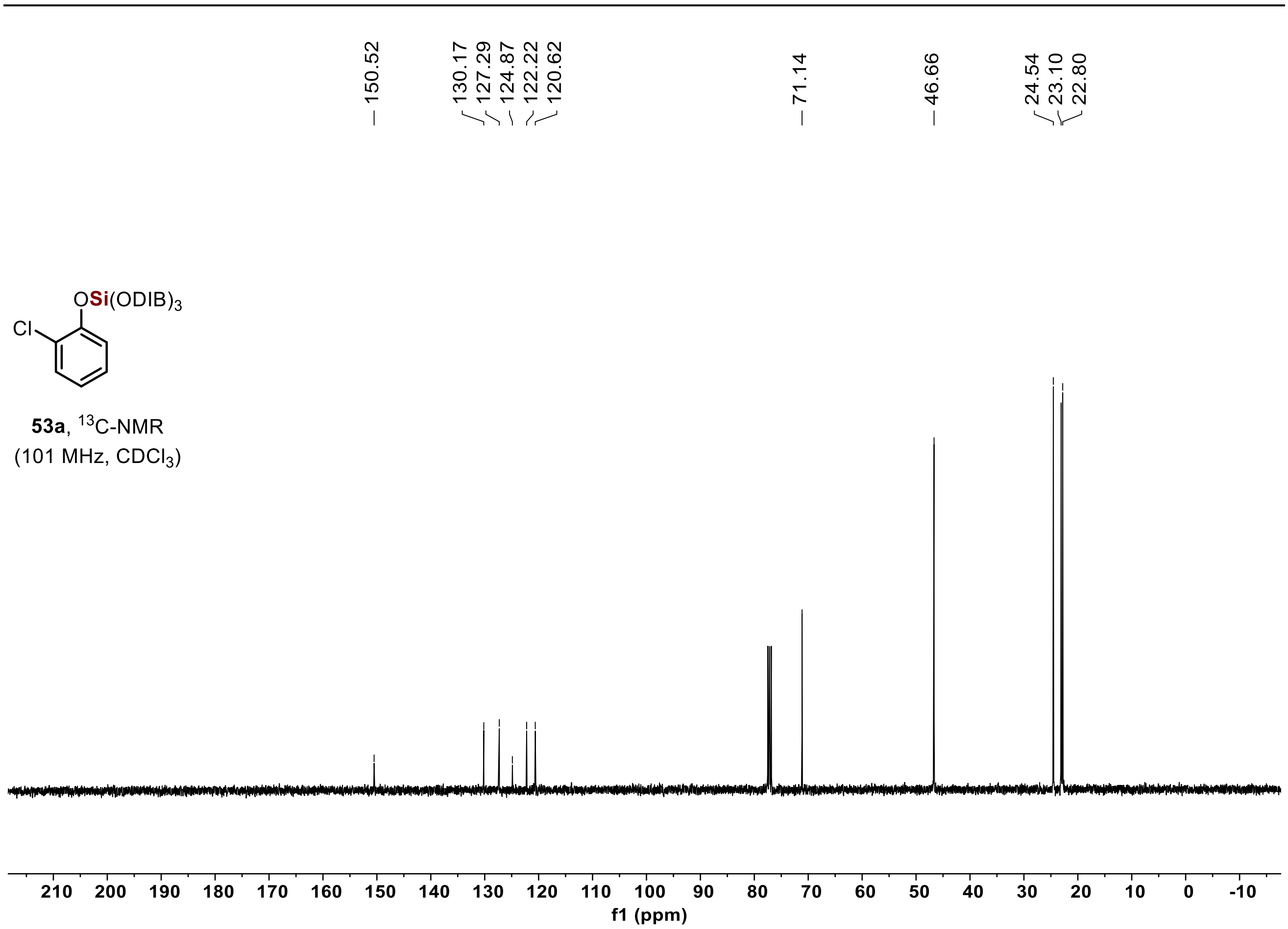
150.52
130.17
127.29
124.87
122.22
120.62
71.14
46.66
24.54
23.10
22.80
OSi(ODIB)3
Cl
53a, 13C-NMR
(101 MHz, CDCl3)
210 200 190 180 170 160 150 140 130 120 110 100 90 80 70 60 50 40 30 20 10 0 -10
f1 (ppm)

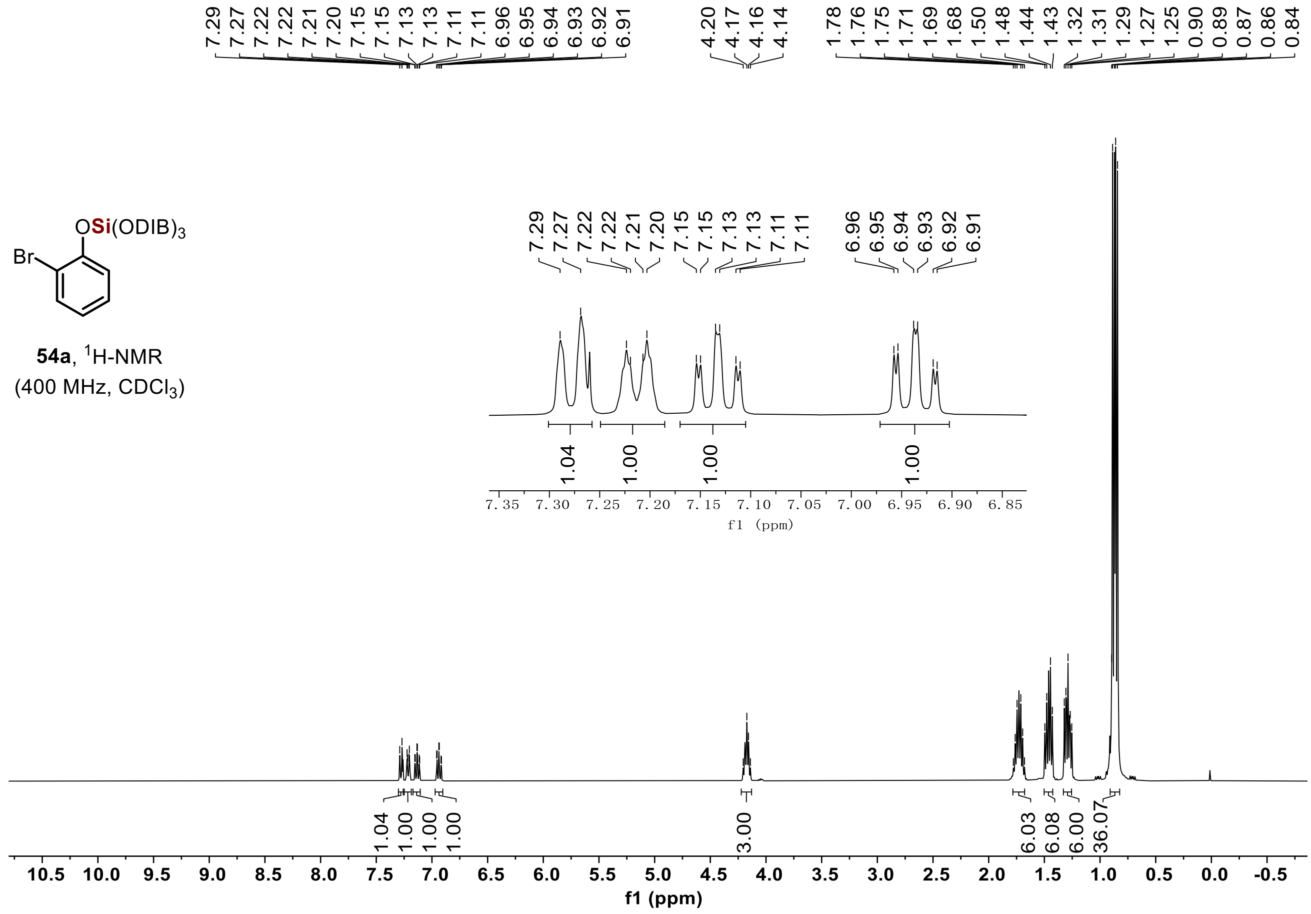
7.29 7.27 7.22 7.22 7.21 7.20 7.15 7.15 7.13 7.13 7.11 7.11 6.96 6.95 6.94 6.93 6.92 6.91
4.20 4.17 4.16 4.14
1.78 1.76 1.75 1.71 1.69 1.68 1.50 1.48 1.44 1.43 1.32 1.31 1.29 1.27 1.25 0.90 0.89 0.87 0.86 0.84
OSi(ODIB)3
Br
54a, 1H-NMR
(400 MHz, CDCl3)
1.04
1.00
1.00
1.00
7.35 7.30 7.25 7.20 7.15 7.10 7.05 7.00 6.95 6.90 6.85
f1 (ppm)
1.04
1.00
1.00
1.00
3.00
6.03
6.08
6.00
36.07
10.5 10.0 9.5 9.0 8.5 8.0 7.5 7.0 6.5 6.0 5.5 5.0 4.5 4.0 3.5 3.0 2.5 2.0 1.5 1.0 0.5 0.0 -0.5
f1 (ppm)

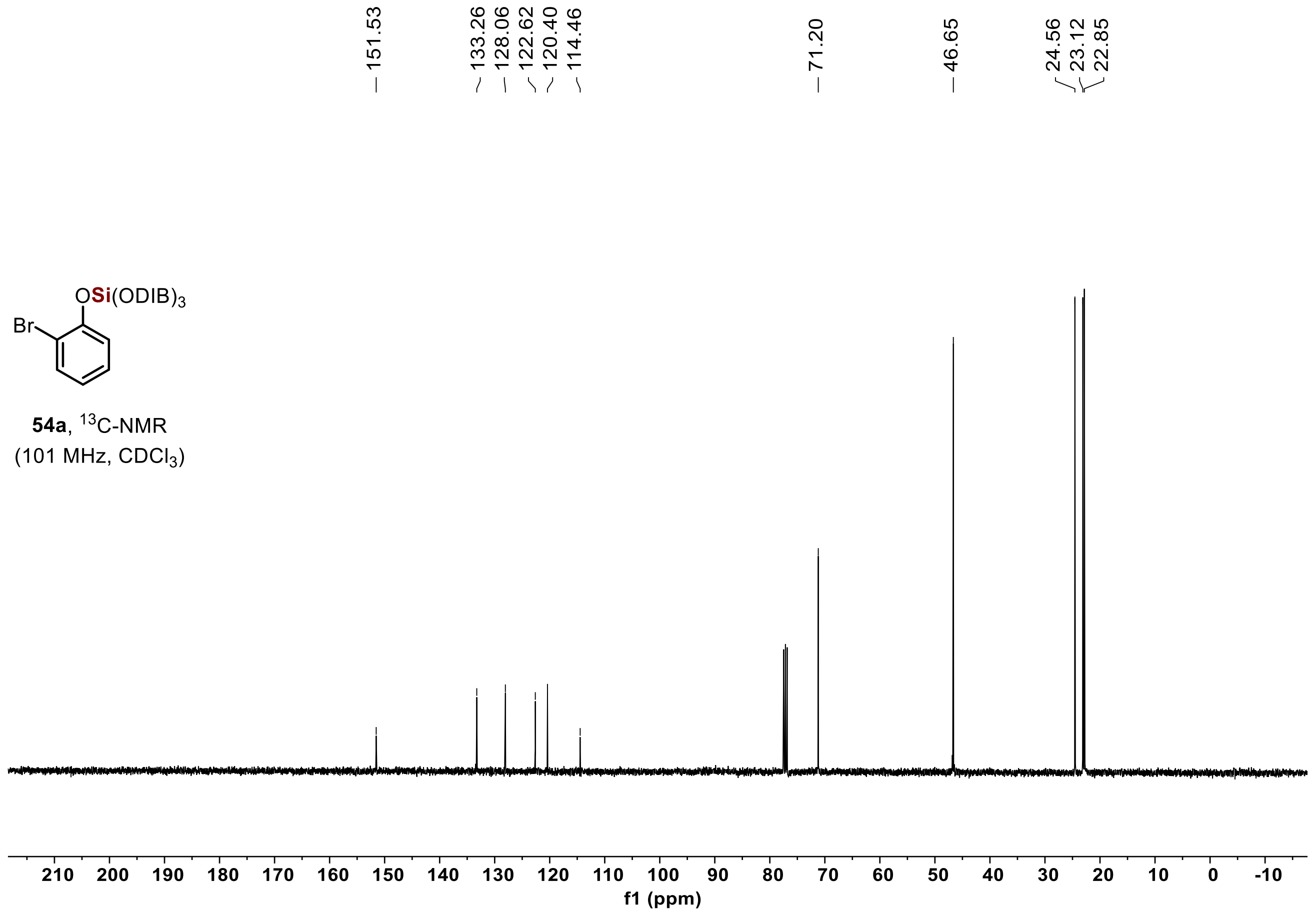
151.53
133.26
128.06
122.62
120.40
114.46
71.20
46.65
24.56
23.12
22.85
OSi(ODIB)3
Br
54a, 13C-NMR
(101 MHz, CDCl3)
f1 (ppm)

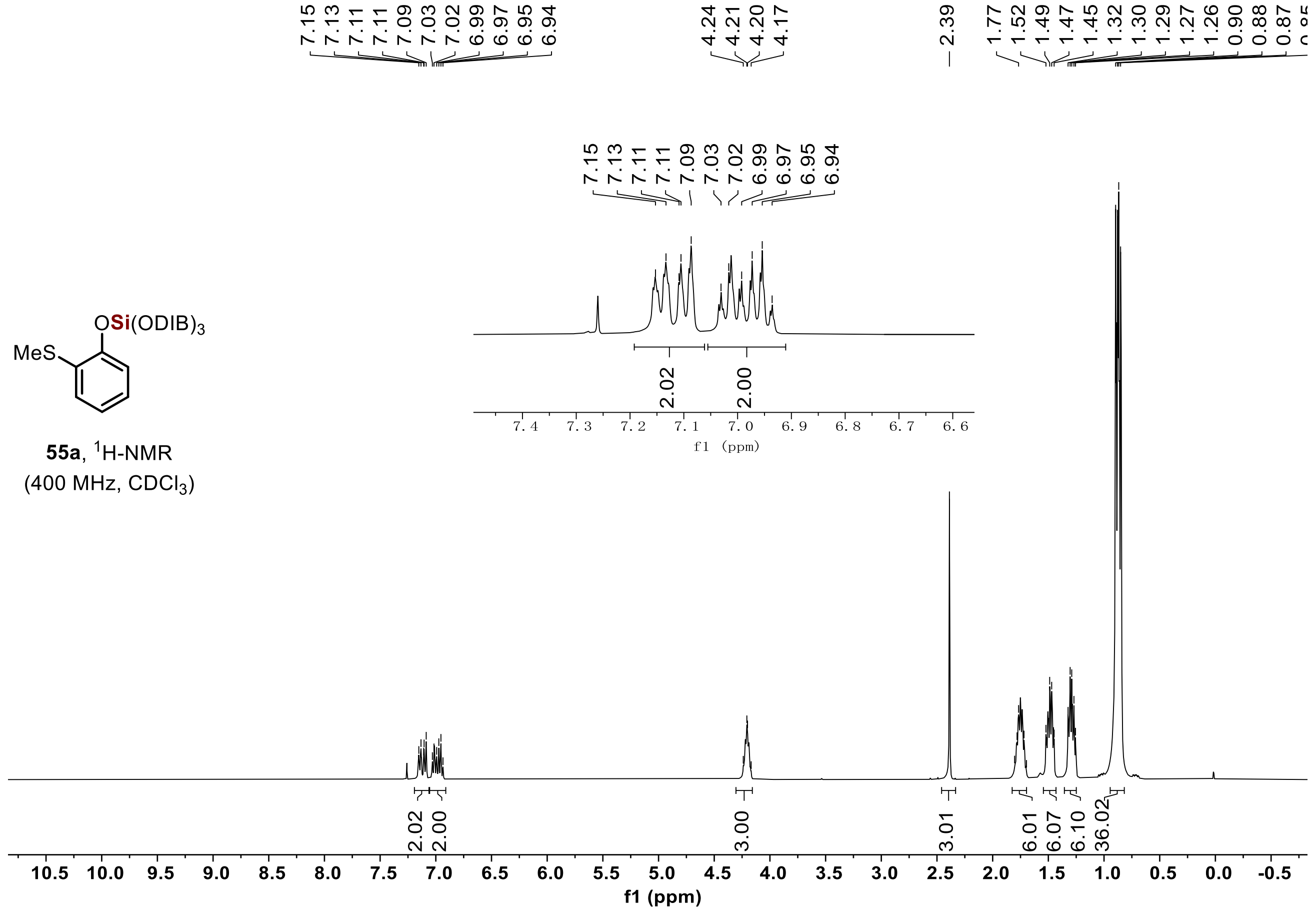
7.15
7.13
7.11
7.11
7.09
7.03
7.02
6.99
6.97
6.95
6.94
4.24
4.21
4.20
4.17
2.39
1.77
1.52
1.49
1.47
1.45
1.32
1.30
1.29
1.27
1.26
0.90
0.88
0.87
OSi(ODIB)3
MeS
55a, 1H-NMR
(400 MHz, CDCl3)
2.02
2.00
3.00
3.01
6.01
6.07
6.10
36.02
f1 (ppm)

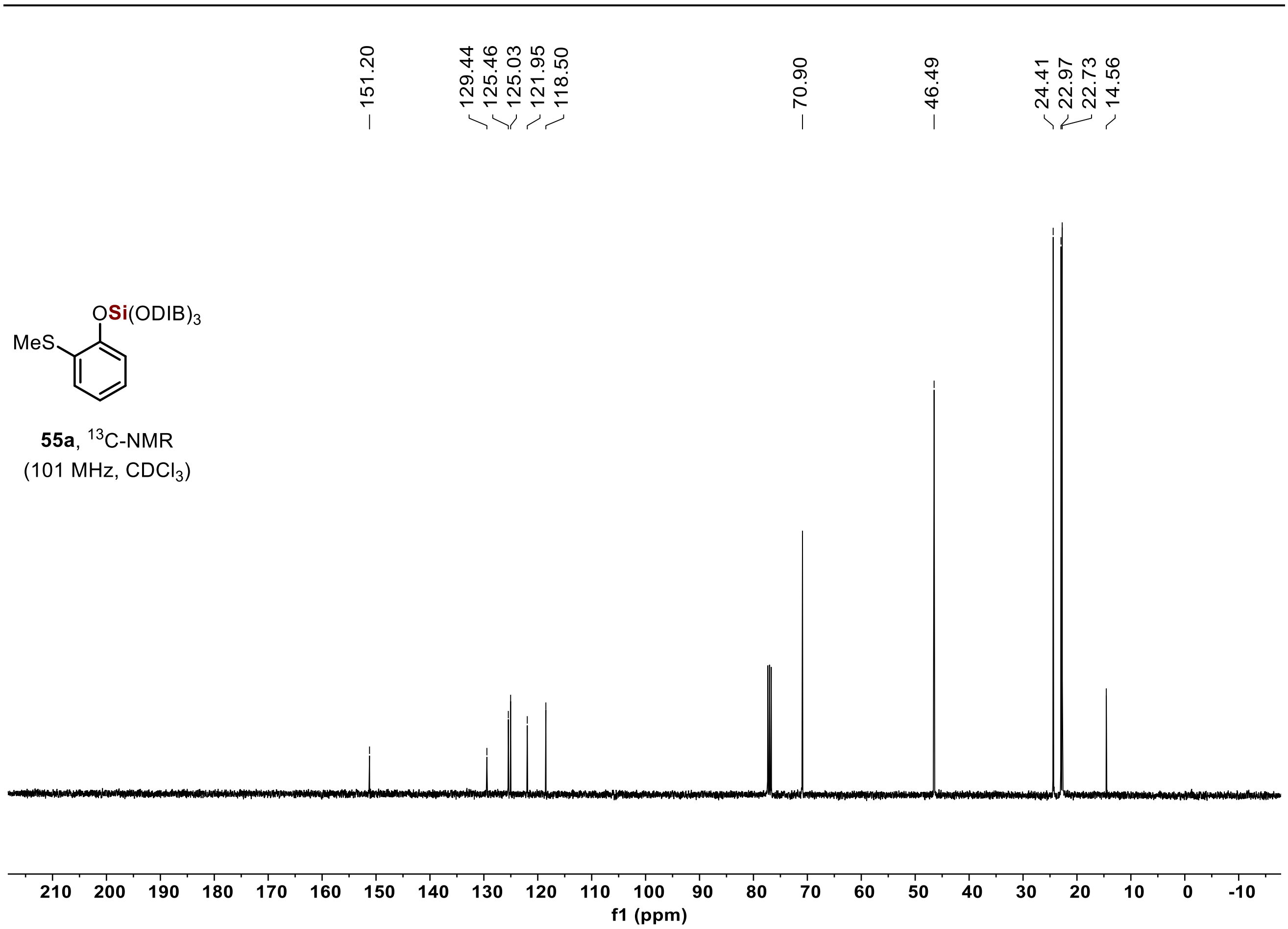

151.20
129.44
125.46
125.03
121.95
118.50
70.90
46.49
24.41
22.97
22.73
14.56
OSi(ODIB)3
MeS
55a, 13C-NMR
(101 MHz, CDCl3)
210 200 190 180 170 160 150 140 130 120 110 100 90 80 70 60 50 40 30 20 10 0 -10
f1 (ppm)

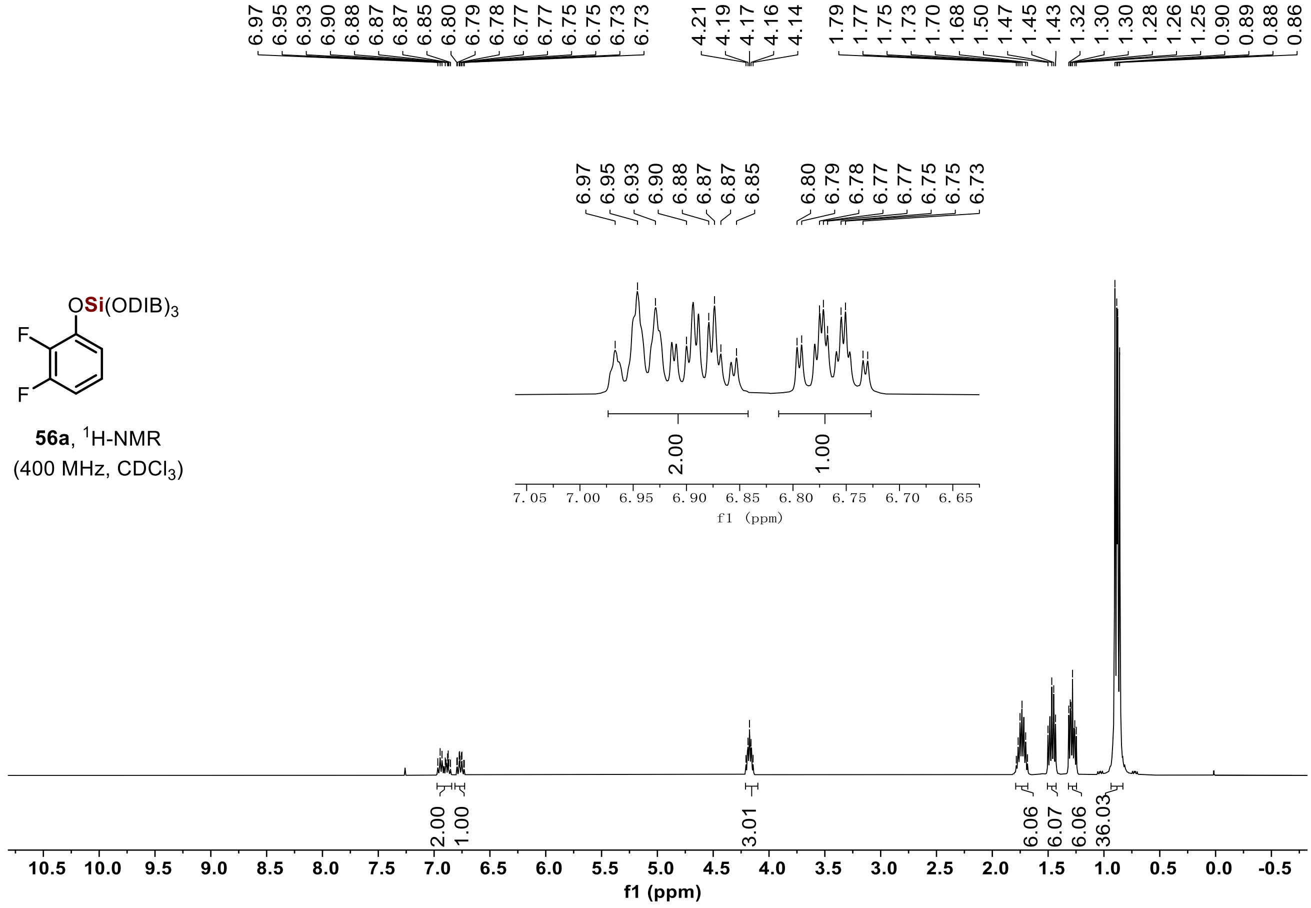

6.97 6.95 6.93 6.90 6.88 6.87 6.87 6.85 6.80 6.79 6.78 6.77 6.77 6.75 6.75 6.73 6.73
4.21 4.19 4.17 4.16 4.14
1.79 1.77 1.75 1.73 1.70 1.68 1.50 1.47 1.45 1.43 1.32 1.30 1.30 1.28 1.26 1.25 0.90 0.89 0.88 0.86
6.97 6.95 6.93 6.90 6.88 6.87 6.87 6.85
6.80 6.79 6.78 6.77 6.77 6.75 6.75 6.73
2.00
1.00
7.05 7.00 6.95 6.90 6.85 6.80 6.75 6.70 6.65
f1 (ppm)
OSi(ODIB)3
F
F
56a, 1H-NMR
(400 MHz, CDCl3)
2.00
1.00
3.01
6.06
6.07
6.06
36.03
10.5 10.0 9.5 9.0 8.5 8.0 7.5 7.0 6.5 6.0 5.5 5.0 4.5 4.0 3.5 3.0 2.5 2.0 1.5 1.0 0.5 0.0 -0.5
f1 (ppm)

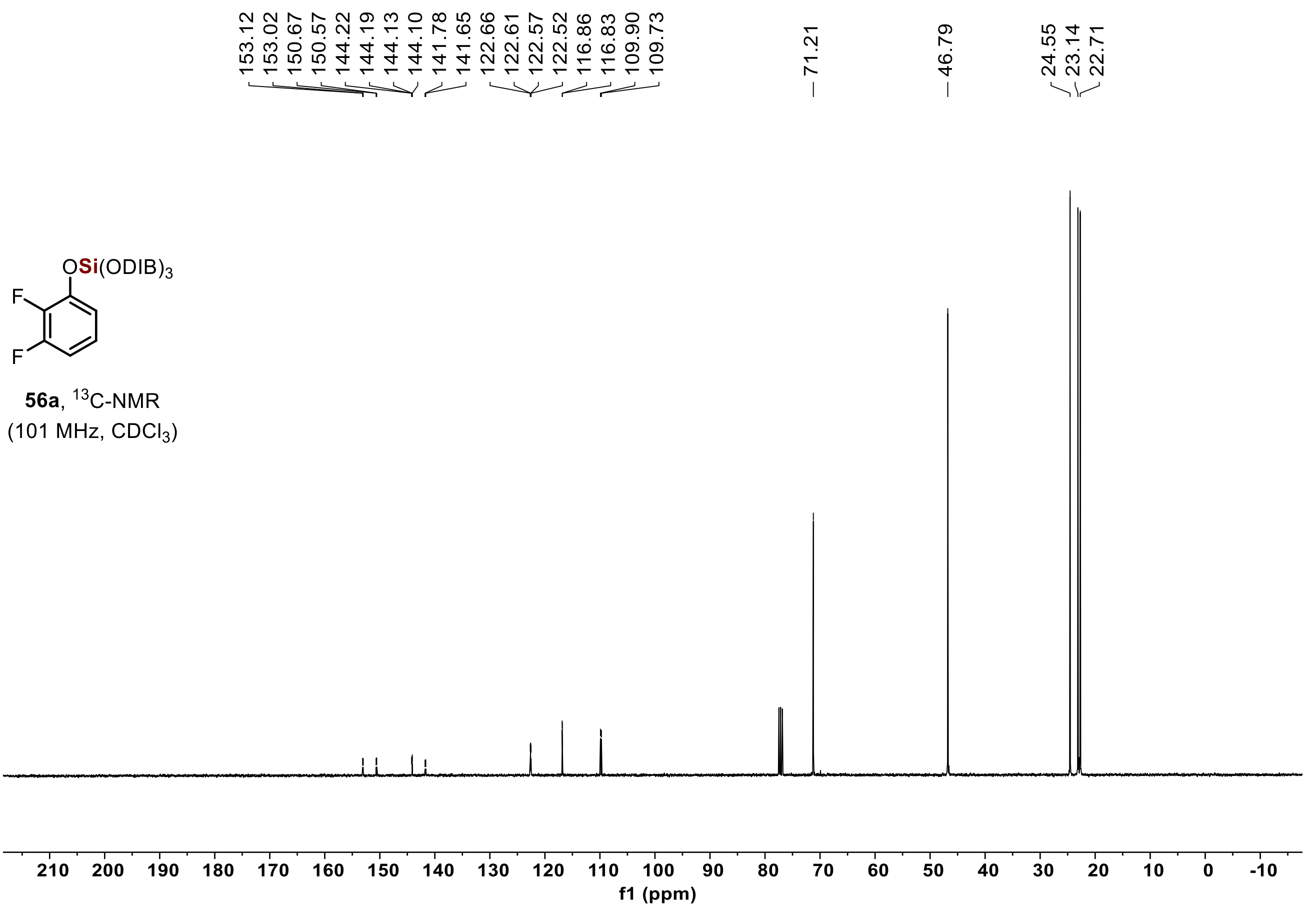
153.12
153.02
150.67
150.57
144.22
144.19
144.13
144.10
141.78
141.65
122.66
122.61
122.57
122.52
116.86
116.83
109.90
109.73
71.21
46.79
24.55
23.14
22.71
OSi(ODIB)3
F
F
56a, 13C-NMR
(101 MHz, CDCl3)
210 200 190 180 170 160 150 140 130 120 110 100 90 80 70 60 50 40 30 20 10 0 -10
f1 (ppm)

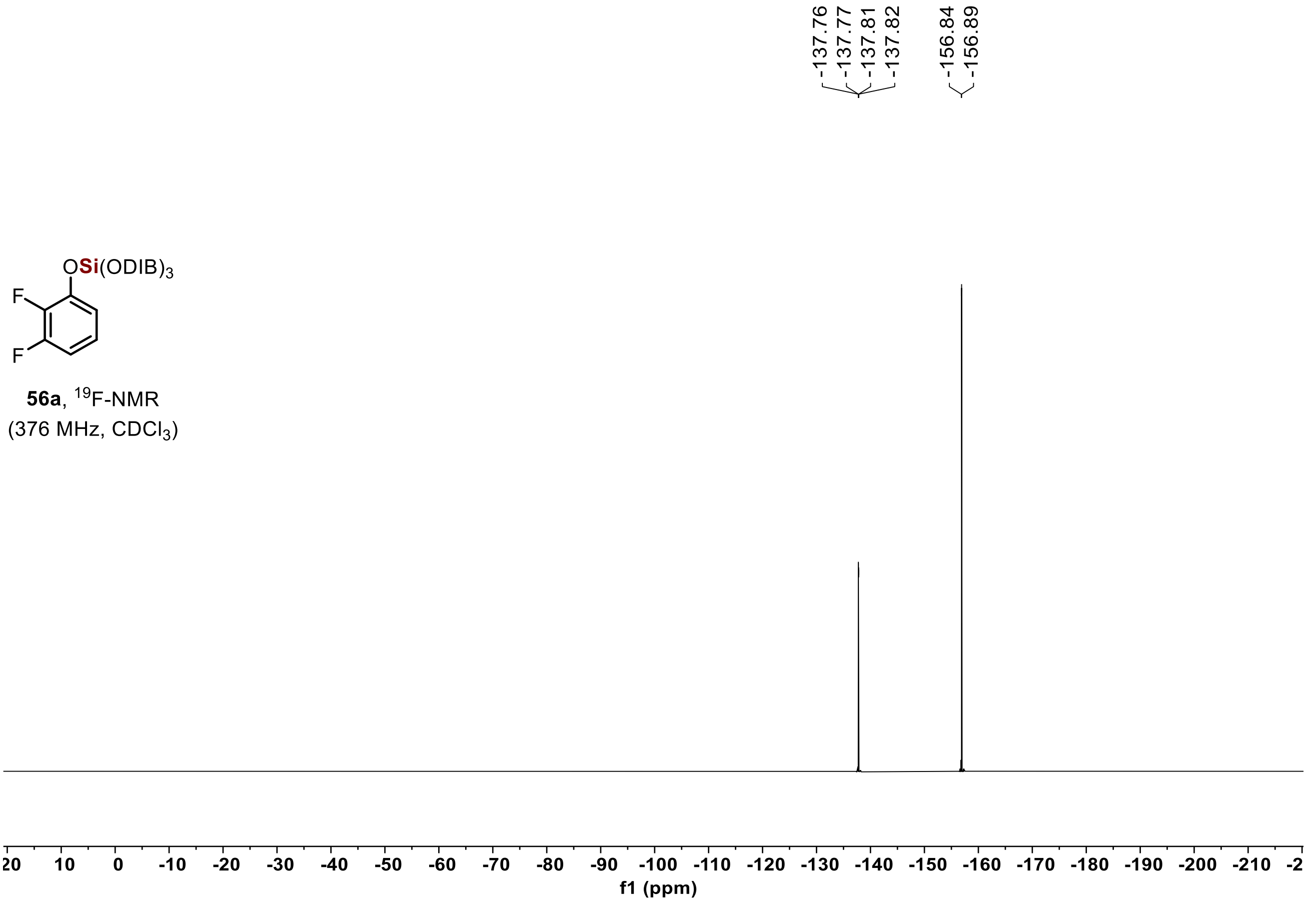
-137.76
-137.77
-137.81
-137.82
-156.84
-156.89
OSi(ODIB)3
F
F
56a, 19F-NMR
(376 MHz, CDCl3)
20 10 0 -10 -20 -30 -40 -50 -60 -70 -80 -90 -100 -110 -120 -130 -140 -150 -160 -170 -180 -190 -200 -210
f1 (ppm)

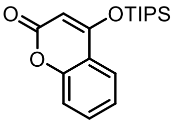

57a, ^{1}H-NMR
(400 MHz, $CDCl_3$)

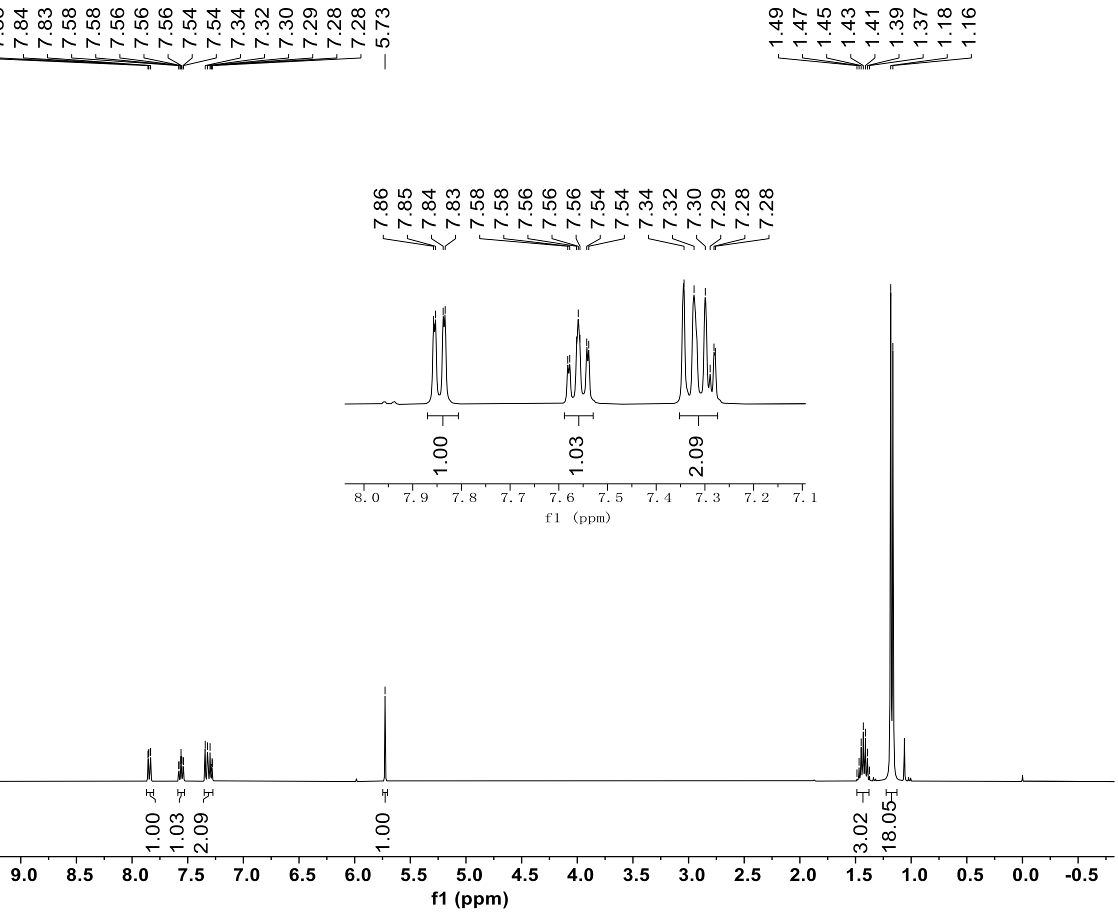

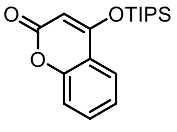

57a, ^{13}C-NMR
(101 MHz, $CDCl_3$)

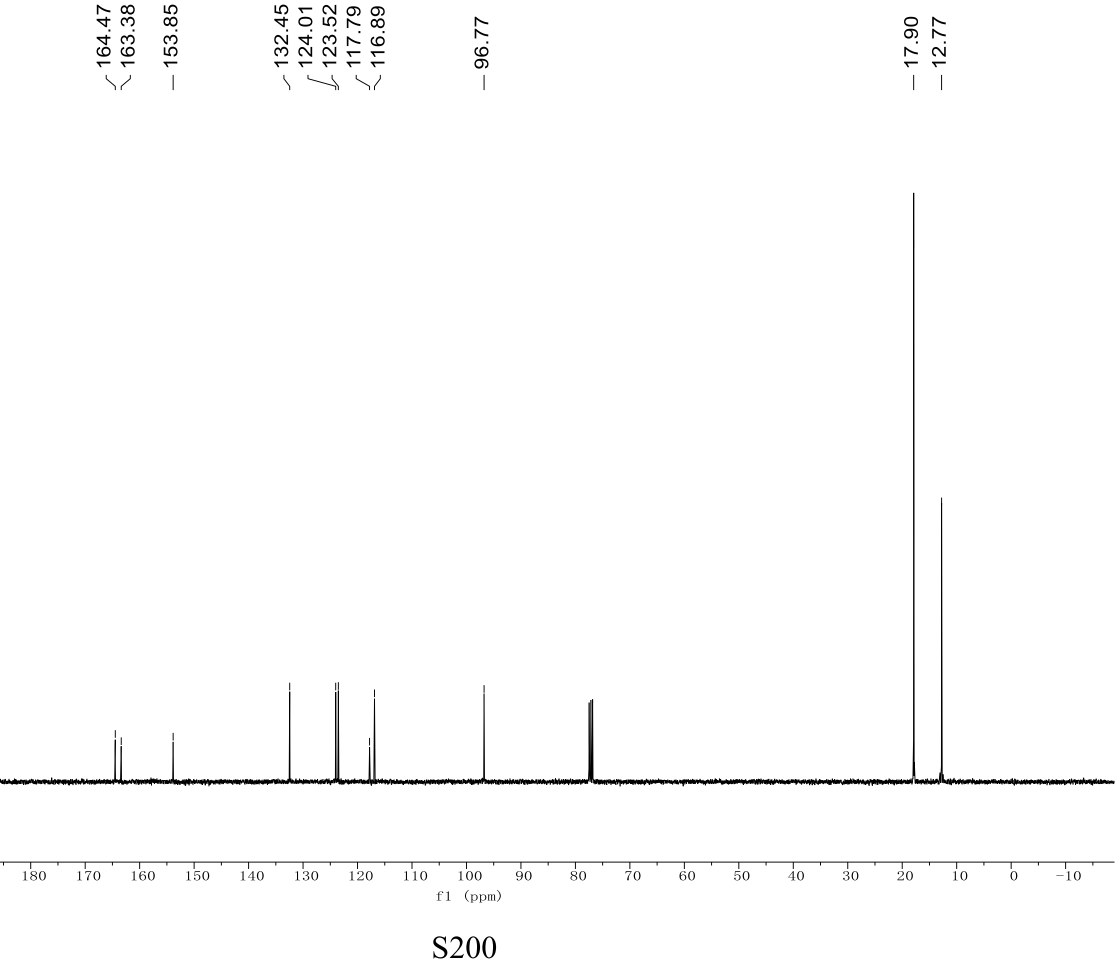

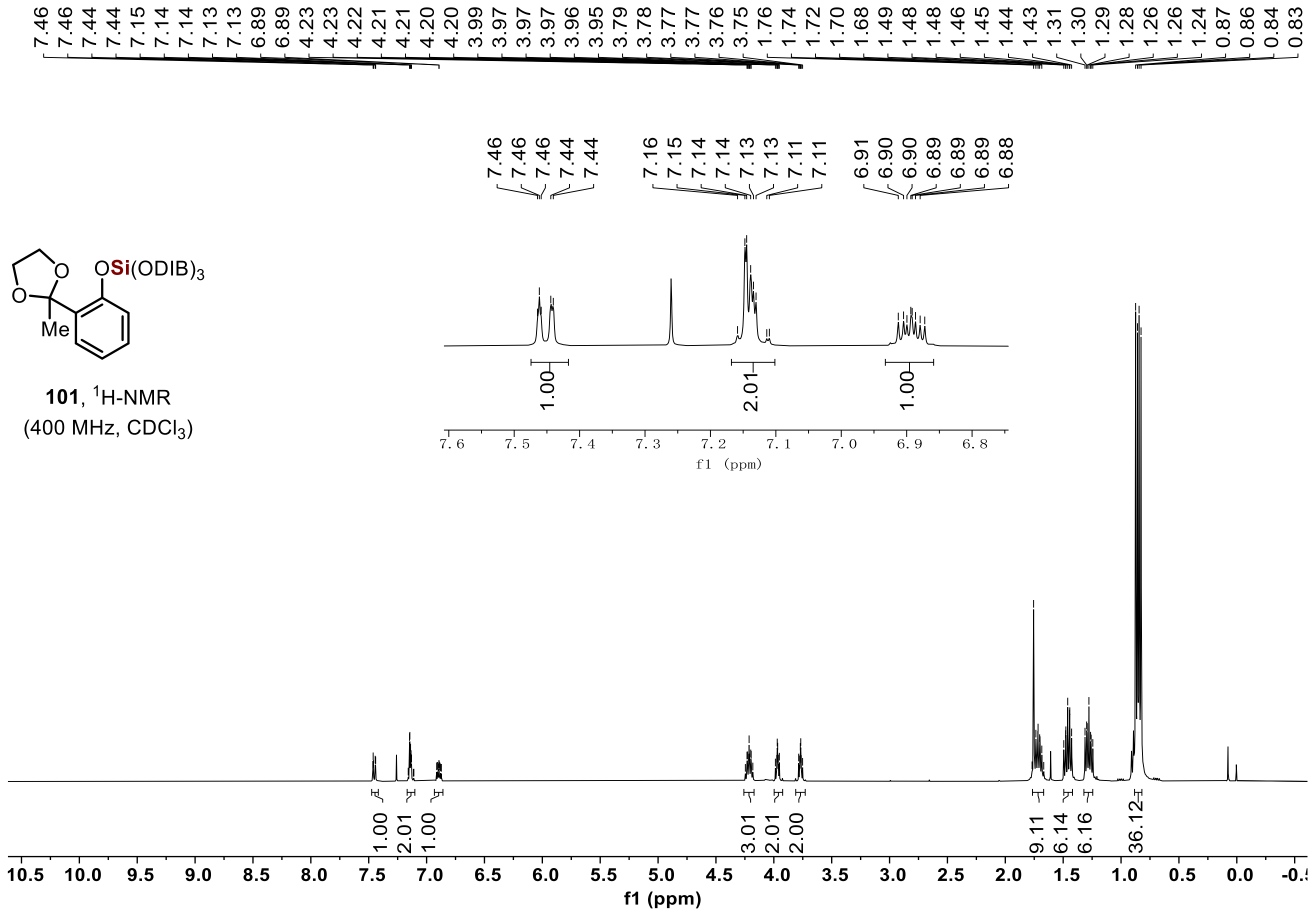
OSi(ODIB)3
Me
101, 1H-NMR
(400 MHz, CDCl3)
f1 (ppm)

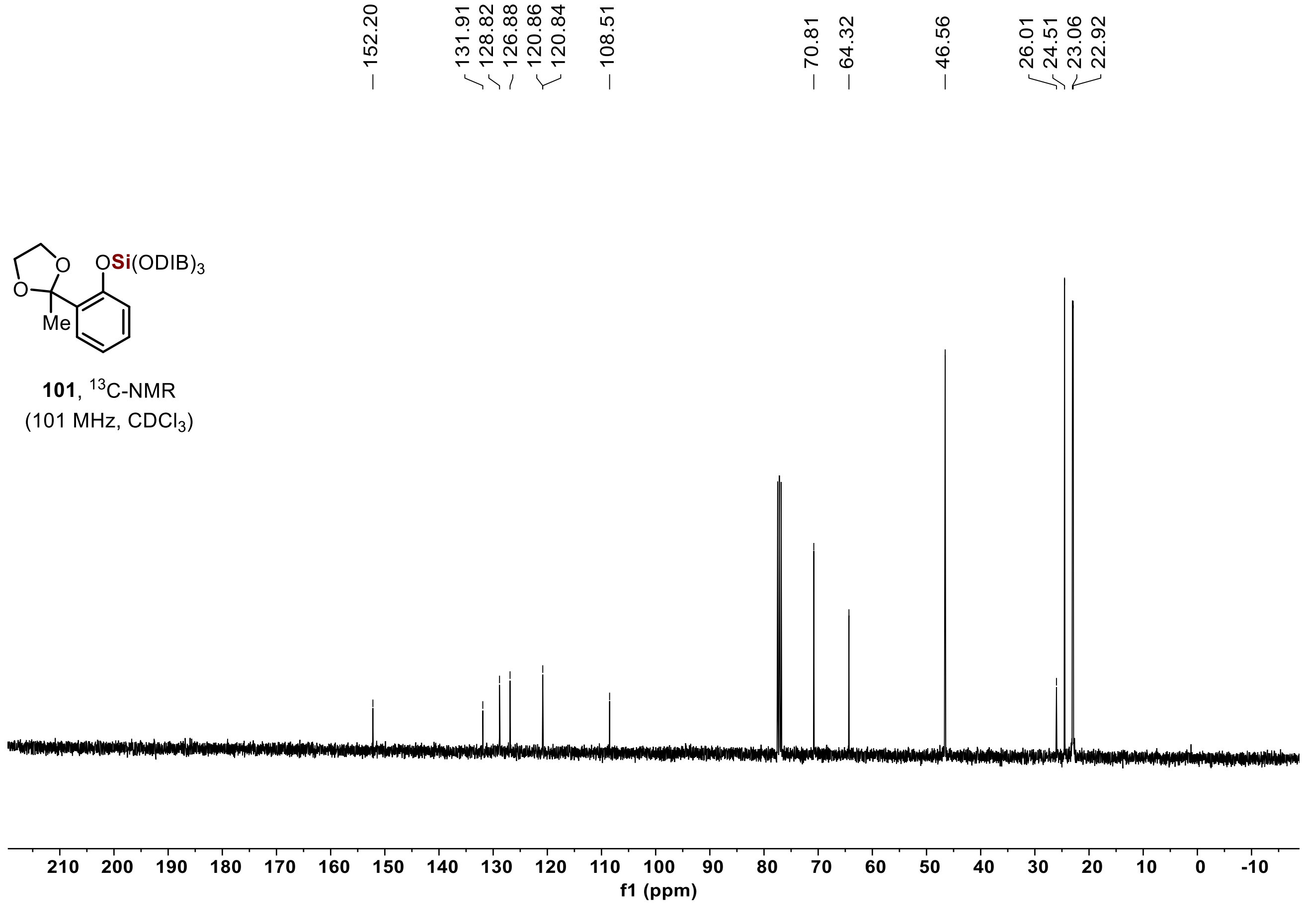
OSi(ODIB)3
Me
101, 13C-NMR
(101 MHz, CDCl3)
f1 (ppm)

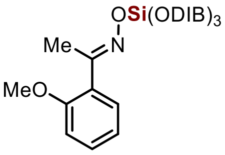

103, ^{1}H-NMR
(400 MHz, $CDCl_3$)

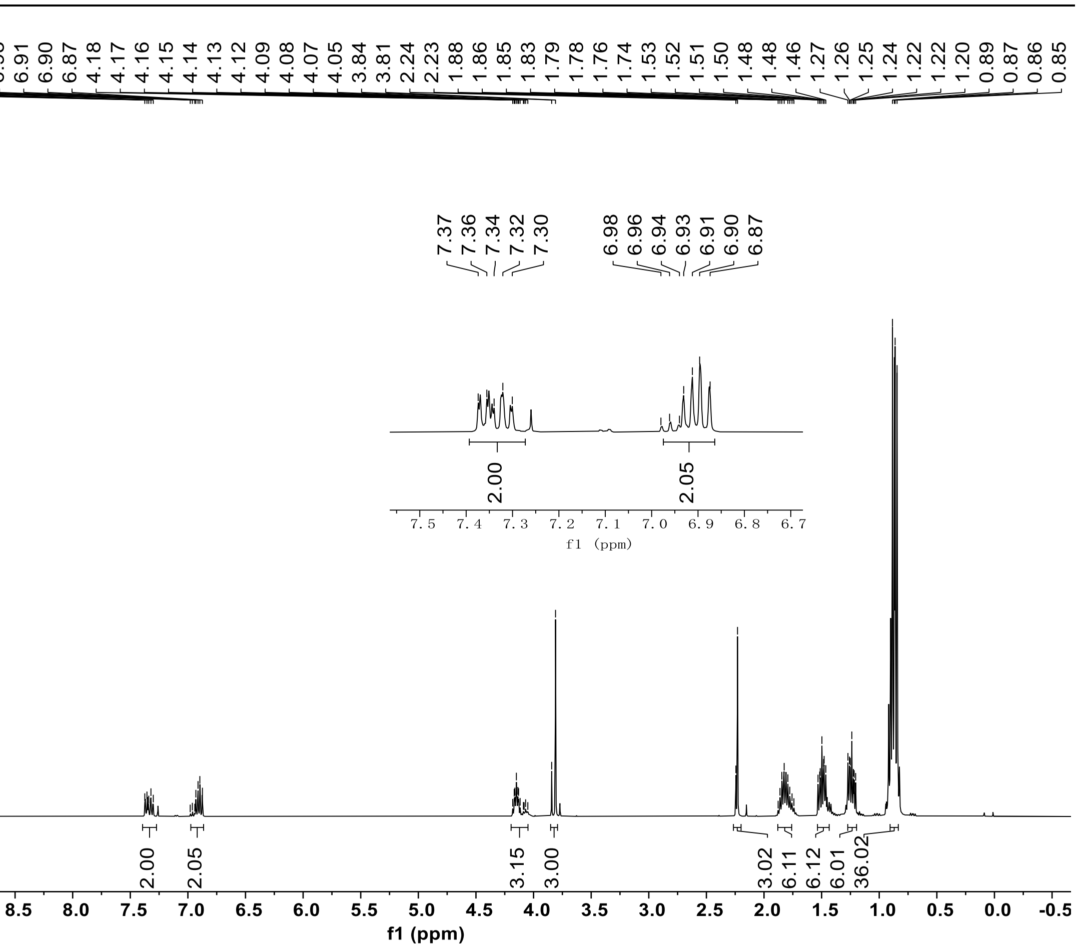

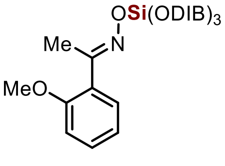

103, ^{13}C-NMR
(101 MHz, $CDCl_3$)

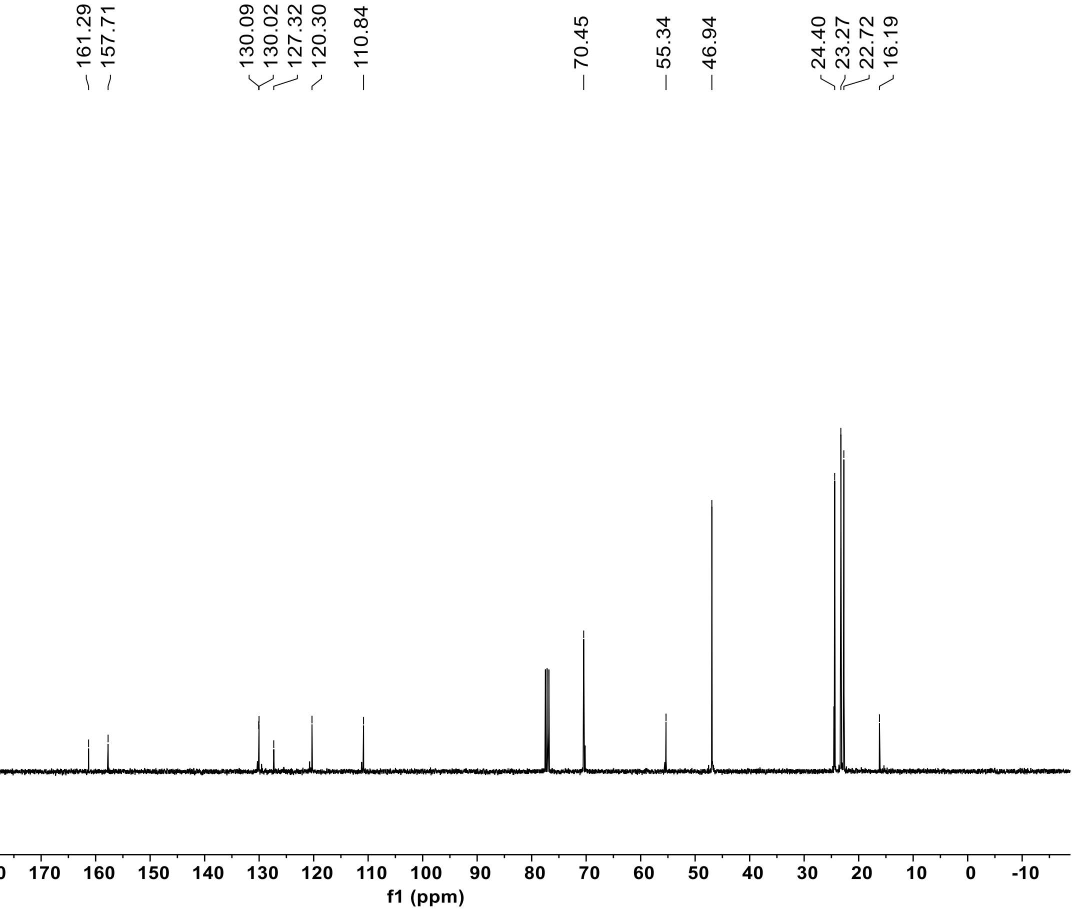

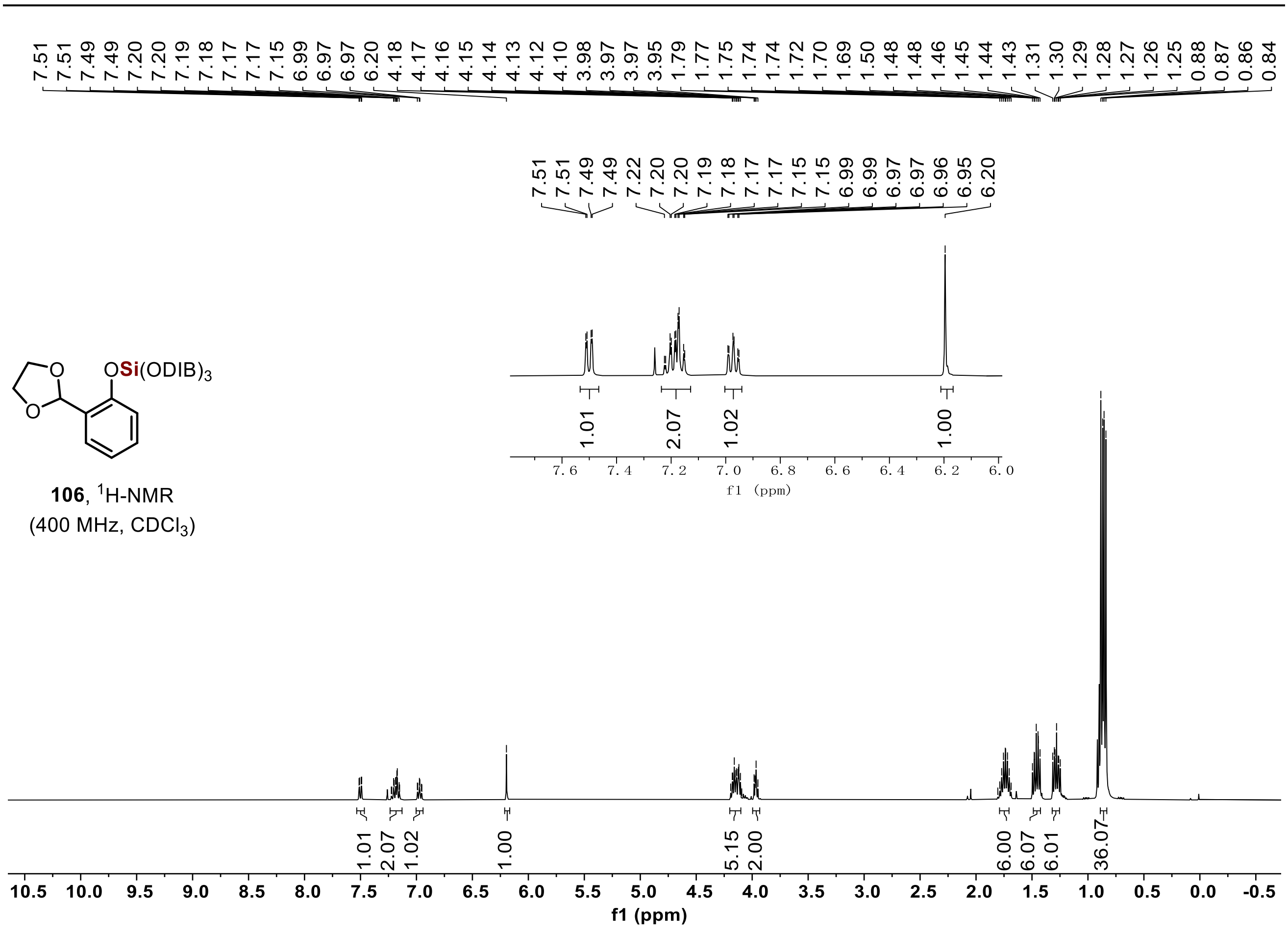

106, ^{1}H-NMR
(400 MHz, $CDCl_3$)

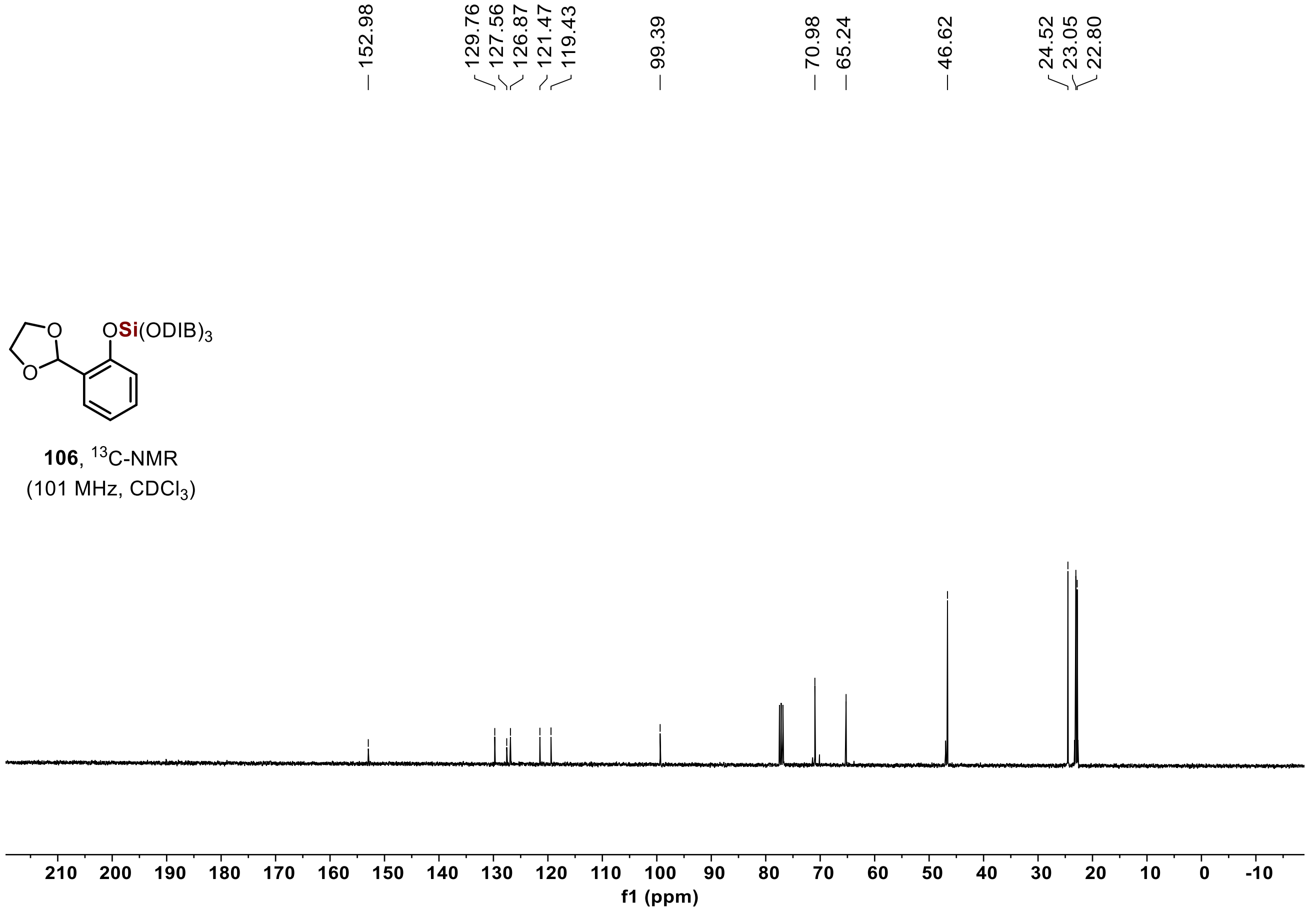

106, ^{13}C-NMR
(101 MHz, $CDCl_3$)

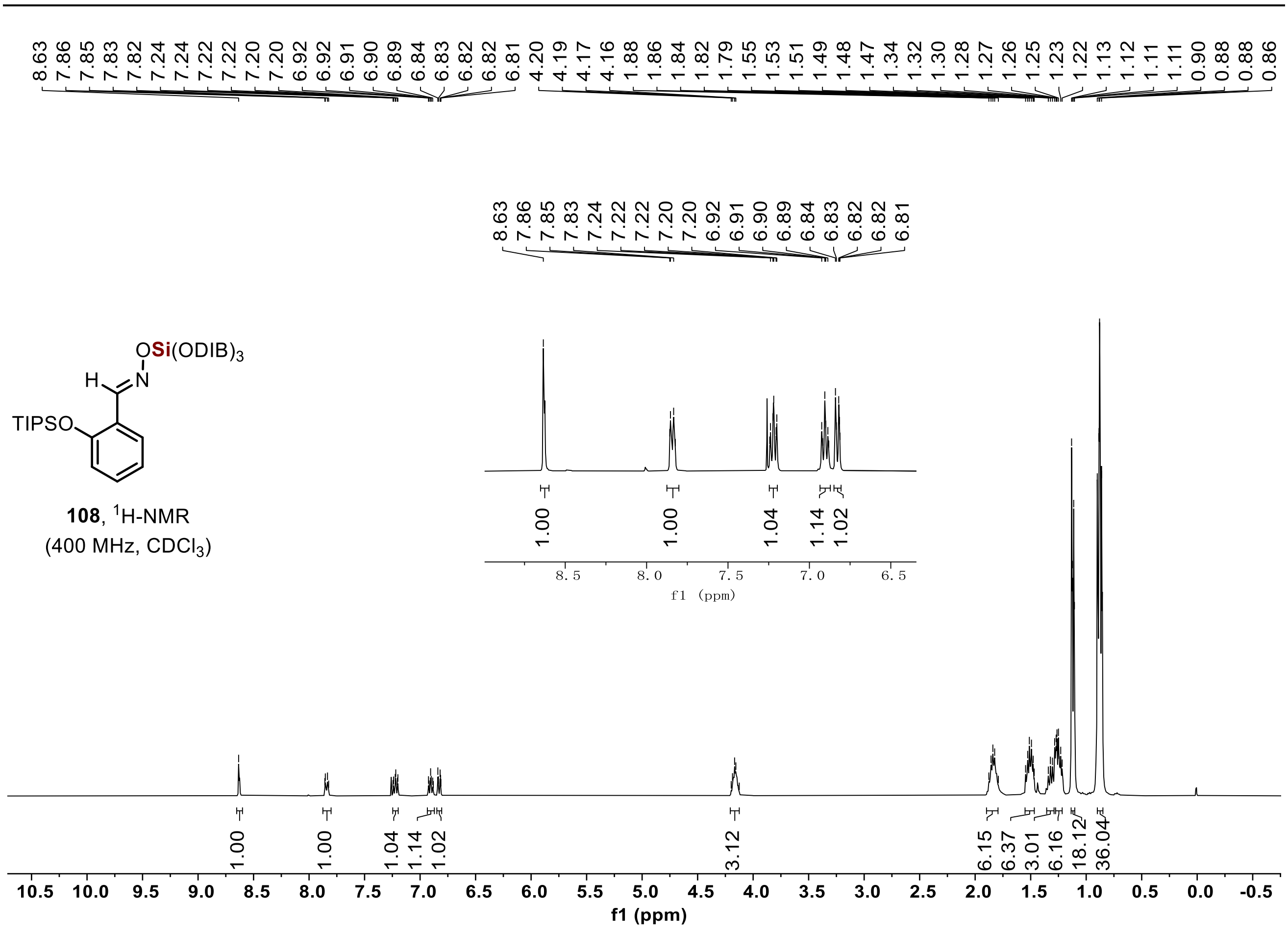

108, ^{1}H-NMR
(400 MHz, $CDCl_3$)

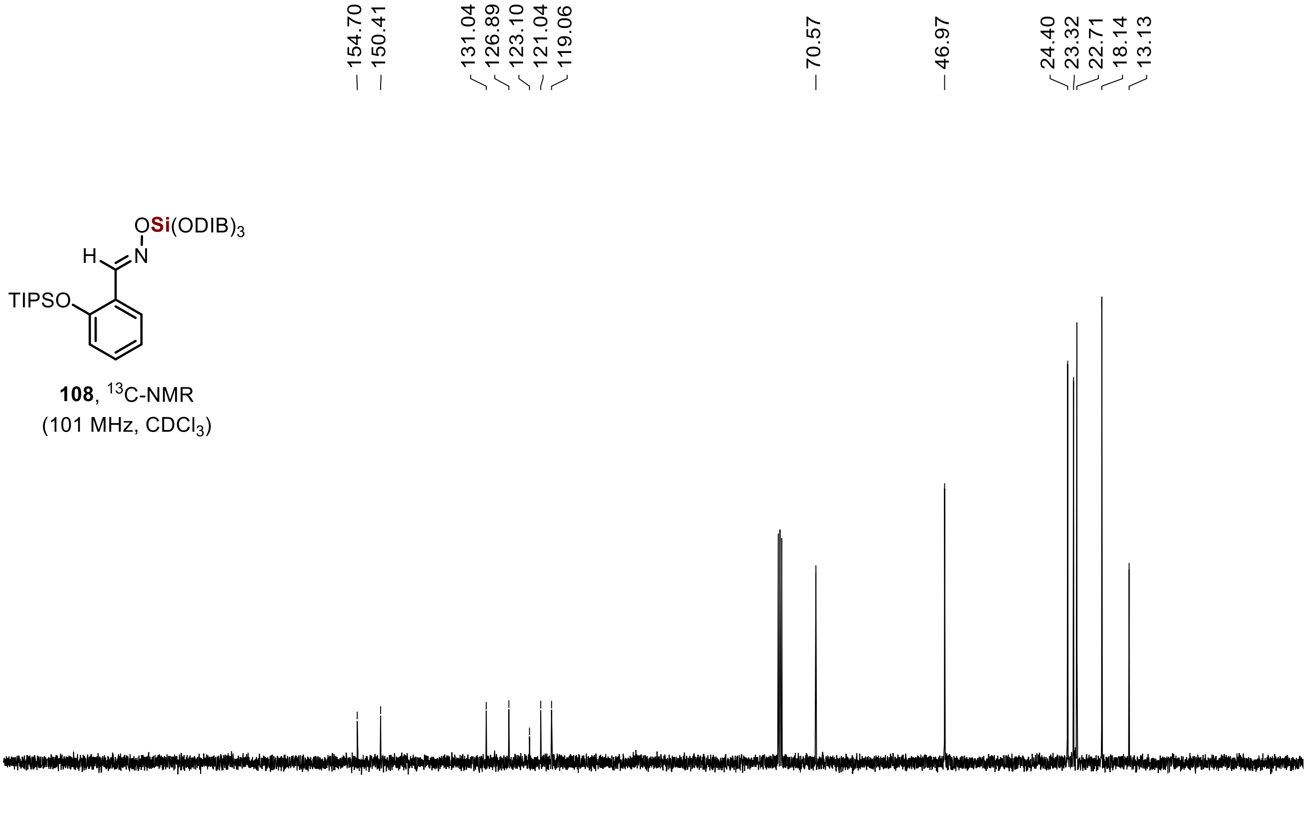

108, ^{13}C-NMR
(101 MHz, $CDCl_3$)

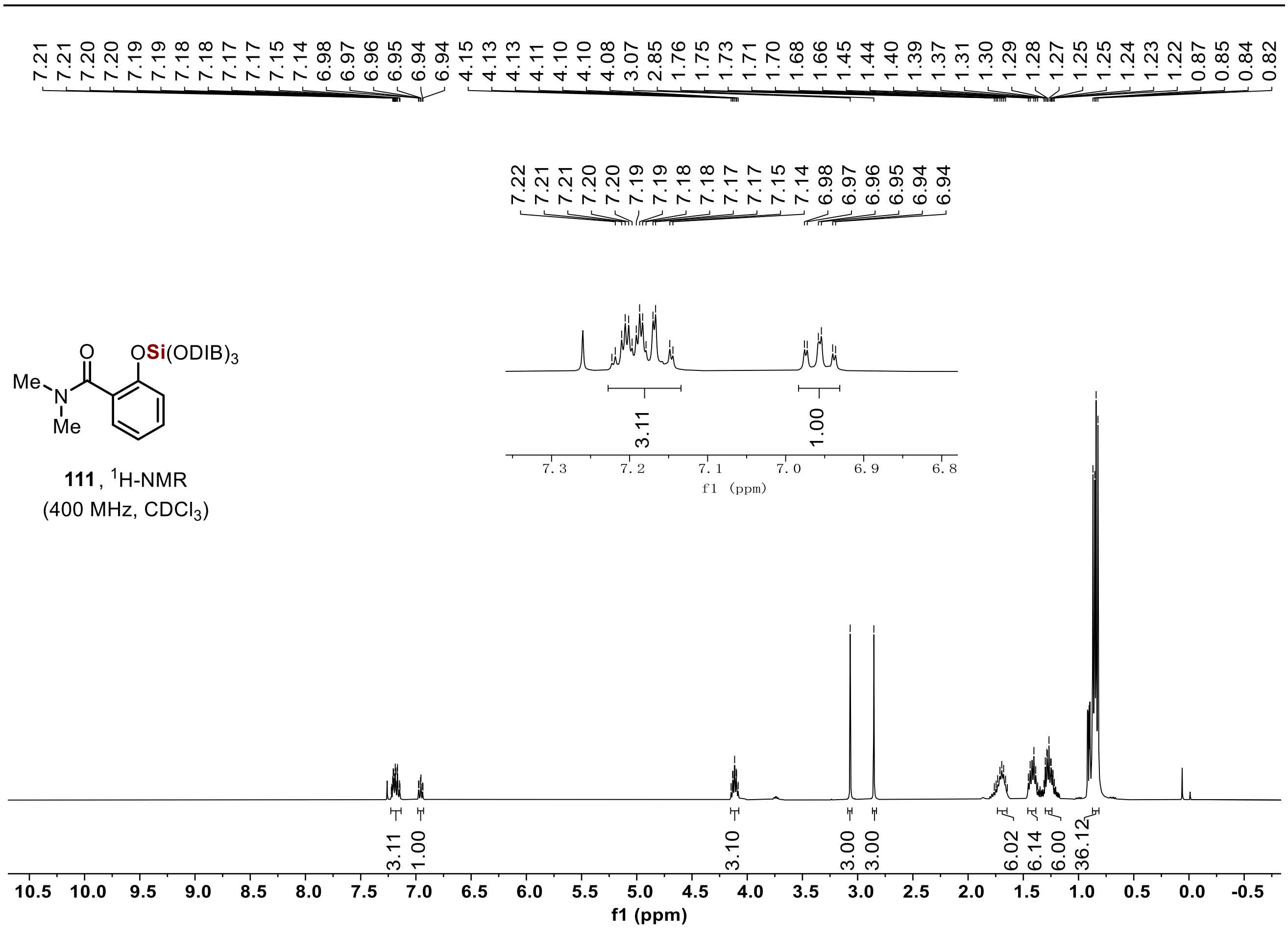

111, ^{1}H-NMR
(400 MHz, $CDCl_3$)

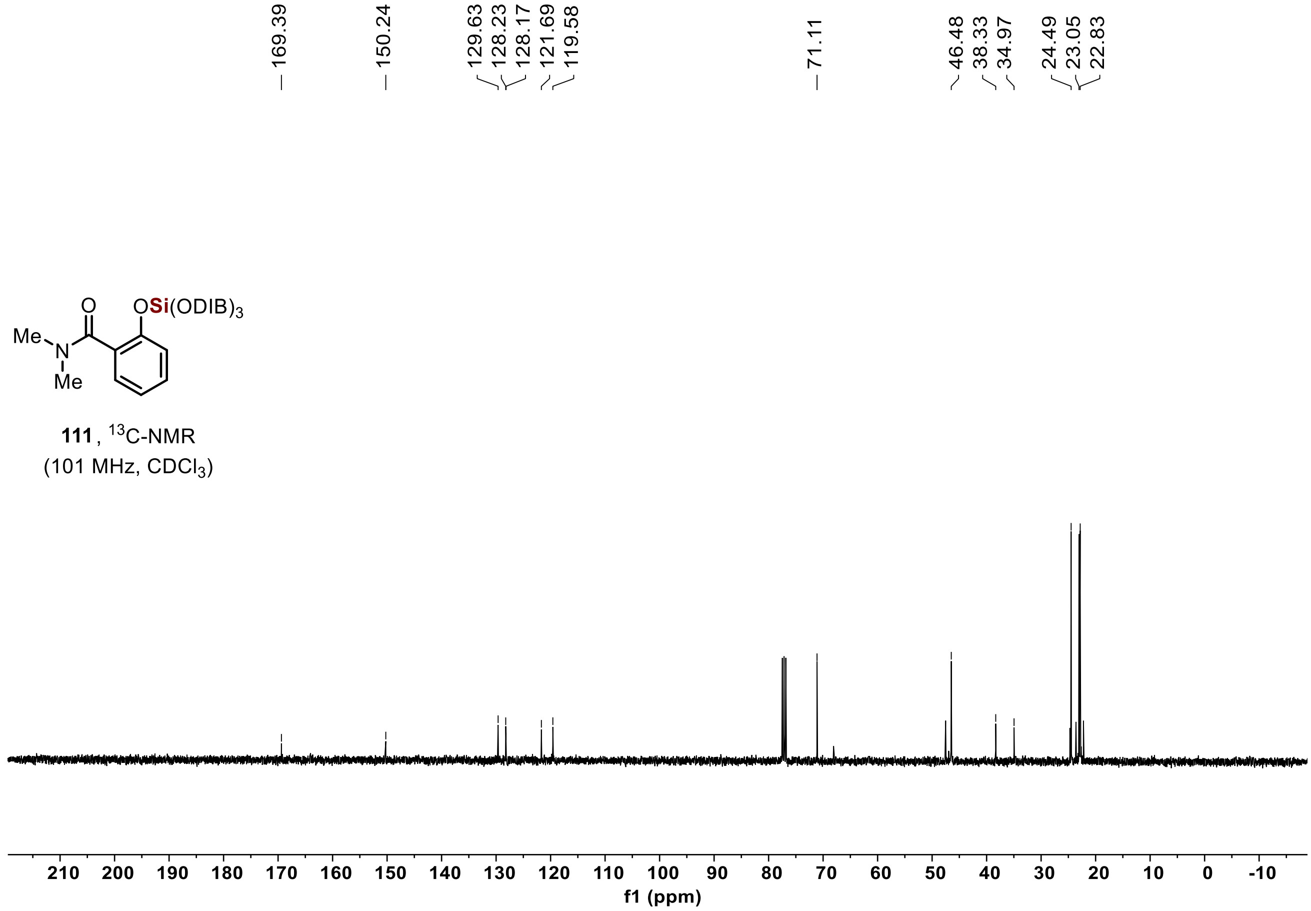

111, ^{13}C-NMR
(101 MHz, $CDCl_3$)

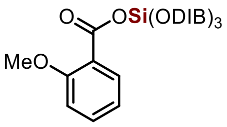

113, ^{1}H-NMR
(400 MHz, $CDCl_3$)

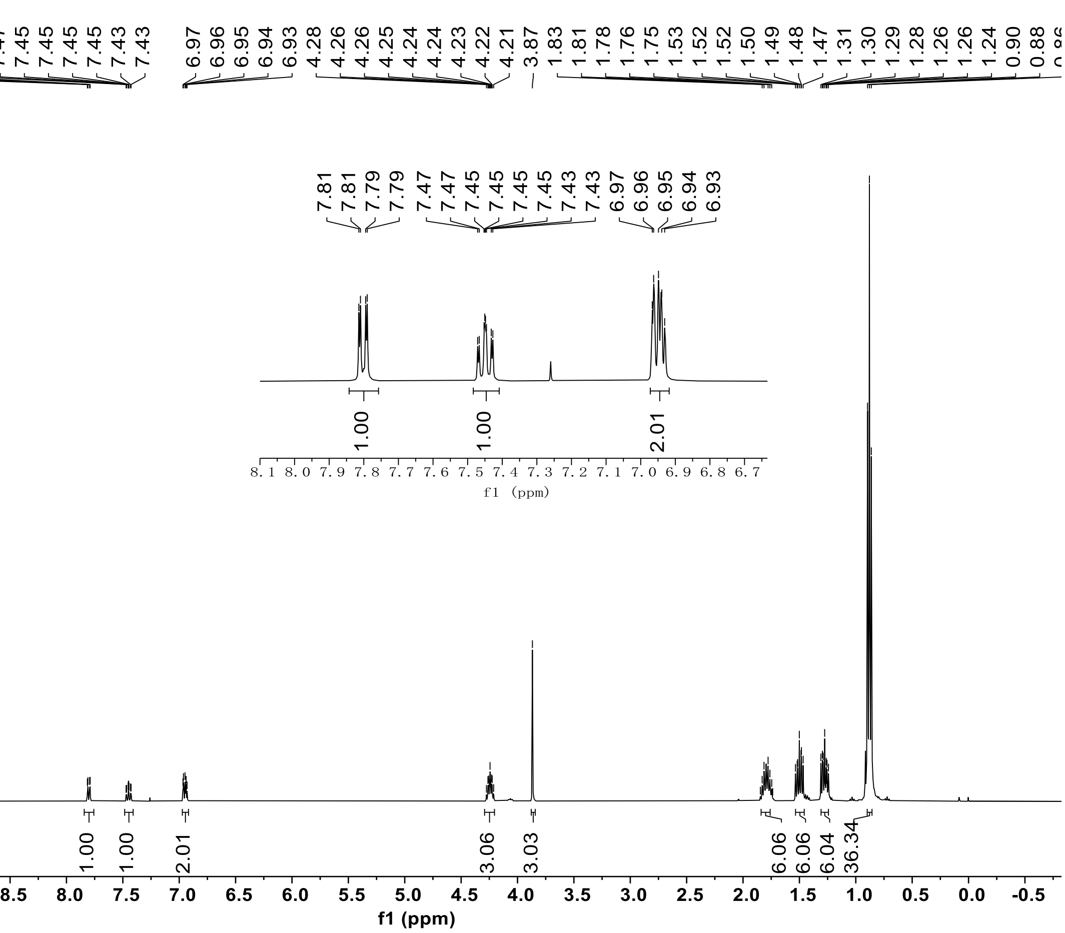

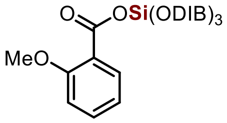

113, ^{13}C-NMR
(101 MHz, $CDCl_3$)

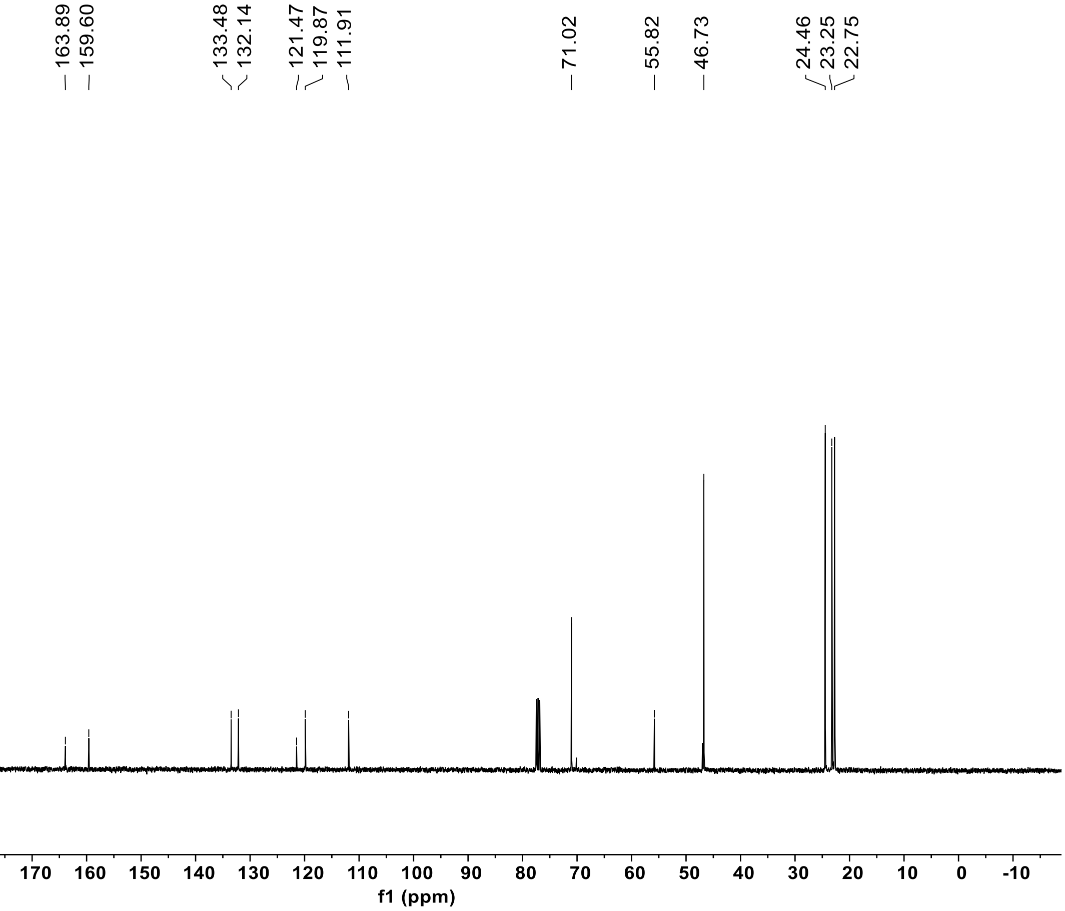

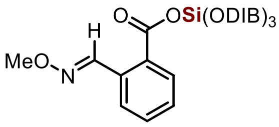

116, ^{1}H-NMR
(400 MHz, $CDCl_3$)

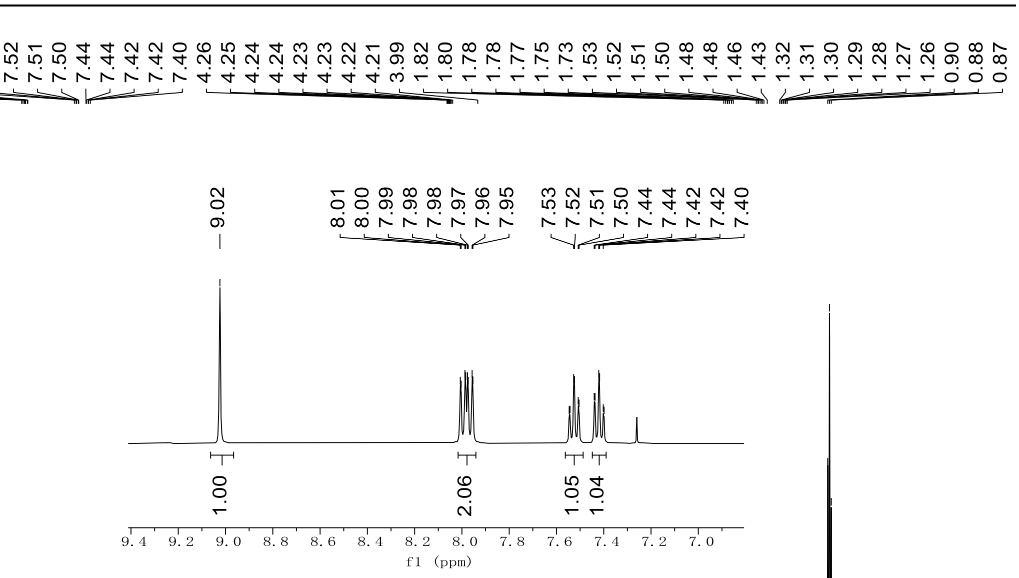

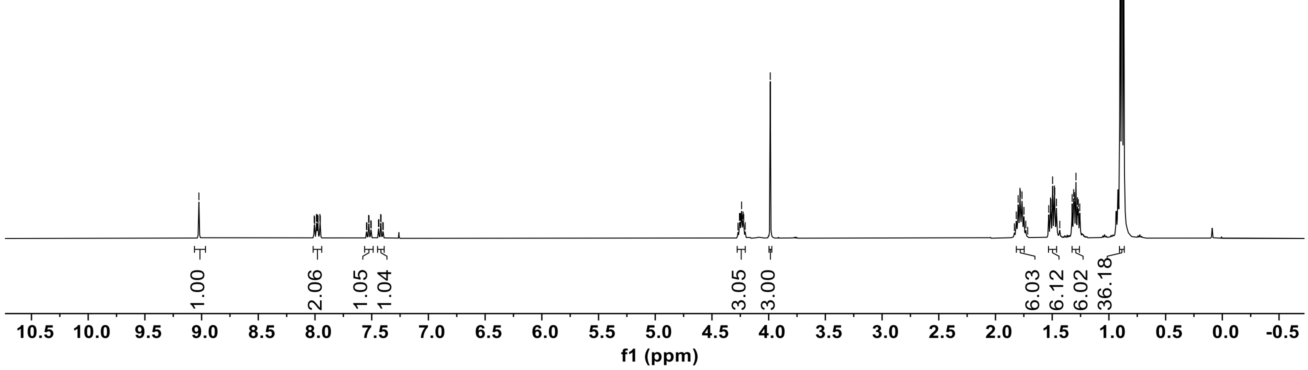

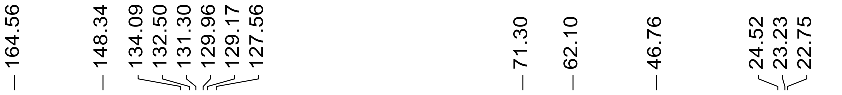

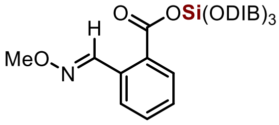

116, ^{13}C-NMR
(101 MHz, $CDCl_3$)

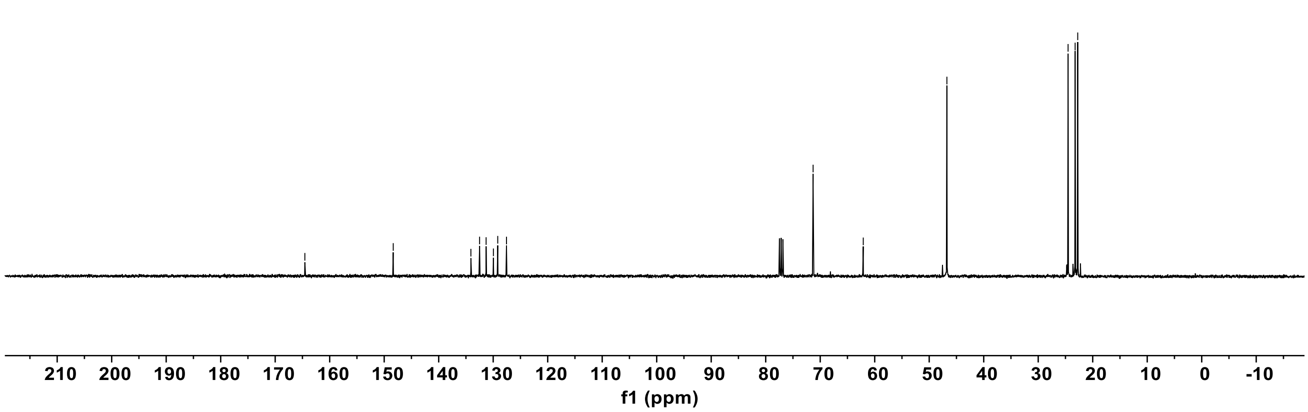

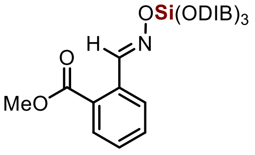

118, ^{1}H-NMR
(400 MHz, $CDCl_3$)

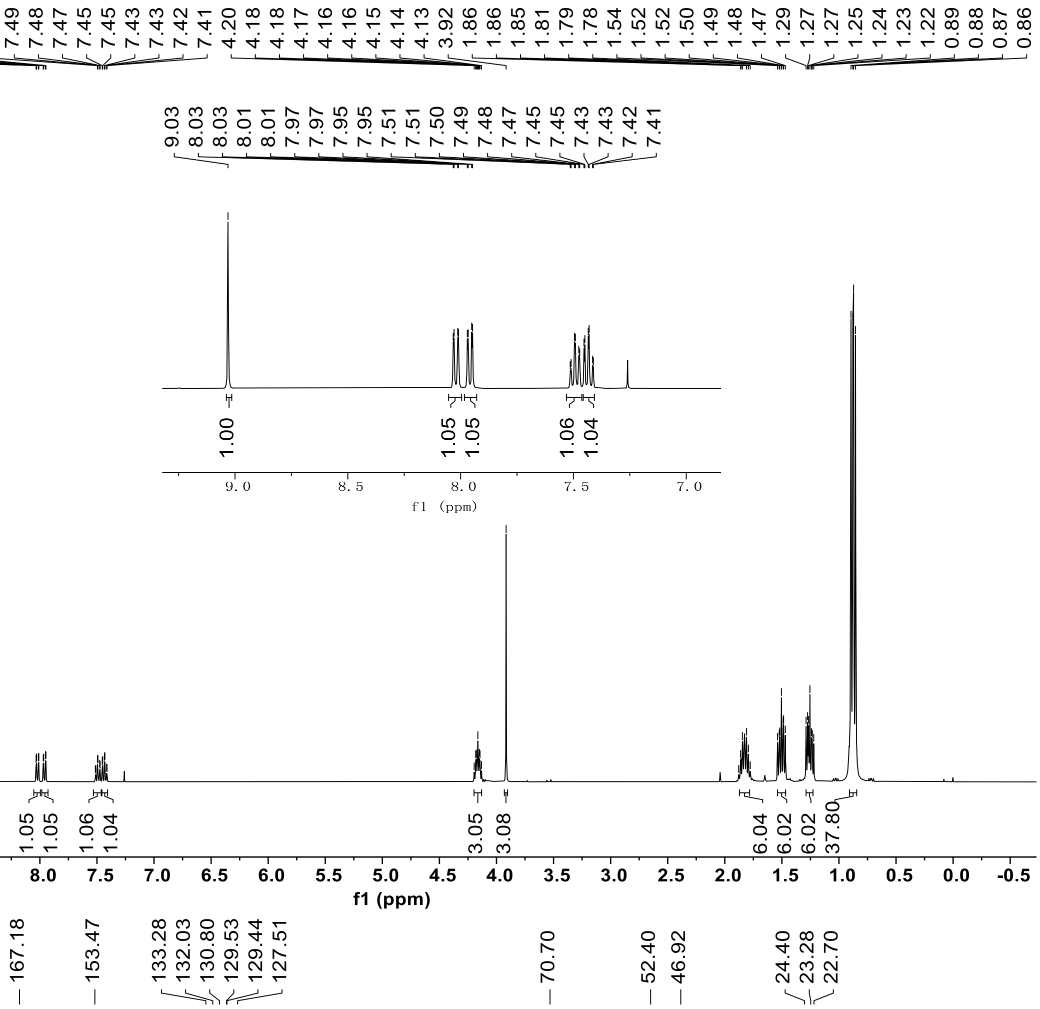

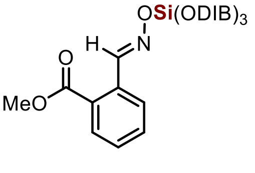

118, ^{13}C-NMR
(101 MHz, $CDCl_3$)

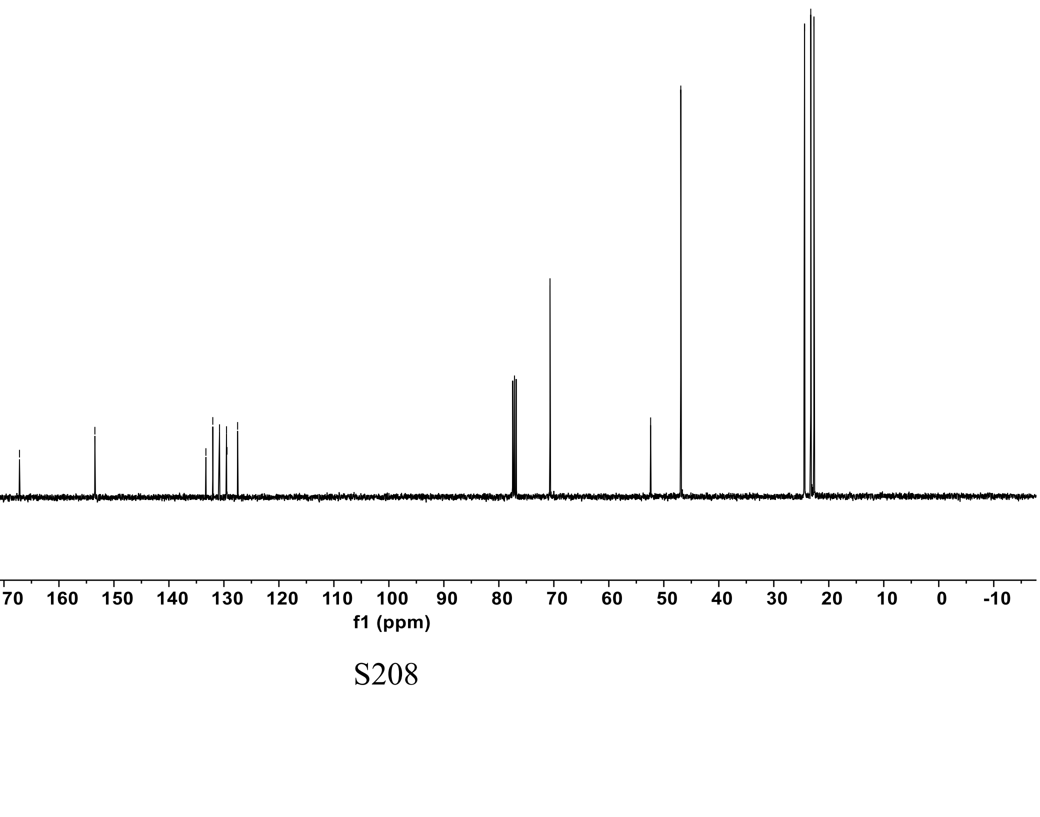

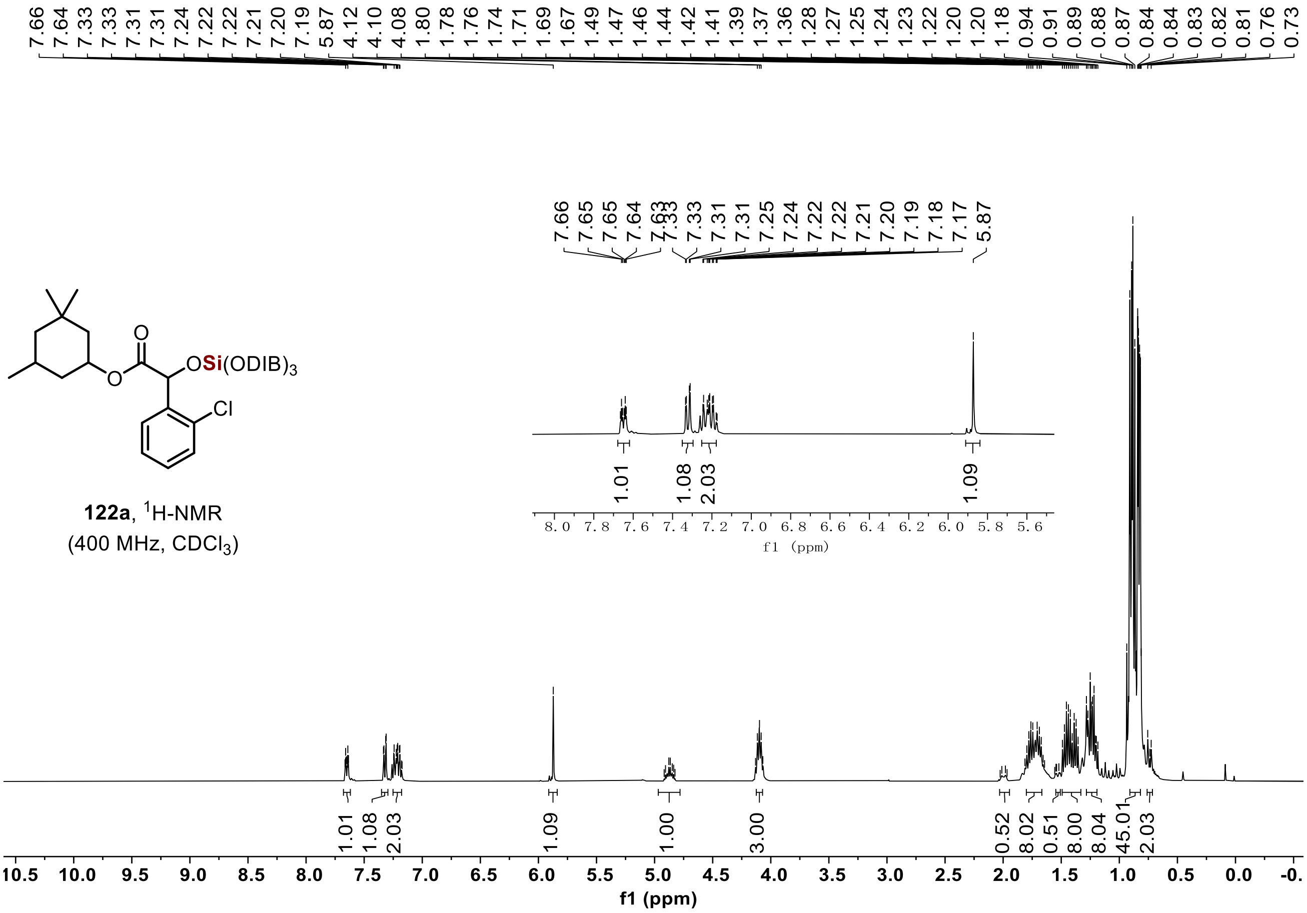

122a, ^{1}H-NMR
(400 MHz, $CDCl_3$)

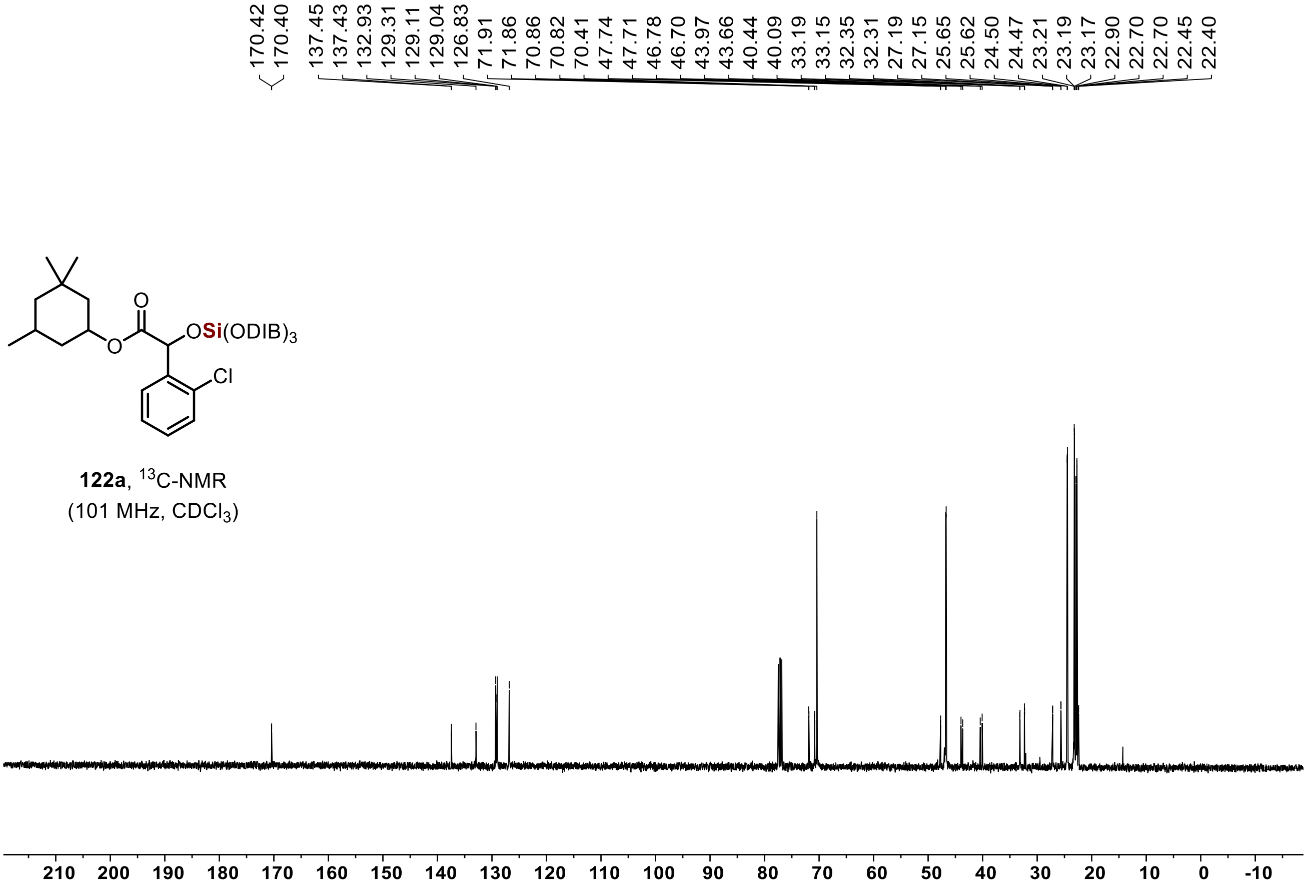

122a, ^{13}C-NMR
(101 MHz, $CDCl_3$)

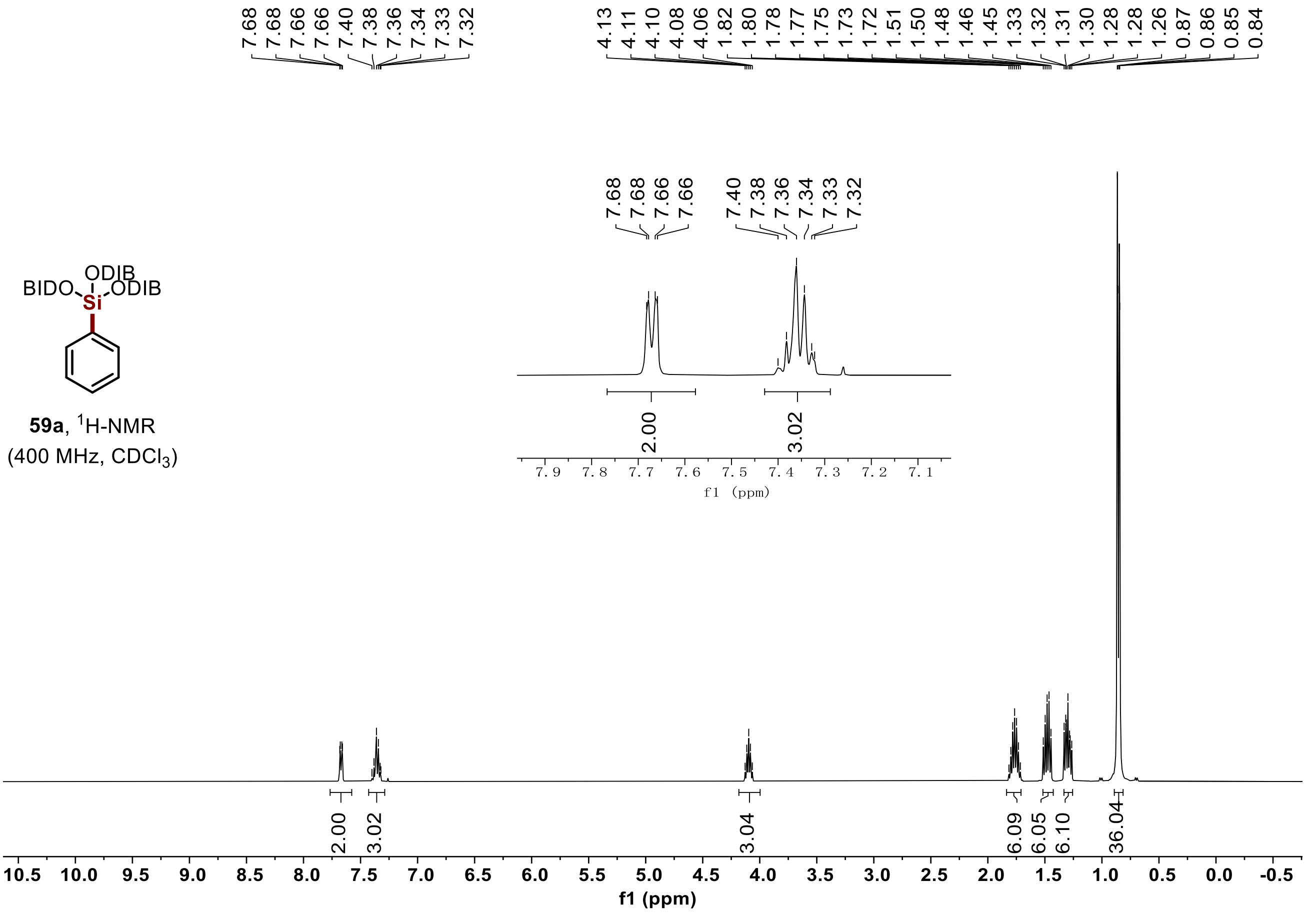
ODIB
BIDO
ODIB
Si
59a, ¹H-NMR
(400 MHz, CDCl₃)
f1 (ppm)

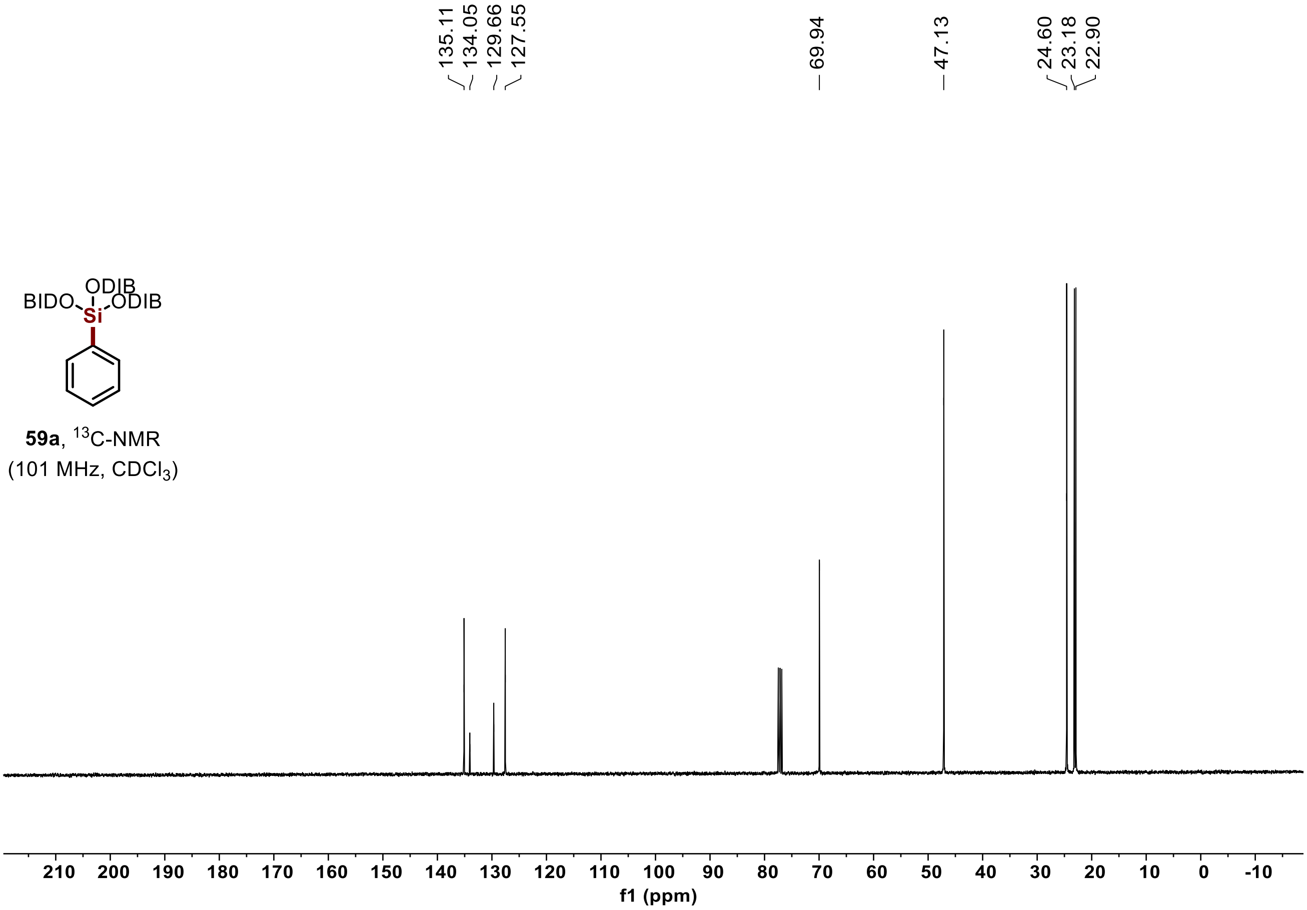
ODIB
BIDO
ODIB
Si
59a, ¹³C-NMR
(101 MHz, CDCl₃)
f1 (ppm)

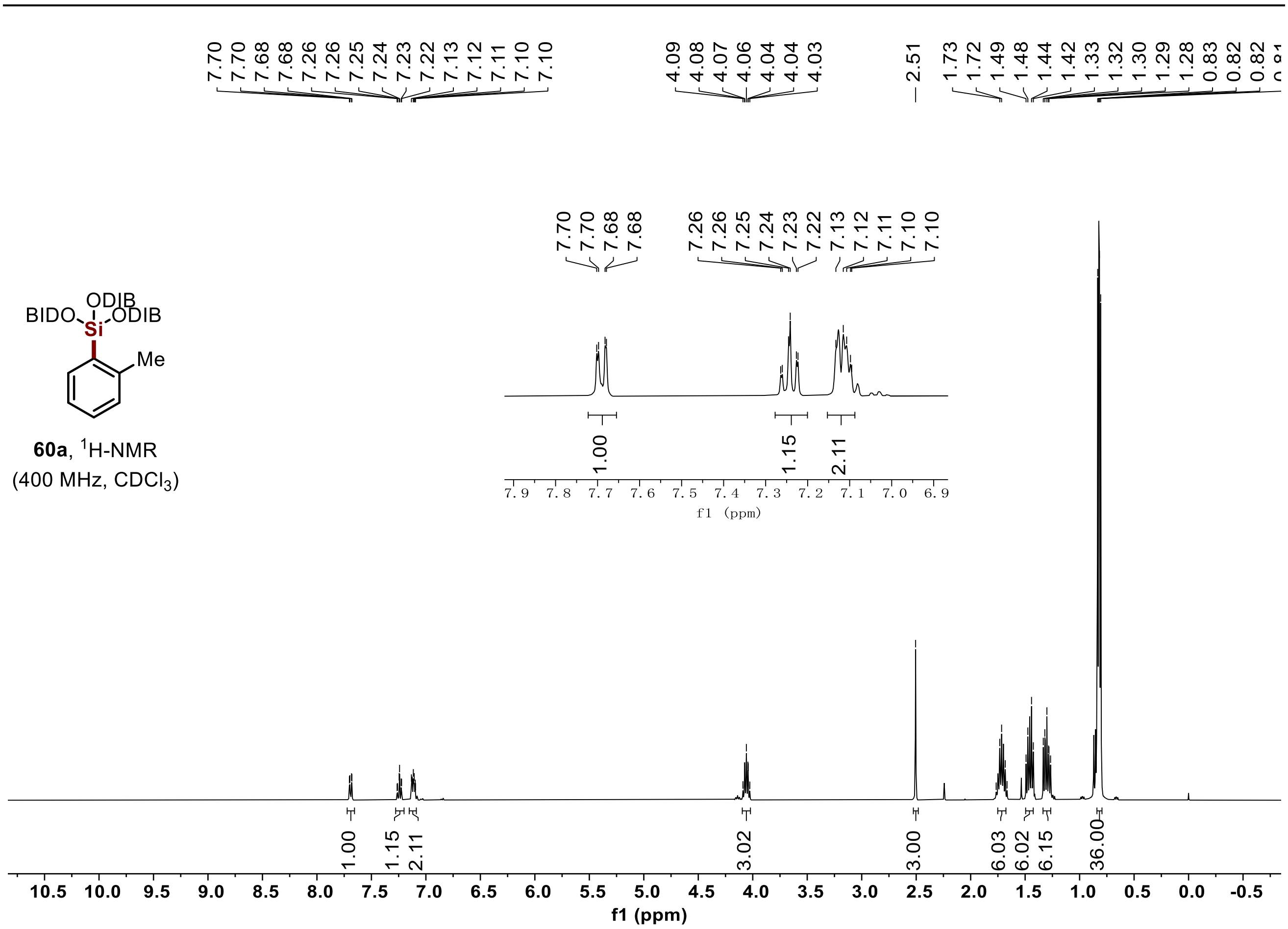
7.70 7.70 7.68 7.68 7.26 7.26 7.25 7.24 7.23 7.22 7.13 7.12 7.11 7.10 7.10
4.09 4.08 4.07 4.06 4.04 4.04 4.03
2.51
1.73 1.72 1.49 1.48 1.44 1.42 1.33 1.32 1.30 1.29 1.28 0.83 0.82 0.82
BIDO ODIB ODIB Si Me
60a, 1H-NMR
(400 MHz, CDCl3)
1.00 1.15 2.11
f1 (ppm)
1.00 1.15 2.11 3.02 3.00 6.03 6.02 6.15 36.00
f1 (ppm)

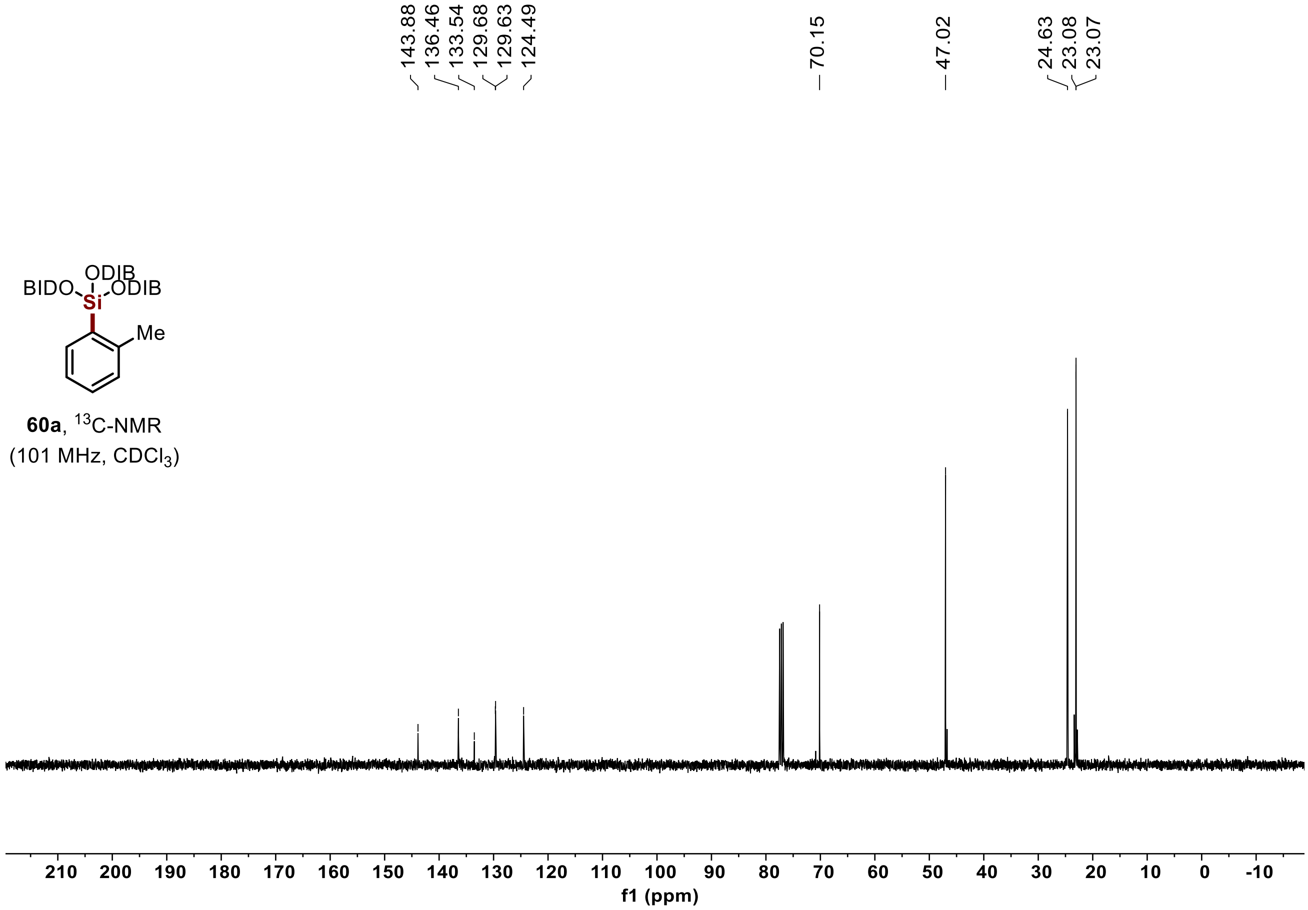
143.88 136.46 133.54 129.68 129.63 124.49
70.15
47.02
24.63 23.08 23.07
BIDO ODIB ODIB Si Me
60a, 13C-NMR
(101 MHz, CDCl3)
f1 (ppm)

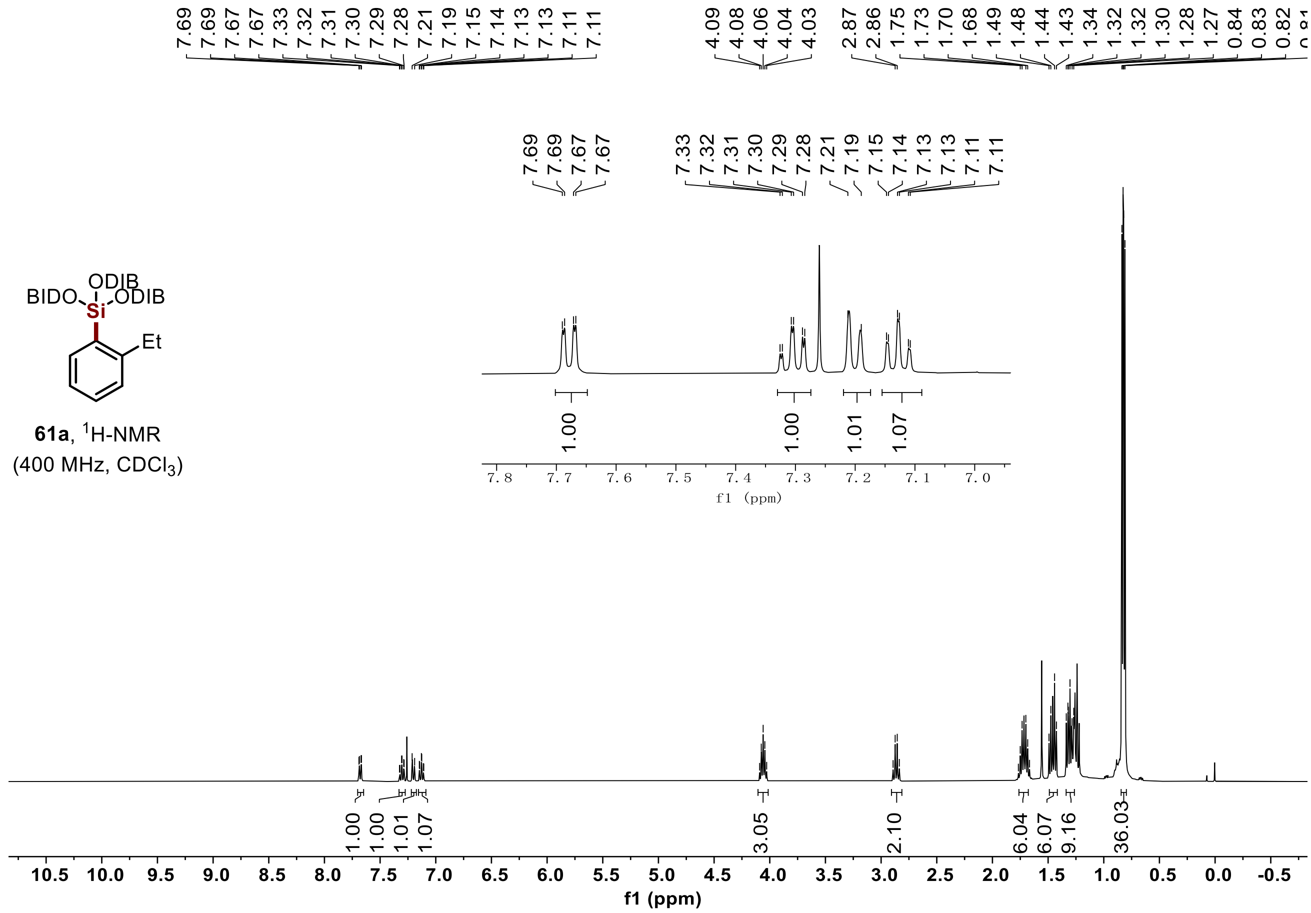

61a, ^{1}H-NMR
(400 MHz, $CDCl_3$)

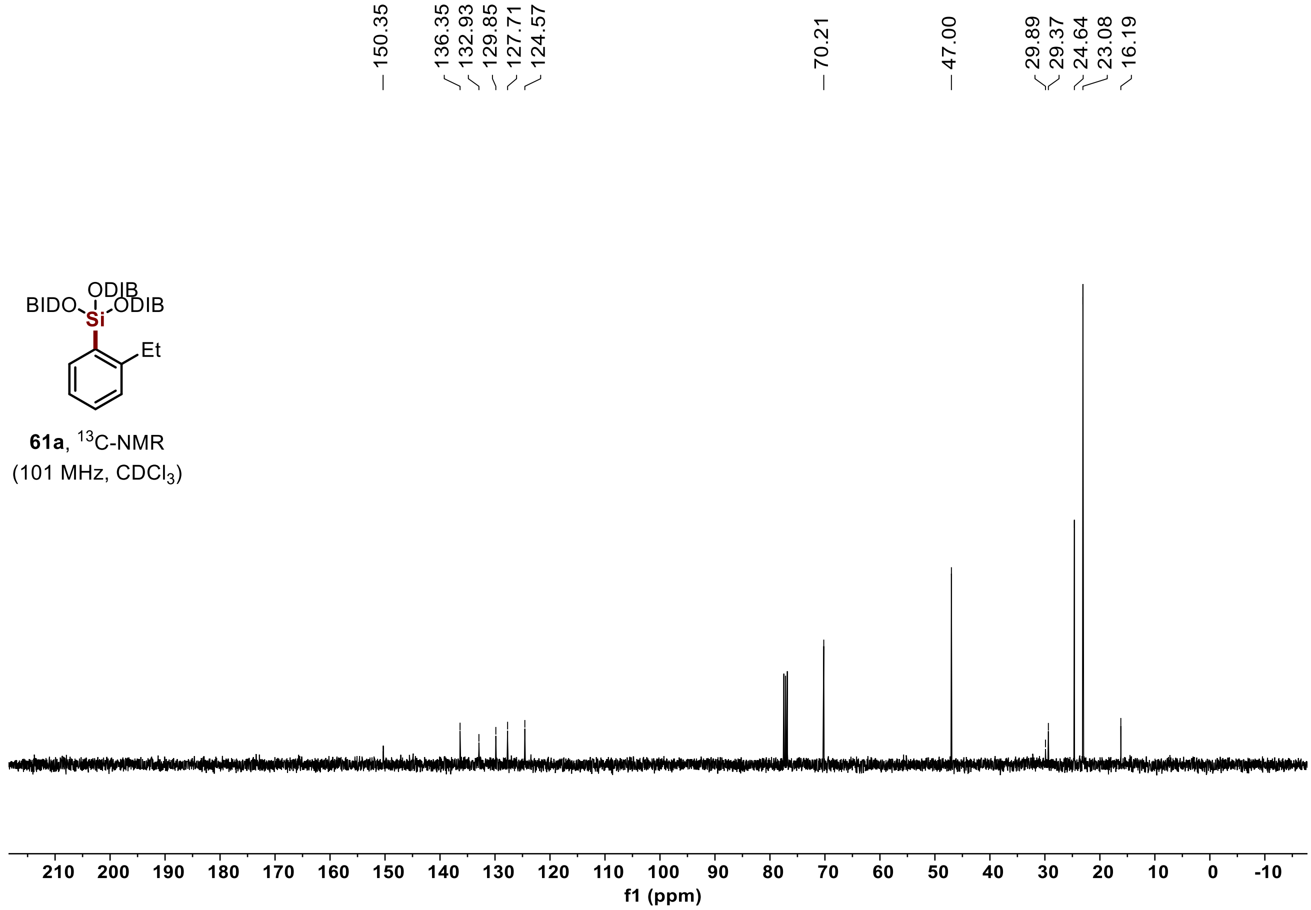

61a, ^{13}C-NMR
(101 MHz, $CDCl_3$)

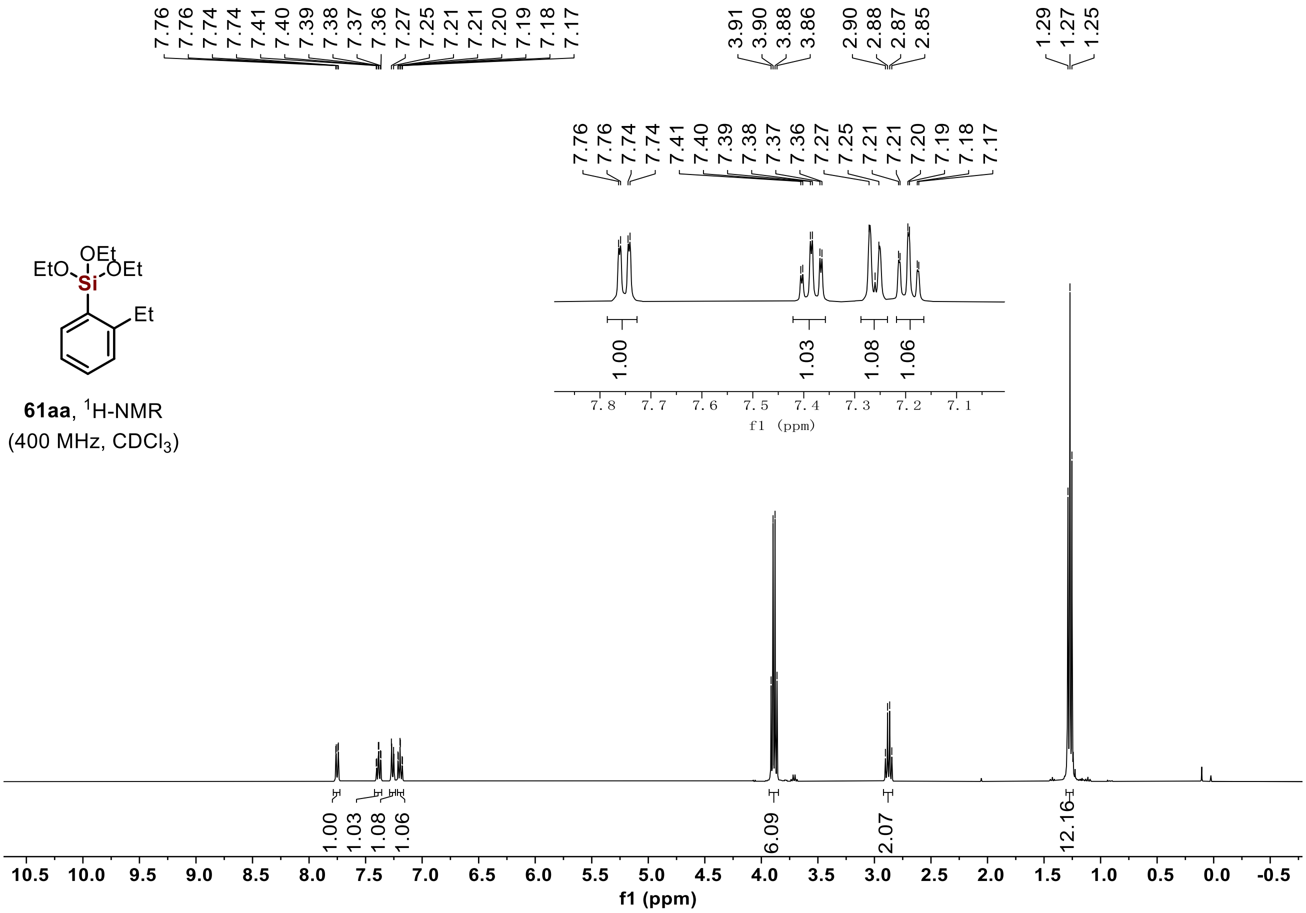

61aa, ^{1}H-NMR
(400 MHz, $CDCl_3$)

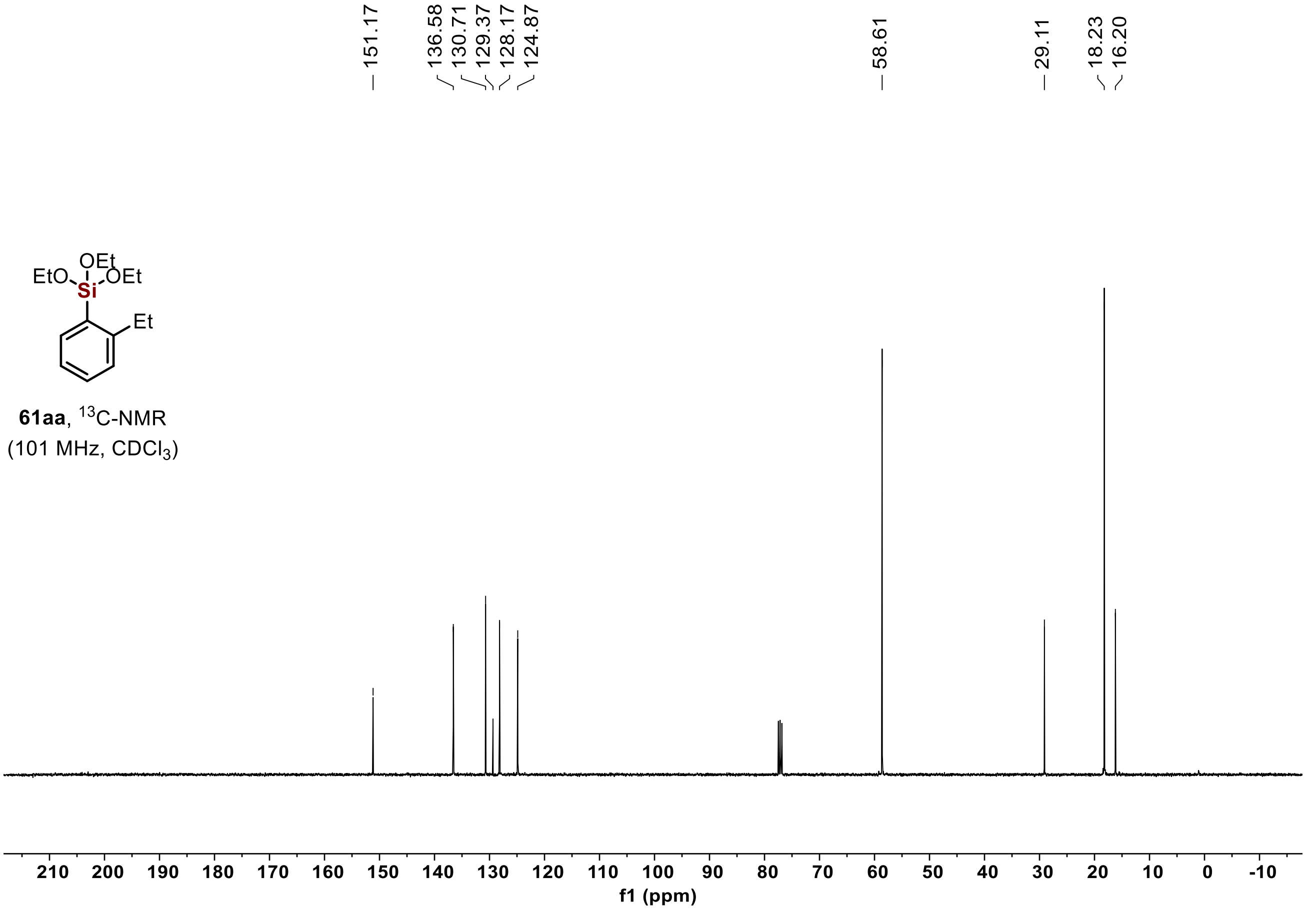

61aa, ^{13}C-NMR
(101 MHz, $CDCl_3$)

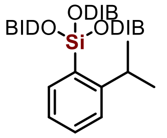

62a, ^{1}H-NMR
(400 MHz, $CDCl_3$)

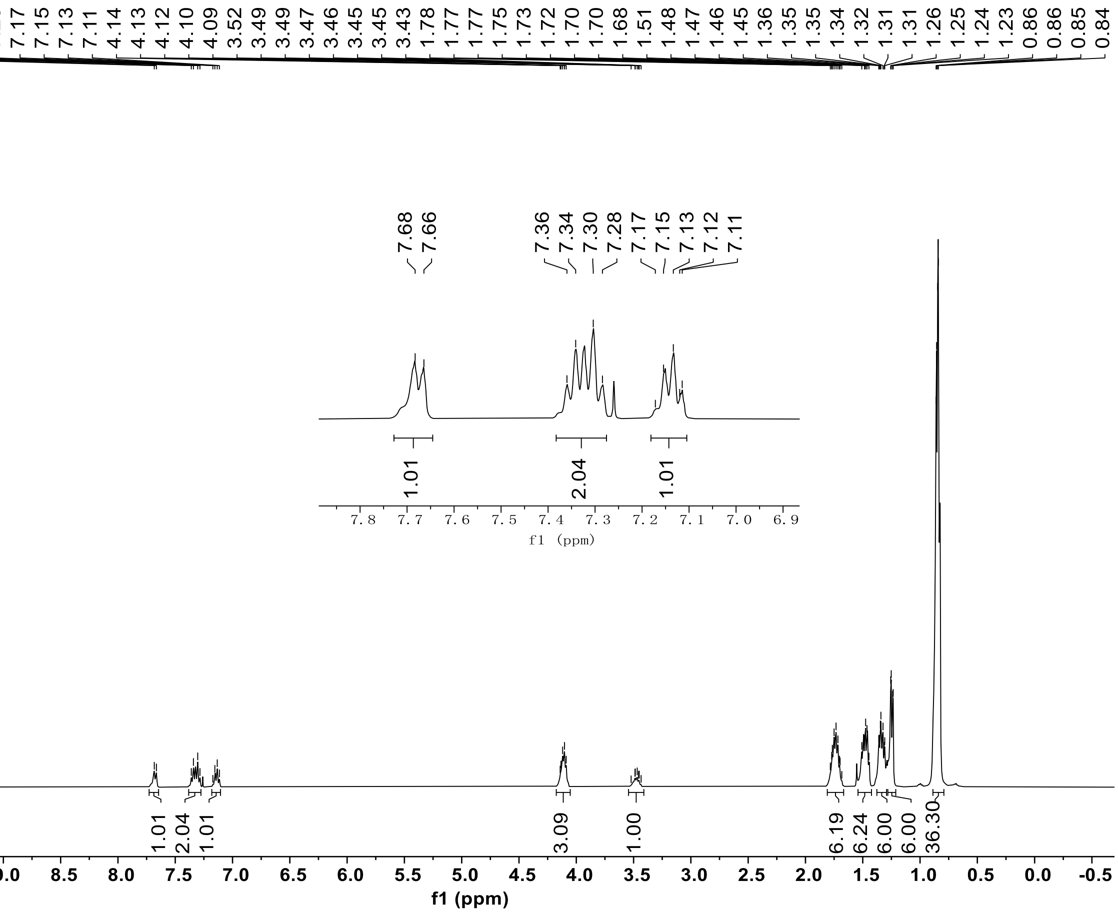

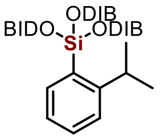

62a, ^{13}C-NMR
(101 MHz, $CDCl_3$)

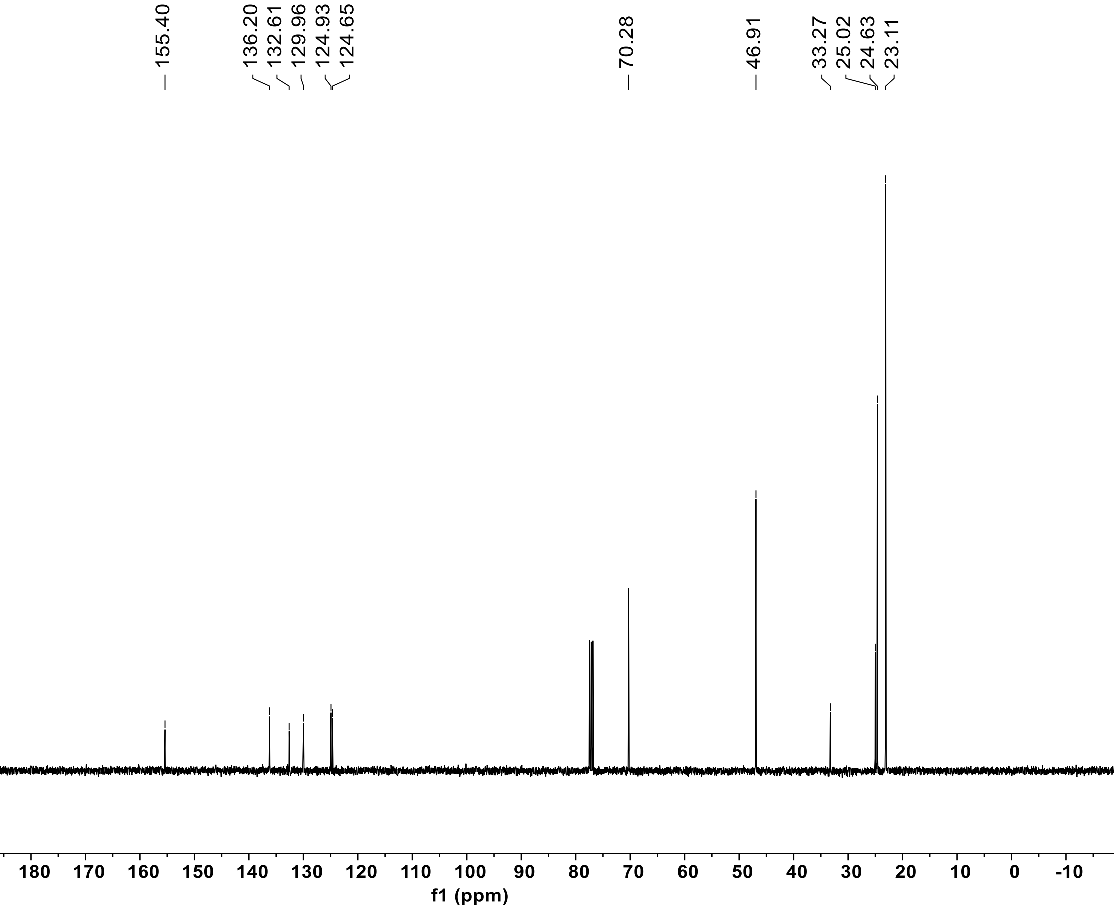

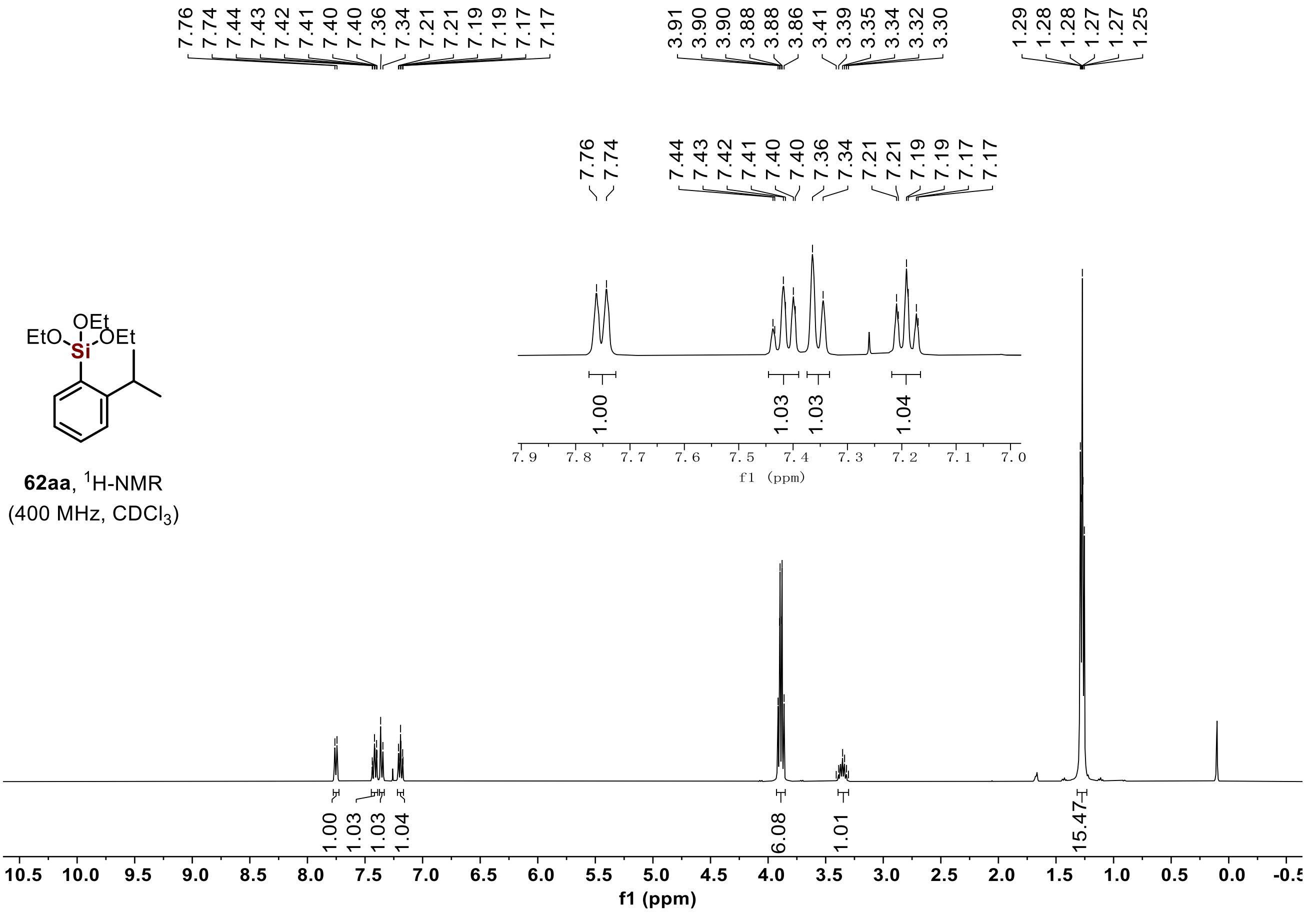

62aa, ^{1}H-NMR
(400 MHz, $CDCl_3$)

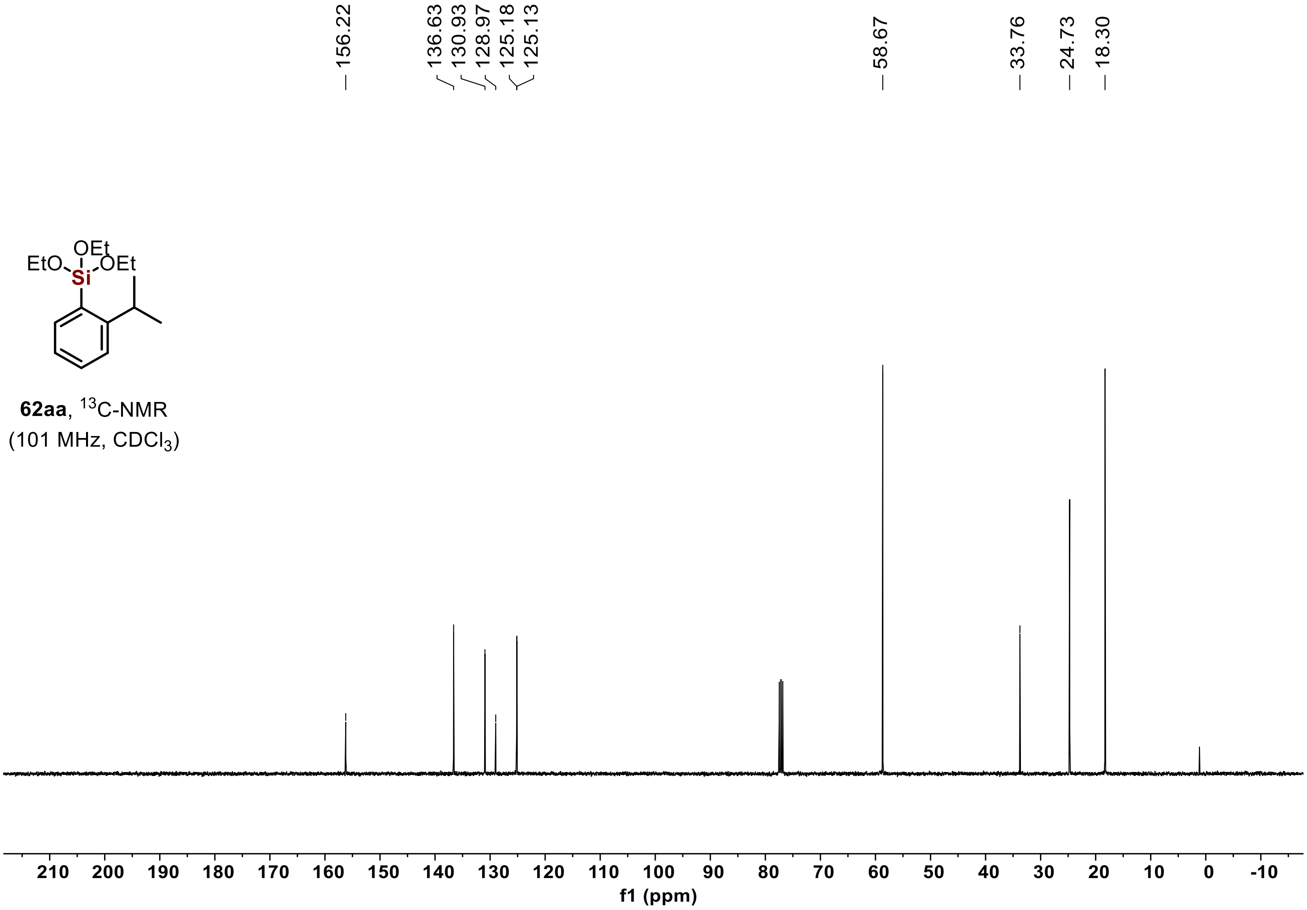

62aa, ^{13}C-NMR
(101 MHz, $CDCl_3$)

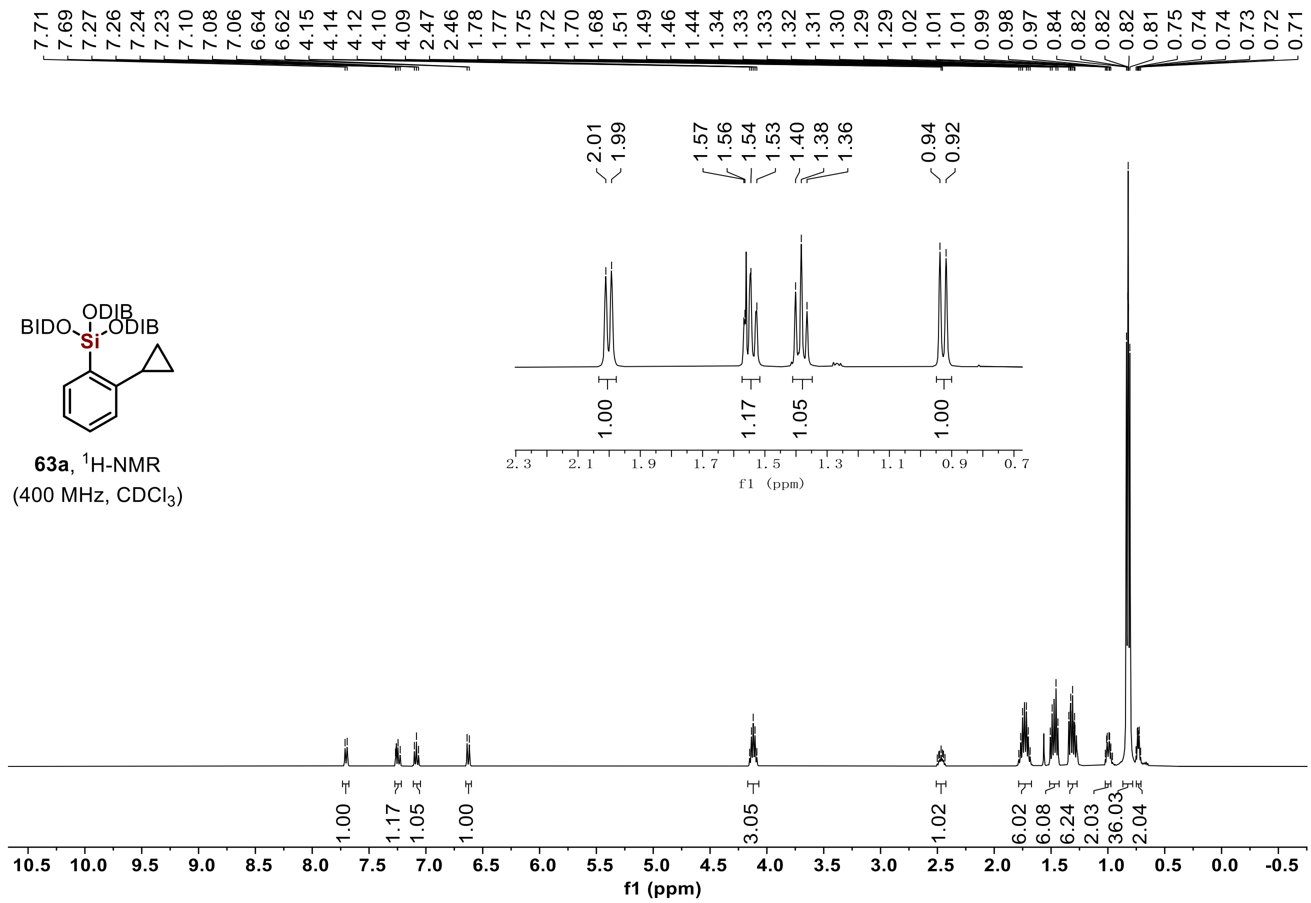

63a, ^{1}H-NMR
(400 MHz, $CDCl_3$)

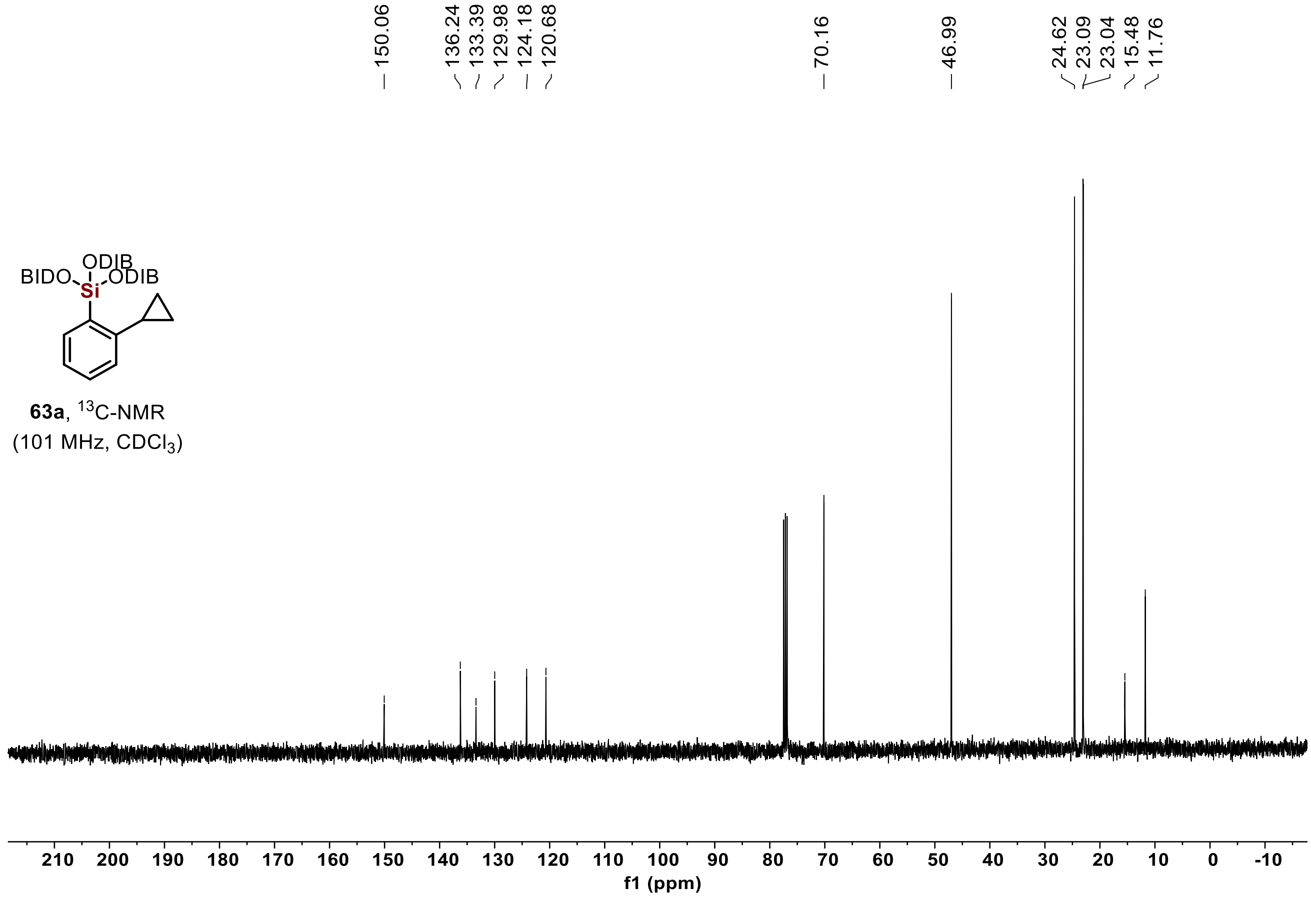

63a, ^{13}C-NMR
(101 MHz, $CDCl_3$)

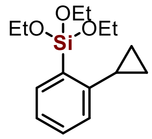

63aa, ^{1}H-NMR
(400 MHz, $CDCl_3$)

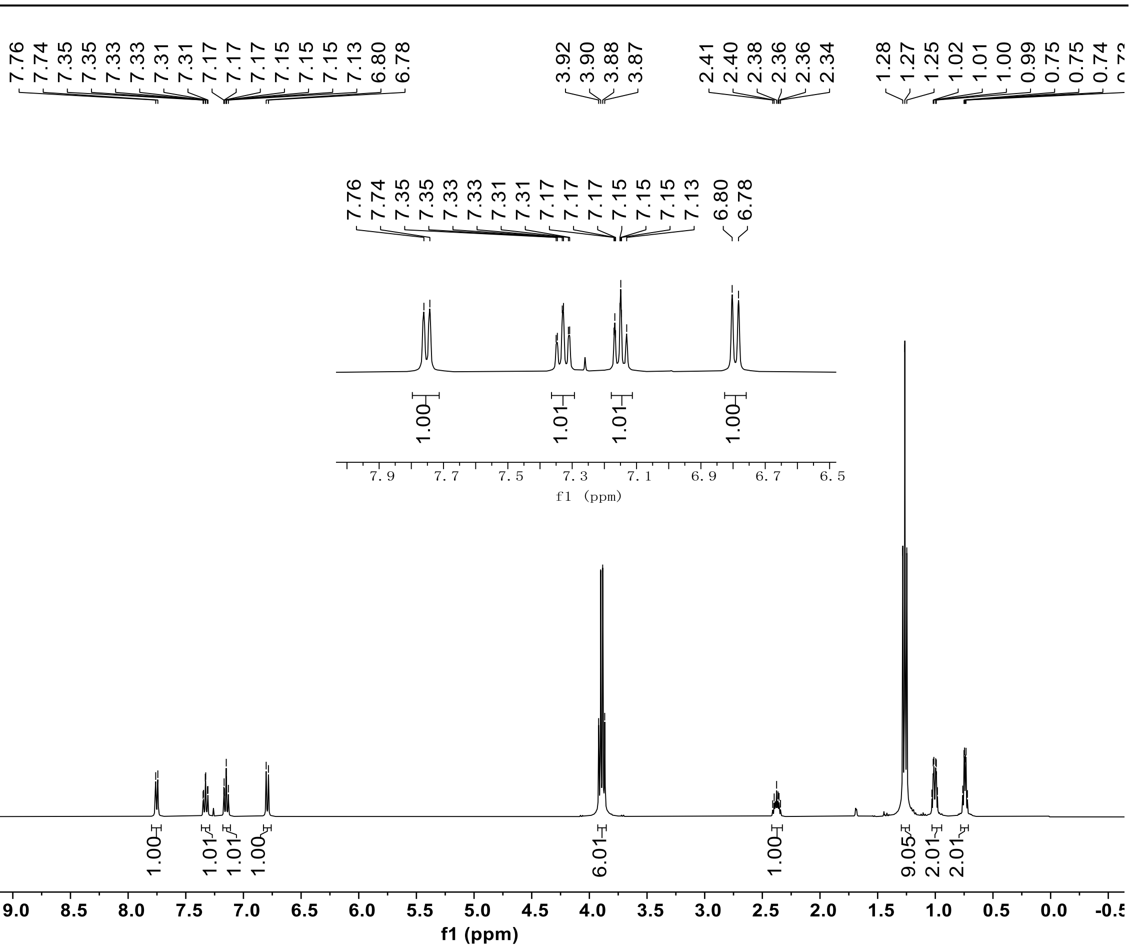

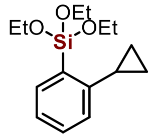

63aa, ^{13}C-NMR
(101 MHz, $CDCl_3$)

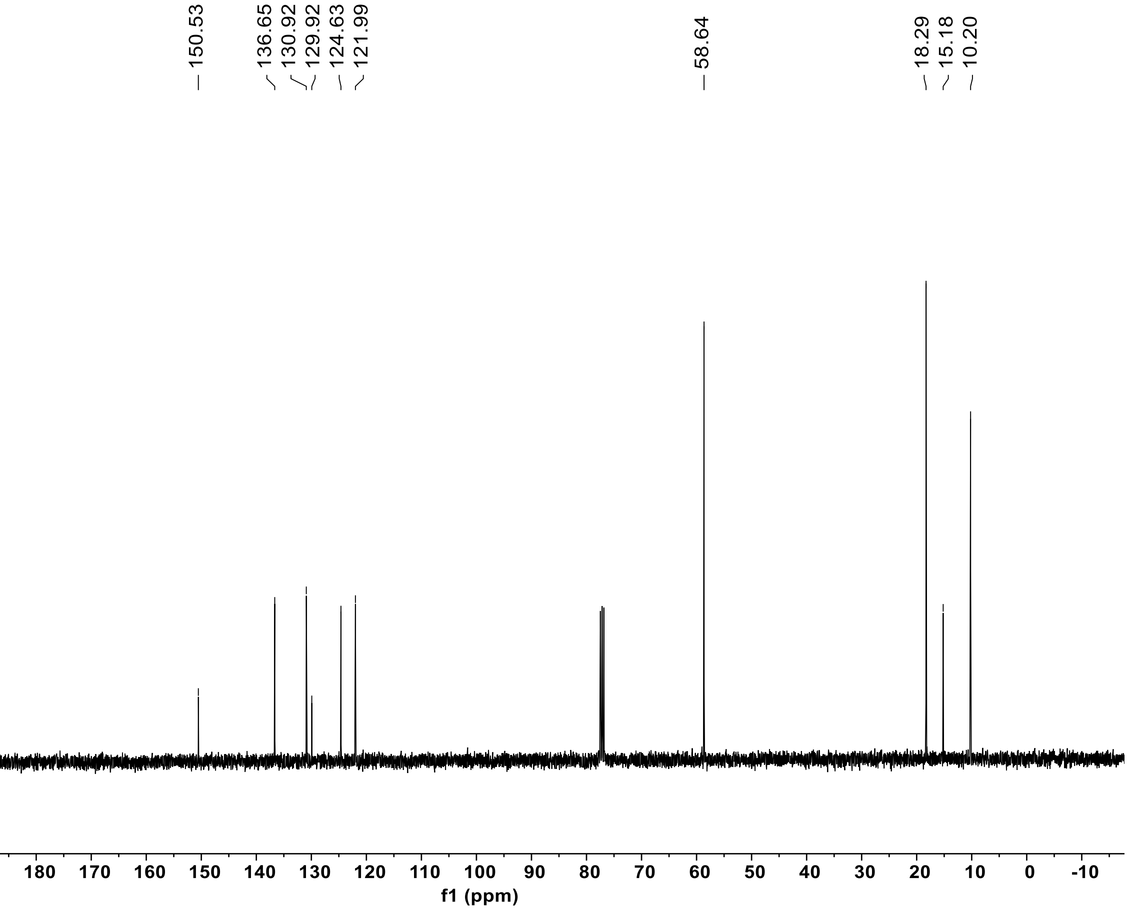

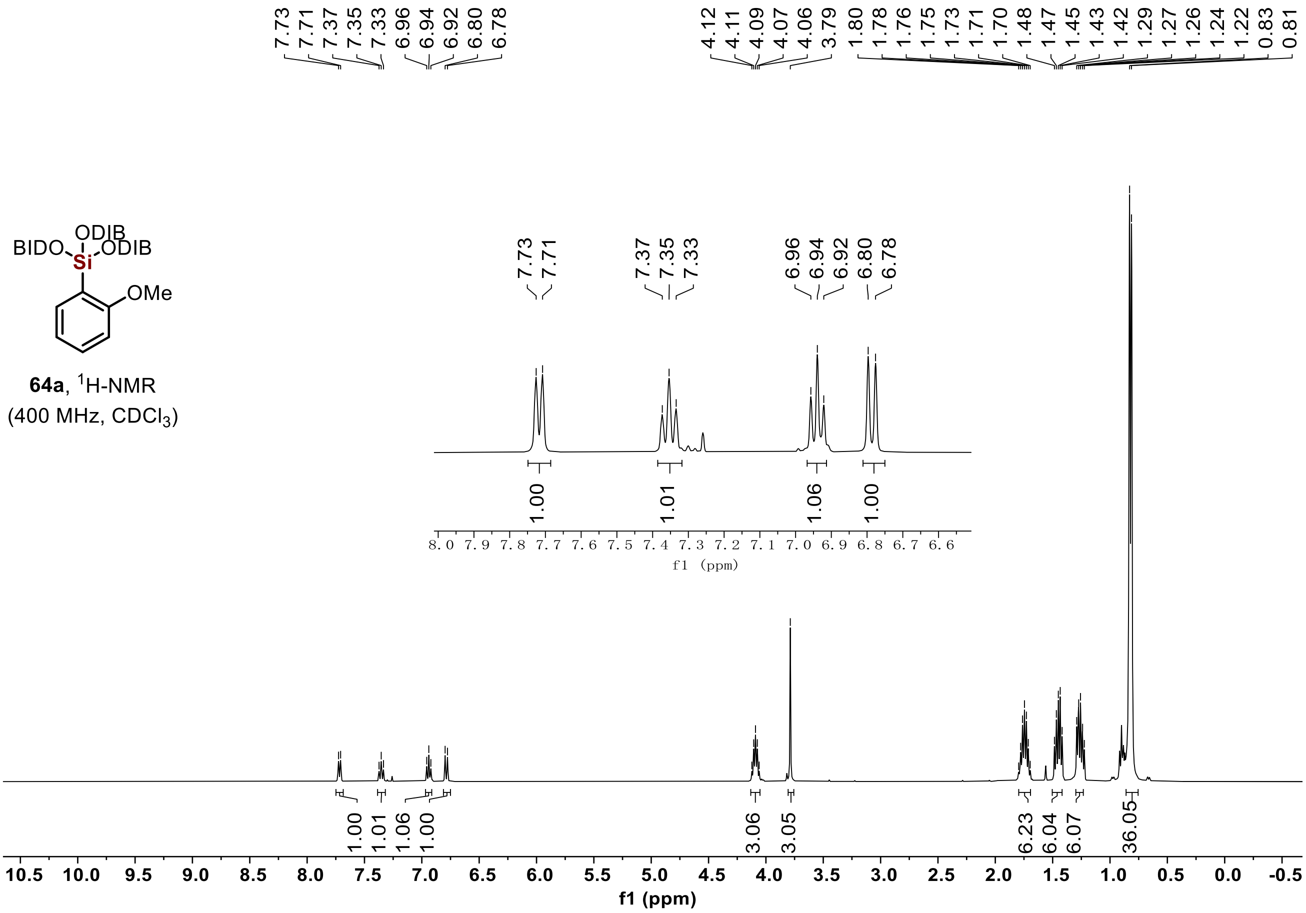

64a, ¹H-NMR
(400 MHz, $CDCl_3$)

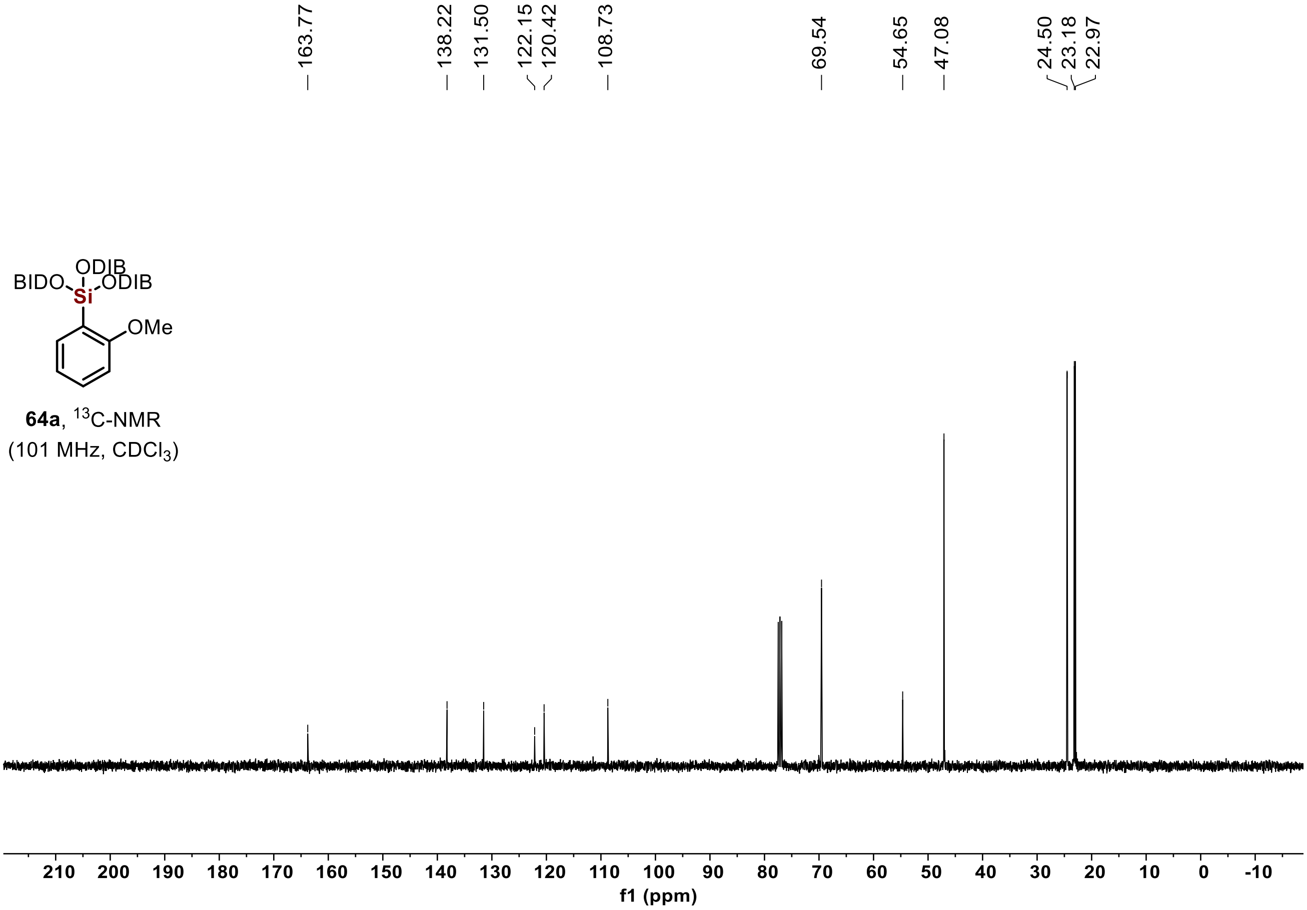

64a, ¹³C-NMR
(101 MHz, $CDCl_3$)

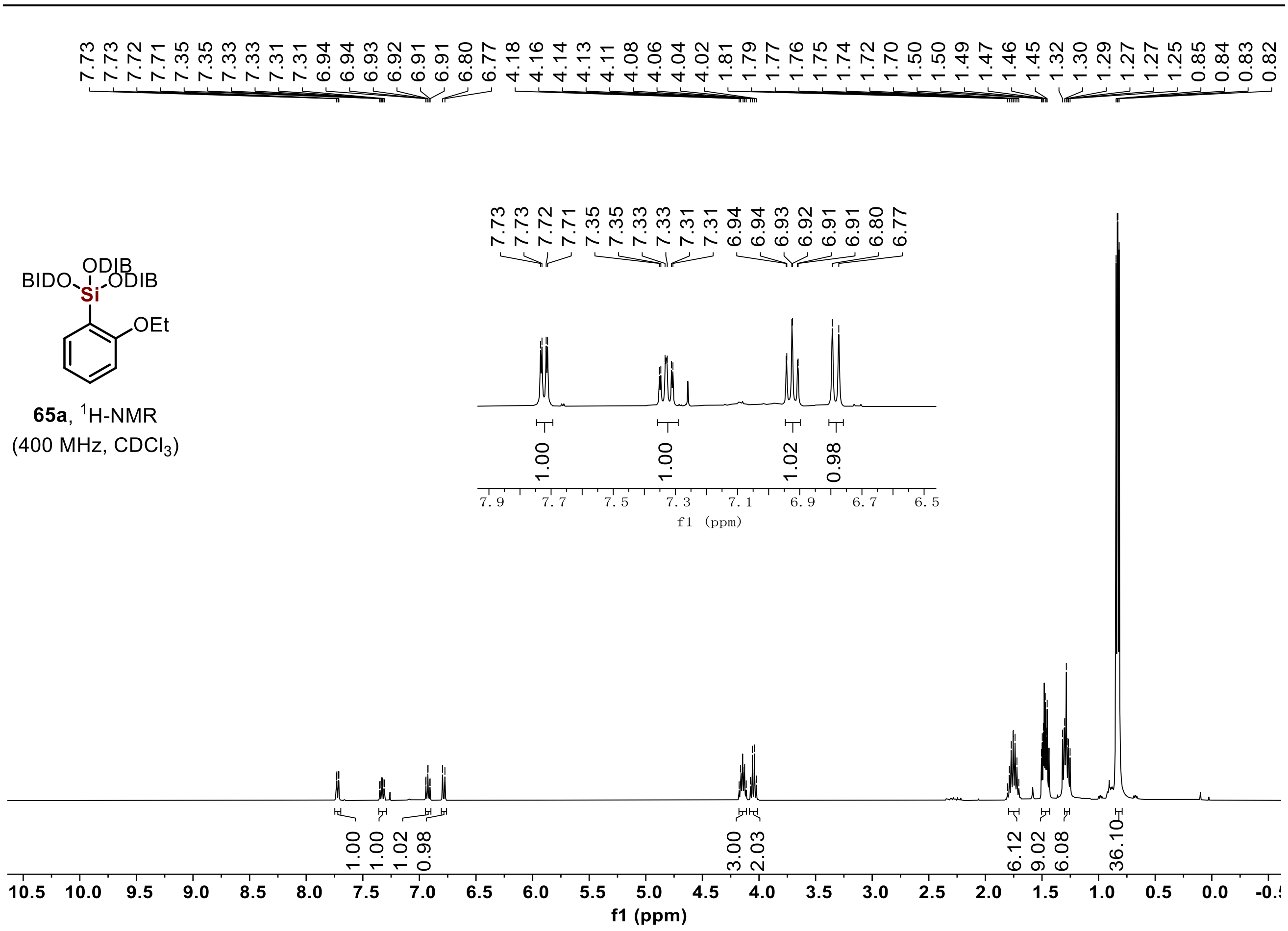

65a, ^{1}H-NMR (400 MHz, $CDCl_3$)

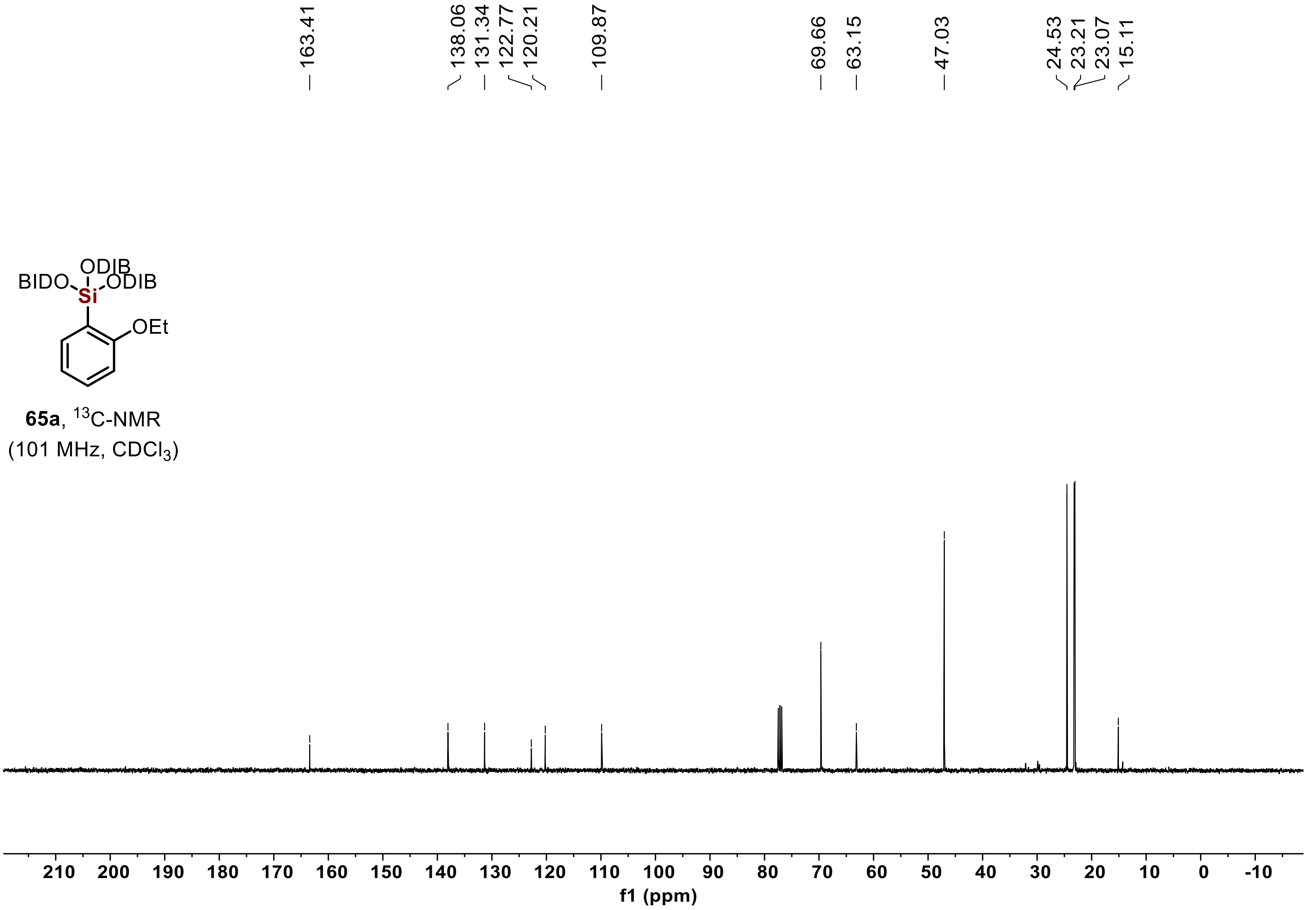

65a, ^{13}C-NMR (101 MHz, $CDCl_3$)

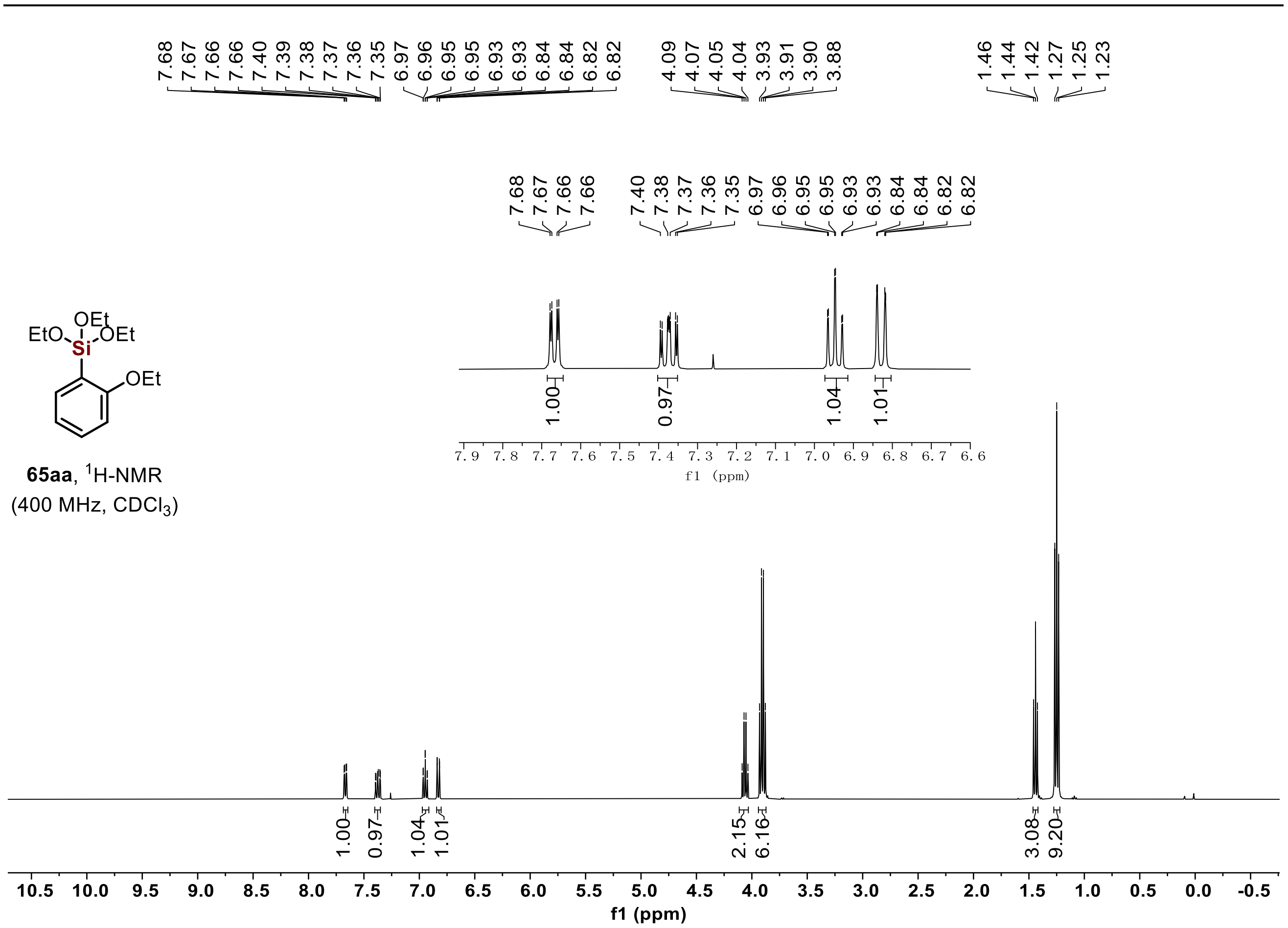

65aa, ^{1}H-NMR
(400 MHz, $CDCl_3$)

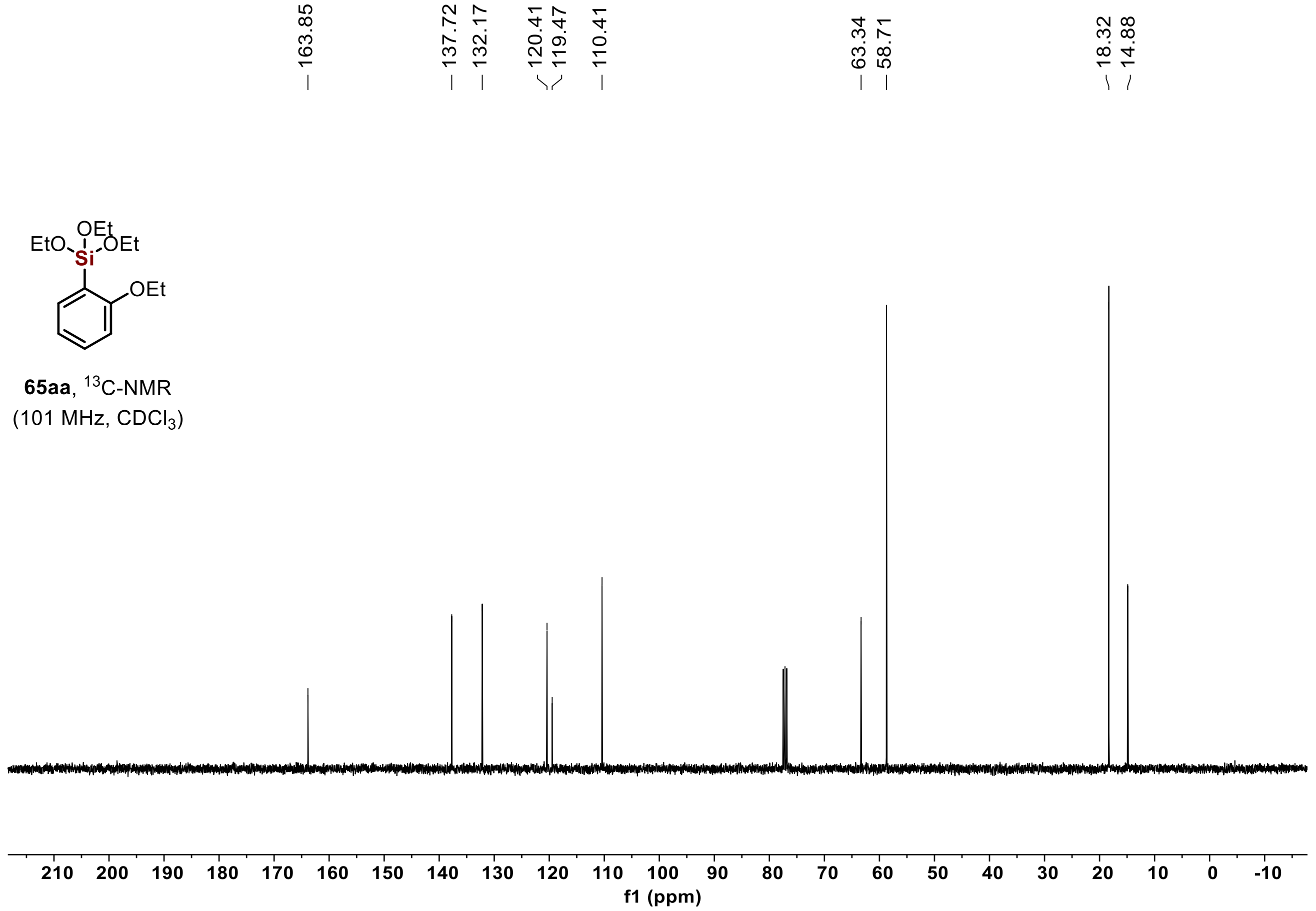

65aa, ^{13}C-NMR
(101 MHz, $CDCl_3$)

66a, ^{1}H-NMR
(400 MHz, $CDCl_3$)

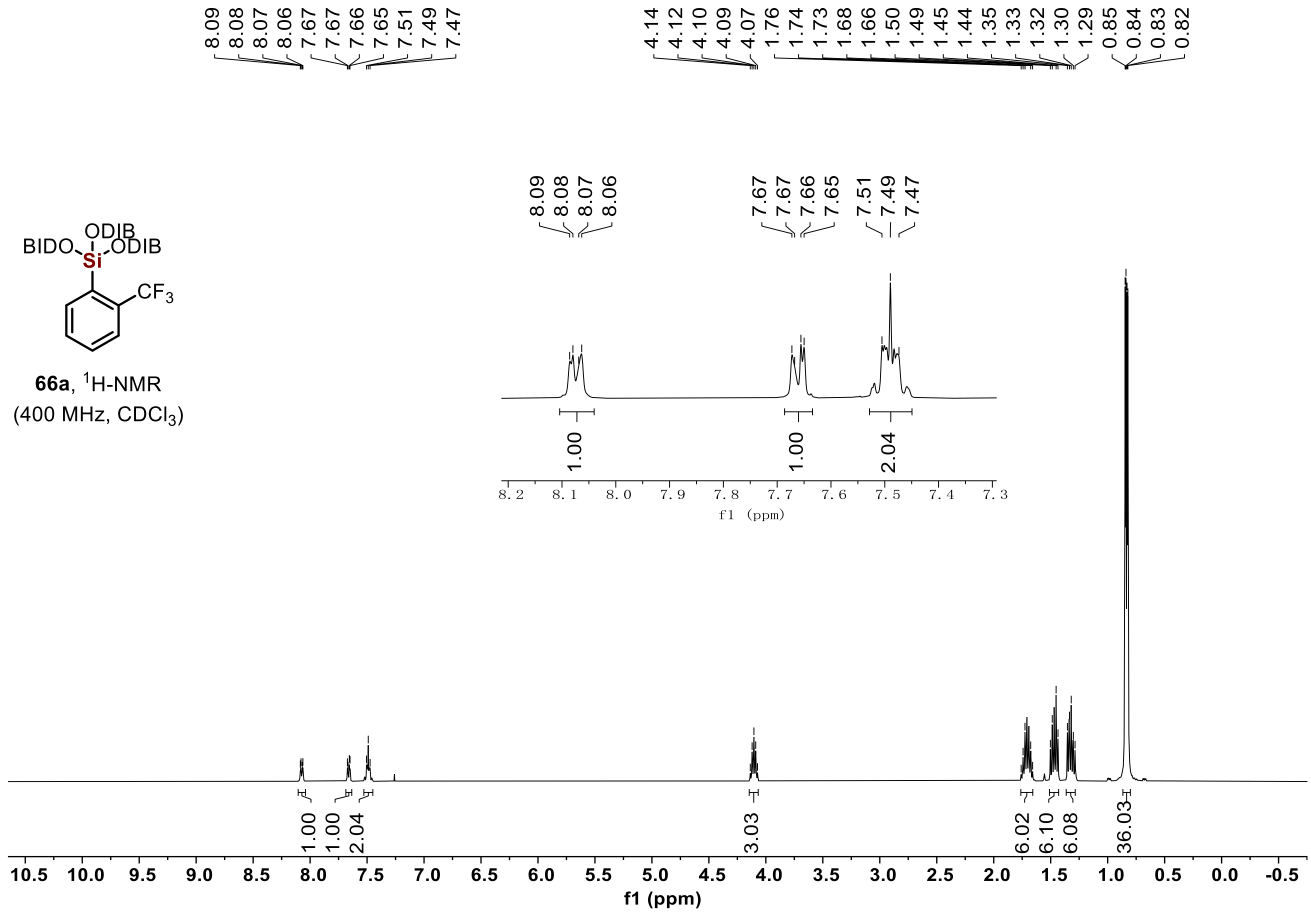

66a, ^{13}C-NMR
(101 MHz, $CDCl_3$)

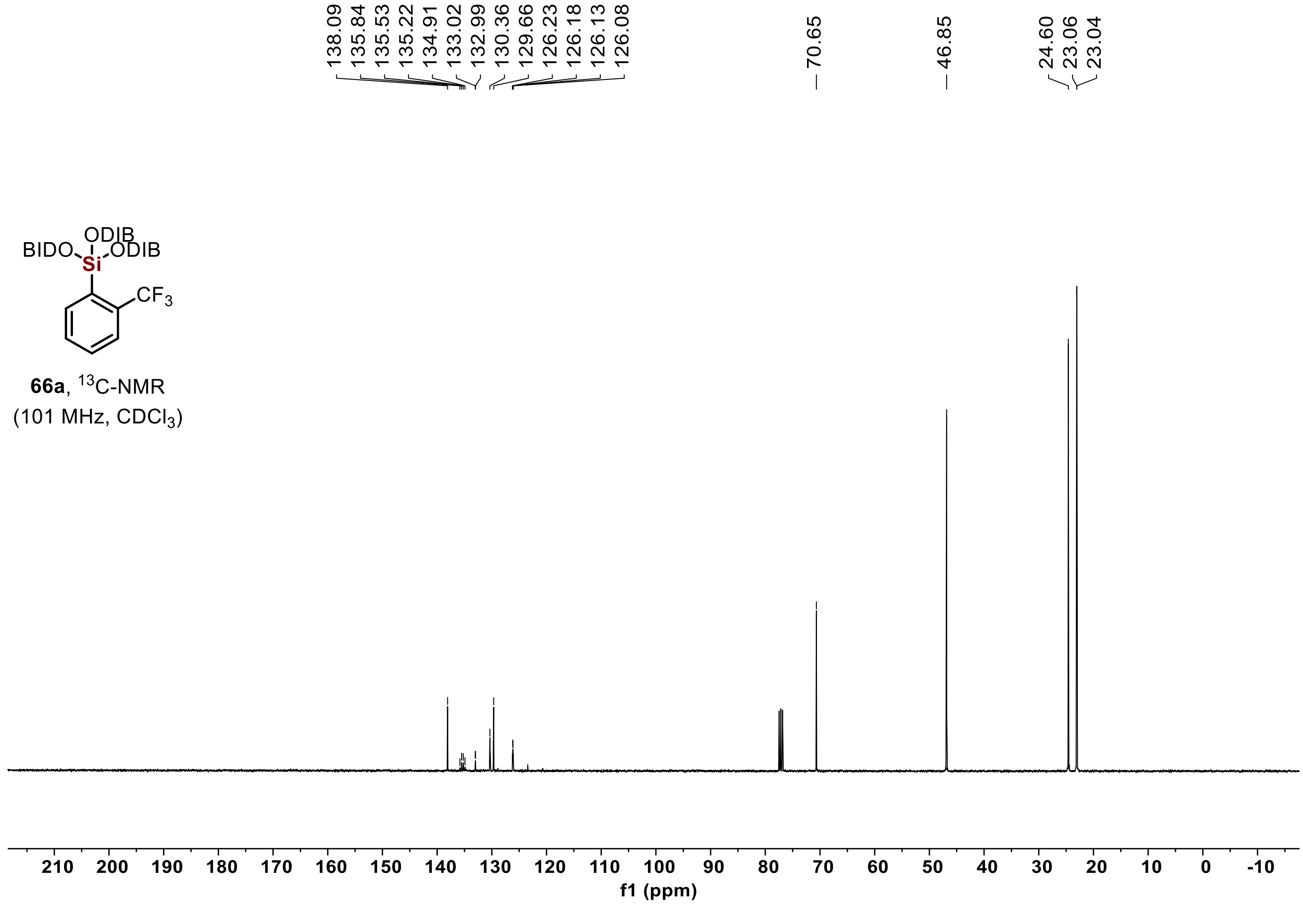

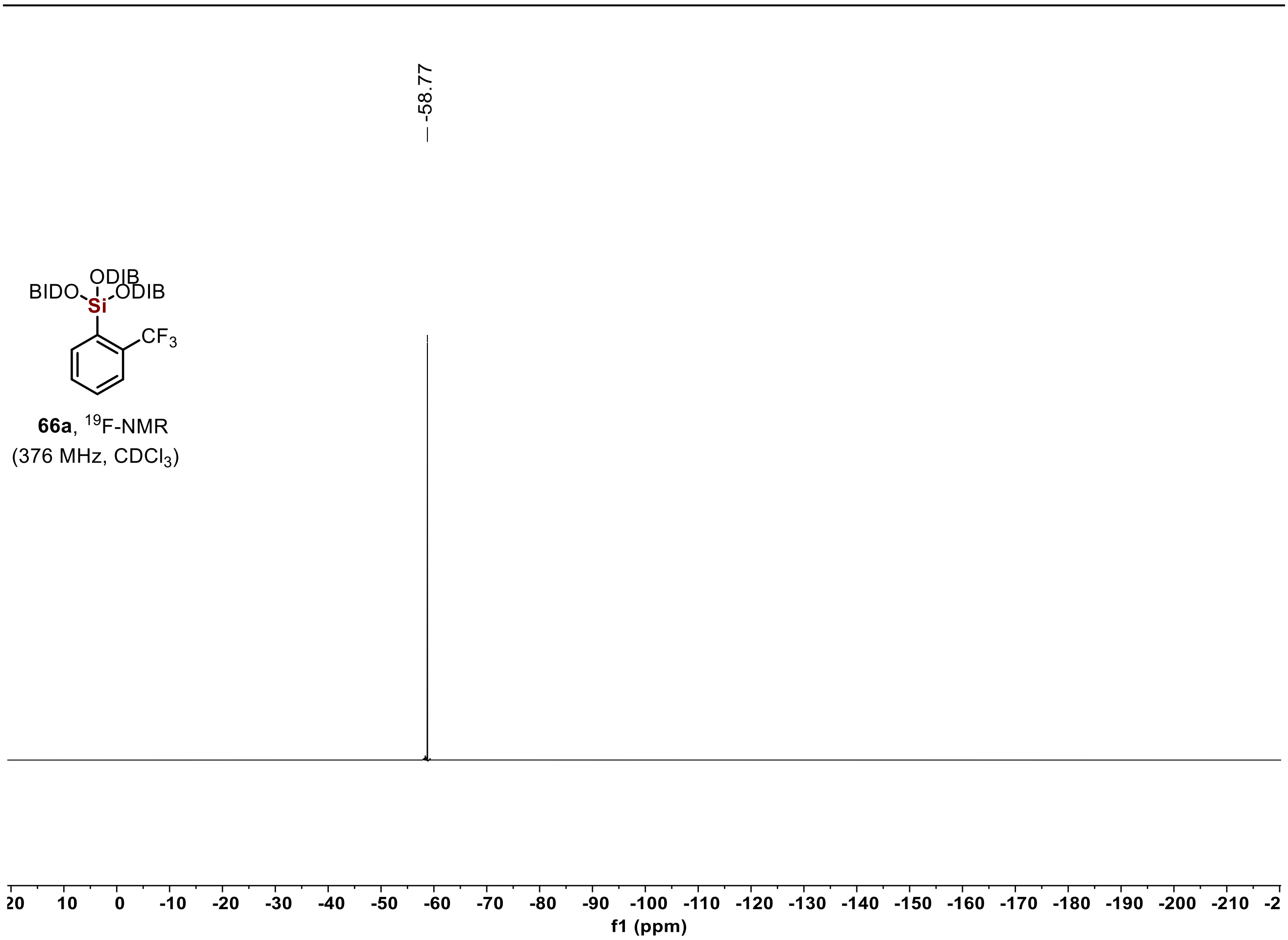

66a, ^{19}F-NMR
(376 MHz, $CDCl_3$)

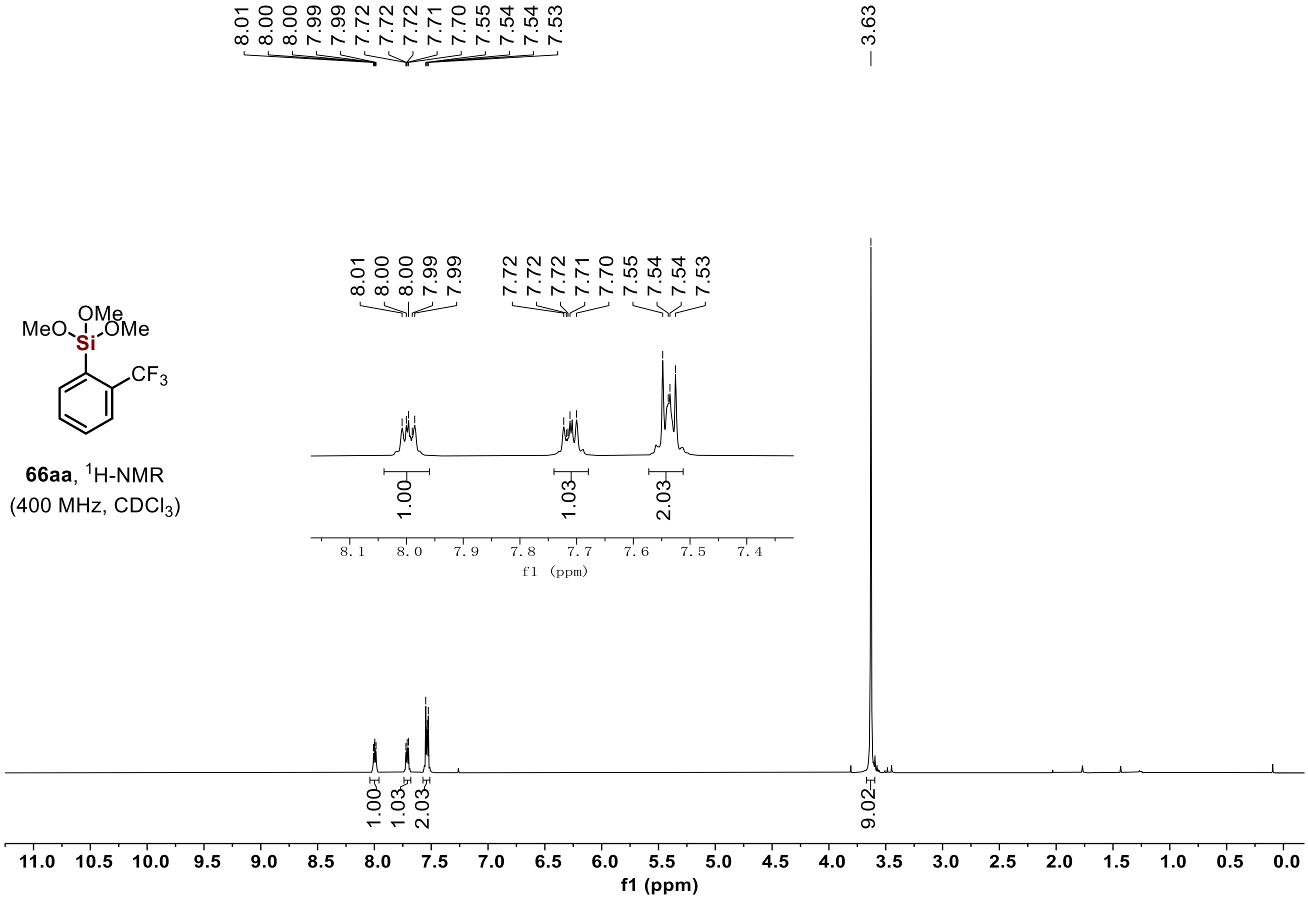

66aa, ^{1}H-NMR
(400 MHz, $CDCl_3$)

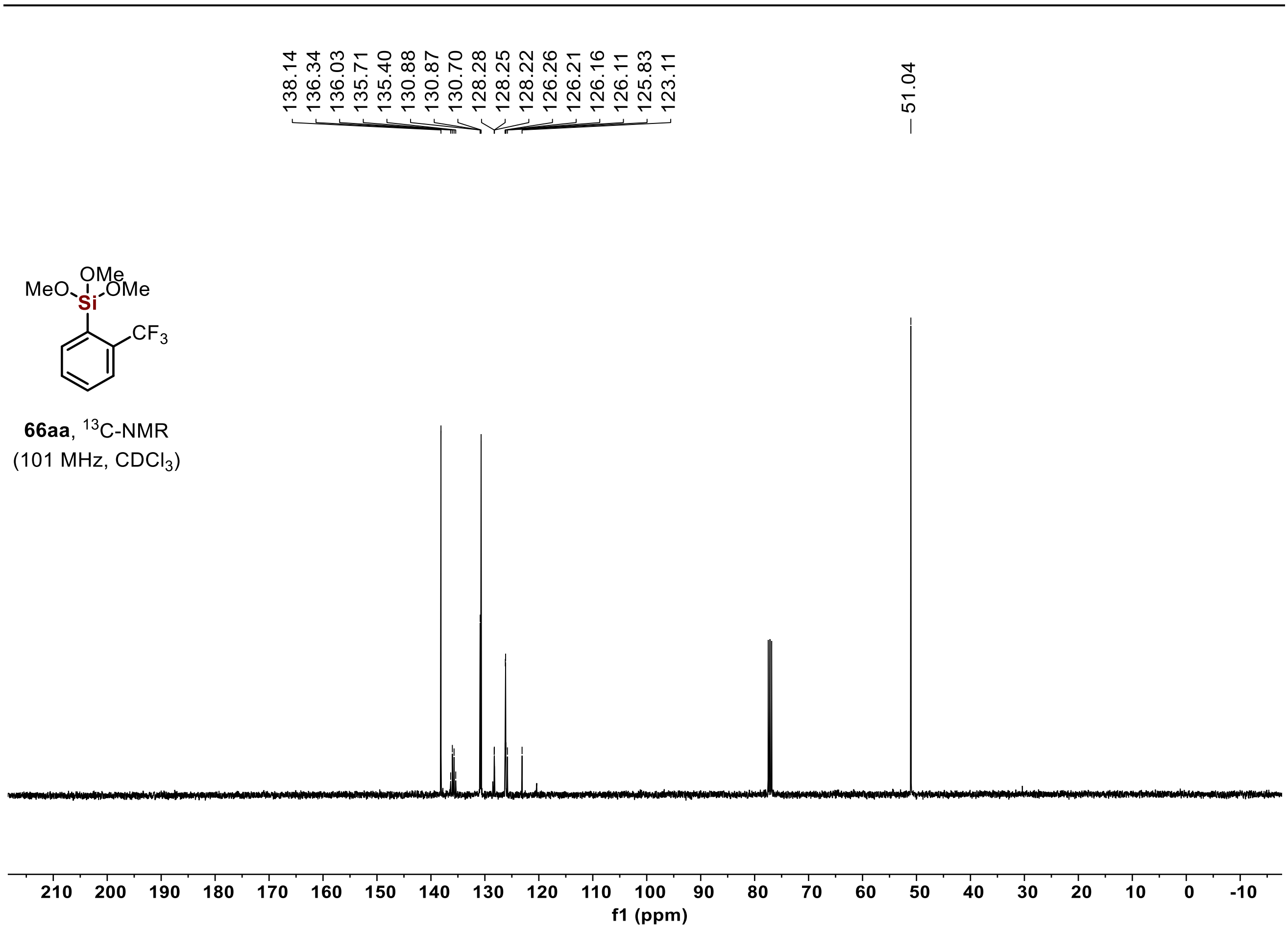
138.14
136.34
136.03
135.71
135.40
130.88
130.87
130.70
128.28
128.25
128.22
126.26
126.21
126.16
126.11
125.83
123.11
51.04
OMe
MeO
Si
OMe
CF3
66aa, 13C-NMR
(101 MHz, CDCl3)
210 200 190 180 170 160 150 140 130 120 110 100 90 80 70 60 50 40 30 20 10 0 -10
f1 (ppm)

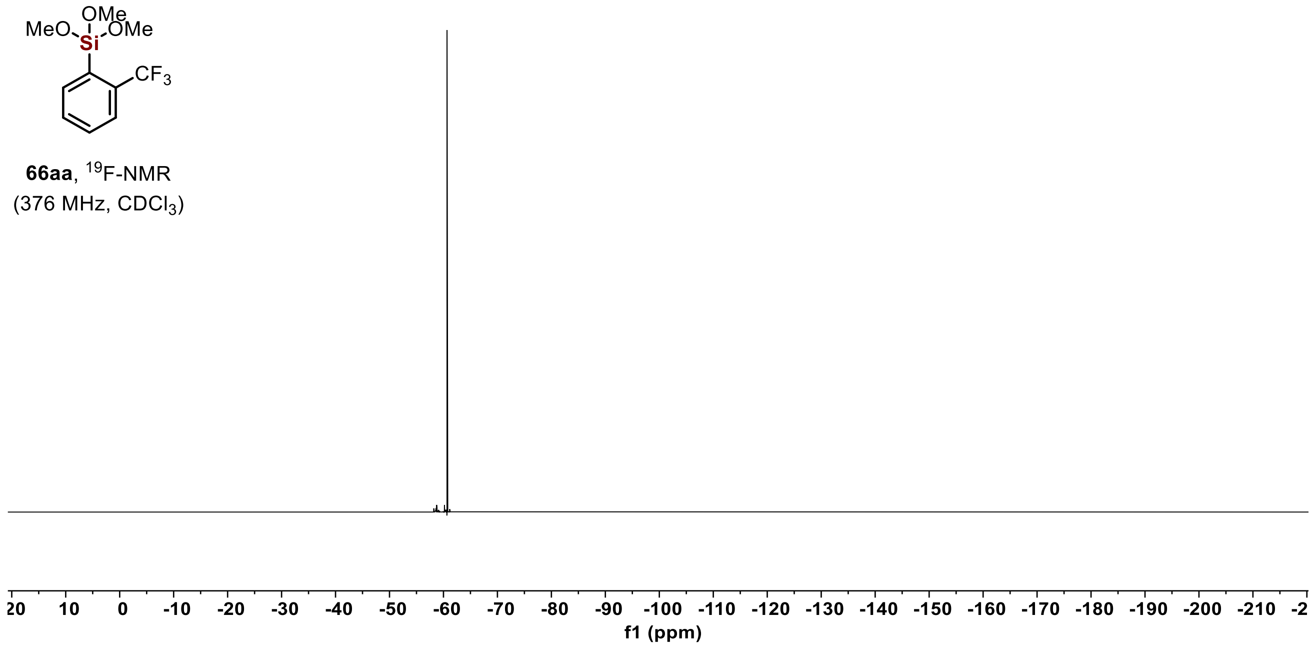
OMe
MeO
Si
OMe
CF3
66aa, 19F-NMR
(376 MHz, CDCl3)
20 10 0 -10 -20 -30 -40 -50 -60 -70 -80 -90 -100 -110 -120 -130 -140 -150 -160 -170 -180 -190 -200 -210
f1 (ppm)

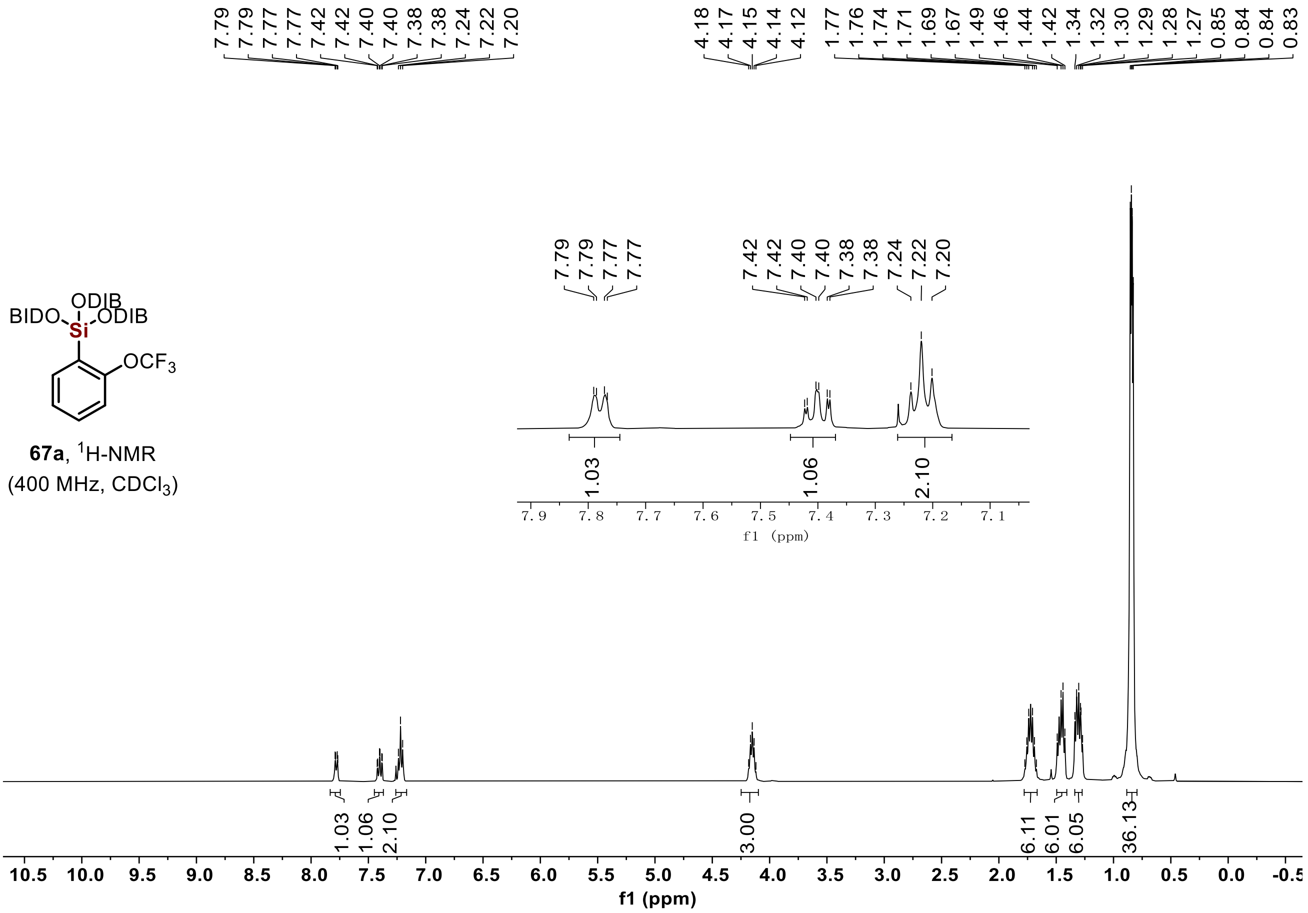

67a, ^{1}H-NMR
(400 MHz, $CDCl_3$)

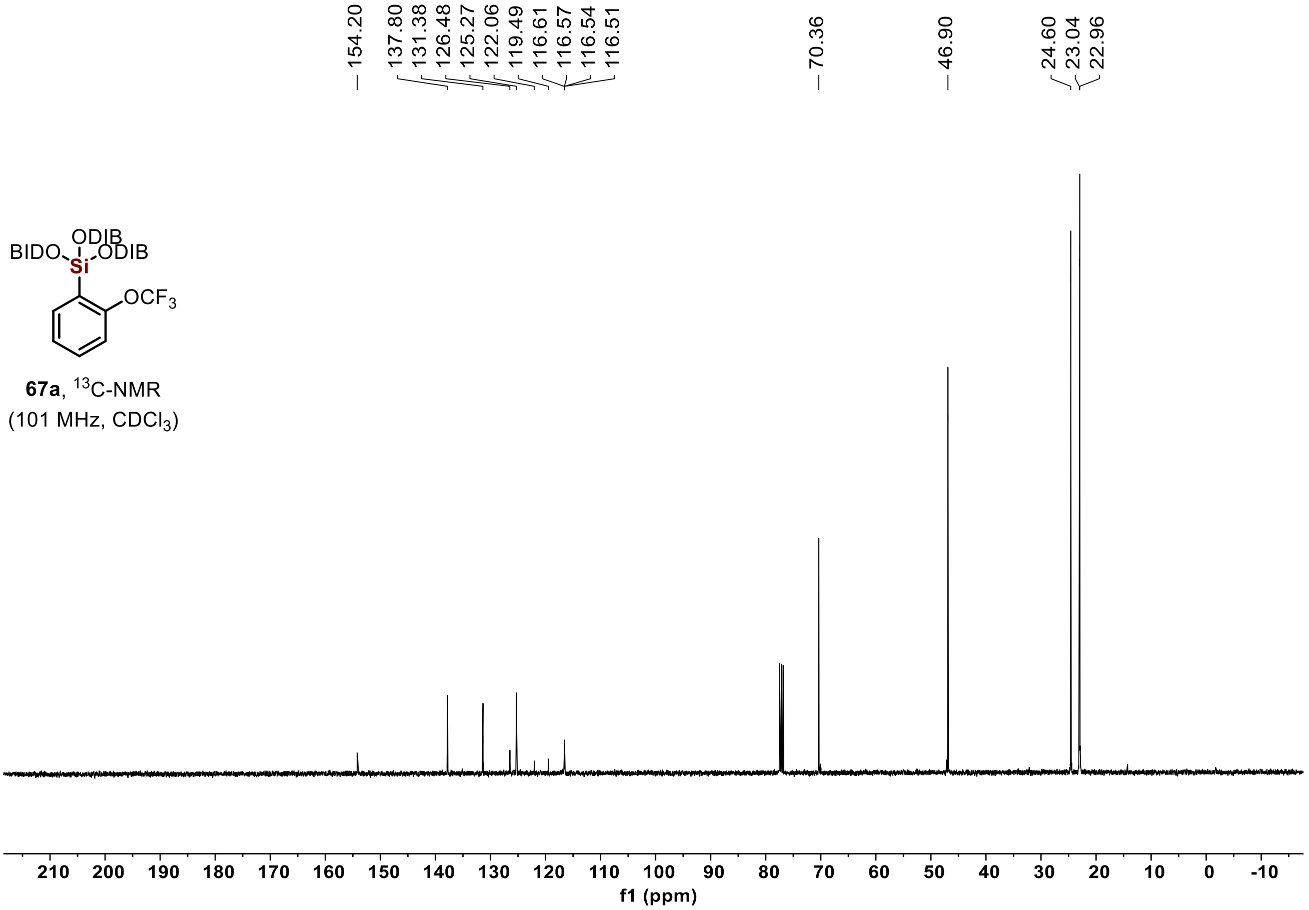

67a, ^{13}C-NMR
(101 MHz, $CDCl_3$)

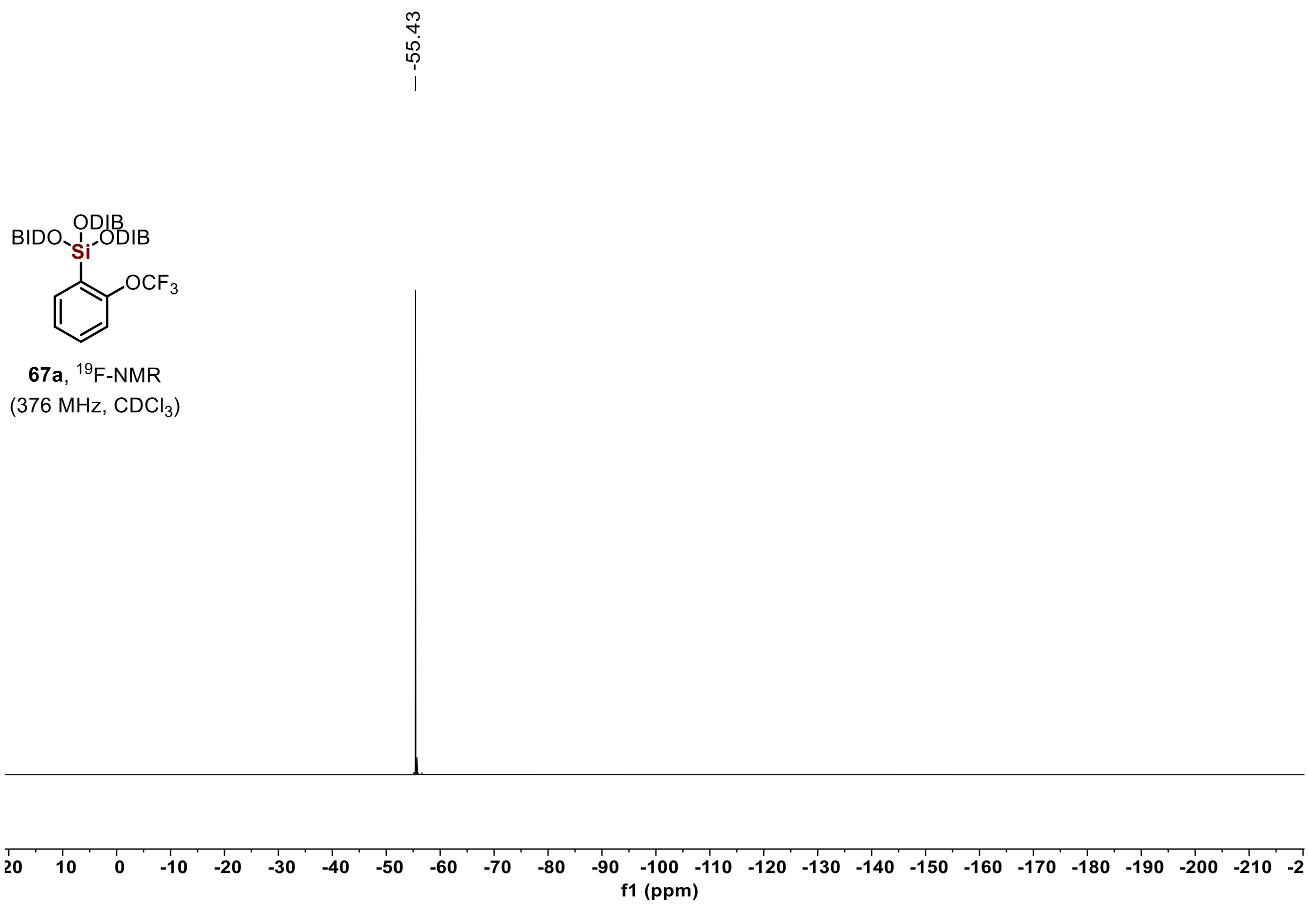

67a, ^{19}F-NMR
(376 MHz, $CDCl_3$)

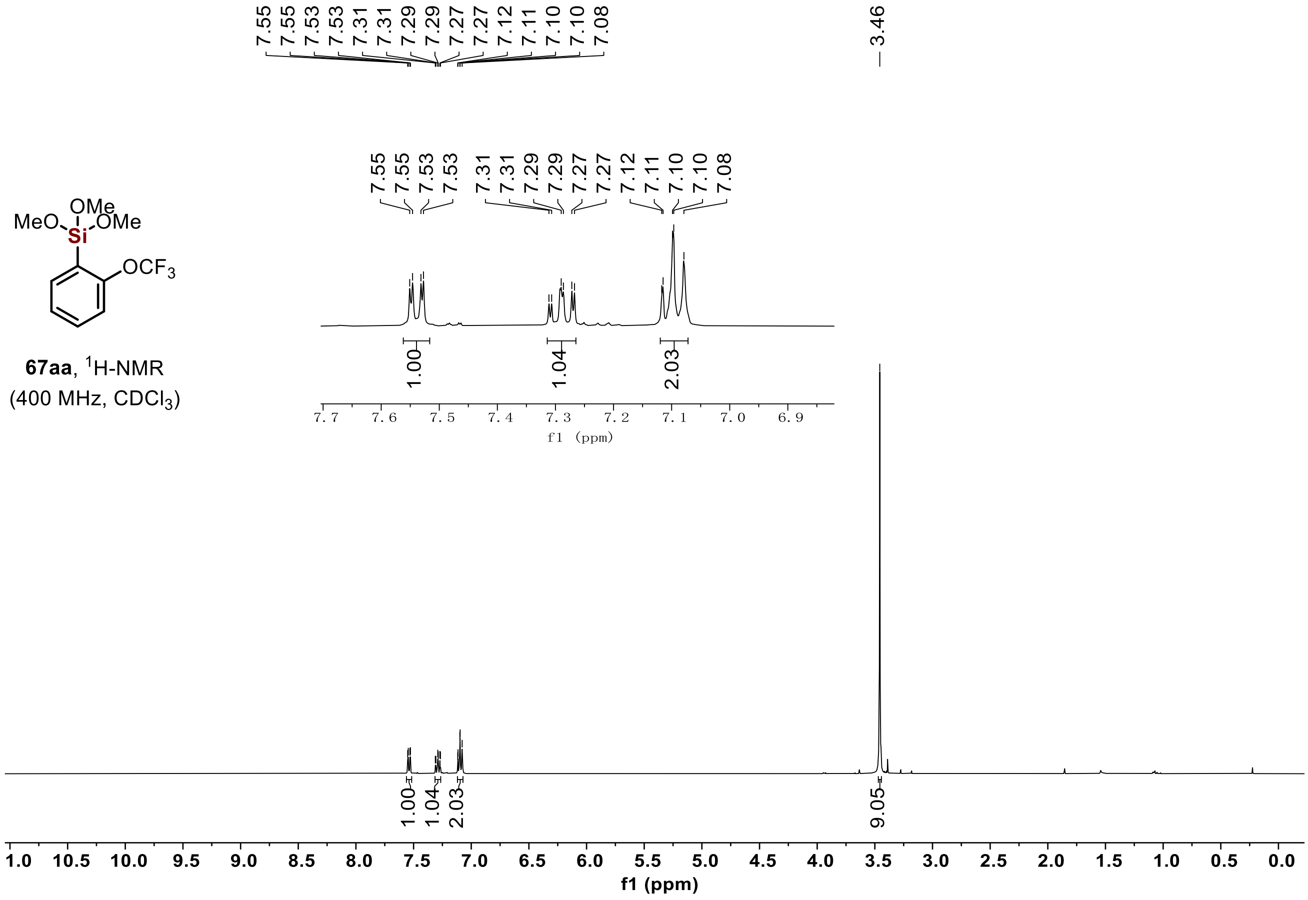

67aa, ^{1}H-NMR
(400 MHz, $CDCl_3$)

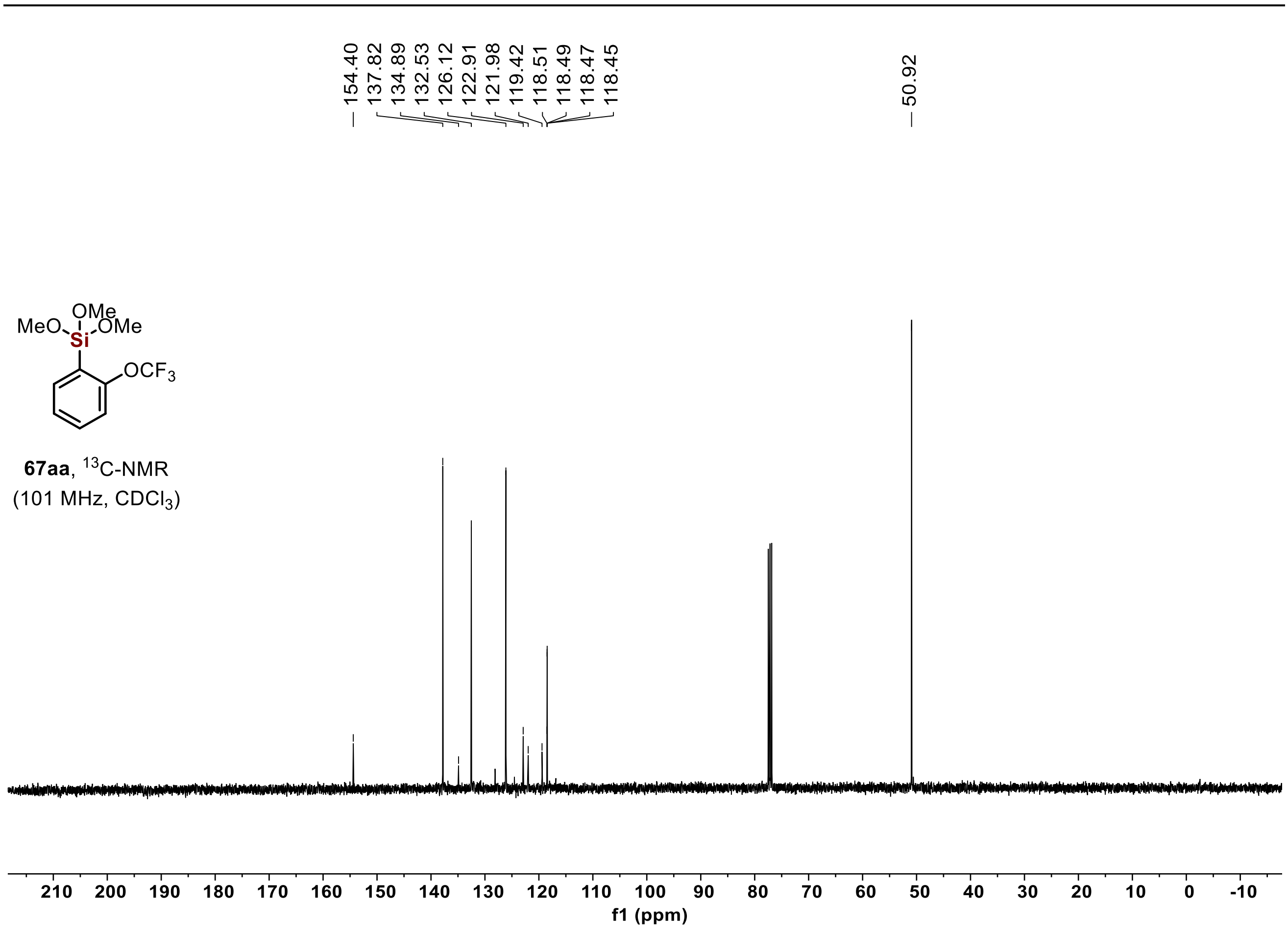

67aa, ^{13}C-NMR
(101 MHz, $CDCl_3$)

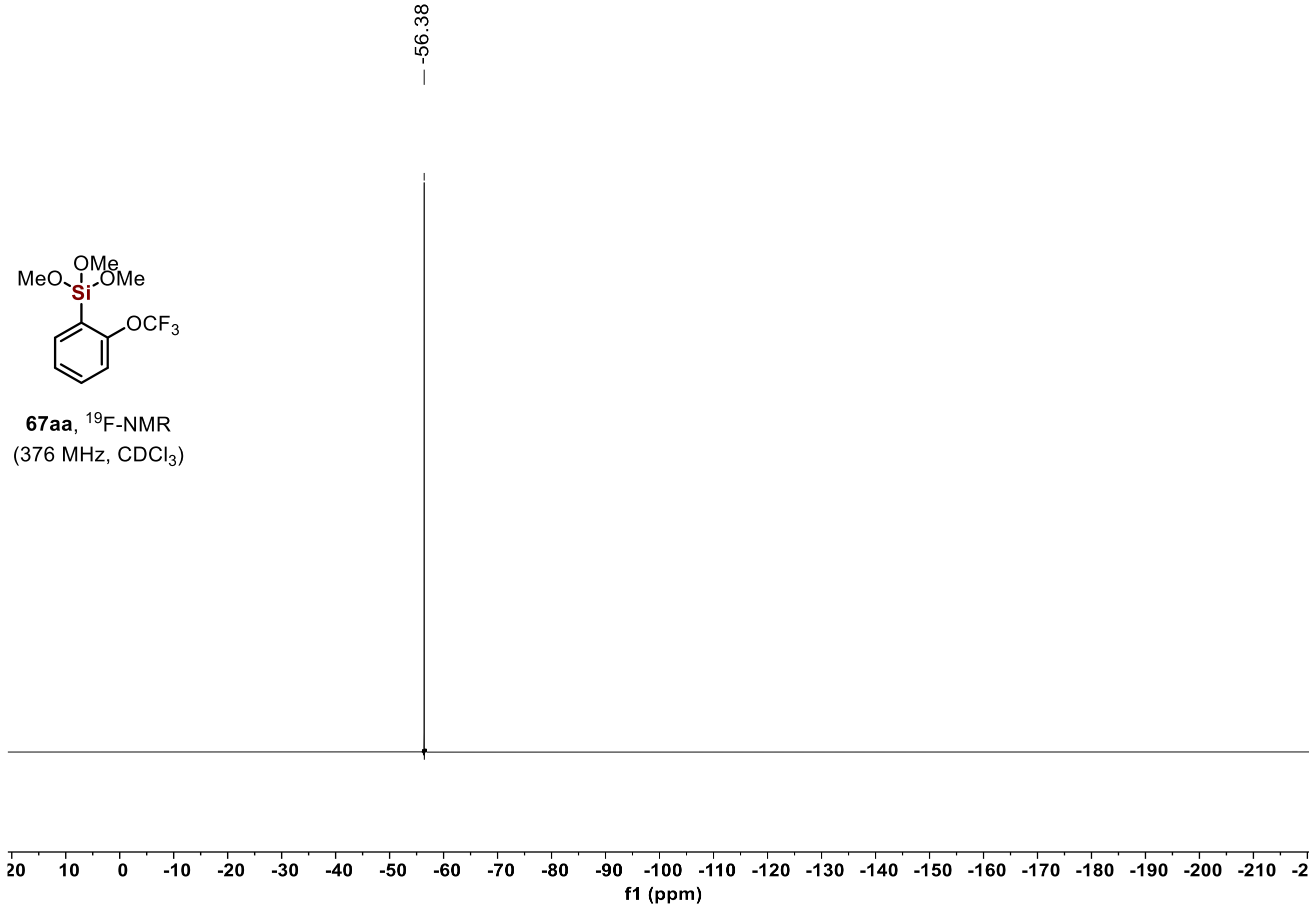

67aa, ^{19}F-NMR
(376 MHz, $CDCl_3$)

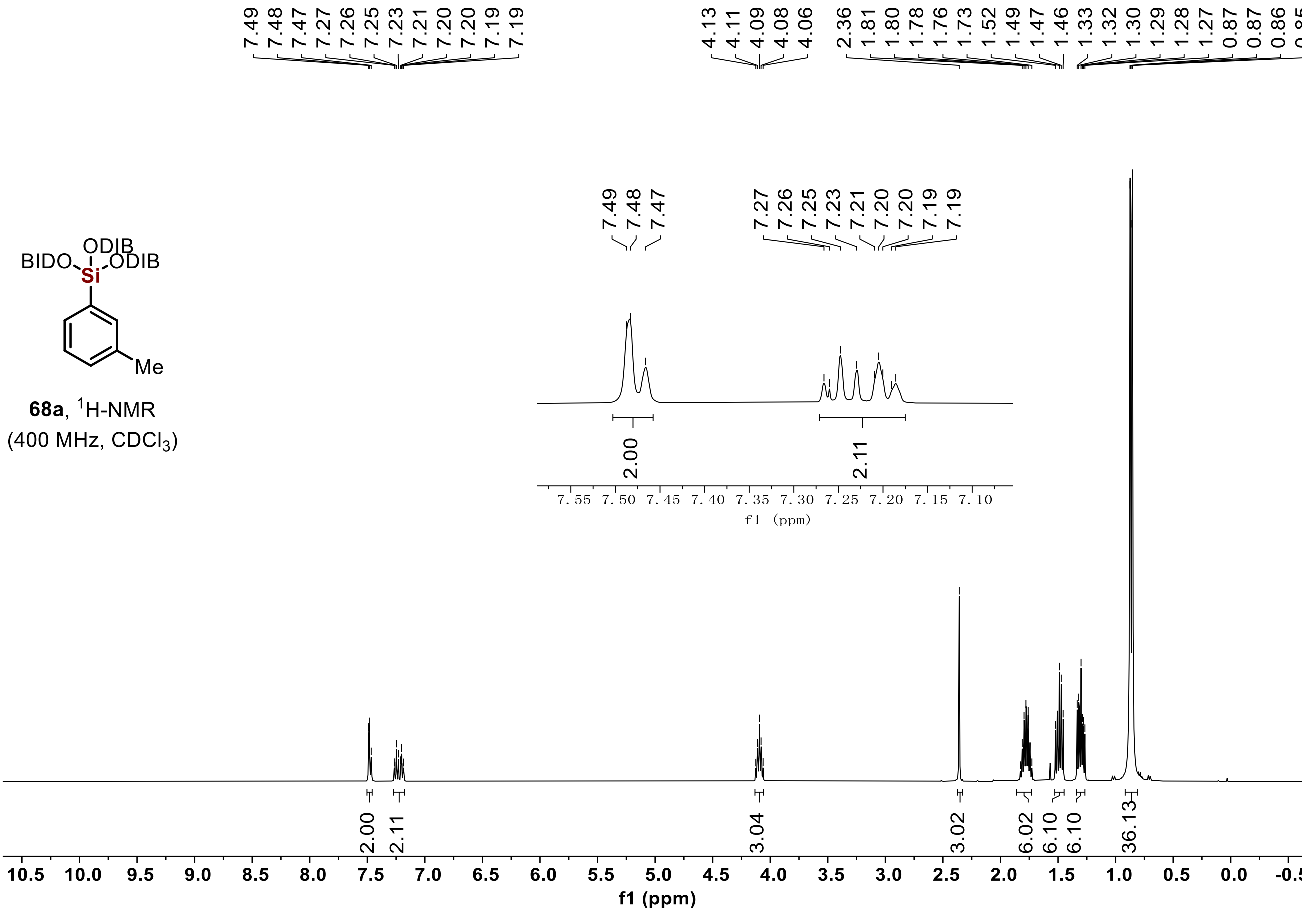
BIDO ODIB ODIB Si Me
68a, 1H-NMR
(400 MHz, CDCl3)
f1 (ppm)

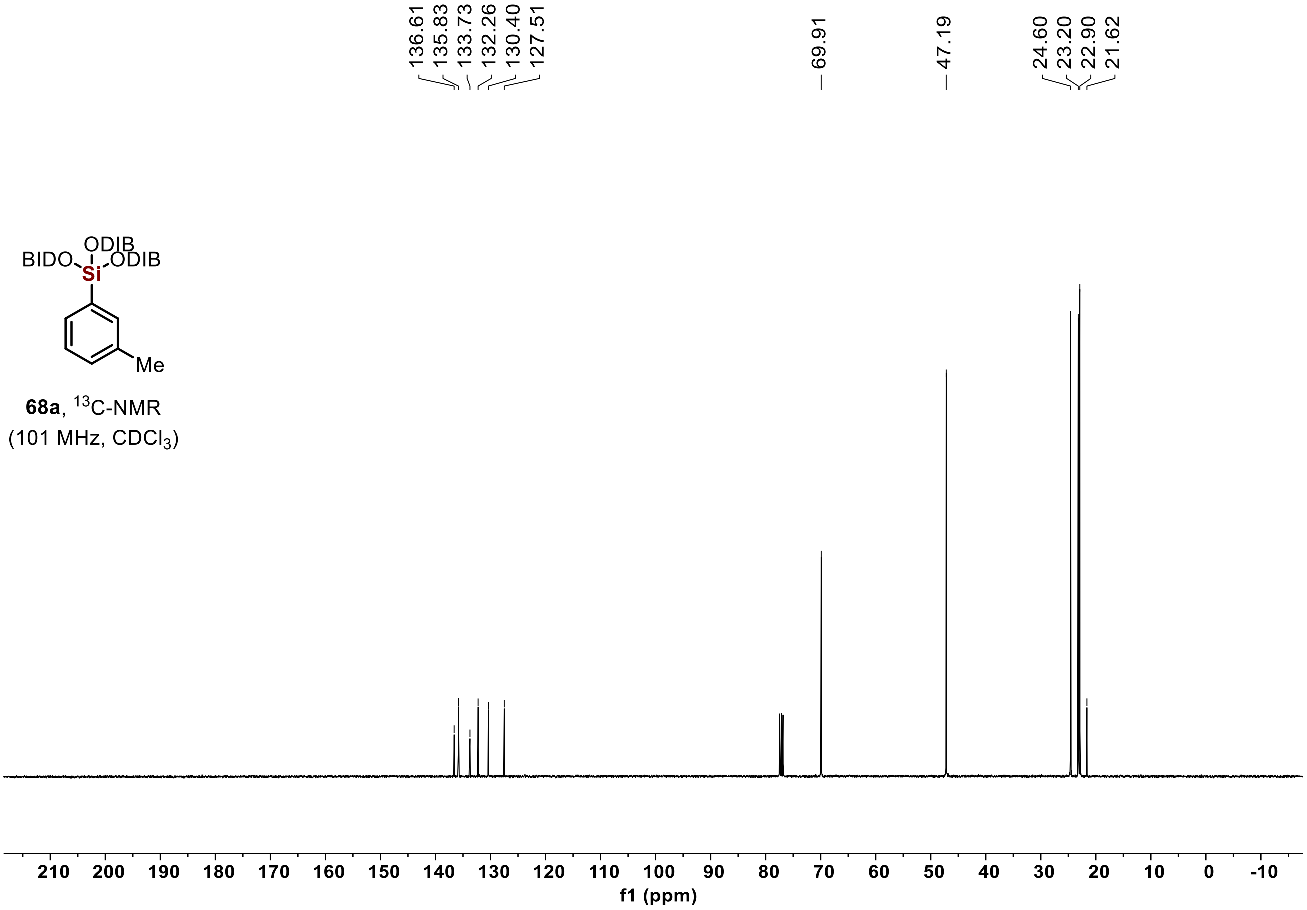
BIDO ODIB ODIB Si Me
68a, 13C-NMR
(101 MHz, CDCl3)
f1 (ppm)

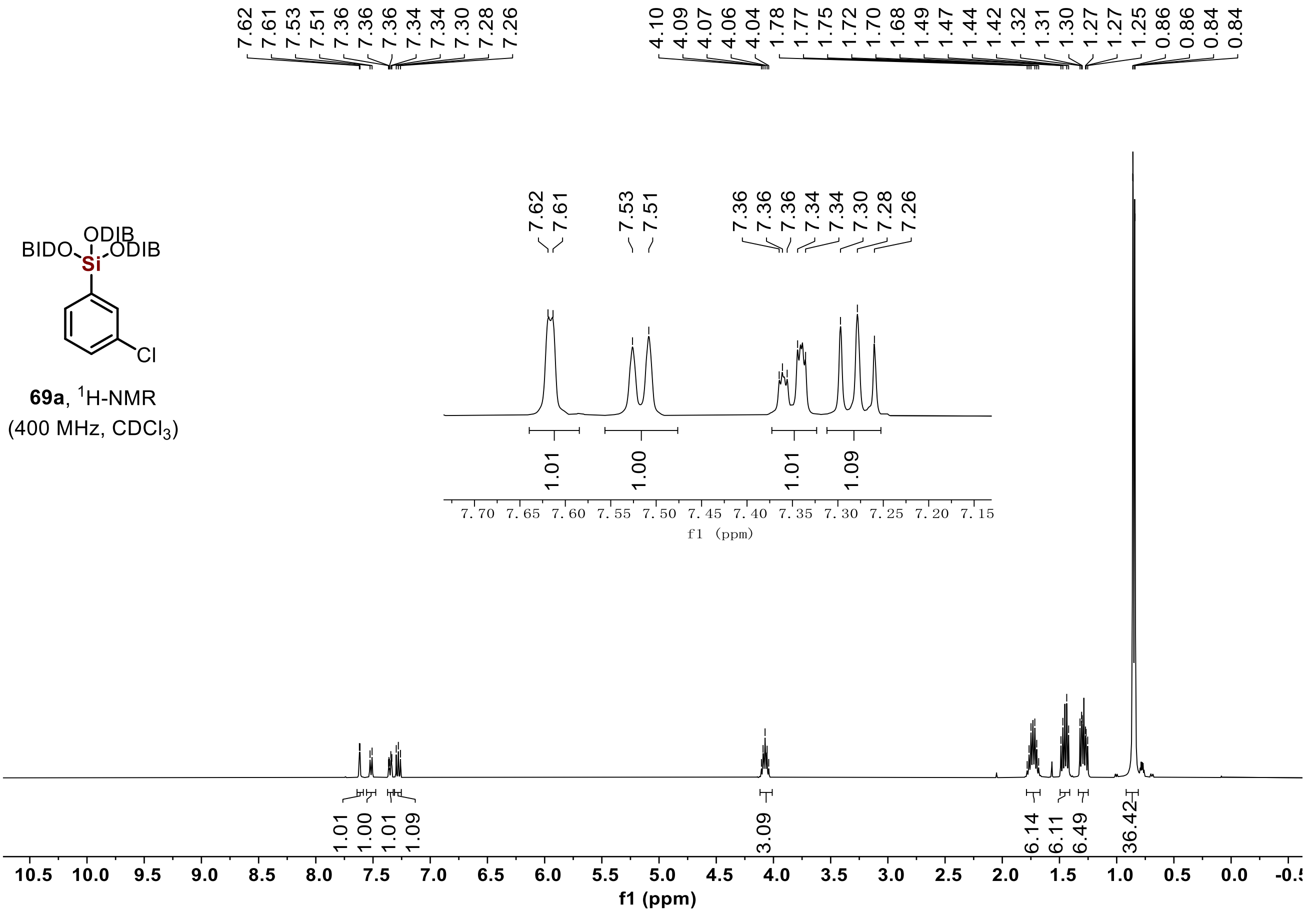
7.62
7.61
7.53
7.51
7.36
7.36
7.36
7.34
7.34
7.30
7.28
7.26
4.10
4.09
4.07
4.06
4.04
1.78
1.77
1.75
1.72
1.70
1.68
1.49
1.47
1.44
1.42
1.32
1.31
1.30
1.27
1.27
1.25
0.86
0.86
0.84
0.84
ODIB
BIDO
Si
ODIB
Cl
69a, ¹H-NMR
(400 MHz, CDCl₃)
1.01
1.00
1.01
1.09
f1 (ppm)
3.09
6.14
6.11
6.49
36.42
f1 (ppm)

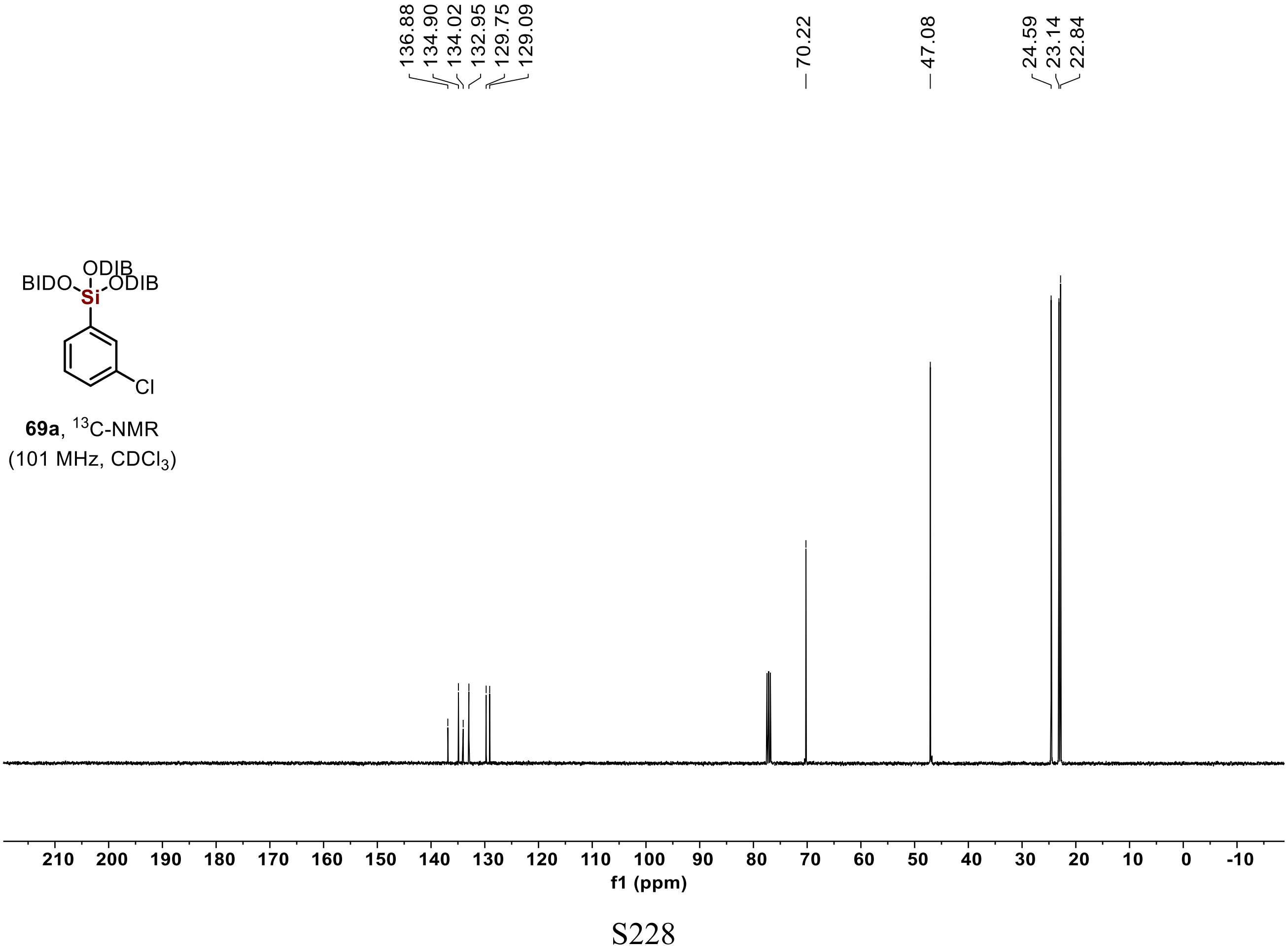
136.88
134.90
134.02
132.95
129.75
129.09
70.22
47.08
24.59
23.14
22.84
ODIB
BIDO
Si
ODIB
Cl
69a, ¹³C-NMR
(101 MHz, CDCl₃)
f1 (ppm)

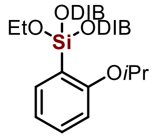

70a, ^{1}H-NMR
(400 MHz, $CDCl_3$)

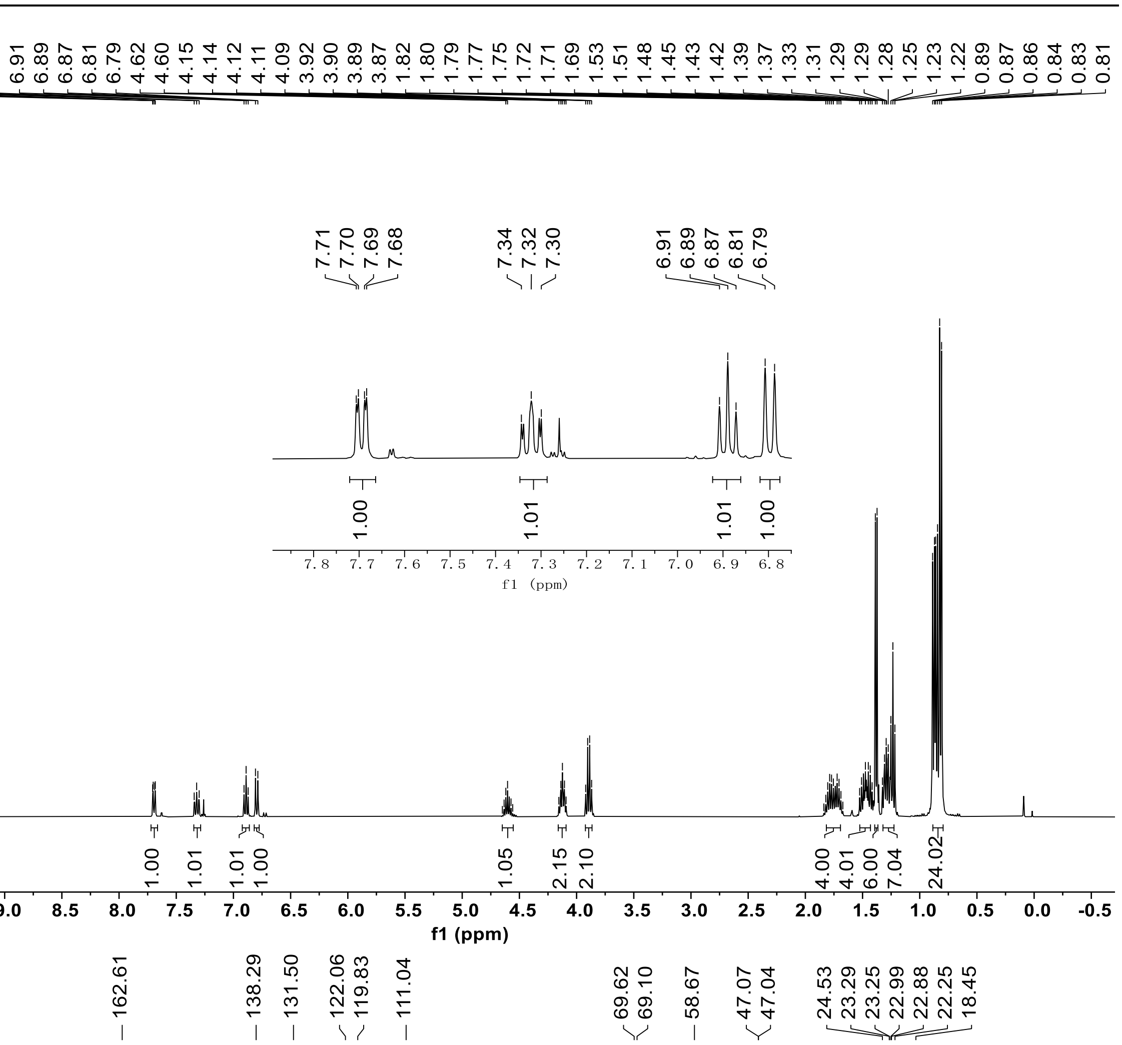

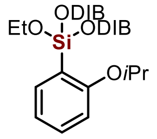

70a, ^{13}C-NMR
(101 MHz, $CDCl_3$)

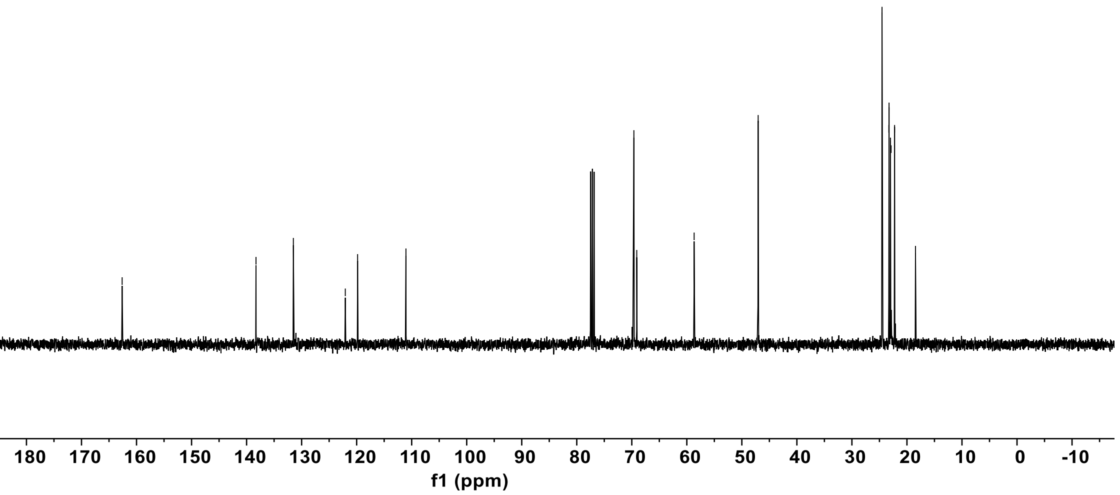

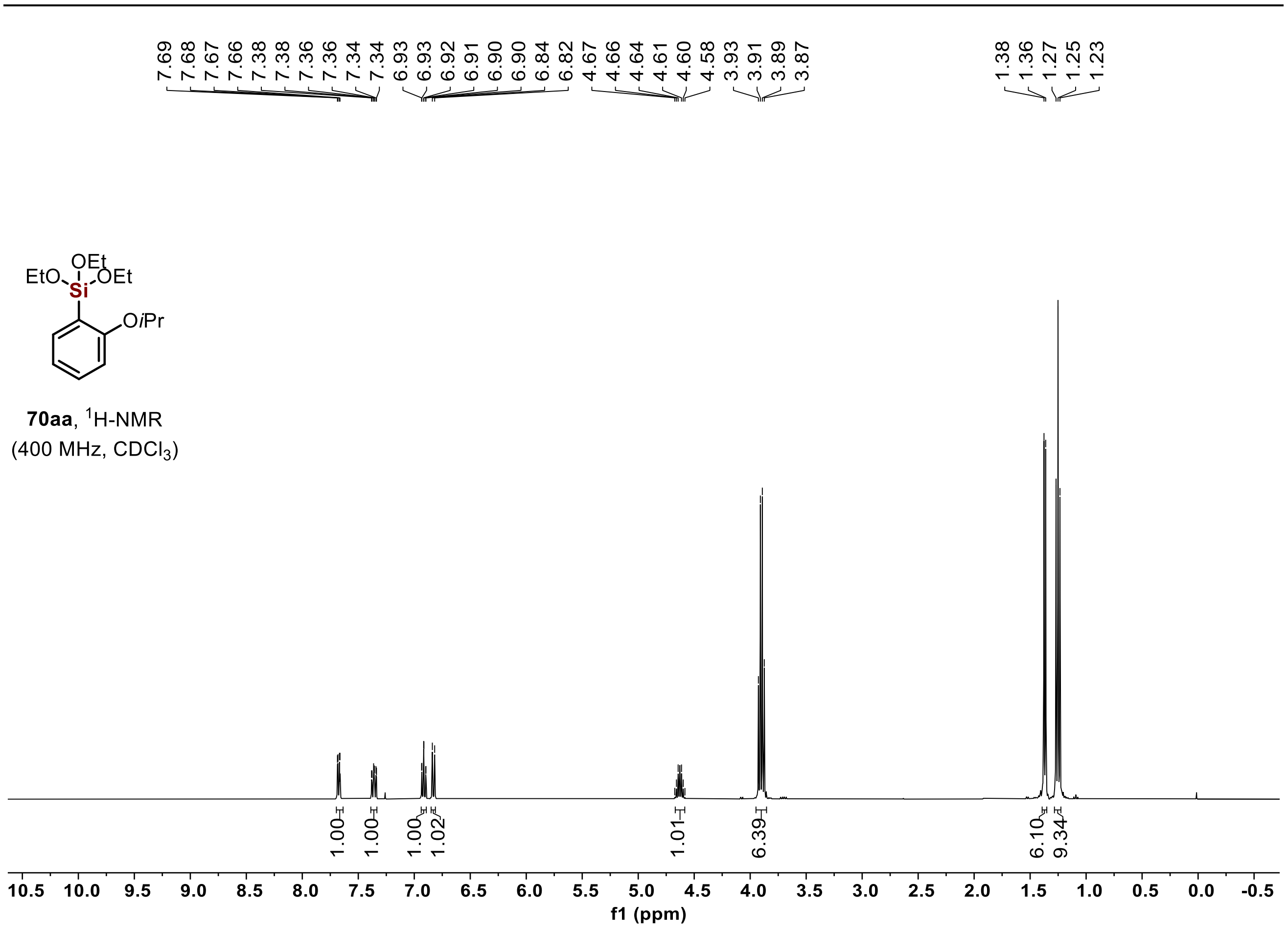

EtO OEt OEt Si OiPr
70aa, 1H-NMR
(400 MHz, CDCl3)
7.69 7.68 7.67 7.66 7.38 7.38 7.36 7.36 7.34 7.34 6.93 6.93 6.92 6.91 6.90 6.90 6.84 6.82 4.67 4.66 4.64 4.61 4.60 4.58 3.93 3.91 3.89 3.87 1.38 1.36 1.27 1.25 1.23
1.00 1.00 1.00 1.02 1.01 6.39 6.10 9.34
f1 (ppm)

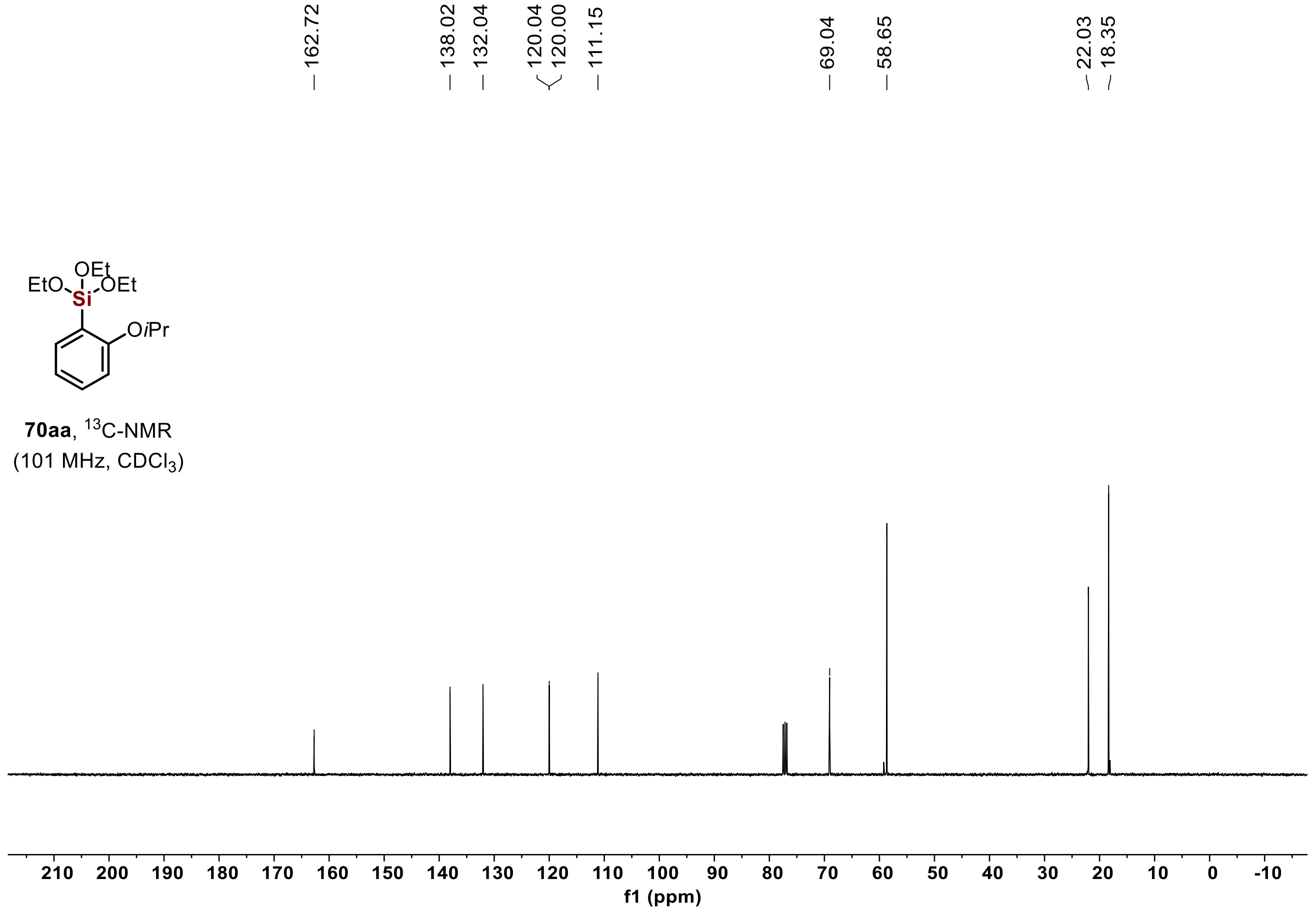

EtO OEt OEt Si OiPr
70aa, 13C-NMR
(101 MHz, CDCl3)
162.72 138.02 132.04 120.04 120.00 111.15 69.04 58.65 22.03 18.35
f1 (ppm)

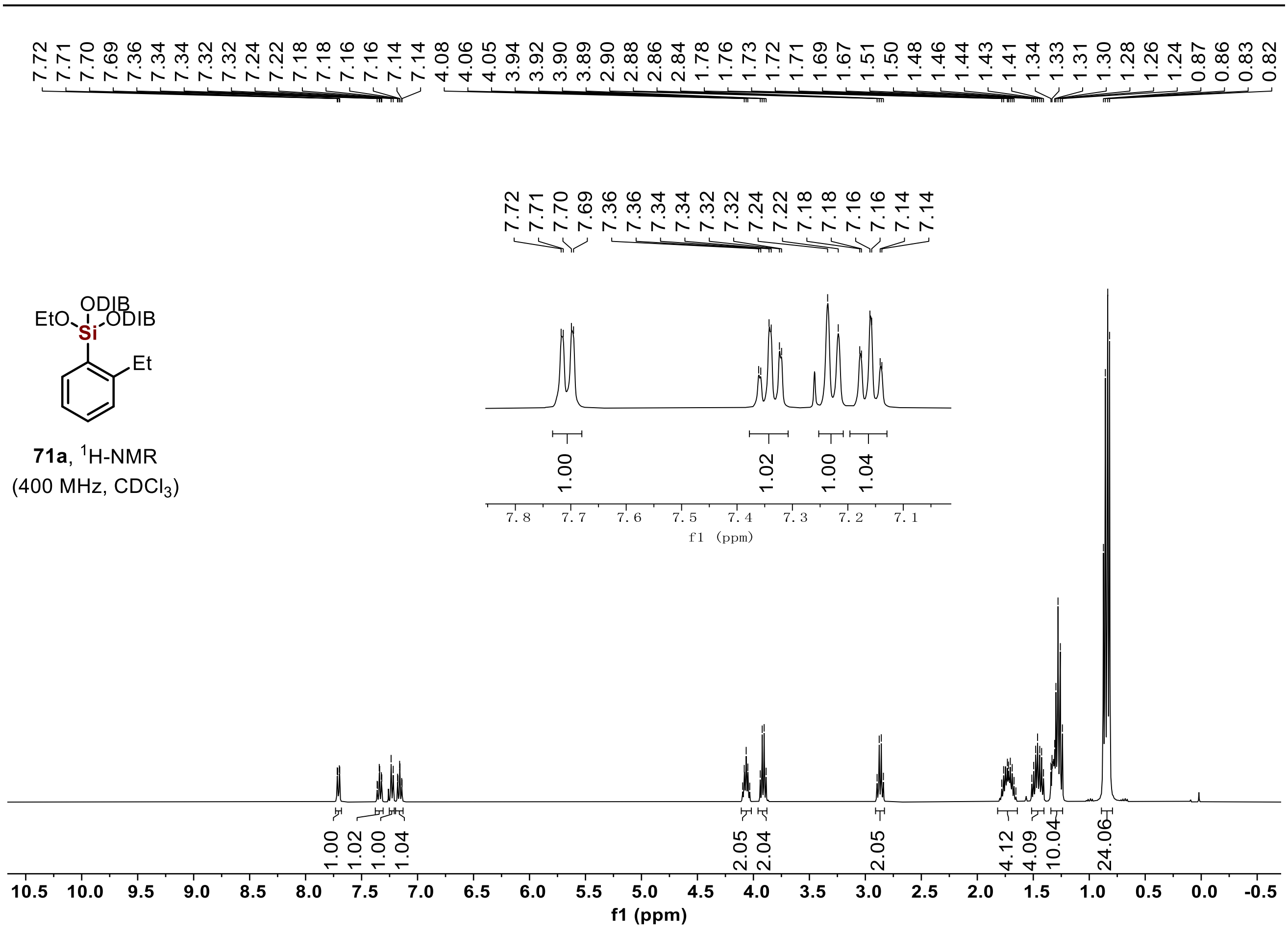

71a, ^{1}H-NMR
(400 MHz, $CDCl_3$)

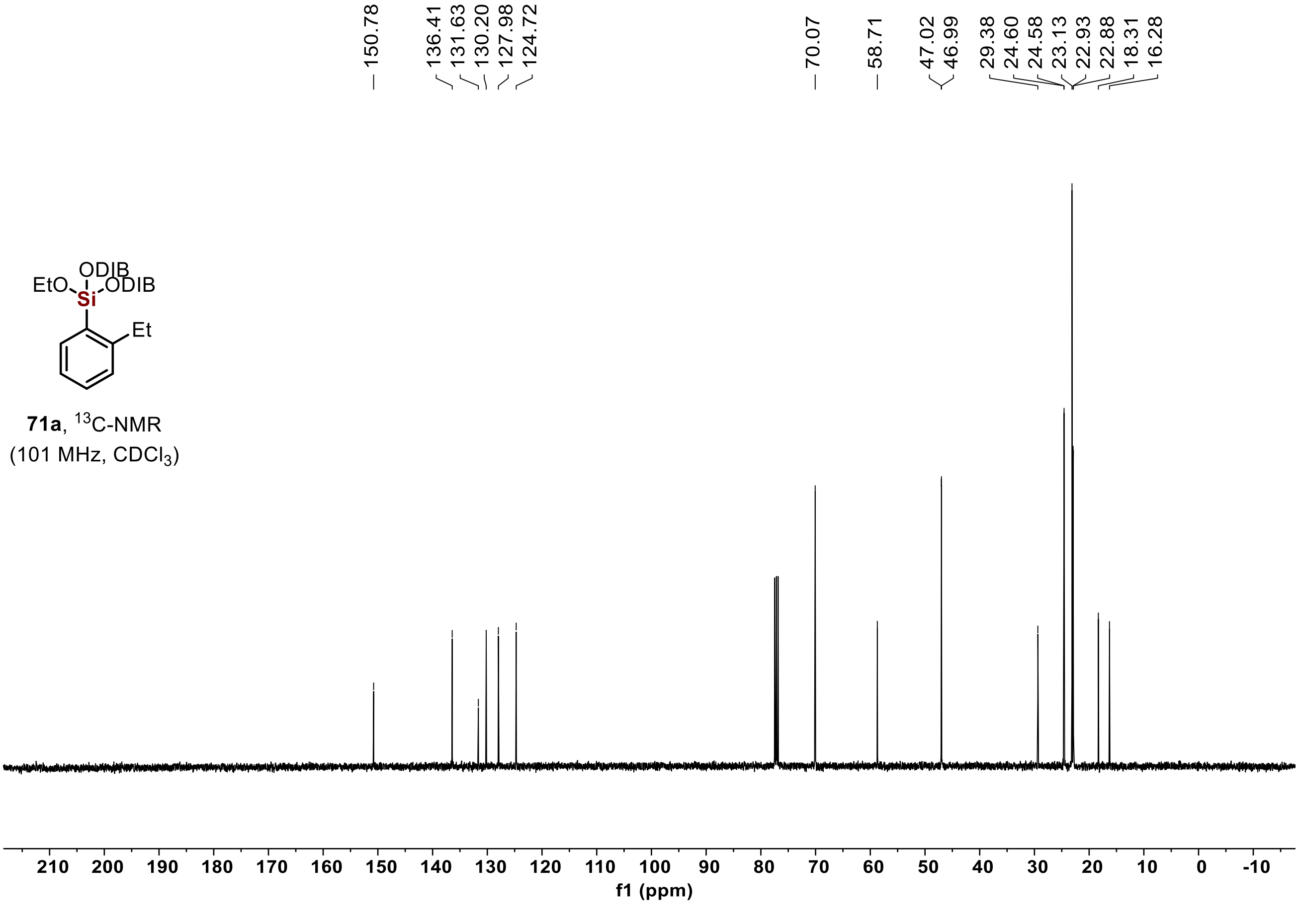

71a, ^{13}C-NMR
(101 MHz, $CDCl_3$)

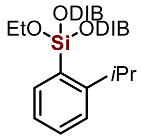

72a, ^{1}H-NMR
(400 MHz, $CDCl_3$)

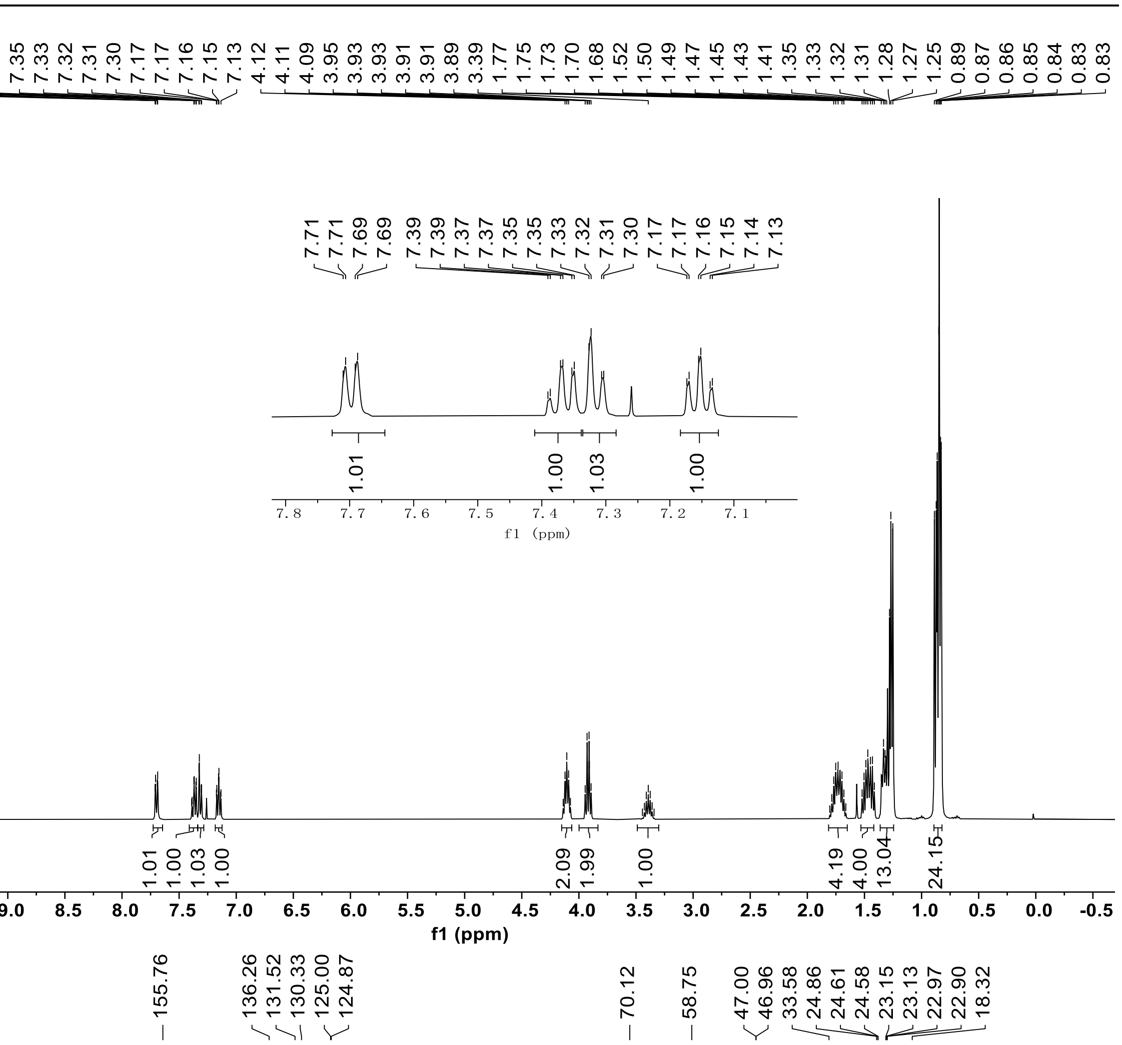

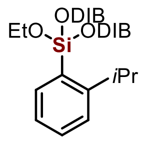

72a, ^{13}C-NMR
(101 MHz, $CDCl_3$)

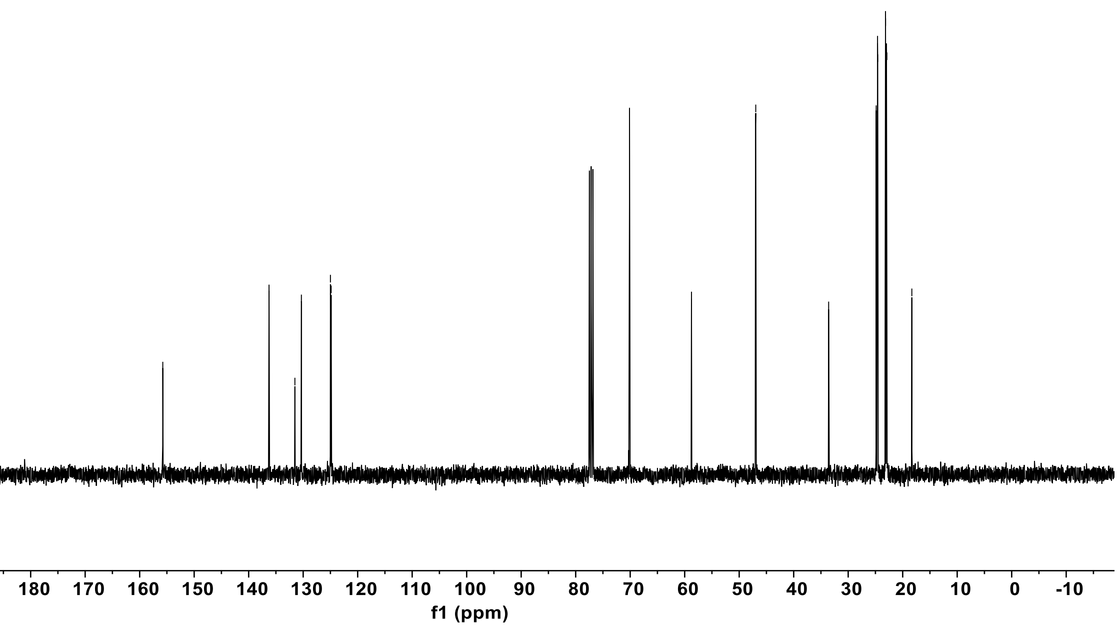

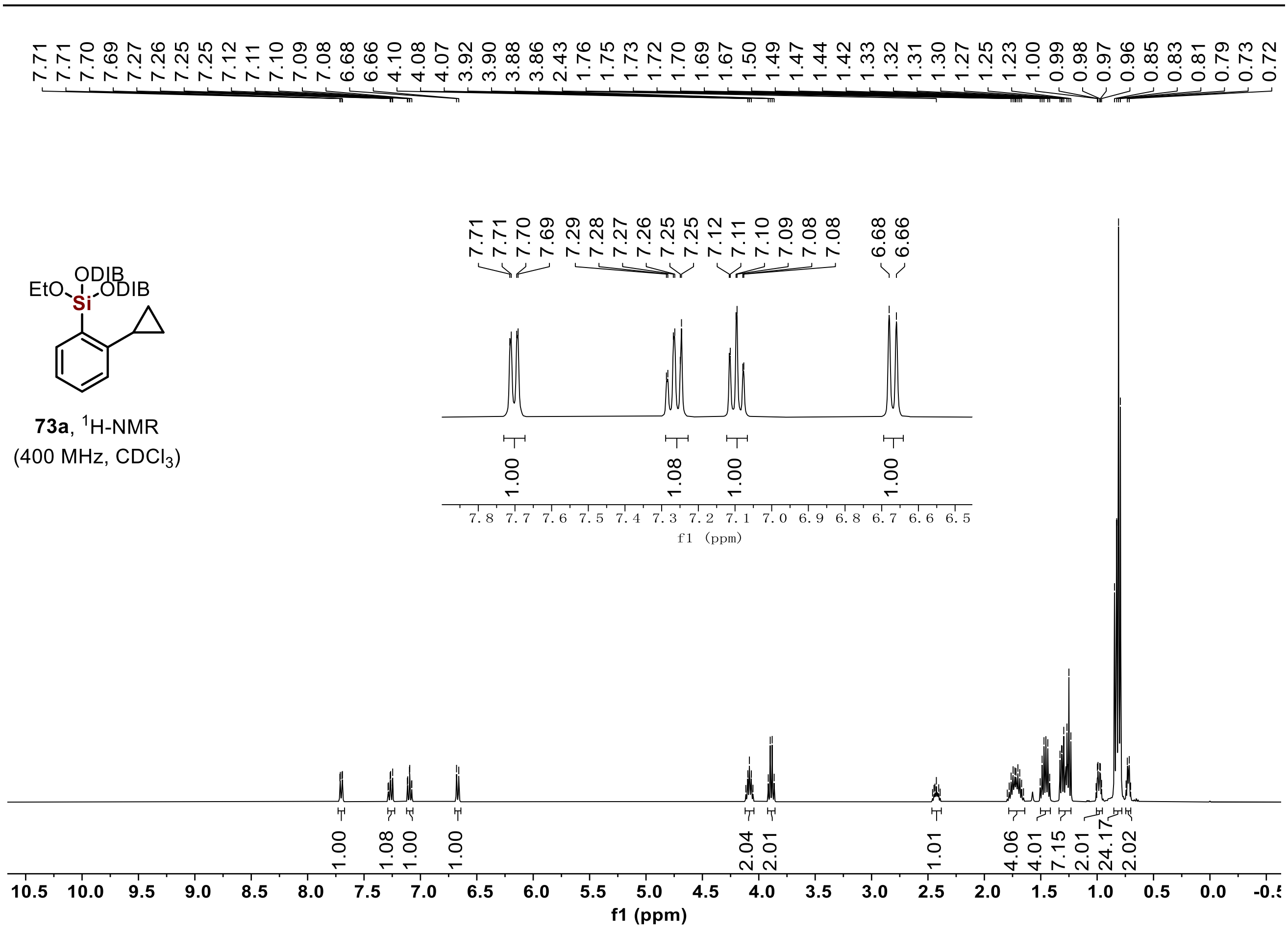
EtO
Si
ODIB
ODIB
73a, ¹H-NMR
(400 MHz, CDCl3)
f1 (ppm)

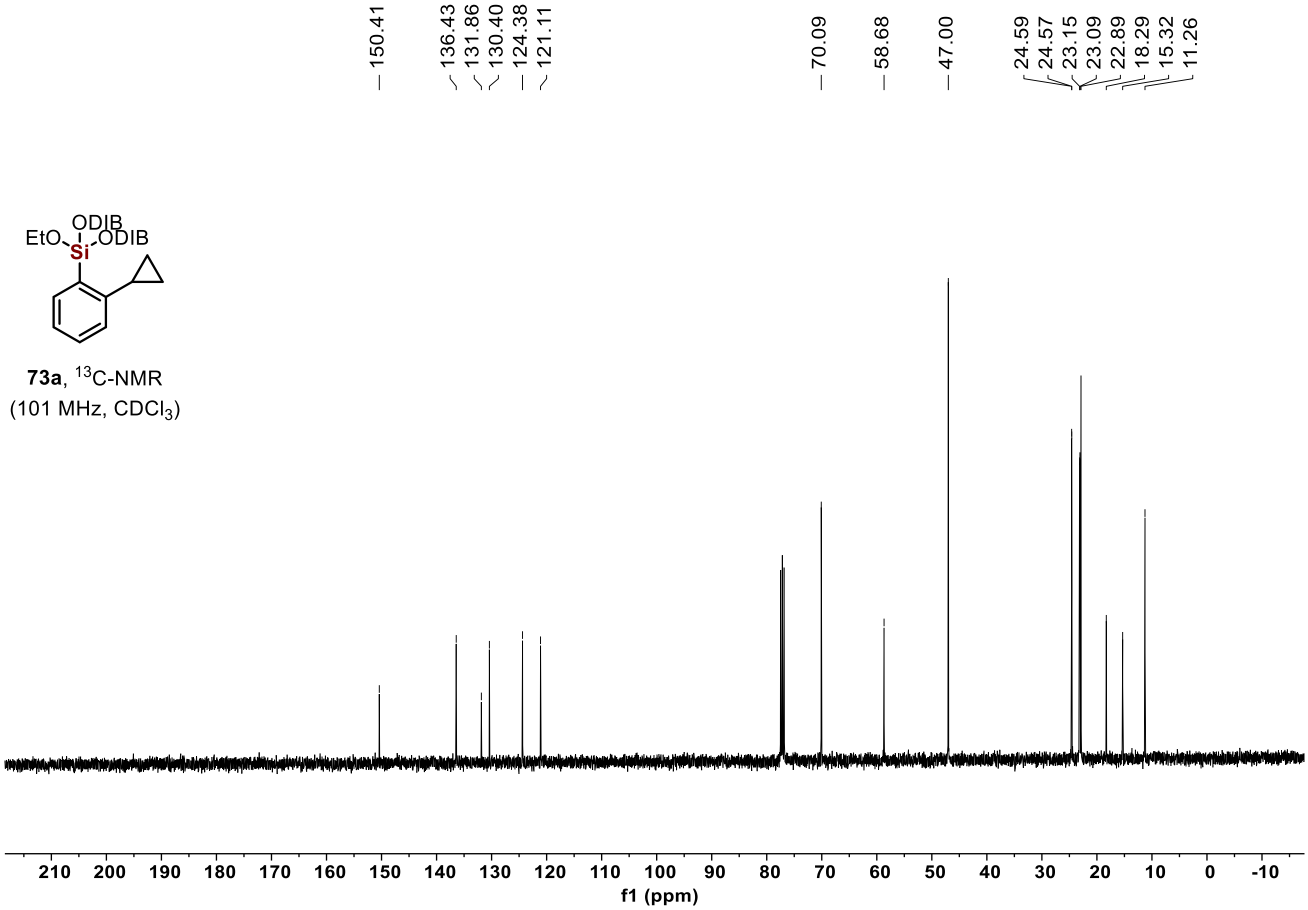
EtO
Si
ODIB
ODIB
73a, ¹³C-NMR
(101 MHz, CDCl3)
150.41
136.43
131.86
130.40
124.38
121.11
70.09
58.68
47.00
24.59
24.57
23.15
23.09
22.89
18.29
15.32
11.26
f1 (ppm)

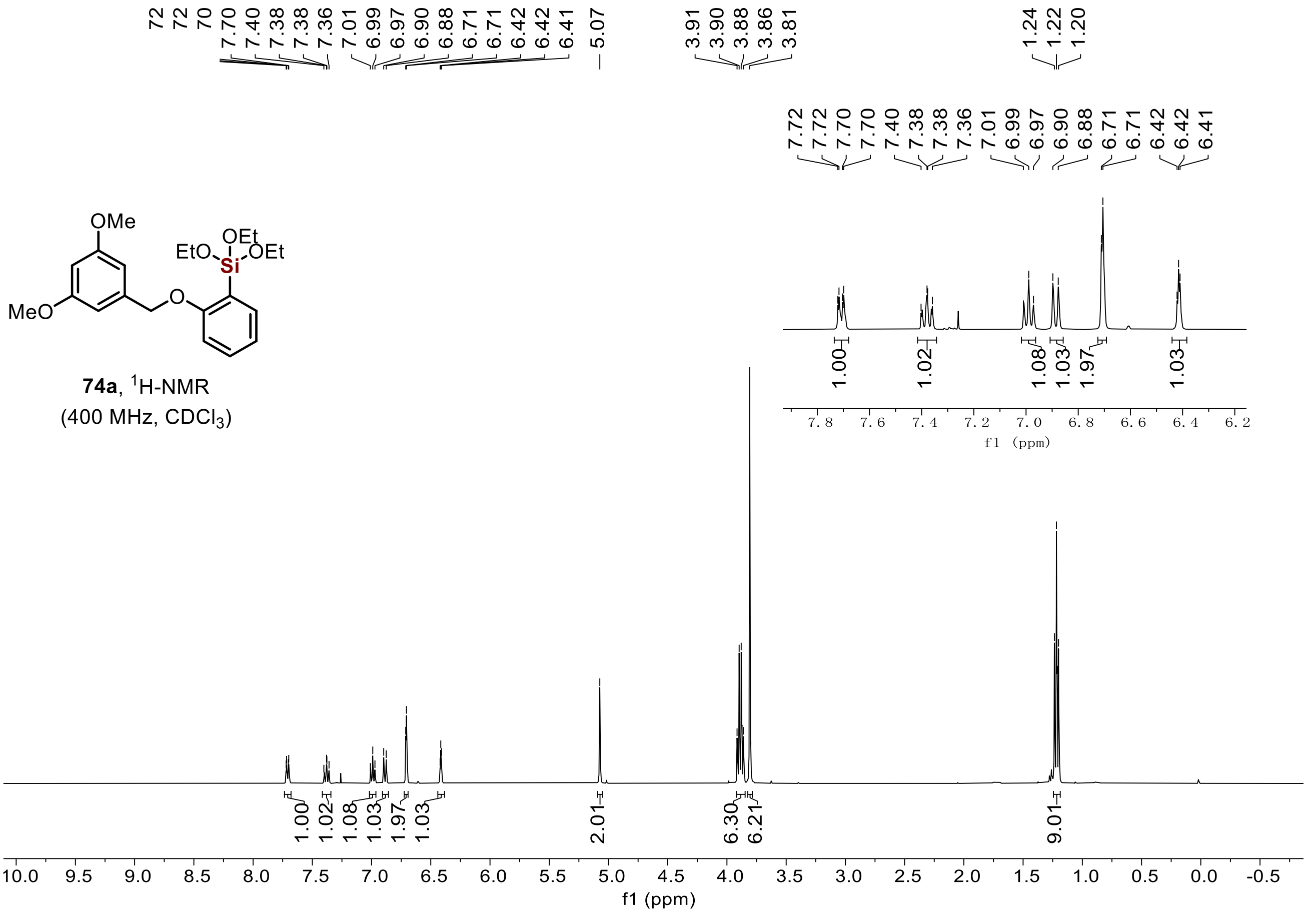

74a, ^{1}H-NMR
(400 MHz, $CDCl_3$)

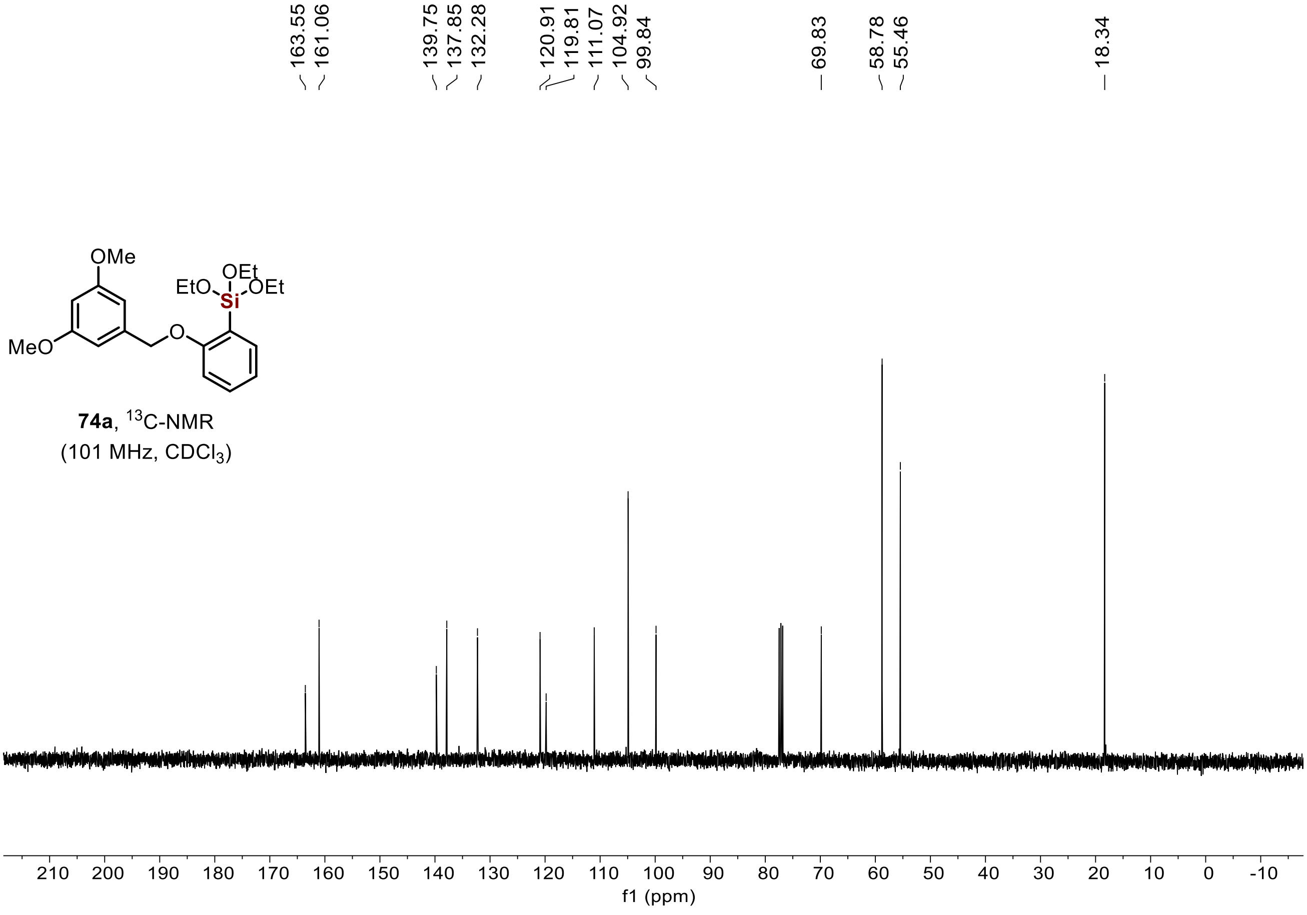

74a, ^{13}C-NMR
(101 MHz, $CDCl_3$)

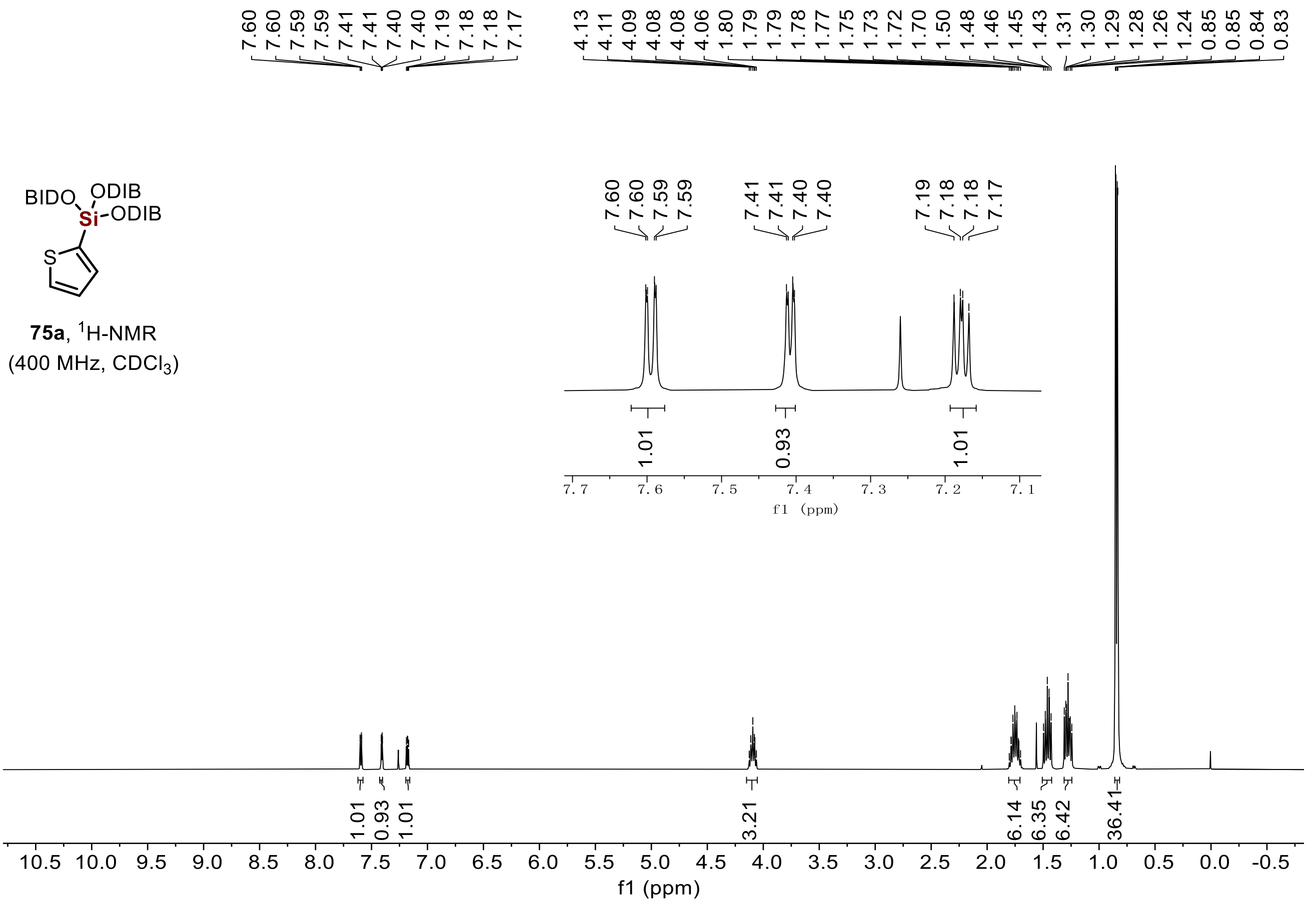

7.60 7.60 7.59 7.59 7.41 7.41 7.40 7.40 7.19 7.18 7.18 7.17
4.13 4.11 4.09 4.08 4.08 4.06 1.80 1.79 1.79 1.78 1.77 1.75 1.73 1.72 1.70 1.50 1.48 1.46 1.45 1.43 1.31 1.30 1.29 1.28 1.26 1.24 0.85 0.85 0.84 0.83
BIDO ODIB Si ODIB S
75a, ^{1}H-NMR
(400 MHz, $CDCl_3$)
1.01 0.93 1.01
1.01 0.93 1.01 3.21 6.14 6.35 6.42 36.41
f1 (ppm)

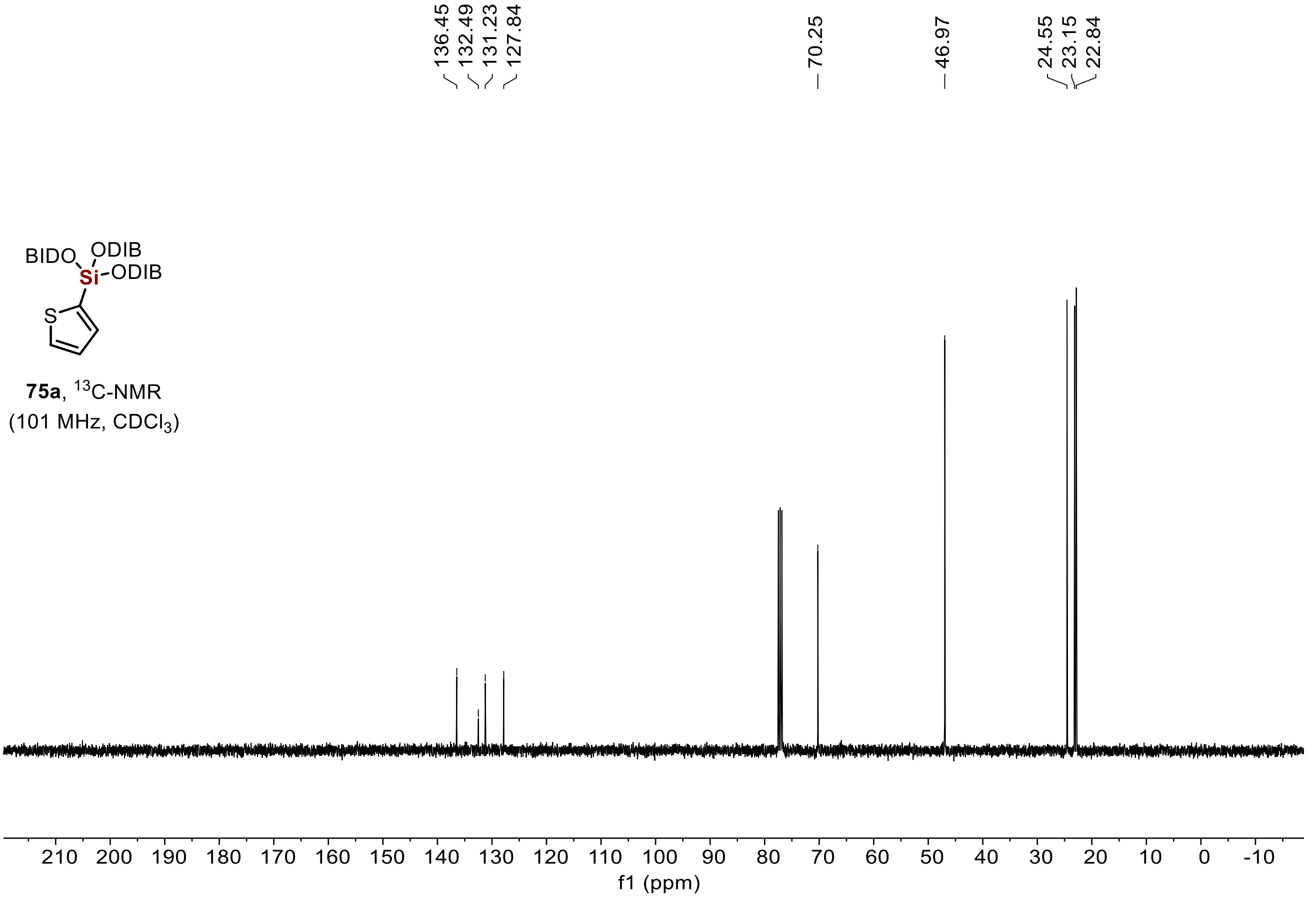

136.45 132.49 131.23 127.84 70.25 46.97 24.55 23.15 22.84
BIDO ODIB Si ODIB S
75a, ^{13}C-NMR
(101 MHz, $CDCl_3$)
f1 (ppm)

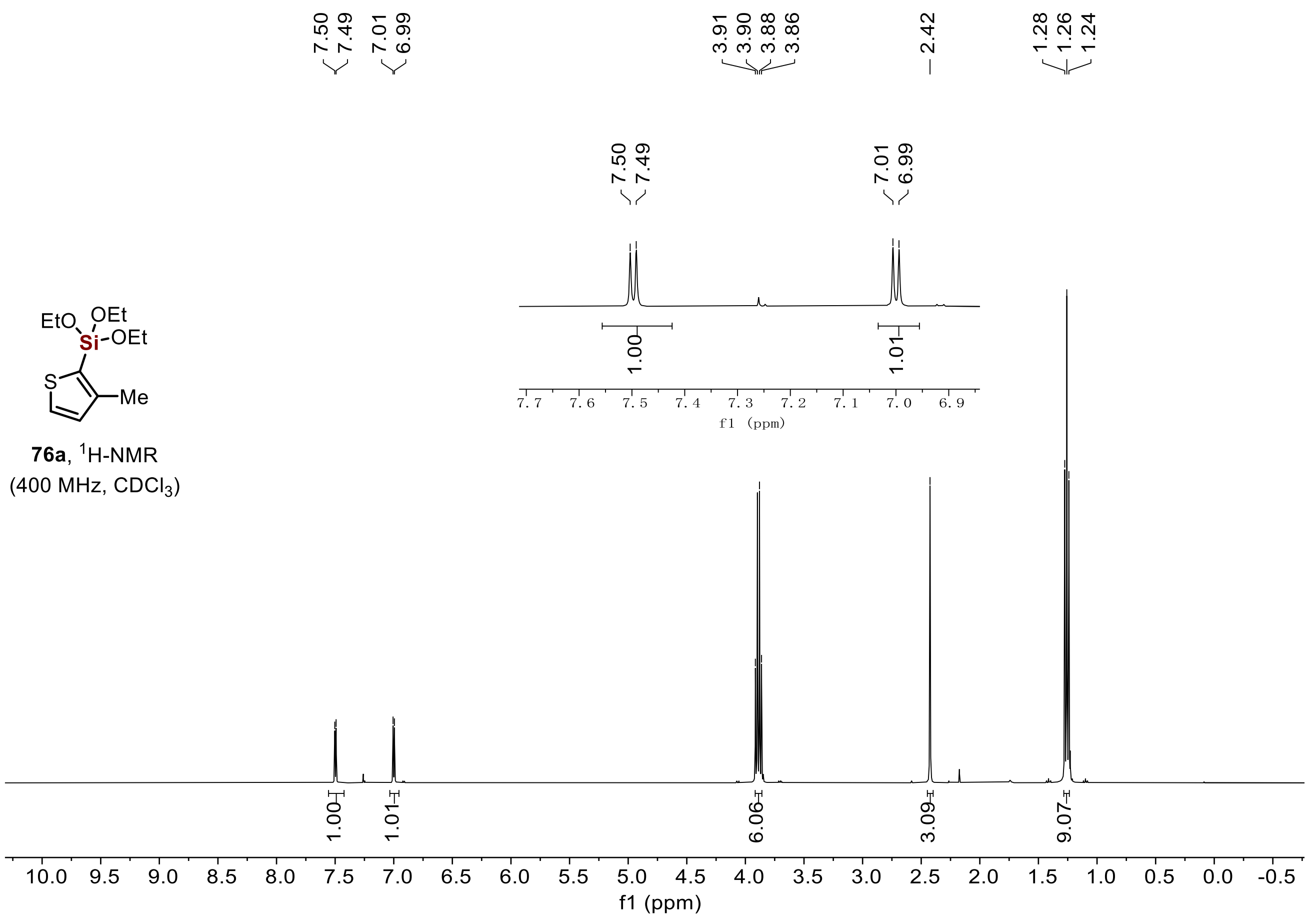
7.50
7.49
7.01
6.99
3.91
3.90
3.88
3.86
2.42
1.28
1.26
1.24
EtO OEt Si OEt S Me
76a, 1H-NMR
(400 MHz, CDCl3)
1.00
1.01
6.06
3.09
9.07
f1 (ppm)

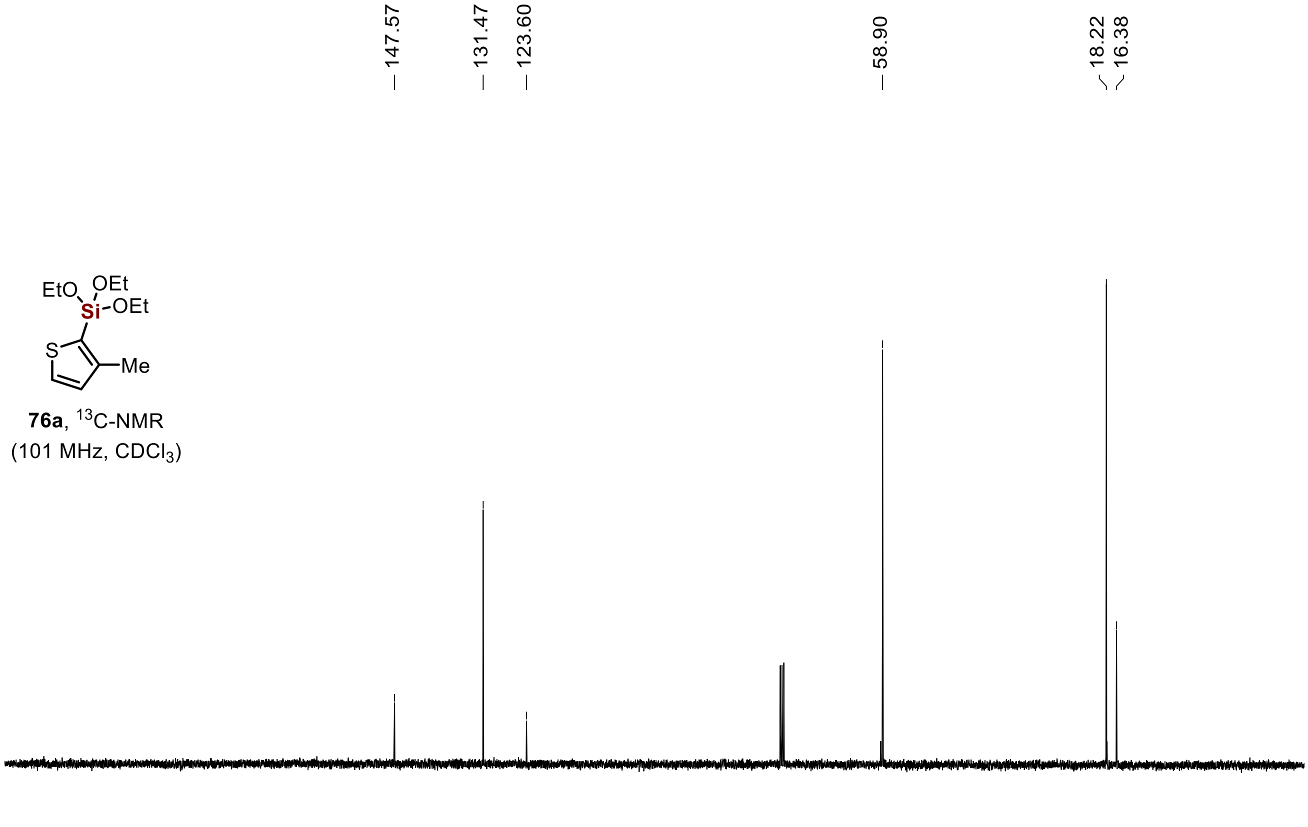
147.57
131.47
123.60
58.90
18.22
16.38
EtO OEt Si OEt S Me
76a, 13C-NMR
(101 MHz, CDCl3)
f1 (ppm)

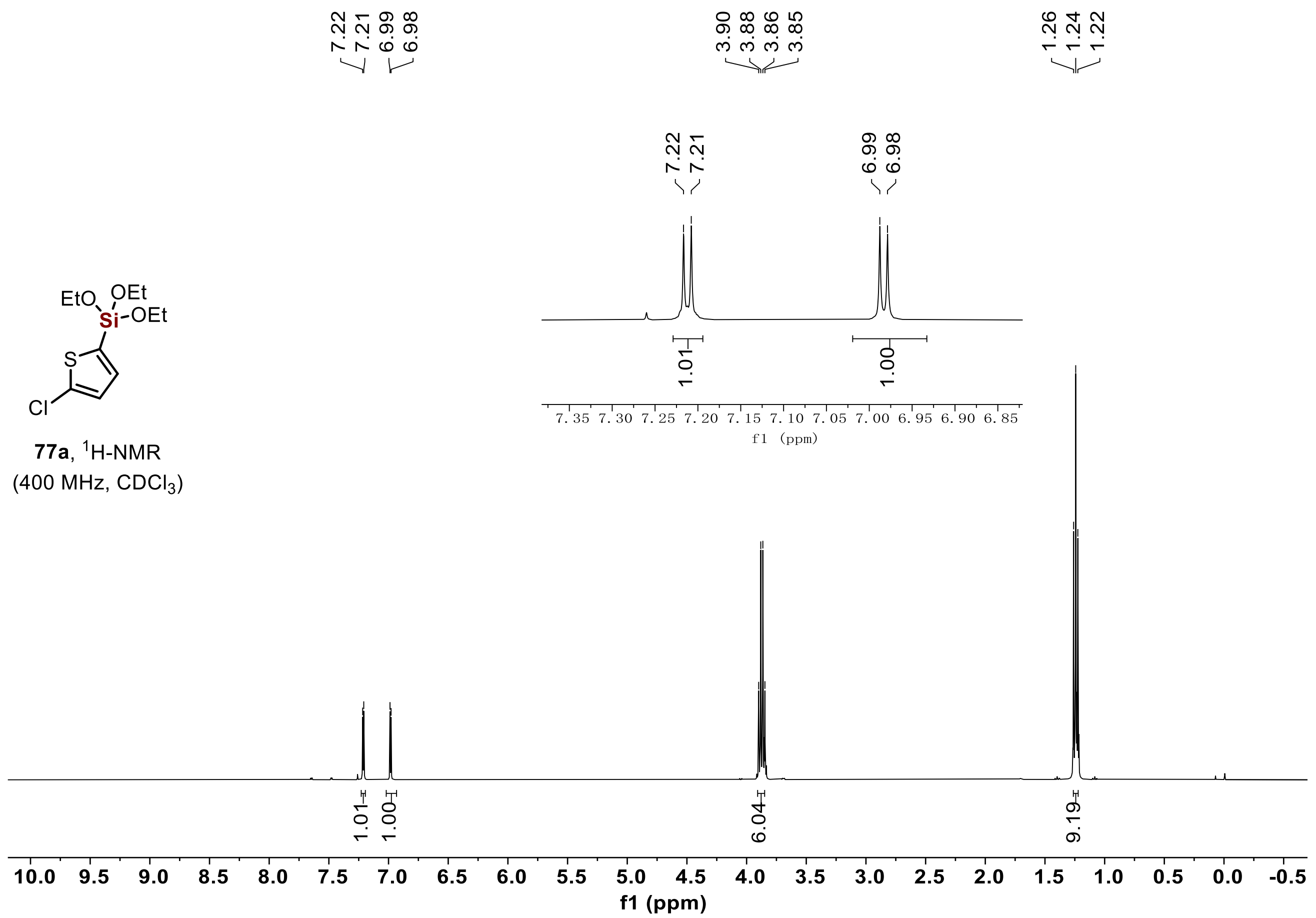

77a, ^{1}H-NMR (400 MHz, $CDCl_3$)

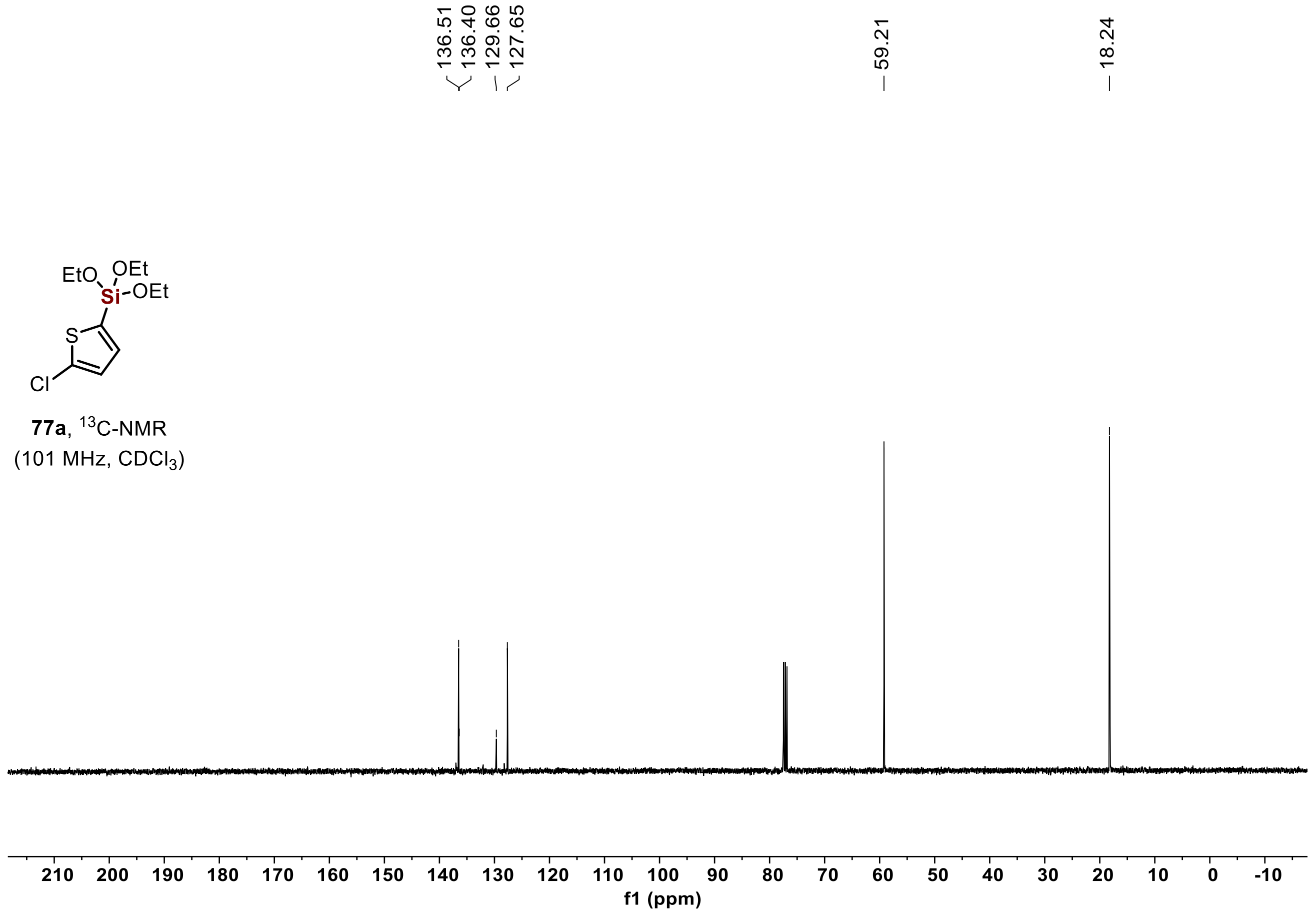

77a, ^{13}C-NMR (101 MHz, $CDCl_3$)

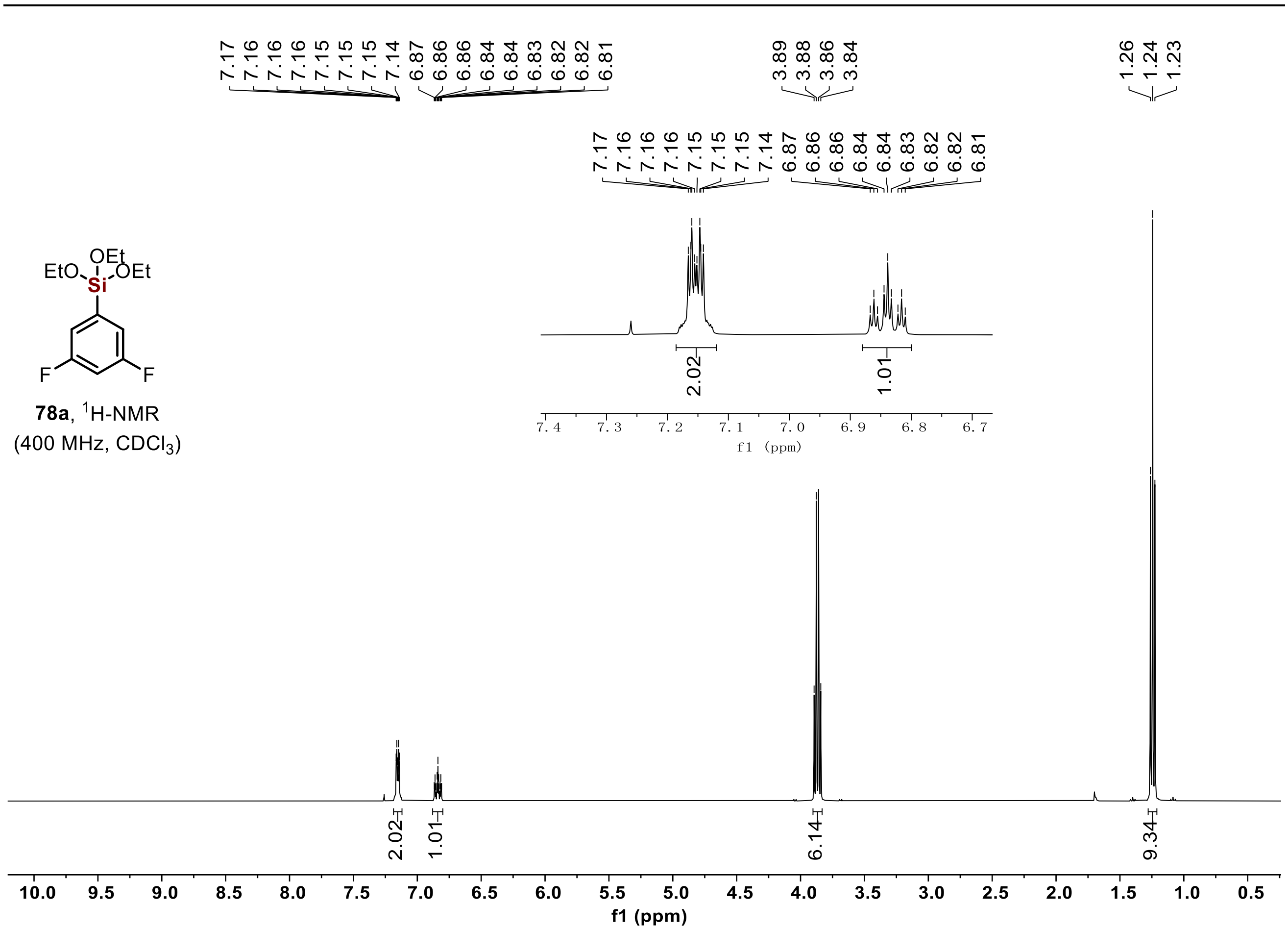
7.17 7.16 7.16 7.16 7.15 7.15 7.15 7.14 6.87 6.86 6.86 6.84 6.84 6.83 6.82 6.82 6.81
3.89 3.88 3.86 3.84
1.26 1.24 1.23
EtO OEt OEt Si
F F
78a, ^{1}H-NMR
(400 MHz, $CDCl_3$)
2.02
1.01
6.14
9.34
f1 (ppm)

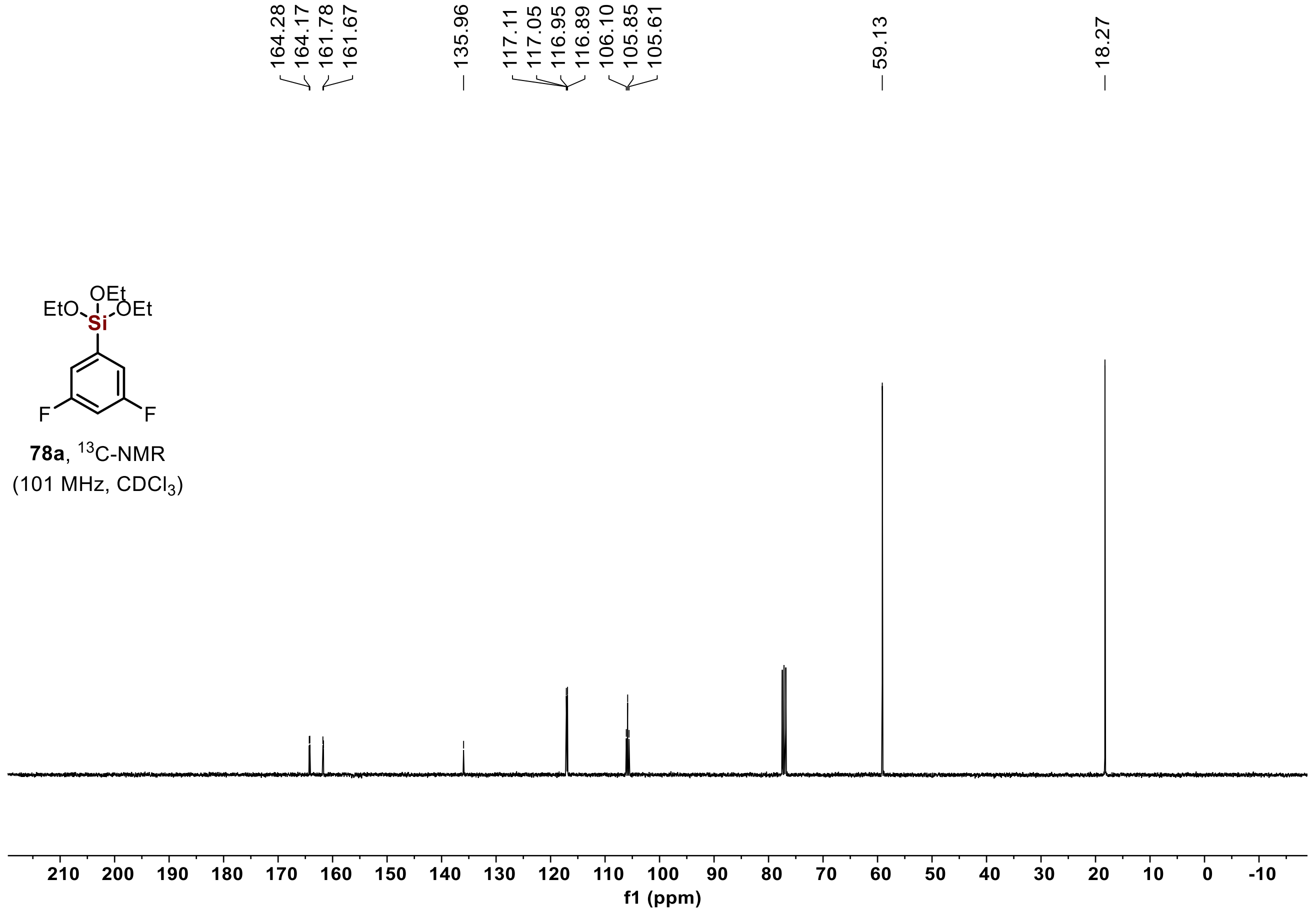
164.28 164.17 161.78 161.67
135.96
117.11 117.05 116.95 116.89 106.10 105.85 105.61
59.13
18.27
EtO OEt OEt Si
F F
78a, ^{13}C-NMR
(101 MHz, $CDCl_3$)
f1 (ppm)

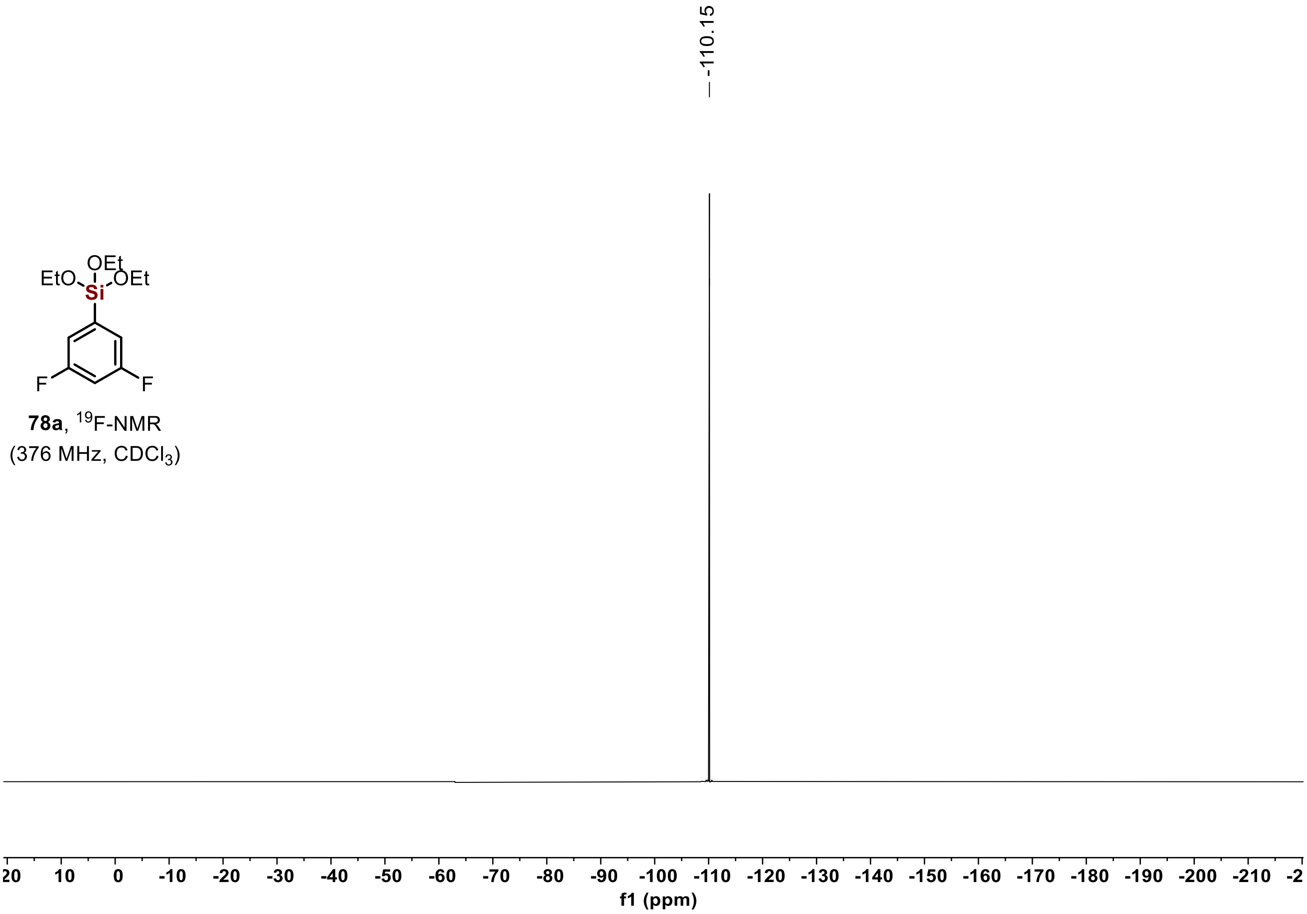

78a, ^{19}F-NMR
(376 MHz, $CDCl_3$)

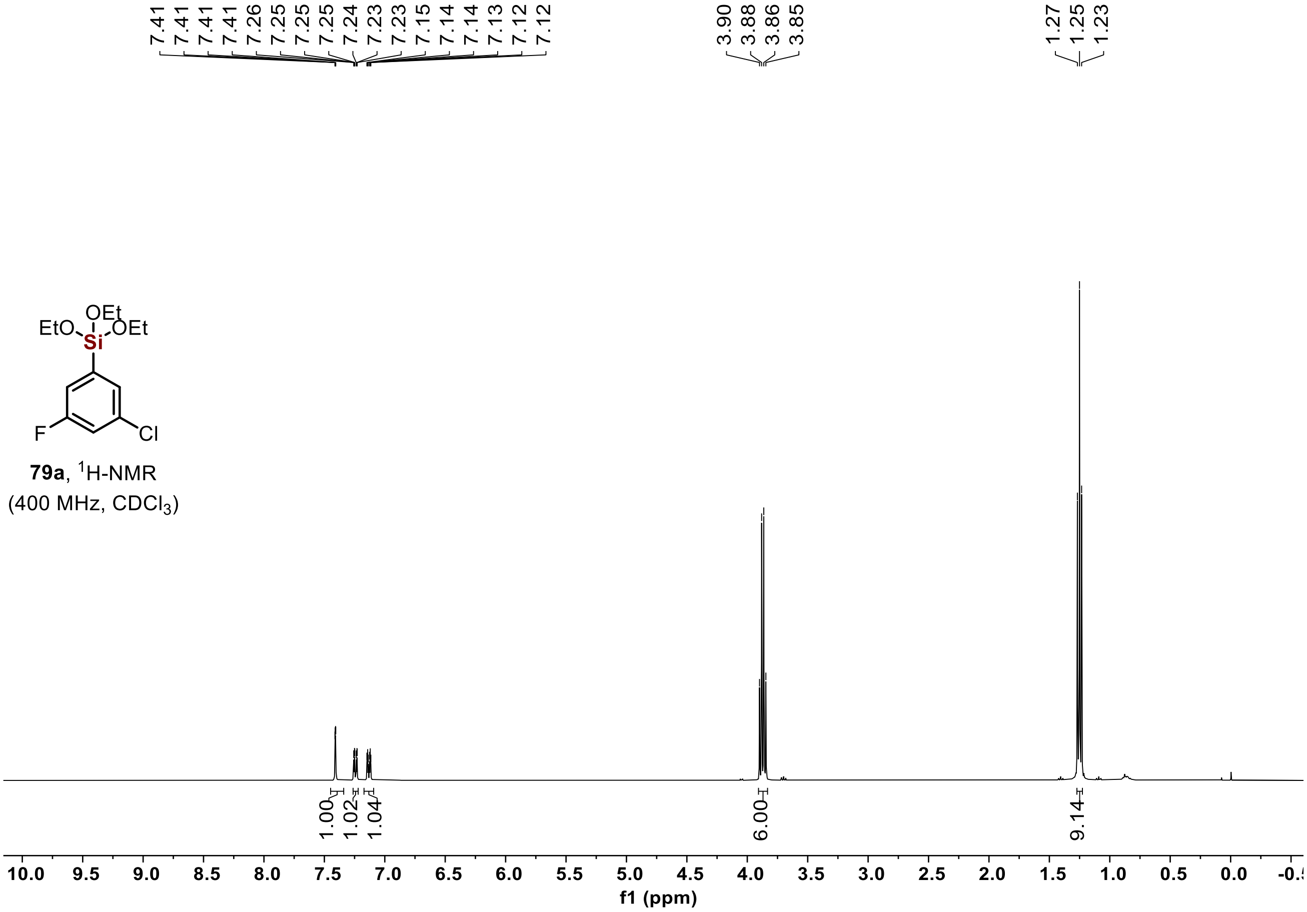

79a, ^{1}H-NMR
(400 MHz, $CDCl_3$)

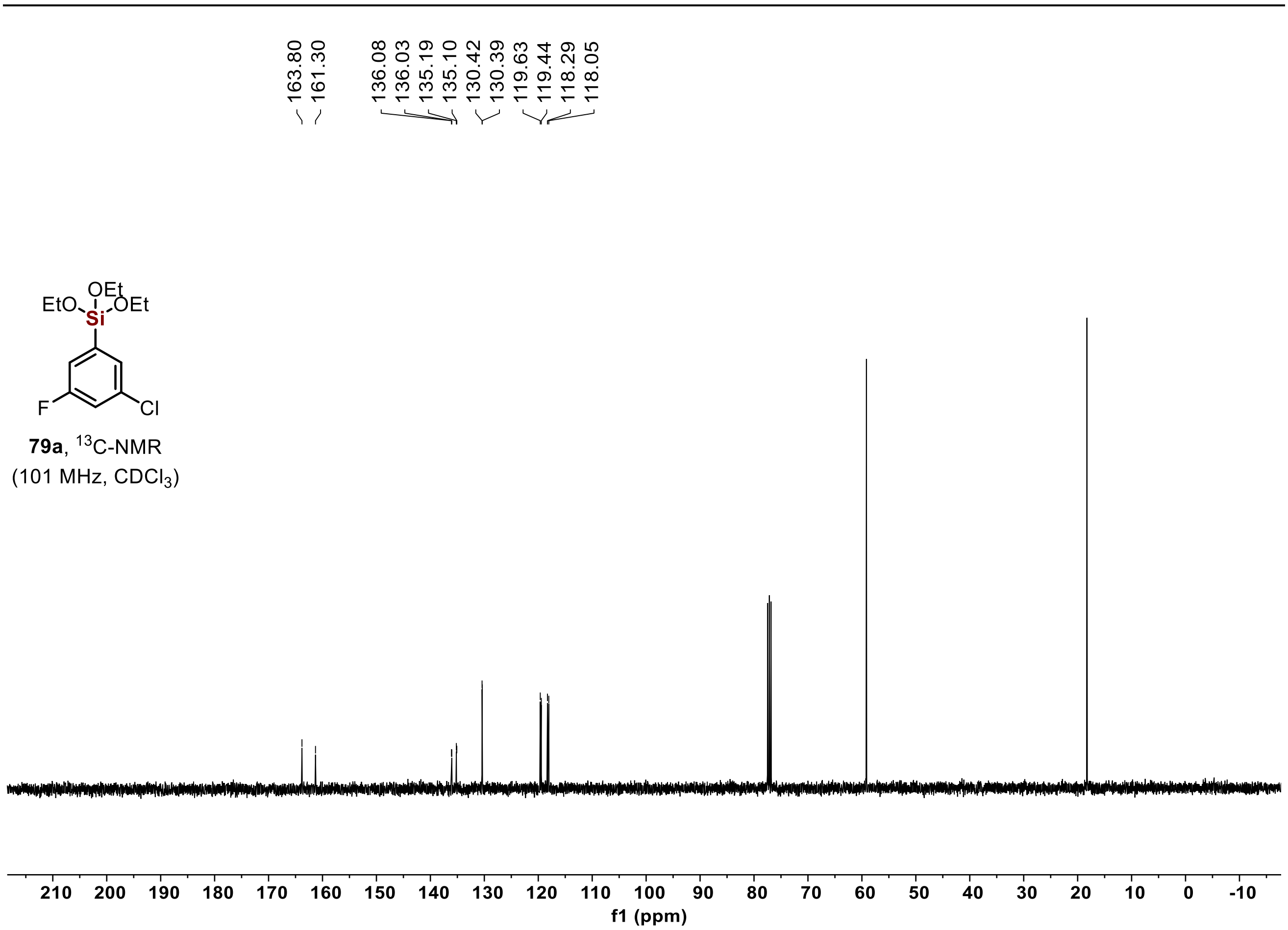

79a, ^{13}C-NMR
(101 MHz, $CDCl_3$)

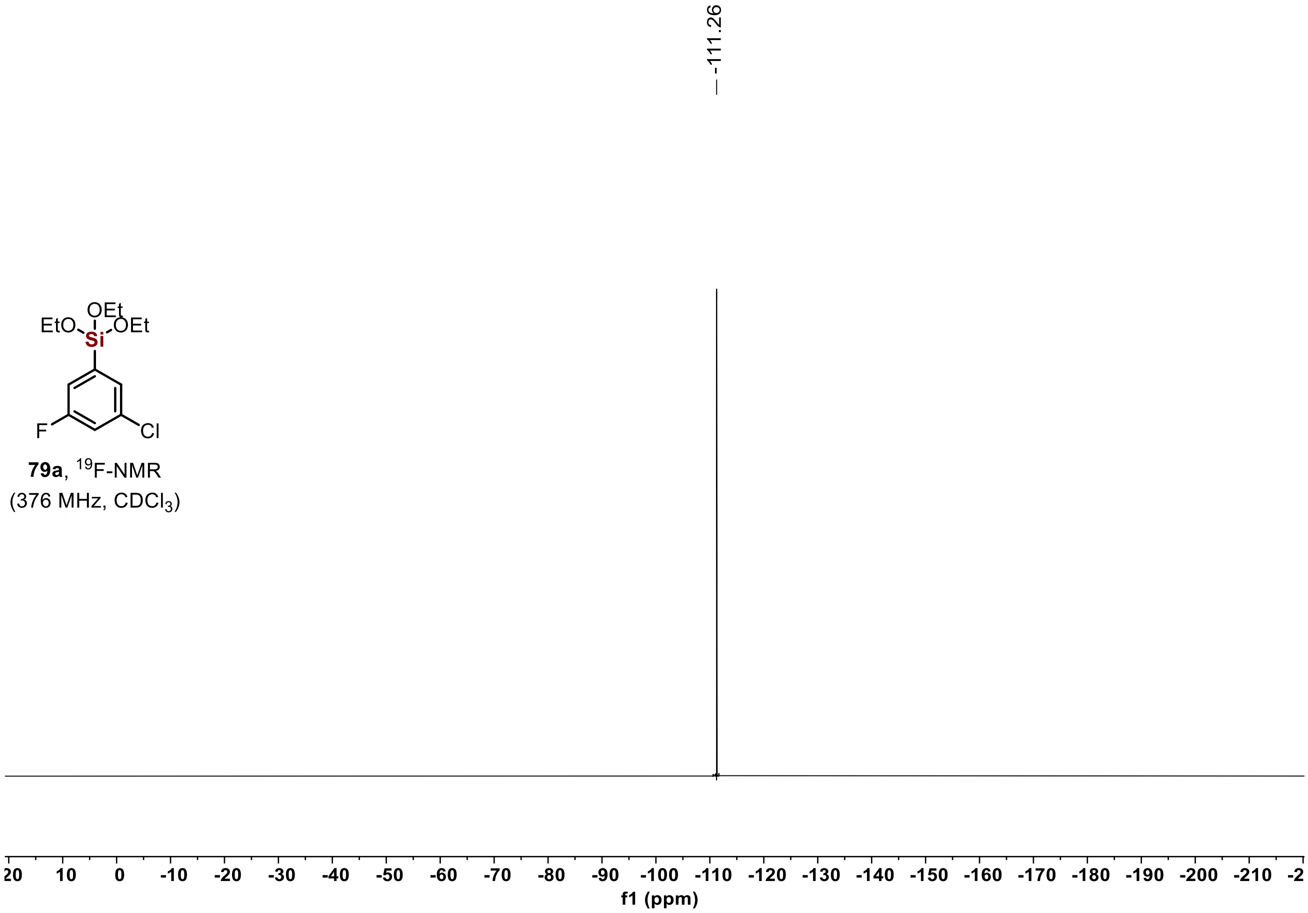

79a, ^{19}F-NMR
(376 MHz, $CDCl_3$)

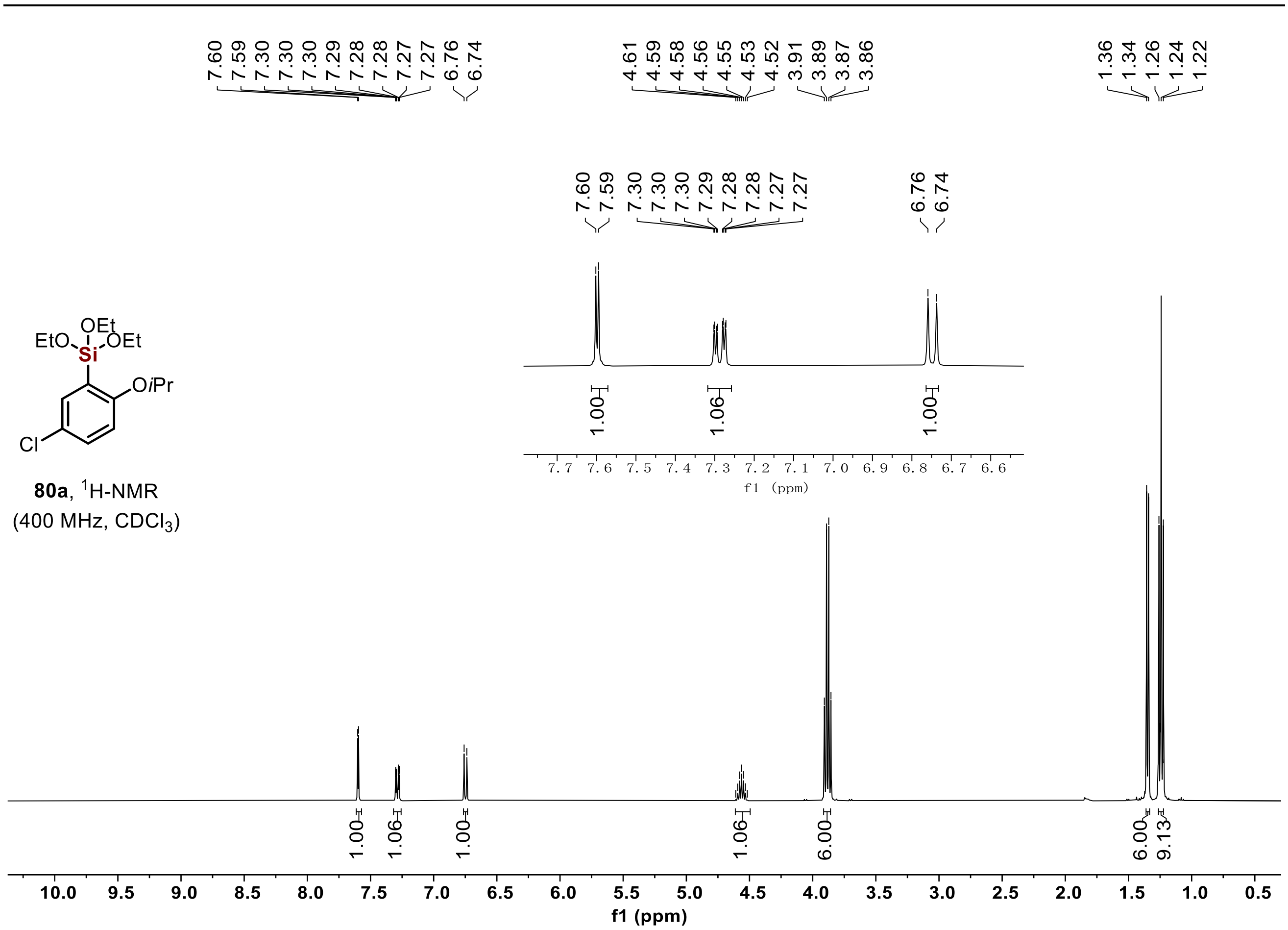

80a, ^{1}H-NMR
(400 MHz, $CDCl_3$)

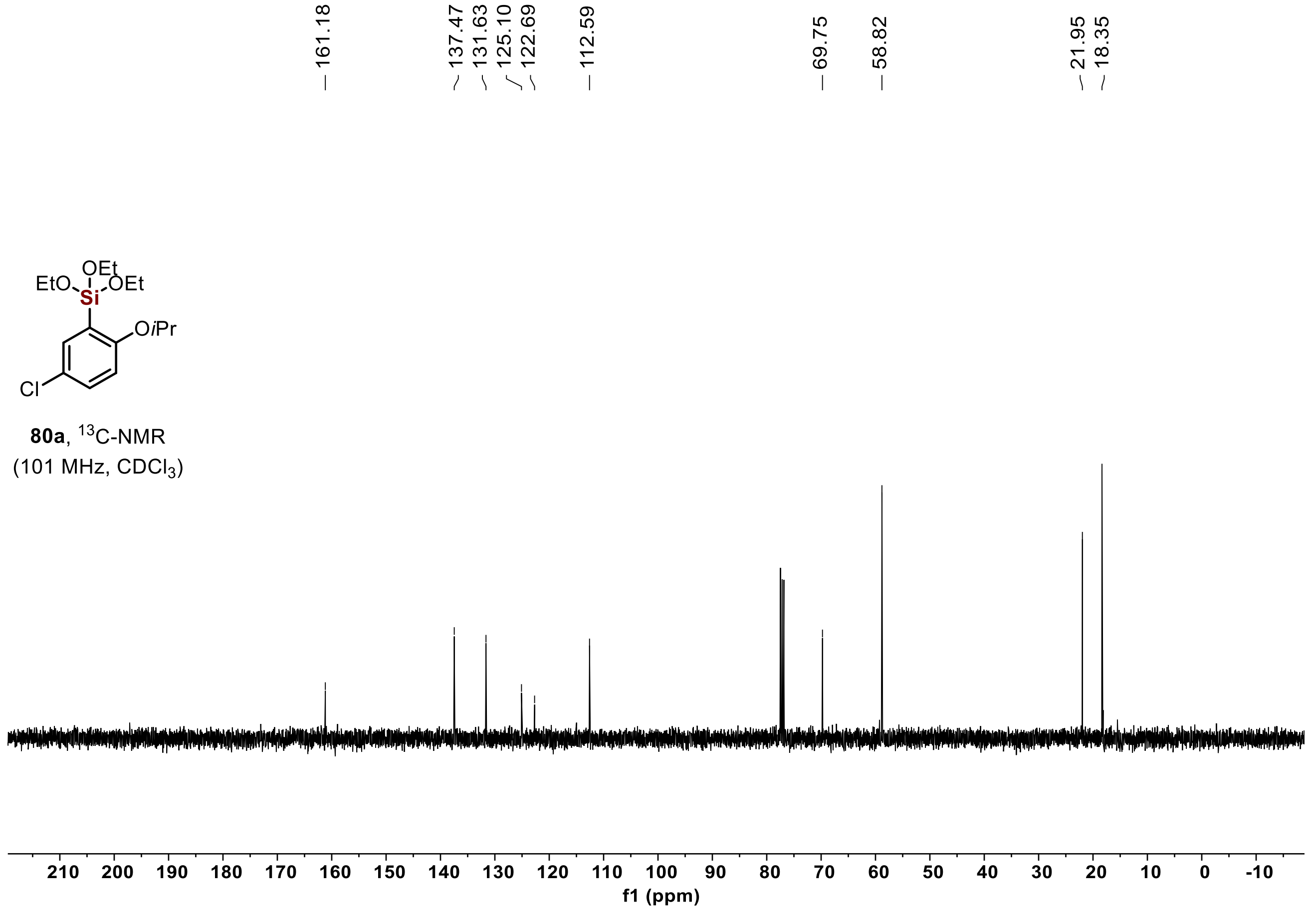

80a, ^{13}C-NMR
(101 MHz, $CDCl_3$)

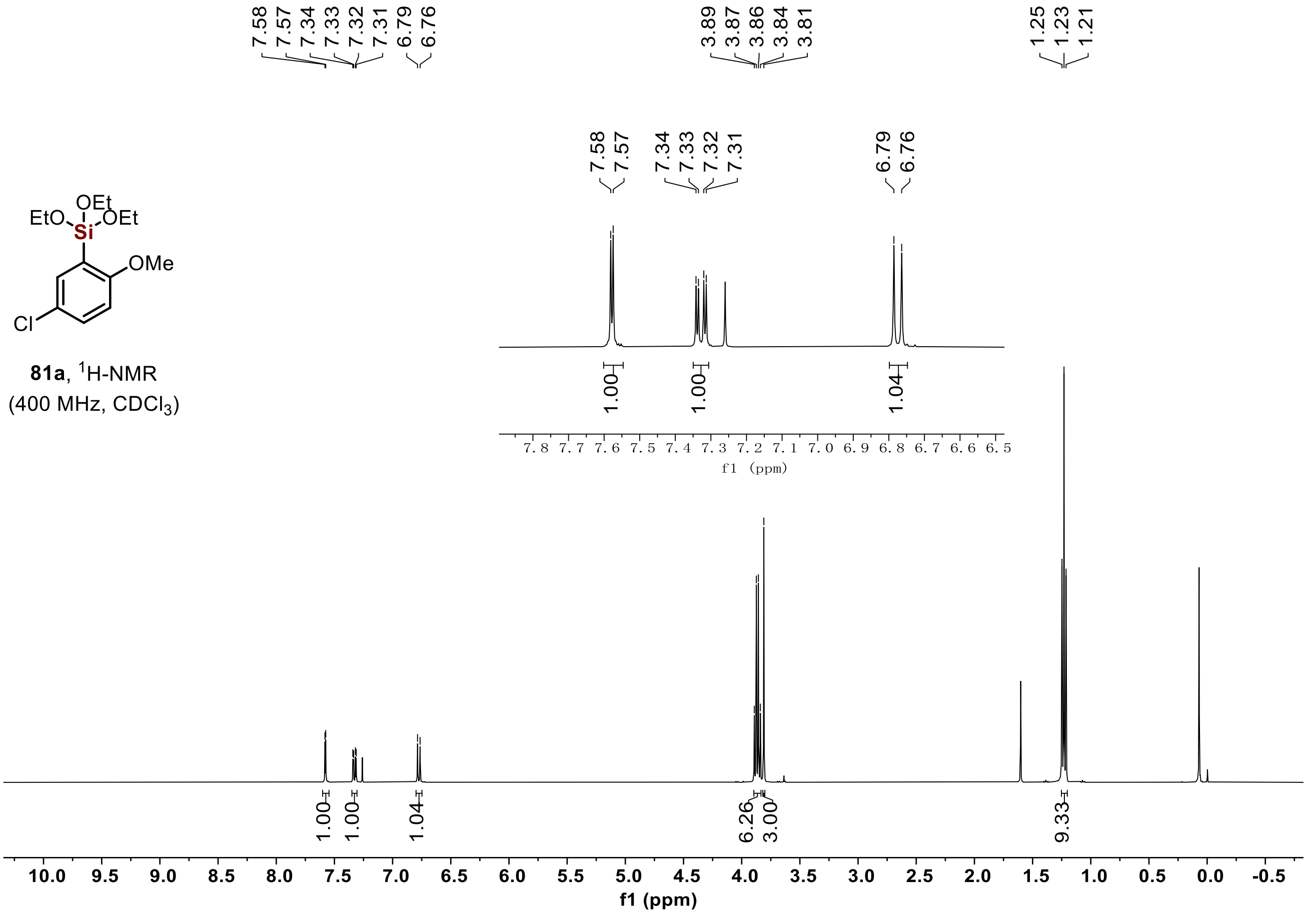

81a, ^{1}H-NMR
(400 MHz, $CDCl_3$)

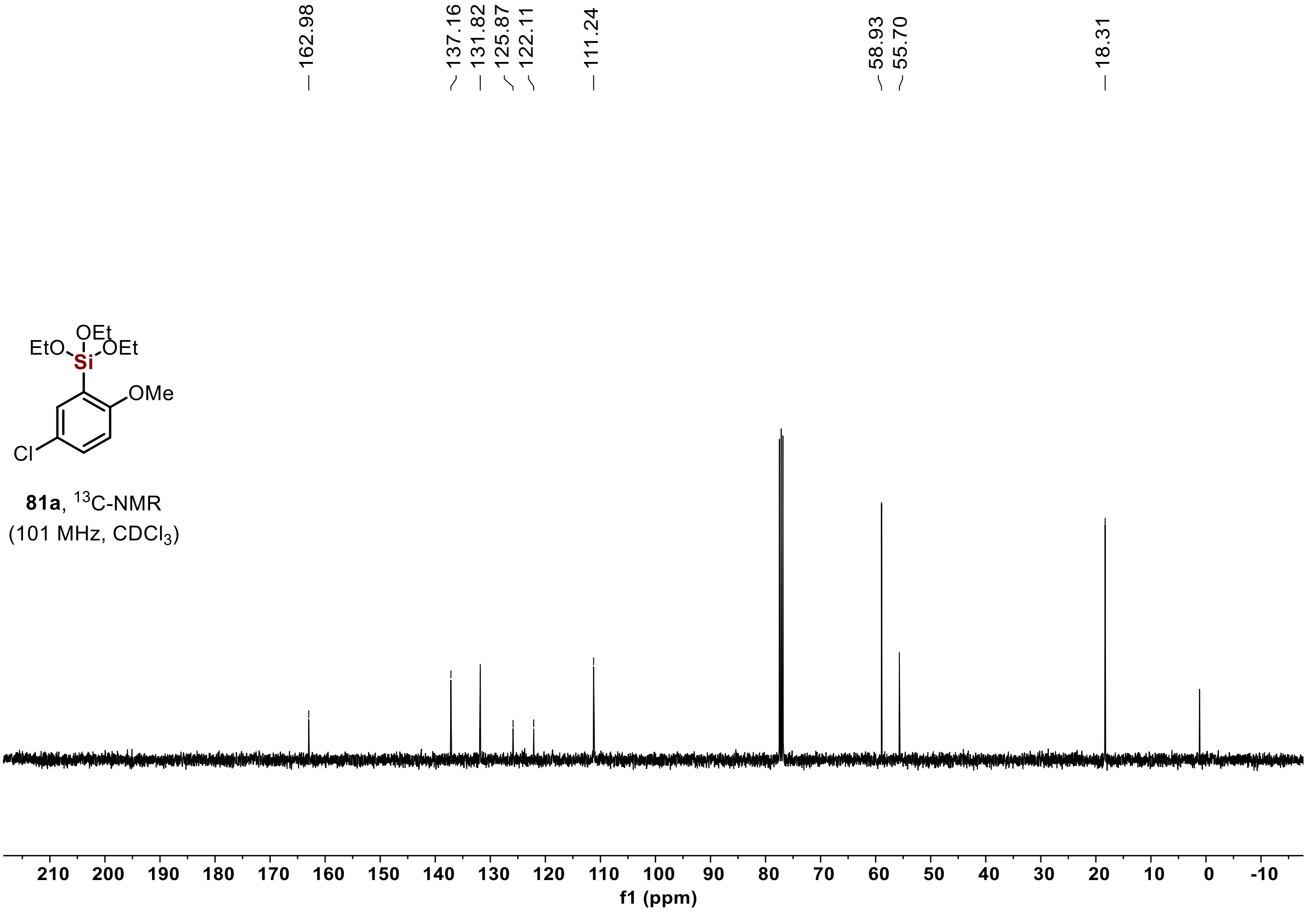

81a, ^{13}C-NMR
(101 MHz, $CDCl_3$)

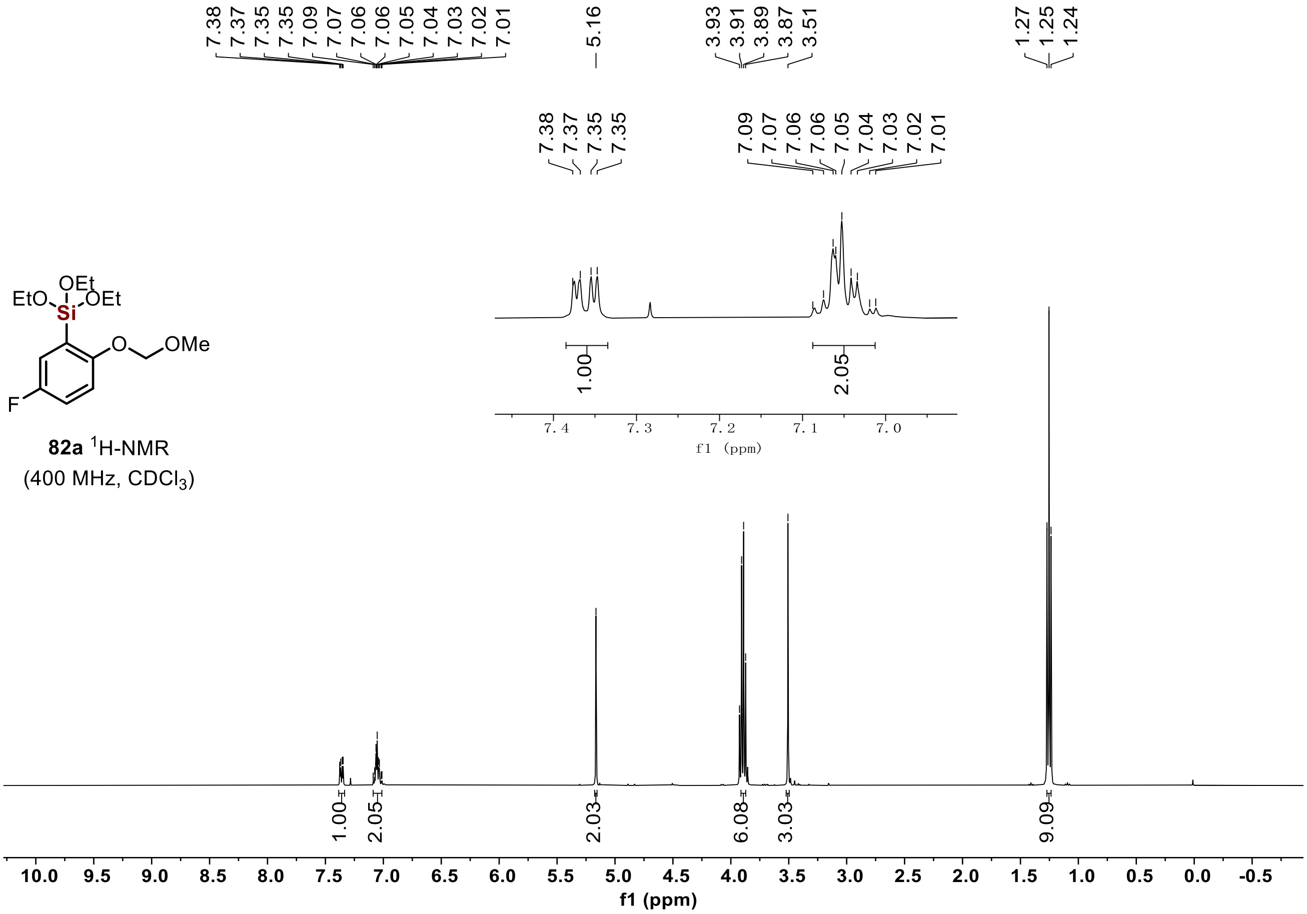

82a ^{1}H-NMR
(400 MHz, $CDCl_3$)

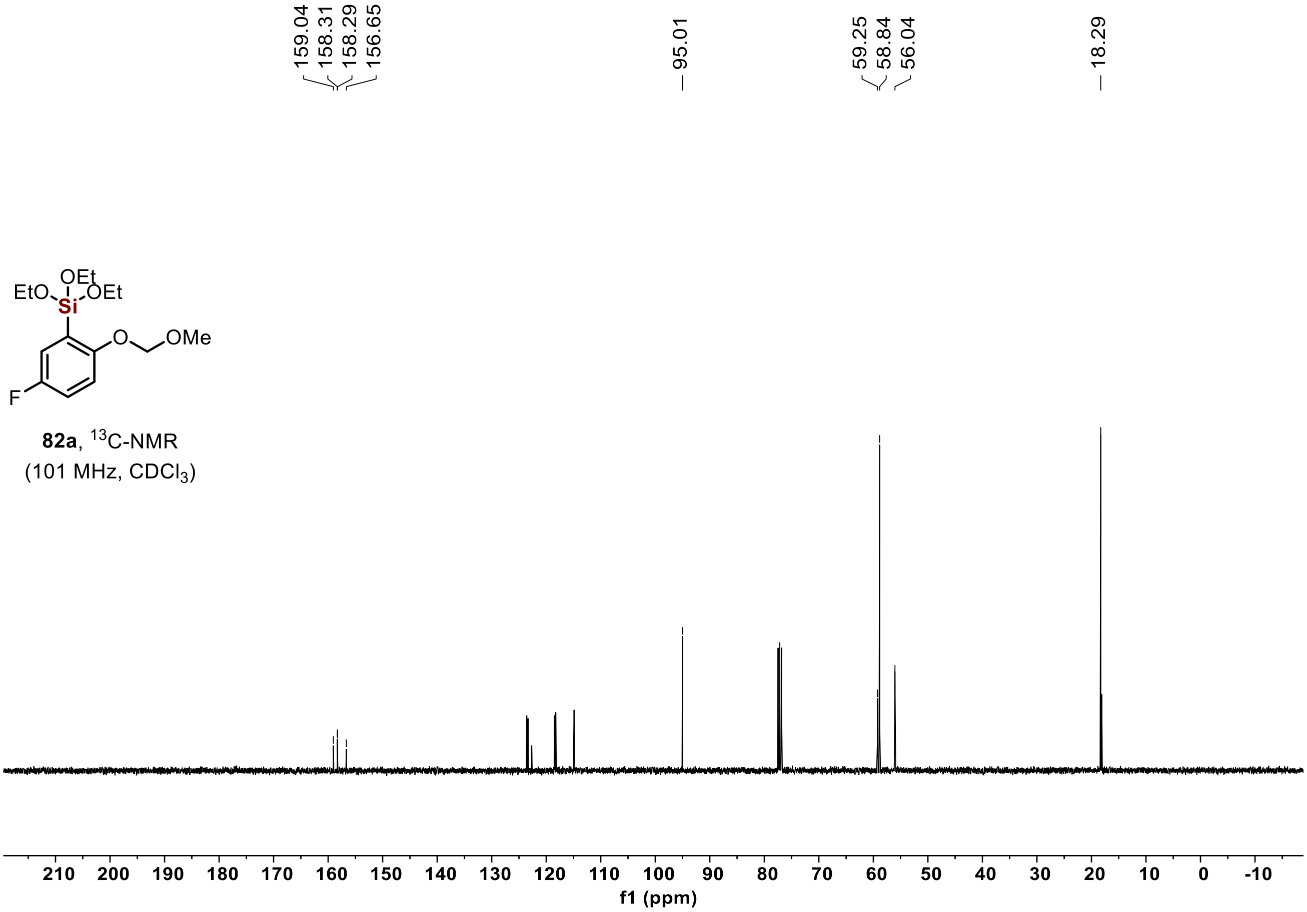

82a, ^{13}C-NMR
(101 MHz, $CDCl_3$)

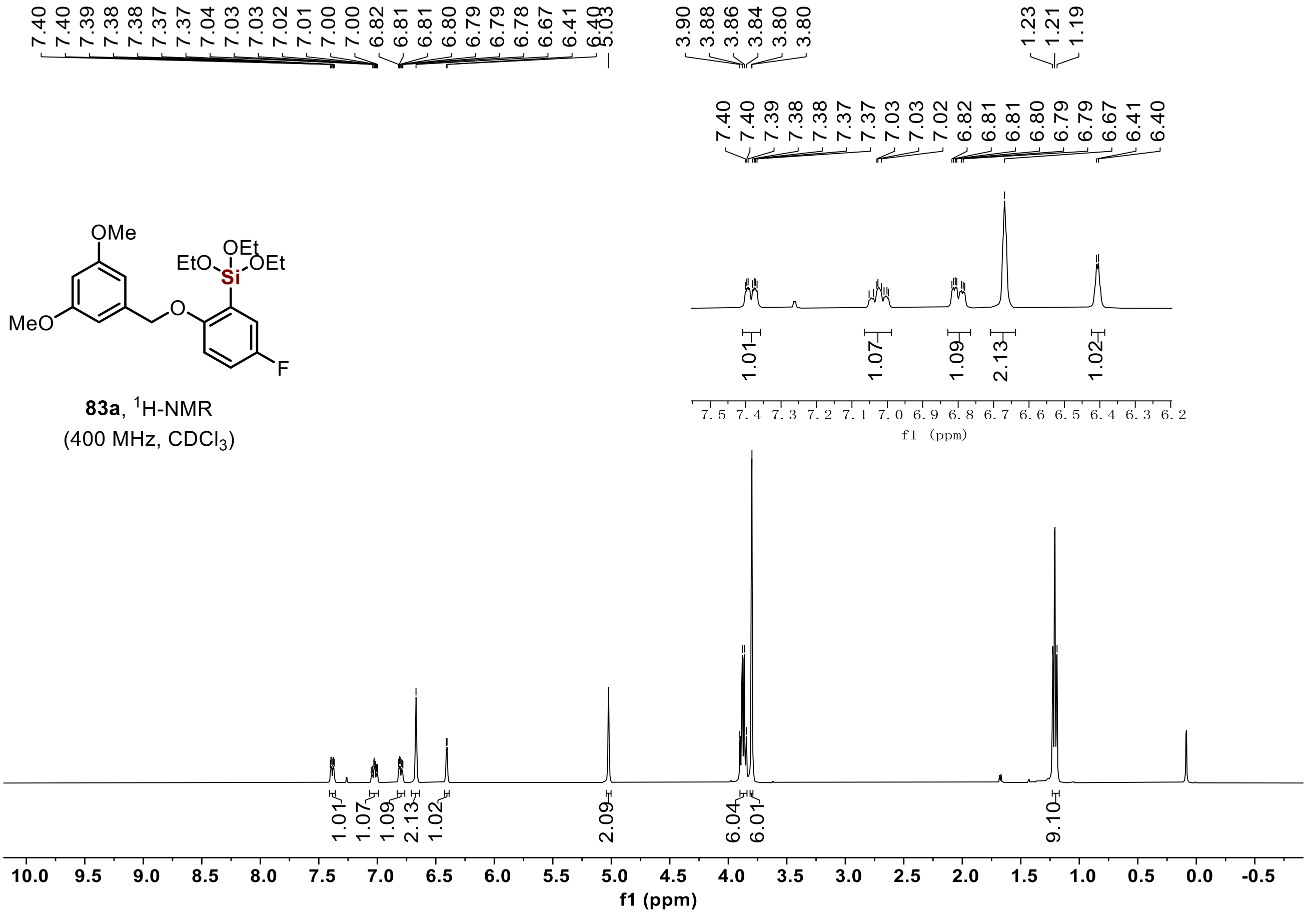

83a, ^{1}H-NMR
(400 MHz, $CDCl_3$)

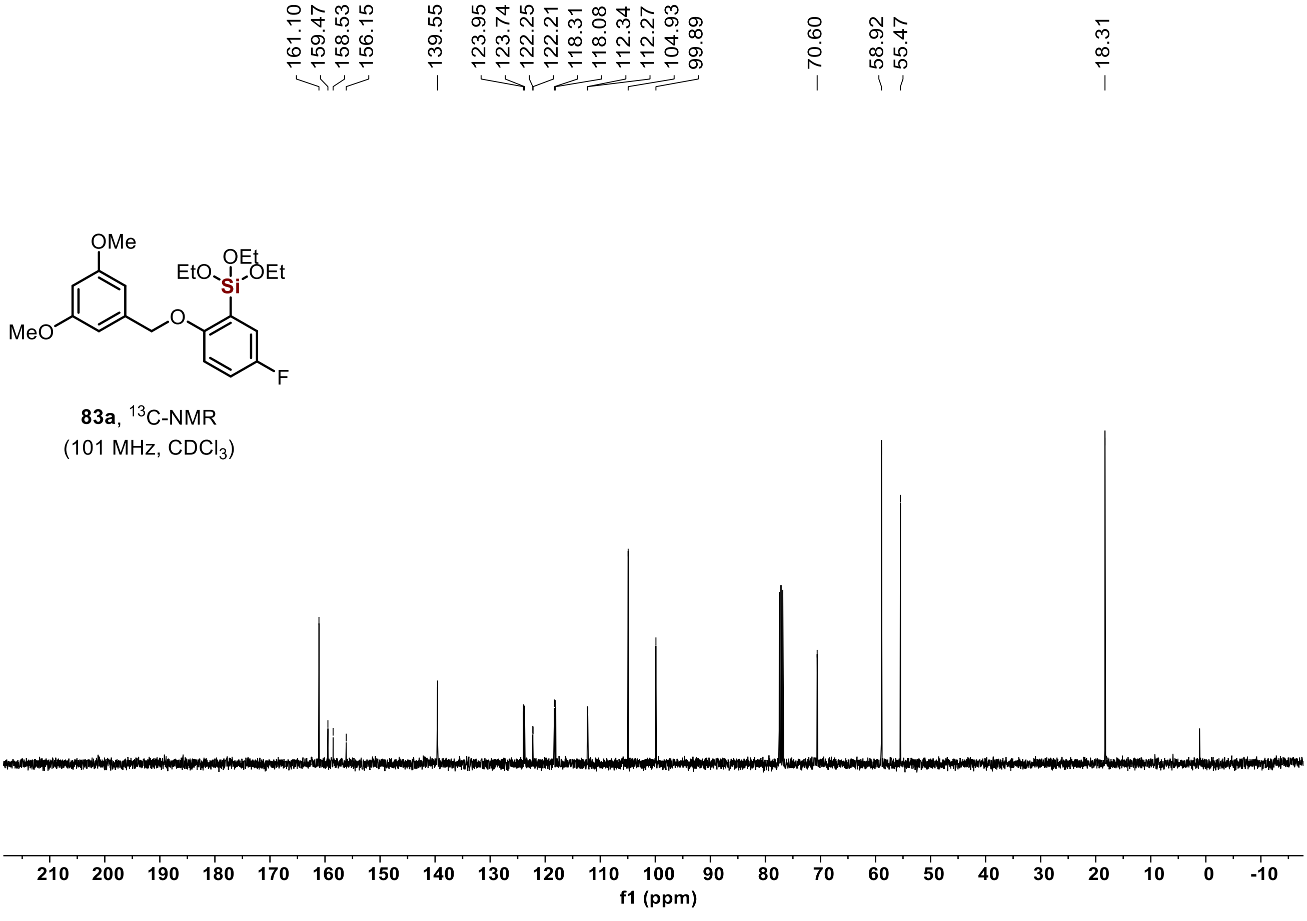

83a, ^{13}C-NMR
(101 MHz, $CDCl_3$)

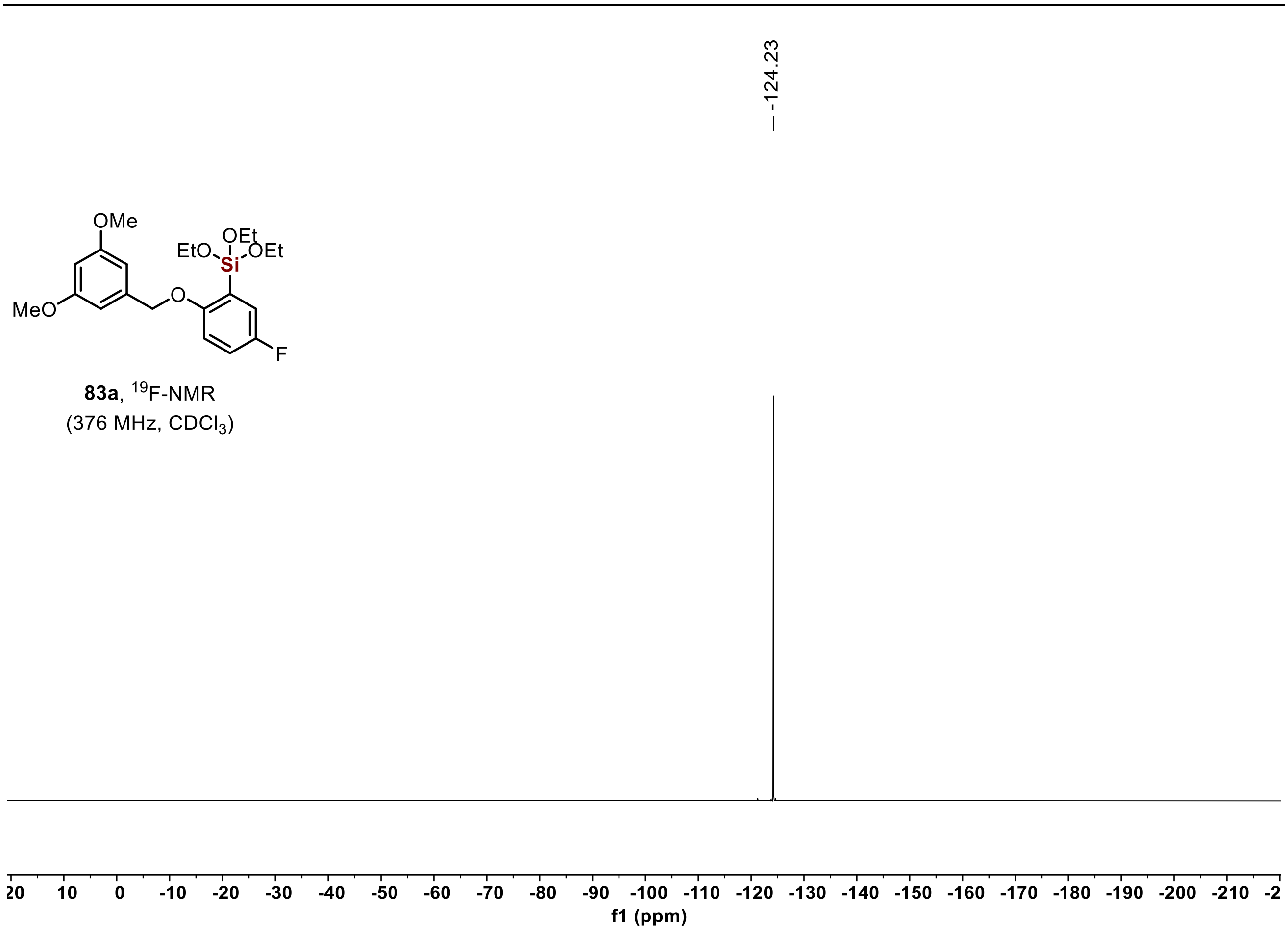
-124.23
OMe
EtO
Si
OEt
OEt
MeO
O
F
83a, 19F-NMR
(376 MHz, CDCl3)
20 10 0 -10 -20 -30 -40 -50 -60 -70 -80 -90 -100 -110 -120 -130 -140 -150 -160 -170 -180 -190 -200 -210 -2
f1 (ppm)

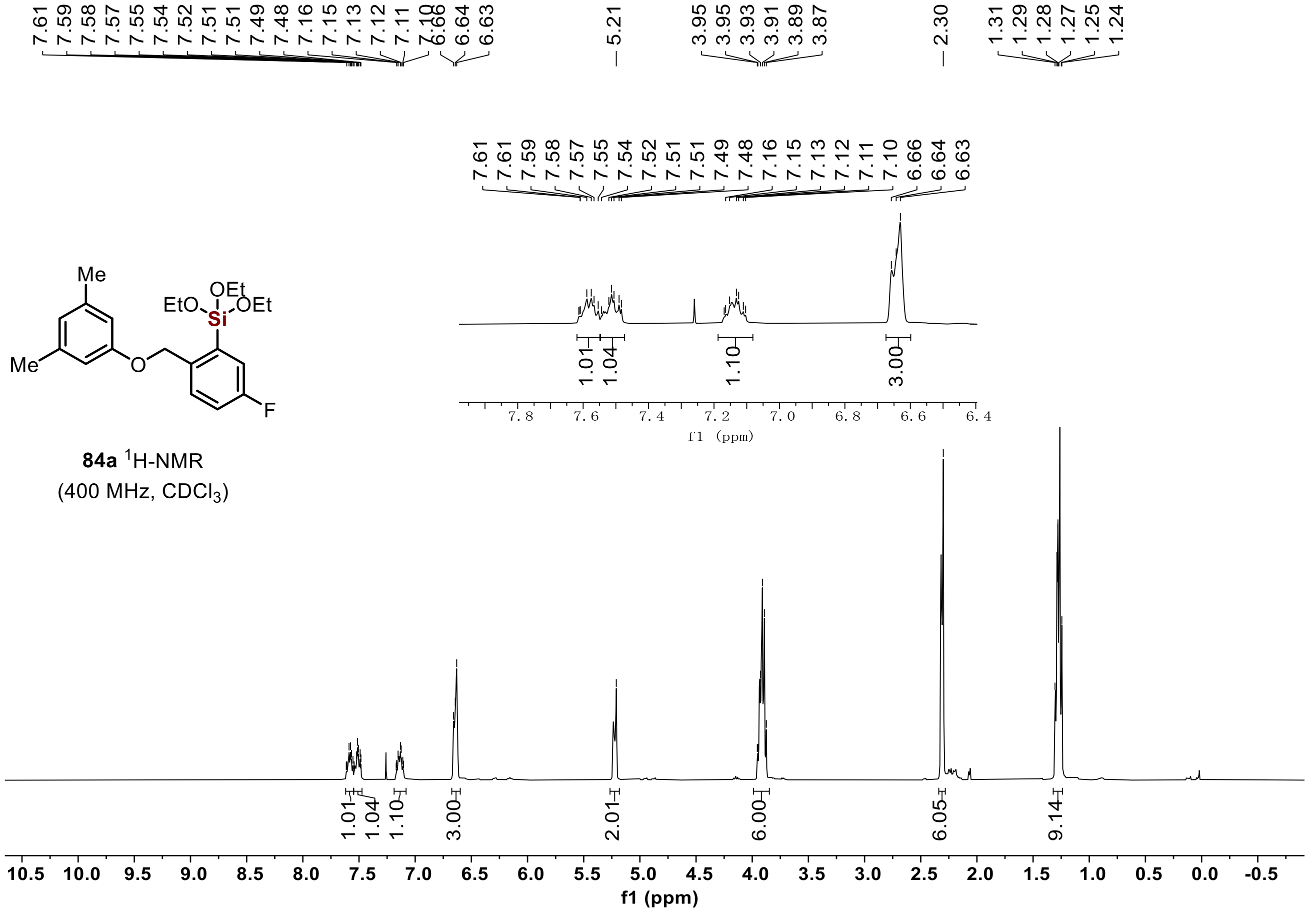
7.61 7.59 7.58 7.57 7.55 7.54 7.52 7.51 7.51 7.49 7.48 7.16 7.15 7.13 7.12 7.11 7.10 6.66 6.64 6.63
5.21
3.95 3.95 3.93 3.91 3.89 3.87
2.30
1.31 1.29 1.28 1.27 1.25 1.24
7.61 7.61 7.59 7.58 7.57 7.55 7.54 7.52 7.51 7.51 7.49 7.48 7.16 7.15 7.13 7.12 7.11 7.10 6.66 6.64 6.63
1.01 1.04 1.10 3.00
7.8 7.6 7.4 7.2 7.0 6.8 6.6 6.4
f1 (ppm)
Me
EtO
Si
OEt
OEt
Me
O
F
84a 1H-NMR
(400 MHz, CDCl3)
1.01 1.04 1.10 3.00 2.01 6.00 6.05 9.14
10.5 10.0 9.5 9.0 8.5 8.0 7.5 7.0 6.5 6.0 5.5 5.0 4.5 4.0 3.5 3.0 2.5 2.0 1.5 1.0 0.5 0.0 -0.5
f1 (ppm)

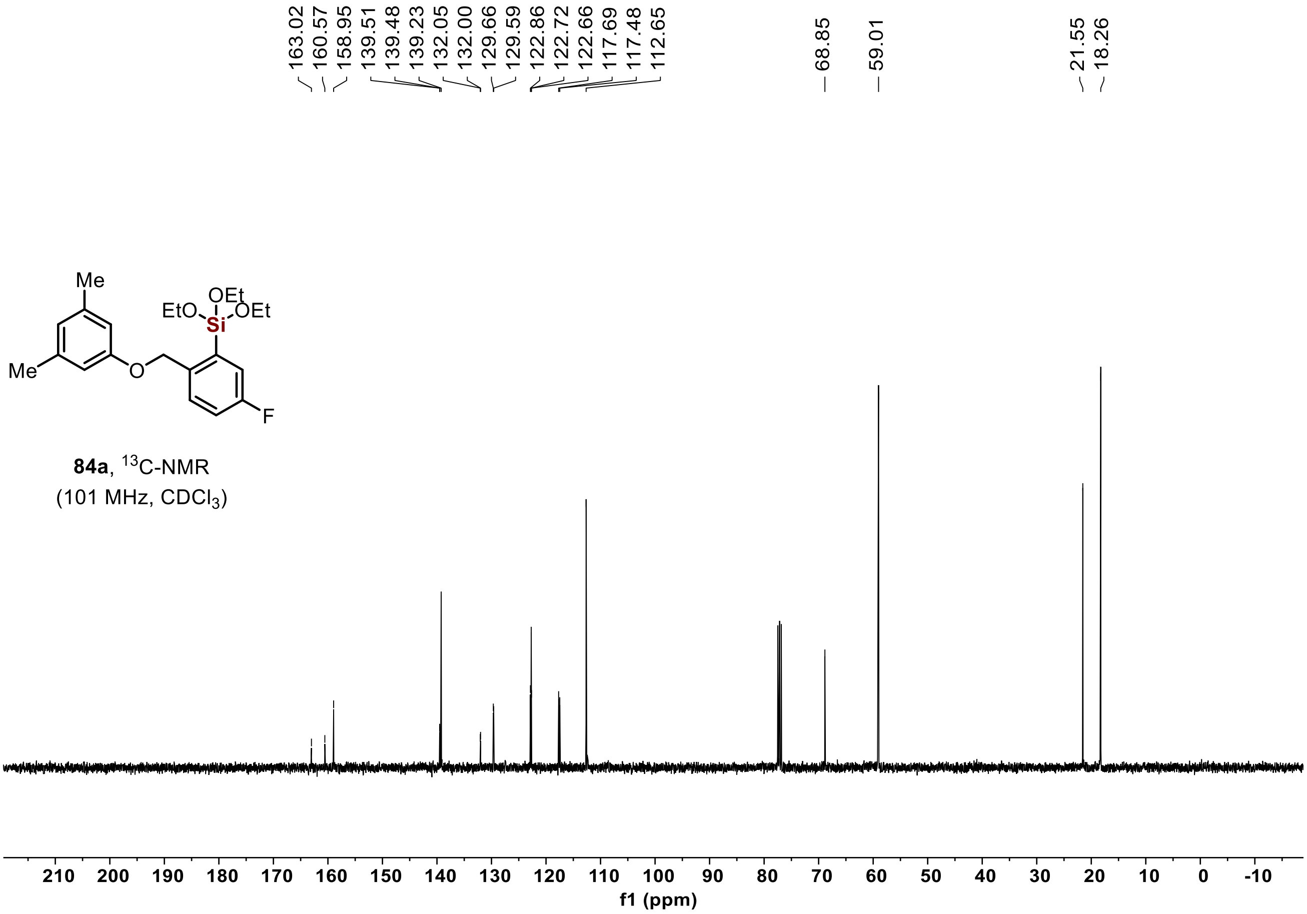

84a, ^{13}C-NMR
(101 MHz, $CDCl_3$)

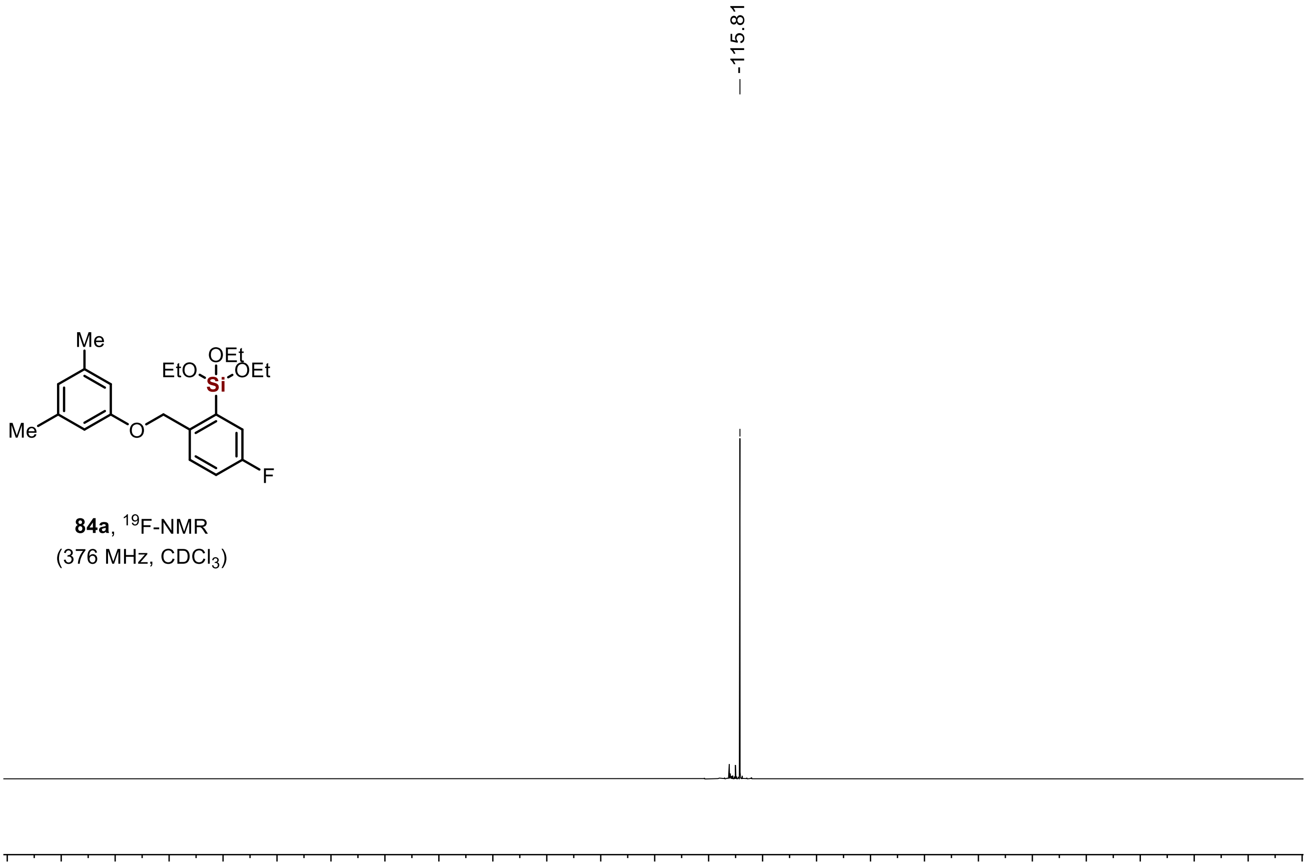

84a, ^{19}F-NMR
(376 MHz, $CDCl_3$)

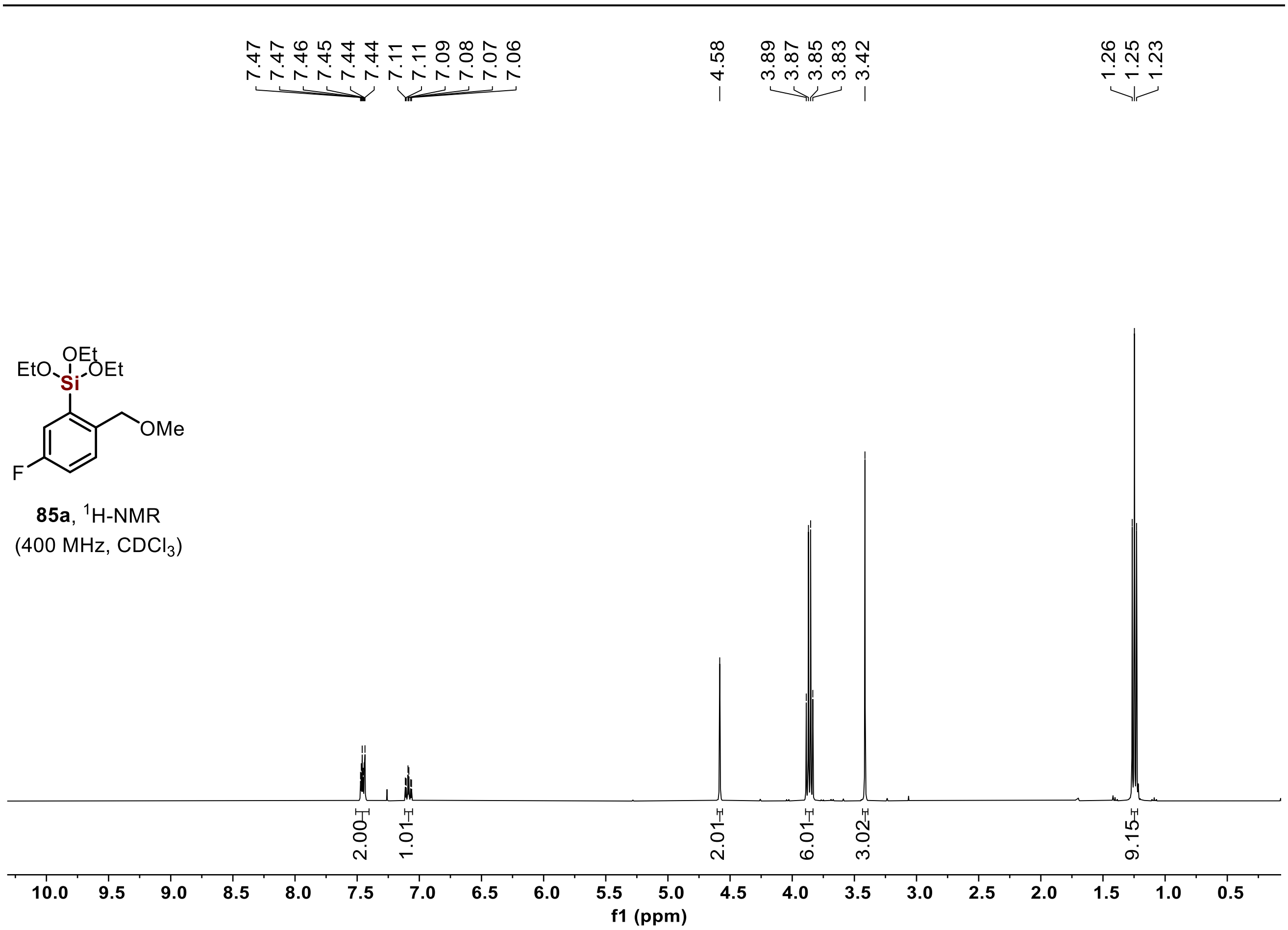

85a, ^{1}H-NMR
(400 MHz, $CDCl_3$)

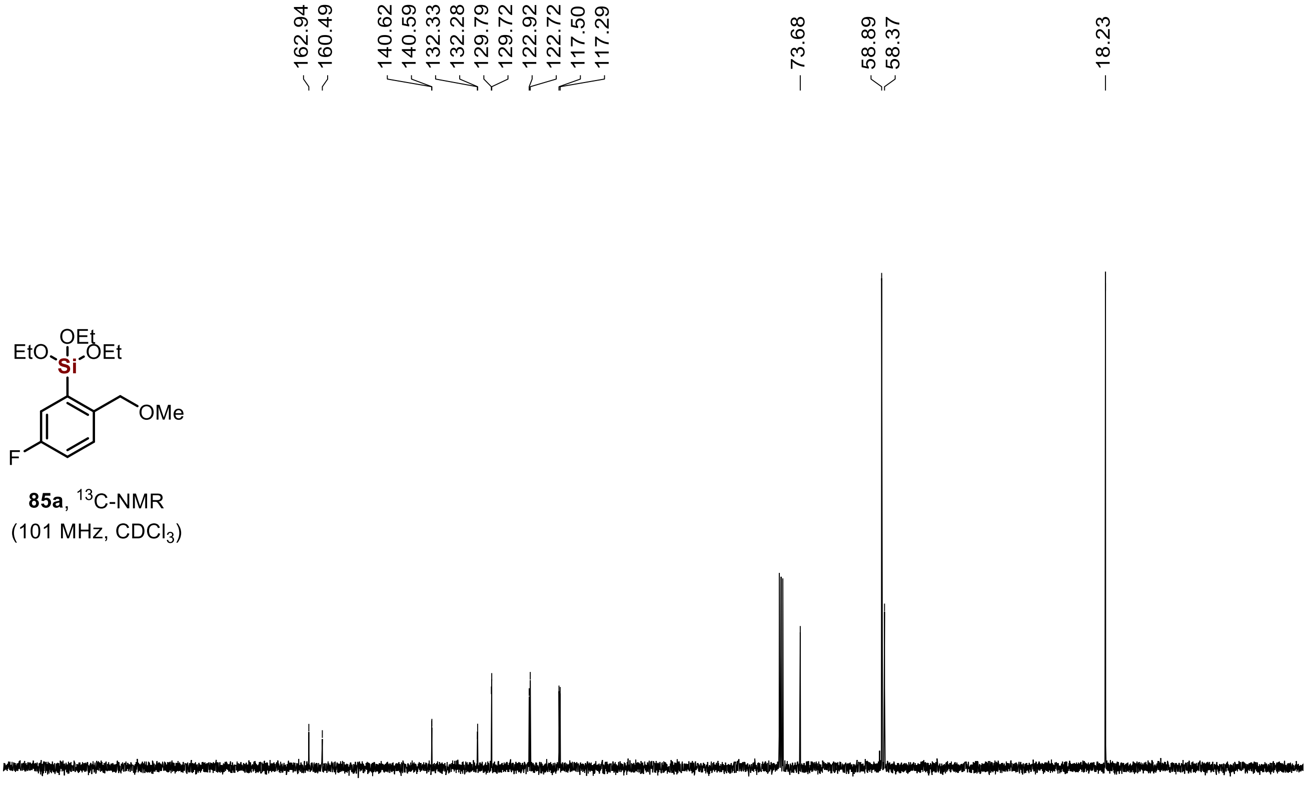

85a, ^{13}C-NMR
(101 MHz, $CDCl_3$)

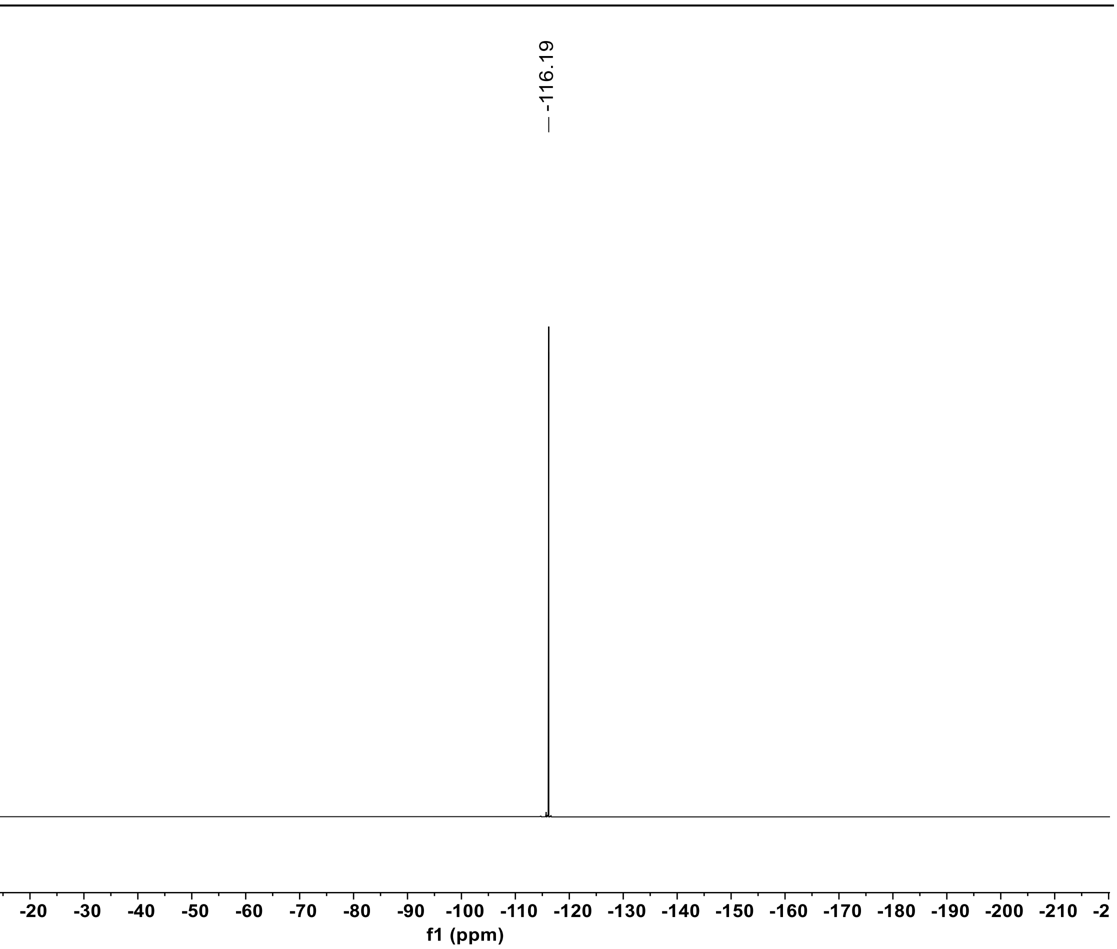

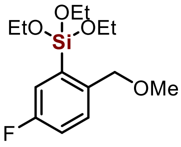

85a, ^{19}F-NMR
(376 MHz, $CDCl_3$)

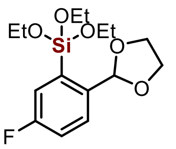

86a, ^{1}H-NMR
(400 MHz, $CDCl_3$)

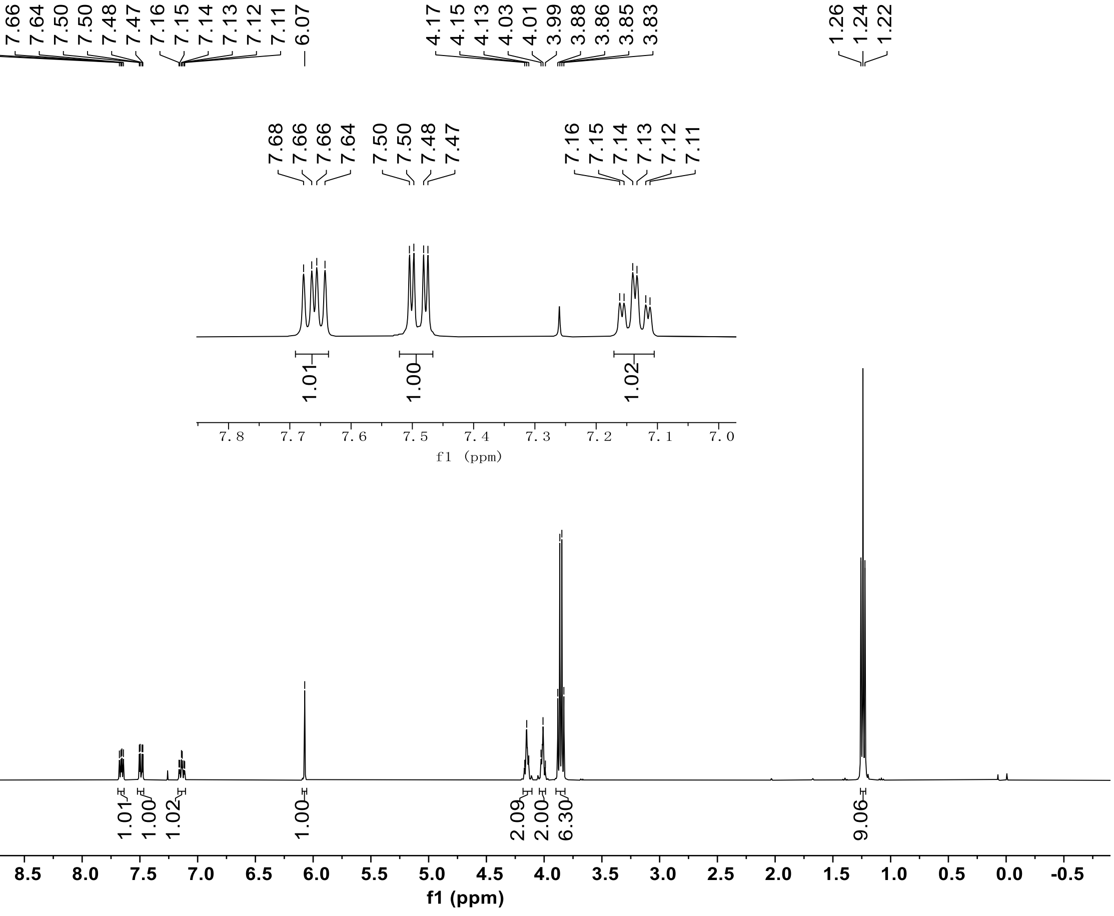

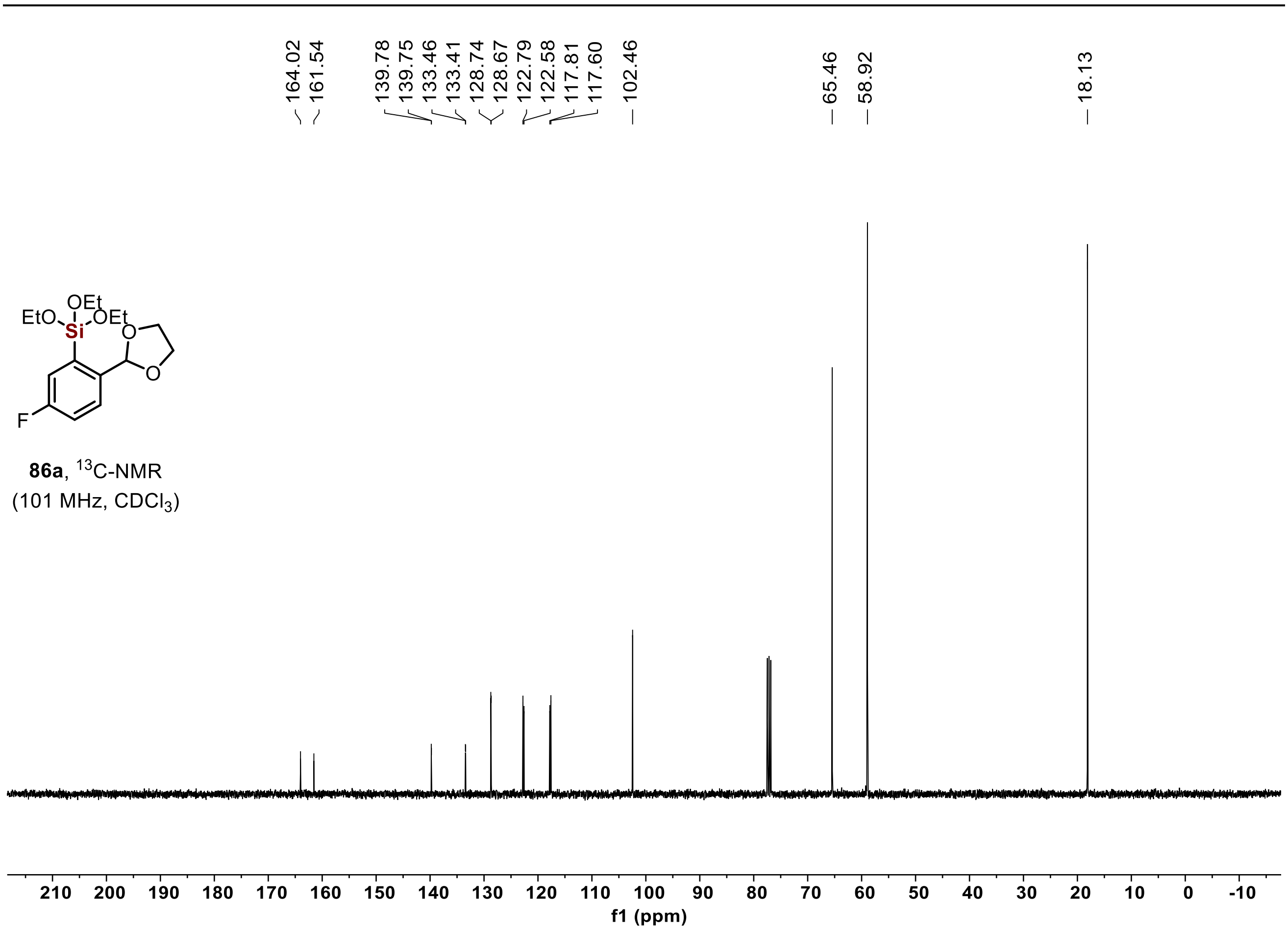

86a, ^{13}C-NMR
(101 MHz, $CDCl_3$)

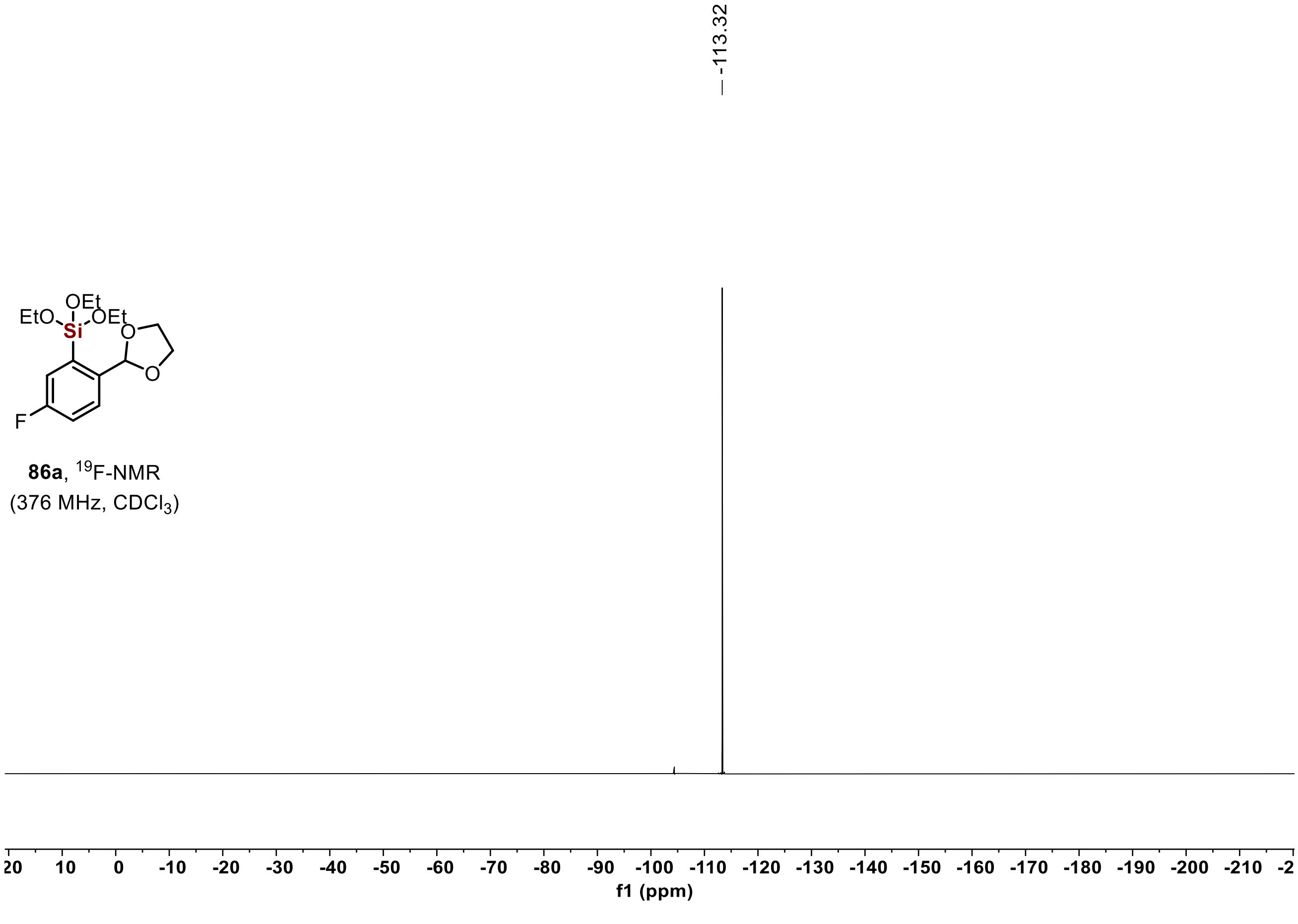

86a, ^{19}F-NMR
(376 MHz, $CDCl_3$)

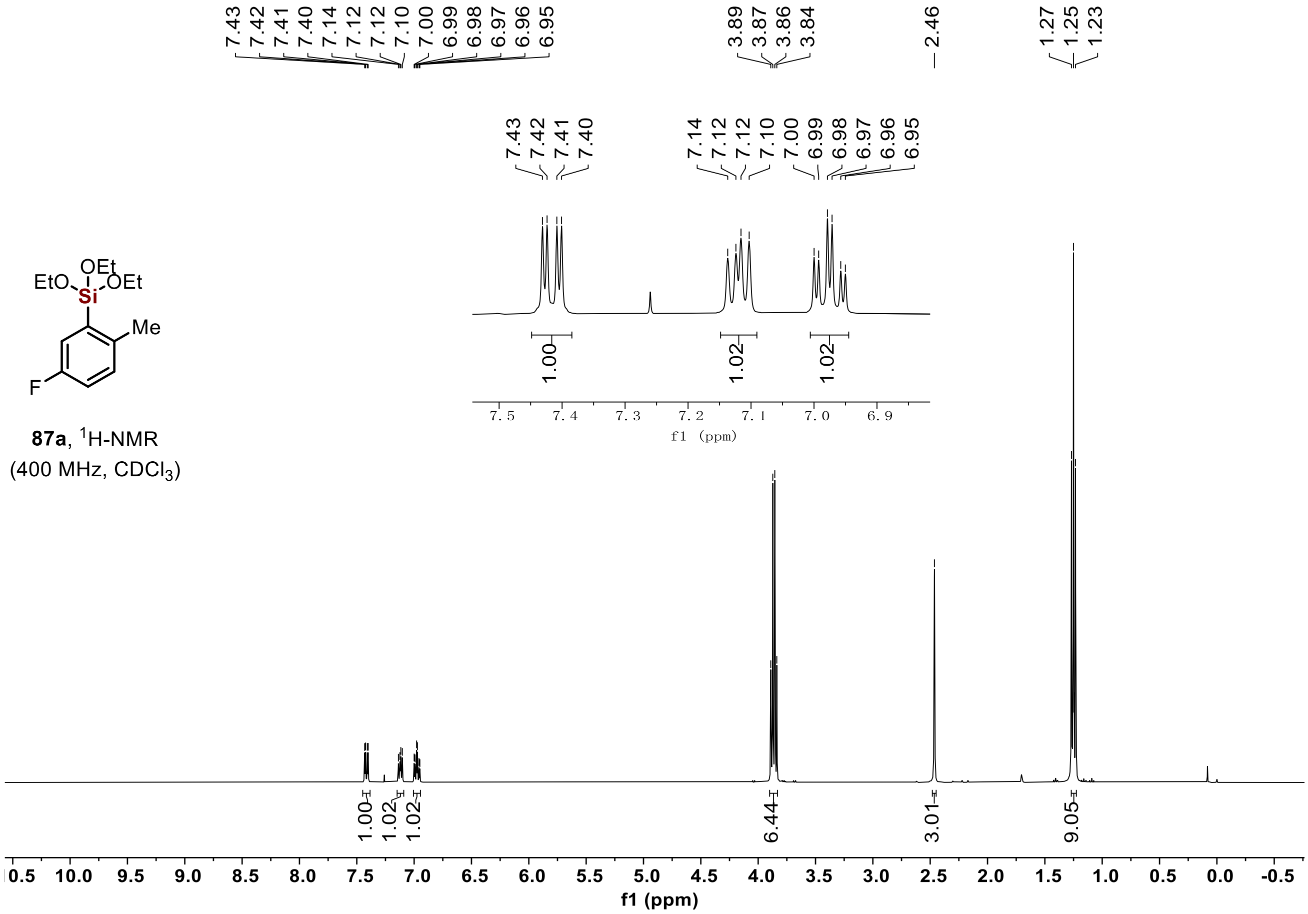

87a, ^{1}H-NMR
(400 MHz, $CDCl_3$)

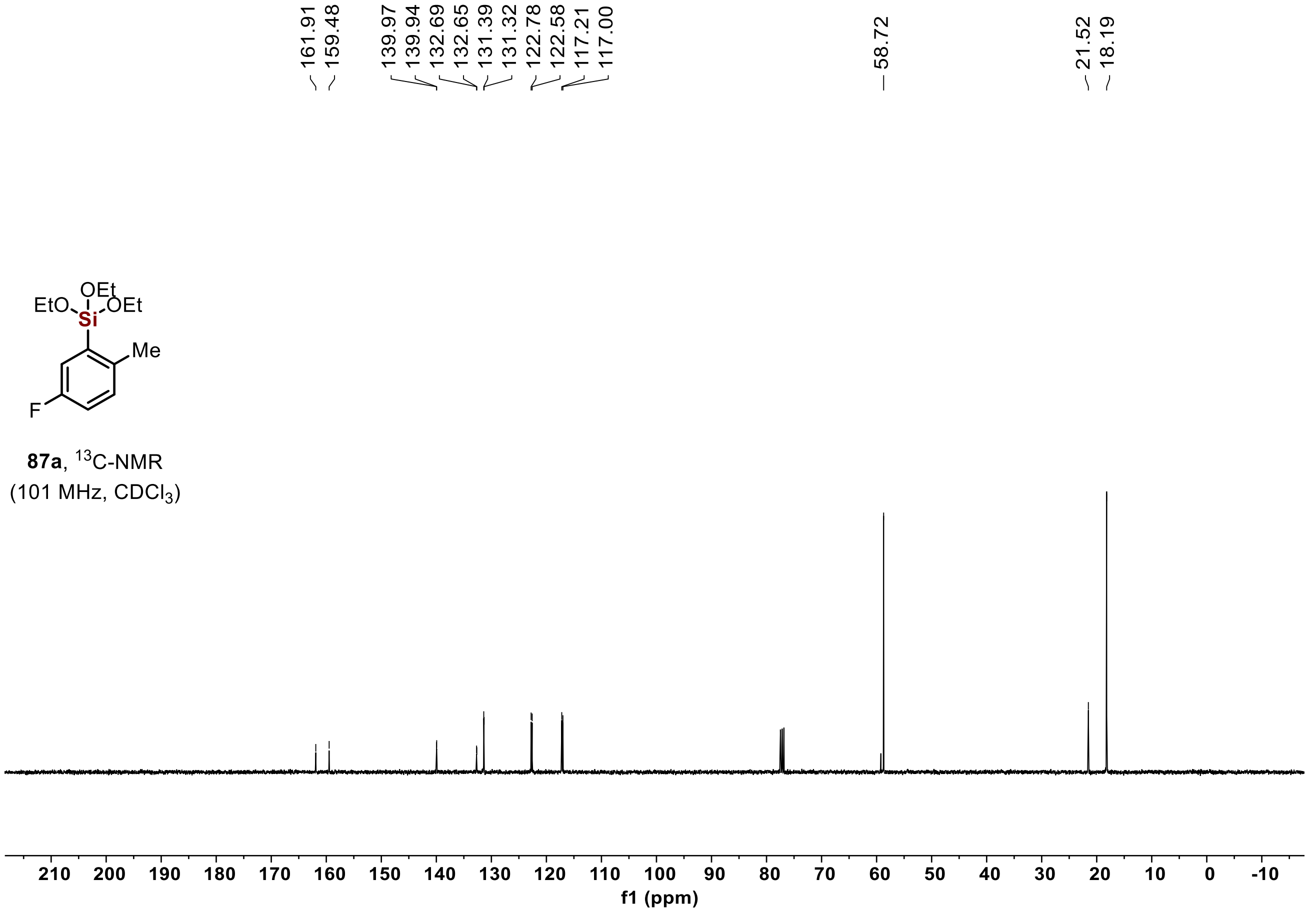

87a, ^{13}C-NMR
(101 MHz, $CDCl_3$)

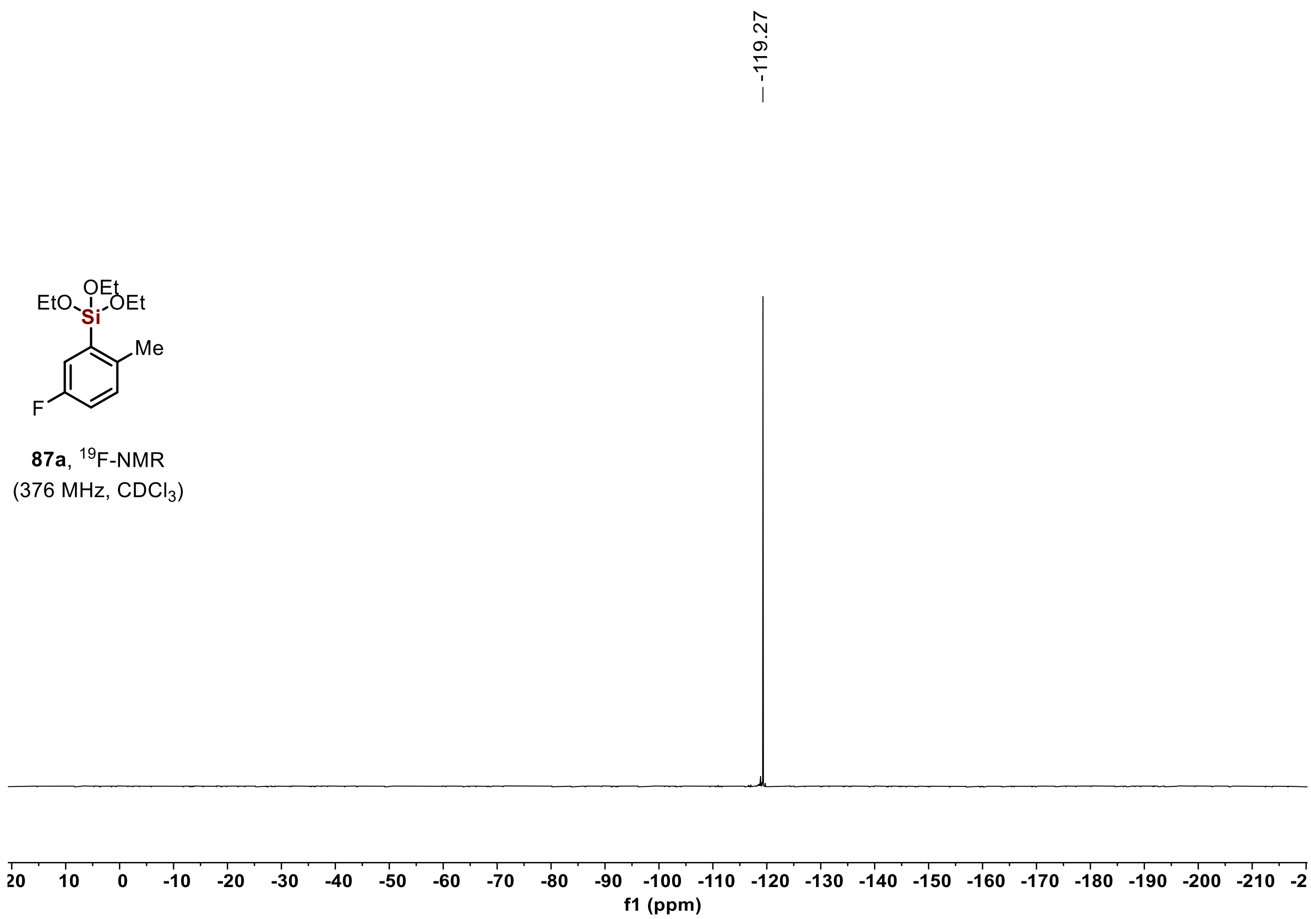

87a, ^{19}F-NMR
(376 MHz, $CDCl_3$)

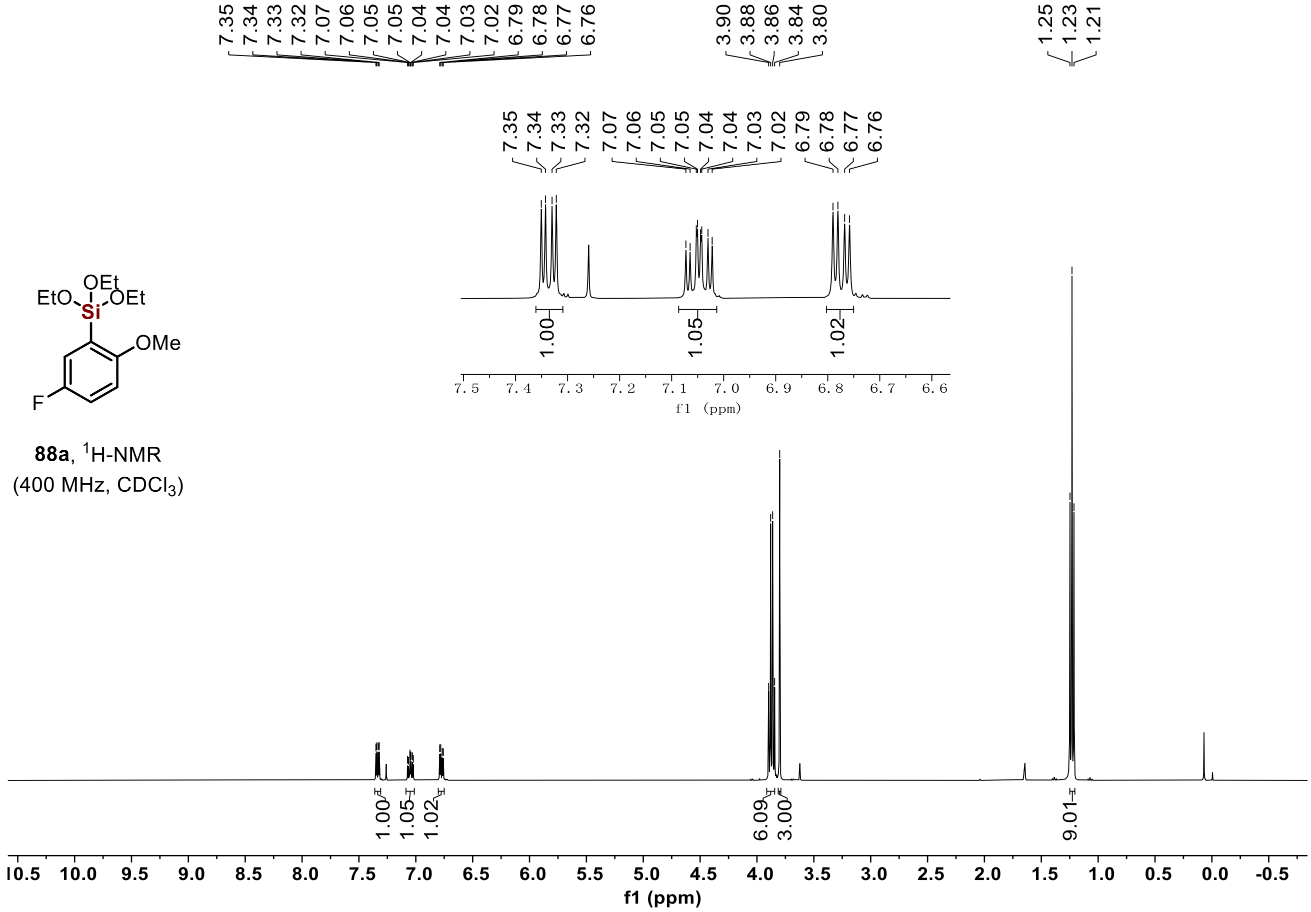

88a, ^{1}H-NMR
(400 MHz, $CDCl_3$)

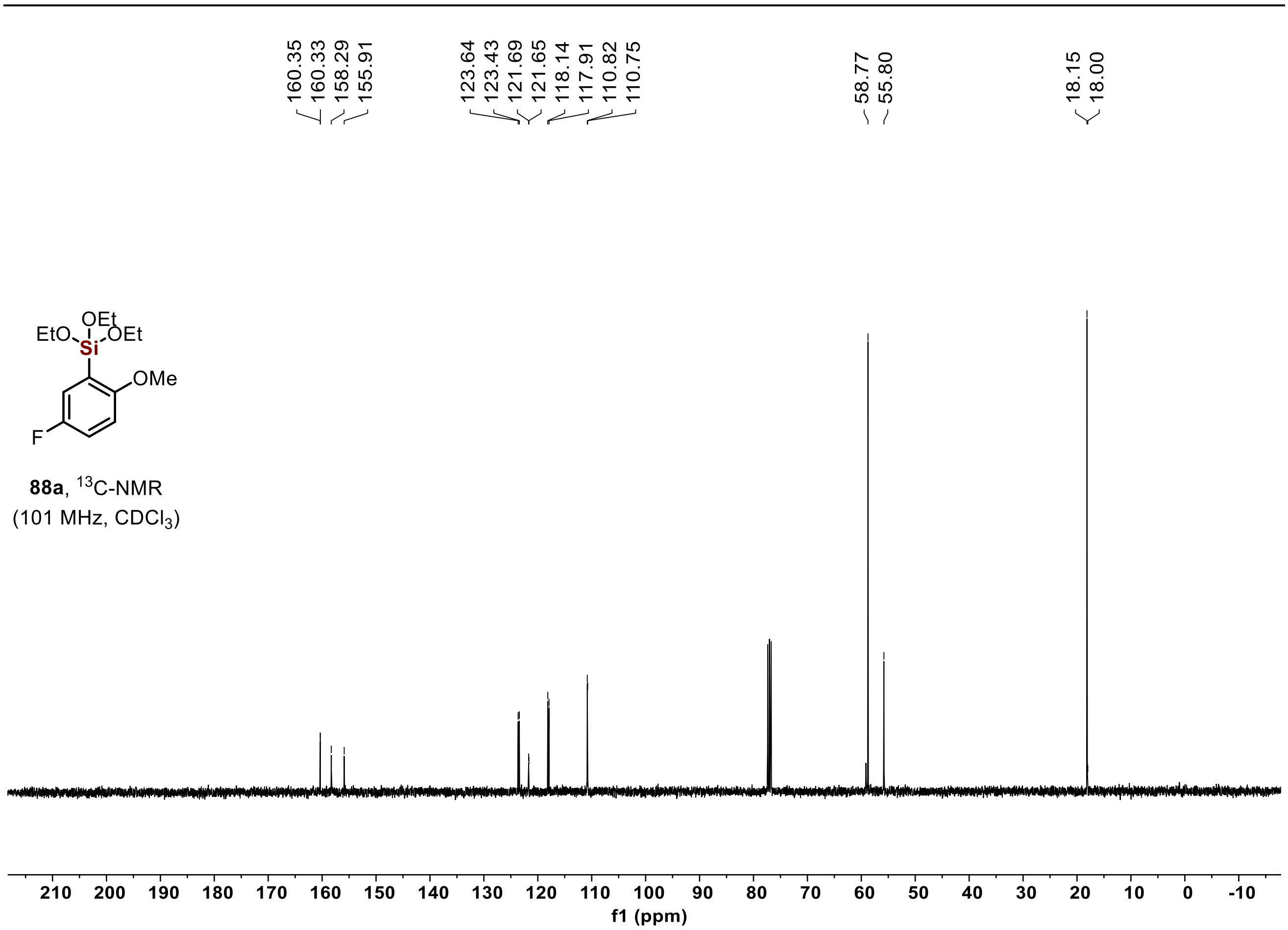

88a, ^{13}C-NMR
(101 MHz, $CDCl_3$)

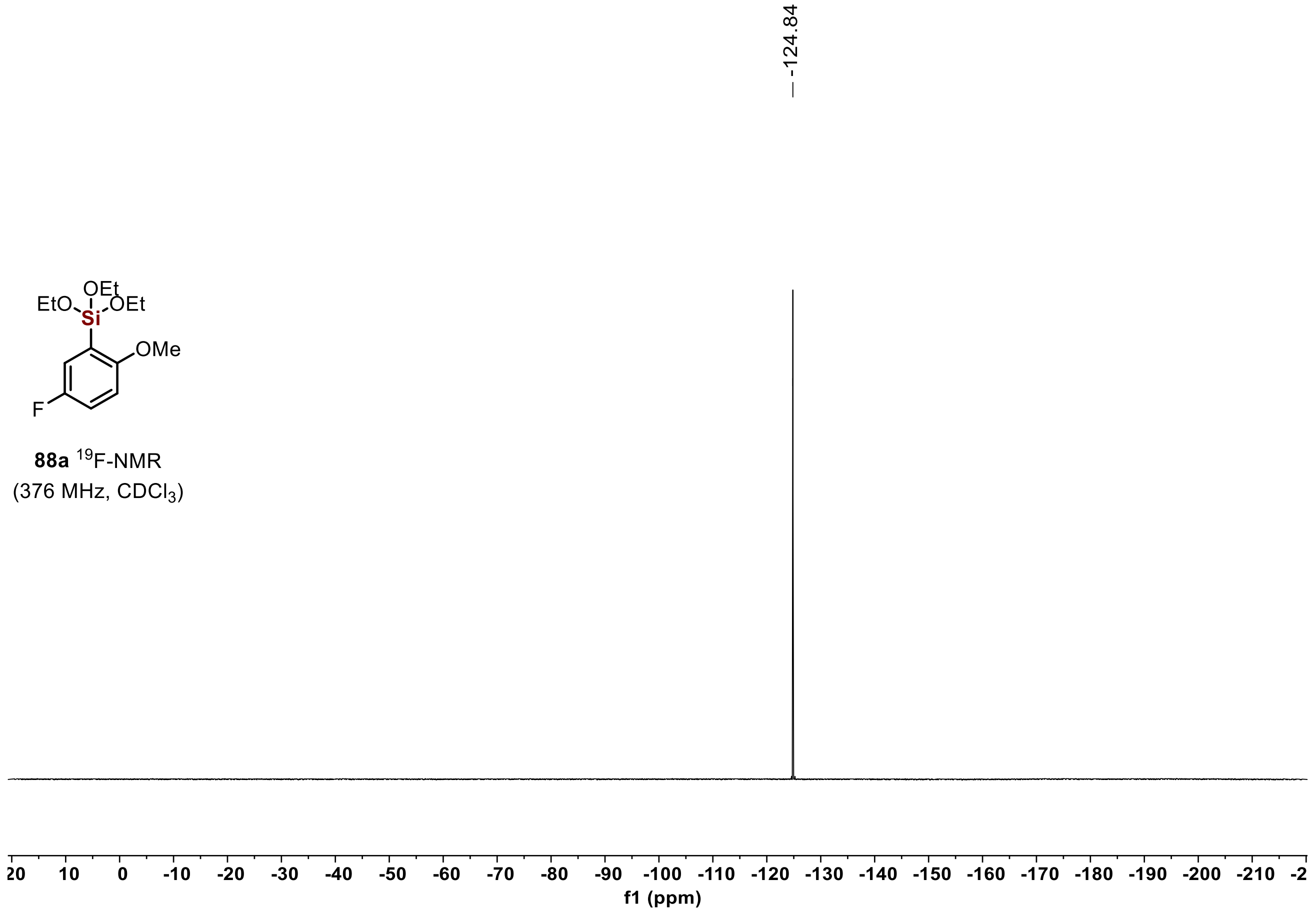

88a ^{19}F-NMR
(376 MHz, $CDCl_3$)

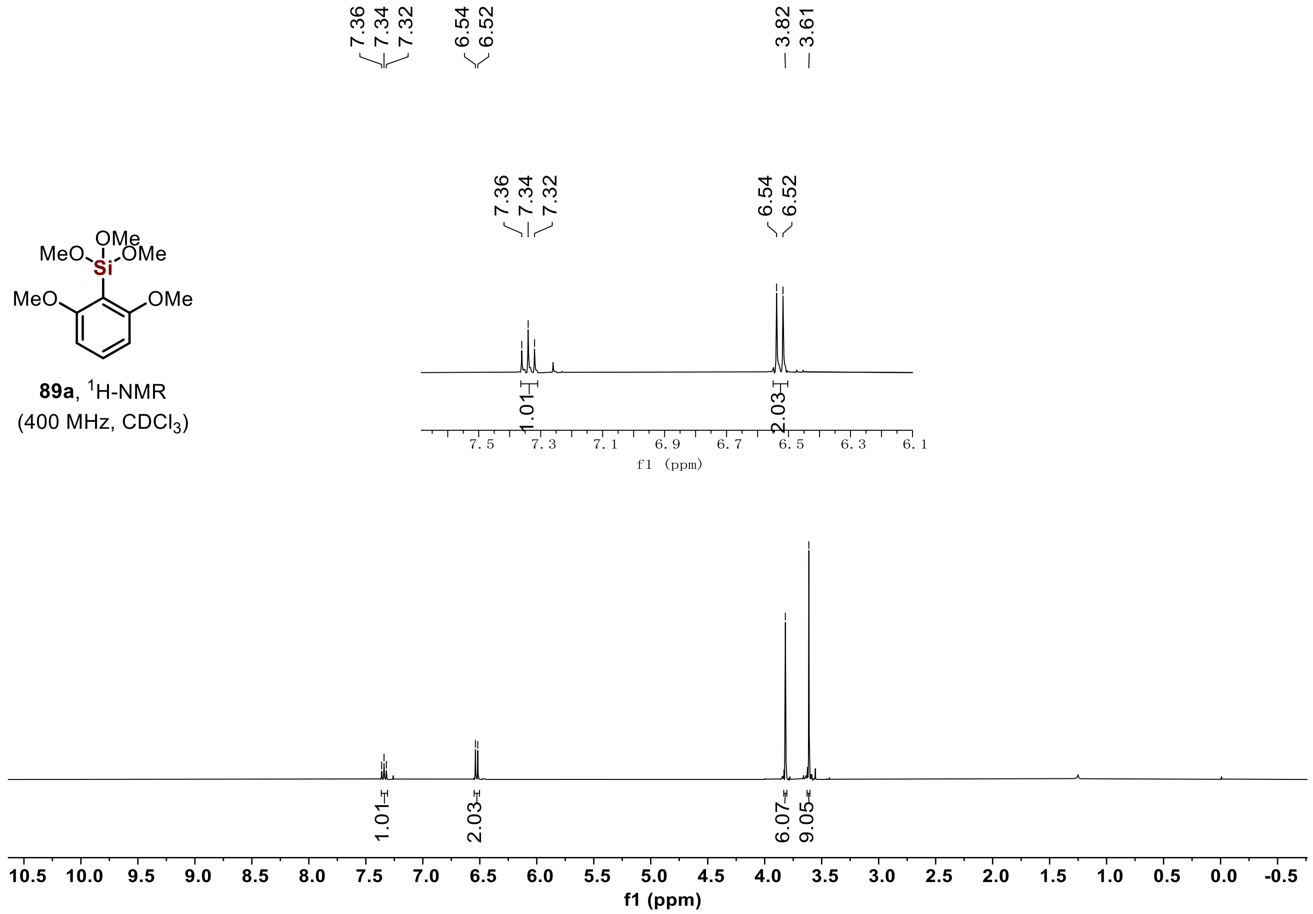

7.36
7.34
7.32
6.54
6.52
3.82
3.61
MeO
OMe
Si
MeO
OMe
89a, ^{1}H-NMR
(400 MHz, $CDCl_3$)
1.01
2.03
6.07
9.05
f1 (ppm)

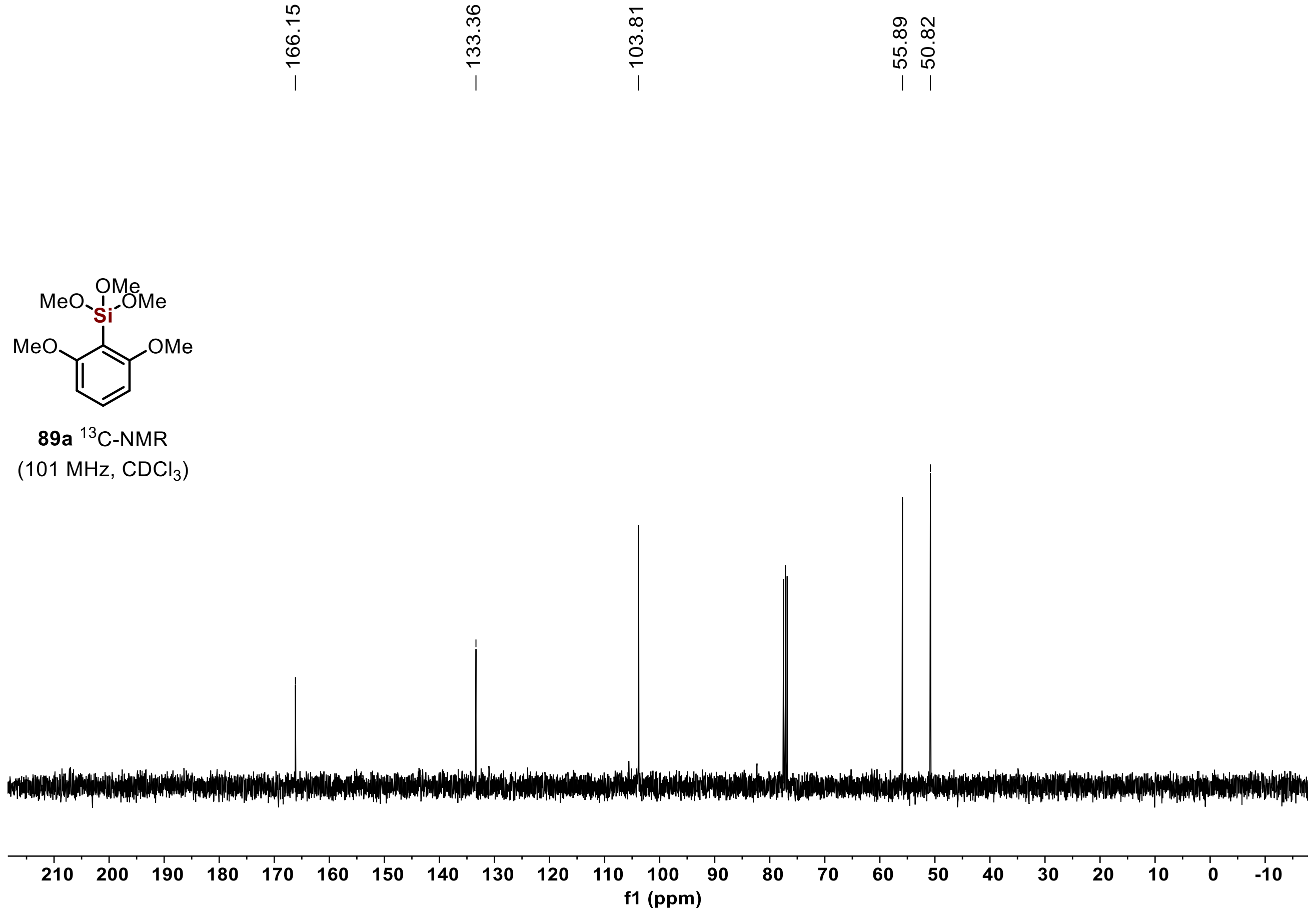

166.15
133.36
103.81
55.89
50.82
MeO
OMe
Si
MeO
OMe
89a ^{13}C-NMR
(101 MHz, $CDCl_3$)
f1 (ppm)

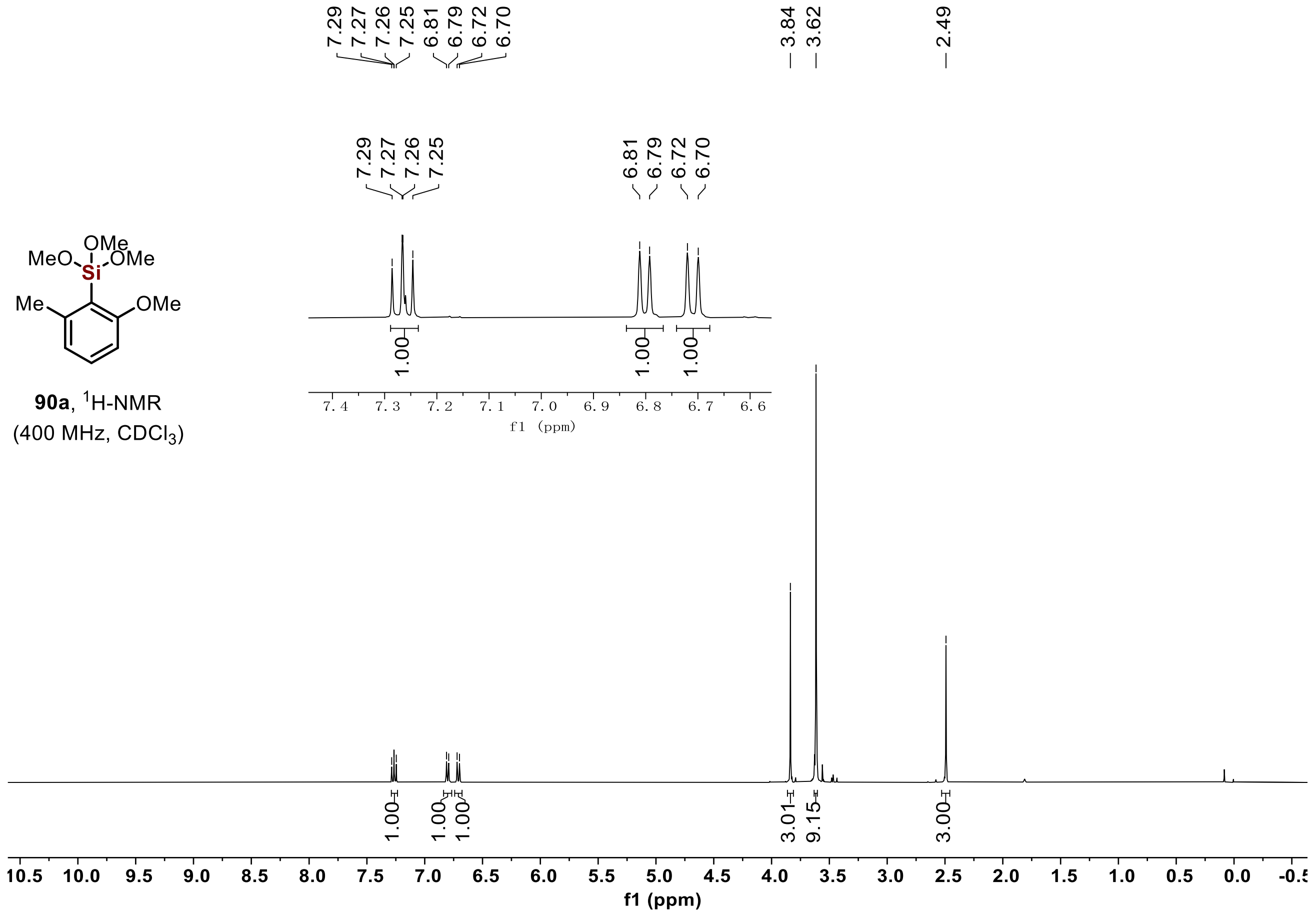

90a, ^{1}H-NMR
(400 MHz, $CDCl_3$)

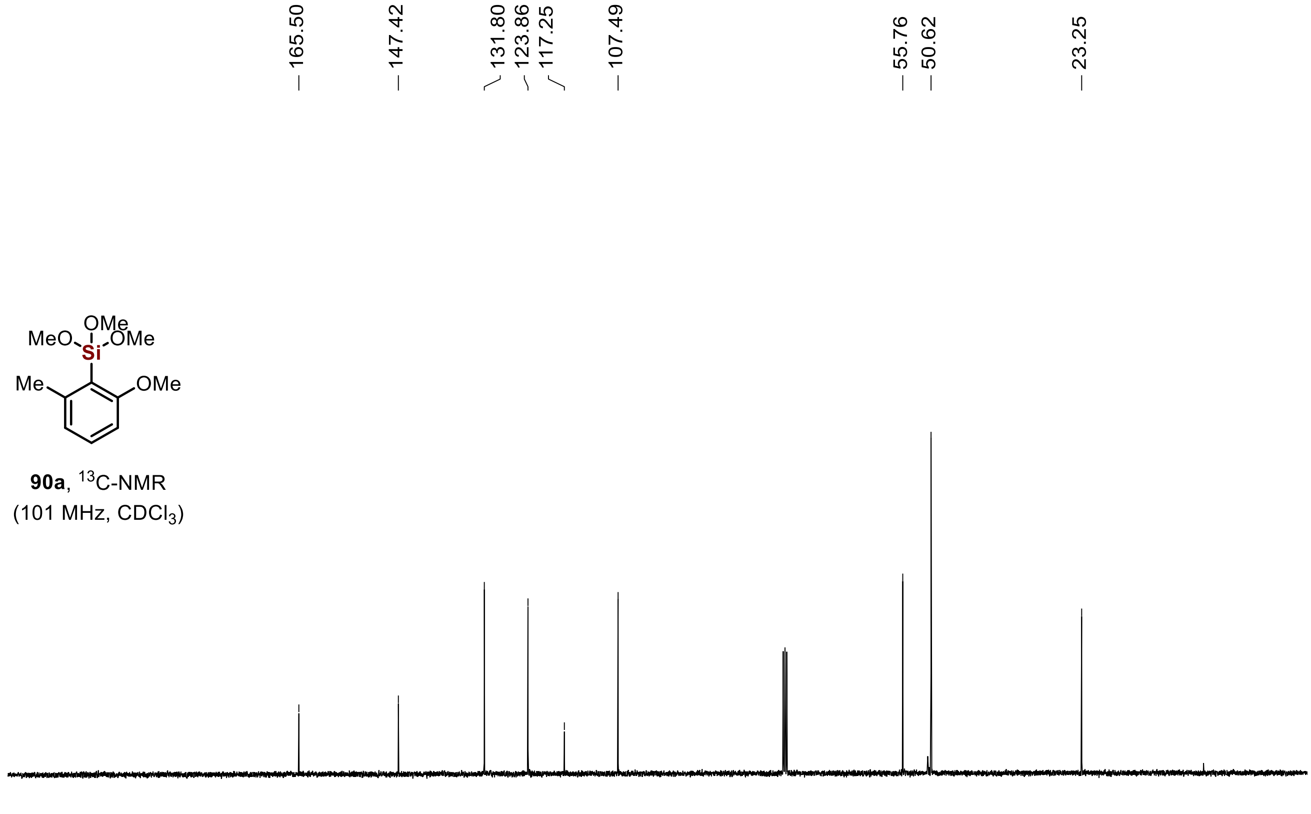

90a, ^{13}C-NMR
(101 MHz, $CDCl_3$)

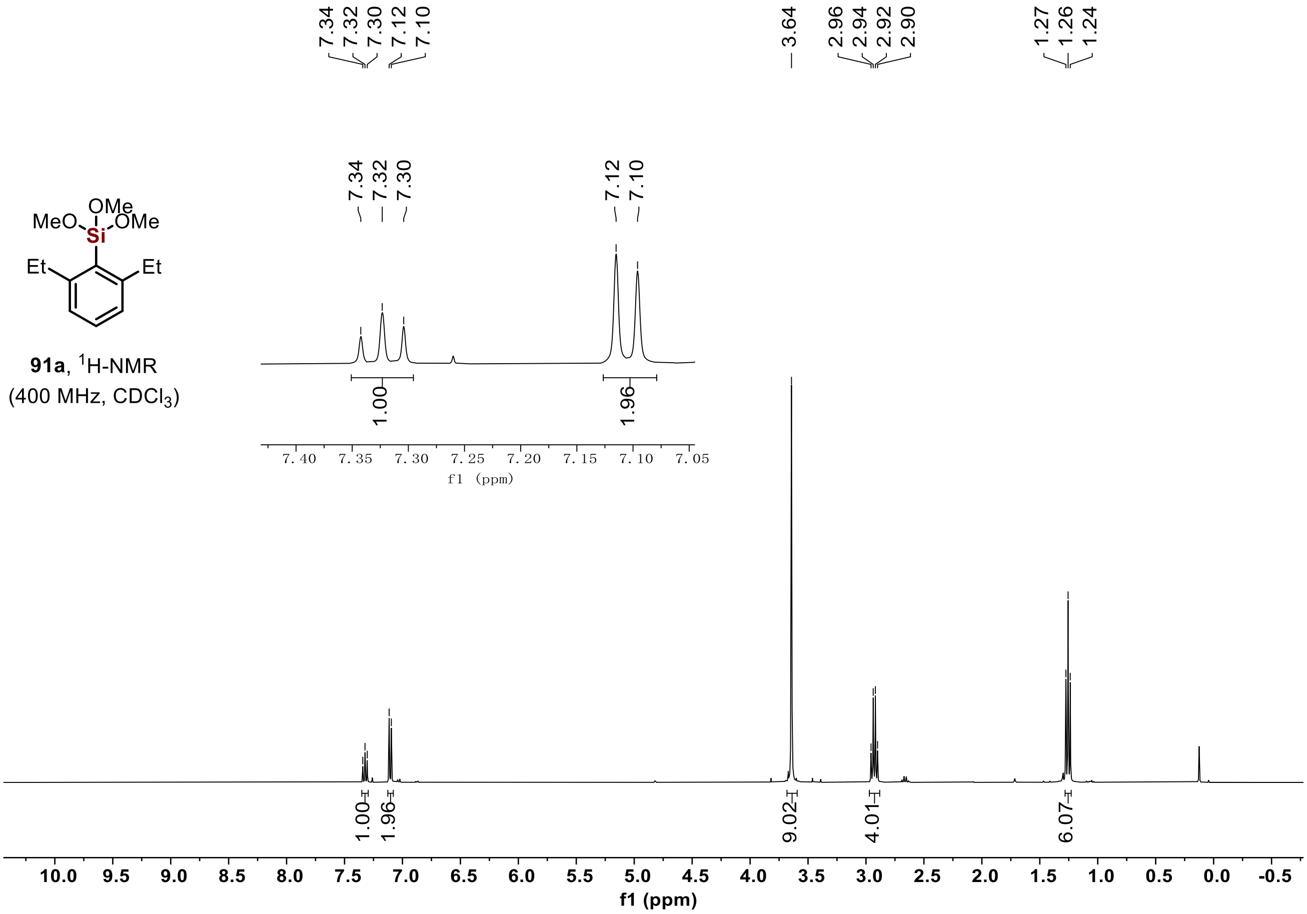

91a, ^{1}H-NMR
(400 MHz, $CDCl_3$)

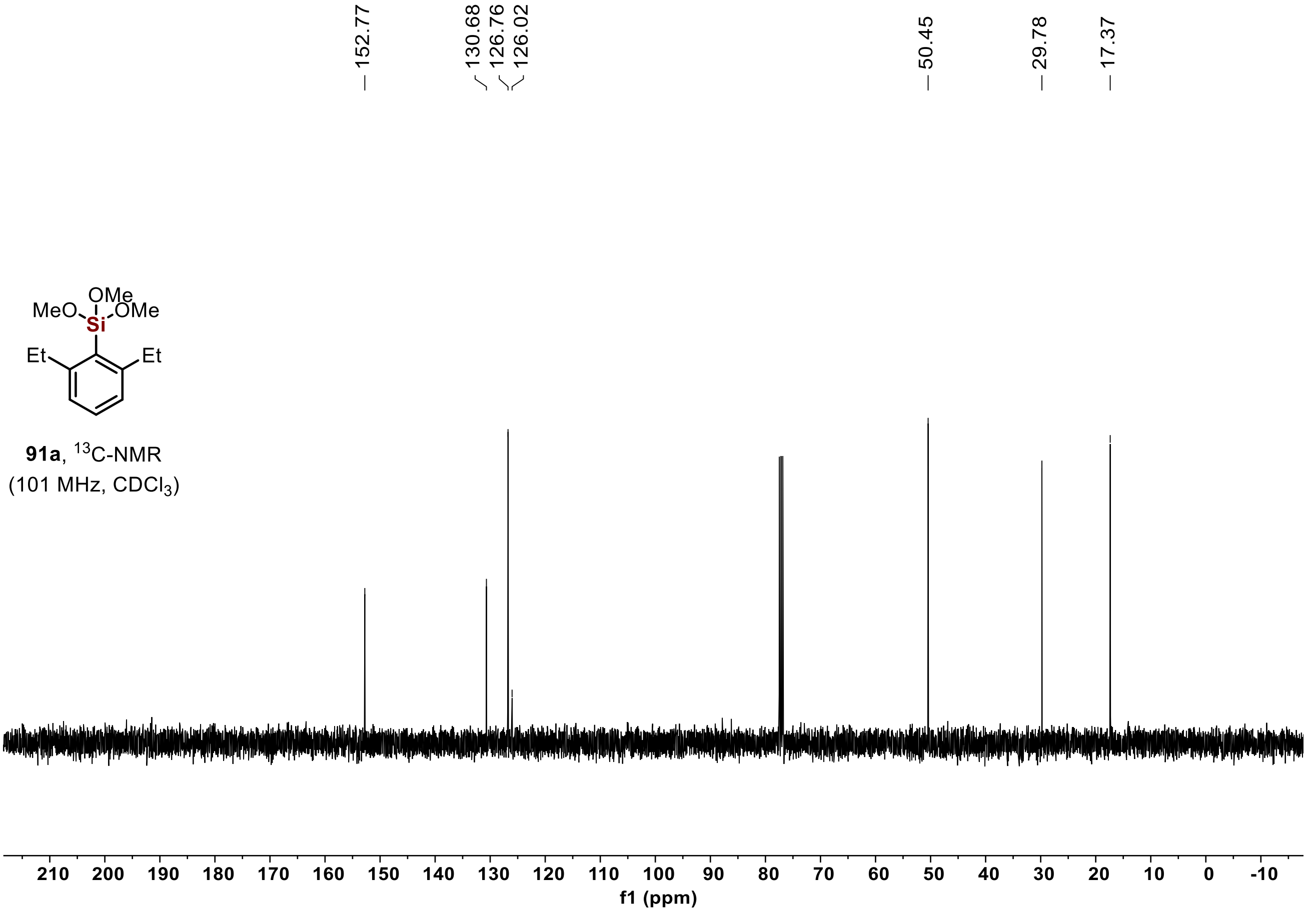

91a, ^{13}C-NMR
(101 MHz, $CDCl_3$)

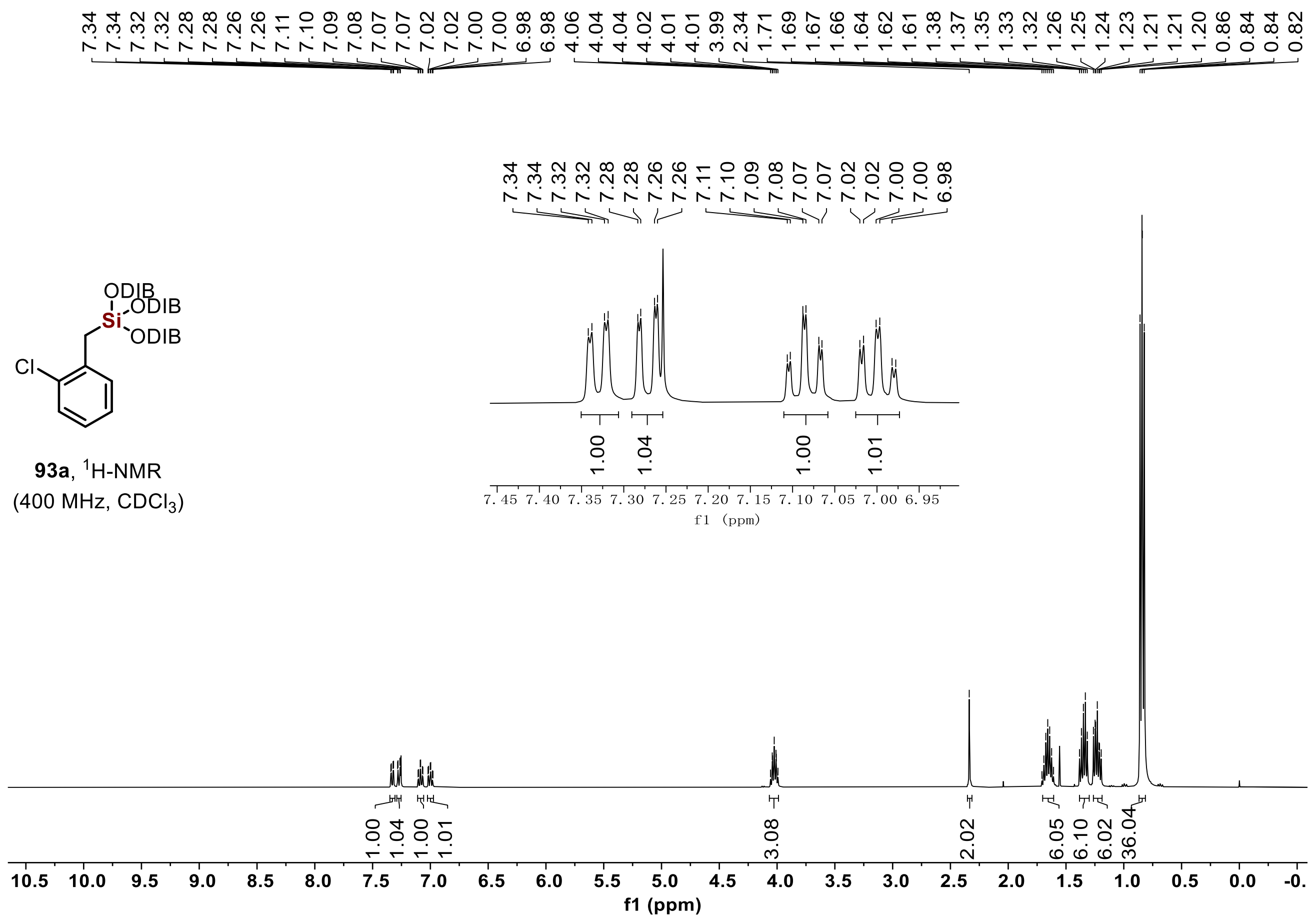

93a, ^{1}H-NMR
(400 MHz, $CDCl_3$)

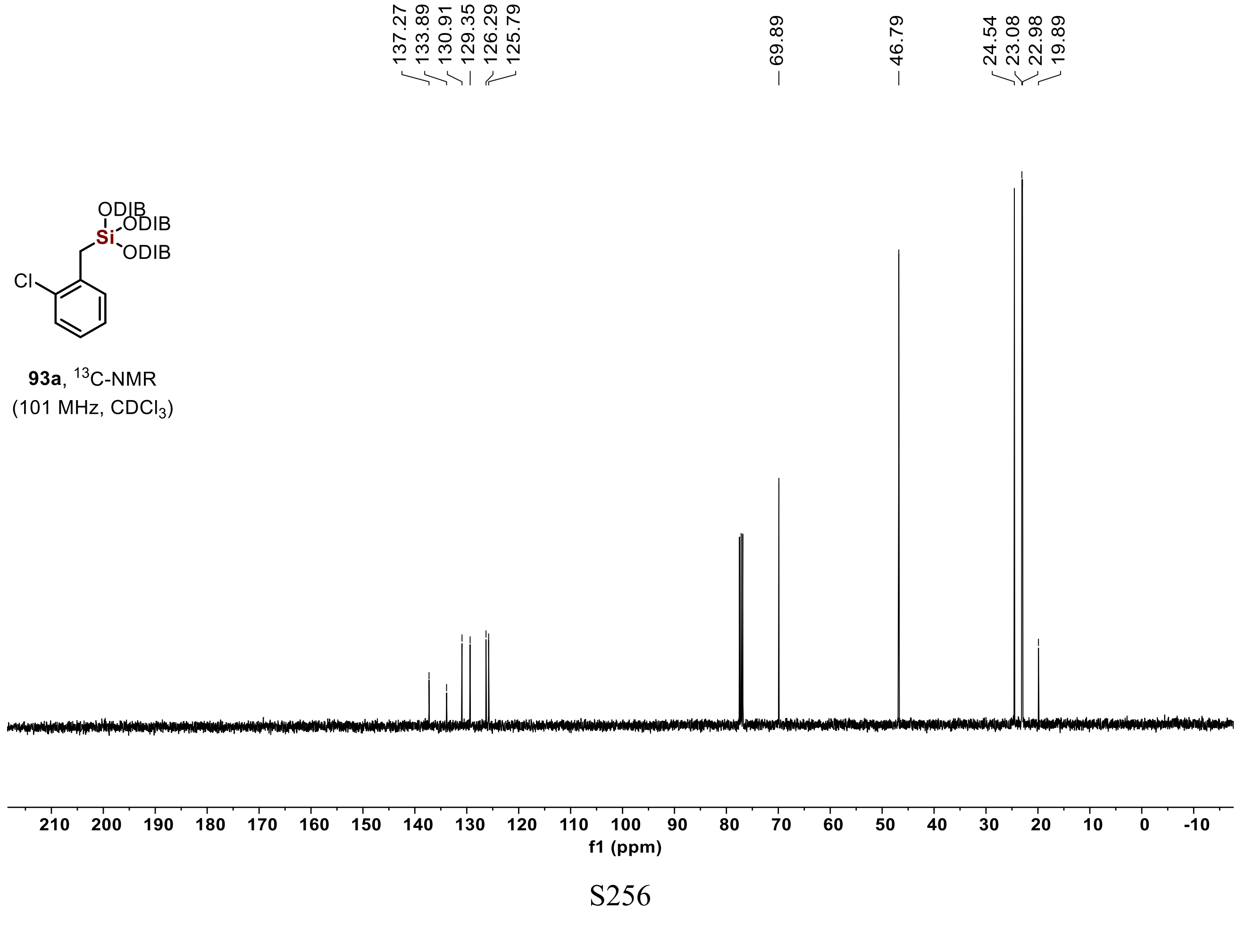

93a, ^{13}C-NMR
(101 MHz, $CDCl_3$)

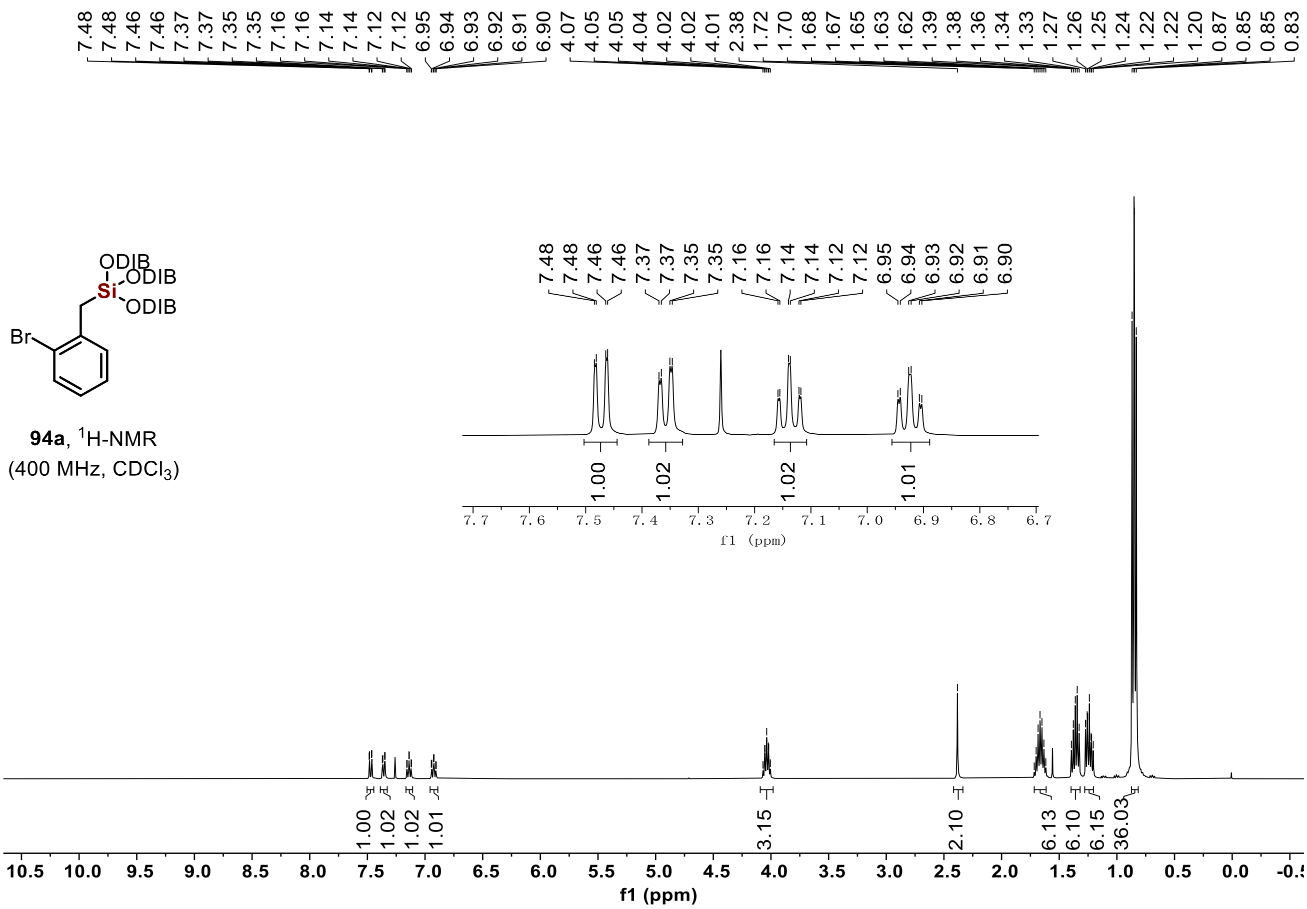

94a, ^{1}H-NMR
(400 MHz, $CDCl_3$)

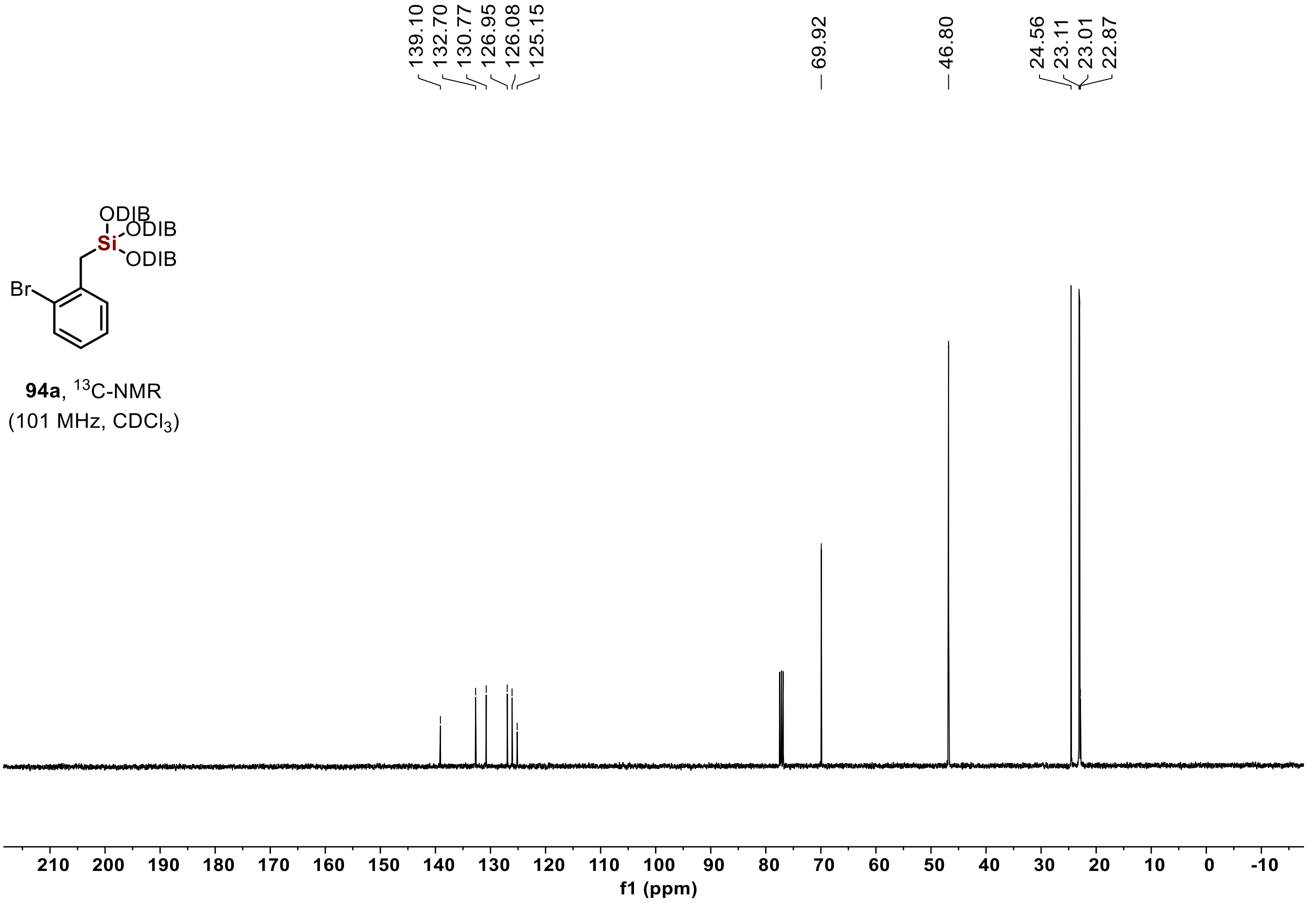

94a, ^{13}C-NMR
(101 MHz, $CDCl_3$)

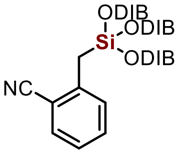

95a, ^{1}H-NMR
(400 MHz, $CDCl_3$)

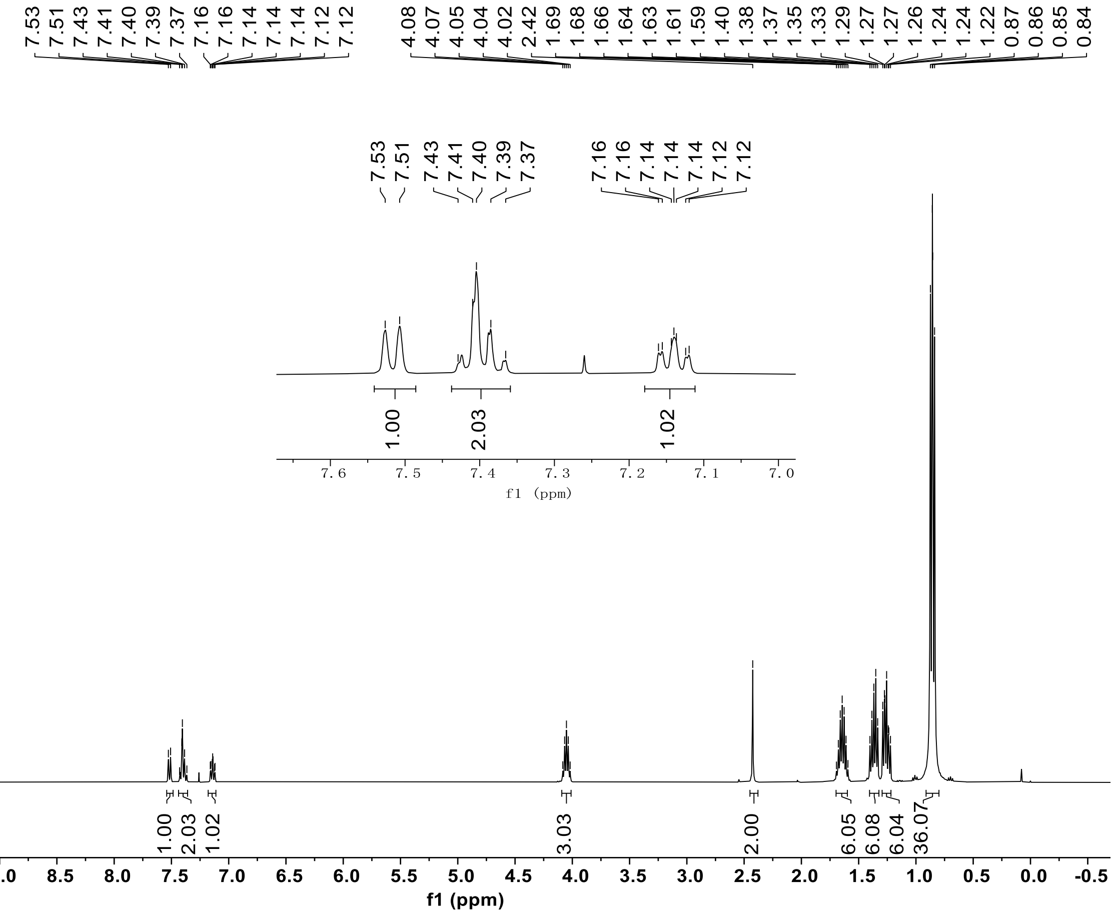

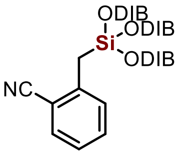

95a, ^{13}C-NMR
(101 MHz, $CDCl_3$)

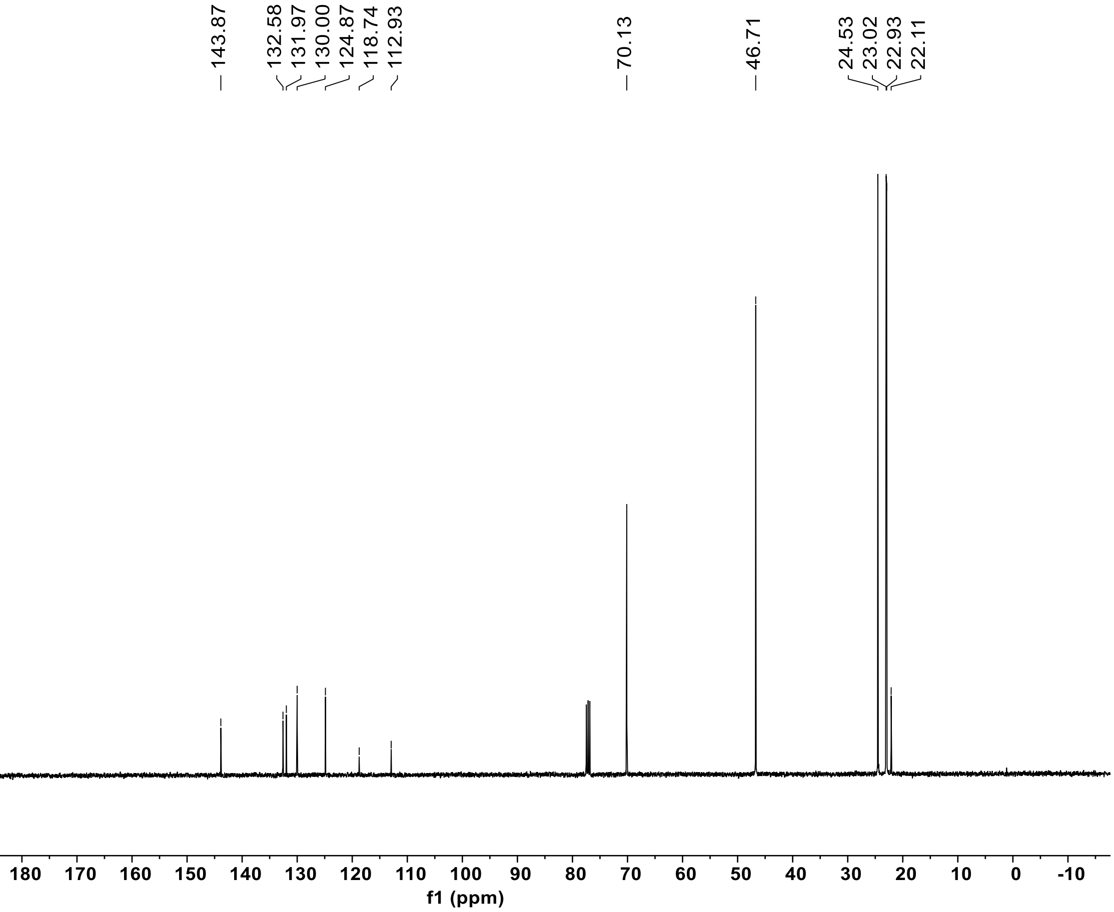

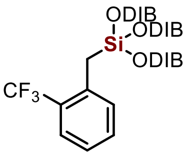

96a, ^{1}H-NMR
(400 MHz, $CDCl_3$)

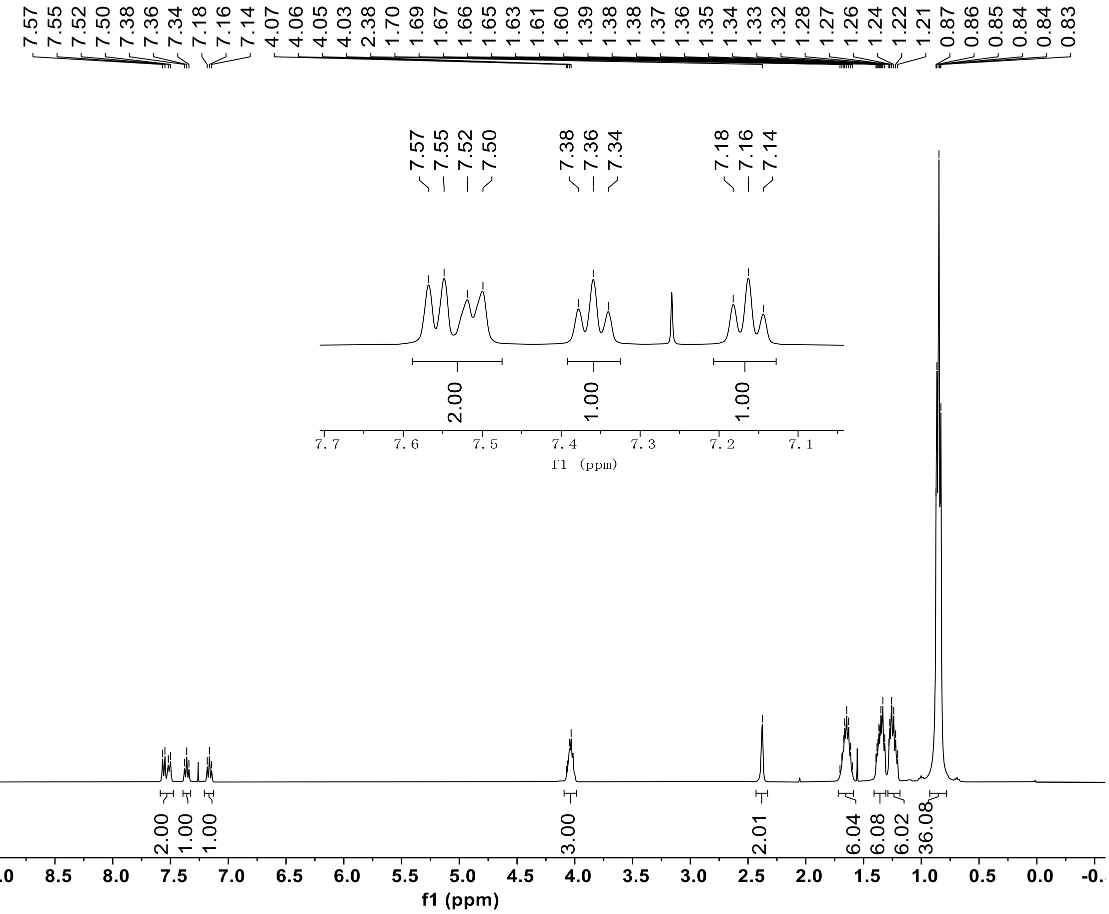

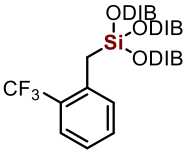

96a, ^{13}C-NMR
(101 MHz, $CDCl_3$)

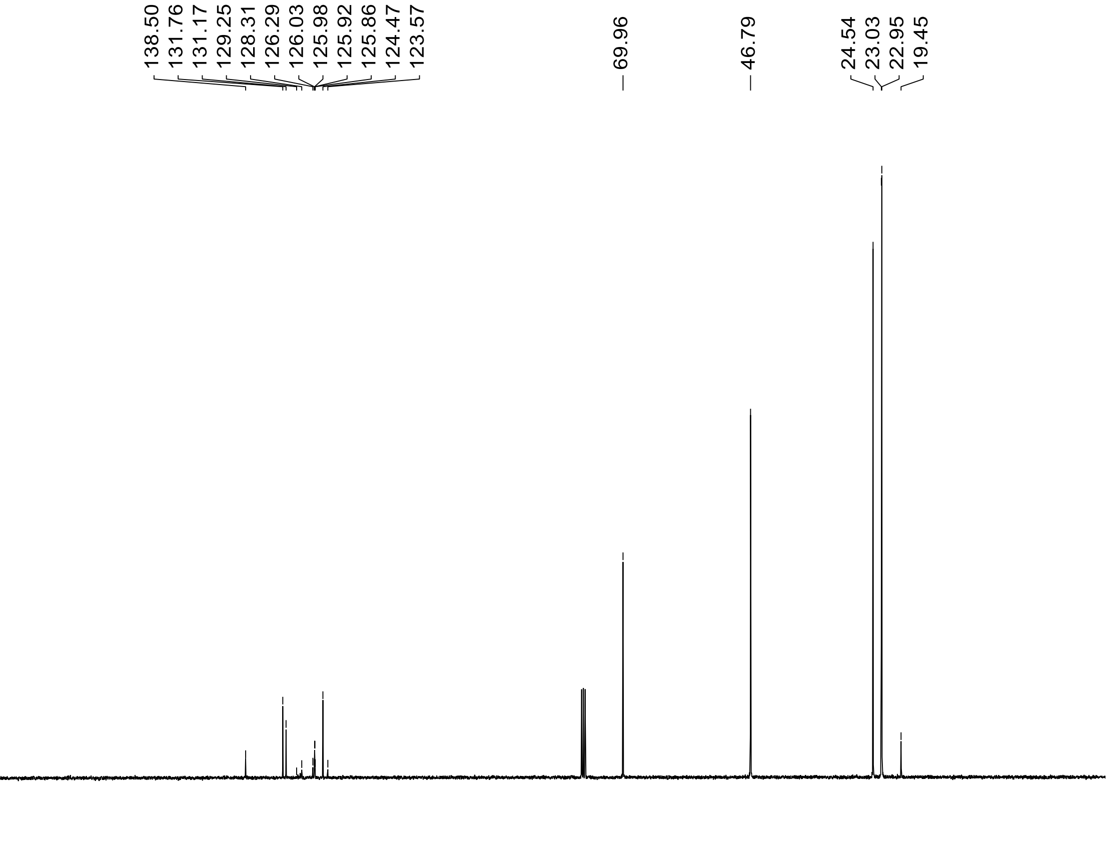

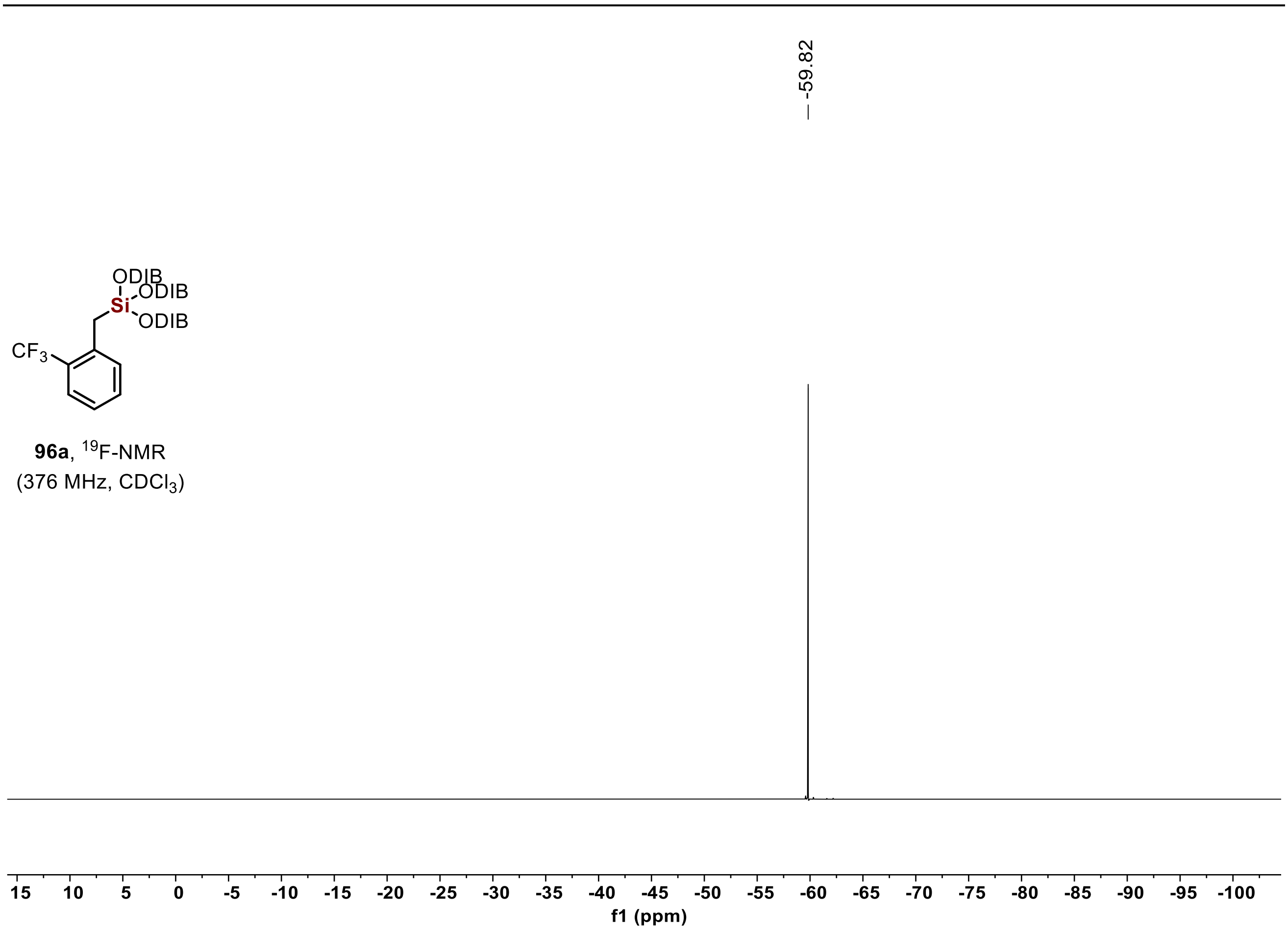

96a, ^{19}F-NMR
(376 MHz, $CDCl_3$)

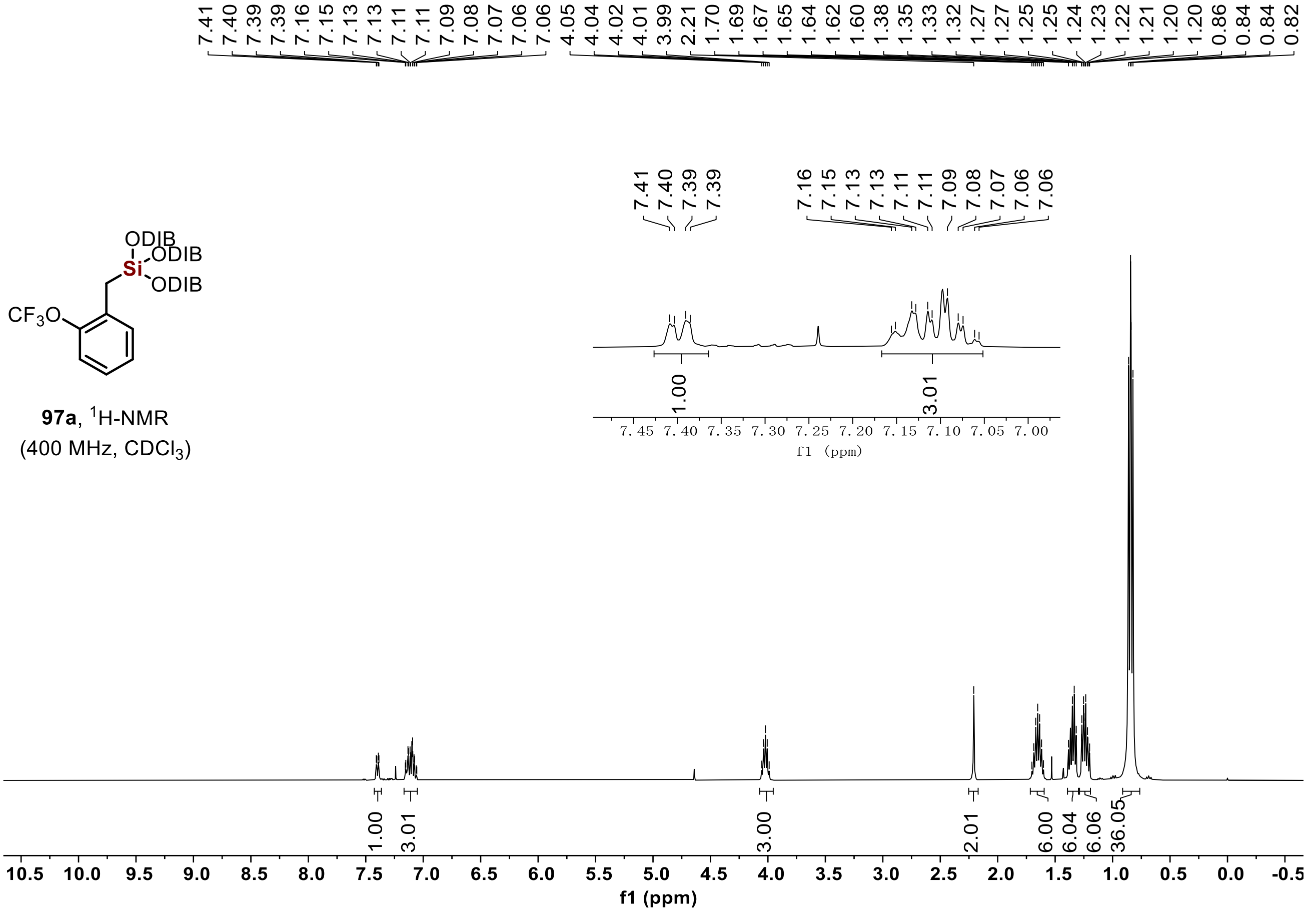

97a, ^{1}H-NMR
(400 MHz, $CDCl_3$)

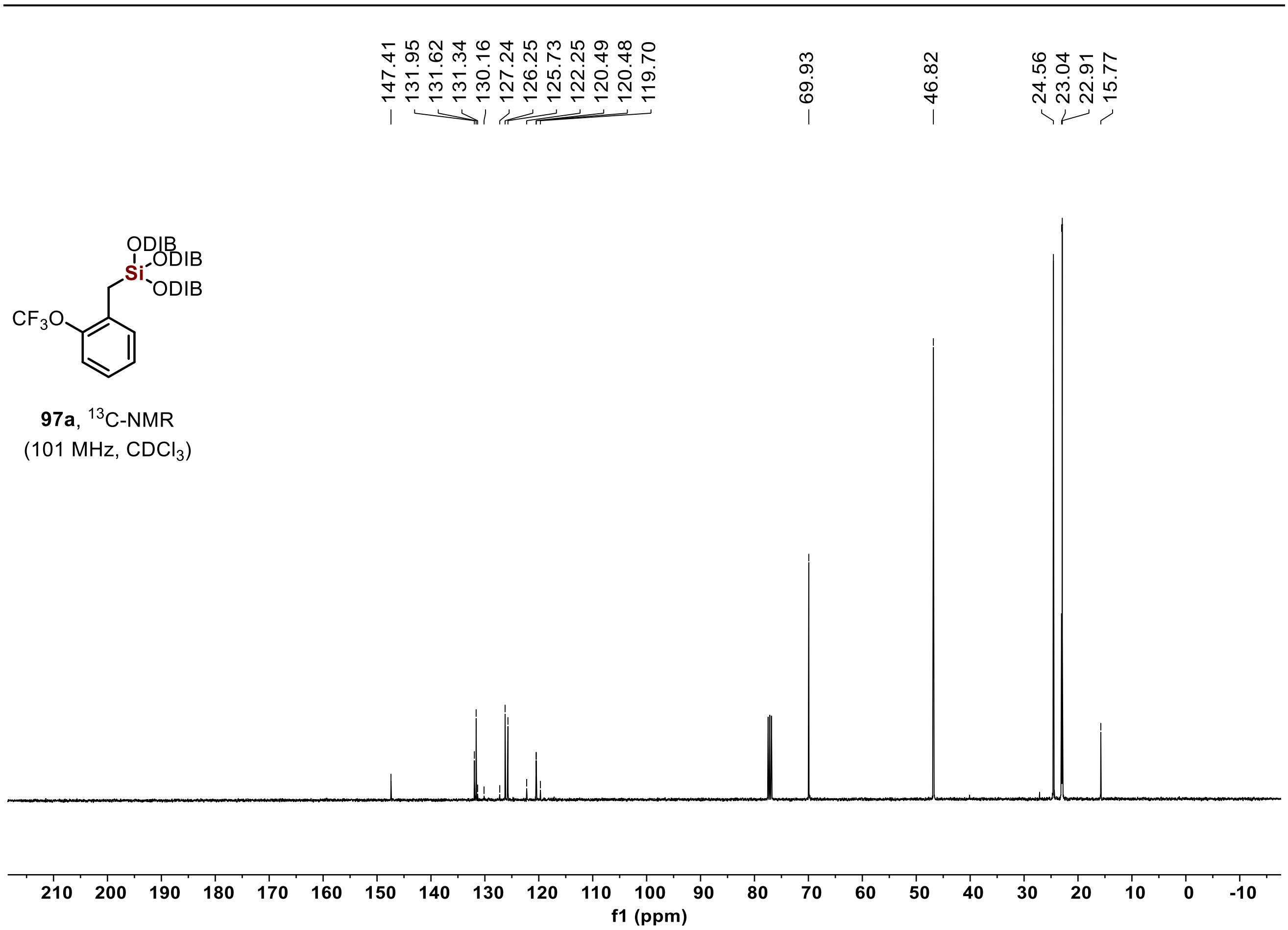

97a, ^{13}C-NMR
(101 MHz, $CDCl_3$)

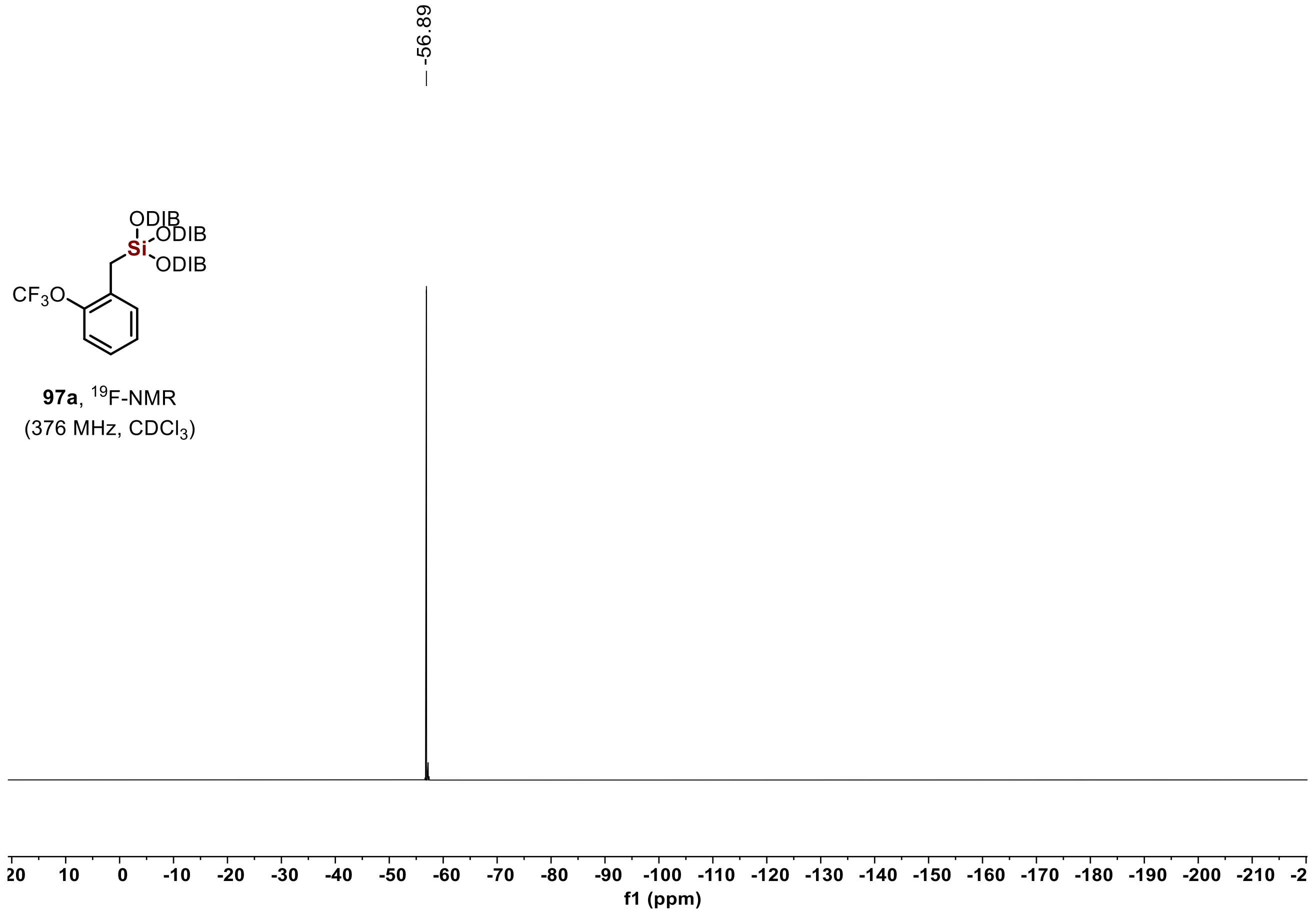

97a, ^{19}F-NMR
(376 MHz, $CDCl_3$)

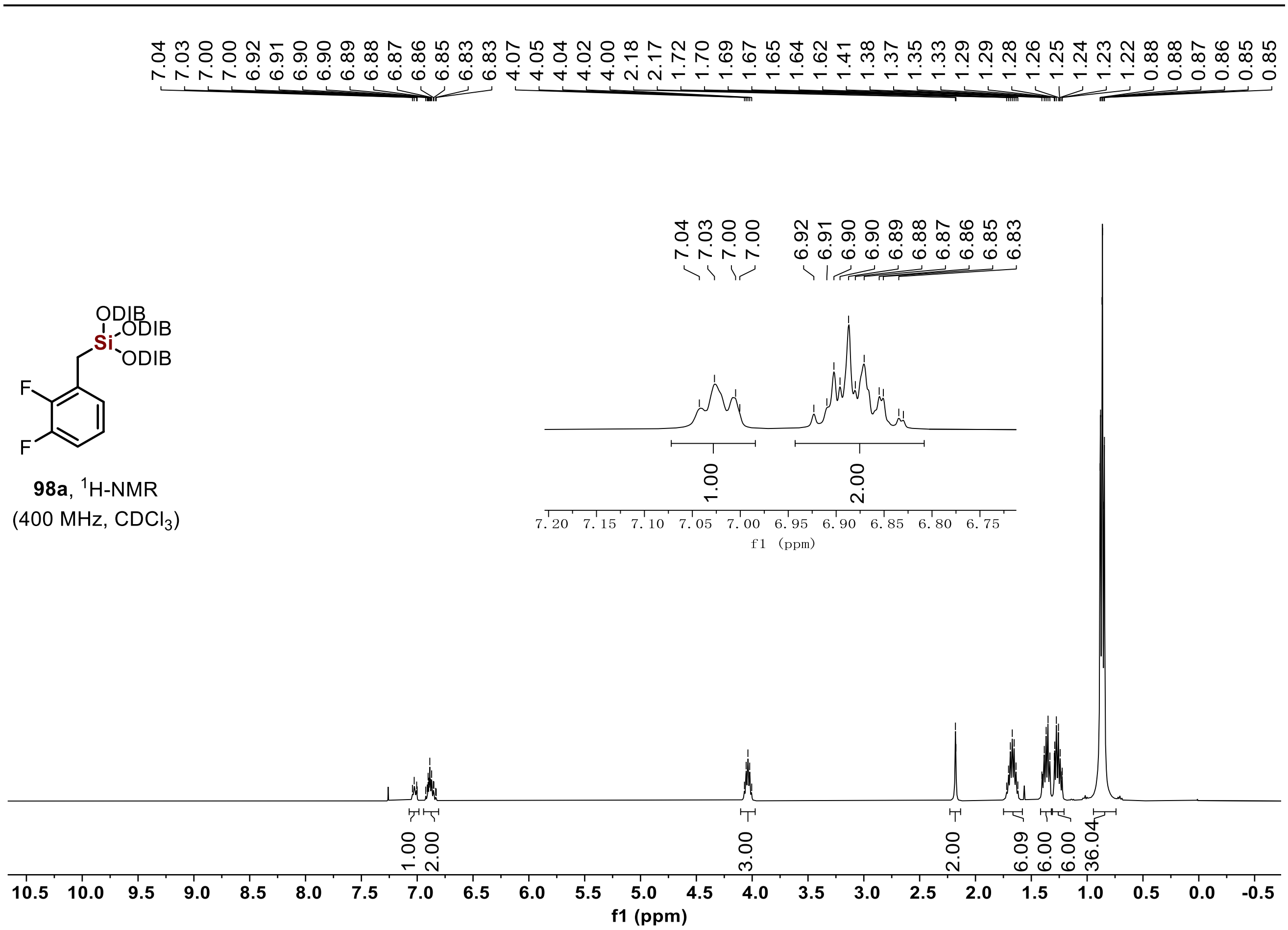
7.04 7.03 7.00 7.00 6.92 6.91 6.90 6.90 6.89 6.88 6.87 6.86 6.85 6.83 6.83 4.07 4.05 4.04 4.02 4.00 2.18 2.17 1.72 1.70 1.69 1.67 1.65 1.64 1.62 1.41 1.38 1.37 1.35 1.33 1.29 1.28 1.26 1.25 1.24 1.23 1.22 0.88 0.88 0.87 0.86 0.85 0.85
ODIB
ODIB
Si
ODIB
F
F
98a, 1H-NMR
(400 MHz, CDCl3)
1.00
2.00
f1 (ppm)
1.00 2.00 3.00 2.00 6.09 6.00 6.00 36.04
f1 (ppm)

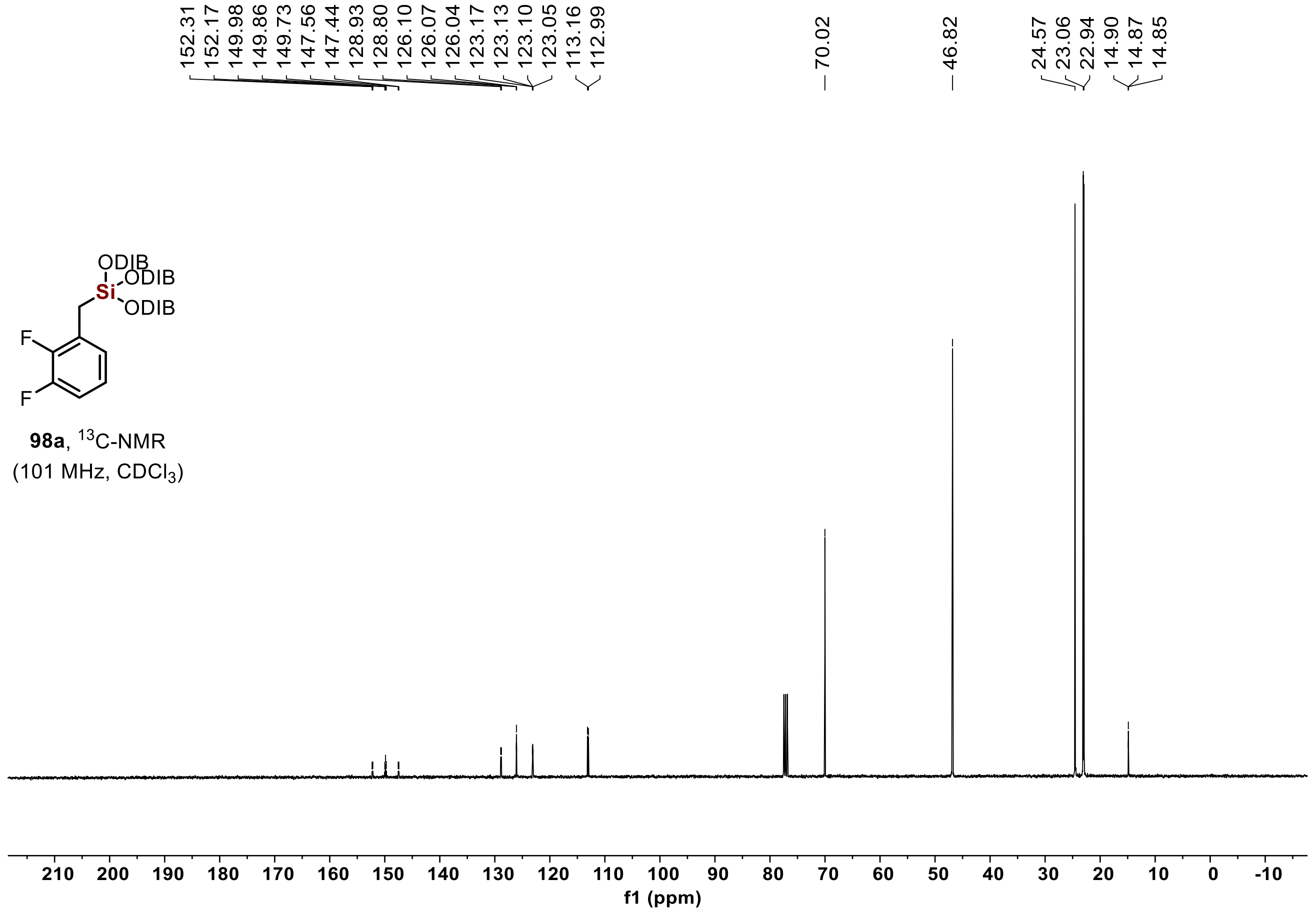
152.31 152.17 149.98 149.86 149.73 147.56 147.44 128.93 128.80 126.10 126.07 126.04 123.17 123.13 123.10 123.05 113.16 112.99
70.02
46.82
24.57 23.06 22.94 14.90 14.87 14.85
ODIB
ODIB
Si
ODIB
F
F
98a, 13C-NMR
(101 MHz, CDCl3)
f1 (ppm)

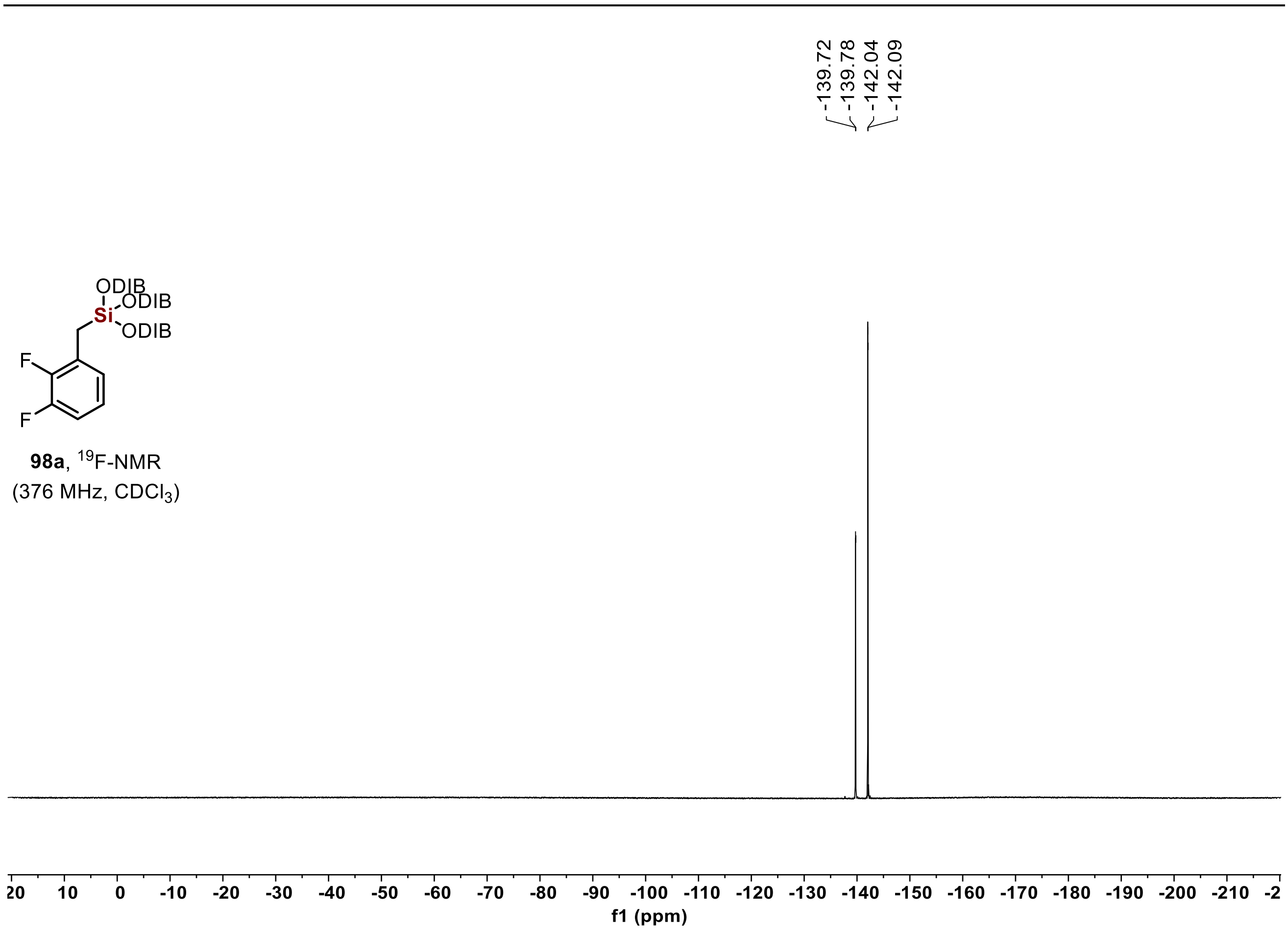

98a, ^{19}F-NMR
(376 MHz, $CDCl_3$)

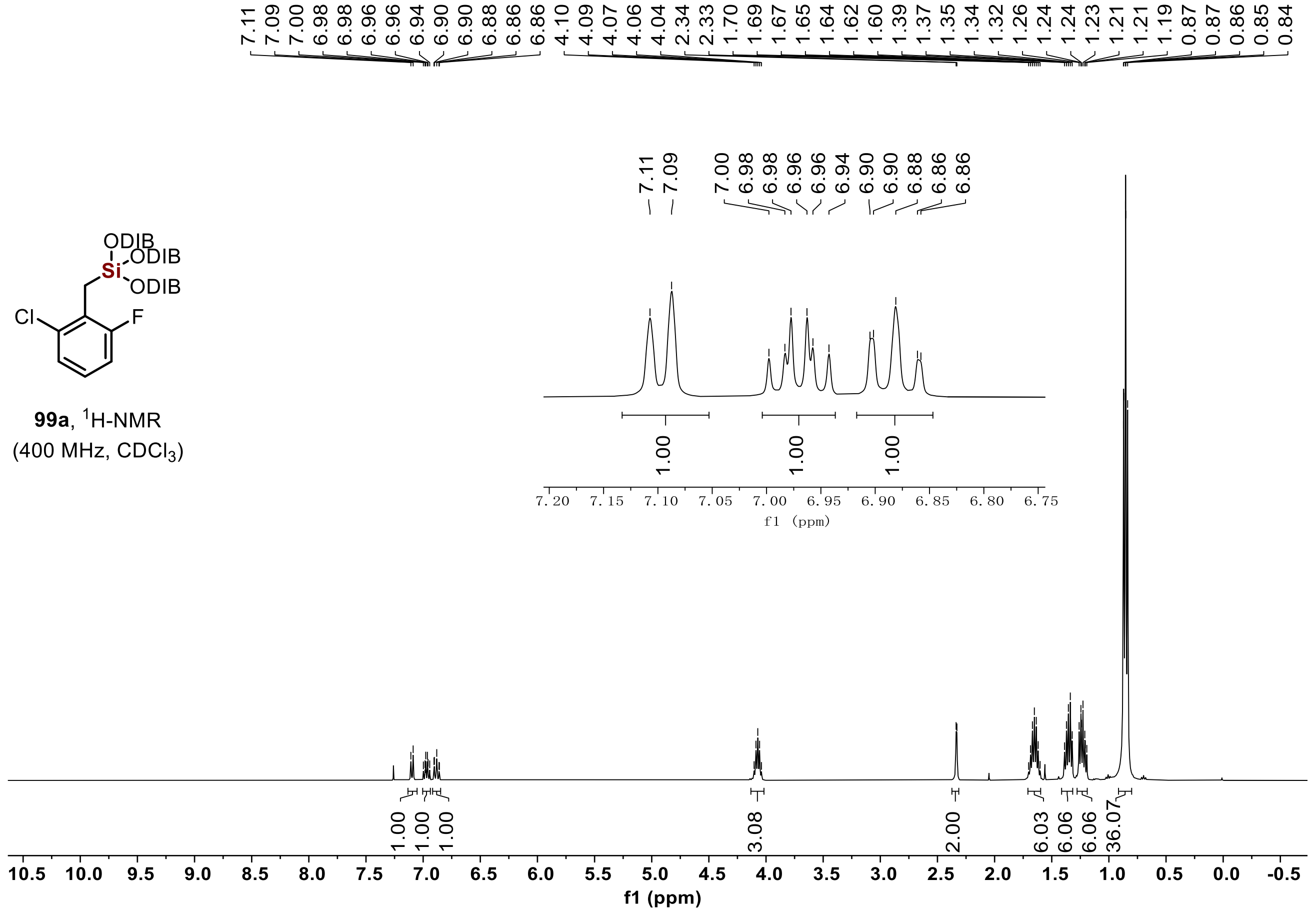

99a, ^{1}H-NMR
(400 MHz, $CDCl_3$)

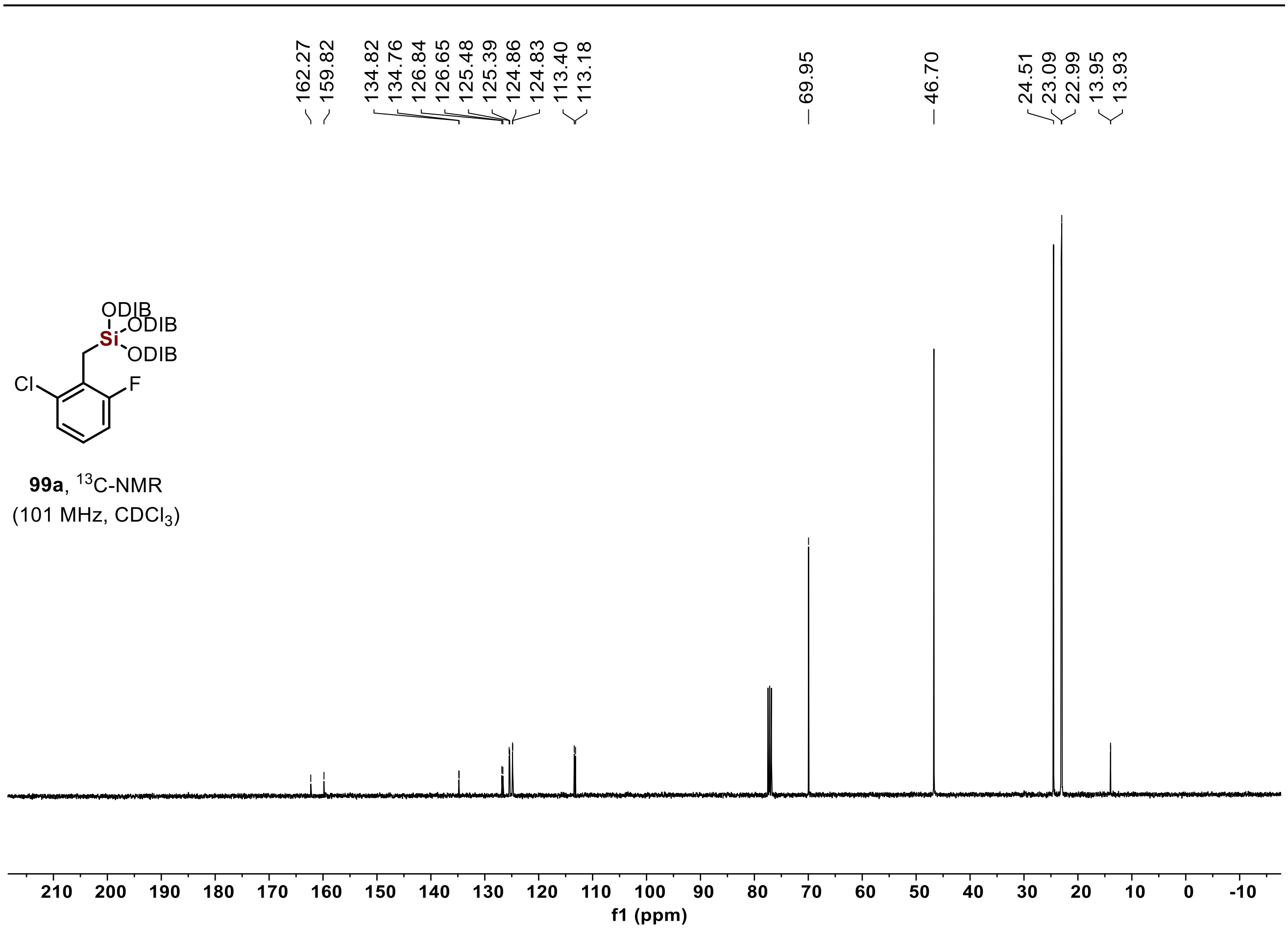

99a, ^{13}C-NMR
(101 MHz, $CDCl_3$)

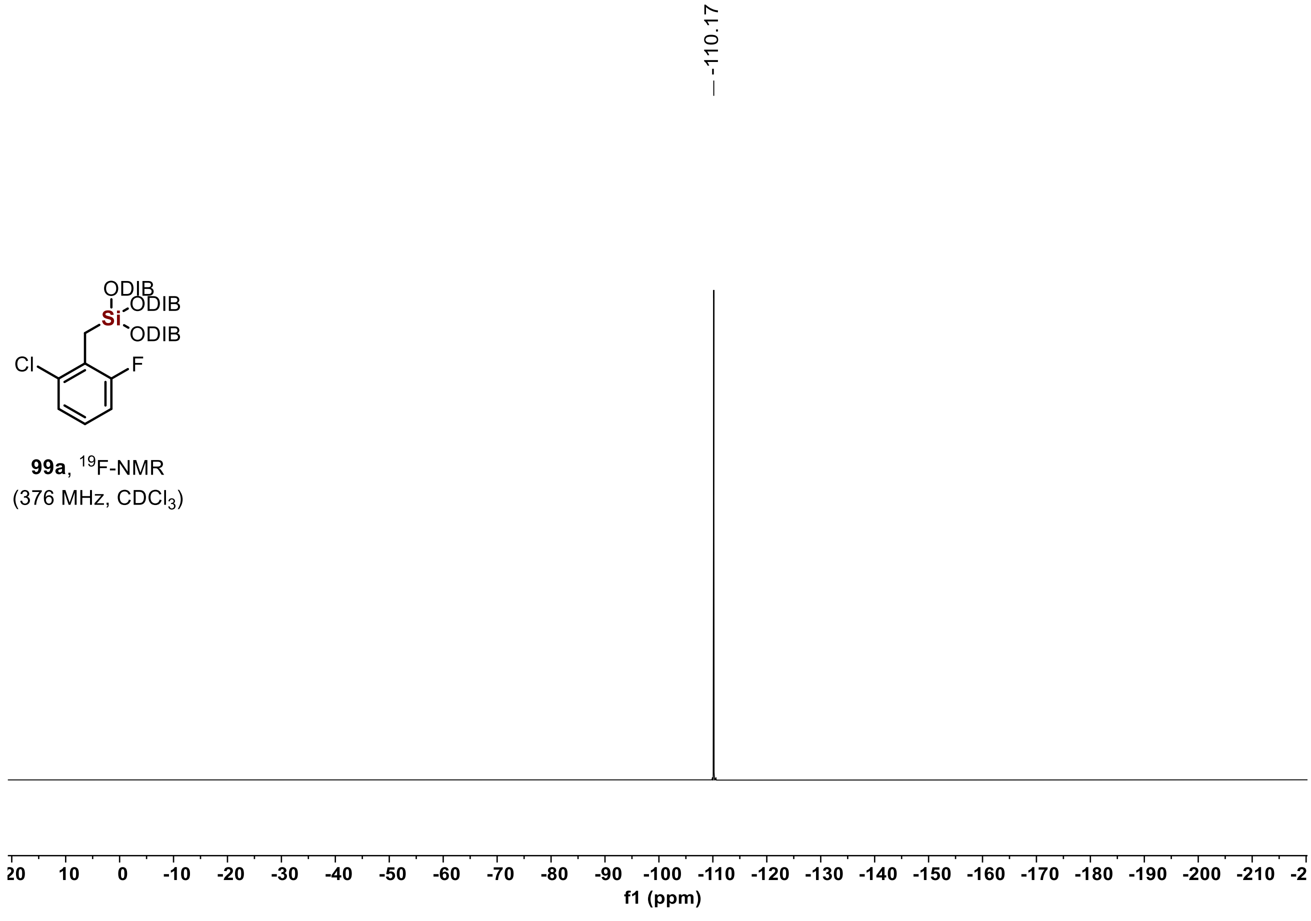

99a, ^{19}F-NMR
(376 MHz, $CDCl_3$)

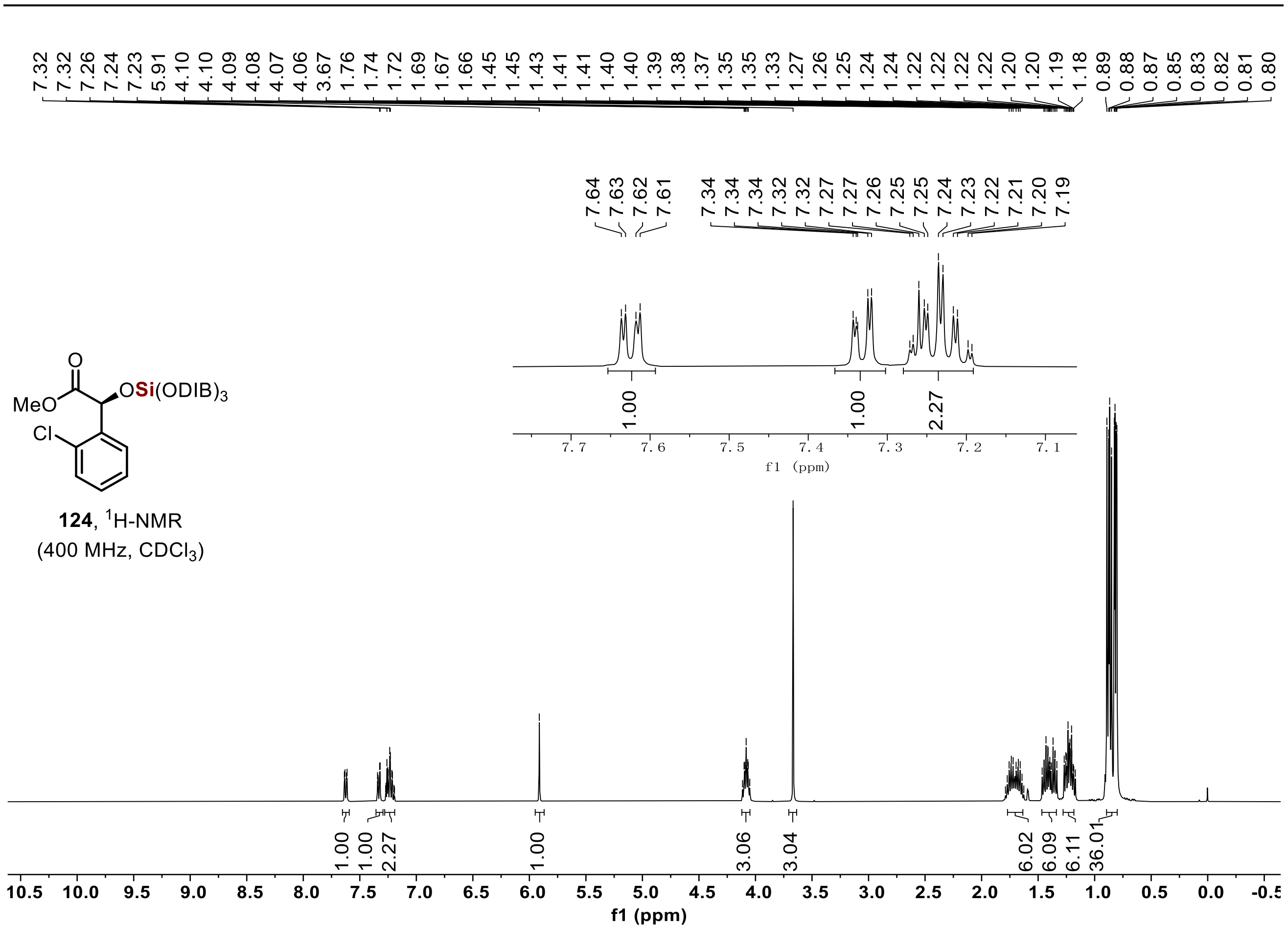

MeO
OSi(ODIB)3
Cl
124, 1H-NMR
(400 MHz, CDCl3)
f1 (ppm)

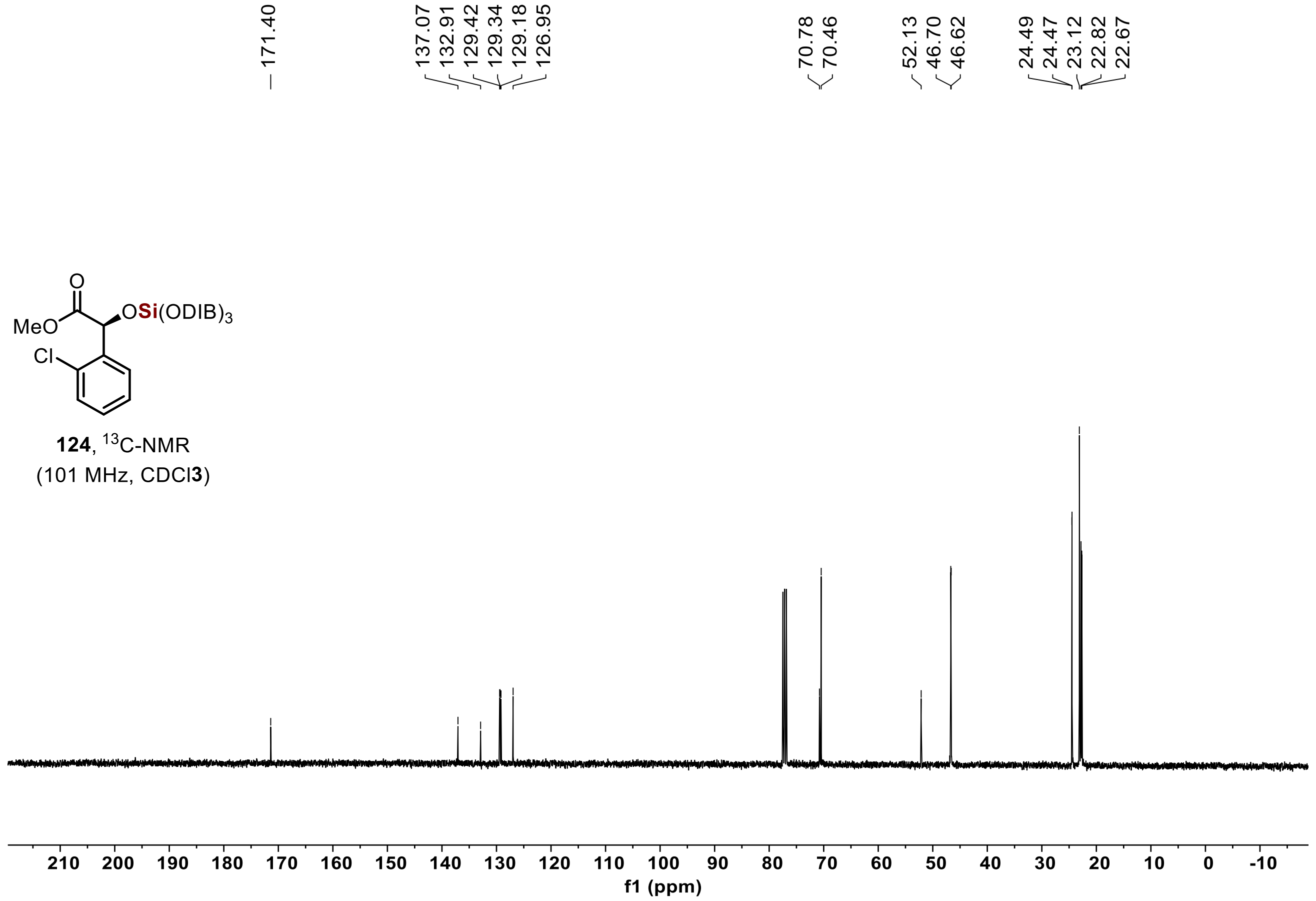

MeO
OSi(ODIB)3
Cl
124, 13C-NMR
(101 MHz, CDCl3)
f1 (ppm)

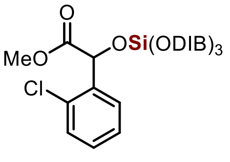

147a, ^{1}H-NMR
(400 MHz, $CDCl_3$)

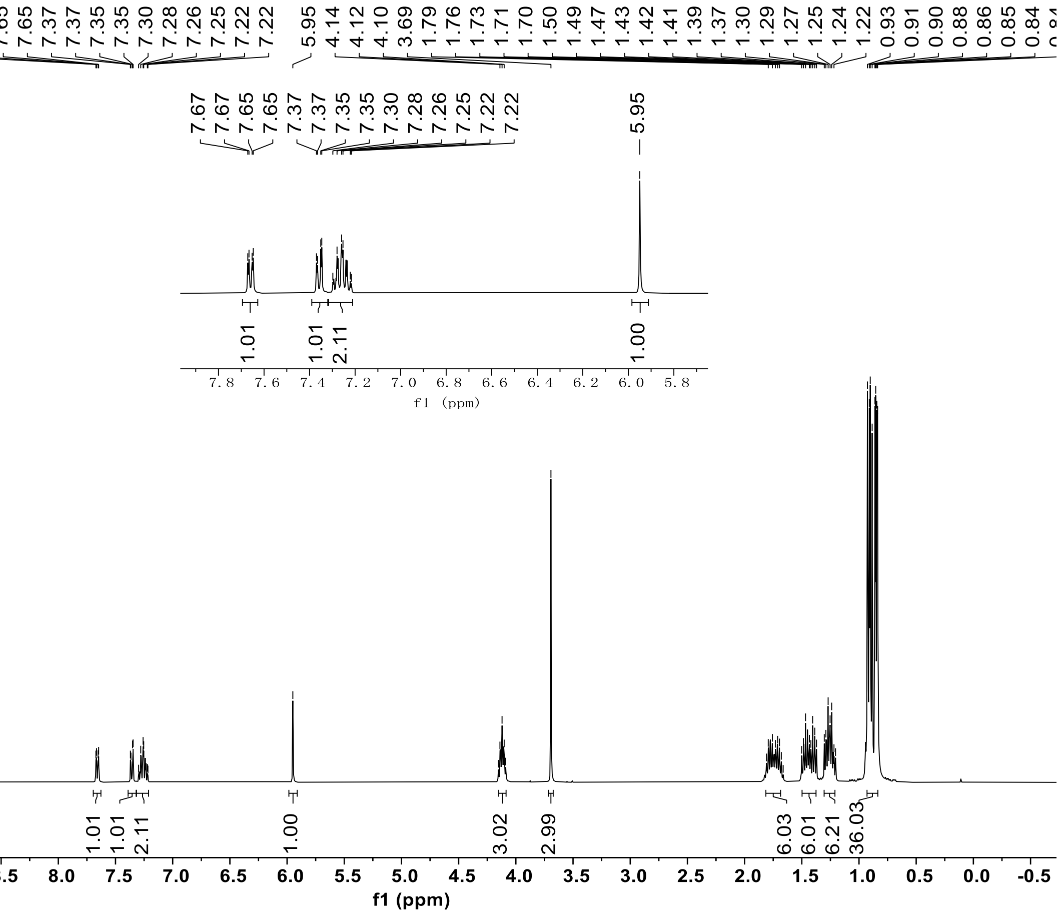

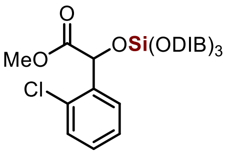

147a, ^{13}C-NMR
(101 MHz, $CDCl_3$)

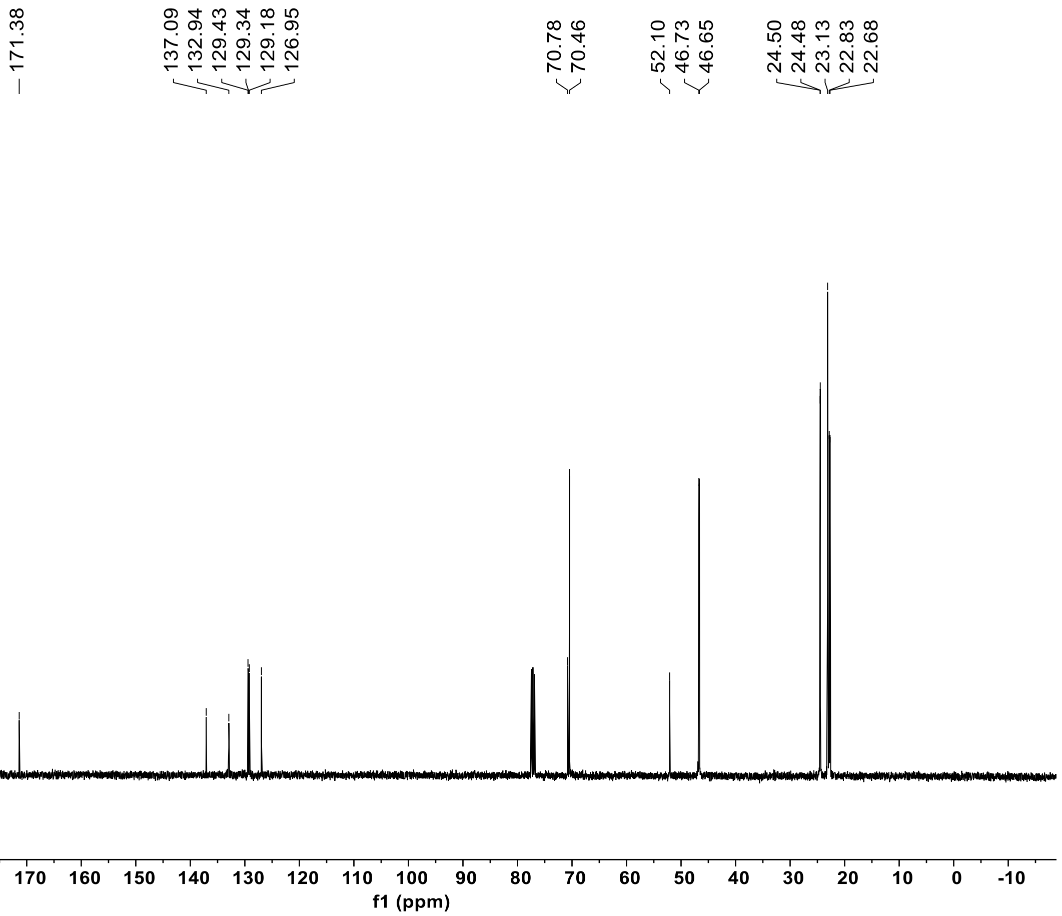

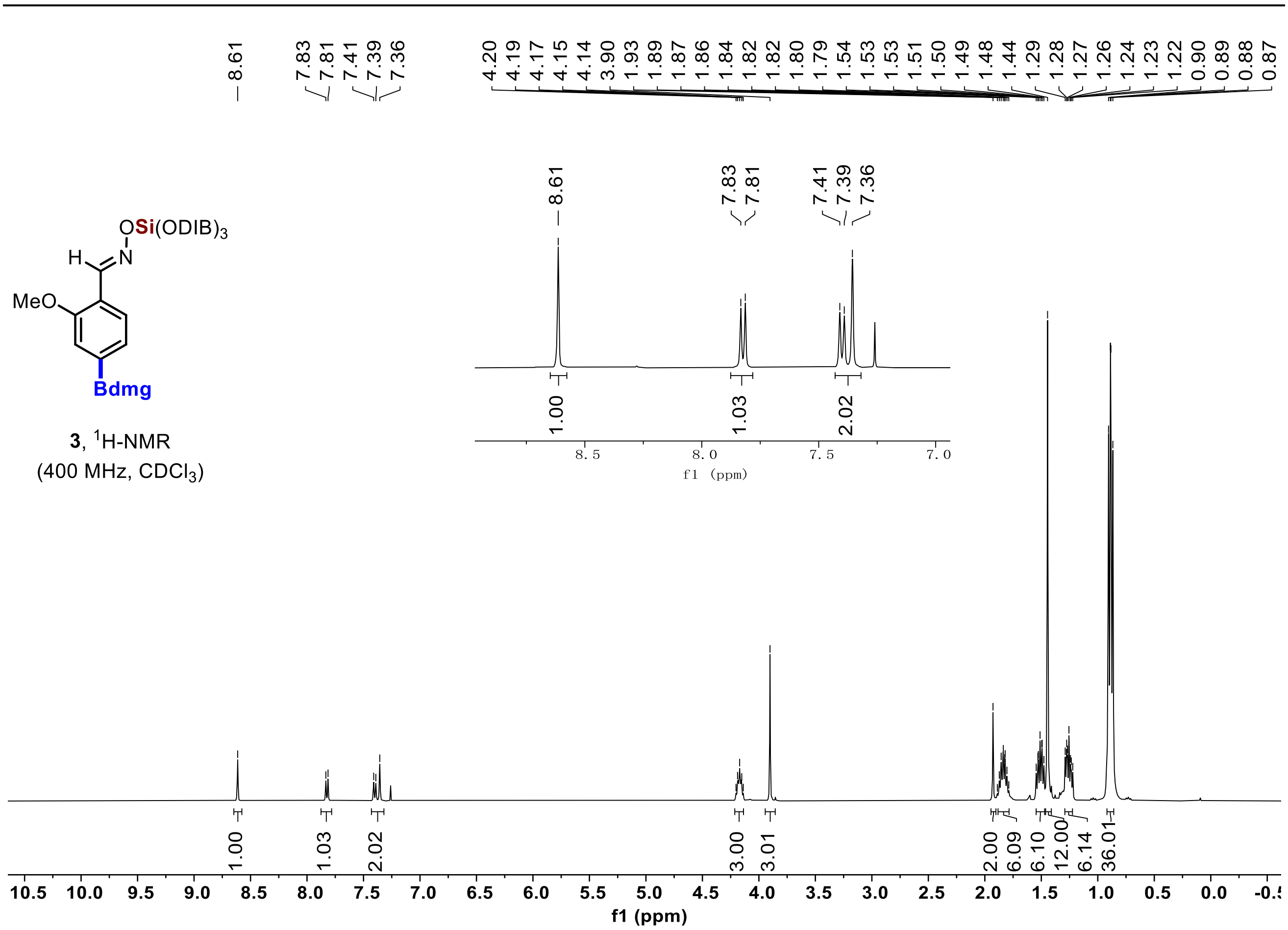

3, ^{1}H-NMR (400 MHz, $CDCl_3$)

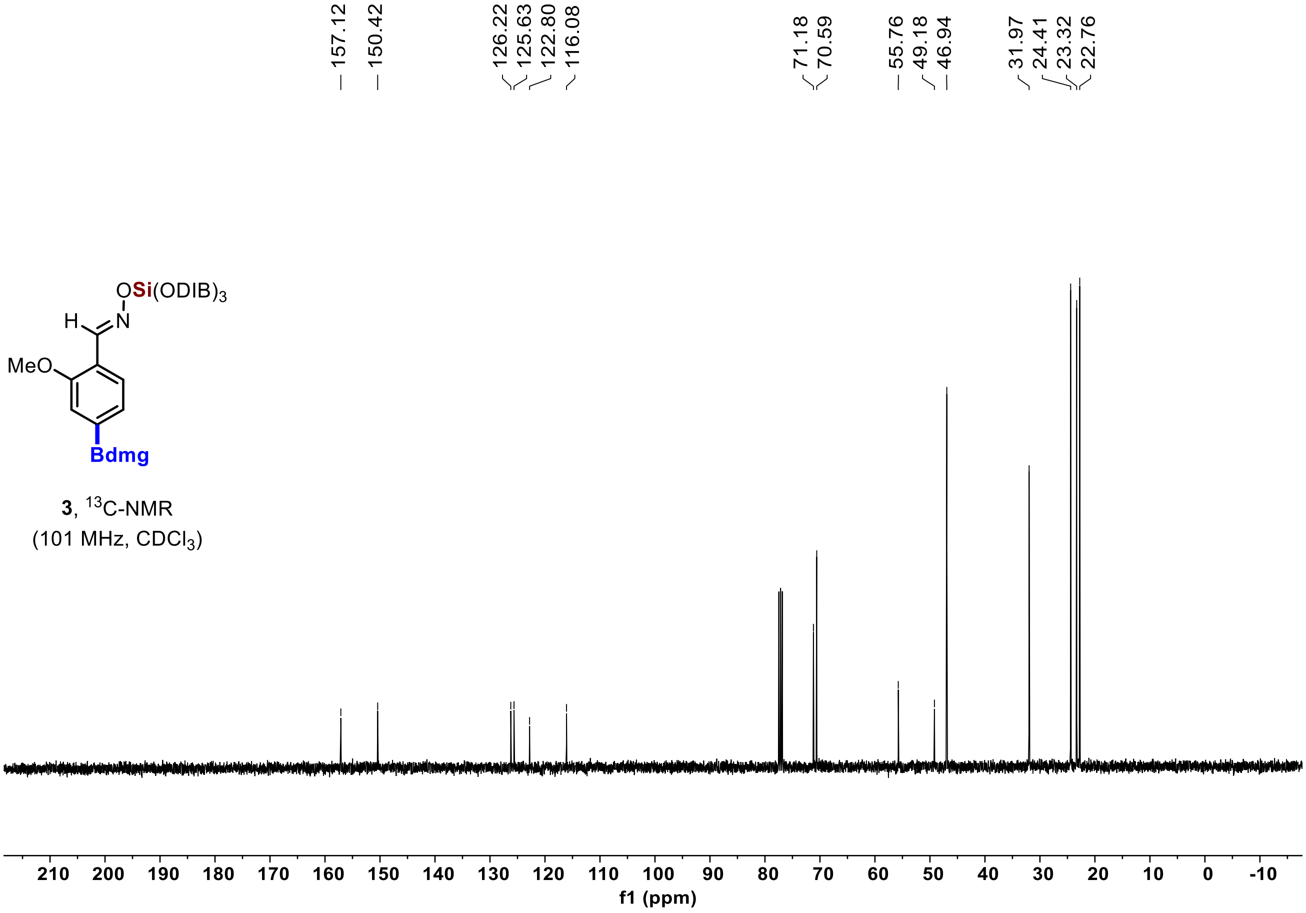

3, ^{13}C-NMR (101 MHz, $CDCl_3$)

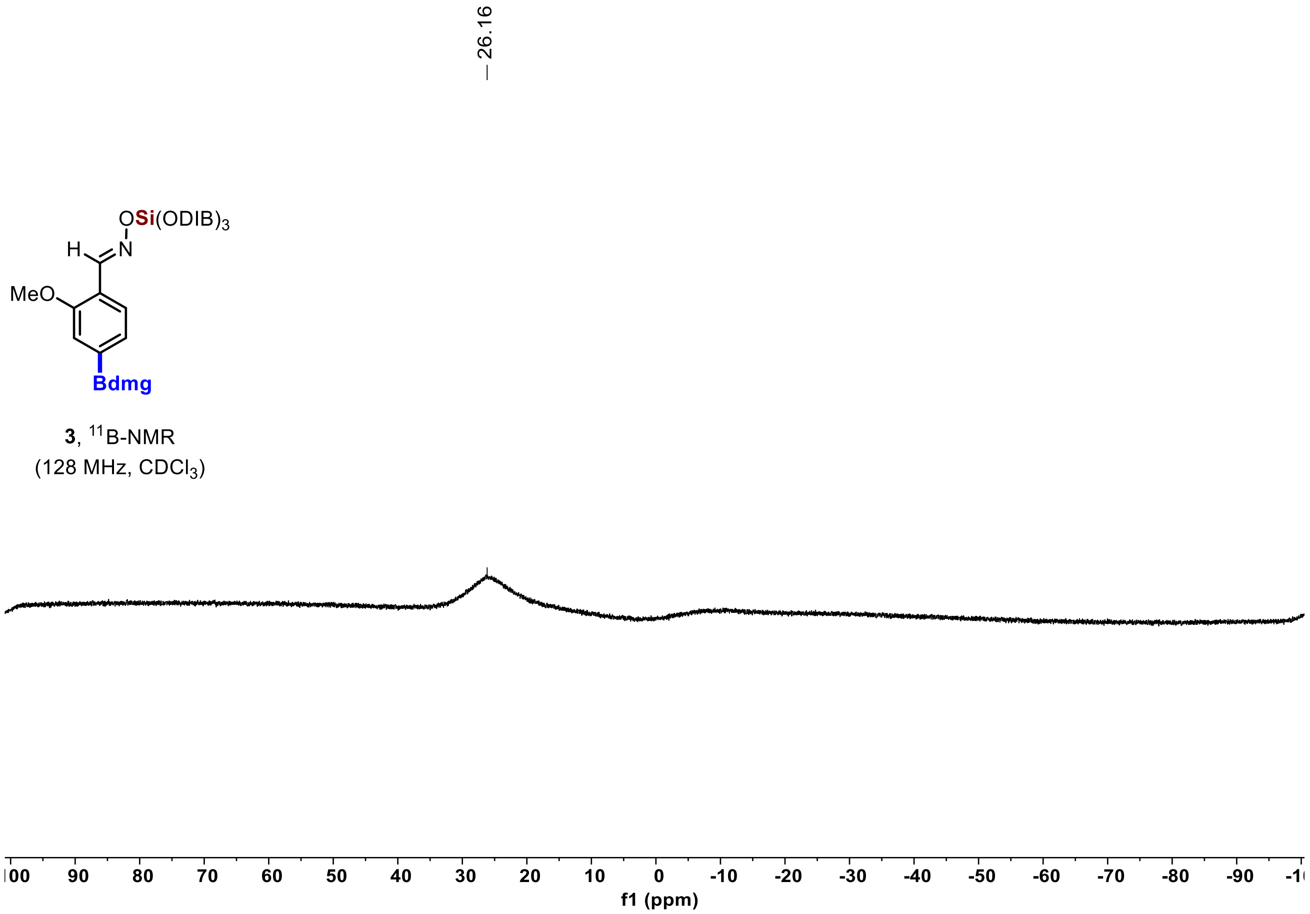

NOESY spectrum of 3 (400 MHz, CDCl₃)

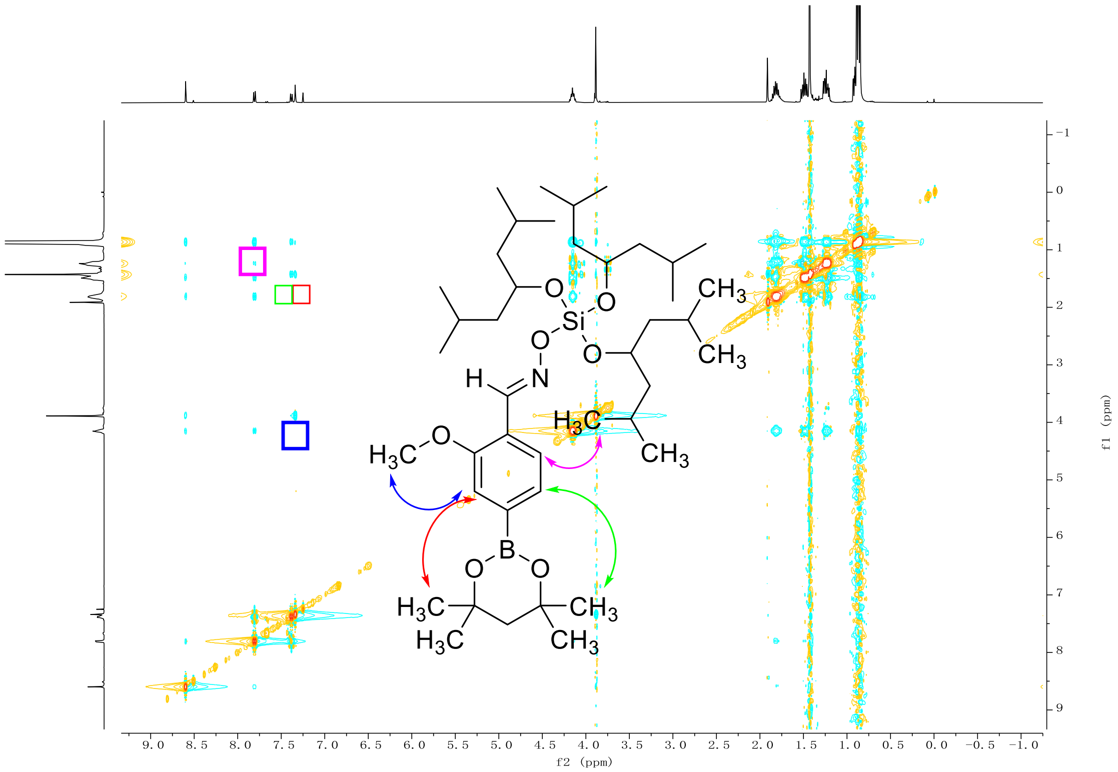

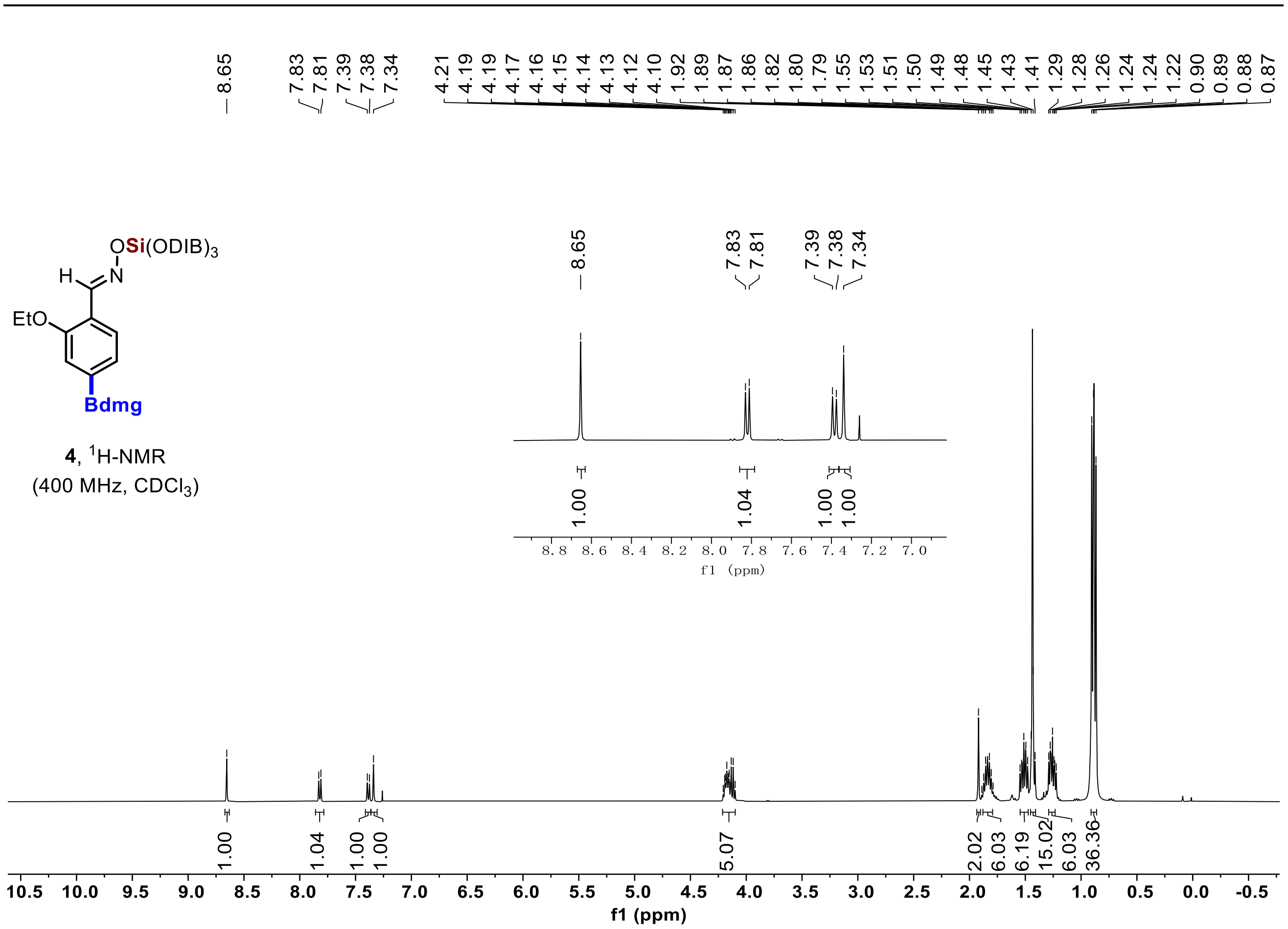
OSi(ODIB)3
H
N
EtO
Bdmg
4, 1H-NMR
(400 MHz, CDCl3)
8.65
7.83
7.81
7.39
7.38
7.34
1.00
1.04
1.00
1.00
5.07
2.02
6.03
6.19
15.02
6.03
36.36
f1 (ppm)

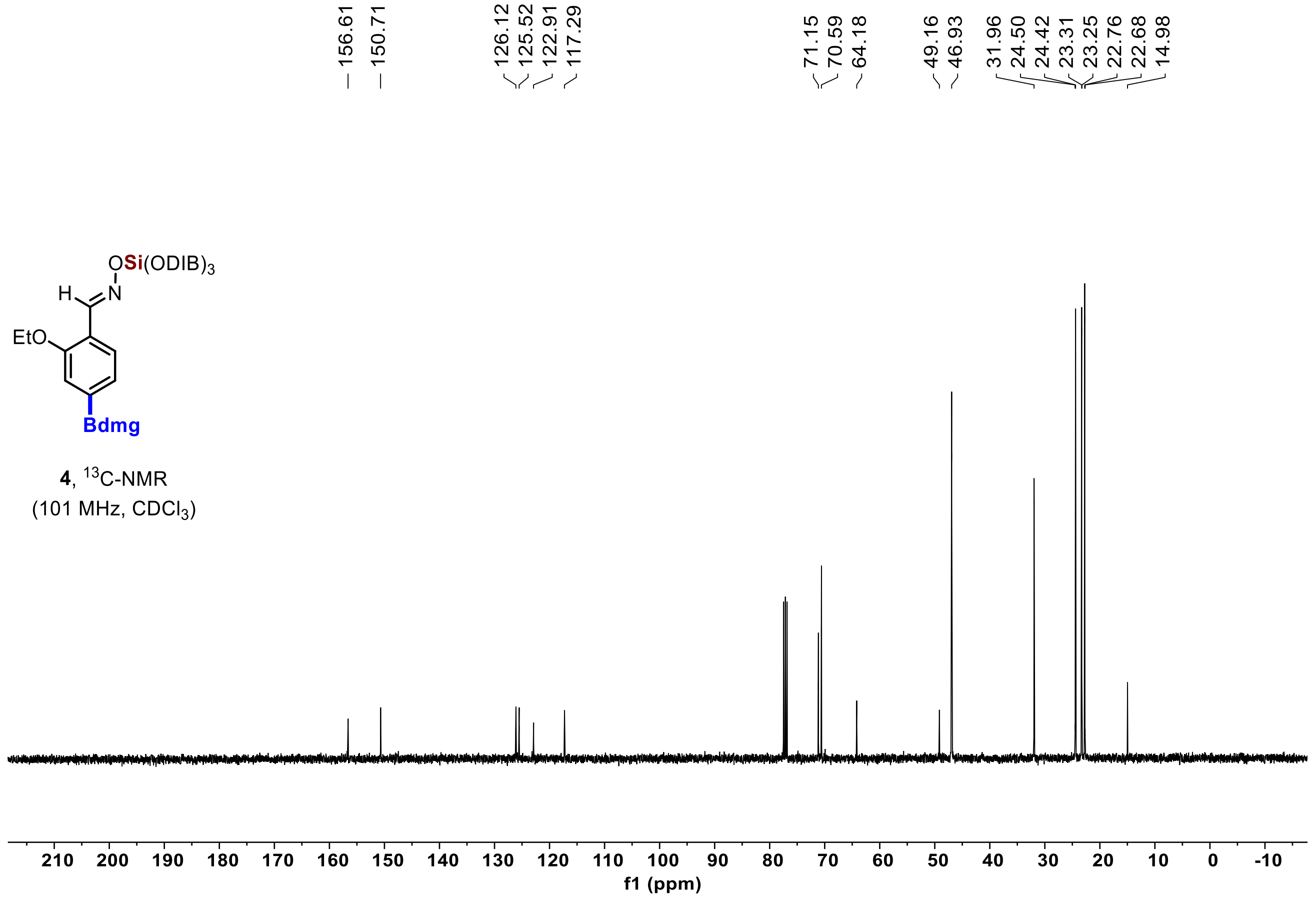
156.61
150.71
126.12
125.52
122.91
117.29
71.15
70.59
64.18
49.16
46.93
31.96
24.50
24.42
23.31
23.25
22.76
22.68
14.98
OSi(ODIB)3
H
N
EtO
Bdmg
4, 13C-NMR
(101 MHz, CDCl3)
f1 (ppm)

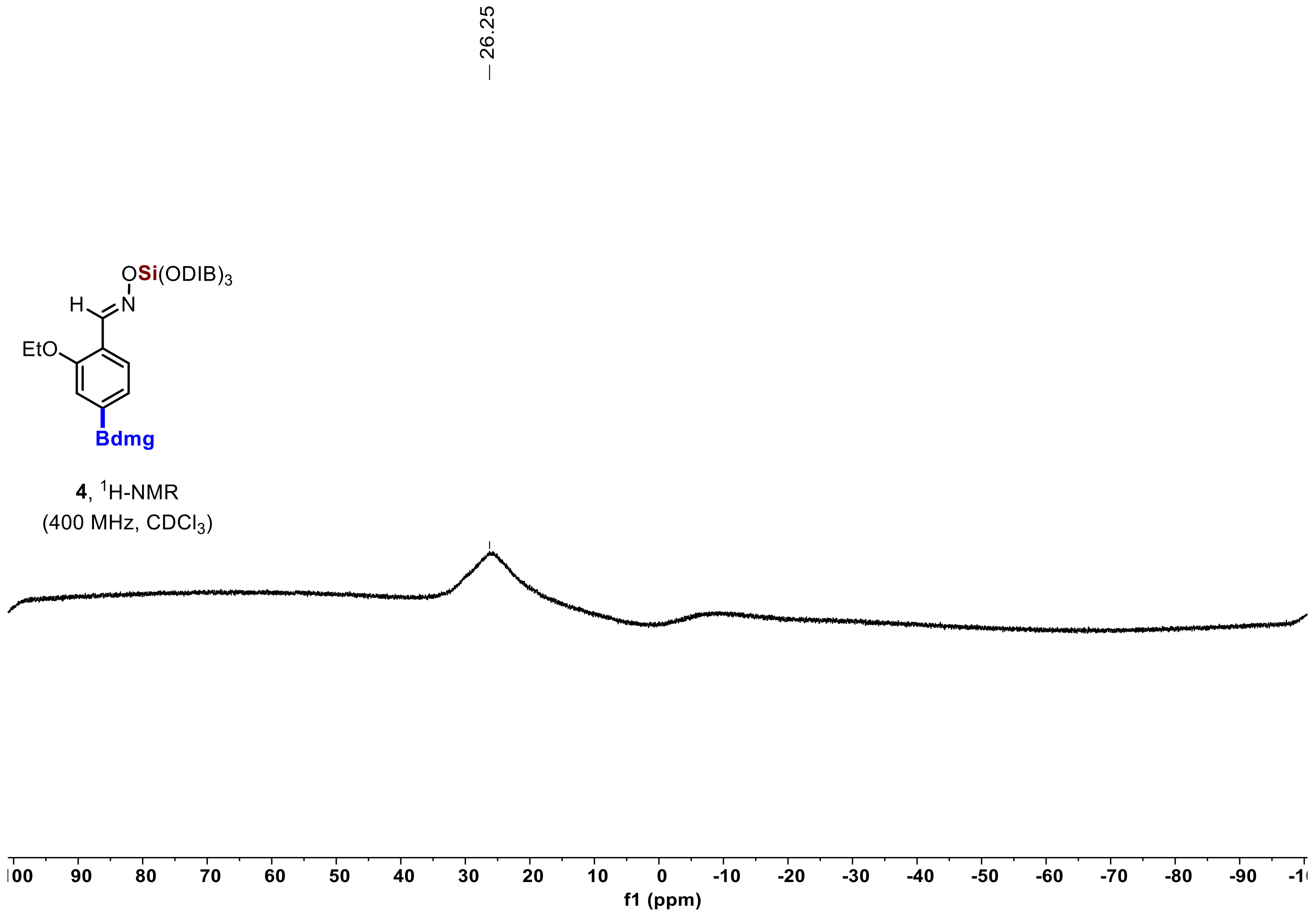

26.25
OSi(ODIB)3
H
N
EtO
Bdmg
4, 1H-NMR
(400 MHz, CDCl3)
100 90 80 70 60 50 40 30 20 10 0 -10 -20 -30 -40 -50 -60 -70 -80 -90
f1 (ppm)

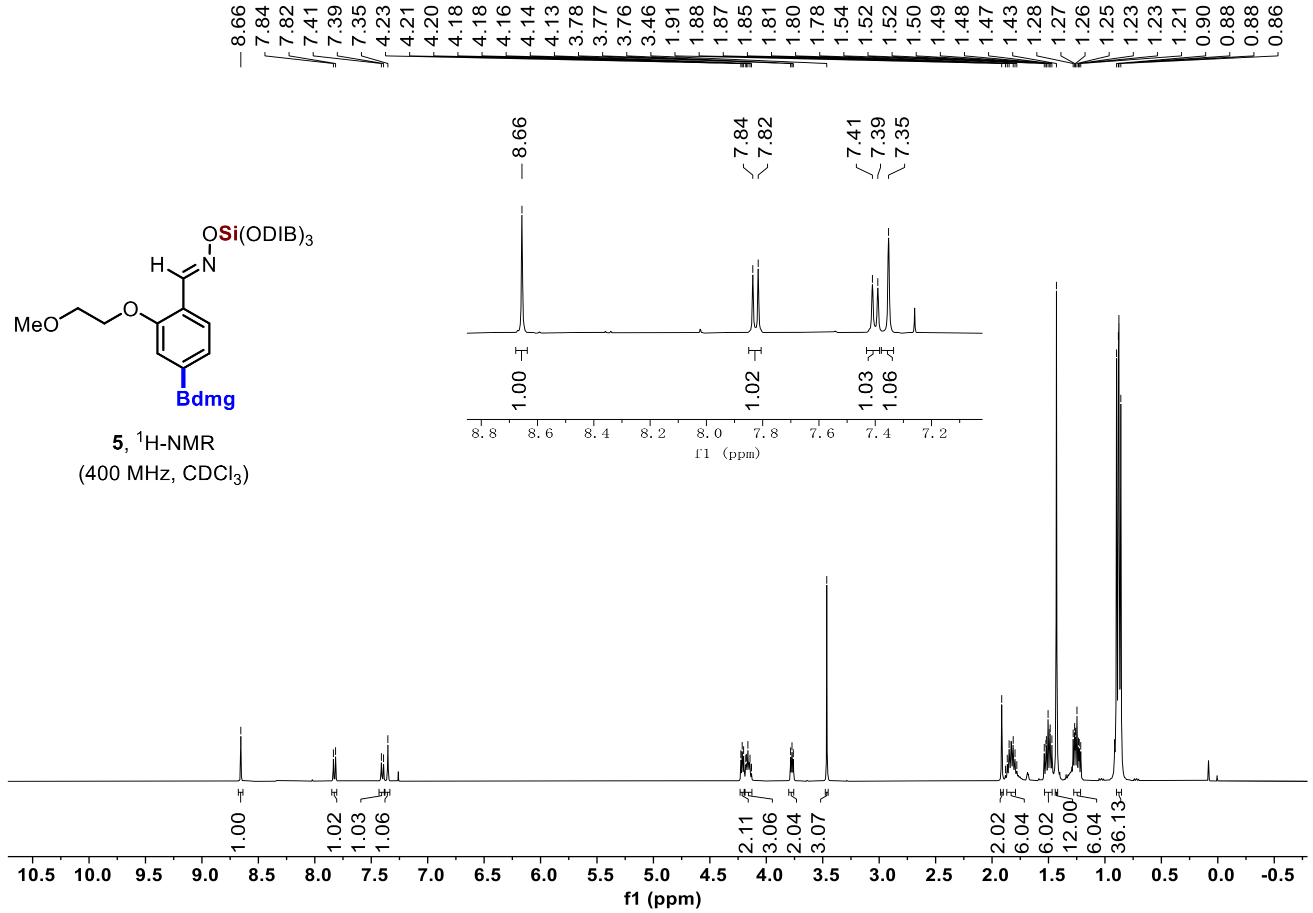

8.66 7.84 7.82 7.41 7.39 7.35 4.23 4.21 4.20 4.18 4.18 4.16 4.14 4.13 3.78 3.77 3.76 3.46 1.91 1.88 1.87 1.85 1.81 1.80 1.78 1.54 1.52 1.52 1.50 1.49 1.48 1.47 1.43 1.28 1.27 1.26 1.25 1.23 1.23 1.21 0.90 0.88 0.88 0.86
OSi(ODIB)3
H
N
MeO
O
Bdmg
5, 1H-NMR
(400 MHz, CDCl3)
1.00 1.02 1.03 1.06
8.8 8.6 8.4 8.2 8.0 7.8 7.6 7.4 7.2
f1 (ppm)
1.00 1.02 1.03 1.06 2.11 3.06 2.04 3.07 2.02 6.04 6.02 12.00 6.04 36.13
10.5 10.0 9.5 9.0 8.5 8.0 7.5 7.0 6.5 6.0 5.5 5.0 4.5 4.0 3.5 3.0 2.5 2.0 1.5 1.0 0.5 0.0 -0.5
f1 (ppm)

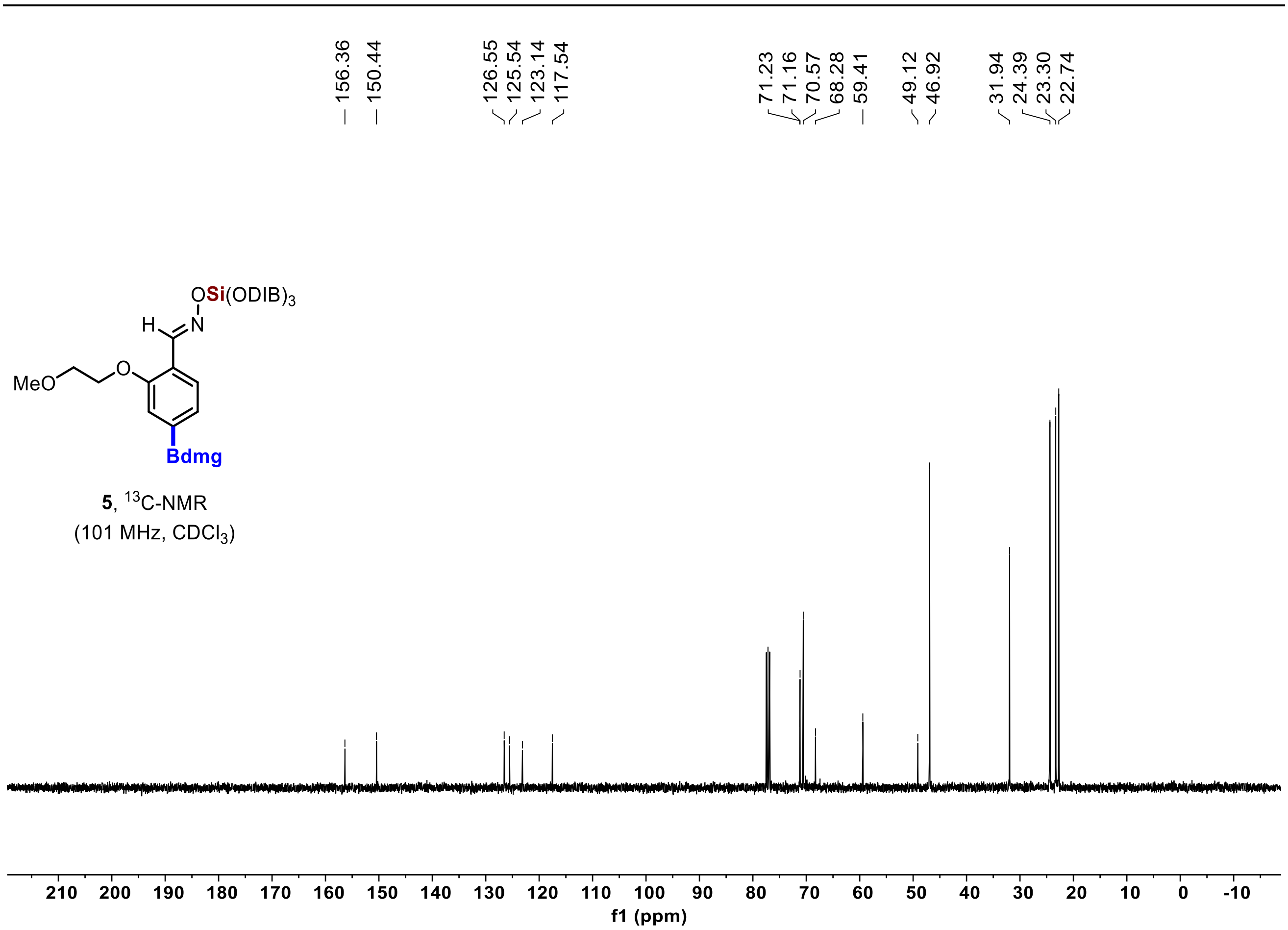
156.36
150.44
126.55
125.54
123.14
117.54
71.23
71.16
70.57
68.28
59.41
49.12
46.92
31.94
24.39
23.30
22.74
OSi(ODIB)3
H
N
MeO
O
Bdmg
5, 13C-NMR
(101 MHz, CDCl3)
210
200
190
180
170
160
150
140
130
120
110
100
90
80
70
60
50
40
30
20
10
0
-10
f1 (ppm)

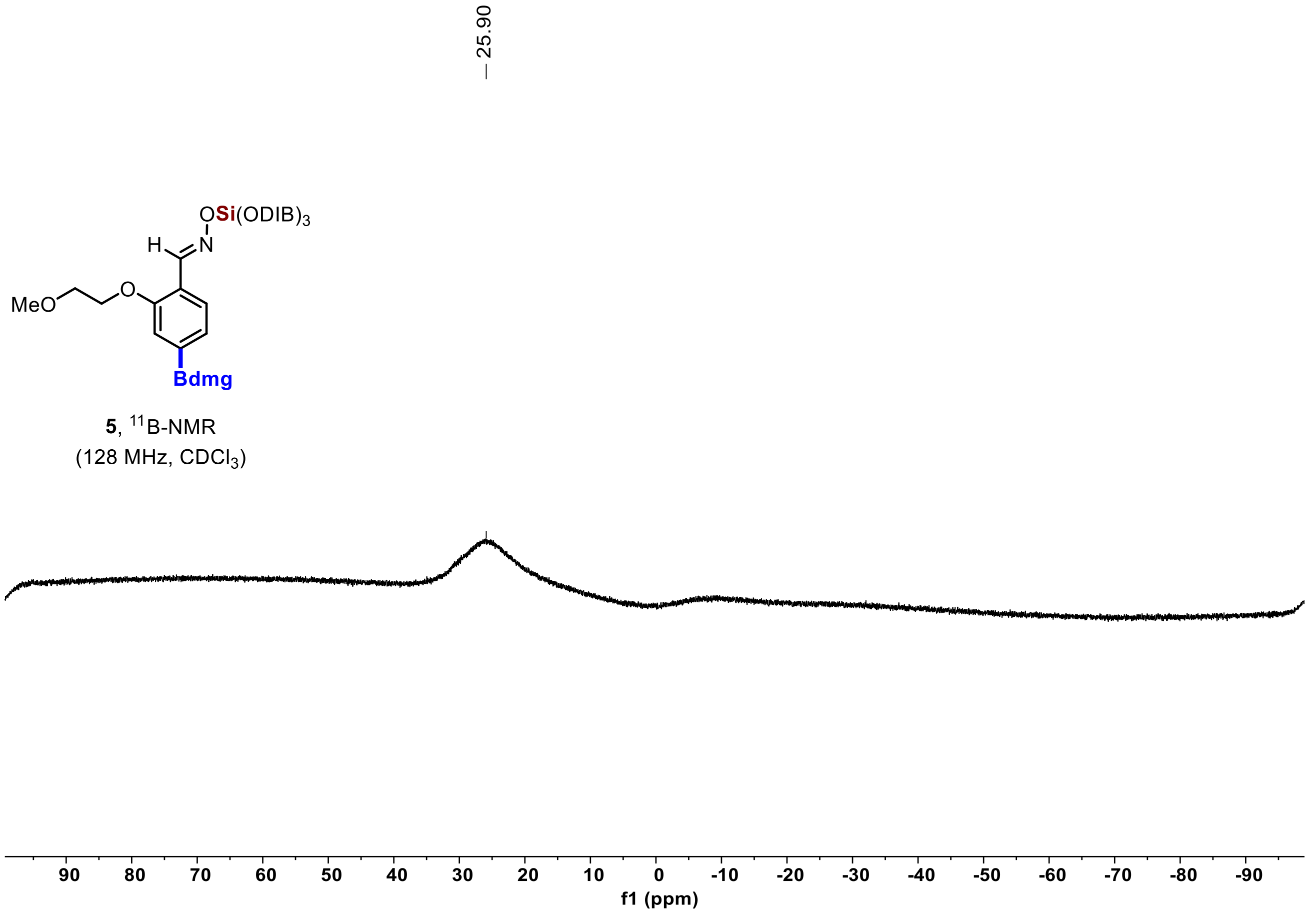
25.90
OSi(ODIB)3
H
N
MeO
O
Bdmg
5, 11B-NMR
(128 MHz, CDCl3)
90
80
70
60
50
40
30
20
10
0
-10
-20
-30
-40
-50
-60
-70
-80
-90
f1 (ppm)

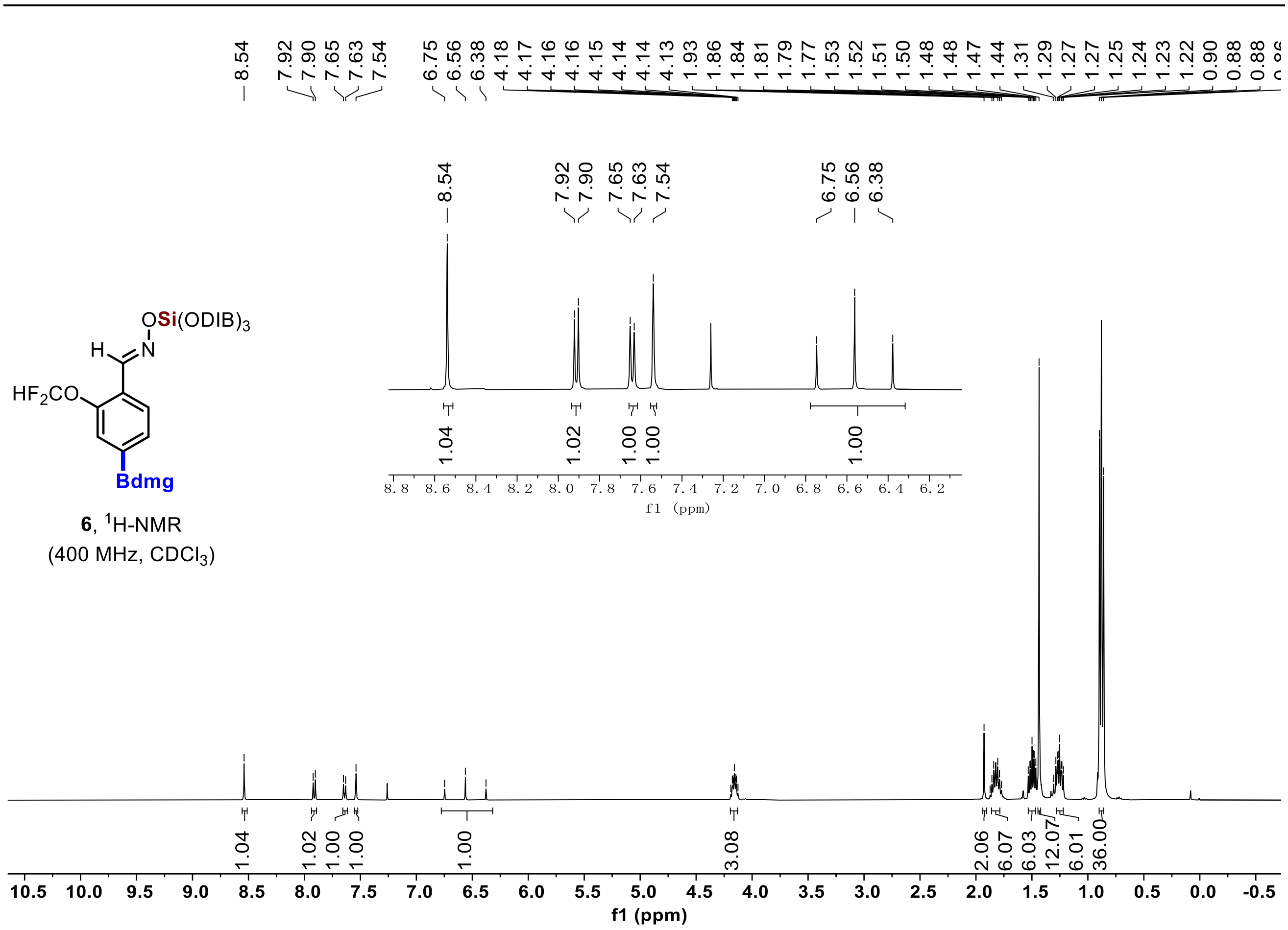

6, ^{1}H-NMR
(400 MHz, $CDCl_3$)

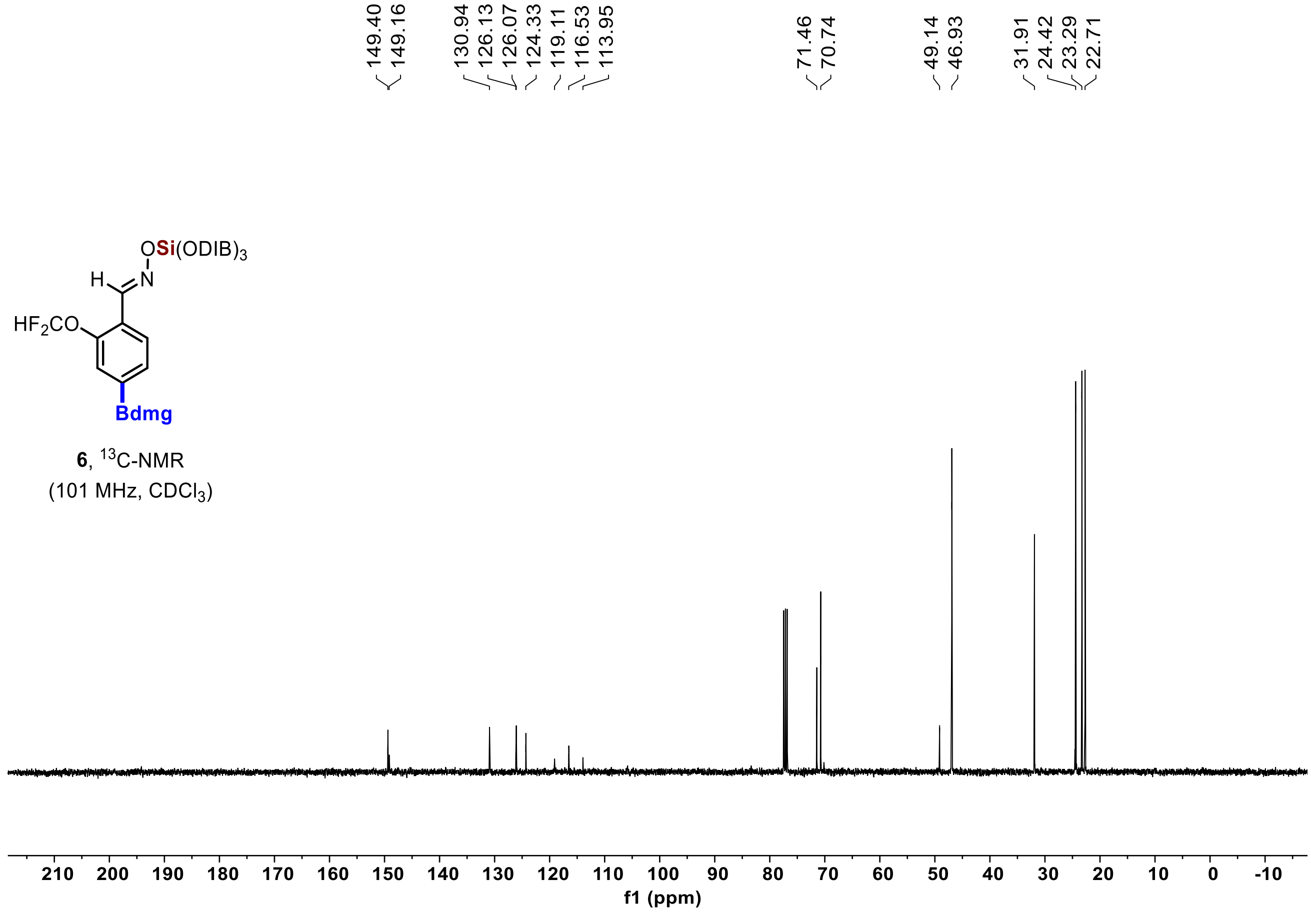

6, ^{13}C-NMR
(101 MHz, $CDCl_3$)

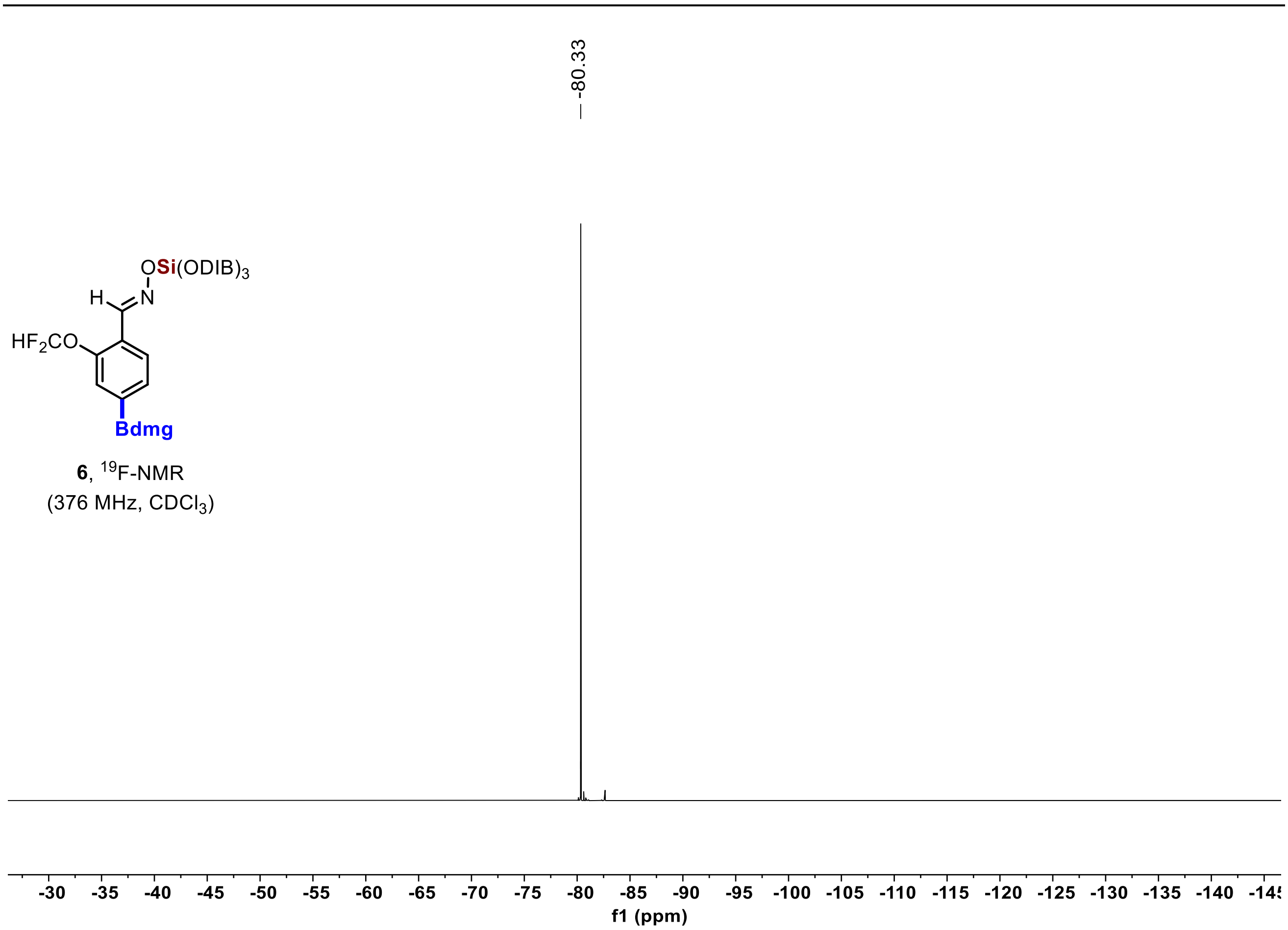
-80.33
OSi(ODIB)3
H
N
HF2CO
Bdmg
6, 19F-NMR
(376 MHz, CDCl3)
-30 -35 -40 -45 -50 -55 -60 -65 -70 -75 -80 -85 -90 -95 -100 -105 -110 -115 -120 -125 -130 -135 -140
f1 (ppm)

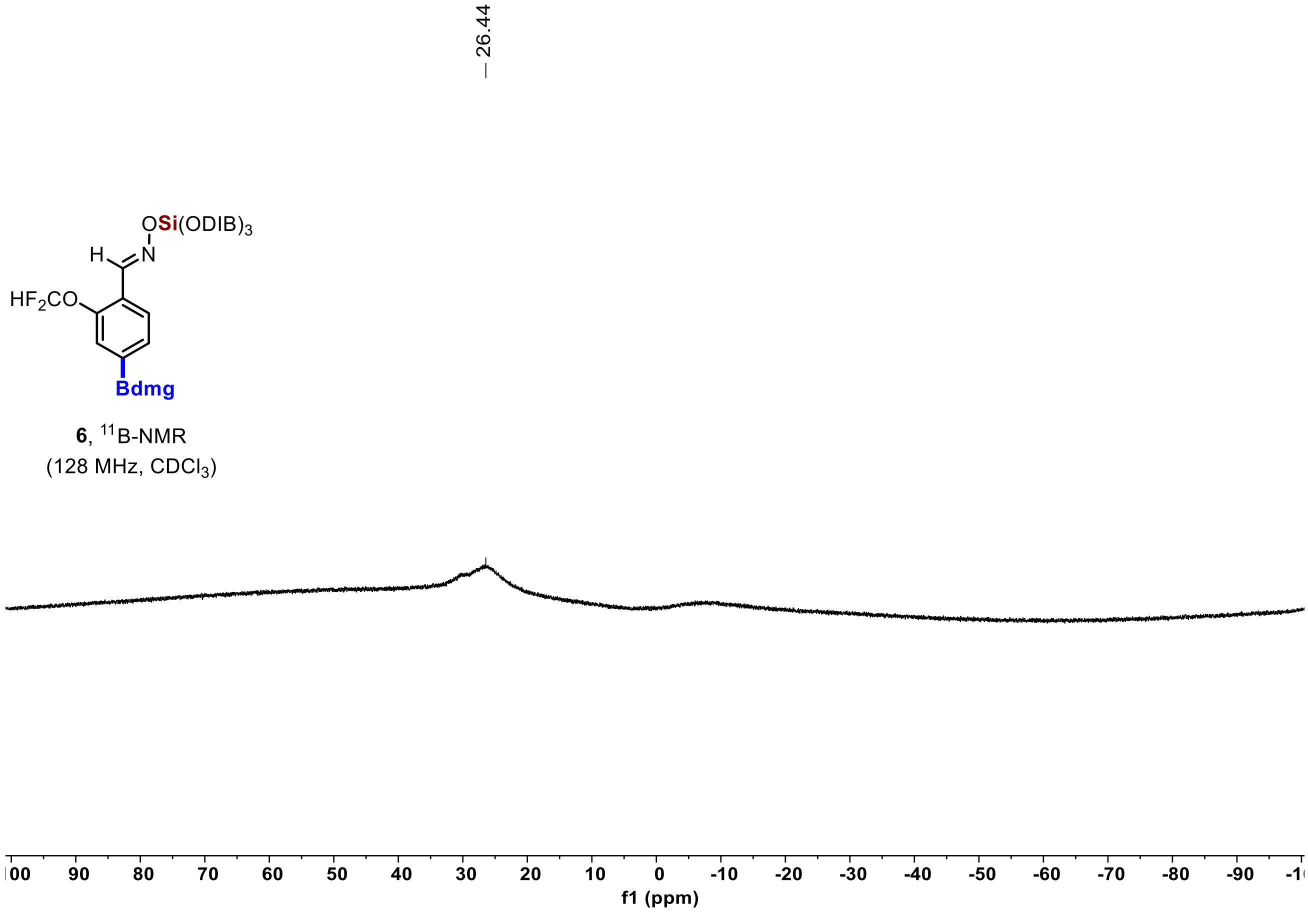
26.44
OSi(ODIB)3
H
N
HF2CO
Bdmg
6, 11B-NMR
(128 MHz, CDCl3)
90 80 70 60 50 40 30 20 10 0 -10 -20 -30 -40 -50 -60 -70 -80 -90
f1 (ppm)

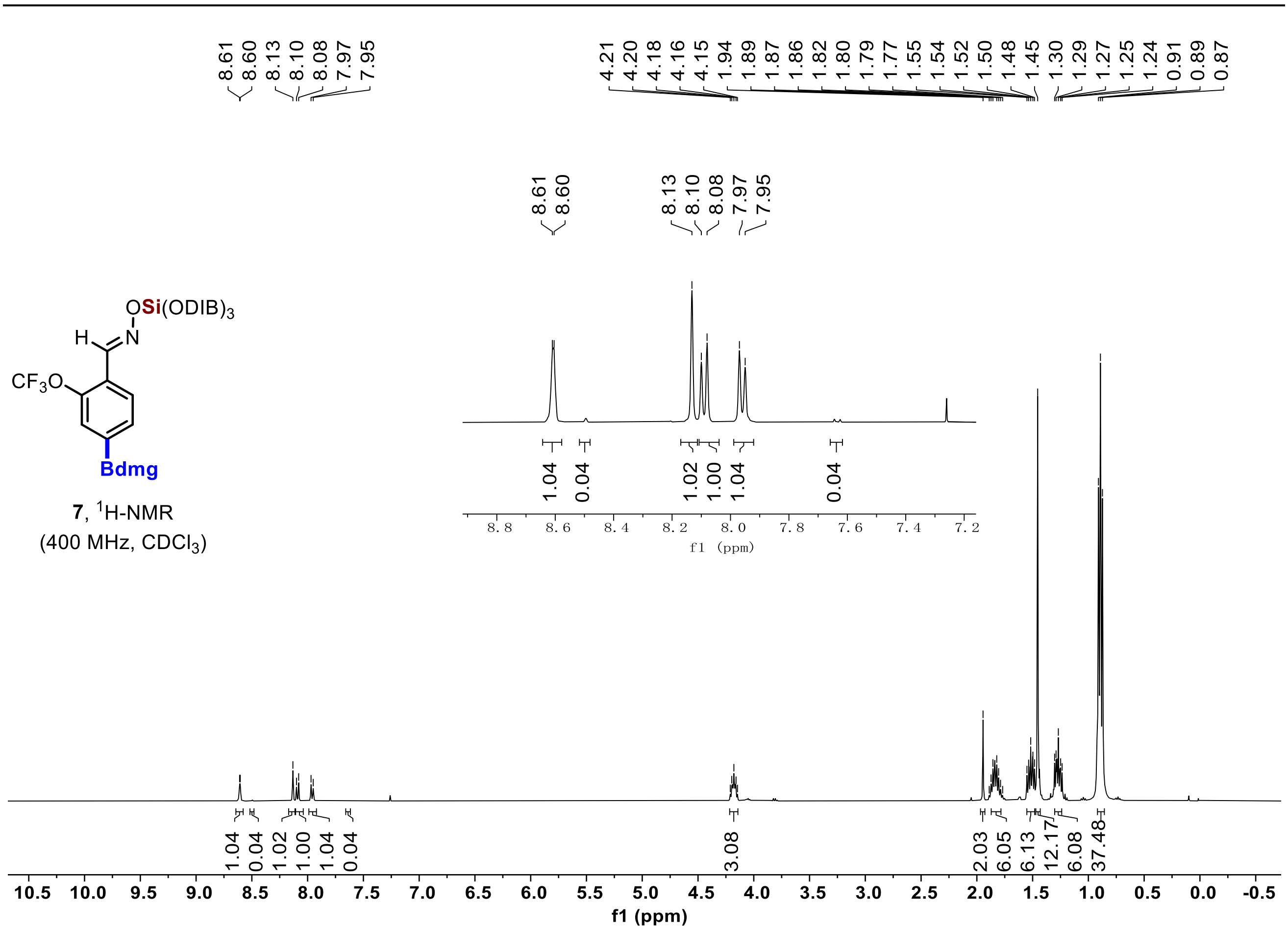
OSi(ODIB)3
H
N
CF3O
Bdmg
7, 1H-NMR
(400 MHz, CDCl3)
f1 (ppm)

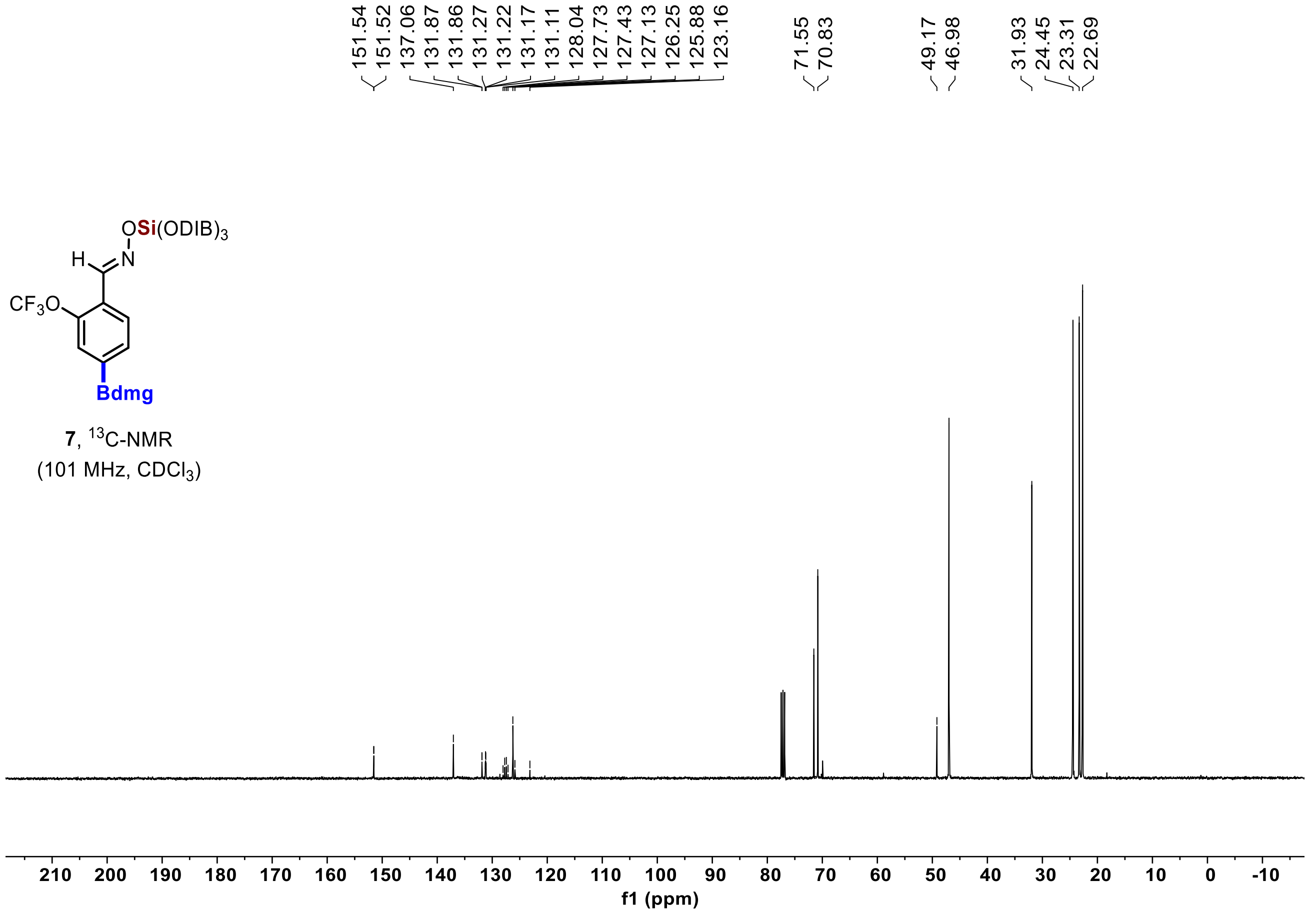
OSi(ODIB)3
H
N
CF3O
Bdmg
7, 13C-NMR
(101 MHz, CDCl3)
f1 (ppm)

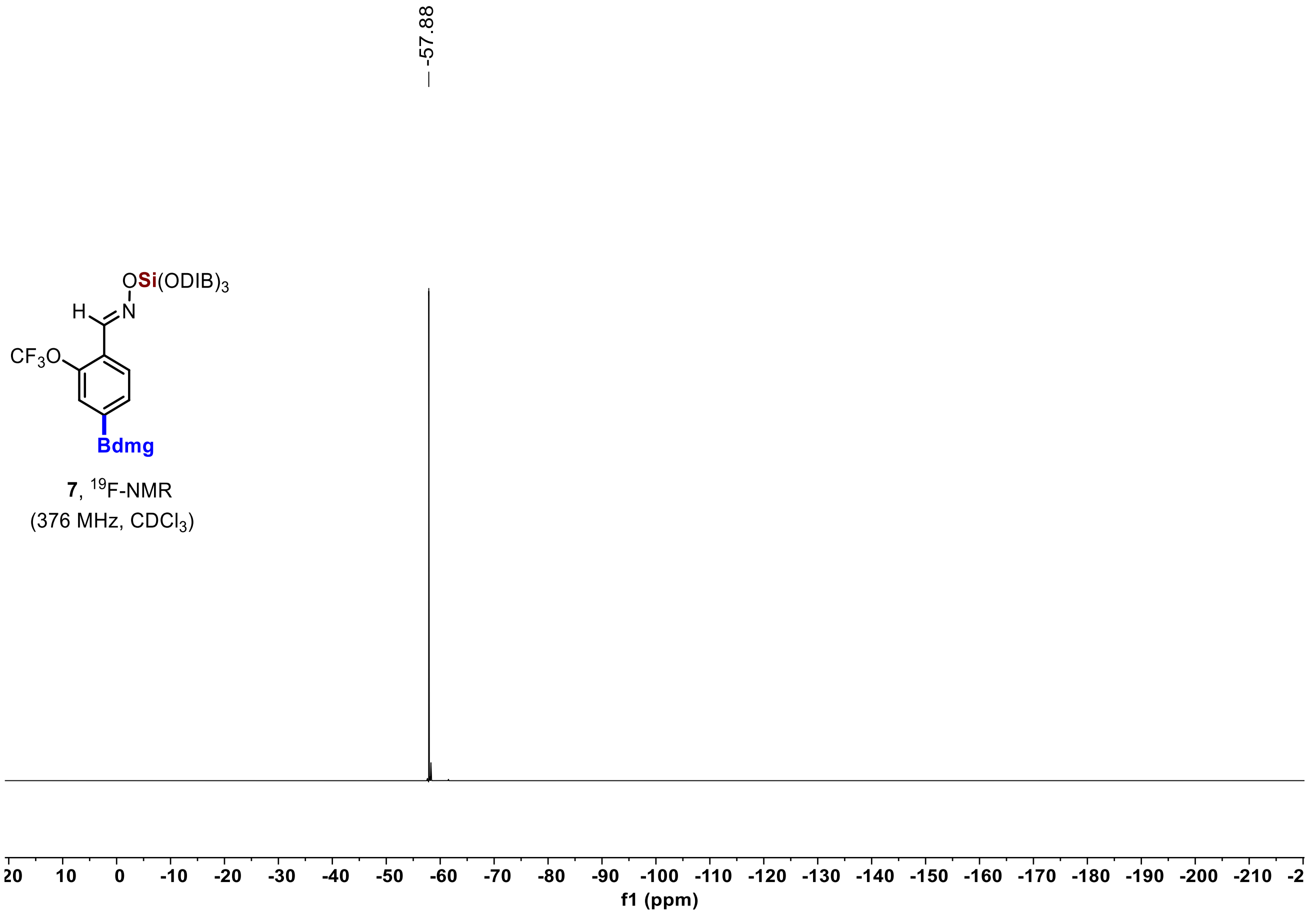

-57.88
OSi(ODIB)3
H
N
CF3O
Bdmg
7, 19F-NMR
(376 MHz, CDCl3)
20 10 0 -10 -20 -30 -40 -50 -60 -70 -80 -90 -100 -110 -120 -130 -140 -150 -160 -170 -180 -190 -200 -210
f1 (ppm)

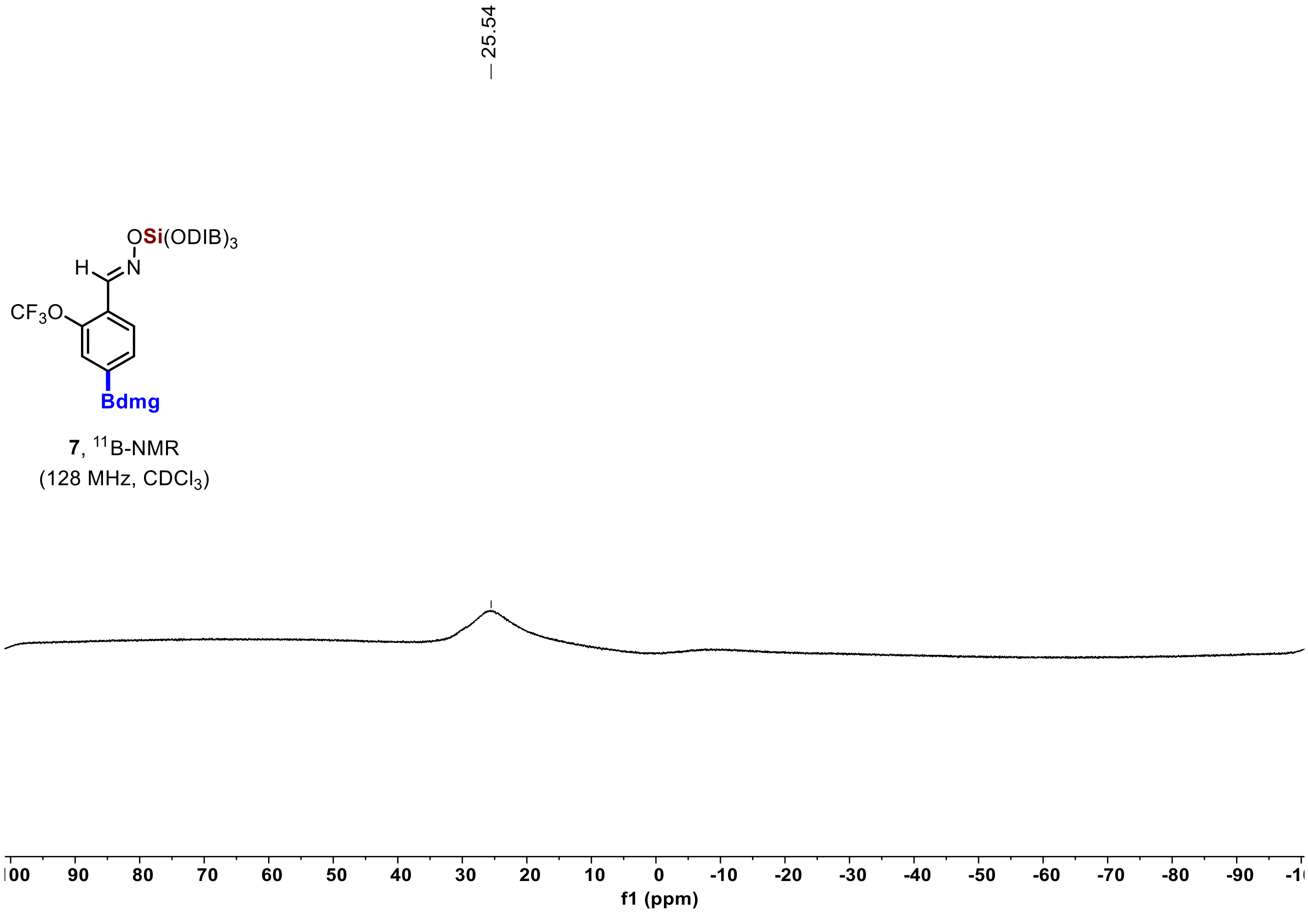

25.54
OSi(ODIB)3
H
N
CF3O
Bdmg
7, 11B-NMR
(128 MHz, CDCl3)
90 80 70 60 50 40 30 20 10 0 -10 -20 -30 -40 -50 -60 -70 -80 -90
f1 (ppm)

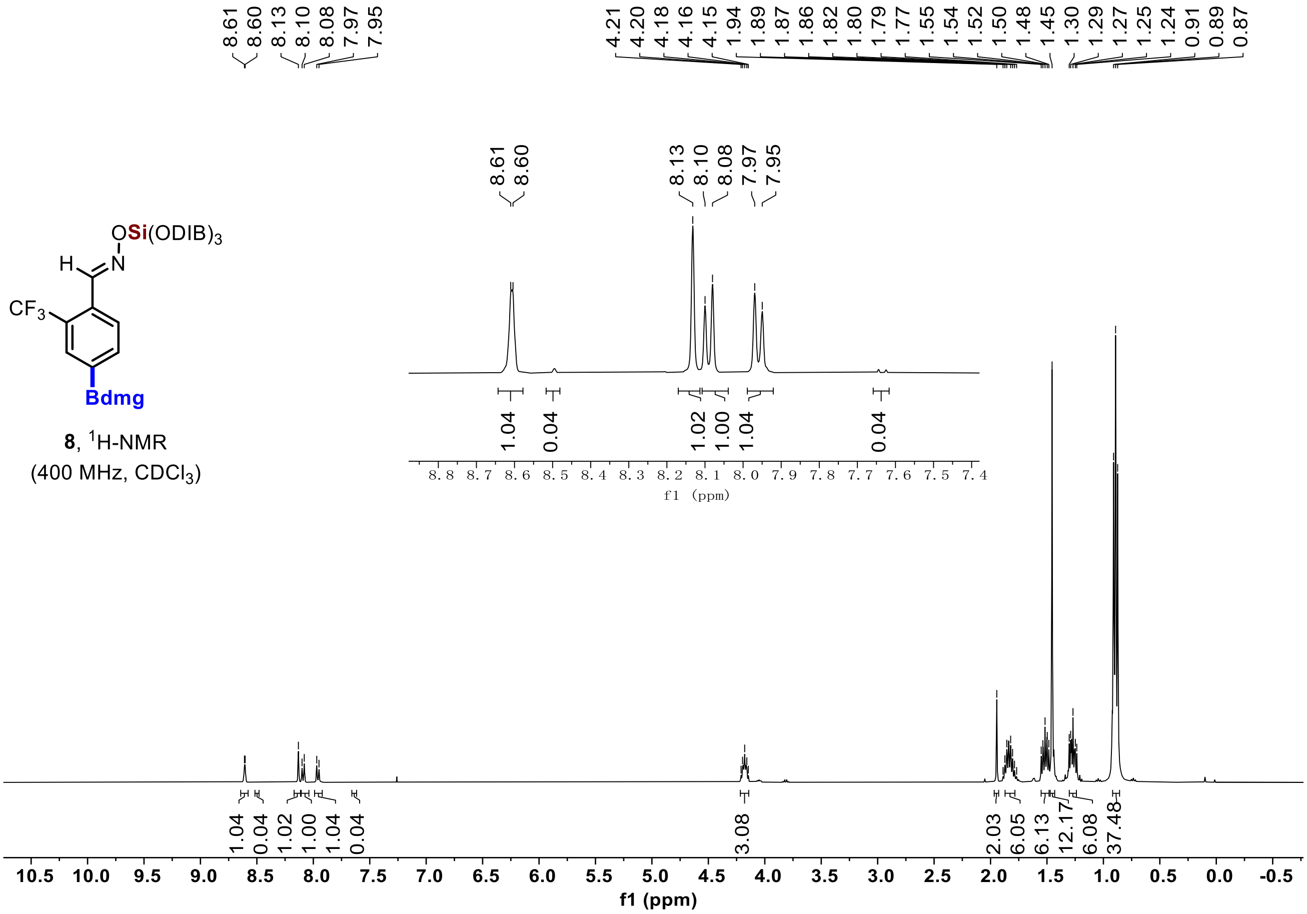
8.61 8.60 8.13 8.10 8.08 7.97 7.95
4.21 4.20 4.18 4.16 4.15 1.94 1.89 1.87 1.86 1.82 1.80 1.79 1.77 1.55 1.54 1.52 1.50 1.48 1.45 1.30 1.29 1.27 1.25 1.24 0.91 0.89 0.87
OSi(ODIB)3
H
N
CF3
Bdmg
8, 1H-NMR
(400 MHz, CDCl3)
1.04 0.04 1.02 1.00 1.04 0.04
f1 (ppm)
1.04 0.04 1.02 1.00 1.04 0.04 3.08 2.03 6.05 6.13 12.17 6.08 37.48
f1 (ppm)

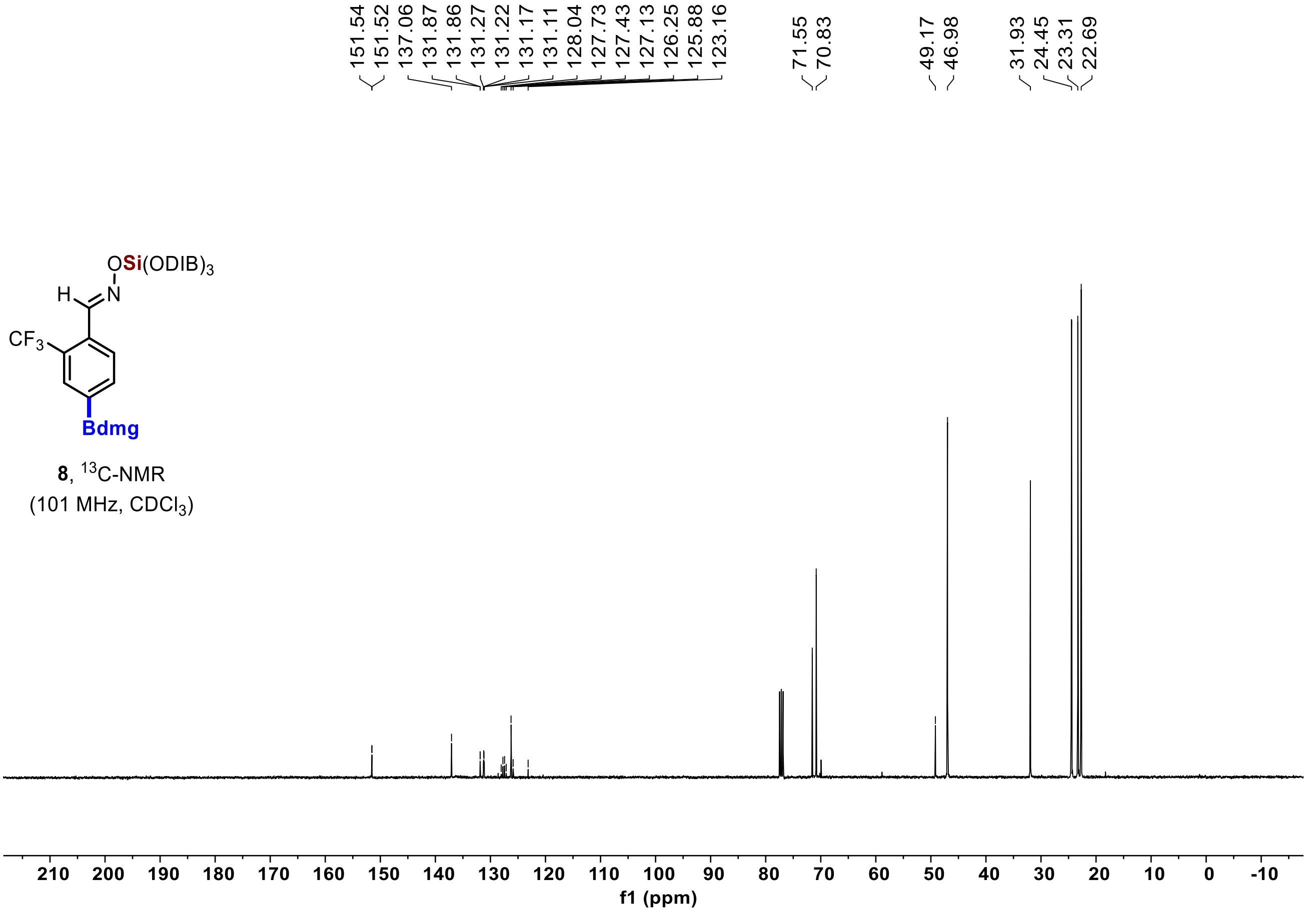
151.54 151.52 137.06 131.87 131.86 131.27 131.22 131.17 131.11 128.04 127.73 127.43 127.13 126.25 125.88 123.16
71.55 70.83
49.17 46.98
31.93 24.45 23.31 22.69
OSi(ODIB)3
H
N
CF3
Bdmg
8, 13C-NMR
(101 MHz, CDCl3)
f1 (ppm)

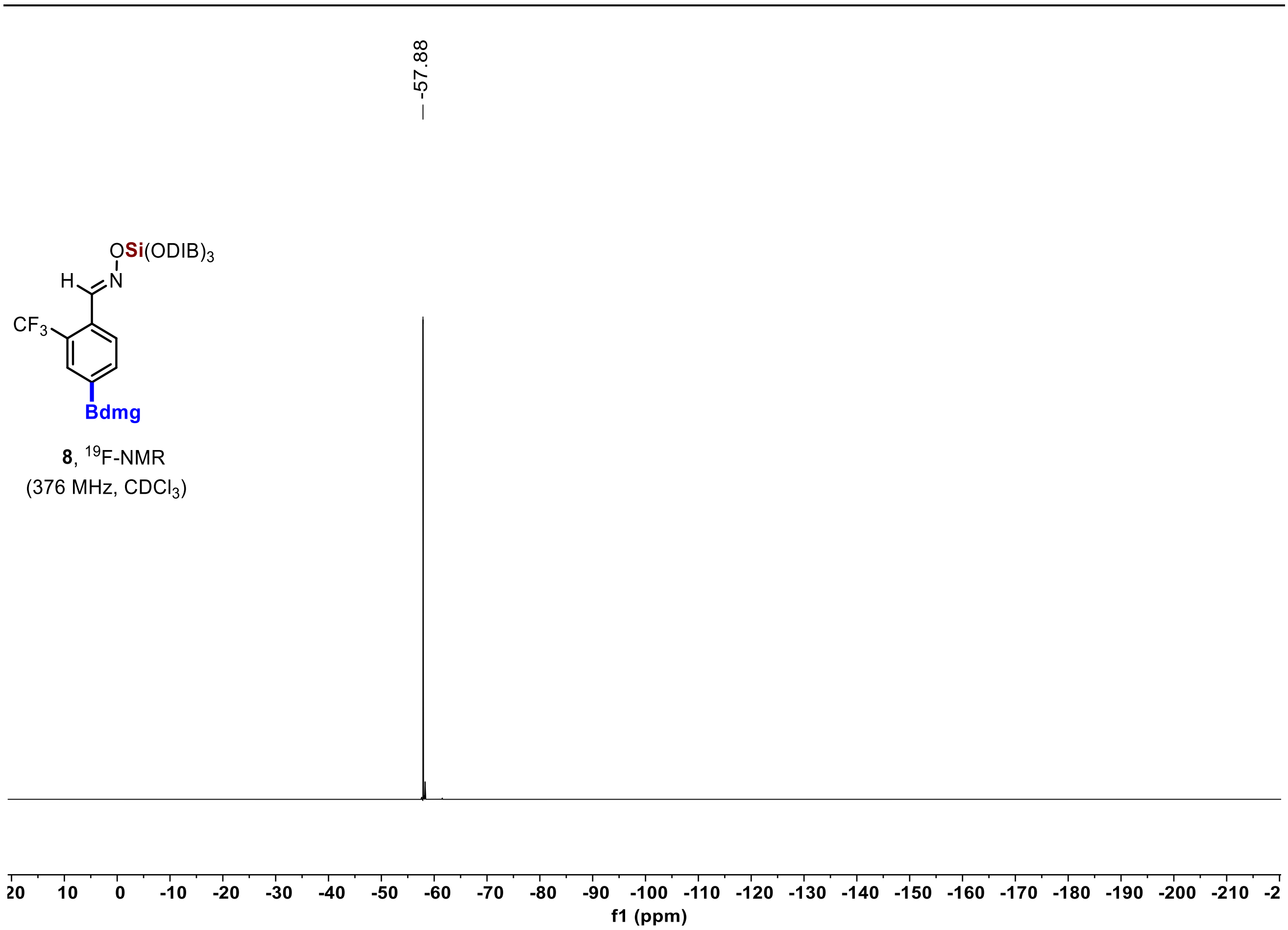

-57.88
OSi(ODIB)3
H
N
CF3
Bdmg
8, 19F-NMR
(376 MHz, CDCl3)
20 10 0 -10 -20 -30 -40 -50 -60 -70 -80 -90 -100 -110 -120 -130 -140 -150 -160 -170 -180 -190 -200 -210
f1 (ppm)

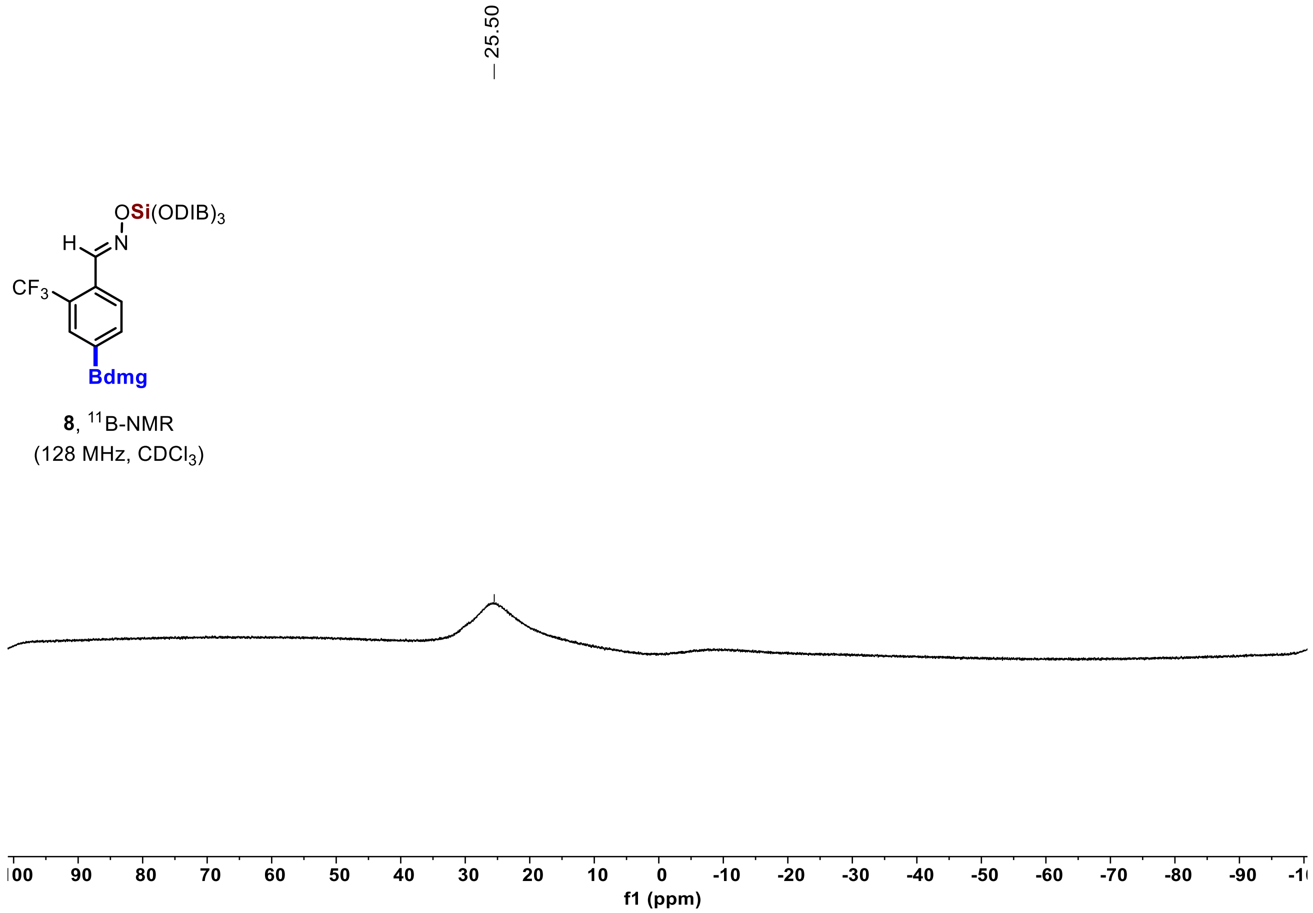

25.50
OSi(ODIB)3
H
N
CF3
Bdmg
8, 11B-NMR
(128 MHz, CDCl3)
90 80 70 60 50 40 30 20 10 0 -10 -20 -30 -40 -50 -60 -70 -80 -90
f1 (ppm)

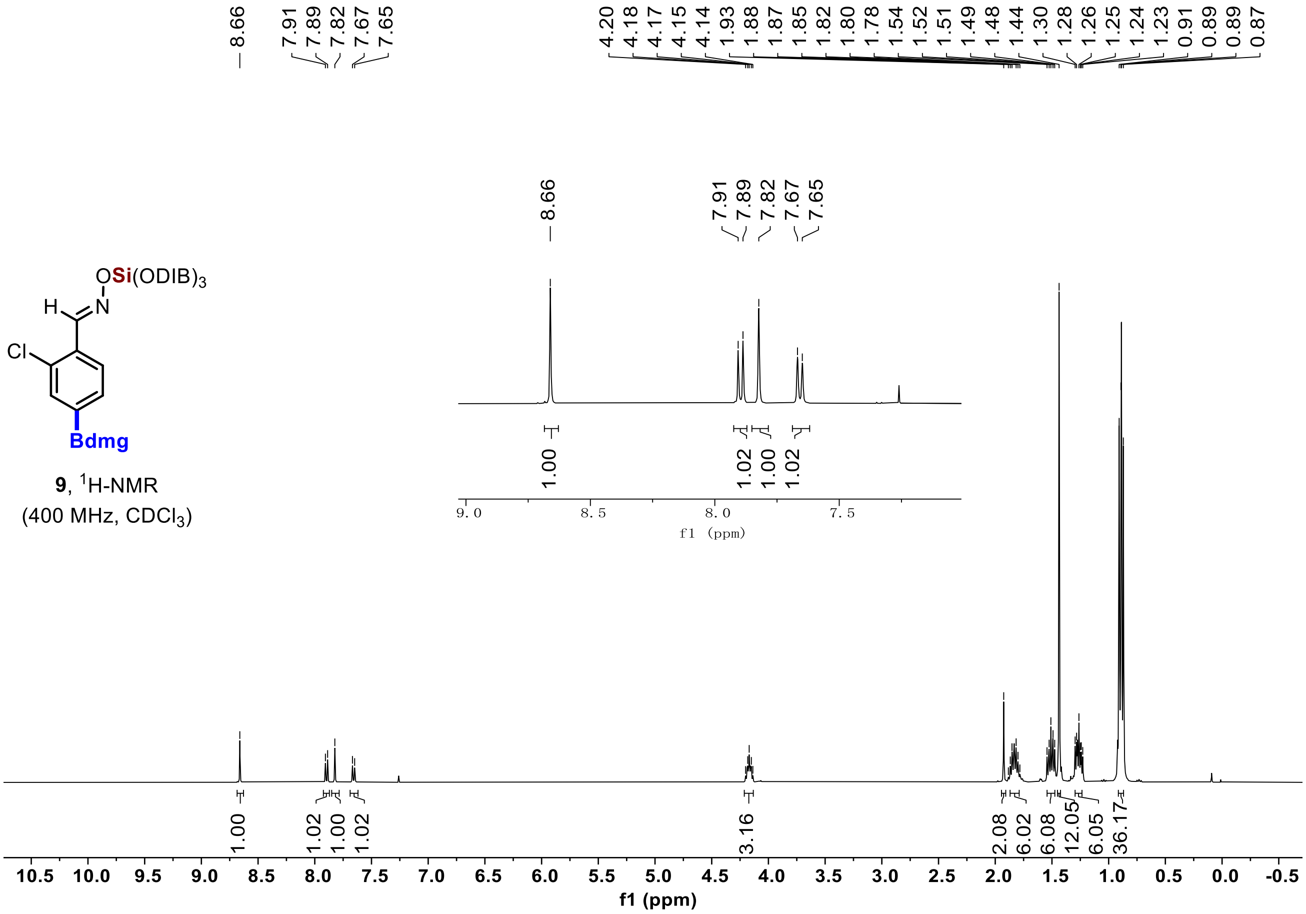

8.66
7.91 7.89 7.82 7.67 7.65
4.20 4.18 4.17 4.15 4.14 1.93 1.88 1.87 1.85 1.82 1.80 1.78 1.54 1.52 1.51 1.49 1.48 1.44 1.30 1.28 1.26 1.25 1.24 1.23 0.91 0.89 0.89 0.87
OSi(ODIB)3
H
N
Cl
Bdmg
9, 1H-NMR
(400 MHz, CDCl3)
1.00
1.02
1.00
1.02
9.0 8.5 8.0 7.5
f1 (ppm)
1.00 1.02 1.00 1.02 3.16 2.08 6.02 6.08 12.05 6.05 36.17
10.5 10.0 9.5 9.0 8.5 8.0 7.5 7.0 6.5 6.0 5.5 5.0 4.5 4.0 3.5 3.0 2.5 2.0 1.5 1.0 0.5 0.0 -0.5
f1 (ppm)

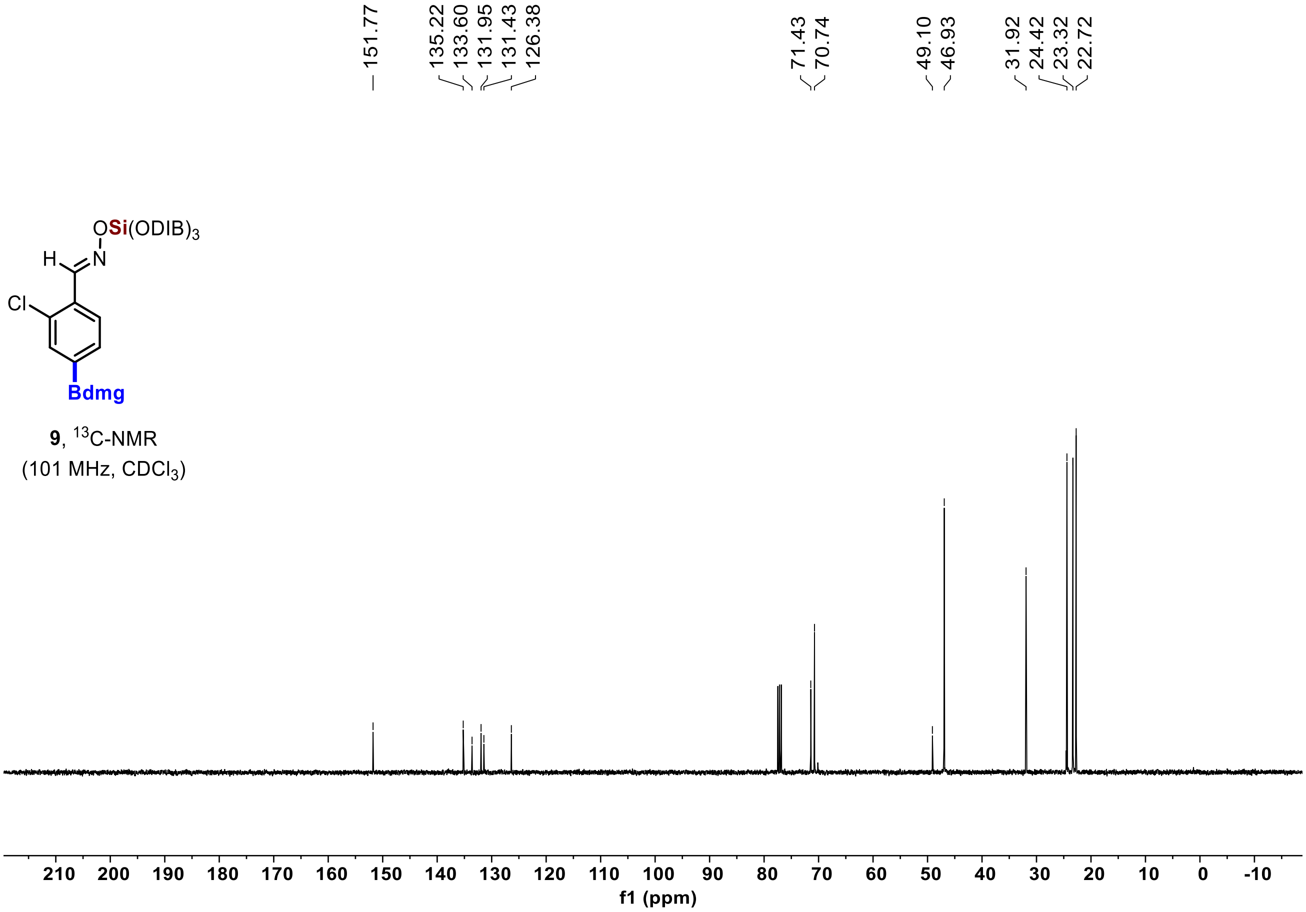

151.77
135.22 133.60 131.95 131.43 126.38
71.43 70.74
49.10 46.93
31.92 24.42 23.32 22.72
OSi(ODIB)3
H
N
Cl
Bdmg
9, 13C-NMR
(101 MHz, CDCl3)
210 200 190 180 170 160 150 140 130 120 110 100 90 80 70 60 50 40 30 20 10 0 -10
f1 (ppm)

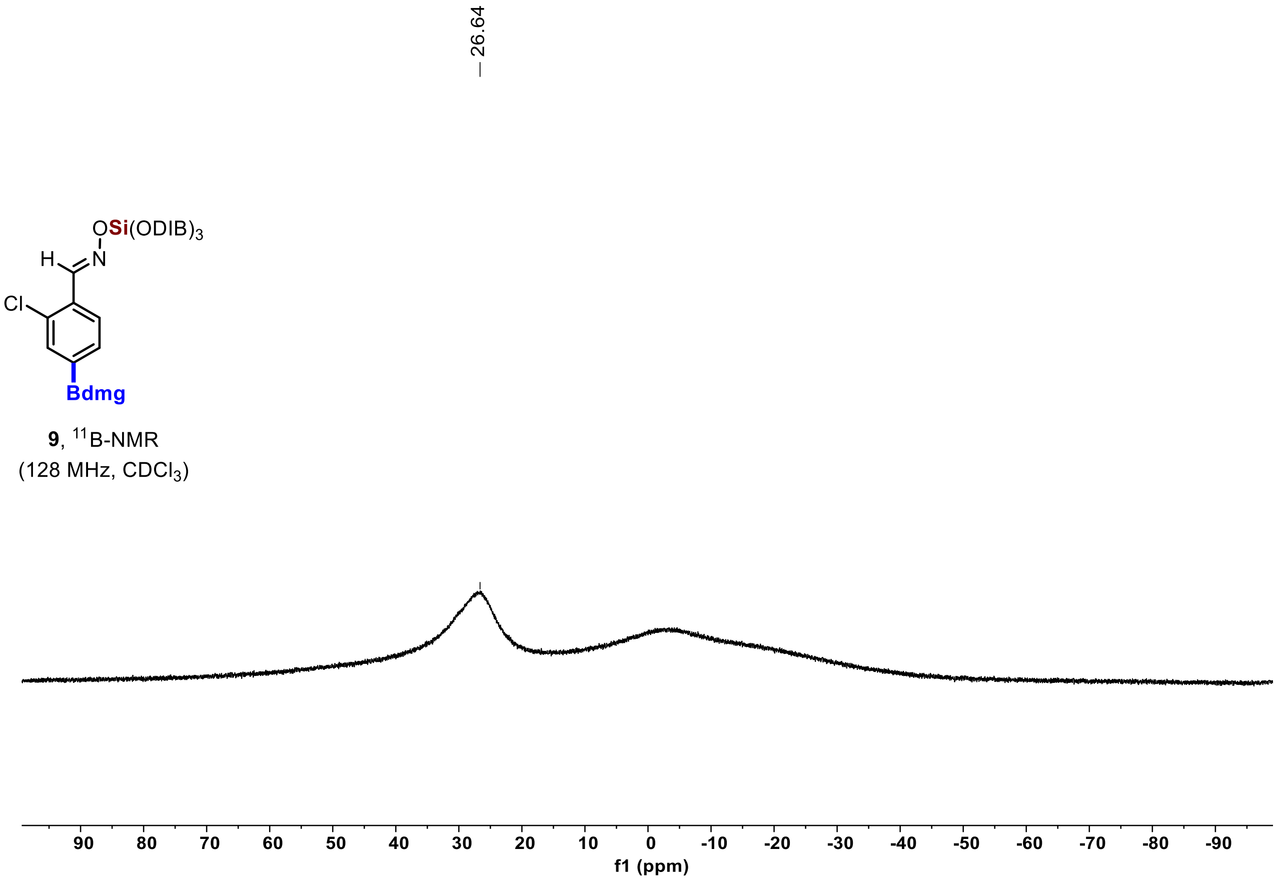

NOESY spectrum of 9 (400 MHz, $CDCl_3$)

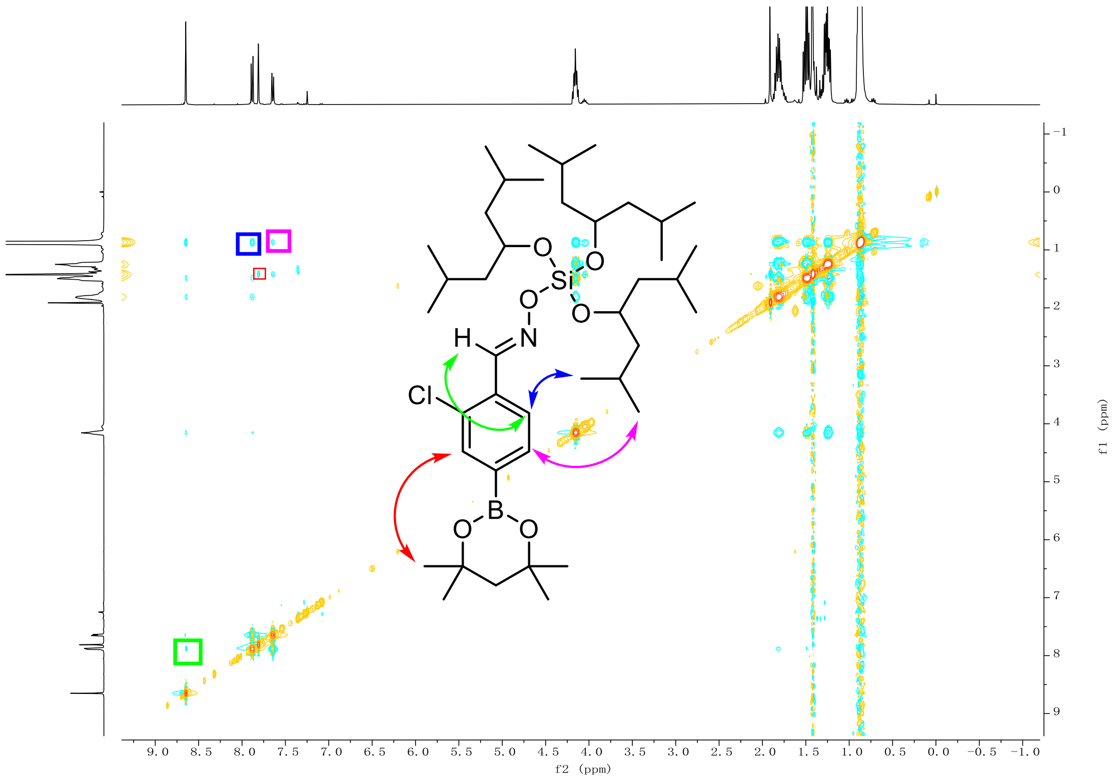

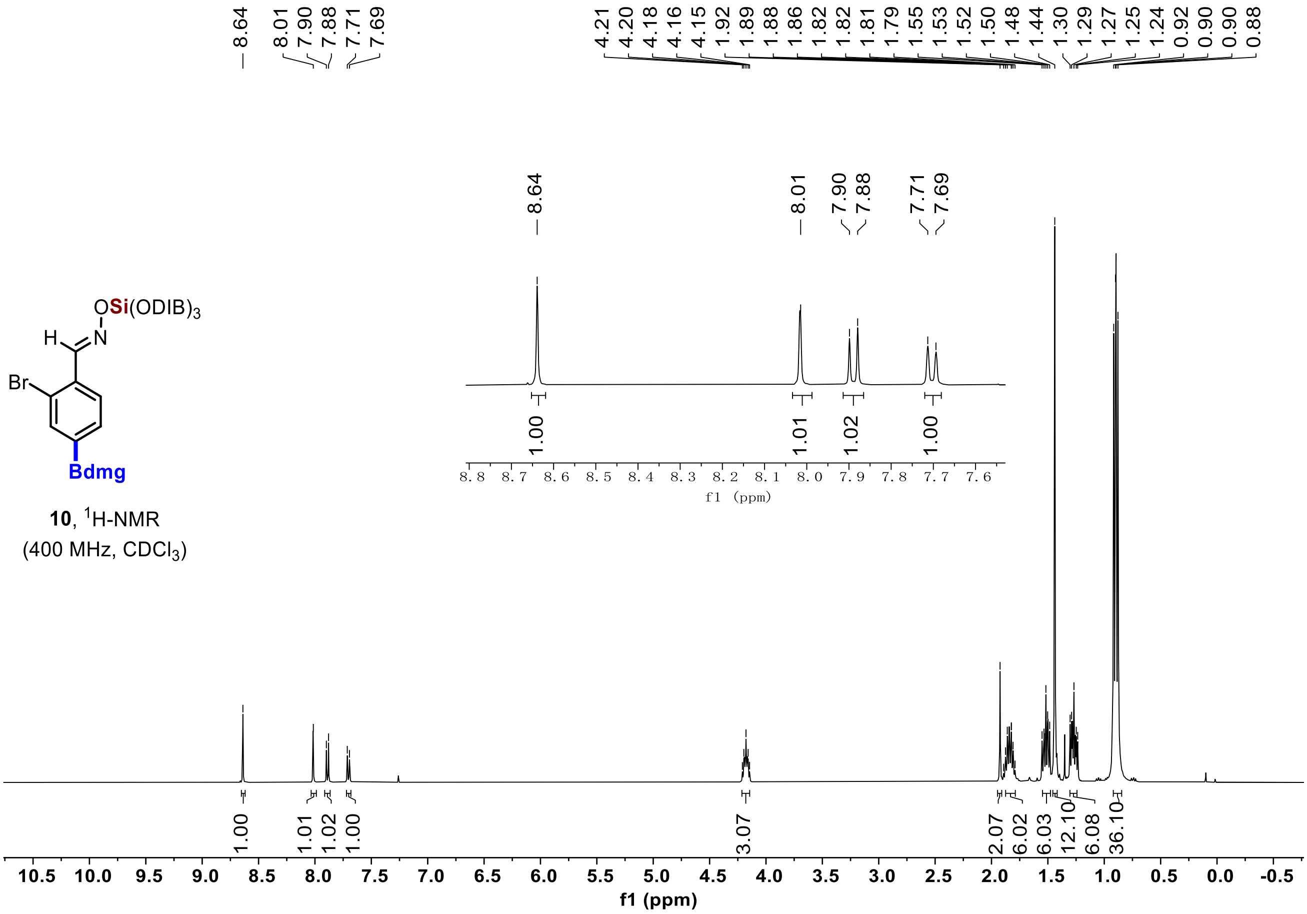

10, ^{1}H-NMR
(400 MHz, $CDCl_3$)

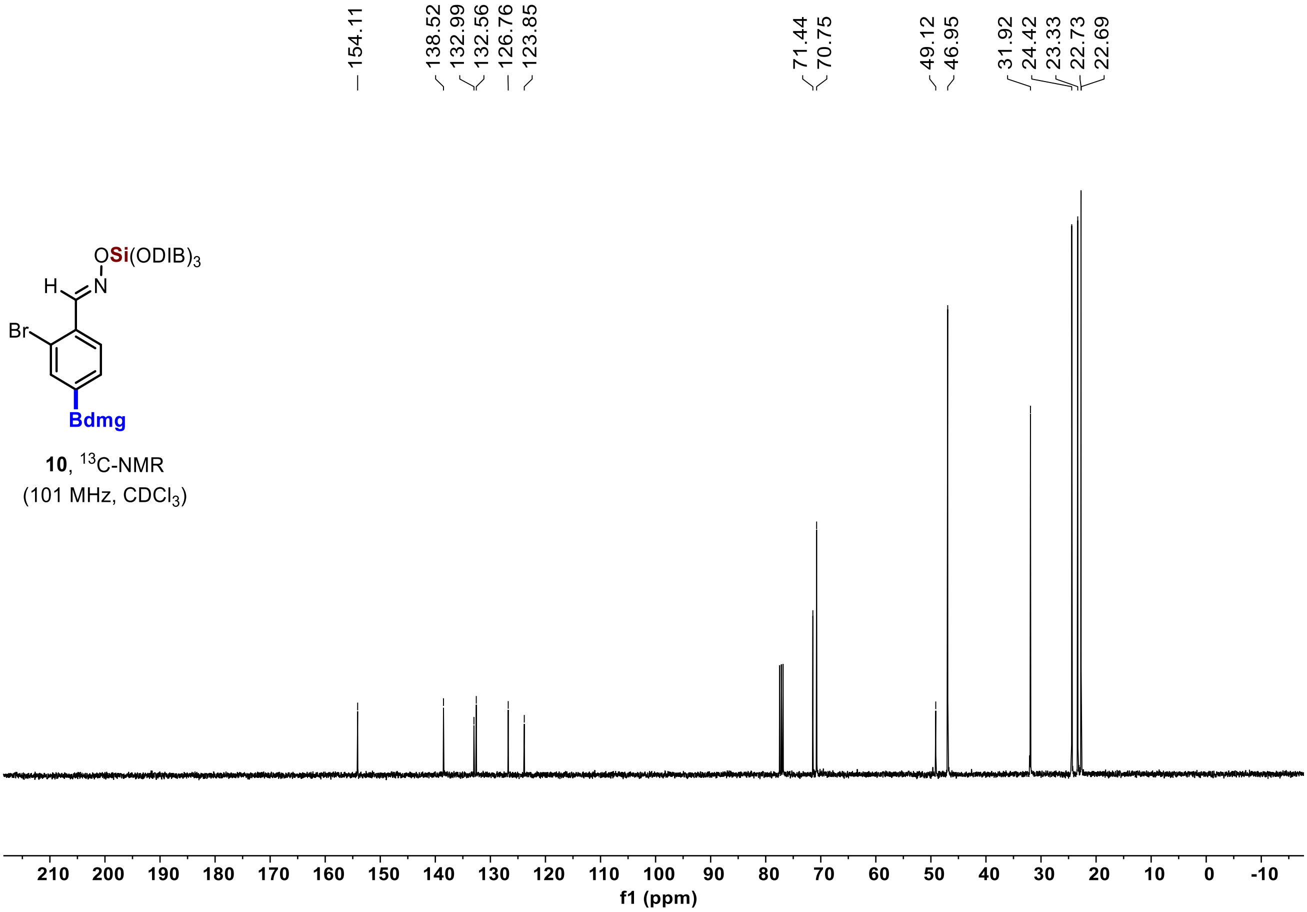

10, ^{13}C-NMR
(101 MHz, $CDCl_3$)

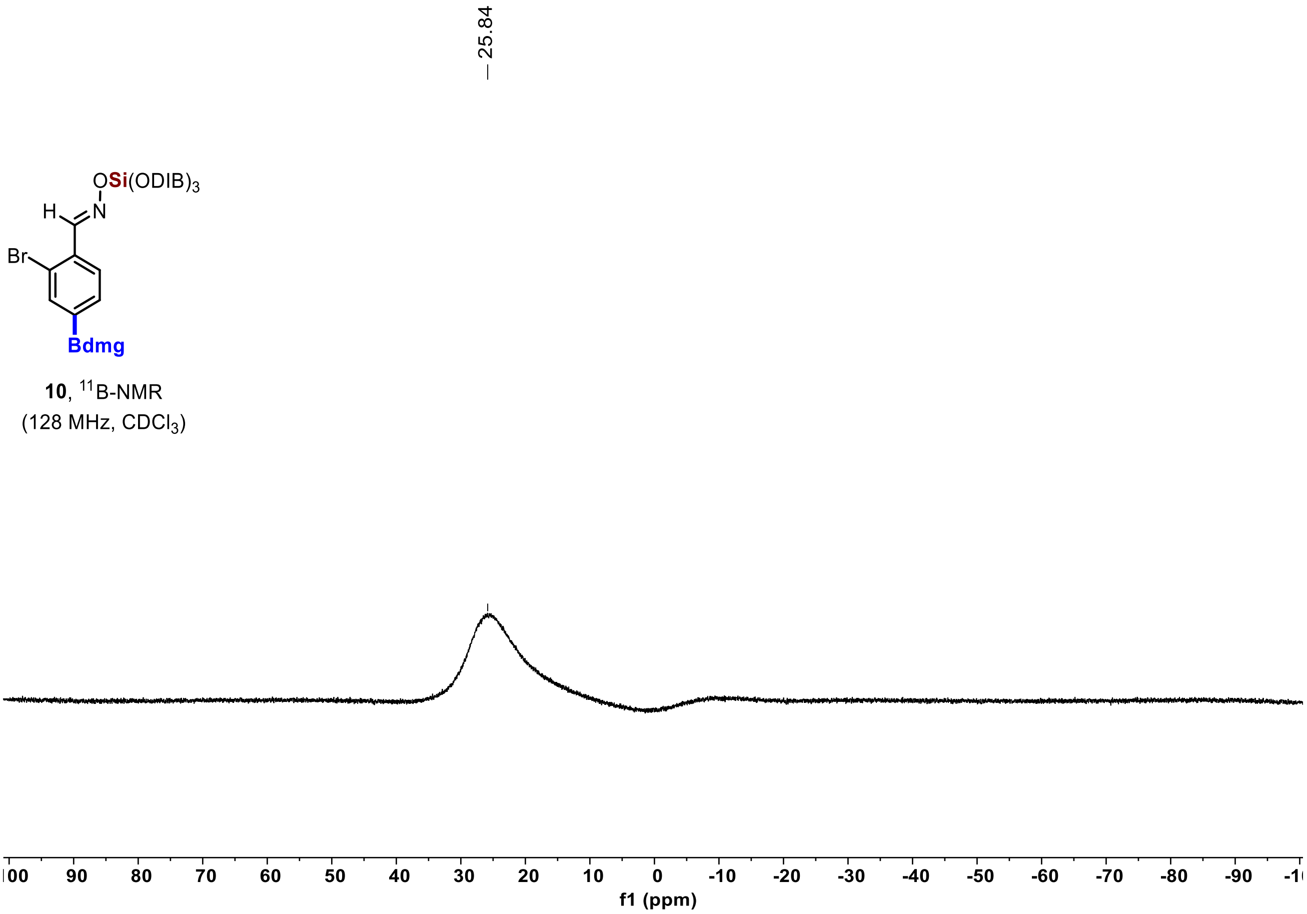

10, ^{11}B-NMR
(128 MHz, $CDCl_3$)

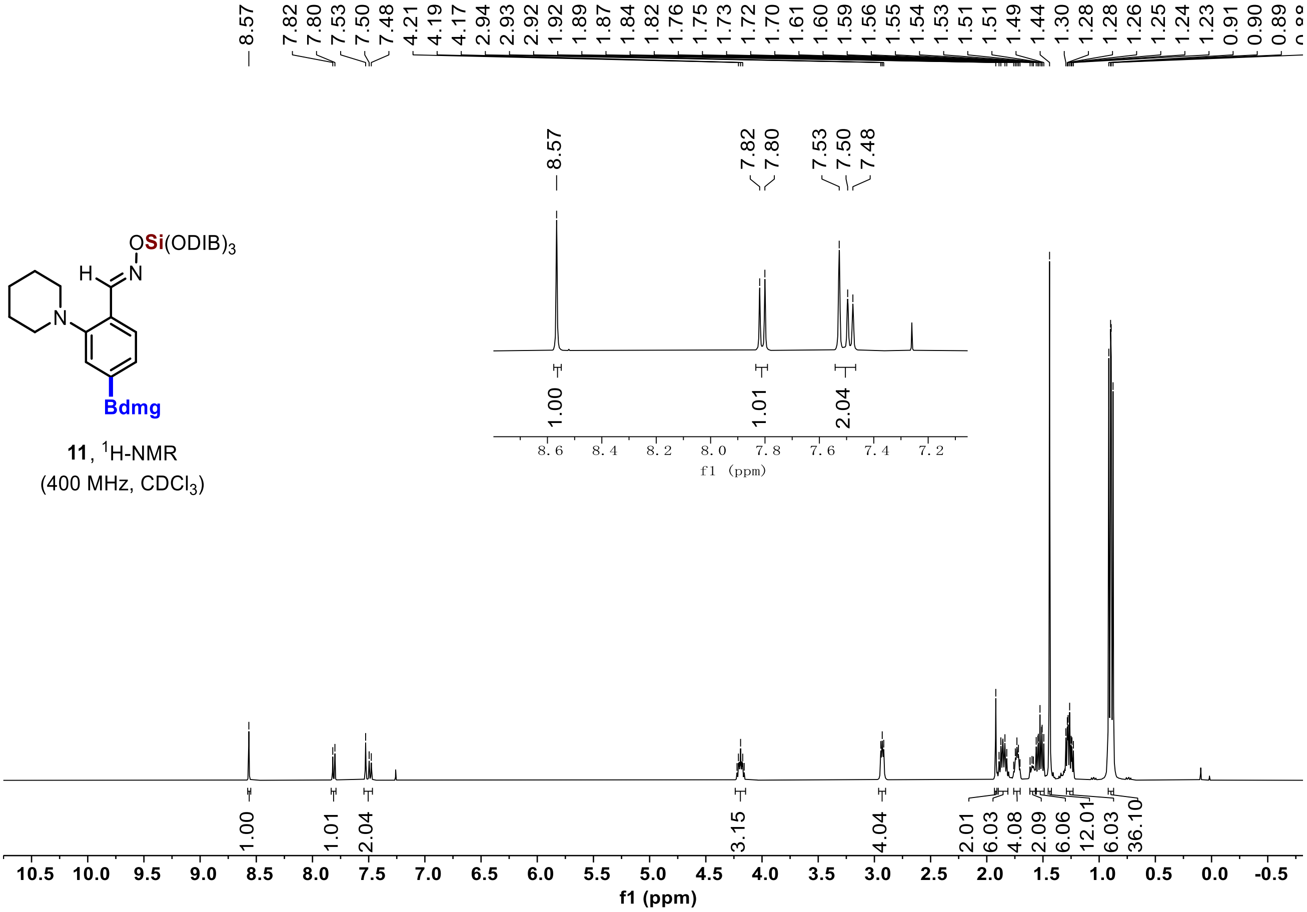

11, ^{1}H-NMR
(400 MHz, $CDCl_3$)

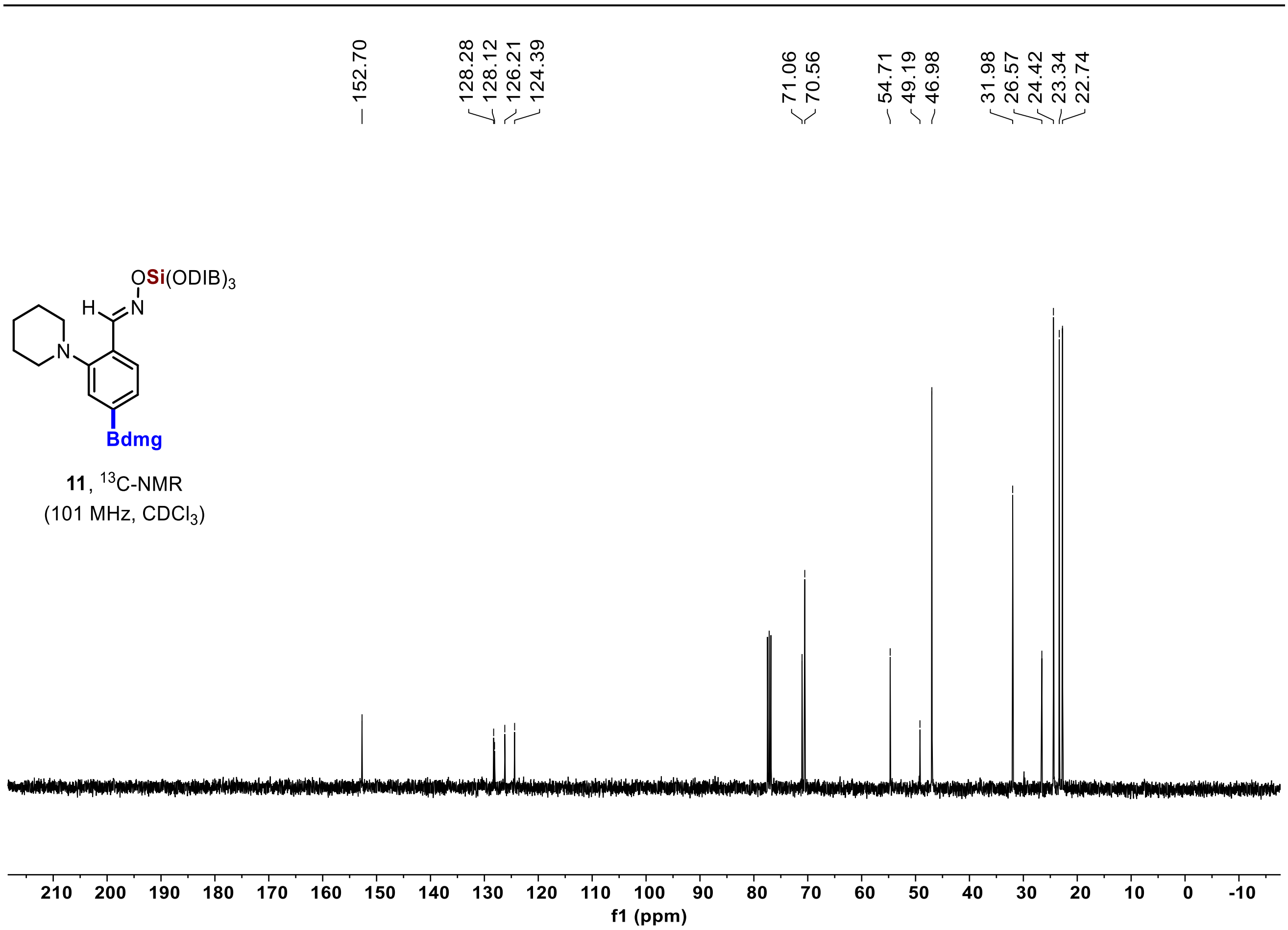

11, ^{13}C-NMR
(101 MHz, $CDCl_3$)

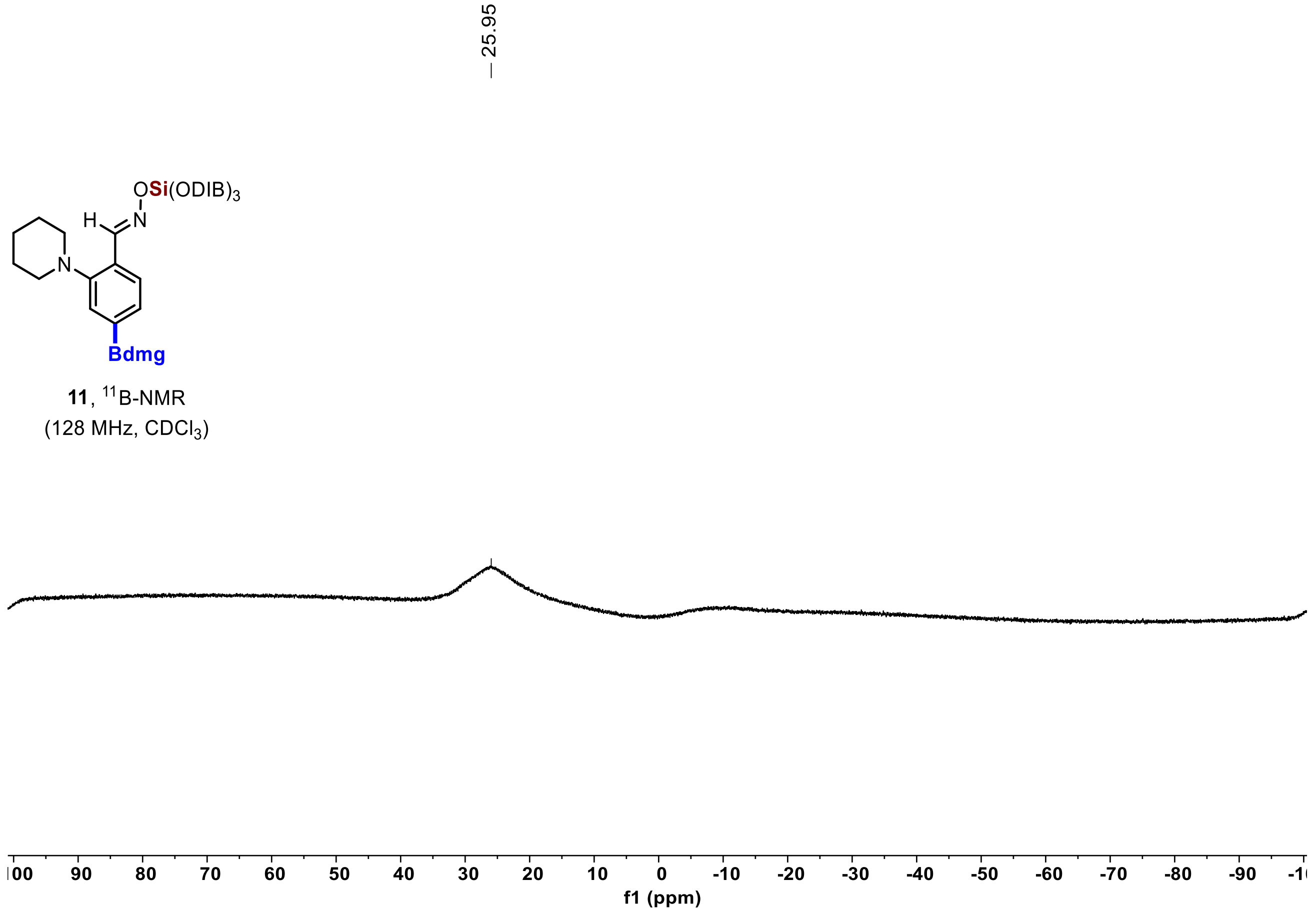

11, ^{11}B-NMR
(128 MHz, $CDCl_3$)

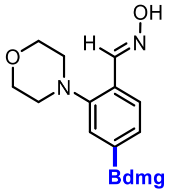

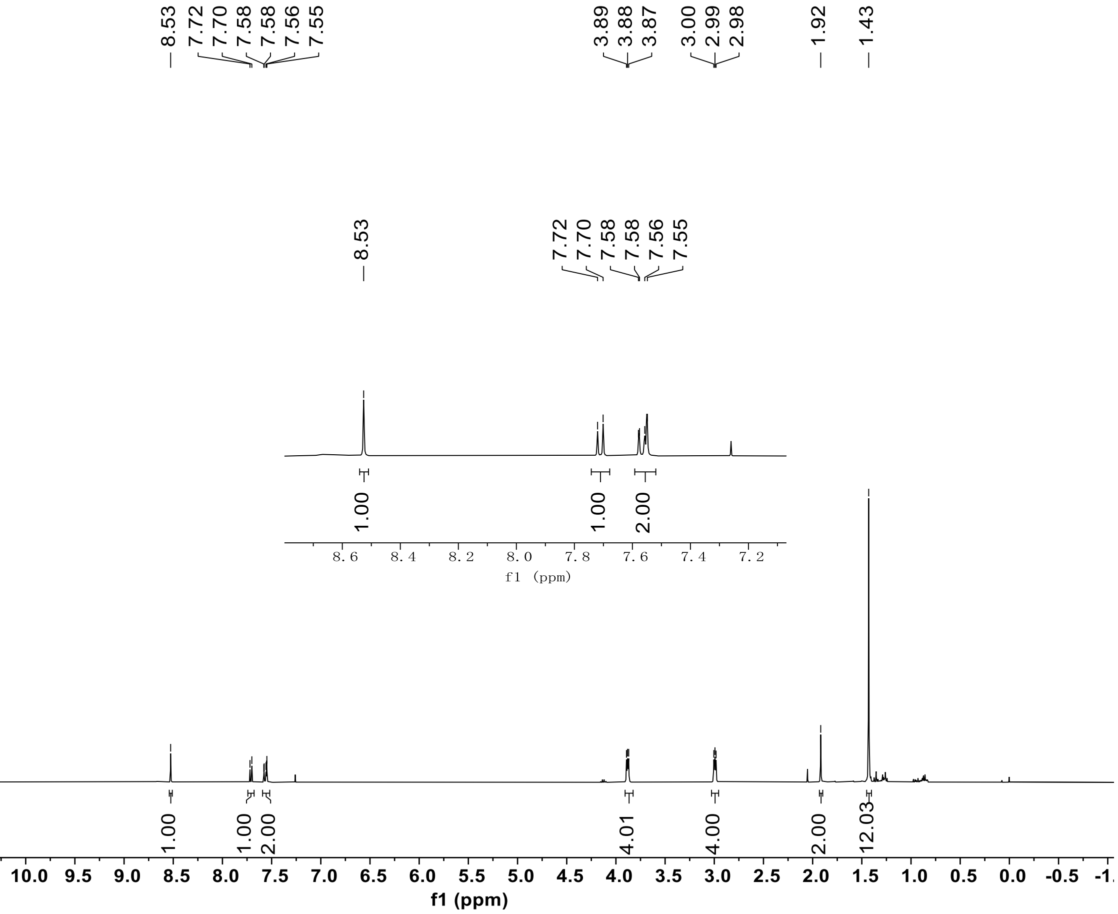

12a, ^{1}H-NMR
(400 MHz, $CDCl_3$)

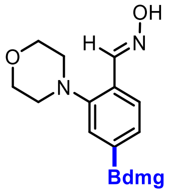

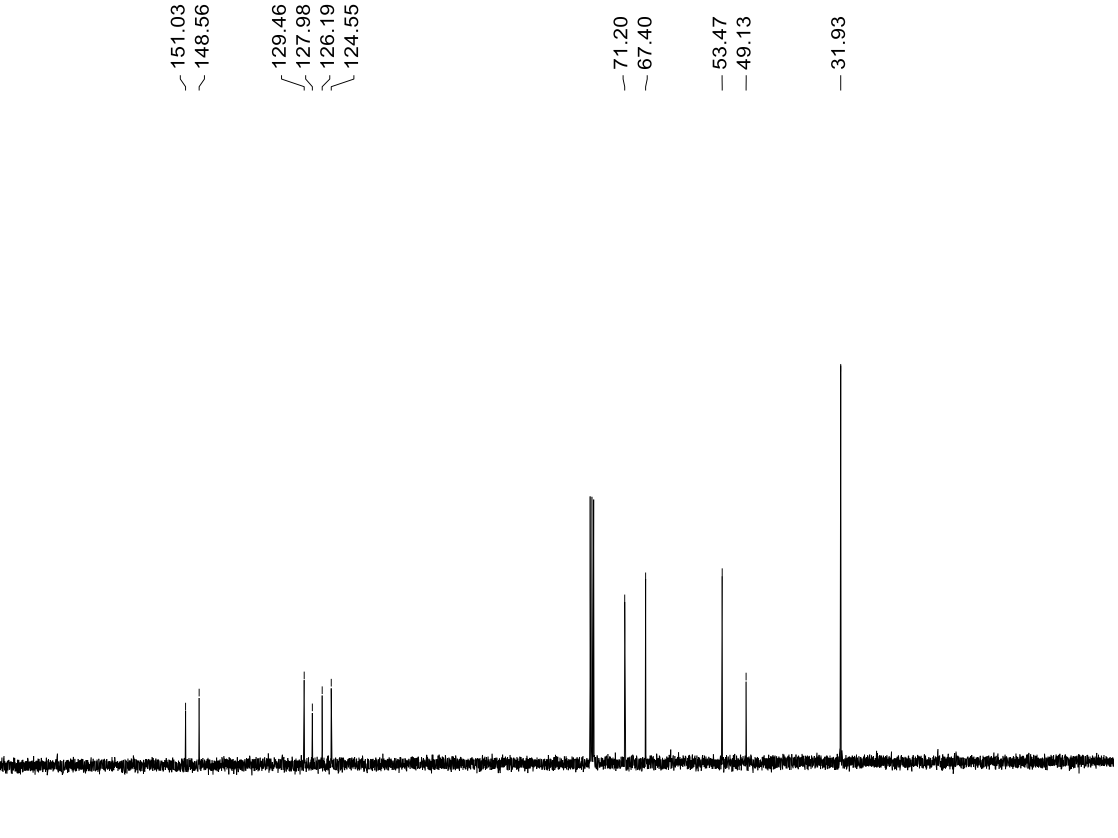

12a, ^{13}C-NMR
(101 MHz, $CDCl_3$)

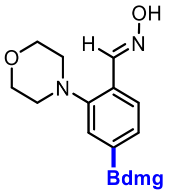

12a, ^{11}B-NMR
(128 MHz, $CDCl_3$)

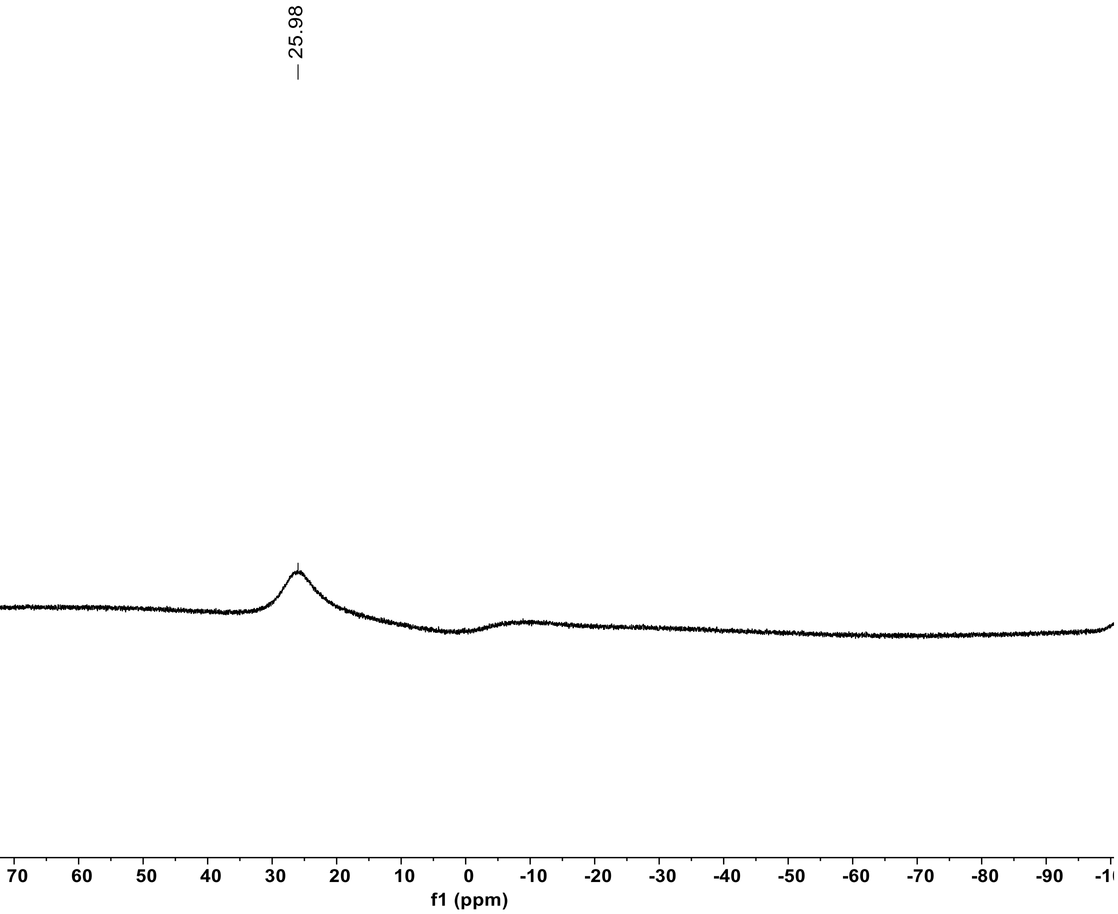

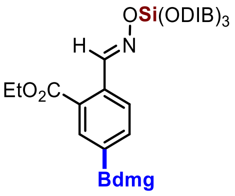

13, ^{1}H-NMR
(400 MHz, $CDCl_3$)

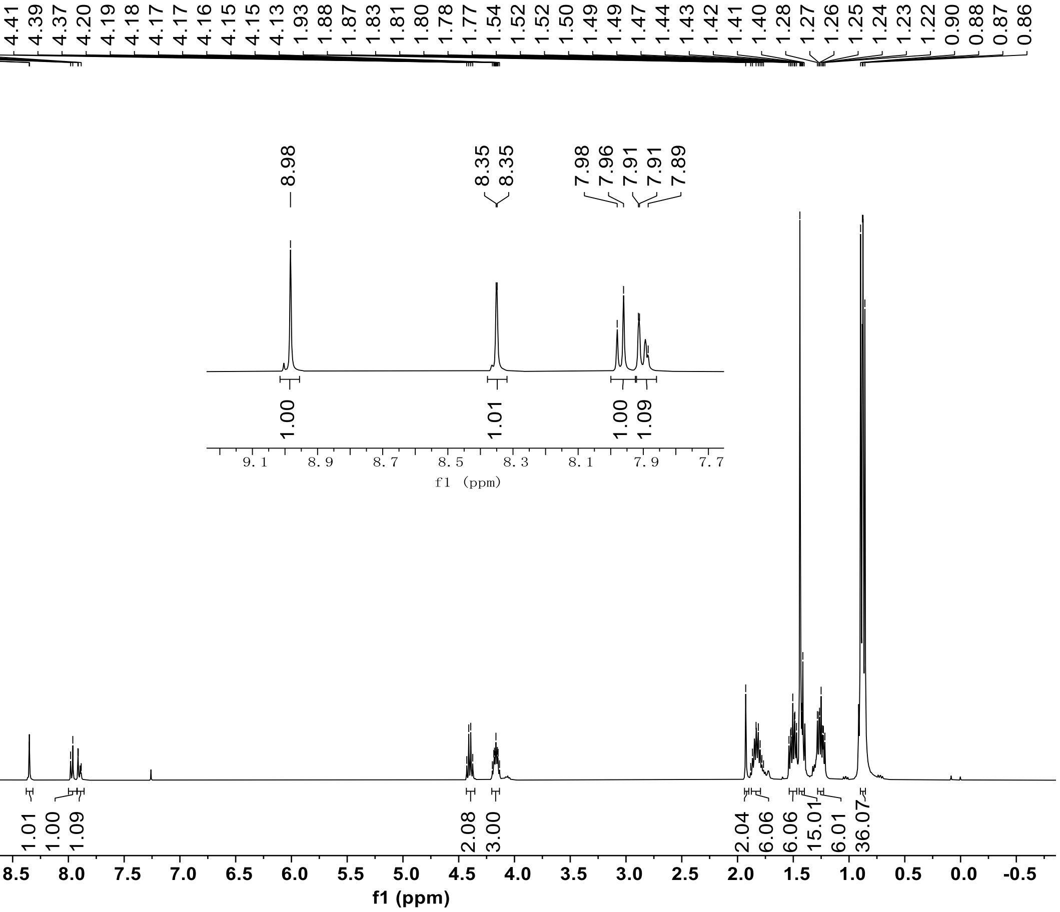

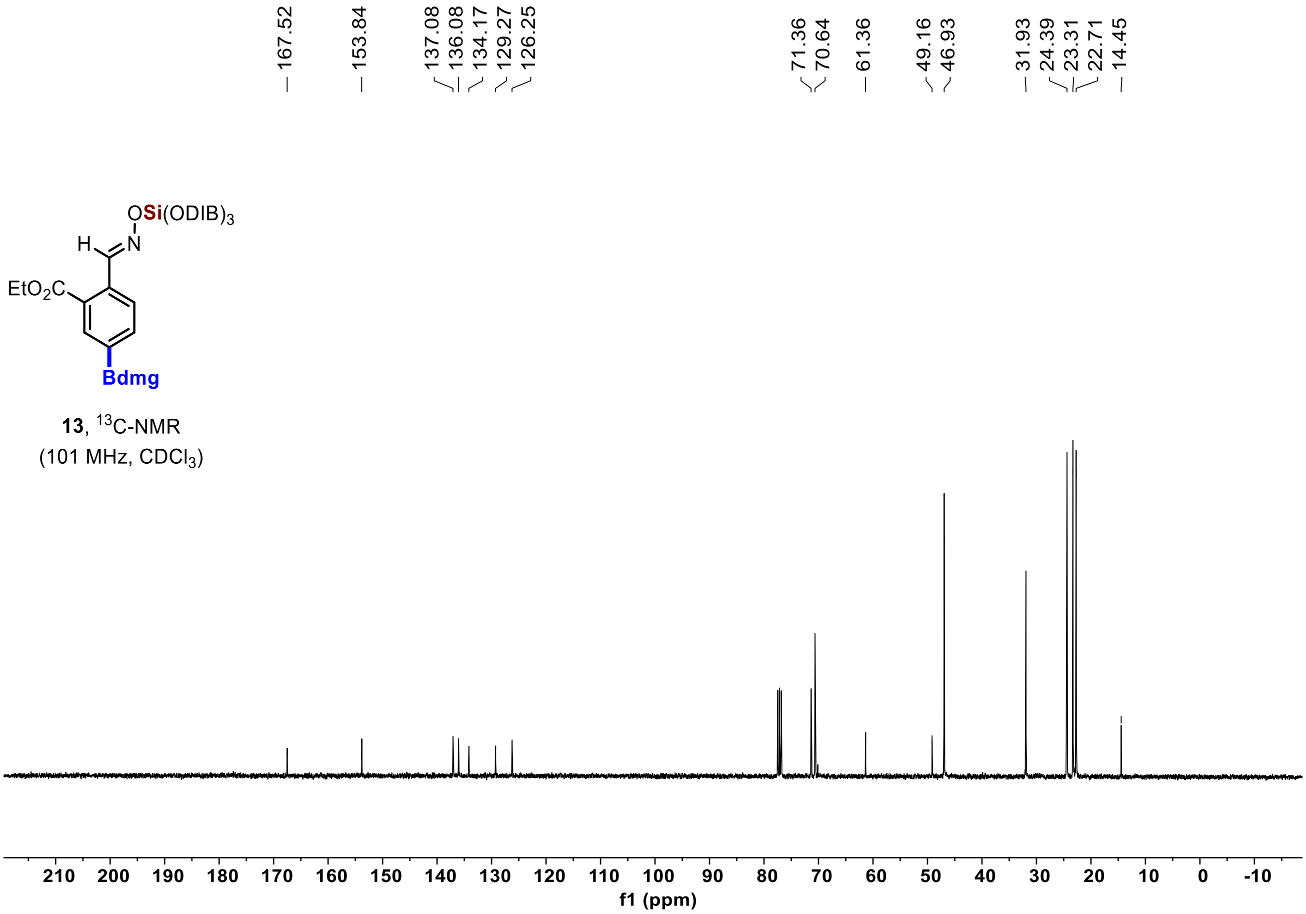
167.52
153.84
137.08
136.08
134.17
129.27
126.25
71.36
70.64
61.36
49.16
46.93
31.93
24.39
23.31
22.71
14.45
OSi(ODIB)3
H
N
EtO2C
Bdmg
13, 13C-NMR
(101 MHz, CDCl3)
f1 (ppm)

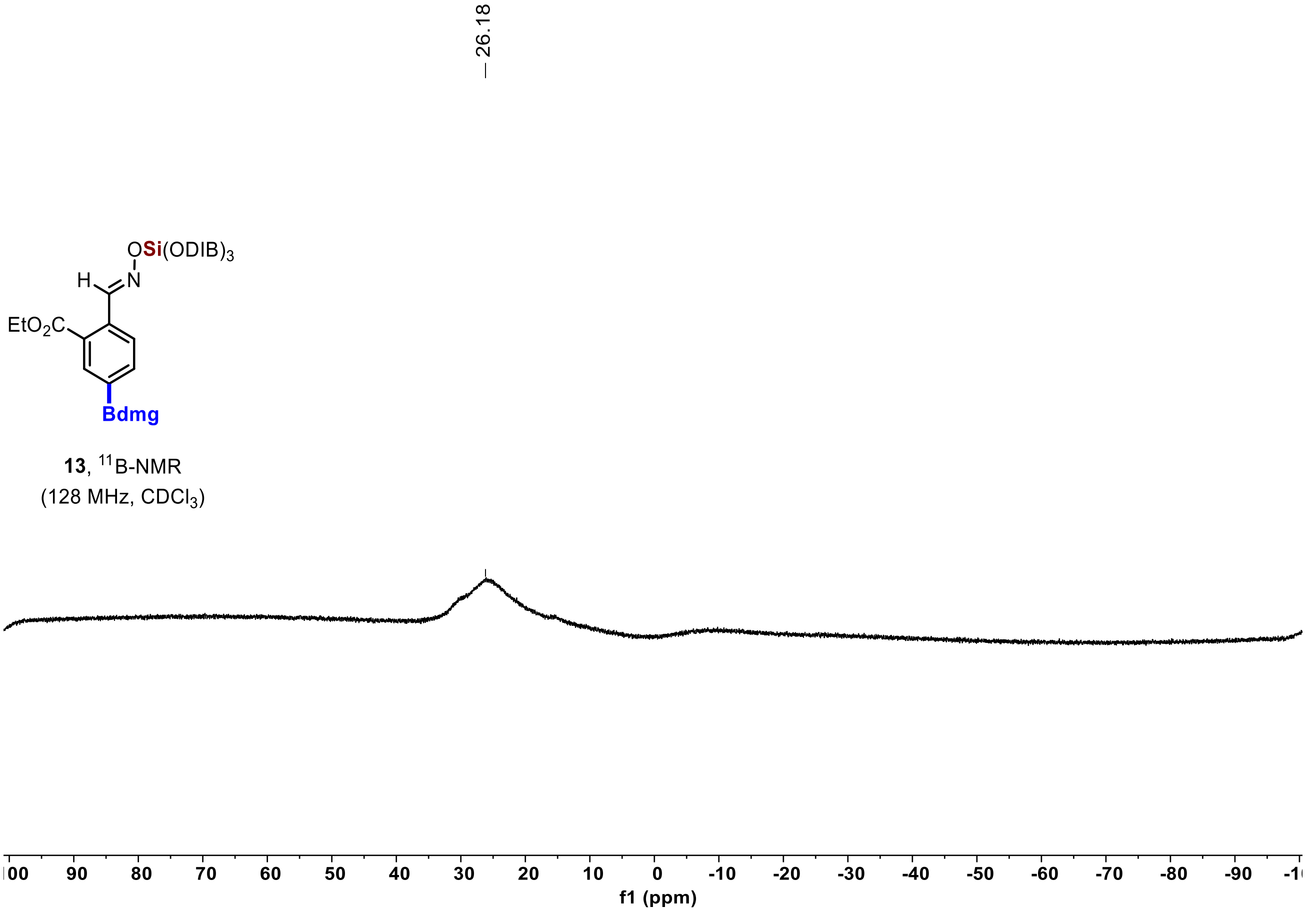
26.18
OSi(ODIB)3
H
N
EtO2C
Bdmg
13, 11B-NMR
(128 MHz, CDCl3)
f1 (ppm)

NOESY spectrum of 13 (400 MHz, CDCl3)

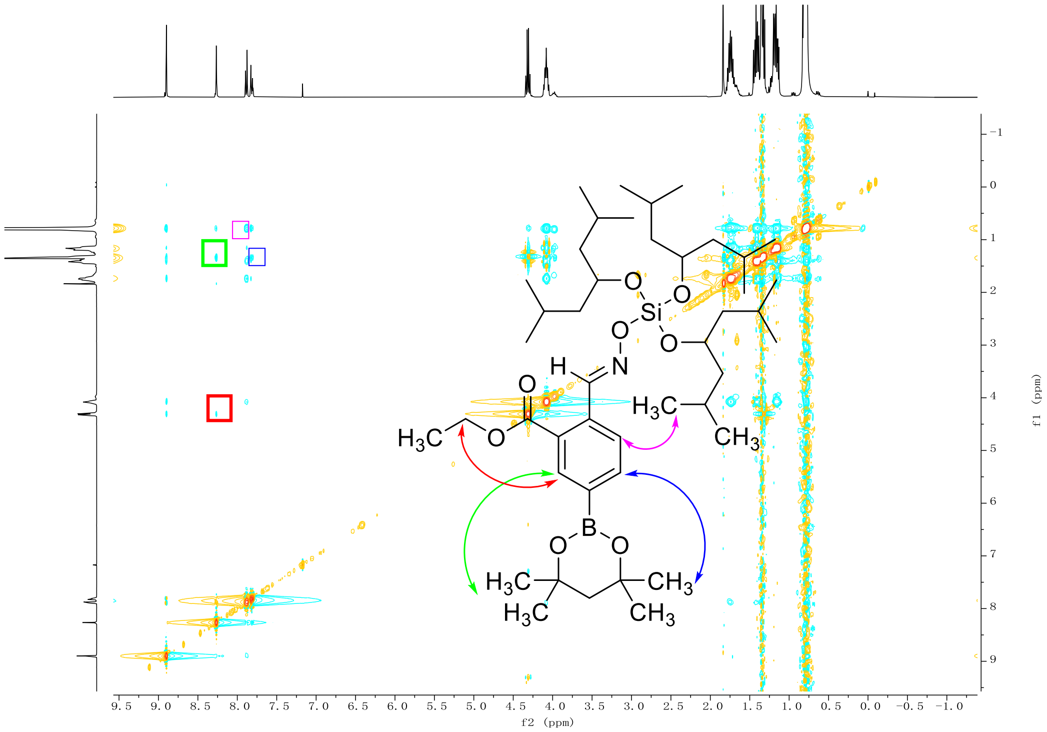

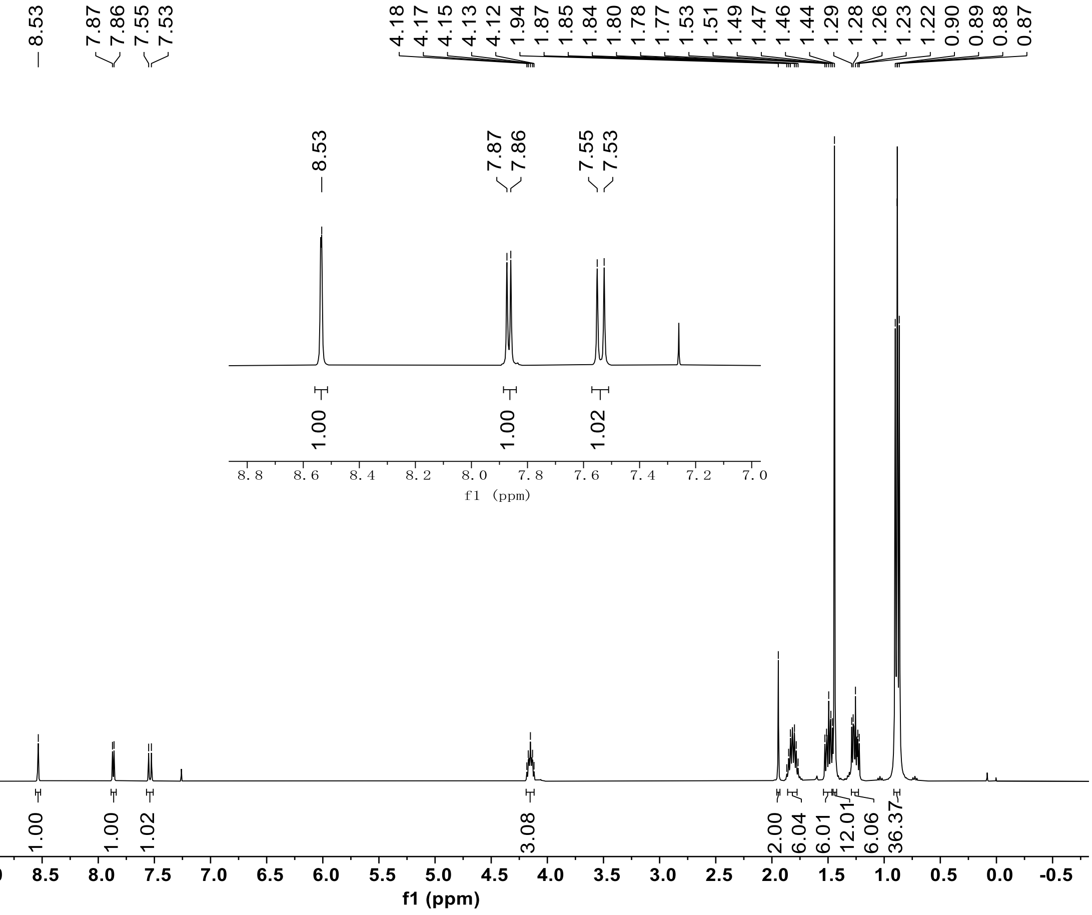

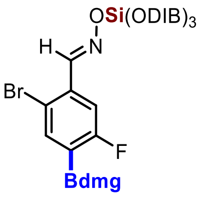

14, ^{1}H-NMR
(400 MHz, $CDCl_3$)

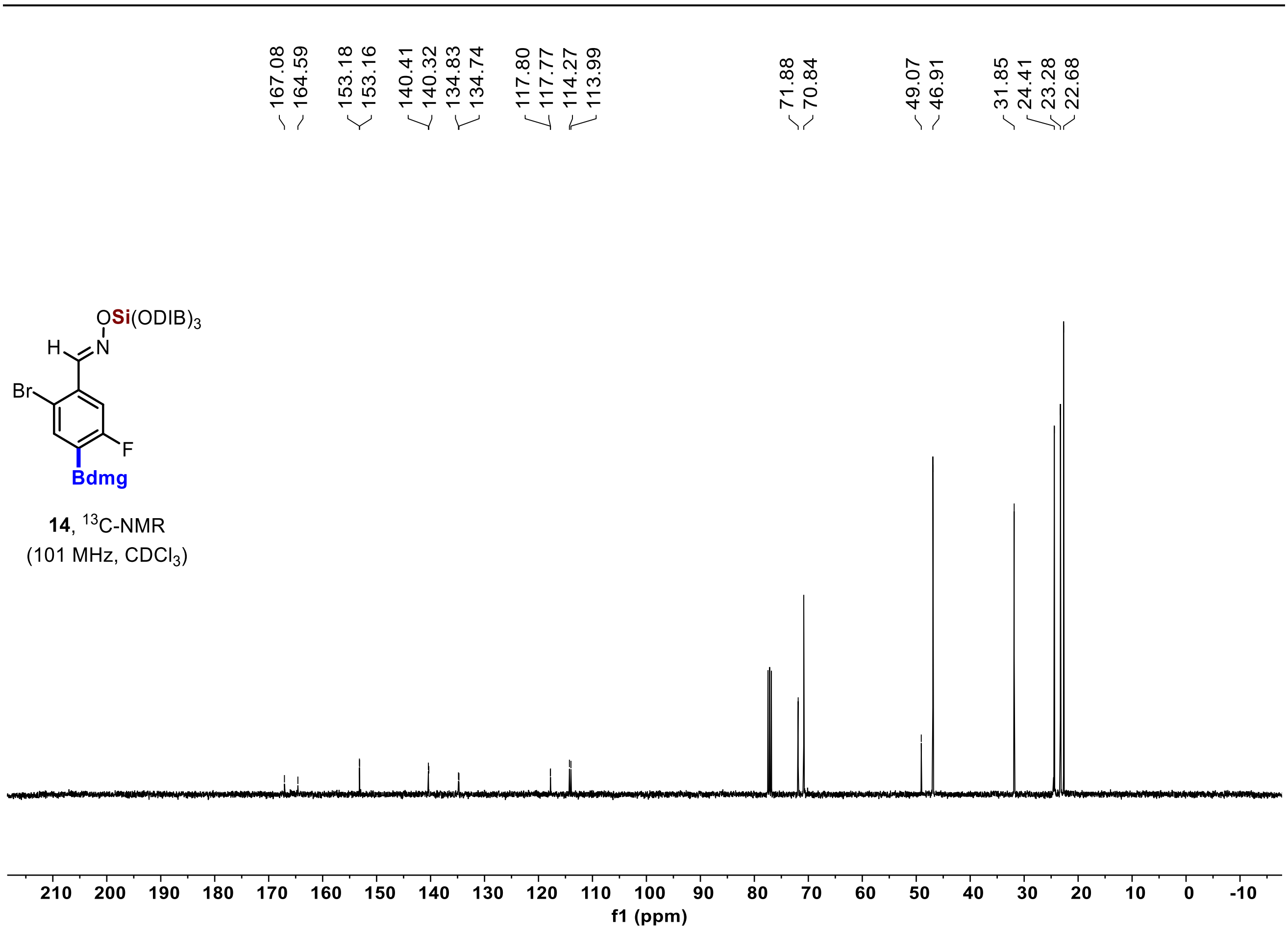
167.08
164.59
153.18
153.16
140.41
140.32
134.83
134.74
117.80
117.77
114.27
113.99
71.88
70.84
49.07
46.91
31.85
24.41
23.28
22.68
OSi(ODIB)3
H
N
Br
F
Bdmg
14, 13C-NMR
(101 MHz, CDCl3)
210 200 190 180 170 160 150 140 130 120 110 100 90 80 70 60 50 40 30 20 10 0 -10
f1 (ppm)

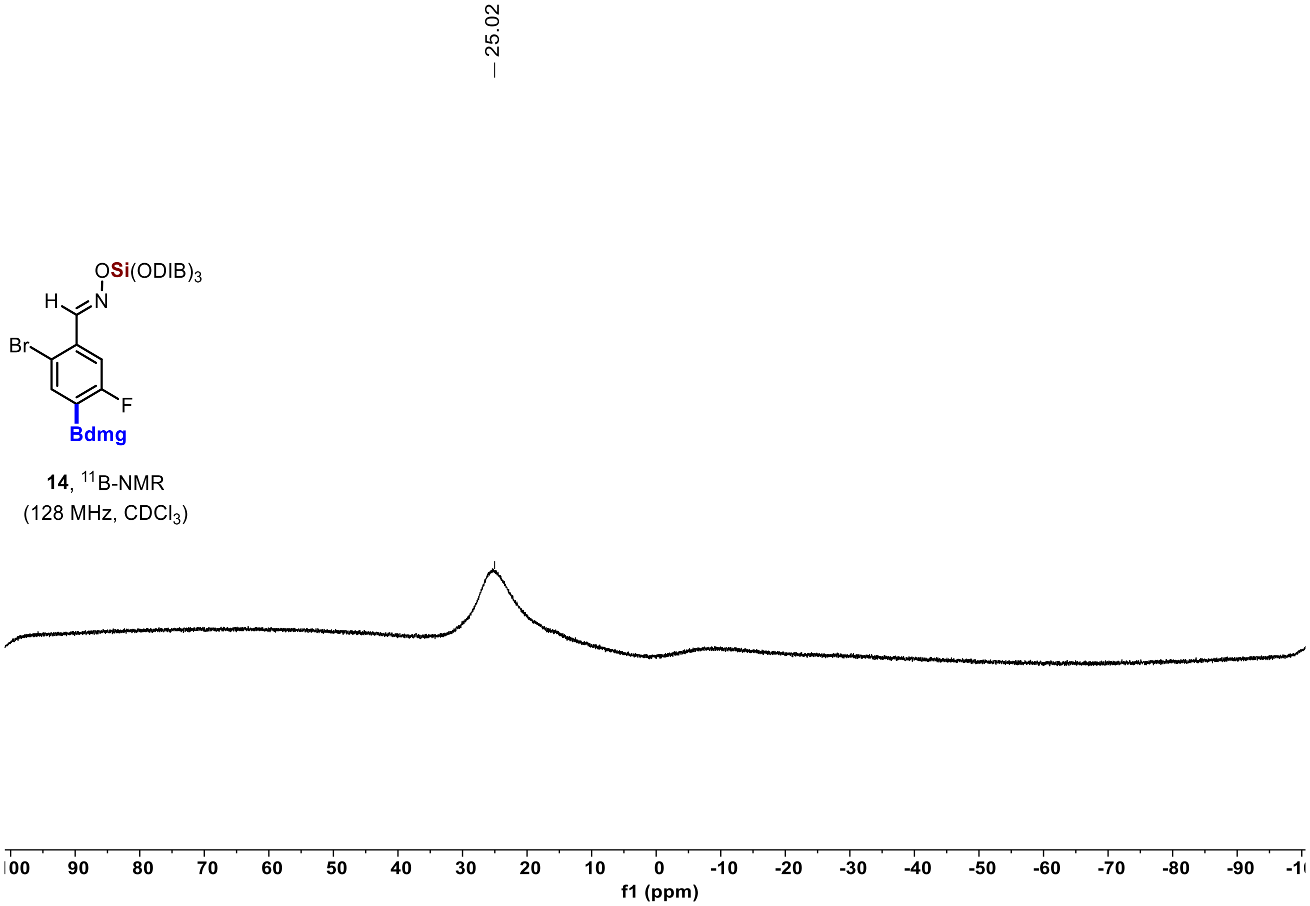
25.02
OSi(ODIB)3
H
N
Br
F
Bdmg
14, 11B-NMR
(128 MHz, CDCl3)
90 80 70 60 50 40 30 20 10 0 -10 -20 -30 -40 -50 -60 -70 -80 -90
f1 (ppm)

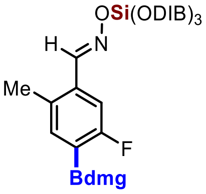

15, ^{1}H-NMR
(400 MHz, $CDCl_3$)

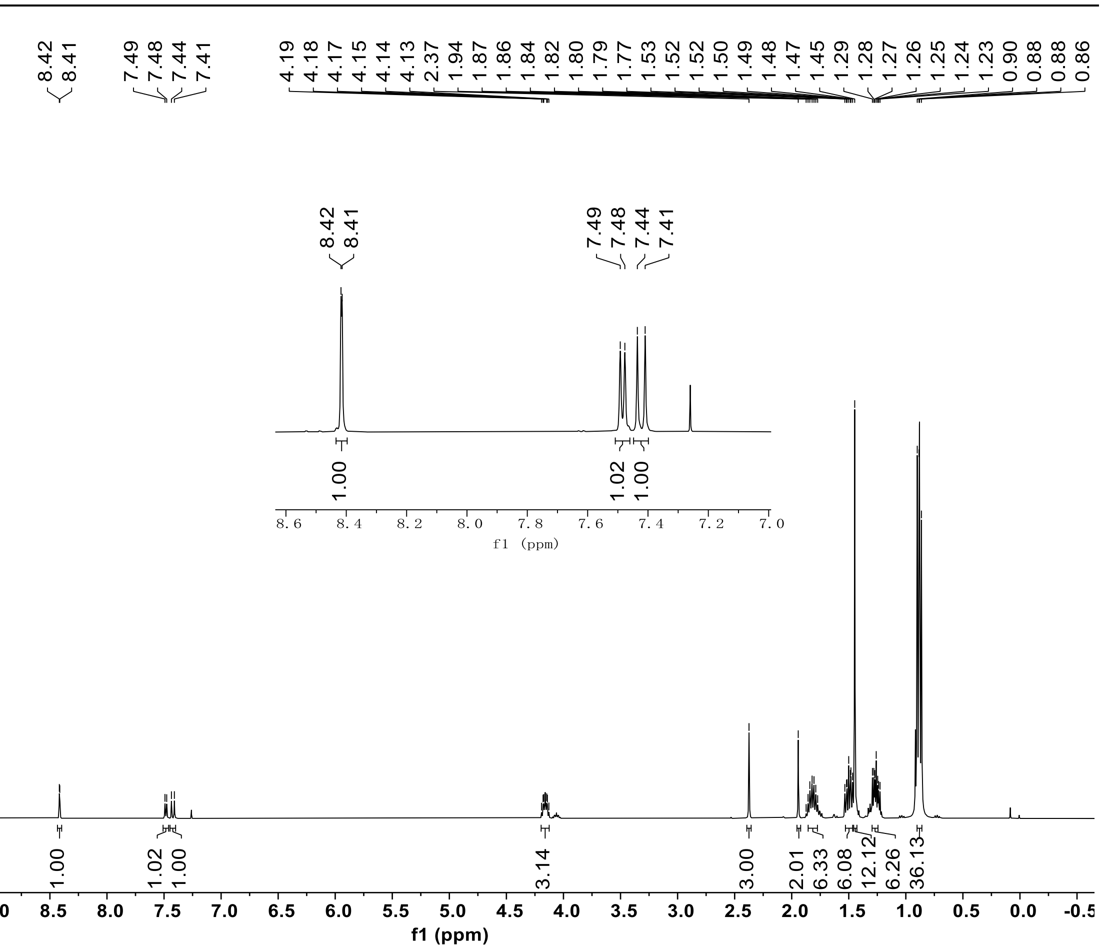

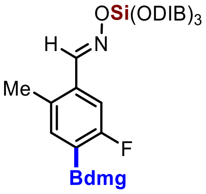

15, ^{13}C-NMR
(101 MHz, $CDCl_3$)

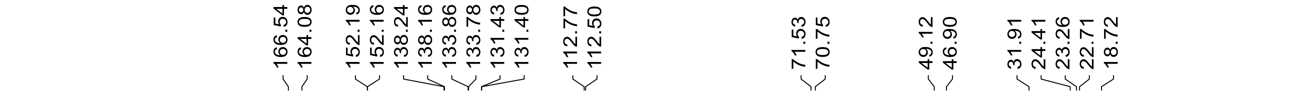

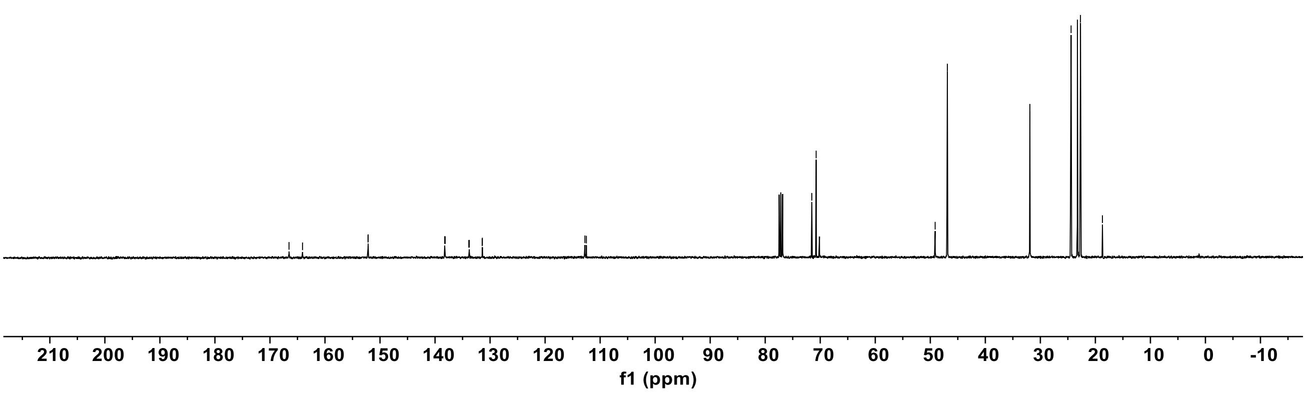

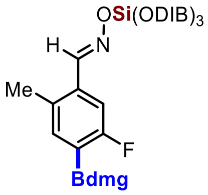

15, ^{19}F-NMR
(376 MHz, $CDCl_3$)

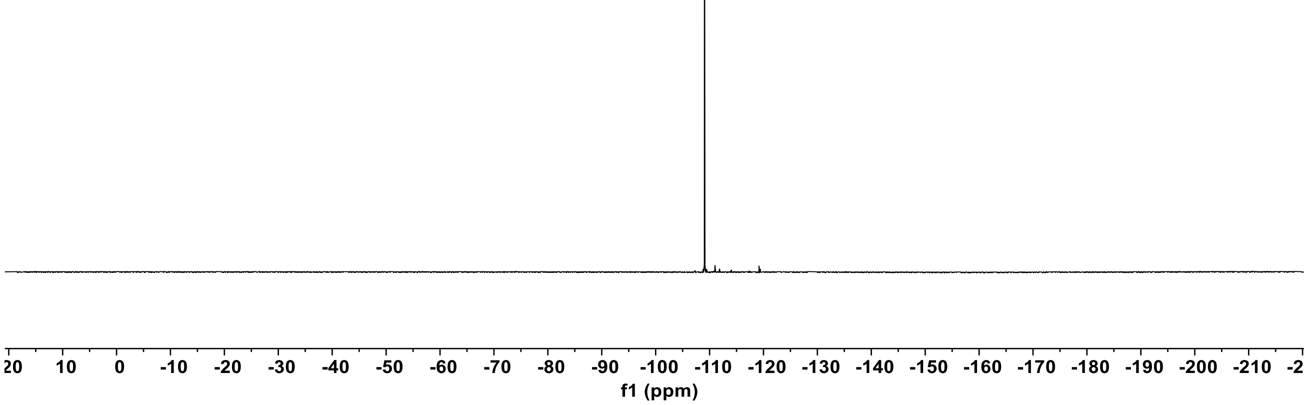

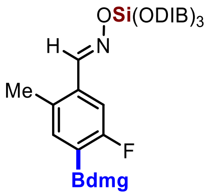

15, ^{11}B-NMR
(128 MHz, $CDCl_3$)

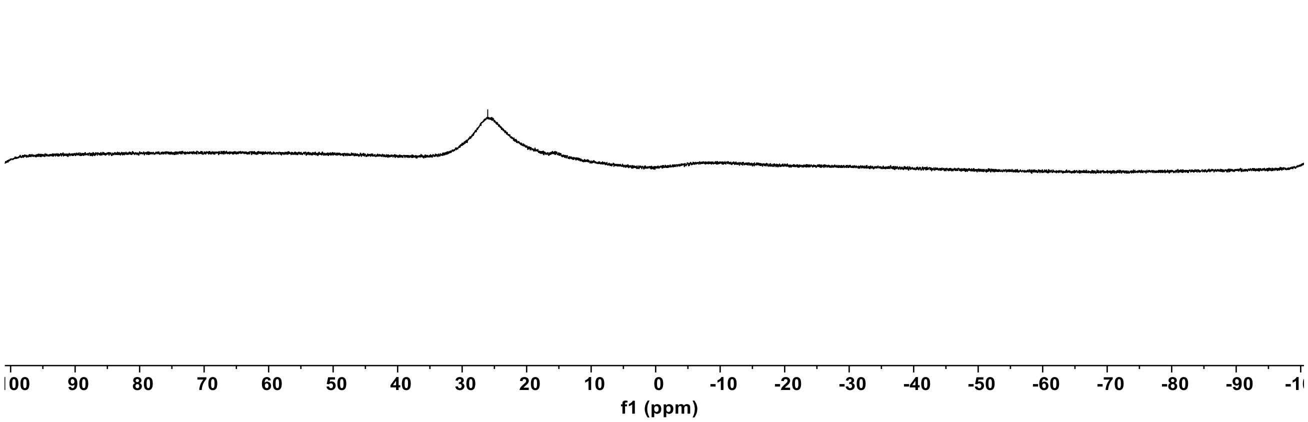

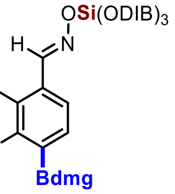

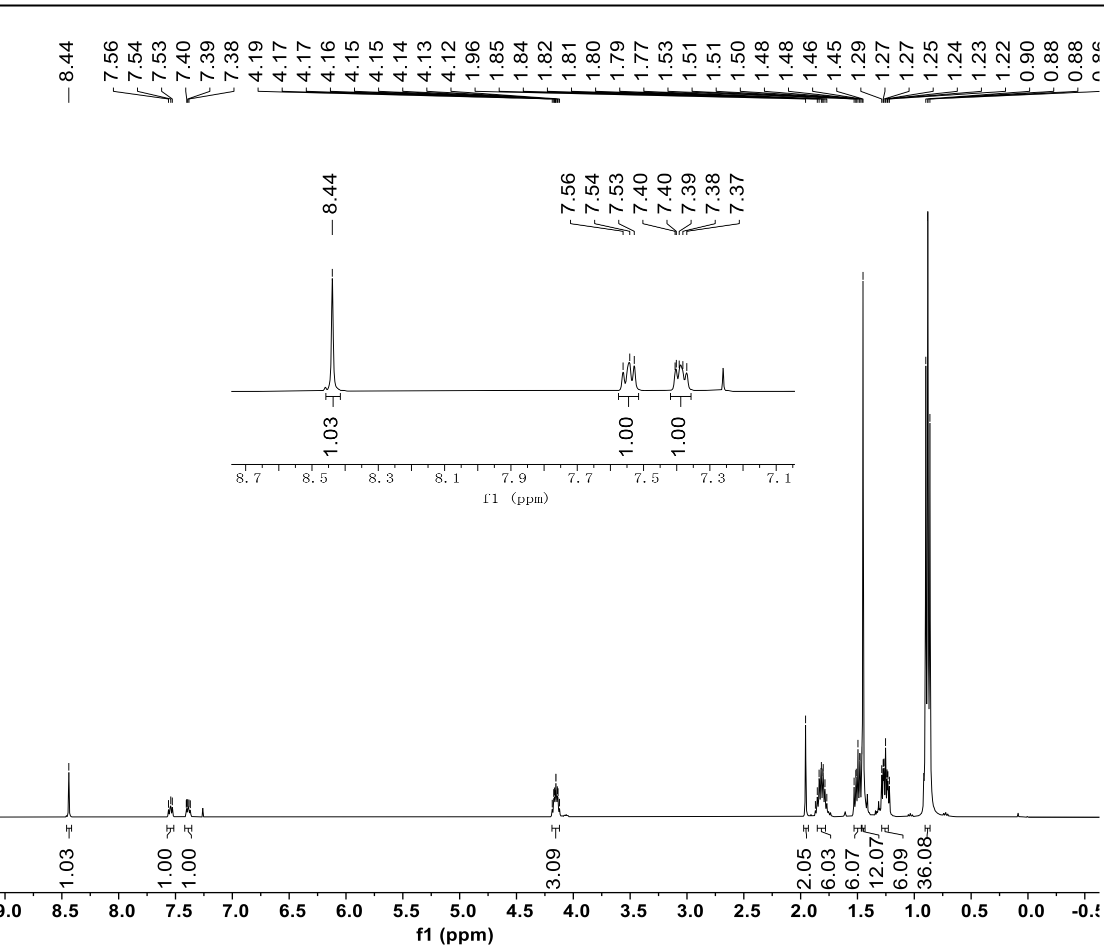

16, ^{1}H-NMR
(400 MHz, $CDCl_3$)

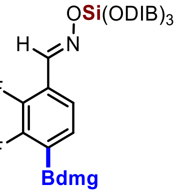

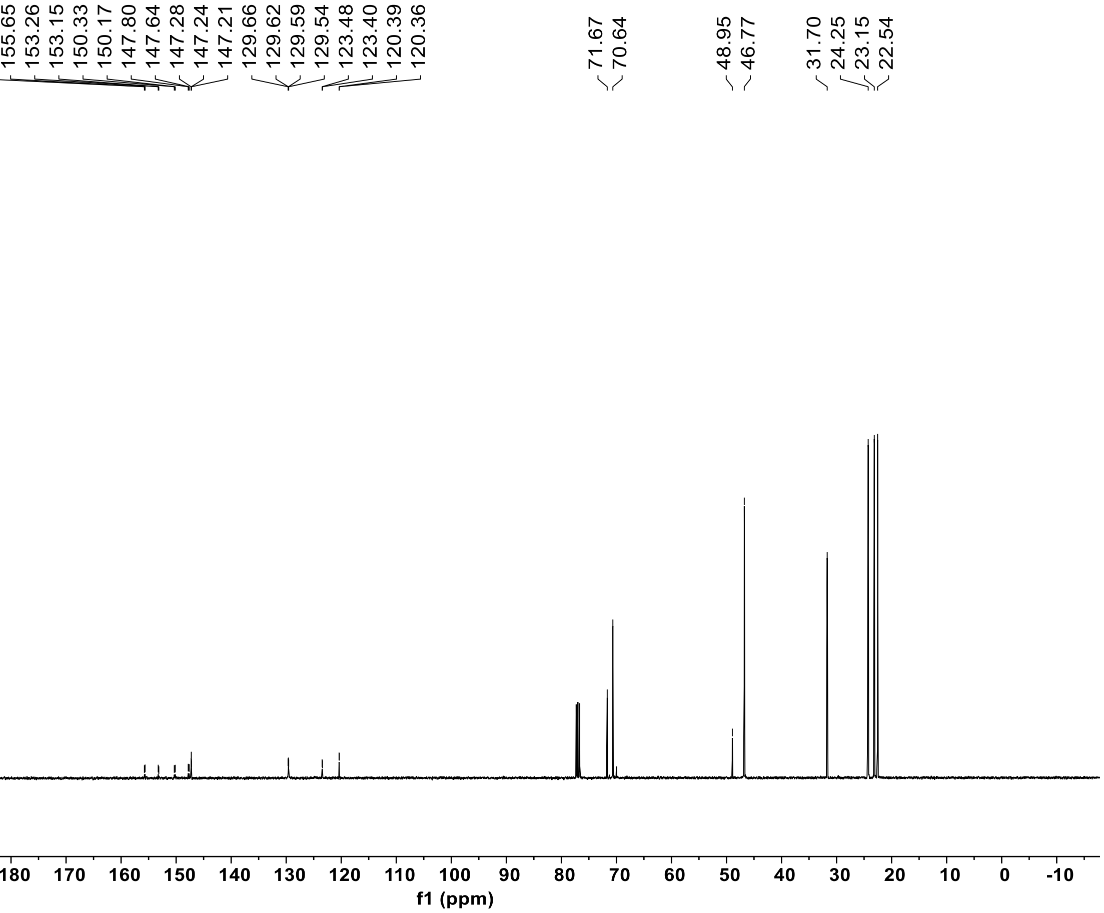

16, ^{13}C-NMR
(101 MHz, $CDCl_3$)

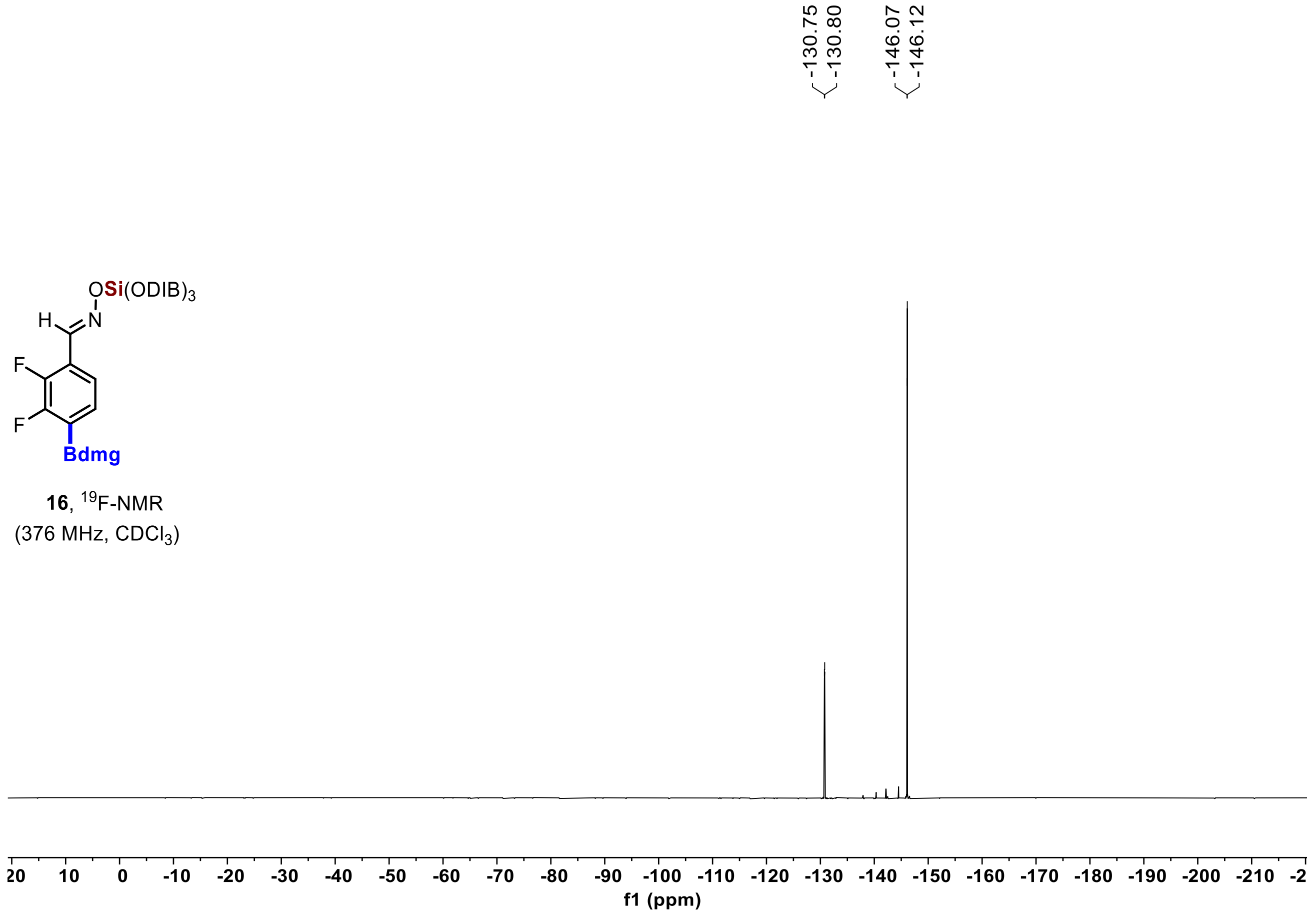

16, ^{19}F-NMR
(376 MHz, $CDCl_3$)

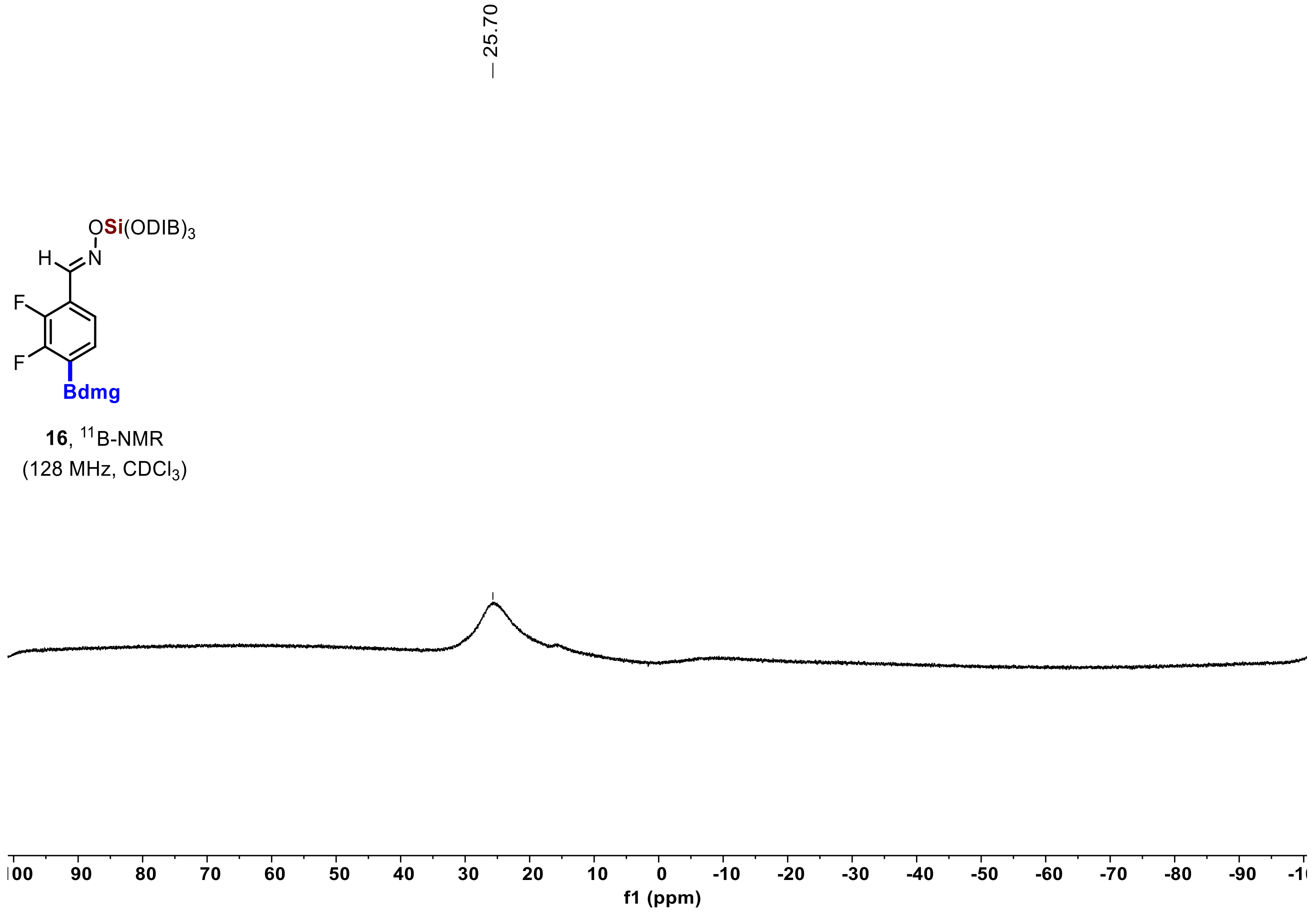

16, ^{11}B-NMR
(128 MHz, $CDCl_3$)

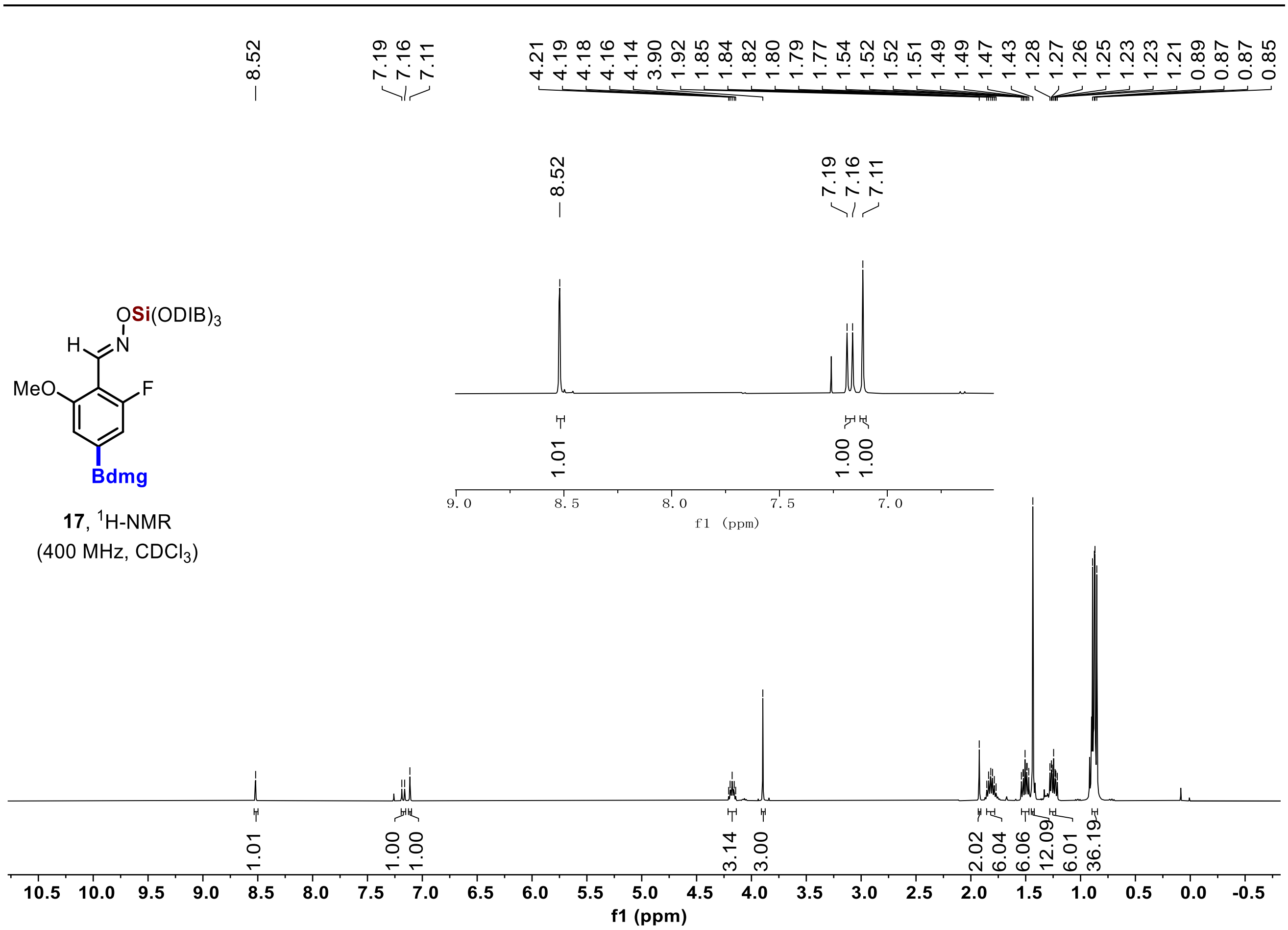
OSi(ODIB)3
H
N
MeO
F
Bdmg
17, 1H-NMR
(400 MHz, CDCl3)
8.52
7.19
7.16
7.11
4.21 4.19 4.18 4.16 4.14 3.90 1.92 1.85 1.84 1.82 1.80 1.79 1.77 1.54 1.52 1.52 1.51 1.49 1.49 1.47 1.43 1.28 1.27 1.26 1.25 1.23 1.23 1.21 0.89 0.87 0.87 0.85
1.01
1.00
1.00
3.14
3.00
2.02
6.04
6.06
12.09
6.01
36.19
f1 (ppm)

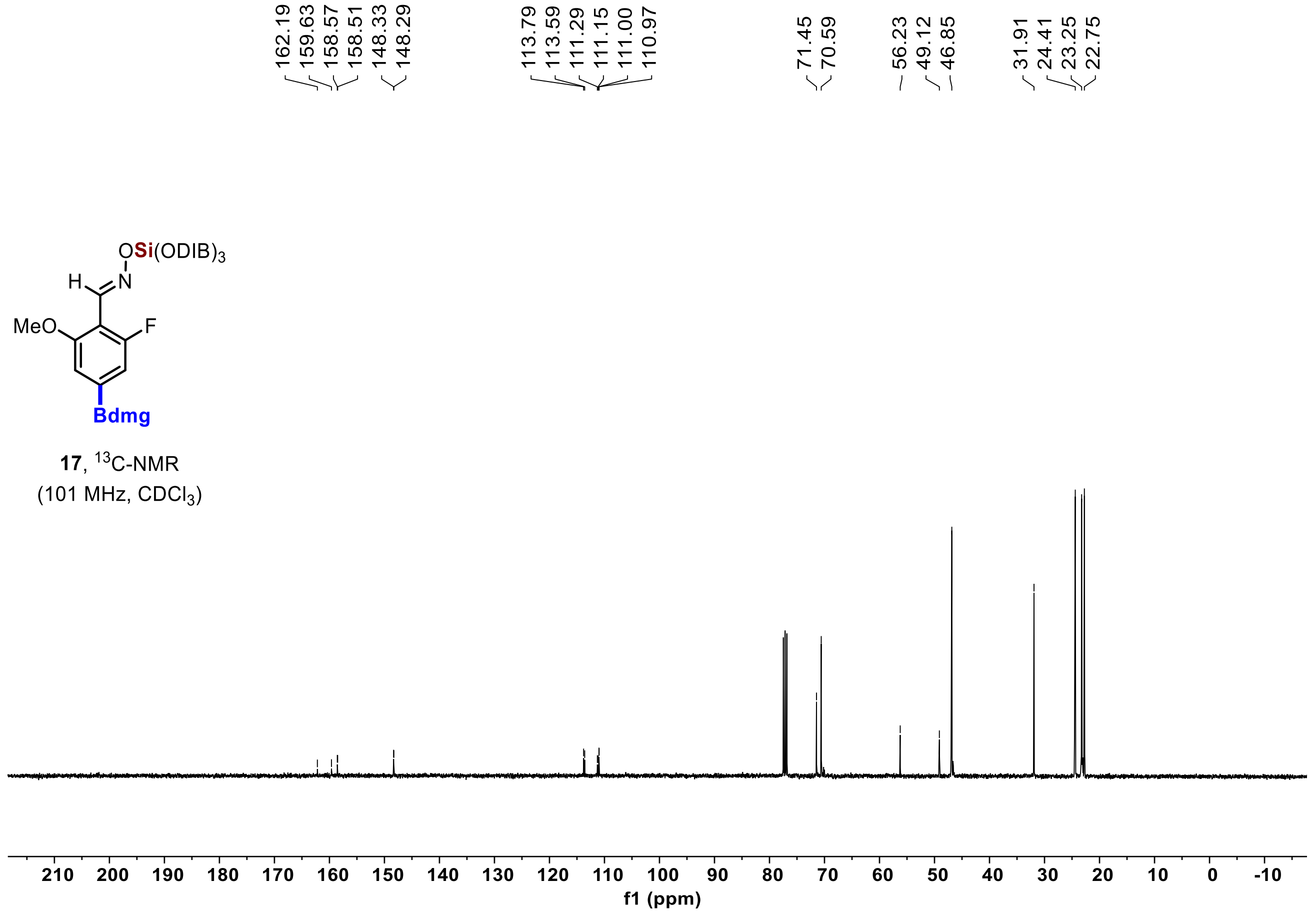
OSi(ODIB)3
H
N
MeO
F
Bdmg
17, 13C-NMR
(101 MHz, CDCl3)
162.19 159.63 158.57 158.51 148.33 148.29
113.79 113.59 111.29 111.15 111.00 110.97
71.45 70.59
56.23 49.12 46.85
31.91 24.41 23.25 22.75
f1 (ppm)

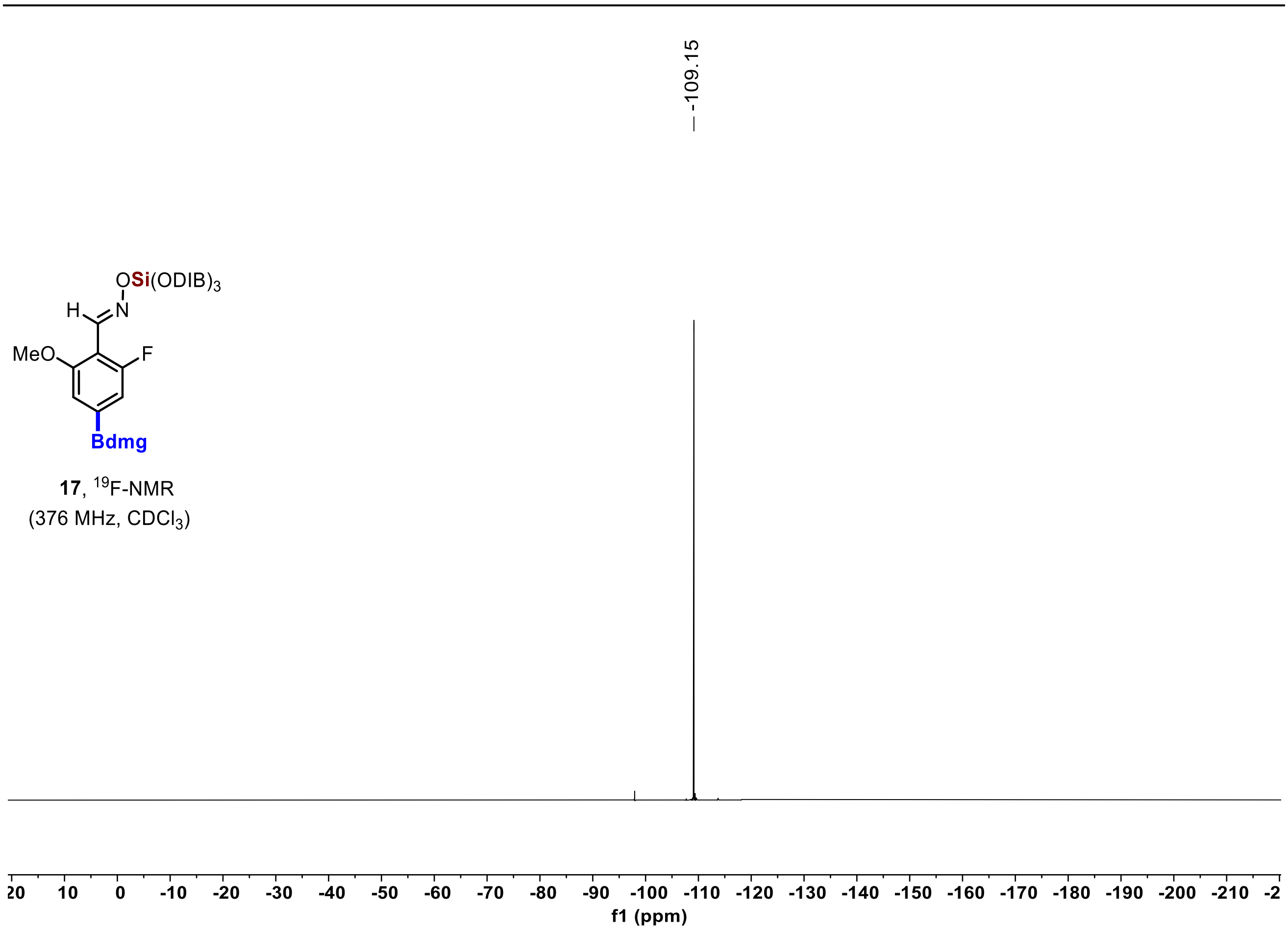

-109.15
OSi(ODIB)3
H
N
MeO
F
Bdmg
17, 19F-NMR
(376 MHz, CDCl3)
20 10 0 -10 -20 -30 -40 -50 -60 -70 -80 -90 -100 -110 -120 -130 -140 -150 -160 -170 -180 -190 -200 -210
f1 (ppm)

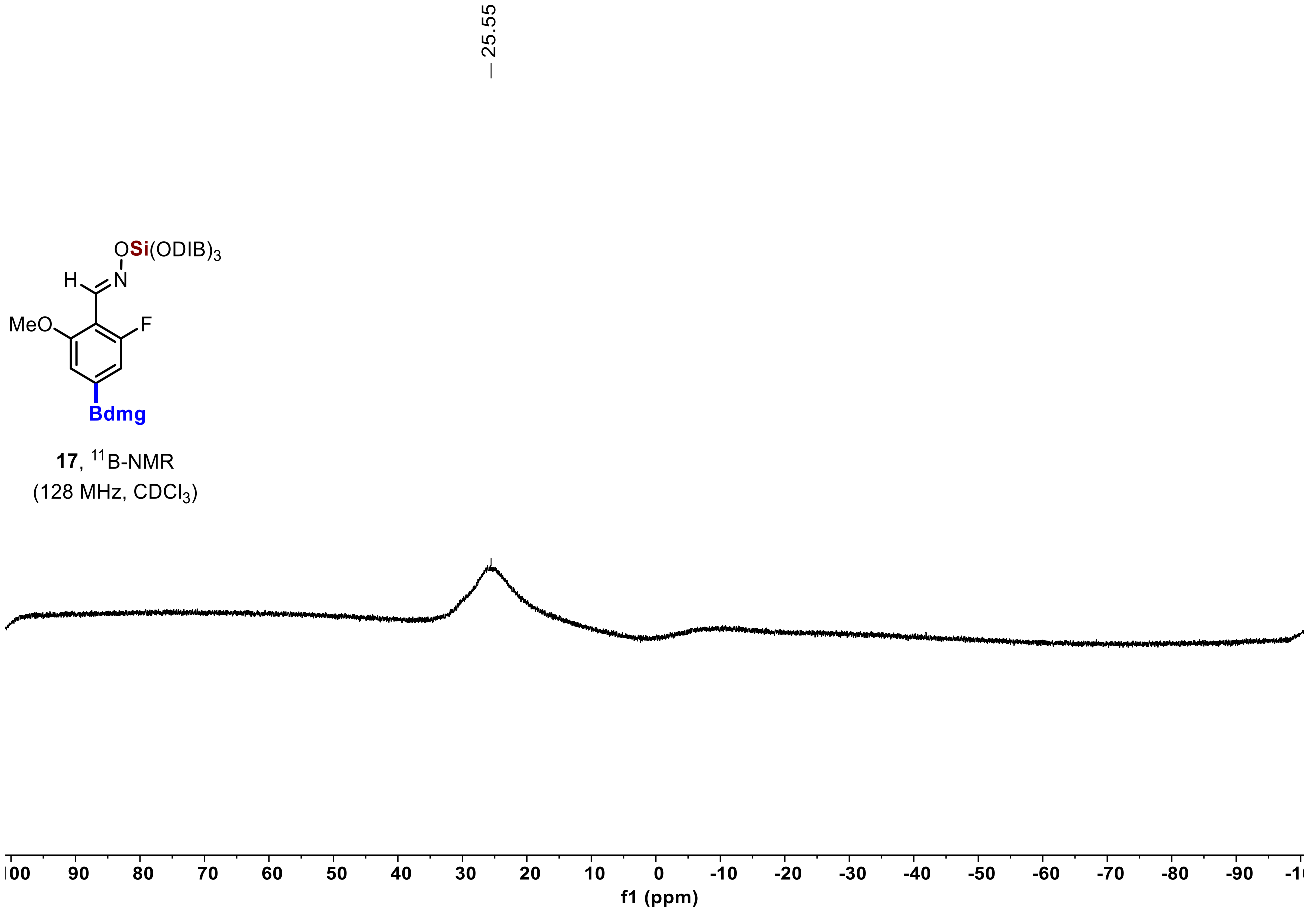

25.55
OSi(ODIB)3
H
N
MeO
F
Bdmg
17, 11B-NMR
(128 MHz, CDCl3)
90 80 70 60 50 40 30 20 10 0 -10 -20 -30 -40 -50 -60 -70 -80 -90
f1 (ppm)

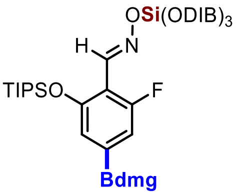

18, ^{1}H-NMR
(400 MHz, $CDCl_3$)

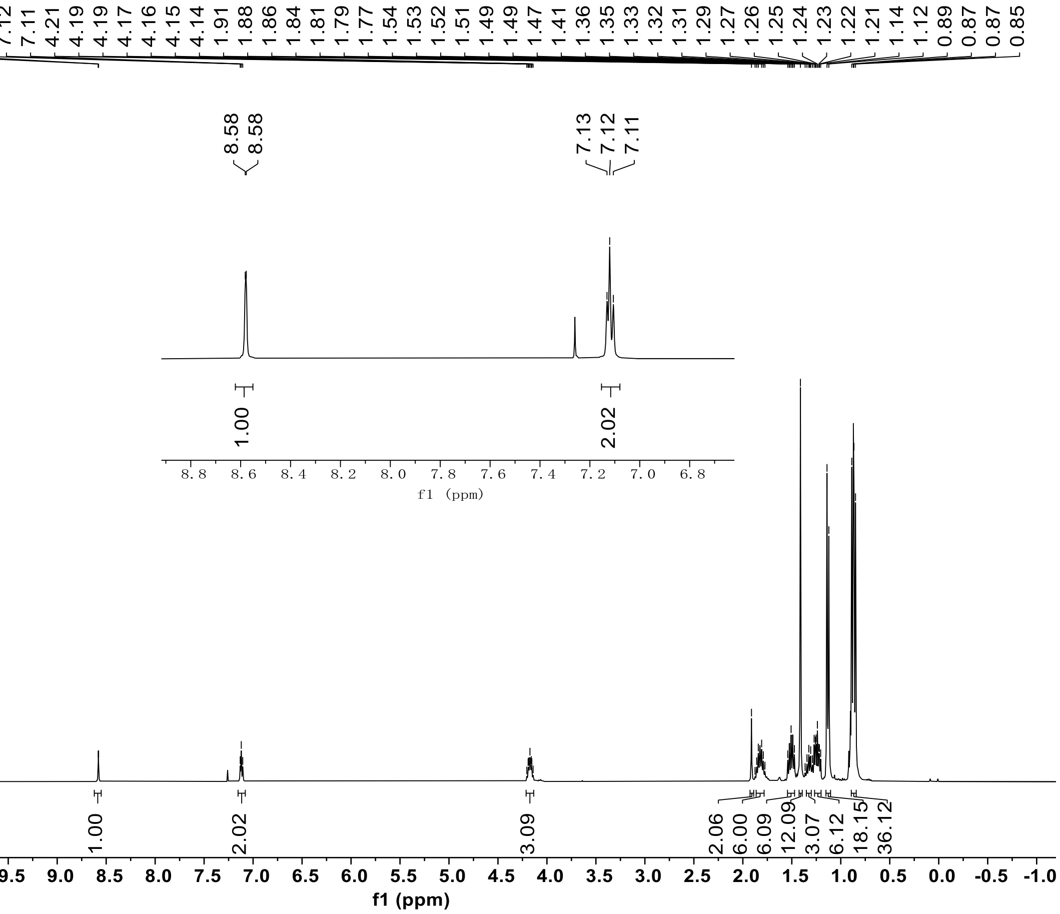

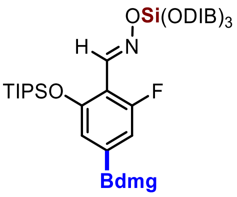

18, ^{13}C-NMR
(101 MHz, $CDCl_3$)

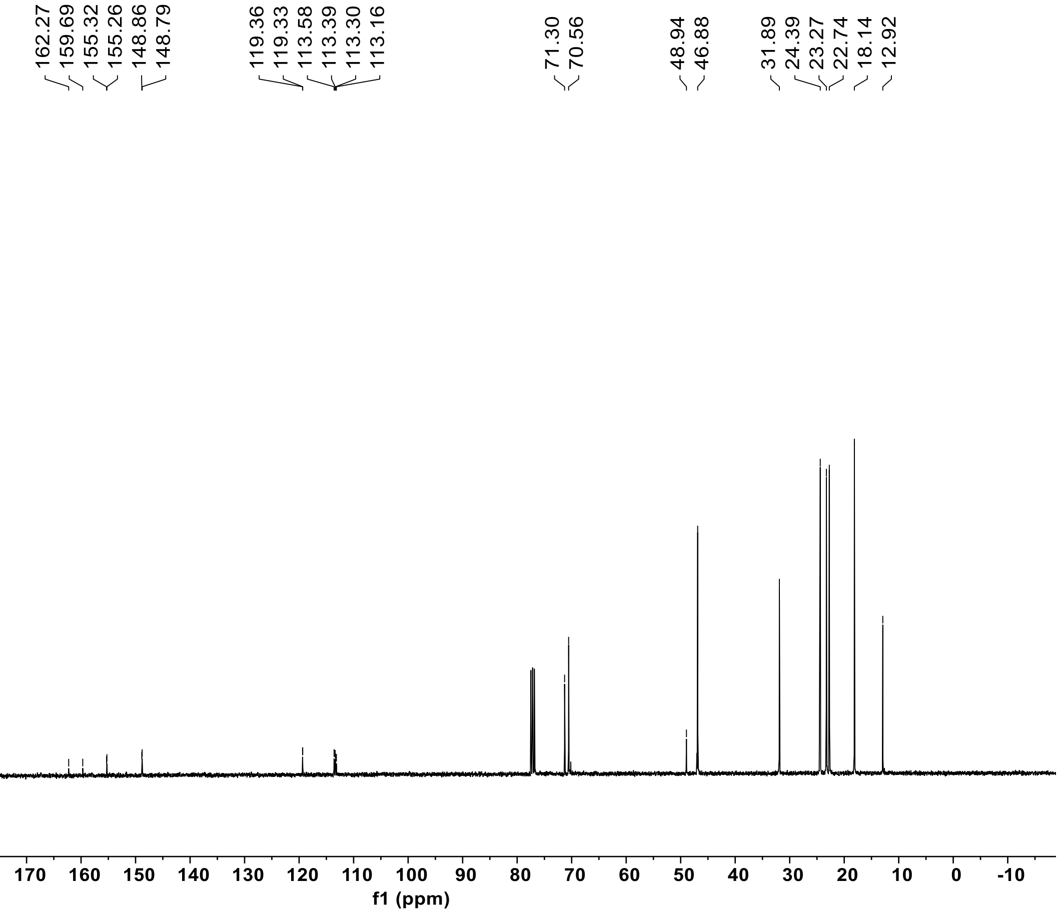

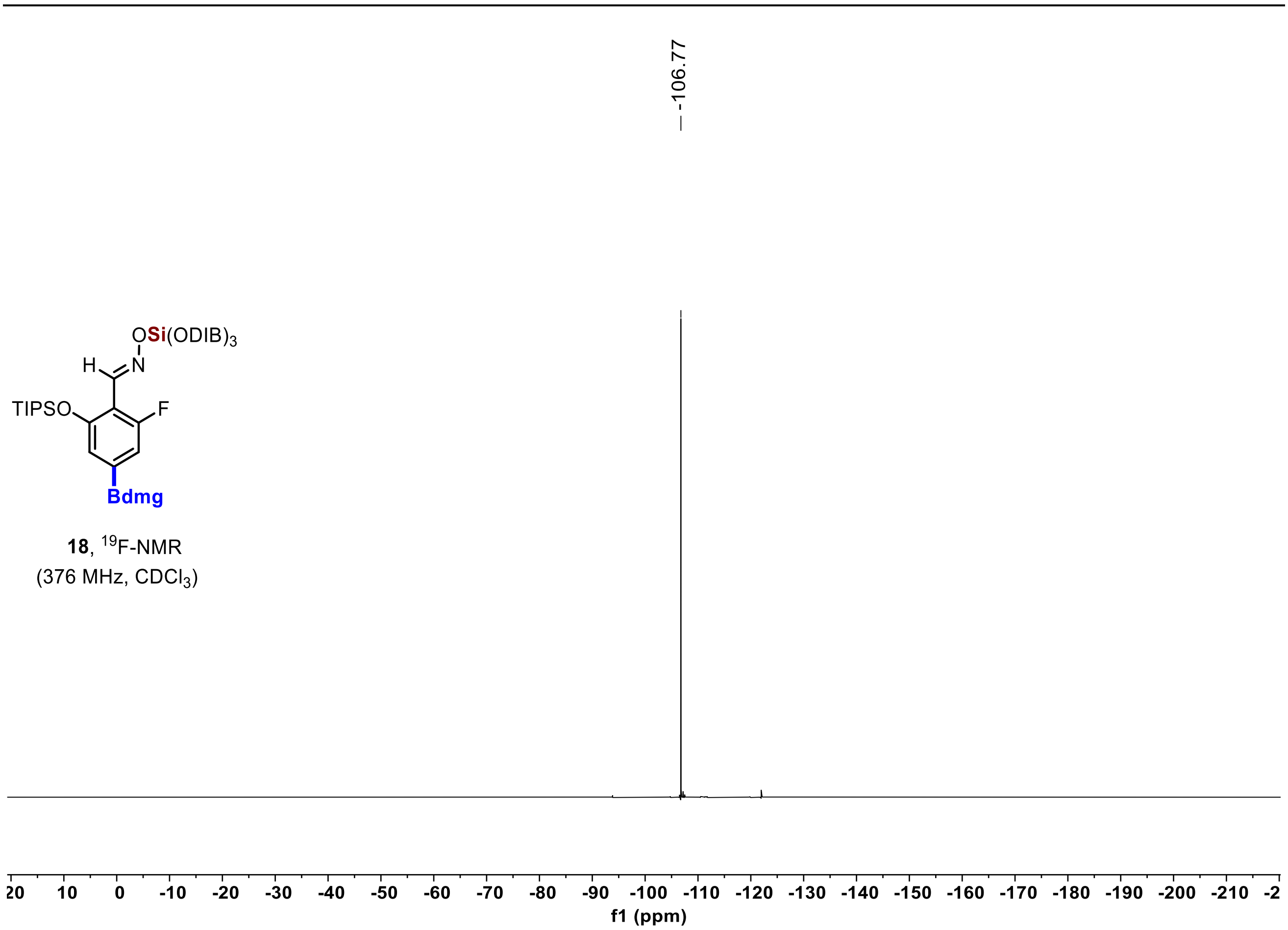
-106.77
OSi(ODIB)3
H
N
TIPSO
F
Bdmg
18, 19F-NMR
(376 MHz, CDCl3)
f1 (ppm)

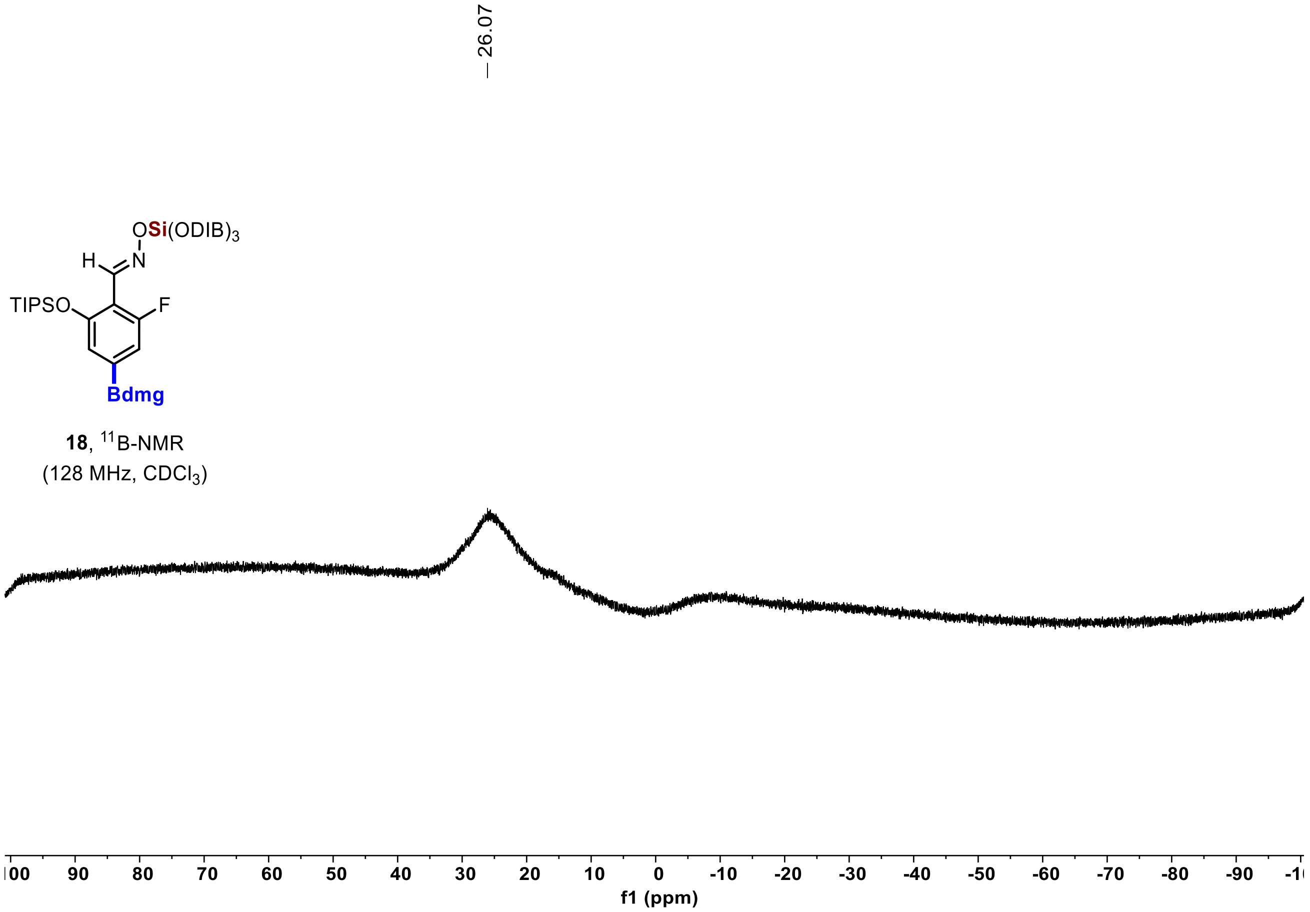
26.07
OSi(ODIB)3
H
N
TIPSO
F
Bdmg
18, 11B-NMR
(128 MHz, CDCl3)
f1 (ppm)

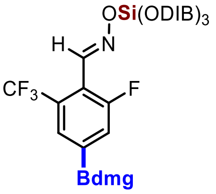

19, ^{1}H-NMR
(400 MHz, $CDCl_3$)

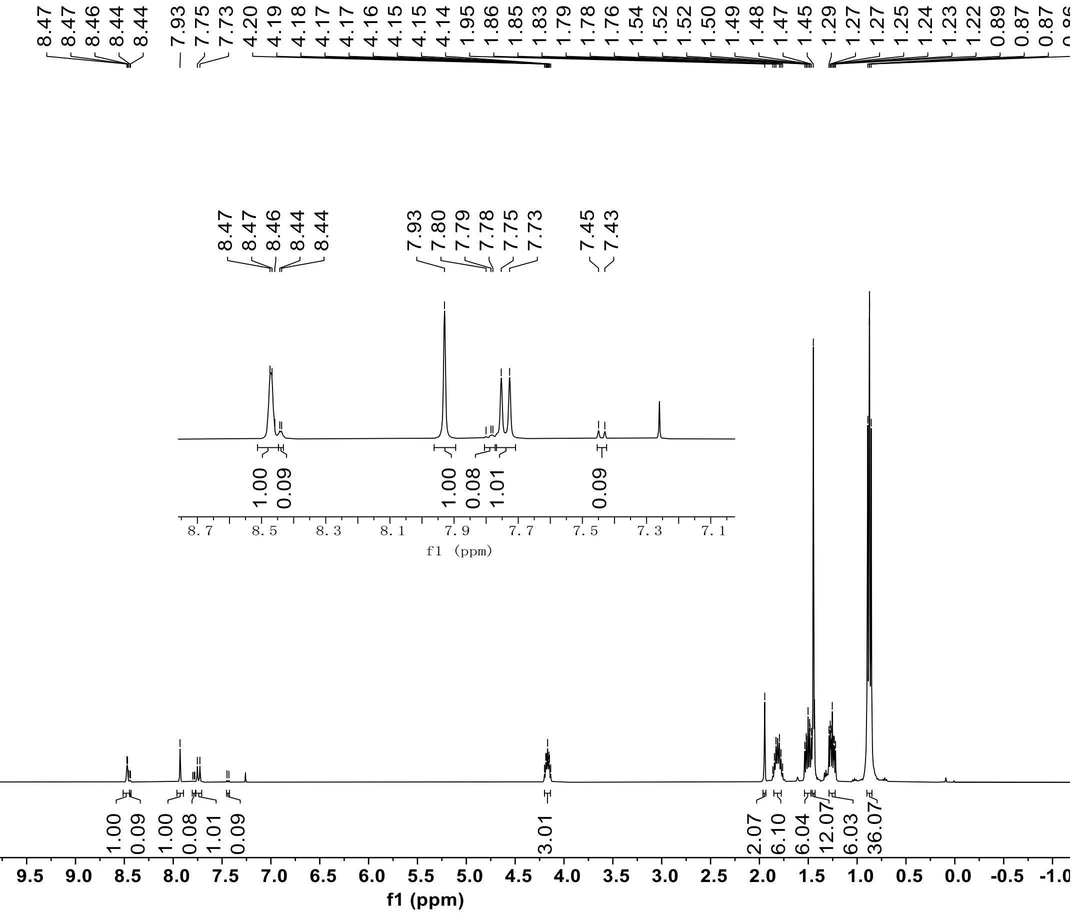

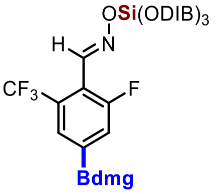

19, ^{13}C-NMR
(101 MHz, $CDCl_3$)

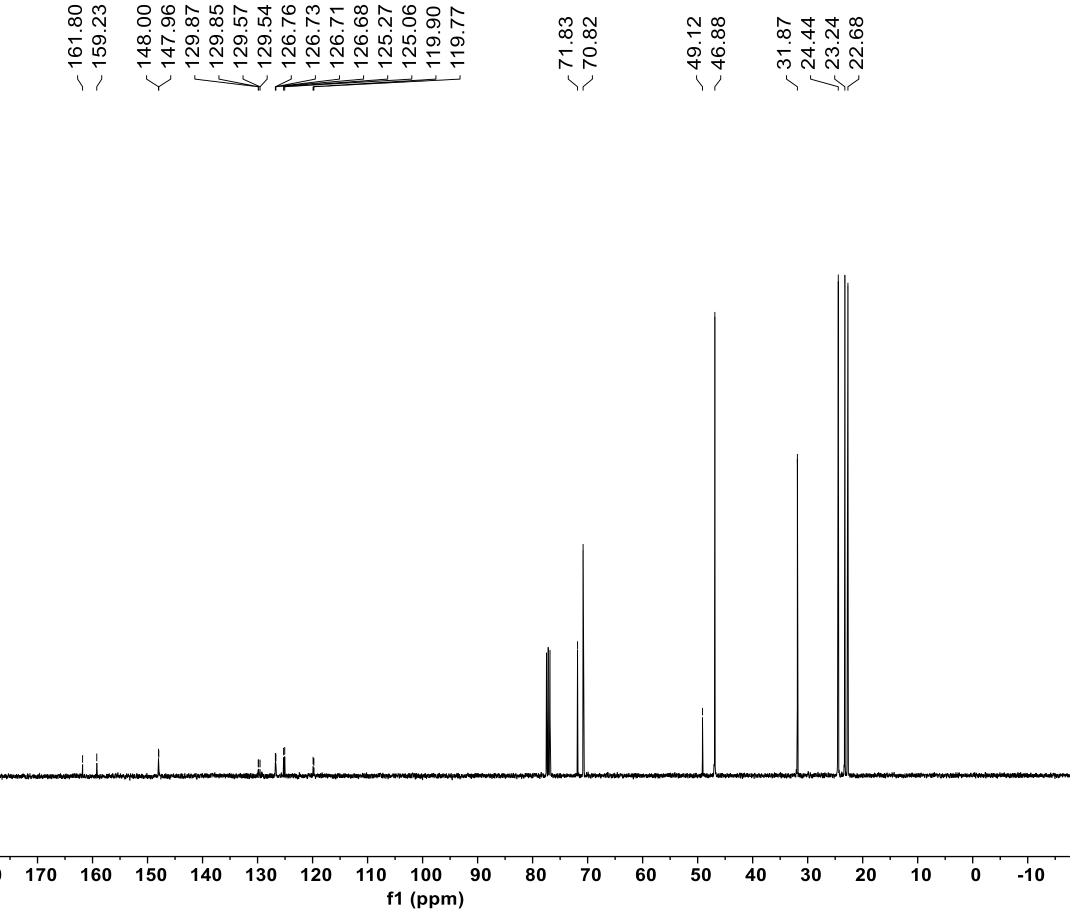

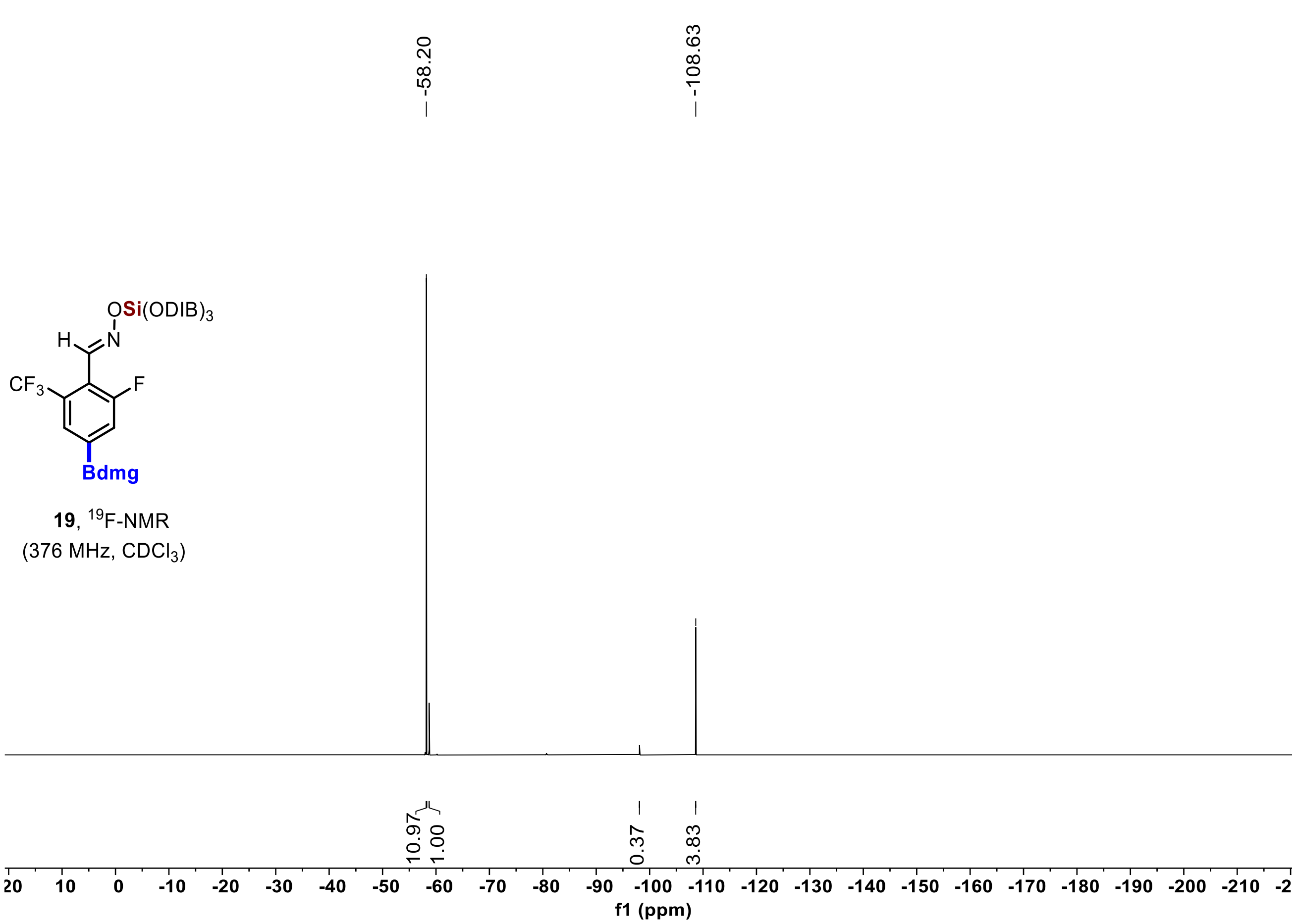

OSi(ODIB)3
H
N
CF3
F
Bdmg
19, 19F-NMR
(376 MHz, CDCl3)
-58.20
-108.63
10.97
1.00
0.37
3.83
f1 (ppm)

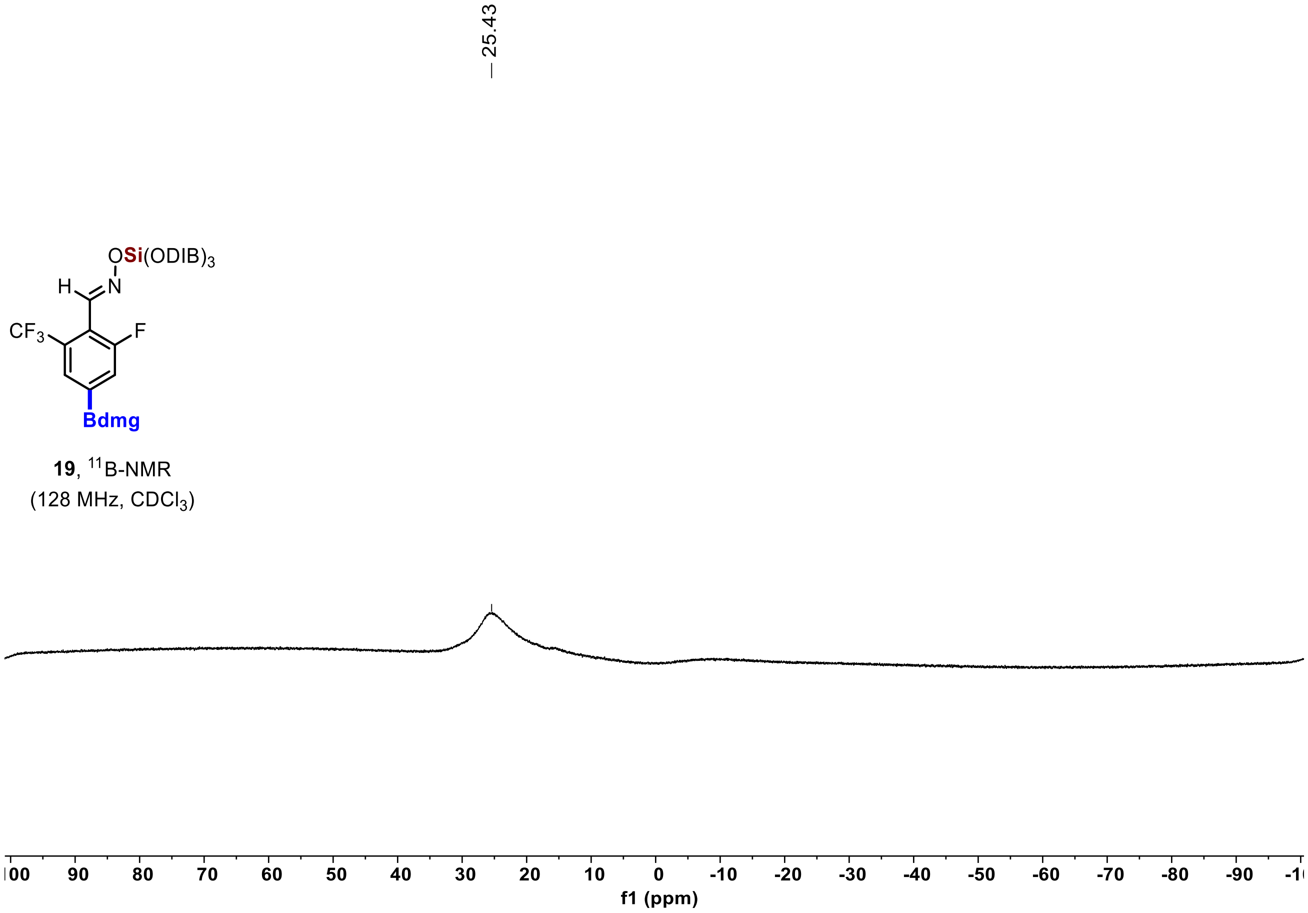

25.43
OSi(ODIB)3
H
N
CF3
F
Bdmg
19, 11B-NMR
(128 MHz, CDCl3)
f1 (ppm)

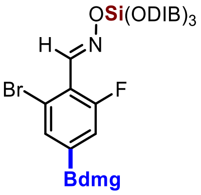

20, ^{1}H-NMR
(400 MHz, CDCl$_3$)

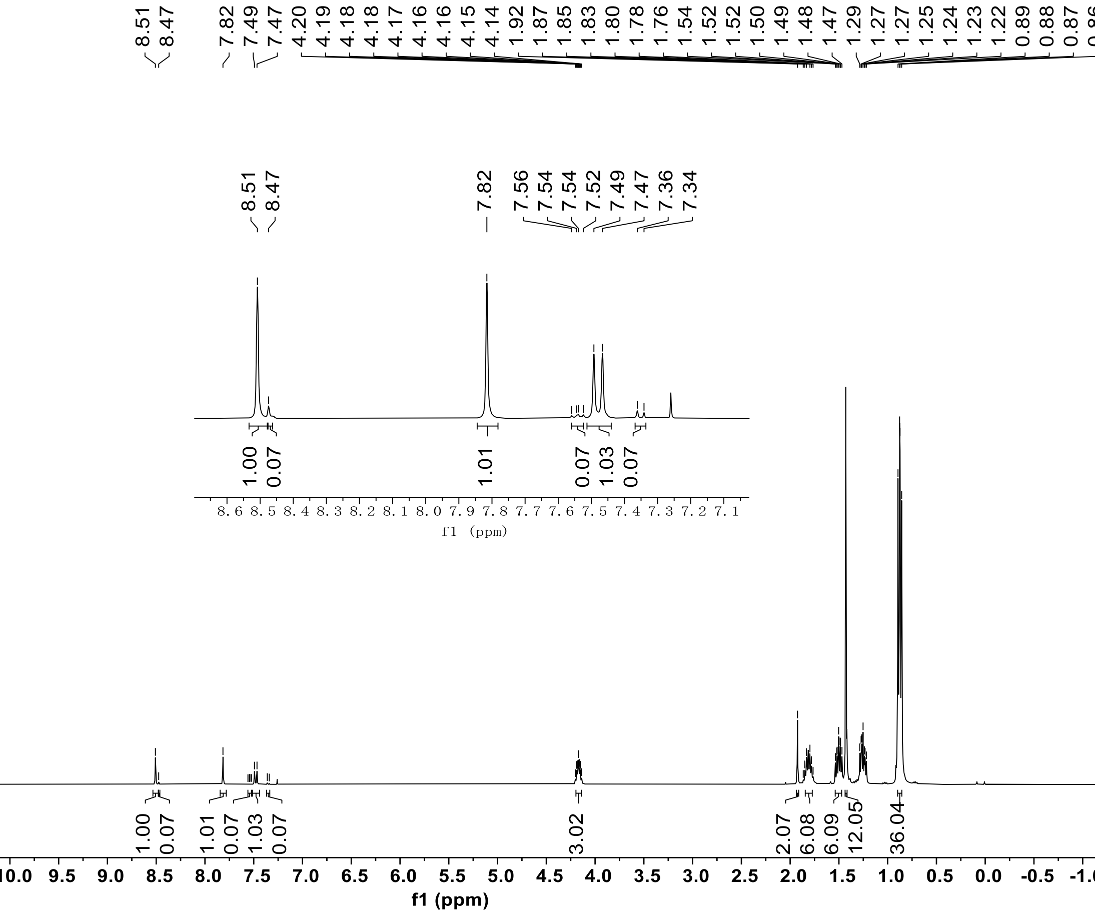

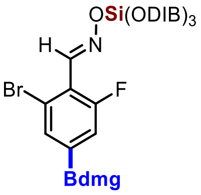

20, ^{13}C-NMR
(101 MHz, CDCl$_3$)

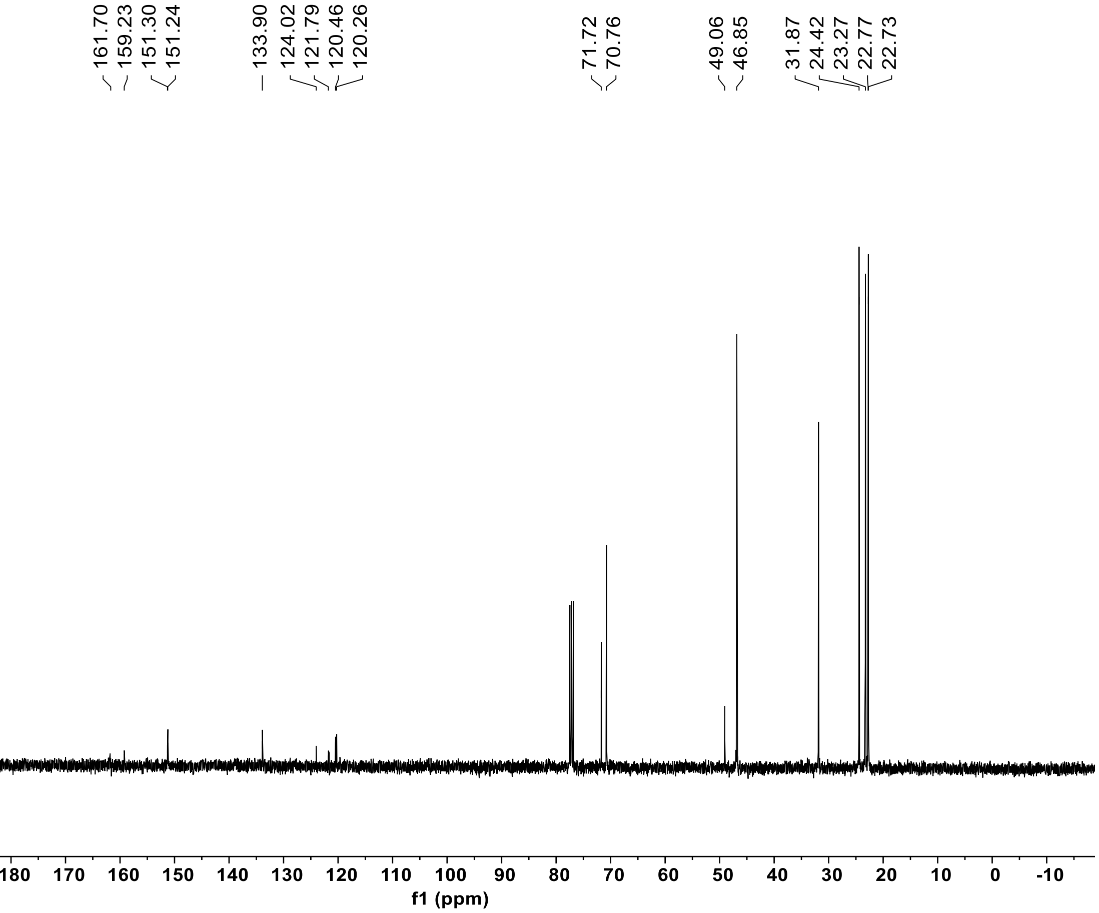

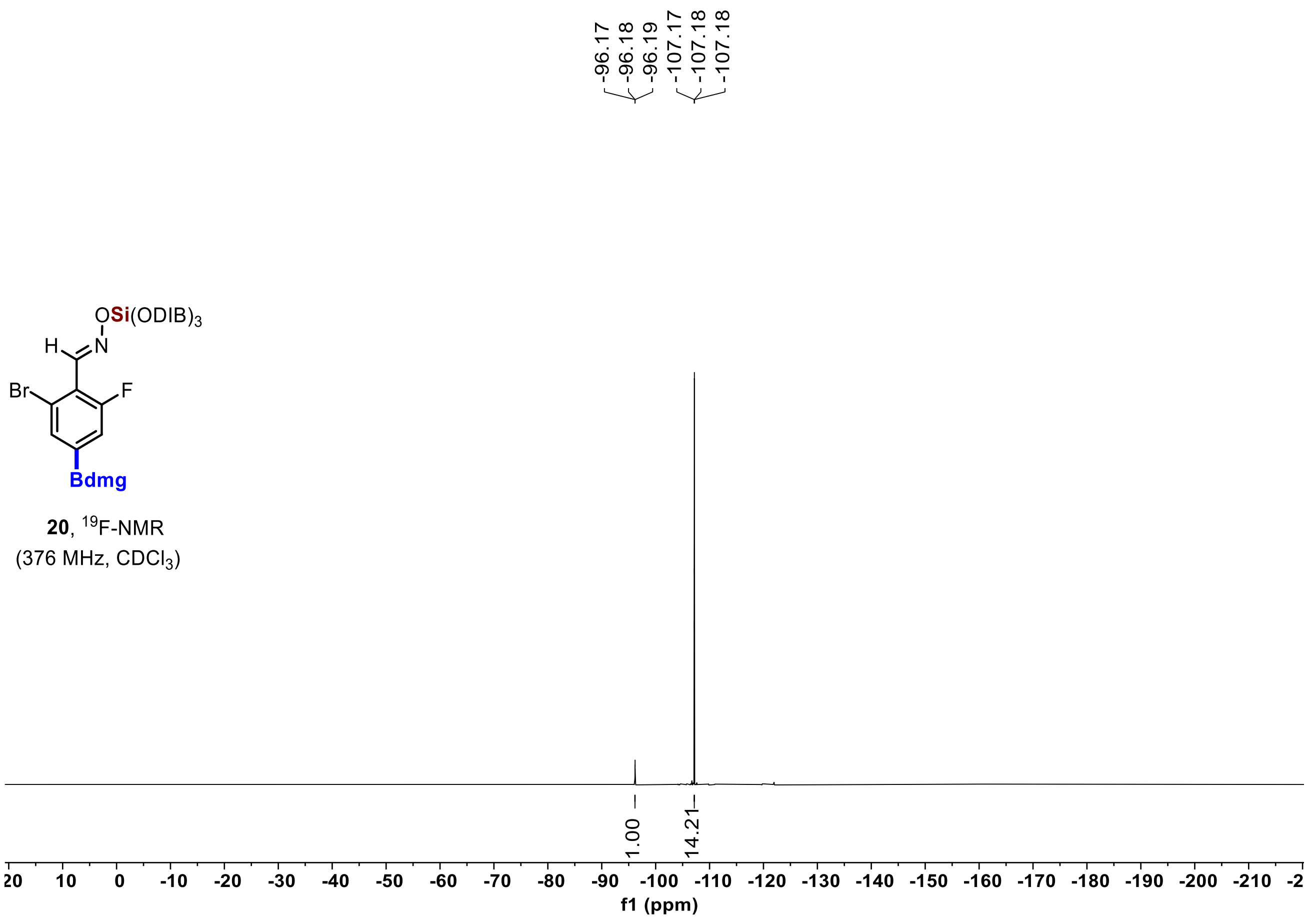

-96.17
-96.18
-96.19
-107.17
-107.18
-107.18
OSi(ODIB)3
H
N
Br
F
Bdmg
20, 19F-NMR
(376 MHz, CDCl3)
1.00
14.21
20 10 0 -10 -20 -30 -40 -50 -60 -70 -80 -90 -100 -110 -120 -130 -140 -150 -160 -170 -180 -190 -200 -210 -2
f1 (ppm)

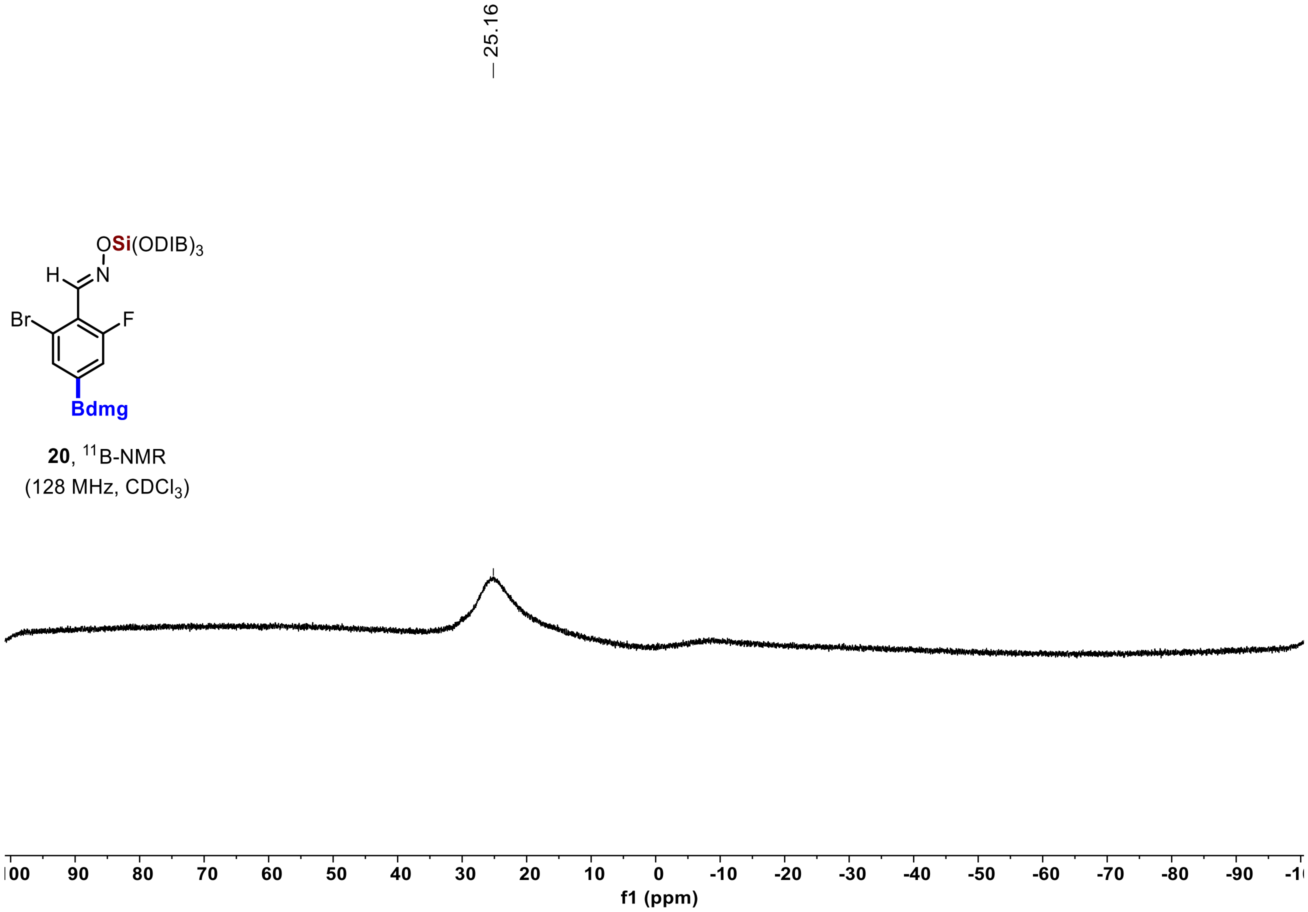

25.16
OSi(ODIB)3
H
N
Br
F
Bdmg
20, 11B-NMR
(128 MHz, CDCl3)
100 90 80 70 60 50 40 30 20 10 0 -10 -20 -30 -40 -50 -60 -70 -80 -90 -1
f1 (ppm)

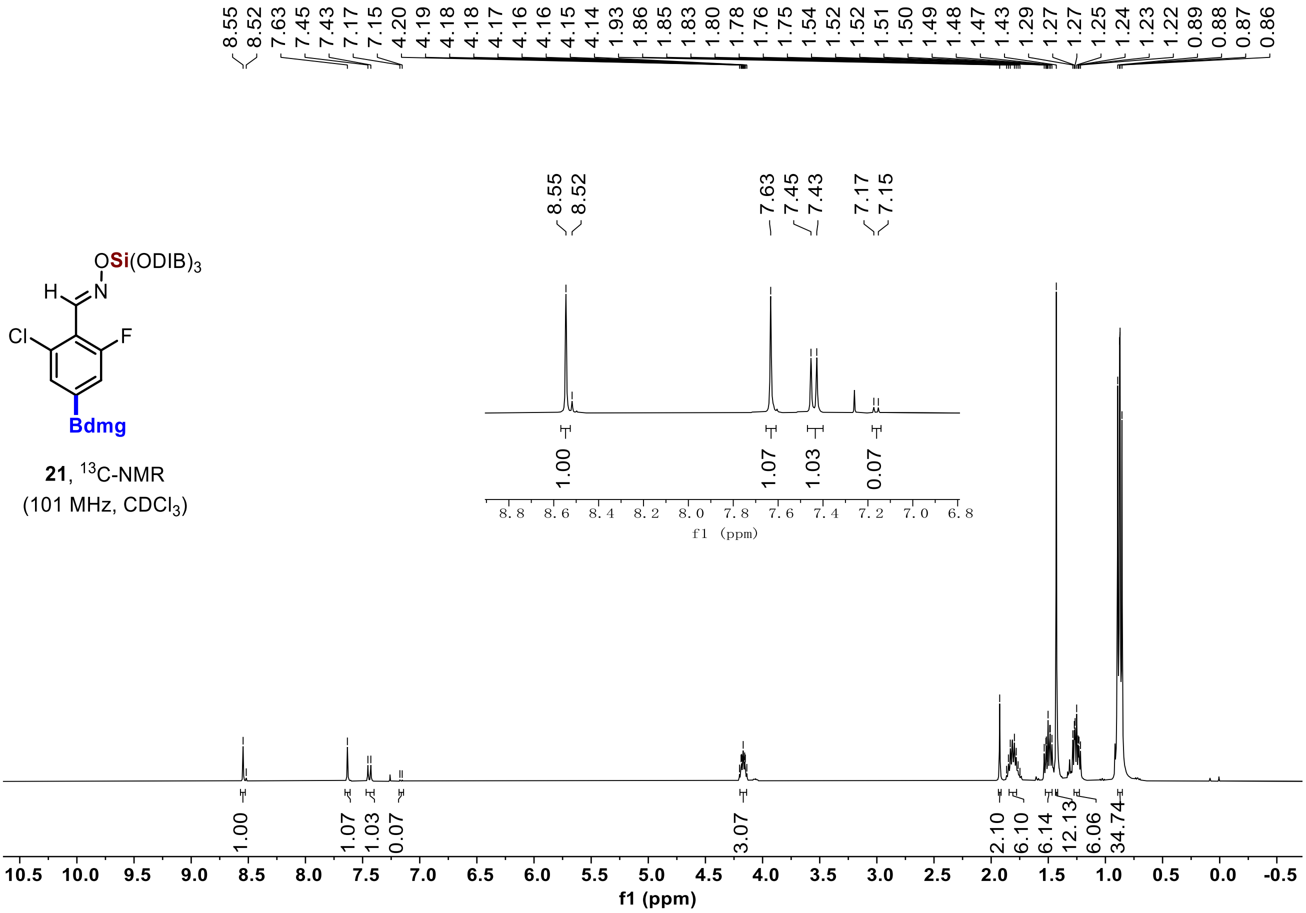
8.55 8.52 7.63 7.45 7.43 7.17 7.15 4.20 4.19 4.18 4.18 4.17 4.16 4.16 4.15 4.14 1.93 1.86 1.85 1.83 1.80 1.78 1.76 1.75 1.54 1.52 1.52 1.51 1.50 1.49 1.48 1.47 1.43 1.29 1.27 1.27 1.25 1.24 1.23 1.22 0.89 0.88 0.87 0.86
OSi(ODIB)3
H
N
Cl
F
Bdmg
21, 13C-NMR
(101 MHz, CDCl3)
8.55 8.52
7.63 7.45 7.43
7.17 7.15
1.00 1.07 1.03 0.07
f1 (ppm)
1.00 1.07 1.03 0.07 3.07 2.10 6.10 6.14 12.13 6.06 34.74
f1 (ppm)

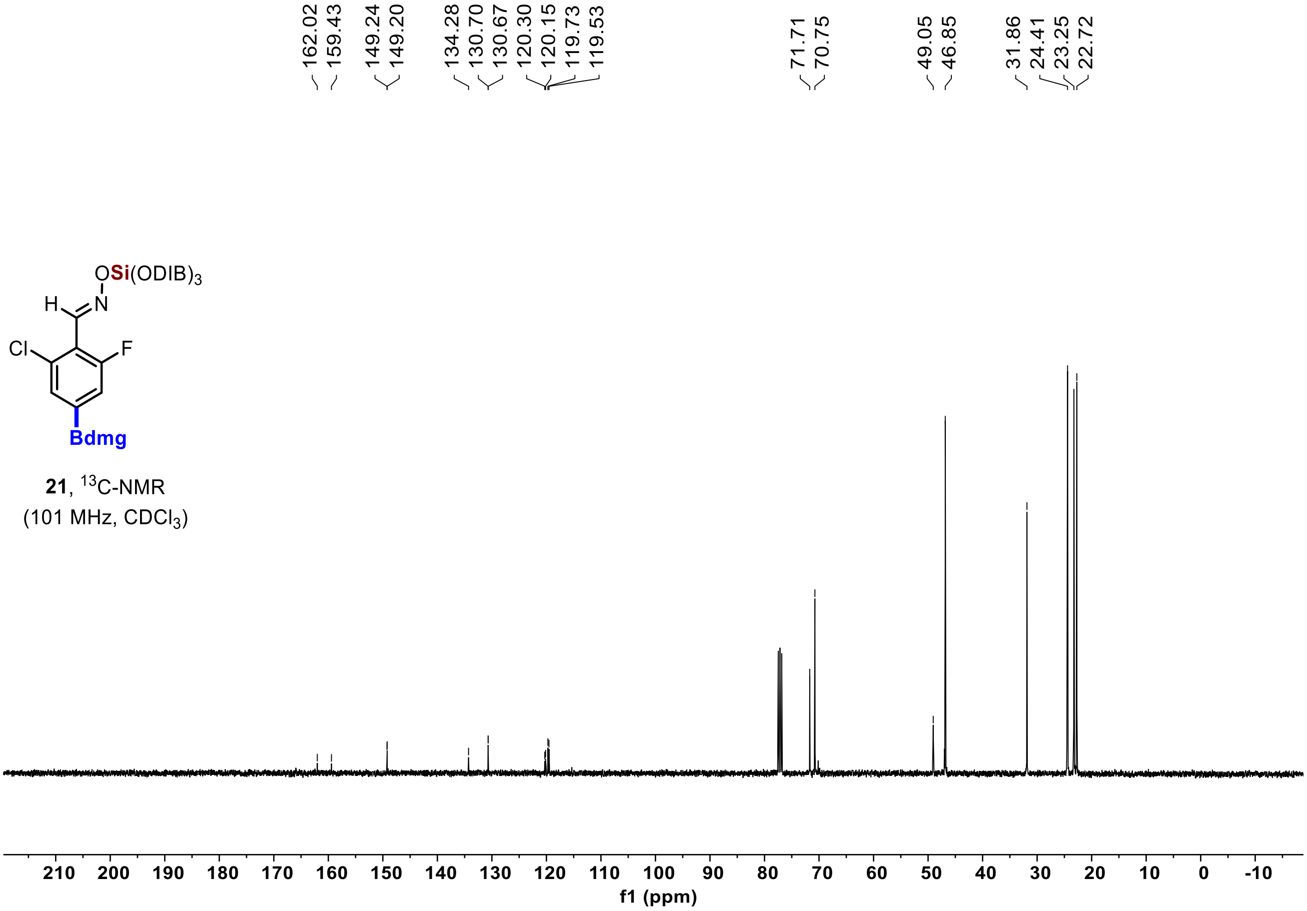
162.02 159.43 149.24 149.20 134.28 130.70 130.67 120.30 120.15 119.73 119.53 71.71 70.75 49.05 46.85 31.86 24.41 23.25 22.72
OSi(ODIB)3
H
N
Cl
F
Bdmg
21, 13C-NMR
(101 MHz, CDCl3)
f1 (ppm)

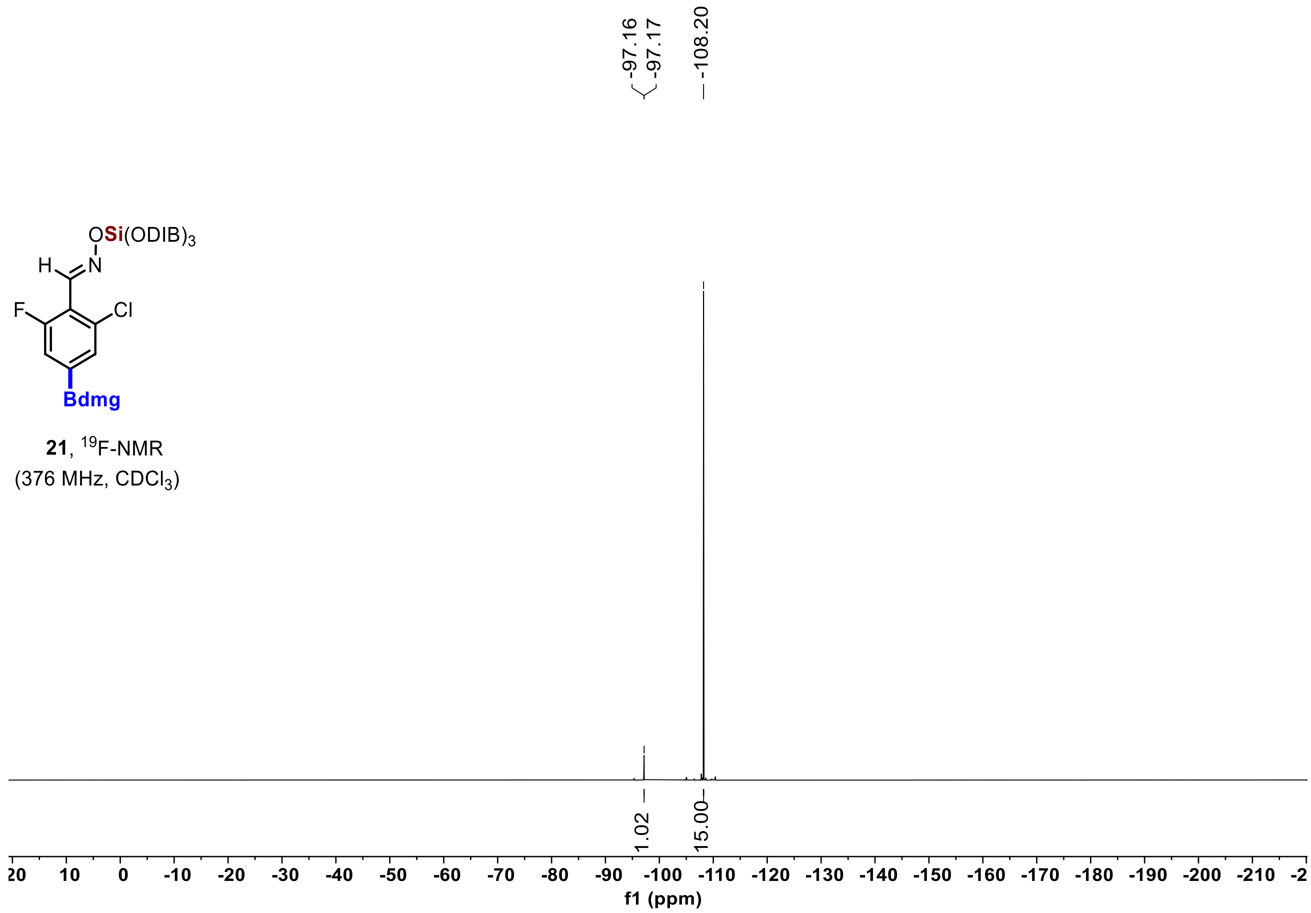
-97.16
-97.17
-108.20
OSi(ODIB)3
H
N
F
Cl
Bdmg
21, 19F-NMR
(376 MHz, CDCl3)
1.02
15.00
f1 (ppm)

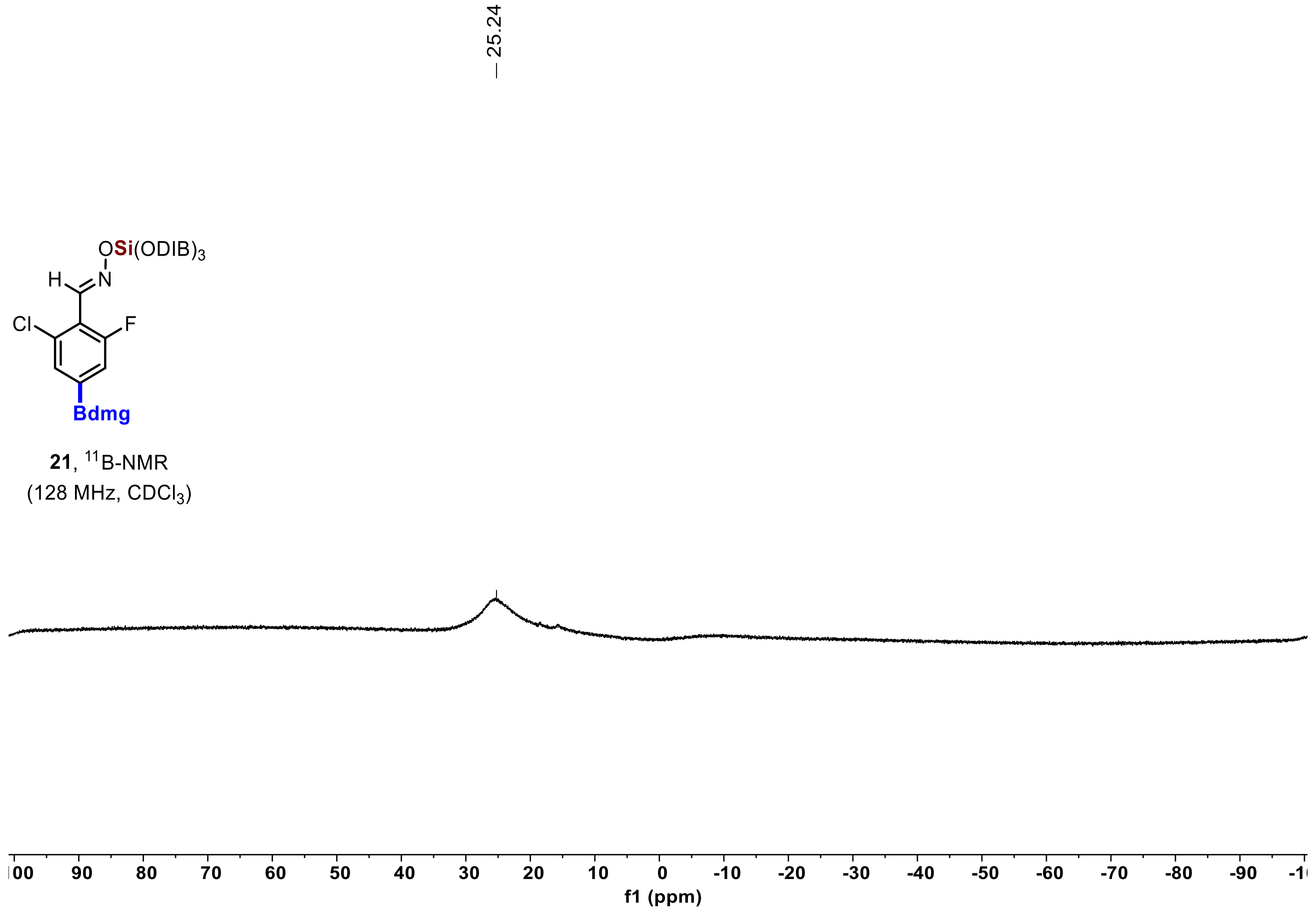
25.24
OSi(ODIB)3
H
N
Cl
F
Bdmg
21, 11B-NMR
(128 MHz, CDCl3)
f1 (ppm)

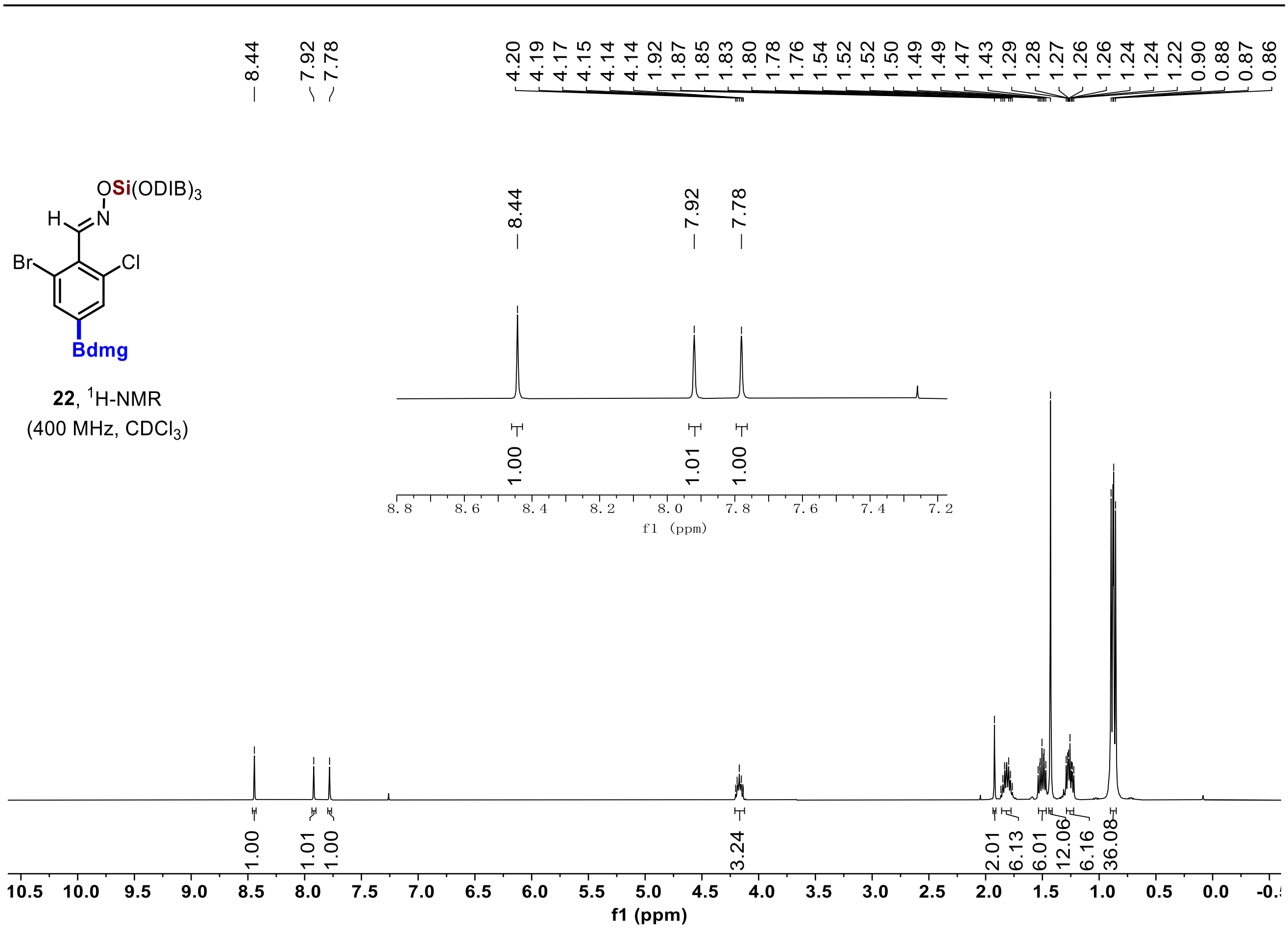

OSi(ODIB)3
H
N
Br
Cl
Bdmg
22, 1H-NMR
(400 MHz, CDCl3)
8.44 7.92 7.78
4.20 4.19 4.17 4.15 4.14 4.14 1.92 1.87 1.85 1.83 1.80 1.78 1.76 1.54 1.52 1.52 1.50 1.49 1.49 1.47 1.43 1.29 1.28 1.27 1.26 1.26 1.24 1.24 1.22 0.90 0.88 0.87 0.86
1.00 1.01 1.00
f1 (ppm)
1.00 1.01 1.00 3.24 2.01 6.13 6.01 12.06 6.16 36.08
f1 (ppm)

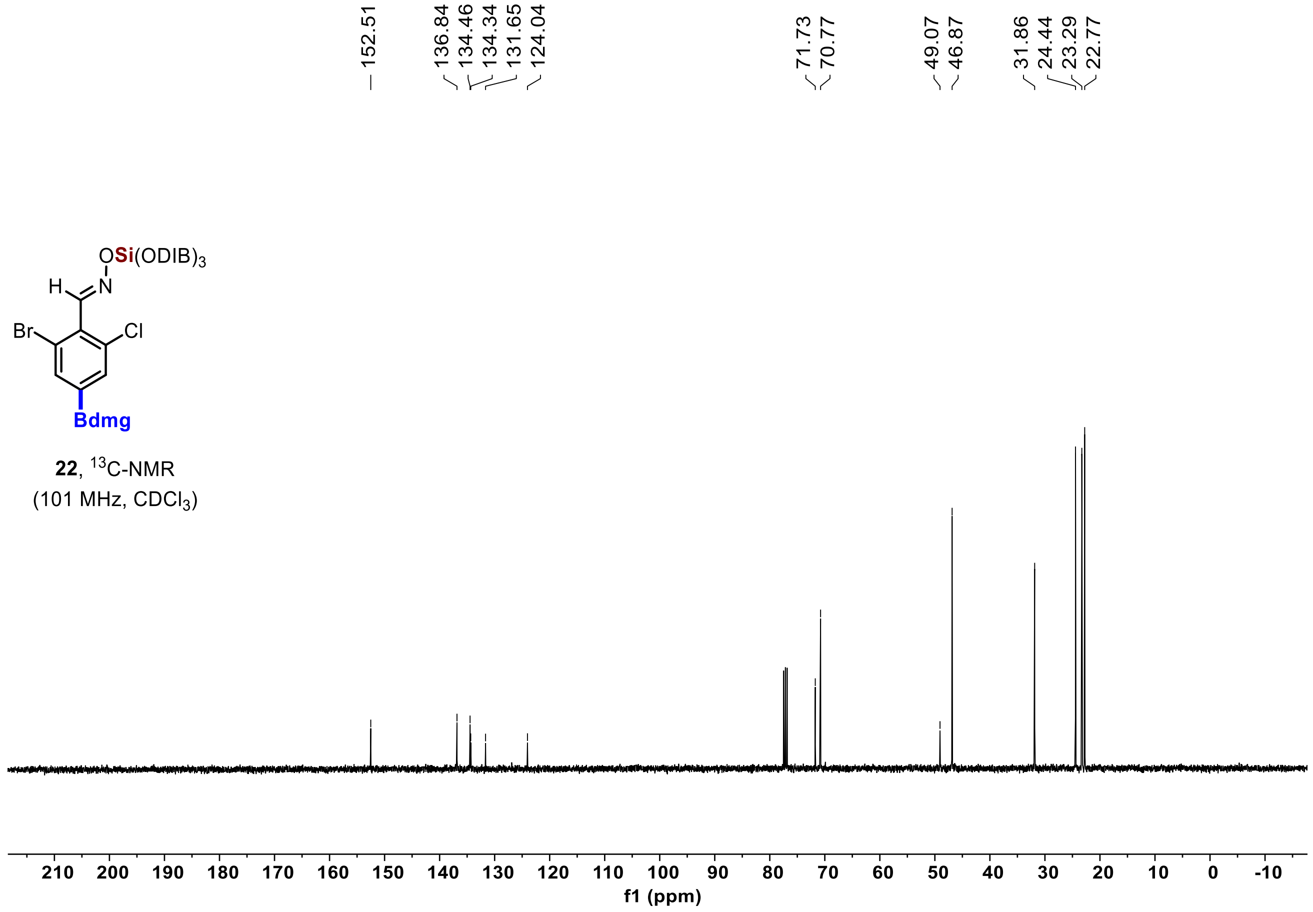

OSi(ODIB)3
H
N
Br
Cl
Bdmg
22, 13C-NMR
(101 MHz, CDCl3)
152.51
136.84 134.46 134.34 131.65 124.04
71.73 70.77
49.07 46.87
31.86 24.44 23.29 22.77
f1 (ppm)

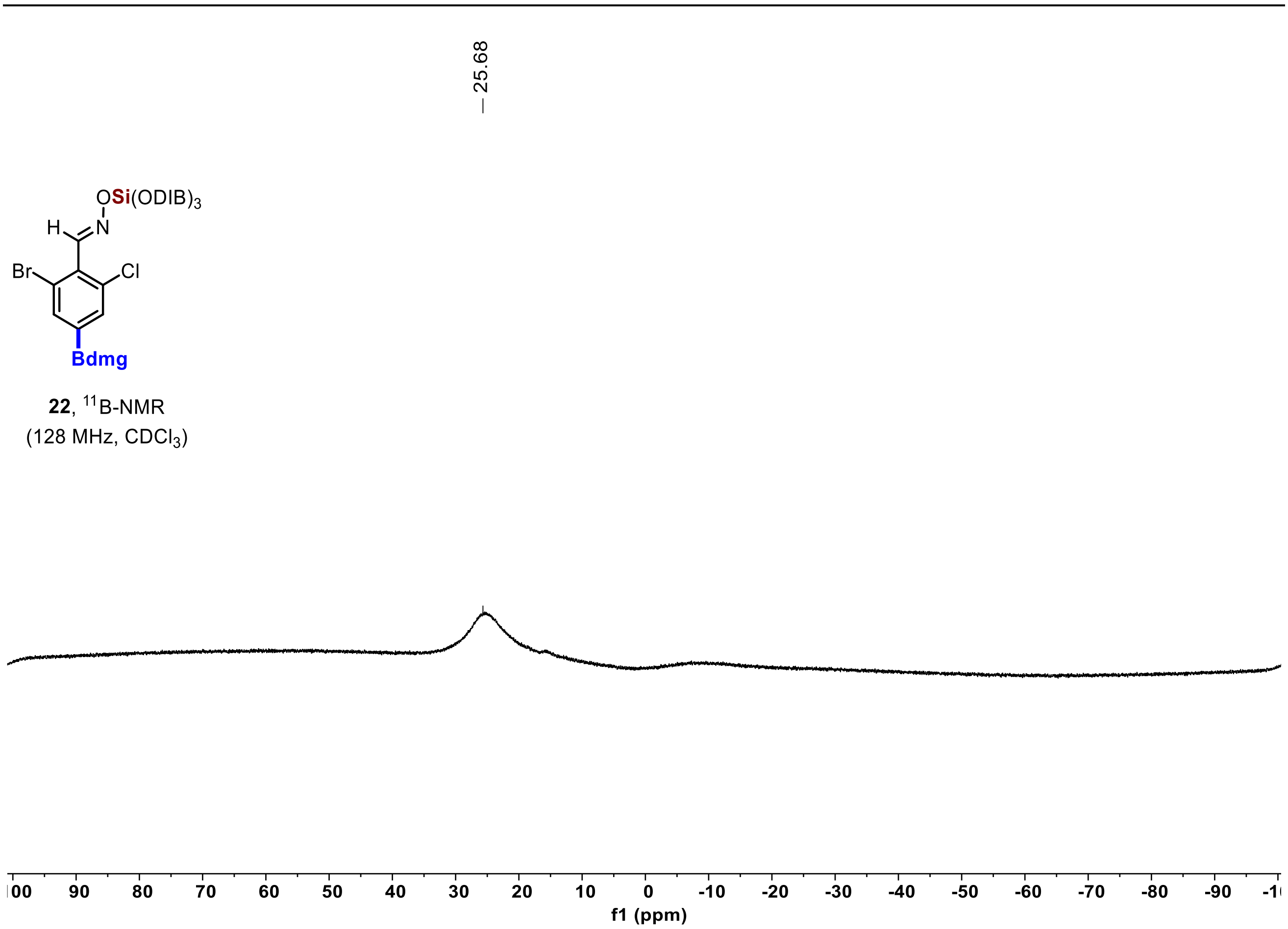
25.68
OSi(ODIB)3
H
N
Br
Cl
Bdmg
22, 11B-NMR
(128 MHz, CDCl3)
90 80 70 60 50 40 30 20 10 0 -10 -20 -30 -40 -50 -60 -70 -80 -90
f1 (ppm)

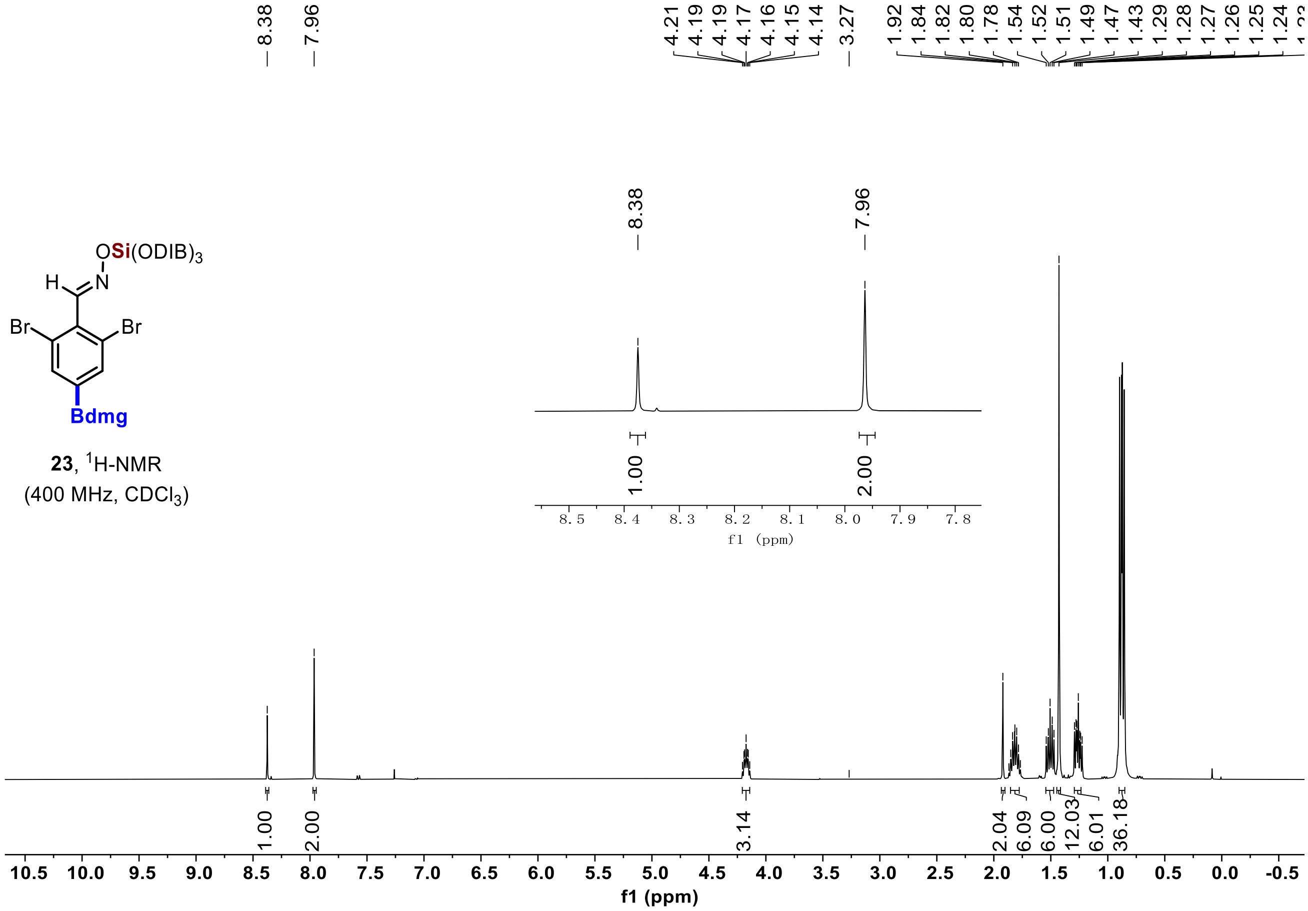
8.38
7.96
4.21
4.19
4.19
4.17
4.16
4.15
4.14
3.27
1.92
1.84
1.82
1.80
1.78
1.54
1.52
1.51
1.49
1.47
1.43
1.29
1.28
1.27
1.26
1.25
1.24
OSi(ODIB)3
H
N
Br
Br
Bdmg
23, 1H-NMR
(400 MHz, CDCl3)
8.38
7.96
1.00
2.00
8.5 8.4 8.3 8.2 8.1 8.0 7.9 7.8
f1 (ppm)
1.00
2.00
3.14
2.04
6.09
6.00
12.03
6.01
36.18
10.5 10.0 9.5 9.0 8.5 8.0 7.5 7.0 6.5 6.0 5.5 5.0 4.5 4.0 3.5 3.0 2.5 2.0 1.5 1.0 0.5 0.0 -0.5
f1 (ppm)

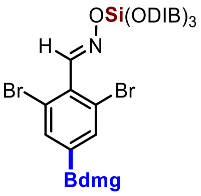

23, ^{13}C-NMR
(101 MHz, $CDCl_3$)

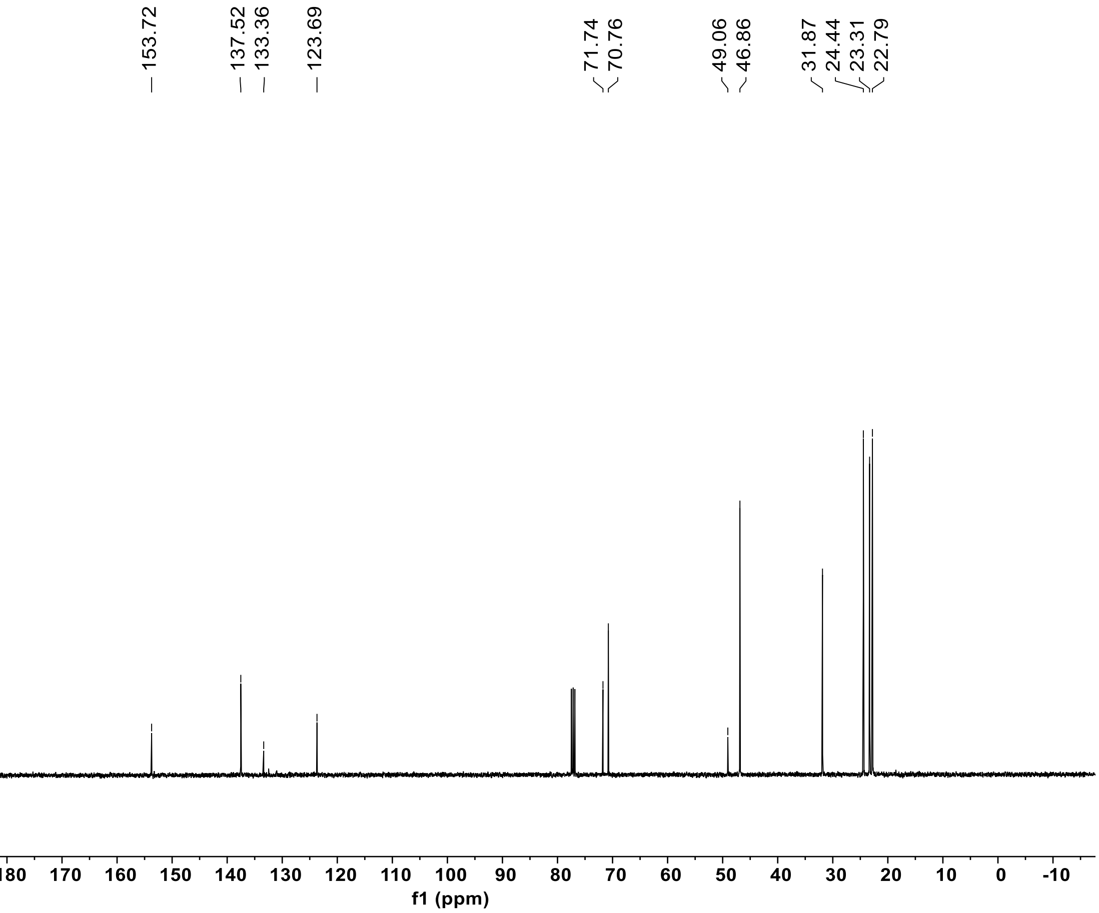

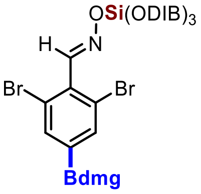

23, ^{11}B-NMR
(128 MHz, $CDCl_3$)

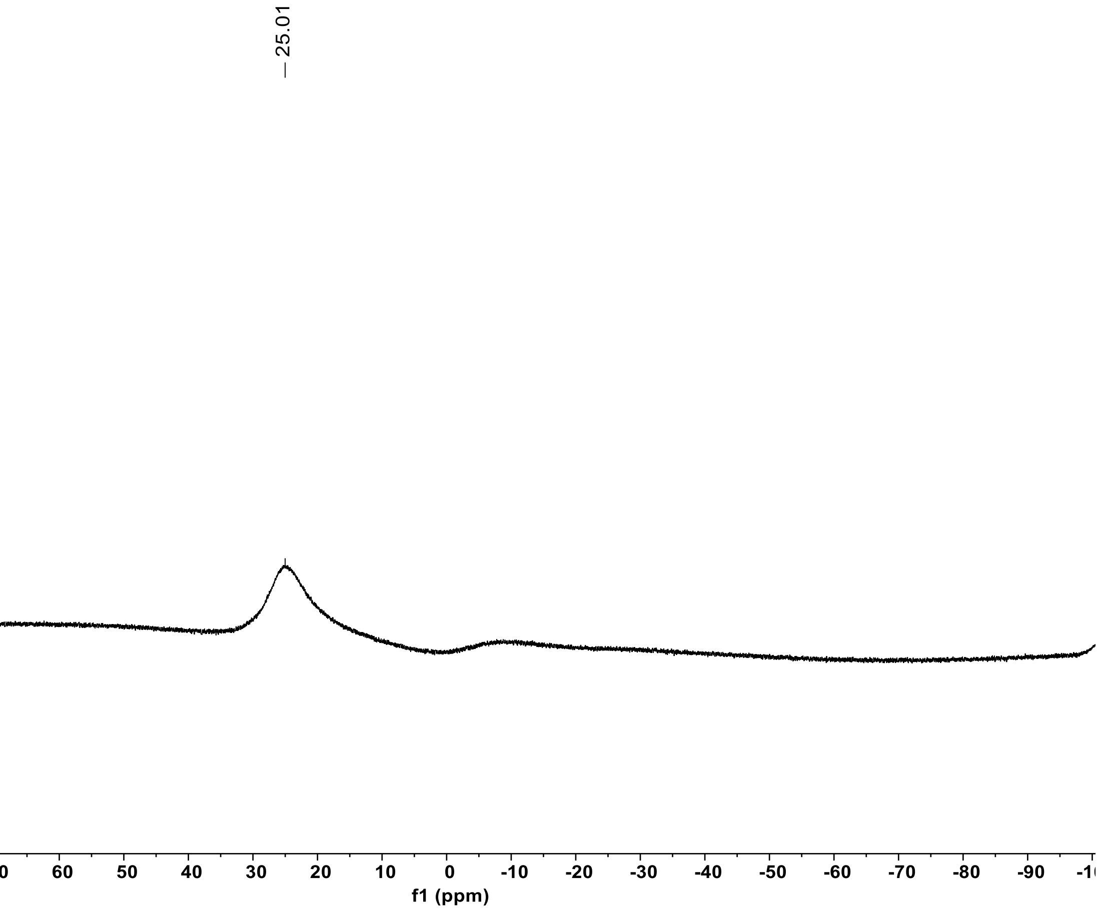

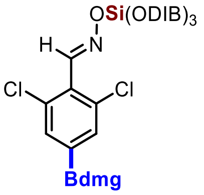

24, ¹H-NMR
(400 MHz, $CDCl_3$)

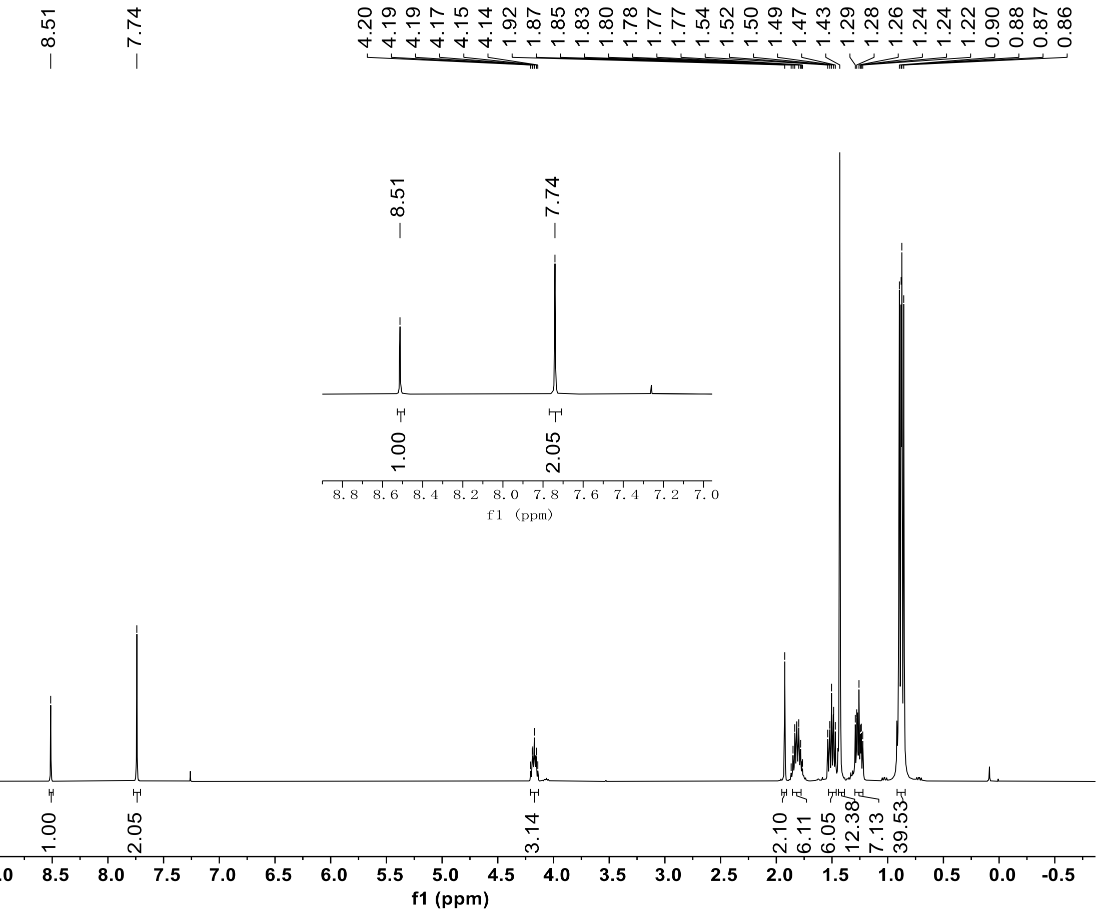

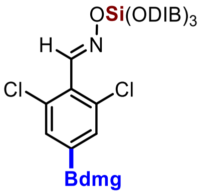

24, ¹³C-NMR
(101 MHz, $CDCl_3$)

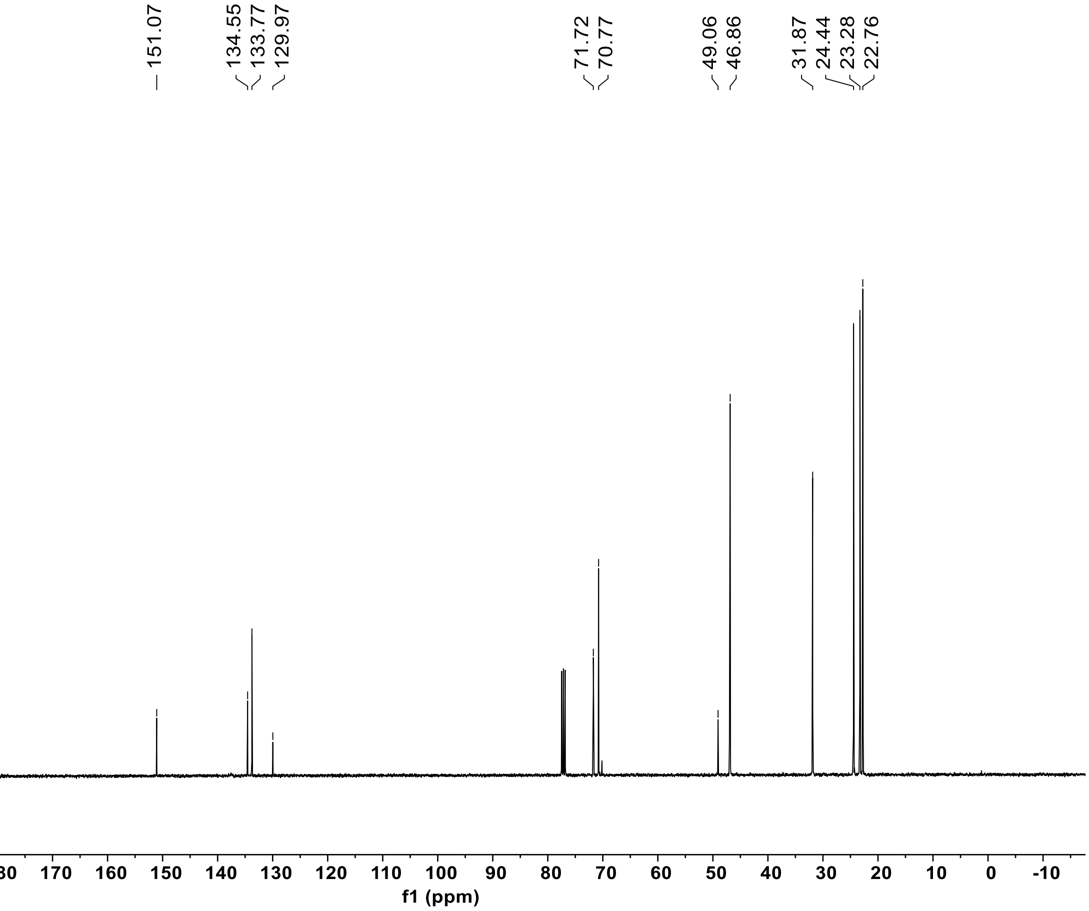

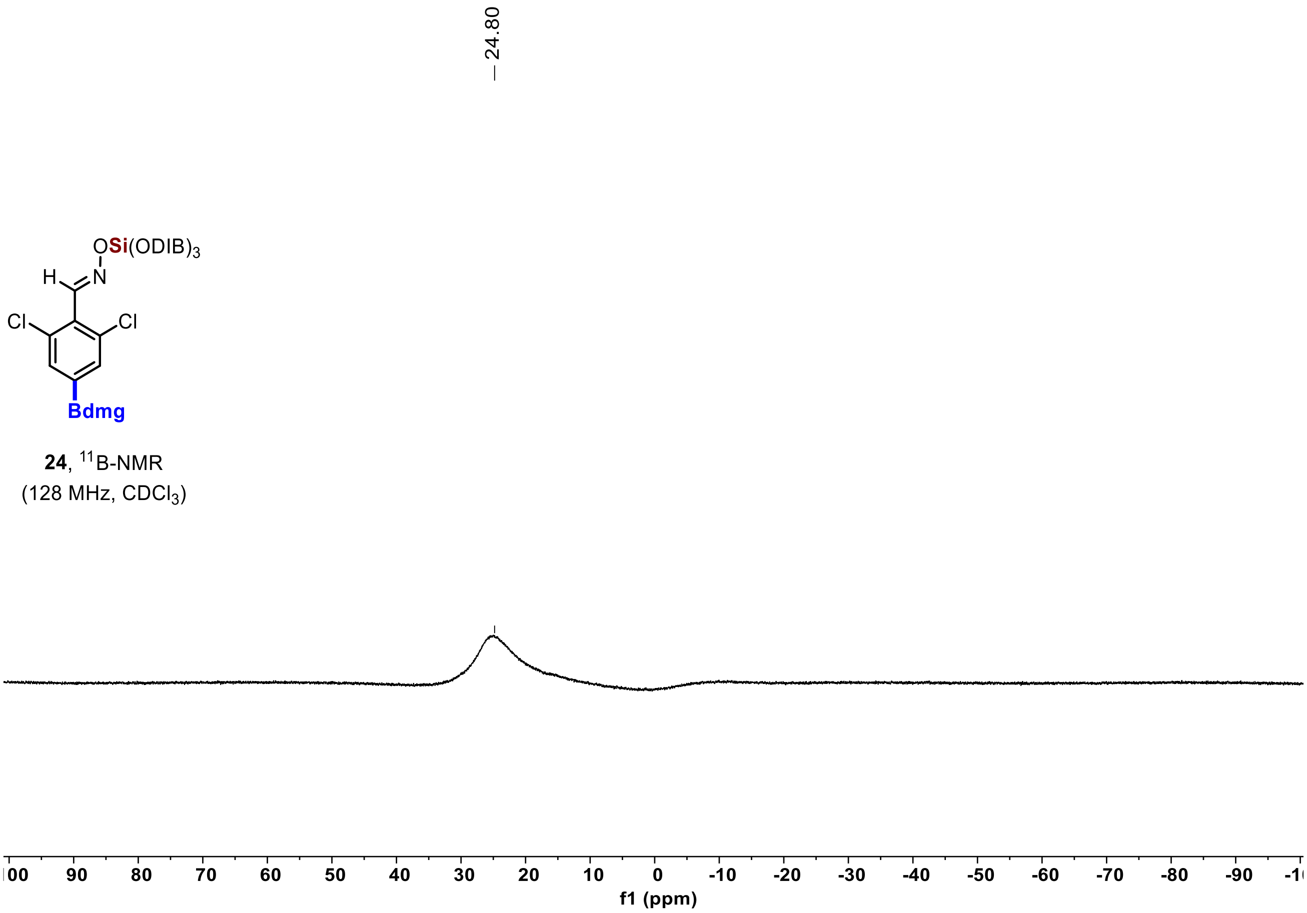
OSi(ODIB)3
H
N
Cl
Cl
Bdmg
24, 11B-NMR
(128 MHz, CDCl3)
24.80
f1 (ppm)

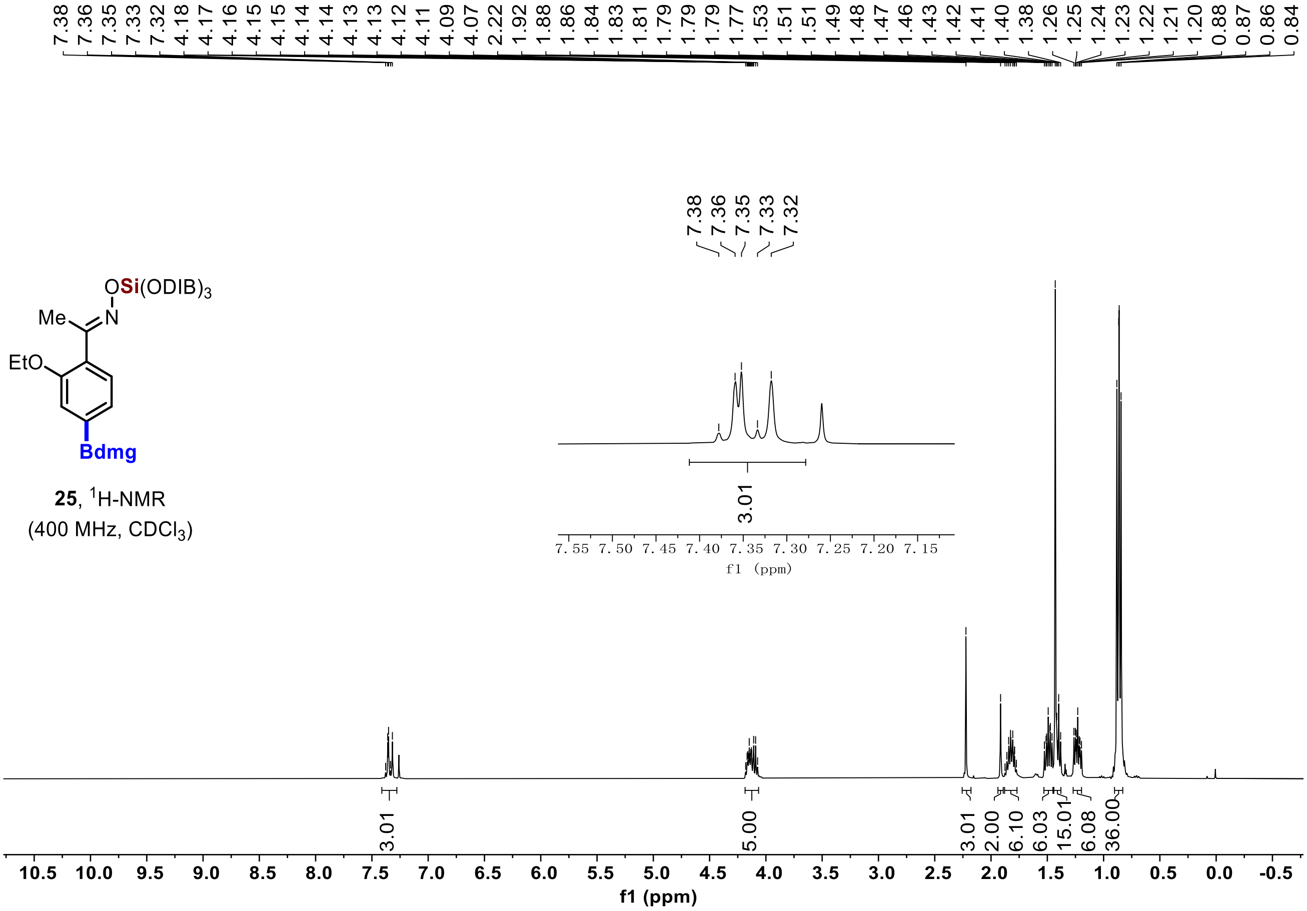
OSi(ODIB)3
Me
N
EtO
Bdmg
25, 1H-NMR
(400 MHz, CDCl3)
7.38 7.36 7.35 7.33 7.32 4.18 4.17 4.16 4.15 4.15 4.14 4.14 4.13 4.13 4.12 4.11 4.09 4.07 2.22 1.92 1.88 1.86 1.84 1.83 1.81 1.79 1.79 1.79 1.77 1.53 1.51 1.51 1.49 1.48 1.47 1.46 1.43 1.42 1.41 1.40 1.38 1.26 1.25 1.24 1.23 1.22 1.21 1.20 0.88 0.87 0.86 0.84
3.01
5.00
3.01 2.00 6.10 6.03 15.01 6.08 36.00
f1 (ppm)

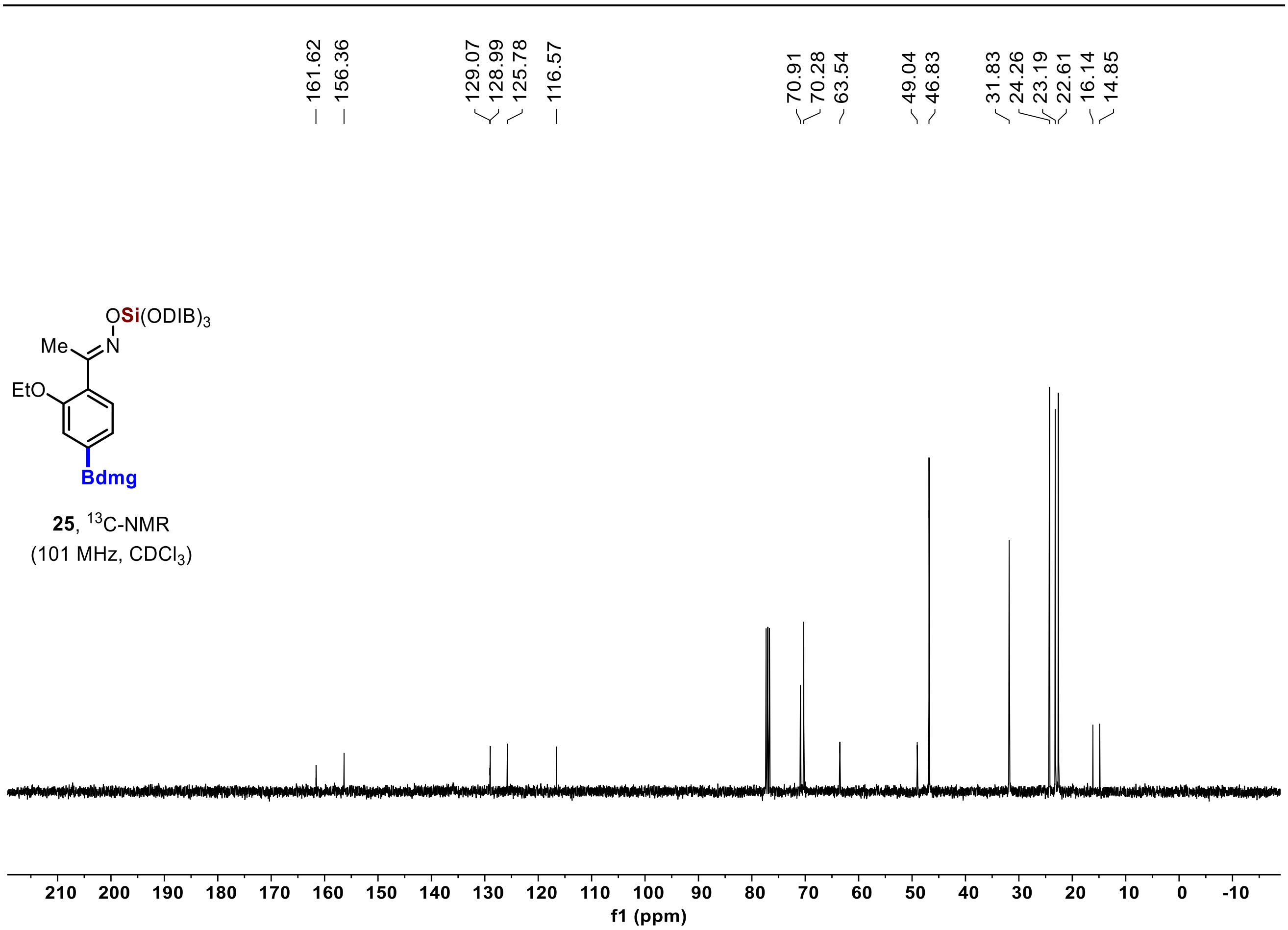
161.62
156.36
129.07
128.99
125.78
116.57
70.91
70.28
63.54
49.04
46.83
31.83
24.26
23.19
22.61
16.14
14.85
OSi(ODIB)3
Me
N
EtO
Bdmg
25, 13C-NMR
(101 MHz, CDCl3)
210 200 190 180 170 160 150 140 130 120 110 100 90 80 70 60 50 40 30 20 10 0 -10
f1 (ppm)

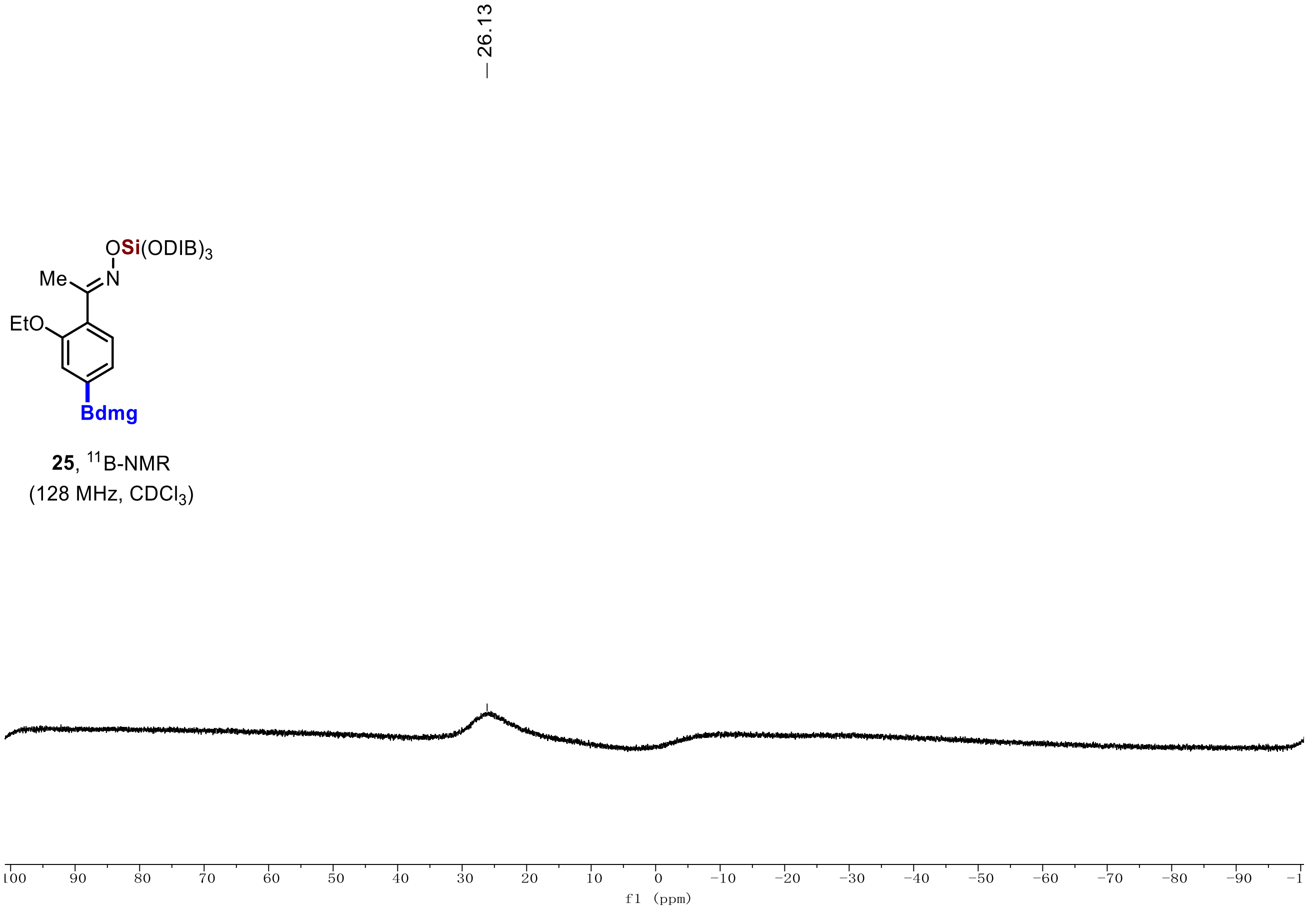
26.13
OSi(ODIB)3
Me
N
EtO
Bdmg
25, 11B-NMR
(128 MHz, CDCl3)
100 90 80 70 60 50 40 30 20 10 0 -10 -20 -30 -40 -50 -60 -70 -80 -90
f1 (ppm)

NOESY spectrum of 25 (400 MHz, $CDCl_3$)

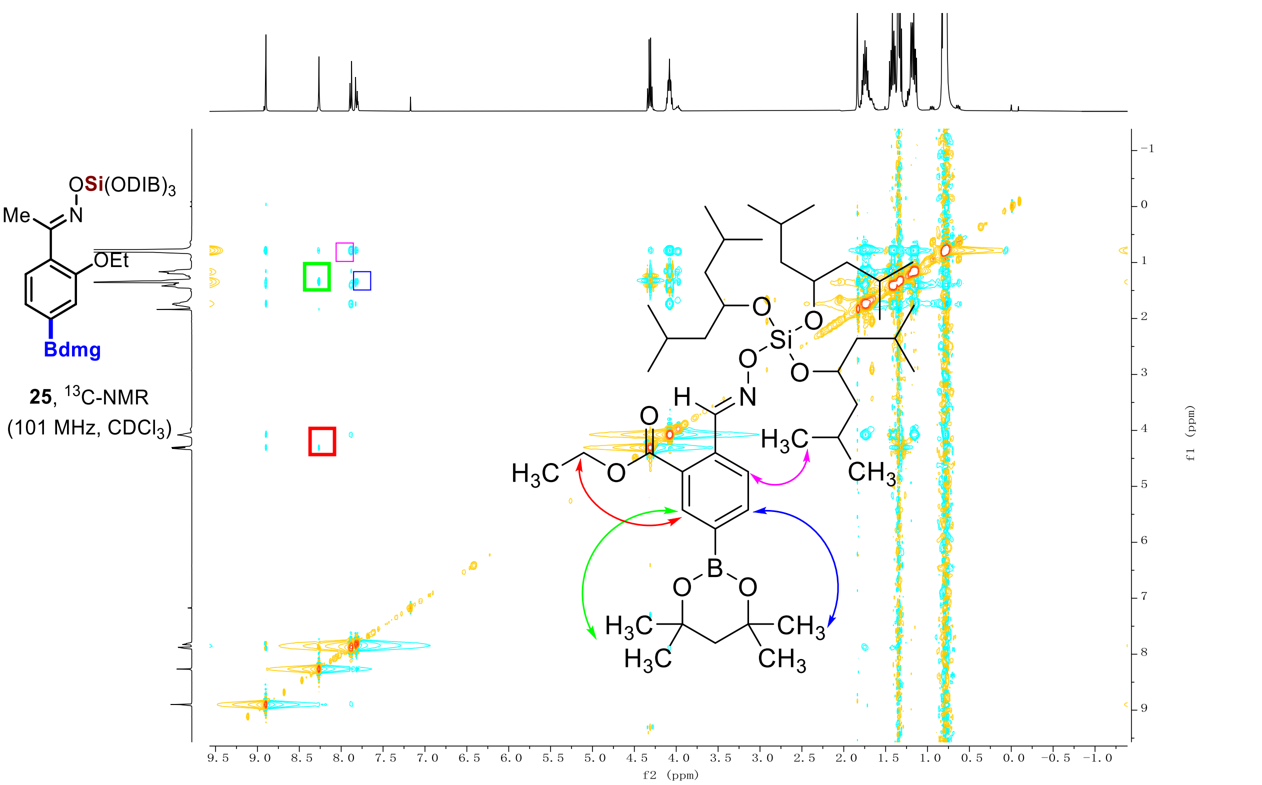

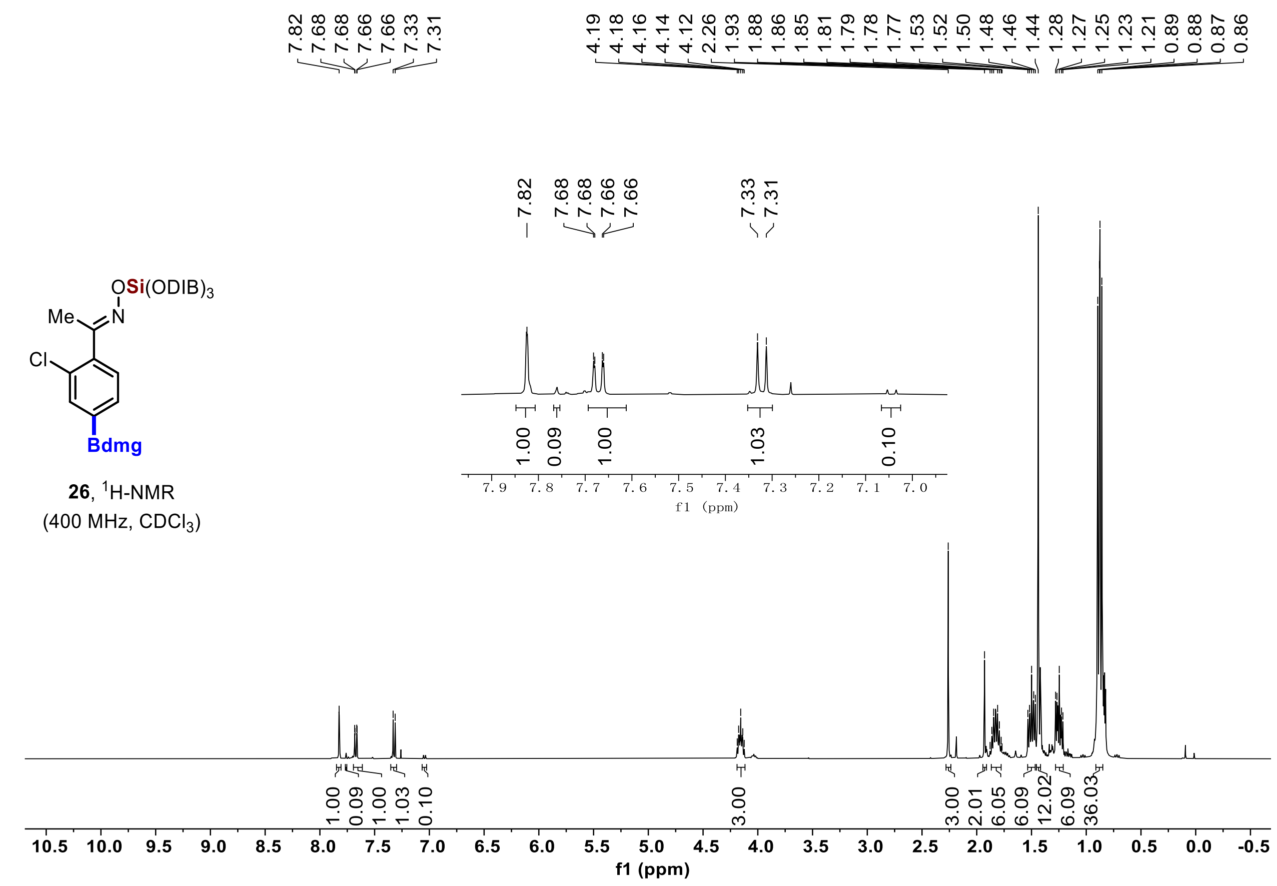

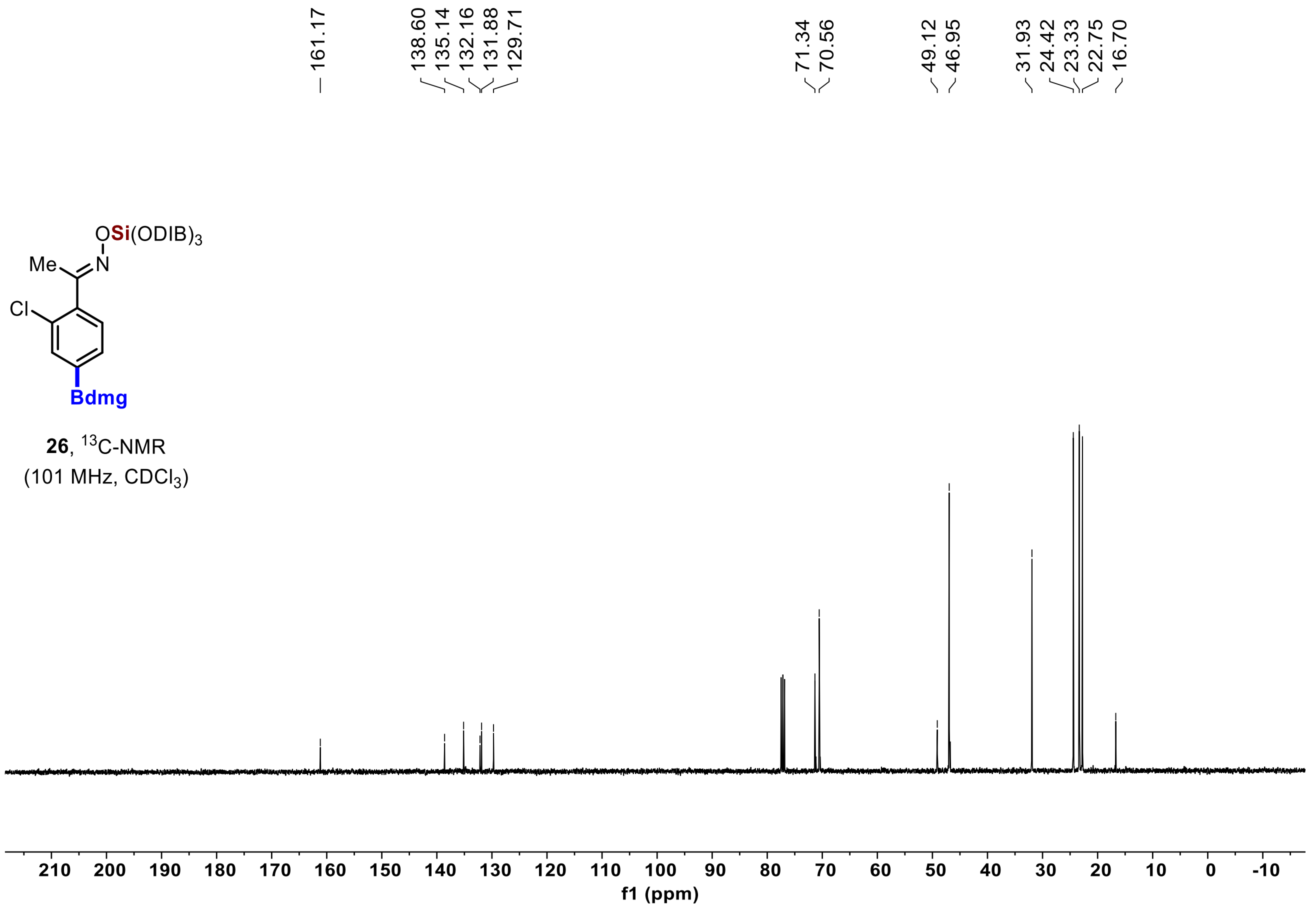

161.17
138.60
135.14
132.16
131.88
129.71
71.34
70.56
49.12
46.95
31.93
24.42
23.33
22.75
16.70
OSi(ODIB)3
Me
N
Cl
Bdmg
26, 13C-NMR
(101 MHz, CDCl3)
210 200 190 180 170 160 150 140 130 120 110 100 90 80 70 60 50 40 30 20 10 0 -10
f1 (ppm)

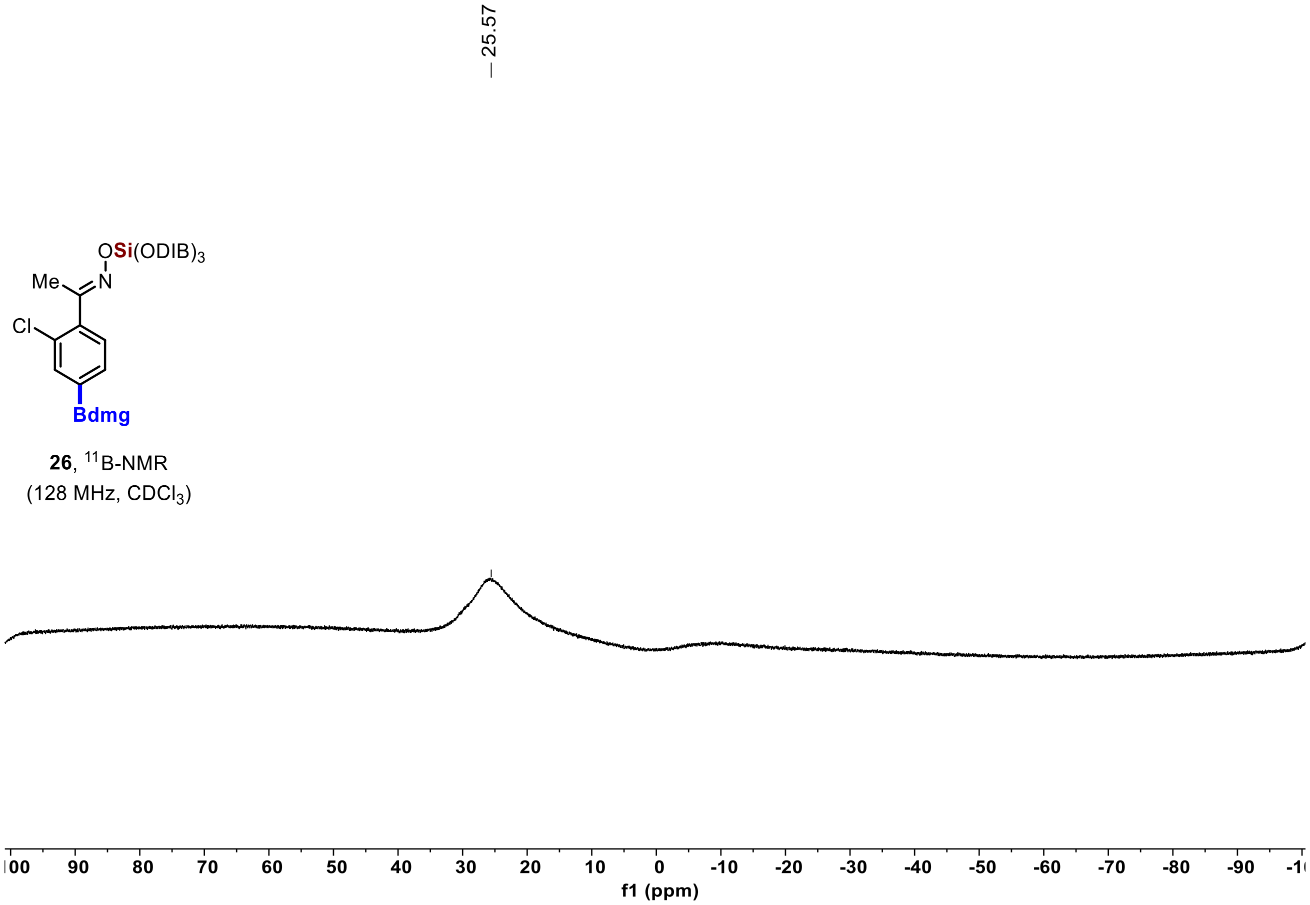

25.57
OSi(ODIB)3
Me
N
Cl
Bdmg
26, 11B-NMR
(128 MHz, CDCl3)
90 80 70 60 50 40 30 20 10 0 -10 -20 -30 -40 -50 -60 -70 -80 -90
f1 (ppm)

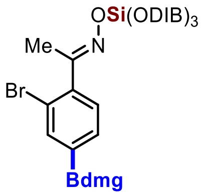

27, ^{1}H-NMR
(400 MHz, $CDCl_3$)

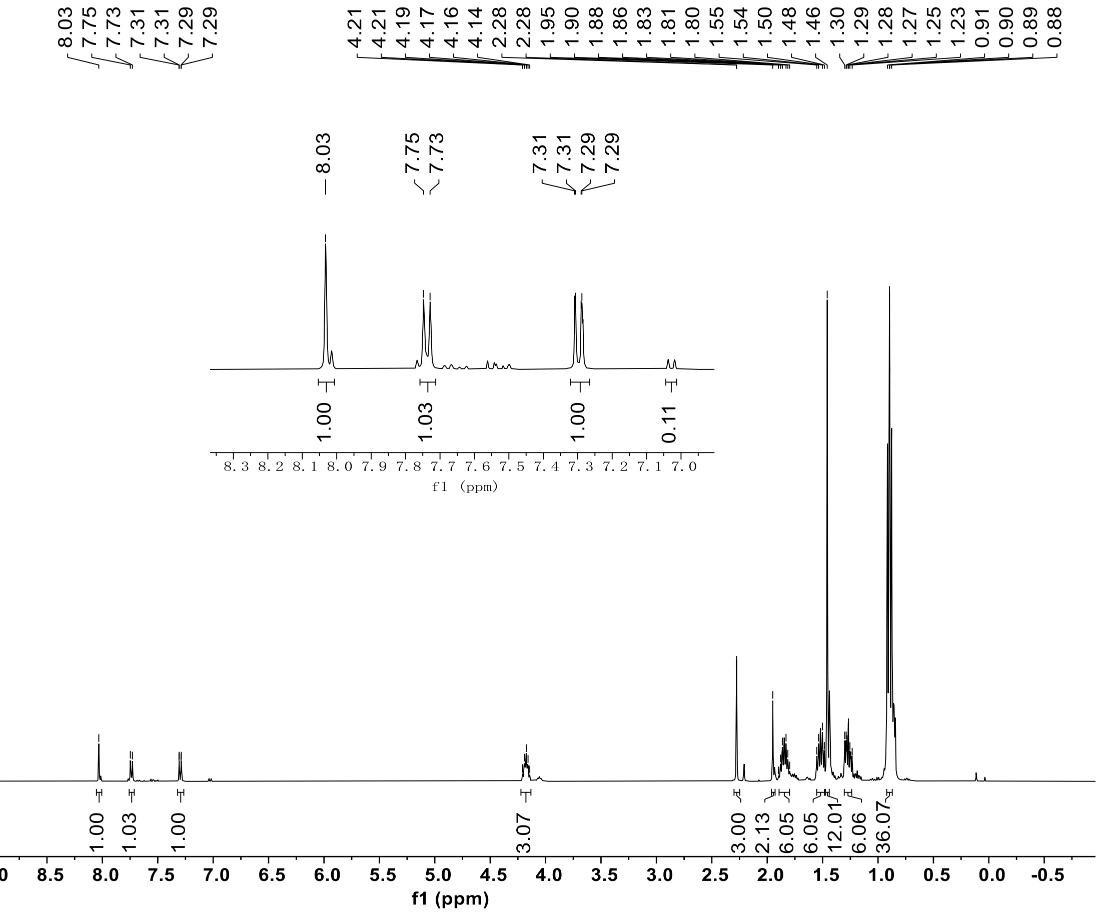

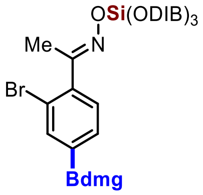

27, ^{13}C-NMR
(101 MHz, $CDCl_3$)

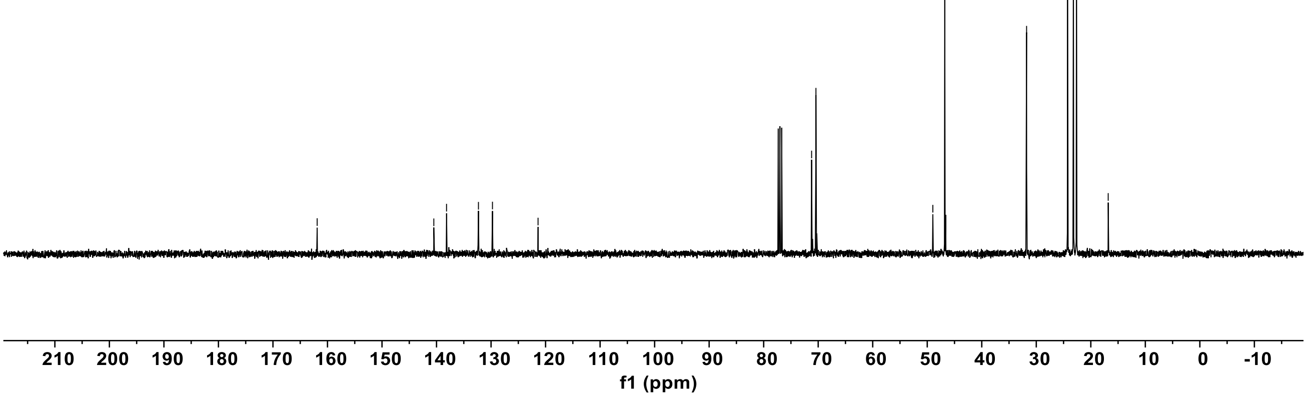

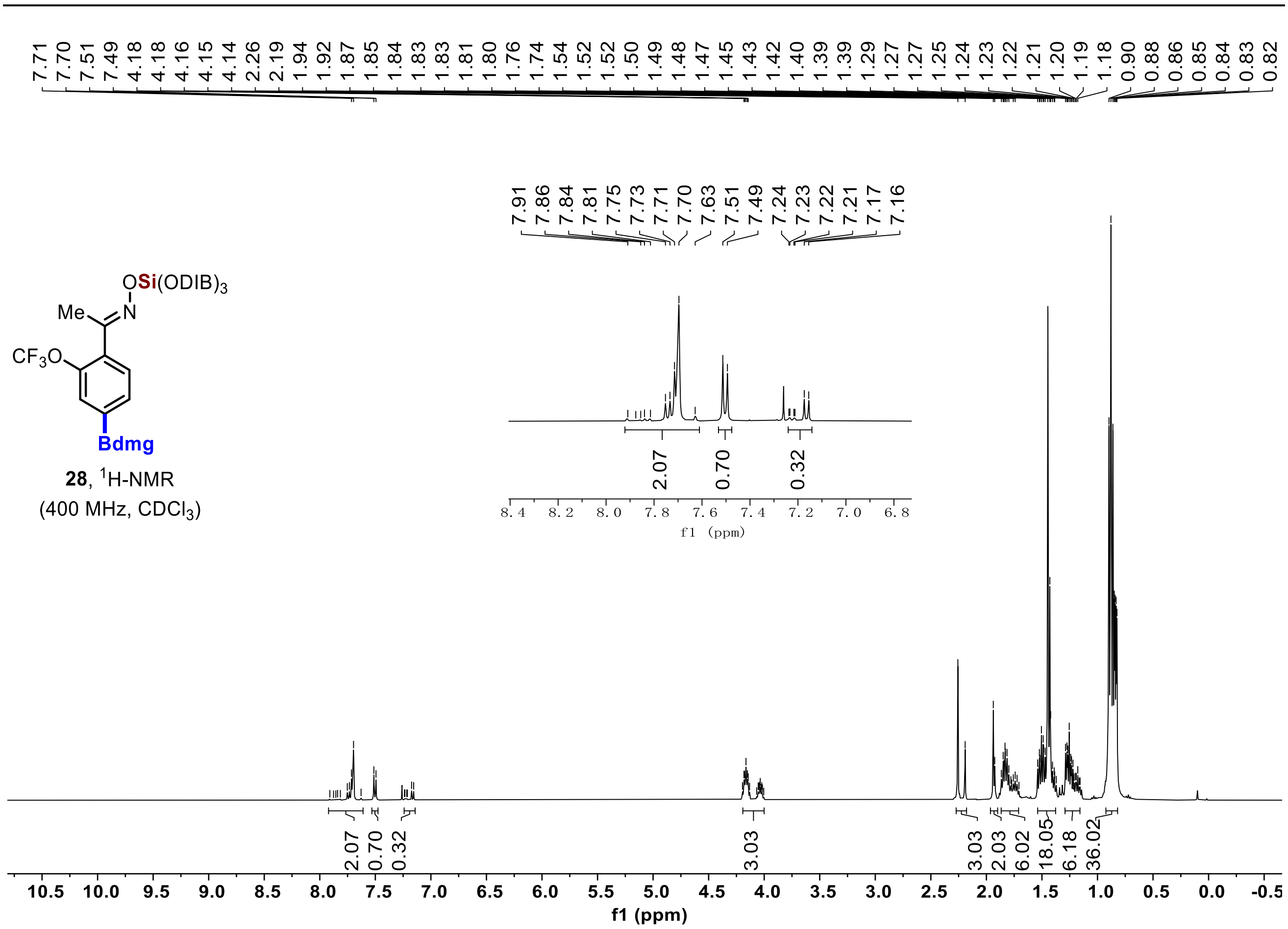

28, ^{1}H-NMR
(400 MHz, $CDCl_3$)

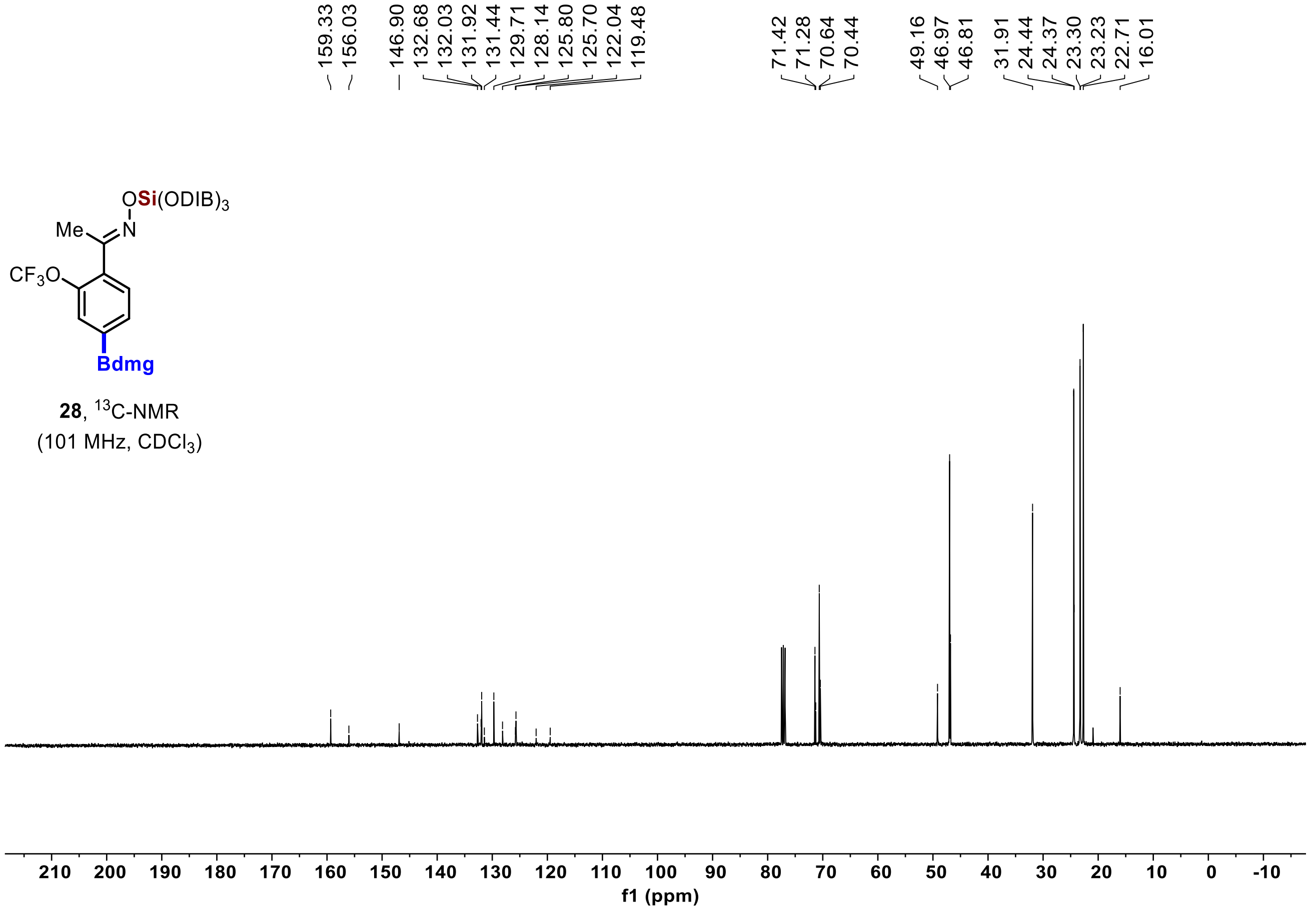

28, ^{13}C-NMR
(101 MHz, $CDCl_3$)

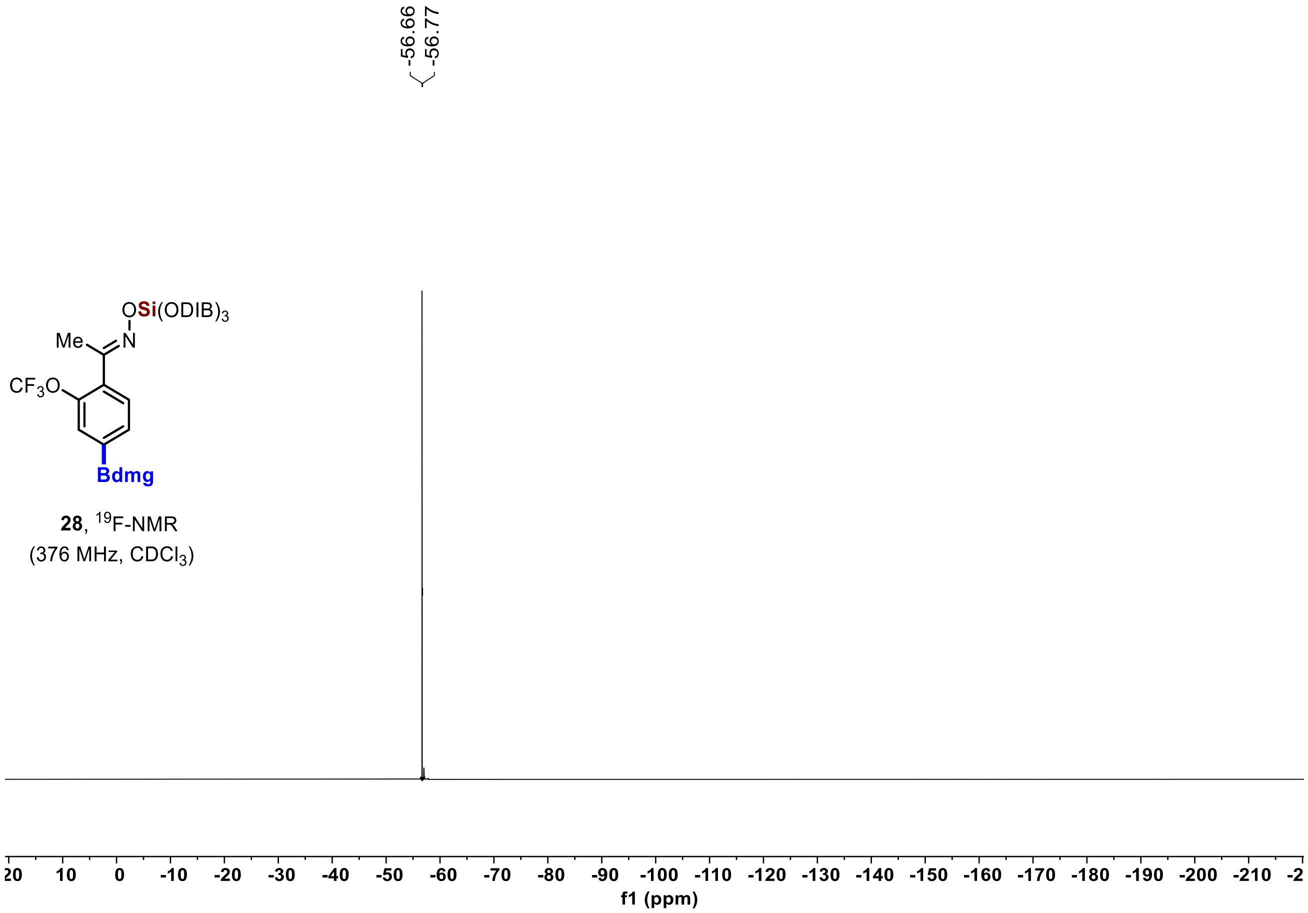
-56.66
-56.77
OSi(ODIB)3
Me
N
CF3O
Bdmg
28, 19F-NMR
(376 MHz, CDCl3)
f1 (ppm)

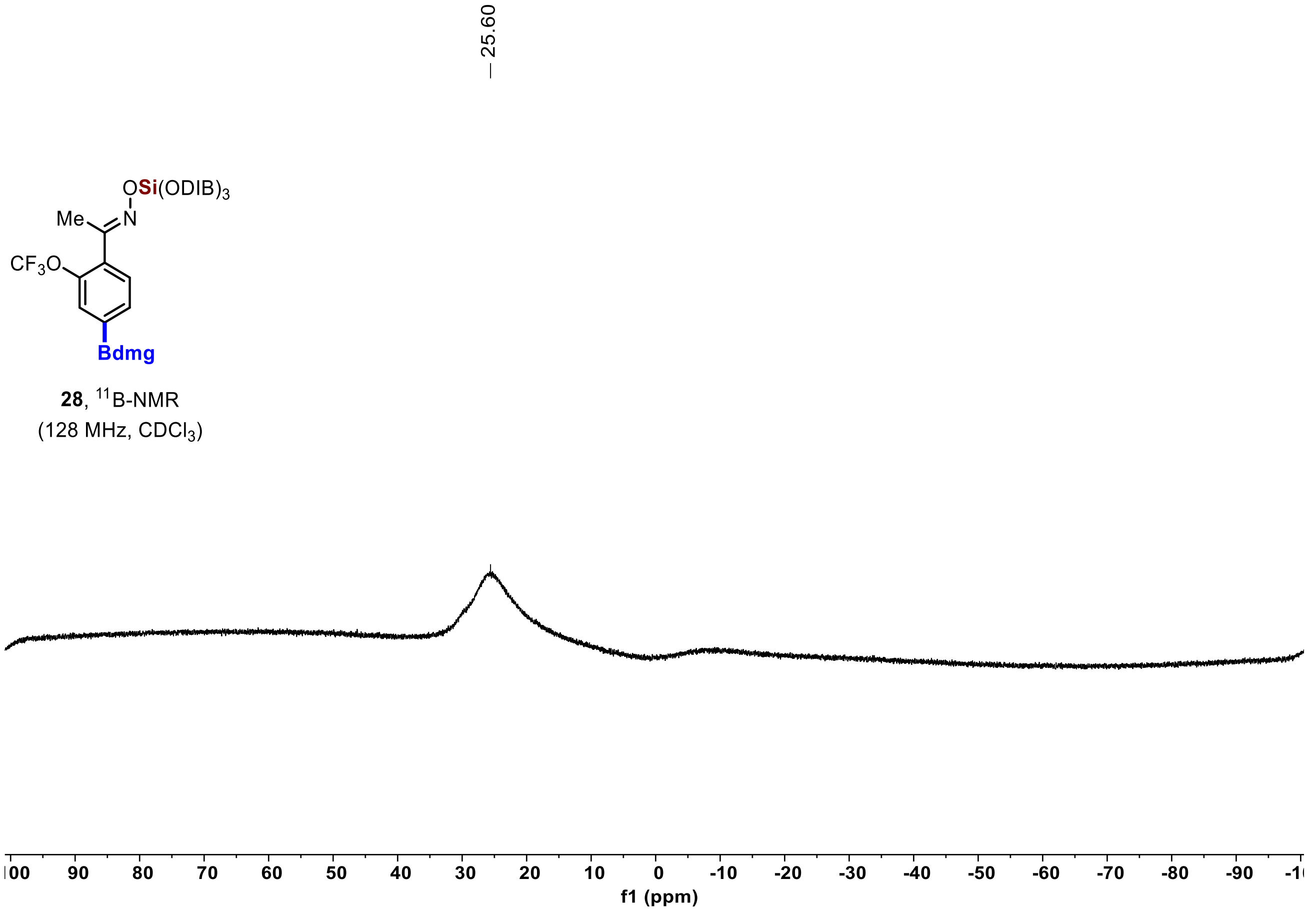
25.60
OSi(ODIB)3
Me
N
CF3O
Bdmg
28, 11B-NMR
(128 MHz, CDCl3)
f1 (ppm)

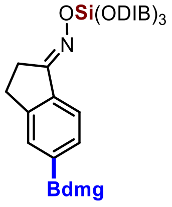

29, ^{1}H-NMR
(400 MHz, $CDCl_3$)

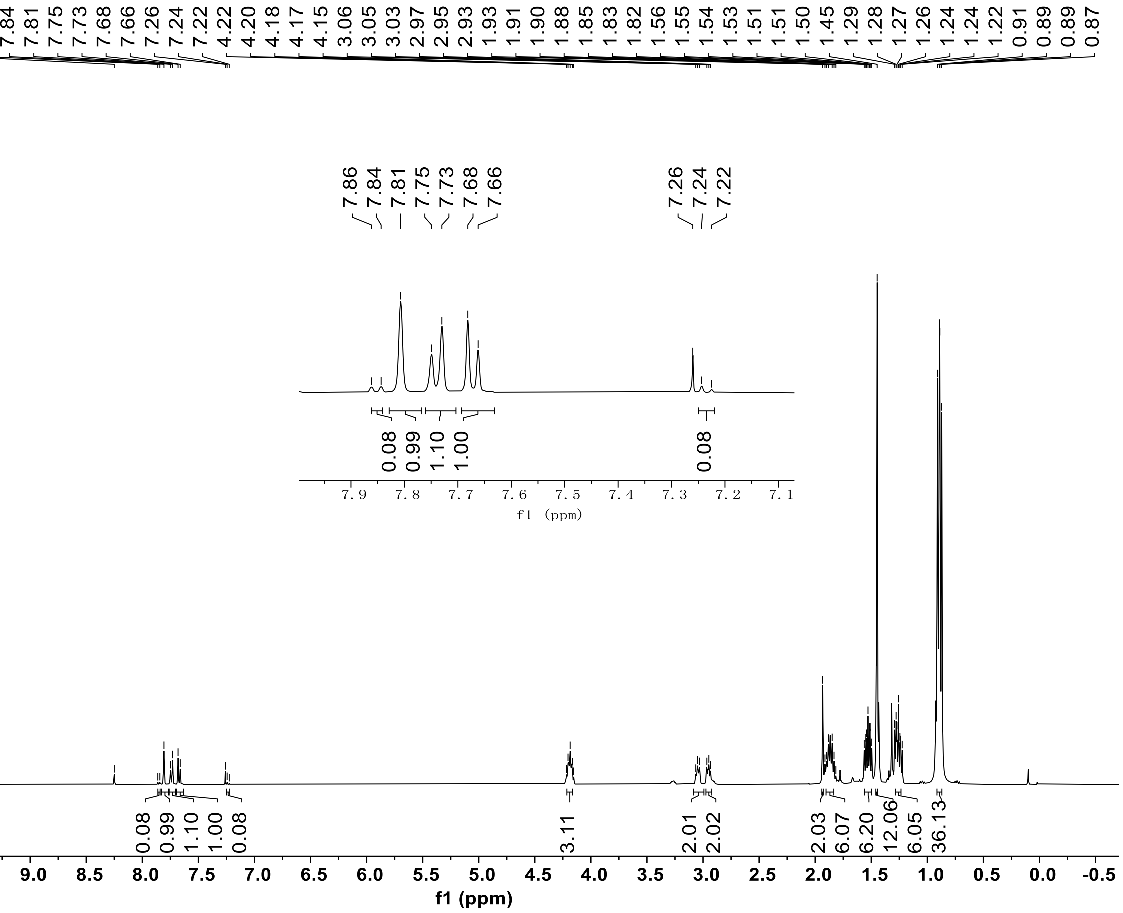

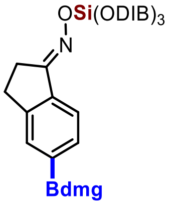

29, ^{13}C-NMR
(101 MHz, $CDCl_3$)

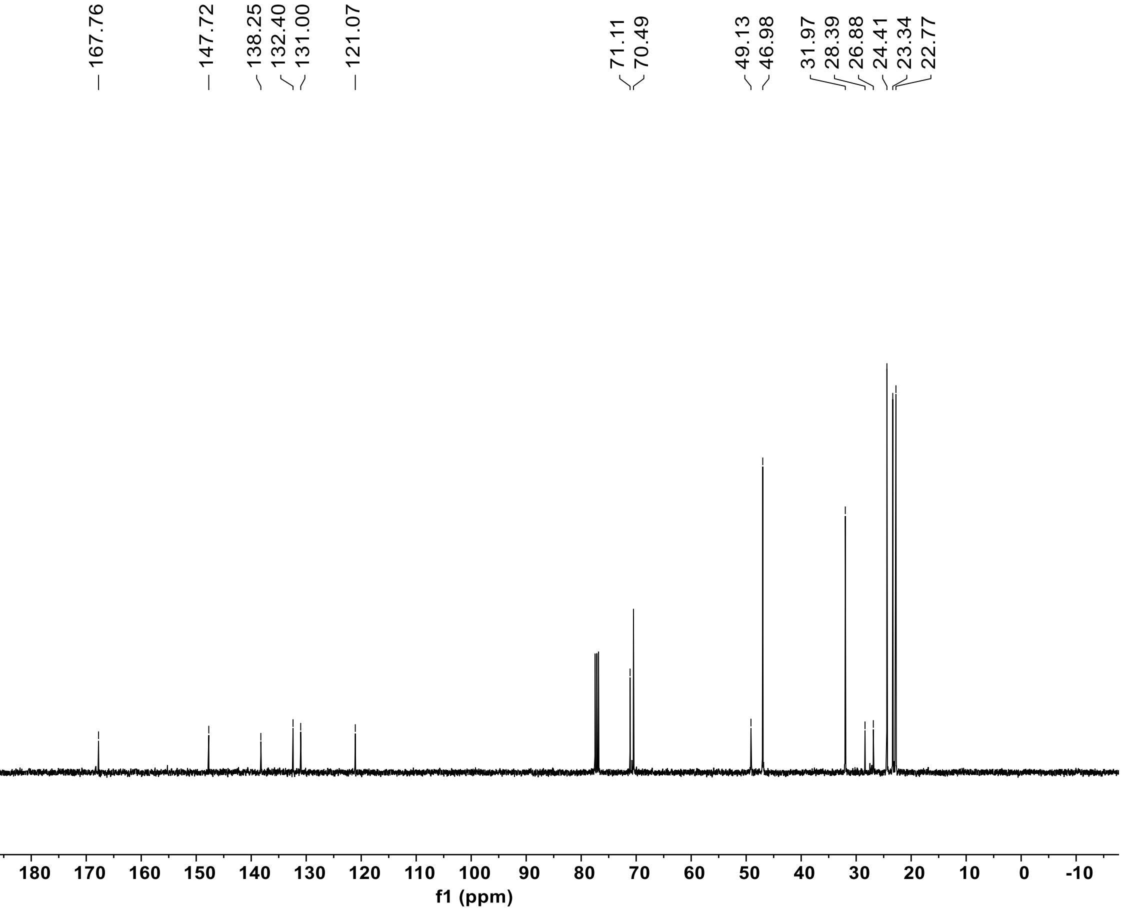

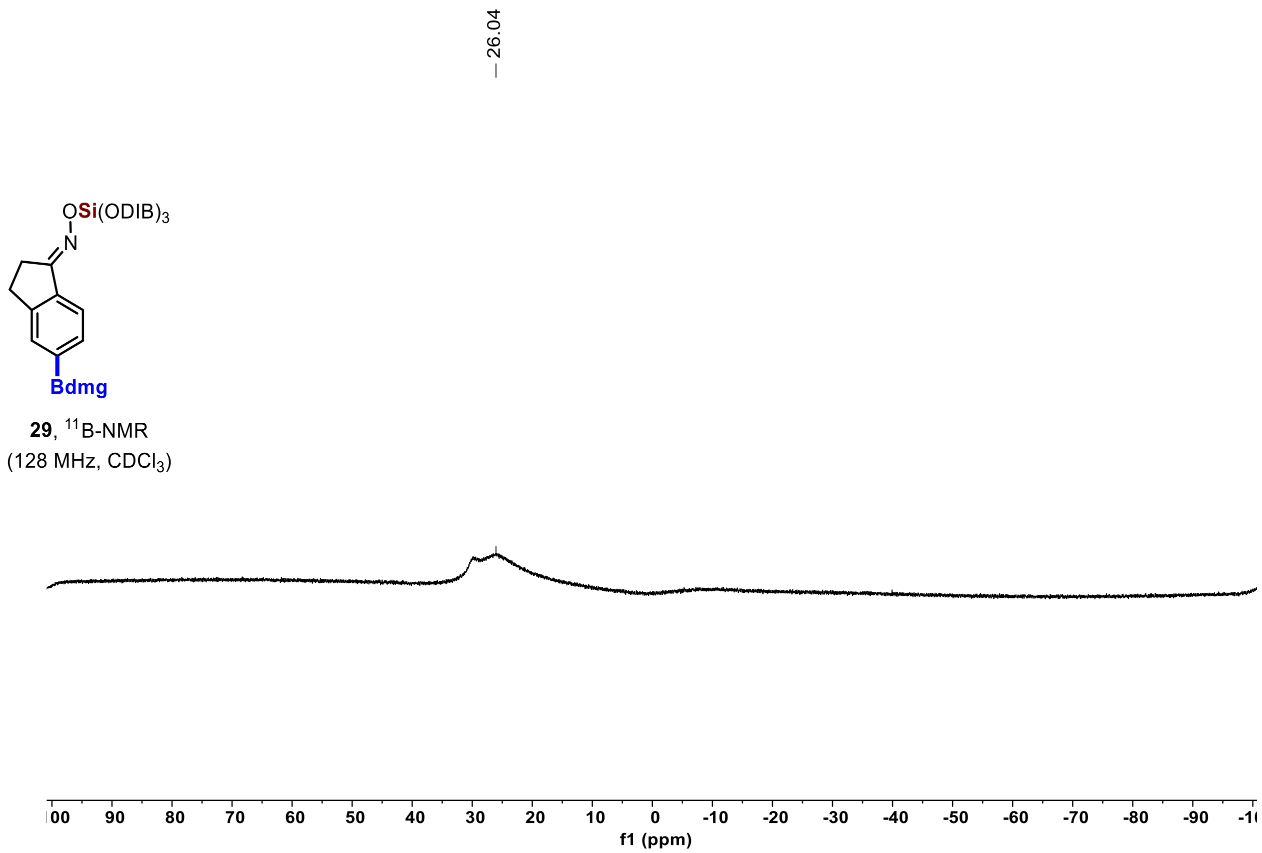

NOESY spectrum of 29 (400 MHz, CDCl$_3$)

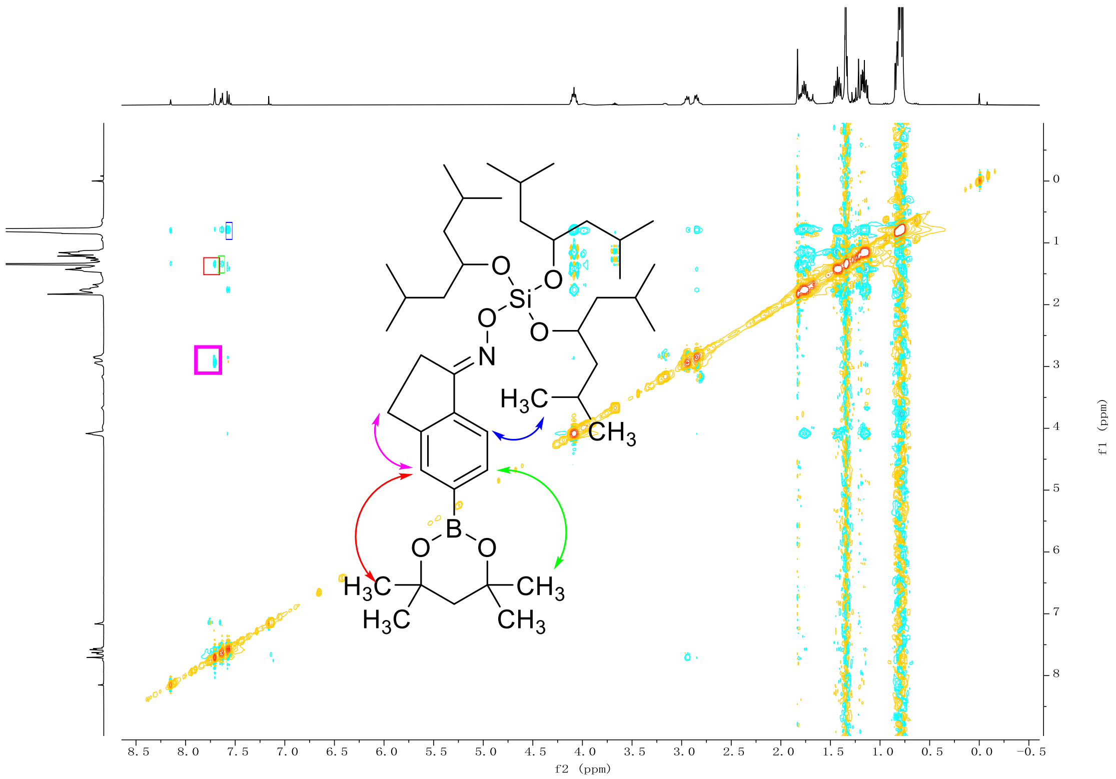

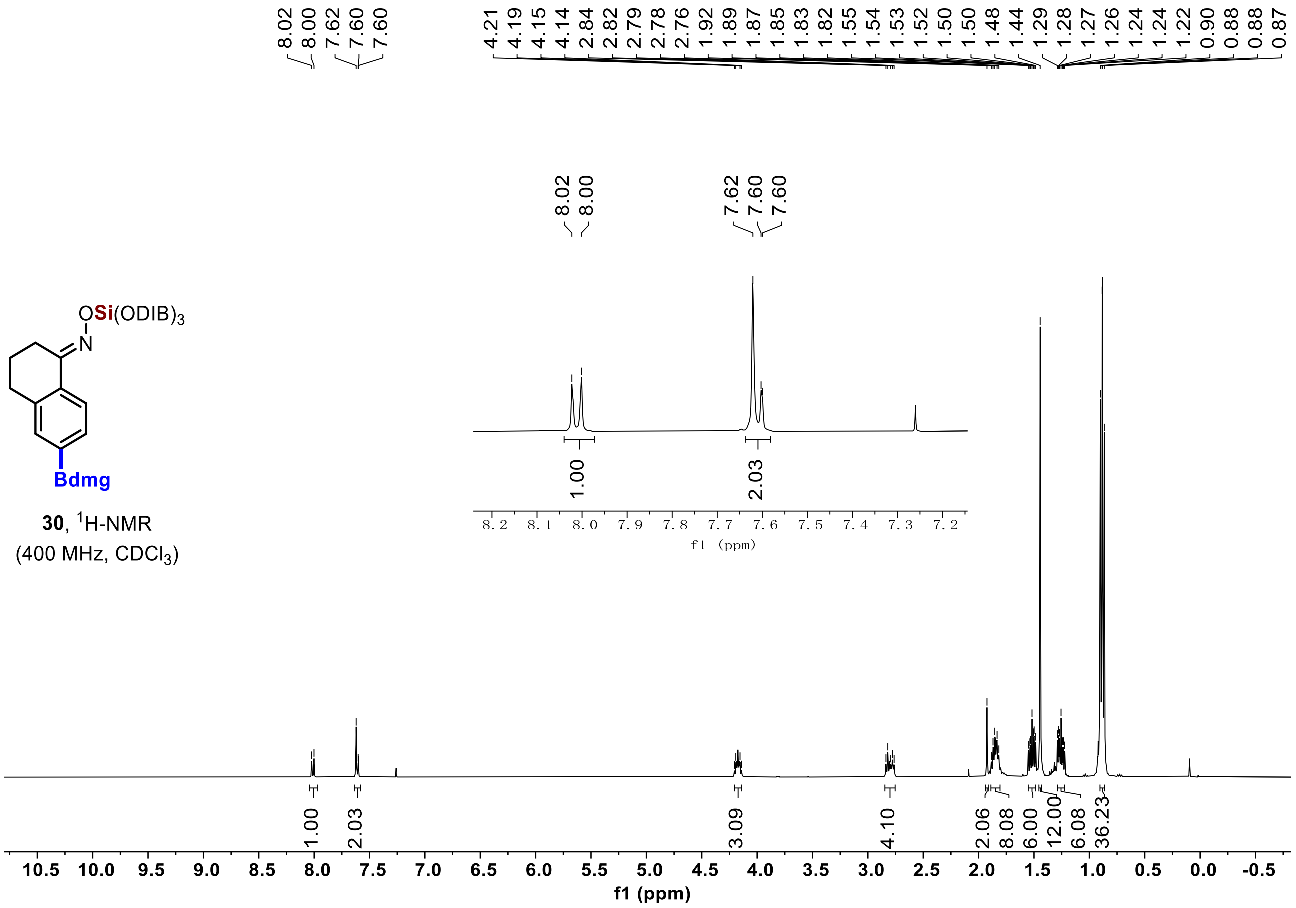

30, ^{1}H-NMR (400 MHz, $CDCl_3$)

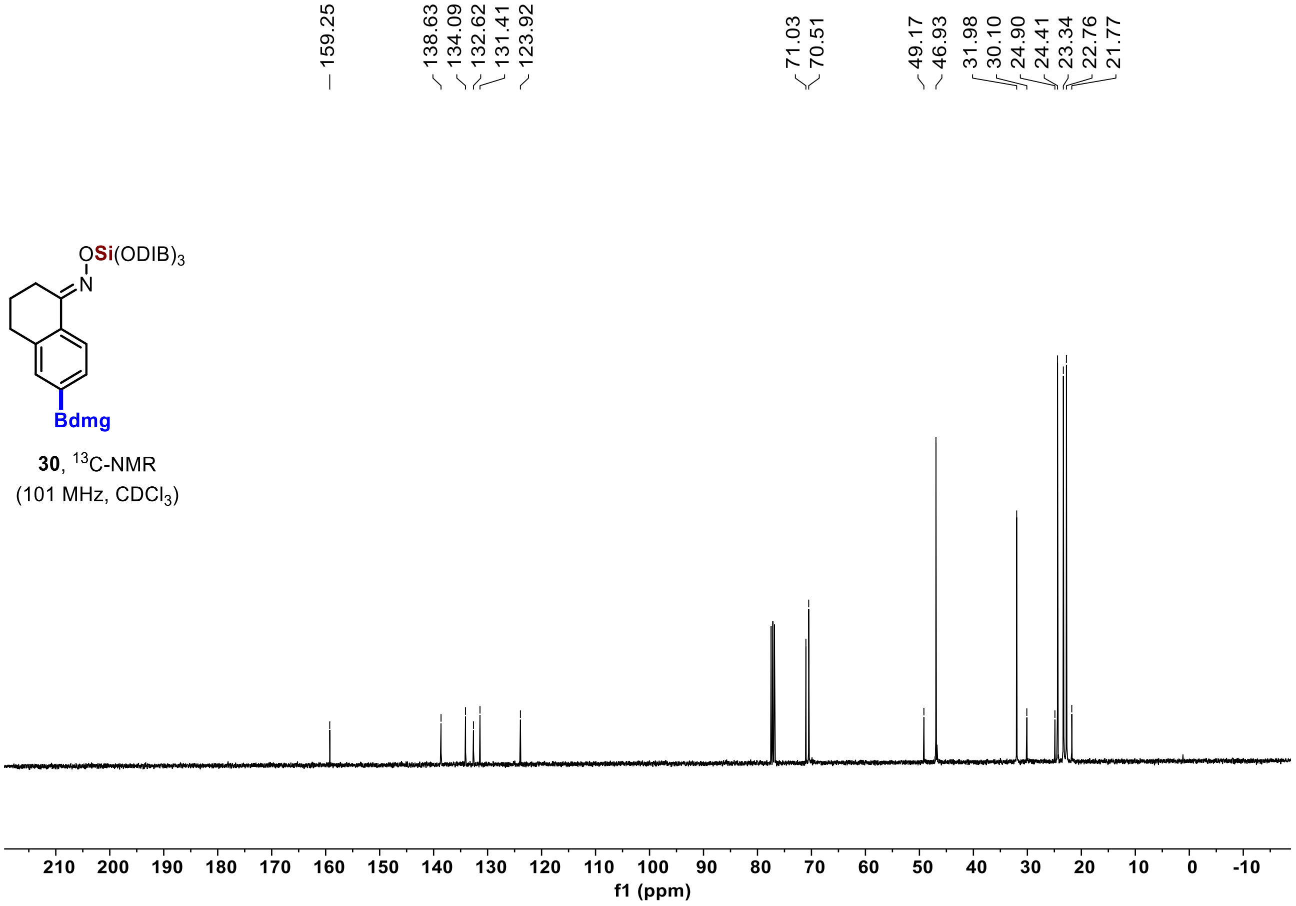

30, ^{13}C-NMR (101 MHz, $CDCl_3$)

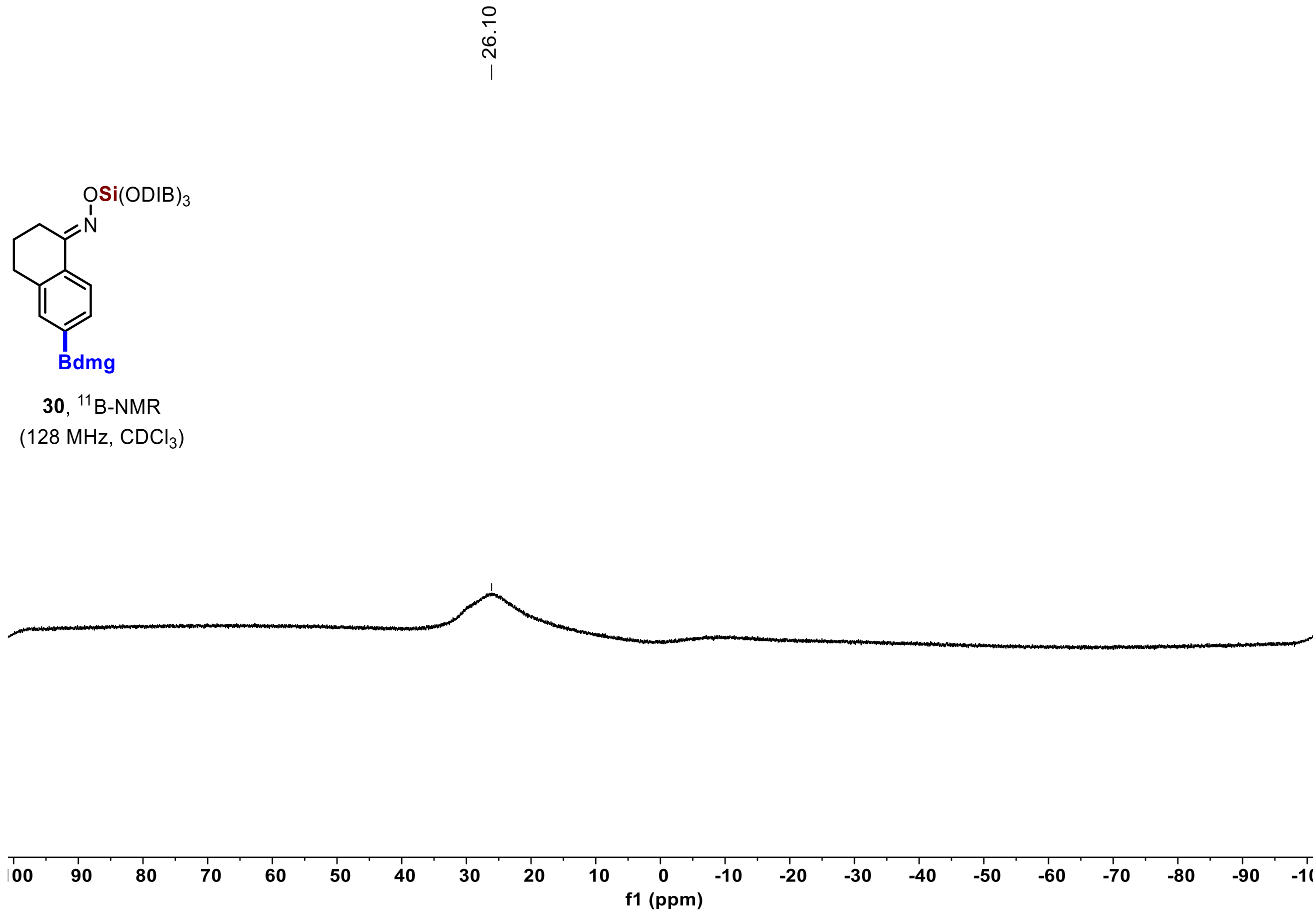

NOESY spectrum of 30 (400 MHz, CDCl$_3$)

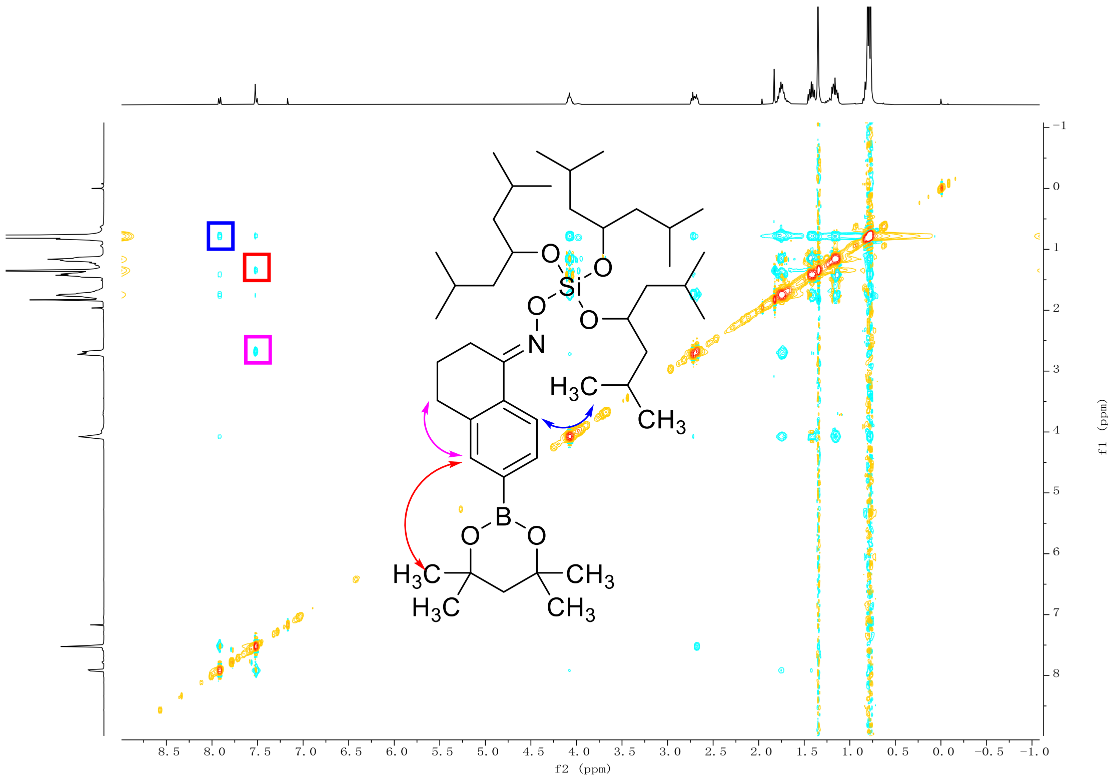

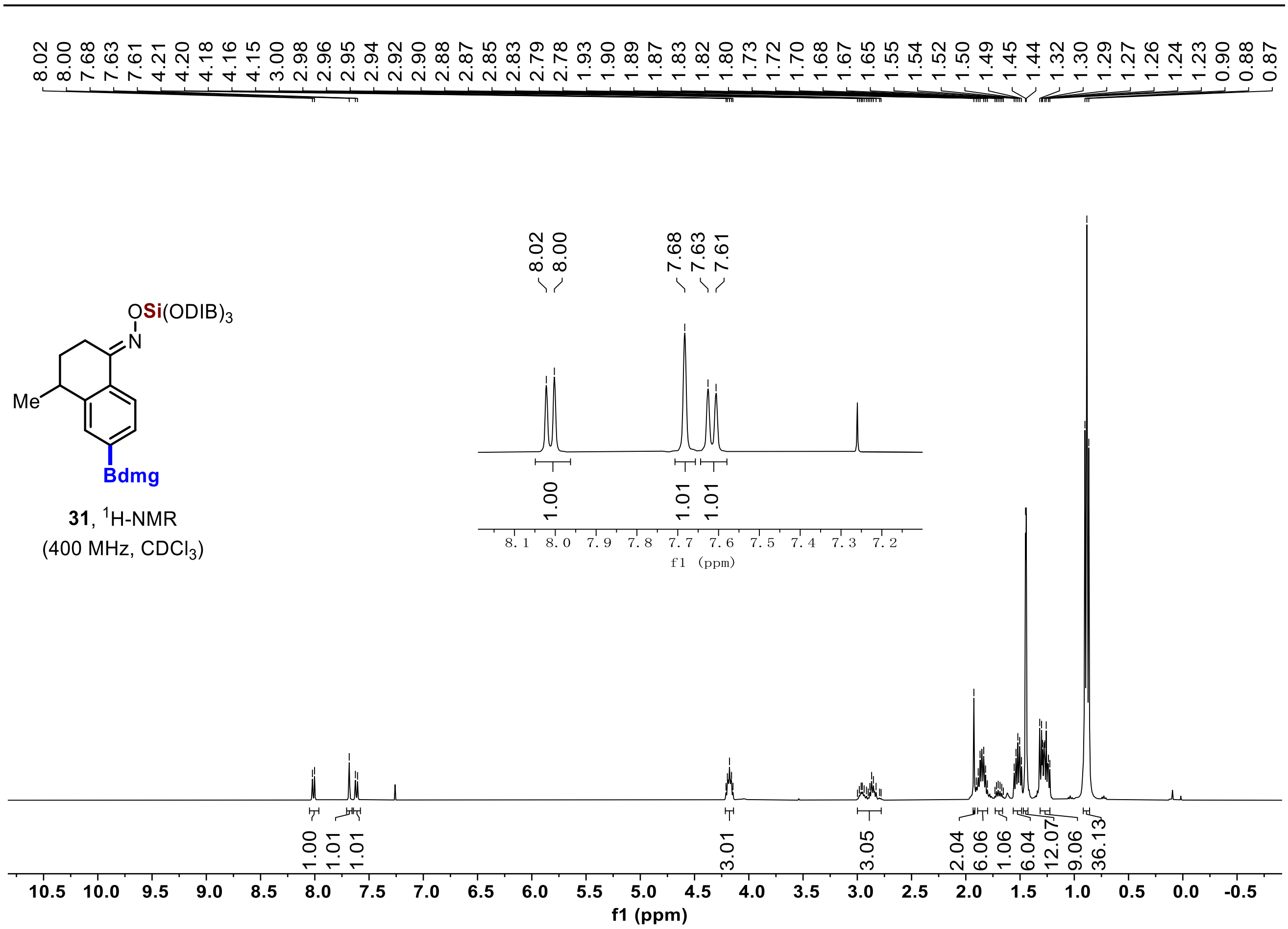
8.02 8.00 7.68 7.63 7.61 4.21 4.20 4.18 4.16 4.15 3.00 2.98 2.96 2.95 2.94 2.92 2.90 2.88 2.87 2.85 2.83 2.79 2.78 1.93 1.90 1.89 1.87 1.83 1.82 1.80 1.73 1.72 1.70 1.68 1.67 1.65 1.55 1.54 1.52 1.50 1.49 1.45 1.44 1.32 1.30 1.29 1.27 1.26 1.24 1.23 0.90 0.88 0.87
OSi(ODIB)3
N
Me
Bdmg
31, 1H-NMR
(400 MHz, CDCl3)
1.00 1.01 1.01 3.01 3.05 2.04 6.06 1.06 6.04 12.07 9.06 36.13
f1 (ppm)

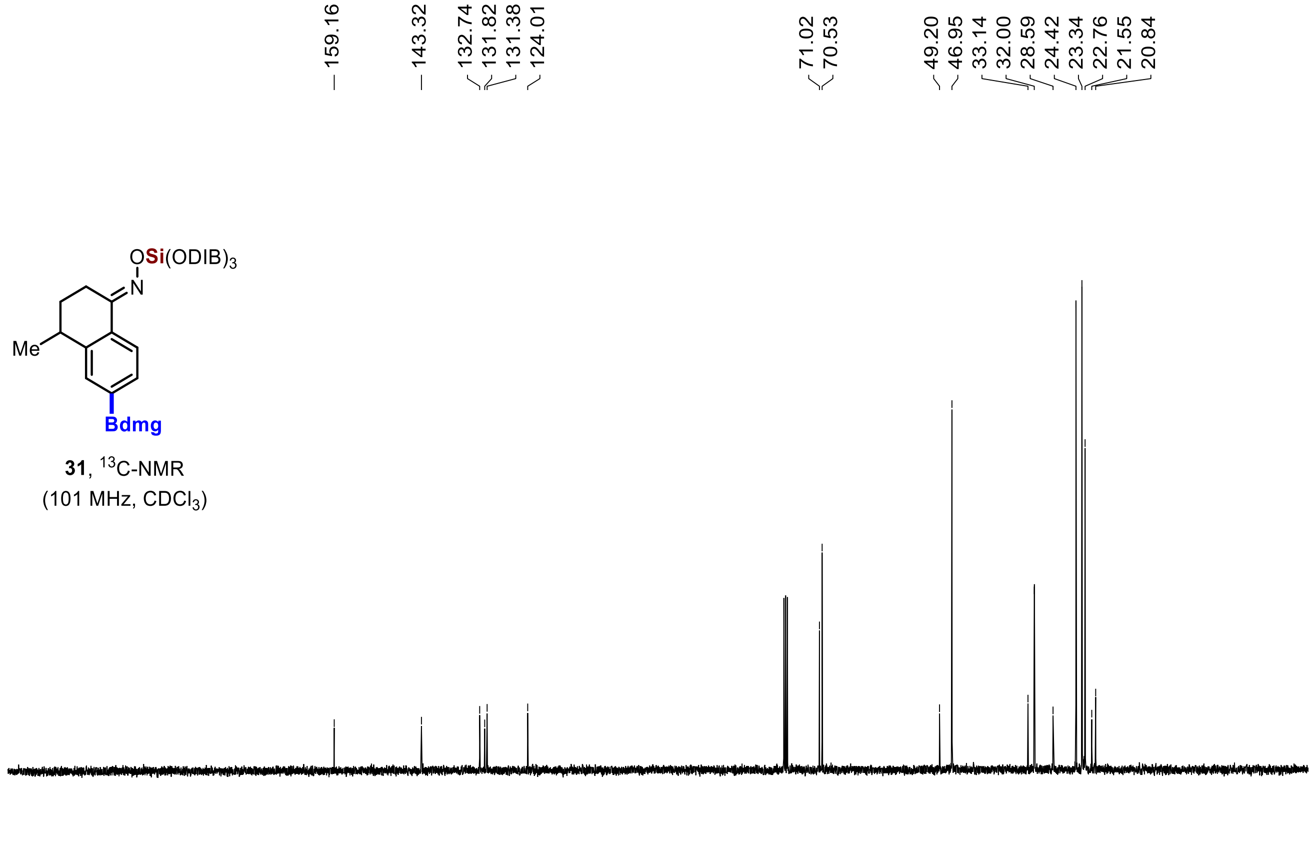
159.16 143.32 132.74 131.82 131.38 124.01 71.02 70.53 49.20 46.95 33.14 32.00 28.59 24.42 23.34 22.76 21.55 20.84
OSi(ODIB)3
N
Me
Bdmg
31, 13C-NMR
(101 MHz, CDCl3)
f1 (ppm)

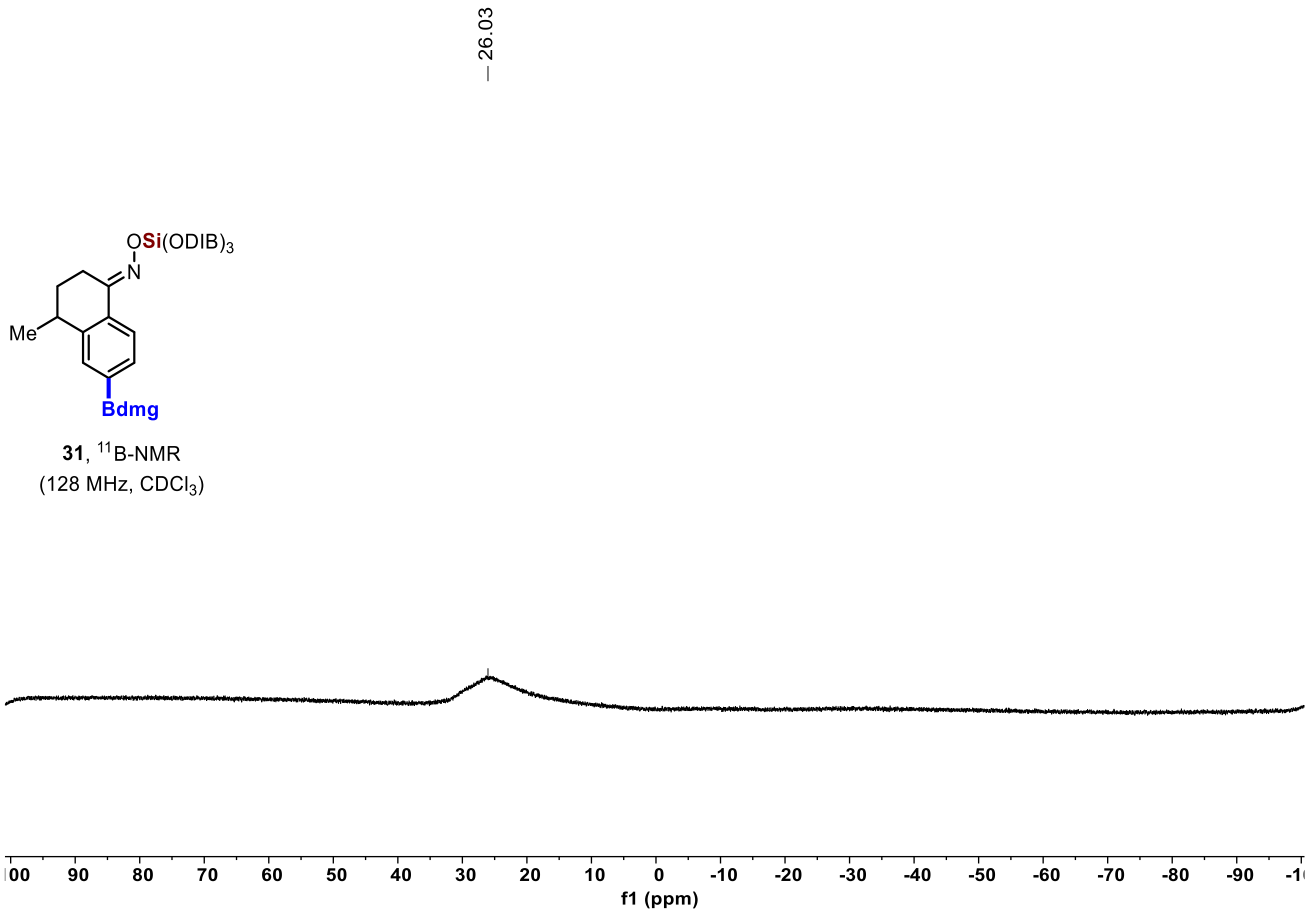

31, ^{11}B-NMR
(128 MHz, $CDCl_3$)

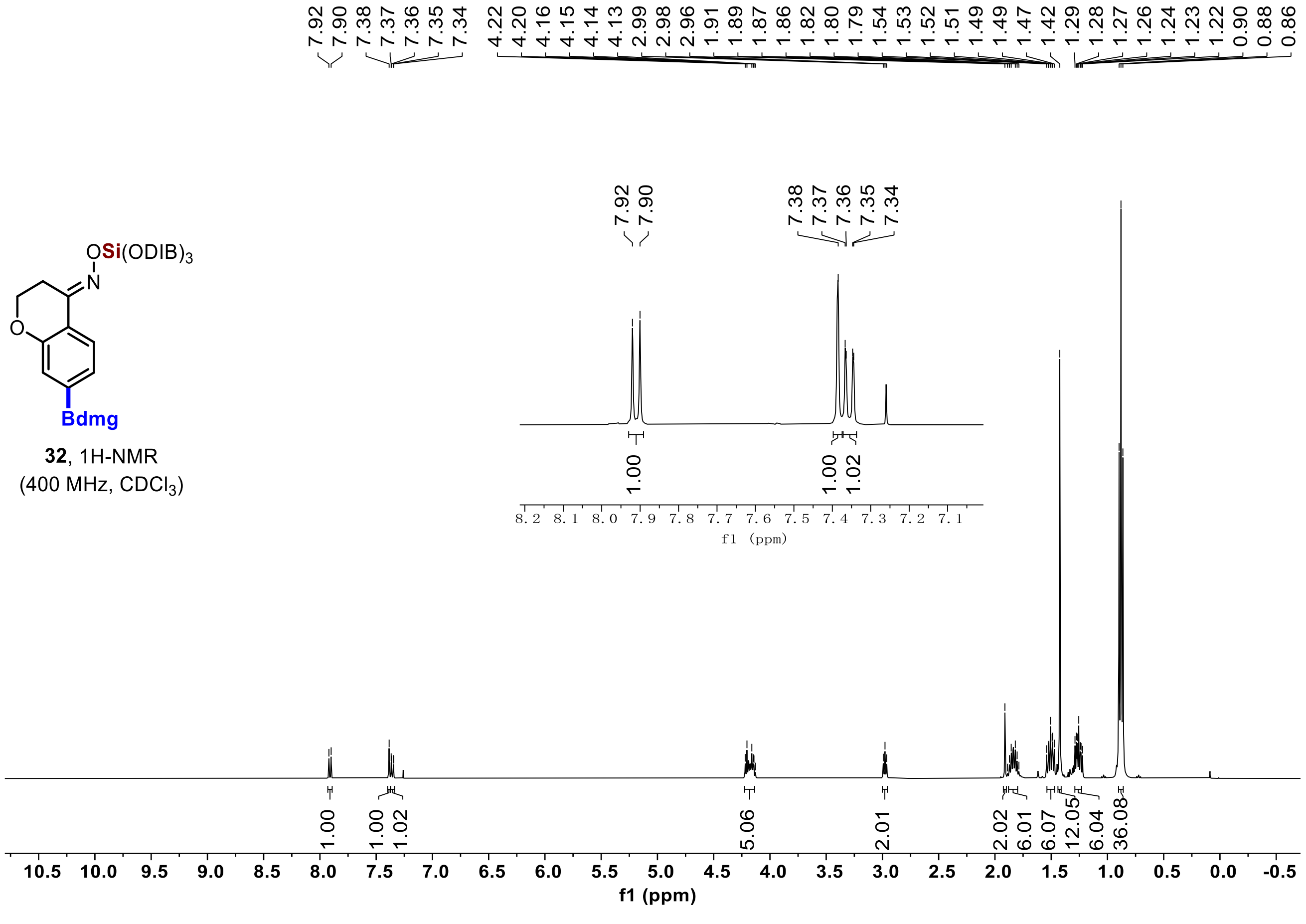

32, 1H-NMR
(400 MHz, $CDCl_3$)

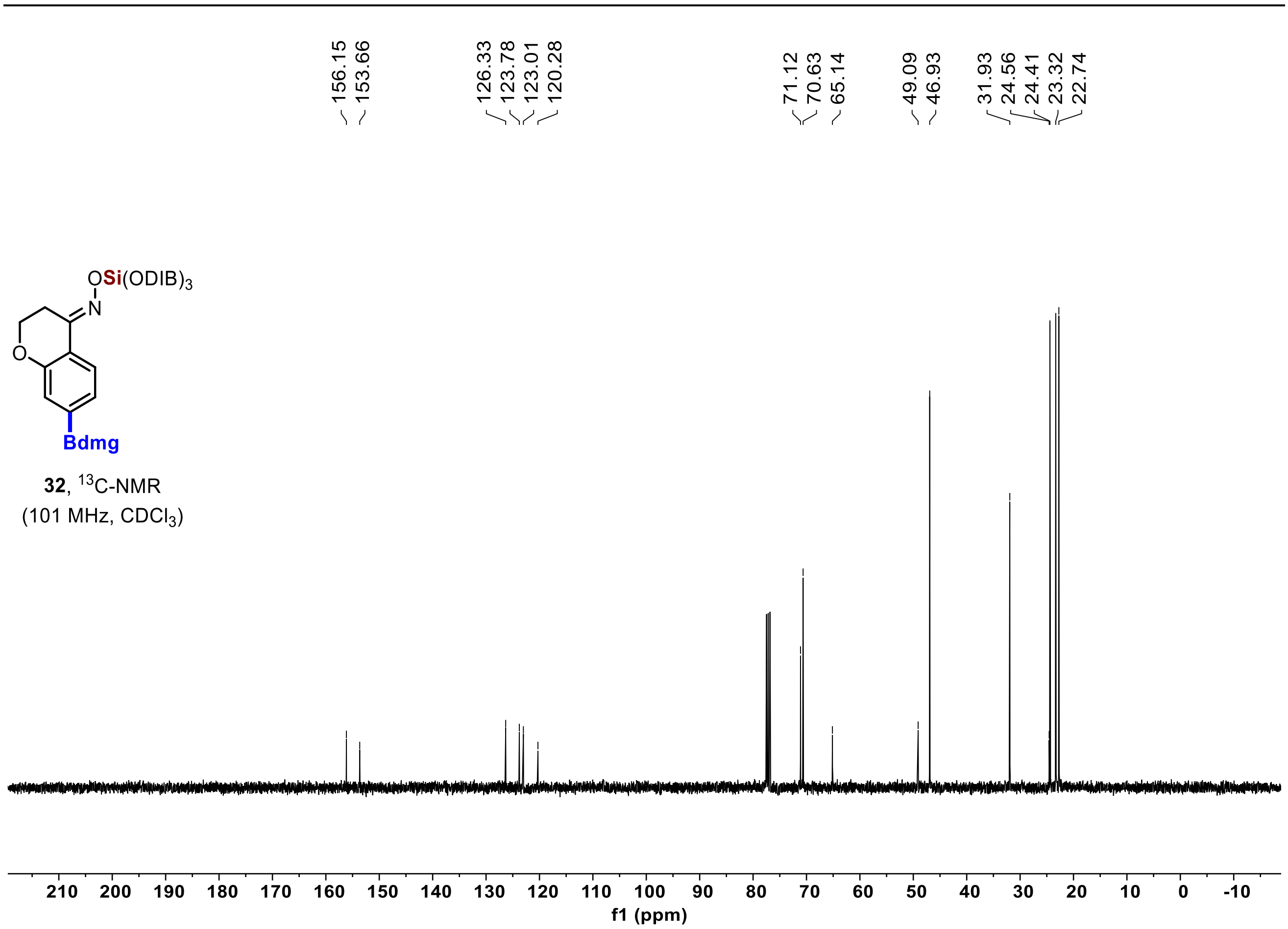

32, ^{13}C-NMR
(101 MHz, $CDCl_3$)

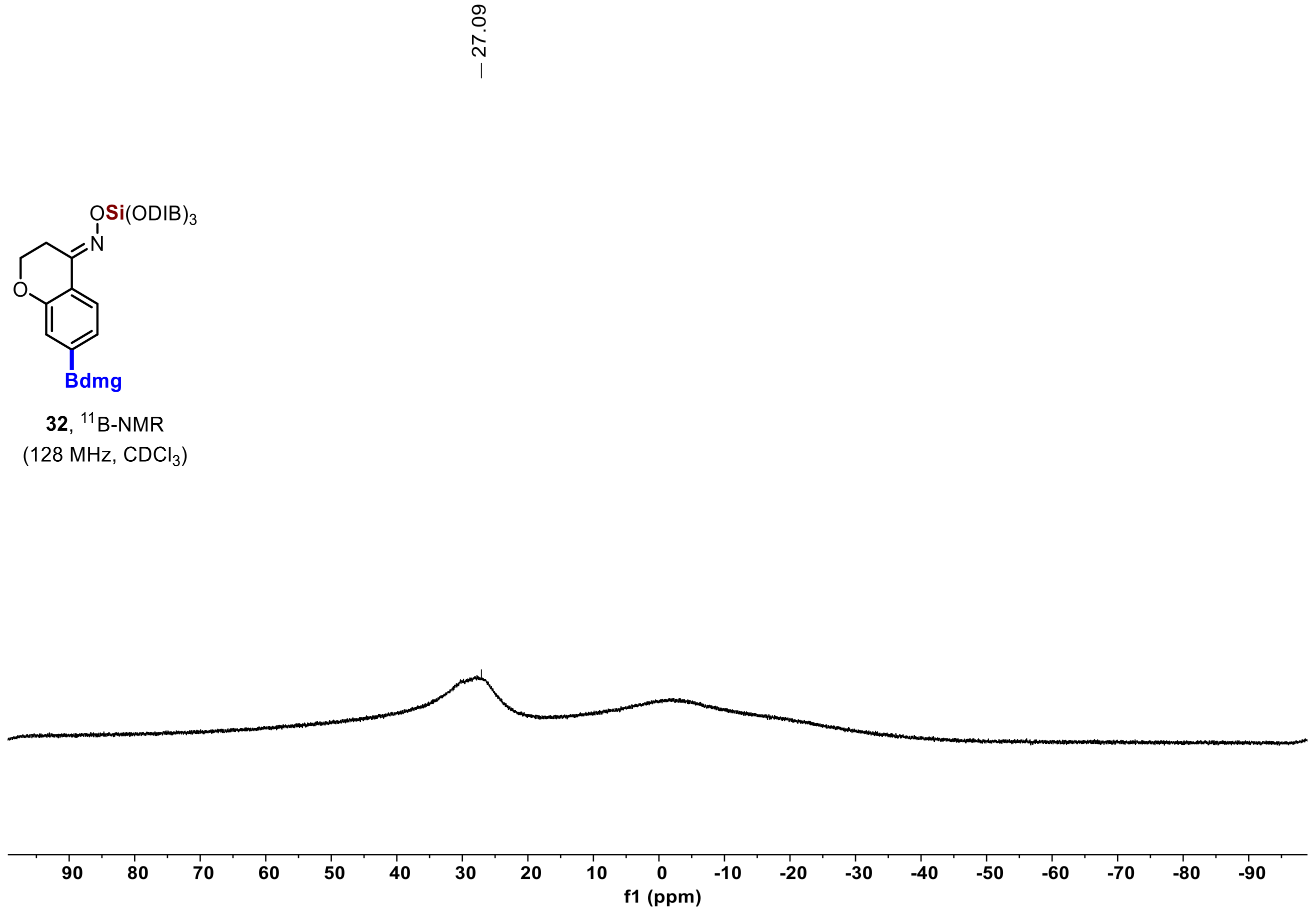

32, ^{11}B-NMR
(128 MHz, $CDCl_3$)

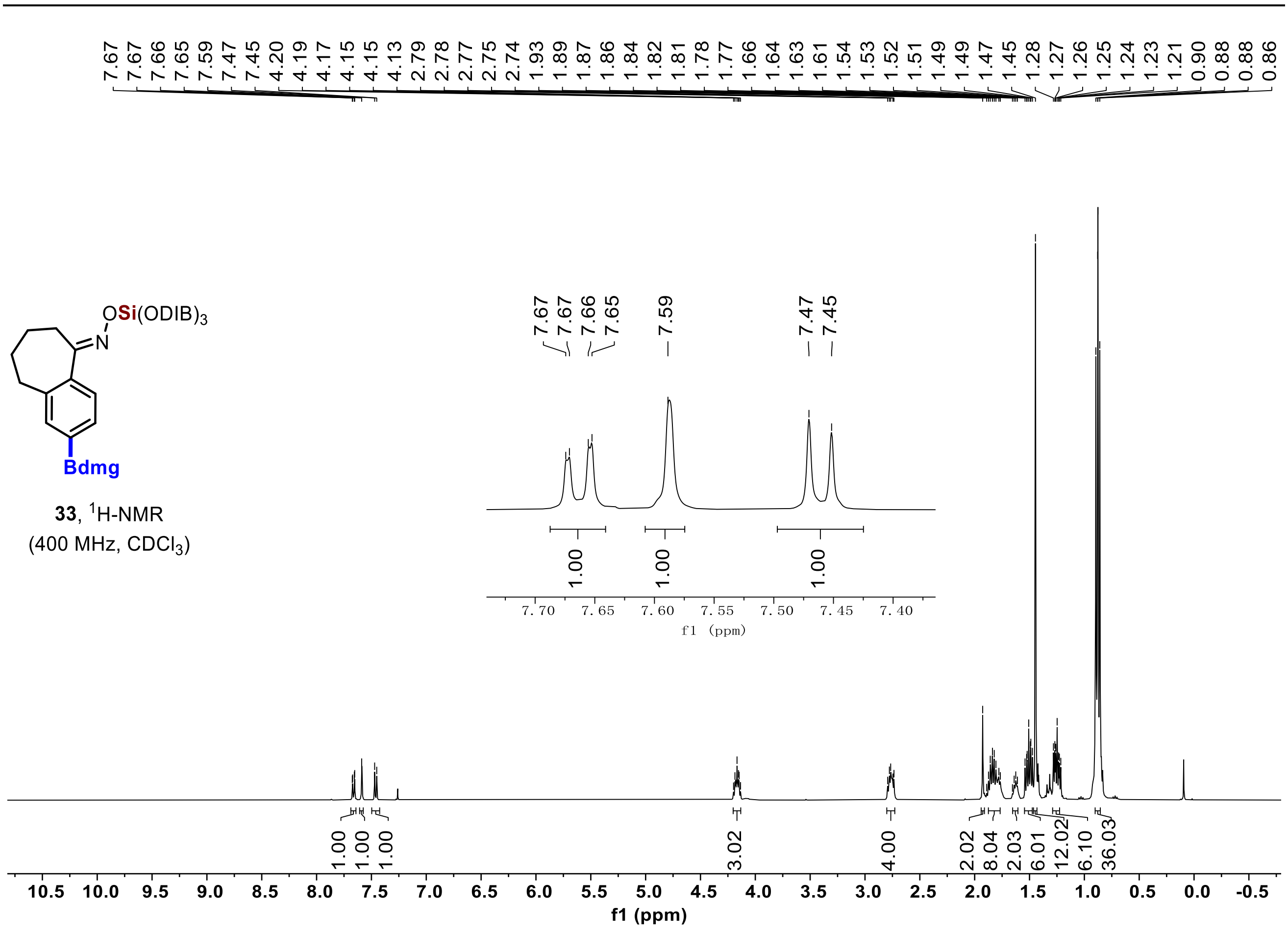

33, ^{1}H-NMR
(400 MHz, $CDCl_3$)

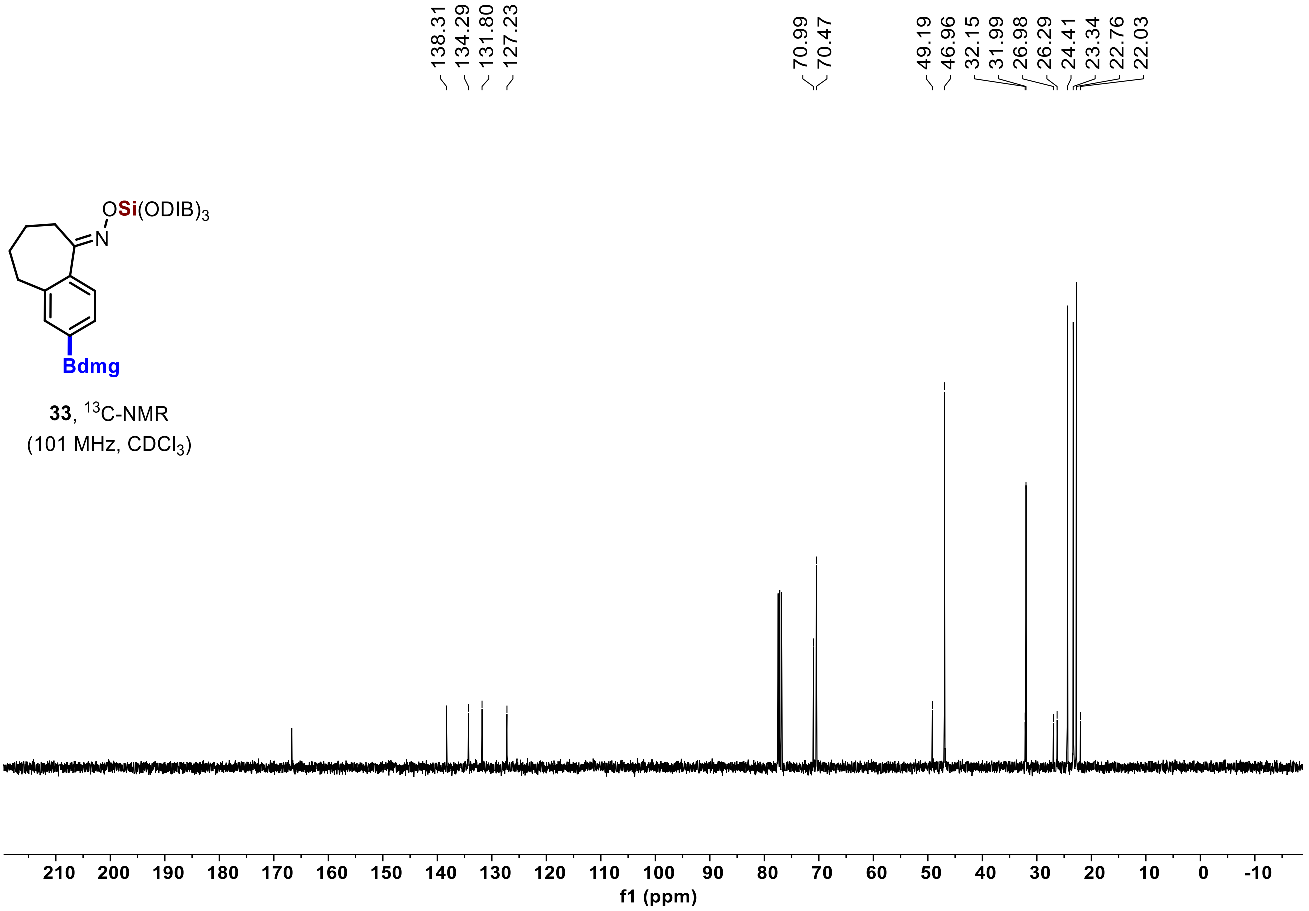

33, ^{13}C-NMR
(101 MHz, $CDCl_3$)

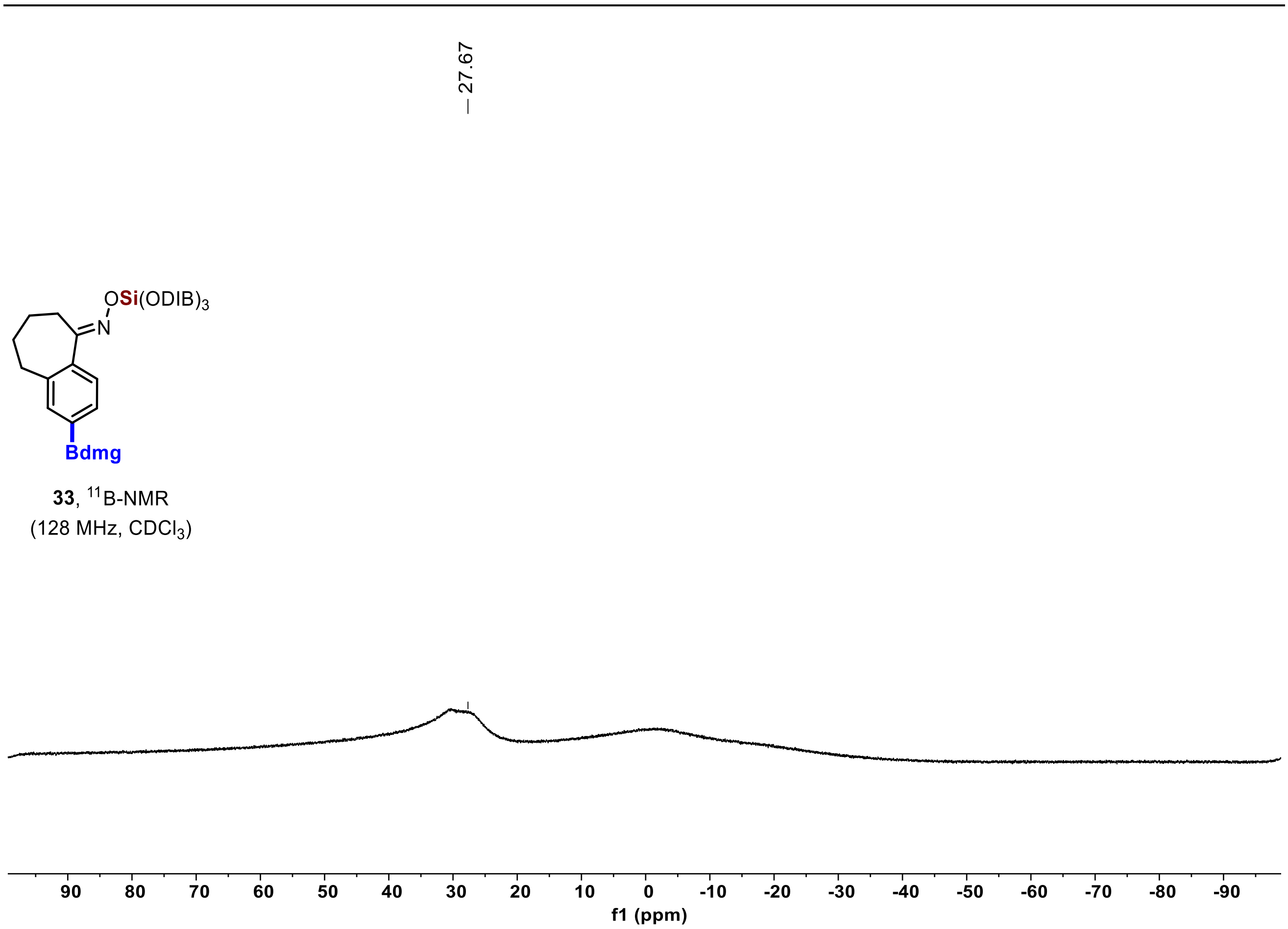
27.67
OSi(ODIB)3
N
Bdmg
33, 11B-NMR
(128 MHz, CDCl3)
90 80 70 60 50 40 30 20 10 0 -10 -20 -30 -40 -50 -60 -70 -80 -90
f1 (ppm)

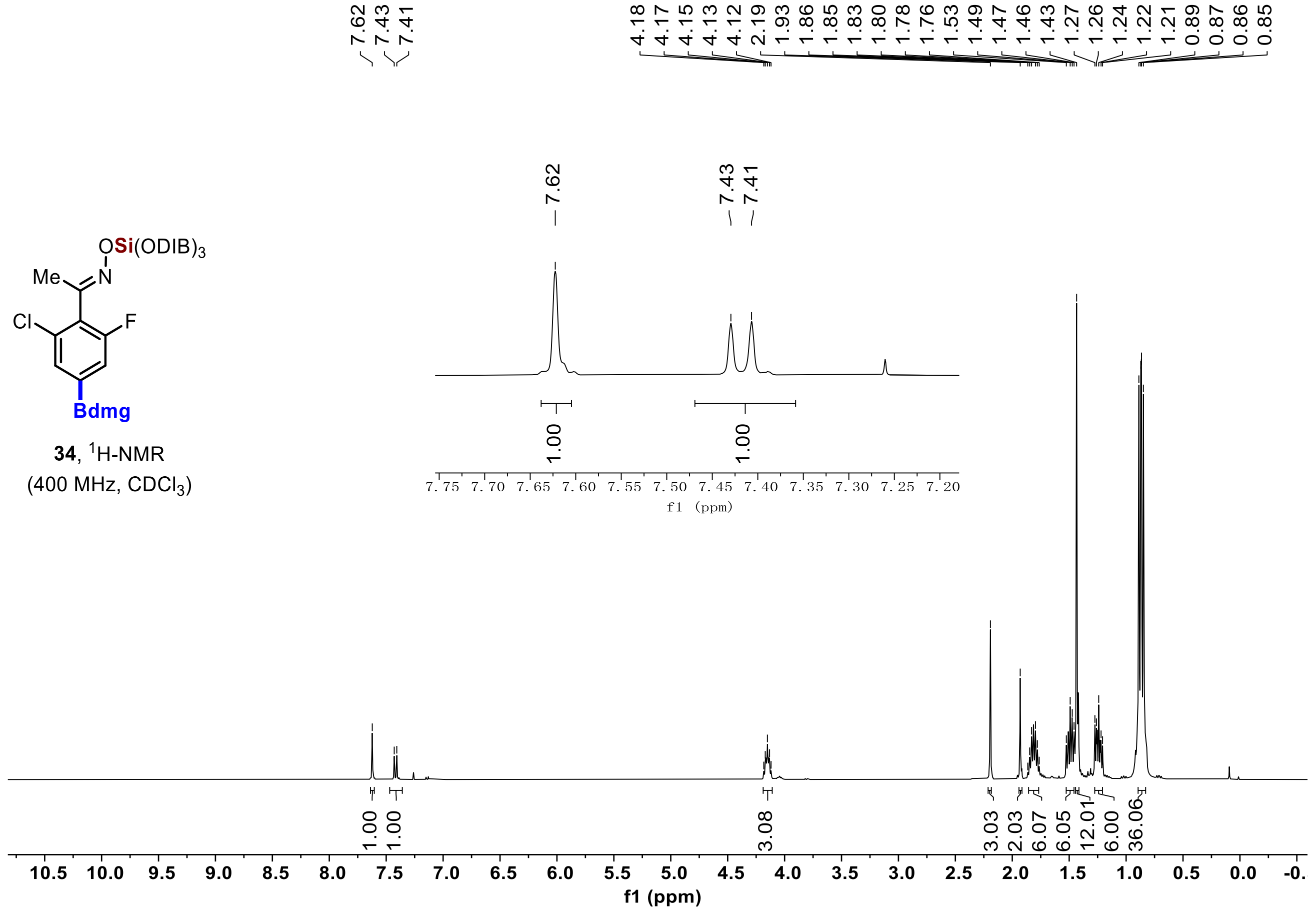
7.62
7.43
7.41
4.18
4.17
4.15
4.13
4.12
2.19
1.93
1.86
1.85
1.83
1.80
1.78
1.76
1.53
1.49
1.47
1.46
1.43
1.27
1.26
1.24
1.22
1.21
0.89
0.87
0.86
0.85
OSi(ODIB)3
Me
N
Cl
F
Bdmg
34, 1H-NMR
(400 MHz, CDCl3)
1.00
1.00
7.75 7.70 7.65 7.60 7.55 7.50 7.45 7.40 7.35 7.30 7.25 7.20
f1 (ppm)
1.00
1.00
3.08
3.03
2.03
6.07
6.05
12.01
6.00
36.06
10.5 10.0 9.5 9.0 8.5 8.0 7.5 7.0 6.5 6.0 5.5 5.0 4.5 4.0 3.5 3.0 2.5 2.0 1.5 1.0 0.5 0.0 -0.
f1 (ppm)

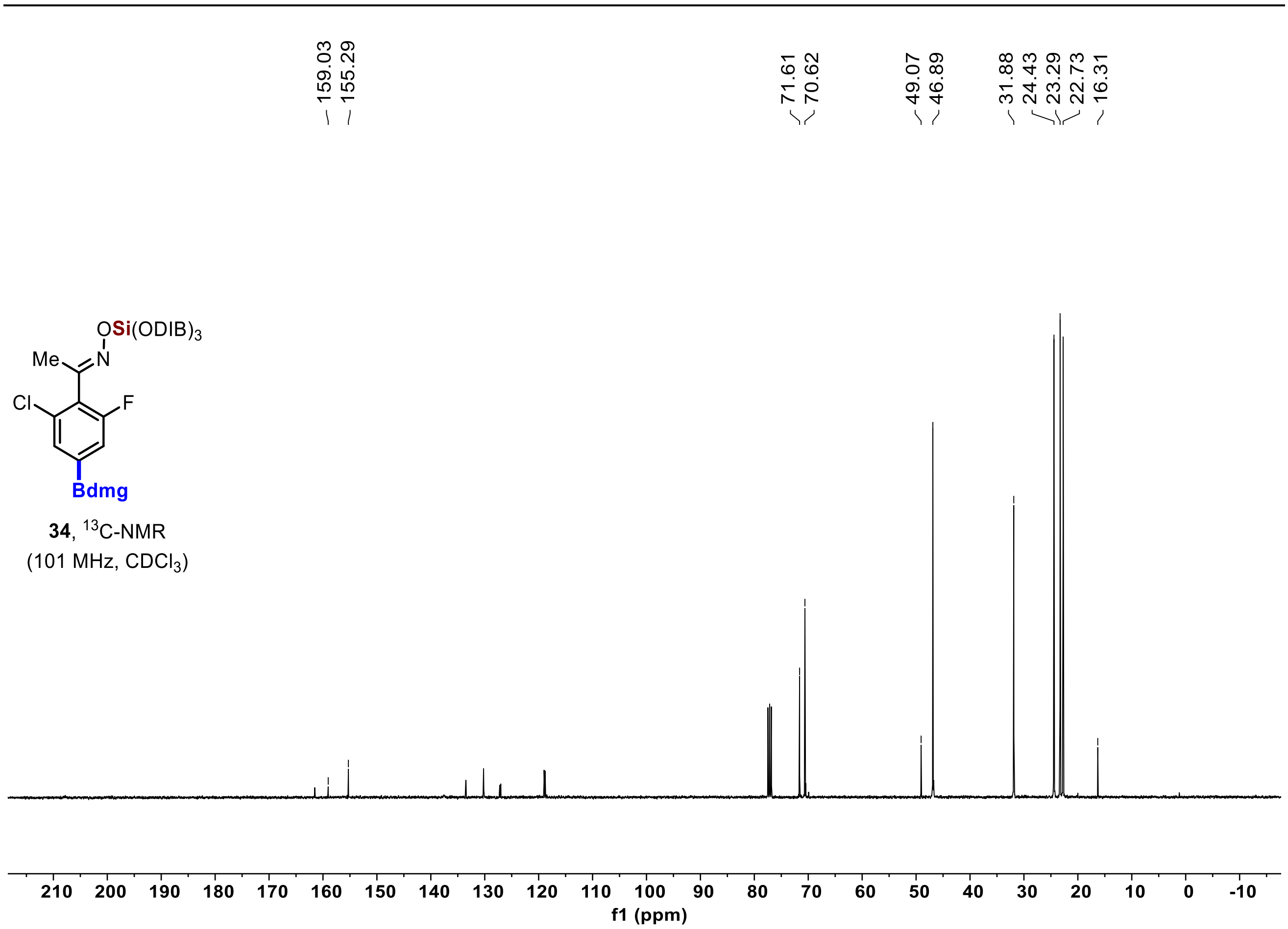

34, ^{13}C-NMR
(101 MHz, $CDCl_3$)

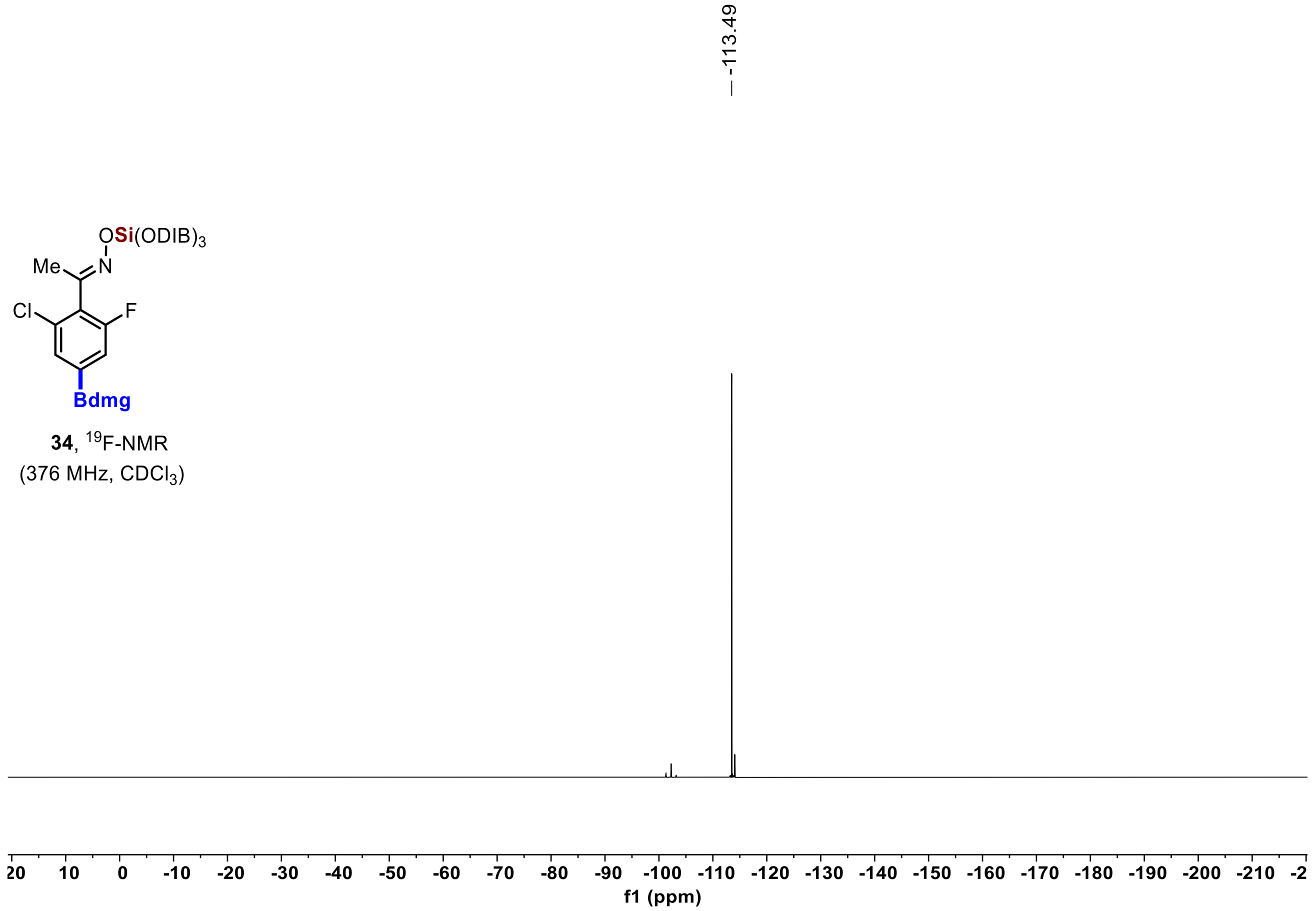

34, ^{19}F-NMR
(376 MHz, $CDCl_3$)

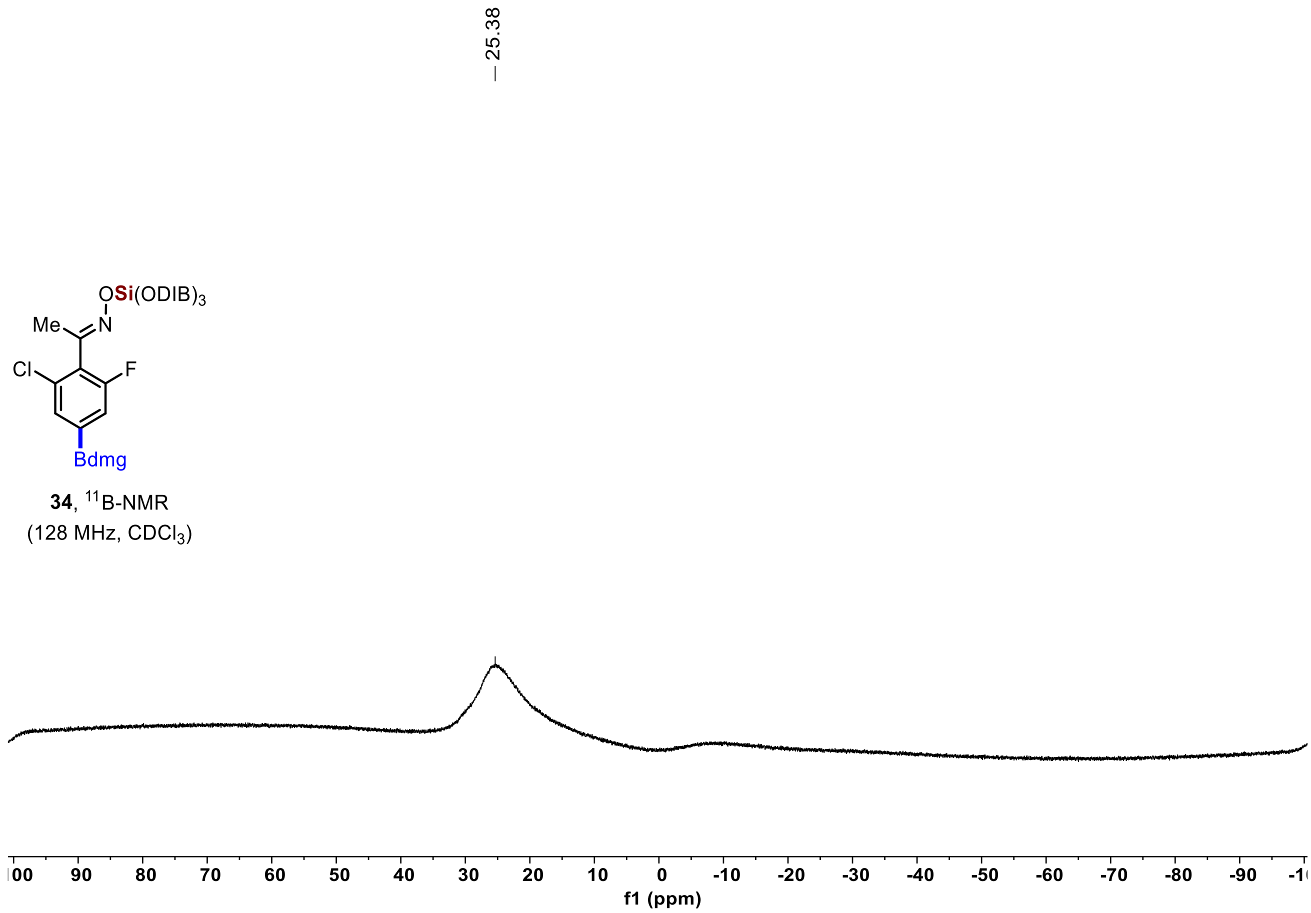
25.38
OSi(ODIB)3
Me
N
Cl
F
Bdmg
34, 11B-NMR
(128 MHz, CDCl3)
f1 (ppm)

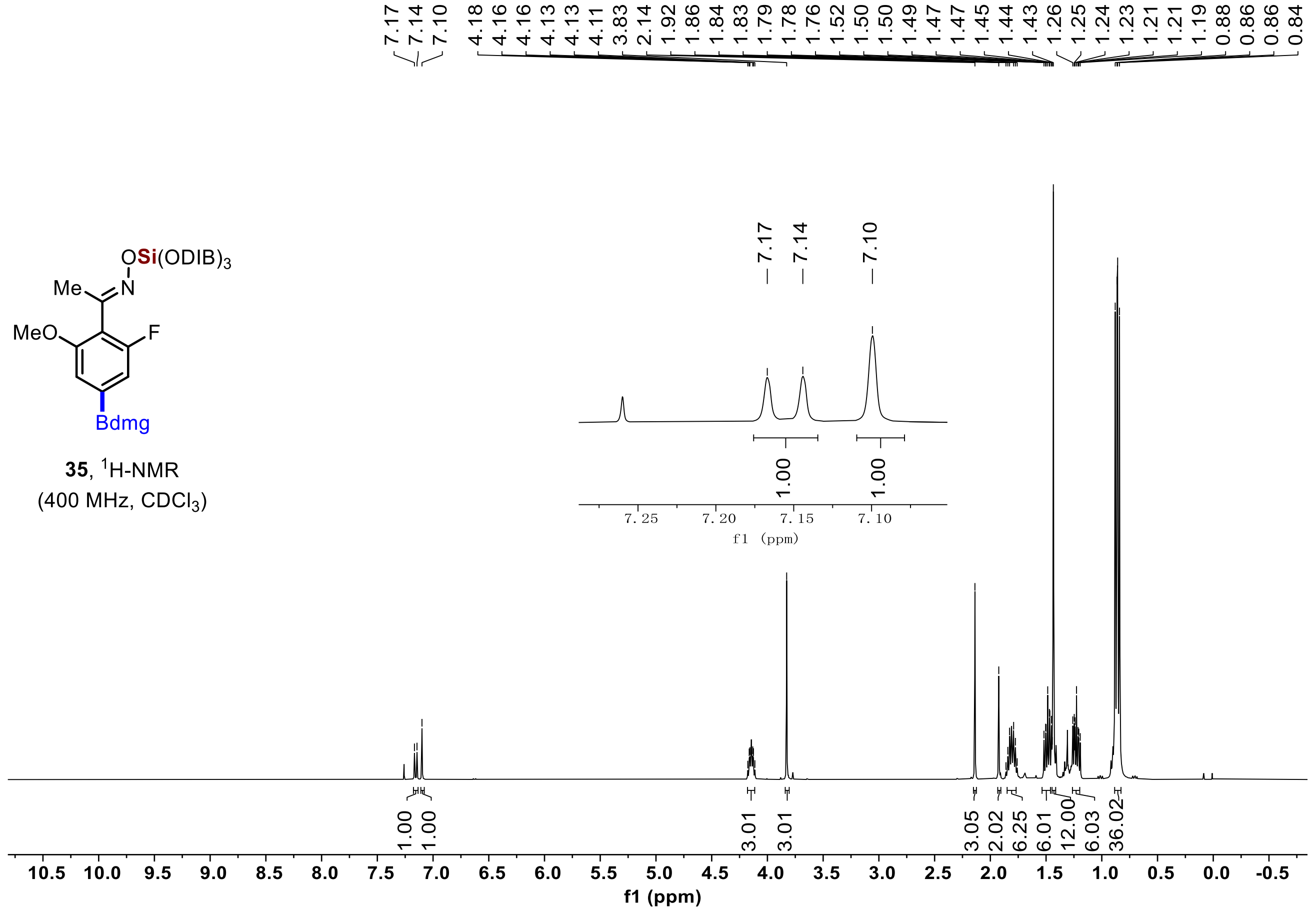
7.17 7.14 7.10 4.18 4.16 4.16 4.13 4.13 4.11 3.83 2.14 1.92 1.86 1.84 1.83 1.79 1.78 1.76 1.52 1.50 1.50 1.49 1.47 1.47 1.45 1.44 1.43 1.26 1.25 1.24 1.23 1.21 1.21 1.19 0.88 0.86 0.86 0.84
OSi(ODIB)3
Me
N
MeO
F
Bdmg
35, 1H-NMR
(400 MHz, CDCl3)
1.00 1.00 3.01 3.01 3.05 2.02 6.25 6.01 12.00 6.03 36.02
f1 (ppm)

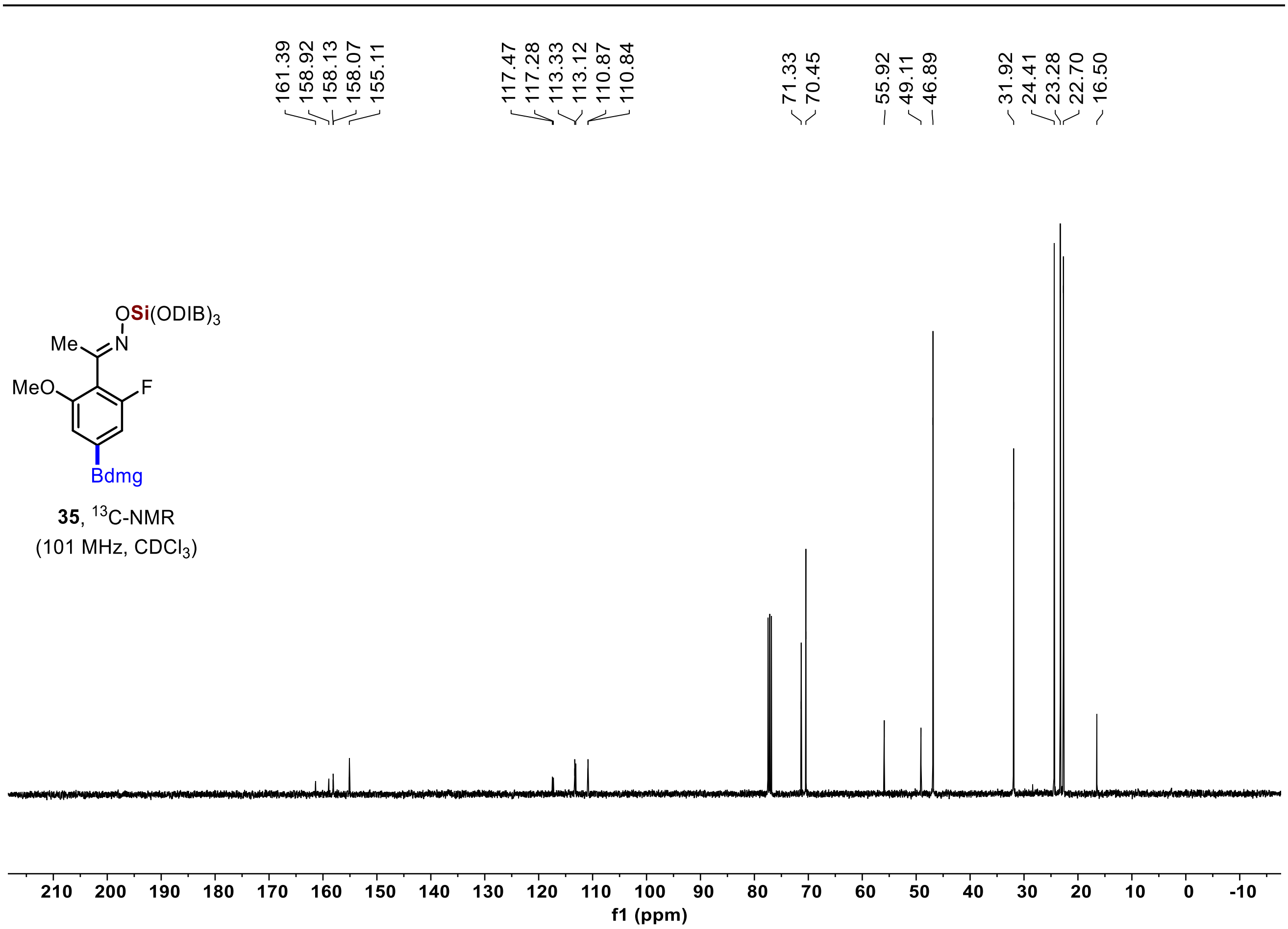
161.39
158.92
158.13
158.07
155.11
117.47
117.28
113.33
113.12
110.87
110.84
71.33
70.45
55.92
49.11
46.89
31.92
24.41
23.28
22.70
16.50
OSi(ODIB)3
Me
N
MeO
F
Bdmg
35, 13C-NMR
(101 MHz, CDCl3)
210 200 190 180 170 160 150 140 130 120 110 100 90 80 70 60 50 40 30 20 10 0 -10
f1 (ppm)

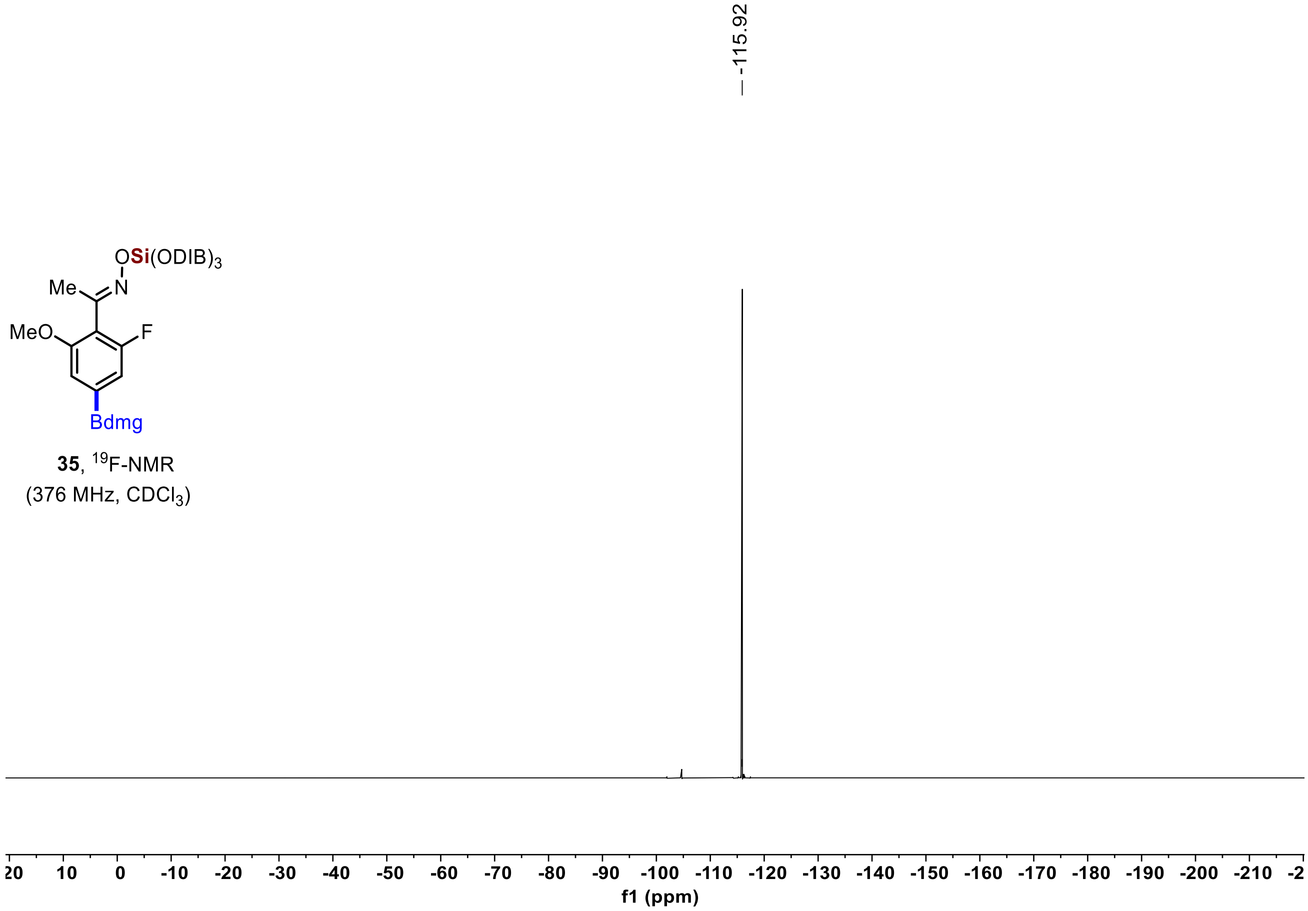
-115.92
OSi(ODIB)3
Me
N
MeO
F
Bdmg
35, 19F-NMR
(376 MHz, CDCl3)
20 10 0 -10 -20 -30 -40 -50 -60 -70 -80 -90 -100 -110 -120 -130 -140 -150 -160 -170 -180 -190 -200 -210 -2
f1 (ppm)

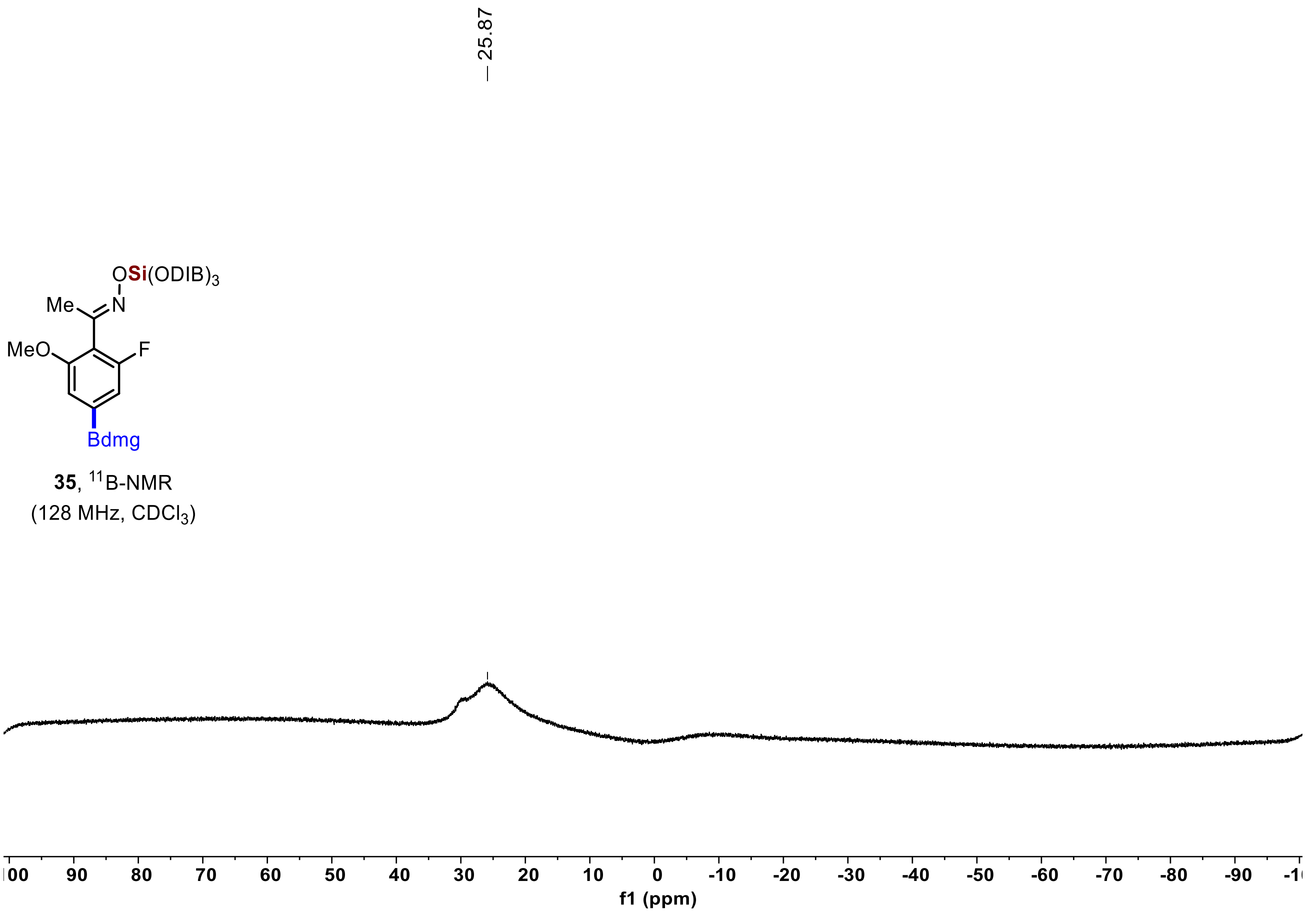

35, ^{11}B-NMR
(128 MHz, $CDCl_3$)

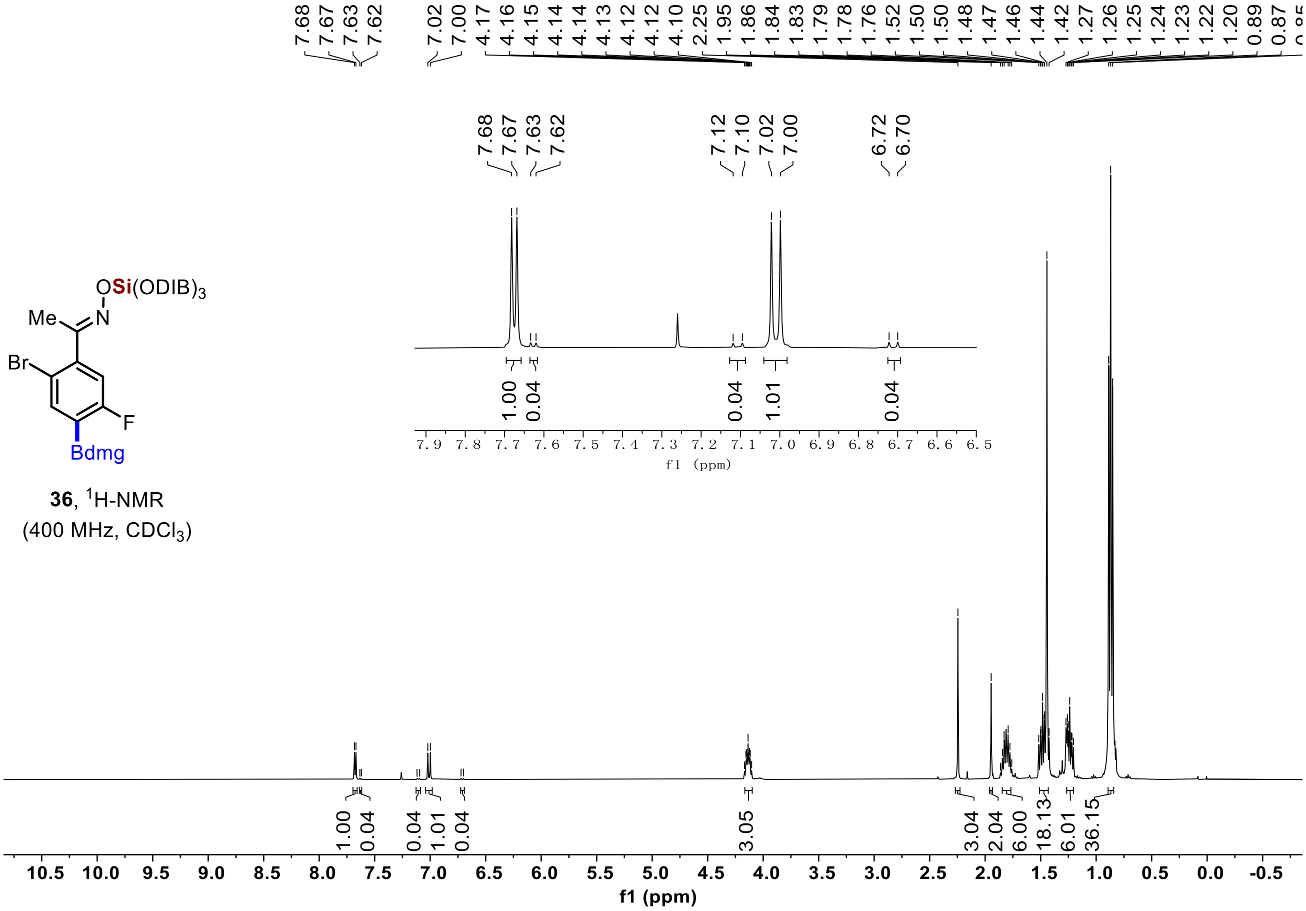

36, ^{1}H-NMR
(400 MHz, $CDCl_3$)

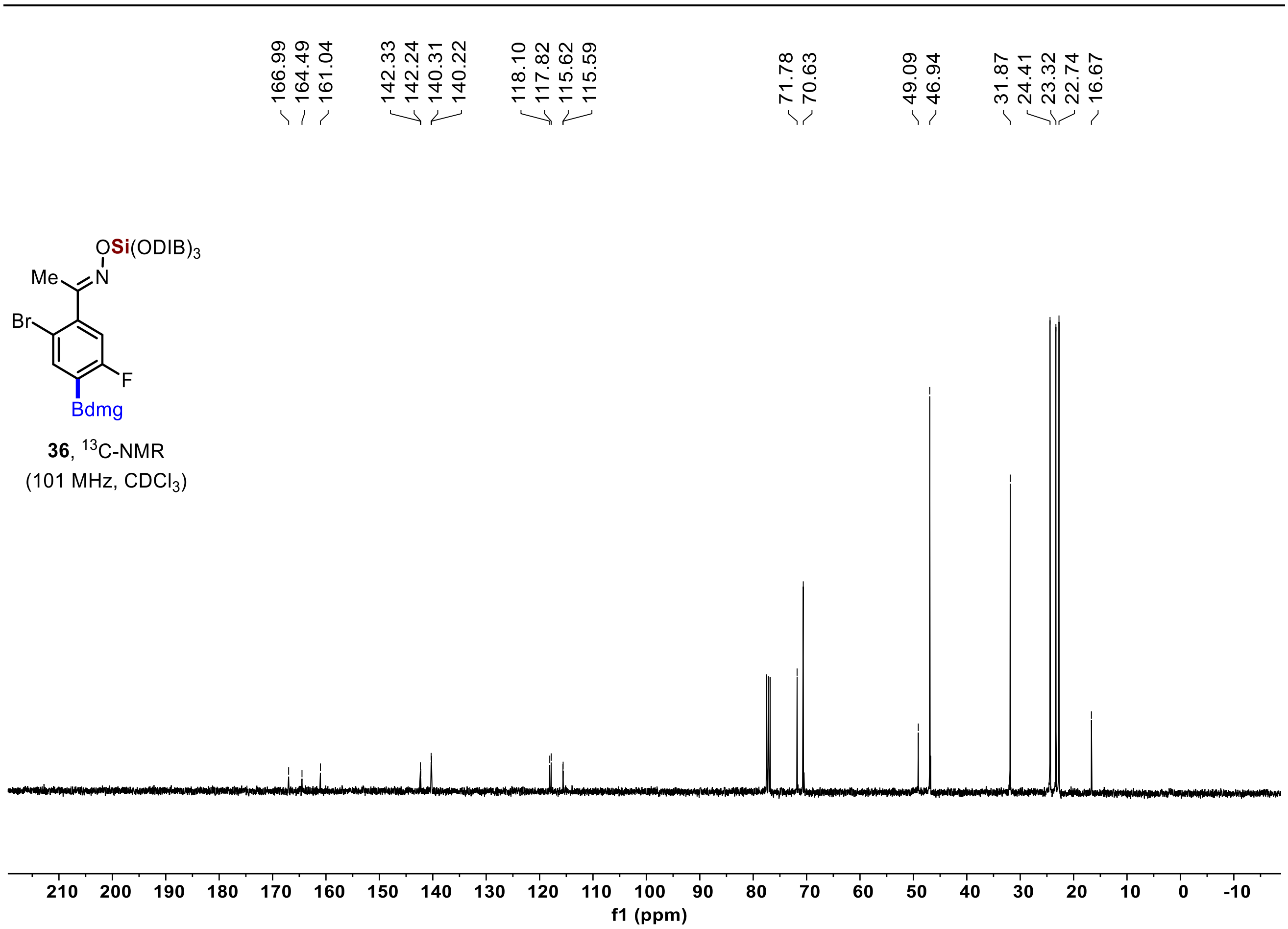
166.99
164.49
161.04
142.33
142.24
140.31
140.22
118.10
117.82
115.62
115.59
71.78
70.63
49.09
46.94
31.87
24.41
23.32
22.74
16.67
OSi(ODIB)3
Me
N
Br
F
Bdmg
36, 13C-NMR
(101 MHz, CDCl3)
210 200 190 180 170 160 150 140 130 120 110 100 90 80 70 60 50 40 30 20 10 0 -10
f1 (ppm)

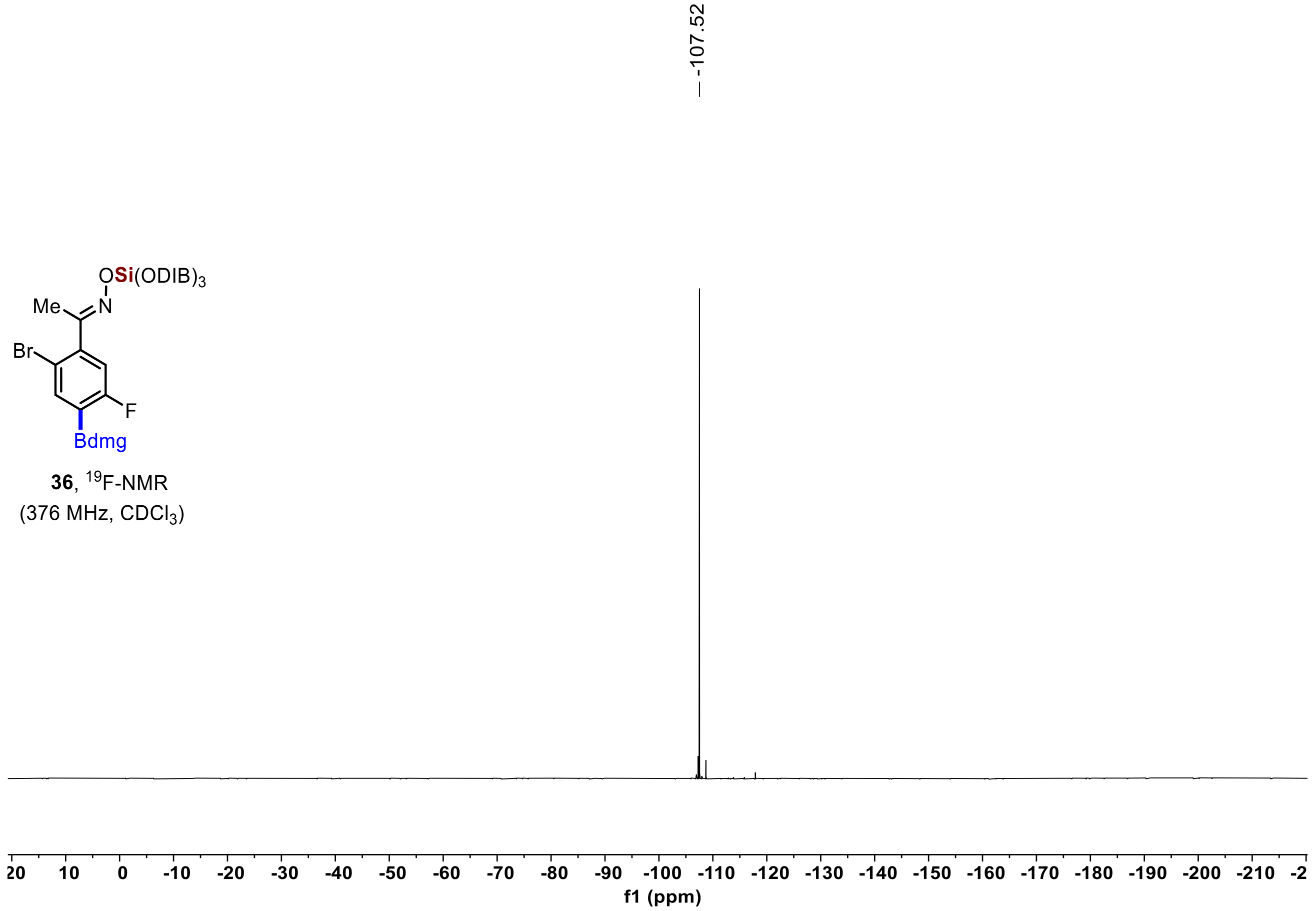
-107.52
OSi(ODIB)3
Me
N
Br
F
Bdmg
36, 19F-NMR
(376 MHz, CDCl3)
20 10 0 -10 -20 -30 -40 -50 -60 -70 -80 -90 -100 -110 -120 -130 -140 -150 -160 -170 -180 -190 -200 -210 -2
f1 (ppm)

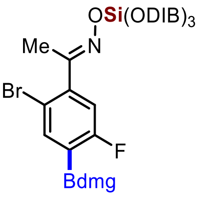

36, ^{11}B-NMR
(128 MHz, $CDCl_3$)

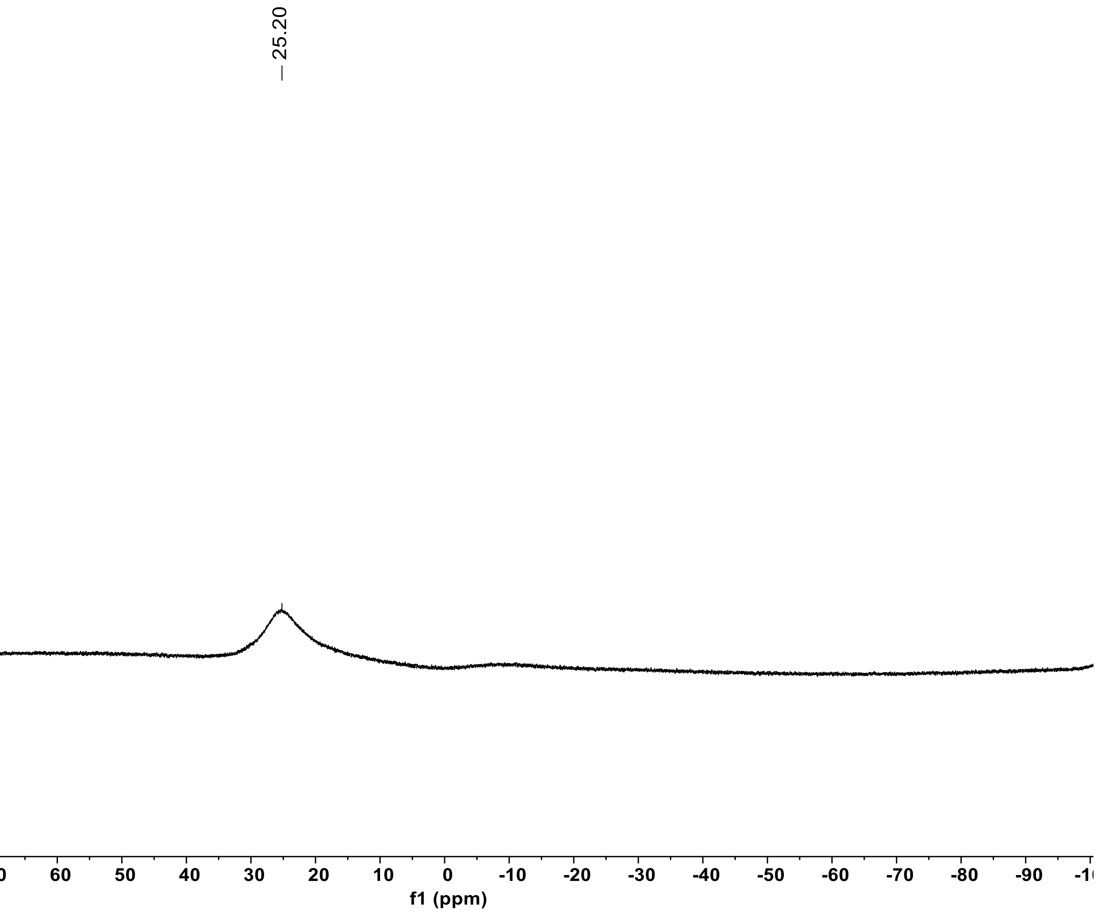

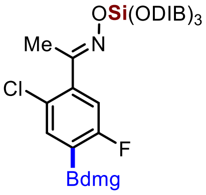

37, ^{1}H-NMR
(400 MHz, $CDCl_3$)

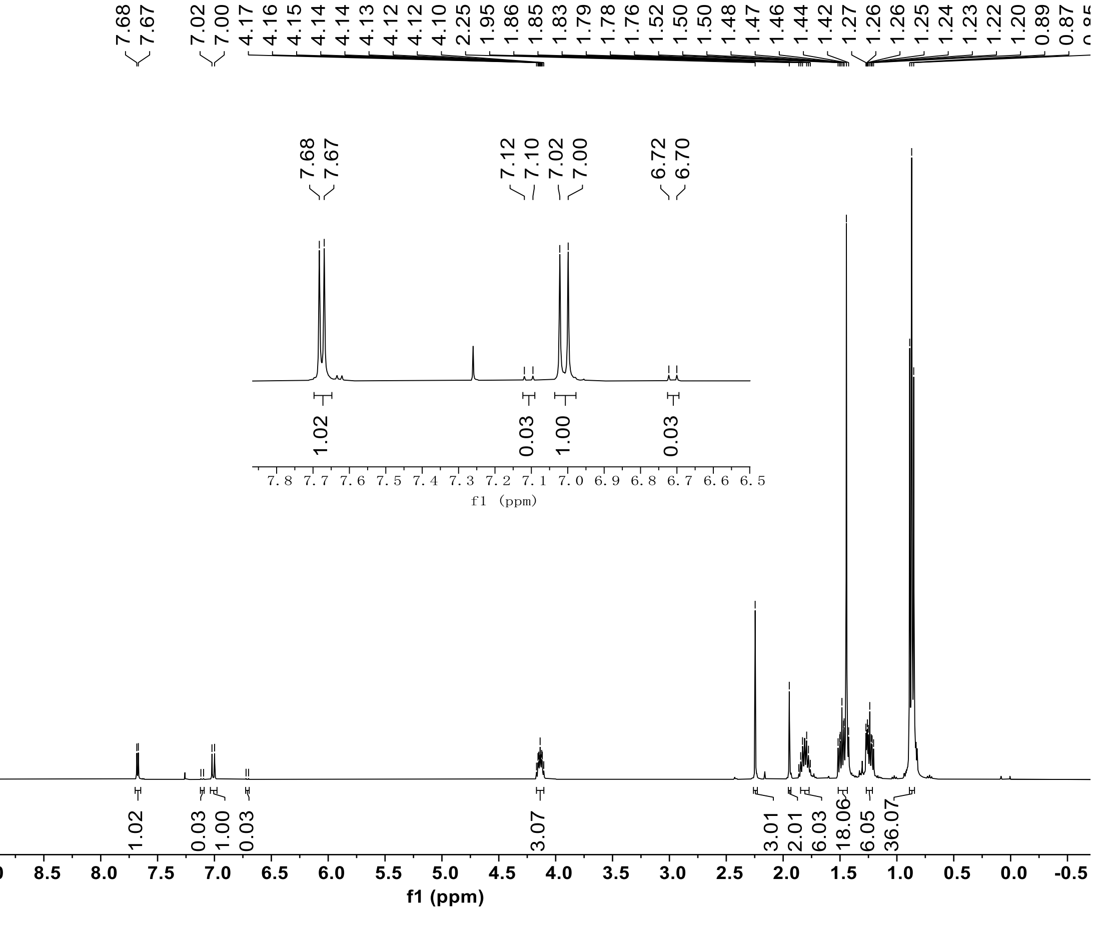

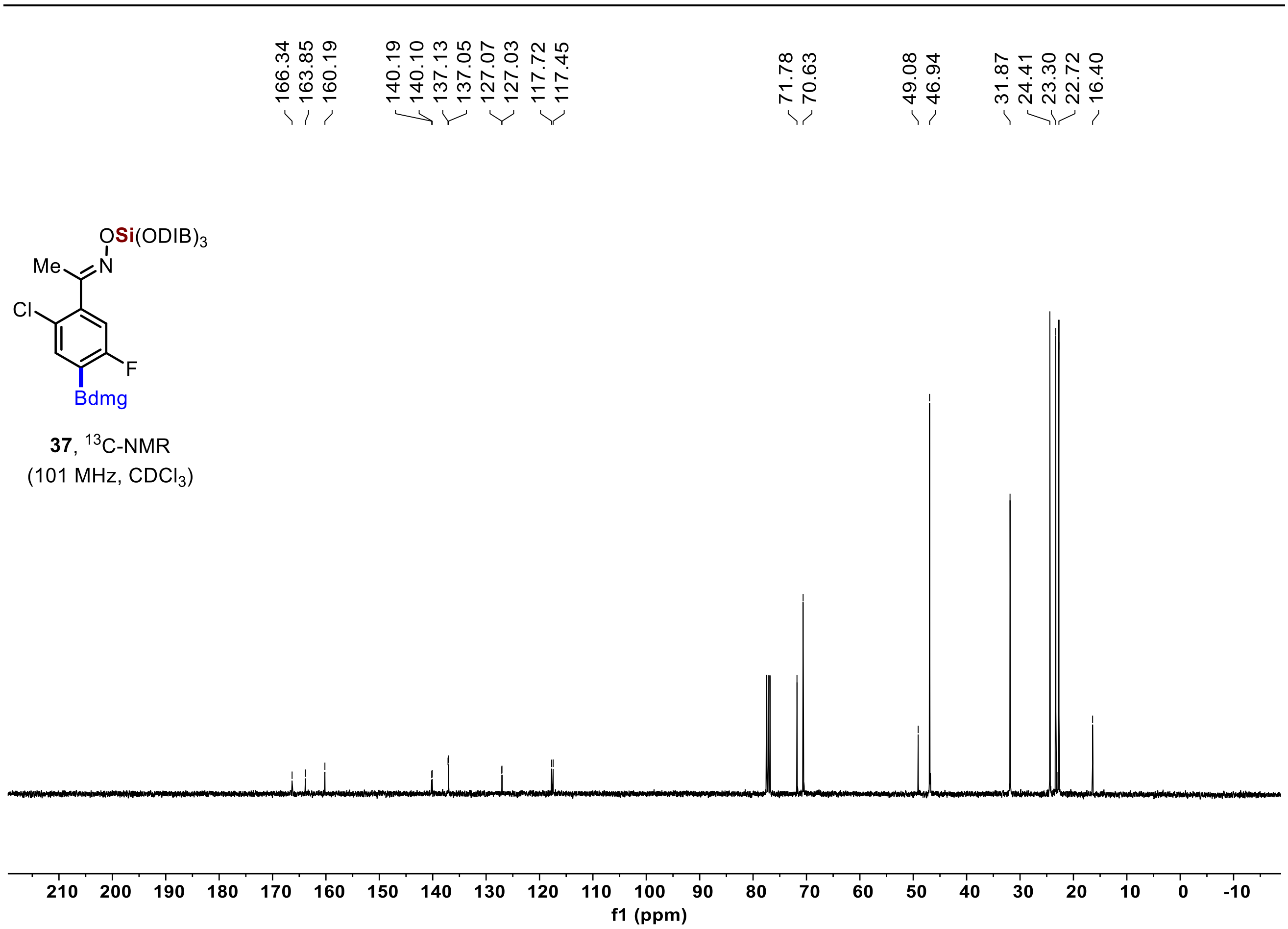
166.34
163.85
160.19
140.19
140.10
137.13
137.05
127.07
127.03
117.72
117.45
71.78
70.63
49.08
46.94
31.87
24.41
23.30
22.72
16.40
OSi(ODIB)3
Me
N
Cl
F
Bdmg
37, 13C-NMR
(101 MHz, CDCl3)
210 200 190 180 170 160 150 140 130 120 110 100 90 80 70 60 50 40 30 20 10 0 -10
f1 (ppm)

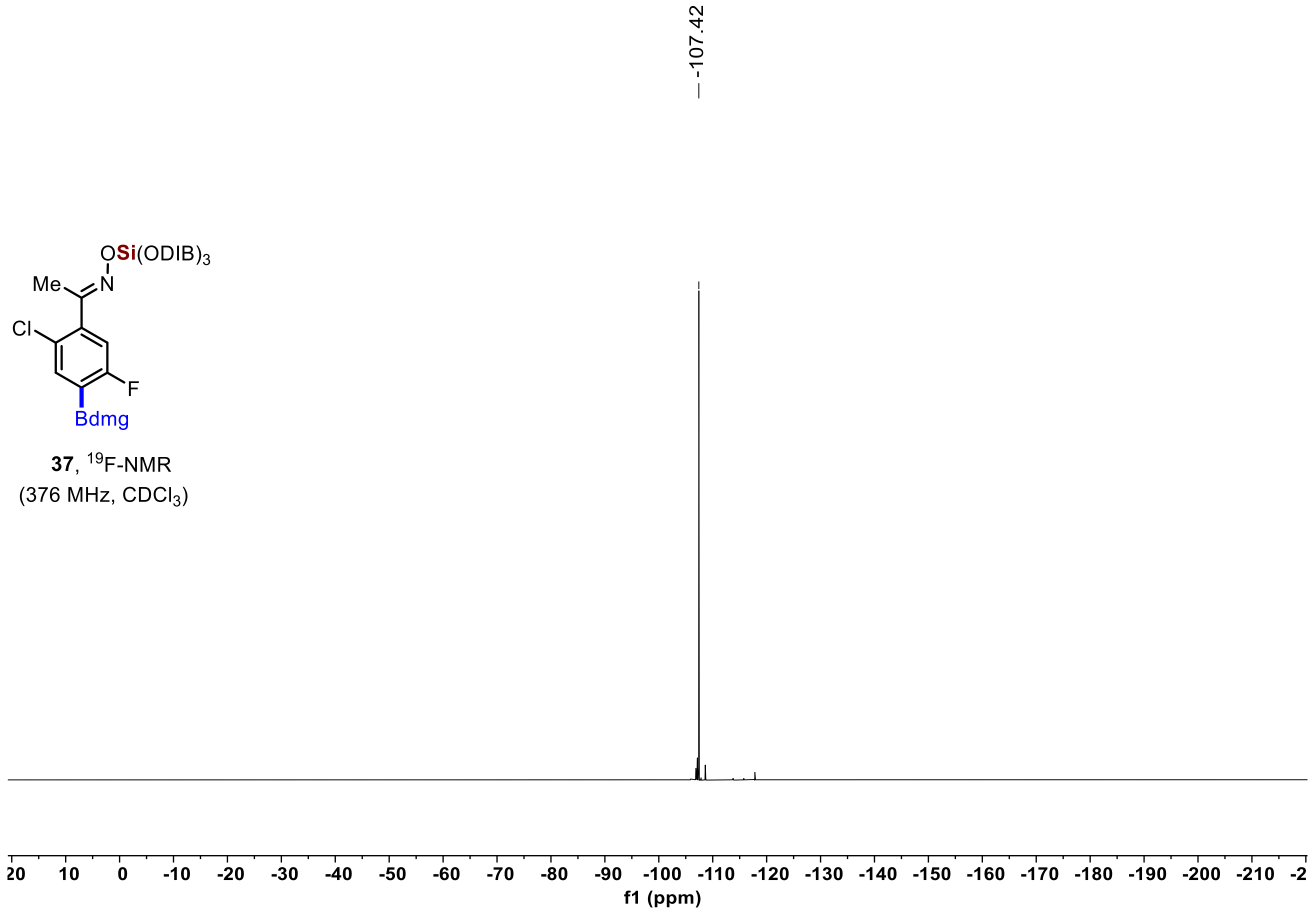
-107.42
OSi(ODIB)3
Me
N
Cl
F
Bdmg
37, 19F-NMR
(376 MHz, CDCl3)
20 10 0 -10 -20 -30 -40 -50 -60 -70 -80 -90 -100 -110 -120 -130 -140 -150 -160 -170 -180 -190 -200 -210
f1 (ppm)

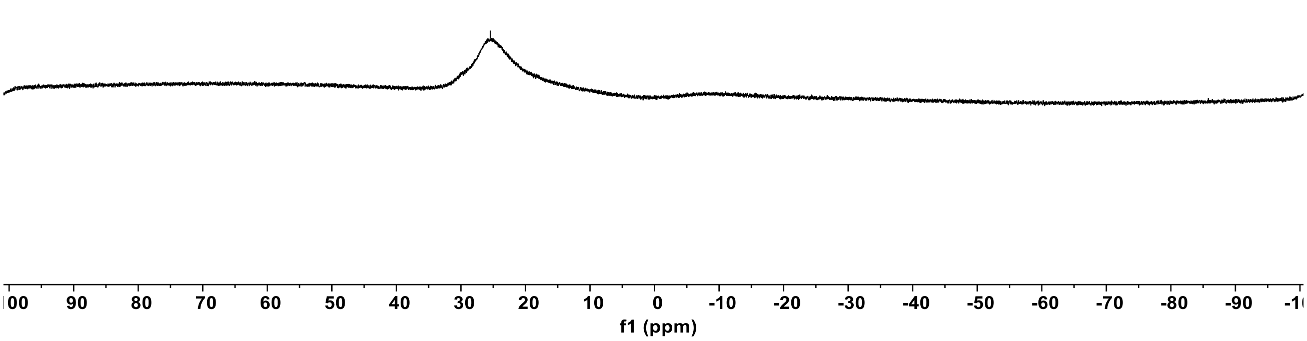

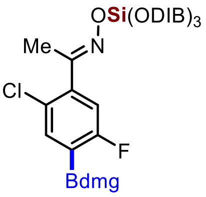

37, ^{11}B-NMR
(128 MHz, $CDCl_3$)

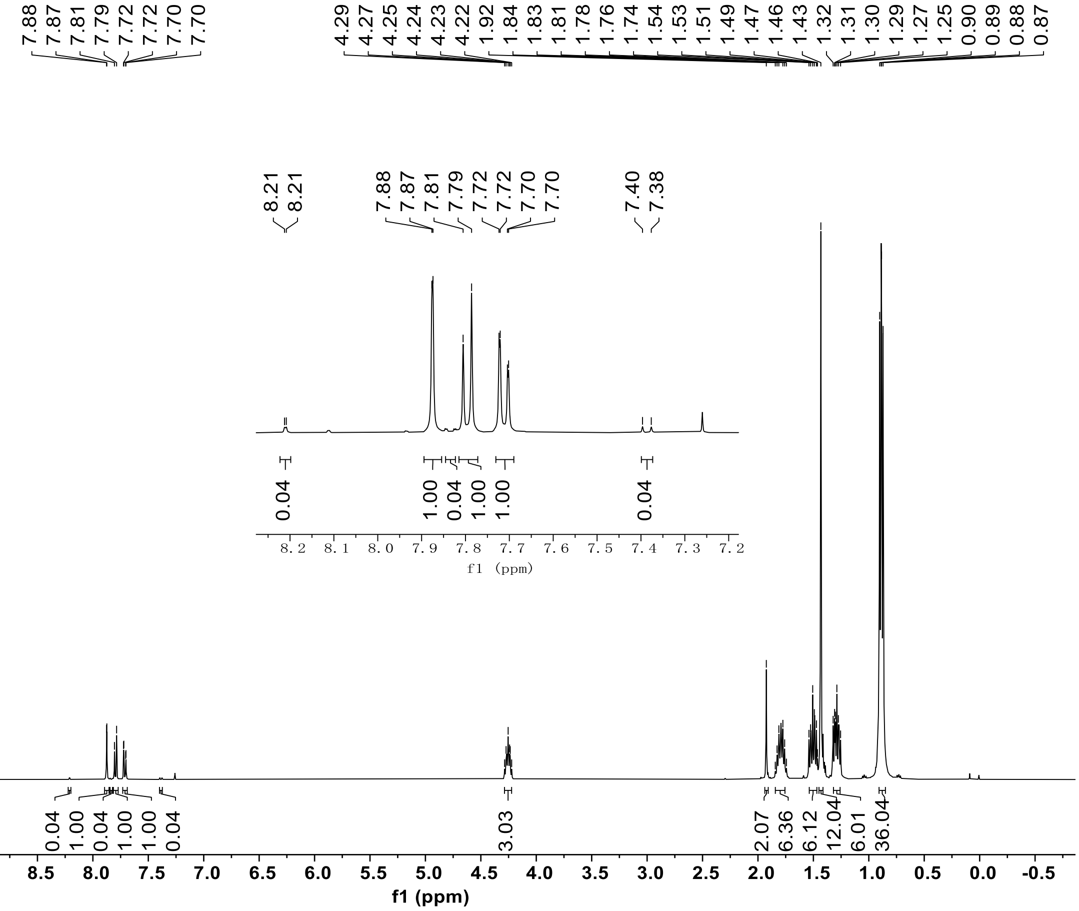

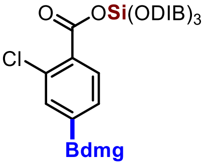

39, ^{1}H-NMR
(400 MHz, $CDCl_3$)

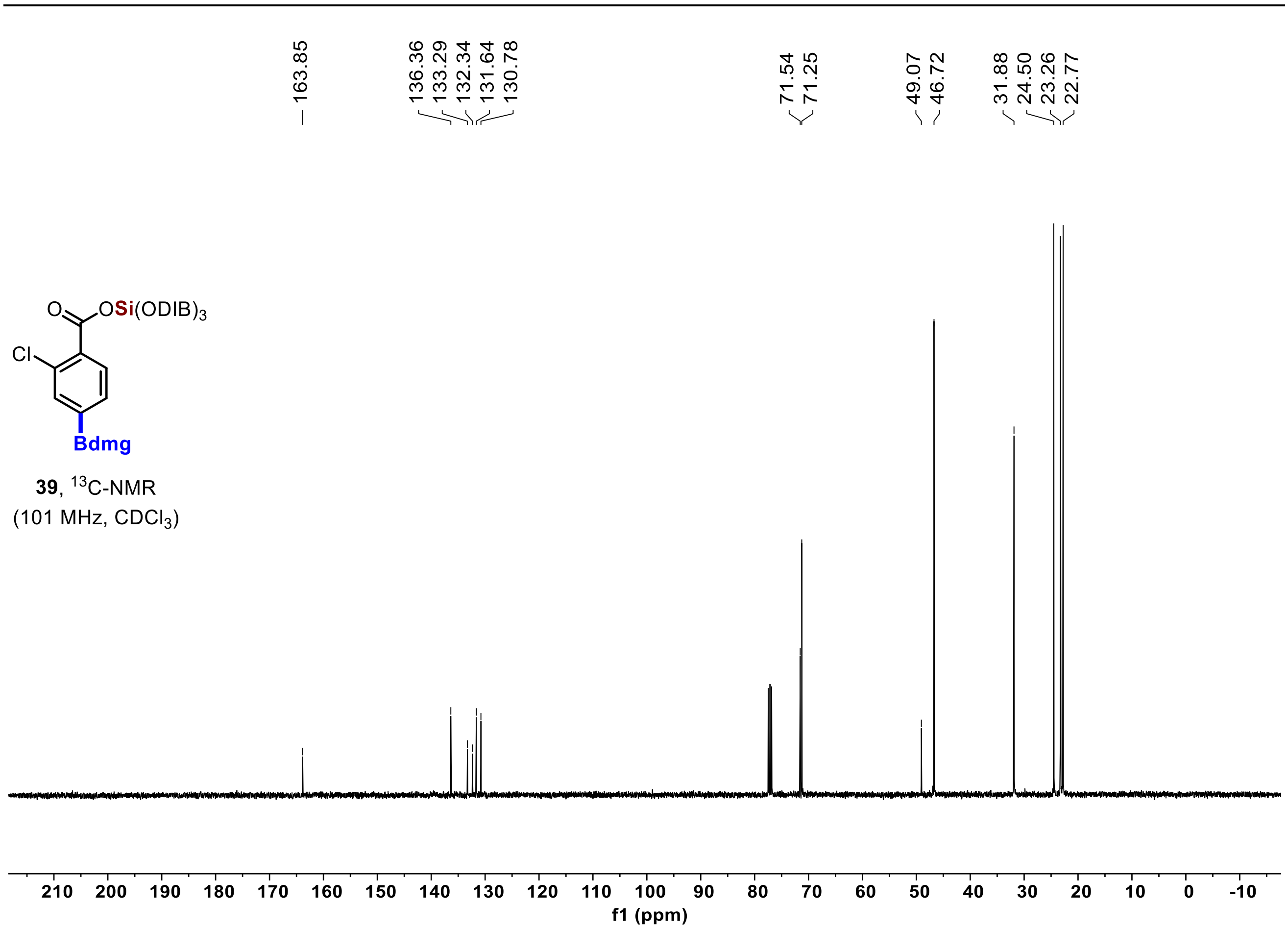
163.85
136.36
133.29
132.34
131.64
130.78
71.54
71.25
49.07
46.72
31.88
24.50
23.26
22.77
O
OSi(ODIB)3
Cl
Bdmg
39, 13C-NMR
(101 MHz, CDCl3)
210 200 190 180 170 160 150 140 130 120 110 100 90 80 70 60 50 40 30 20 10 0 -10
f1 (ppm)

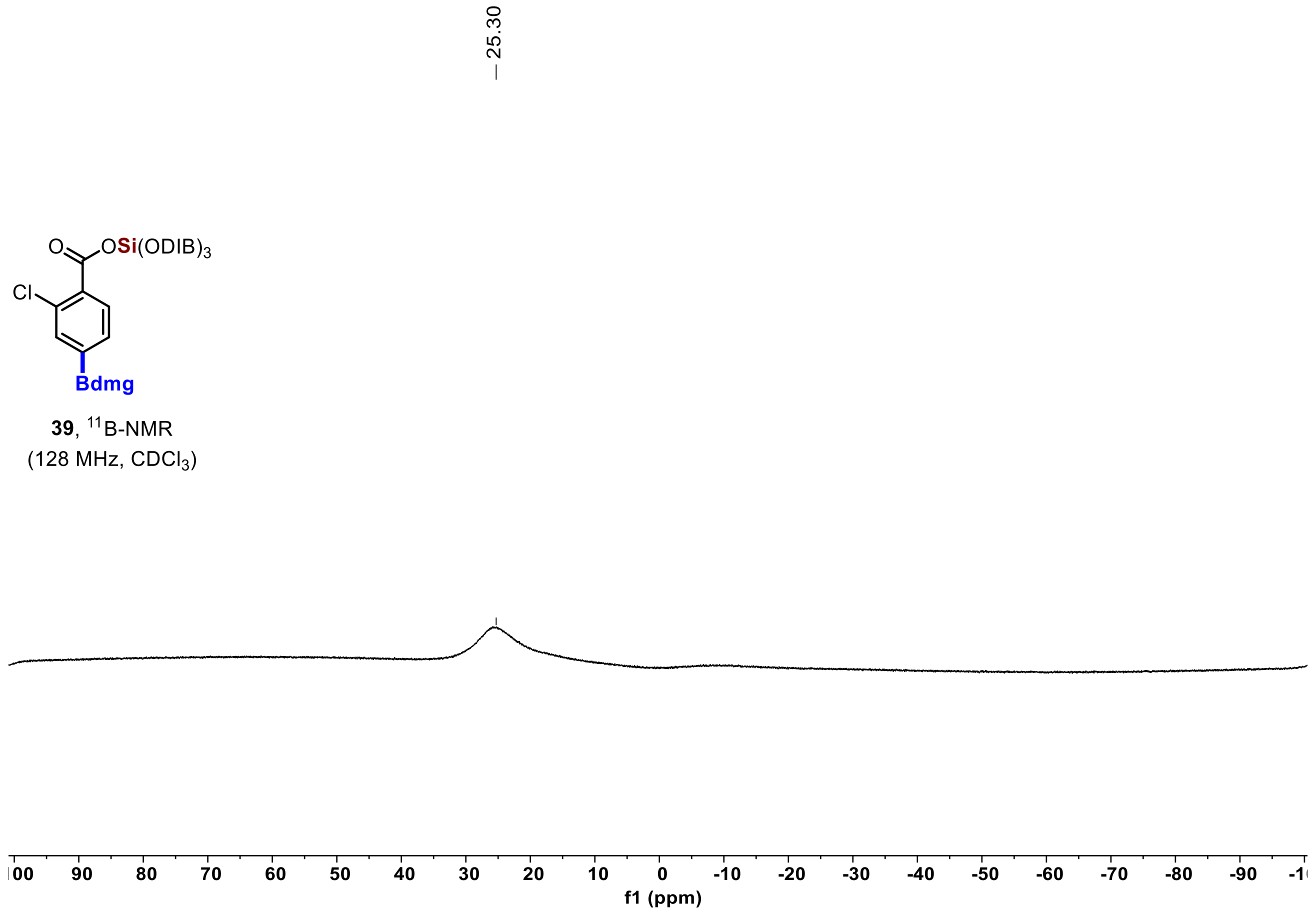
25.30
O
OSi(ODIB)3
Cl
Bdmg
39, 11B-NMR
(128 MHz, CDCl3)
90 80 70 60 50 40 30 20 10 0 -10 -20 -30 -40 -50 -60 -70 -80 -90
f1 (ppm)

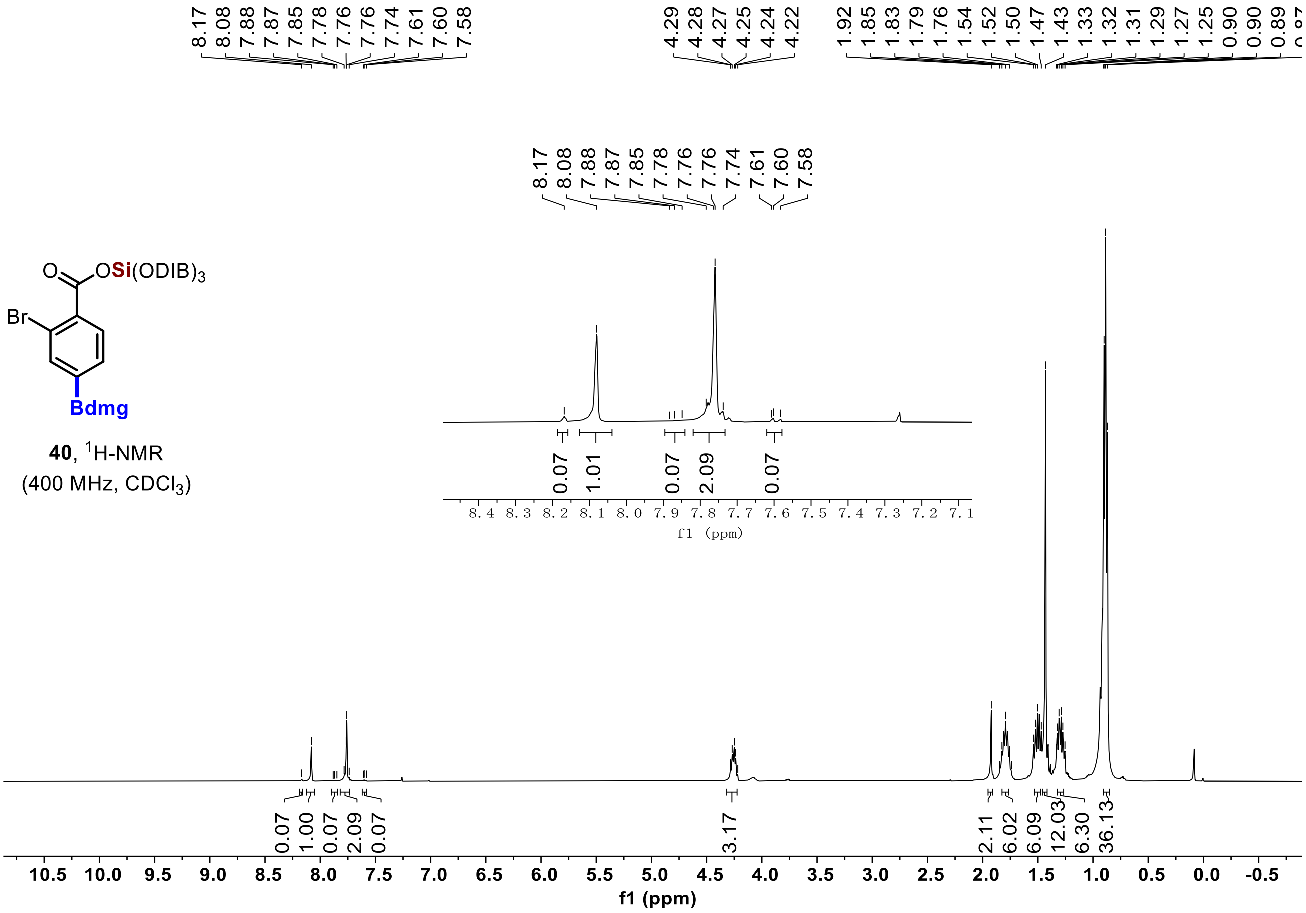

40, ^{1}H-NMR (400 MHz, $CDCl_3$)

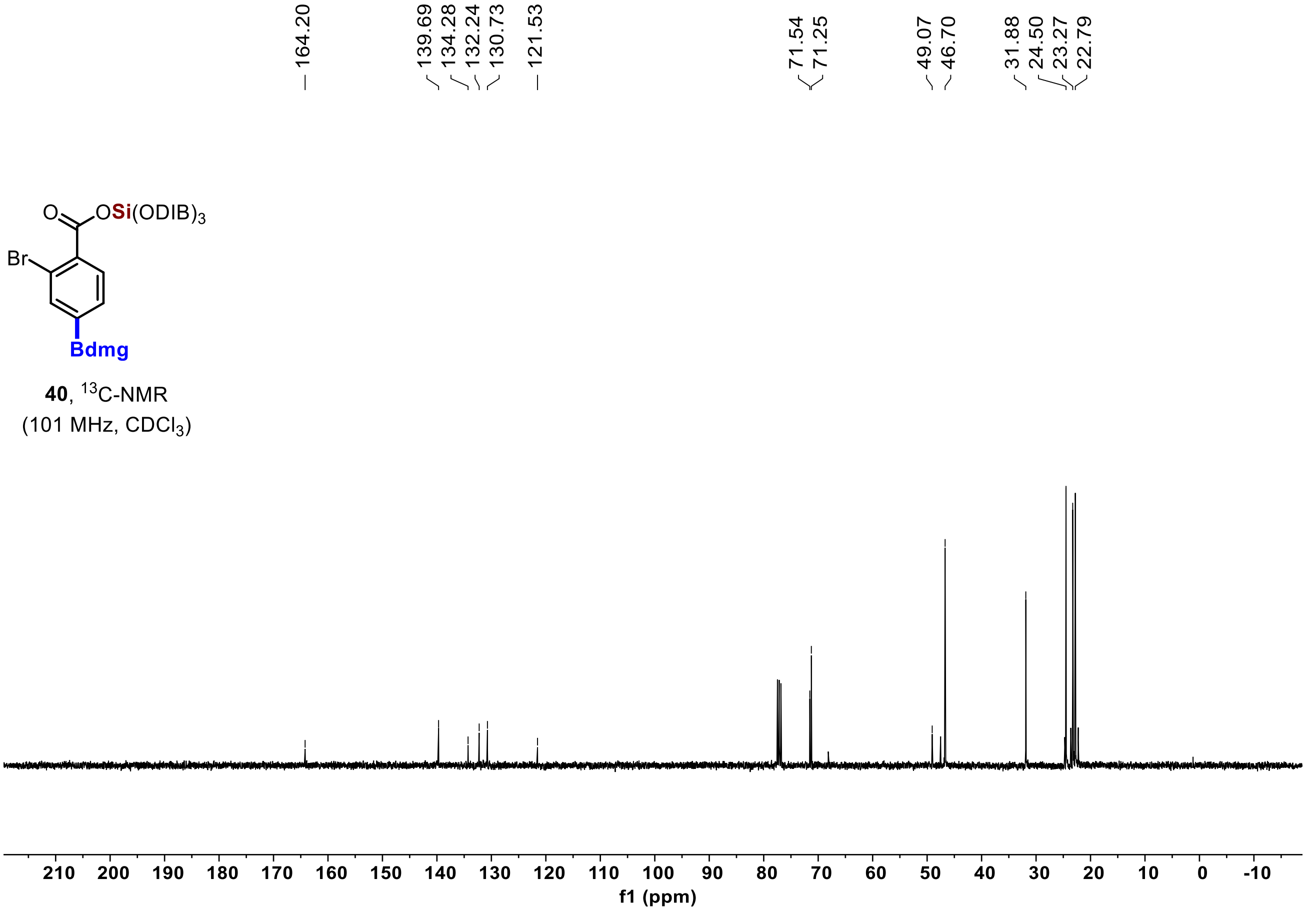

40, ^{13}C-NMR (101 MHz, $CDCl_3$)

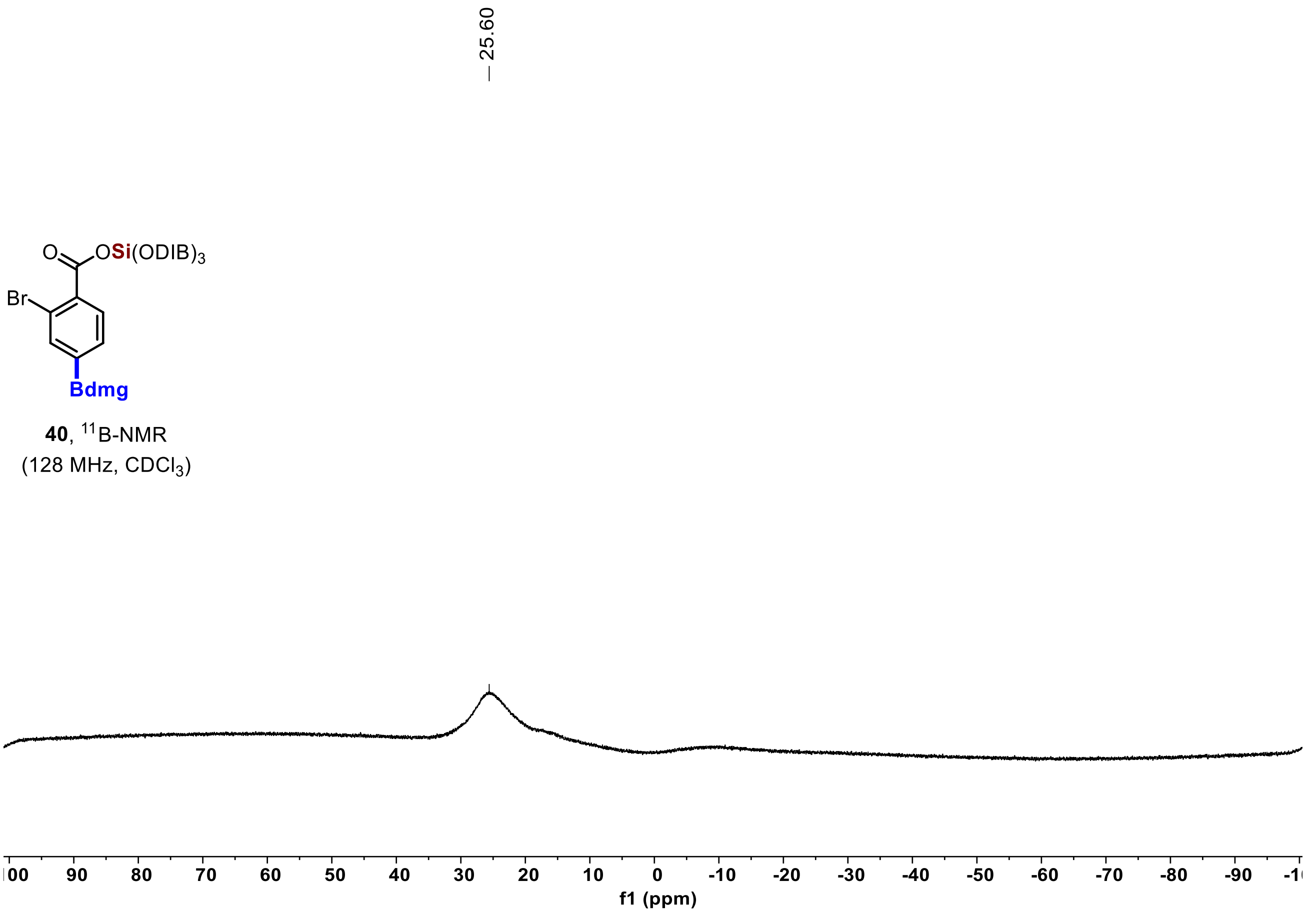

40, ^{11}B-NMR
(128 MHz, $CDCl_3$)

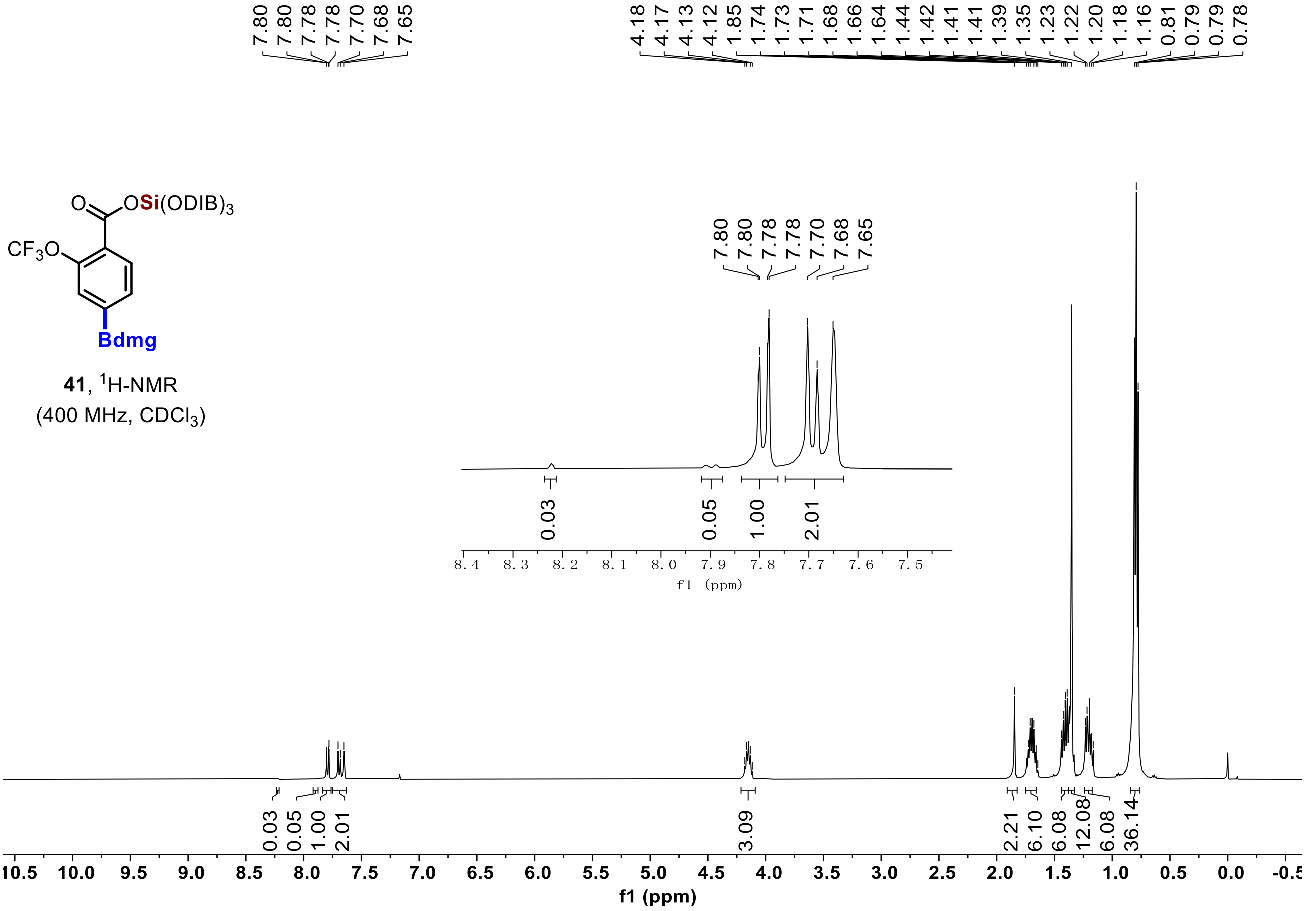

41, ^{1}H-NMR
(400 MHz, $CDCl_3$)

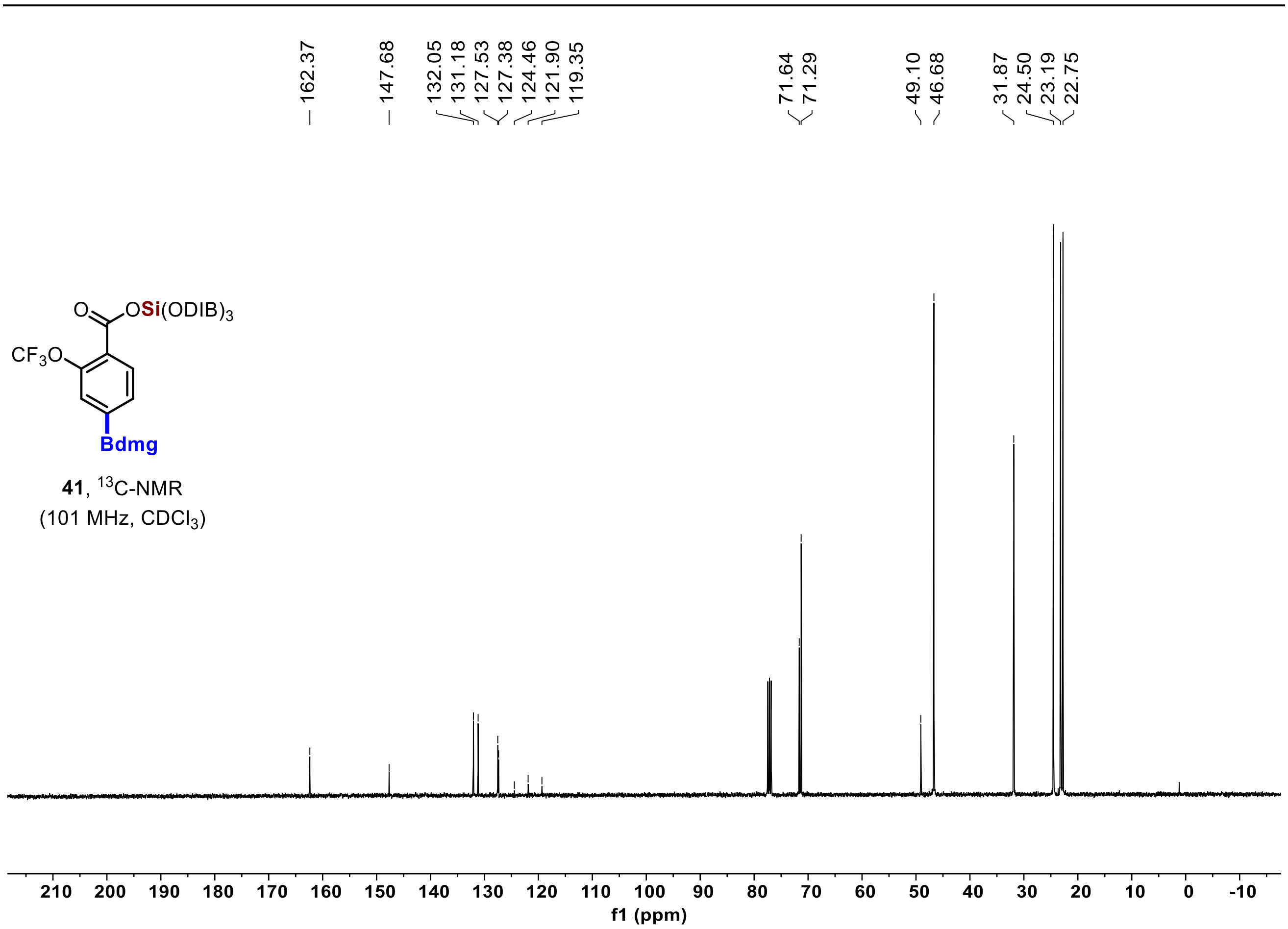
162.37
147.68
132.05
131.18
127.53
127.38
124.46
121.90
119.35
71.64
71.29
49.10
46.68
31.87
24.50
23.19
22.75
O
OSi(ODIB)3
CF3O
Bdmg
41, 13C-NMR
(101 MHz, CDCl3)
210 200 190 180 170 160 150 140 130 120 110 100 90 80 70 60 50 40 30 20 10 0 -10
f1 (ppm)

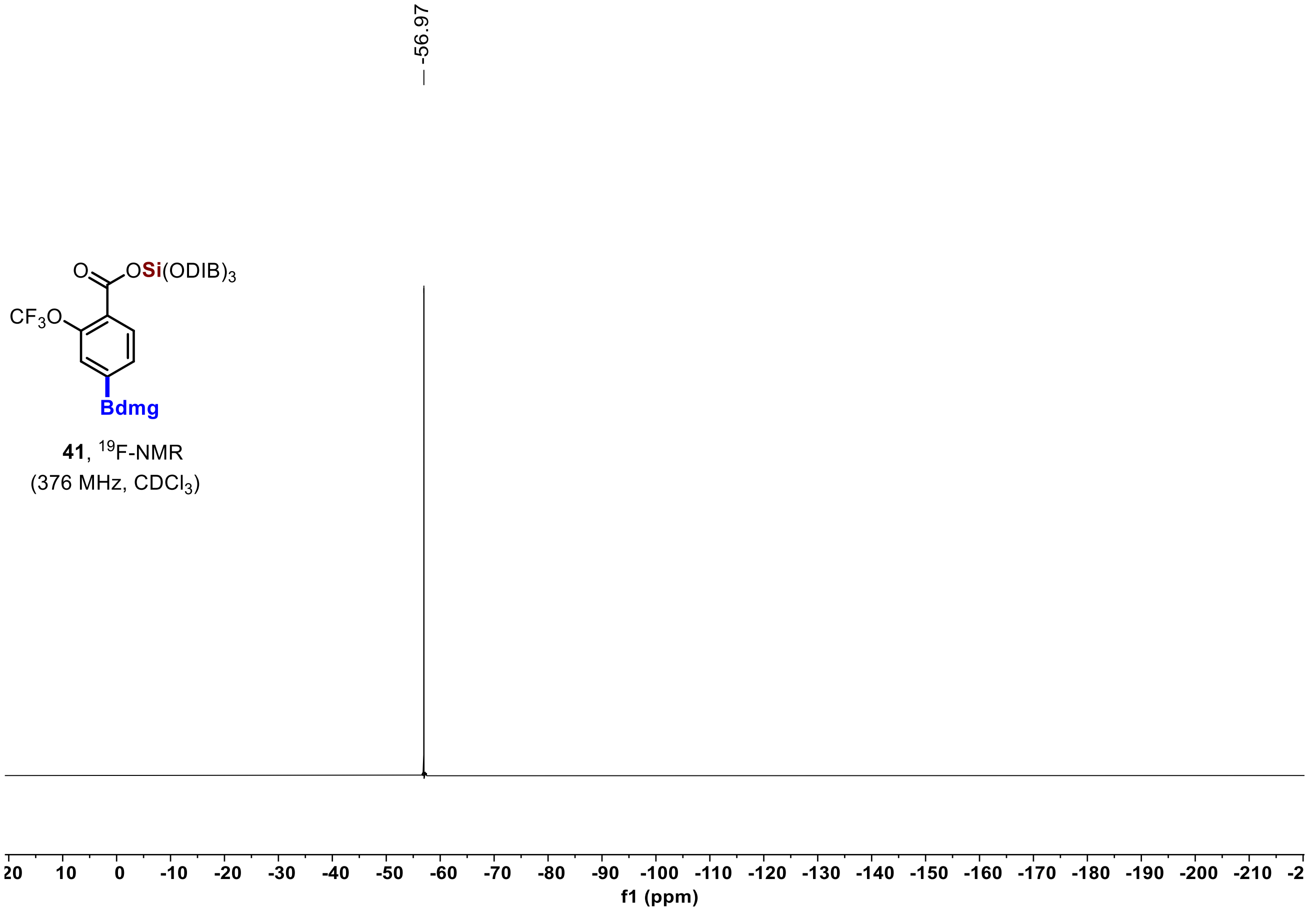
-56.97
O
OSi(ODIB)3
CF3O
Bdmg
41, 19F-NMR
(376 MHz, CDCl3)
20 10 0 -10 -20 -30 -40 -50 -60 -70 -80 -90 -100 -110 -120 -130 -140 -150 -160 -170 -180 -190 -200 -210 -2
f1 (ppm)

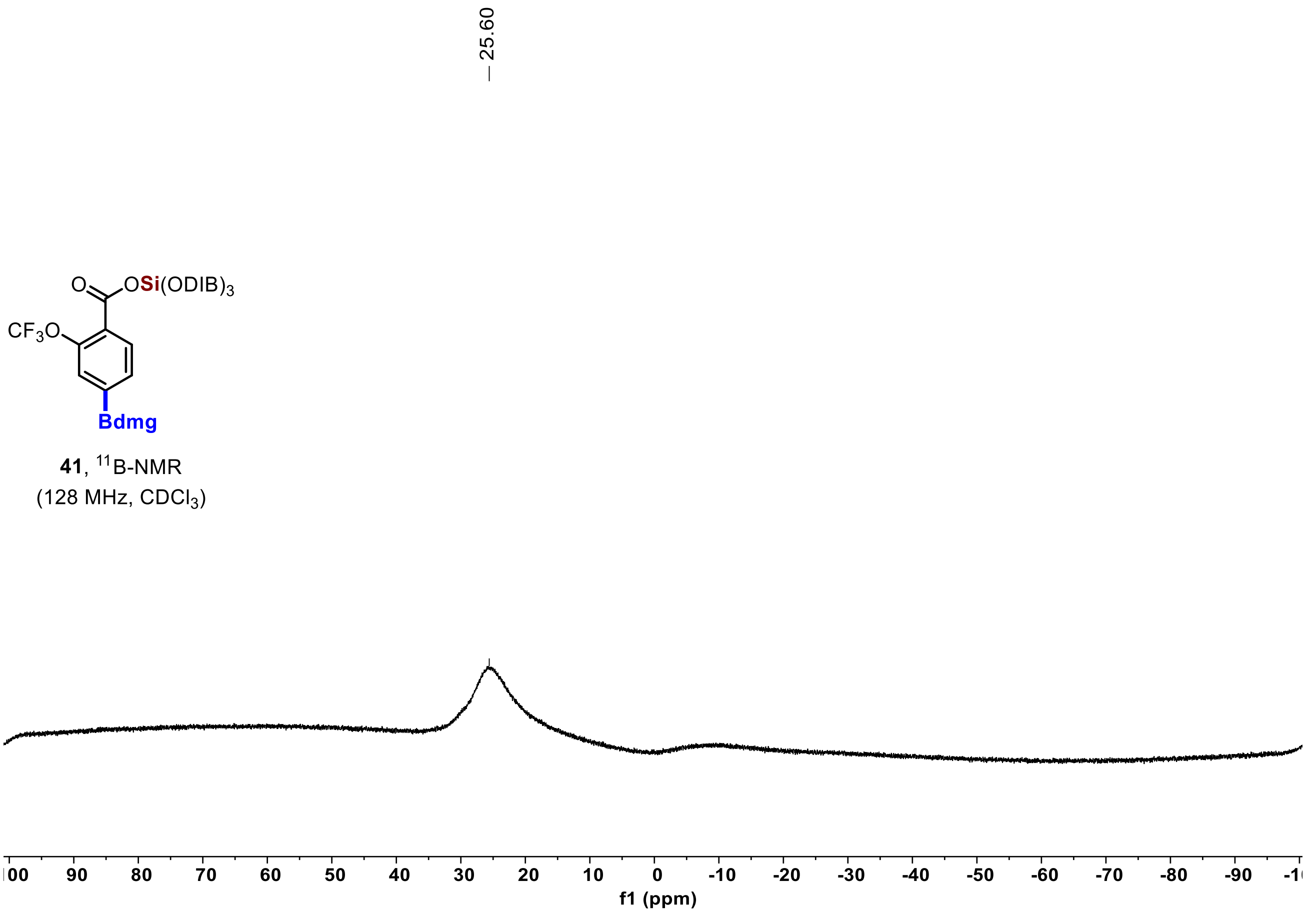

41, ^{11}B-NMR
(128 MHz, $CDCl_3$)

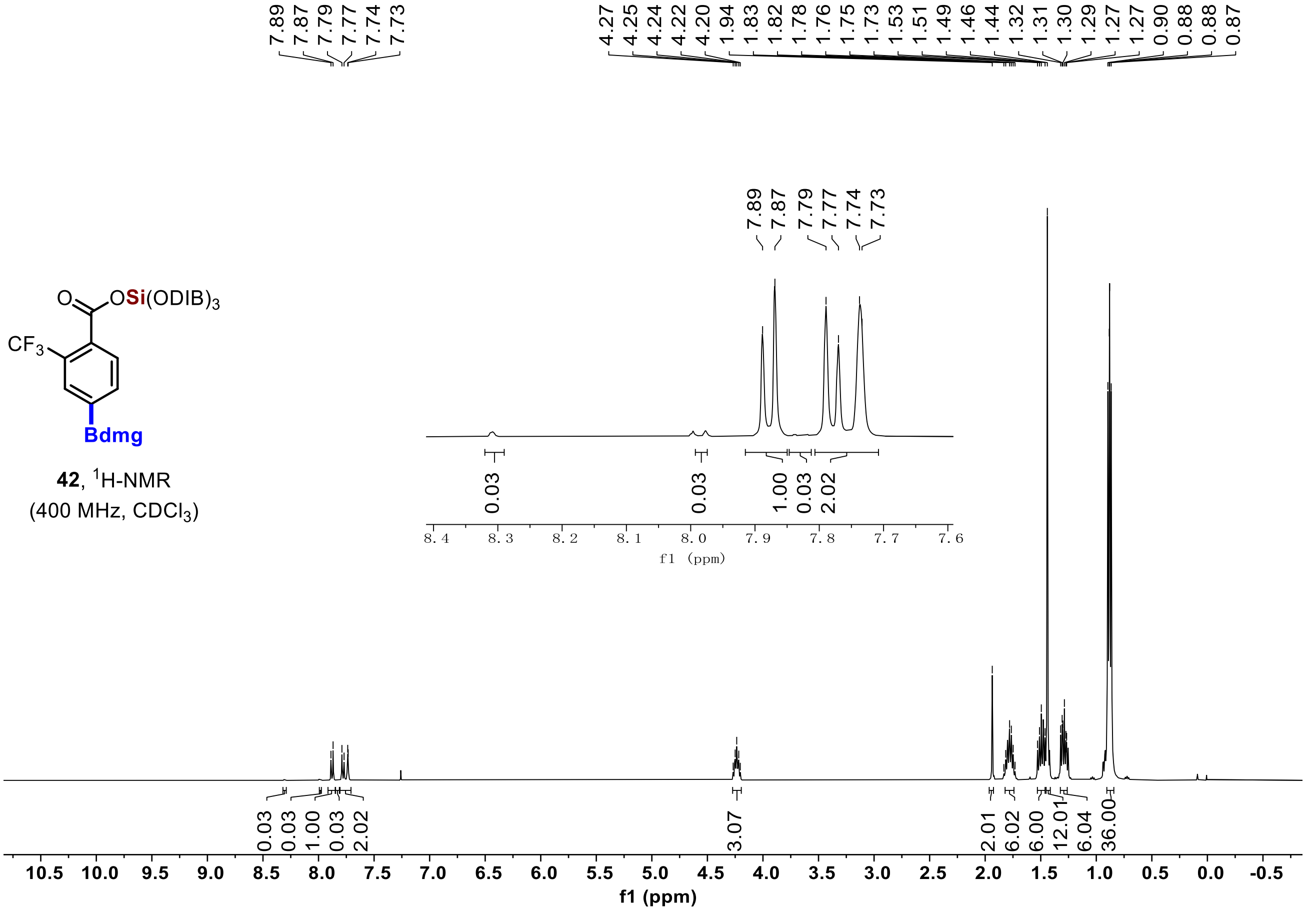

42, ^{1}H-NMR
(400 MHz, $CDCl_3$)

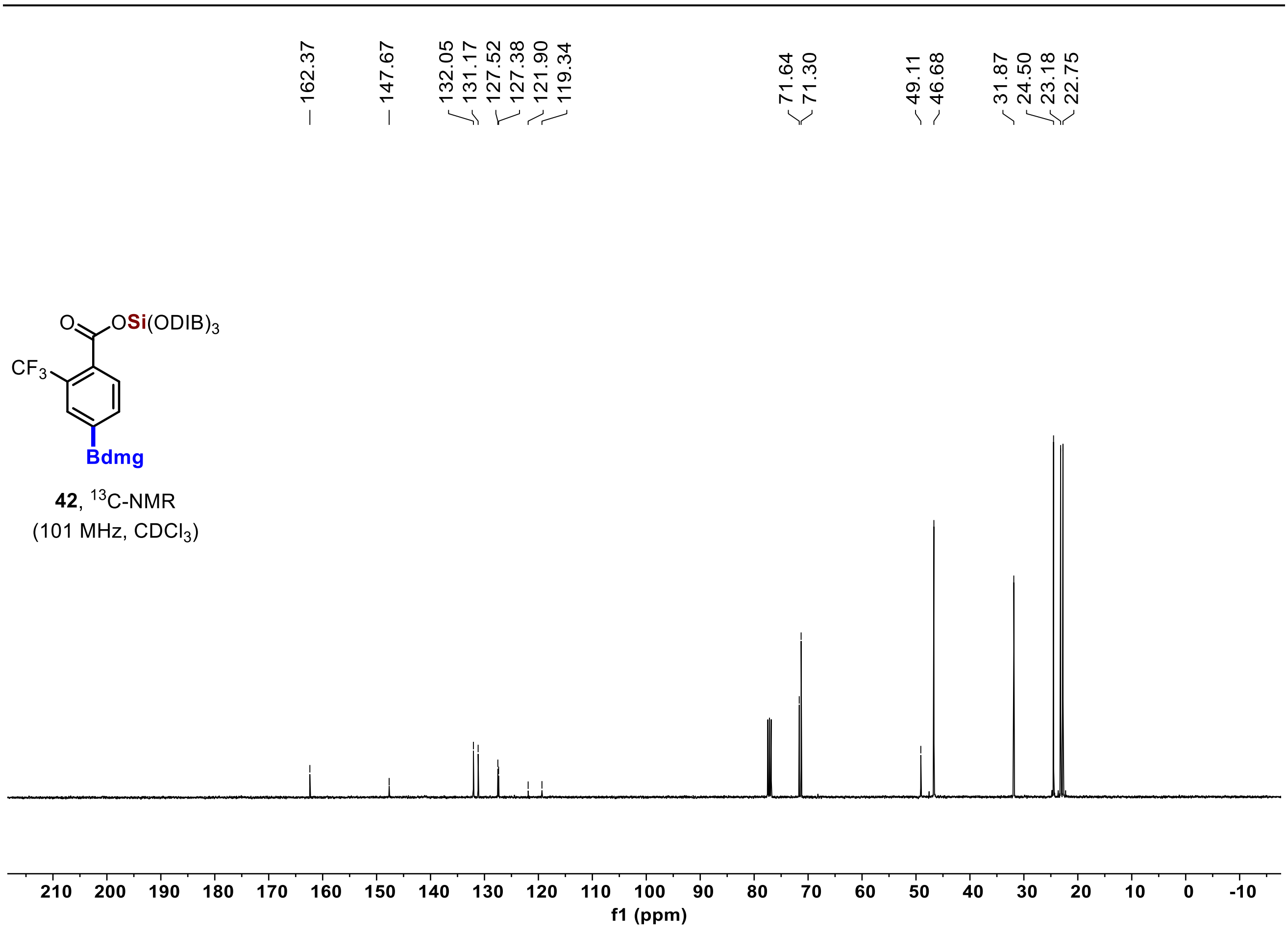

42, ¹³C-NMR
(101 MHz, $CDCl_3$)

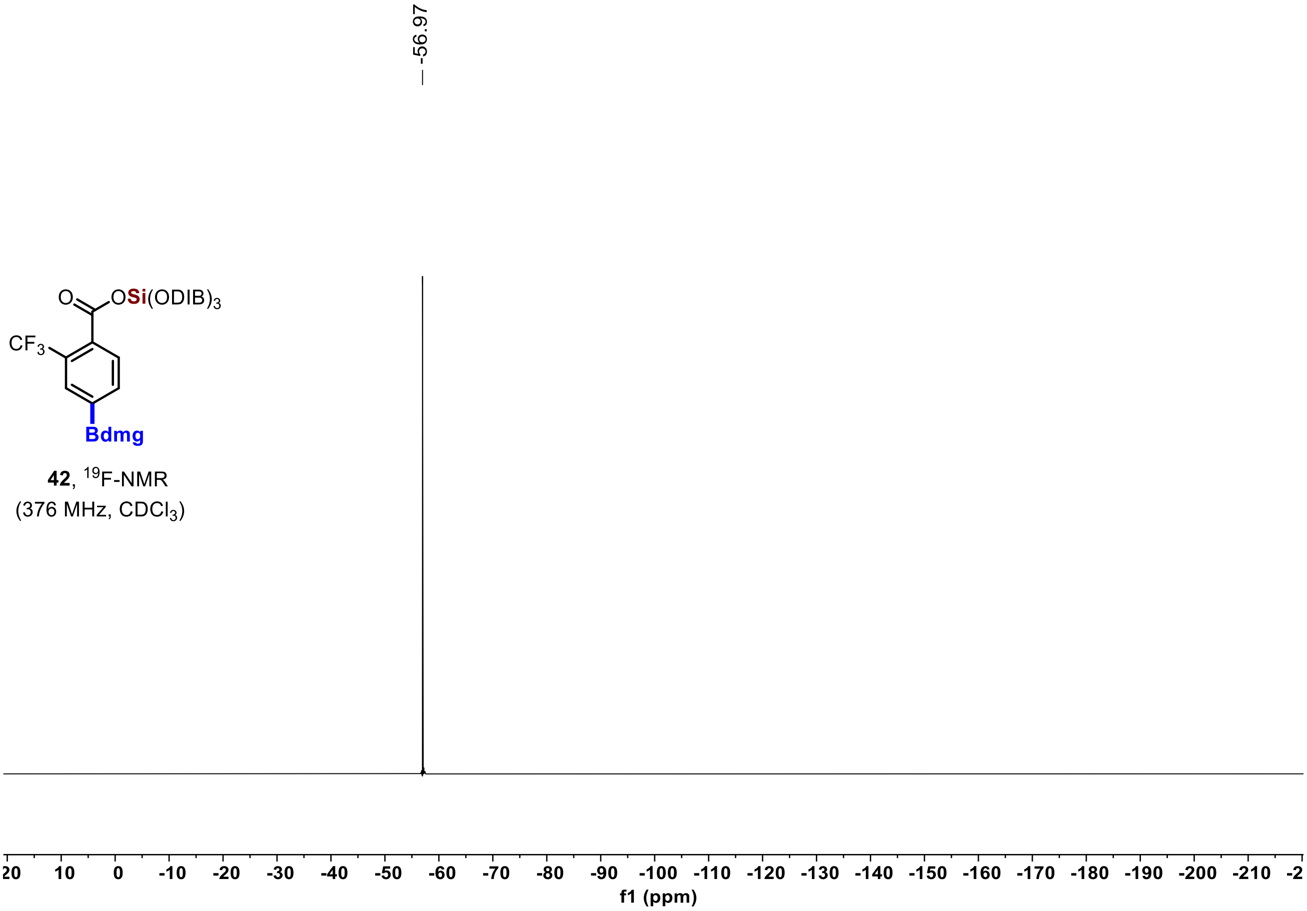

42, ¹⁹F-NMR
(376 MHz, $CDCl_3$)

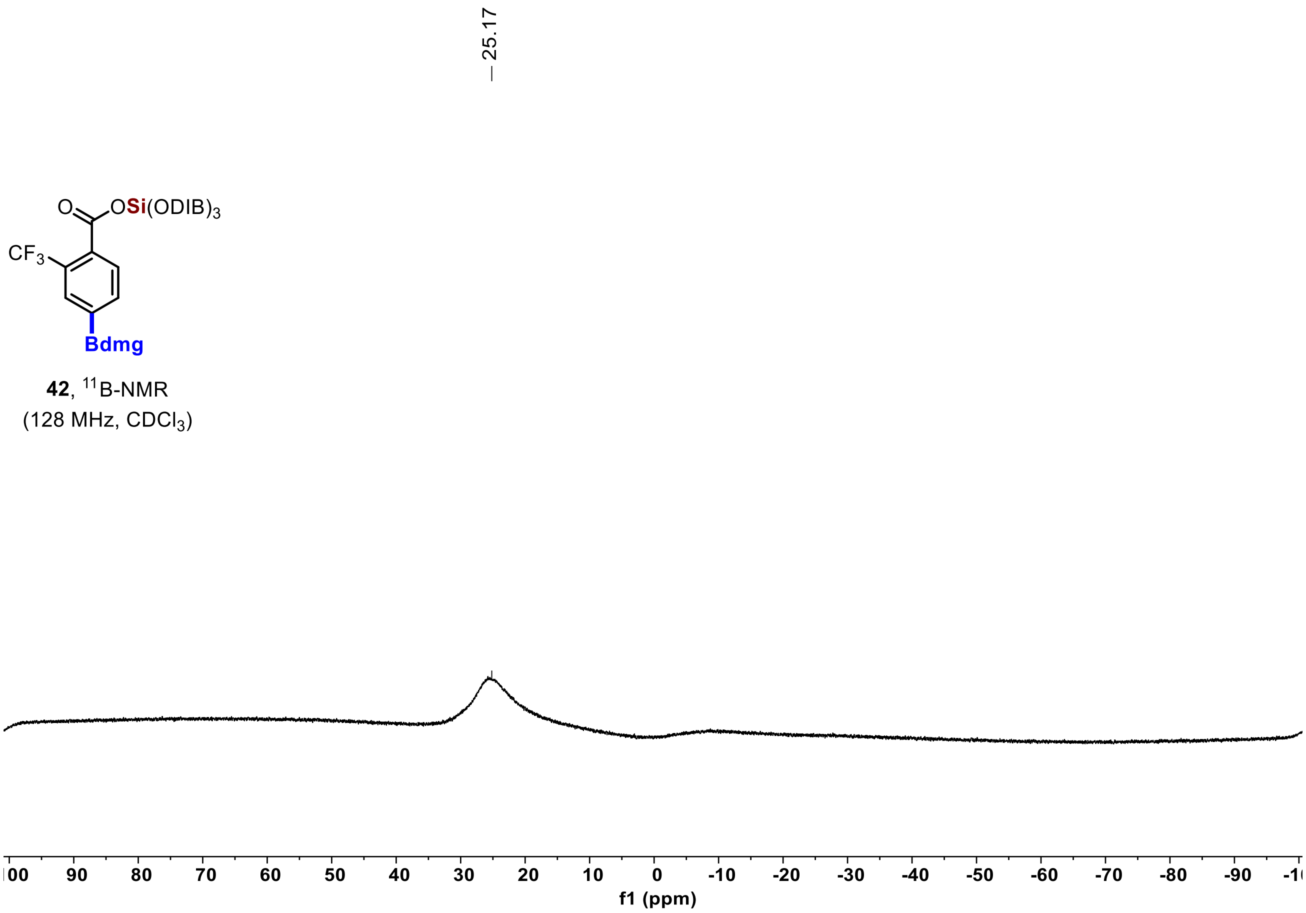
25.17
OSi(ODIB)3
CF3
Bdmg
42, 11B-NMR
(128 MHz, CDCl3)
f1 (ppm)

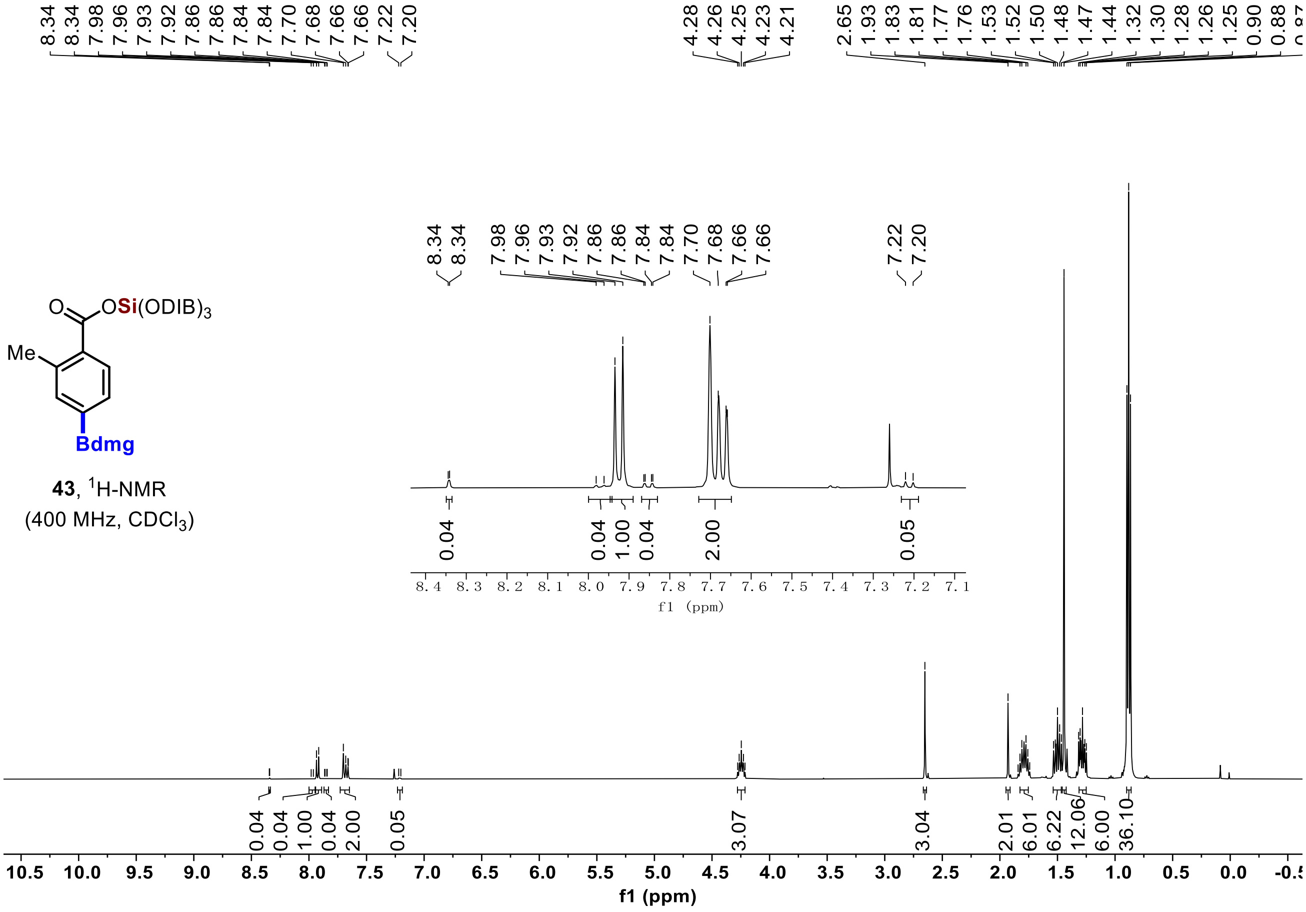
8.34 8.34 7.98 7.96 7.93 7.92 7.86 7.86 7.84 7.84 7.70 7.68 7.66 7.66 7.22 7.20
4.28 4.26 4.25 4.23 4.21
2.65 1.93 1.83 1.81 1.77 1.76 1.53 1.52 1.50 1.48 1.47 1.44 1.32 1.30 1.28 1.26 1.25 0.90 0.88
OSi(ODIB)3
Me
Bdmg
43, 1H-NMR
(400 MHz, CDCl3)
0.04 0.04 1.00 0.04 2.00 0.05 3.07 3.04 2.01 6.01 6.22 12.06 6.00 36.10
f1 (ppm)

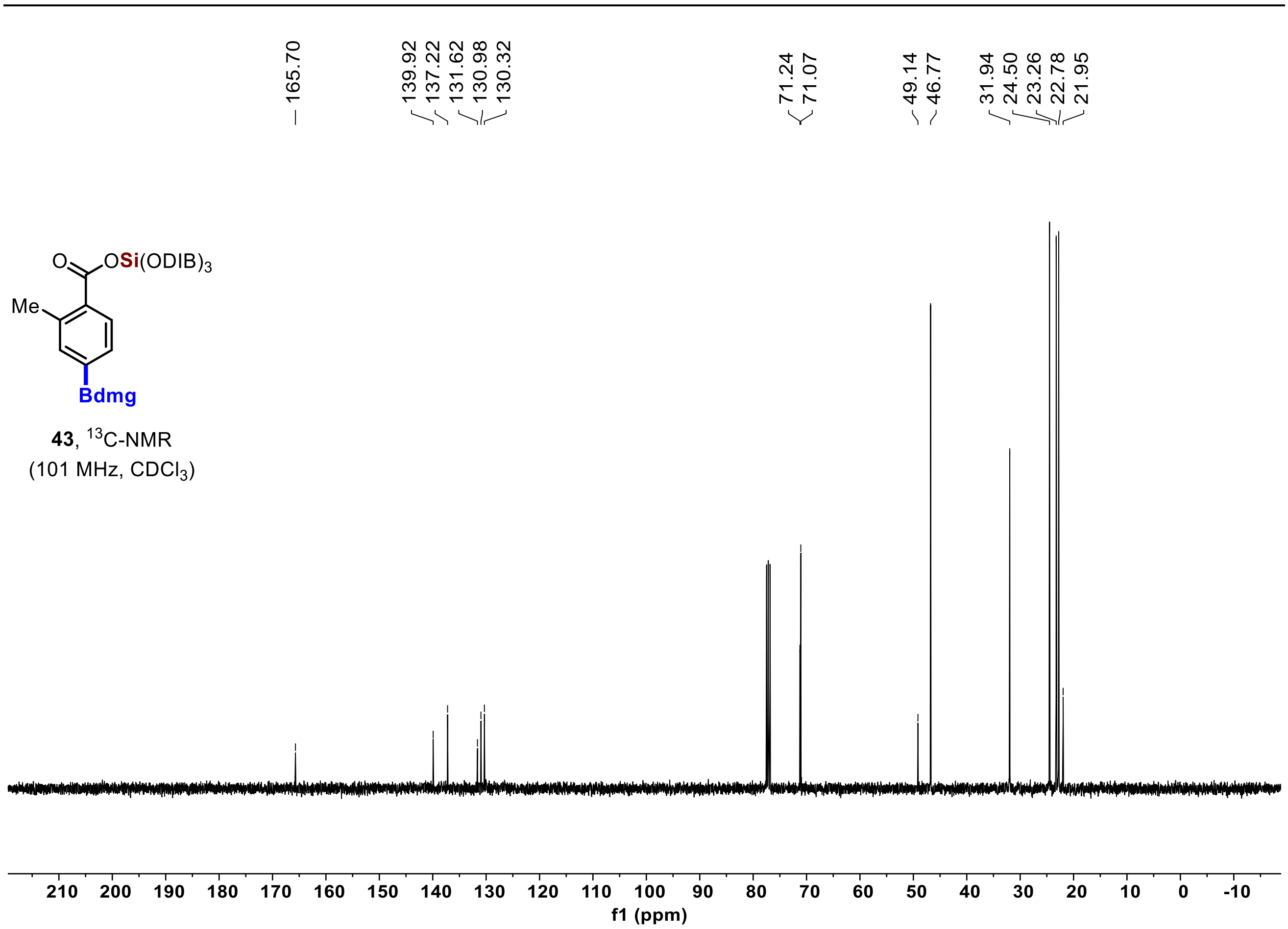
165.70
139.92
137.22
131.62
130.98
130.32
71.24
71.07
49.14
46.77
31.94
24.50
23.26
22.78
21.95
OSi(ODIB)3
Me
Bdmg
43, 13C-NMR
(101 MHz, CDCl3)
f1 (ppm)

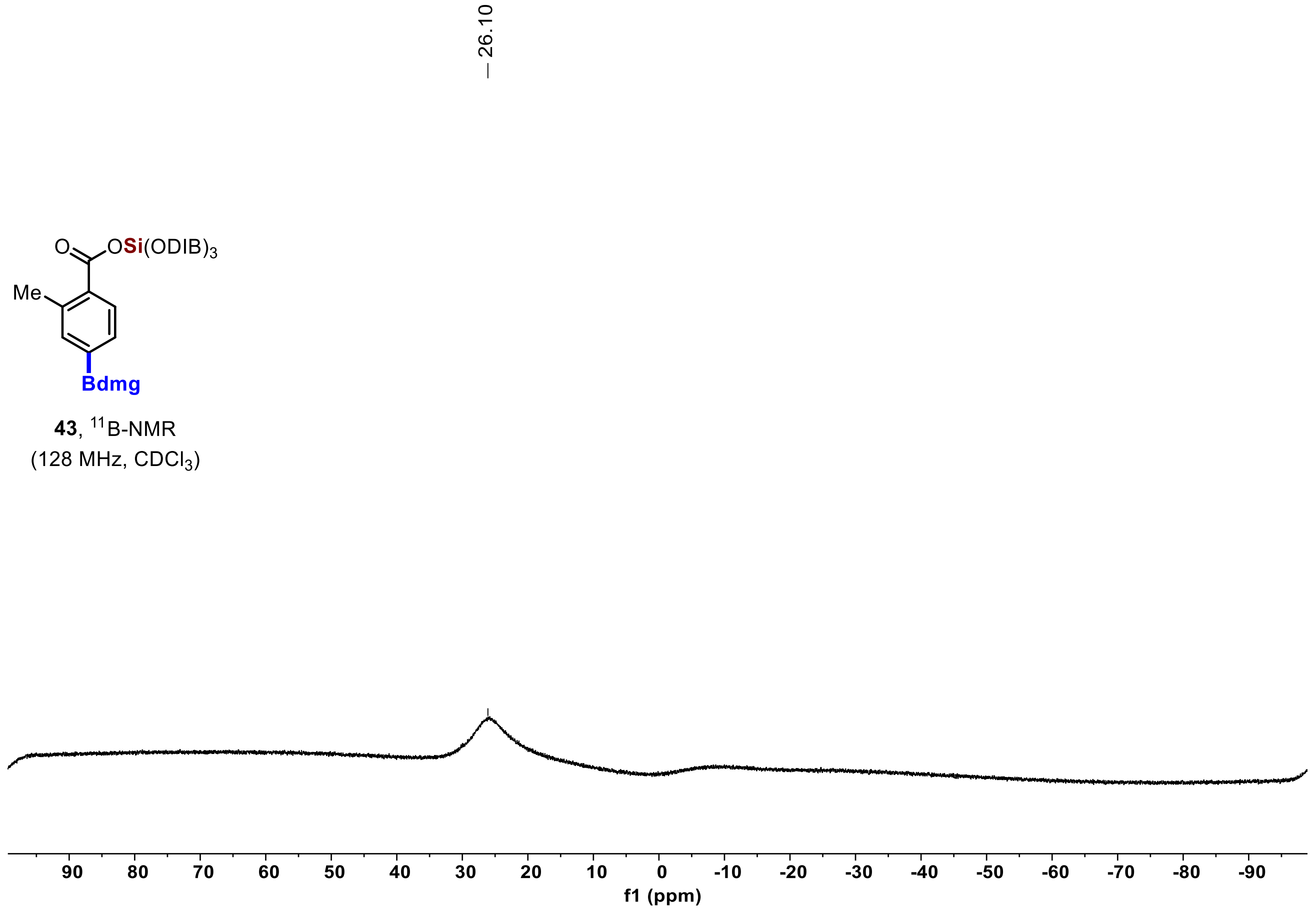
26.10
OSi(ODIB)3
Me
Bdmg
43, 11B-NMR
(128 MHz, CDCl3)
f1 (ppm)

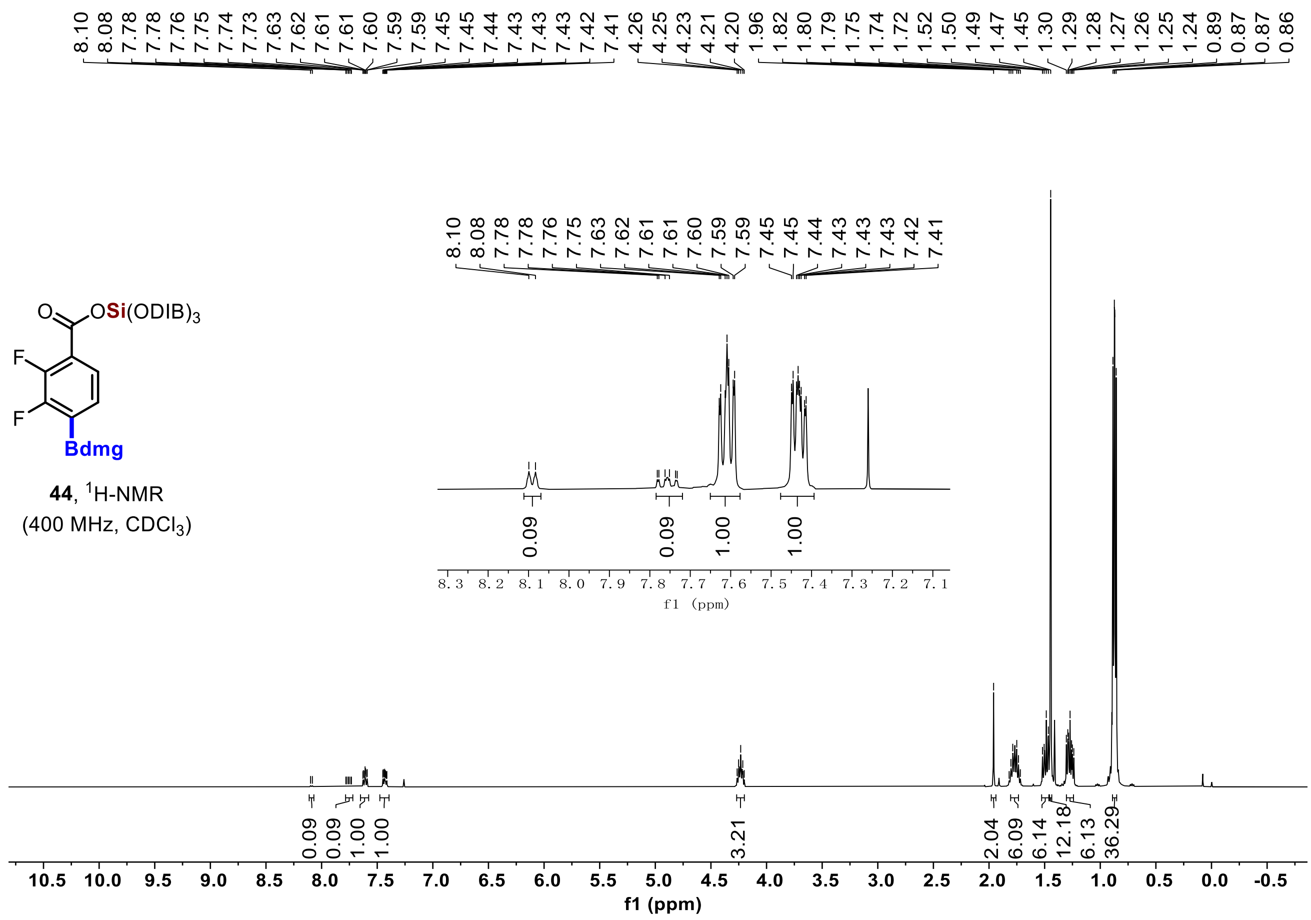

44, ^{1}H-NMR
(400 MHz, $CDCl_3$)

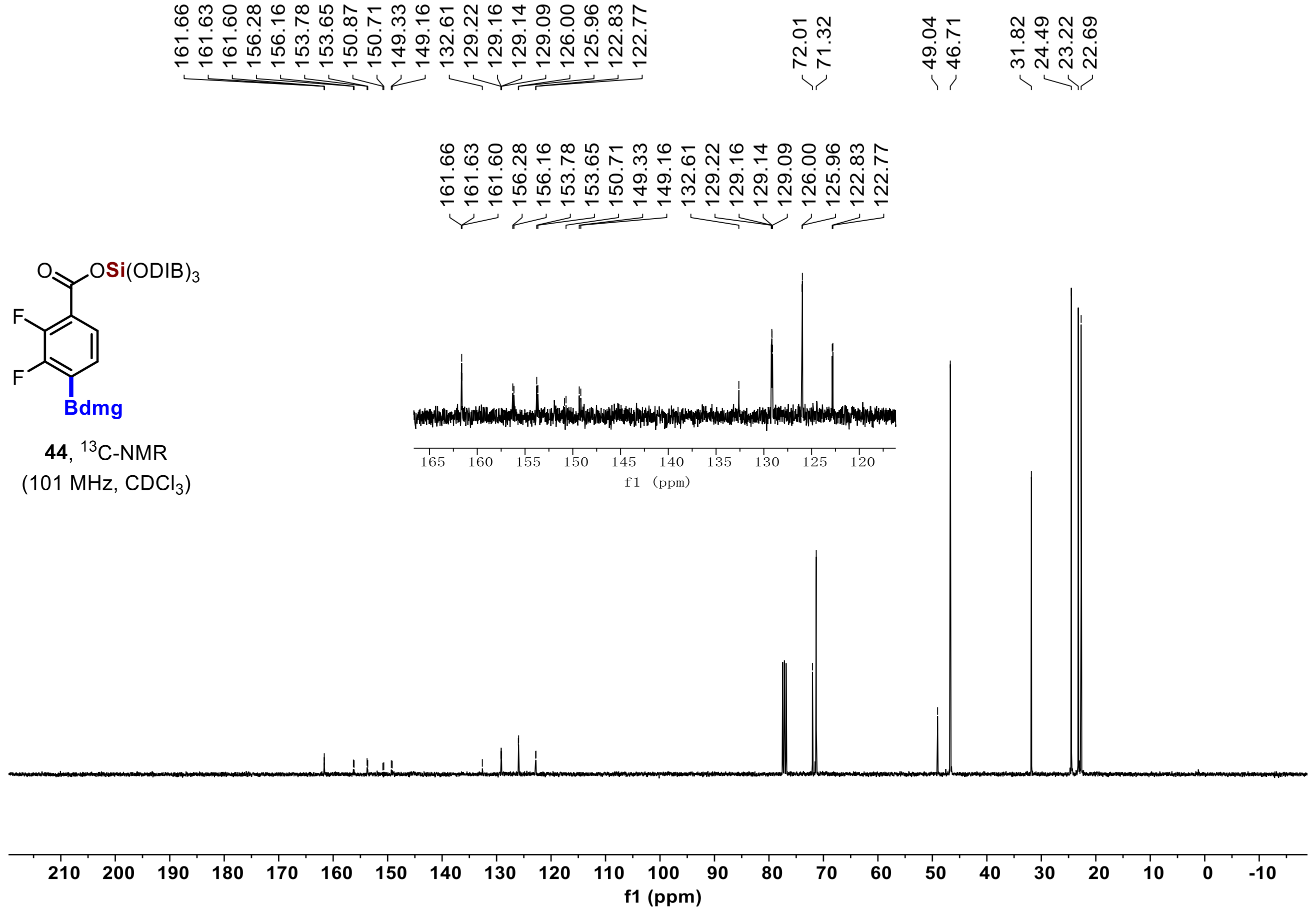

44, ^{13}C-NMR
(101 MHz, $CDCl_3$)

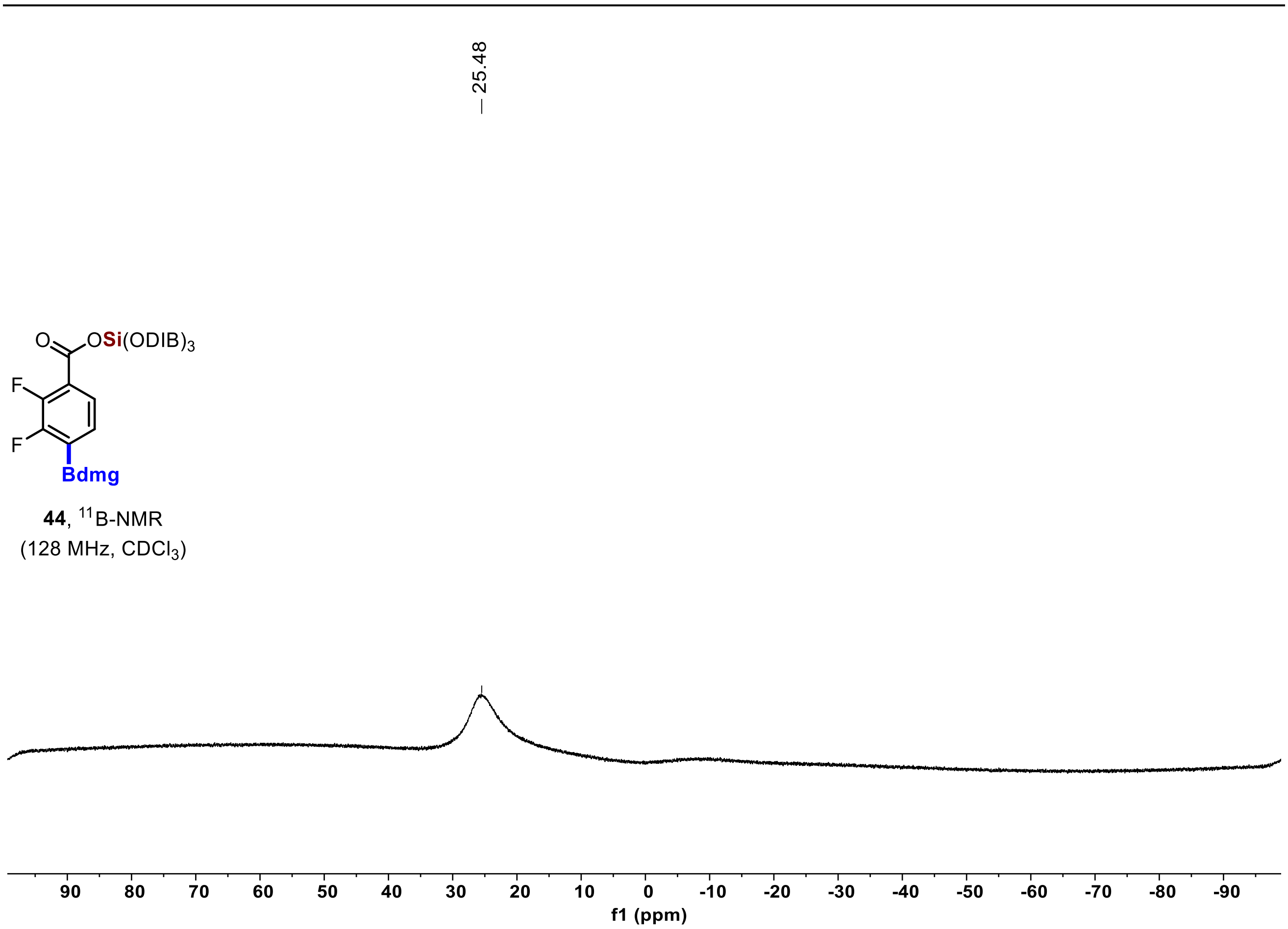

44, ^{11}B-NMR
(128 MHz, $CDCl_3$)

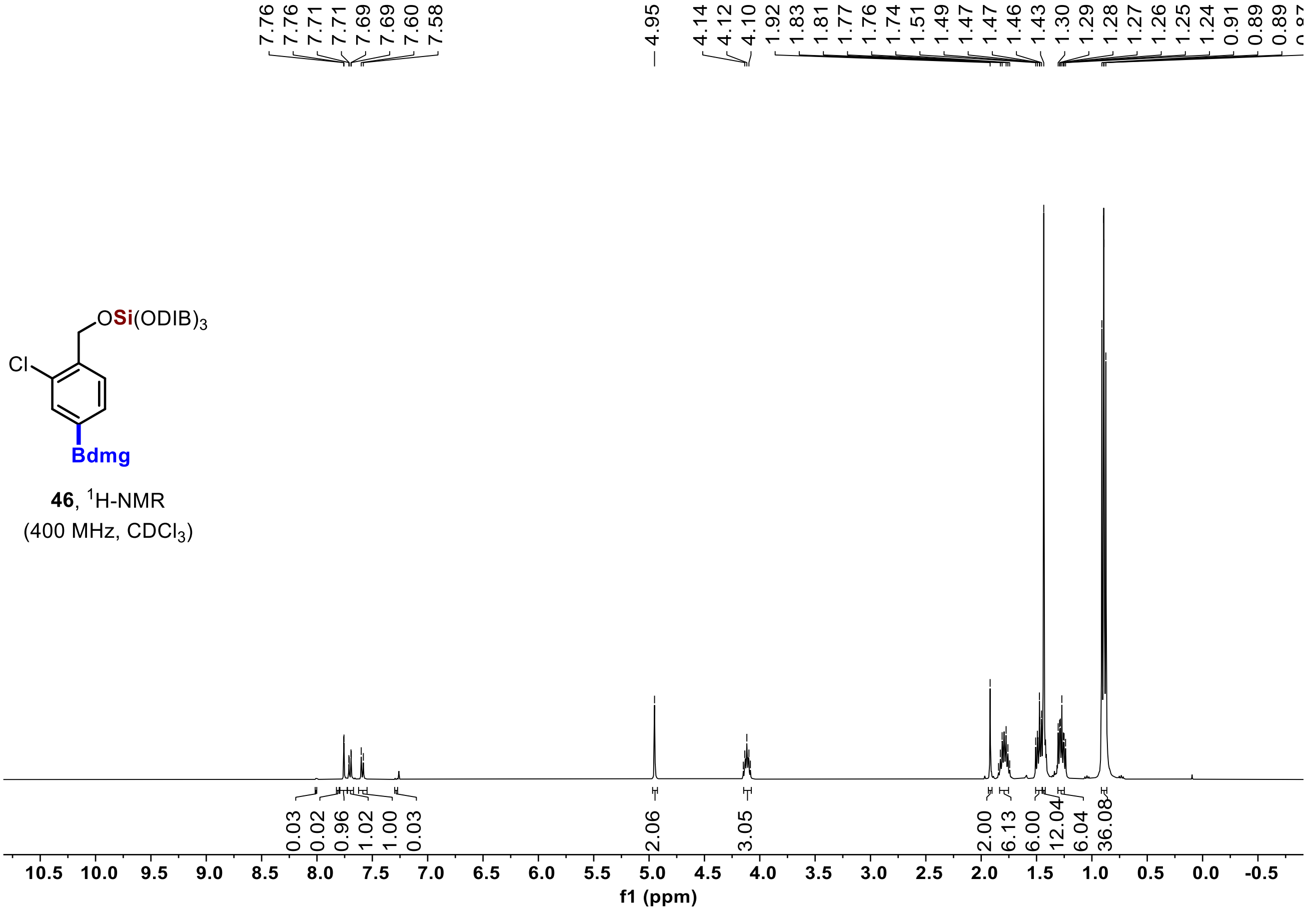

46, ^{1}H-NMR
(400 MHz, $CDCl_3$)

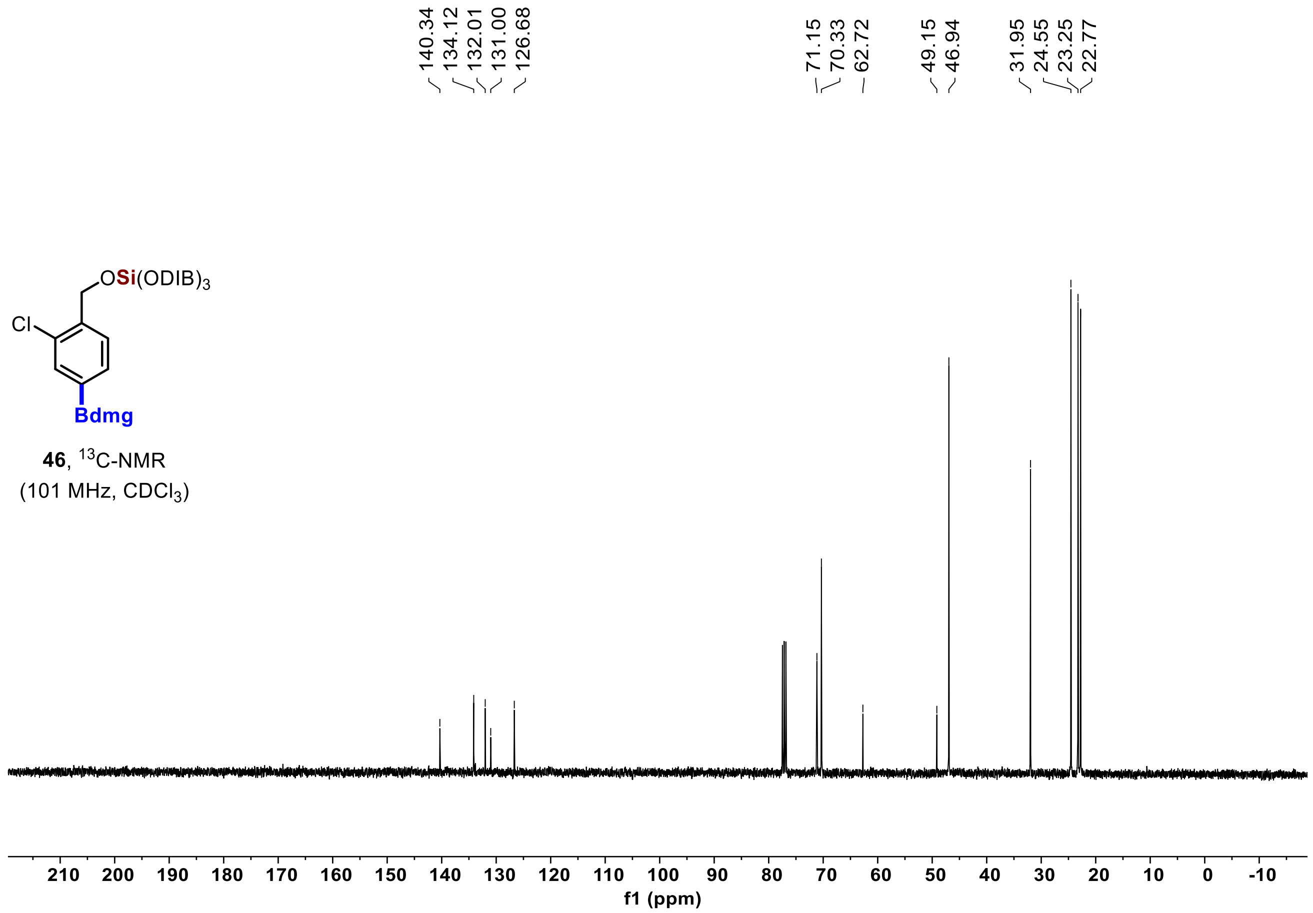

46, ^{13}C-NMR
(101 MHz, $CDCl_3$)

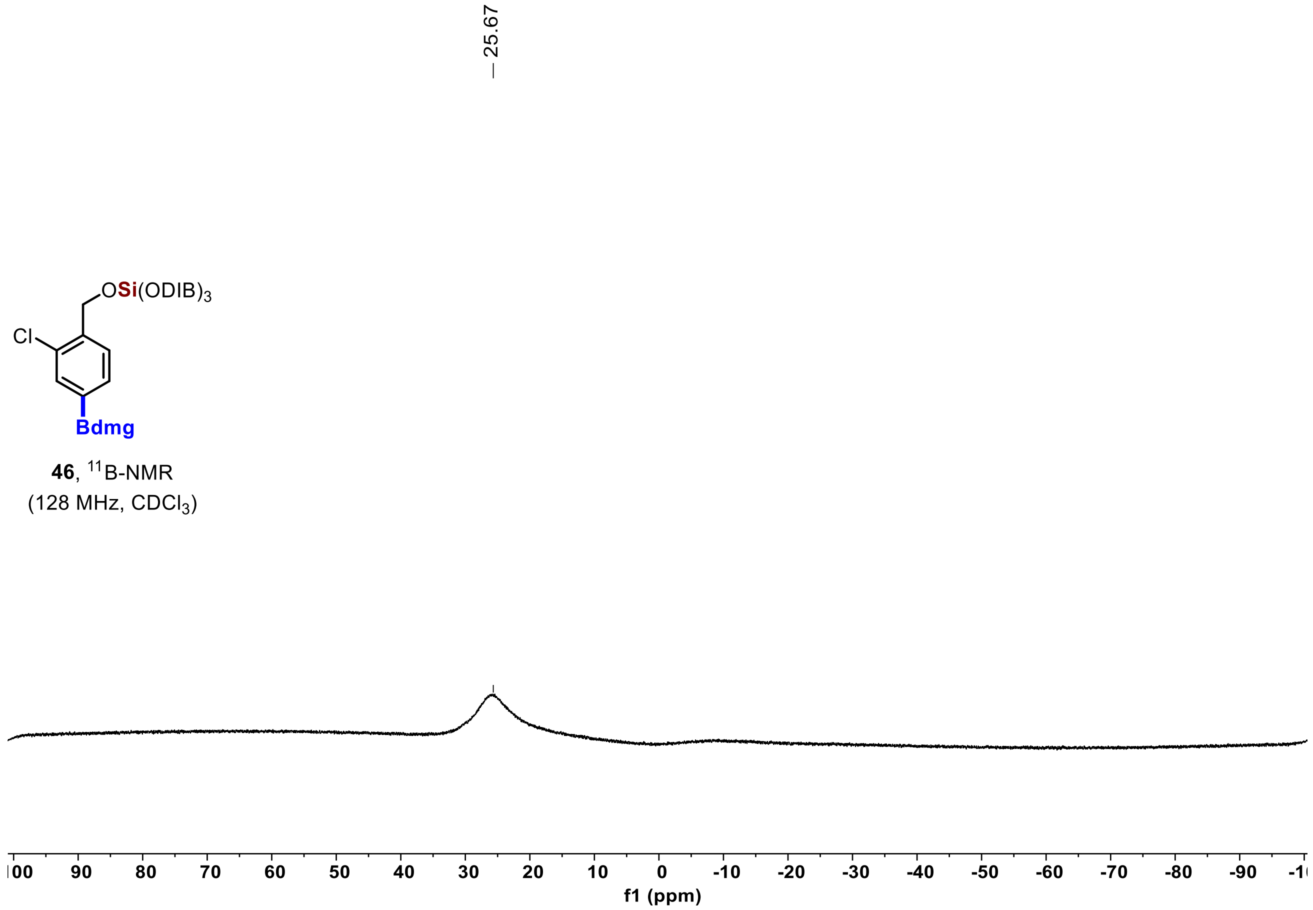

46, ^{11}B-NMR
(128 MHz, $CDCl_3$)

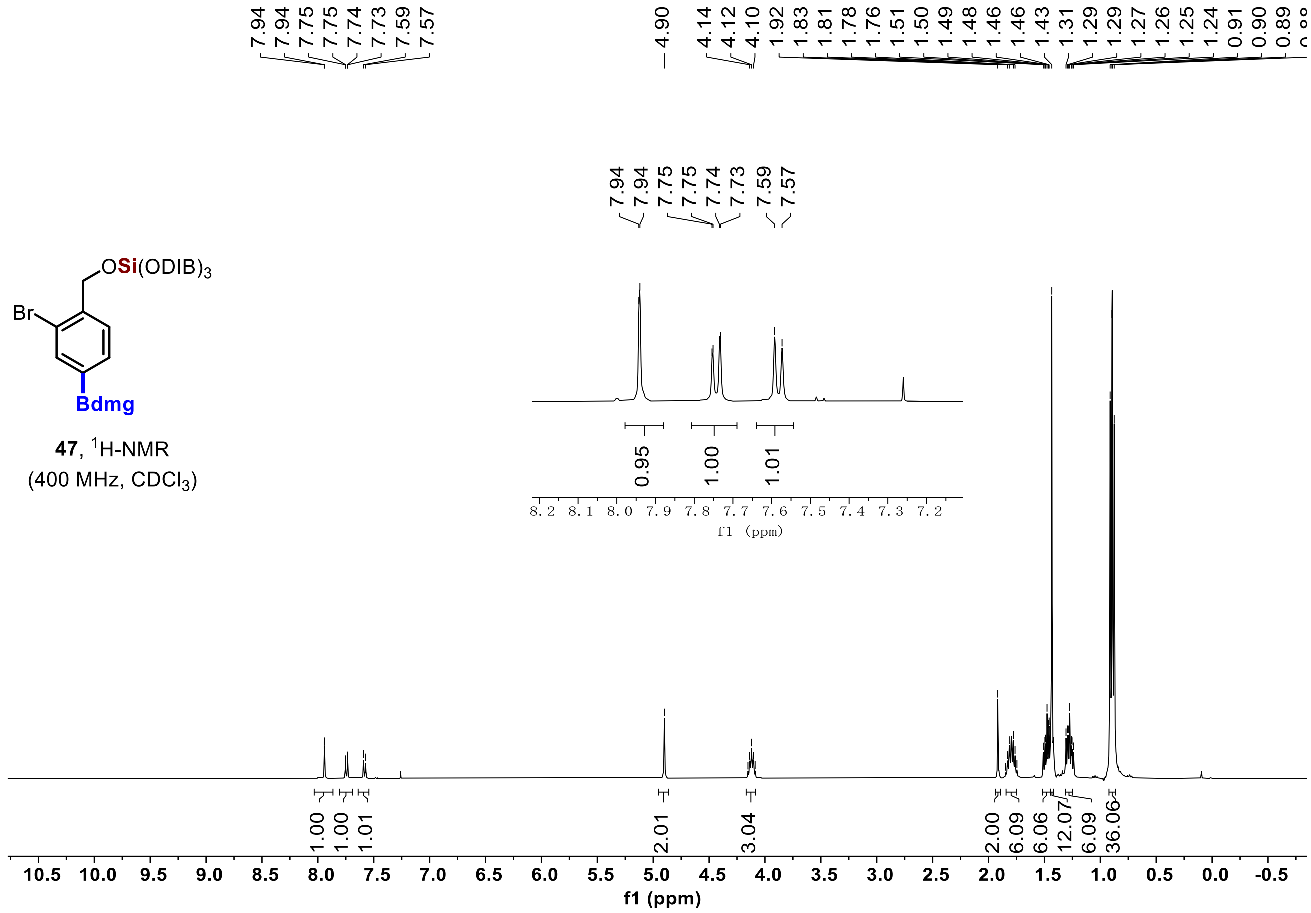

7.94 7.94 7.75 7.75 7.74 7.73 7.59 7.57
4.90
4.14 4.12 4.10 1.92 1.83 1.81 1.78 1.76 1.51 1.50 1.49 1.48 1.46 1.46 1.43 1.31 1.29 1.29 1.27 1.26 1.25 1.24 0.91 0.90 0.89
OSi(ODIB)3
Br
Bdmg
47, 1H-NMR
(400 MHz, CDCl3)
0.95
1.00
1.01
f1 (ppm)
1.00 1.00 1.01 2.01 3.04 2.00 6.09 6.06 12.07 6.09 36.06
f1 (ppm)

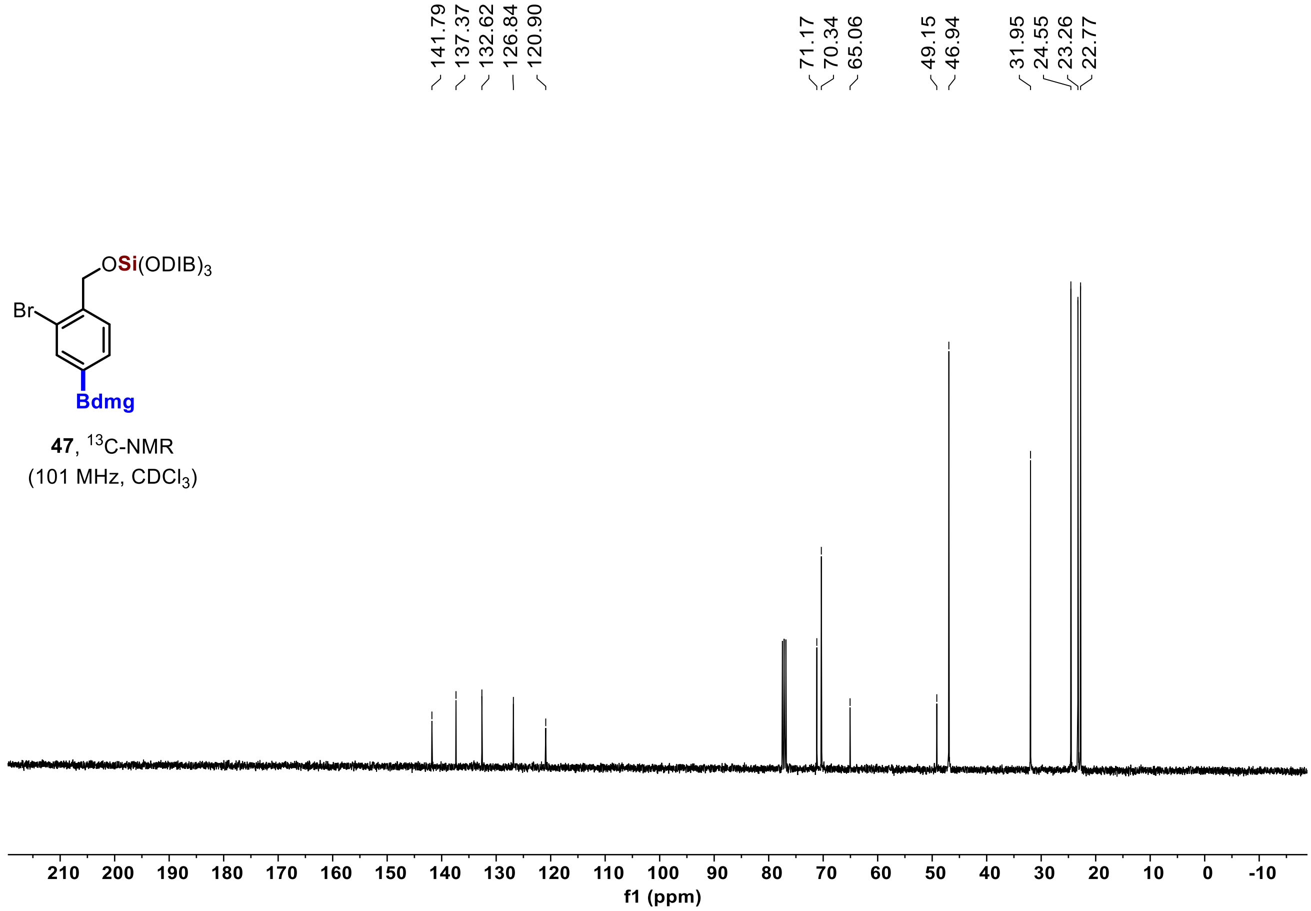

141.79 137.37 132.62 126.84 120.90
71.17 70.34 65.06
49.15 46.94
31.95 24.55 23.26 22.77
OSi(ODIB)3
Br
Bdmg
47, 13C-NMR
(101 MHz, CDCl3)
f1 (ppm)

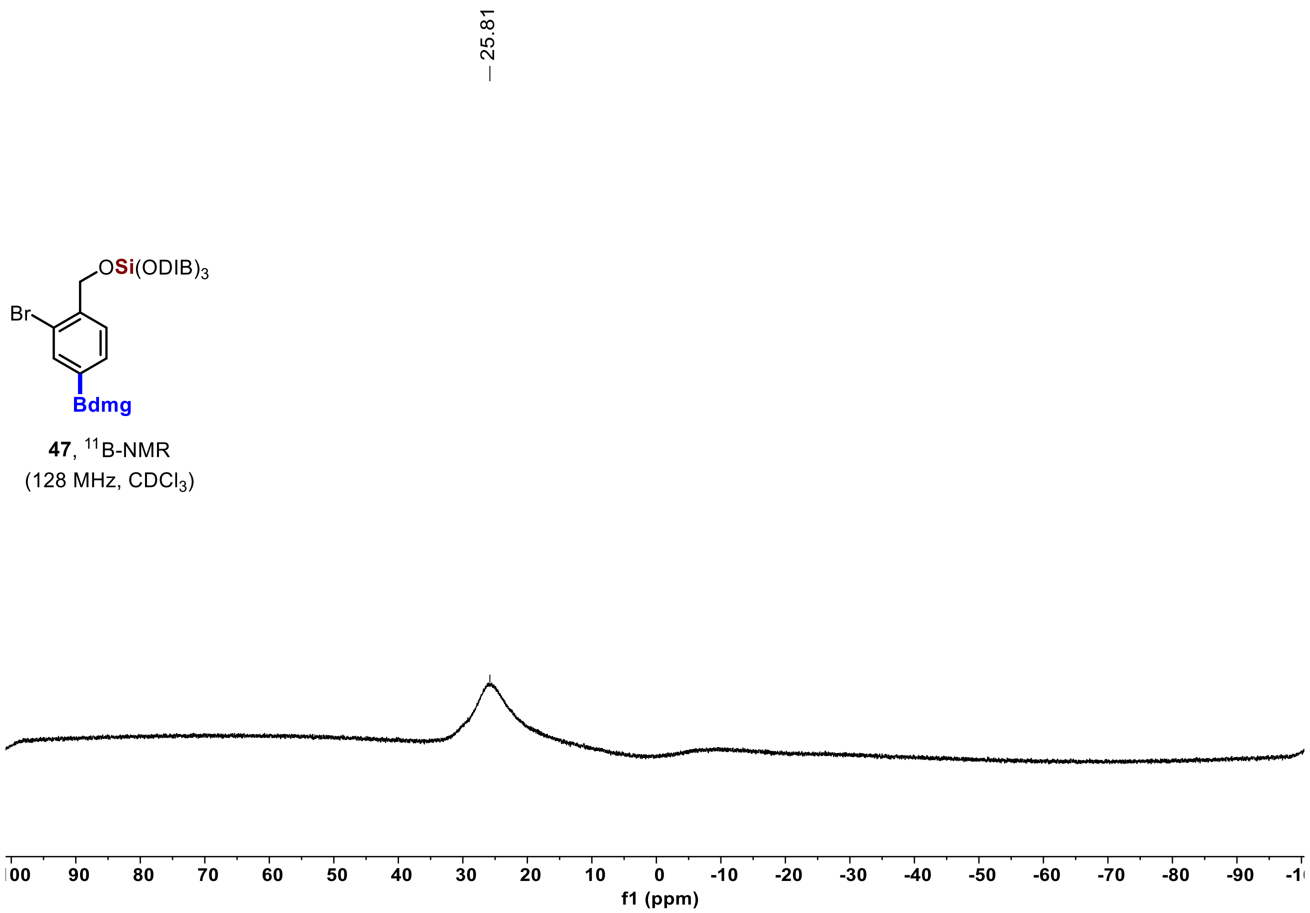

47, ^{11}B-NMR
(128 MHz, $CDCl_3$)

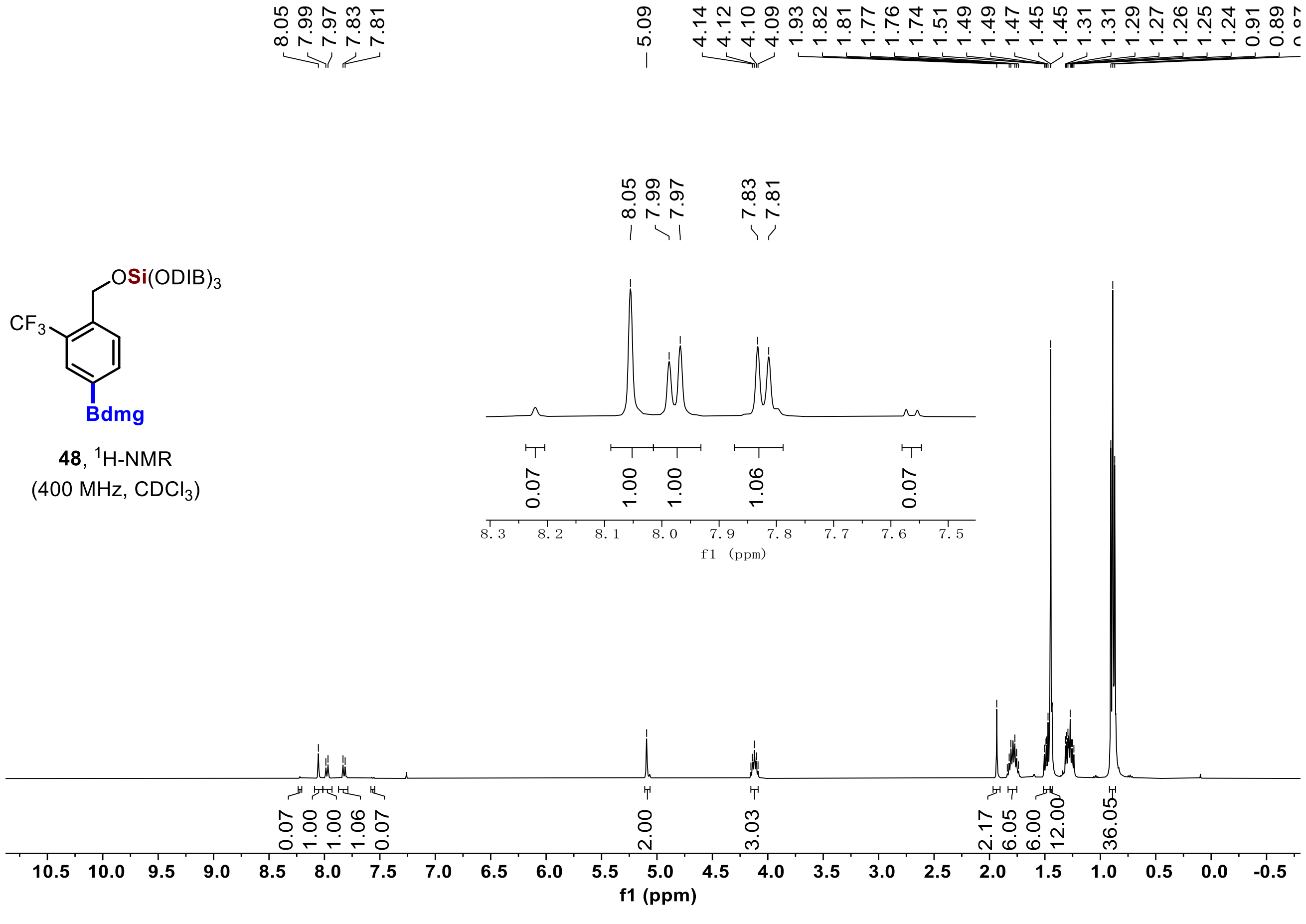

48, ^{1}H-NMR
(400 MHz, $CDCl_3$)

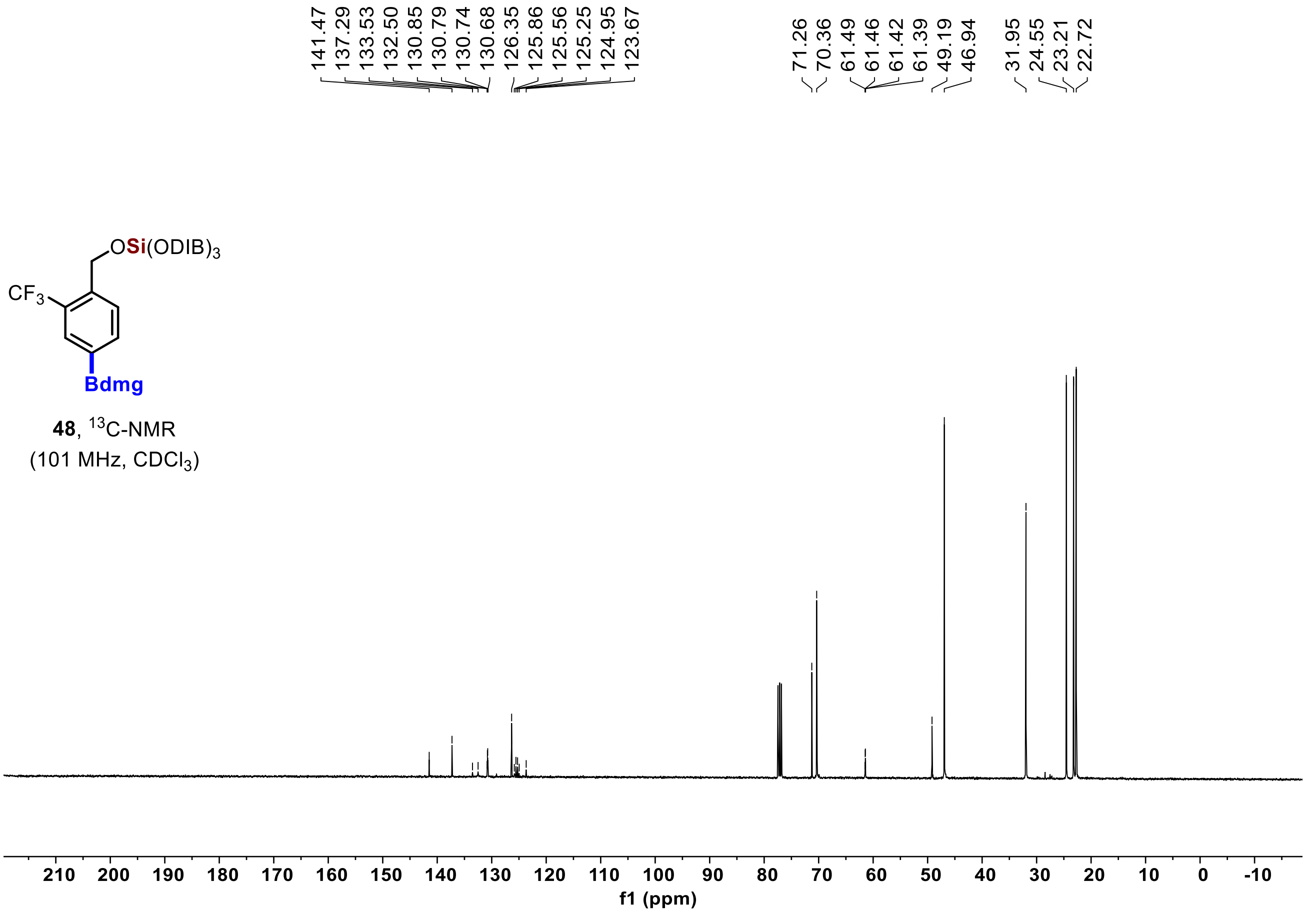

48, ^{13}C-NMR
(101 MHz, $CDCl_3$)

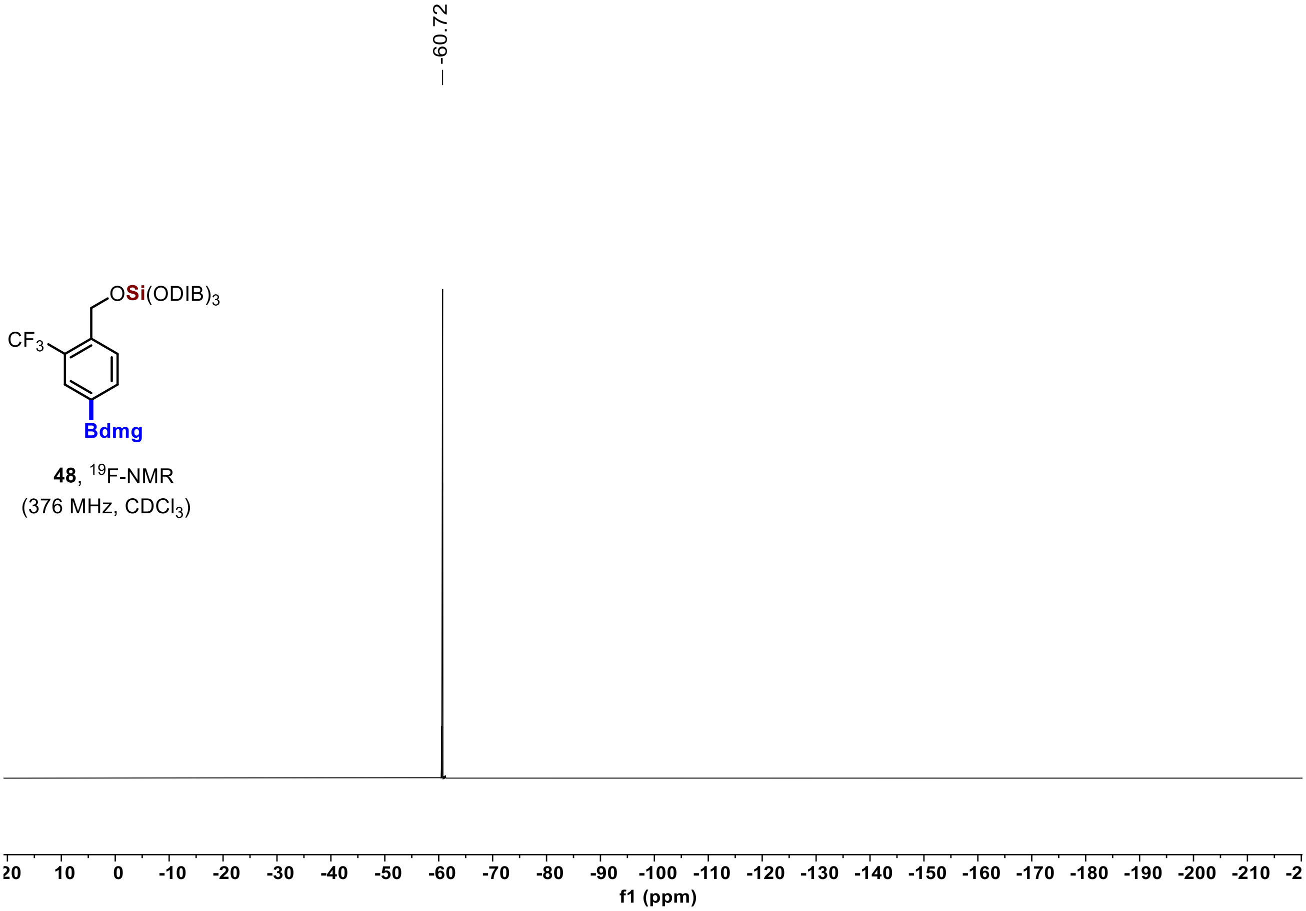

48, ^{19}F-NMR
(376 MHz, $CDCl_3$)

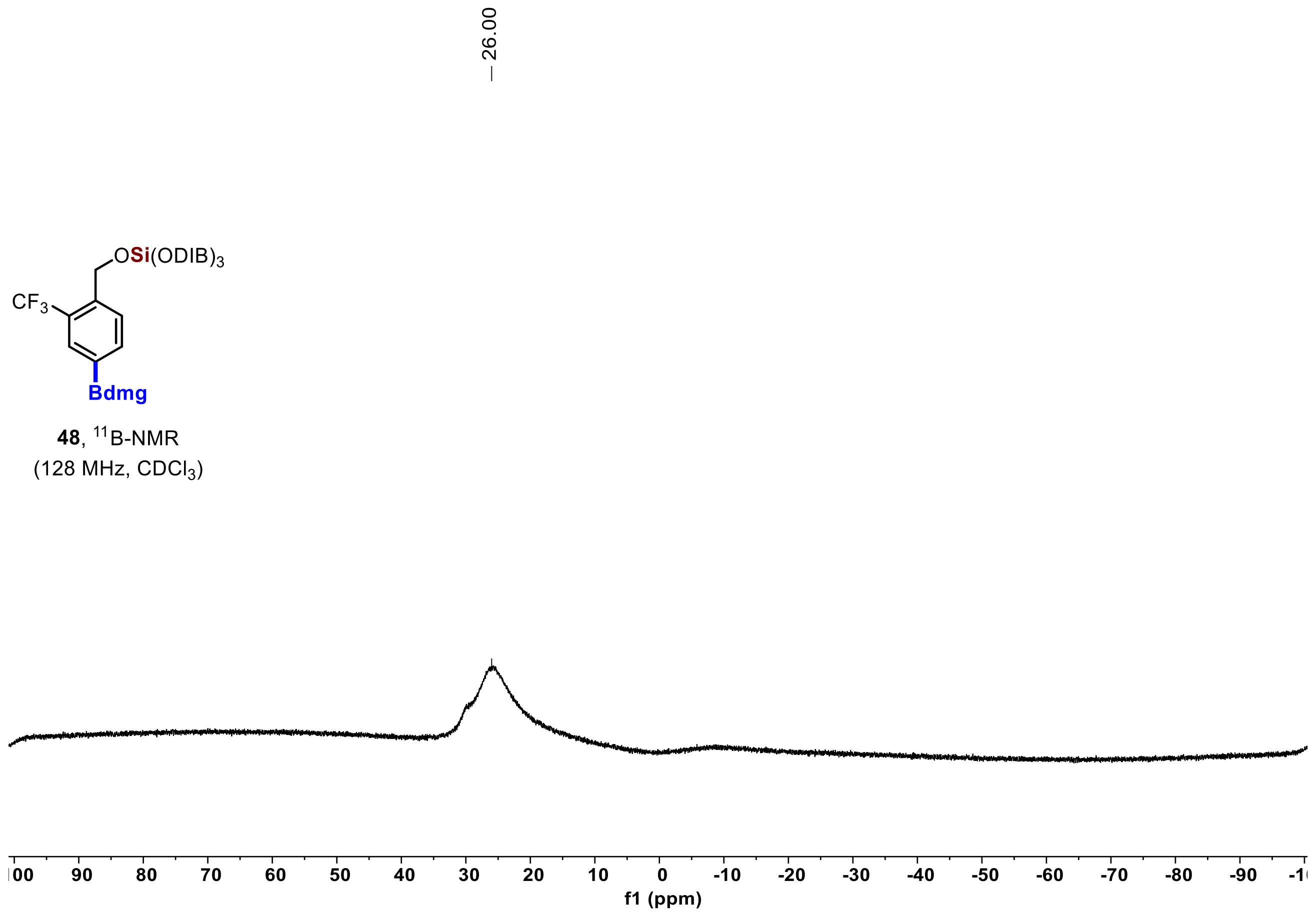

48, ^{11}B-NMR
(128 MHz, $CDCl_3$)

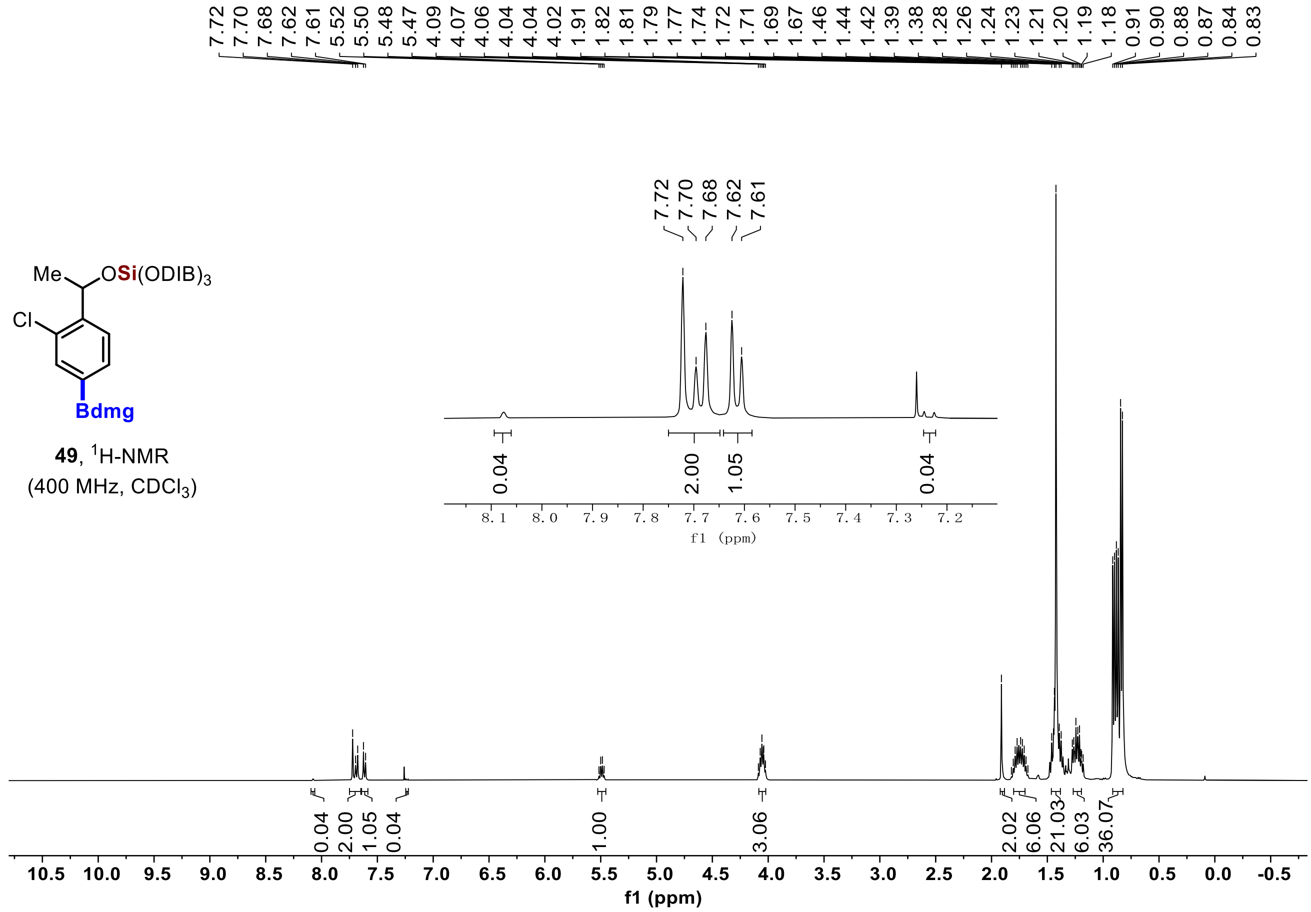

49, ^{1}H-NMR
(400 MHz, $CDCl_3$)

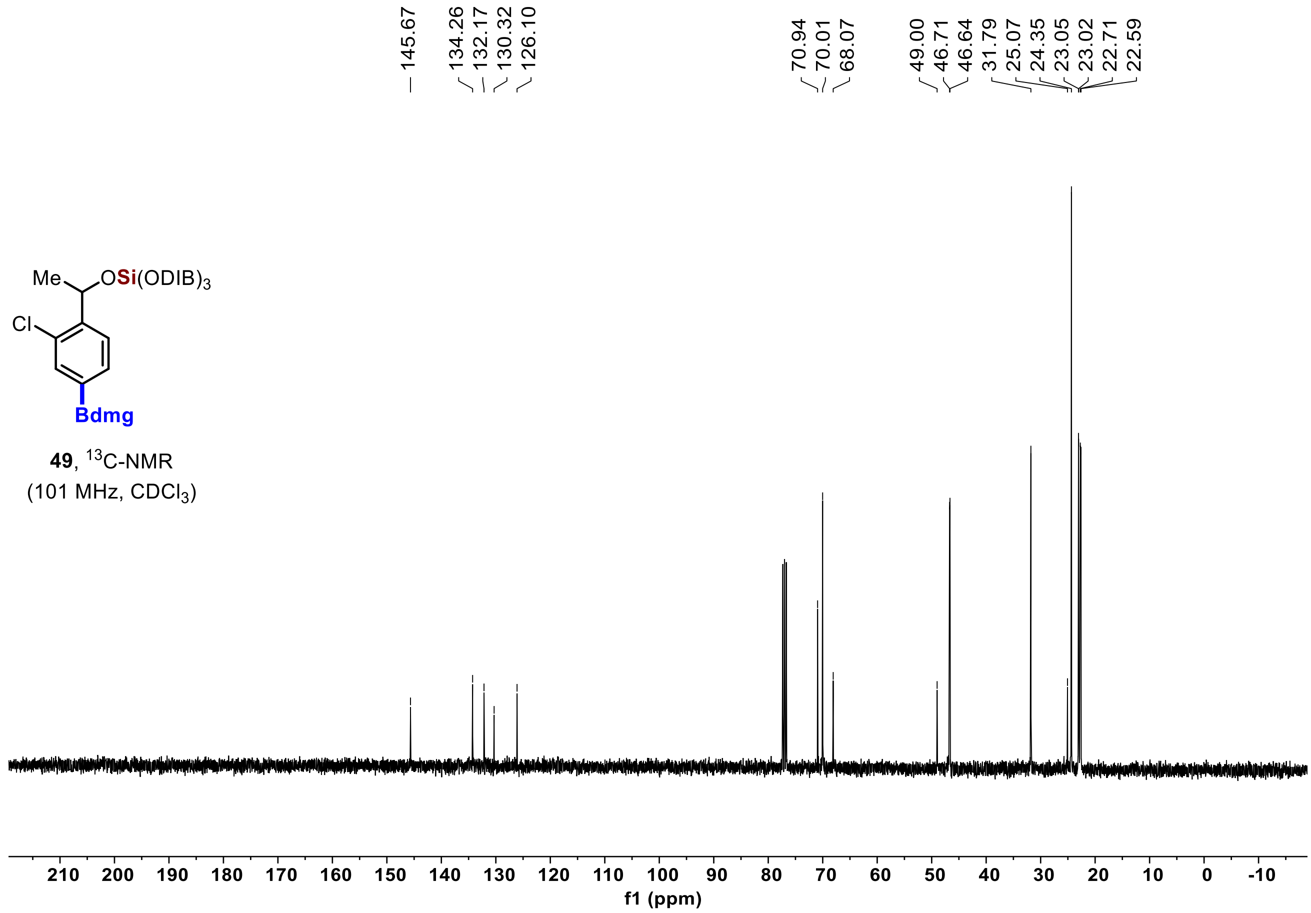
145.67
134.26
132.17
130.32
126.10
70.94
70.01
68.07
49.00
46.71
46.64
31.79
25.07
24.35
23.05
23.02
22.71
22.59
Me
OSi(ODIB)$_3$
Cl
Bdmg
49, ^{13}C-NMR
(101 MHz, $CDCl_3$)
f1 (ppm)

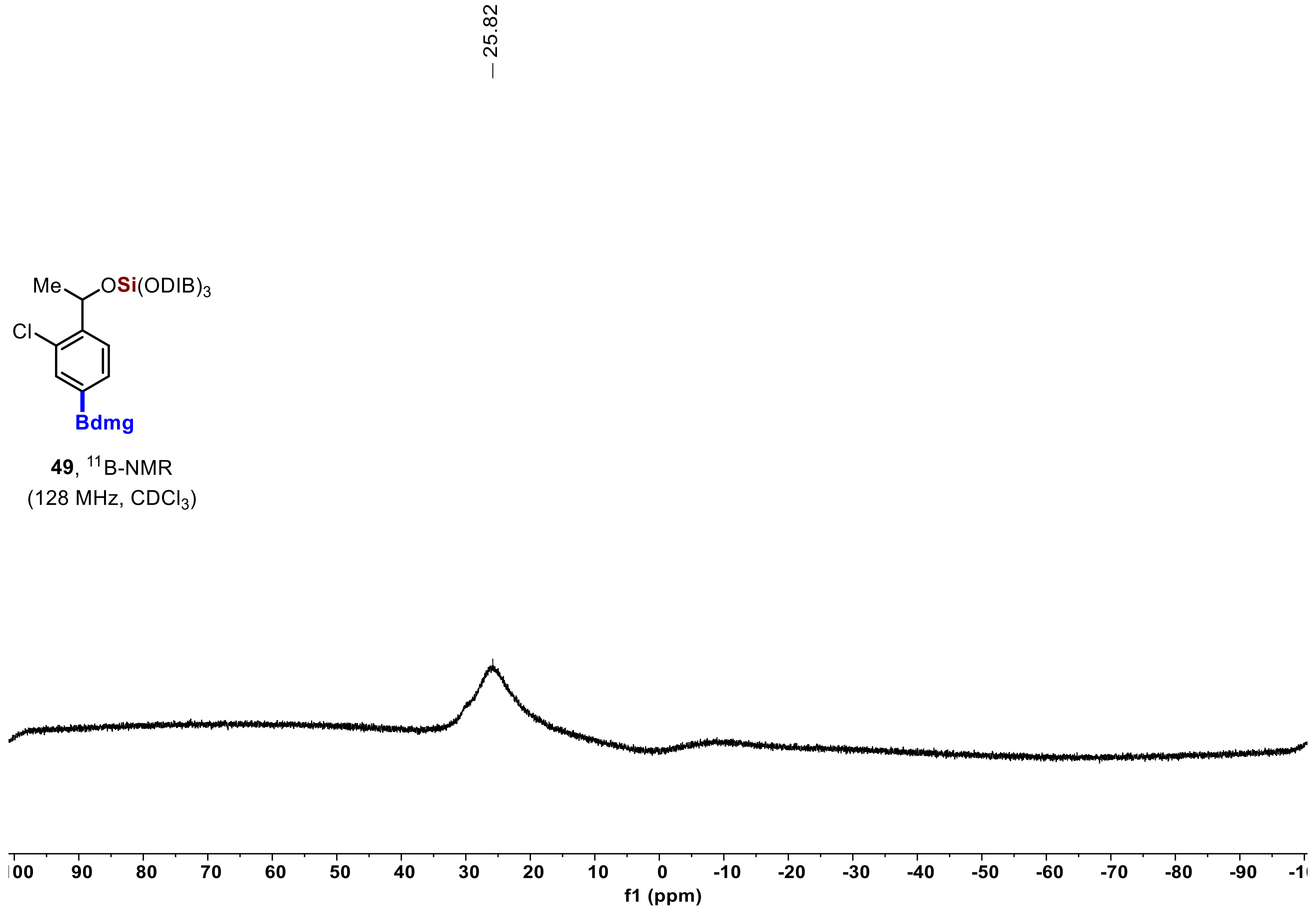
25.82
Me
OSi(ODIB)$_3$
Cl
Bdmg
49, ^{11}B-NMR
(128 MHz, $CDCl_3$)
f1 (ppm)

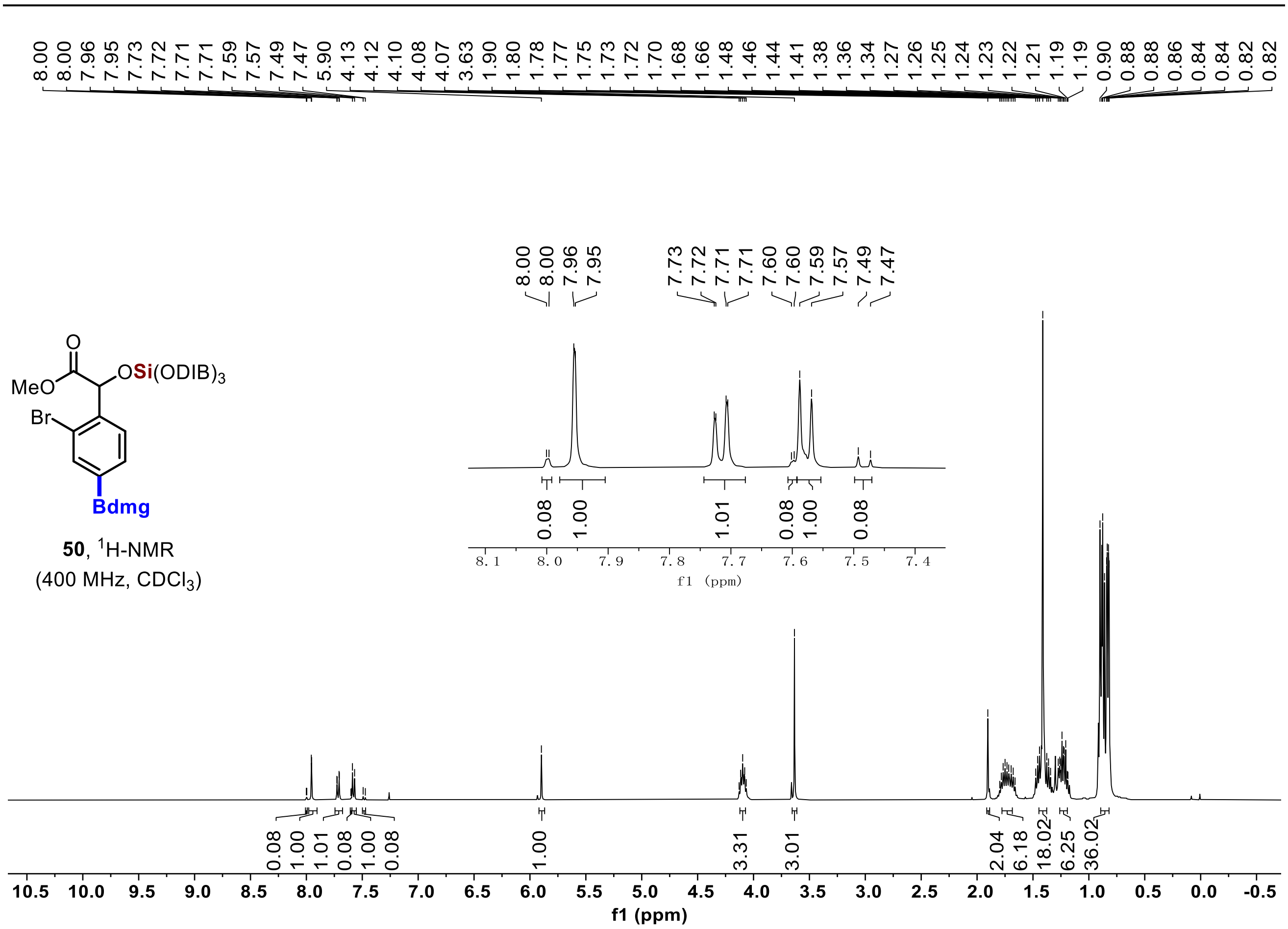
8.00 8.00 7.96 7.95 7.73 7.72 7.71 7.71 7.59 7.57 7.49 7.47 5.90 4.13 4.12 4.10 4.08 4.07 3.63 1.90 1.80 1.78 1.77 1.75 1.73 1.72 1.70 1.68 1.66 1.48 1.46 1.44 1.41 1.38 1.36 1.34 1.27 1.26 1.25 1.24 1.23 1.22 1.21 1.19 1.19 0.90 0.88 0.86 0.84 0.84 0.82 0.82
8.00 8.00 7.96 7.95 7.73 7.72 7.71 7.71 7.60 7.60 7.59 7.57 7.49 7.47
0.08 1.00 1.01 0.08 1.00 0.08
f1 (ppm)
MeO
OSi(ODIB)3
Br
Bdmg
50, 1H-NMR
(400 MHz, CDCl3)
0.08 1.00 1.01 0.08 1.00 0.08 1.00 3.31 3.01 2.04 6.18 18.02 6.25 36.02
f1 (ppm)

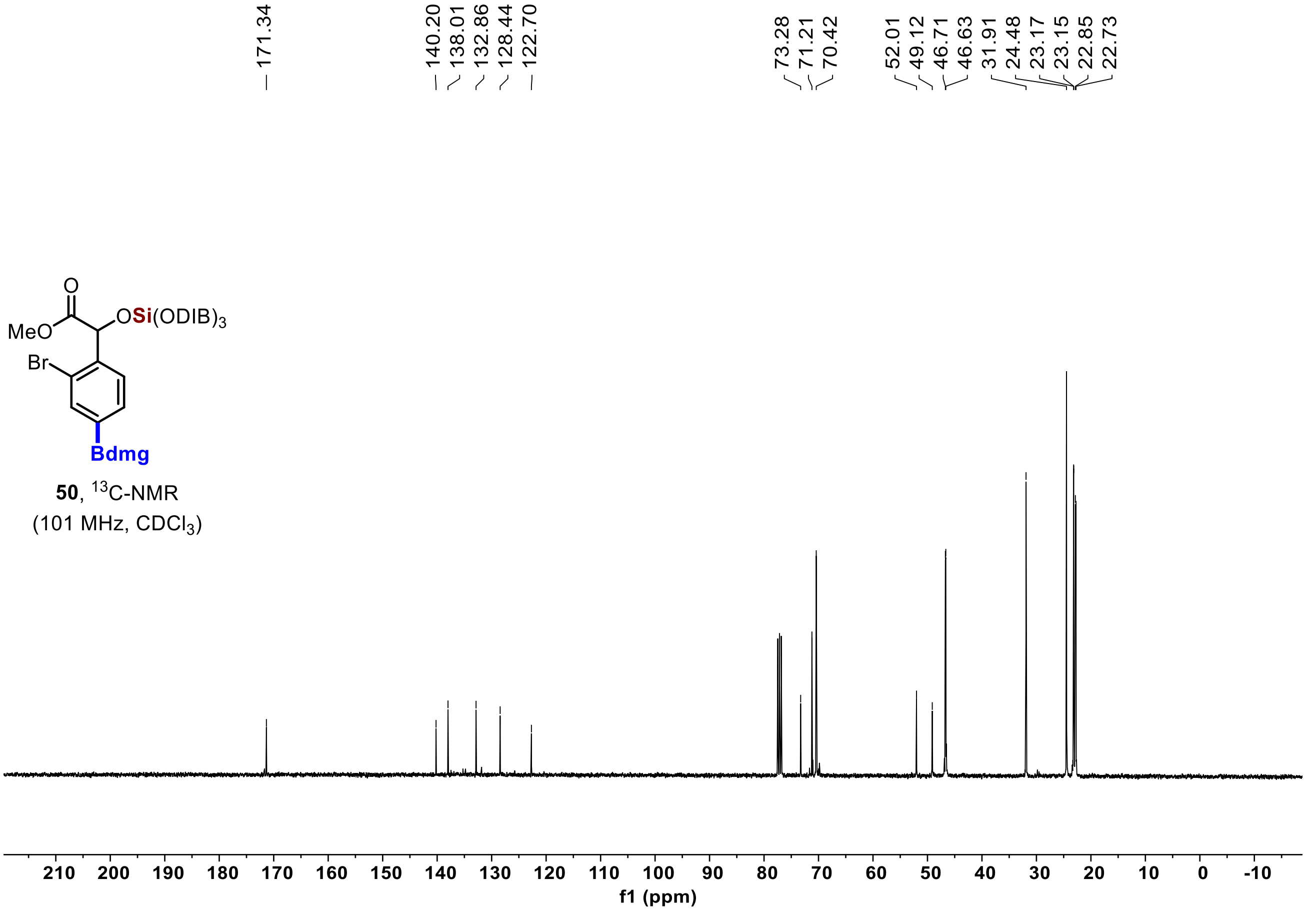
171.34
140.20 138.01 132.86 128.44 122.70
73.28 71.21 70.42
52.01 49.12 46.71 46.63 31.91 24.48 23.17 23.15 22.85 22.73
MeO
OSi(ODIB)3
Br
Bdmg
50, 13C-NMR
(101 MHz, CDCl3)
f1 (ppm)

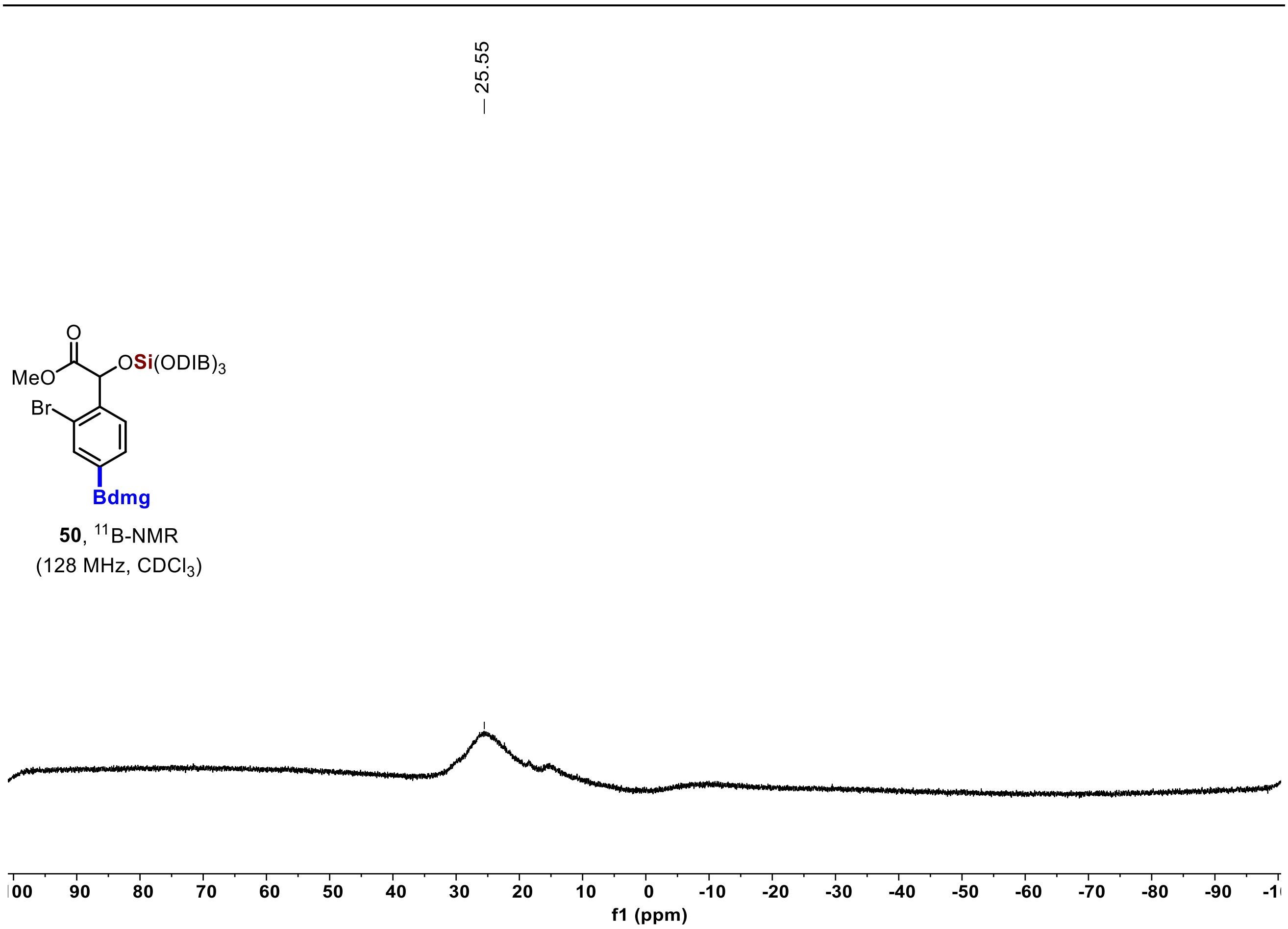

50, ^{11}B-NMR
(128 MHz, $CDCl_3$)

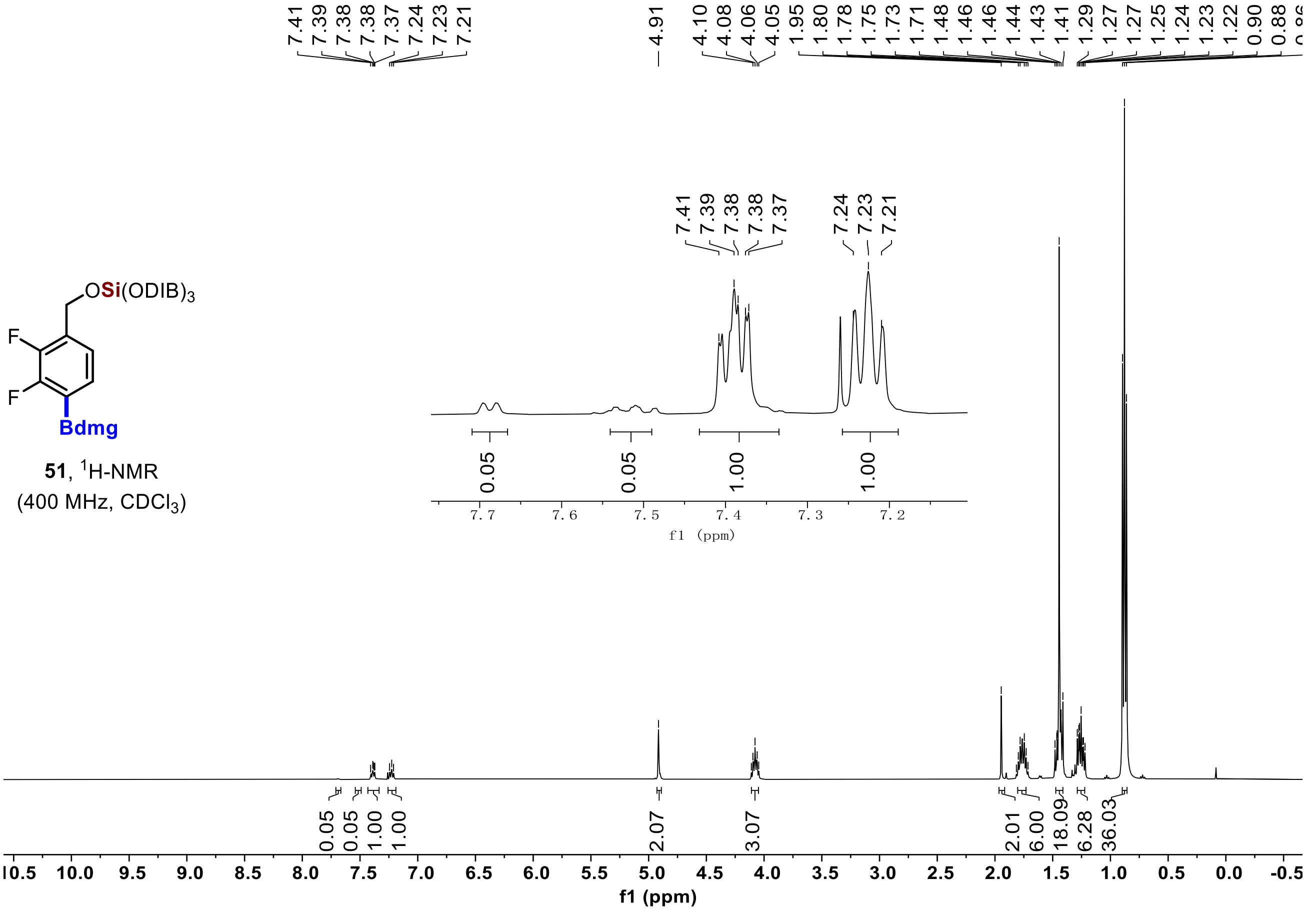

51, ^{1}H-NMR
(400 MHz, $CDCl_3$)

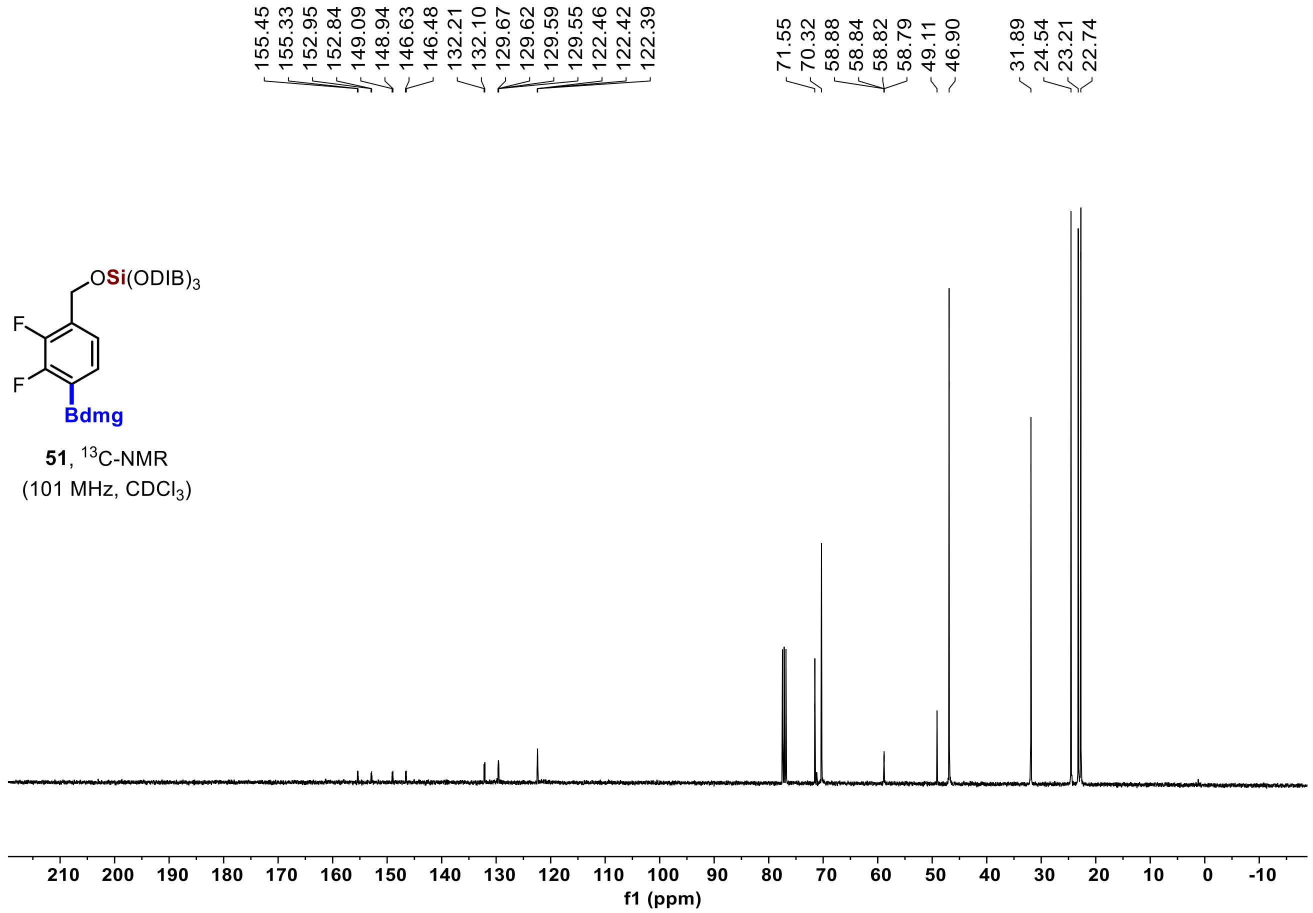

155.45
155.33
152.95
152.84
149.09
148.94
146.63
146.48
132.21
132.10
129.67
129.62
129.59
129.55
122.46
122.42
122.39
71.55
70.32
58.88
58.84
58.82
58.79
49.11
46.90
31.89
24.54
23.21
22.74
OSi(ODIB)3
F
F
Bdmg
51, 13C-NMR
(101 MHz, CDCl3)
210 200 190 180 170 160 150 140 130 120 110 100 90 80 70 60 50 40 30 20 10 0 -10
f1 (ppm)

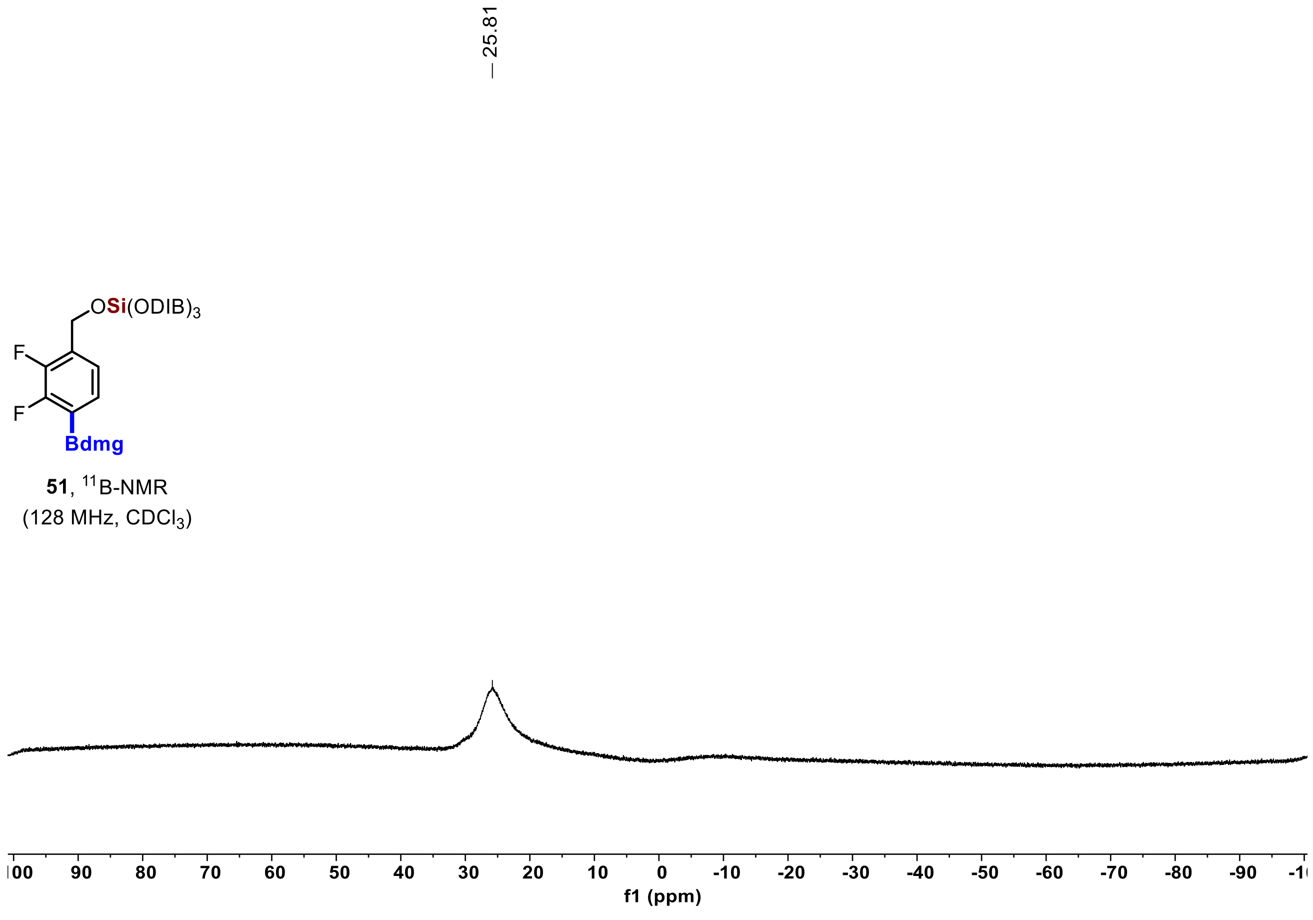

25.81
OSi(ODIB)3
F
F
Bdmg
51, 11B-NMR
(128 MHz, CDCl3)
90 80 70 60 50 40 30 20 10 0 -10 -20 -30 -40 -50 -60 -70 -80 -90
f1 (ppm)

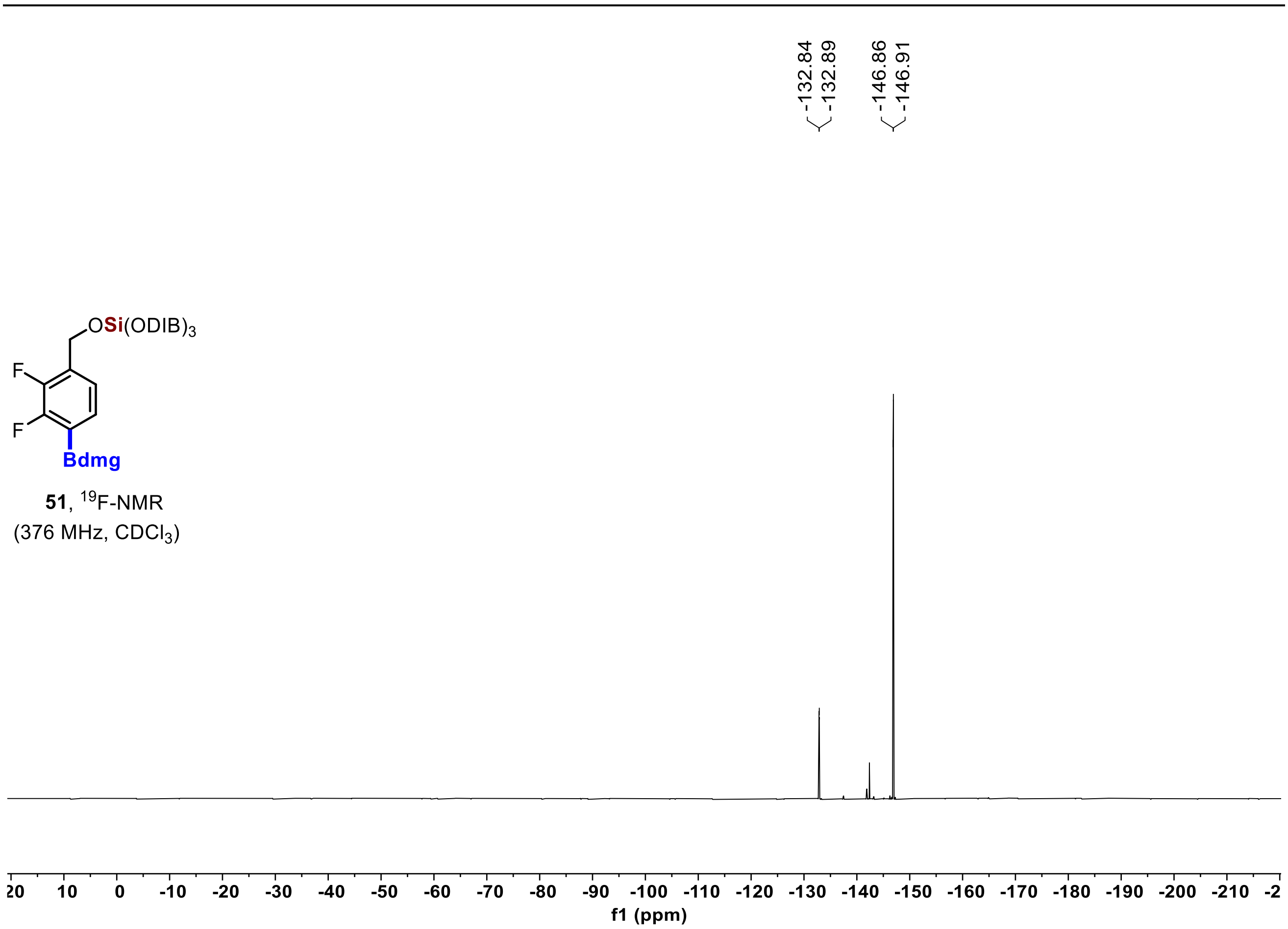

OSi(ODIB)3
F
F
Bdmg
51, 19F-NMR
(376 MHz, CDCl3)
-132.84
-132.89
-146.86
-146.91
f1 (ppm)

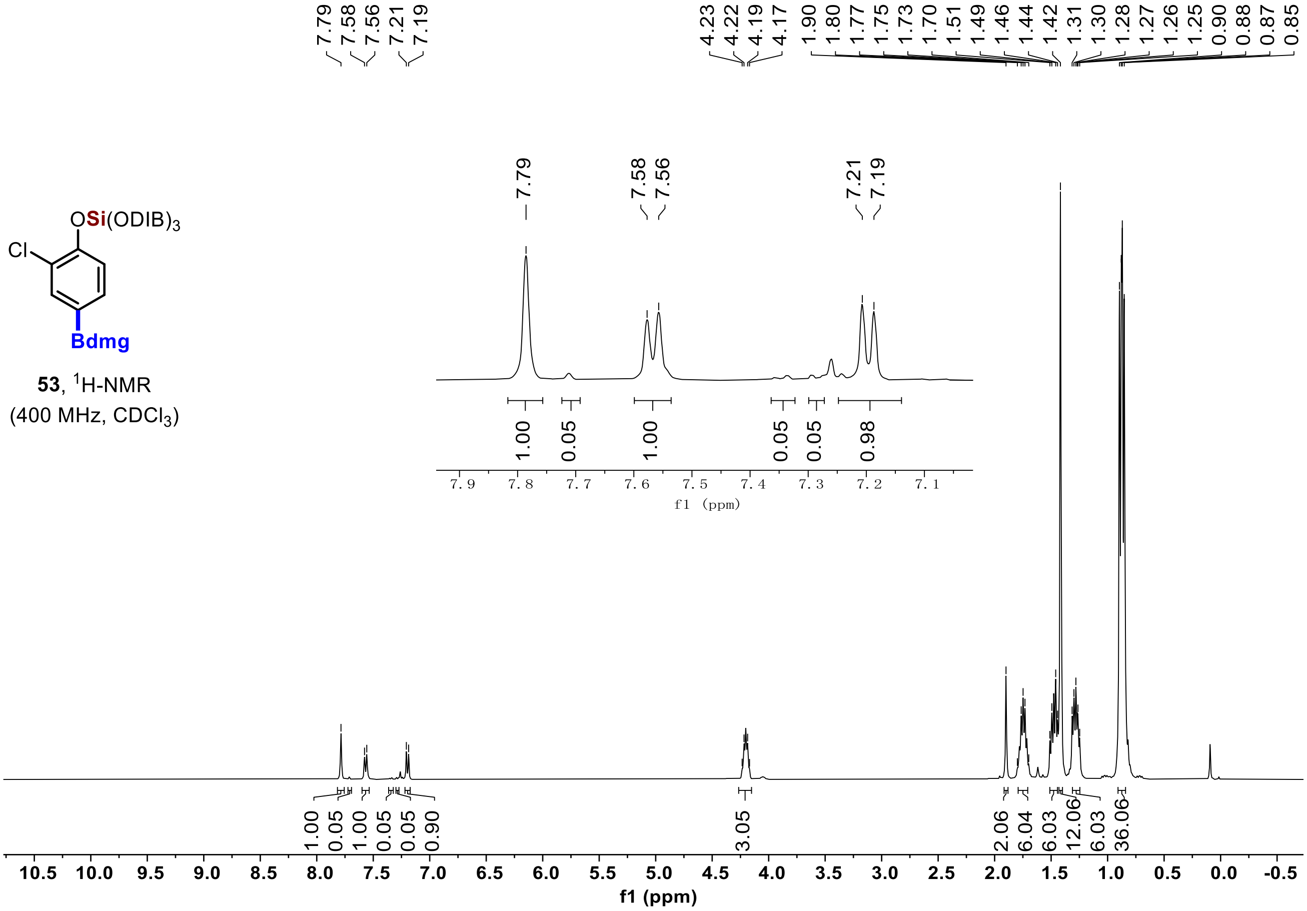

OSi(ODIB)3
Cl
Bdmg
53, 1H-NMR
(400 MHz, CDCl3)
7.79
7.58
7.56
7.21
7.19
4.23
4.22
4.19
4.17
1.90
1.80
1.77
1.75
1.73
1.70
1.51
1.49
1.46
1.44
1.42
1.31
1.30
1.28
1.27
1.26
1.25
0.90
0.88
0.87
0.85
f1 (ppm)

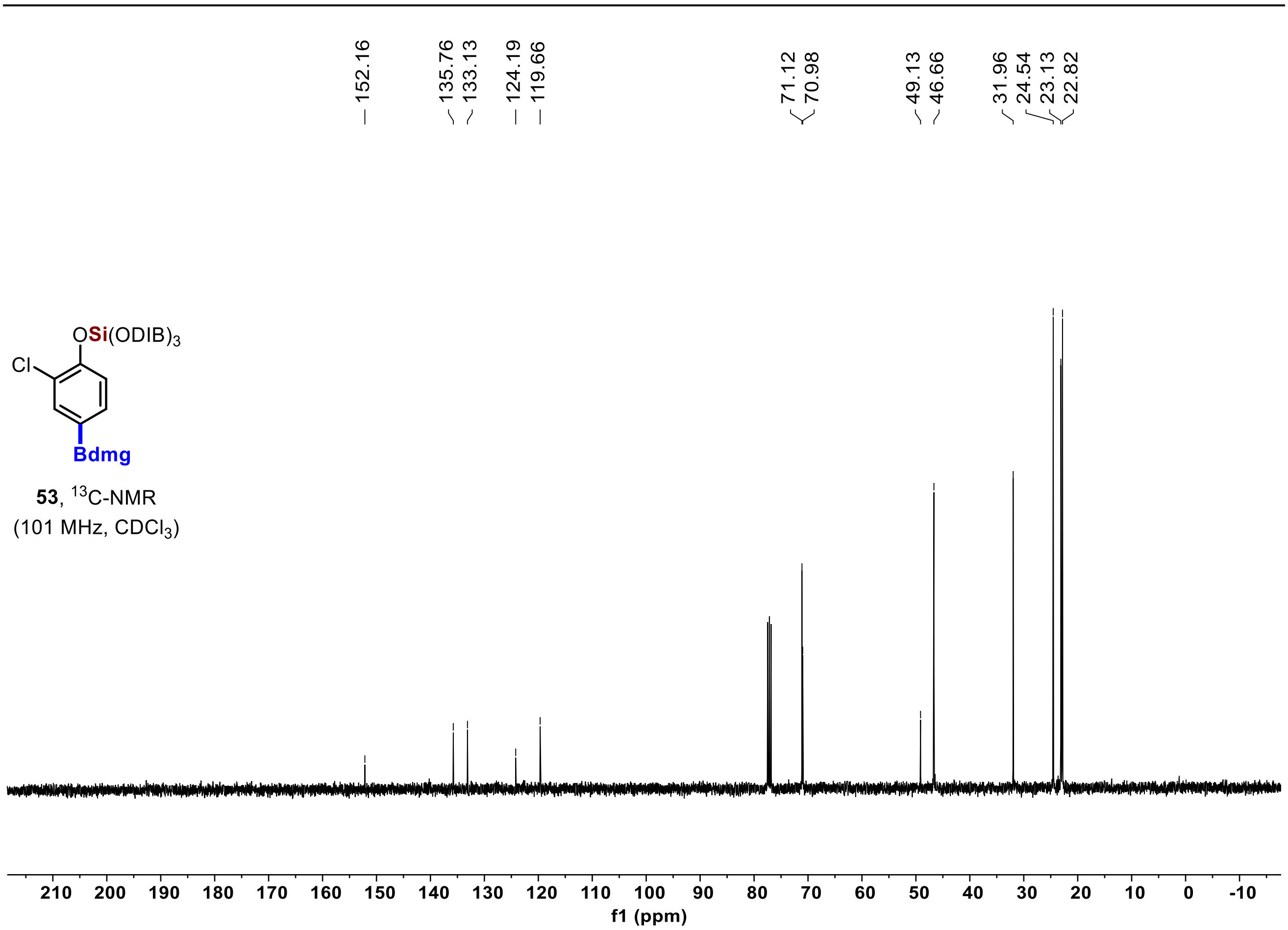

53, ^{13}C-NMR
(101 MHz, $CDCl_3$)

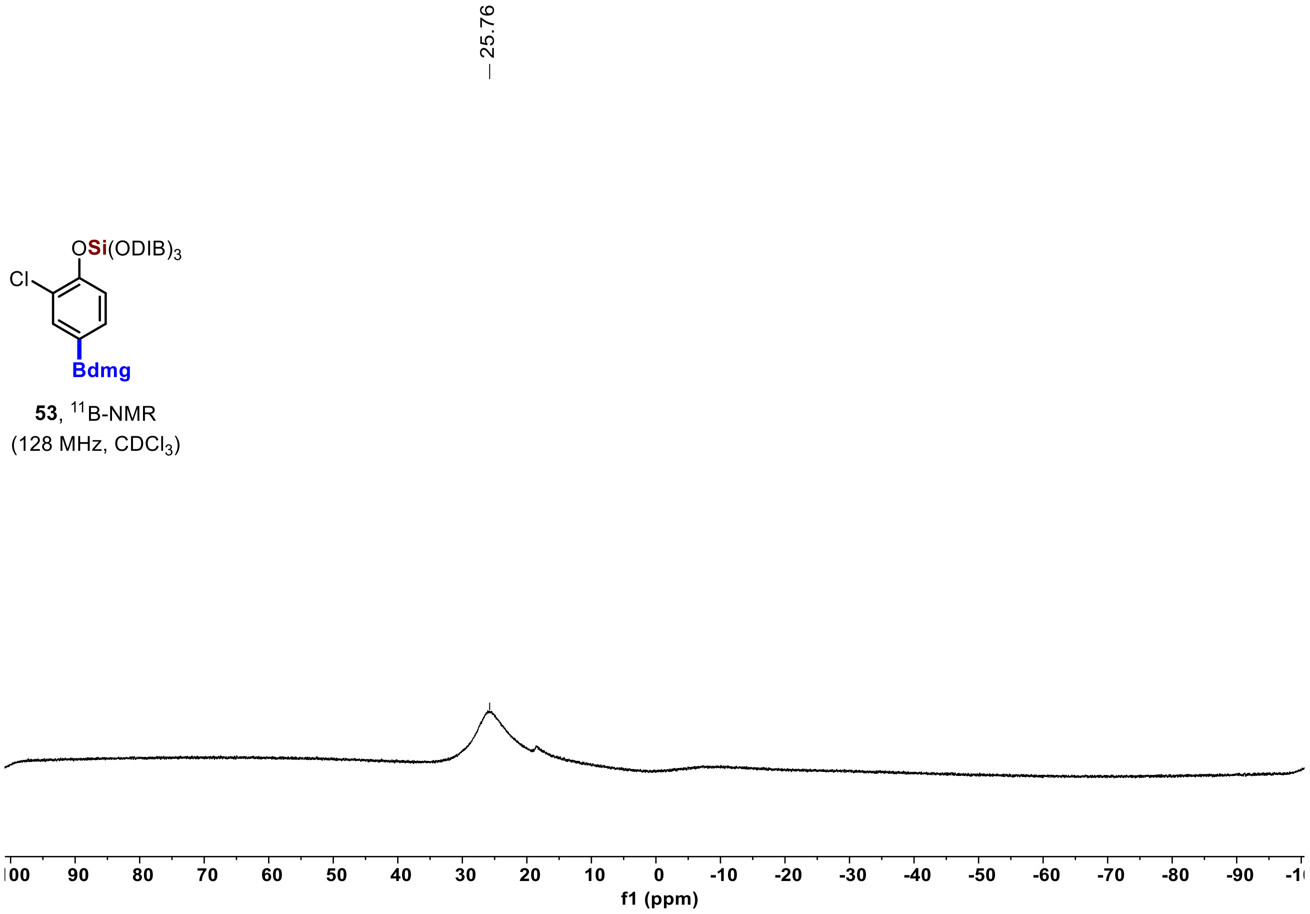

53, ^{11}B-NMR
(128 MHz, $CDCl_3$)

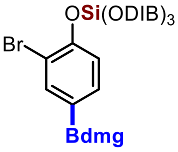

54, ^{1}H-NMR
(400 MHz, $CDCl_3$)

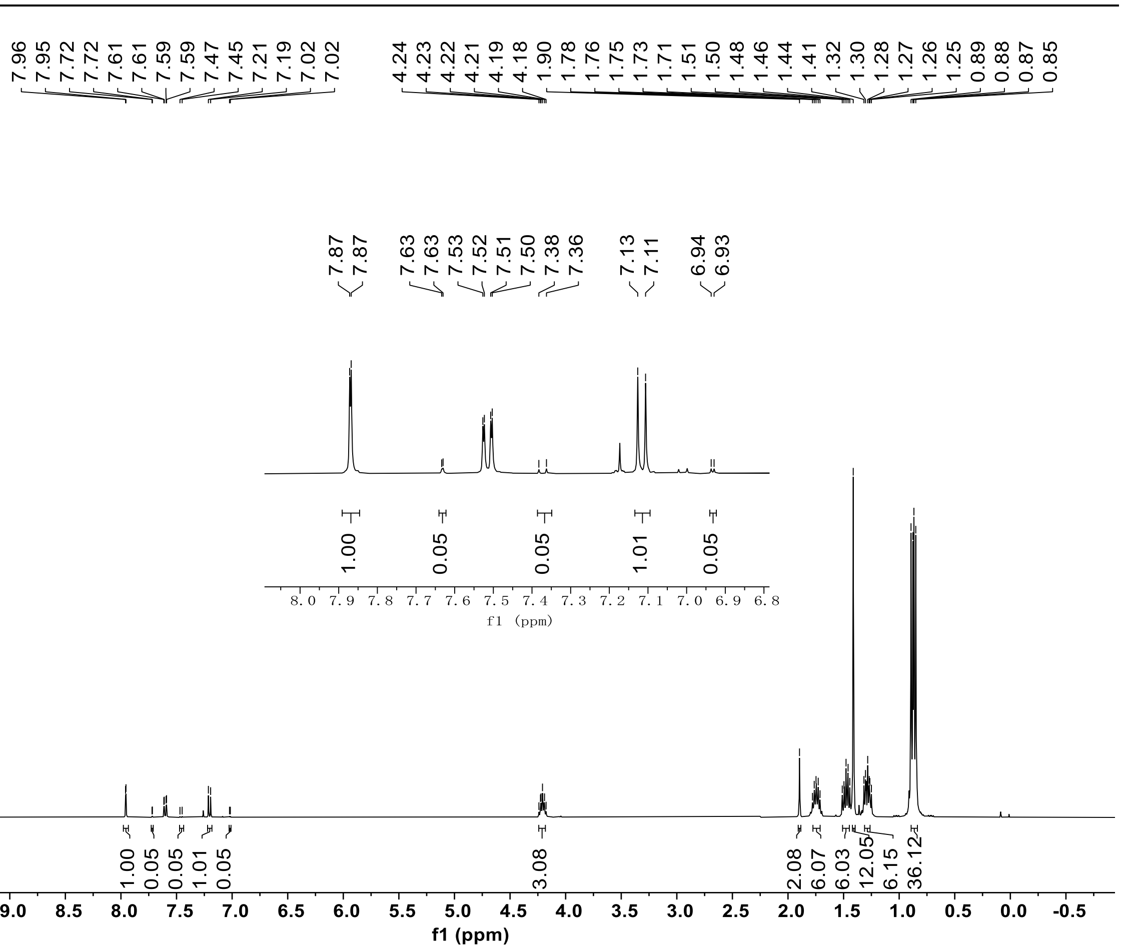

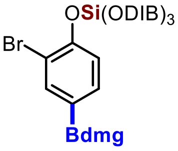

54, ^{13}C-NMR
(101 MHz, $CDCl_3$)

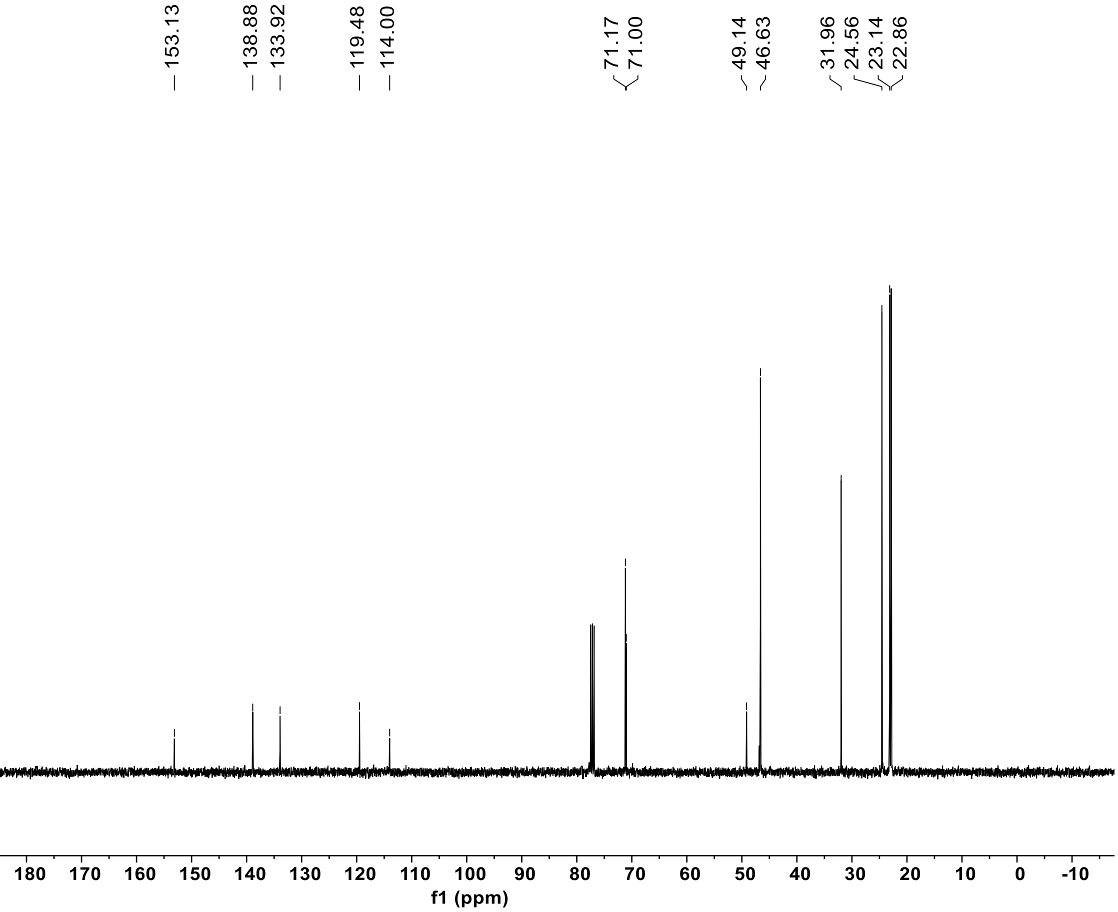

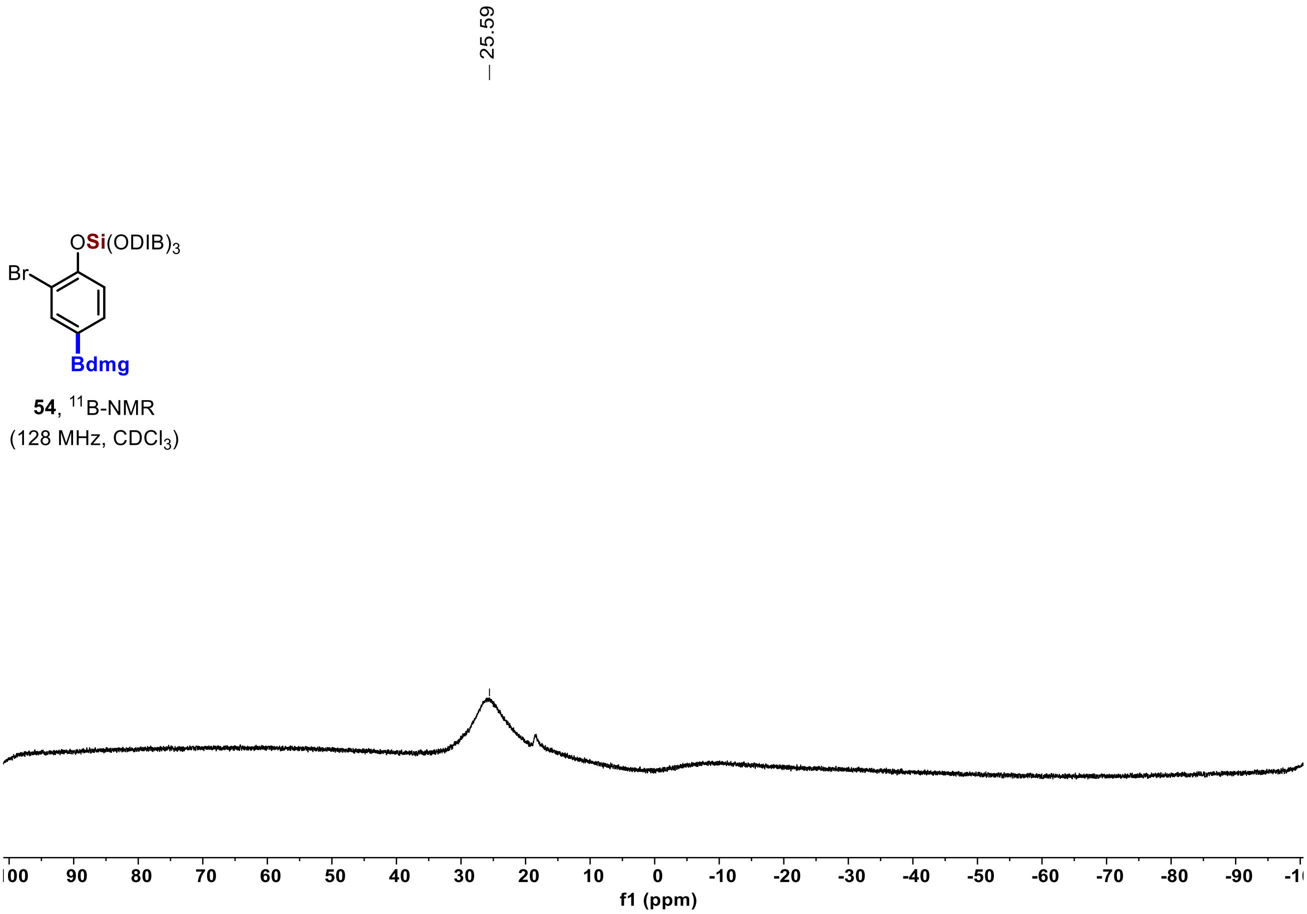

54, ^{11}B-NMR
(128 MHz, $CDCl_3$)

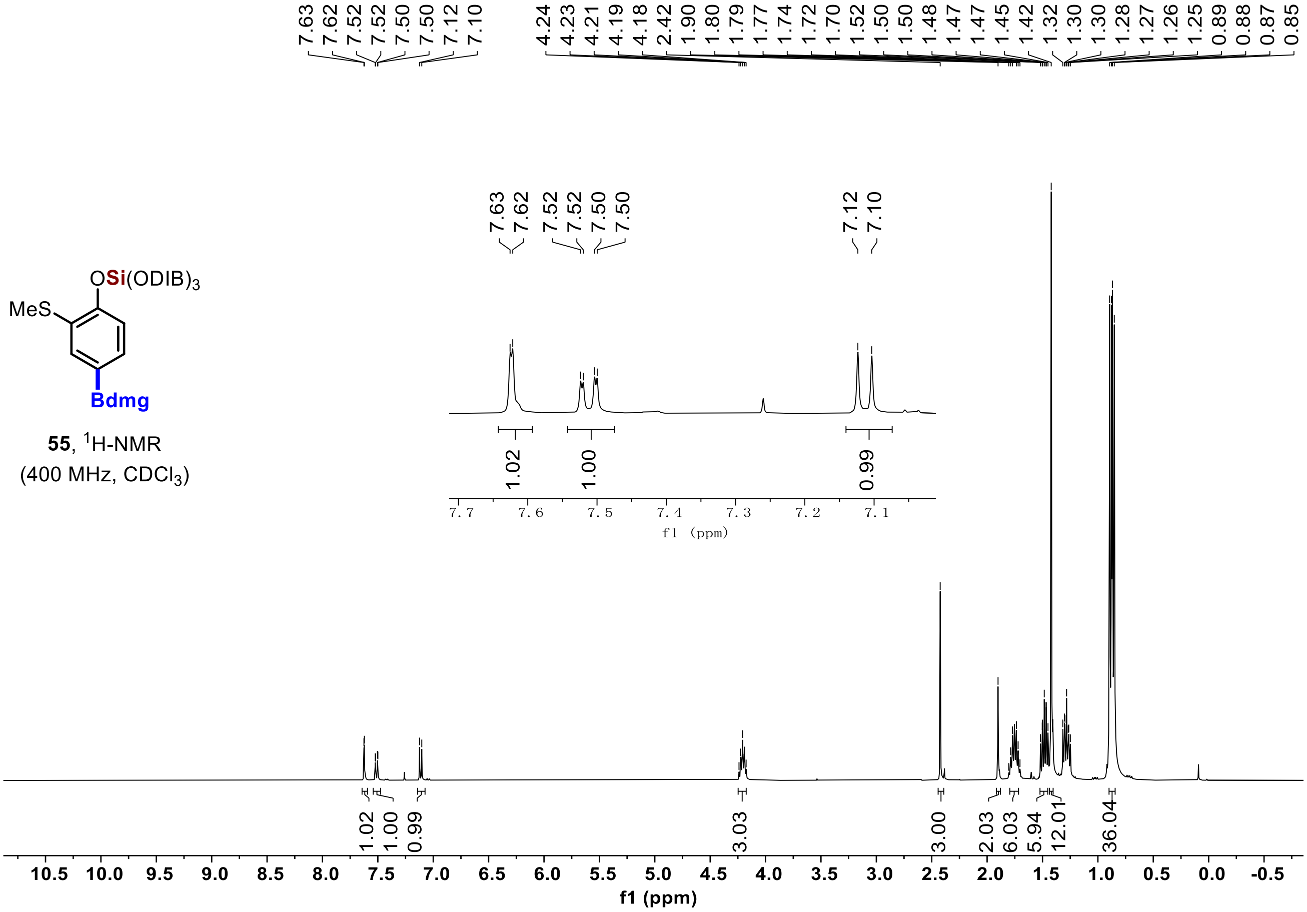

55, ^{1}H-NMR
(400 MHz, $CDCl_3$)

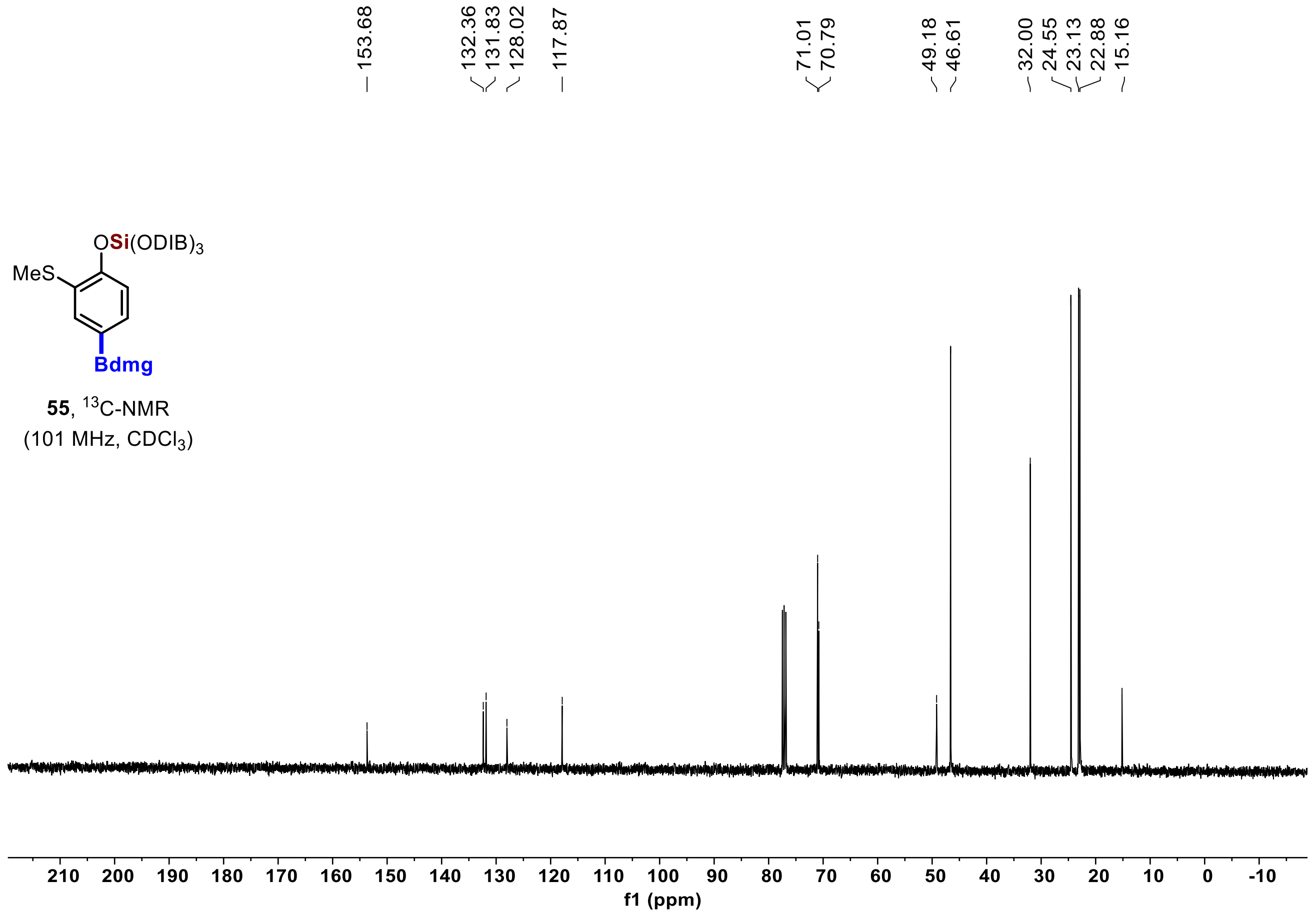

153.68
132.36
131.83
128.02
117.87
71.01
70.79
49.18
46.61
32.00
24.55
23.13
22.88
15.16
OSi(ODIB)3
MeS
Bdmg
55, 13C-NMR
(101 MHz, CDCl3)
f1 (ppm)

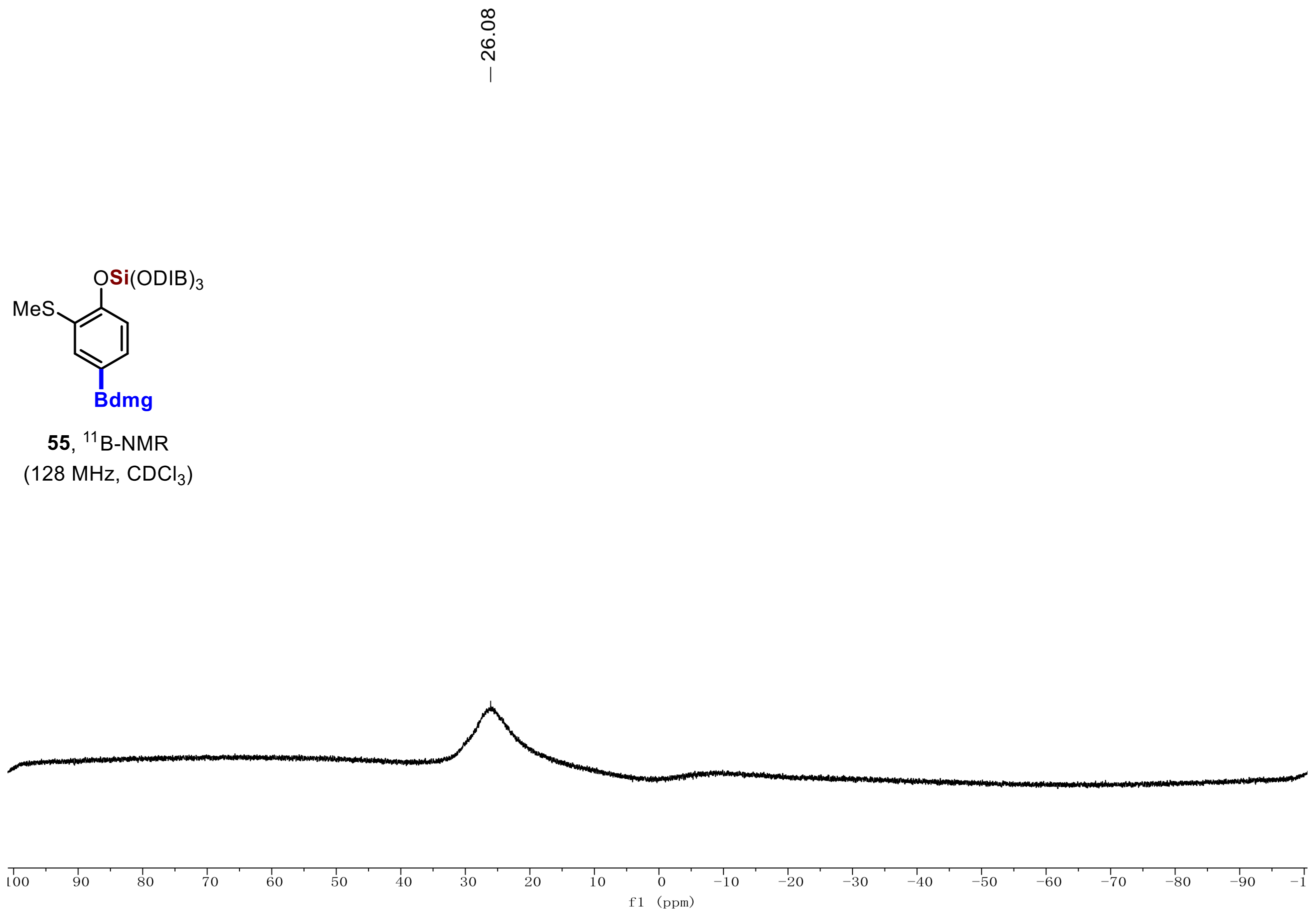

26.08
OSi(ODIB)3
MeS
Bdmg
55, 11B-NMR
(128 MHz, CDCl3)
f1 (ppm)

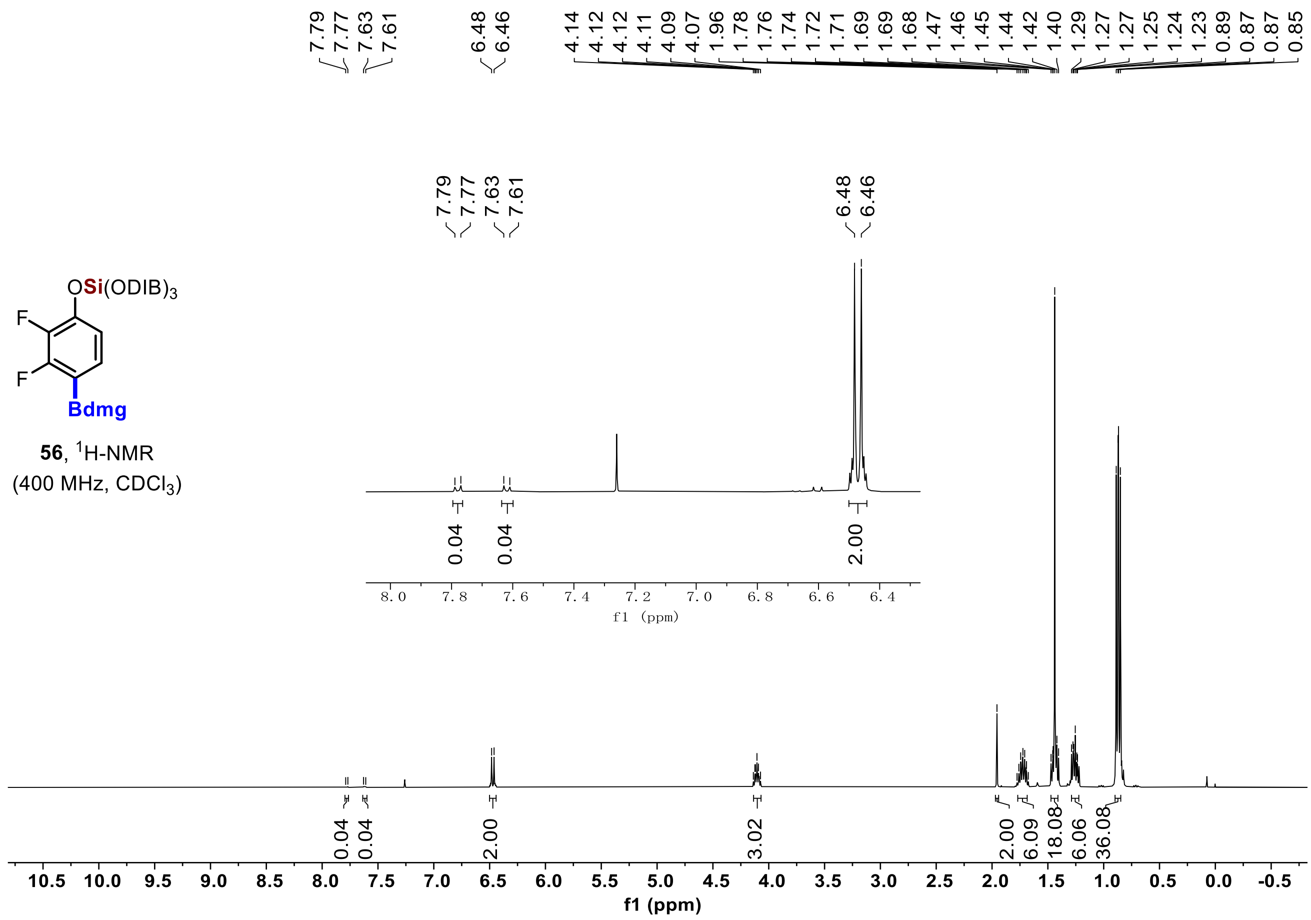
7.79
7.77
7.63
7.61
6.48
6.46
4.14
4.12
4.12
4.11
4.09
4.07
1.96
1.78
1.76
1.74
1.72
1.71
1.69
1.69
1.68
1.47
1.46
1.45
1.44
1.42
1.40
1.29
1.27
1.27
1.25
1.24
1.23
0.89
0.87
0.87
0.85
OSi(ODIB)3
F
F
Bdmg
56, 1H-NMR
(400 MHz, CDCl3)
0.04
0.04
2.00
3.02
2.00
6.09
18.08
6.06
36.08
f1 (ppm)

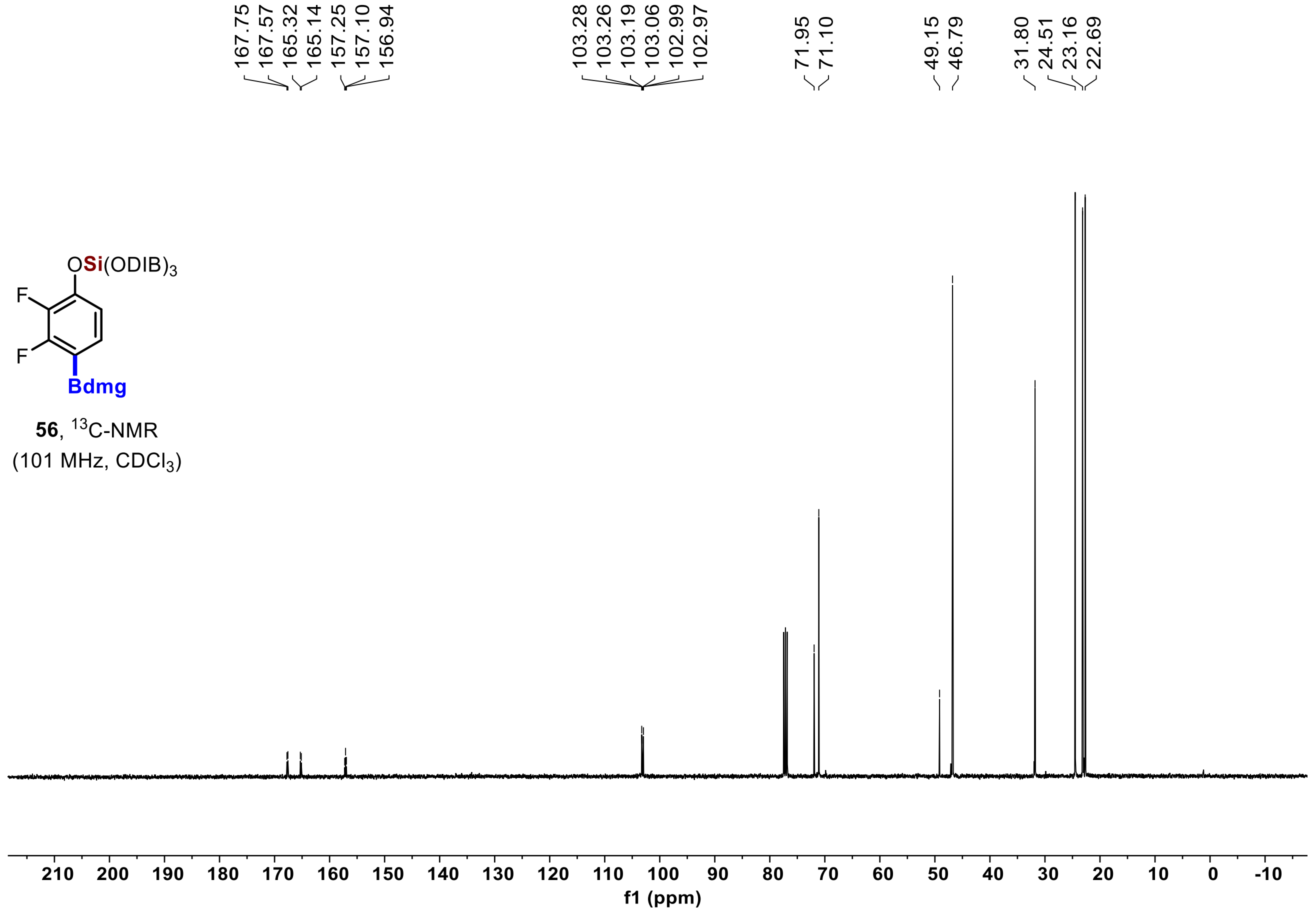
167.75
167.57
165.32
165.14
157.25
157.10
156.94
103.28
103.26
103.19
103.06
102.99
102.97
71.95
71.10
49.15
46.79
31.80
24.51
23.16
22.69
OSi(ODIB)3
F
F
Bdmg
56, 13C-NMR
(101 MHz, CDCl3)
f1 (ppm)

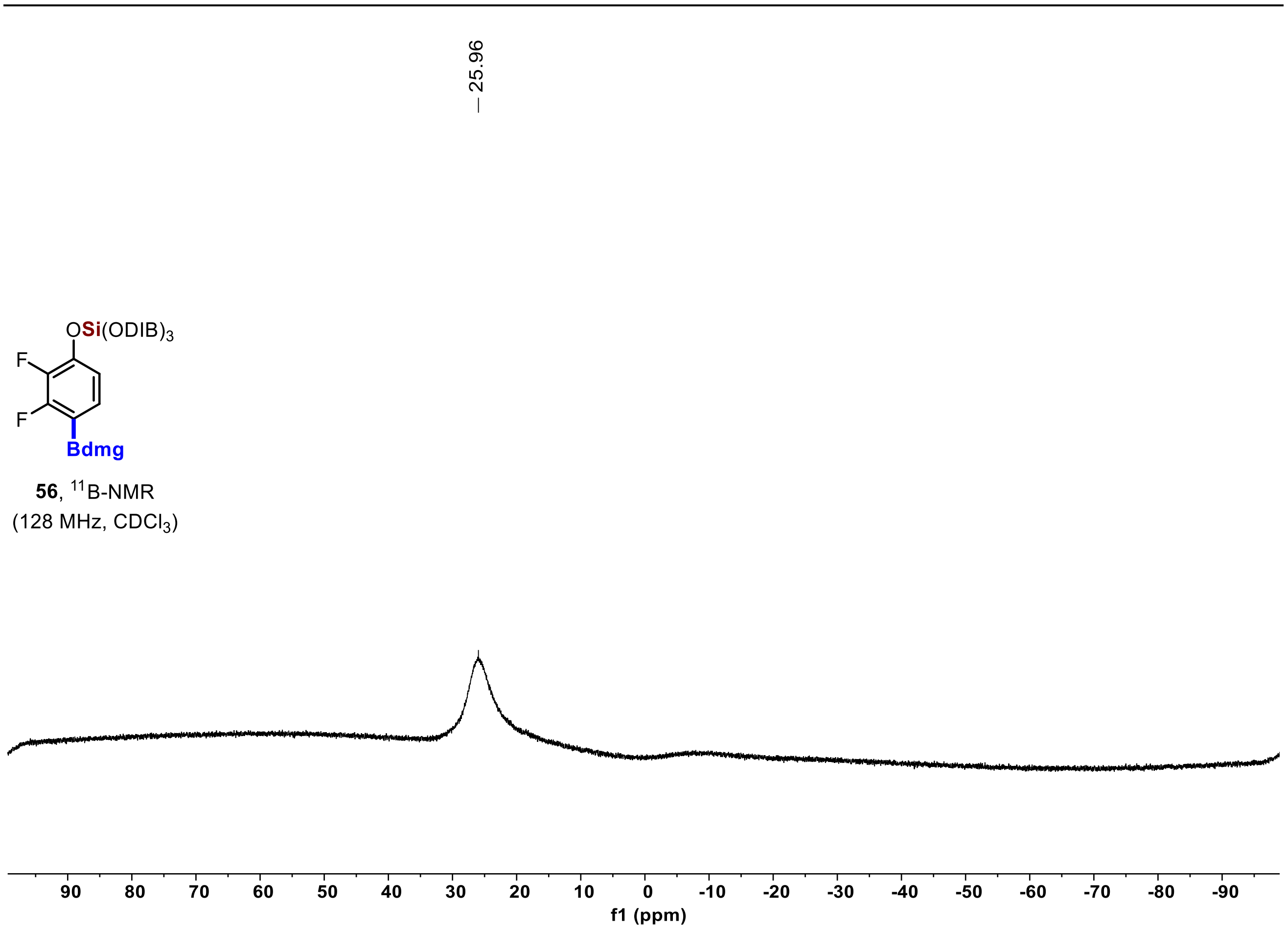

56, ^{11}B-NMR
(128 MHz, $CDCl_3$)

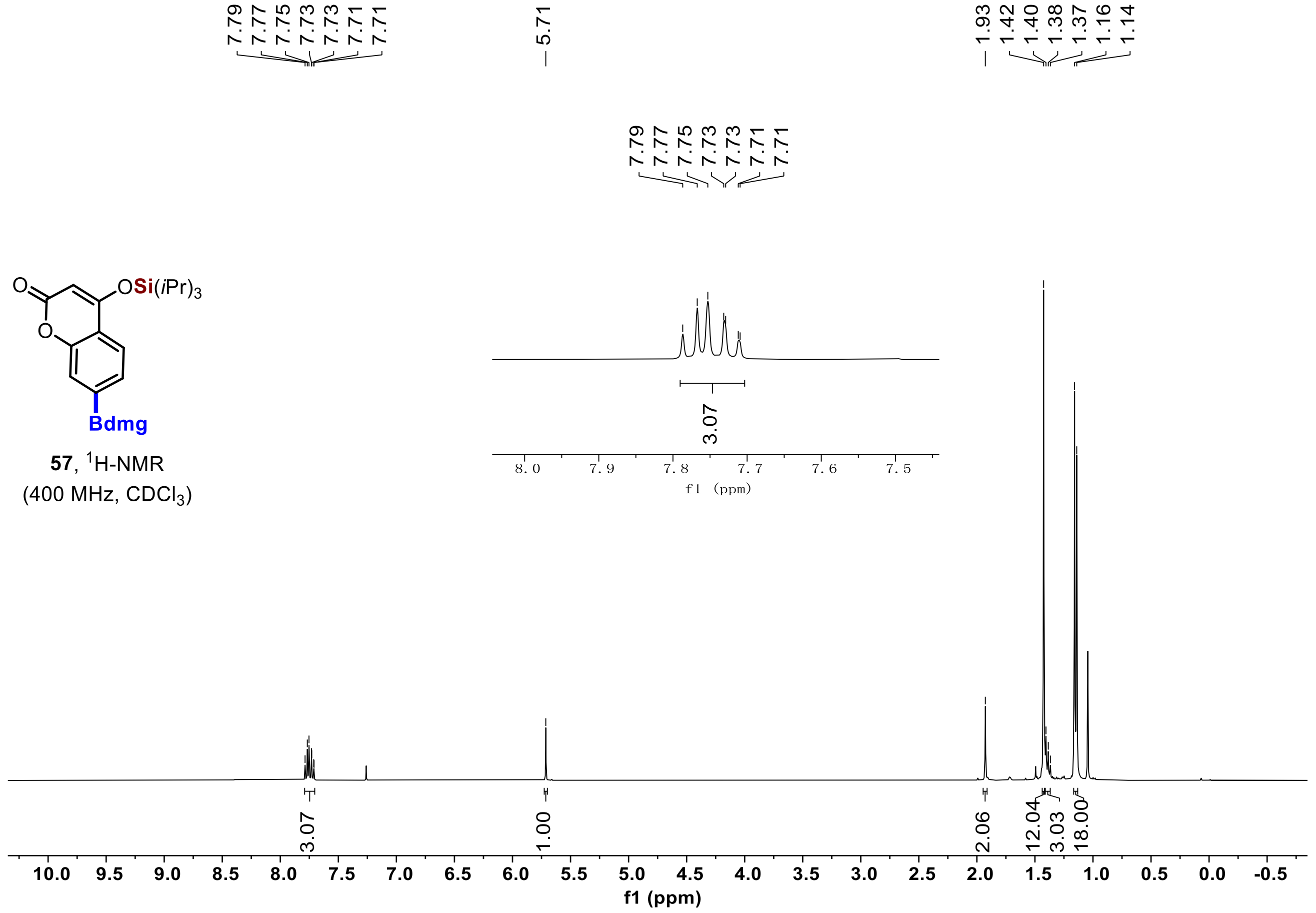

57, ^{1}H-NMR
(400 MHz, $CDCl_3$)

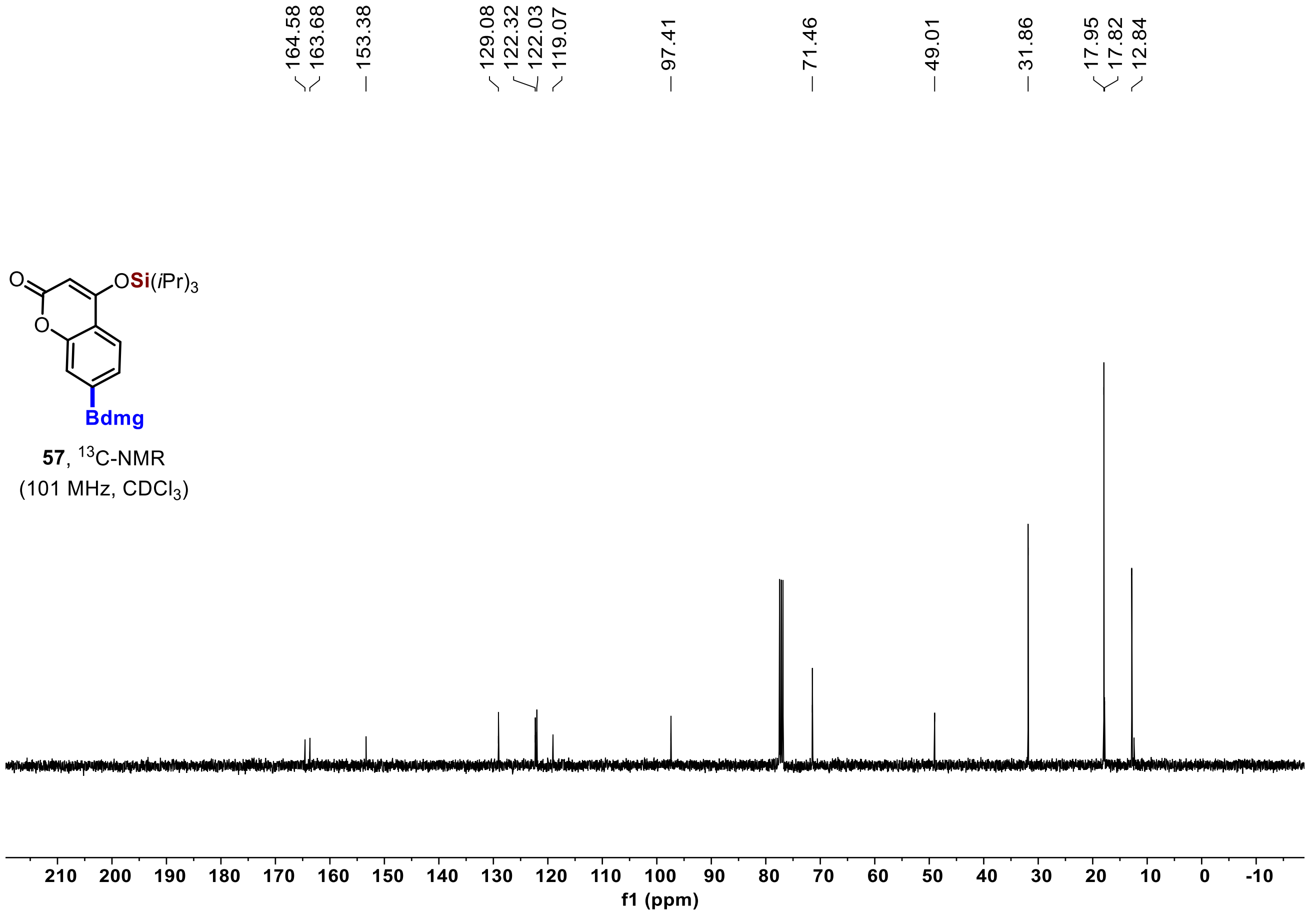

57, ^{13}C-NMR
(101 MHz, $CDCl_3$)

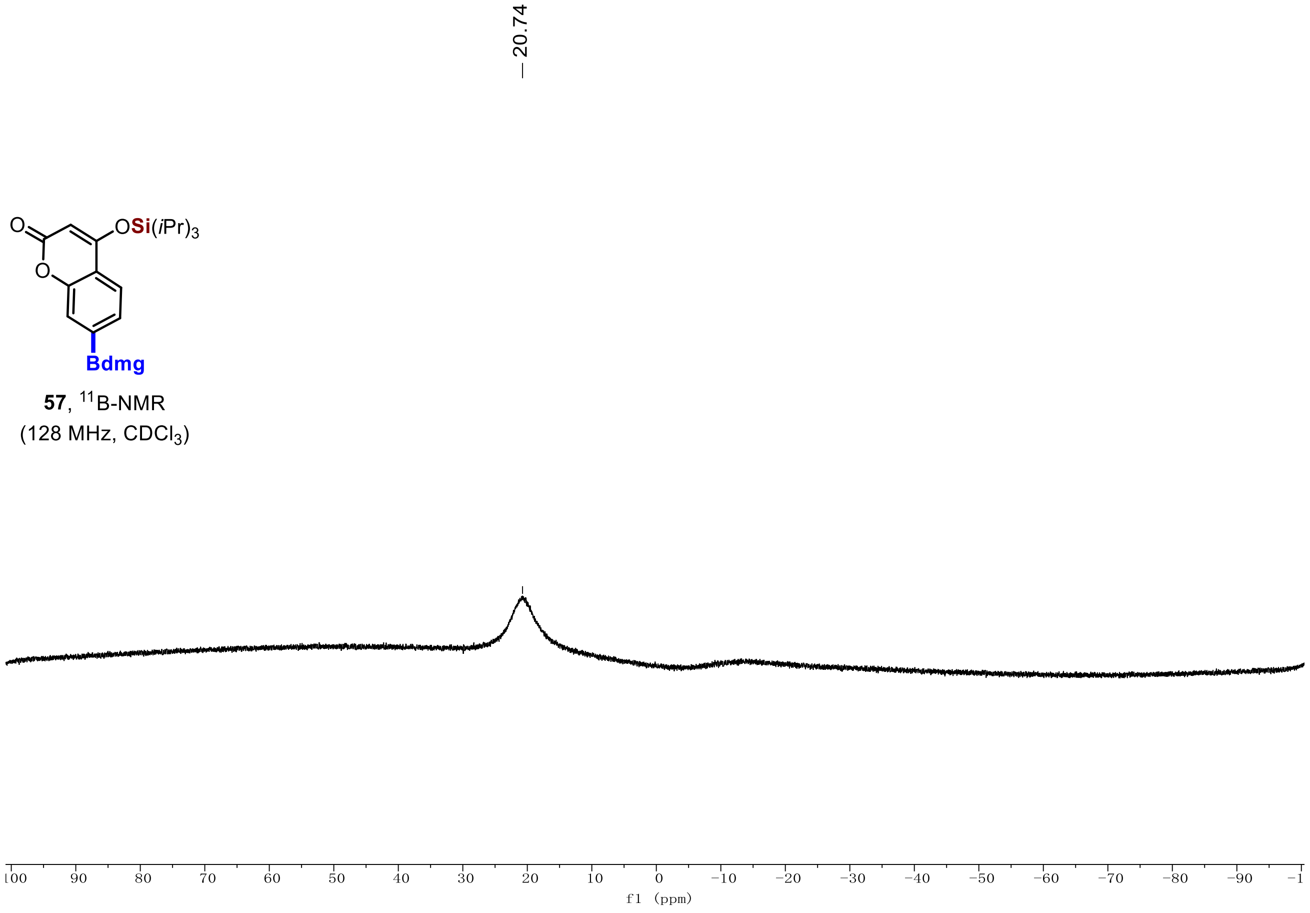

57, ^{11}B-NMR
(128 MHz, $CDCl_3$)

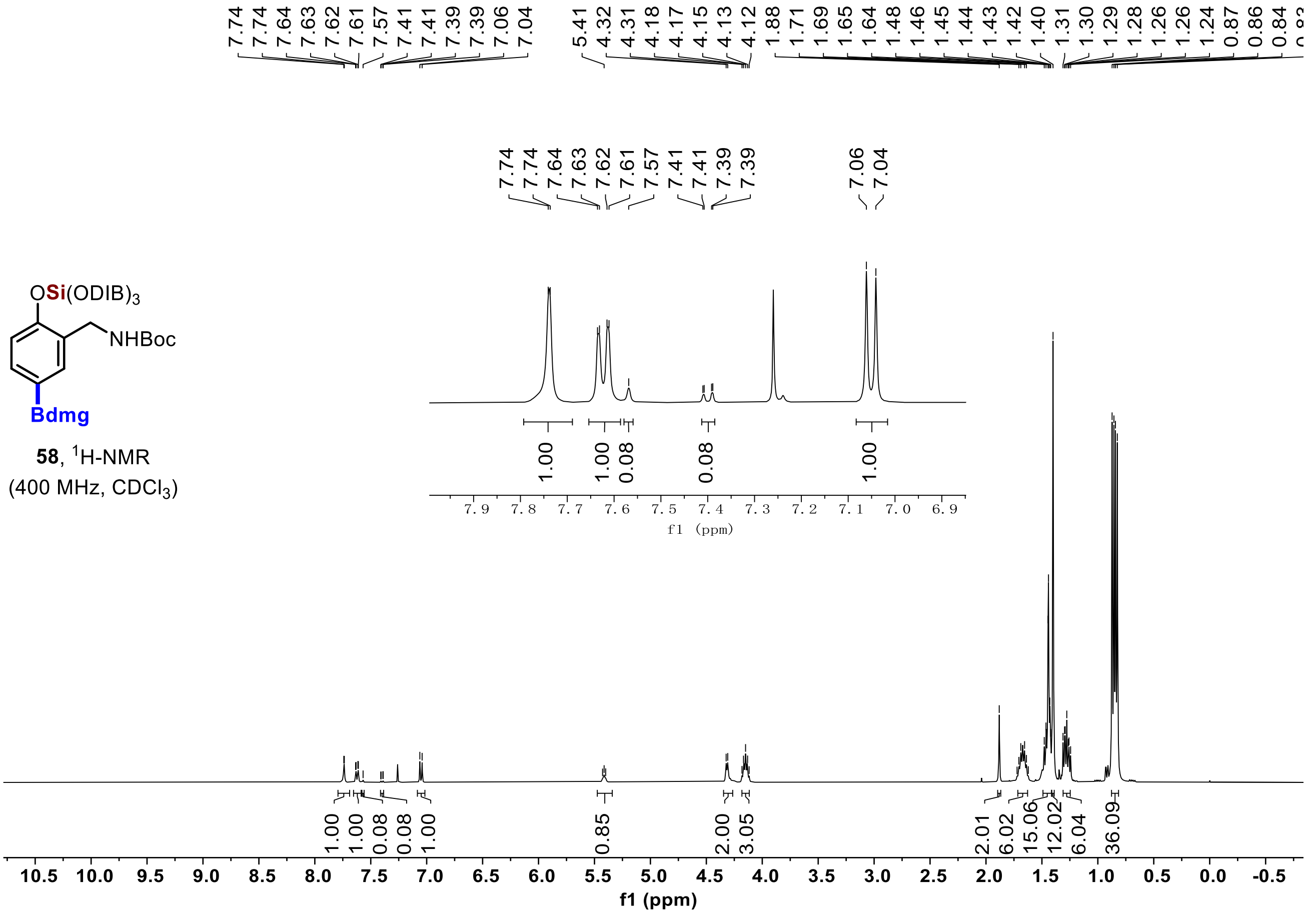

58, ^{1}H-NMR
(400 MHz, $CDCl_3$)

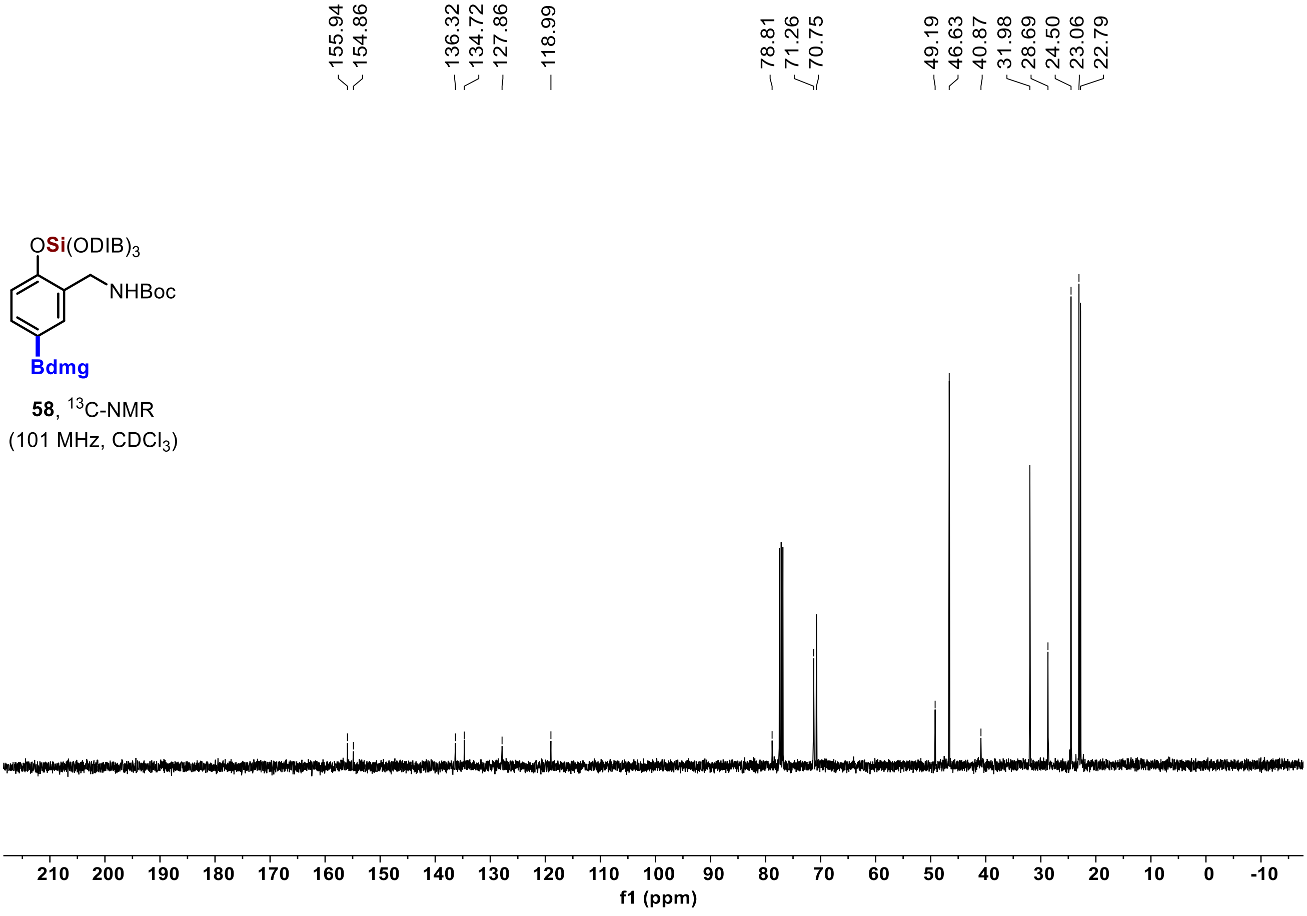

58, ^{13}C-NMR
(101 MHz, $CDCl_3$)

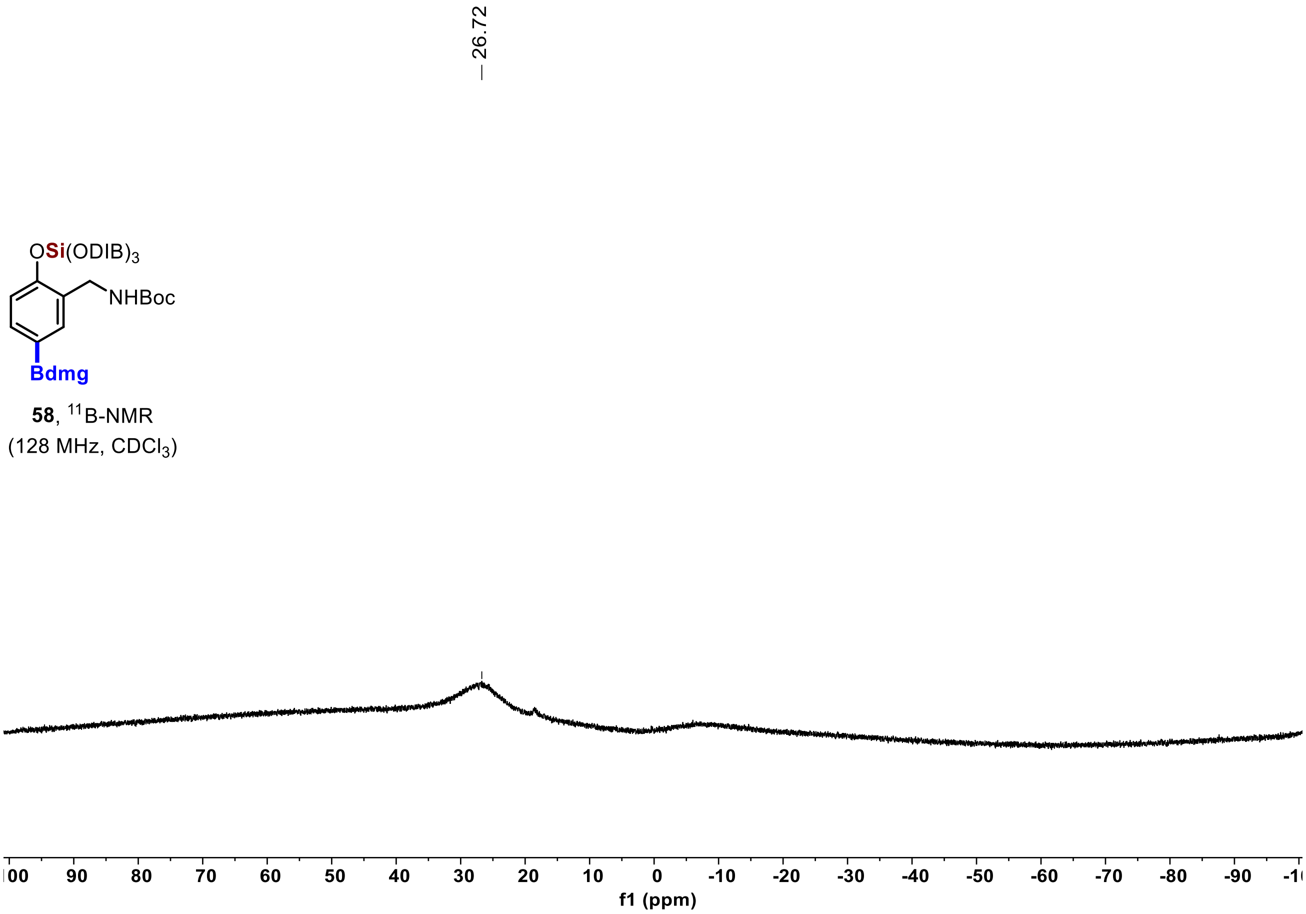

OSi(ODIB)3
NHBoc
Bdmg
58, 11B-NMR
(128 MHz, CDCl3)
26.72
f1 (ppm)

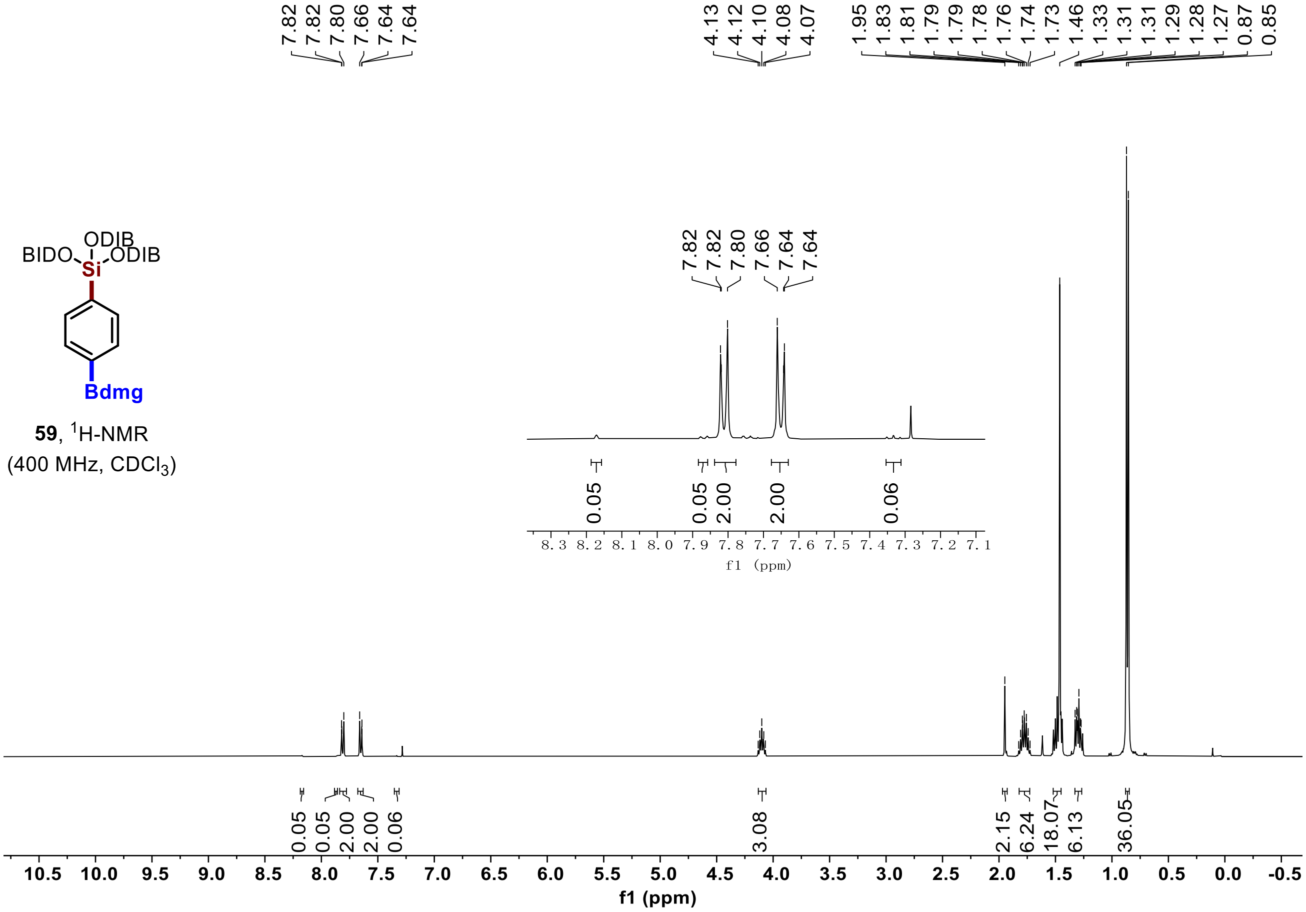

BIDO
ODIB
ODIB
Si
Bdmg
59, 1H-NMR
(400 MHz, CDCl3)
7.82
7.82
7.80
7.66
7.64
7.64
4.13
4.12
4.10
4.08
4.07
1.95
1.83
1.81
1.79
1.79
1.78
1.76
1.74
1.73
1.46
1.33
1.31
1.31
1.29
1.28
1.27
0.87
0.85
0.05
0.05
2.00
2.00
0.06
3.08
2.15
6.24
18.07
6.13
36.05
f1 (ppm)

59, ^{13}C-NMR
(101 MHz, $CDCl_3$)

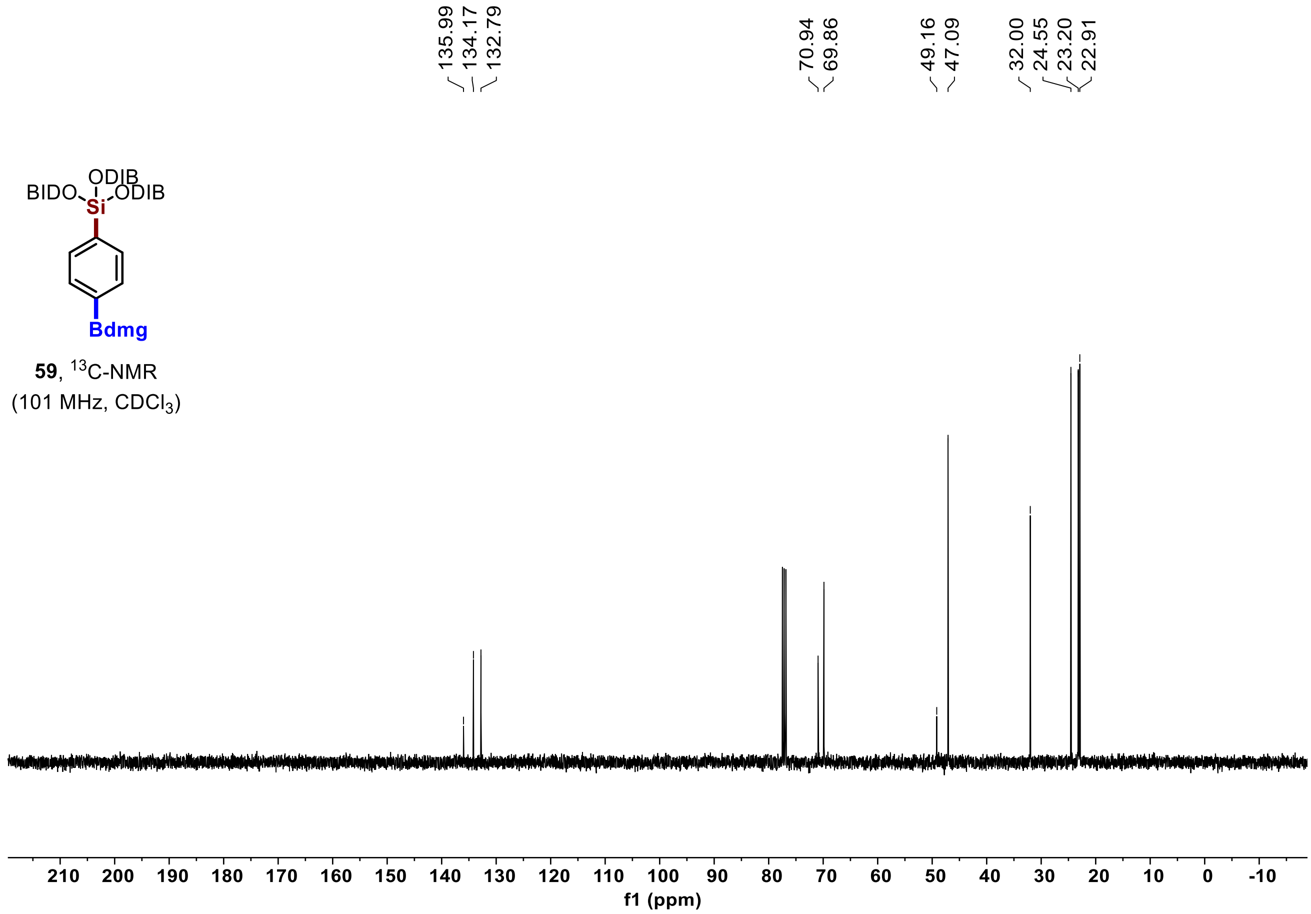

60, ^{1}H-NMR
(400 MHz, $CDCl_3$)

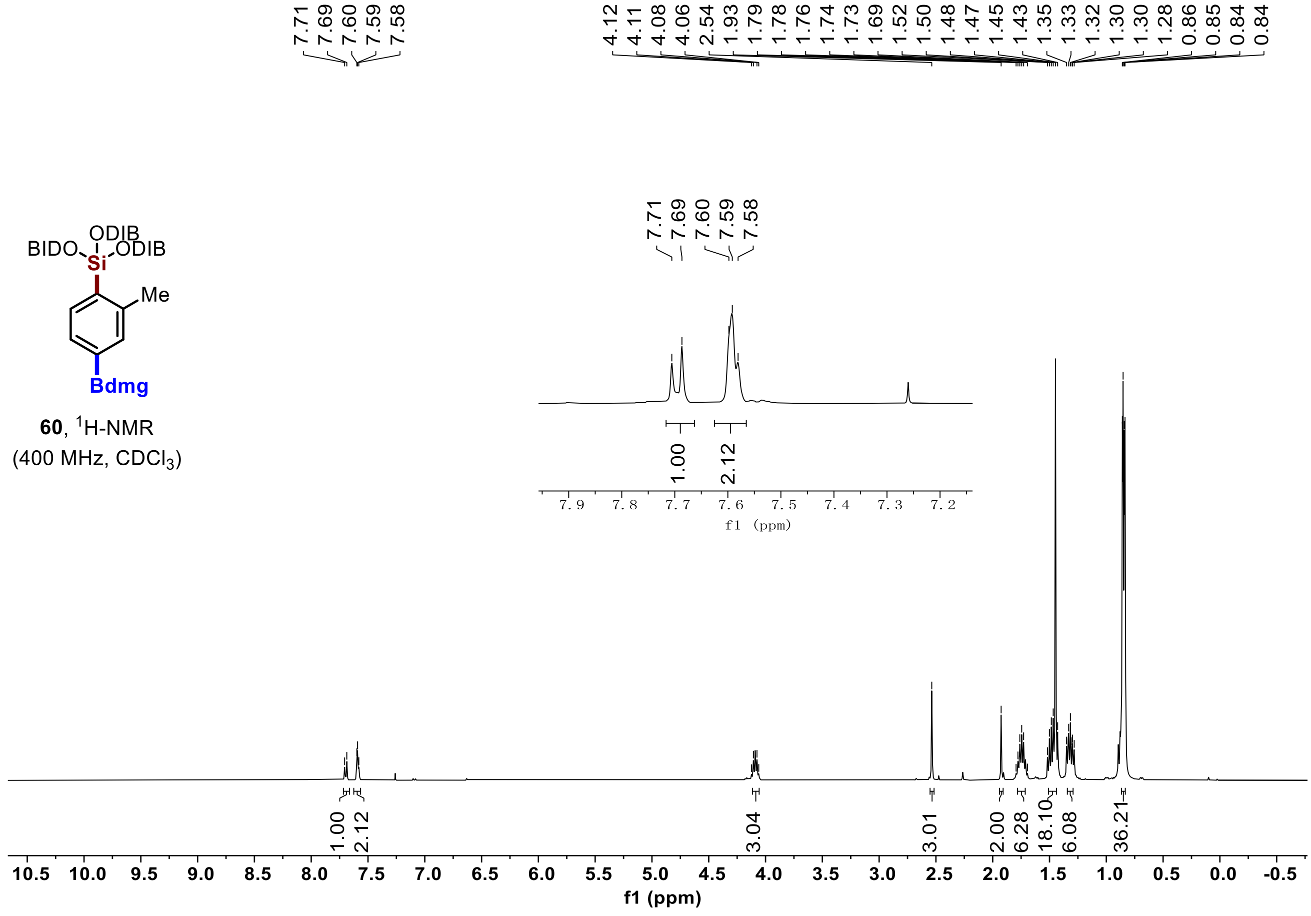

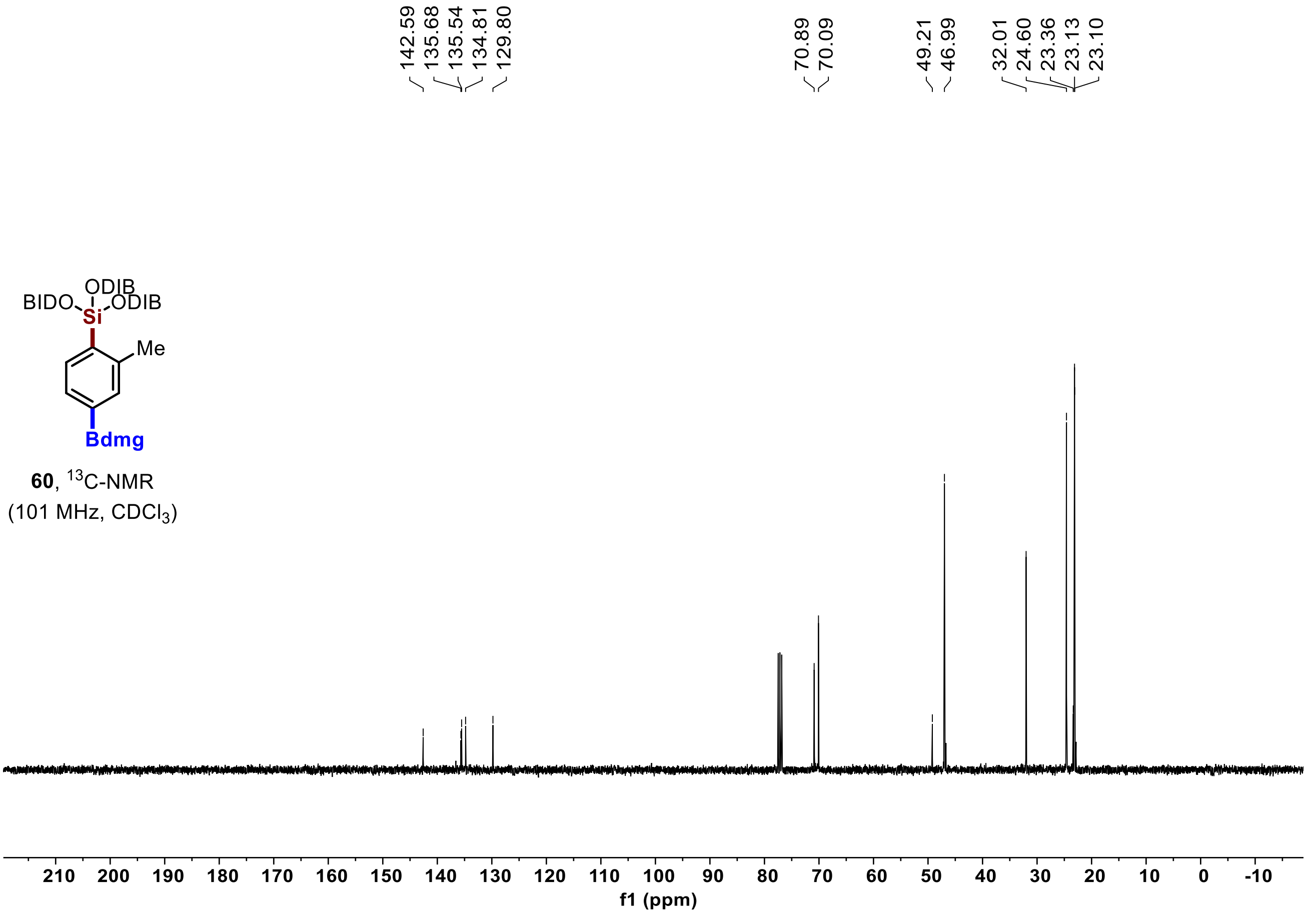

60, ^{13}C-NMR
(101 MHz, $CDCl_3$)

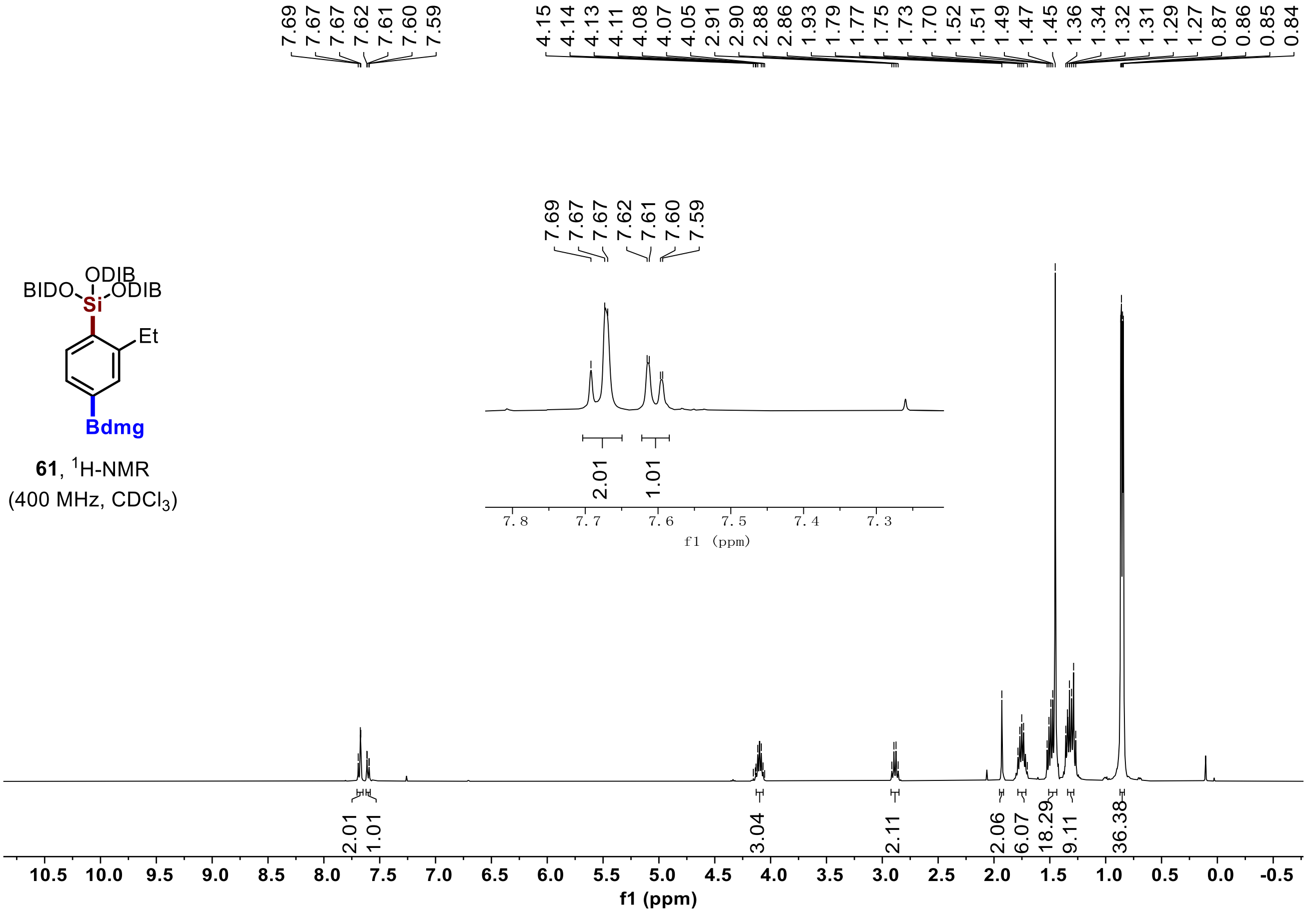

61, ^{1}H-NMR
(400 MHz, $CDCl_3$)

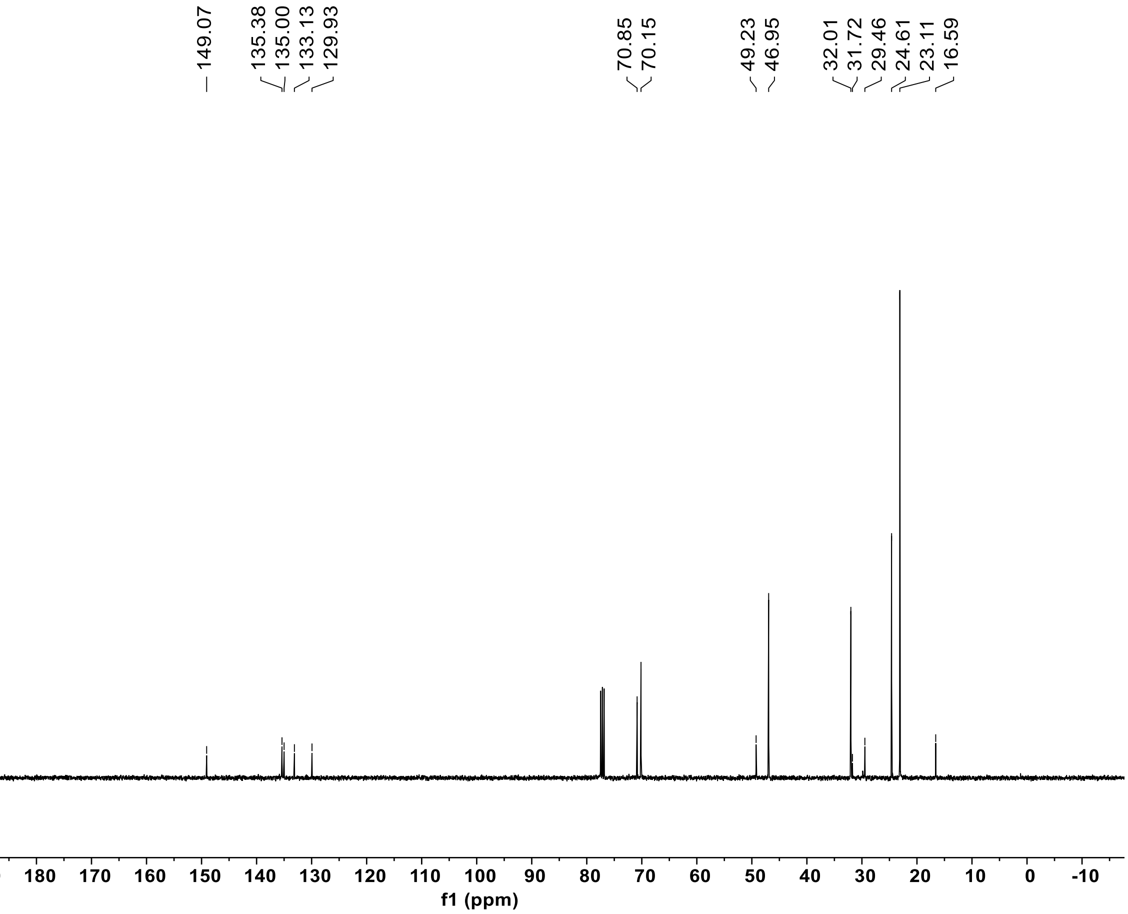

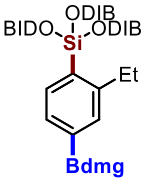

61, ^{13}C-NMR
(101 MHz, $CDCl_3$)

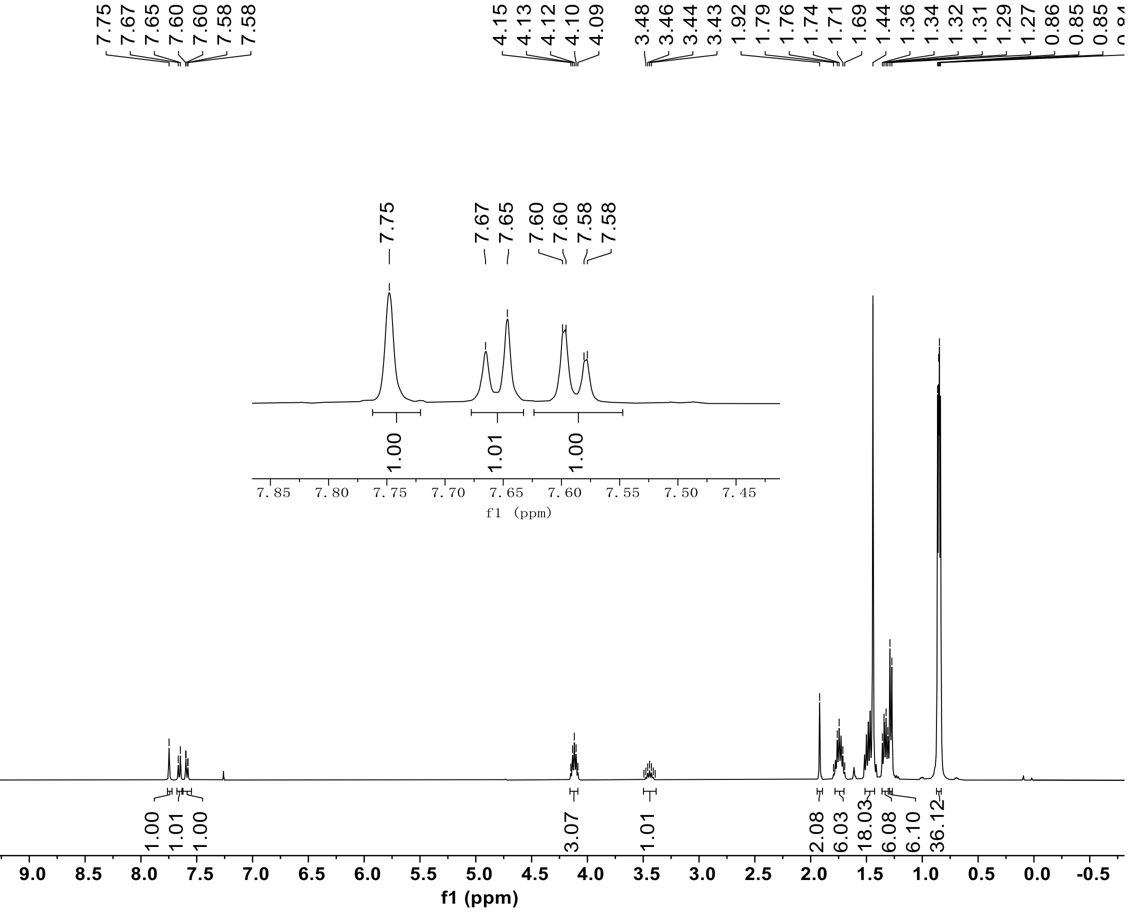

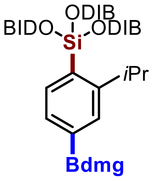

62, ^{1}H-NMR
(400 MHz, $CDCl_3$)

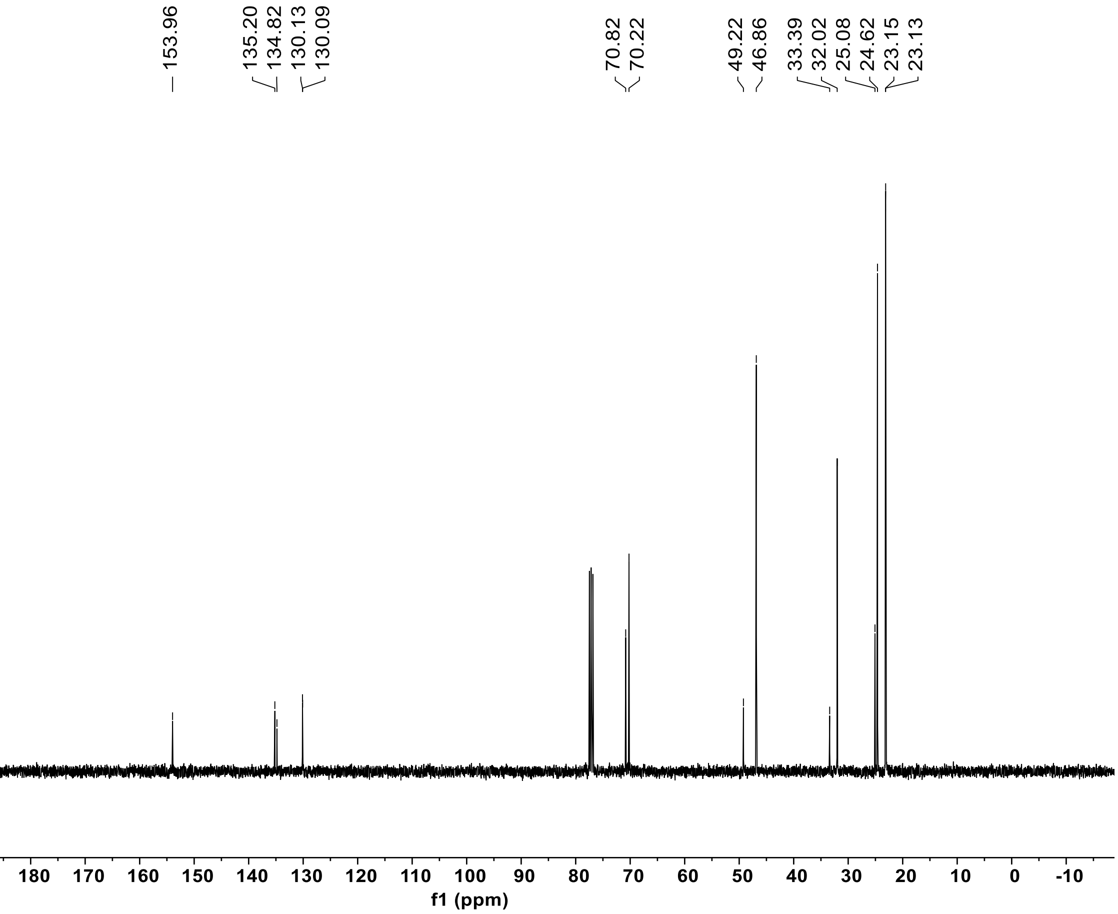

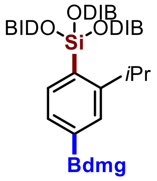

62, ^{13}C-NMR
(101 MHz, $CDCl_3$)

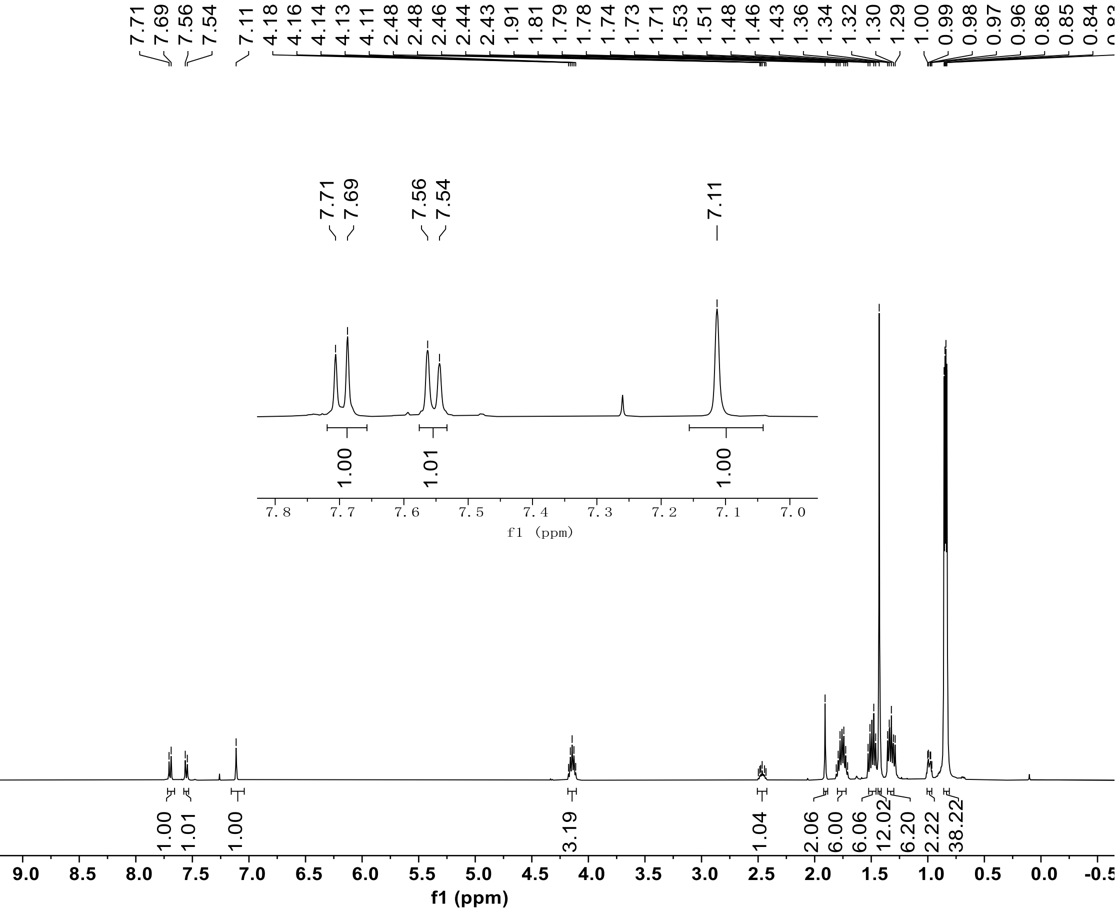

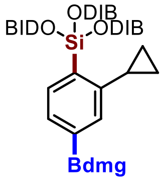

63, ^{1}H-NMR
(400 MHz, $CDCl_3$)

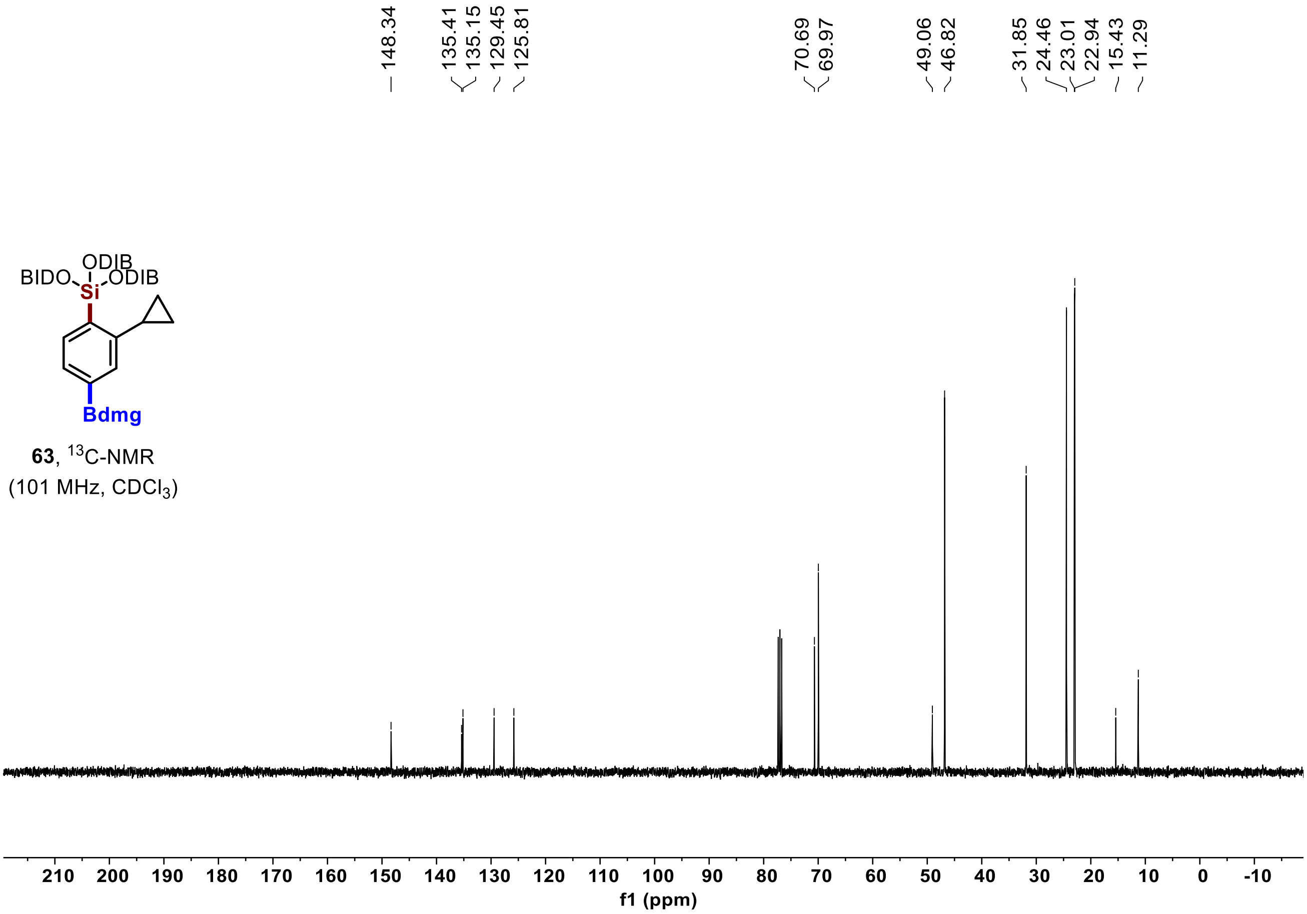

63, ^{13}C-NMR
(101 MHz, $CDCl_3$)

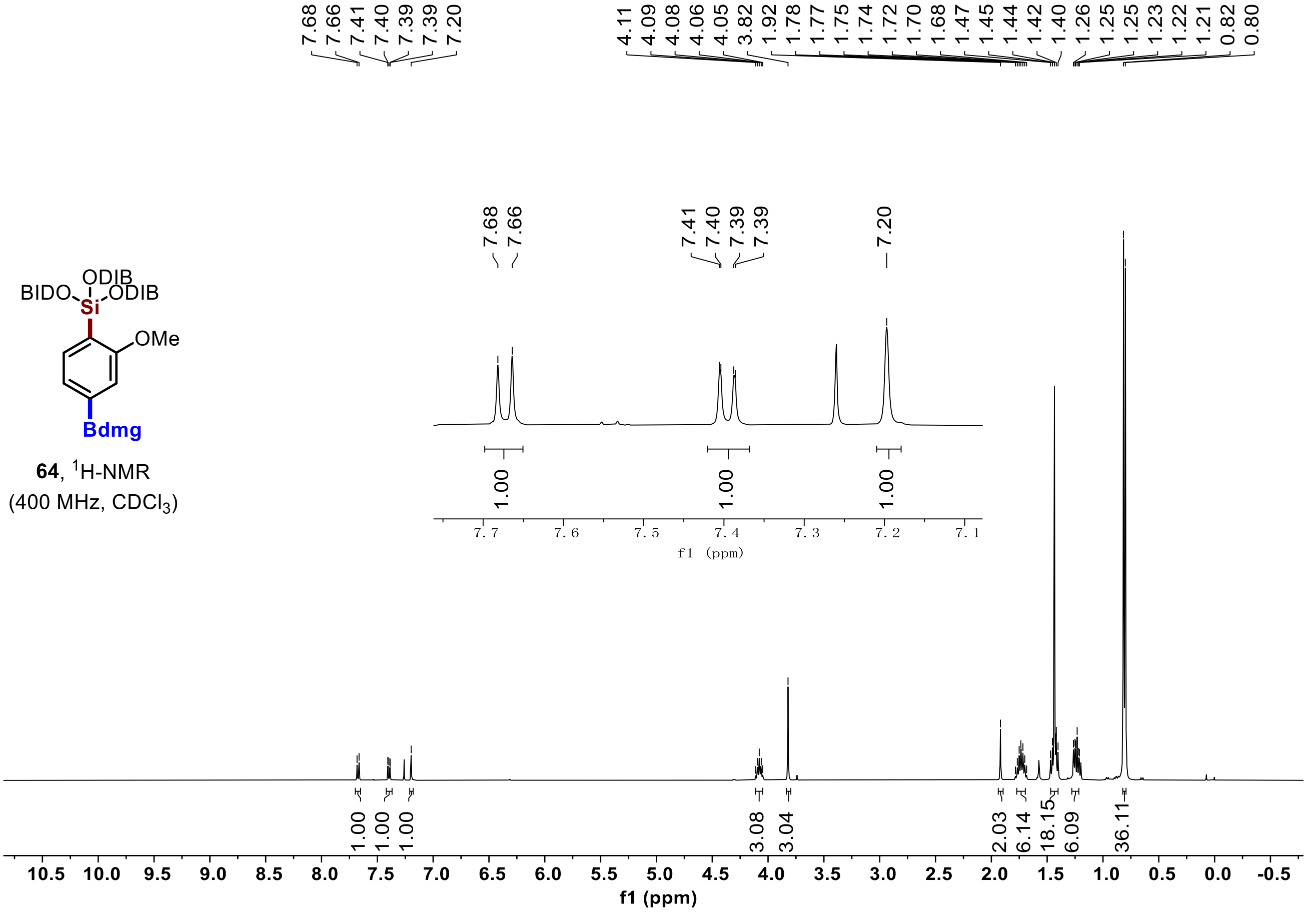

64, ^{1}H-NMR
(400 MHz, $CDCl_3$)

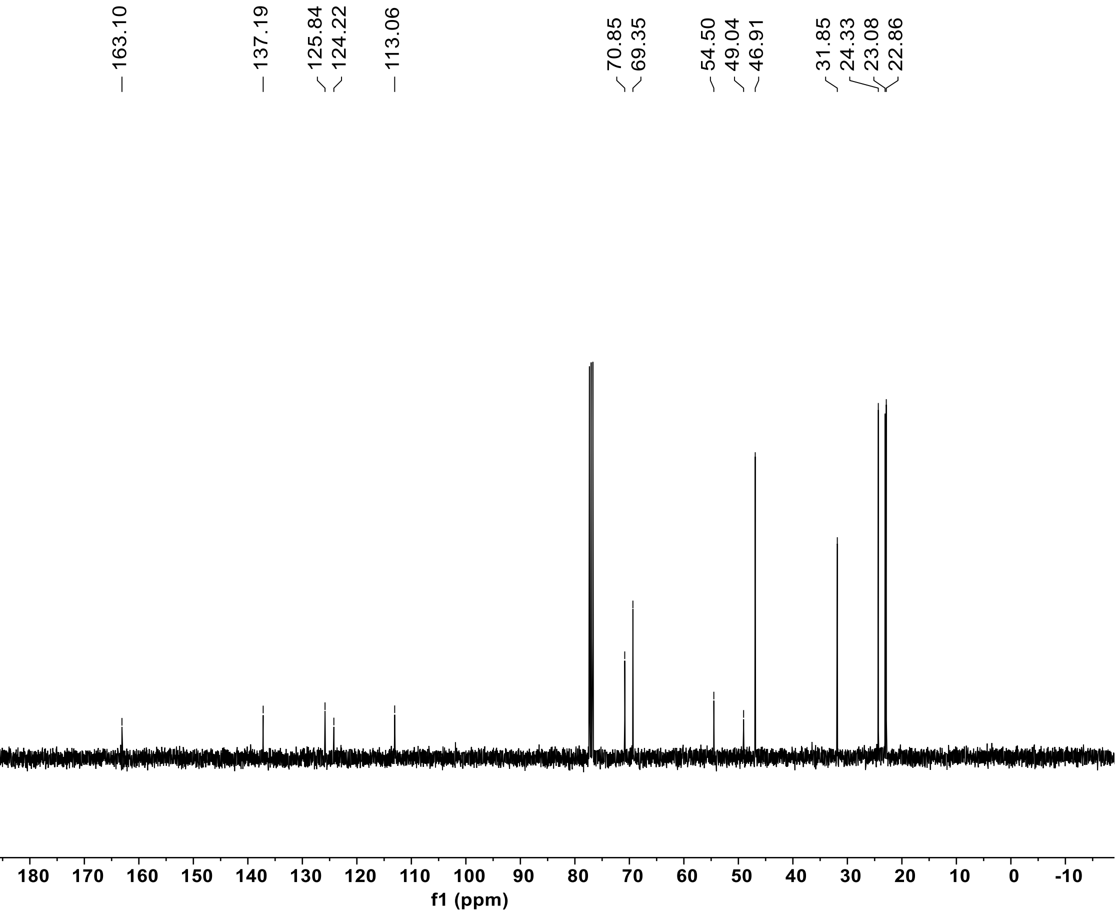

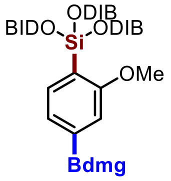

64, ^{13}C-NMR
(101 MHz, $CDCl_3$)

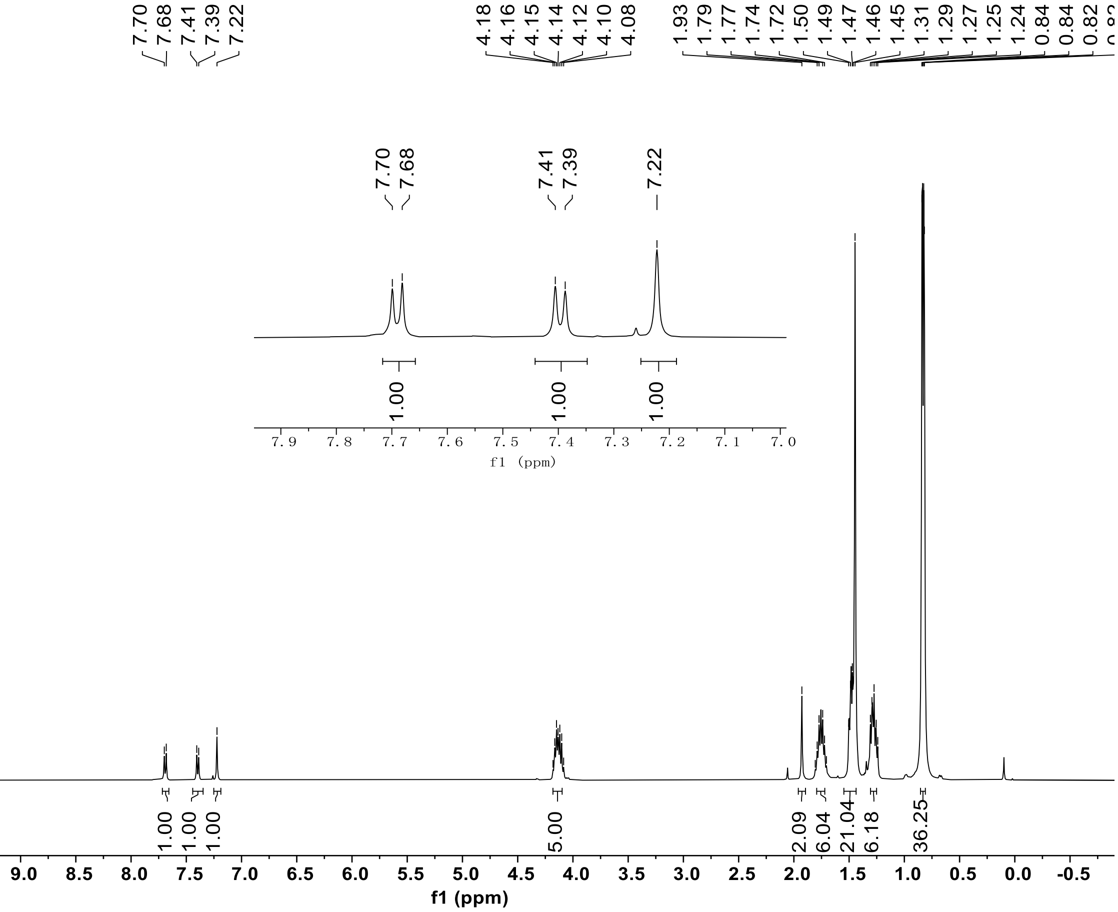

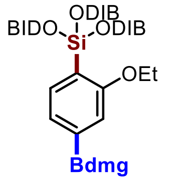

65, ^{1}H-NMR
(400 MHz, $CDCl_3$)

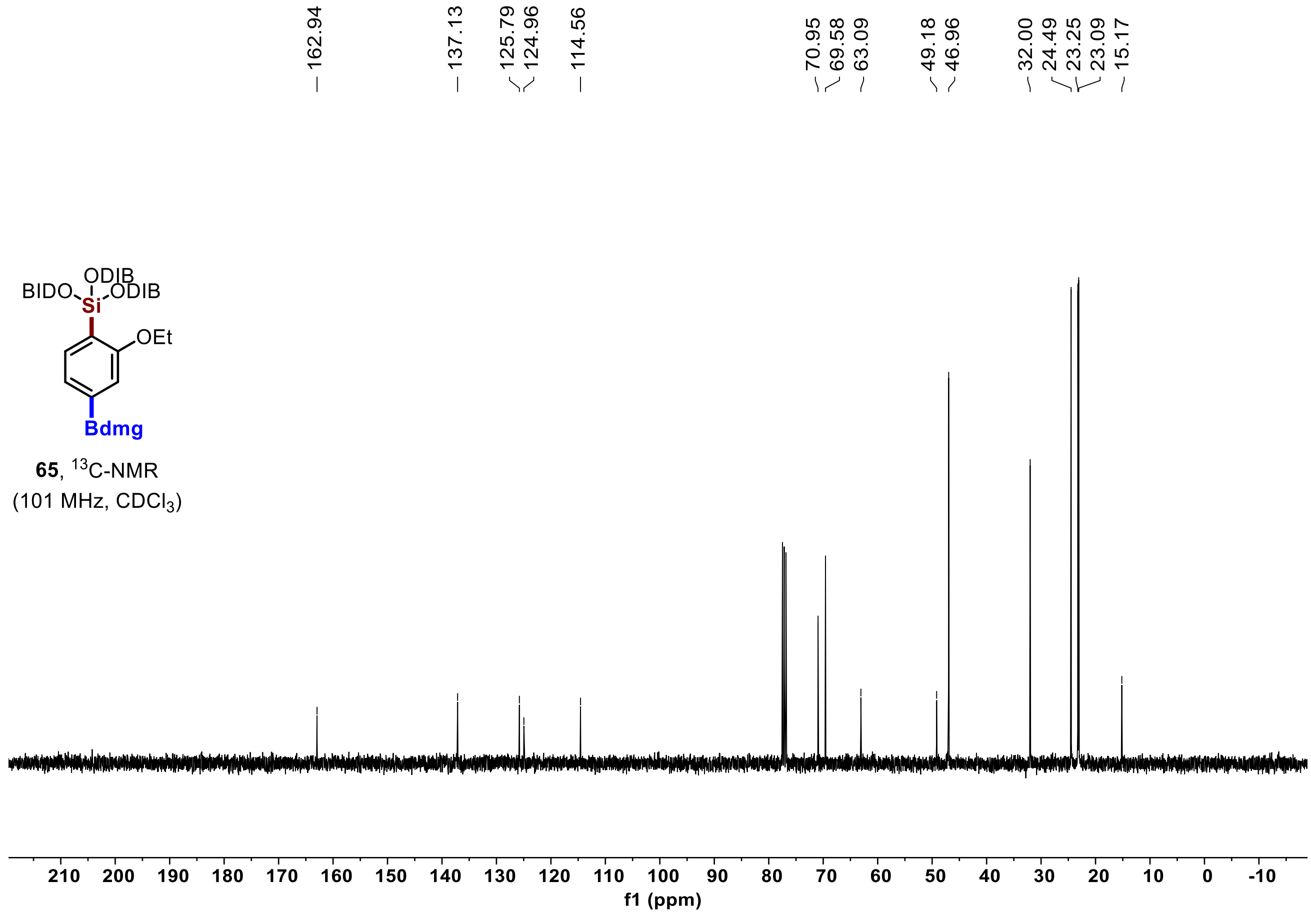

65, ^{13}C-NMR
(101 MHz, $CDCl_3$)

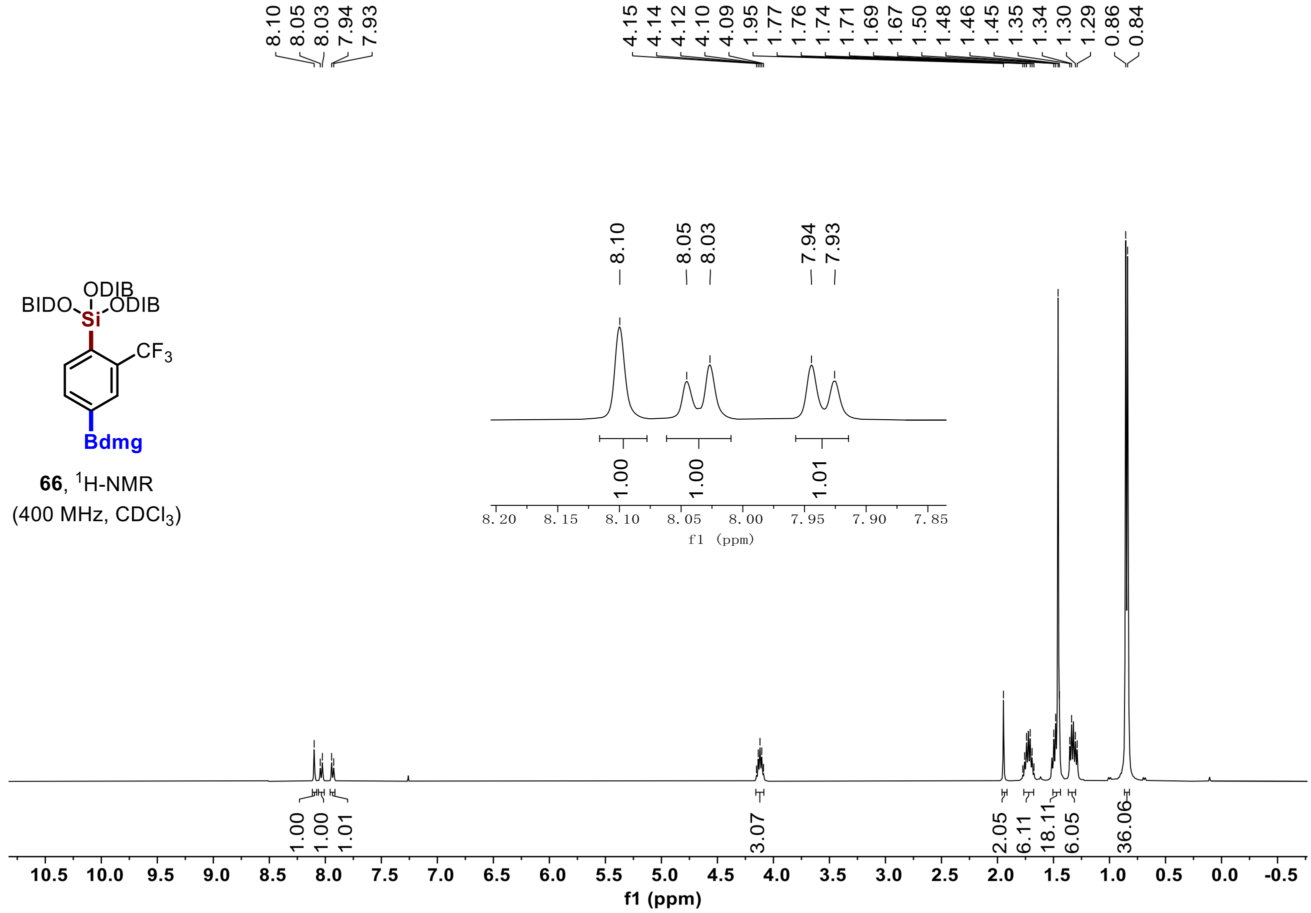

66, ^{1}H-NMR
(400 MHz, $CDCl_3$)

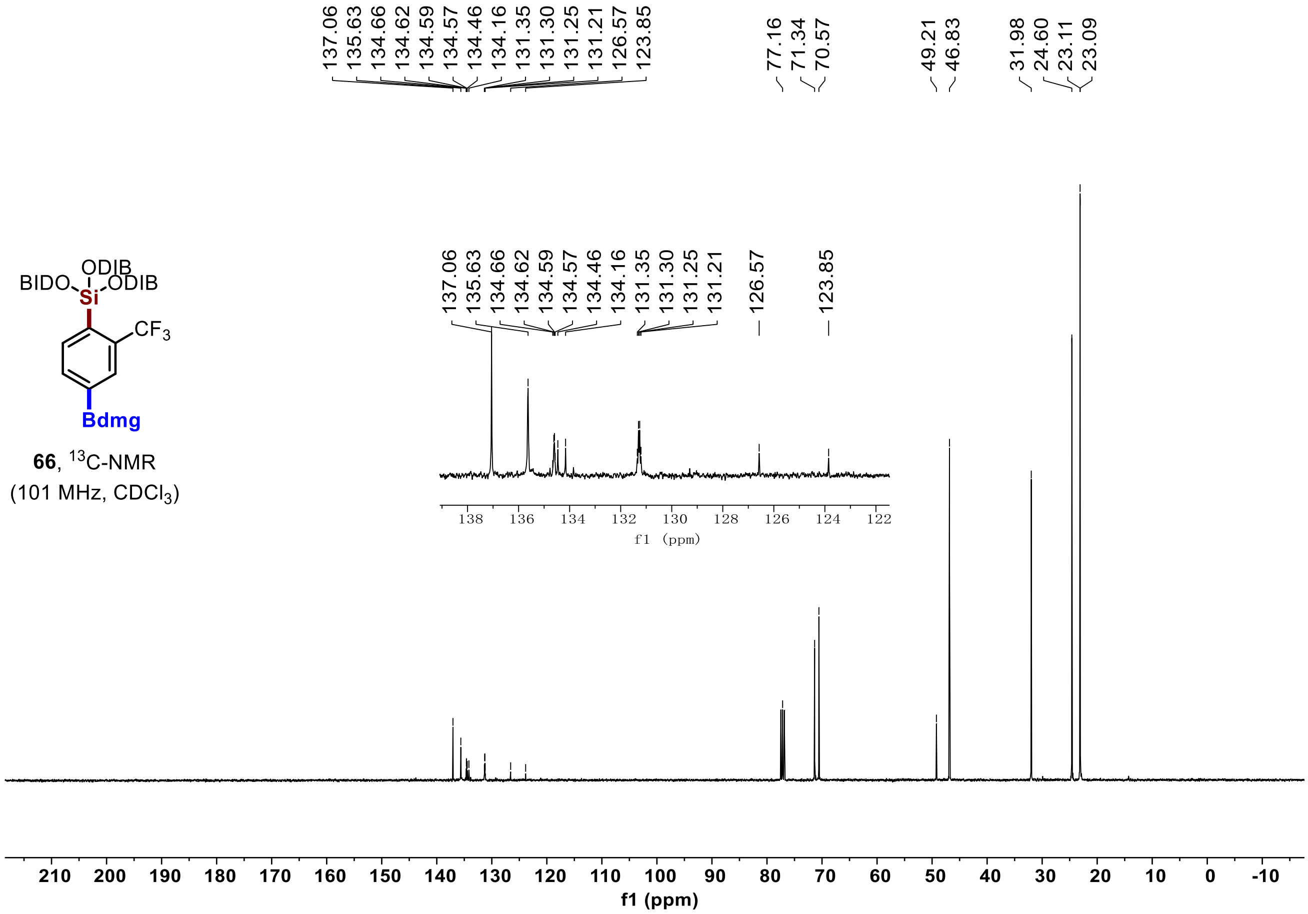
137.06
135.63
134.66
134.62
134.59
134.57
134.46
134.16
131.35
131.30
131.25
131.21
126.57
123.85
77.16
71.34
70.57
49.21
46.83
31.98
24.60
23.11
23.09
BIDO
ODIB
ODIB
Si
CF3
Bdmg
66, 13C-NMR
(101 MHz, CDCl3)
f1 (ppm)

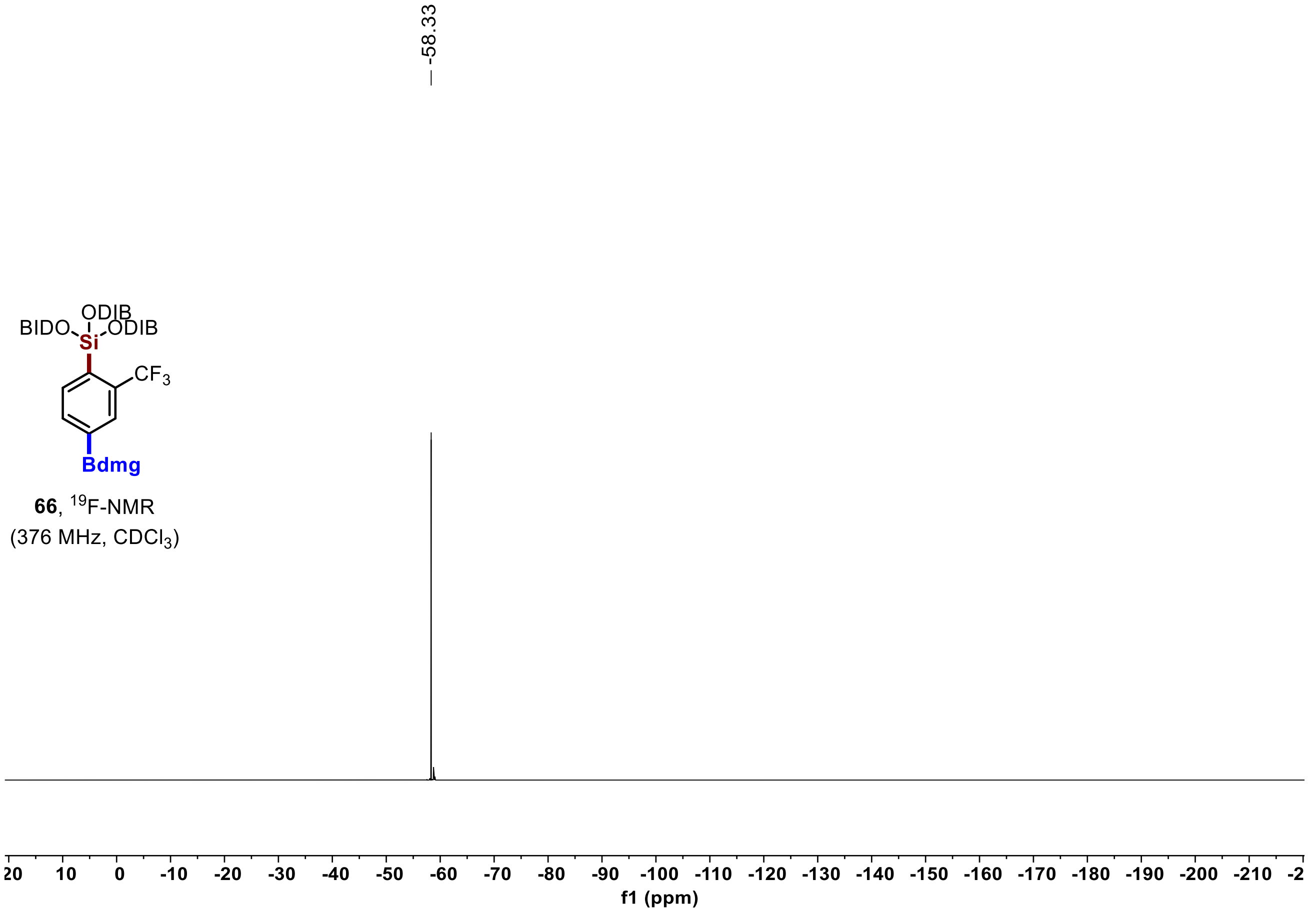
-58.33
BIDO
ODIB
ODIB
Si
CF3
Bdmg
66, 19F-NMR
(376 MHz, CDCl3)
f1 (ppm)

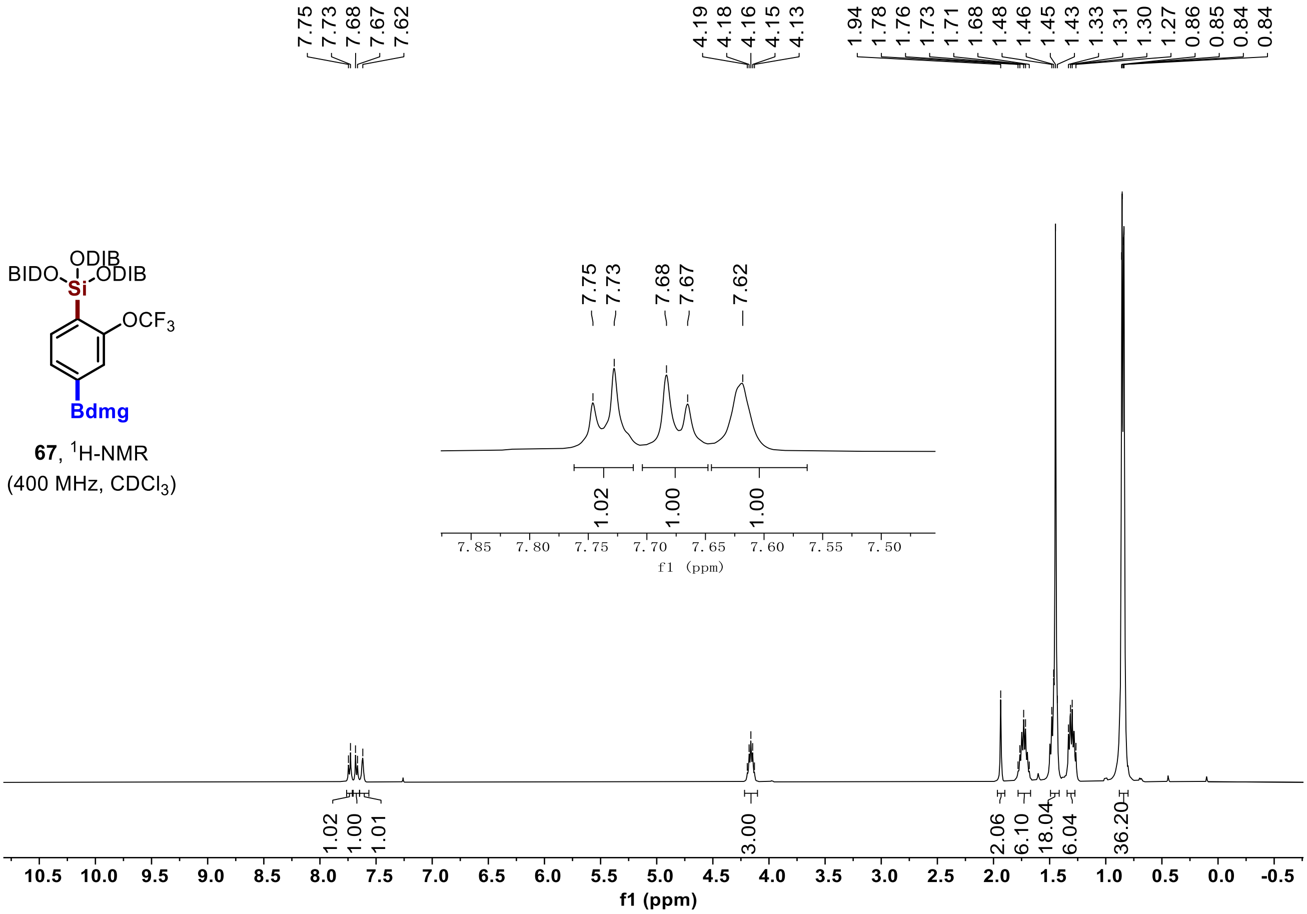

67, ^{1}H-NMR
(400 MHz, $CDCl_3$)

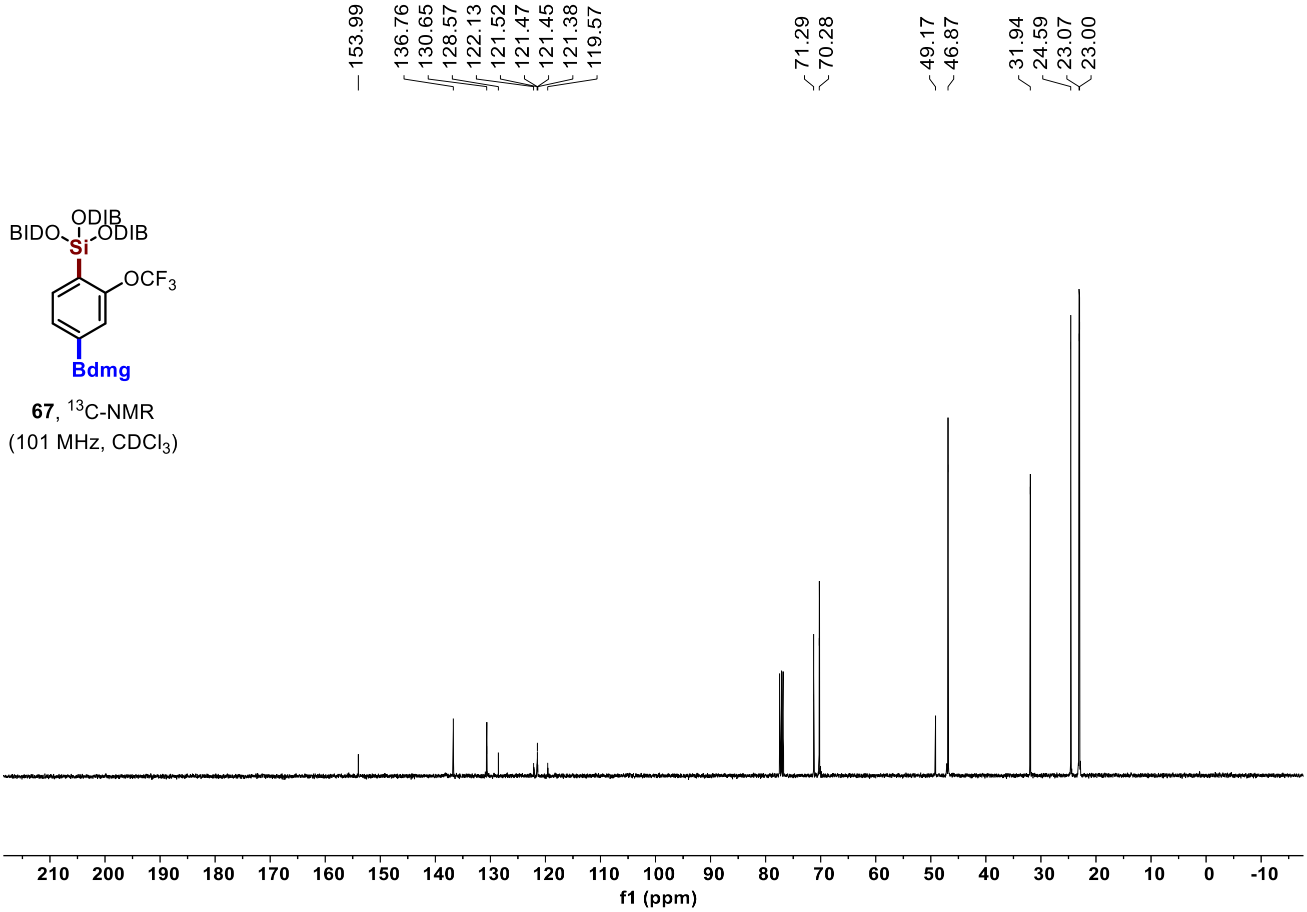

67, ^{13}C-NMR
(101 MHz, $CDCl_3$)

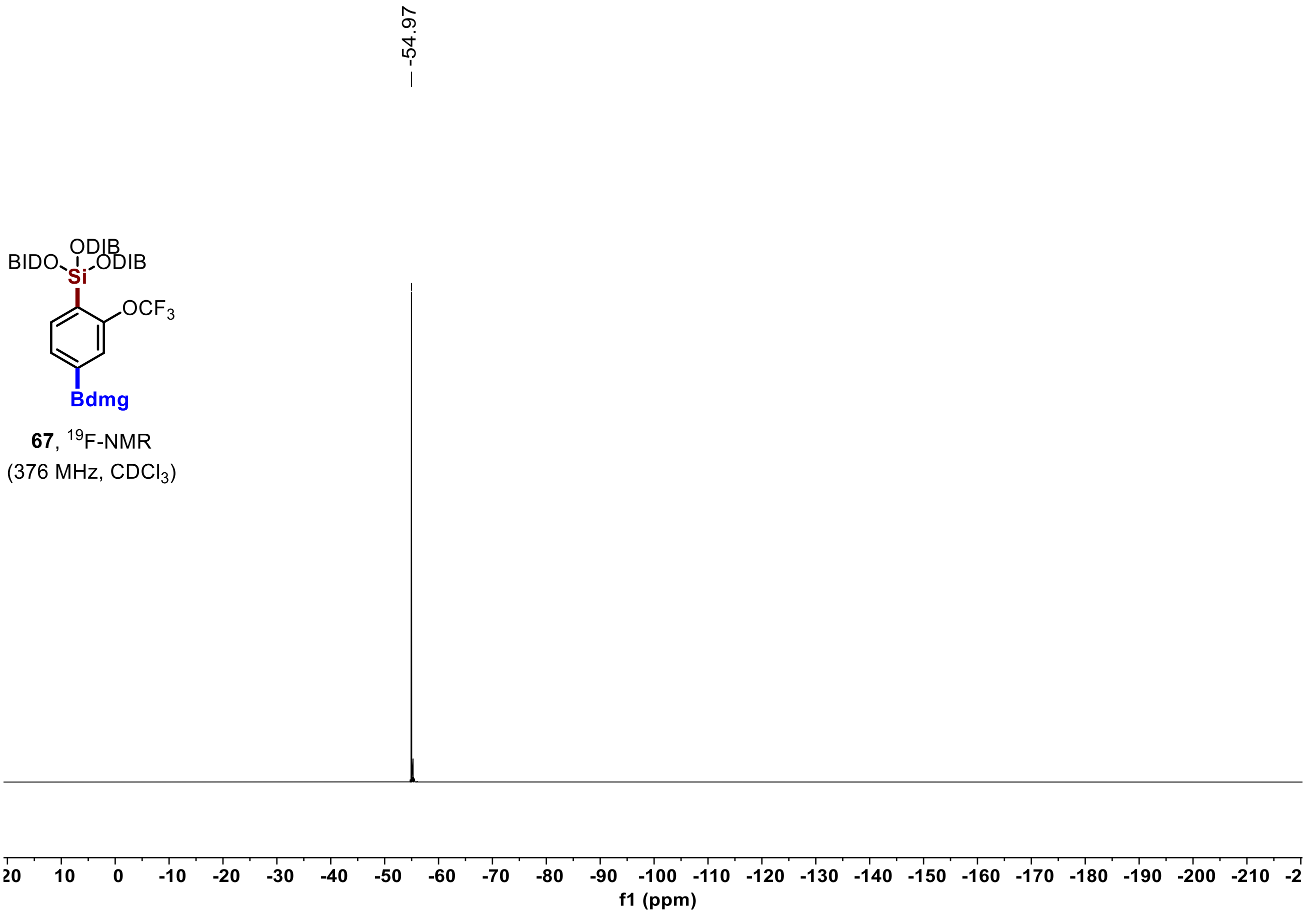
-54.97
BIDO
ODIB
ODIB
Si
OCF3
Bdmg
67, 19F-NMR
(376 MHz, CDCl3)
f1 (ppm)

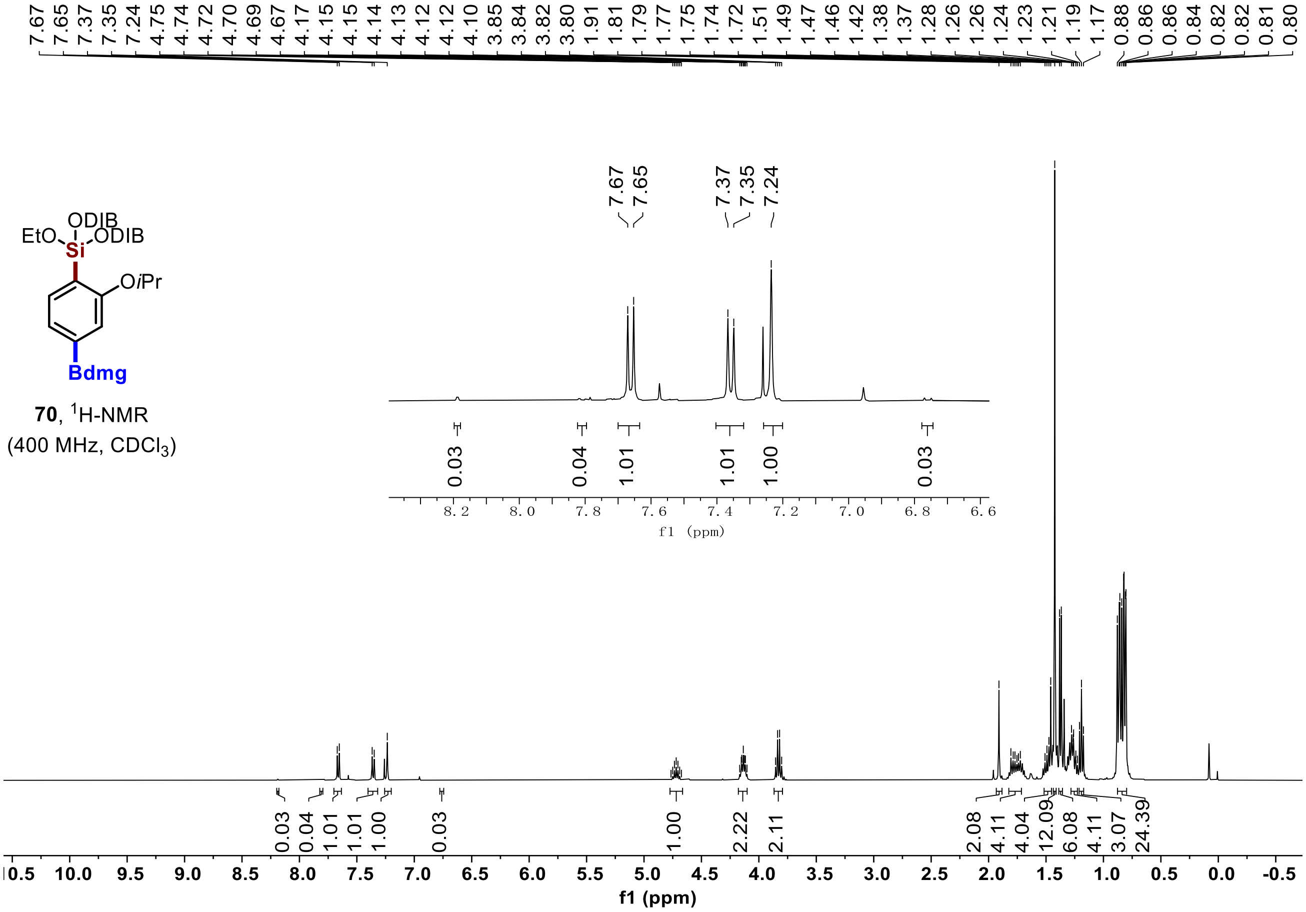
EtO
ODIB
ODIB
Si
OiPr
Bdmg
70, 1H-NMR
(400 MHz, CDCl3)
f1 (ppm)

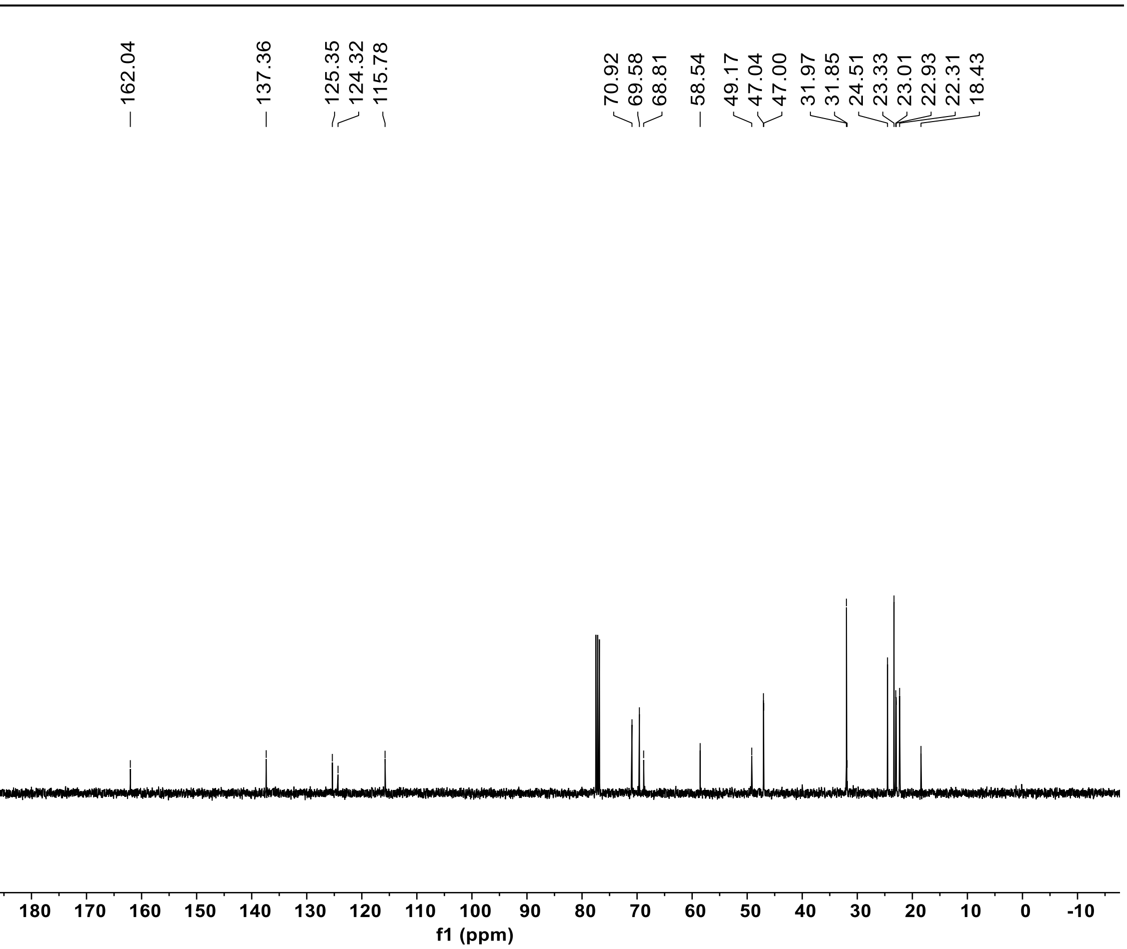

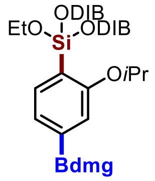

70, ^{13}C-NMR
(101 MHz, $CDCl_3$)

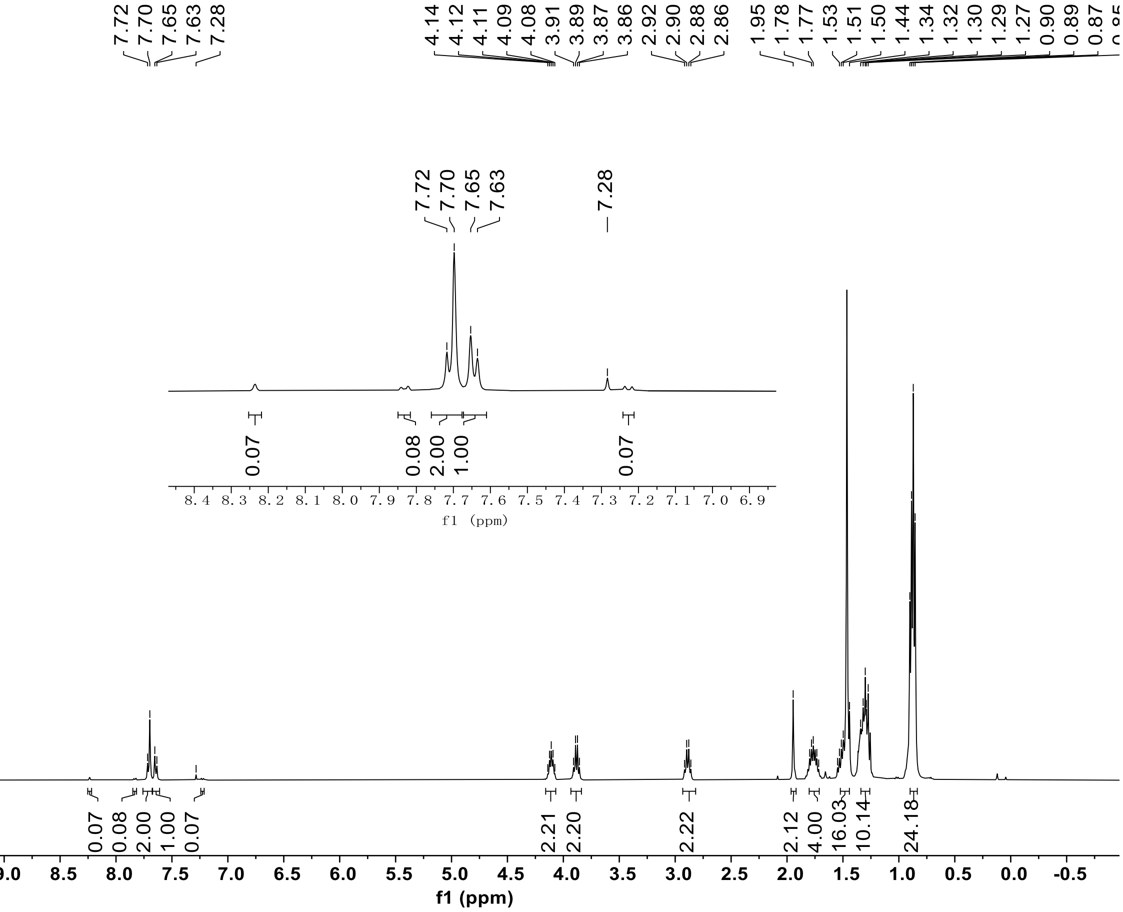

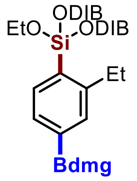

71, ^{1}H-NMR
(400 MHz, $CDCl_3$)

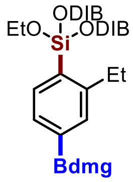

71, ^{13}C-NMR
(101 MHz, $CDCl_3$)

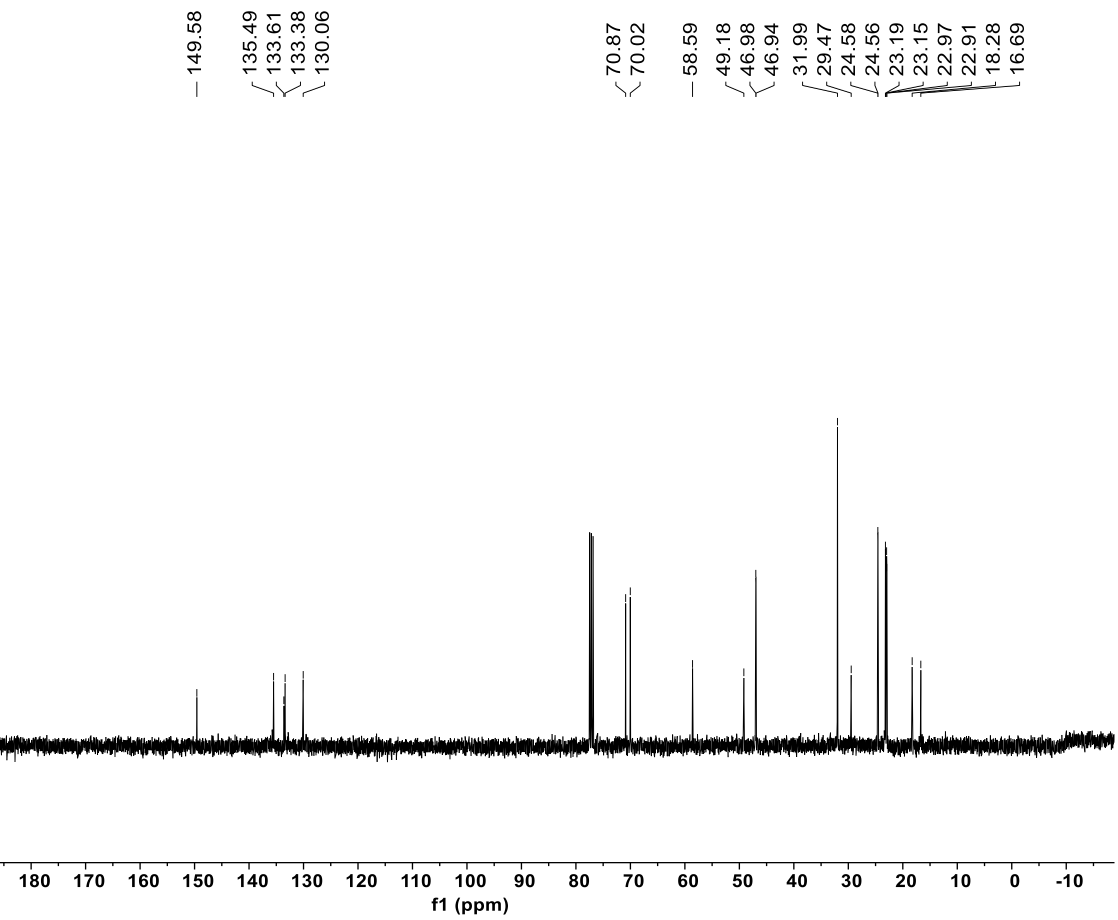

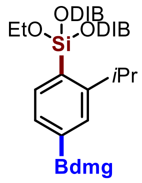

72, ^{1}H-NMR
(400 MHz, $CDCl_3$)

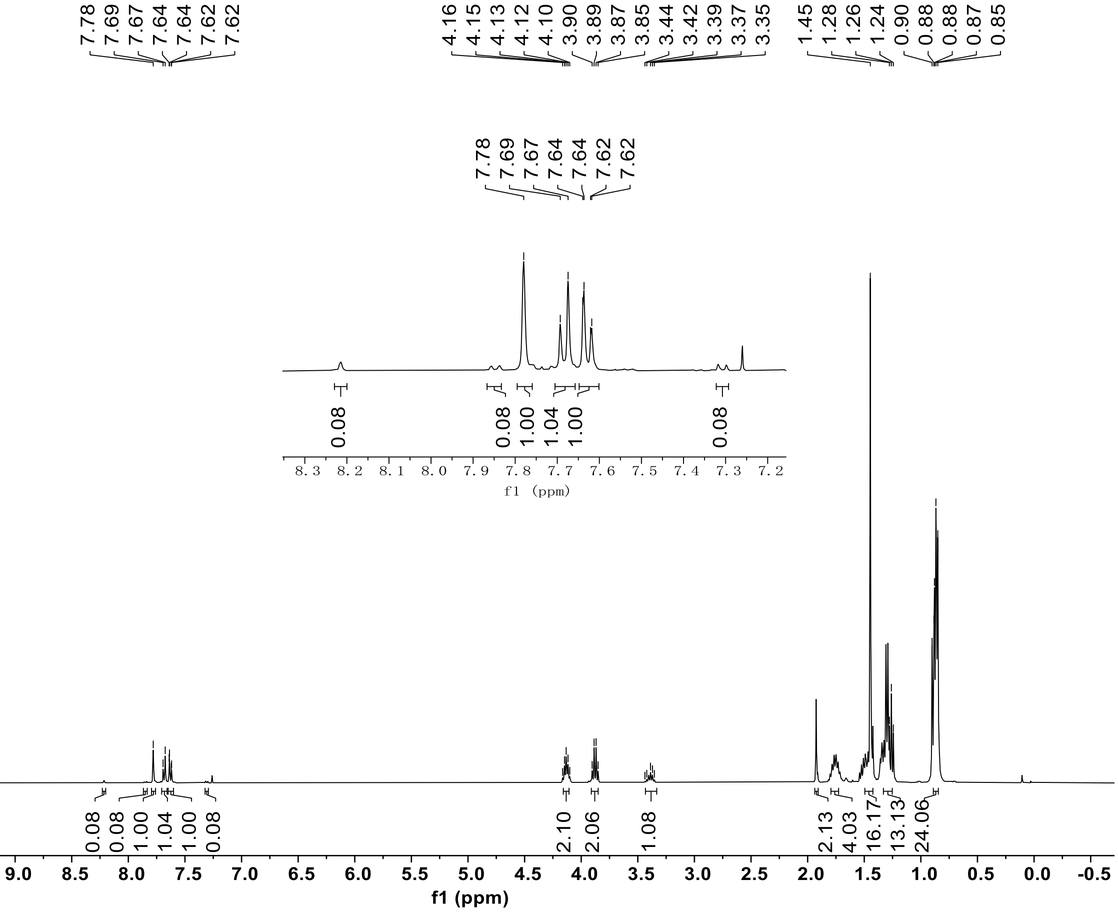

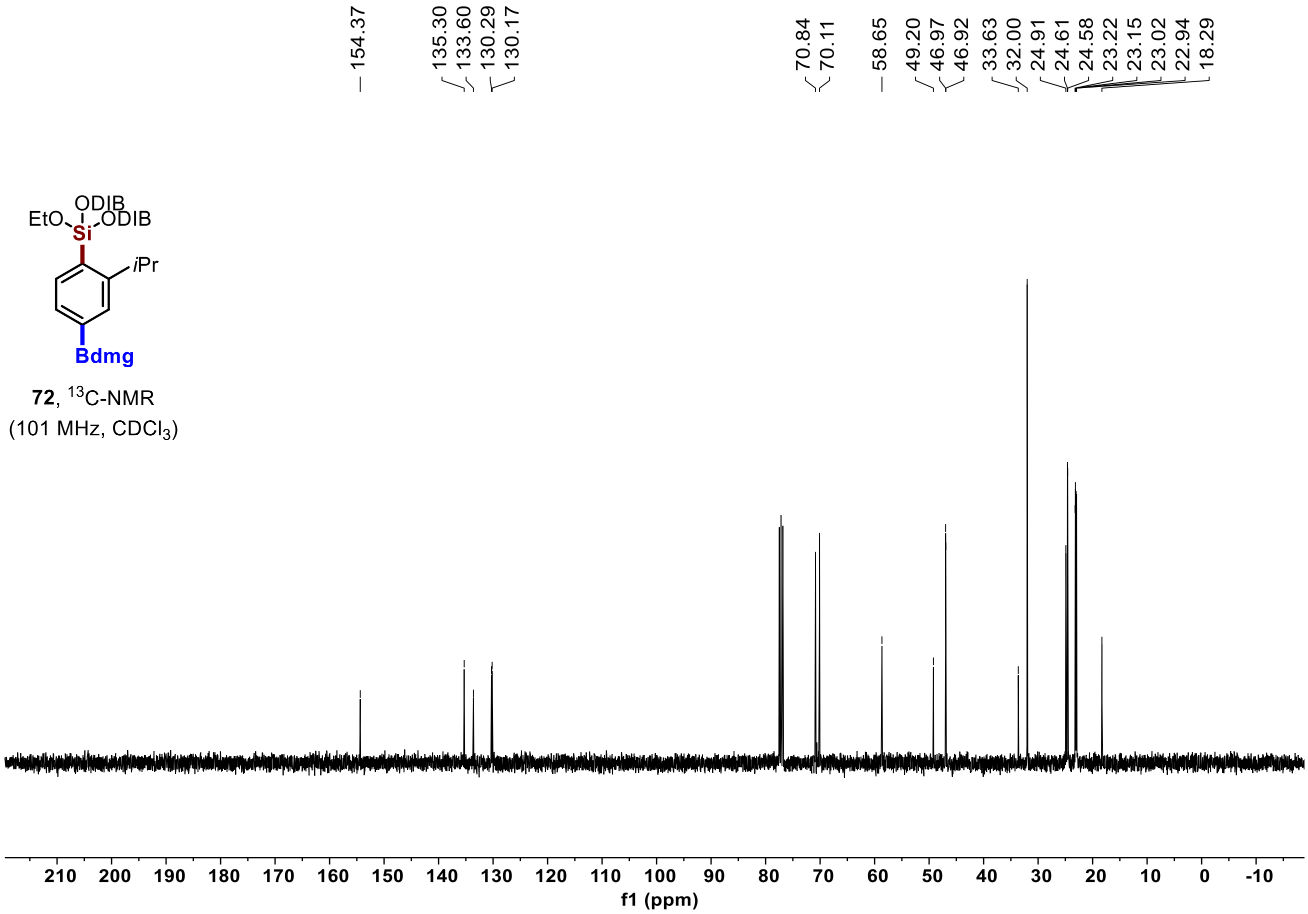

72, ^{13}C-NMR
(101 MHz, CDCl$_3$)

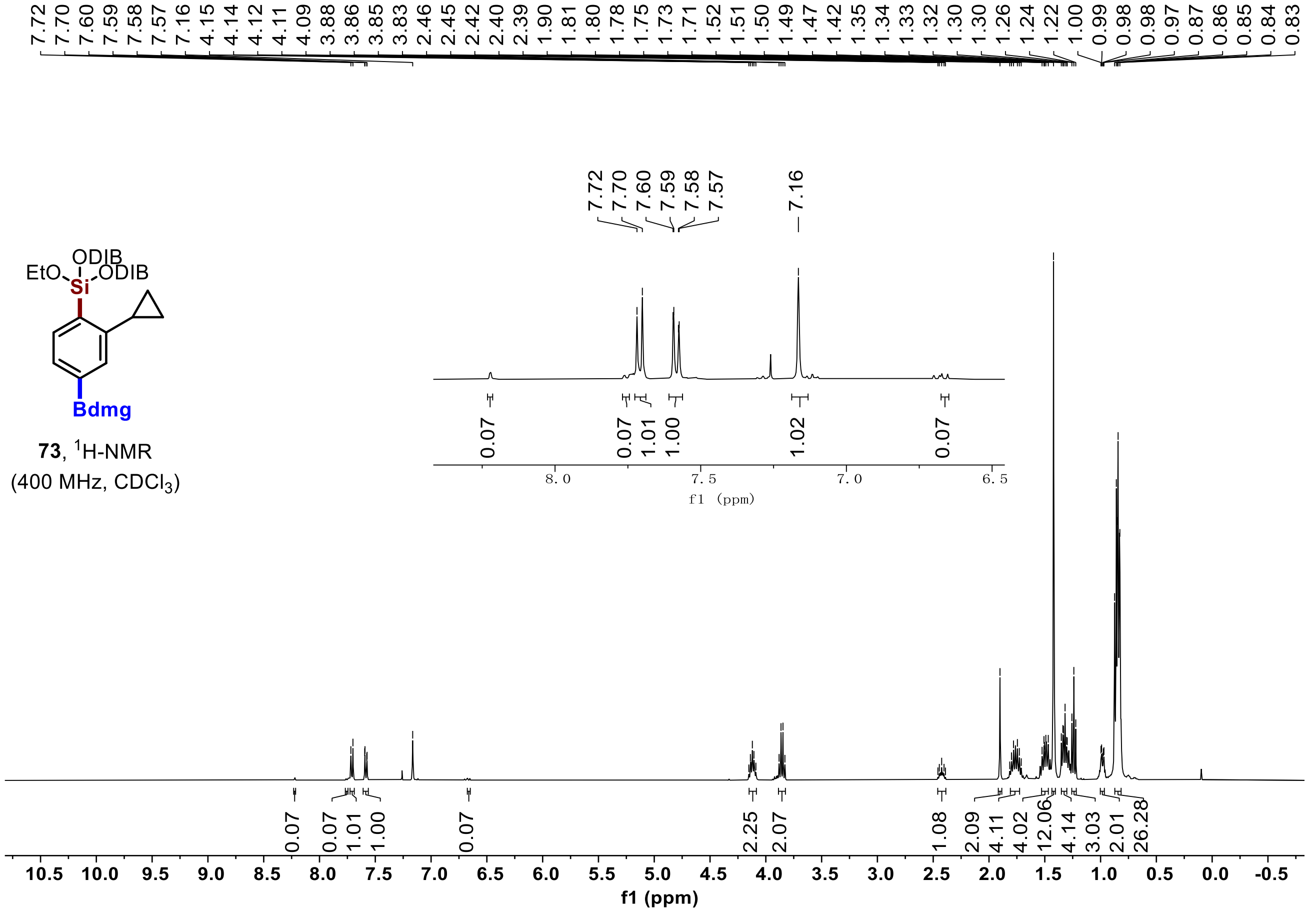

73, ^{1}H-NMR
(400 MHz, CDCl$_3$)

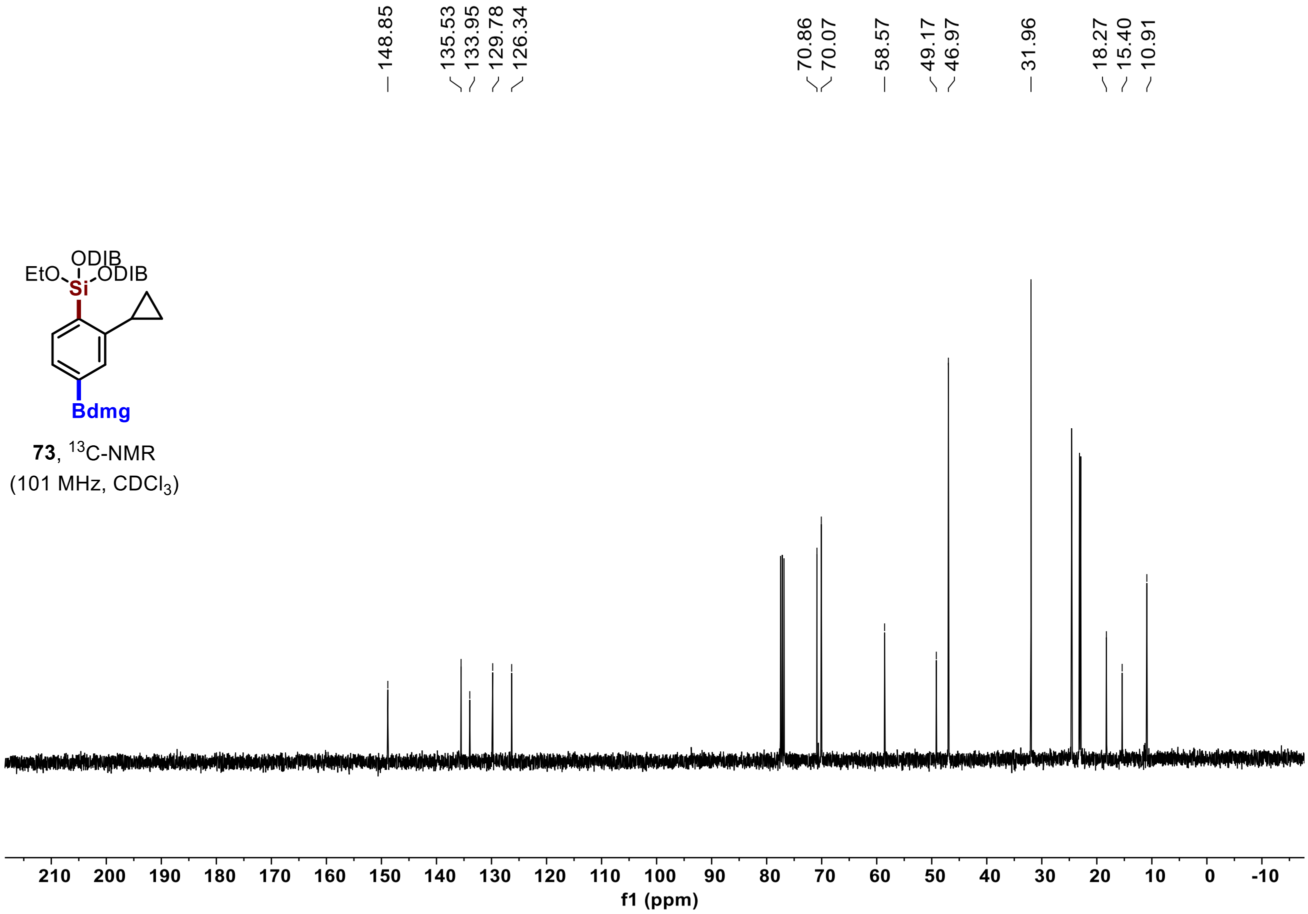

73, ^{13}C-NMR
(101 MHz, $CDCl_3$)

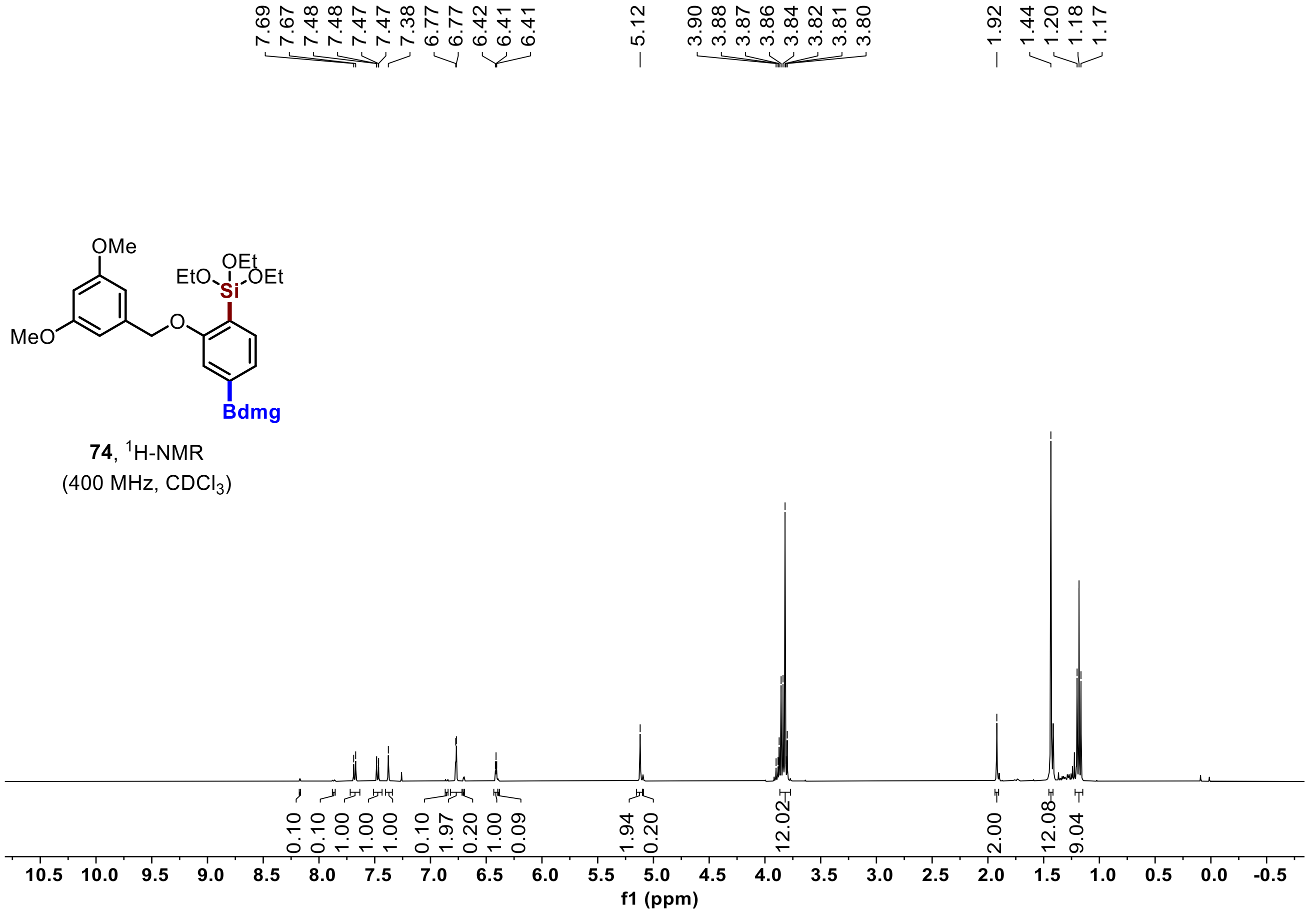

74, ^{1}H-NMR
(400 MHz, $CDCl_3$)

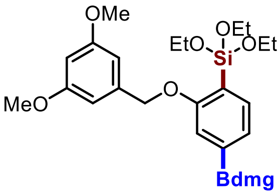

74, ^{13}C-NMR
(101 MHz, $CDCl_3$)

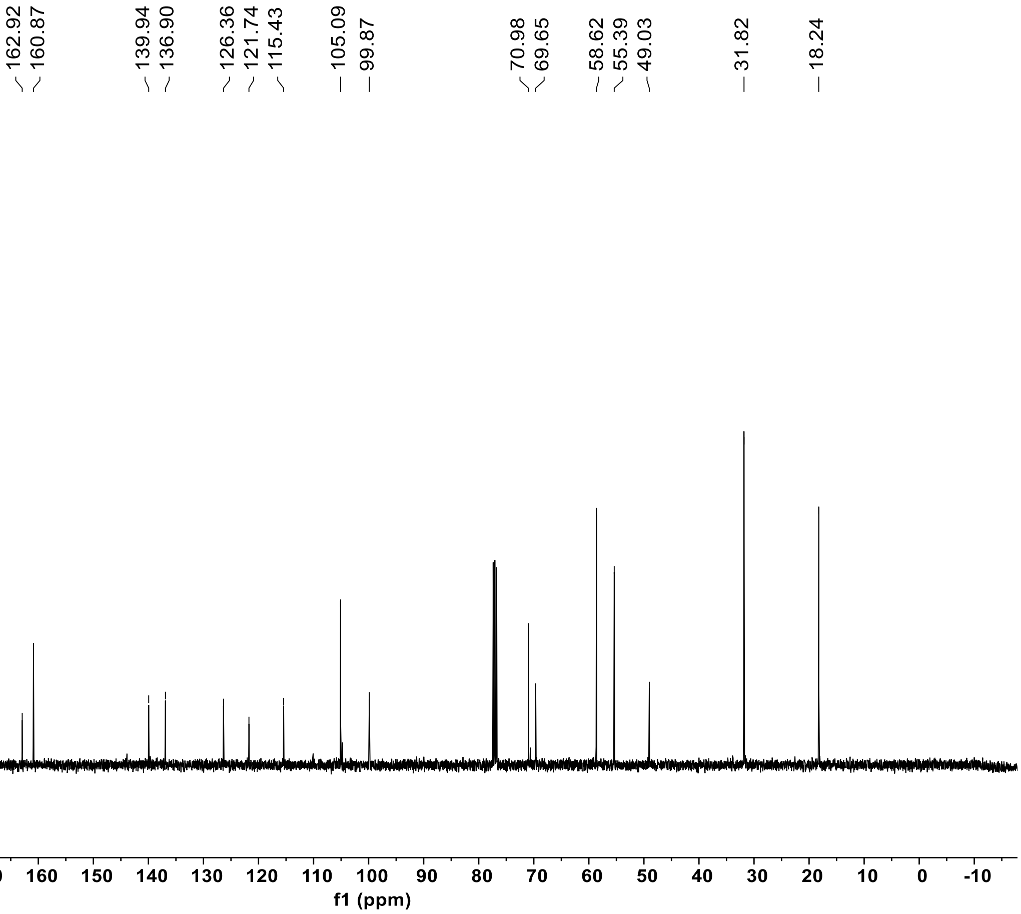

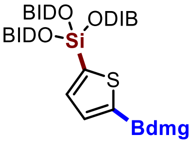

75, ^{1}H-NMR
(400 MHz, $CDCl_3$)

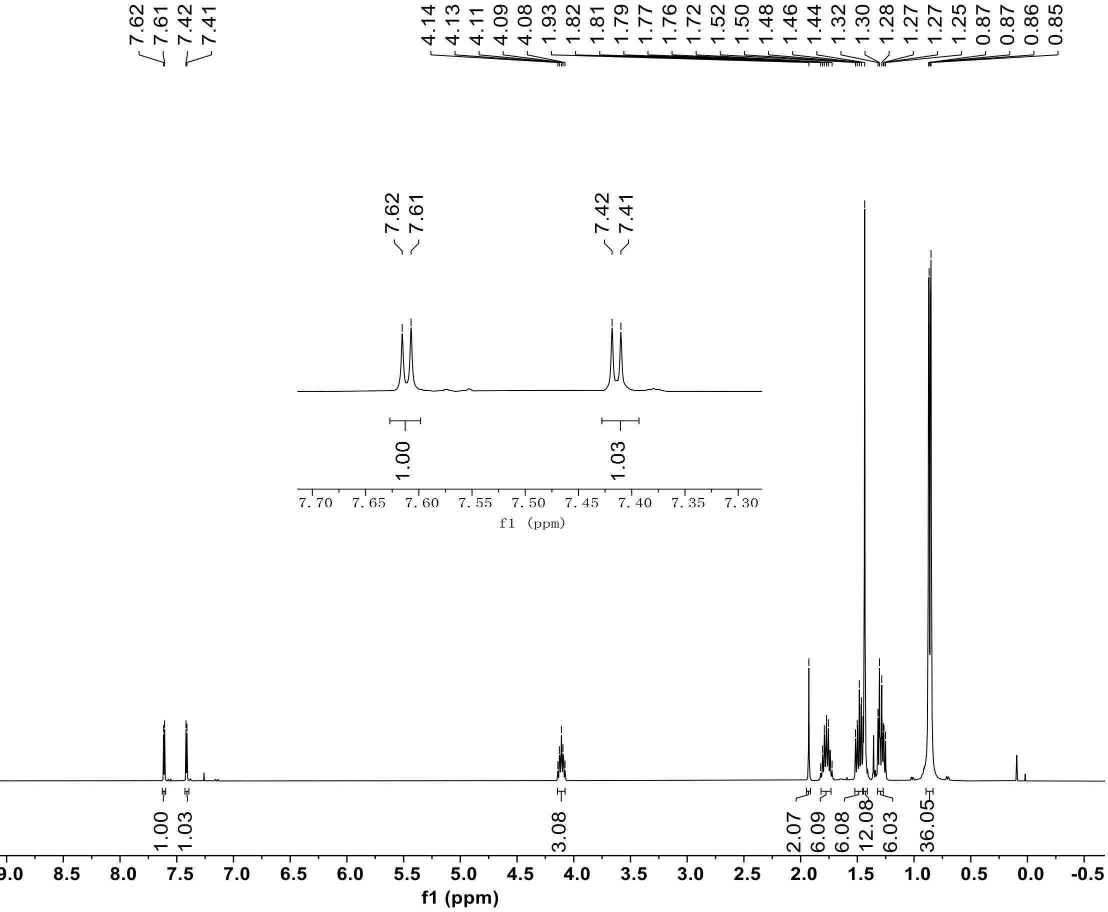

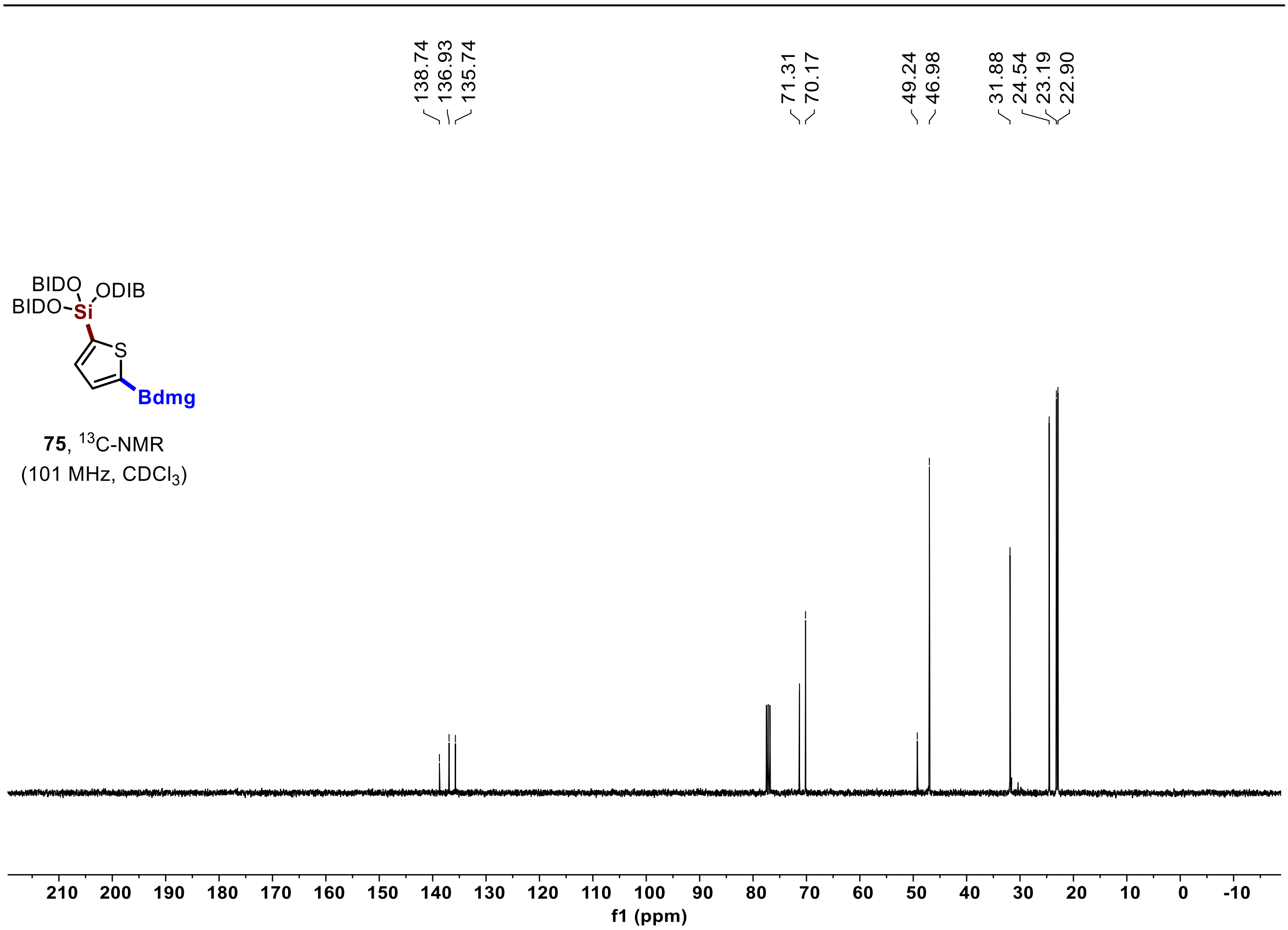
138.74
136.93
135.74
71.31
70.17
49.24
46.98
31.88
24.54
23.19
22.90
BIDO
ODIB
BIDO
Si
S
Bdmg
75, ^{13}C-NMR
(101 MHz, $CDCl_3$)
210 200 190 180 170 160 150 140 130 120 110 100 90 80 70 60 50 40 30 20 10 0 -10
f1 (ppm)

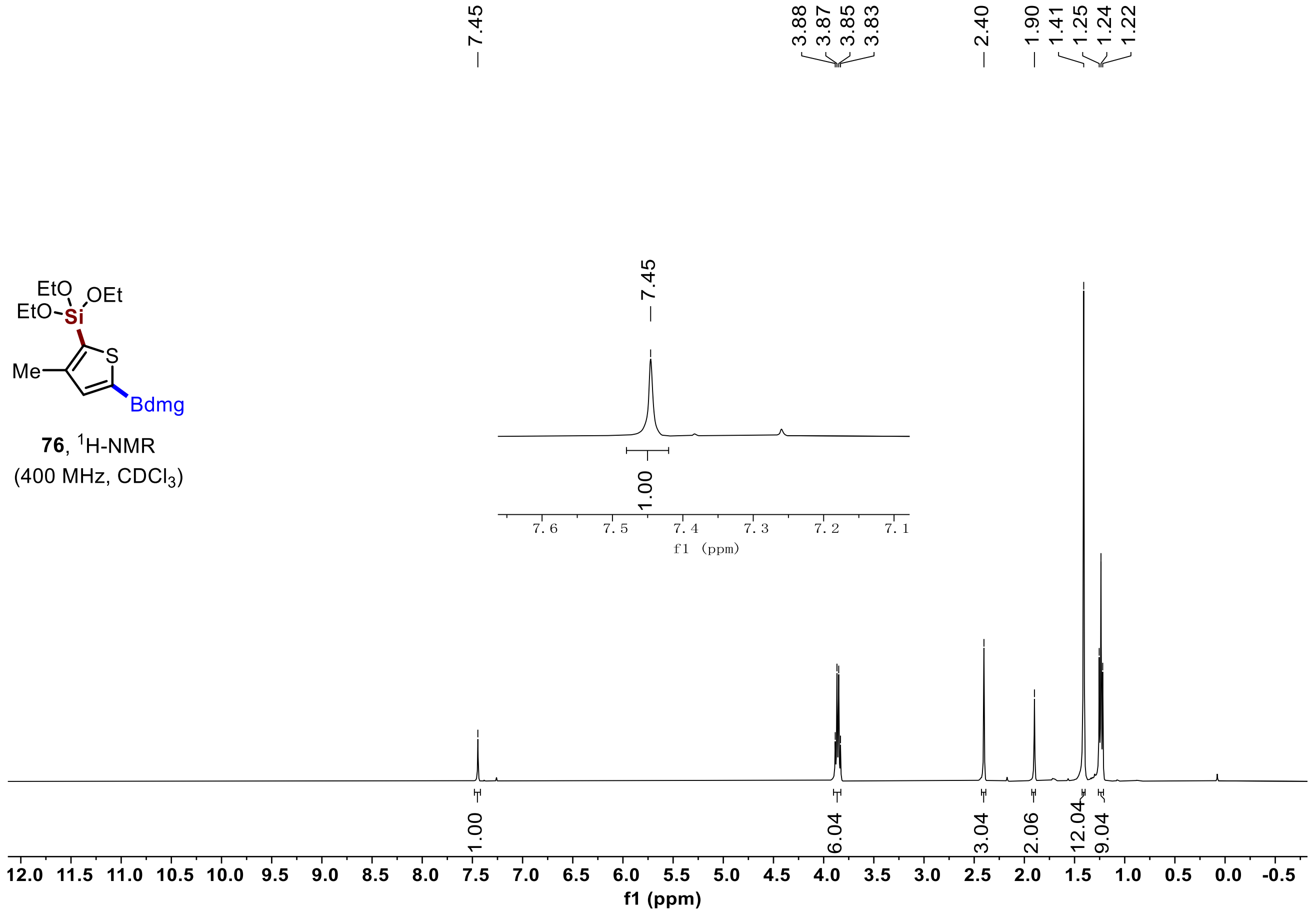
7.45
3.88
3.87
3.85
3.83
2.40
1.90
1.41
1.25
1.24
1.22
EtO
OEt
EtO
Si
S
Me
Bdmg
76, ^{1}H-NMR
(400 MHz, $CDCl_3$)
7.45
1.00
7.6 7.5 7.4 7.3 7.2 7.1
f1 (ppm)
1.00
6.04
3.04
2.06
12.04
9.04
12.0 11.5 11.0 10.5 10.0 9.5 9.0 8.5 8.0 7.5 7.0 6.5 6.0 5.5 5.0 4.5 4.0 3.5 3.0 2.5 2.0 1.5 1.0 0.5 0.0 -0.5
f1 (ppm)

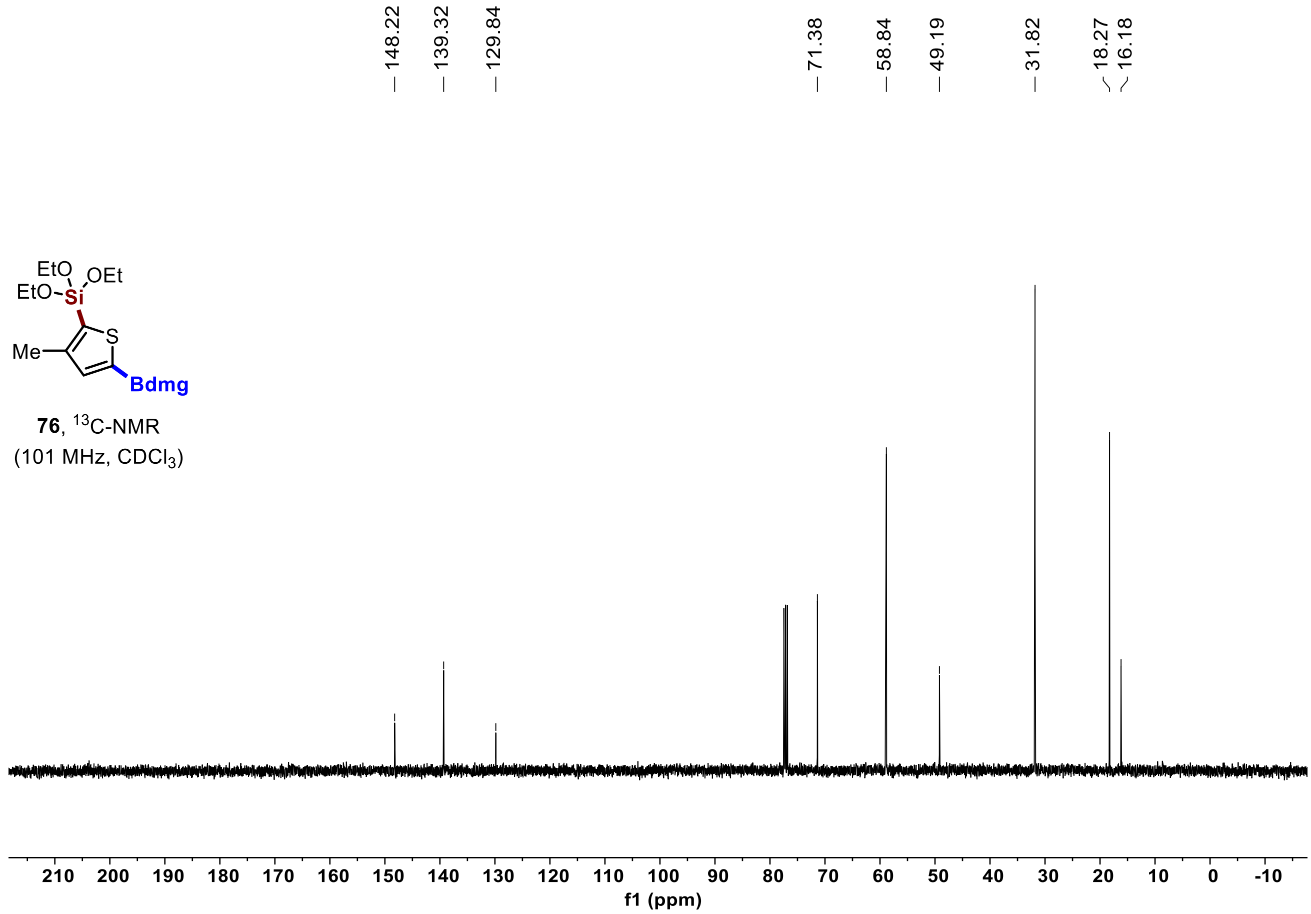

76, ^{13}C-NMR
(101 MHz, $CDCl_3$)

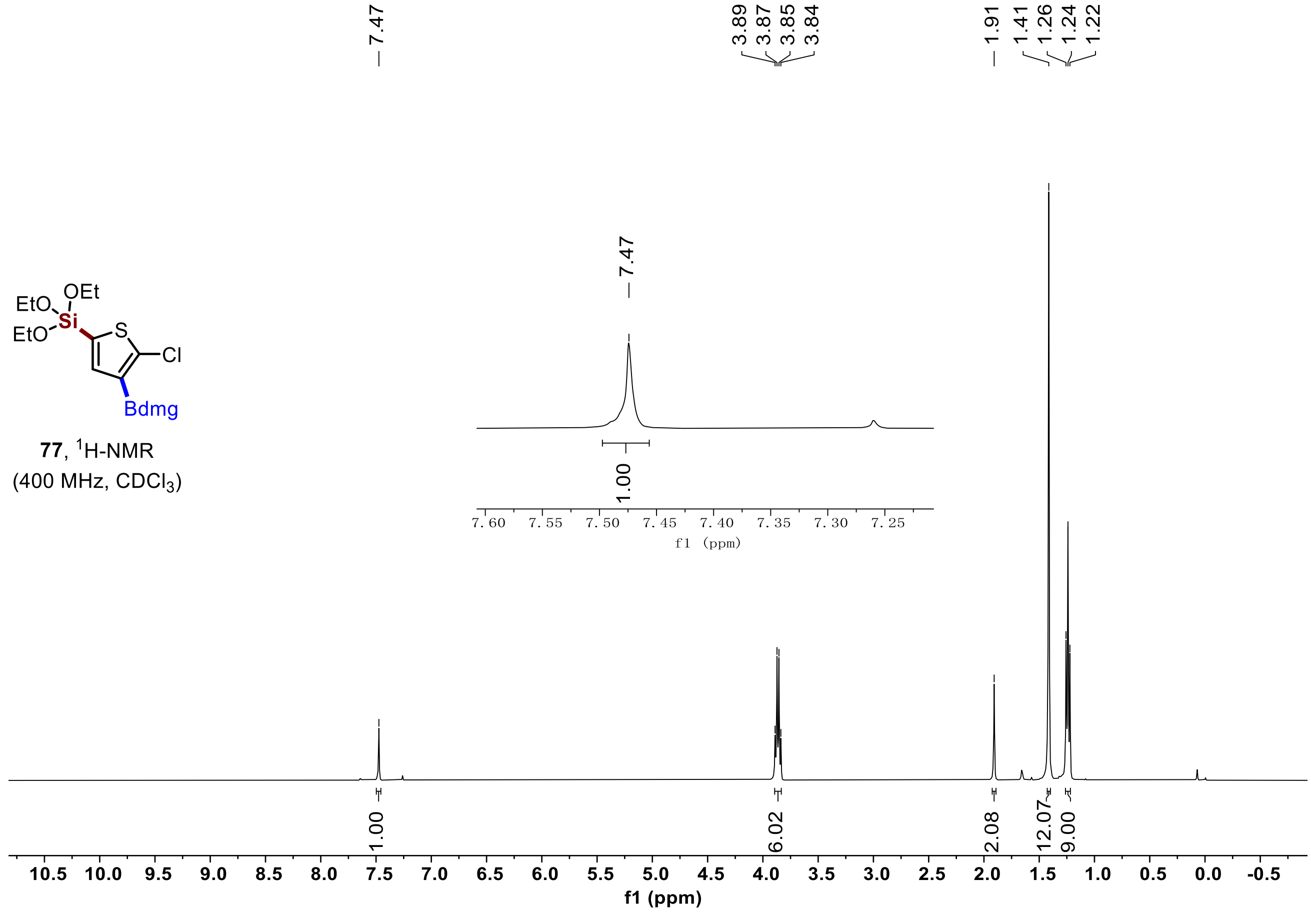

77, ^{1}H-NMR
(400 MHz, $CDCl_3$)

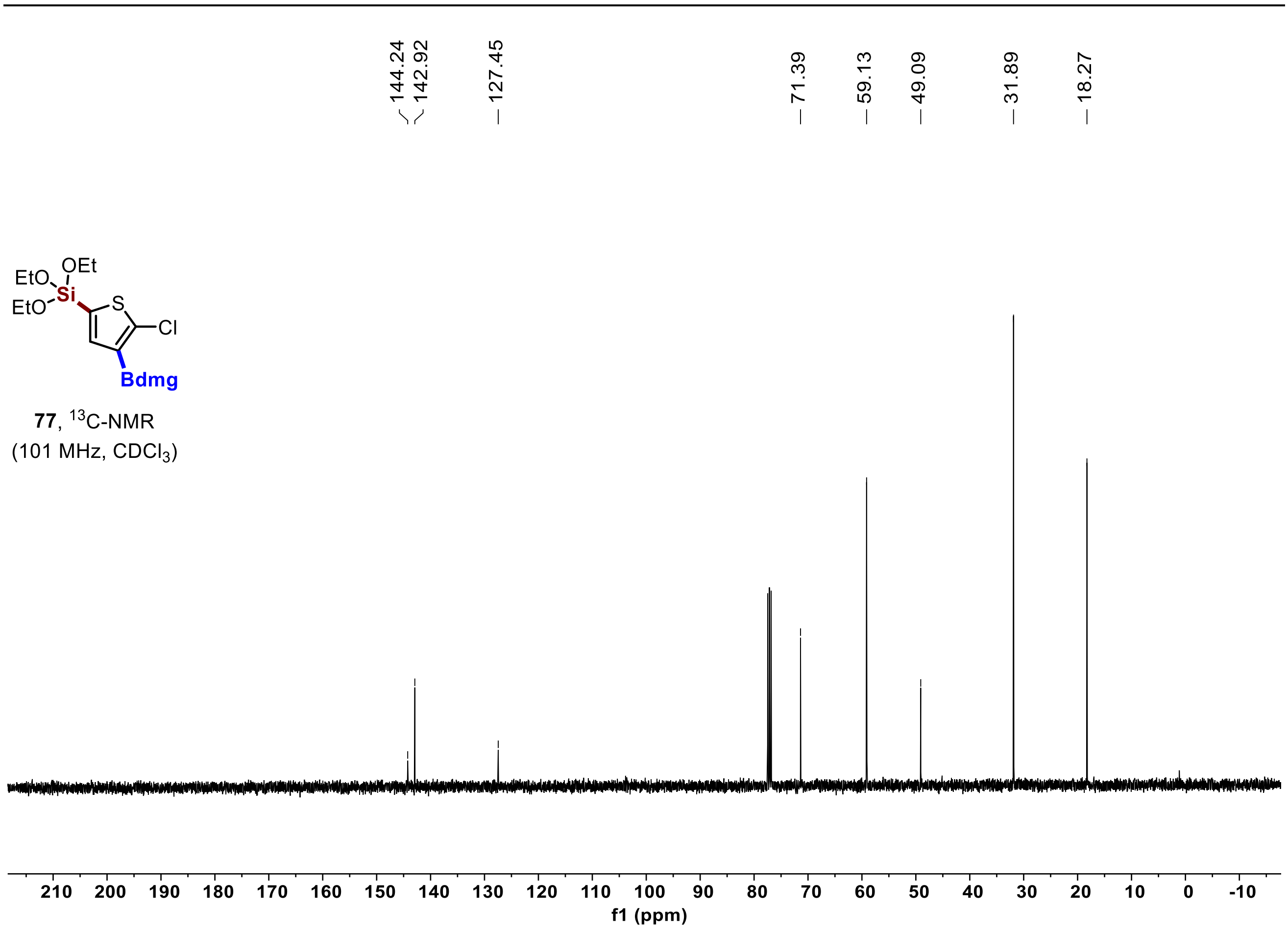

EtO
OEt
EtO
Si
S
Cl
Bdmg
77, ¹³C-NMR
(101 MHz, CDCl3)
144.24
142.92
127.45
71.39
59.13
49.09
31.89
18.27
f1 (ppm)

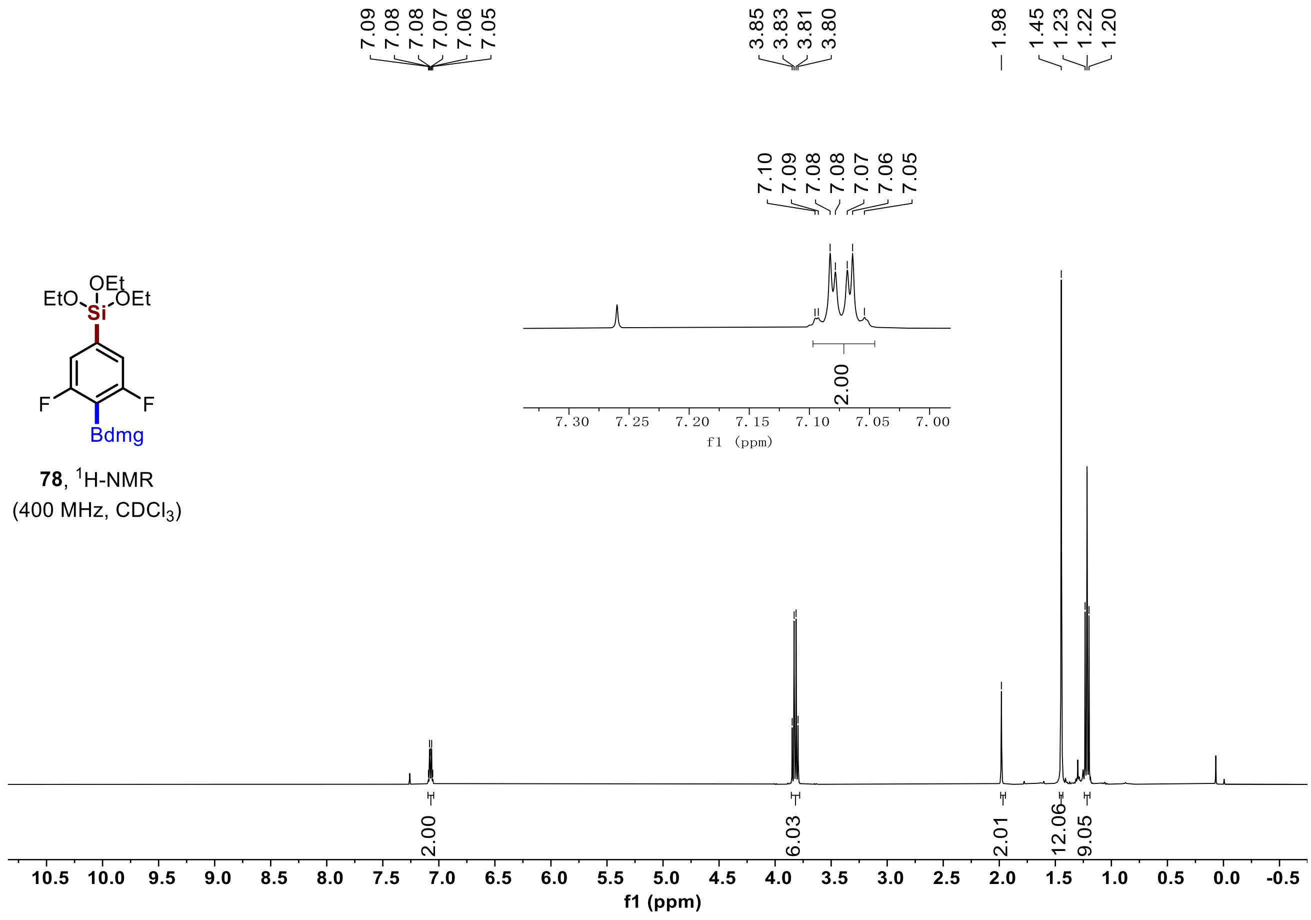

EtO
OEt
OEt
Si
F
F
Bdmg
78, ¹H-NMR
(400 MHz, CDCl3)
7.09
7.08
7.08
7.07
7.06
7.05
3.85
3.83
3.81
3.80
1.98
1.45
1.23
1.22
1.20
7.10
7.09
7.08
7.08
7.07
7.06
7.05
2.00
f1 (ppm)
2.00
6.03
2.01
12.06
9.05
f1 (ppm)

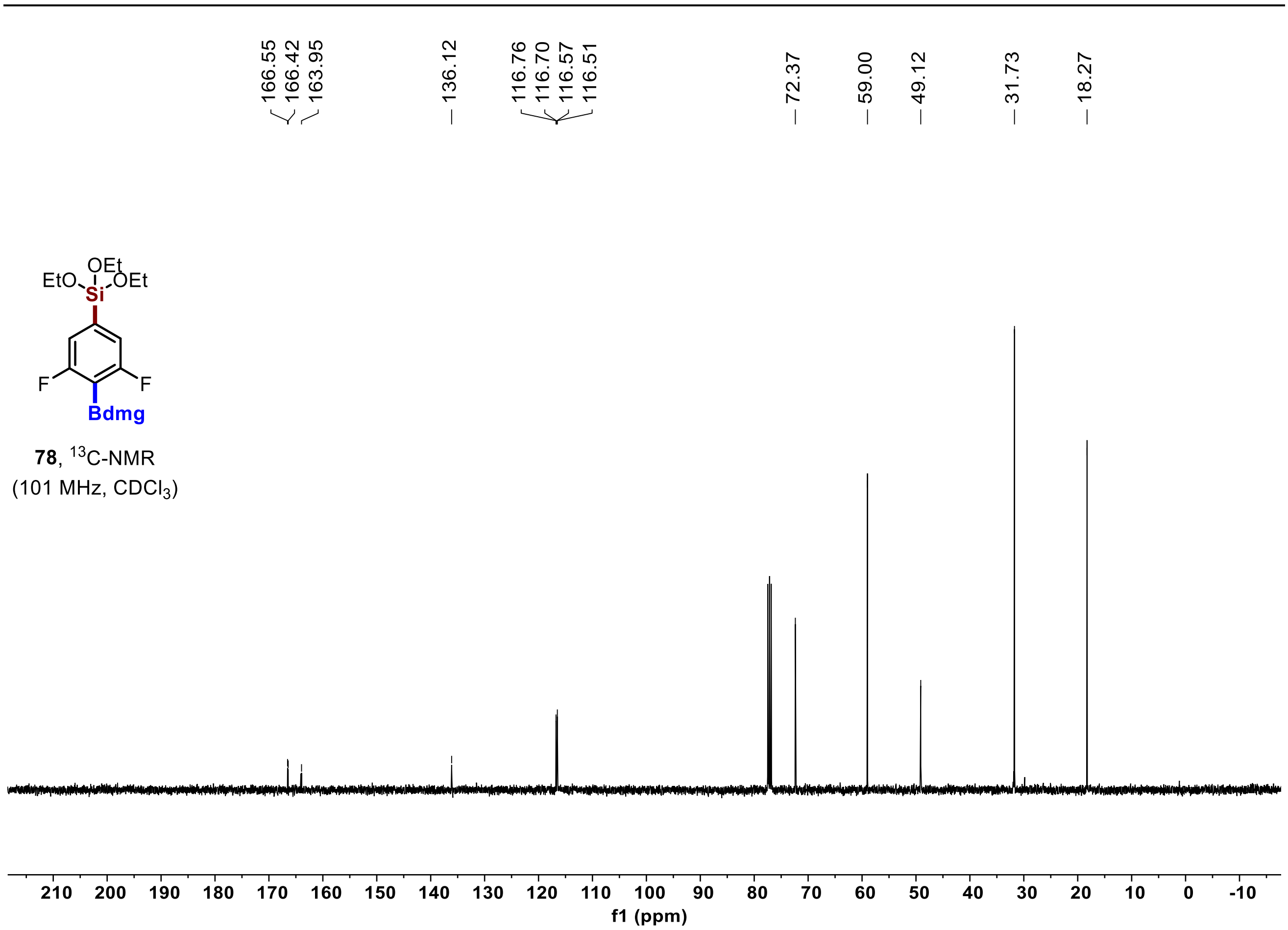

78, ^{13}C-NMR
(101 MHz, $CDCl_3$)

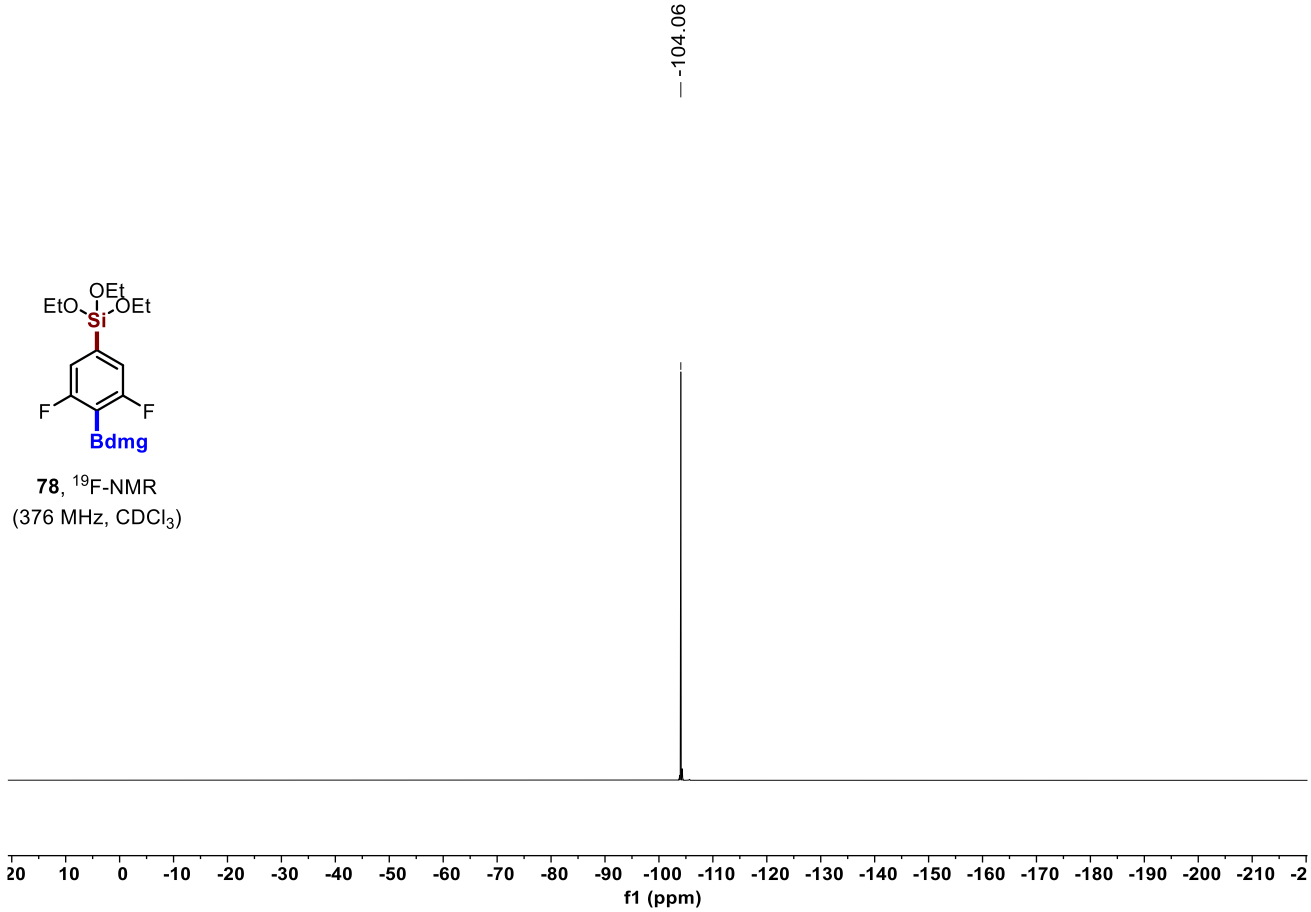

78, ^{19}F-NMR
(376 MHz, $CDCl_3$)

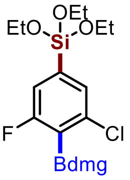

79, ^{1}H-NMR
(400 MHz, $CDCl_3$)

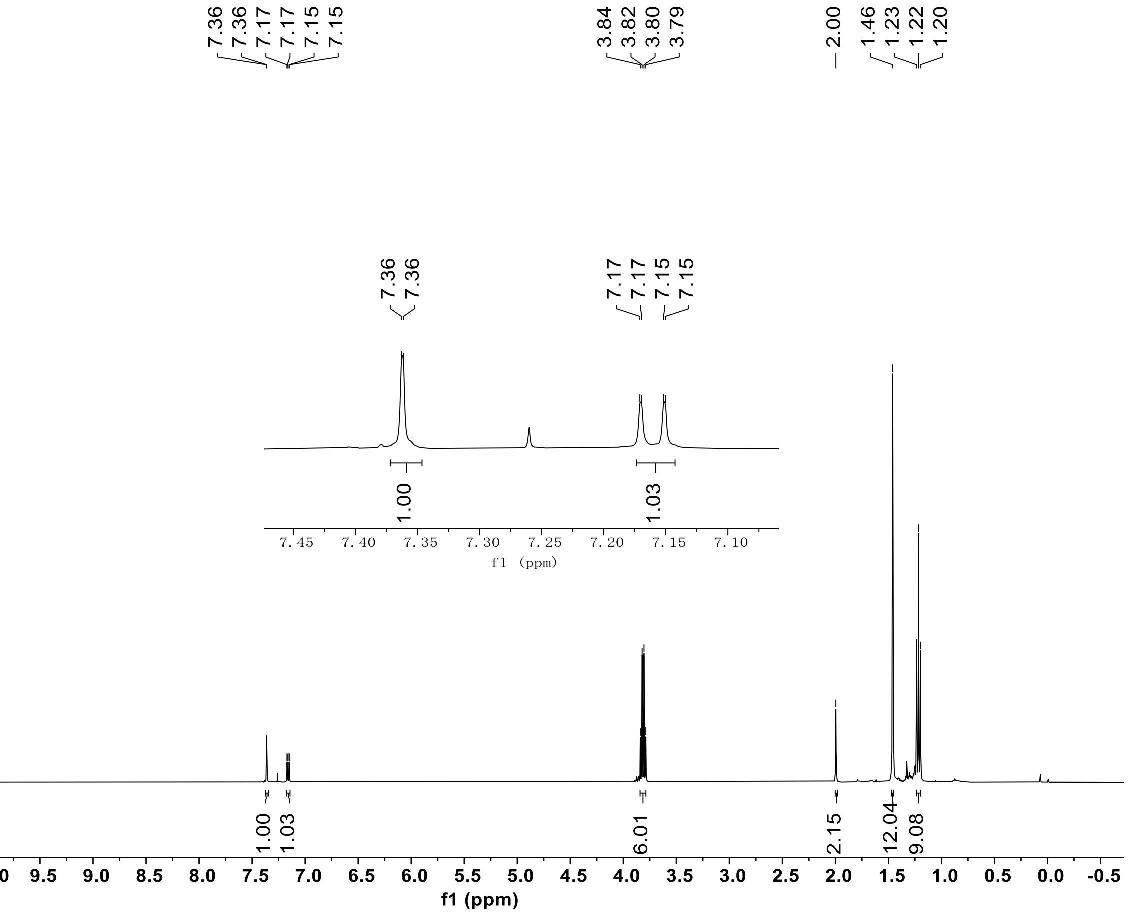

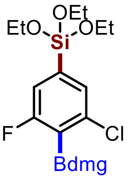

79, ^{13}C-NMR
(101 MHz, $CDCl_3$)

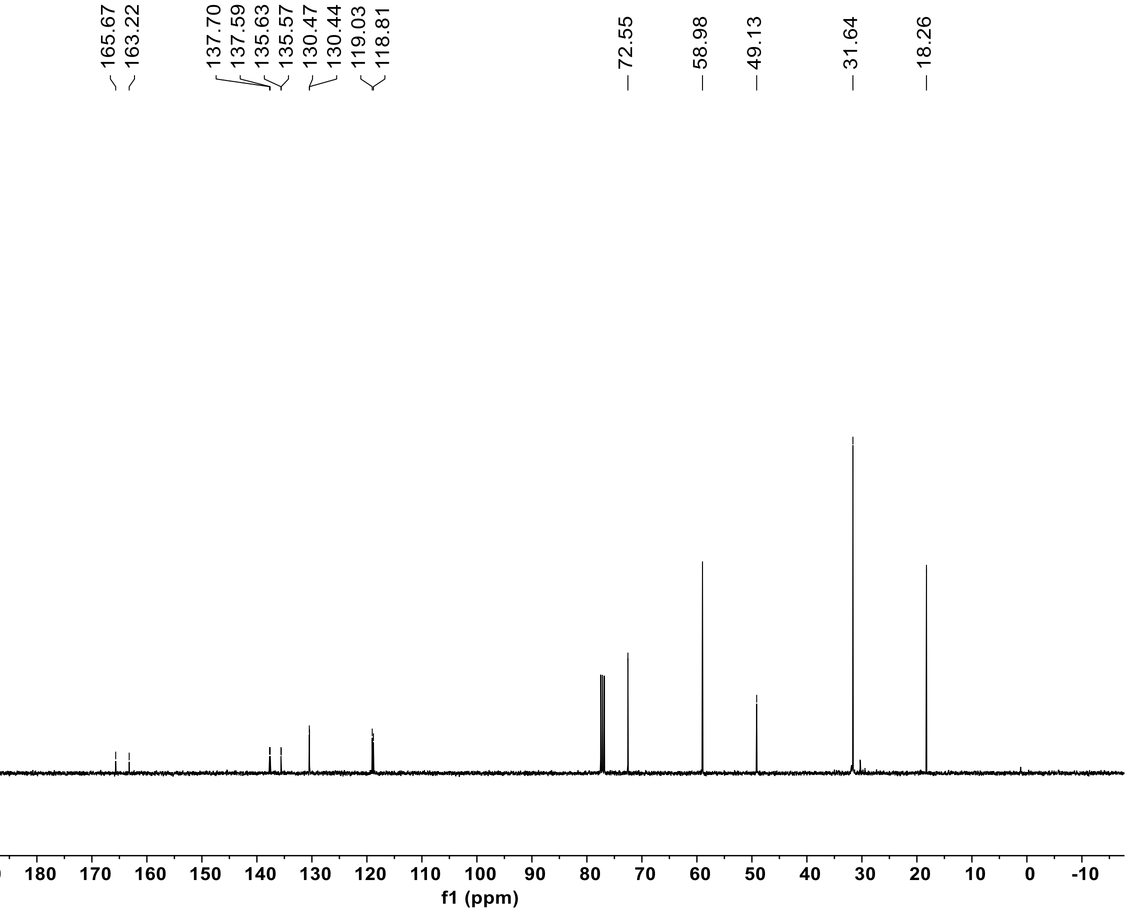

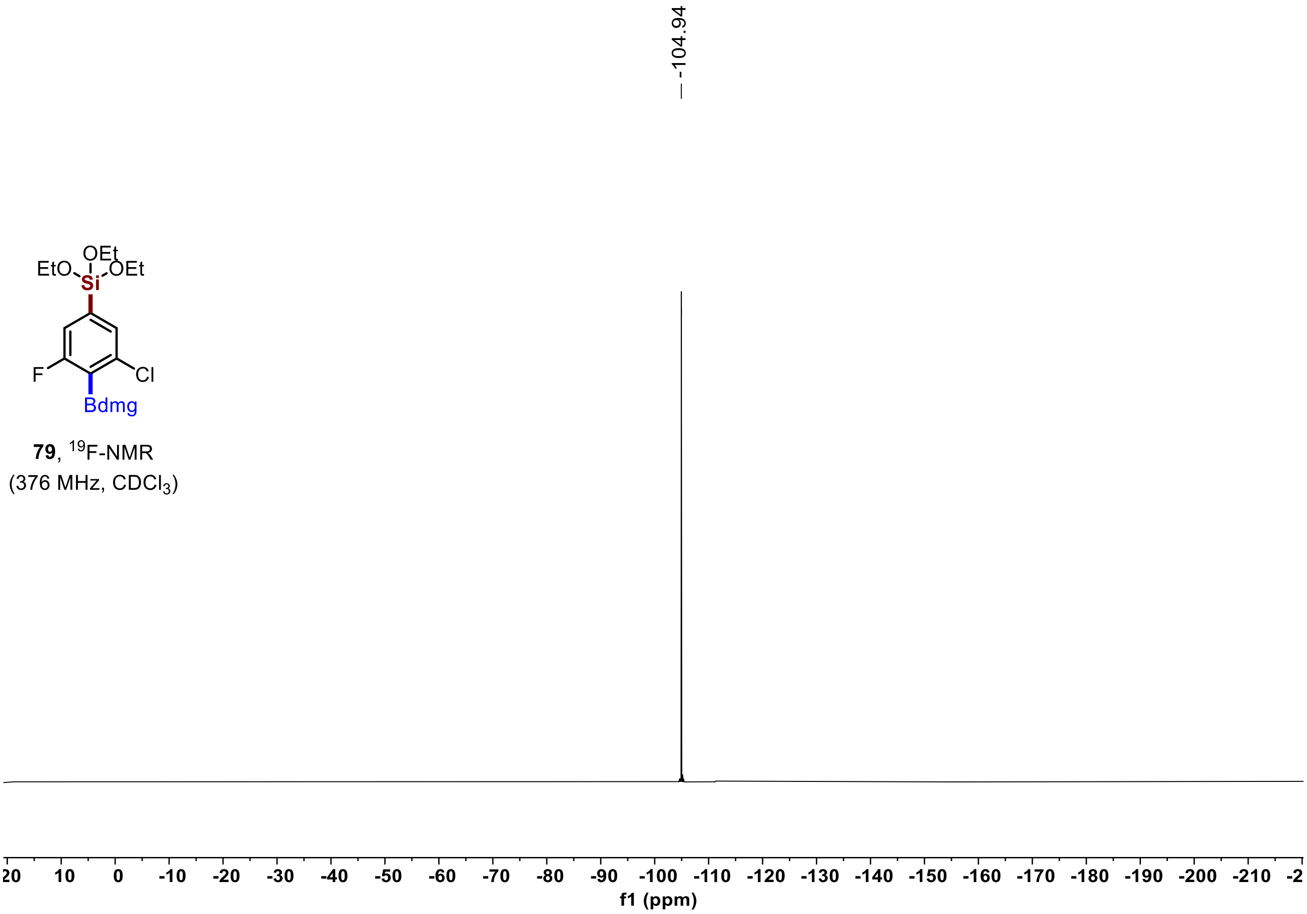

79, ^{19}F-NMR
(376 MHz, $CDCl_3$)

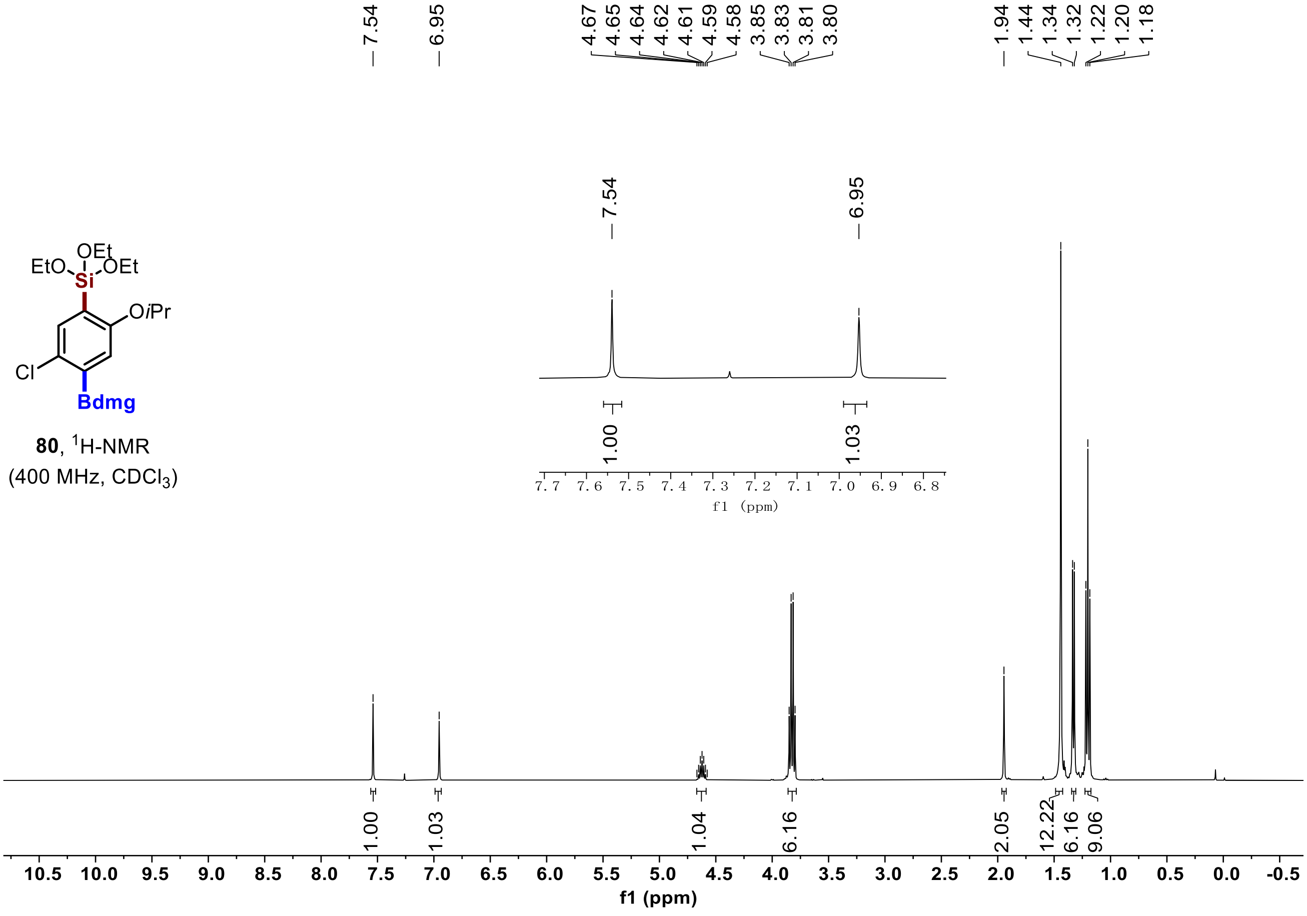

80, ^{1}H-NMR
(400 MHz, $CDCl_3$)

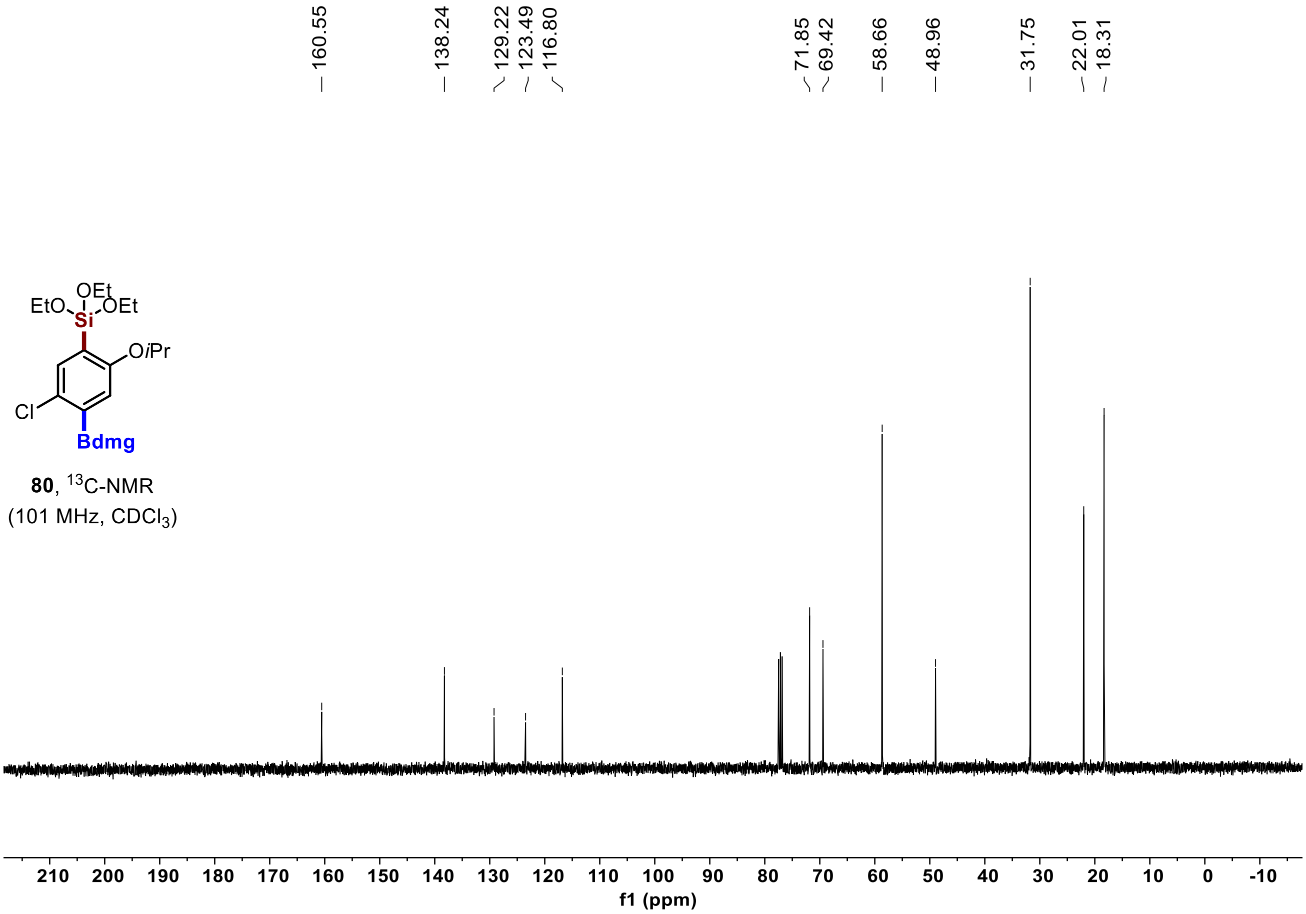
160.55
138.24
129.22
123.49
116.80
71.85
69.42
58.66
48.96
31.75
22.01
18.31
EtO
OEt
OEt
Si
OiPr
Cl
Bdmg
80, 13C-NMR
(101 MHz, CDCl3)
210 200 190 180 170 160 150 140 130 120 110 100 90 80 70 60 50 40 30 20 10 0 -10
f1 (ppm)

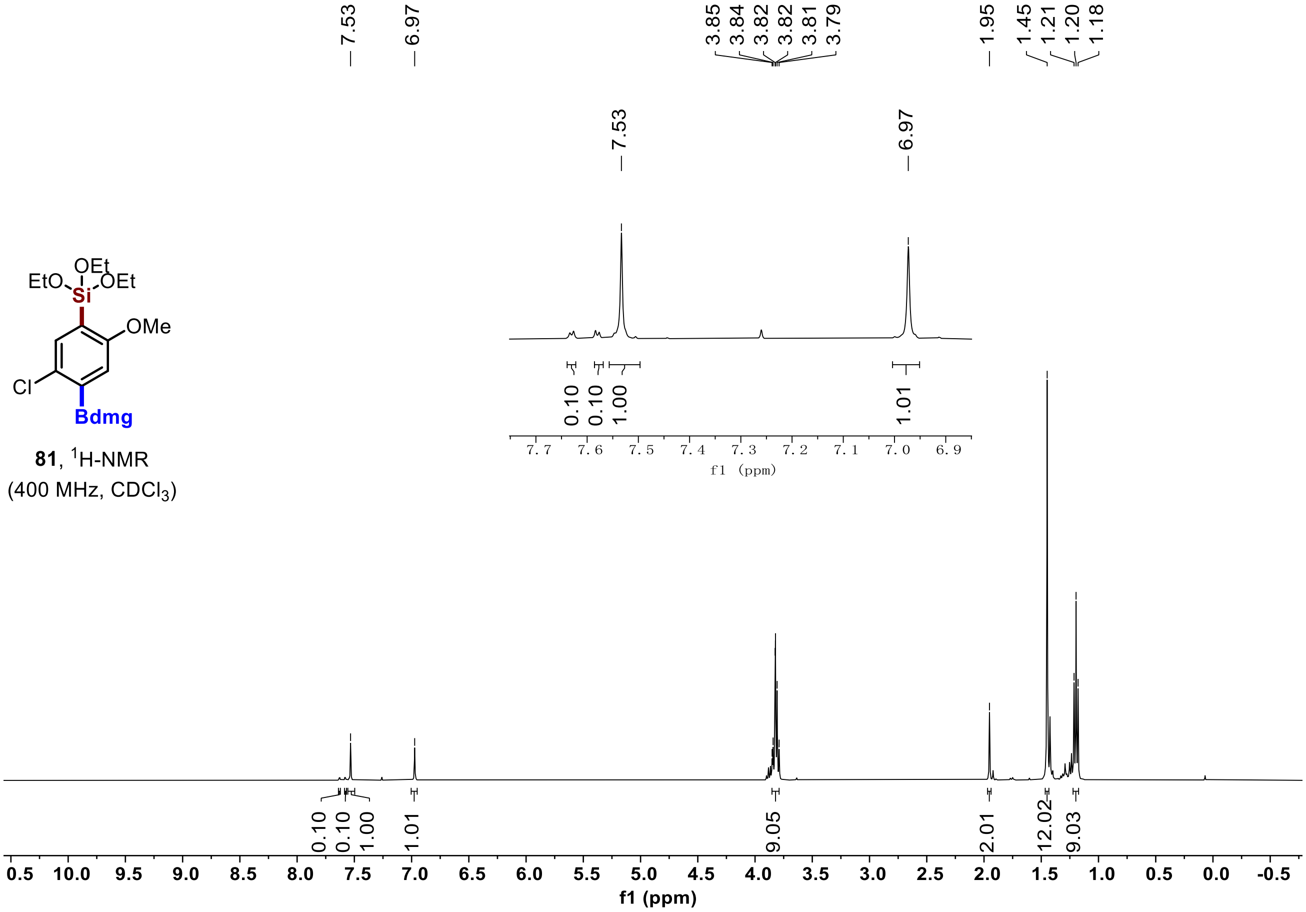
7.53
6.97
3.85
3.84
3.82
3.82
3.81
3.79
1.95
1.45
1.21
1.20
1.18
EtO
OEt
OEt
Si
OMe
Cl
Bdmg
81, 1H-NMR
(400 MHz, CDCl3)
0.10
0.10
1.00
1.01
7.7 7.6 7.5 7.4 7.3 7.2 7.1 7.0 6.9
f1 (ppm)
9.05
2.01
12.02
9.03
0.5 10.0 9.5 9.0 8.5 8.0 7.5 7.0 6.5 6.0 5.5 5.0 4.5 4.0 3.5 3.0 2.5 2.0 1.5 1.0 0.5 0.0 -0.5
f1 (ppm)

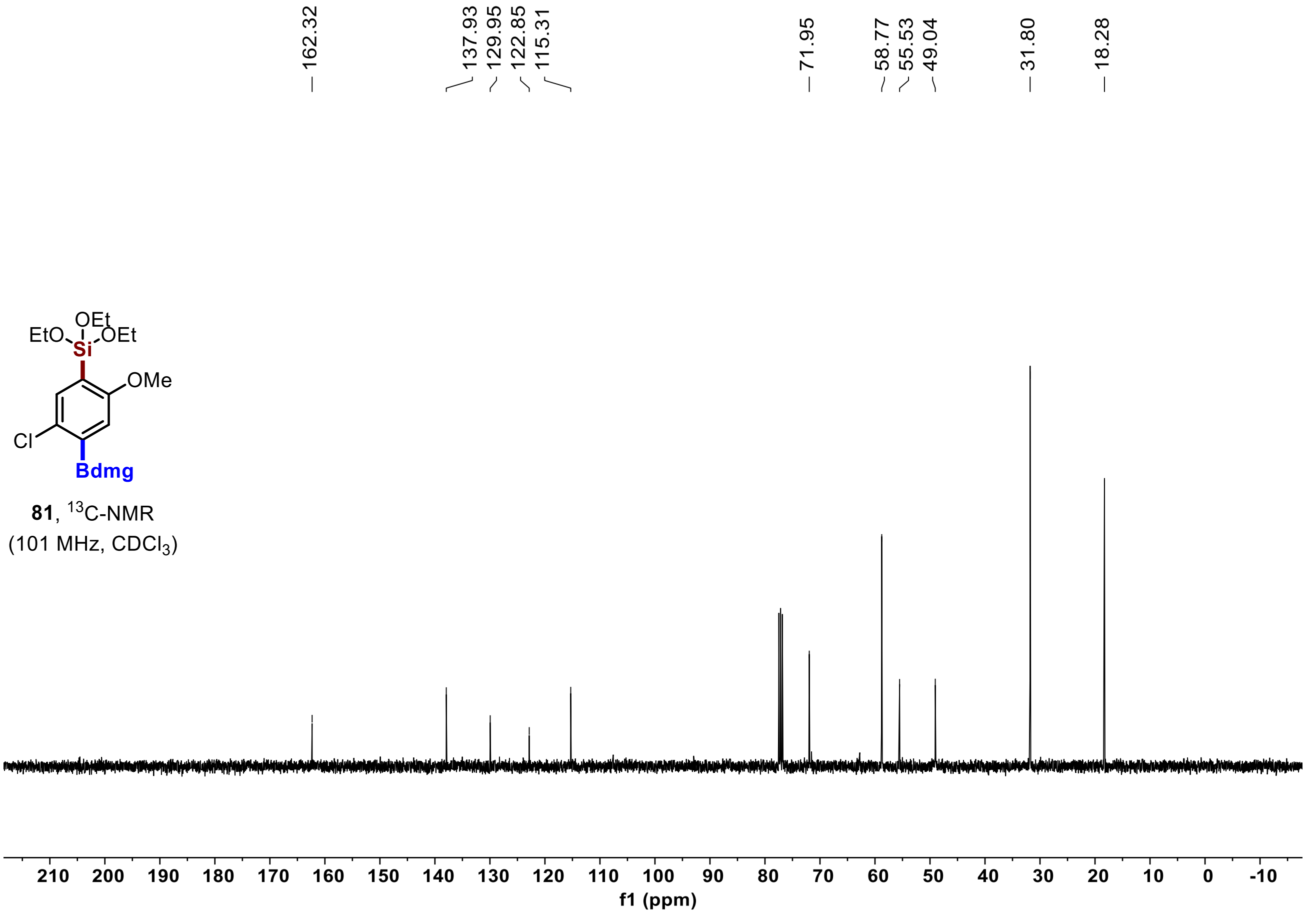

81, ^{13}C-NMR
(101 MHz, $CDCl_3$)

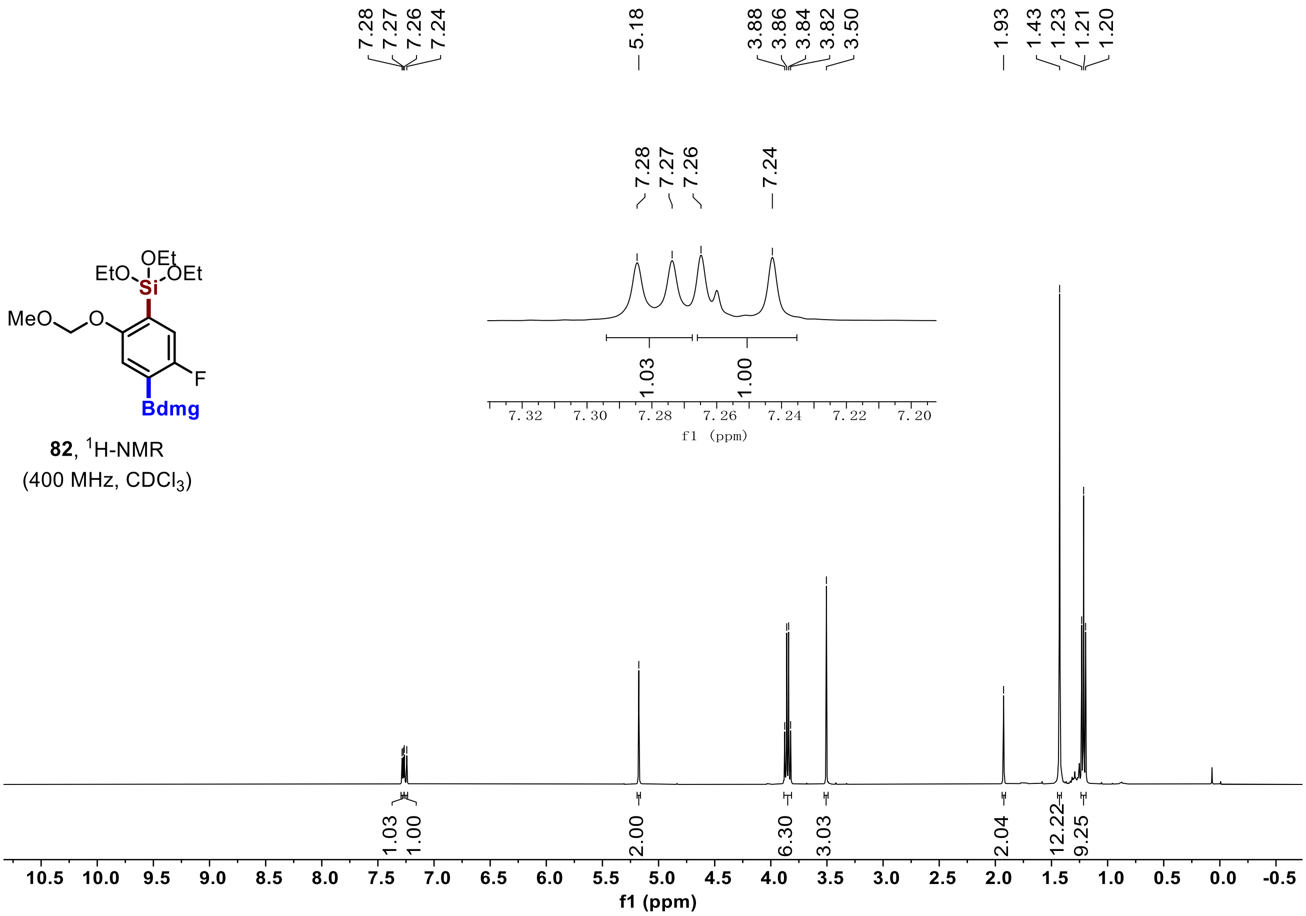

82, ^{1}H-NMR
(400 MHz, $CDCl_3$)

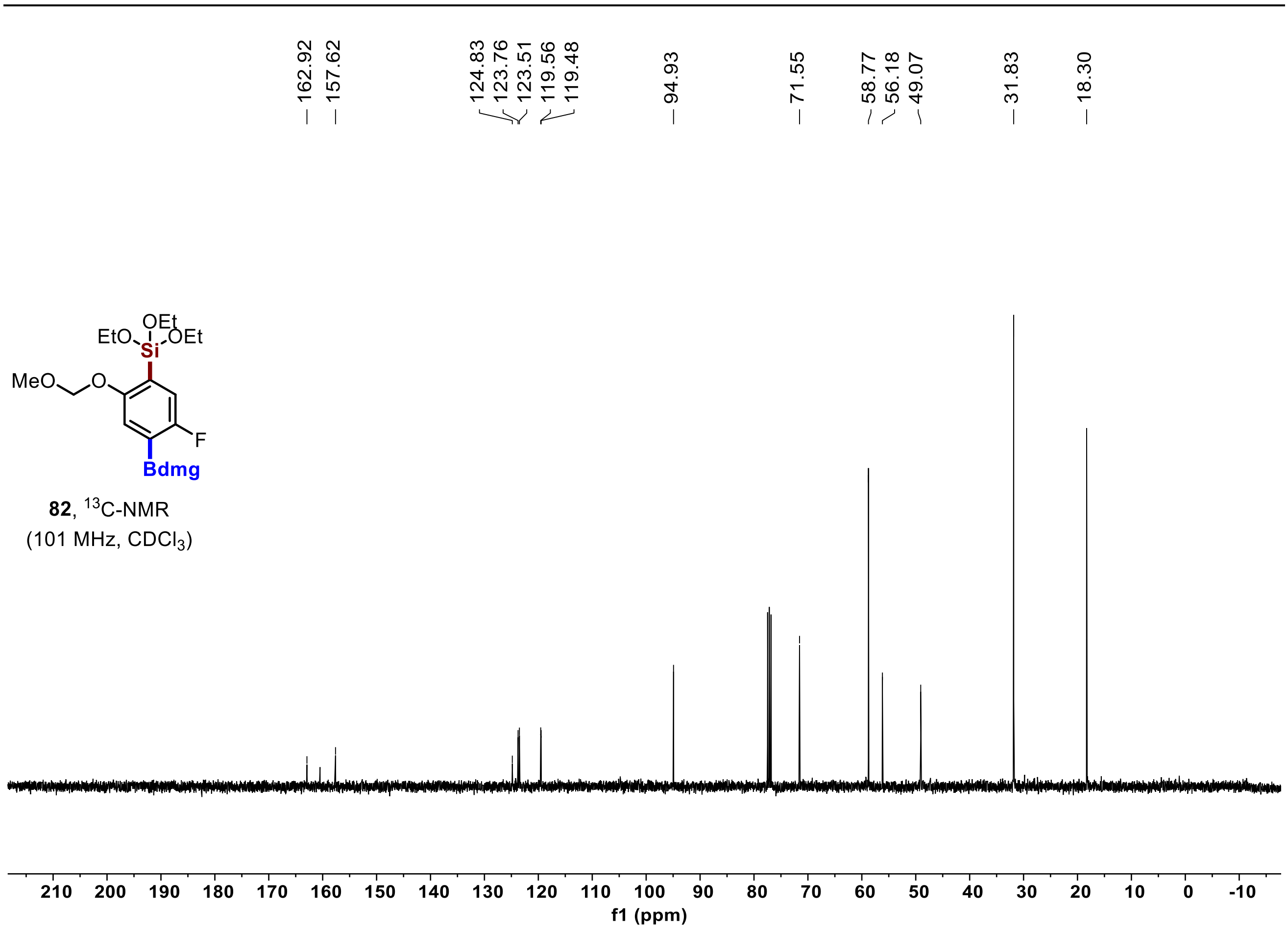

82, ^{13}C-NMR
(101 MHz, $CDCl_3$)

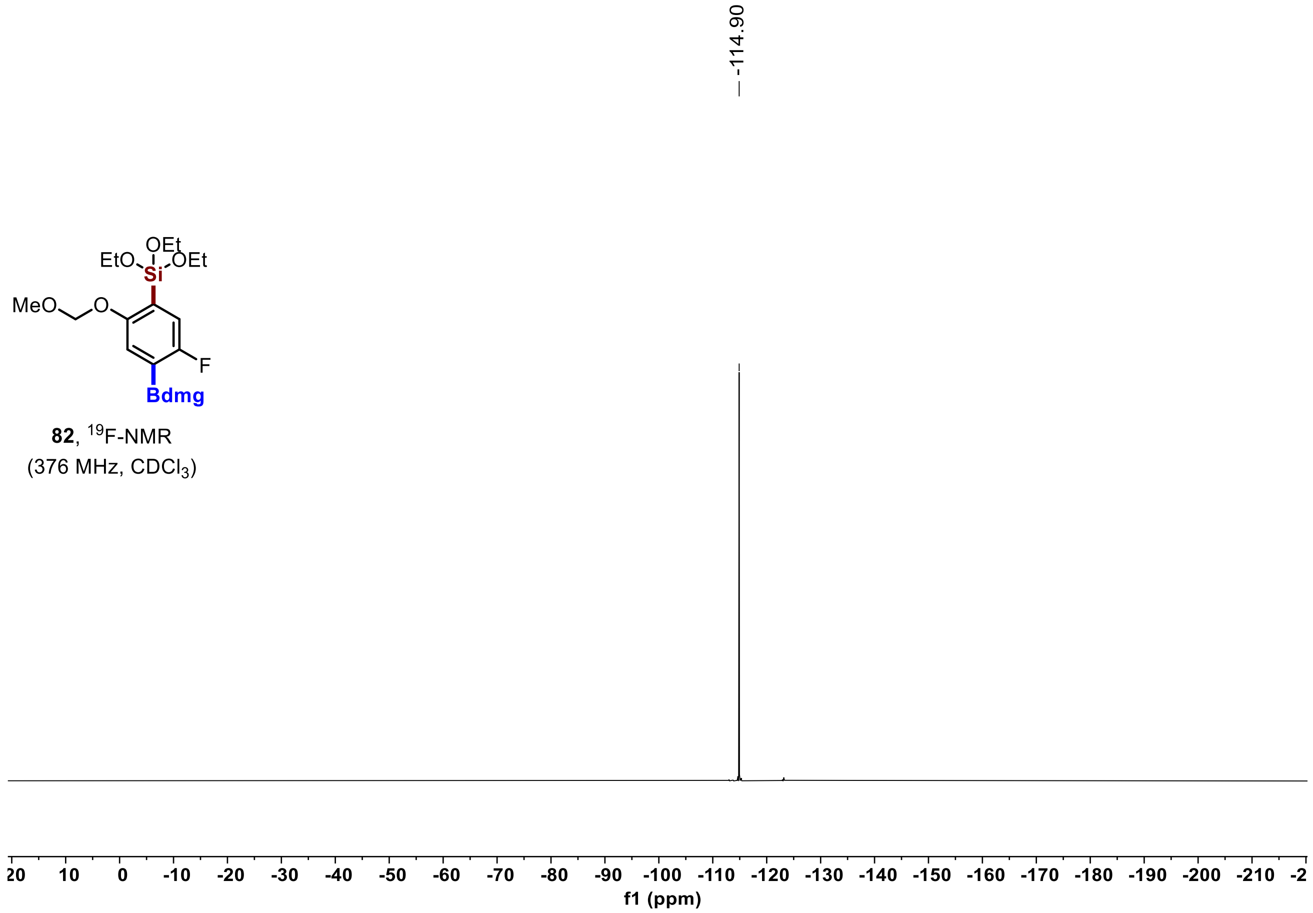

82, ^{19}F-NMR
(376 MHz, $CDCl_3$)

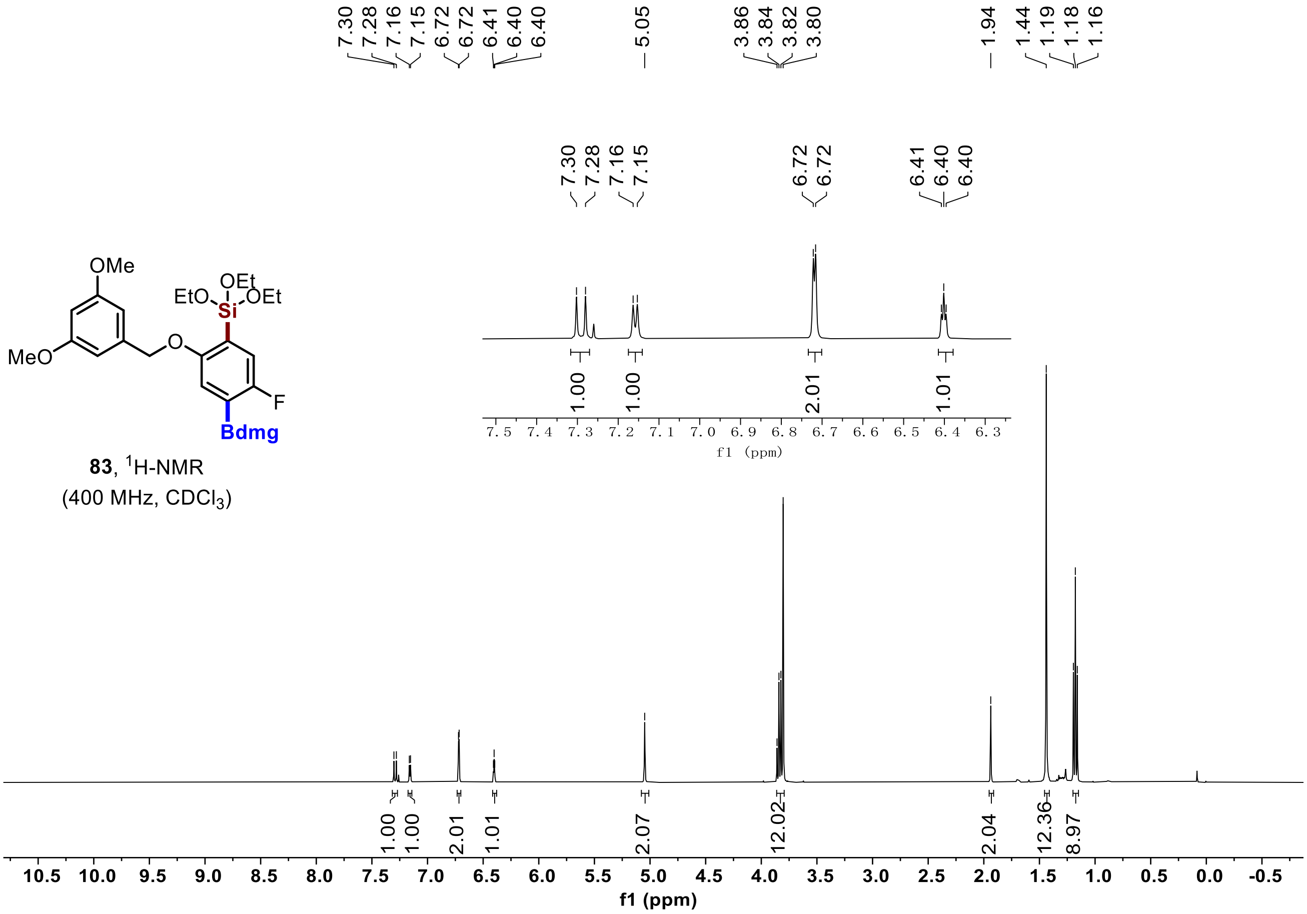

83, ^{1}H-NMR
(400 MHz, $CDCl_3$)

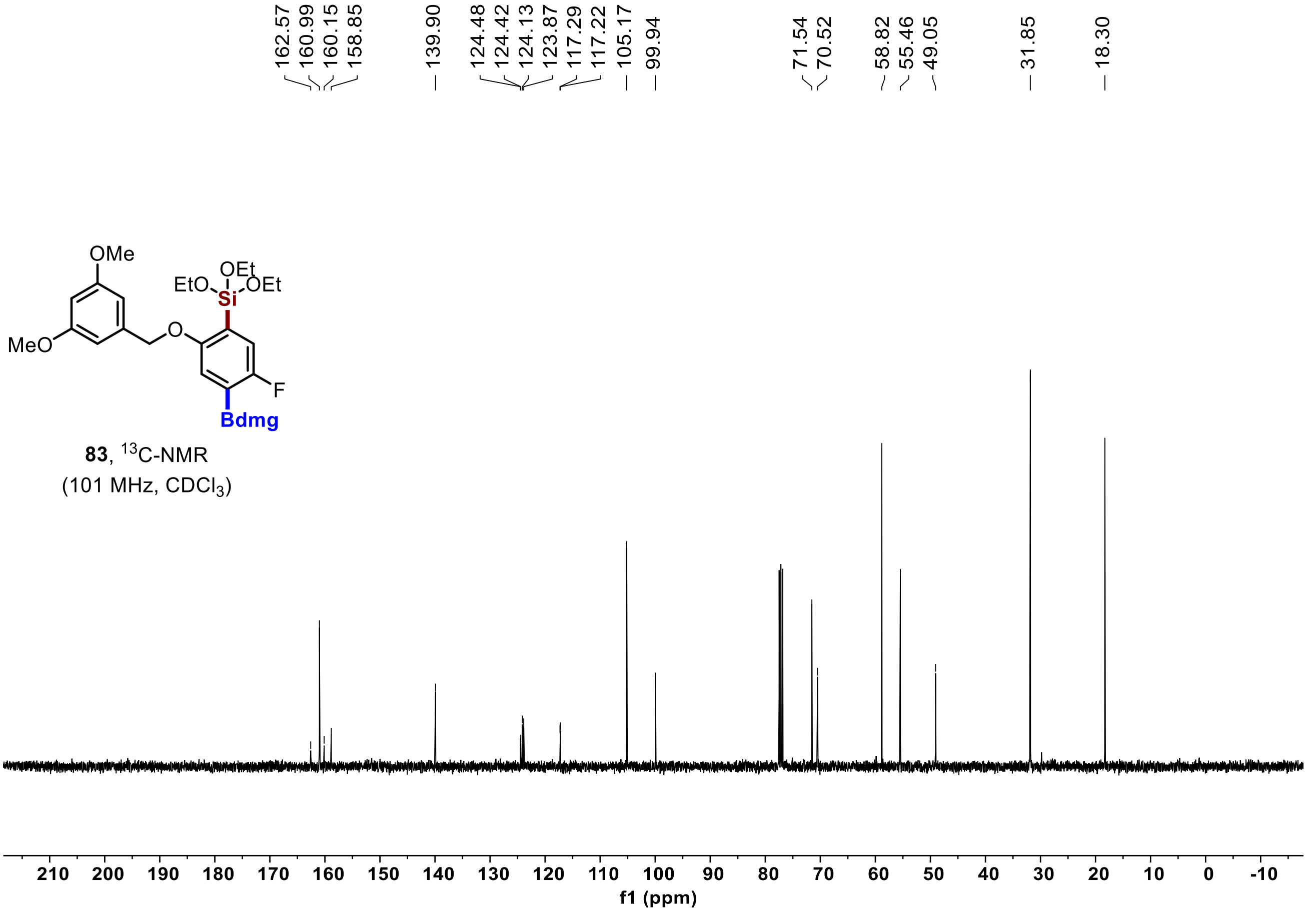

83, ^{13}C-NMR
(101 MHz, $CDCl_3$)

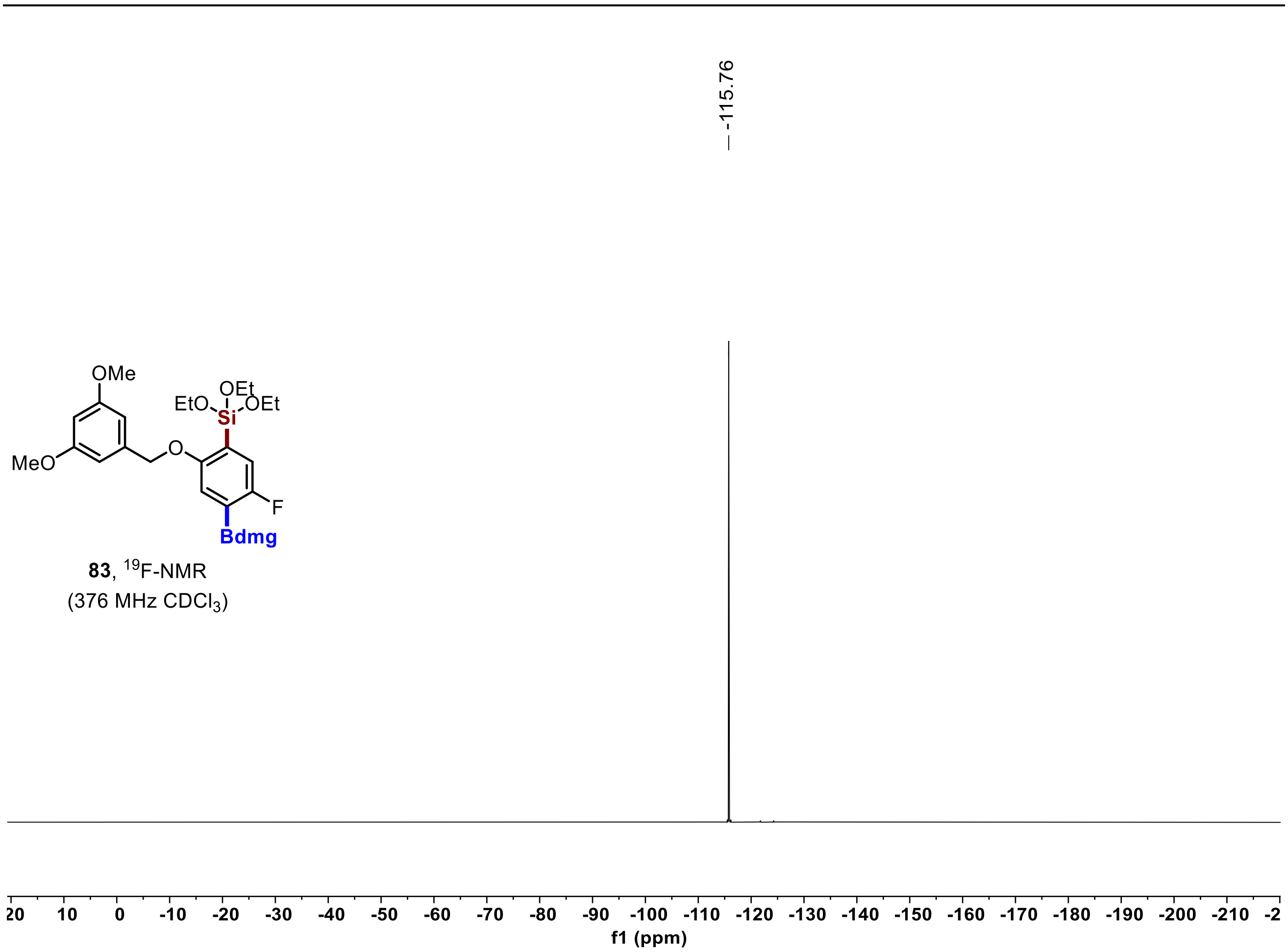
-115.76
OMe
OEt
EtO
Si
OEt
MeO
O
F
Bdmg
83, ^{19}F-NMR
(376 MHz CDCl$_3$)
20 10 0 -10 -20 -30 -40 -50 -60 -70 -80 -90 -100 -110 -120 -130 -140 -150 -160 -170 -180 -190 -200 -210
f1 (ppm)

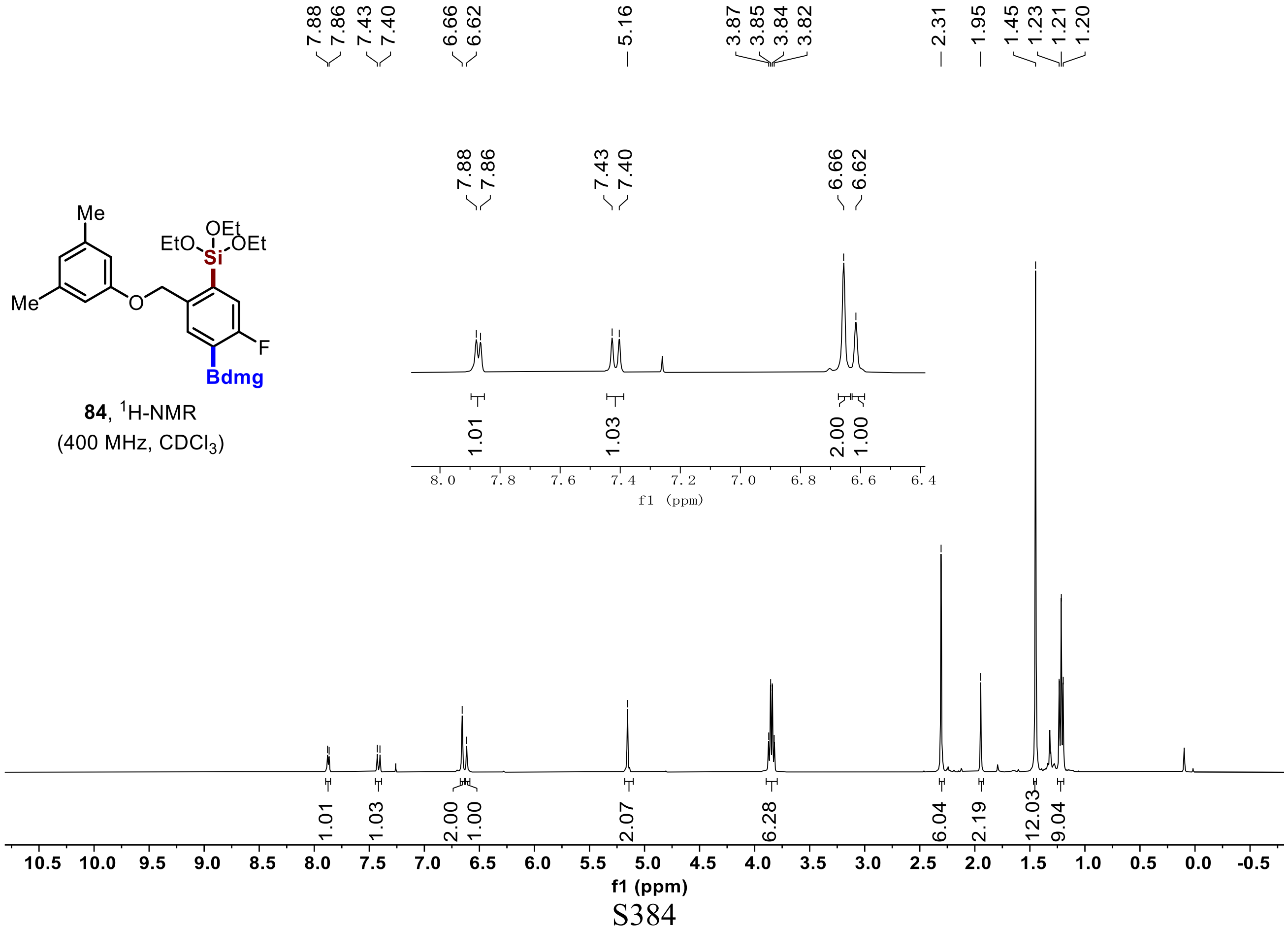
7.88 7.86 7.43 7.40 6.66 6.62 5.16 3.87 3.85 3.84 3.82 2.31 1.95 1.45 1.23 1.21 1.20
Me
OEt
EtO
Si
OEt
Me
O
F
Bdmg
84, ^{1}H-NMR
(400 MHz, CDCl$_3$)
1.01 1.03 2.00 1.00 2.07 6.28 6.04 2.19 12.03 9.04
10.5 10.0 9.5 9.0 8.5 8.0 7.5 7.0 6.5 6.0 5.5 5.0 4.5 4.0 3.5 3.0 2.5 2.0 1.5 1.0 0.5 0.0 -0.5
f1 (ppm)

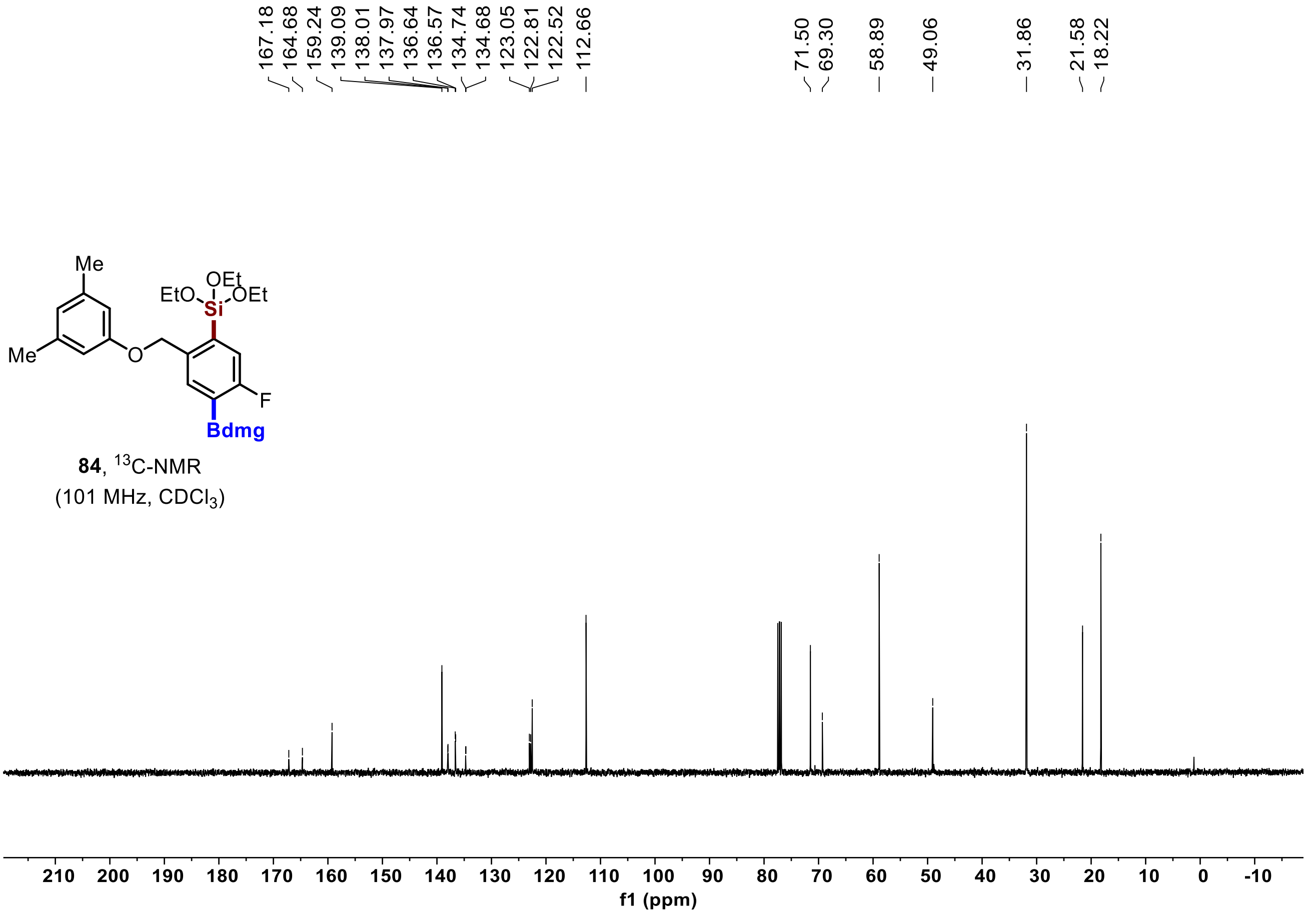

84, ^{13}C-NMR
(101 MHz, $CDCl_3$)

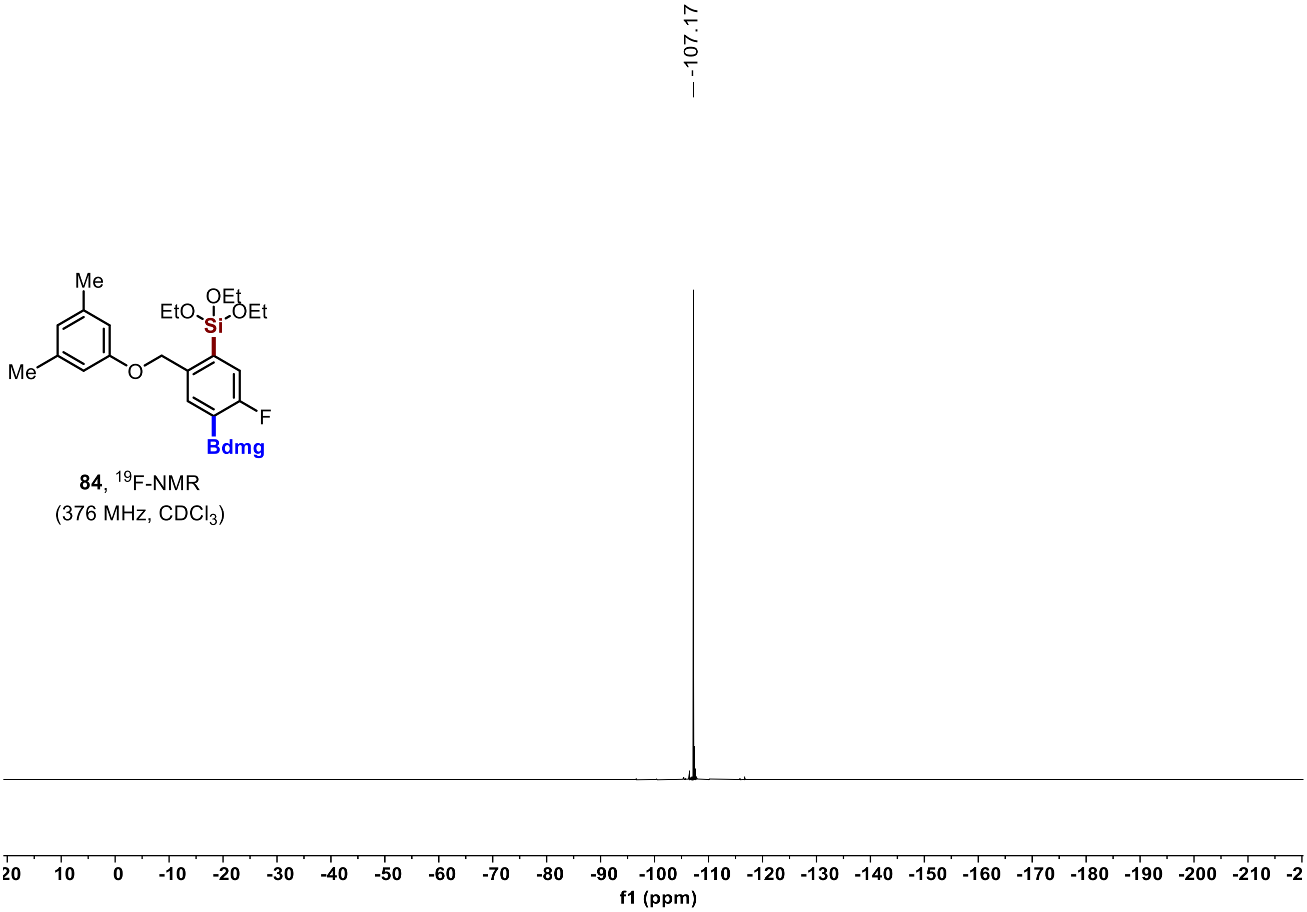

84, ^{19}F-NMR
(376 MHz, $CDCl_3$)

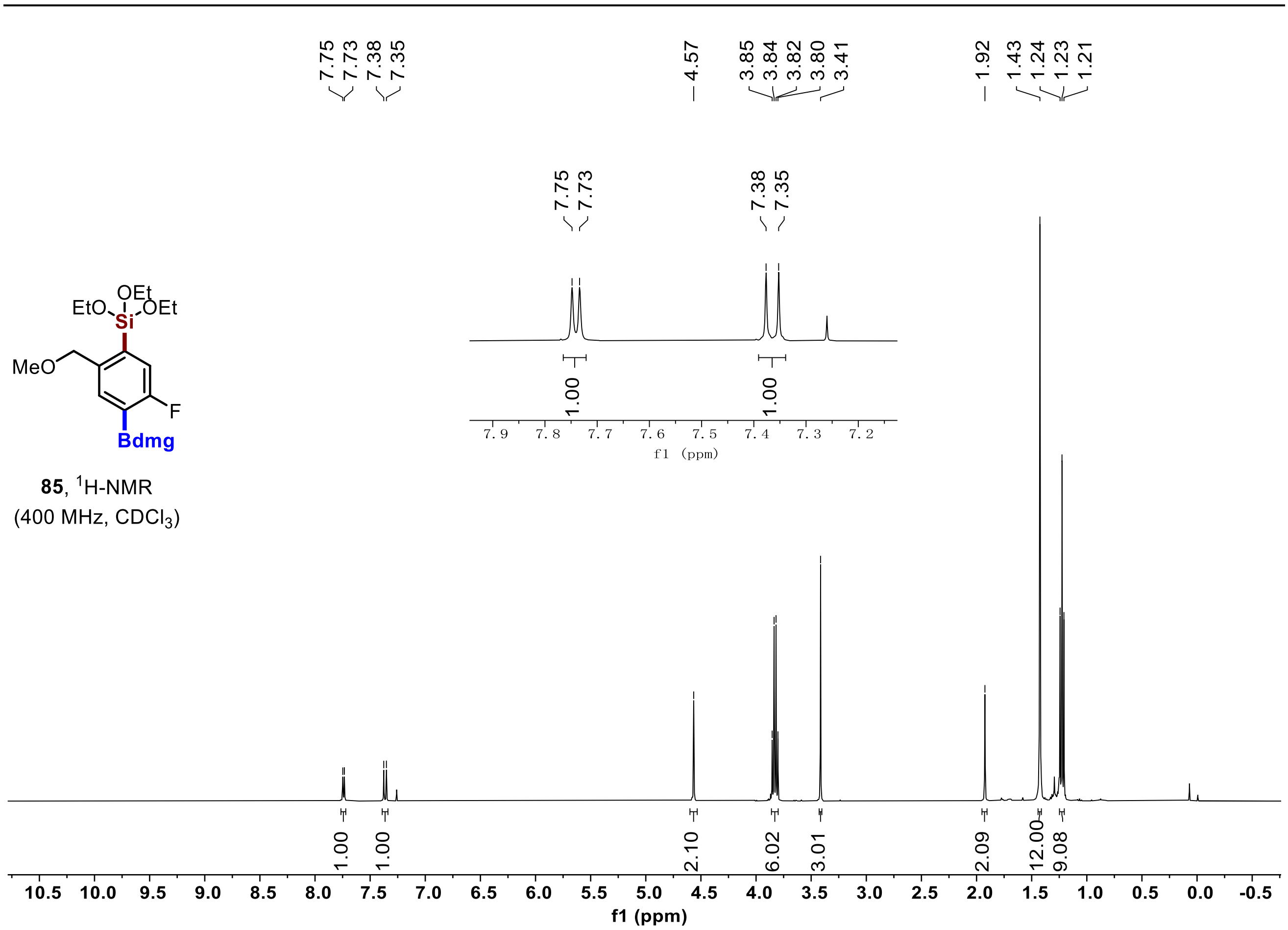

85, ^{1}H-NMR
(400 MHz, $CDCl_3$)

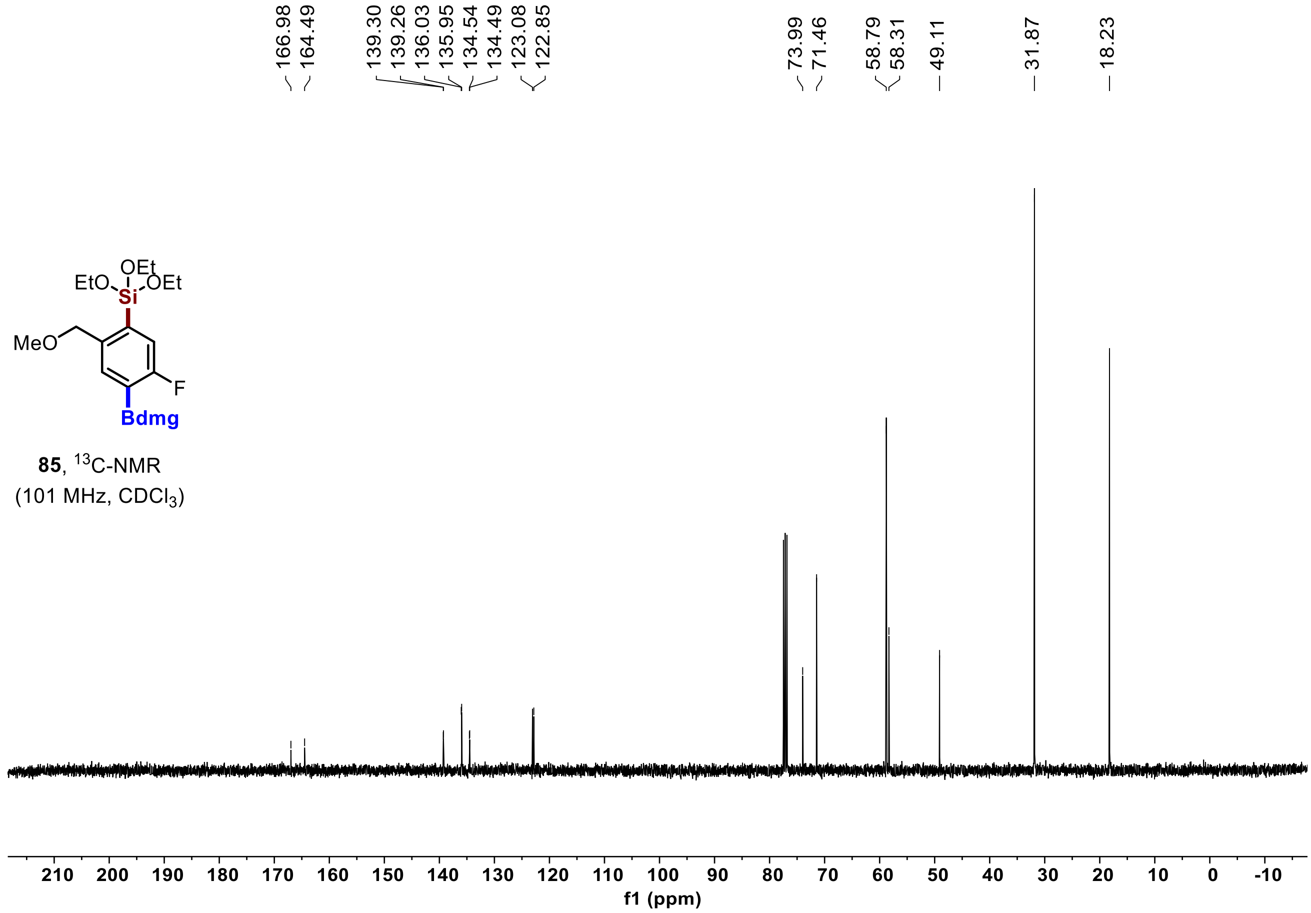

85, ^{13}C-NMR
(101 MHz, $CDCl_3$)

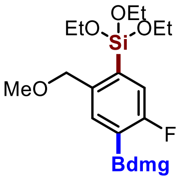

85, ^{19}F-NMR
(376 MHz, $CDCl_3$)

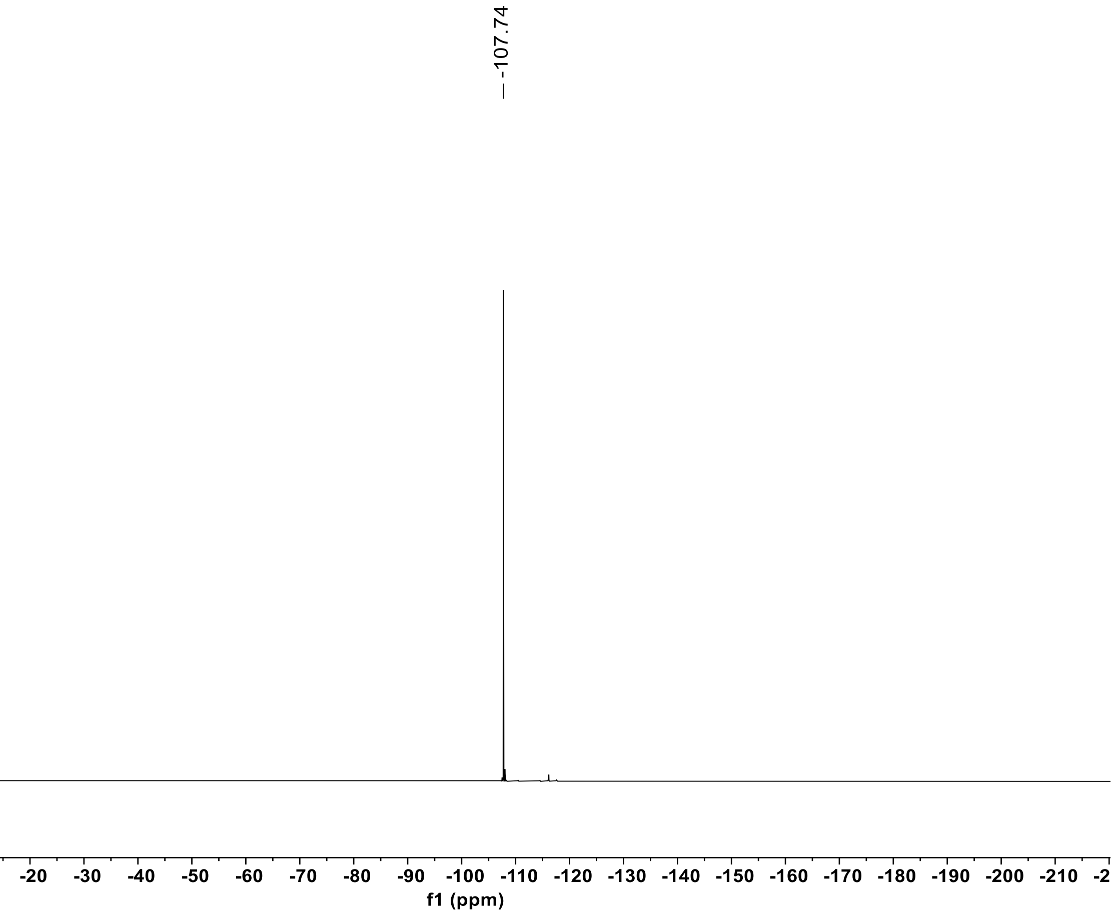

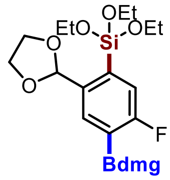

86, ^{1}H-NMR
(400 MHz, $CDCl_3$)

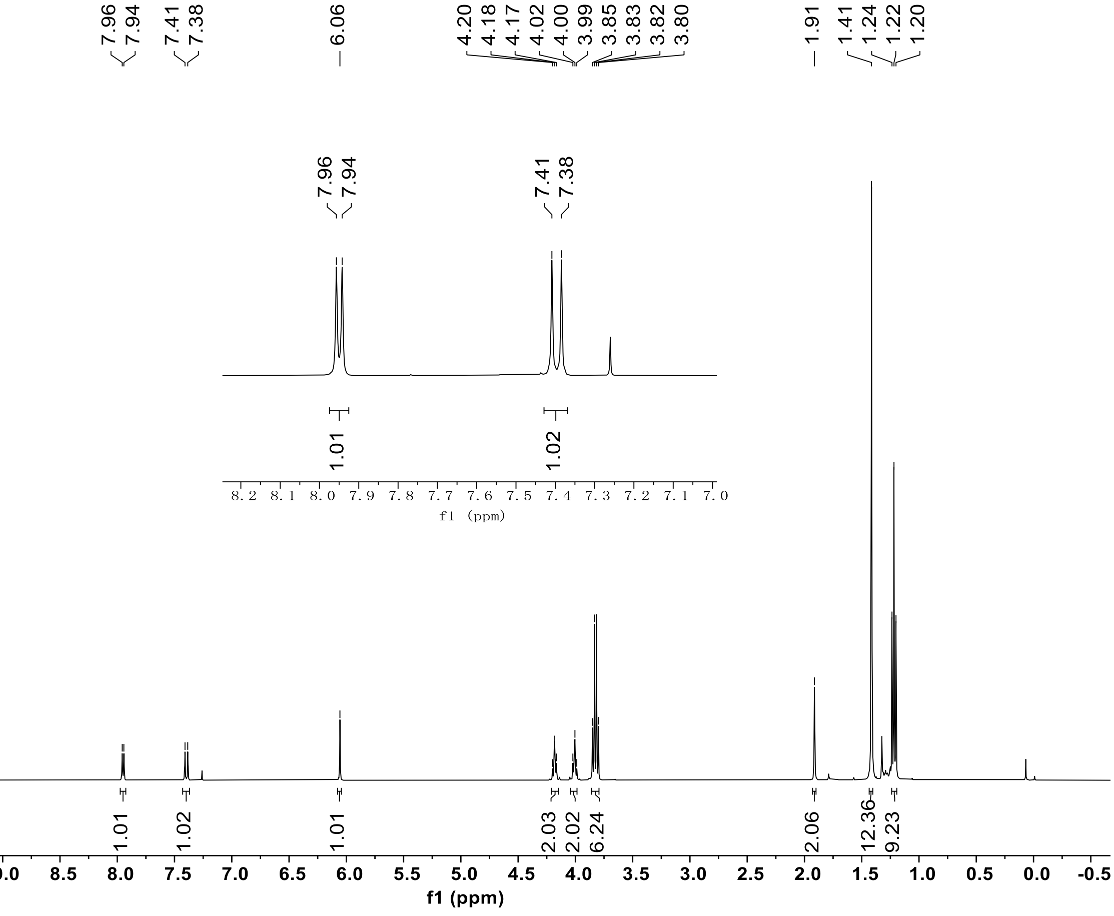

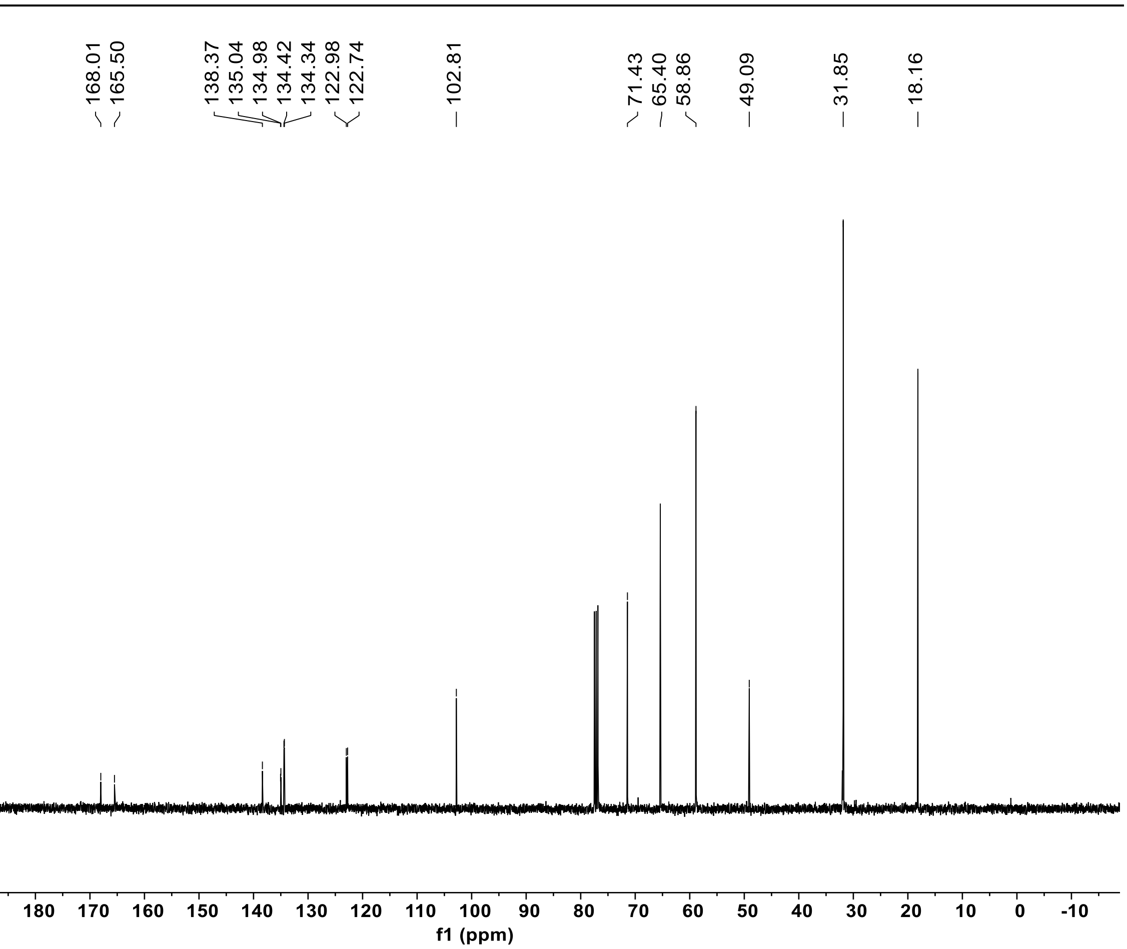

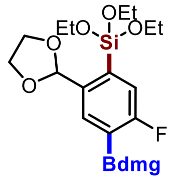

86, ^{13}C-NMR
(101 MHz, $CDCl_3$)

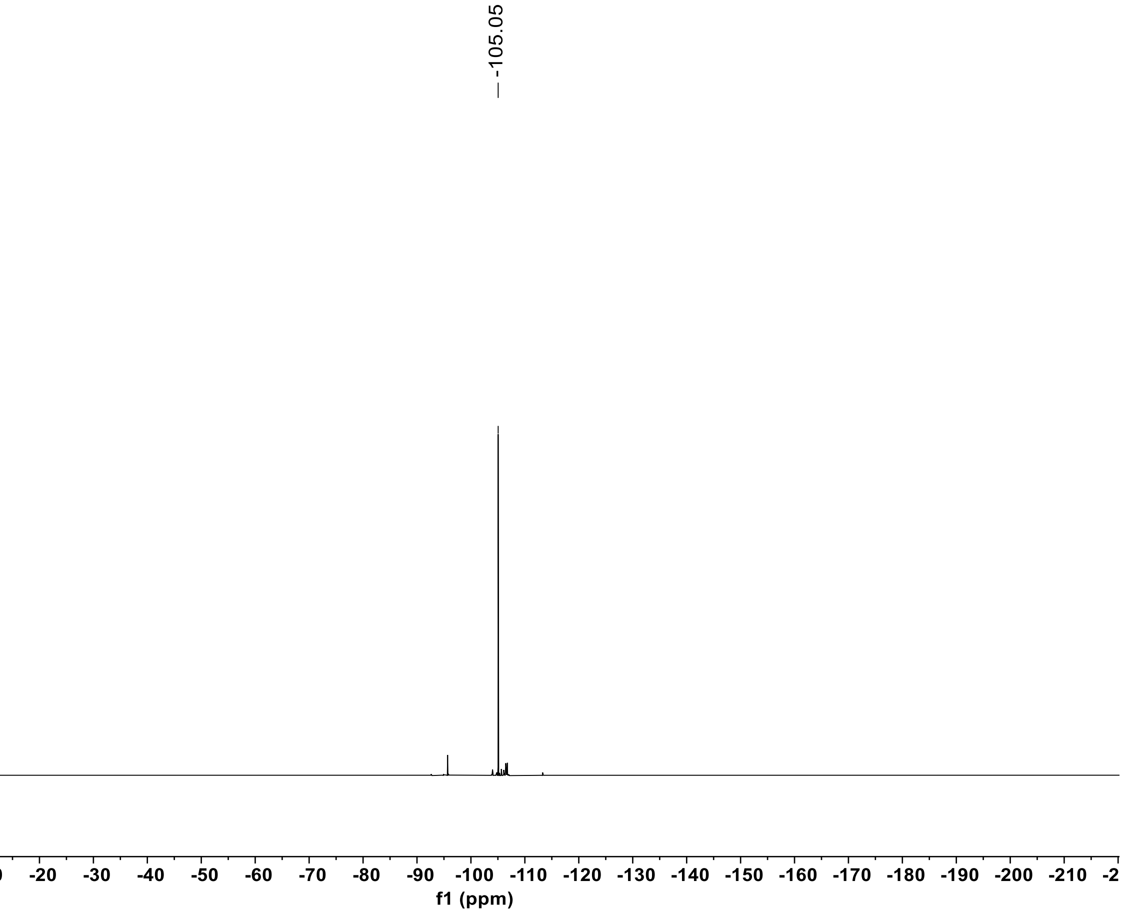

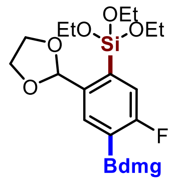

86, ^{19}F-NMR
(376 MHz, $CDCl_3$)

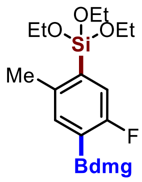

87, ^{1}H-NMR
(400 MHz, $CDCl_3$)

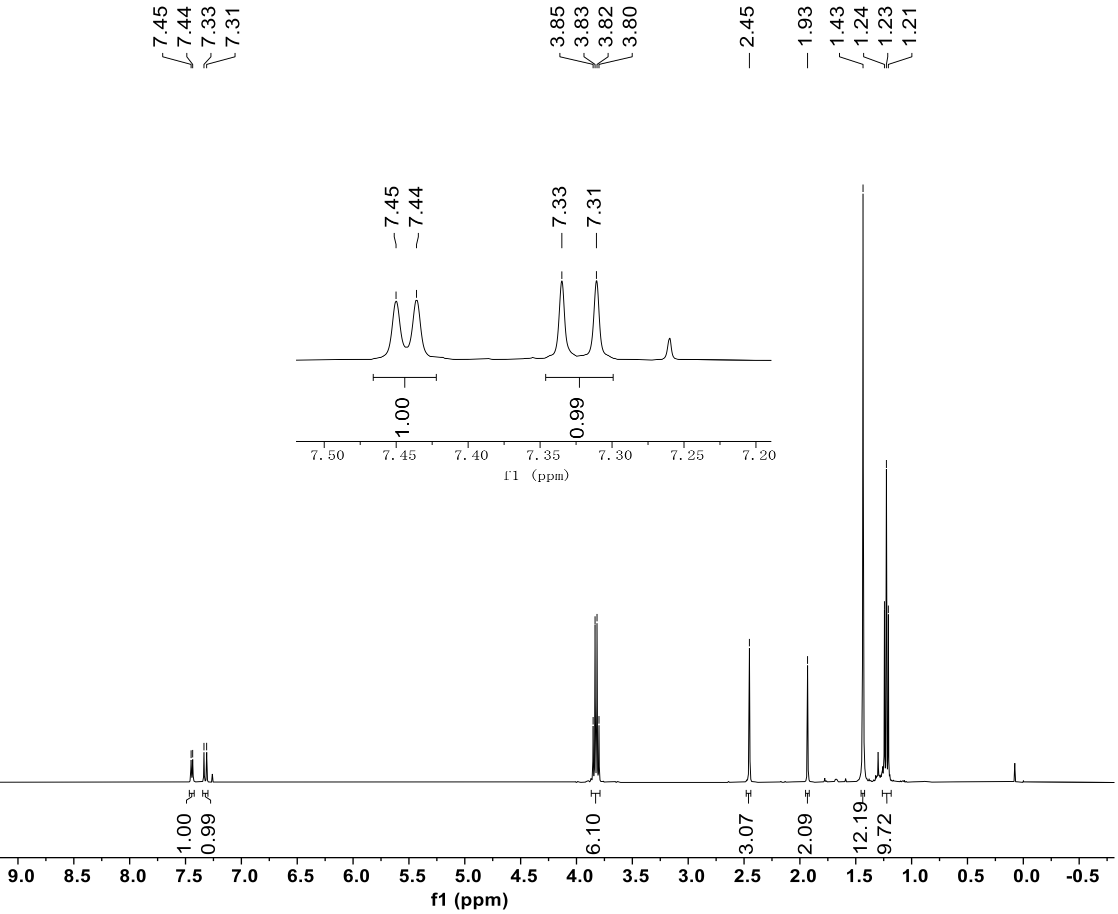

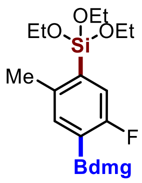

87, ^{13}C-NMR
(101 MHz, $CDCl_3$)

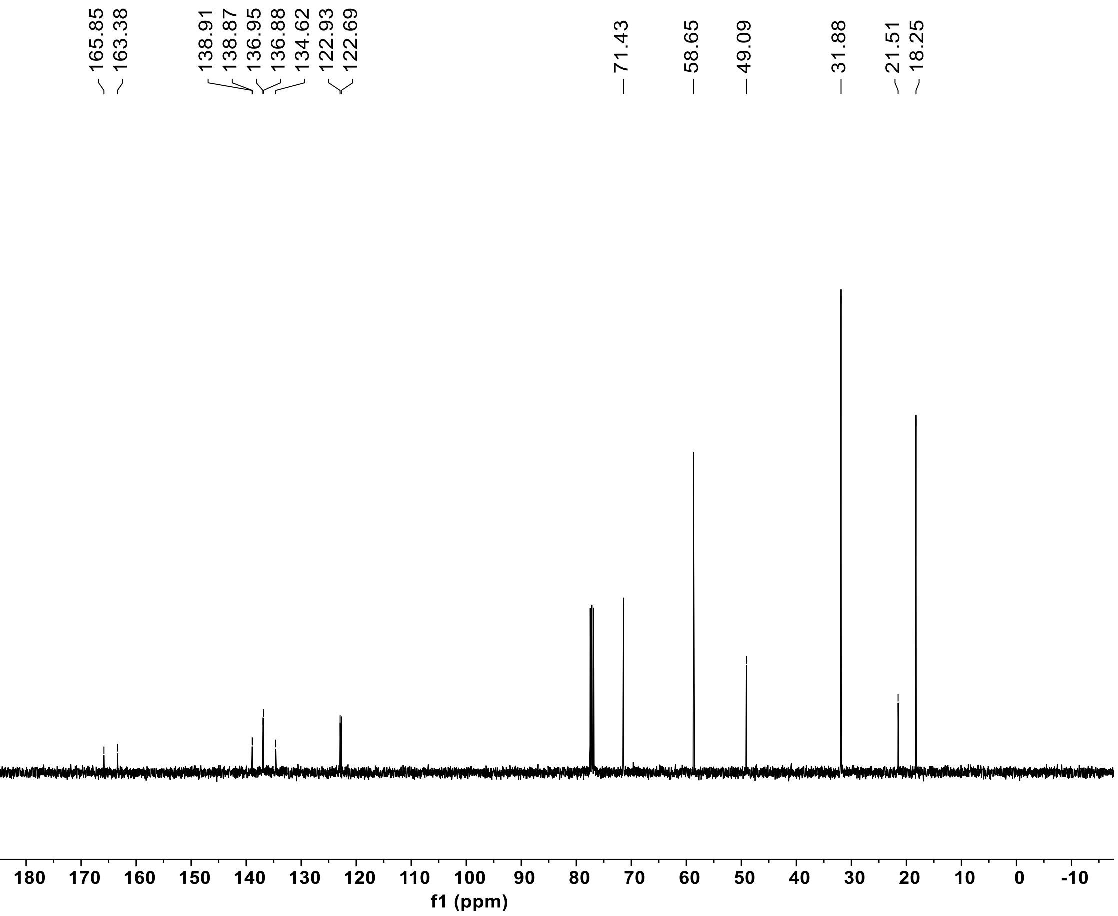

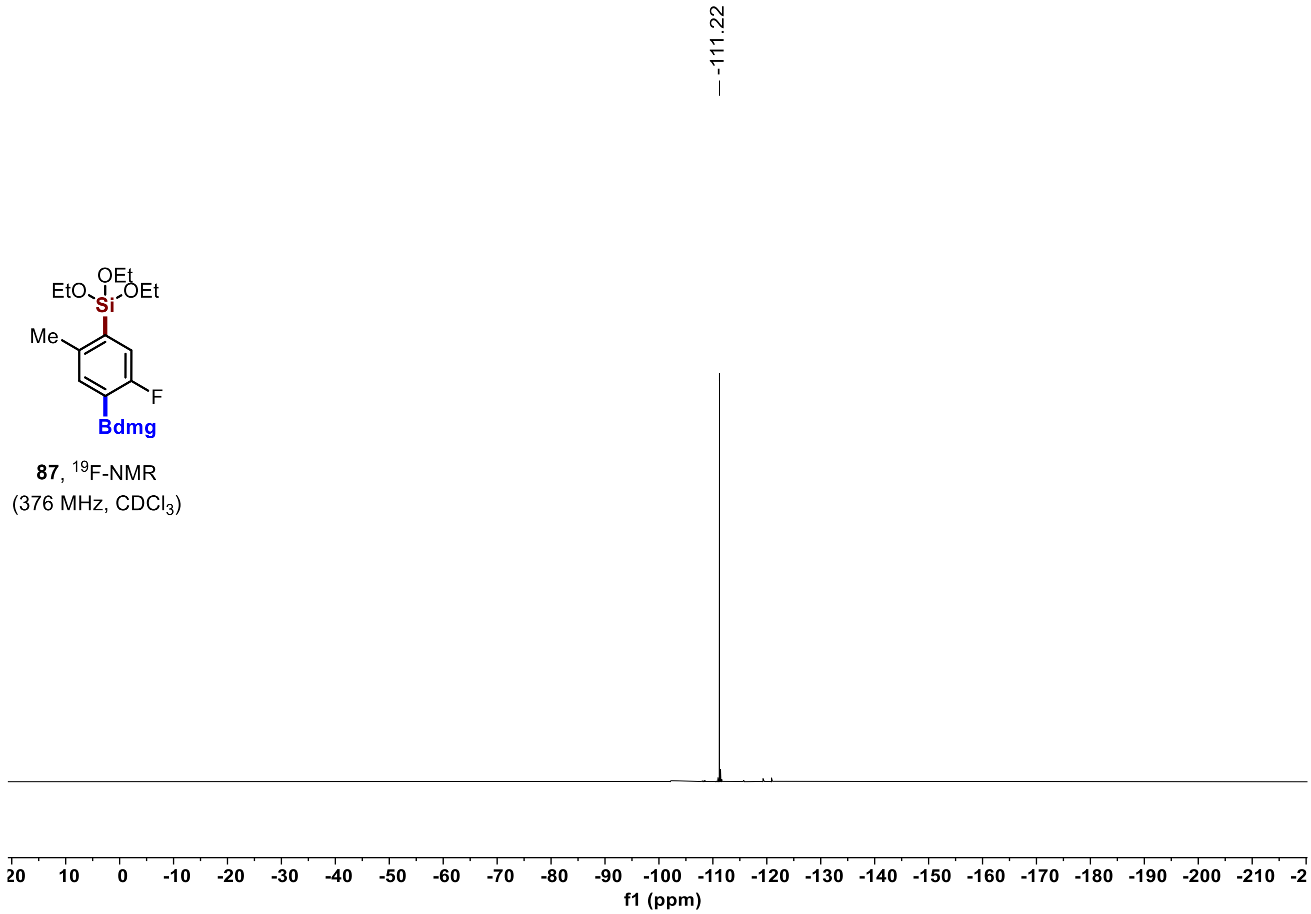

87, ^{19}F-NMR
(376 MHz, $CDCl_3$)

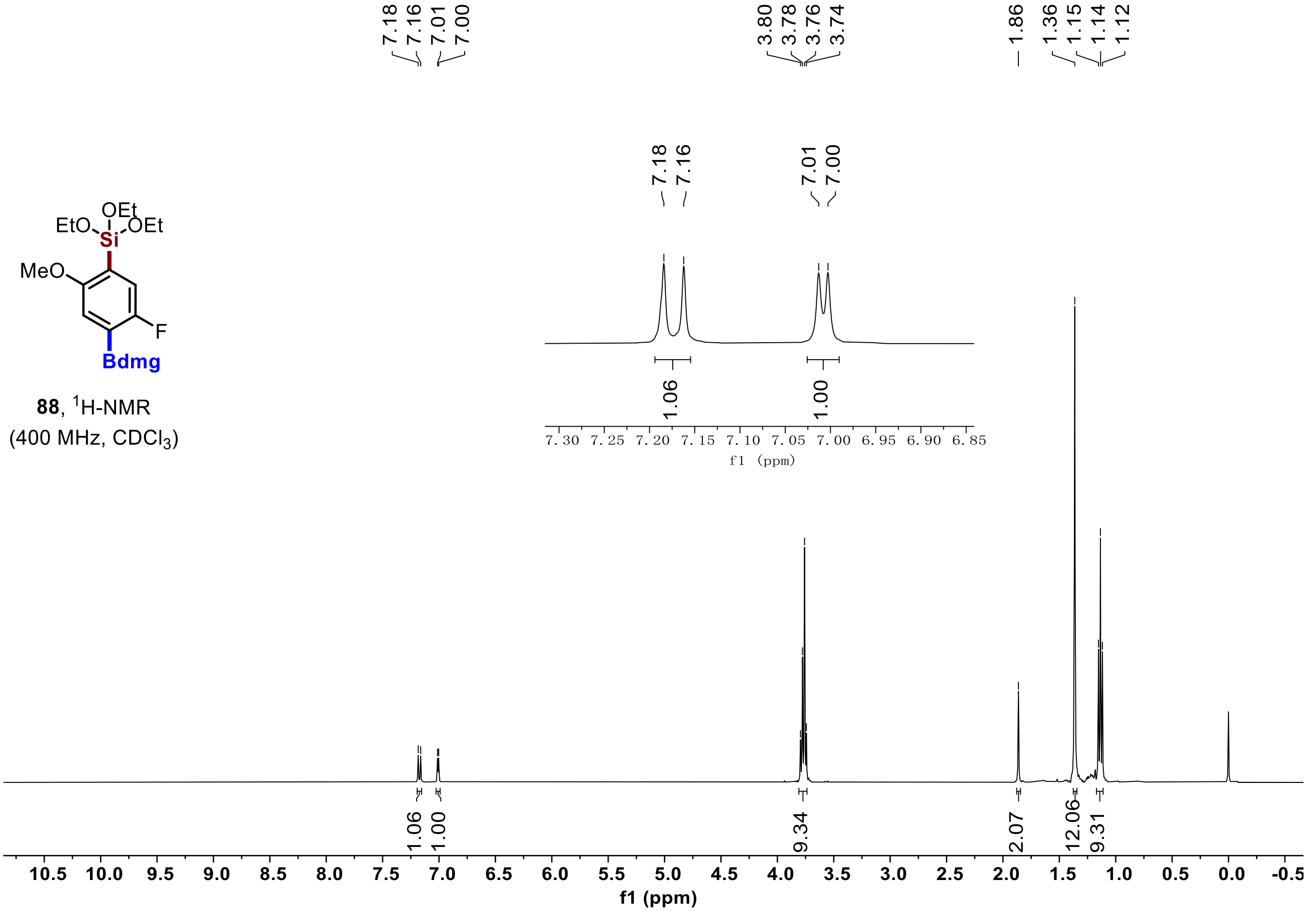

88, ^{1}H-NMR
(400 MHz, $CDCl_3$)

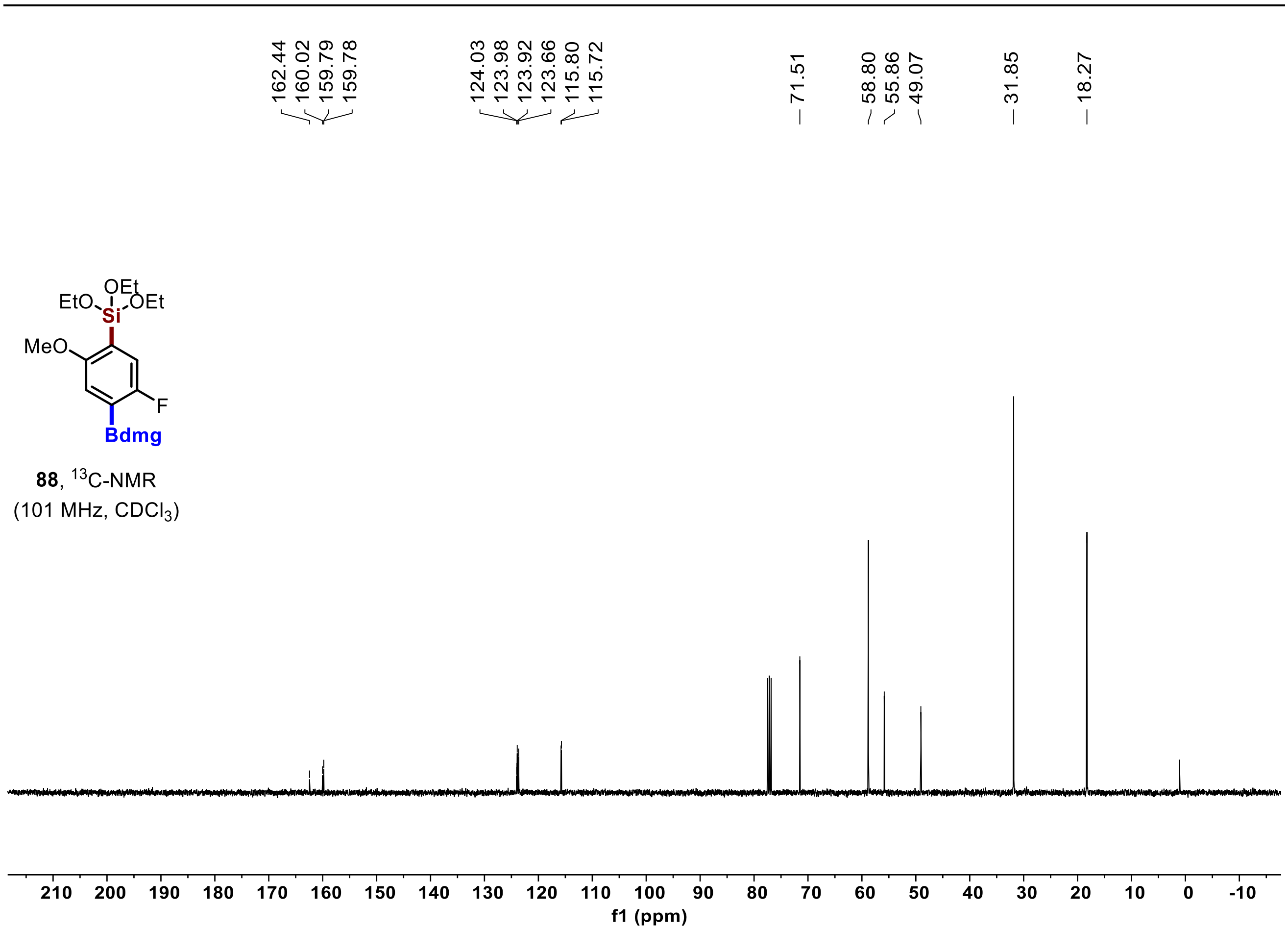
162.44
160.02
159.79
159.78
124.03
123.98
123.92
123.66
115.80
115.72
71.51
58.80
55.86
49.07
31.85
18.27
EtO
OEt
OEt
Si
MeO
F
Bdmg
88, ^{13}C-NMR
(101 MHz, $CDCl_3$)
210 200 190 180 170 160 150 140 130 120 110 100 90 80 70 60 50 40 30 20 10 0 -10
f1 (ppm)

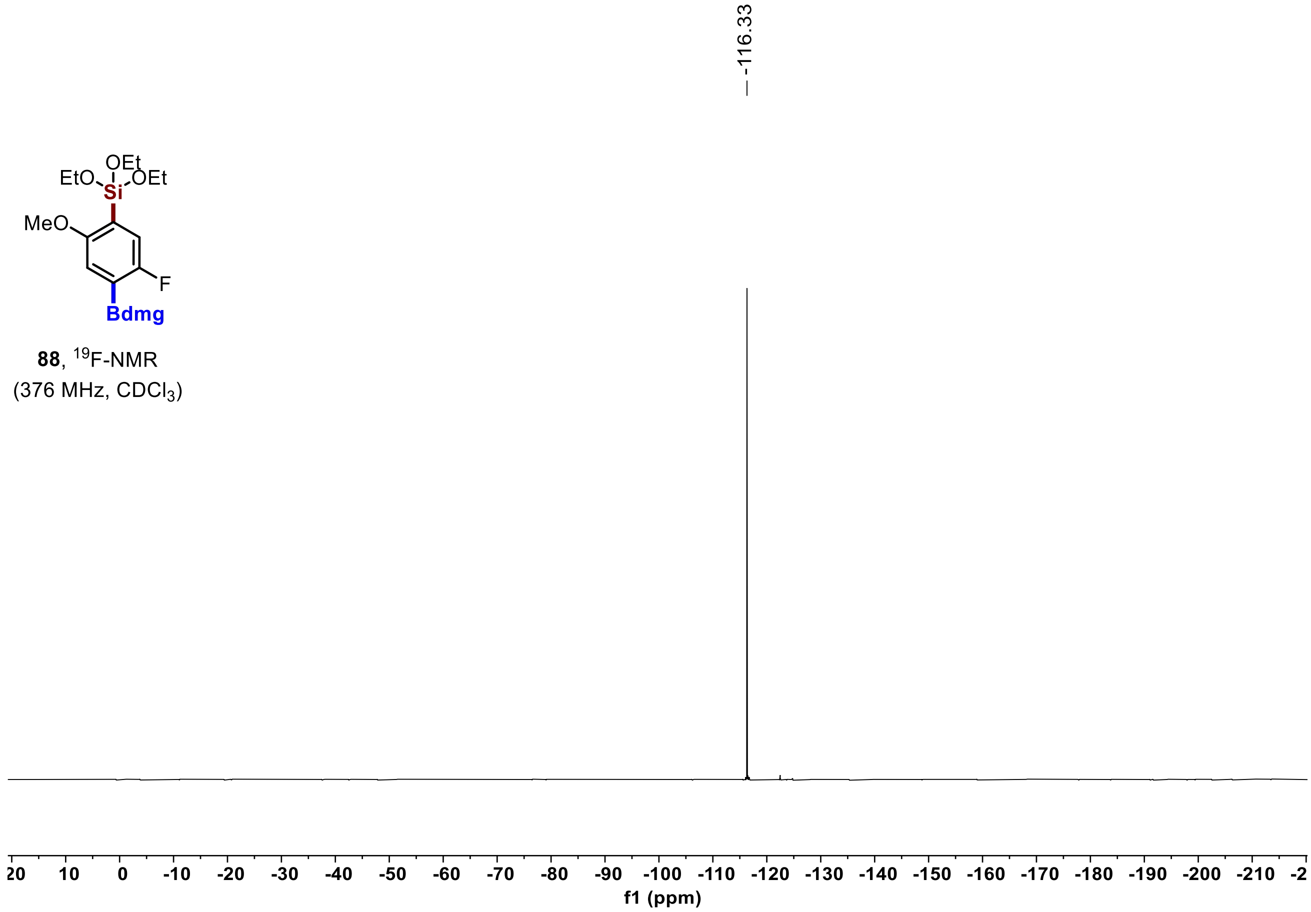
-116.33
EtO
OEt
OEt
Si
MeO
F
Bdmg
88, ^{19}F-NMR
(376 MHz, $CDCl_3$)
20 10 0 -10 -20 -30 -40 -50 -60 -70 -80 -90 -100 -110 -120 -130 -140 -150 -160 -170 -180 -190 -200 -210 -2
f1 (ppm)

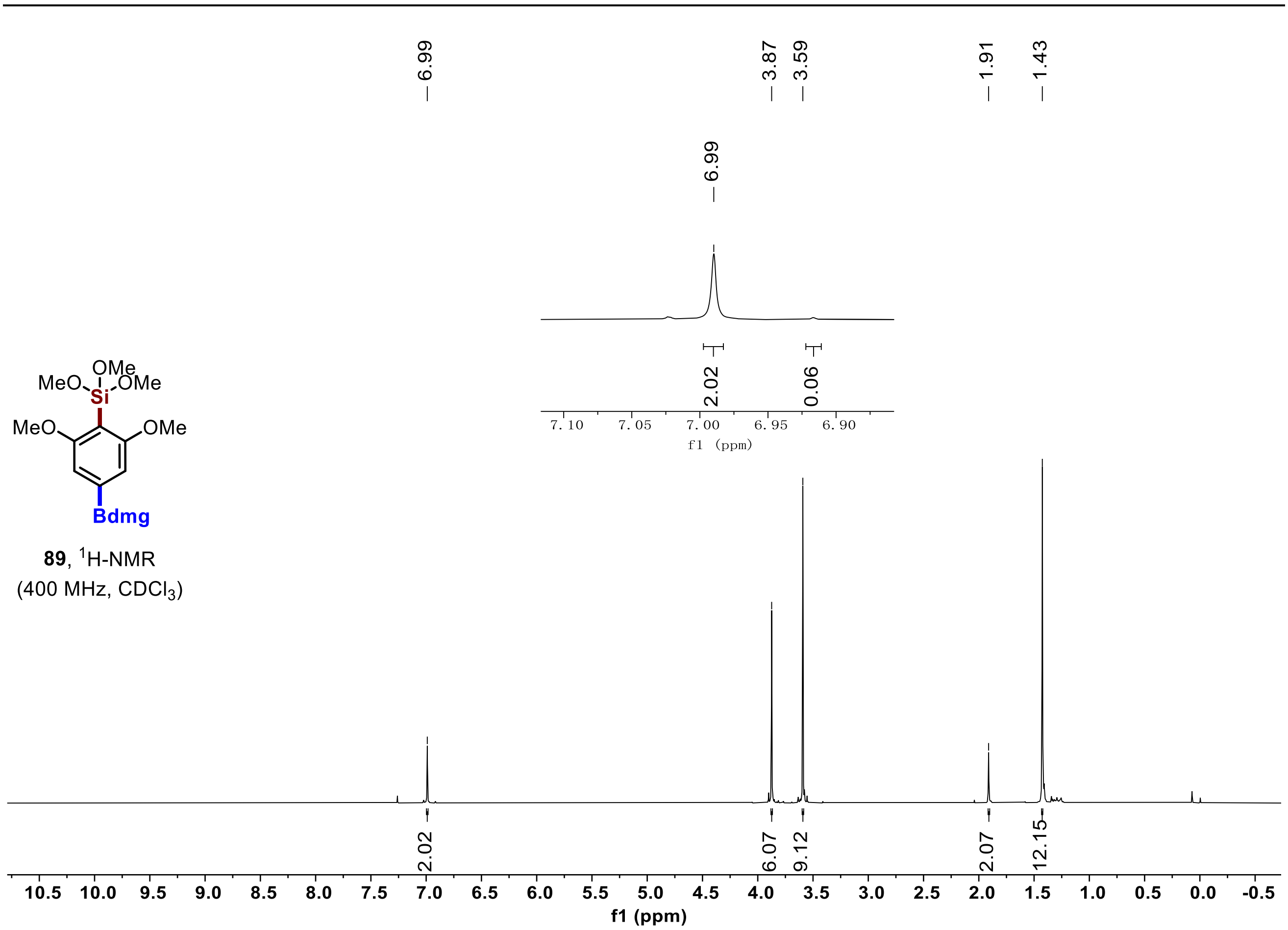
6.99
3.87
3.59
1.91
1.43
2.02
0.06
7.10 7.05 7.00 6.95 6.90
f1 (ppm)
MeO OMe OMe Si
MeO OMe
Bdmg
89, ^{1}H-NMR
(400 MHz, $CDCl_3$)
2.02
6.07
9.12
2.07
12.15
10.5 10.0 9.5 9.0 8.5 8.0 7.5 7.0 6.5 6.0 5.5 5.0 4.5 4.0 3.5 3.0 2.5 2.0 1.5 1.0 0.5 0.0 -0.5
f1 (ppm)

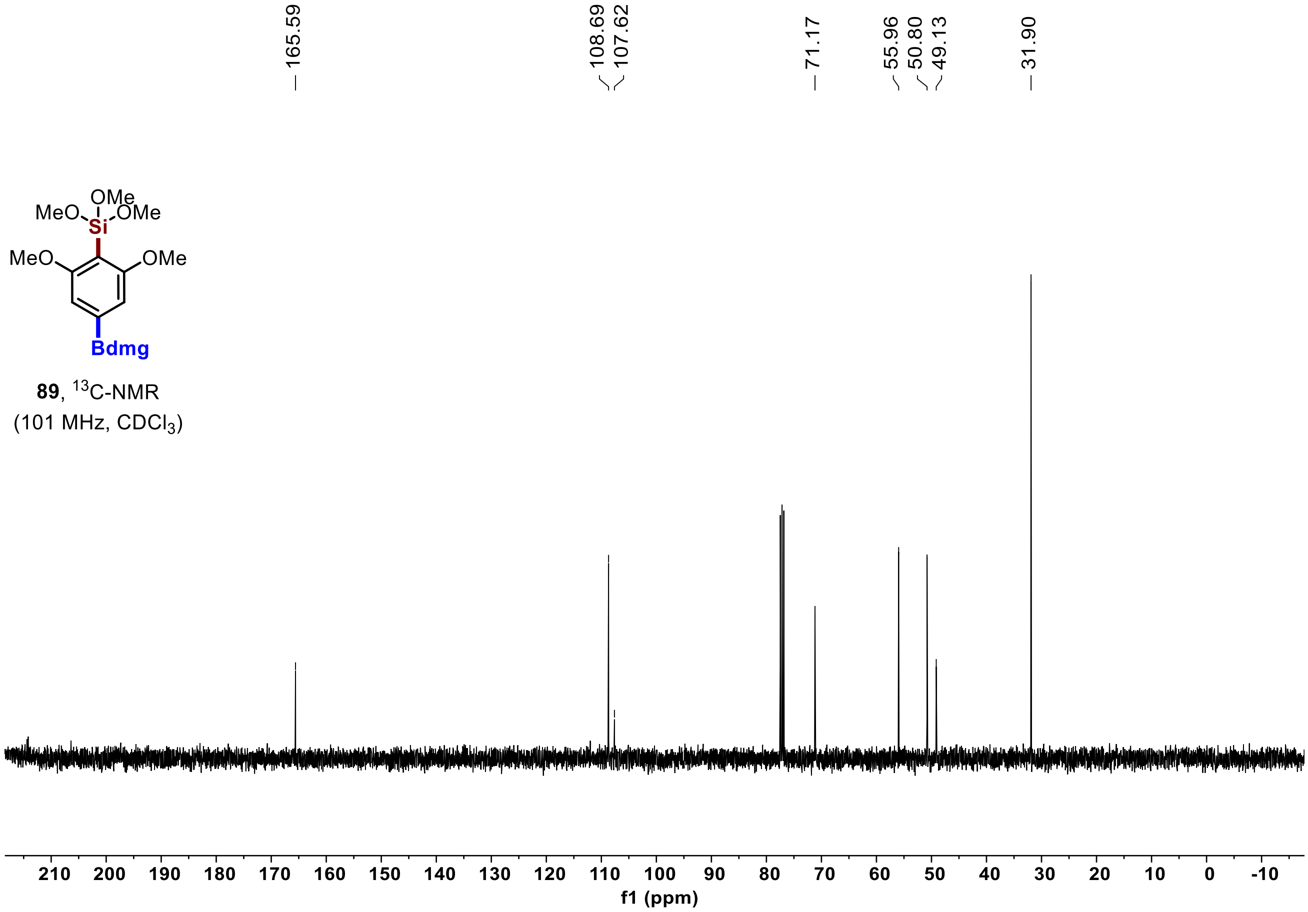
165.59
108.69
107.62
71.17
55.96
50.80
49.13
31.90
MeO OMe OMe Si
MeO OMe
Bdmg
89, ^{13}C-NMR
(101 MHz, $CDCl_3$)
210 200 190 180 170 160 150 140 130 120 110 100 90 80 70 60 50 40 30 20 10 0 -10
f1 (ppm)

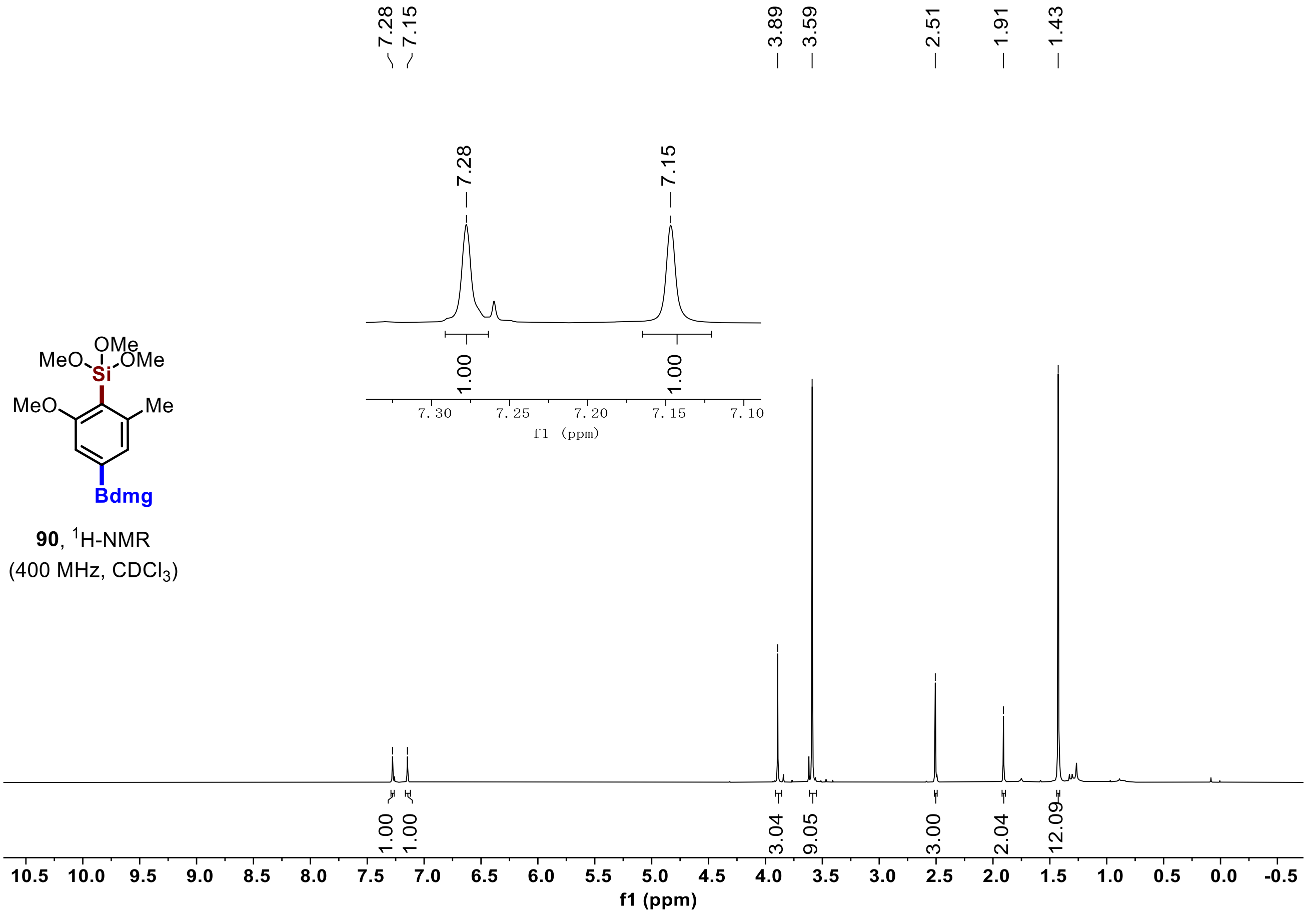

90, ^{1}H-NMR
(400 MHz, $CDCl_3$)

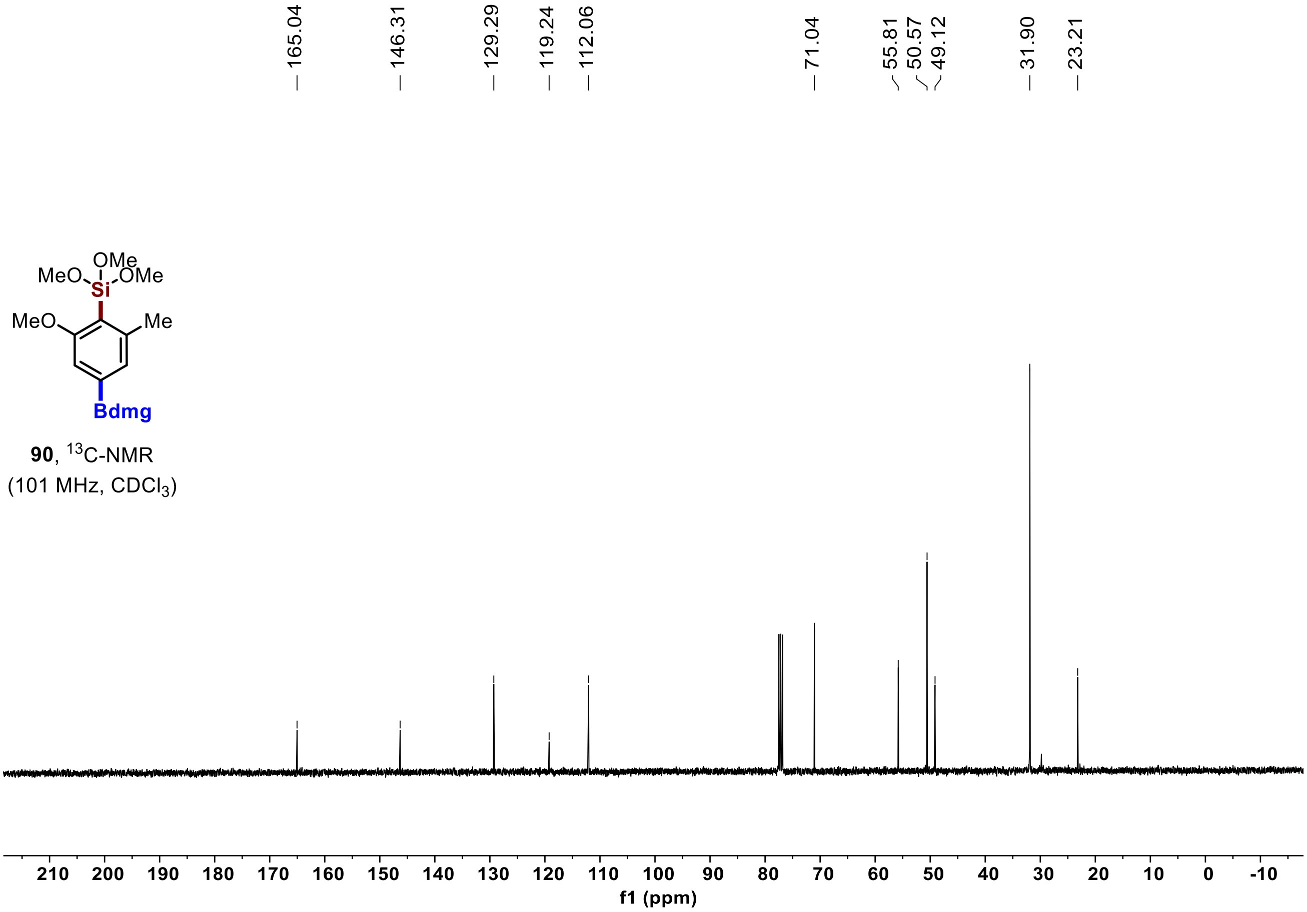

90, ^{13}C-NMR
(101 MHz, $CDCl_3$)

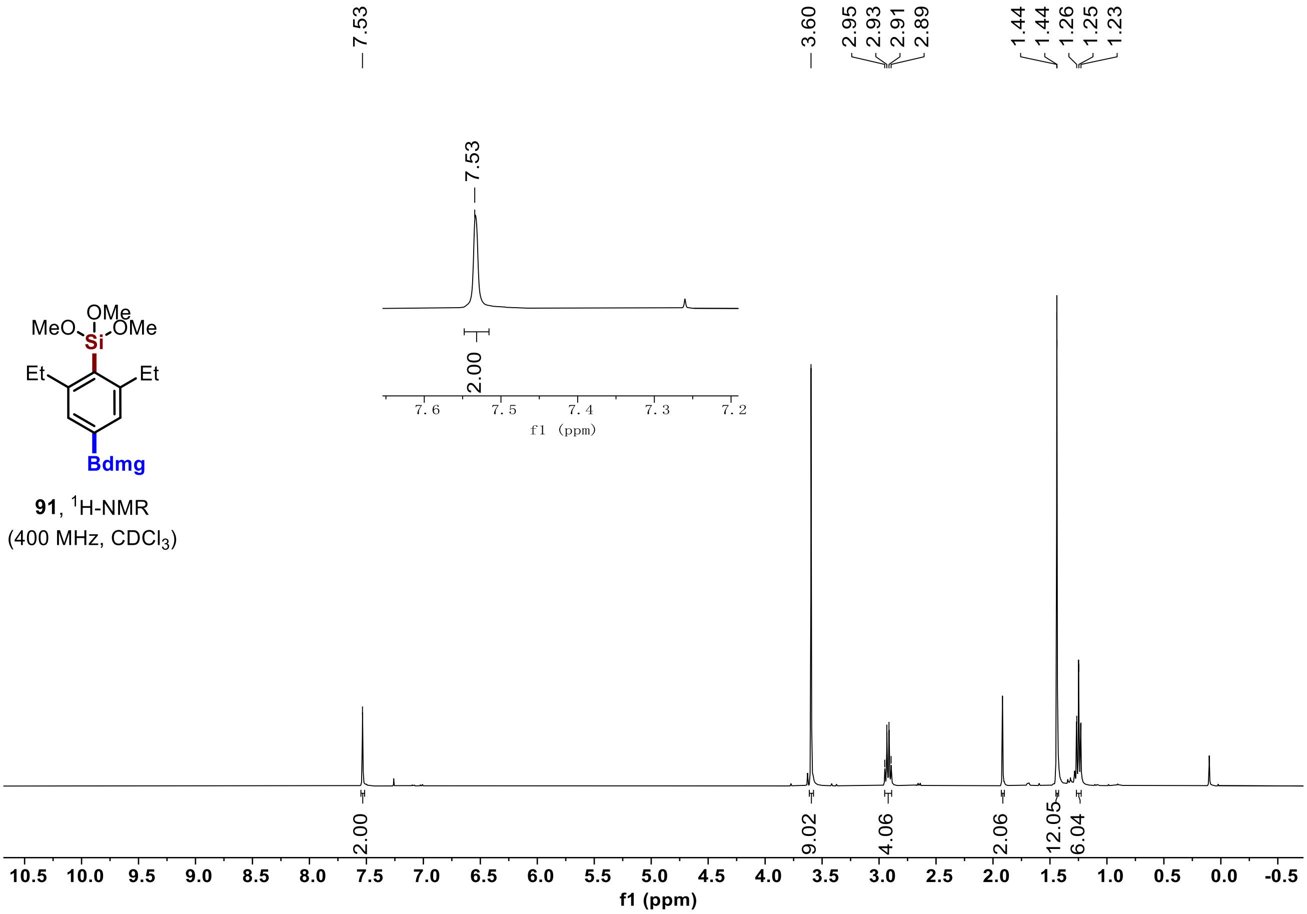

91, ^{1}H-NMR
(400 MHz, $CDCl_3$)

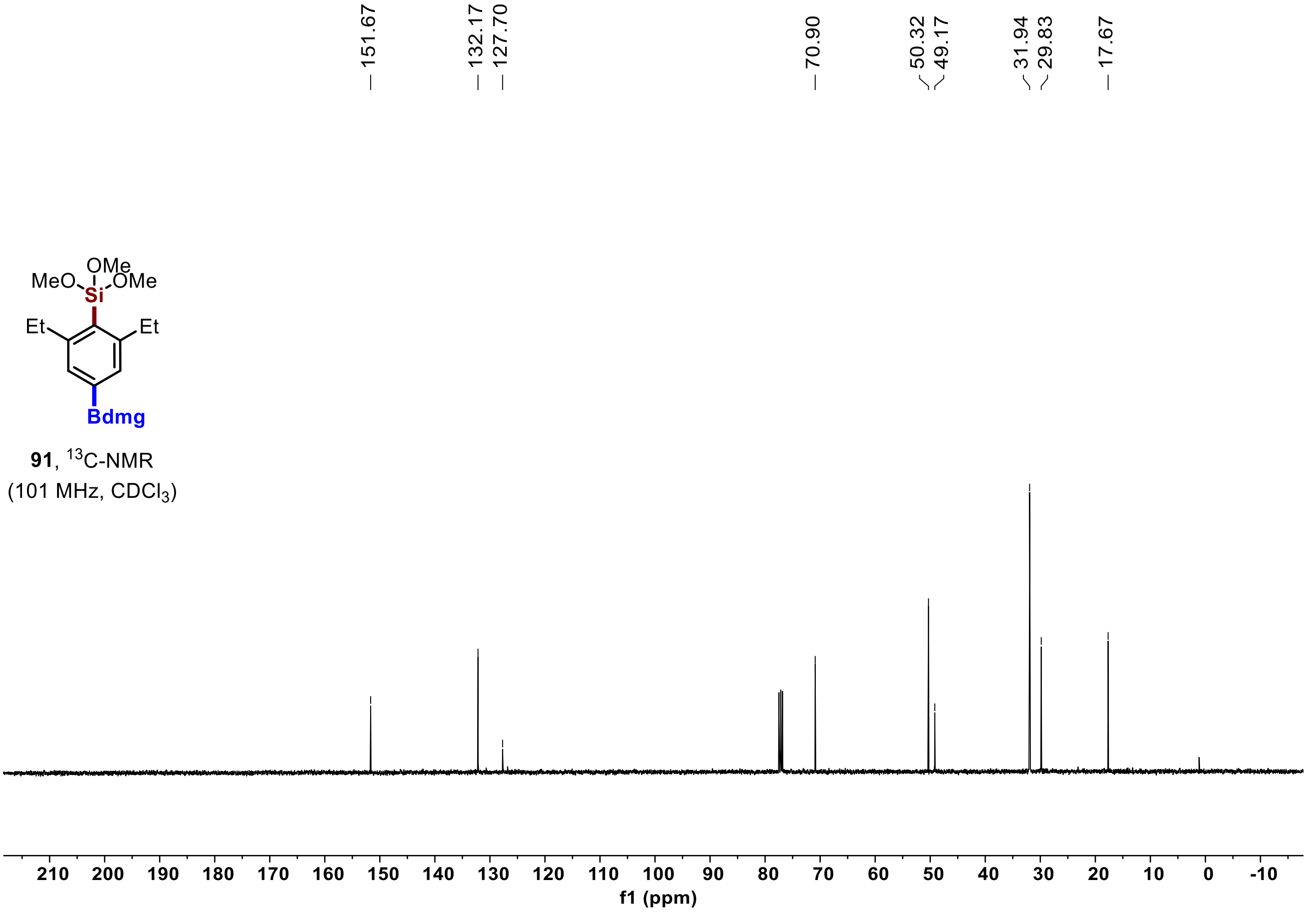

91, ^{13}C-NMR
(101 MHz, $CDCl_3$)

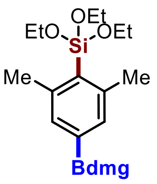

92, ^{1}H-NMR
(400 MHz, $CDCl_3$)

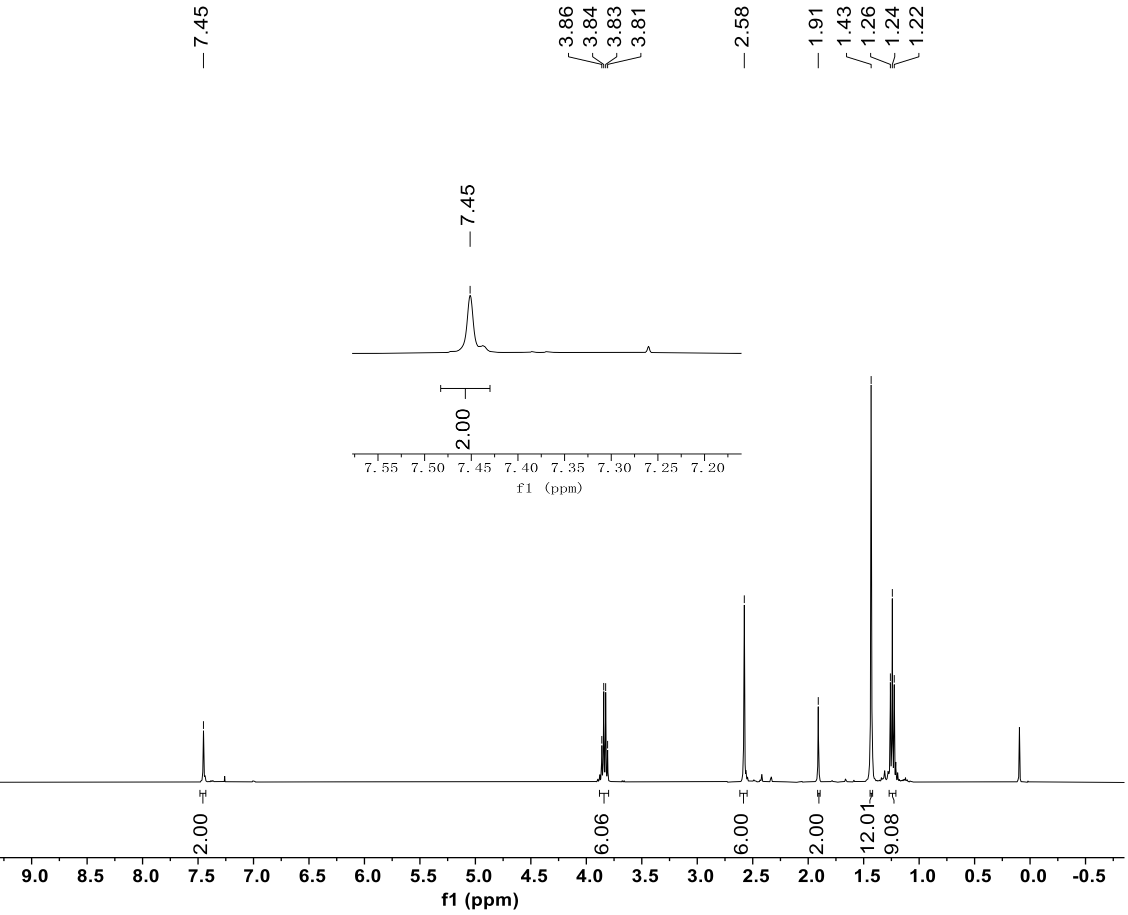

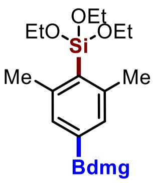

92, ^{13}C-NMR
(101 MHz, $CDCl_3$)

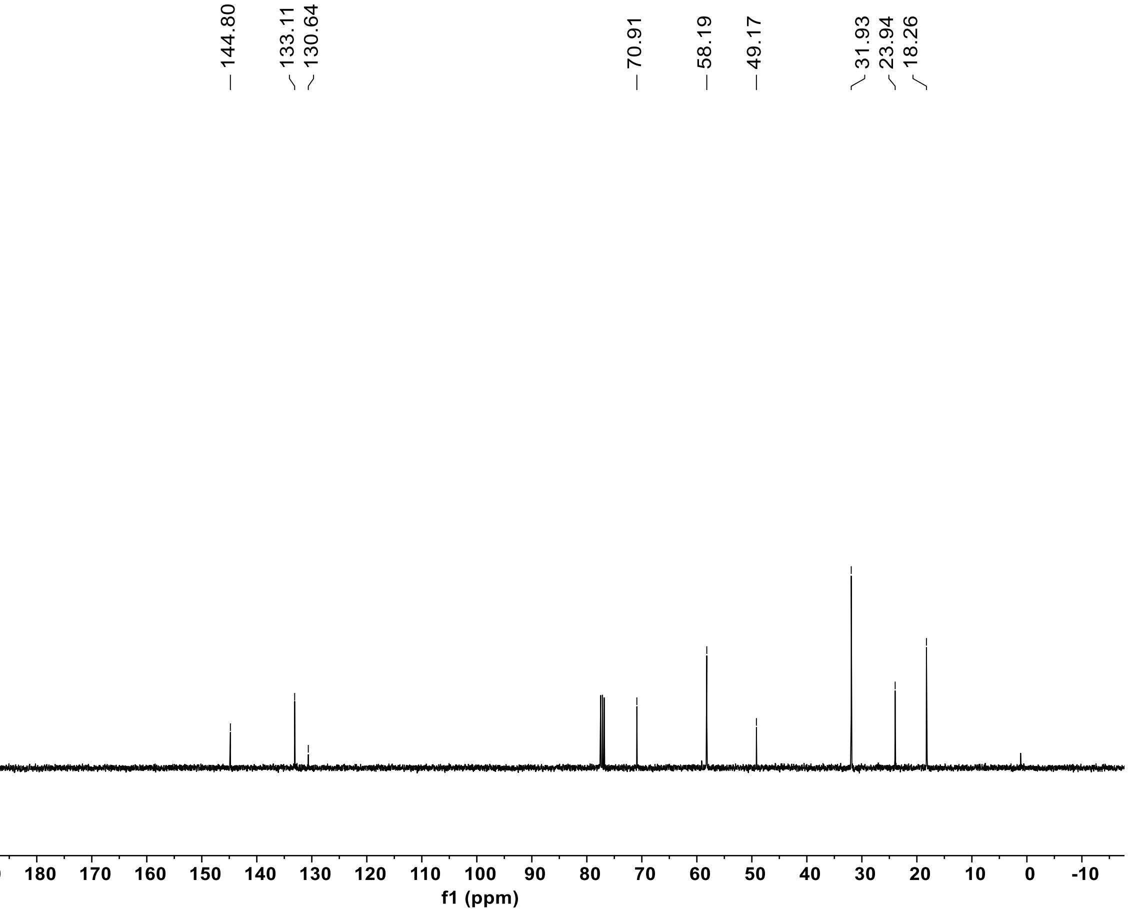

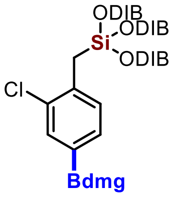

93, ^{1}H-NMR
(400 MHz, $CDCl_3$)

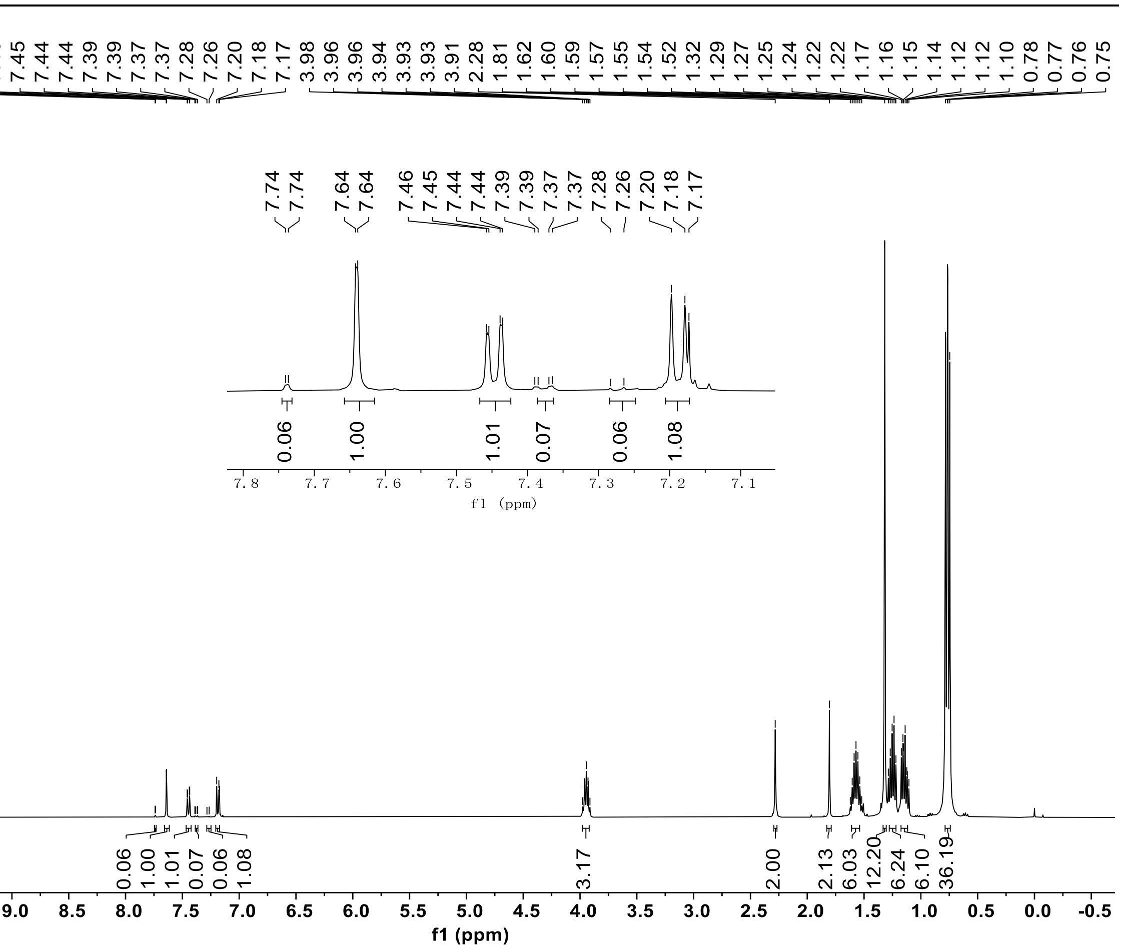

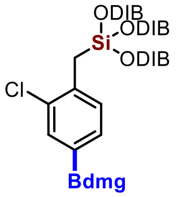

93, ^{13}C-NMR
(101 MHz, $CDCl_3$)

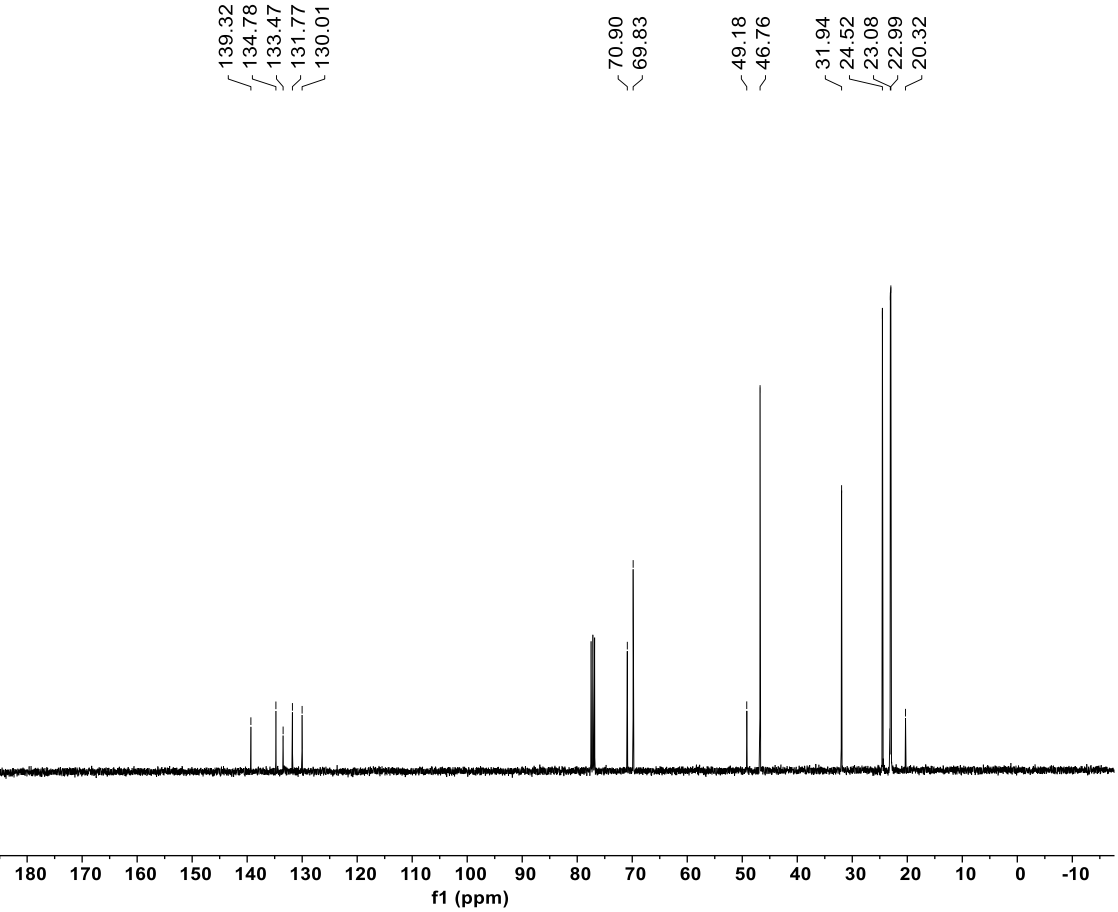

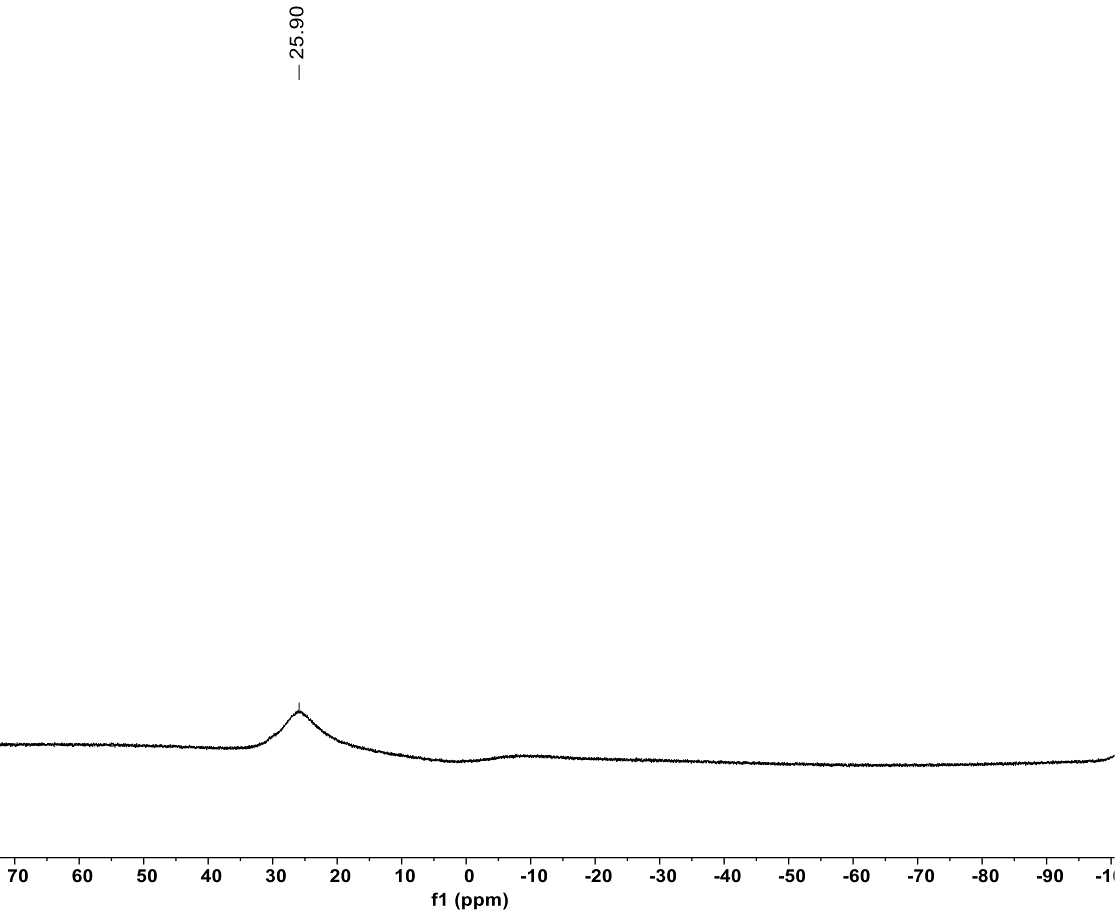

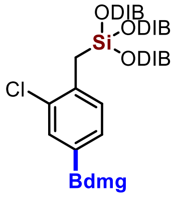

93, ^{11}B-NMR
(128 MHz, $CDCl_3$)

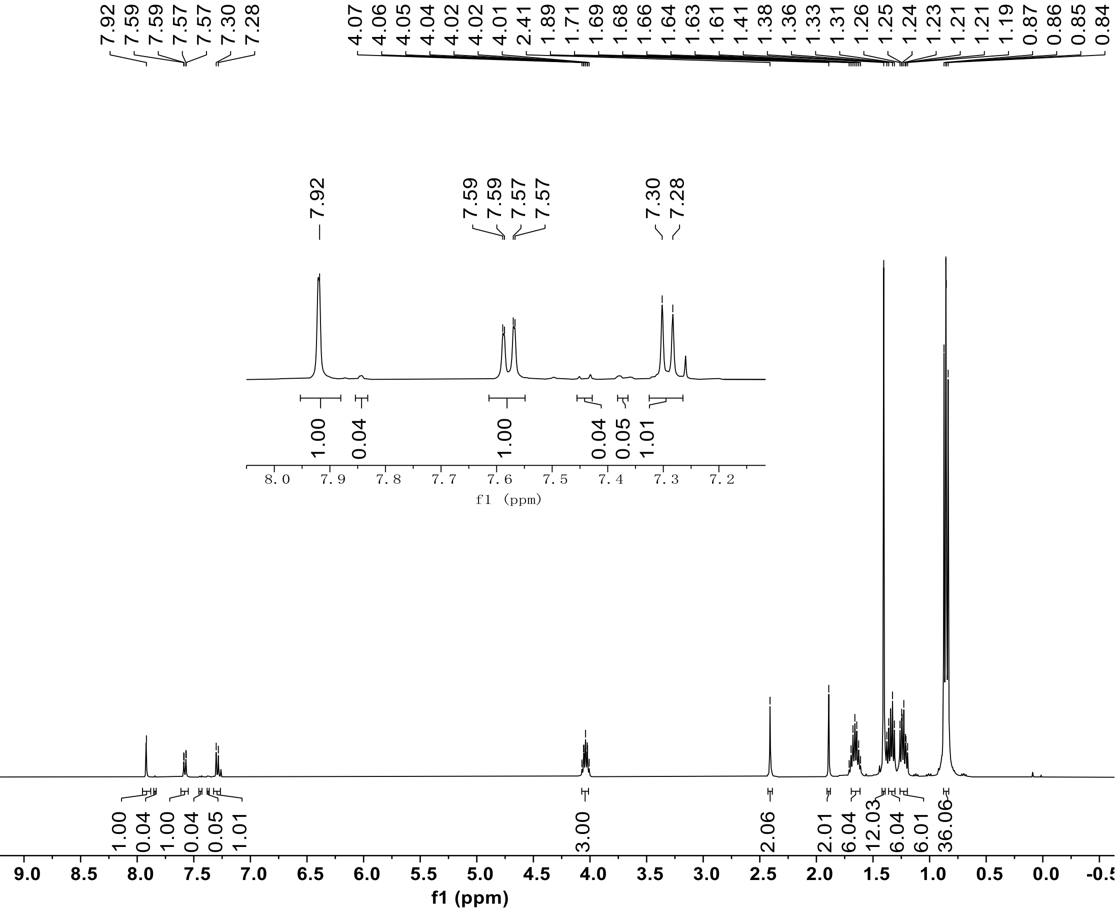

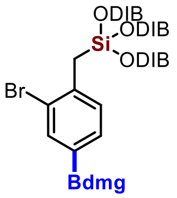

94, ^{1}H-NMR
(400 MHz, $CDCl_3$)

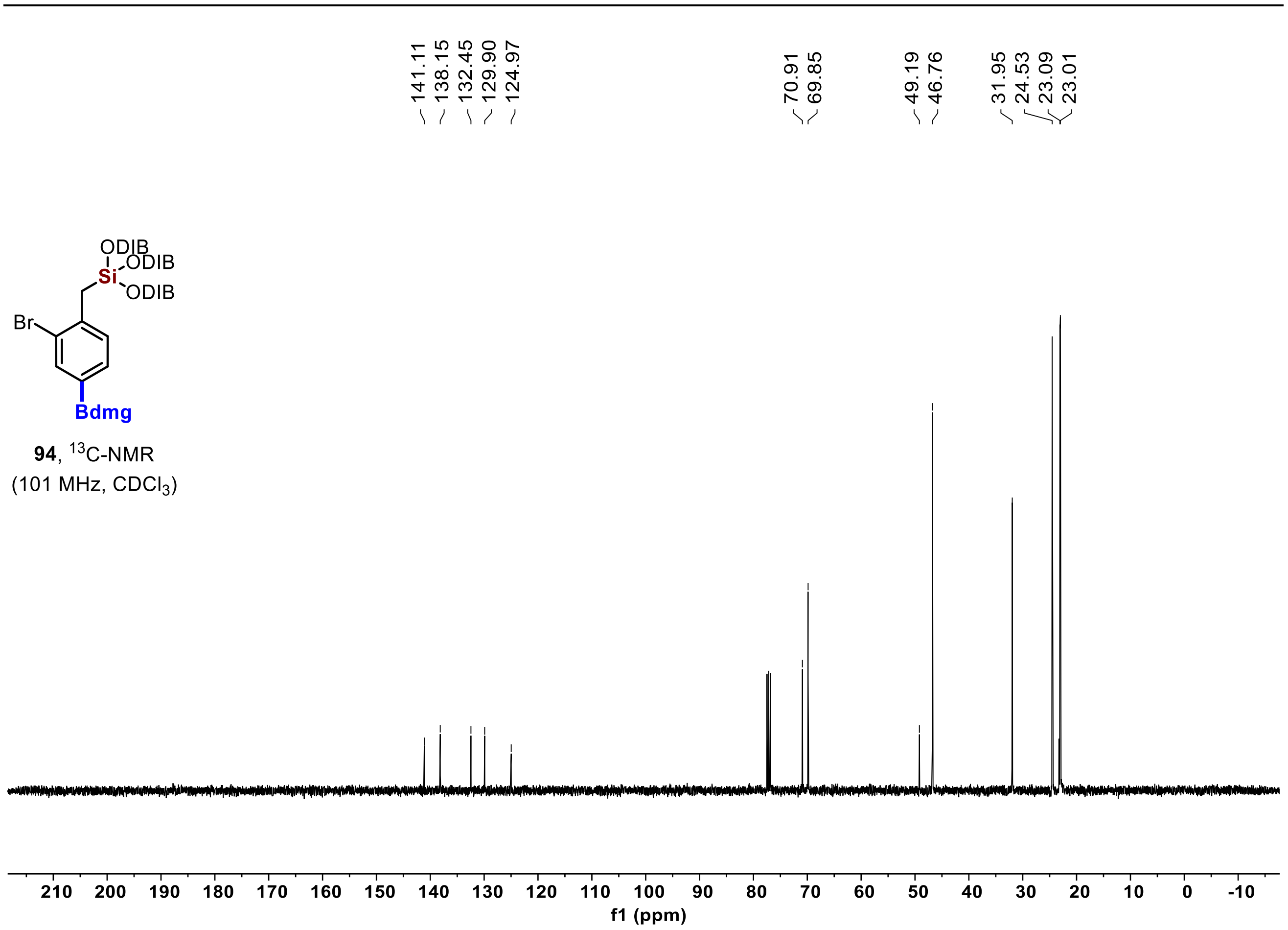
141.11
138.15
132.45
129.90
124.97
70.91
69.85
49.19
46.76
31.95
24.53
23.09
23.01
ODIB
ODIB
Si
ODIB
Br
Bdmg
94, ^{13}C-NMR
(101 MHz, $CDCl_3$)
210 200 190 180 170 160 150 140 130 120 110 100 90 80 70 60 50 40 30 20 10 0 -10
f1 (ppm)

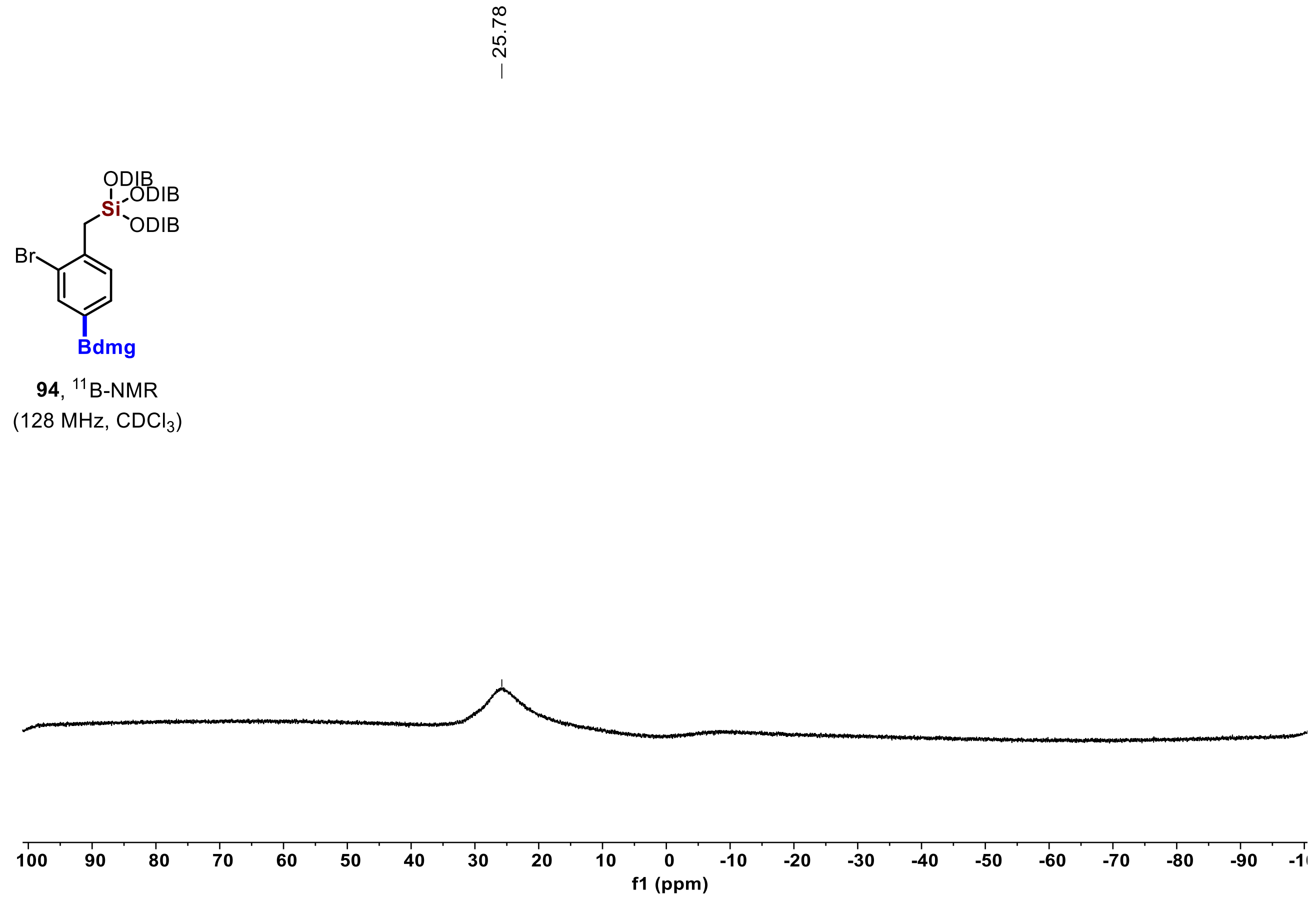
25.78
ODIB
ODIB
Si
ODIB
Br
Bdmg
94, ^{11}B-NMR
(128 MHz, $CDCl_3$)
100 90 80 70 60 50 40 30 20 10 0 -10 -20 -30 -40 -50 -60 -70 -80 -90
f1 (ppm)

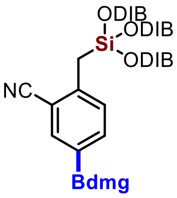

95, ^{1}H-NMR
(400 MHz, $CDCl_3$)

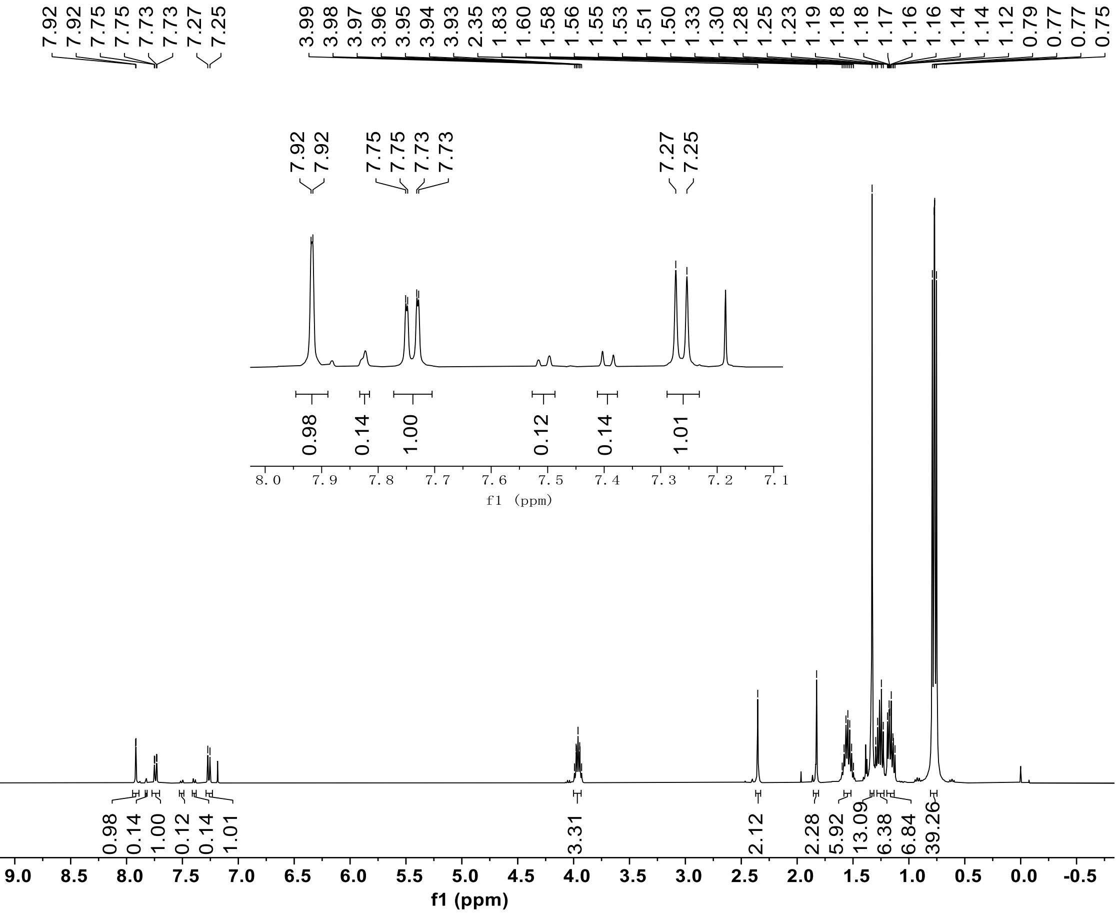

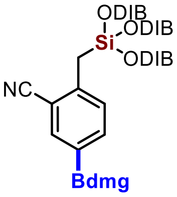

95, ^{13}C-NMR
(101 MHz, $CDCl_3$)

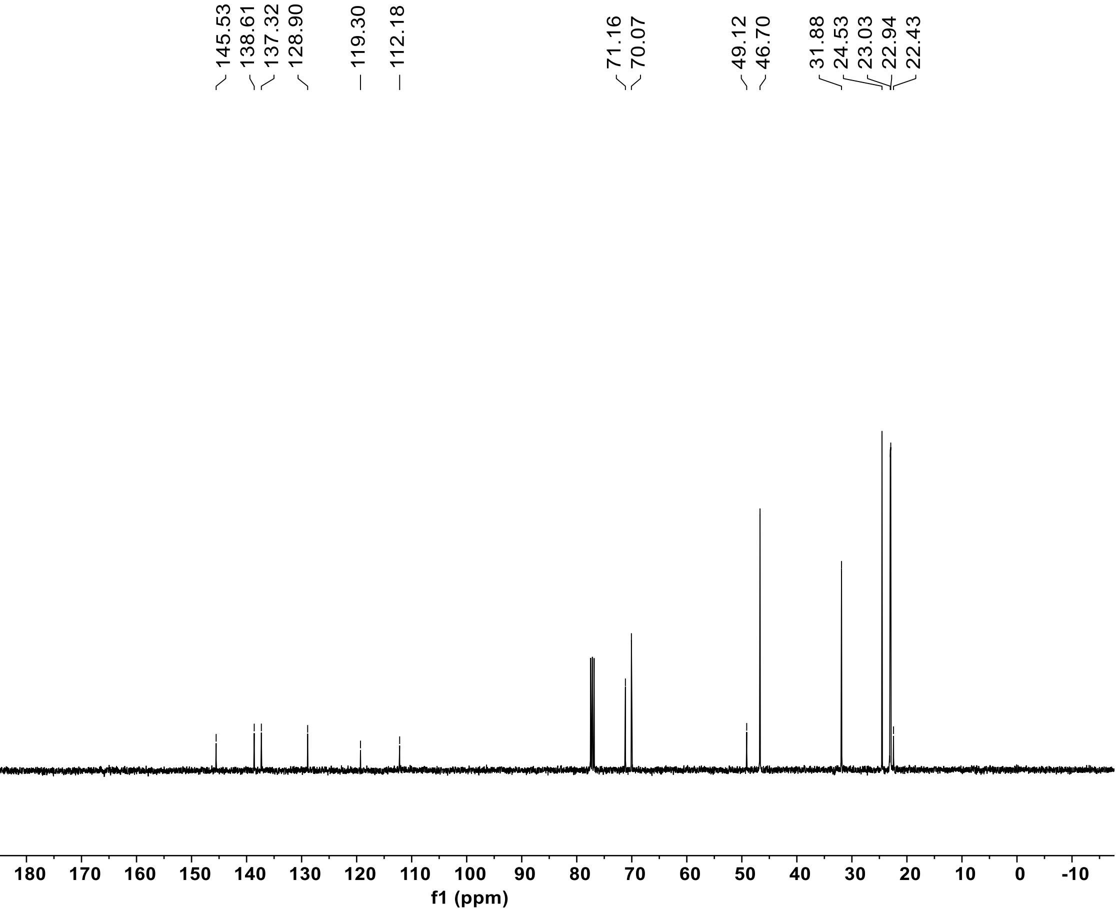

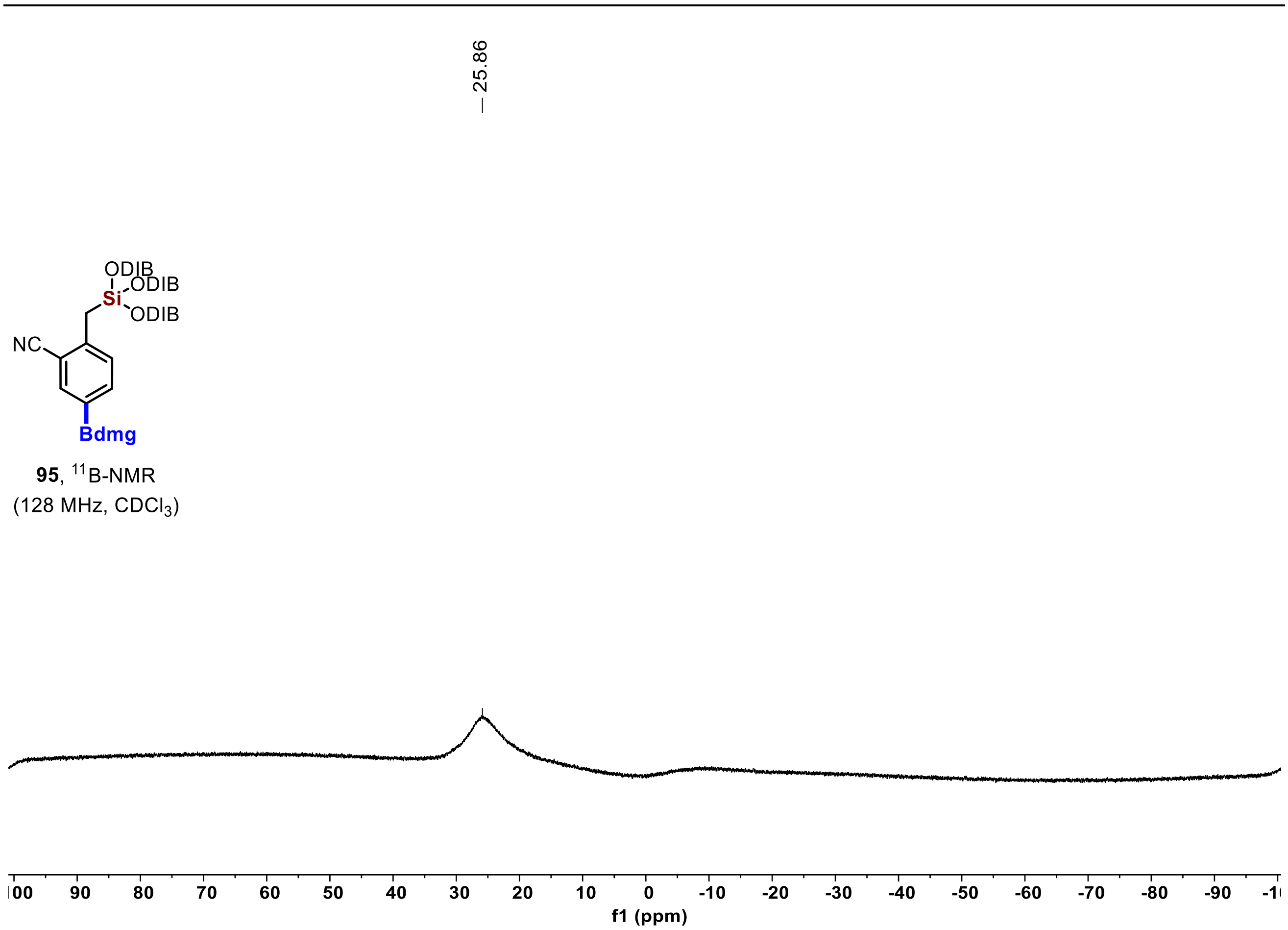

25.86
ODIB
ODIB
Si
ODIB
NC
Bdmg
95, 11B-NMR
(128 MHz, CDCl3)
f1 (ppm)

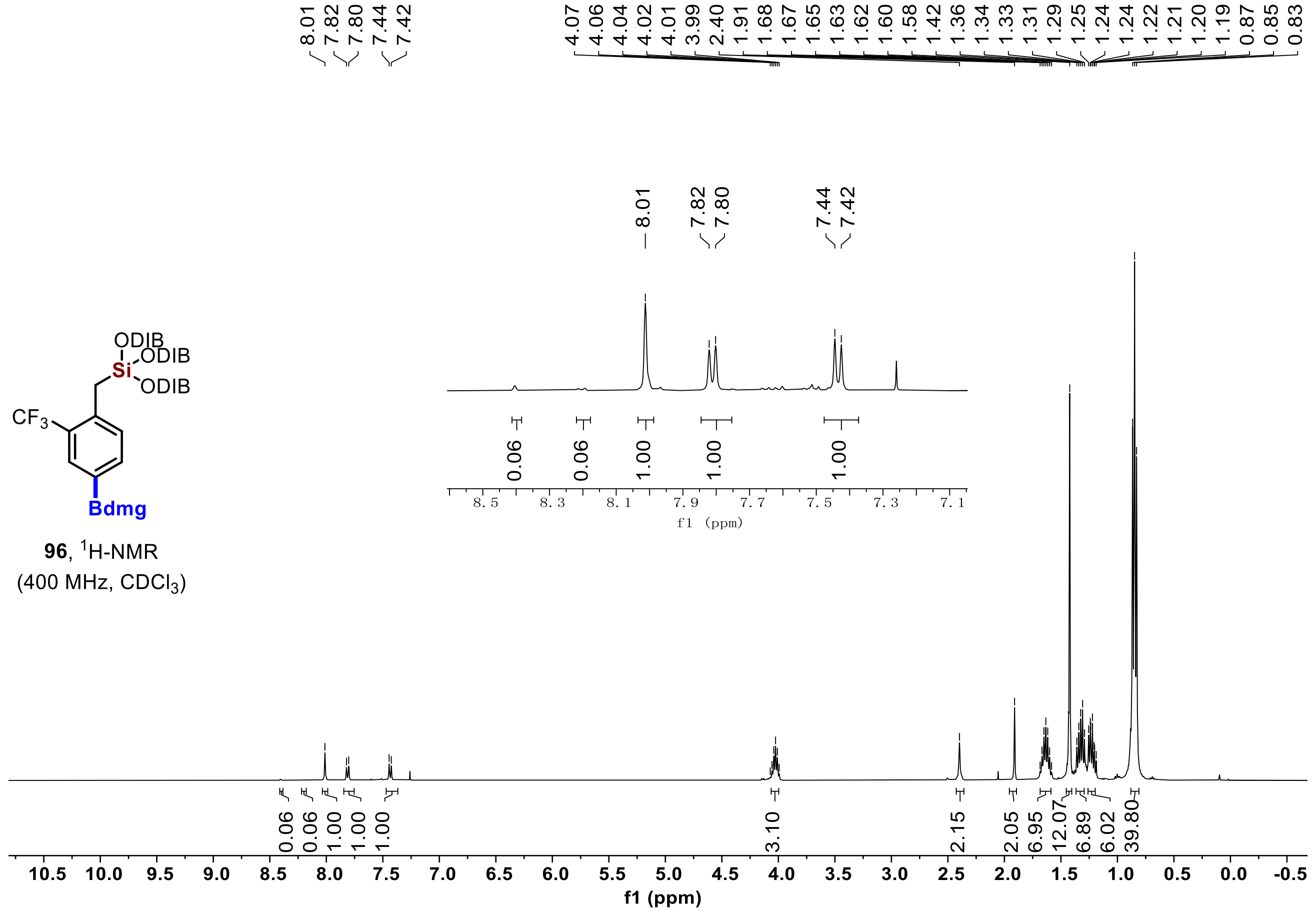

8.01
7.82
7.80
7.44
7.42
4.07
4.06
4.04
4.02
4.01
3.99
2.40
1.91
1.68
1.67
1.65
1.63
1.62
1.60
1.58
1.42
1.36
1.34
1.33
1.31
1.29
1.25
1.24
1.24
1.22
1.21
1.20
1.19
0.87
0.85
0.83
ODIB
ODIB
Si
ODIB
CF3
Bdmg
96, 1H-NMR
(400 MHz, CDCl3)
0.06
0.06
1.00
1.00
1.00
3.10
2.15
2.05
6.95
12.07
6.89
6.02
39.80
f1 (ppm)

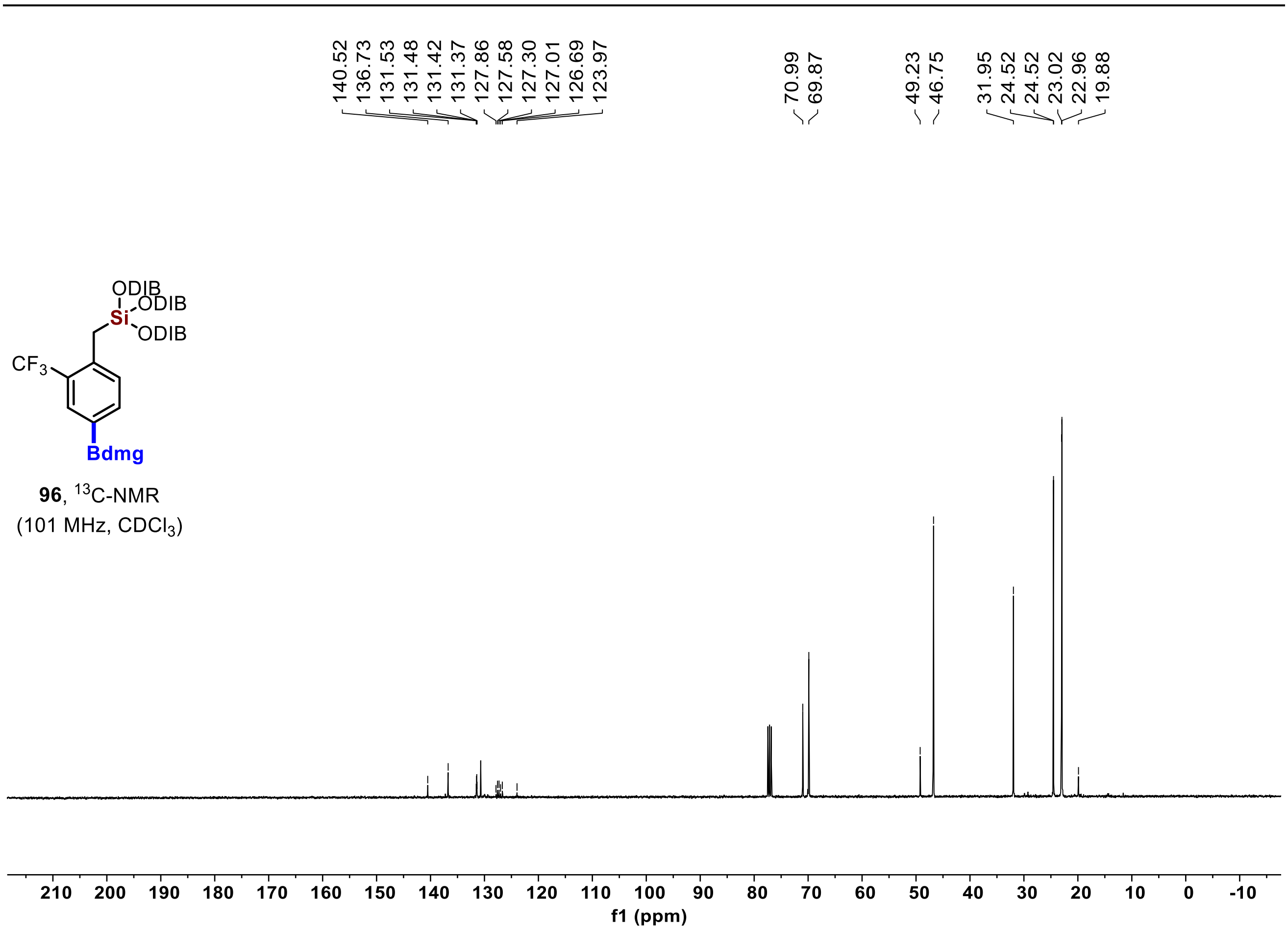
140.52
136.73
131.53
131.48
131.42
131.37
127.86
127.58
127.30
127.01
126.69
123.97
70.99
69.87
49.23
46.75
31.95
24.52
24.52
23.02
22.96
19.88
ODIB
ODIB
Si
ODIB
CF3
Bdmg
96, 13C-NMR
(101 MHz, CDCl3)
210 200 190 180 170 160 150 140 130 120 110 100 90 80 70 60 50 40 30 20 10 0 -10
f1 (ppm)

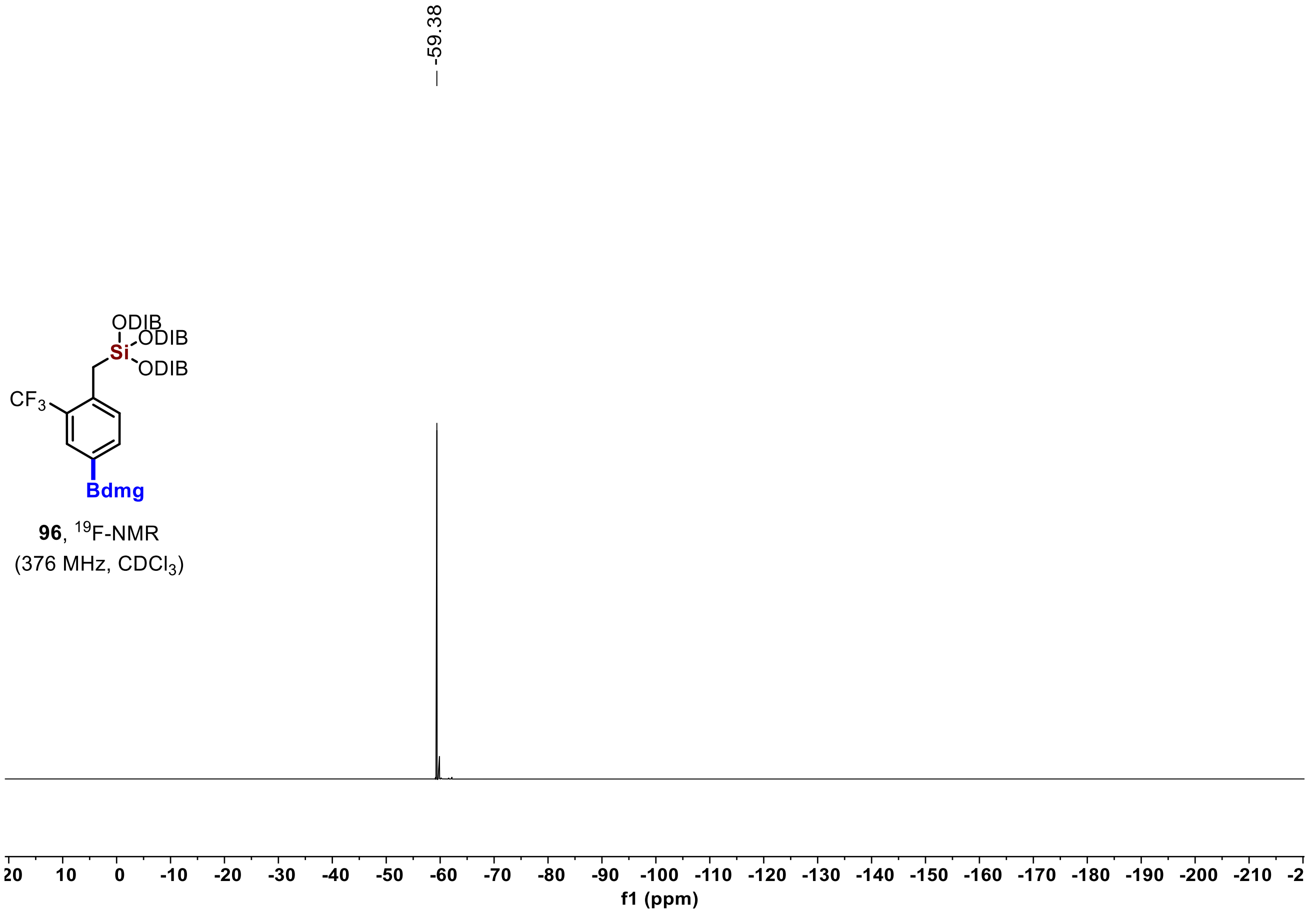
-59.38
ODIB
ODIB
Si
ODIB
CF3
Bdmg
96, 19F-NMR
(376 MHz, CDCl3)
20 10 0 -10 -20 -30 -40 -50 -60 -70 -80 -90 -100 -110 -120 -130 -140 -150 -160 -170 -180 -190 -200 -210
f1 (ppm)

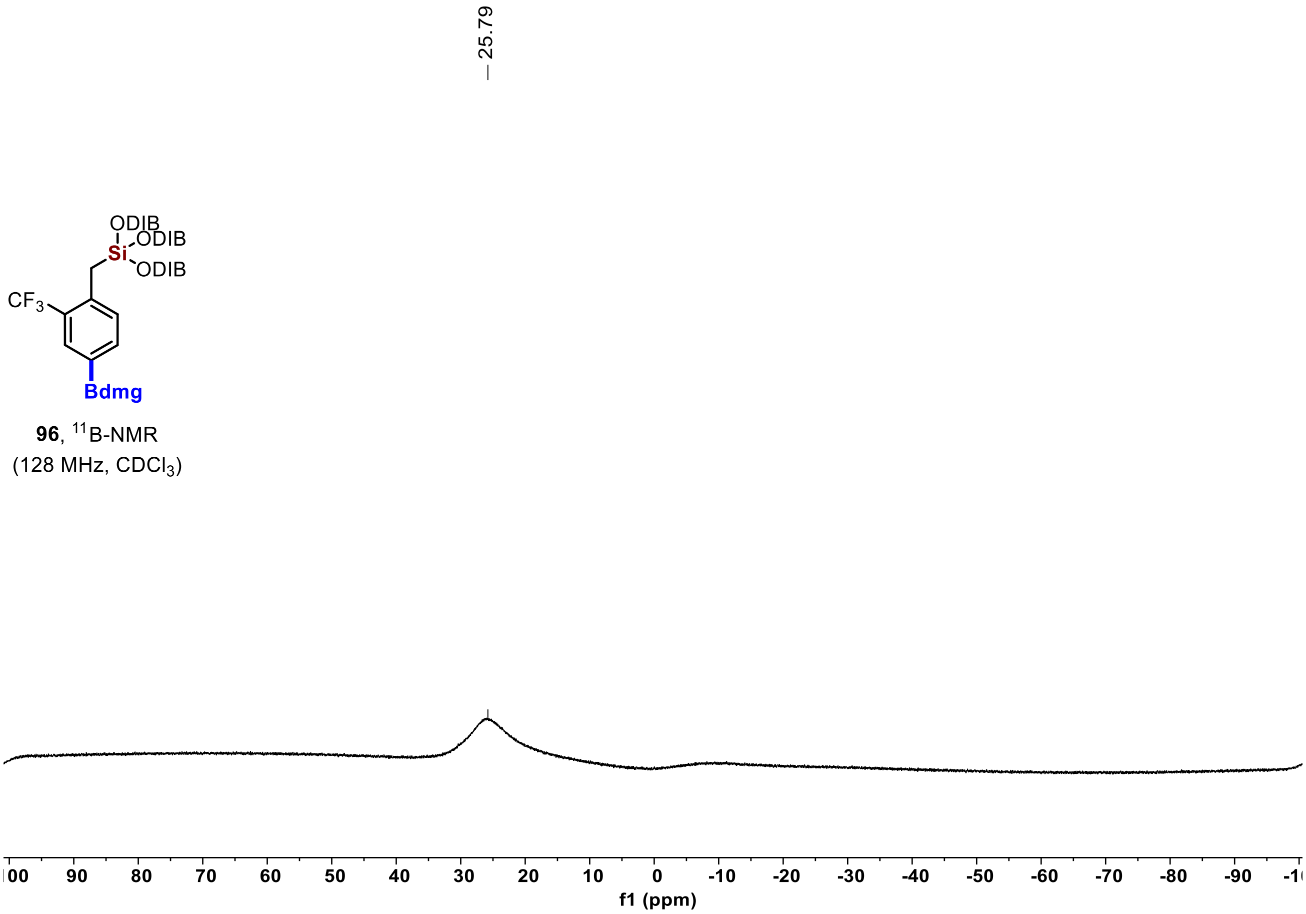

96, ^{11}B-NMR
(128 MHz, $CDCl_3$)

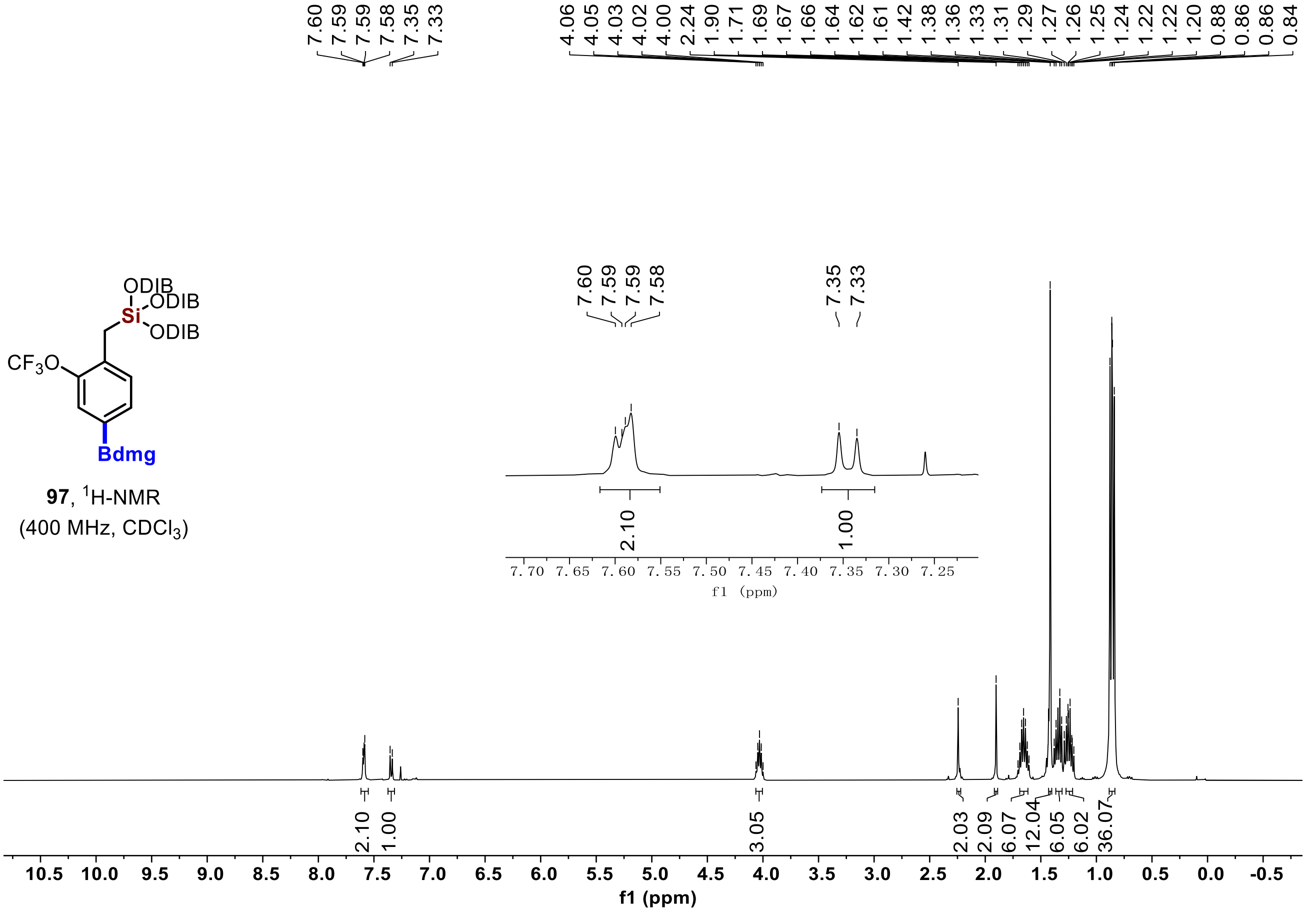

97, ^{1}H-NMR
(400 MHz, $CDCl_3$)

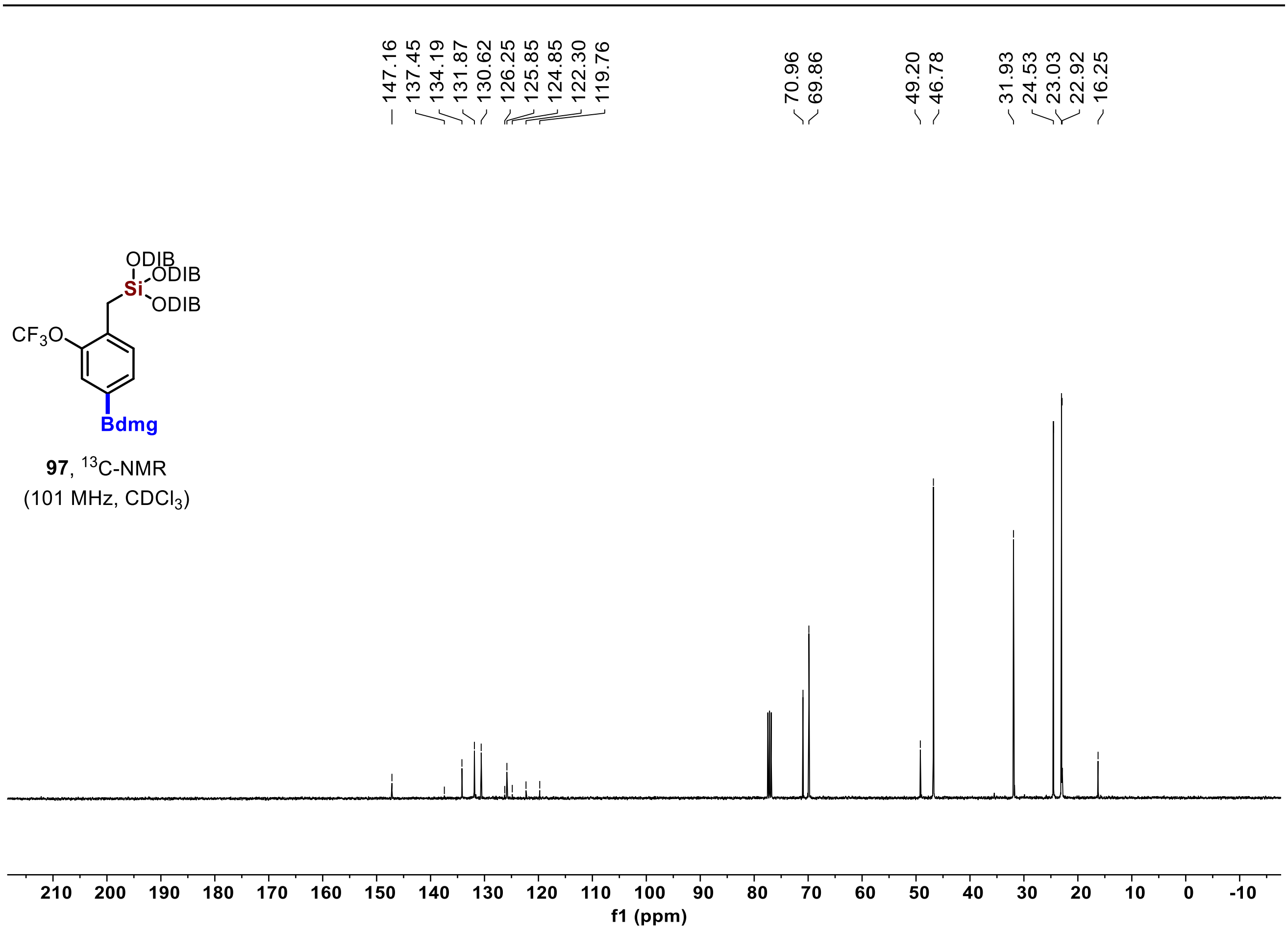
147.16
137.45
134.19
131.87
130.62
126.25
125.85
124.85
122.30
119.76
70.96
69.86
49.20
46.78
31.93
24.53
23.03
22.92
16.25
ODIB
ODIB
Si
ODIB
CF3O
Bdmg
97, 13C-NMR
(101 MHz, CDCl3)
210 200 190 180 170 160 150 140 130 120 110 100 90 80 70 60 50 40 30 20 10 0 -10
f1 (ppm)

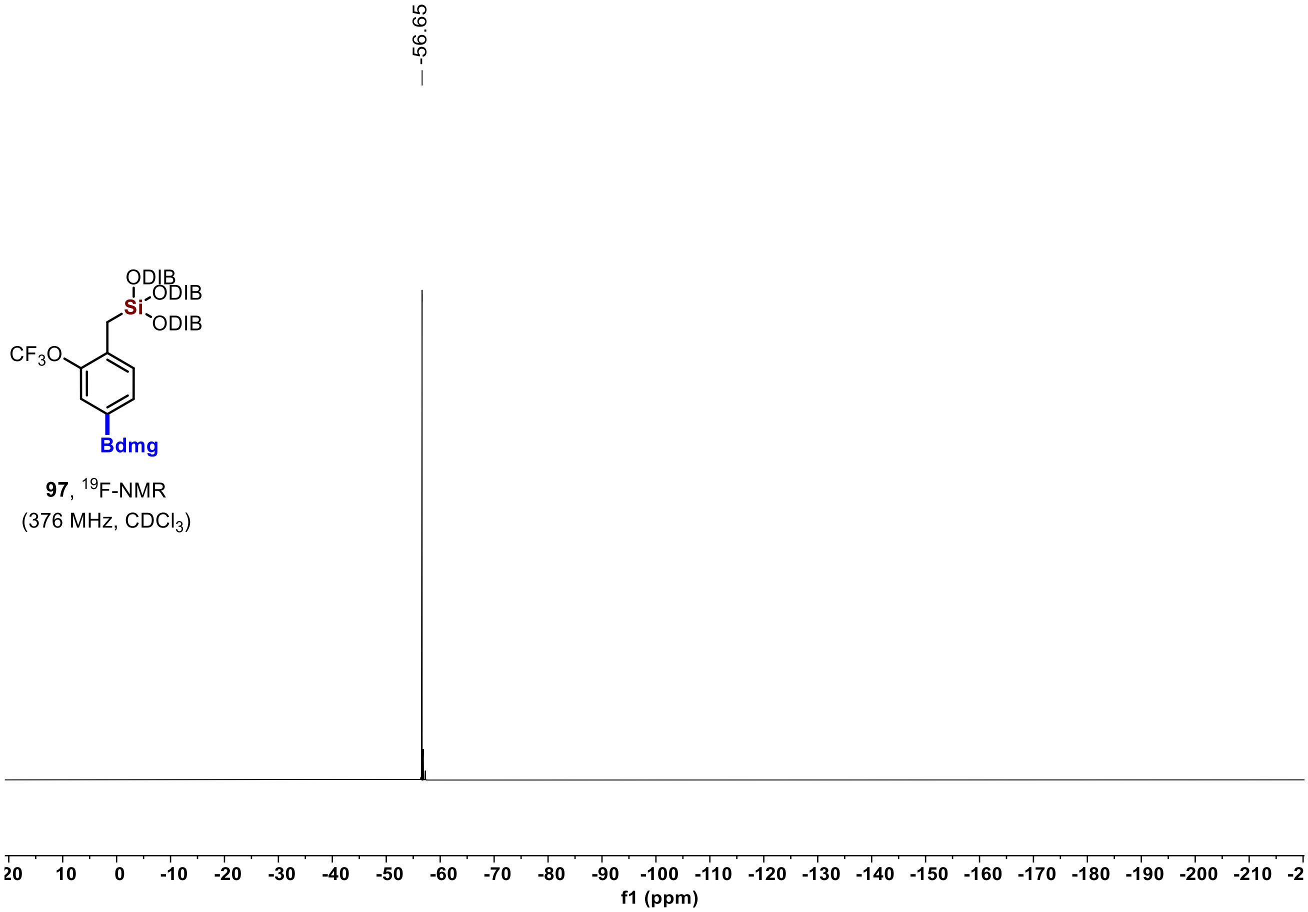
-56.65
ODIB
ODIB
Si
ODIB
CF3O
Bdmg
97, 19F-NMR
(376 MHz, CDCl3)
20 10 0 -10 -20 -30 -40 -50 -60 -70 -80 -90 -100 -110 -120 -130 -140 -150 -160 -170 -180 -190 -200 -210 -2
f1 (ppm)

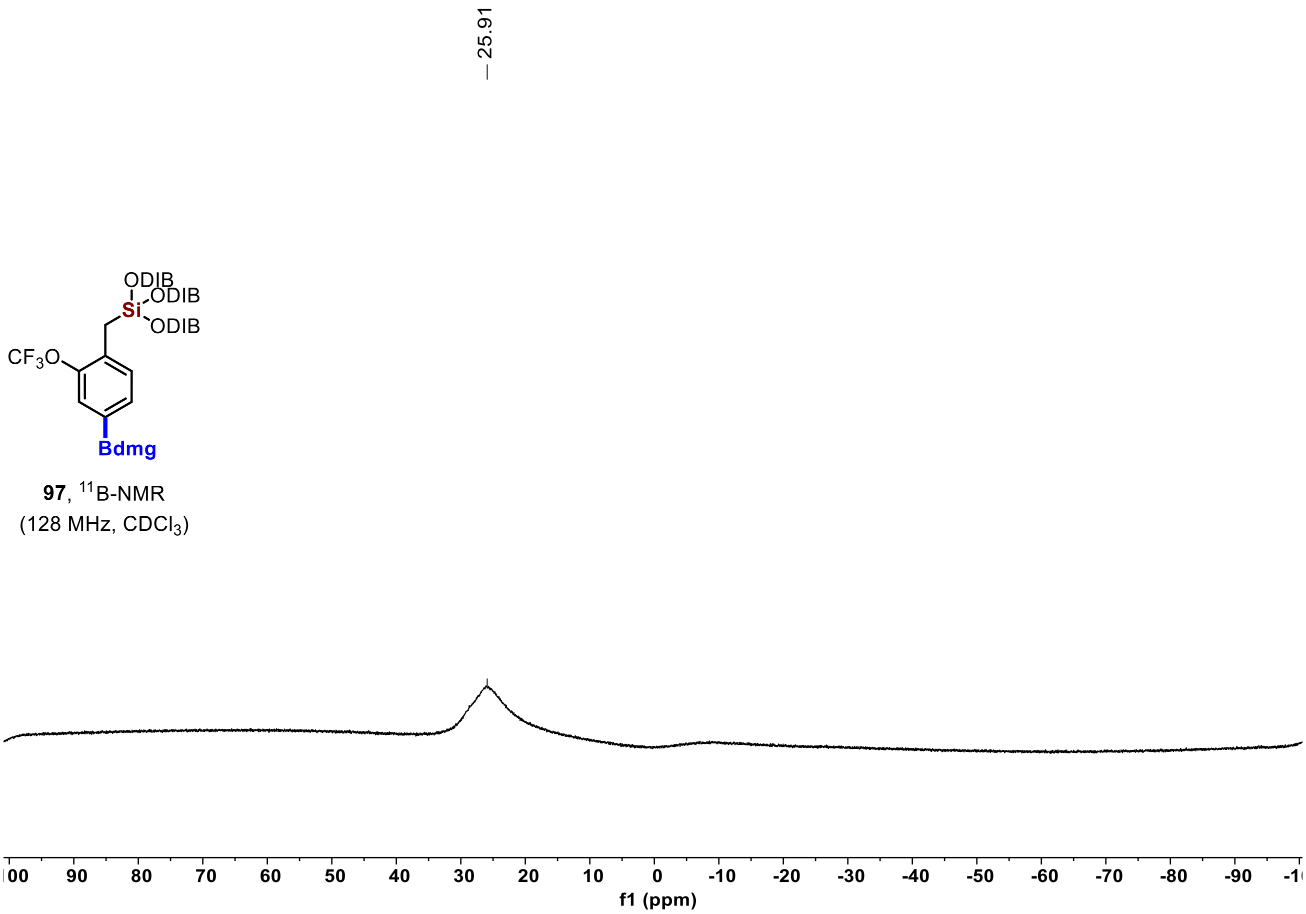

97, ^{11}B-NMR
(128 MHz, $CDCl_3$)

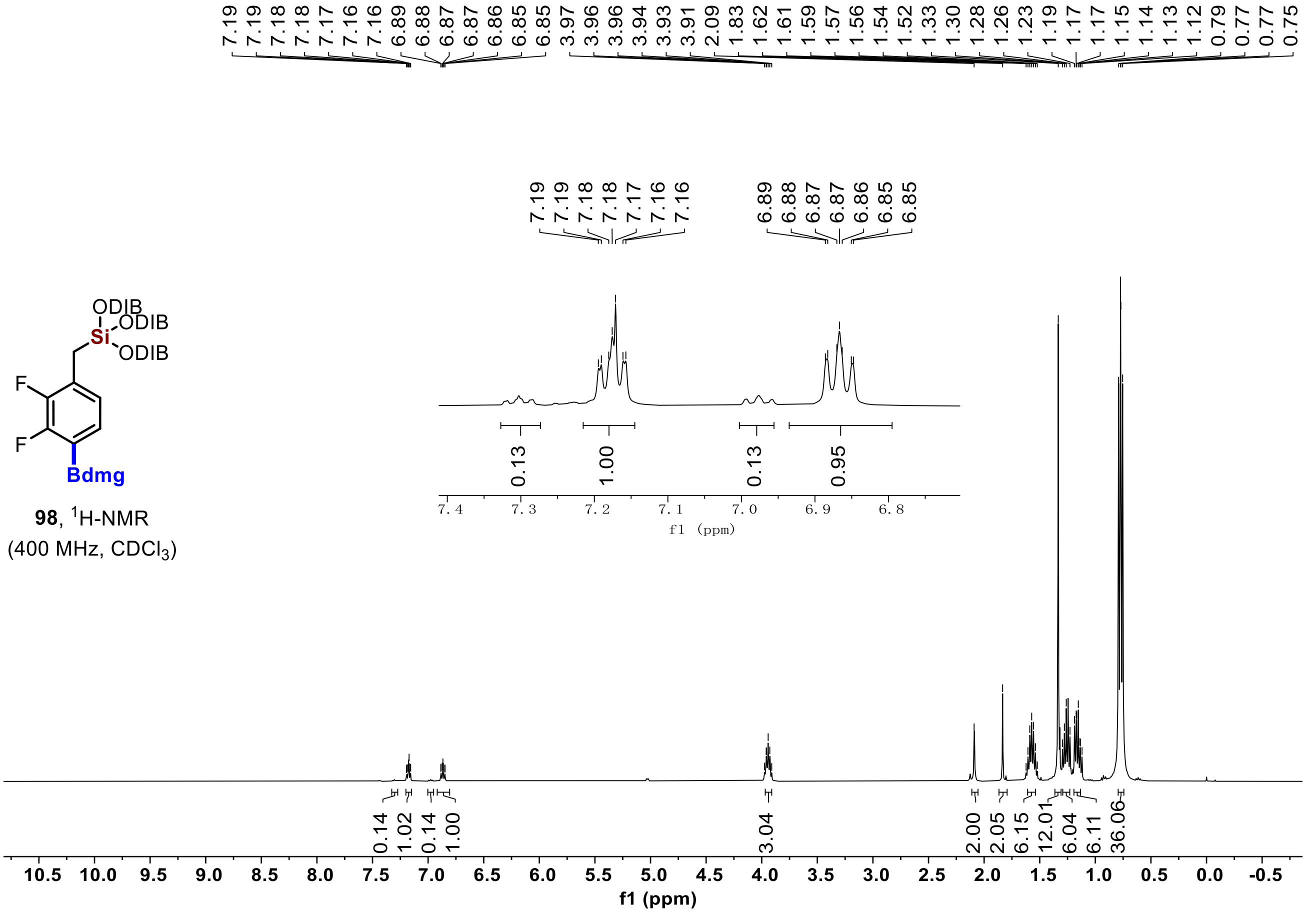

98, ^{1}H-NMR
(400 MHz, $CDCl_3$)

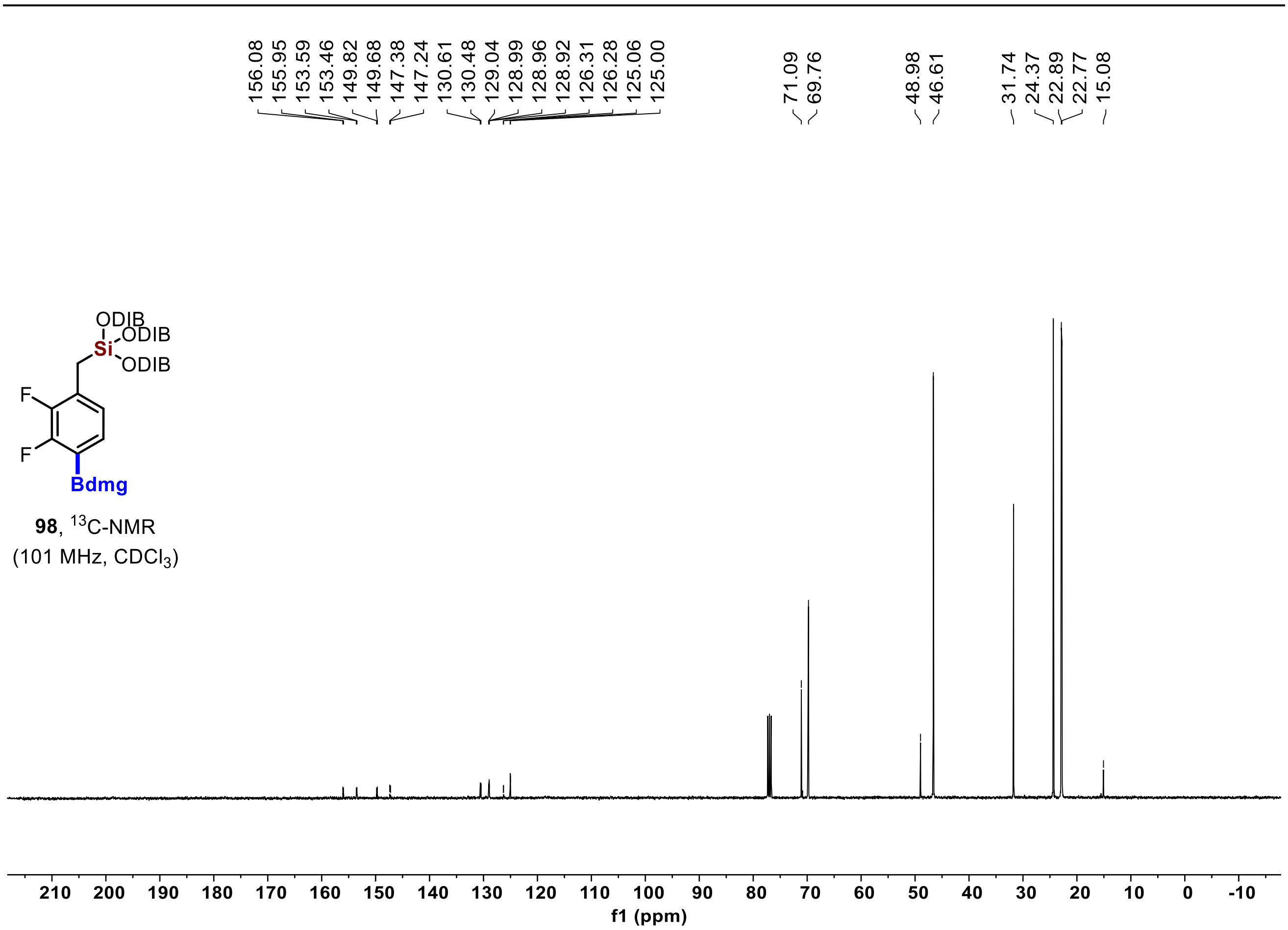

98, ^{13}C-NMR
(101 MHz, $CDCl_3$)

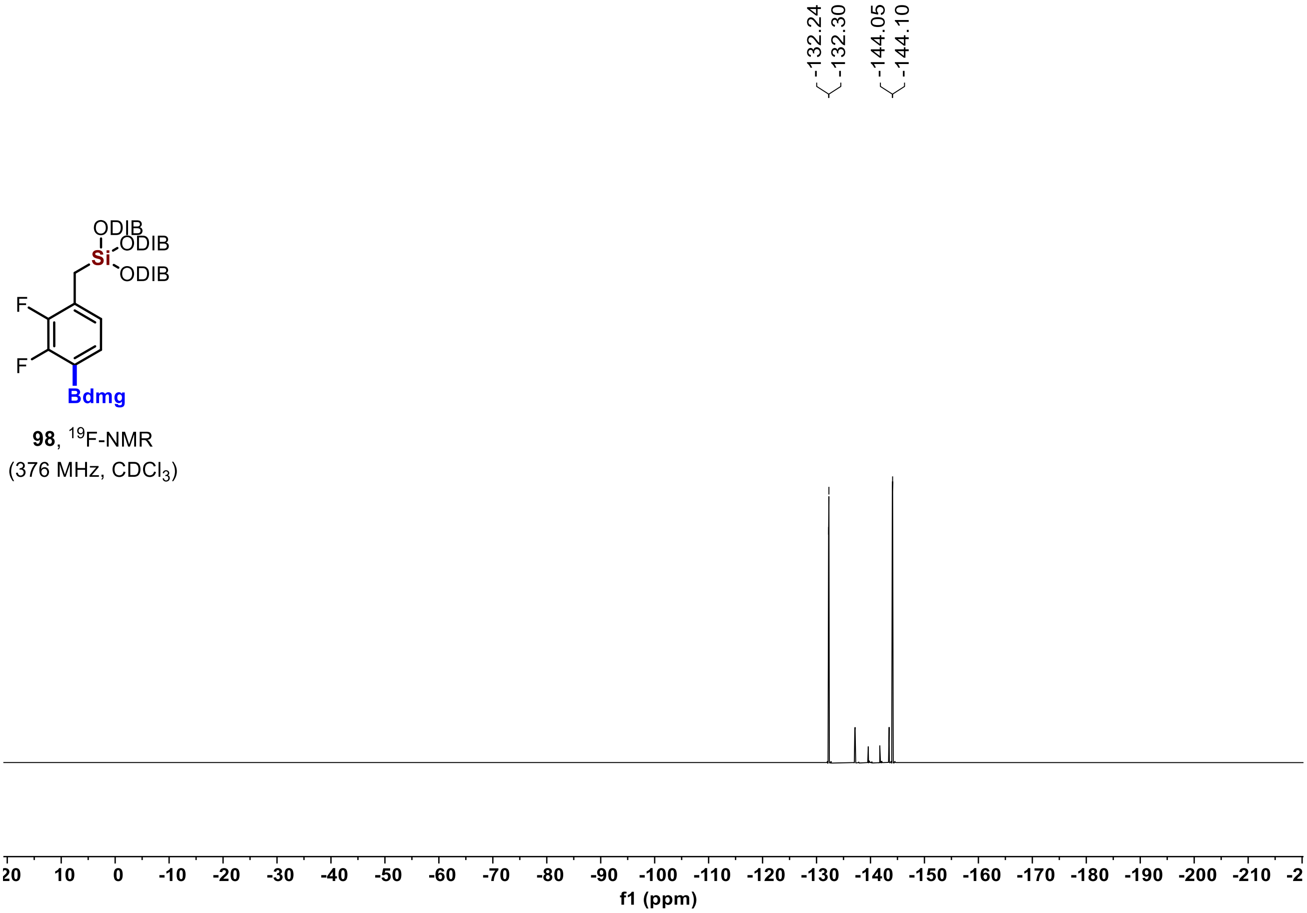

98, ^{19}F-NMR
(376 MHz, $CDCl_3$)

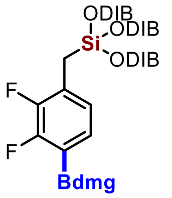

98, ^{11}B-NMR
(128 MHz, $CDCl_3$)

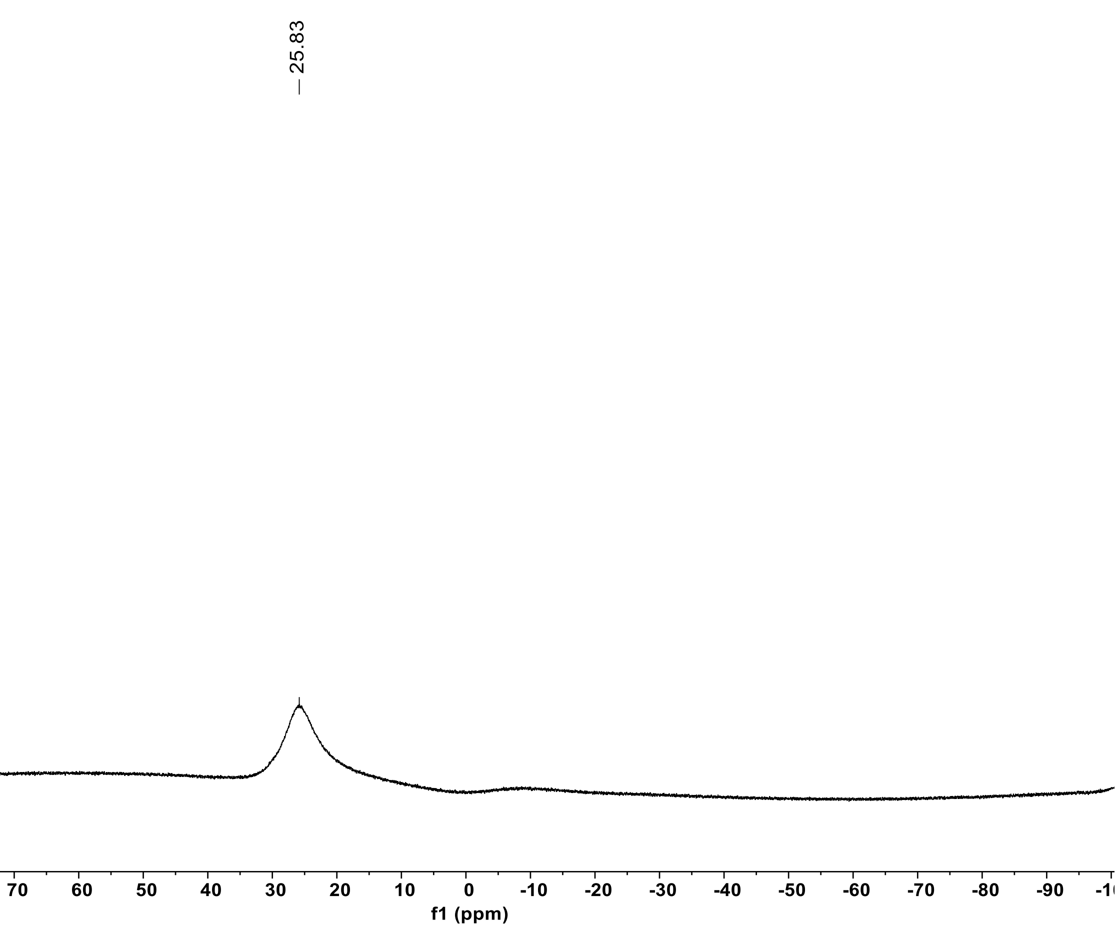

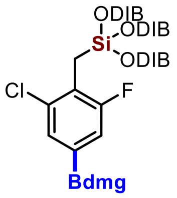

99, ^{1}H-NMR
(400 MHz, $CDCl_3$)

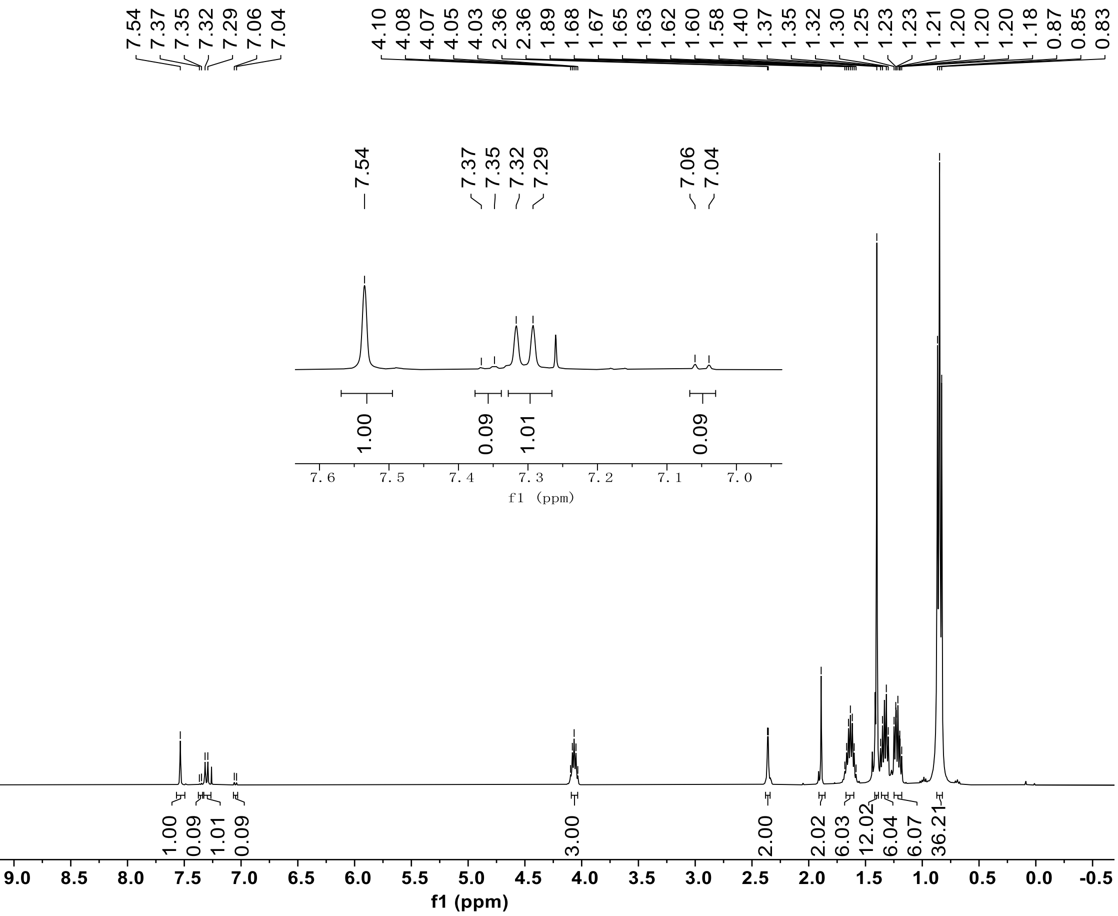

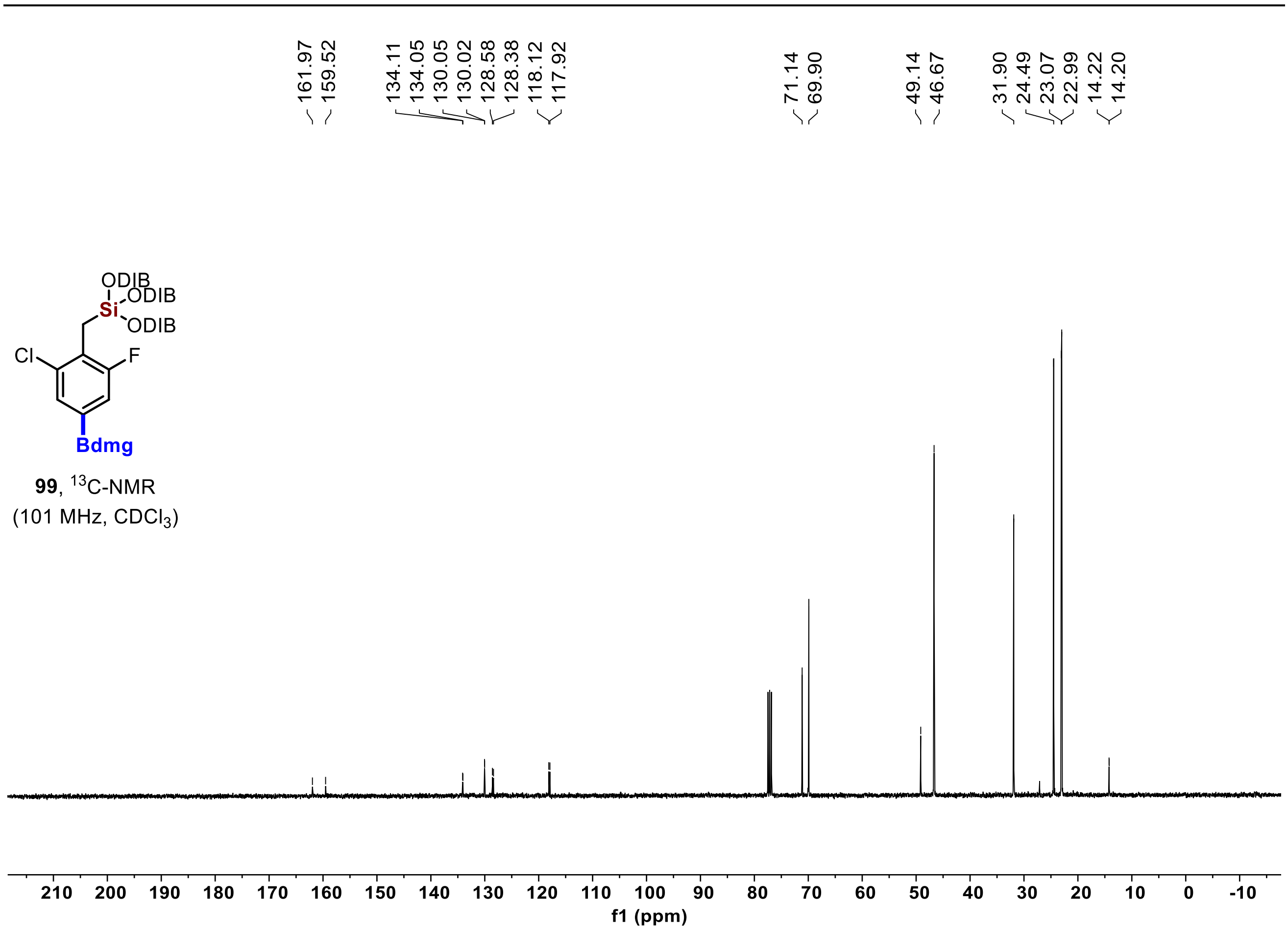
161.97
159.52
134.11
134.05
130.05
130.02
128.58
128.38
118.12
117.92
71.14
69.90
49.14
46.67
31.90
24.49
23.07
22.99
14.22
14.20
ODIB
ODIB
Si
ODIB
Cl
F
Bdmg
99, 13C-NMR
(101 MHz, CDCl3)
f1 (ppm)

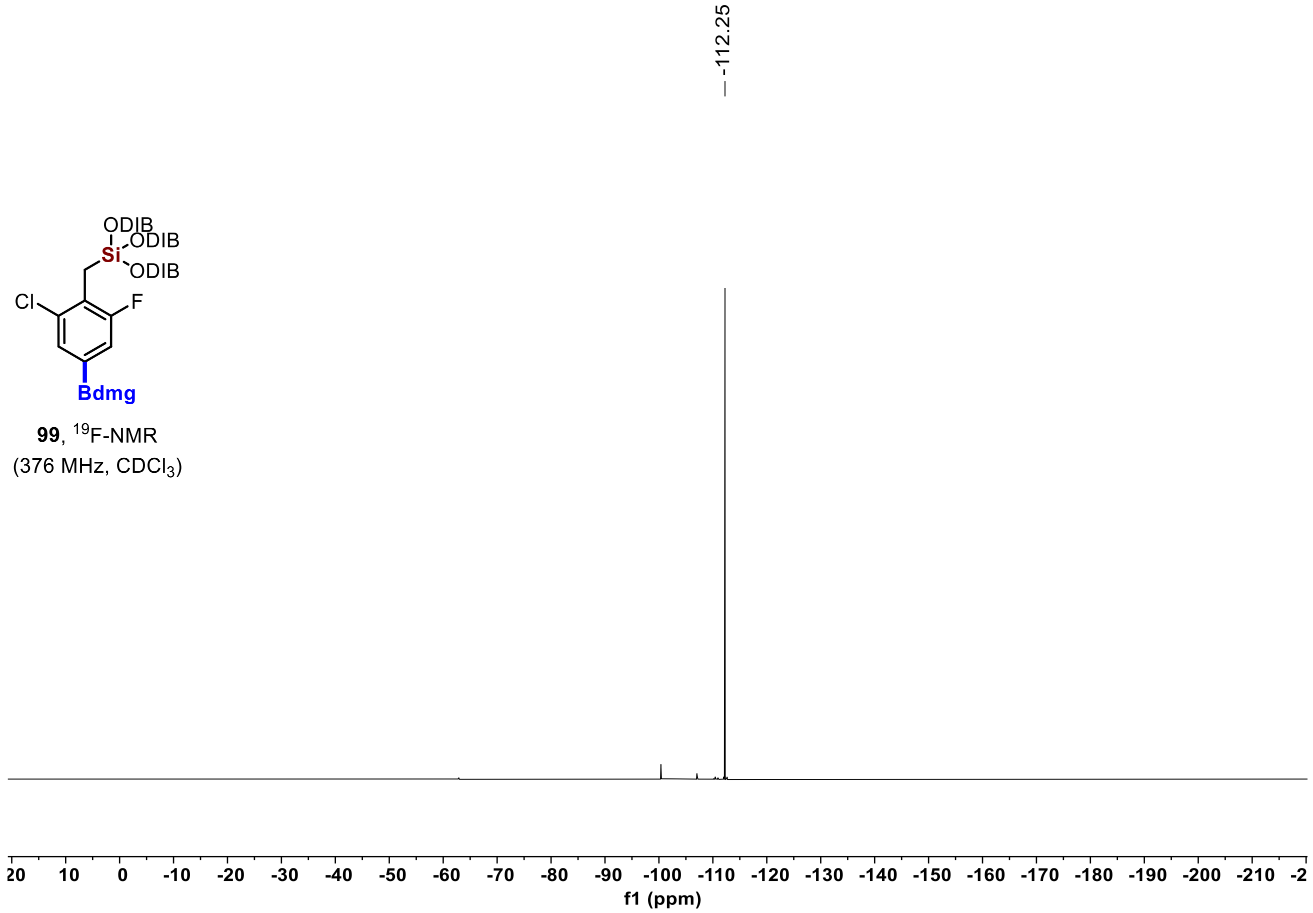
-112.25
ODIB
ODIB
Si
ODIB
Cl
F
Bdmg
99, 19F-NMR
(376 MHz, CDCl3)
f1 (ppm)

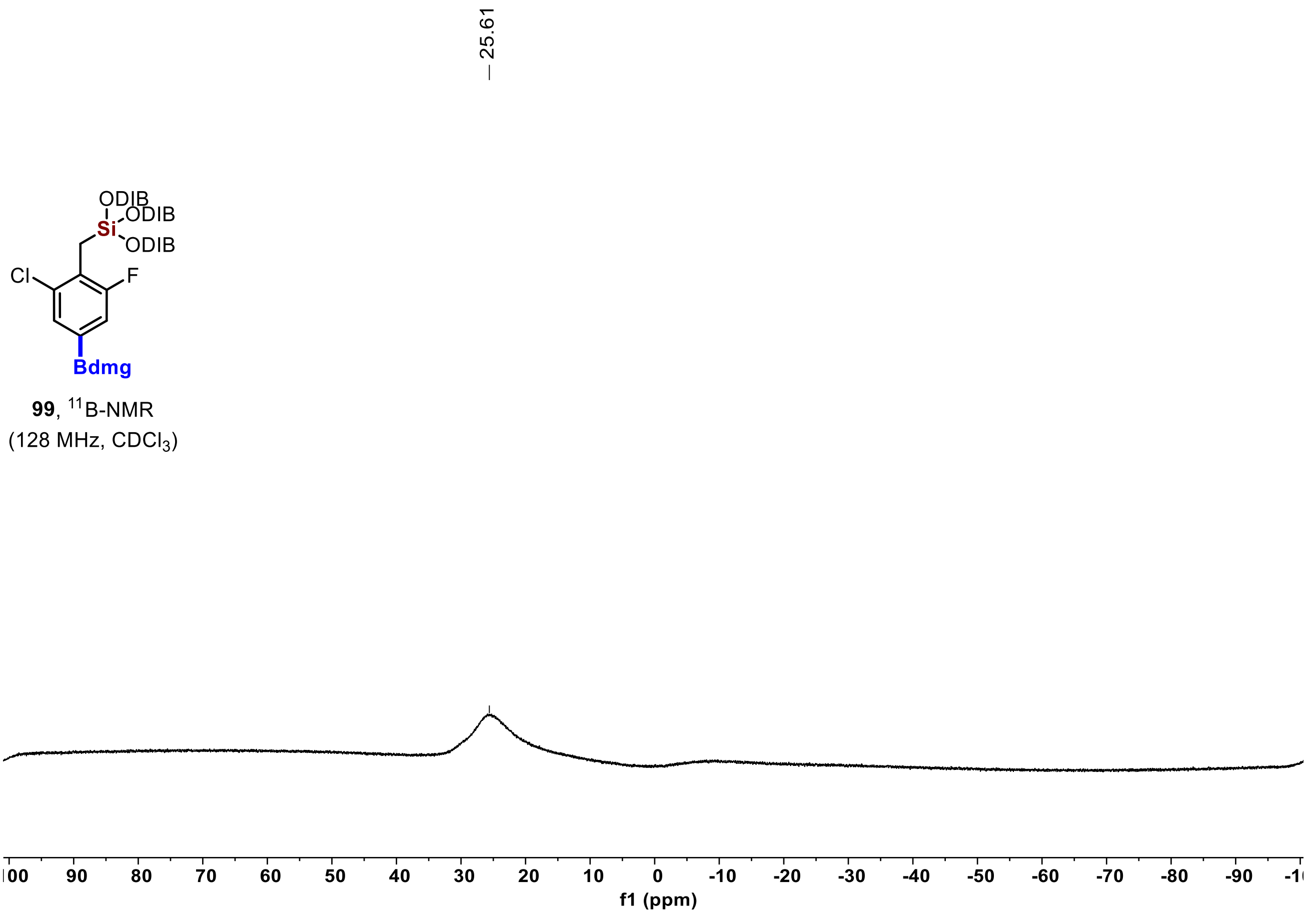

99, ^{11}B-NMR
(128 MHz, $CDCl_3$)

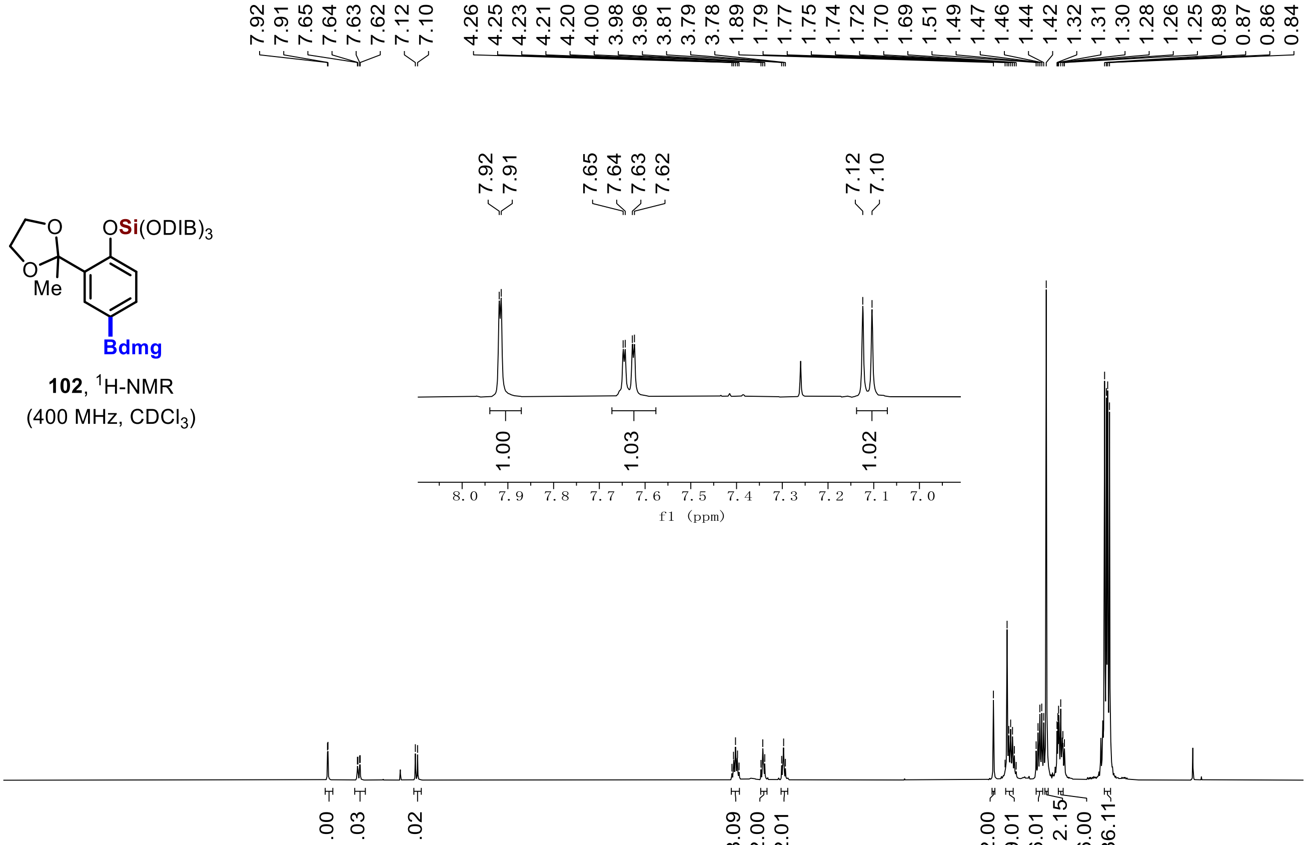

102, ^{1}H-NMR
(400 MHz, $CDCl_3$)

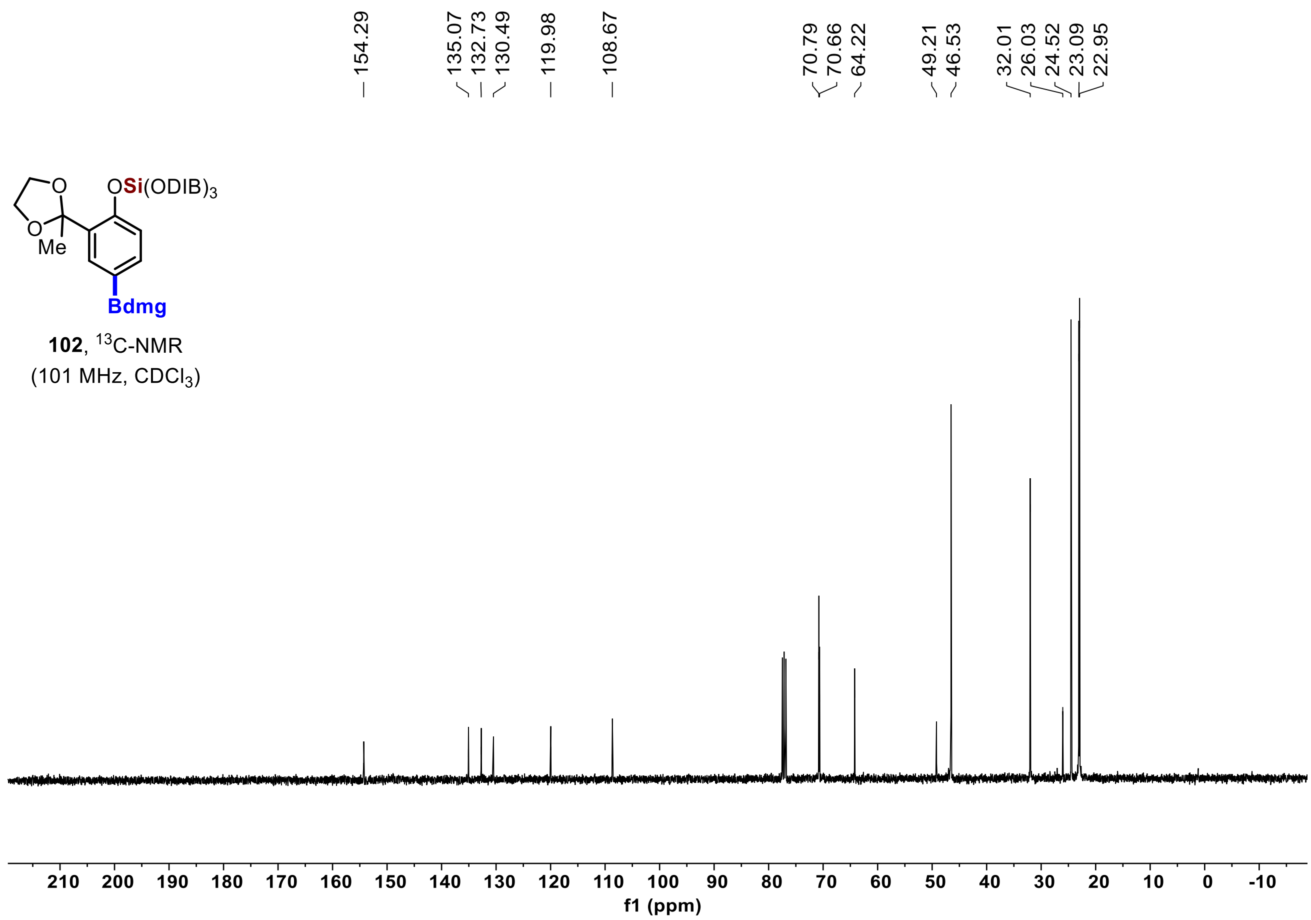

102, ^{13}C-NMR
(101 MHz, $CDCl_3$)

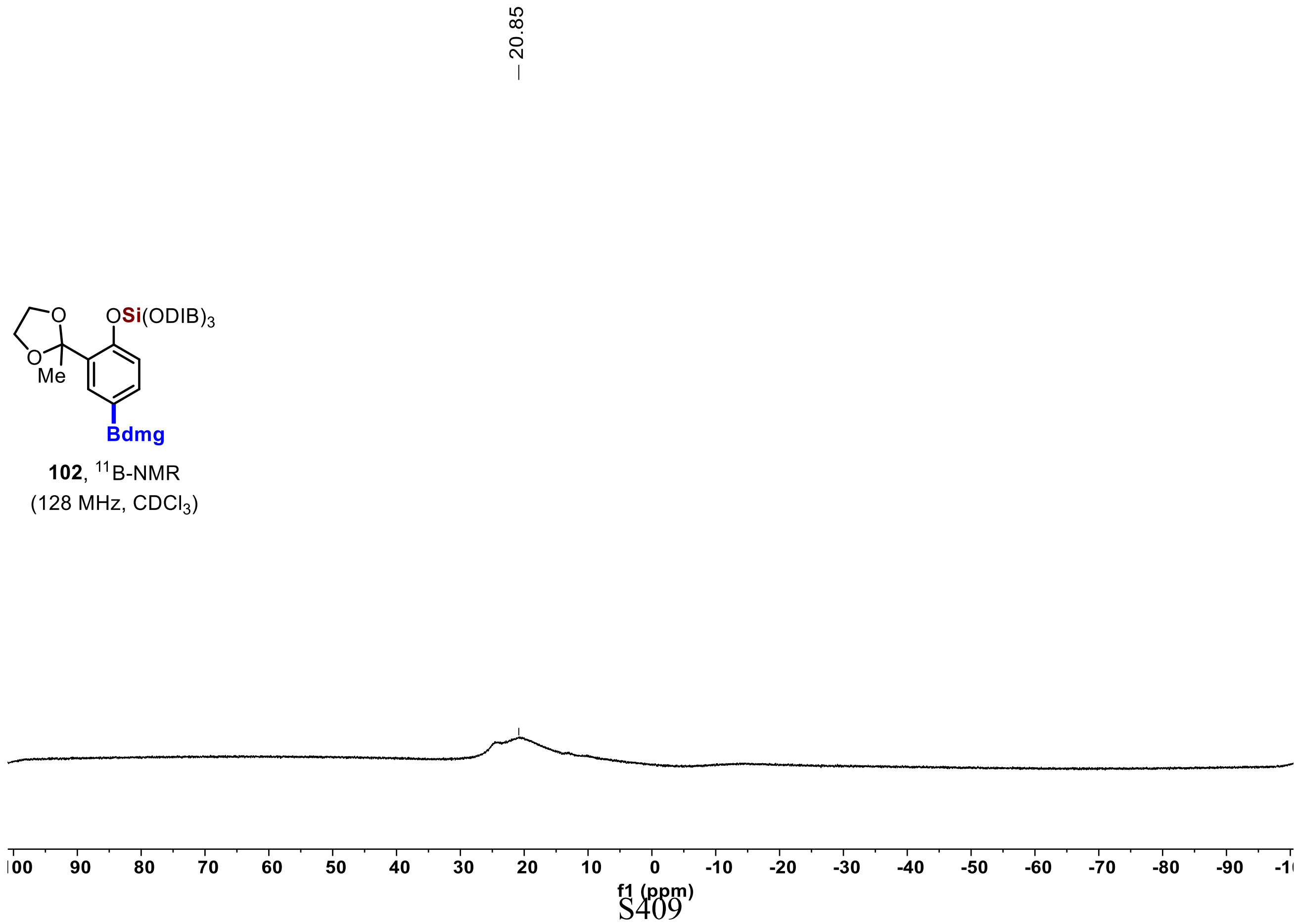

102, ^{11}B-NMR
(128 MHz, $CDCl_3$)

NOESY spectrum of 102 (400 MHz, $CDCl_3$)

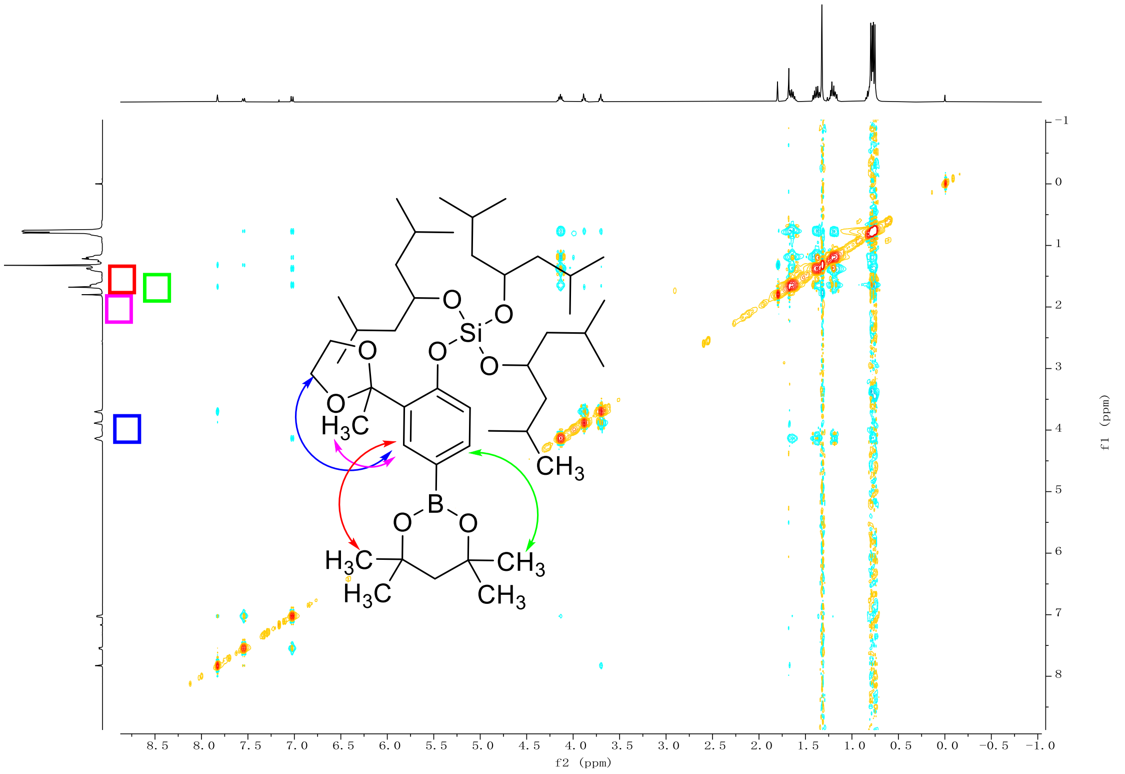

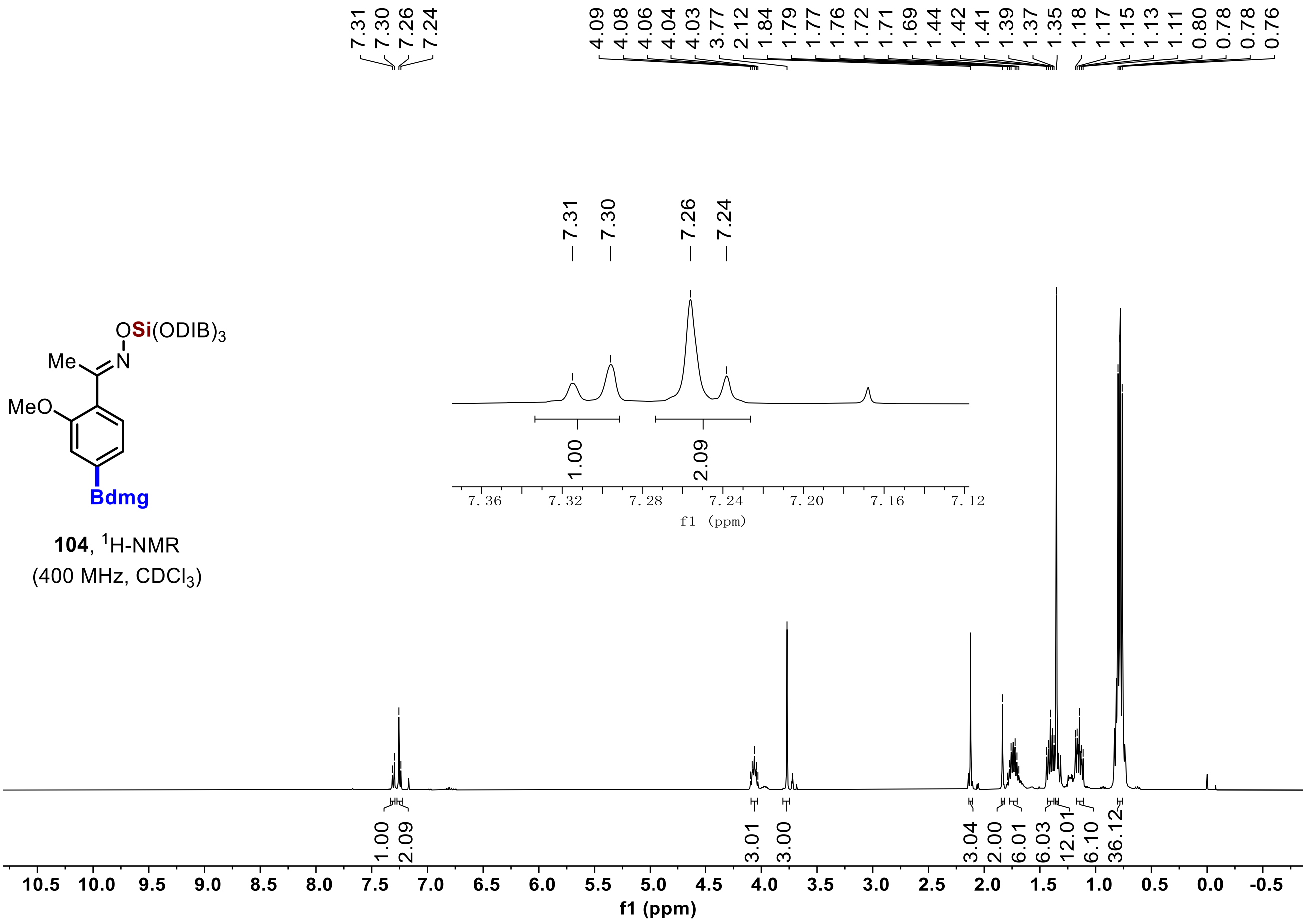

104, ^{1}H-NMR
(400 MHz, $CDCl_3$)

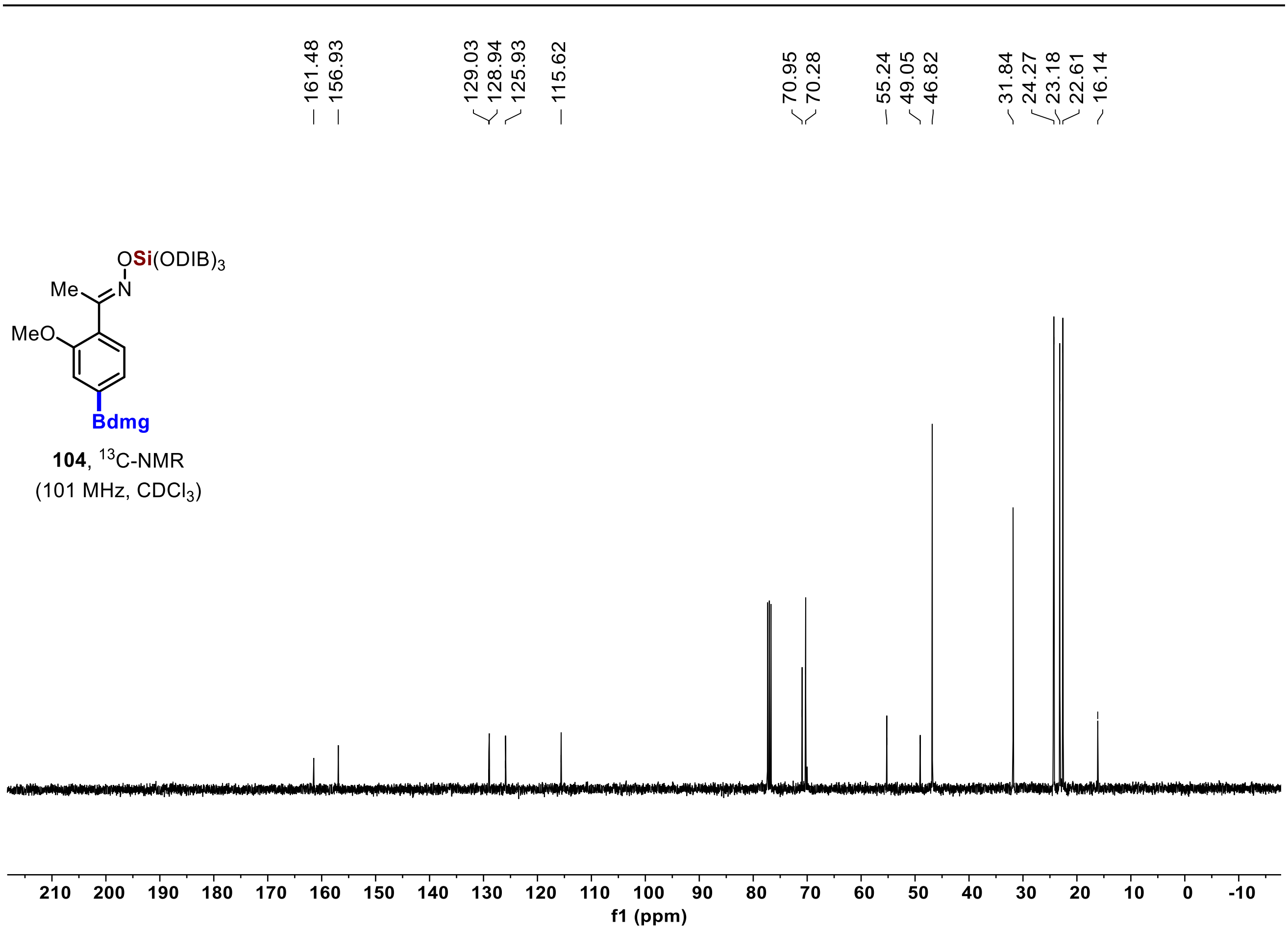
161.48
156.93
129.03
128.94
125.93
115.62
70.95
70.28
55.24
49.05
46.82
31.84
24.27
23.18
22.61
16.14
OSi(ODIB)3
Me
N
MeO
Bdmg
104, 13C-NMR
(101 MHz, CDCl3)
210 200 190 180 170 160 150 140 130 120 110 100 90 80 70 60 50 40 30 20 10 0 -10
f1 (ppm)

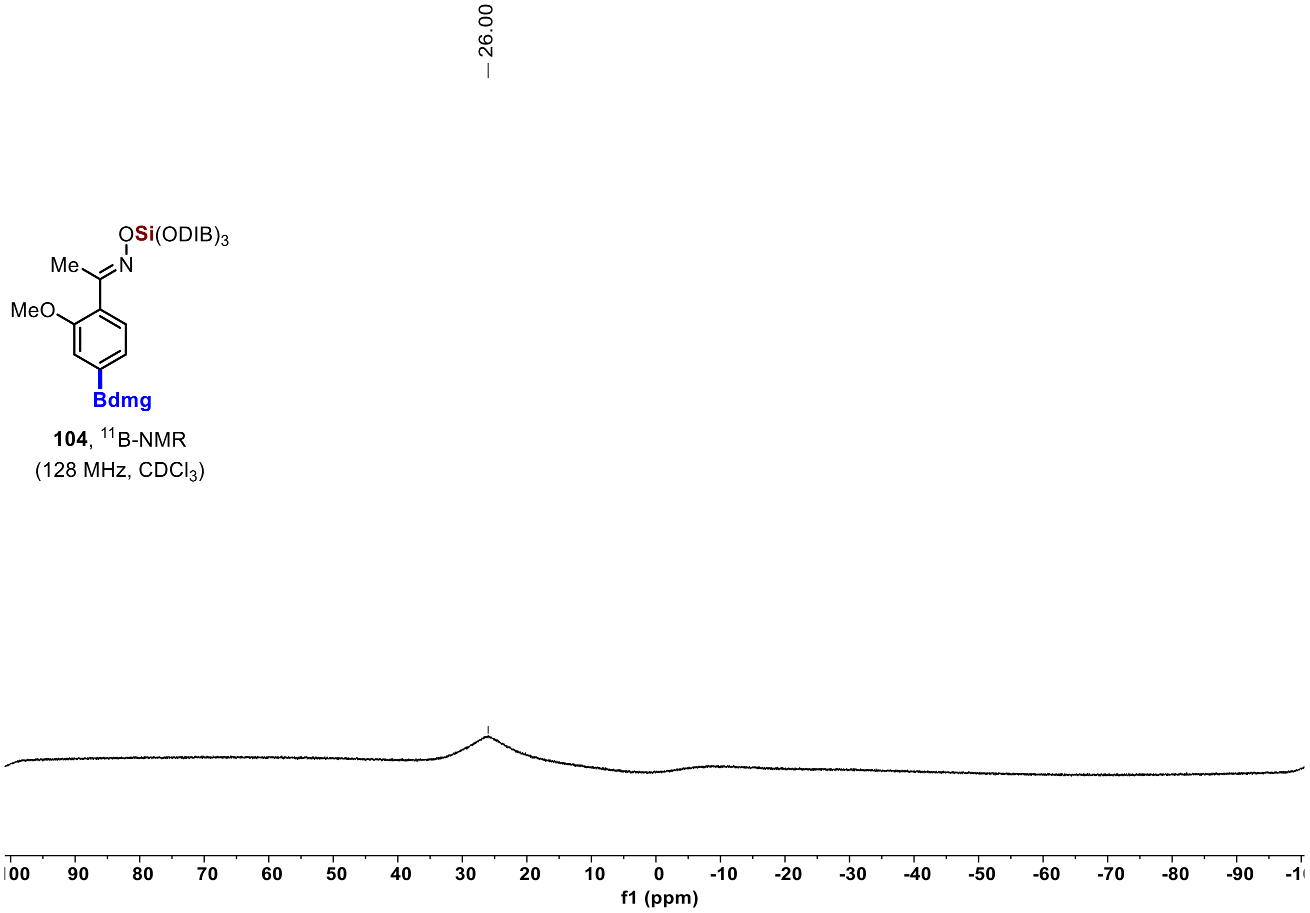
26.00
OSi(ODIB)3
Me
N
MeO
Bdmg
104, 11B-NMR
(128 MHz, CDCl3)
90 80 70 60 50 40 30 20 10 0 -10 -20 -30 -40 -50 -60 -70 -80 -90
f1 (ppm)

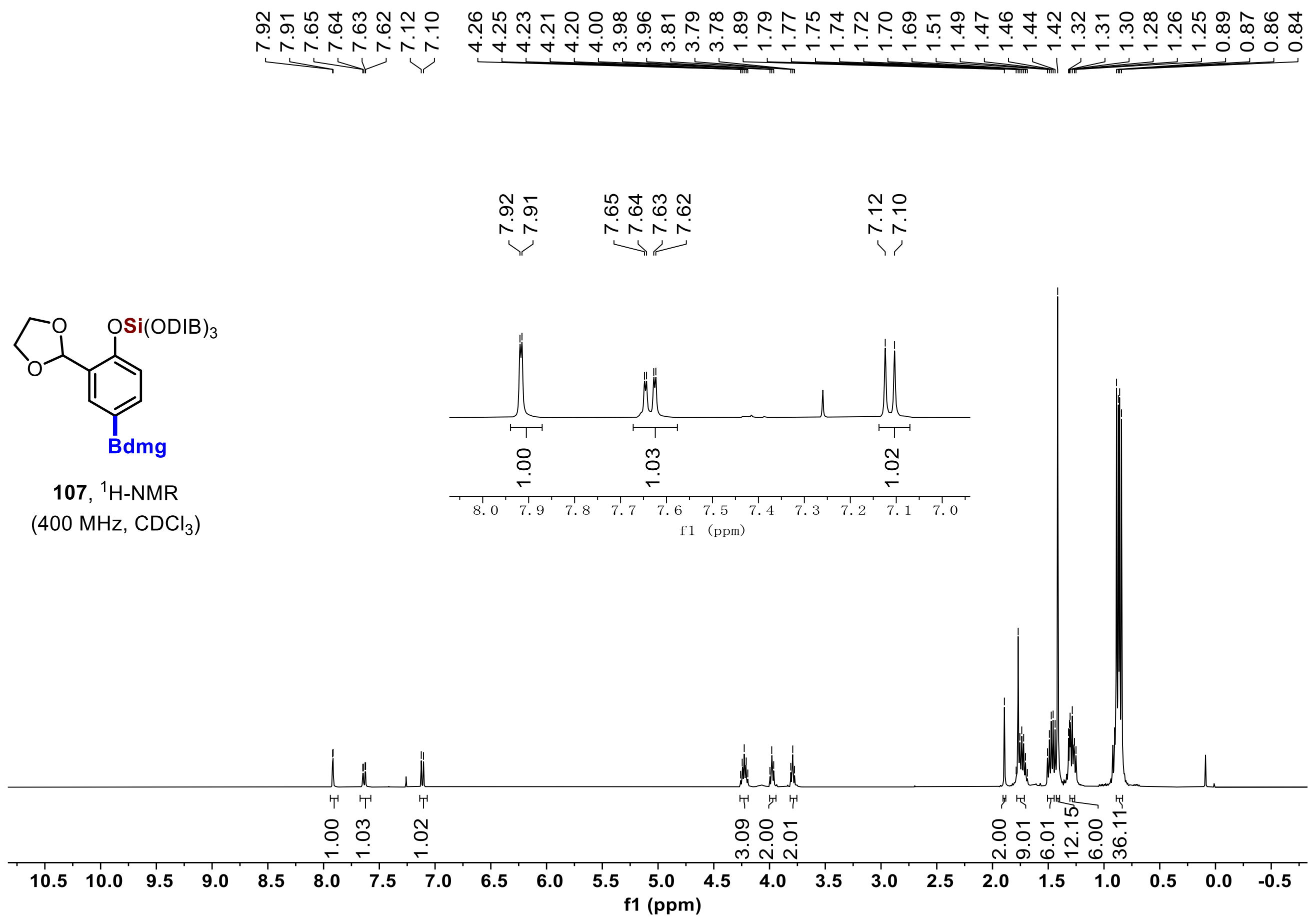
OSi(ODIB)3
Bdmg
107, 1H-NMR
(400 MHz, CDCl3)
f1 (ppm)

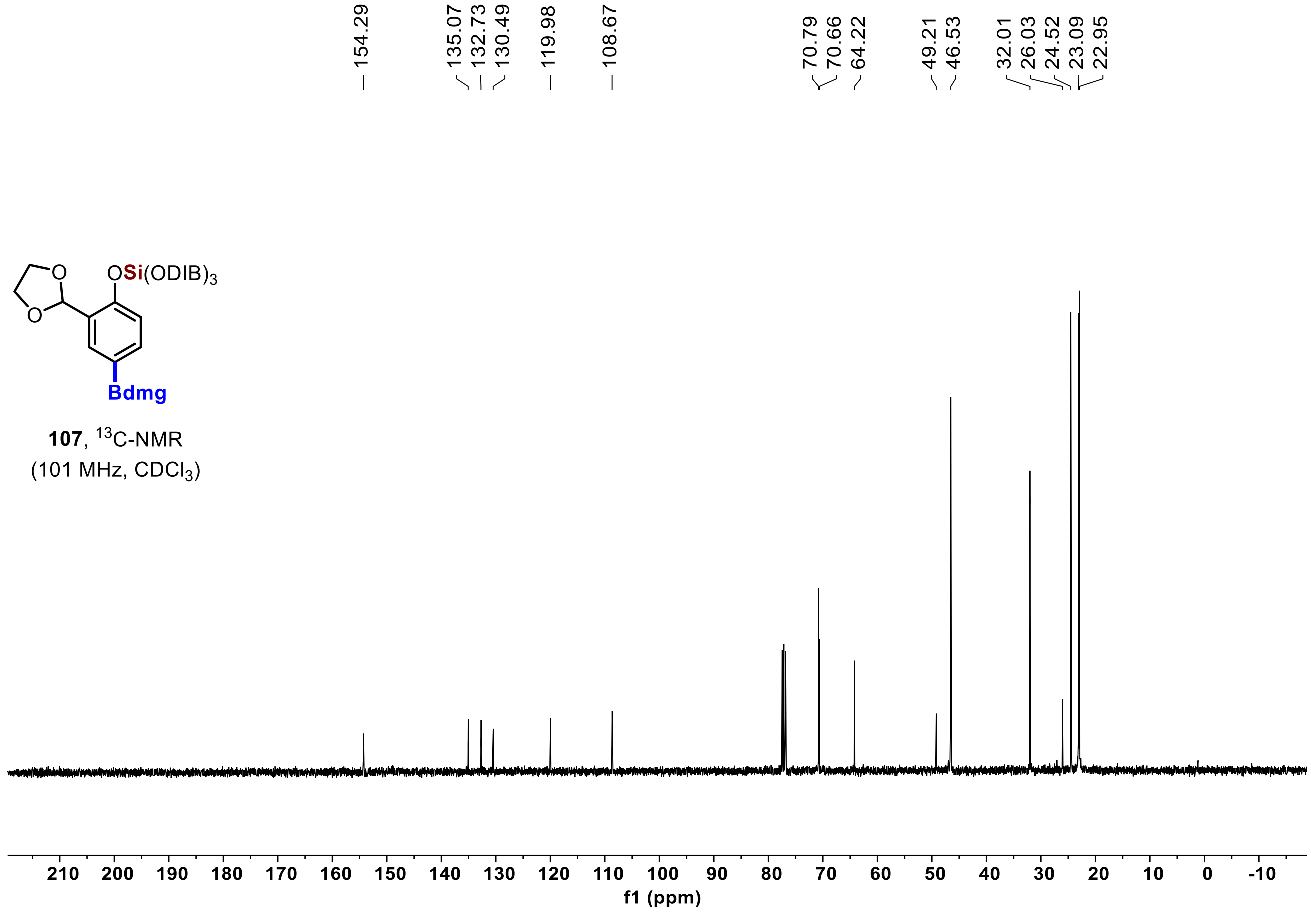
OSi(ODIB)3
Bdmg
107, 13C-NMR
(101 MHz, CDCl3)
f1 (ppm)

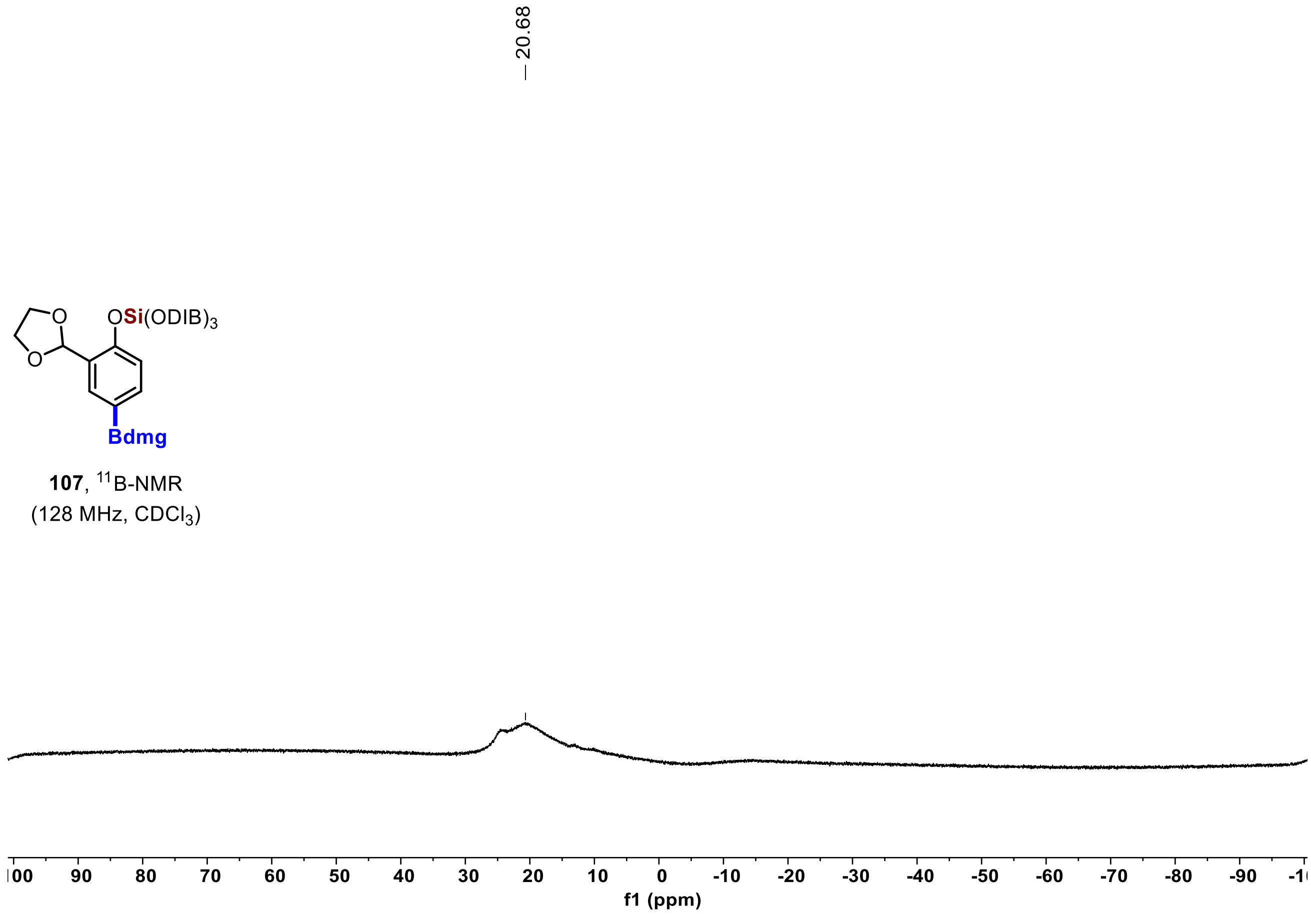

107, ^{11}B-NMR
(128 MHz, $CDCl_3$)

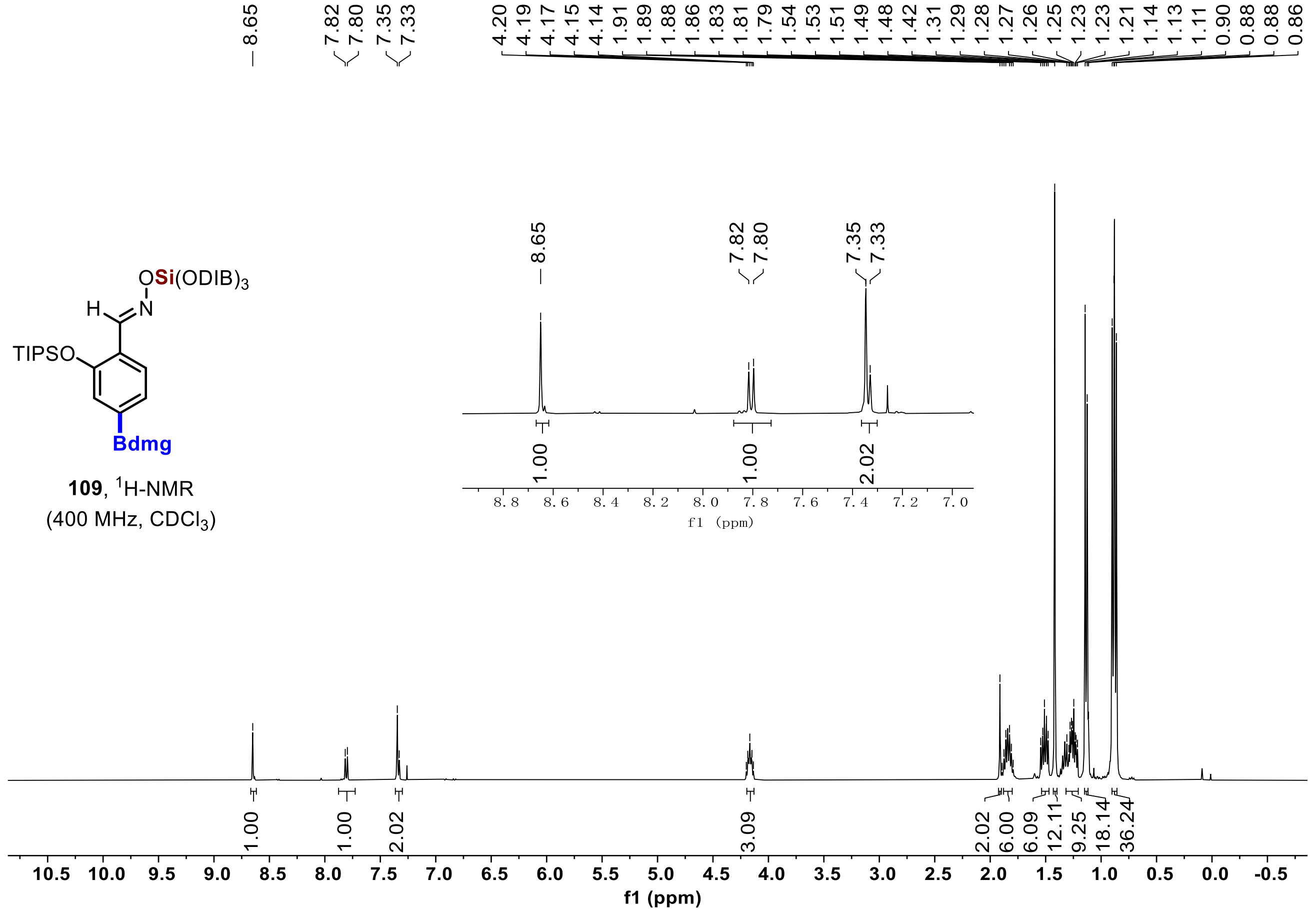

109, ^{1}H-NMR
(400 MHz, $CDCl_3$)

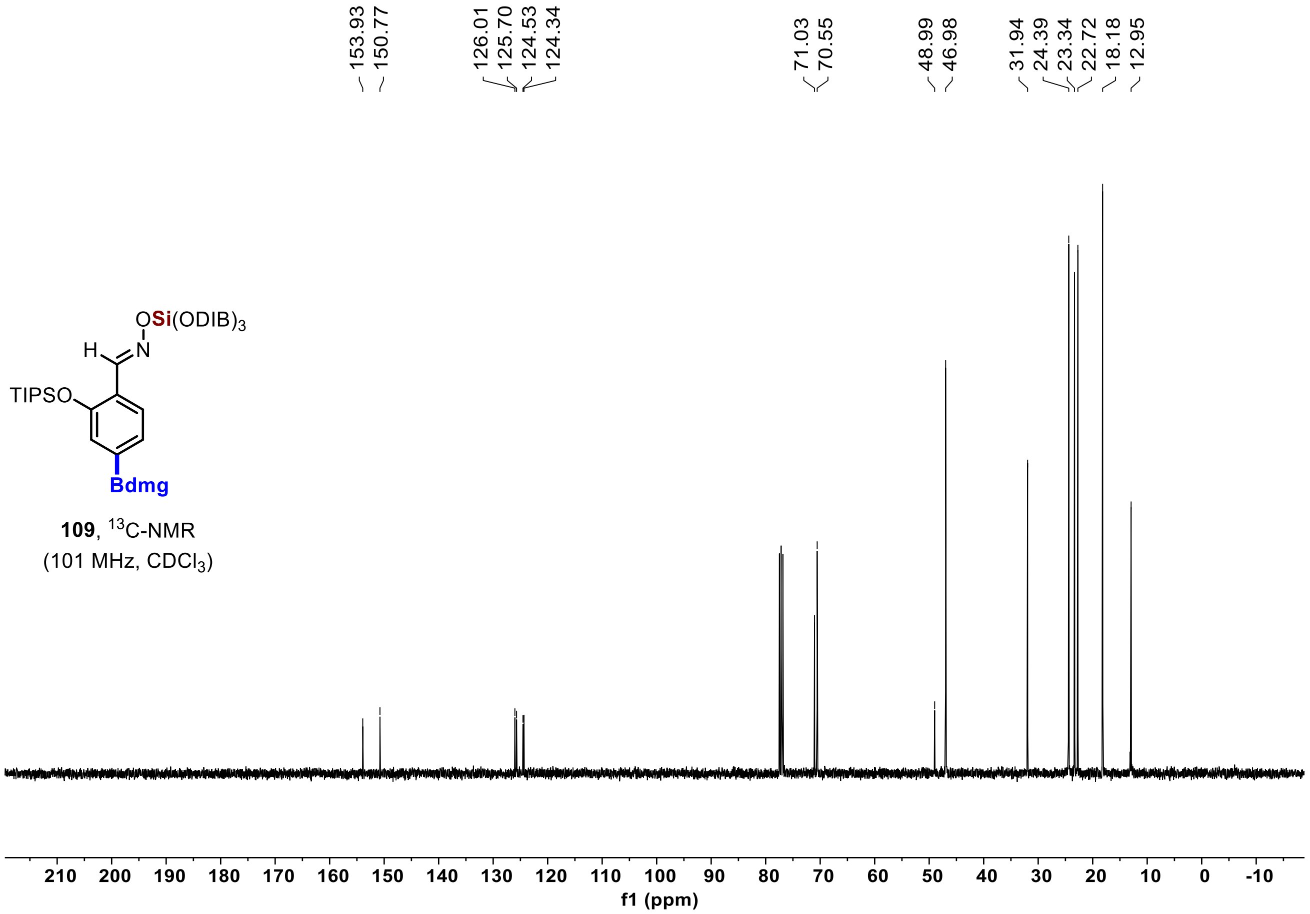
153.93
150.77
126.01
125.70
124.53
124.34
71.03
70.55
48.99
46.98
31.94
24.39
23.34
22.72
18.18
12.95
OSi(ODIB)3
H
N
TIPSO
Bdmg
109, 13C-NMR
(101 MHz, CDCl3)
210 200 190 180 170 160 150 140 130 120 110 100 90 80 70 60 50 40 30 20 10 0 -10
f1 (ppm)

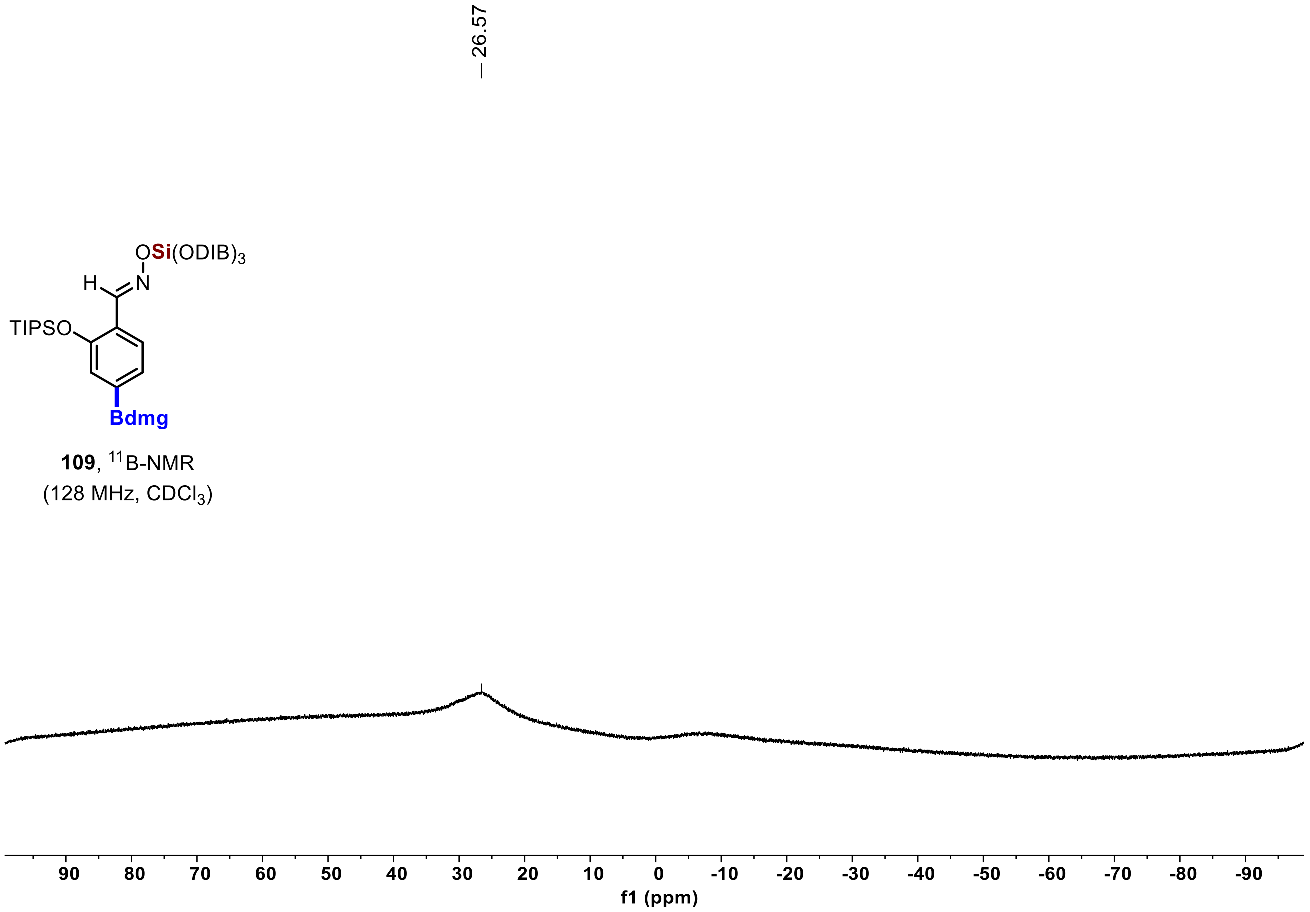
26.57
OSi(ODIB)3
H
N
TIPSO
Bdmg
109, 11B-NMR
(128 MHz, CDCl3)
90 80 70 60 50 40 30 20 10 0 -10 -20 -30 -40 -50 -60 -70 -80 -90
f1 (ppm)

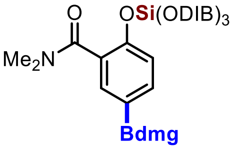

112, ^{1}H-NMR
(400 MHz, $CDCl_3$)

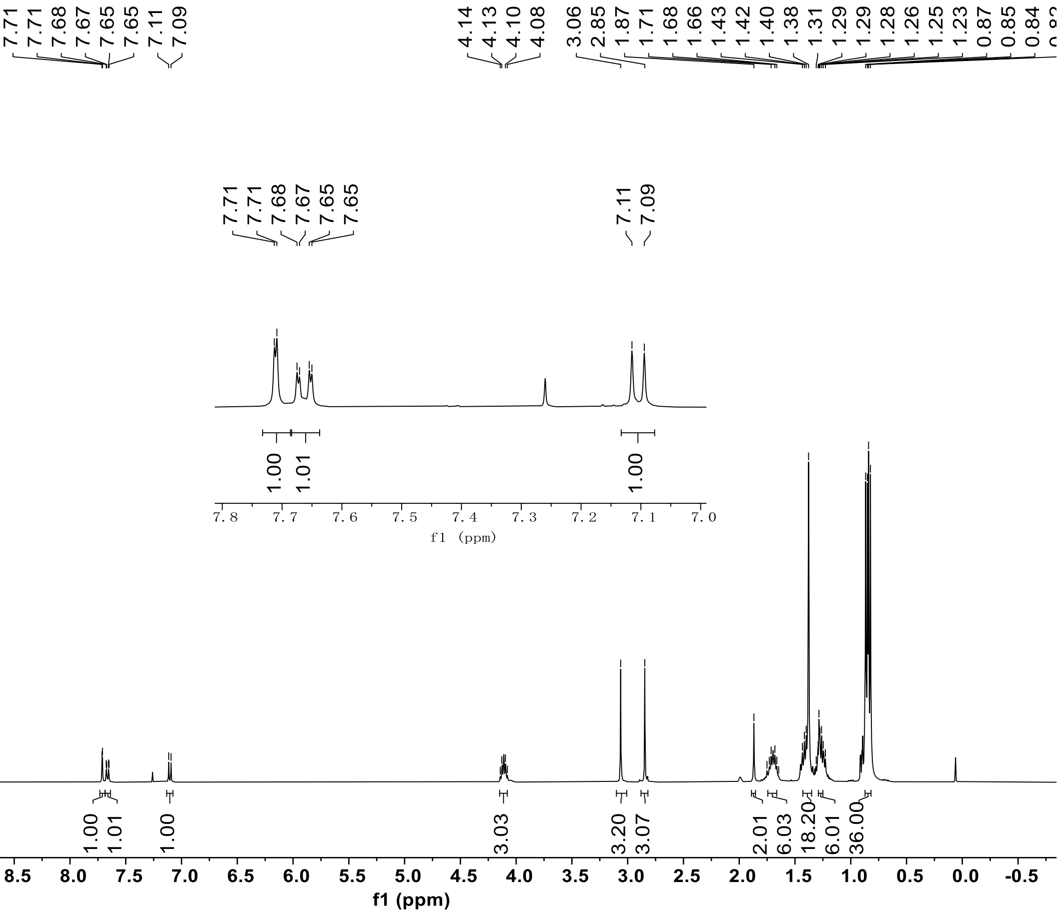

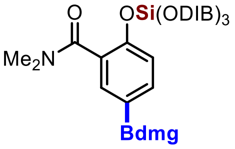

112, ^{13}C-NMR
(101 MHz, $CDCl_3$)

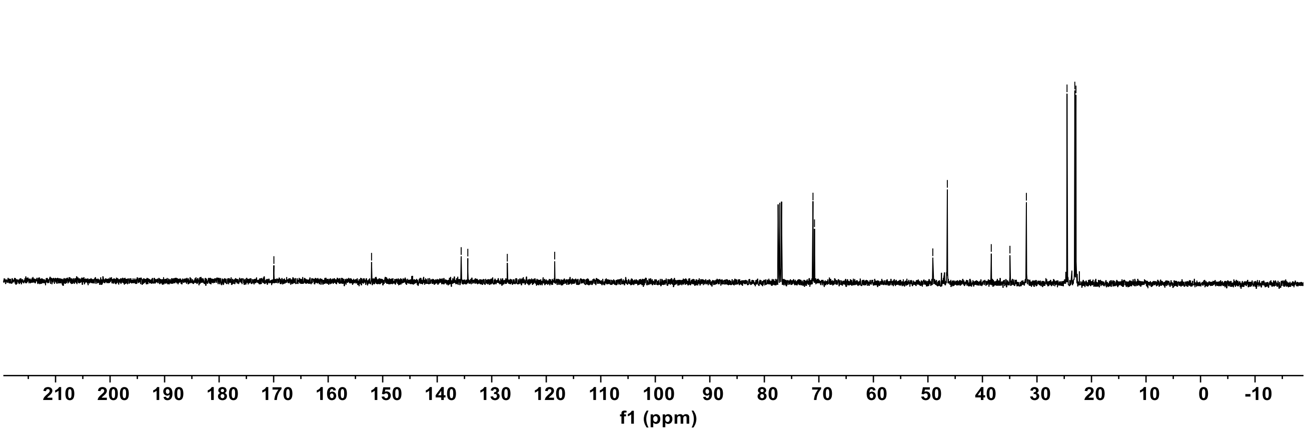

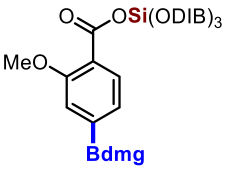

114, ^{1}H-NMR
(400 MHz, $CDCl_3$)

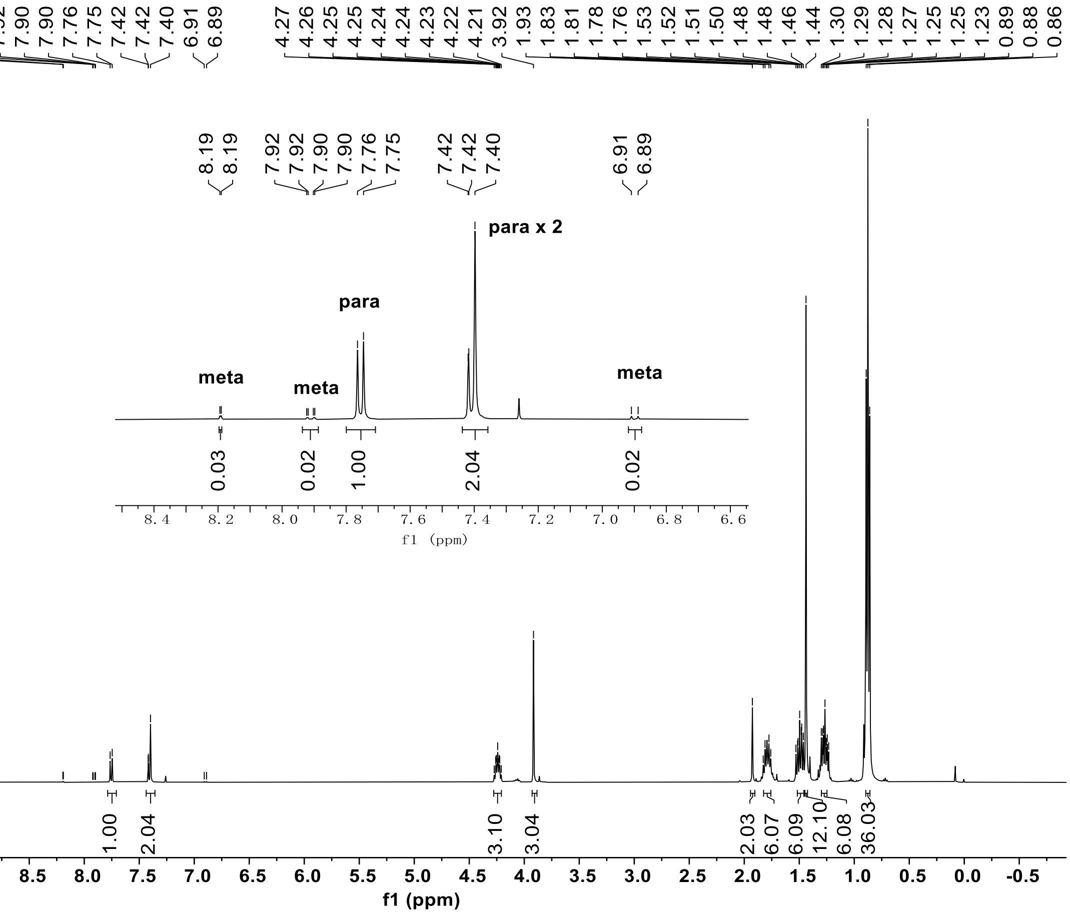

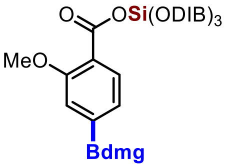

114, ^{13}C-NMR
(101 MHz, $CDCl_3$)

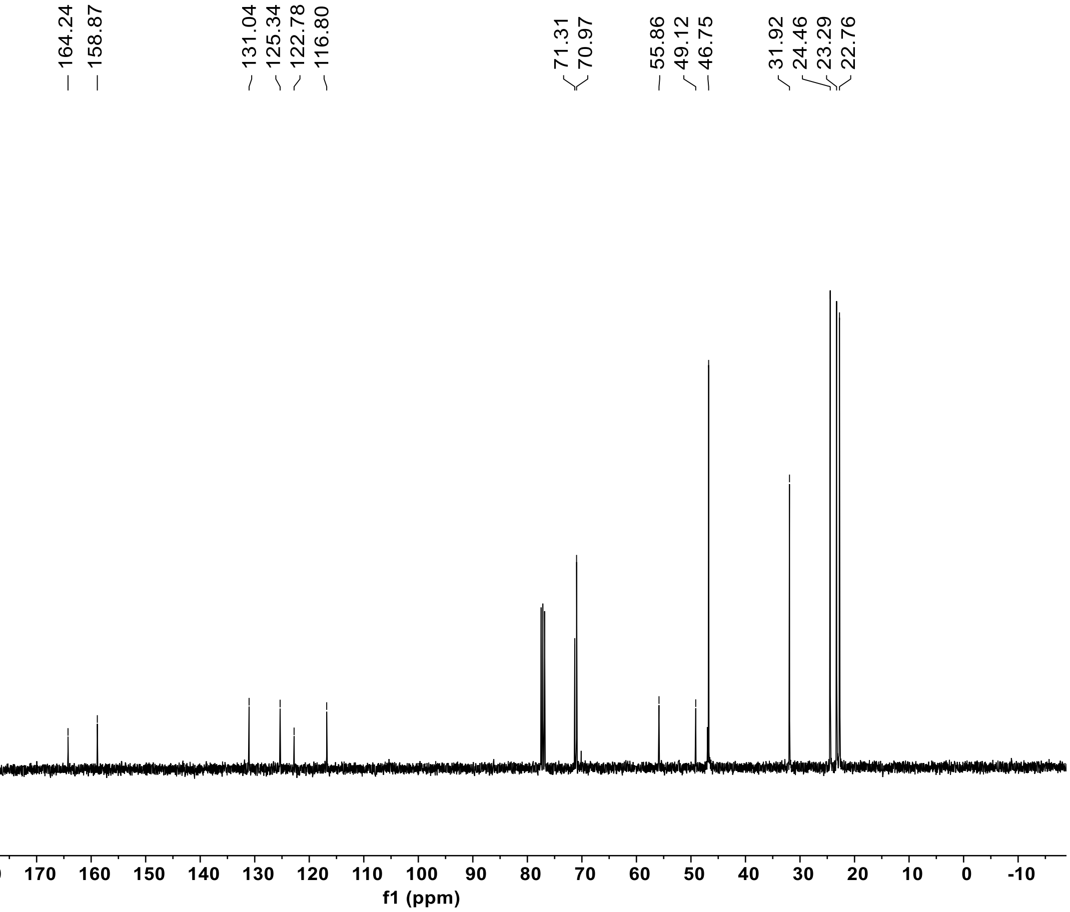

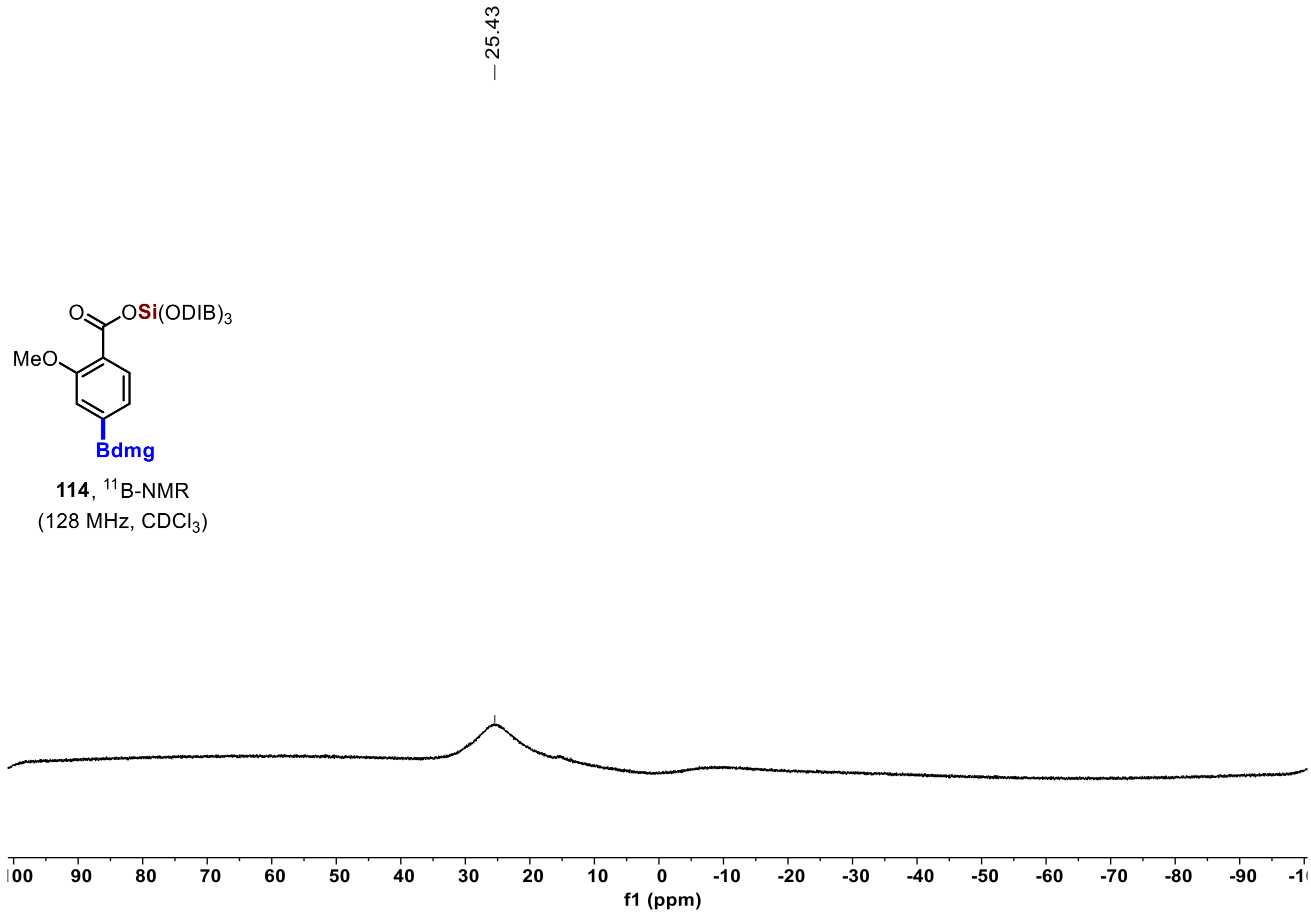

114, ^{11}B-NMR
(128 MHz, $CDCl_3$)

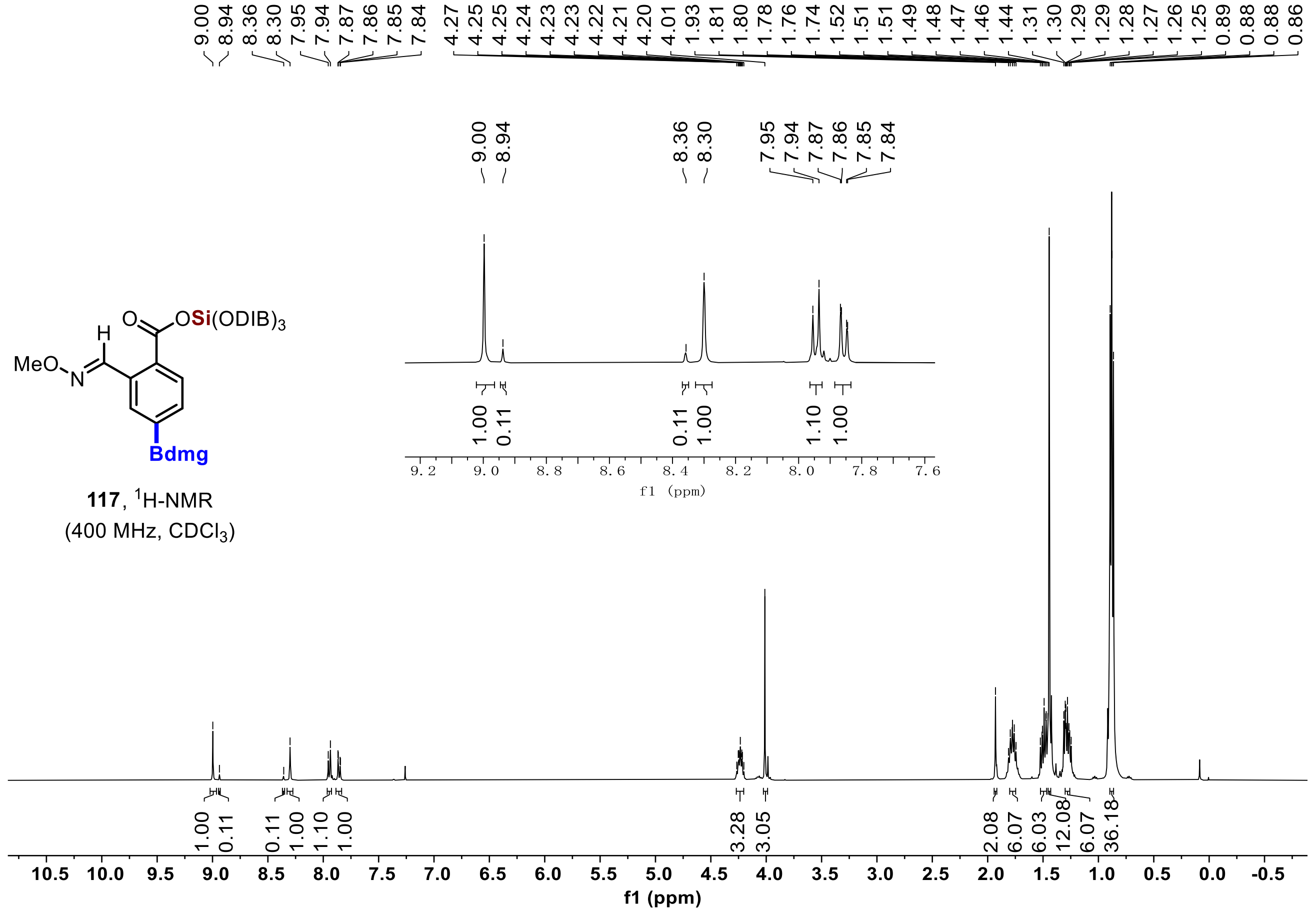

117, ^{1}H-NMR
(400 MHz, $CDCl_3$)

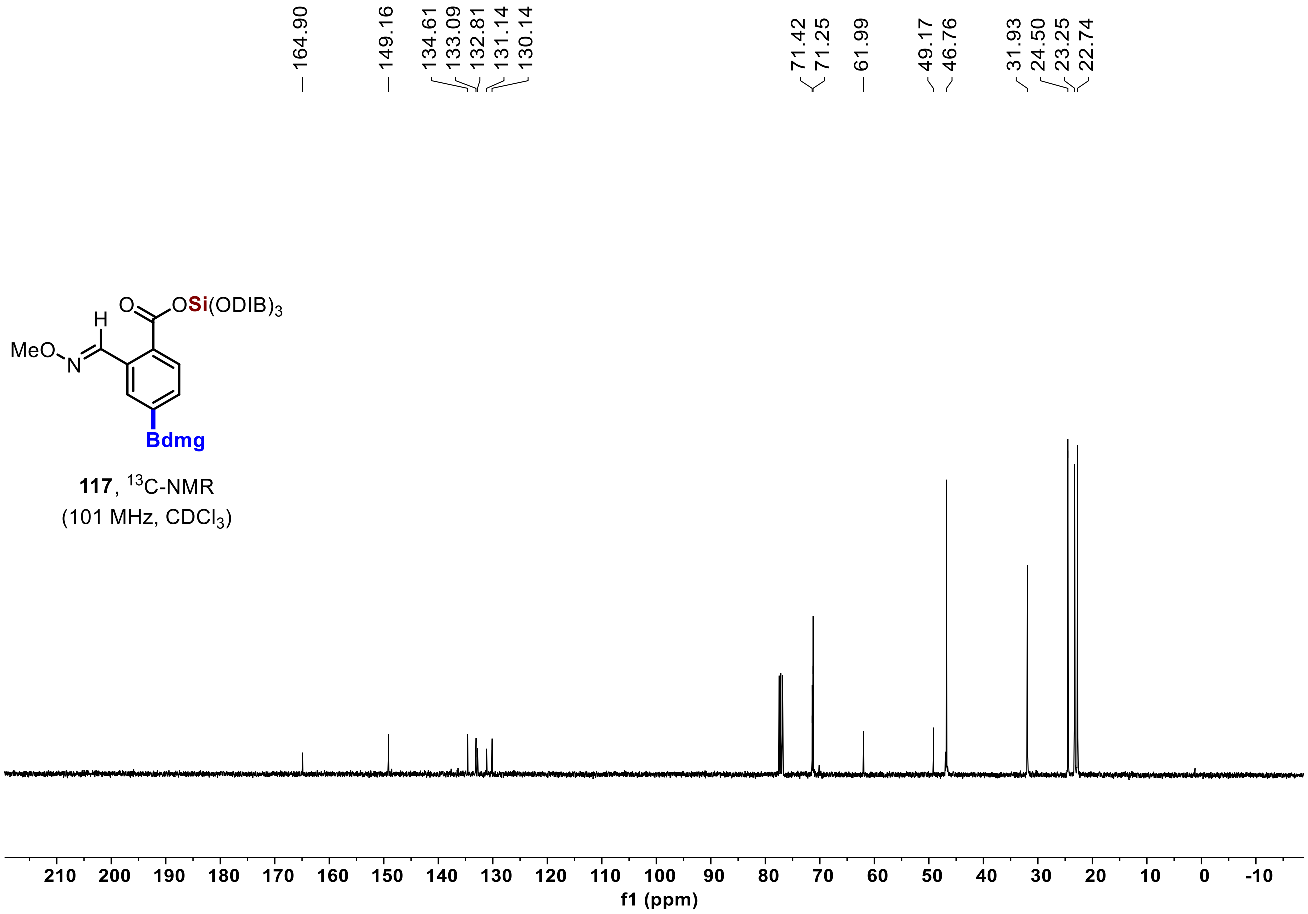
164.90
149.16
134.61
133.09
132.81
131.14
130.14
71.42
71.25
61.99
49.17
46.76
31.93
24.50
23.25
22.74
O
OSi(ODIB)3
H
MeO
N
Bdmg
117, 13C-NMR
(101 MHz, CDCl3)
210 200 190 180 170 160 150 140 130 120 110 100 90 80 70 60 50 40 30 20 10 0 -10
f1 (ppm)

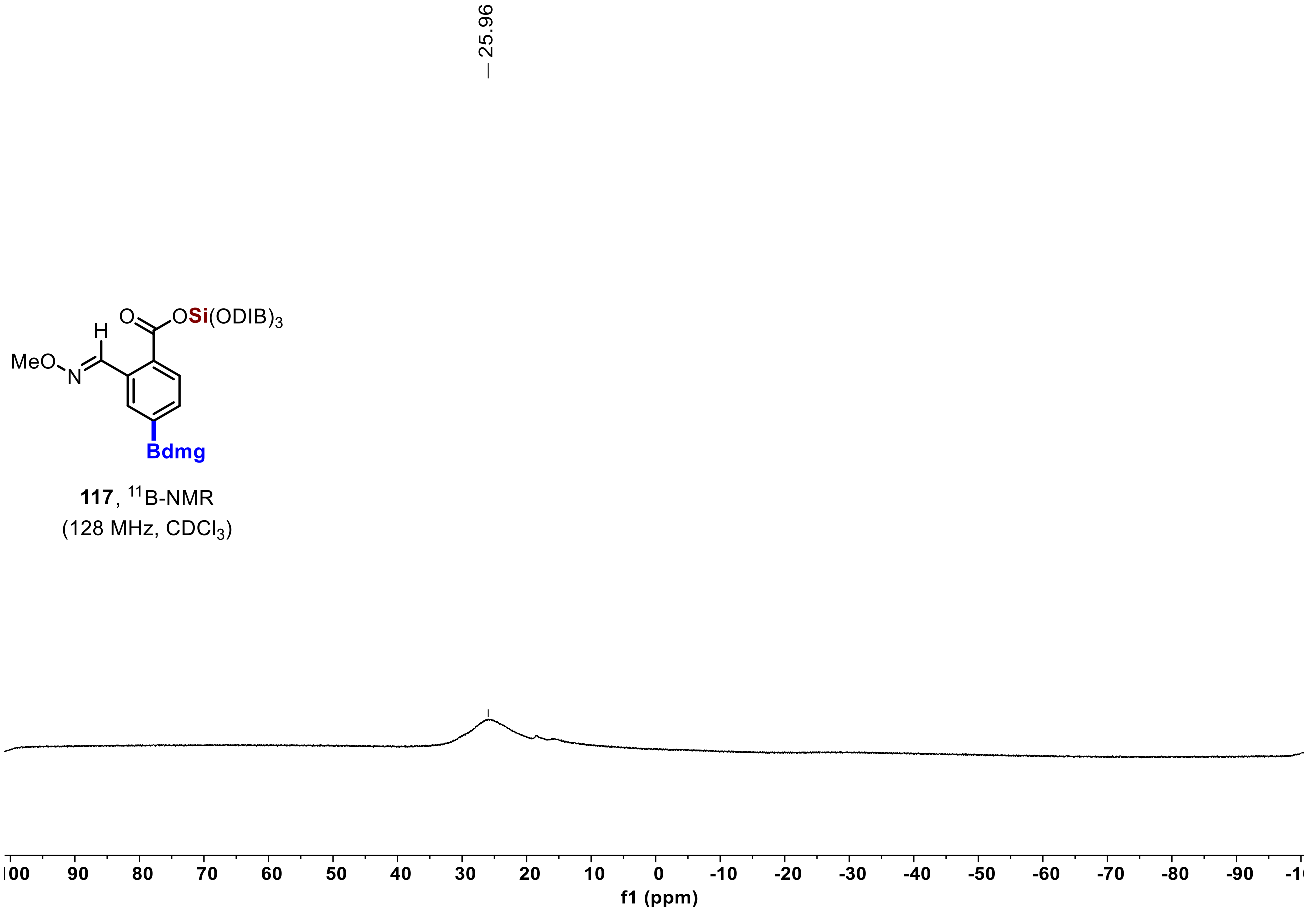
25.96
O
OSi(ODIB)3
H
MeO
N
Bdmg
117, 11B-NMR
(128 MHz, CDCl3)
90 80 70 60 50 40 30 20 10 0 -10 -20 -30 -40 -50 -60 -70 -80 -90
f1 (ppm)

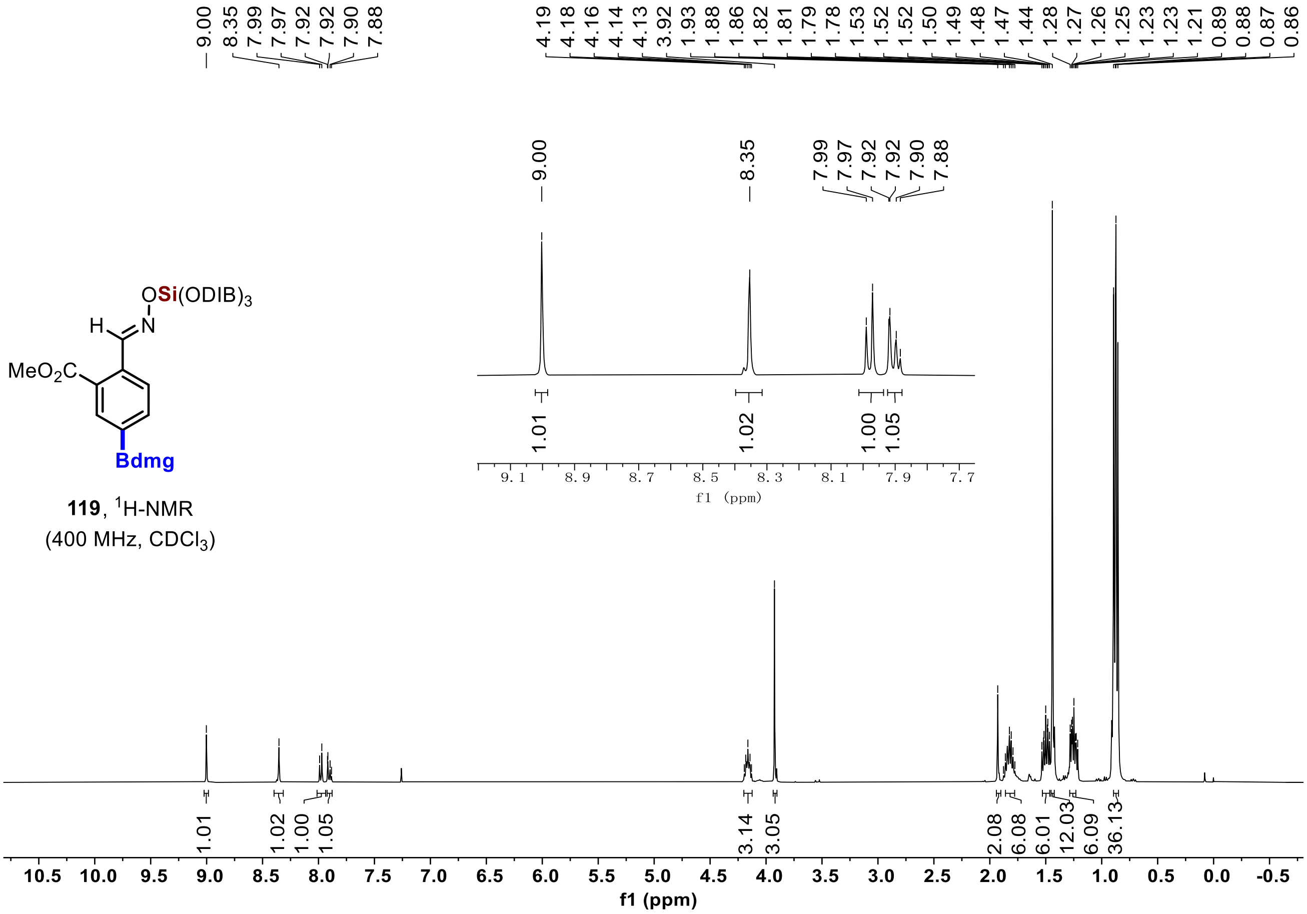

119, ^{1}H-NMR
(400 MHz, $CDCl_3$)

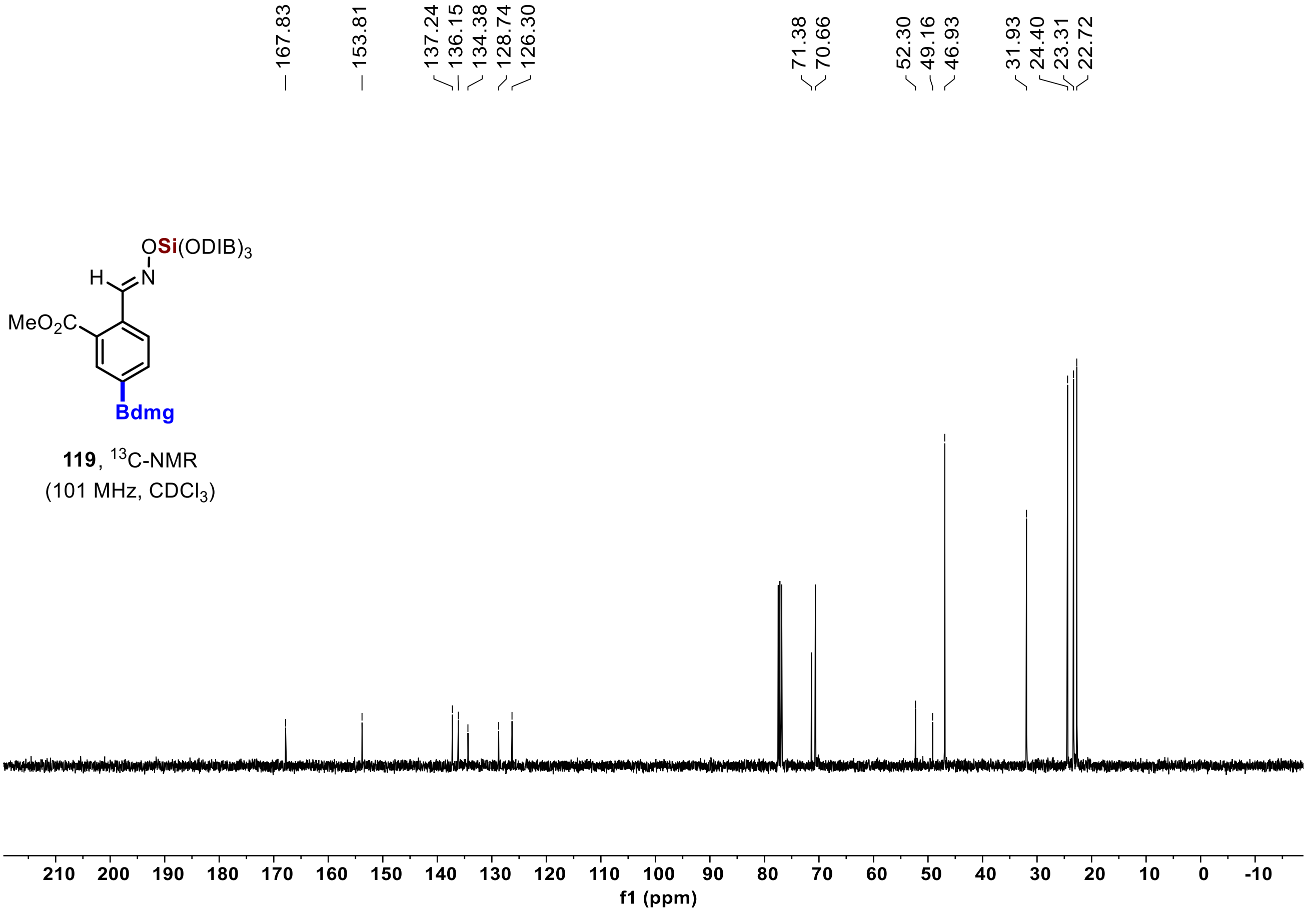

119, ^{13}C-NMR
(101 MHz, $CDCl_3$)

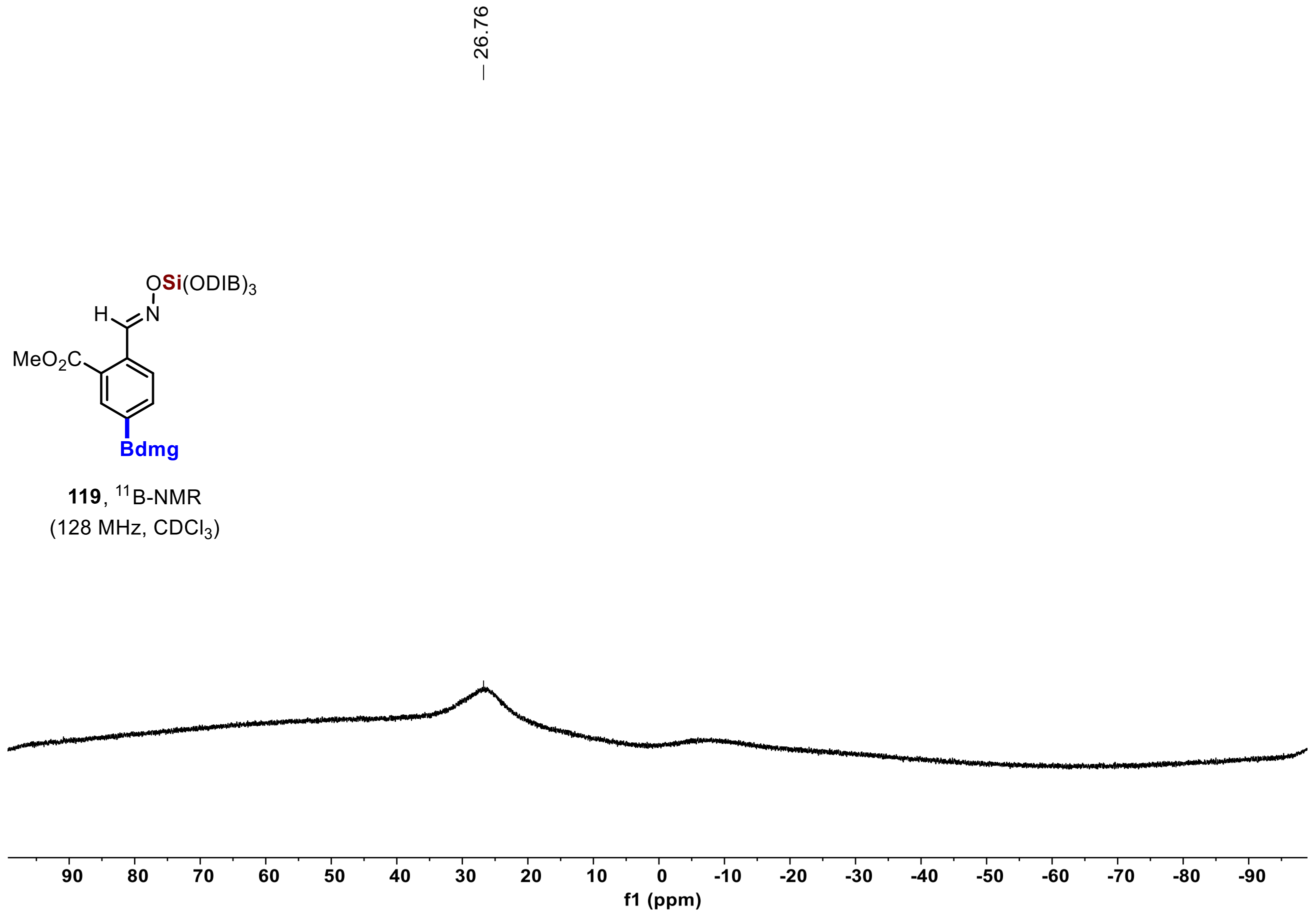
26.76
OSi(ODIB)3
H
N
MeO2C
Bdmg
119, 11B-NMR
(128 MHz, CDCl3)
f1 (ppm)

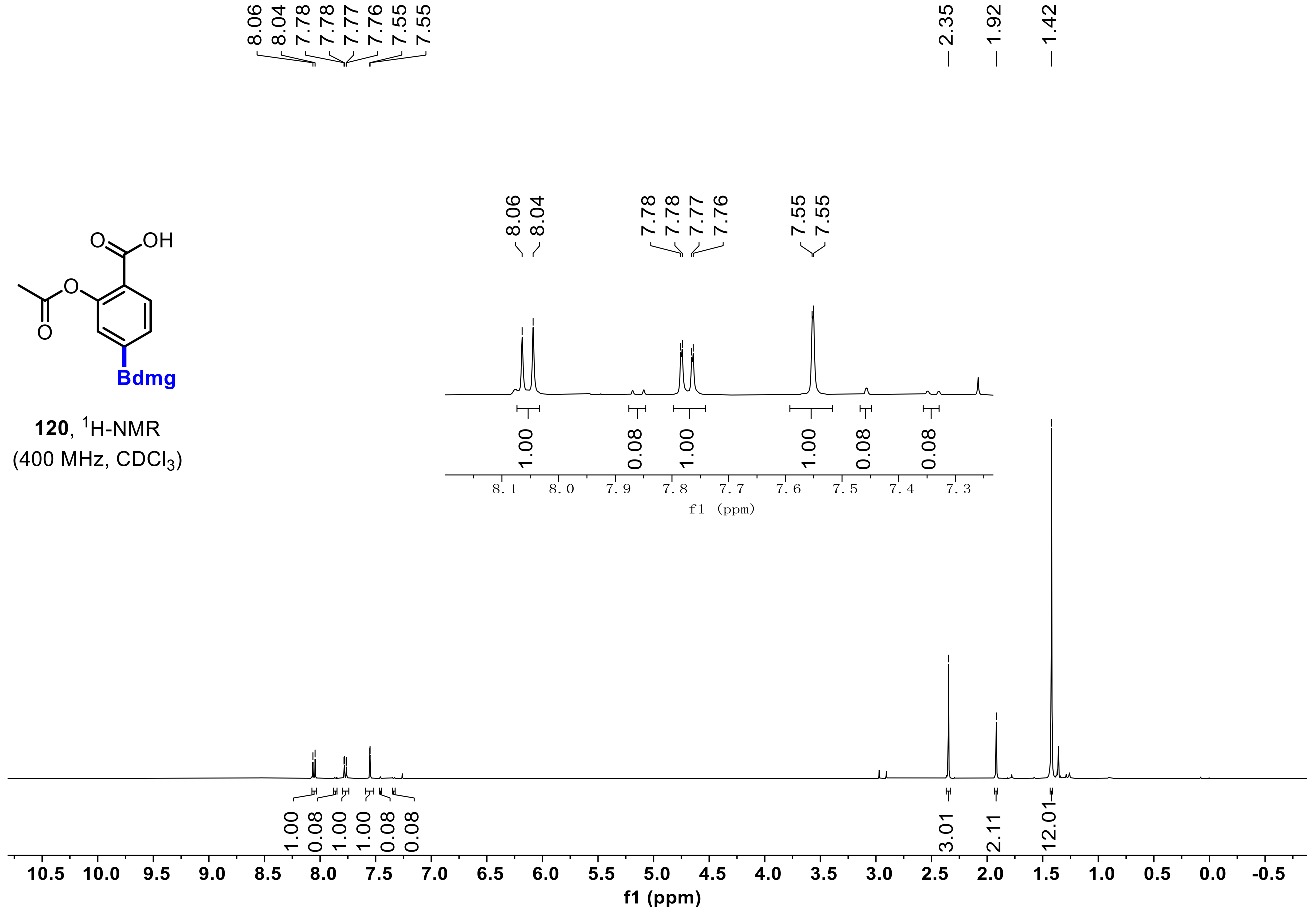
8.06
8.04
7.78
7.78
7.77
7.76
7.55
7.55
2.35
1.92
1.42
O
OH
O
O
Bdmg
120, 1H-NMR
(400 MHz, CDCl3)
1.00
0.08
1.00
1.00
0.08
0.08
3.01
2.11
12.01
f1 (ppm)

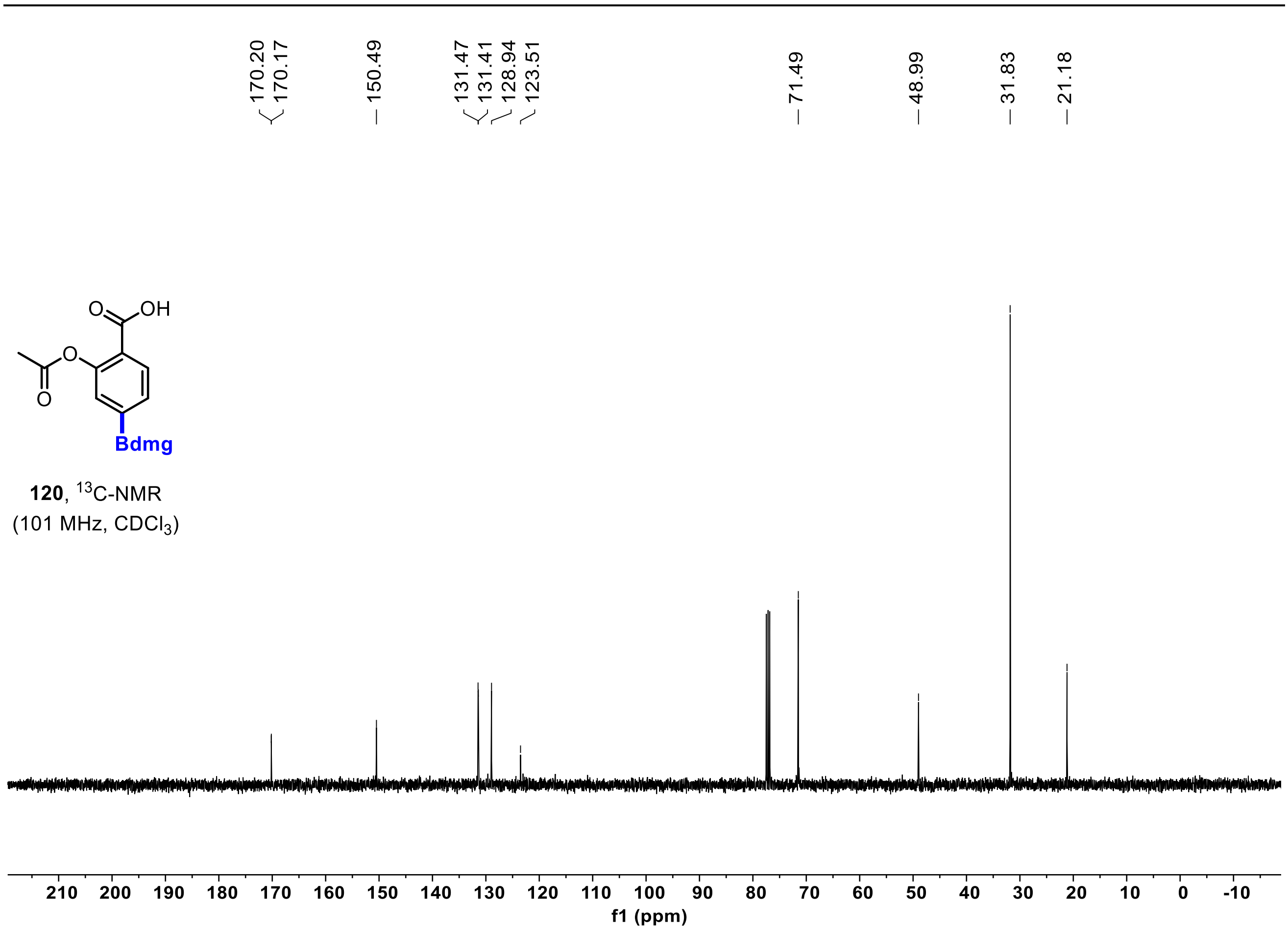

120, ^{13}C-NMR
(101 MHz, $CDCl_3$)

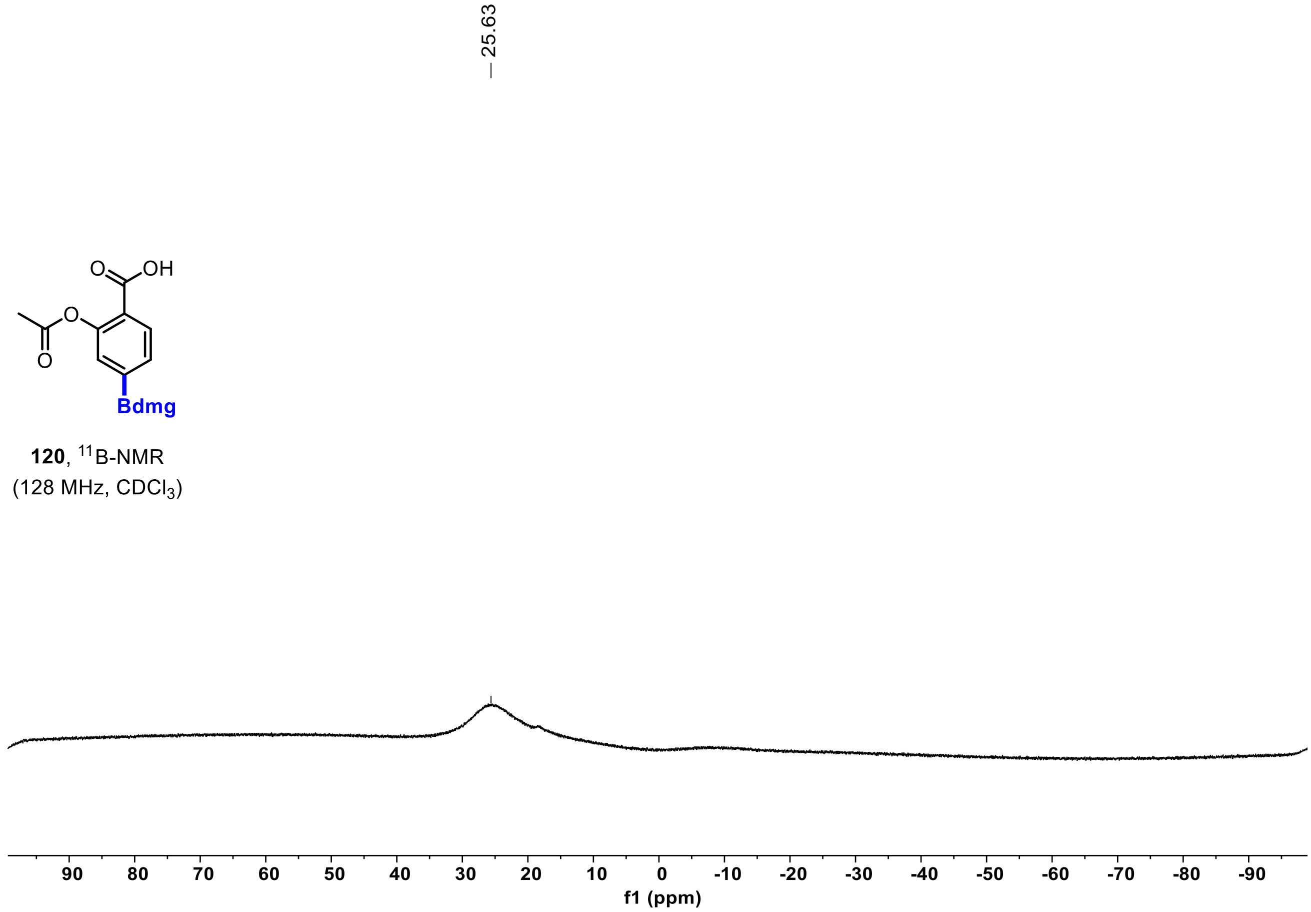

120, ^{11}B-NMR
(128 MHz, $CDCl_3$)

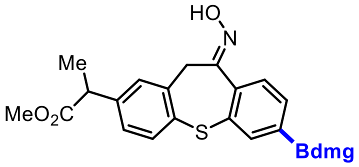

121, ^{1}H-NMR
(400 MHz, $CDCl_3$)

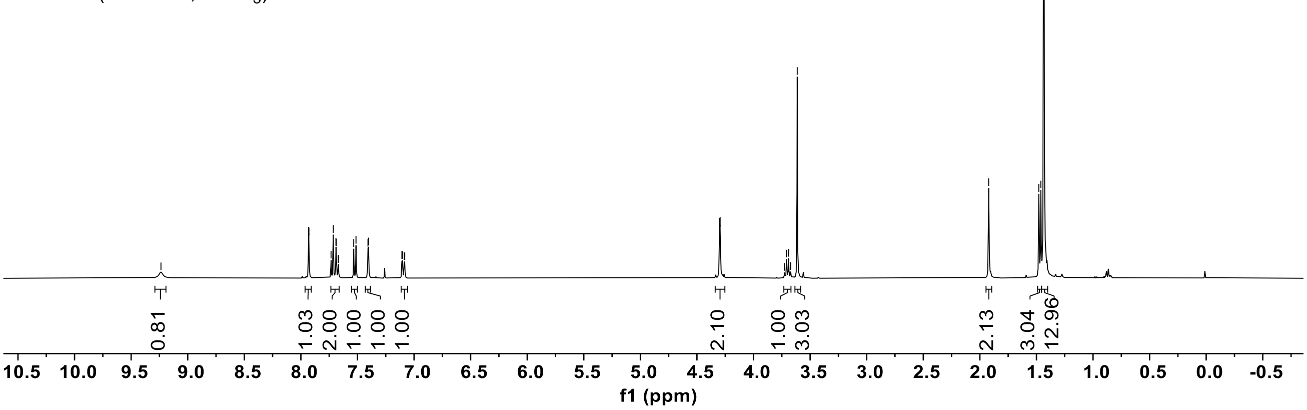

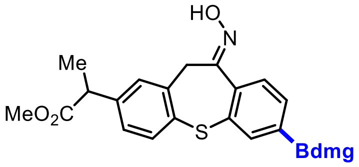

121, ^{13}C-NMR
(101 MHz, $CDCl_3$)

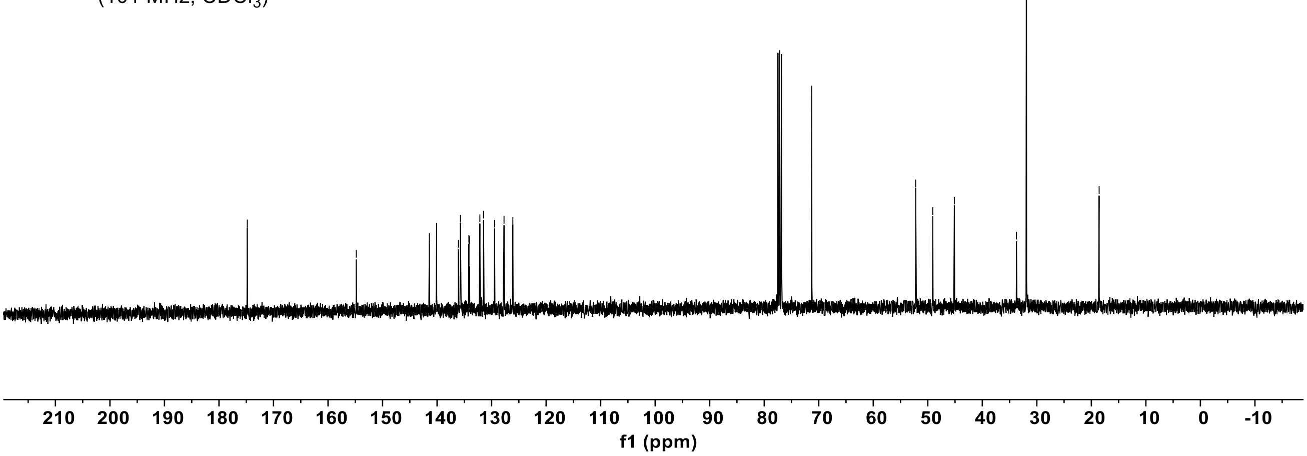

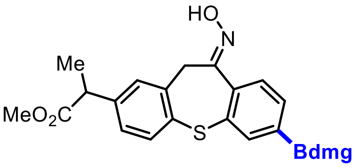

121, ^{11}B-NMR
(128 MHz, $CDCl_3$)

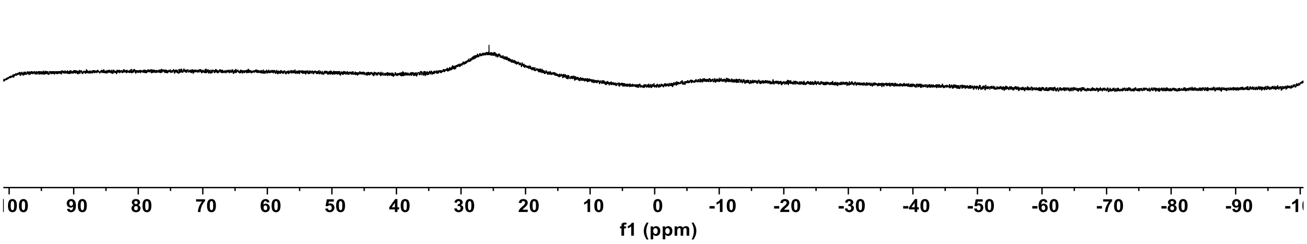

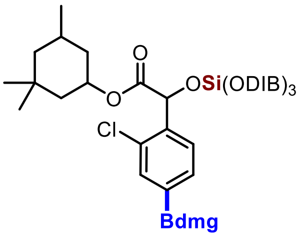

122, ^{1}H-NMR
(400 MHz, $CDCl_3$)

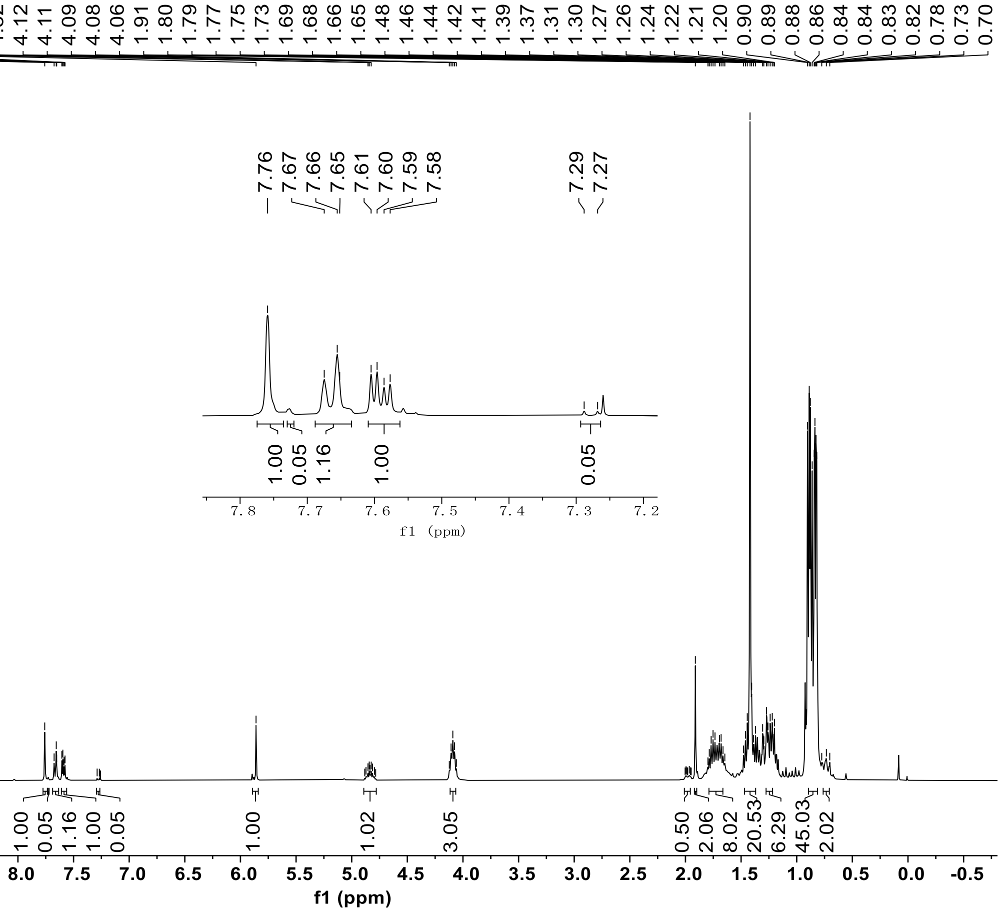

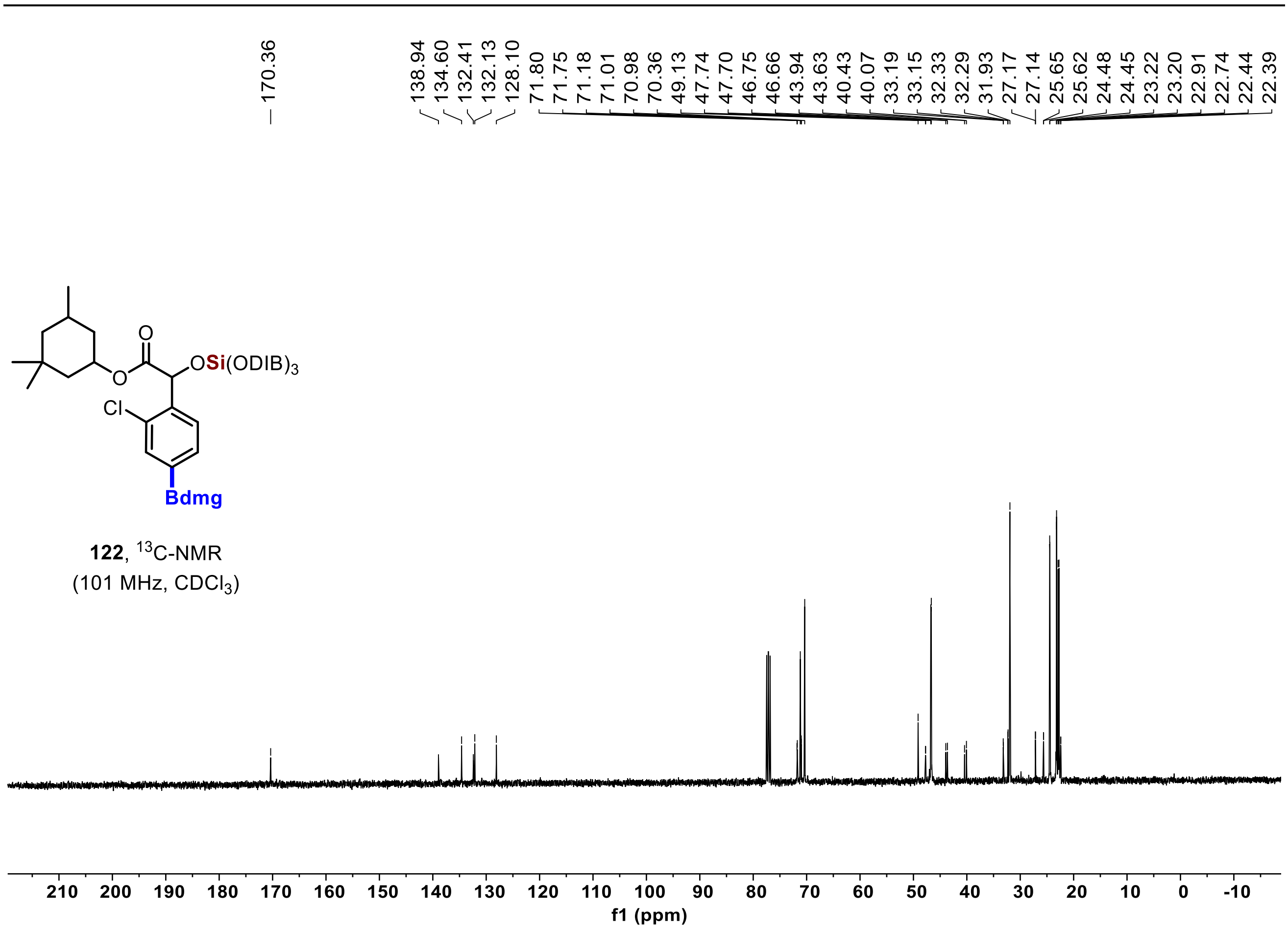

122, ^{13}C-NMR
(101 MHz, $CDCl_3$)

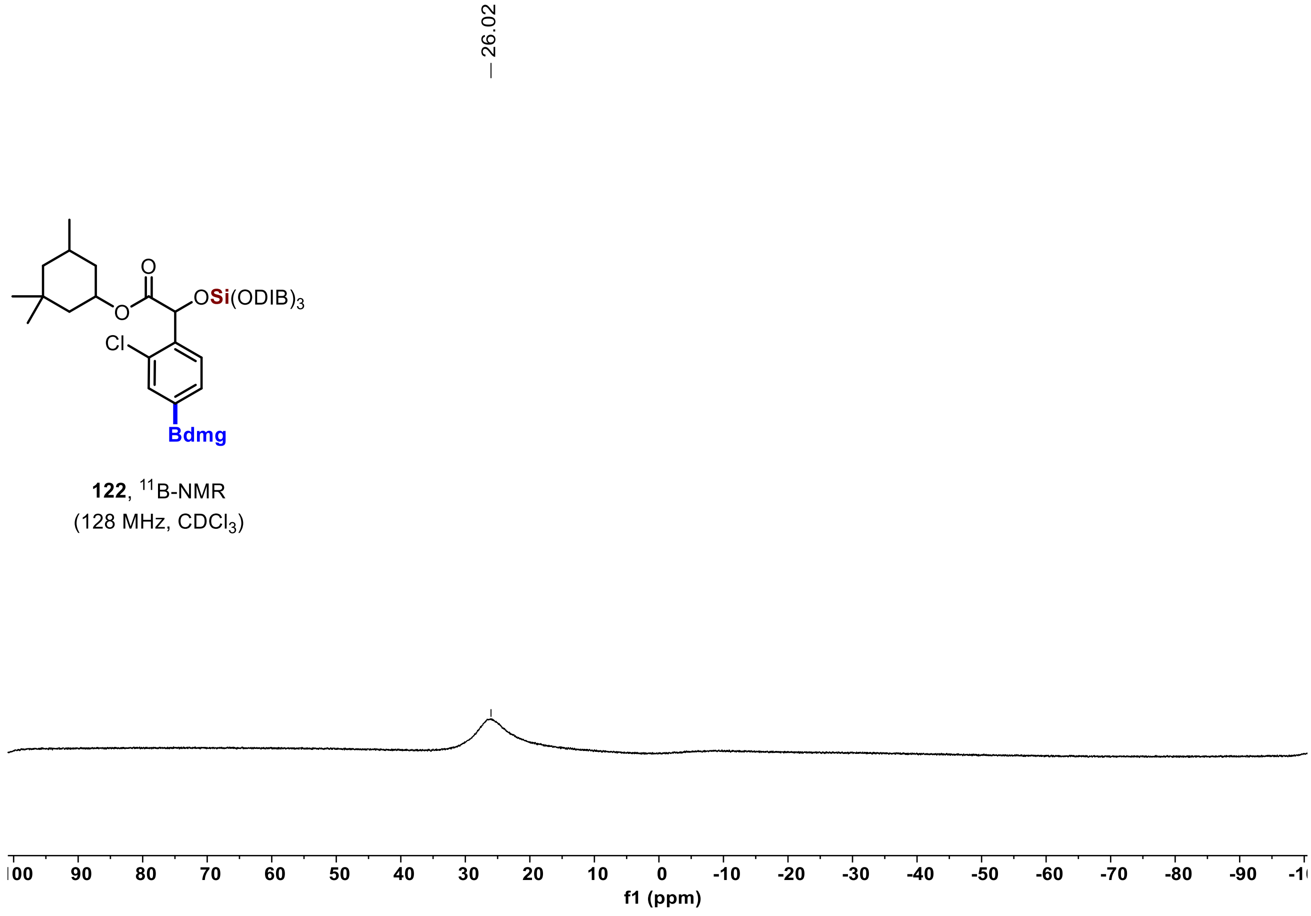

122, ^{11}B-NMR
(128 MHz, $CDCl_3$)

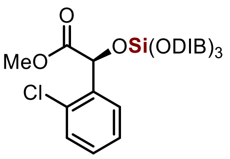

125, ^{1}H-NMR
(400 MHz, $CDCl_3$)

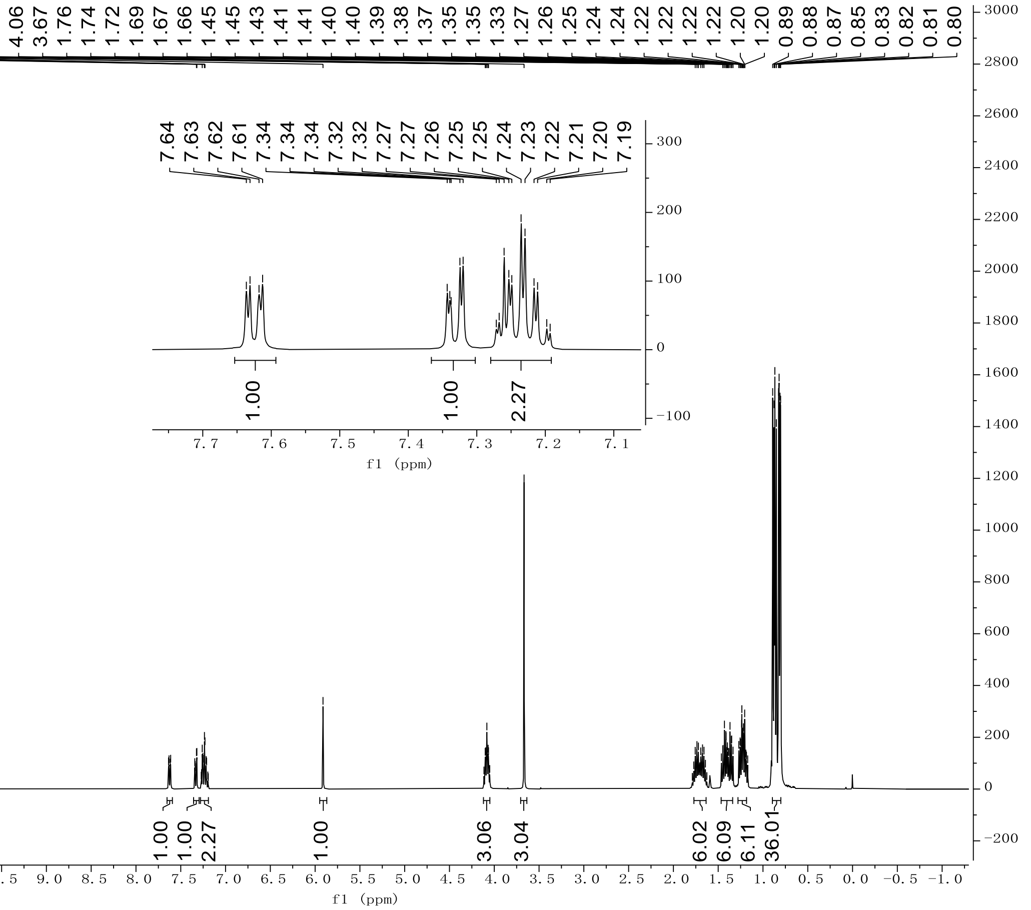

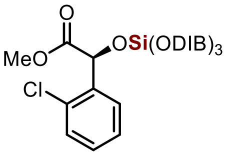

125, ^{13}C-NMR
(101 MHz, $CDCl_3$)

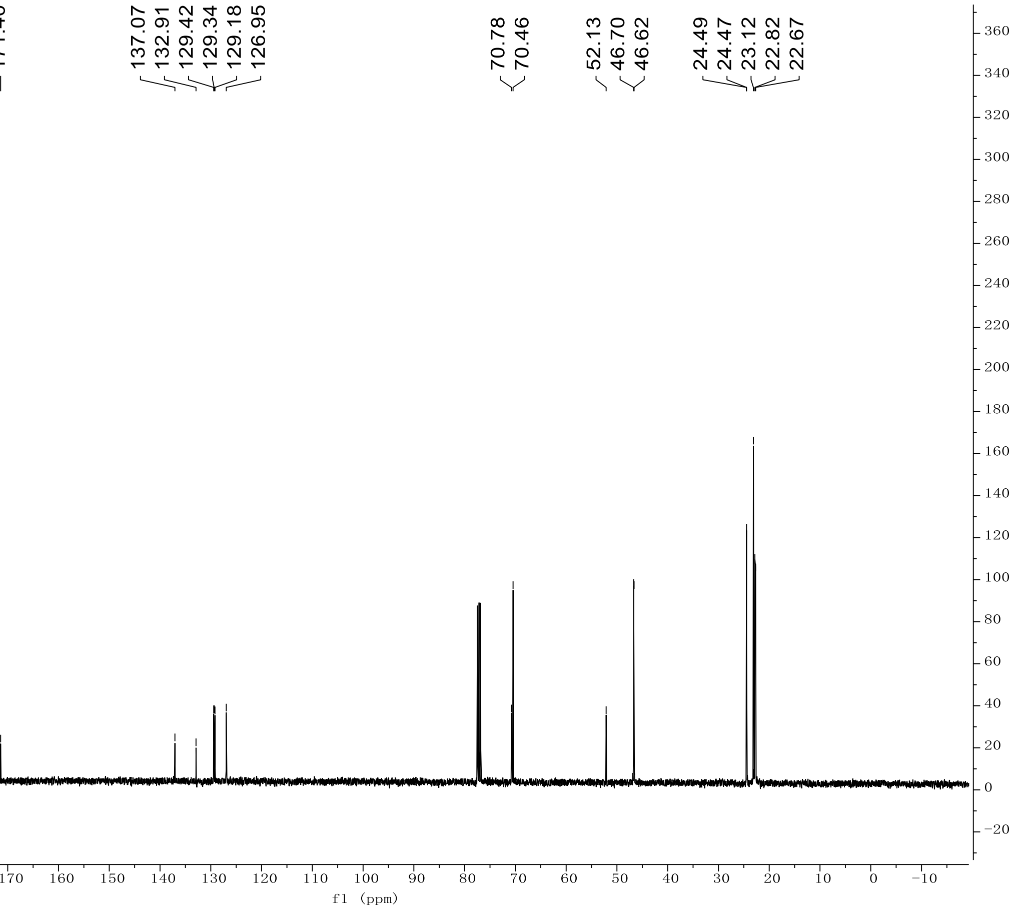

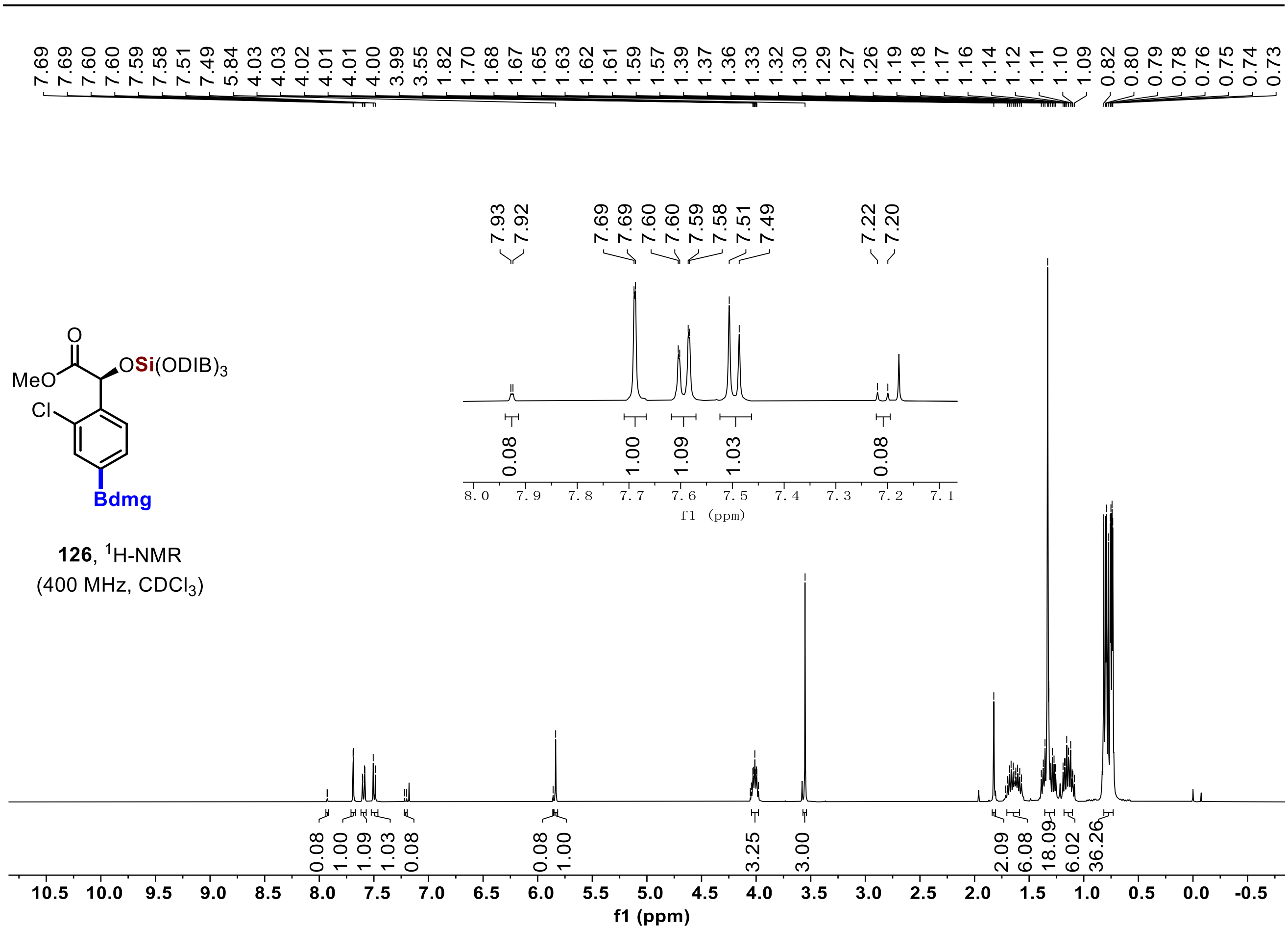

126, ^{1}H-NMR
(400 MHz, $CDCl_3$)

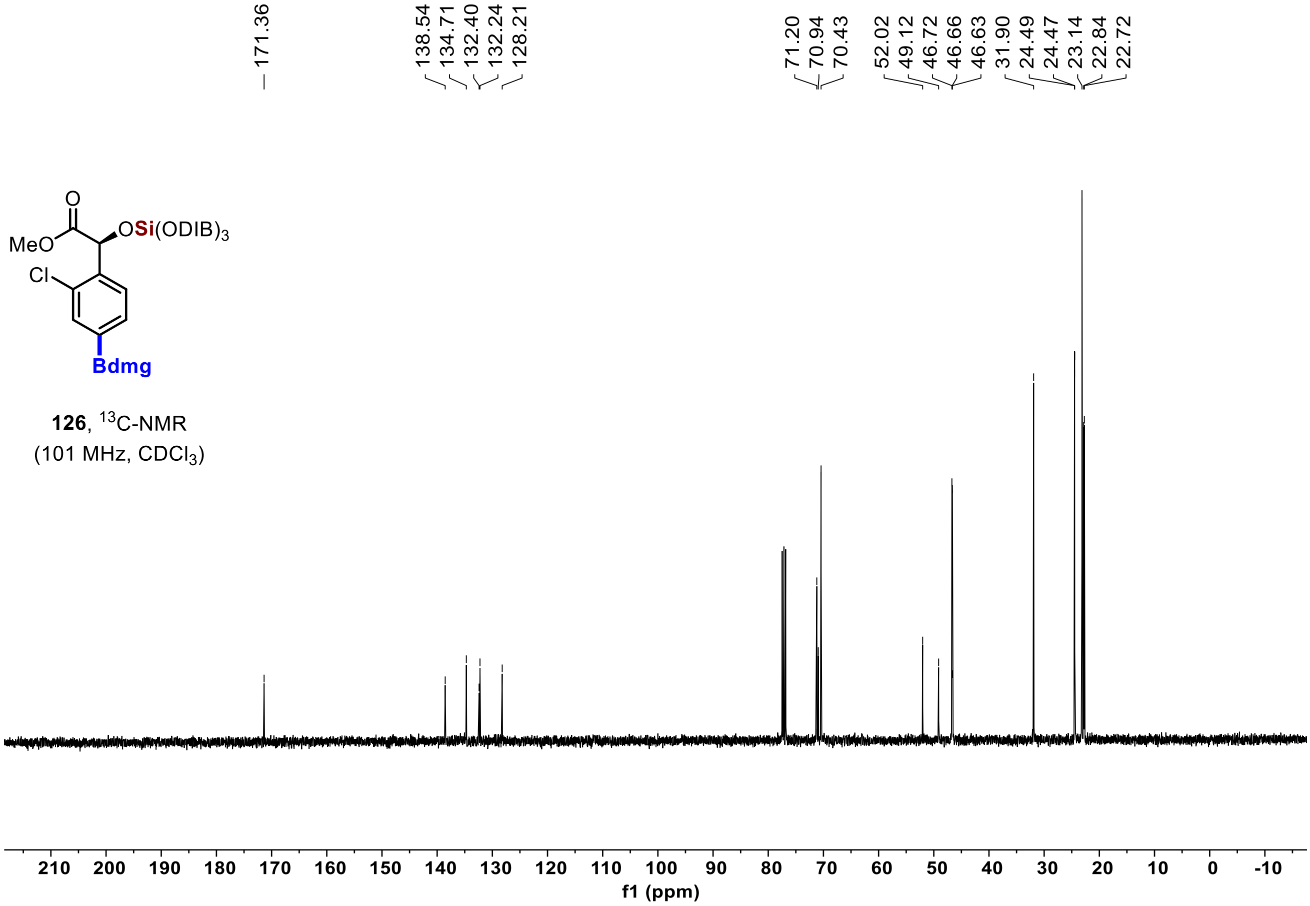

126, ^{13}C-NMR
(101 MHz, $CDCl_3$)

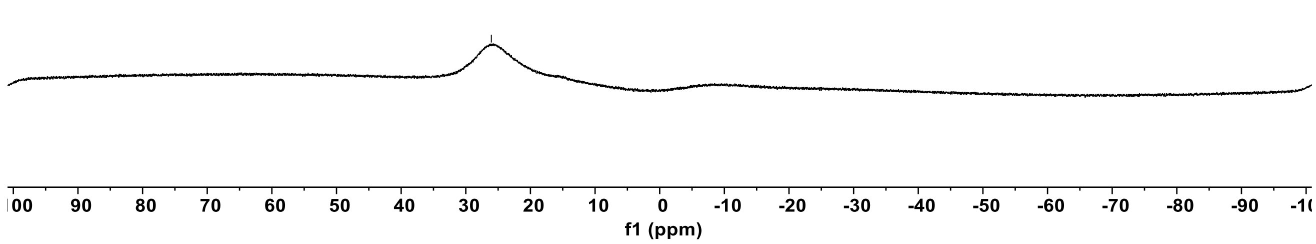

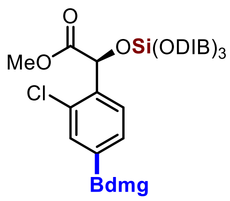

126, ^{11}B-NMR
(128 MHz, $CDCl_3$)

NOESY spectrum of 126 (400 MHz, $CDCl_3$)

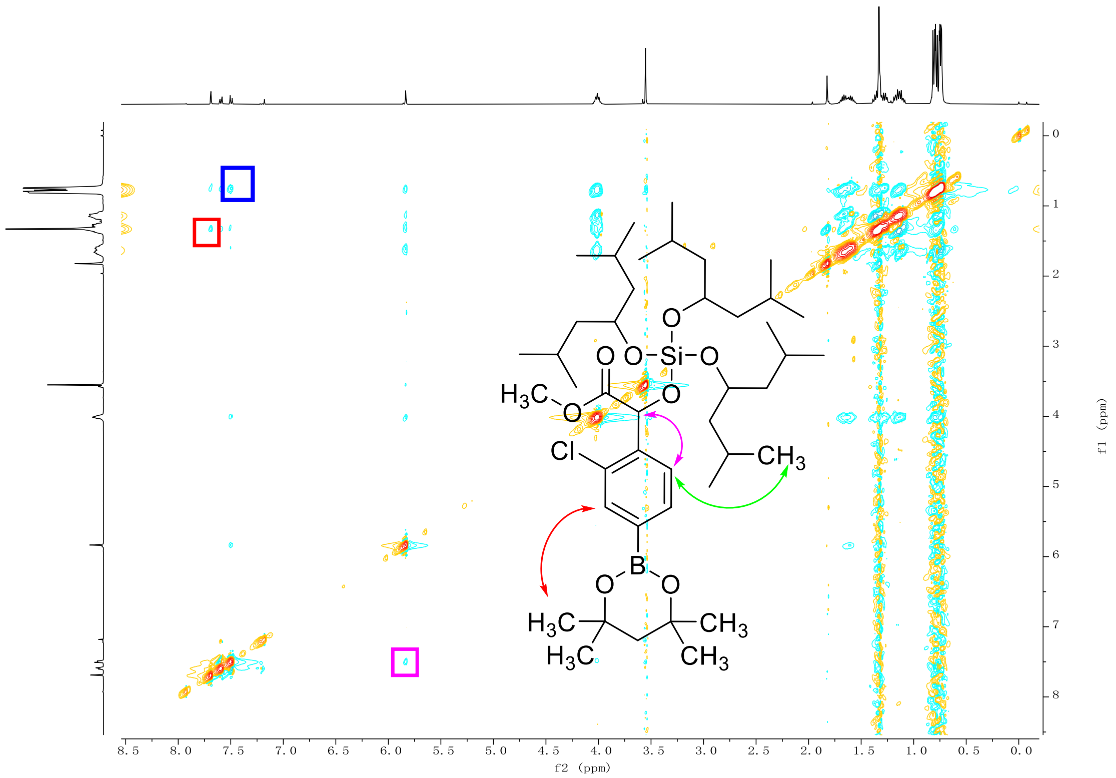

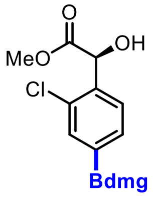

127, ^{1}H-NMR
(400 MHz, $CDCl_3$)

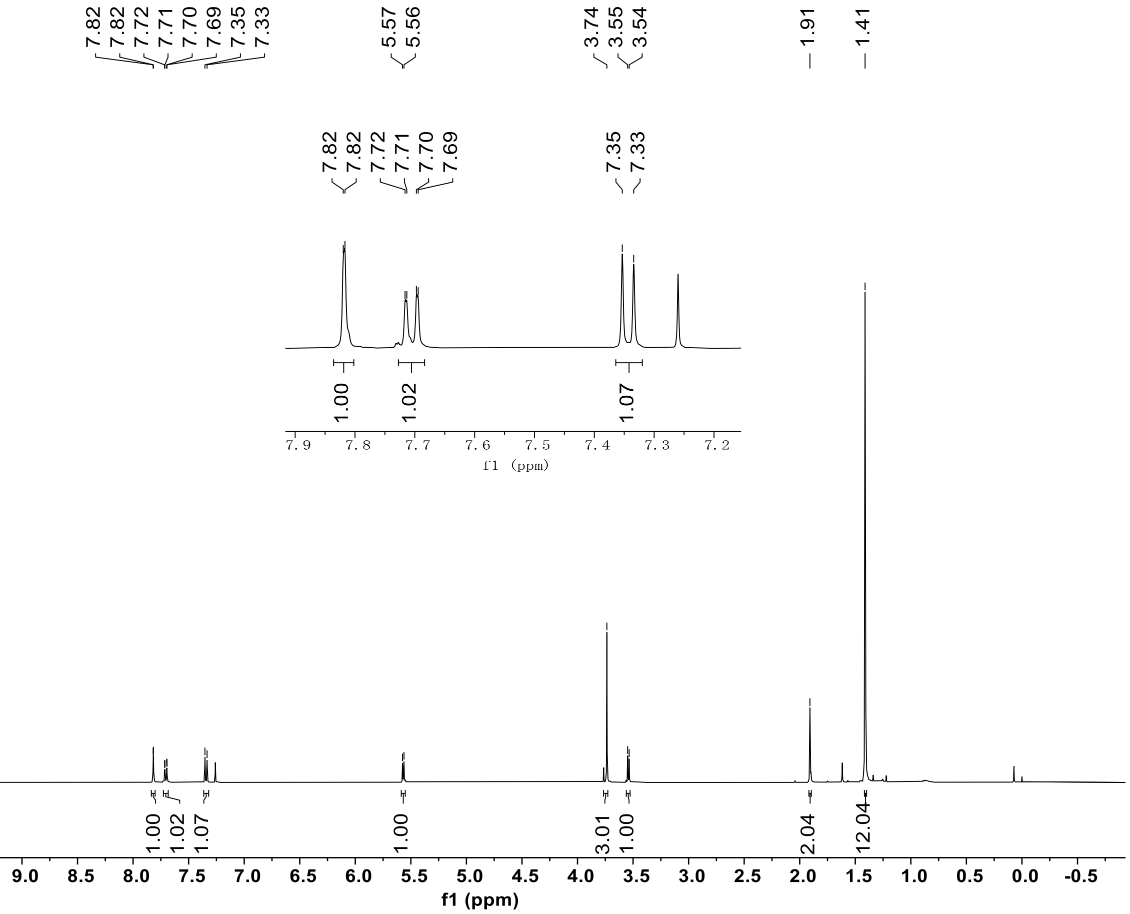

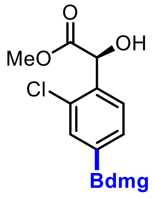

127, ^{13}C-NMR
(101 MHz, $CDCl_3$)

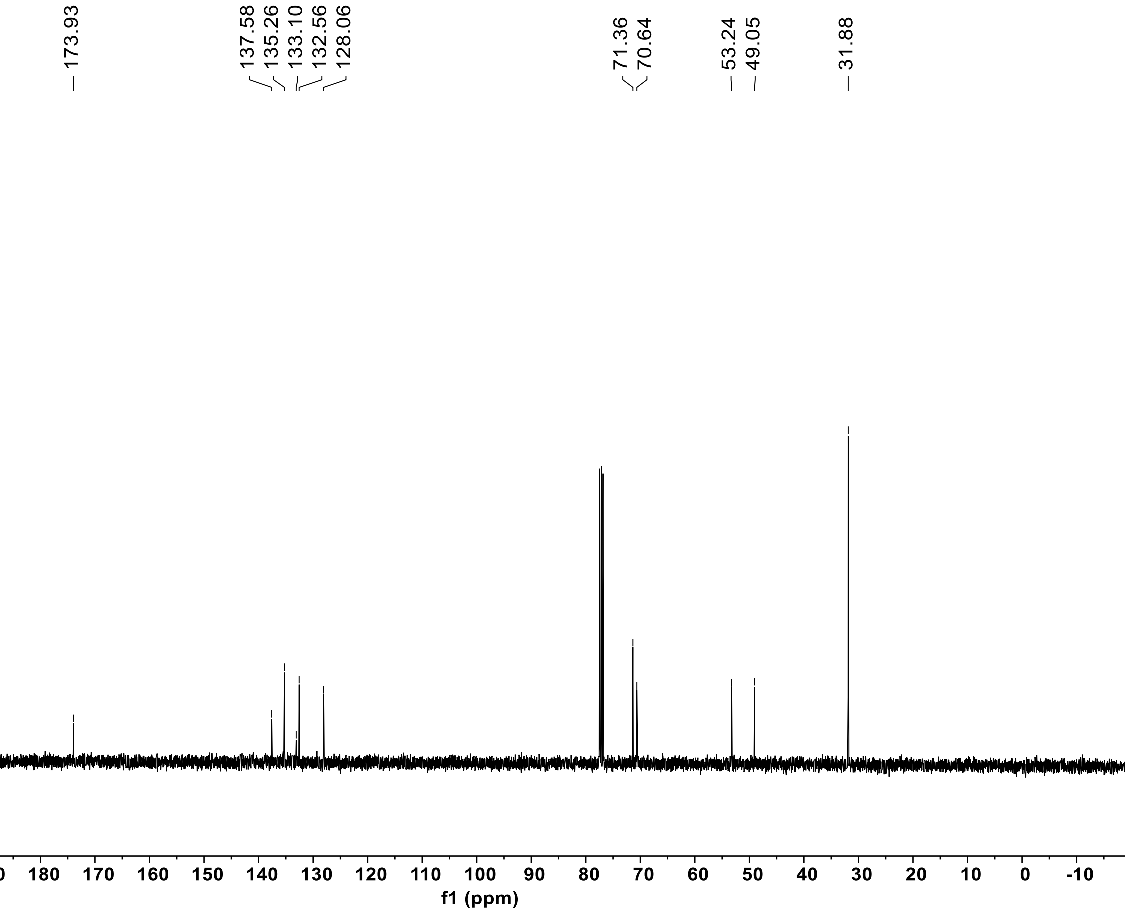

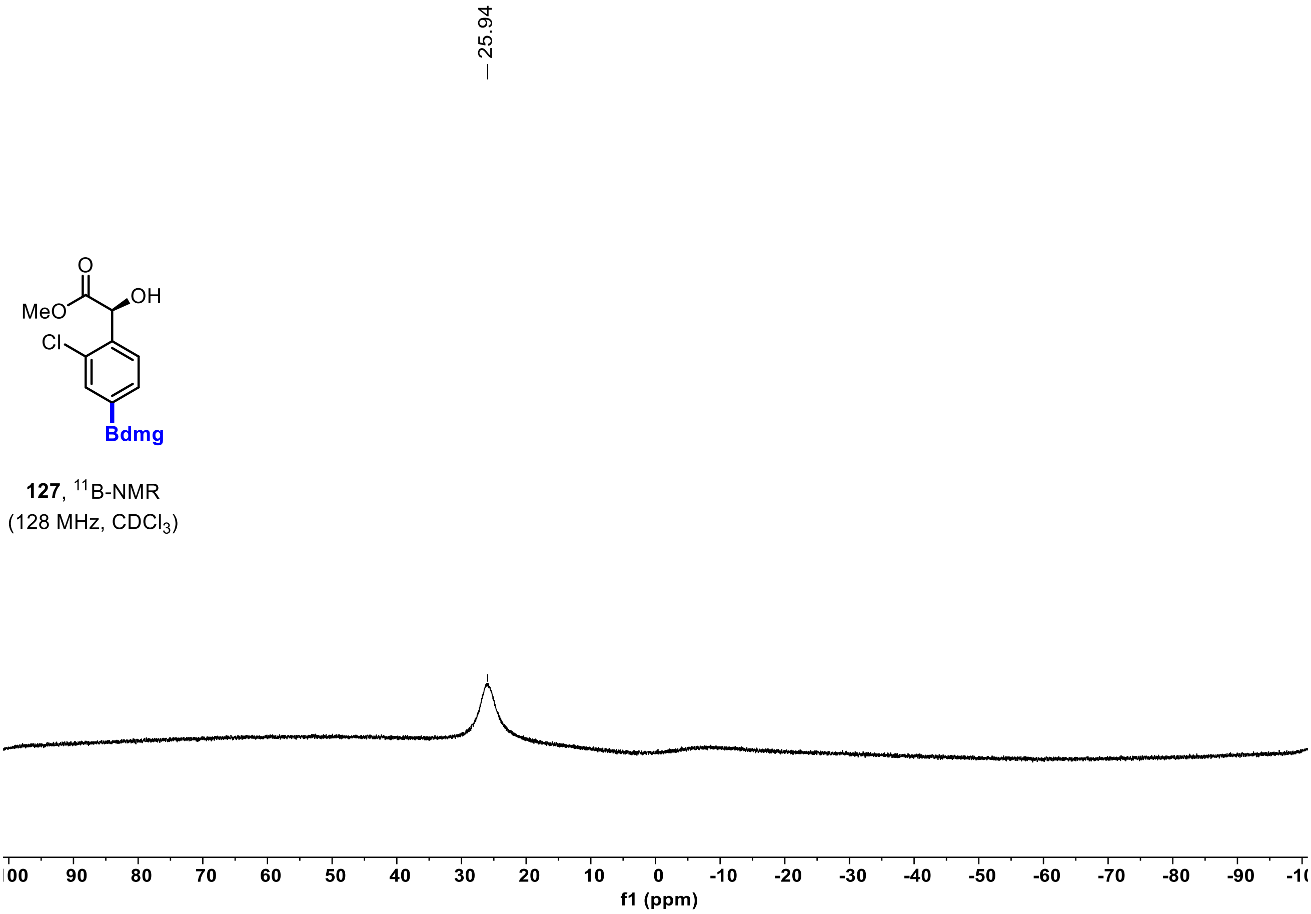

127, ^{11}B-NMR
(128 MHz, $CDCl_3$)

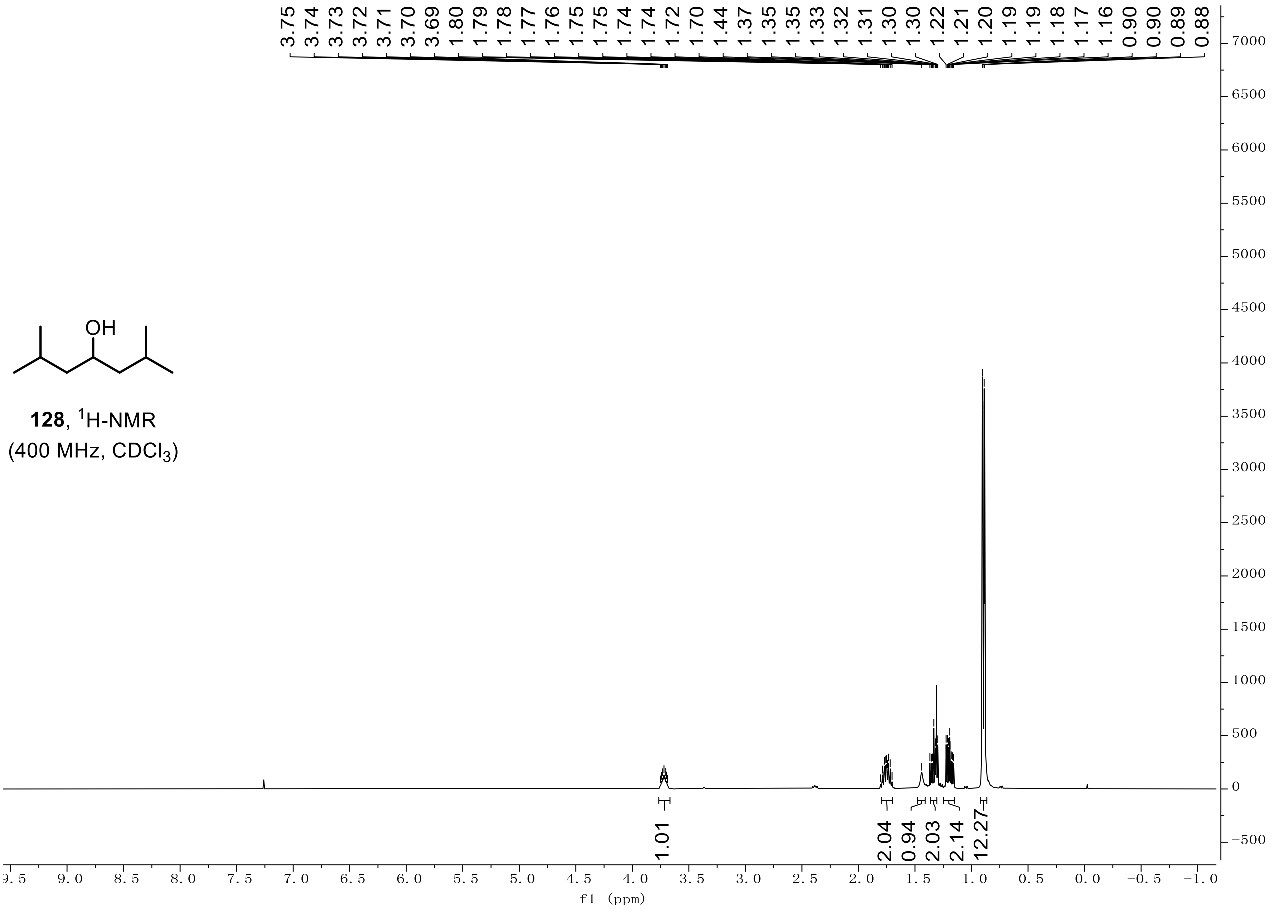

128, ^{1}H-NMR
(400 MHz, $CDCl_3$)

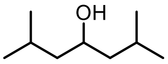

128, ^{13}C-NMR
(101 MHz, $CDCl_3$)

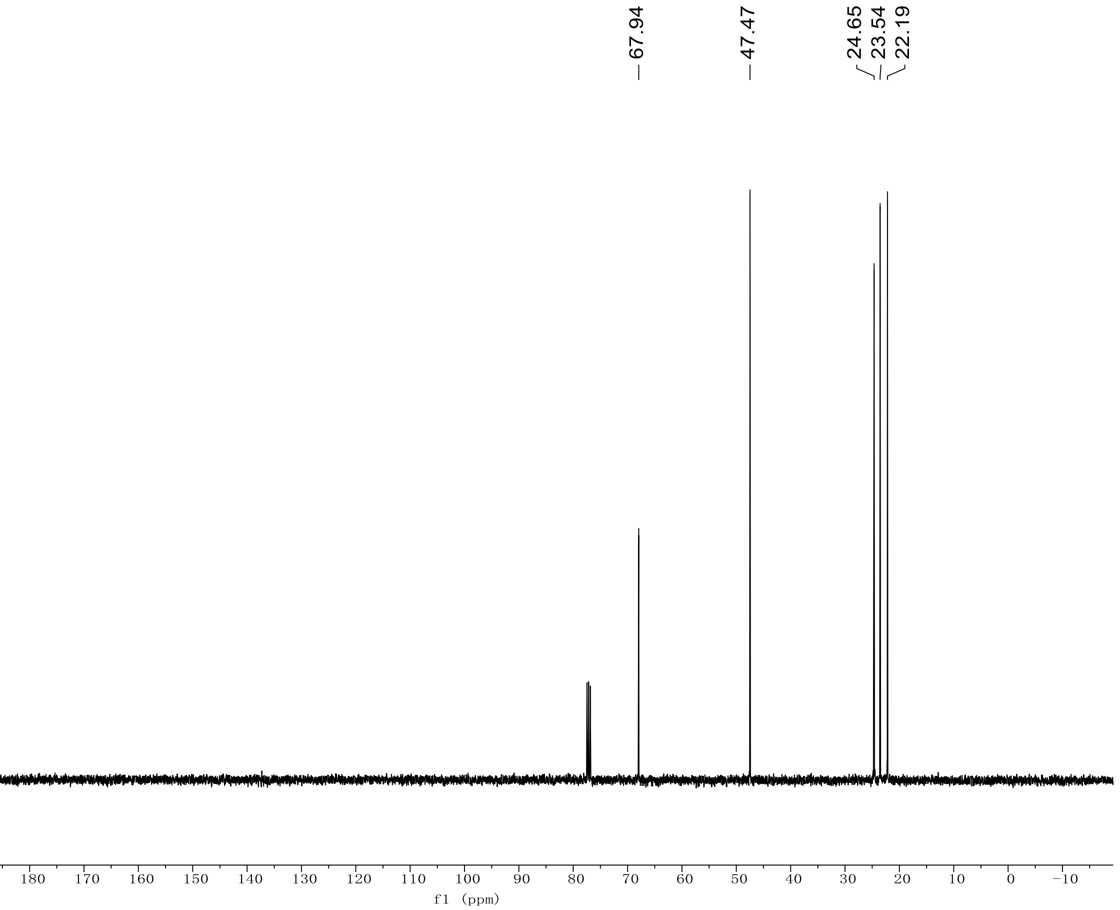

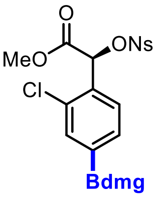

129, ^{1}H-NMR
(400 MHz, $CDCl_3$)

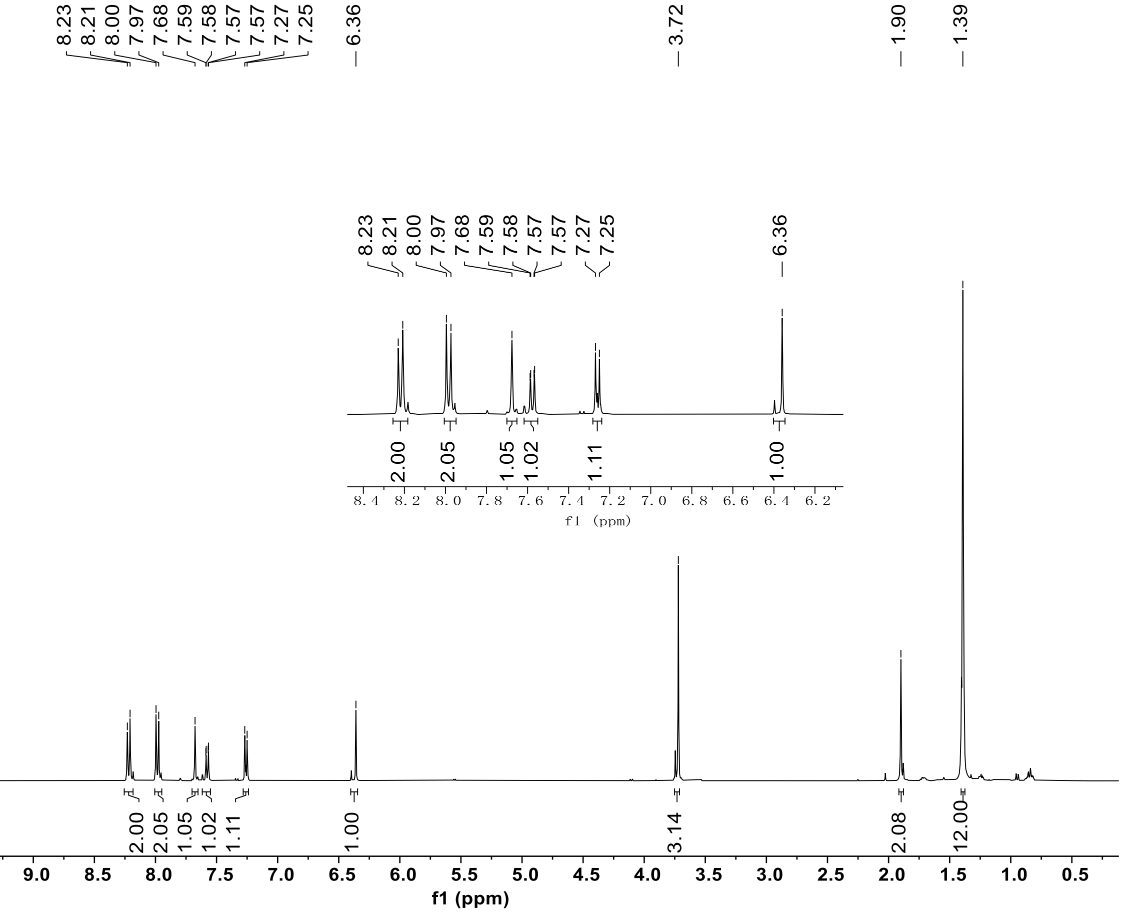

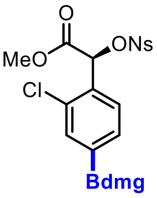

129, ^{13}C-NMR
(101 MHz, $CDCl_3$)

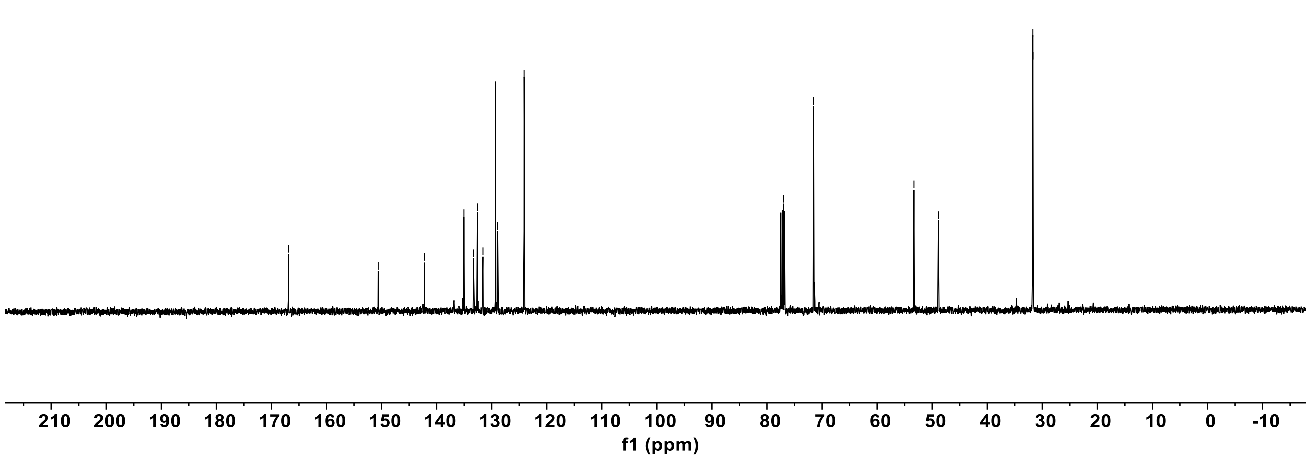

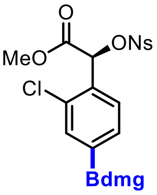

129, ^{11}B-NMR
(128 MHz, $CDCl_3$)

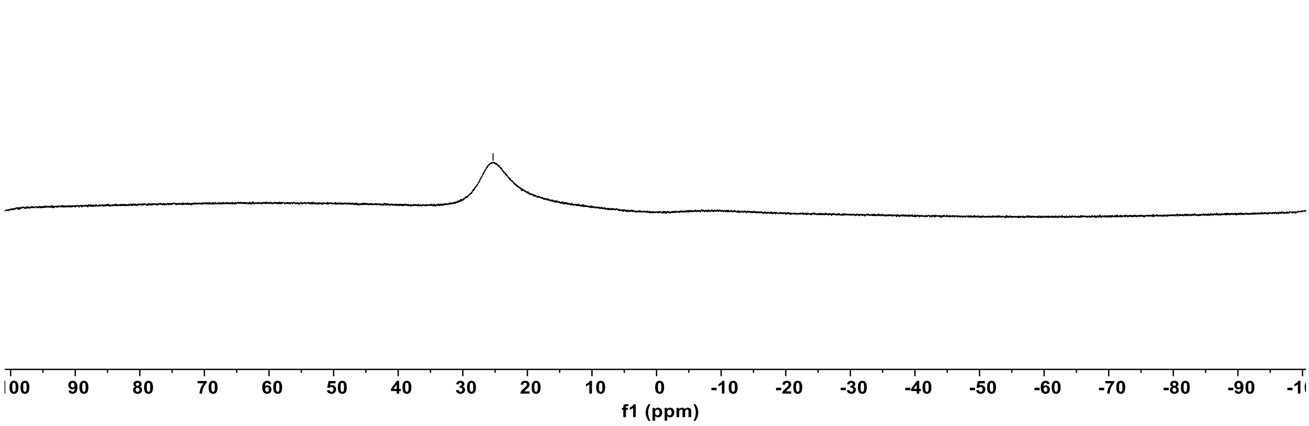

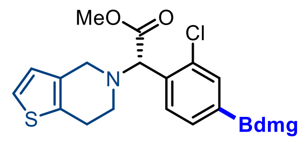

130, ^{1}H-NMR
(400 MHz, $CDCl_3$)

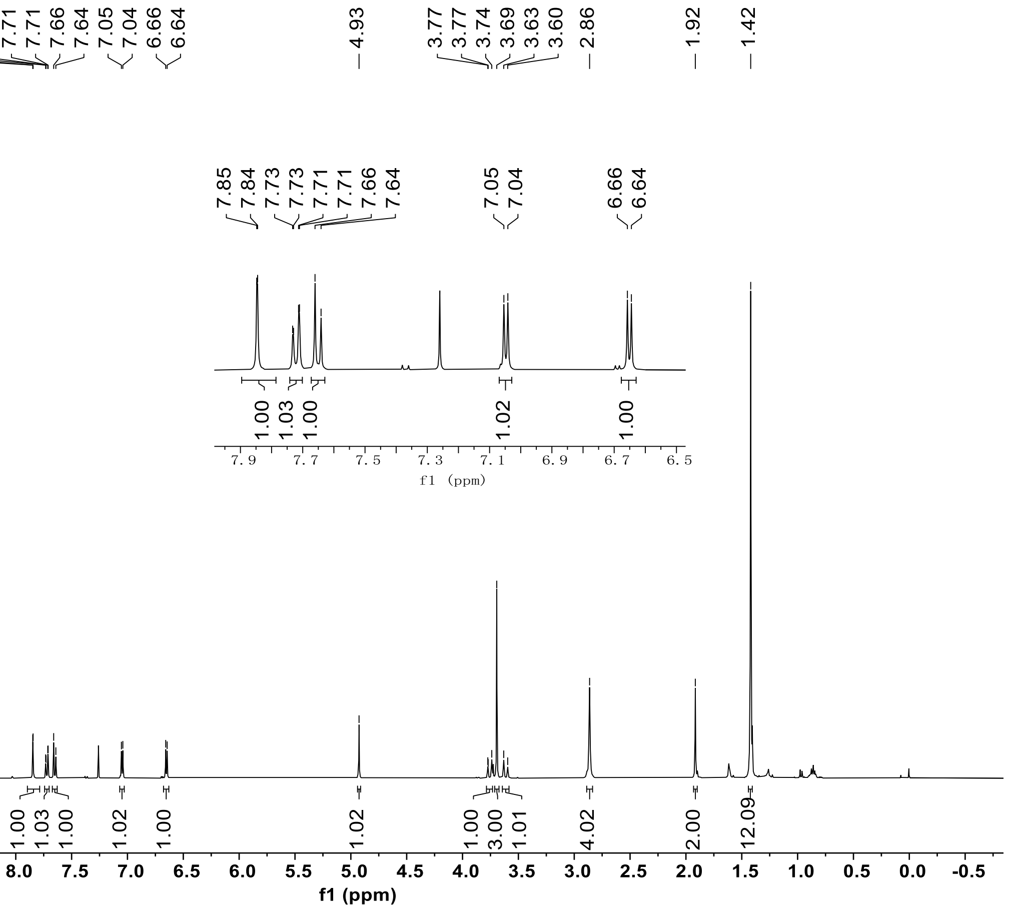

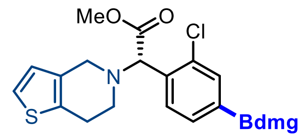

130, ^{13}C-NMR
(101 MHz, $CDCl_3$)

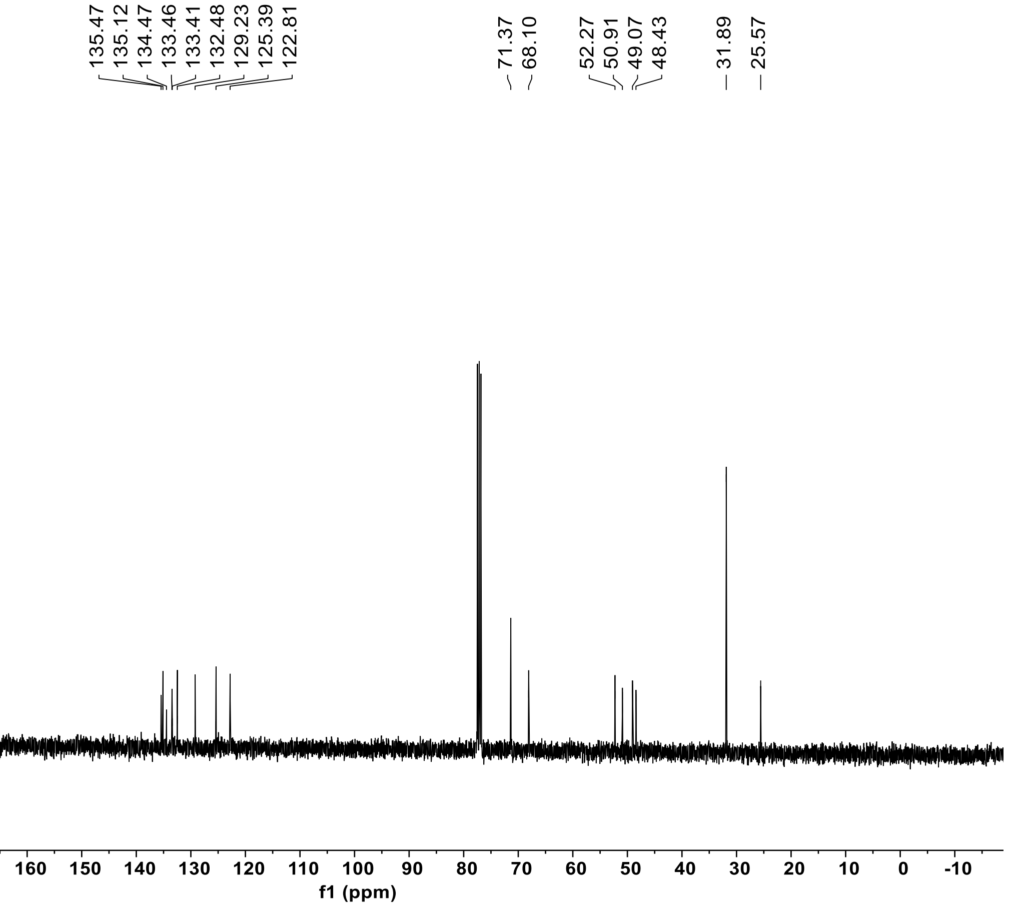

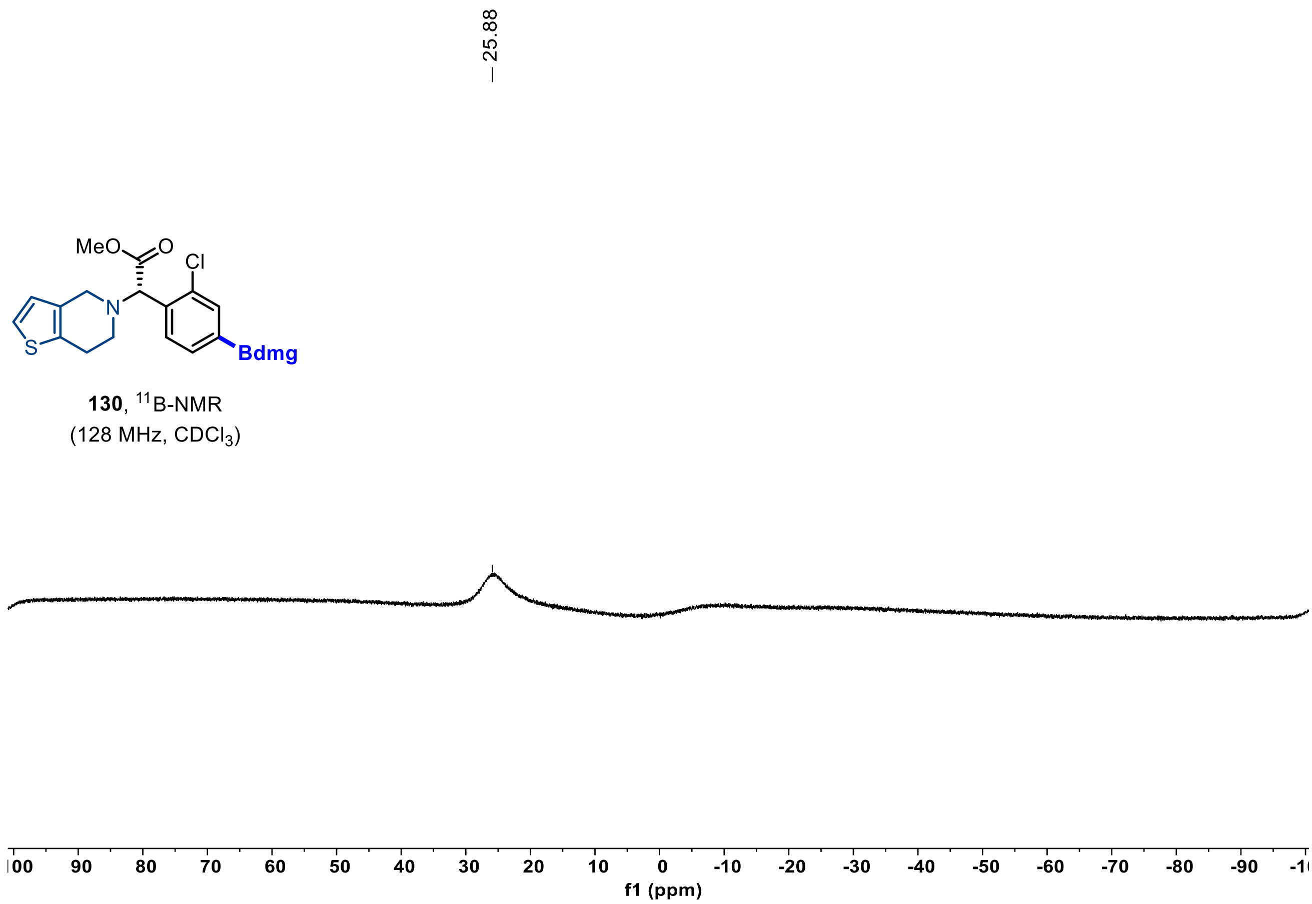

130, ^{11}B-NMR
(128 MHz, $CDCl_3$)

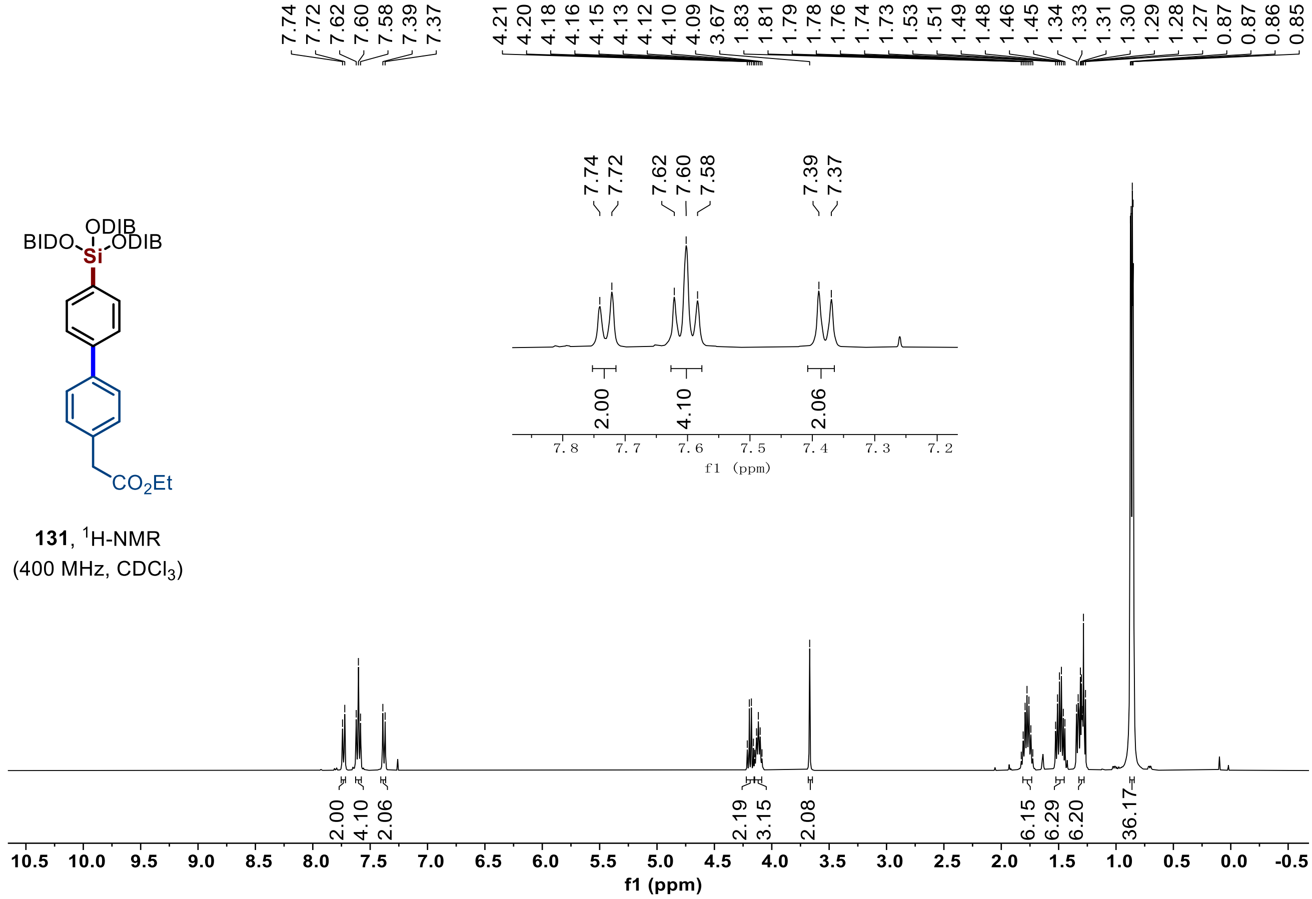

131, ^{1}H-NMR
(400 MHz, $CDCl_3$)

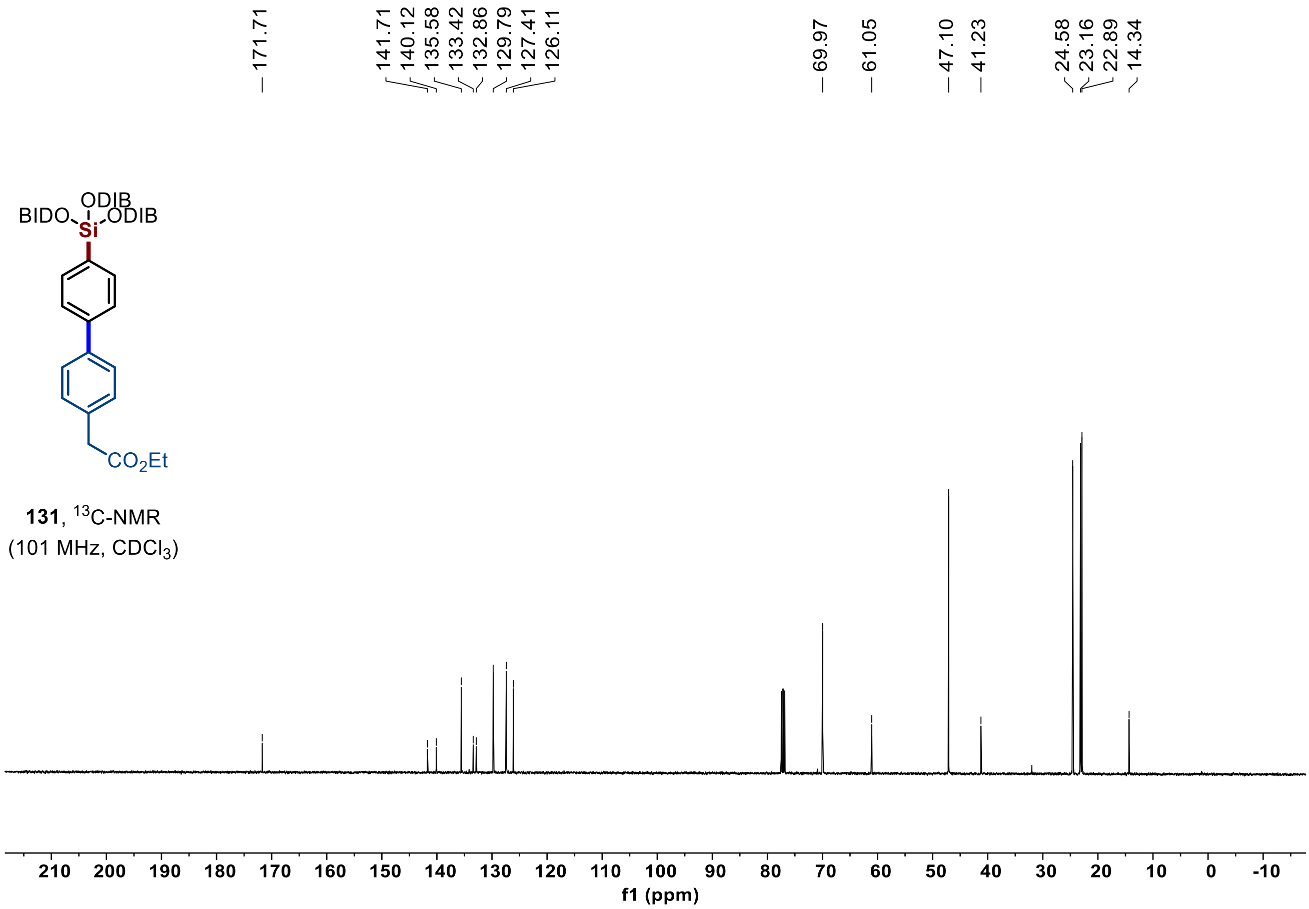
171.71
141.71
140.12
135.58
133.42
132.86
129.79
127.41
126.11
69.97
61.05
47.10
41.23
24.58
23.16
22.89
14.34
BIDO
ODIB
ODIB
Si
CO2Et
131, 13C-NMR
(101 MHz, CDCl3)
f1 (ppm)

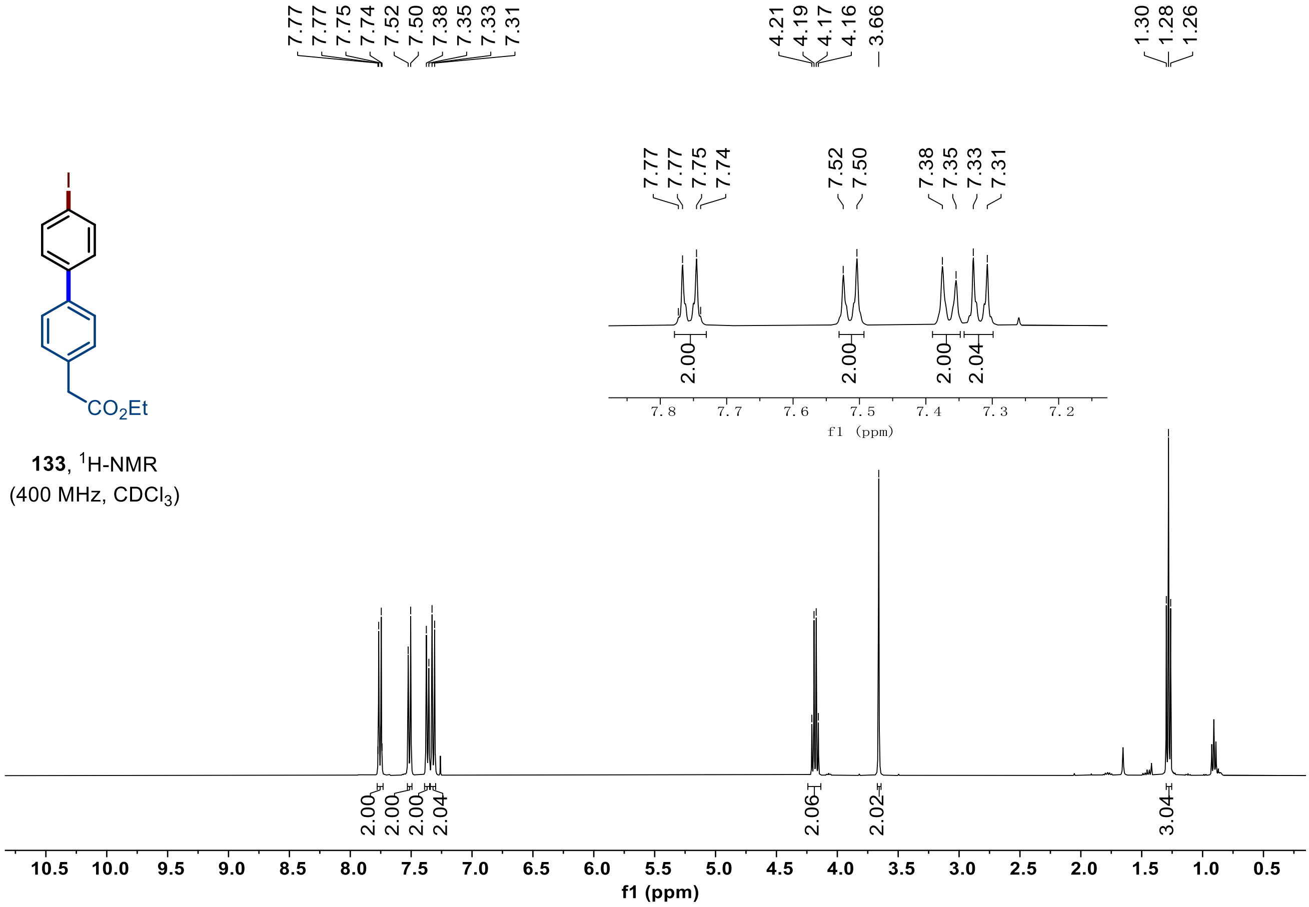
7.77
7.77
7.75
7.74
7.52
7.50
7.38
7.35
7.33
7.31
4.21
4.19
4.17
4.16
3.66
1.30
1.28
1.26
I
CO2Et
133, 1H-NMR
(400 MHz, CDCl3)
2.00
2.00
2.00
2.04
2.06
2.02
3.04
f1 (ppm)

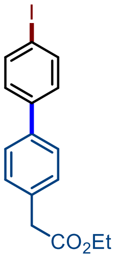

133, ^{13}C-NMR
(101 MHz, $CDCl_3$)

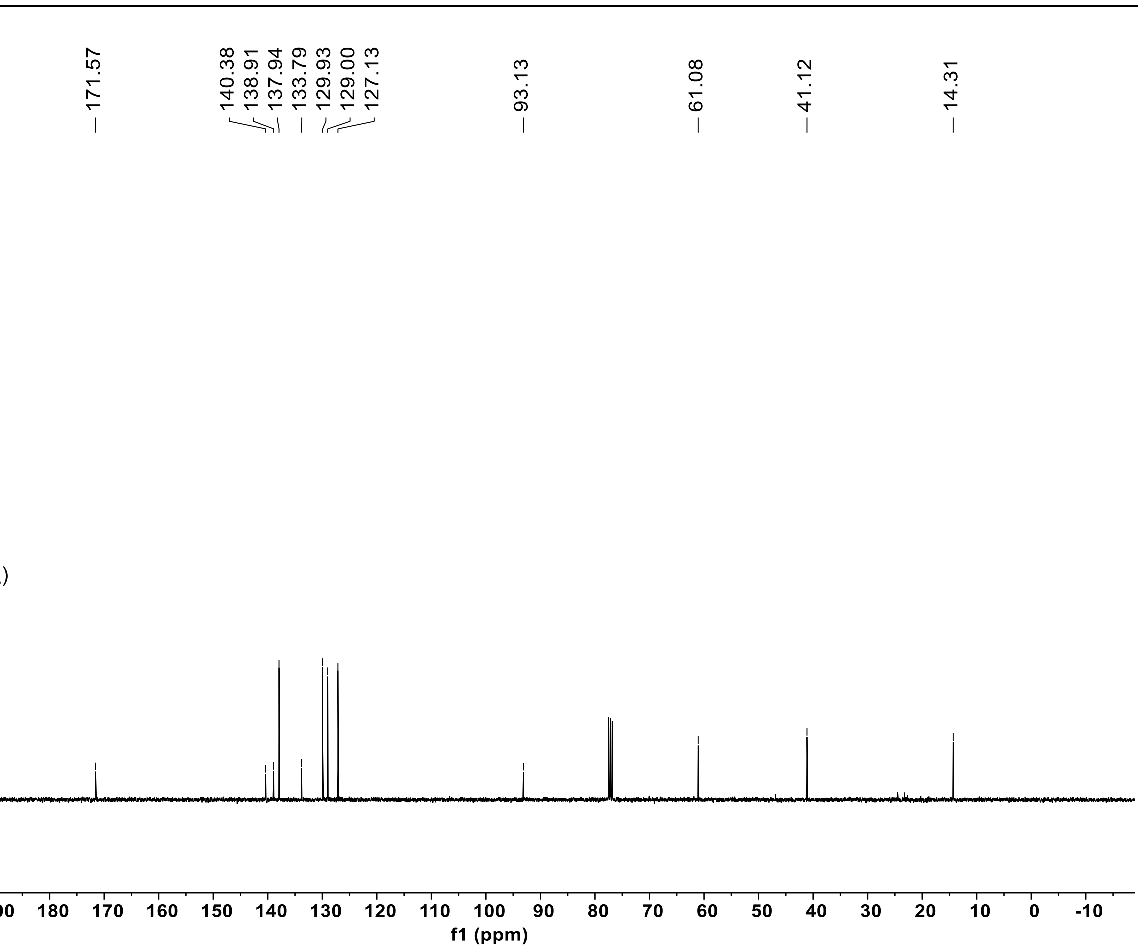

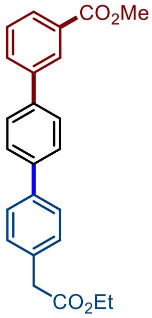

134, ^{1}H-NMR
(400 MHz, $CDCl_3$)

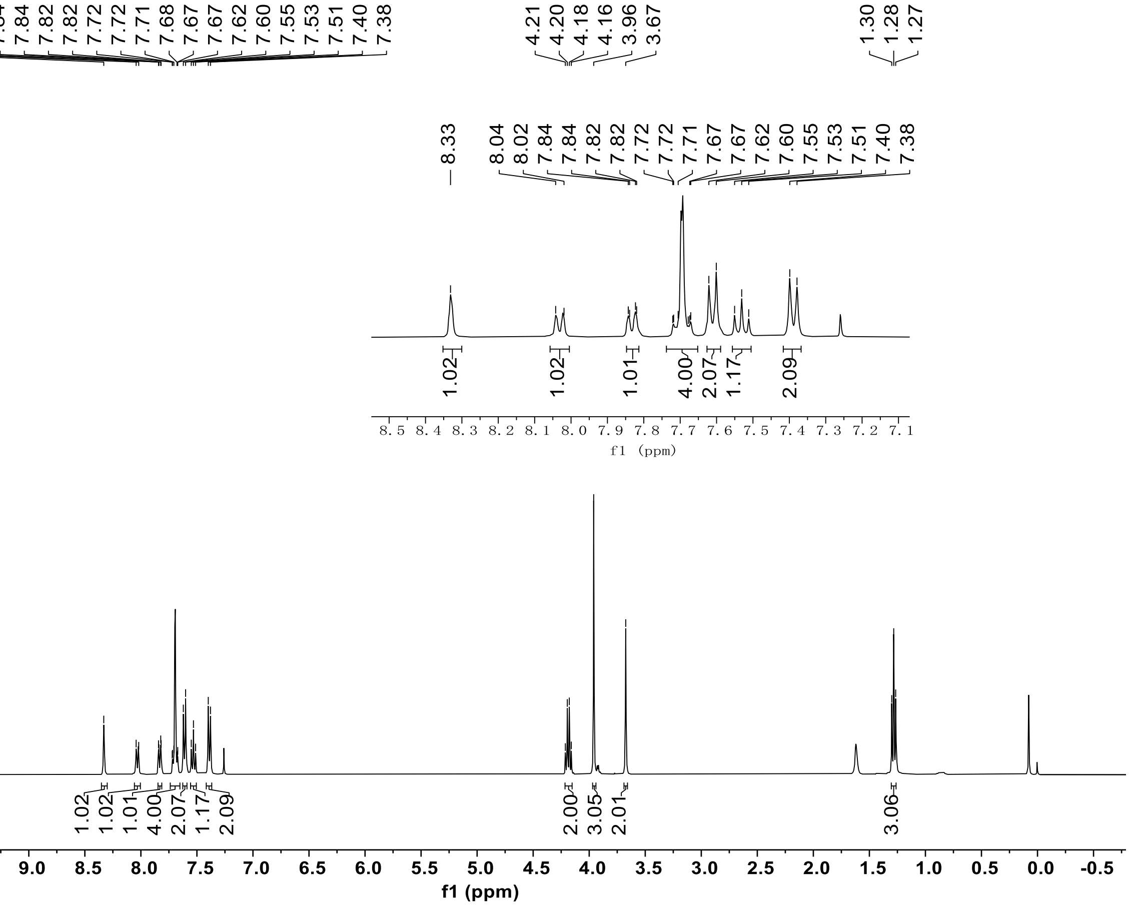

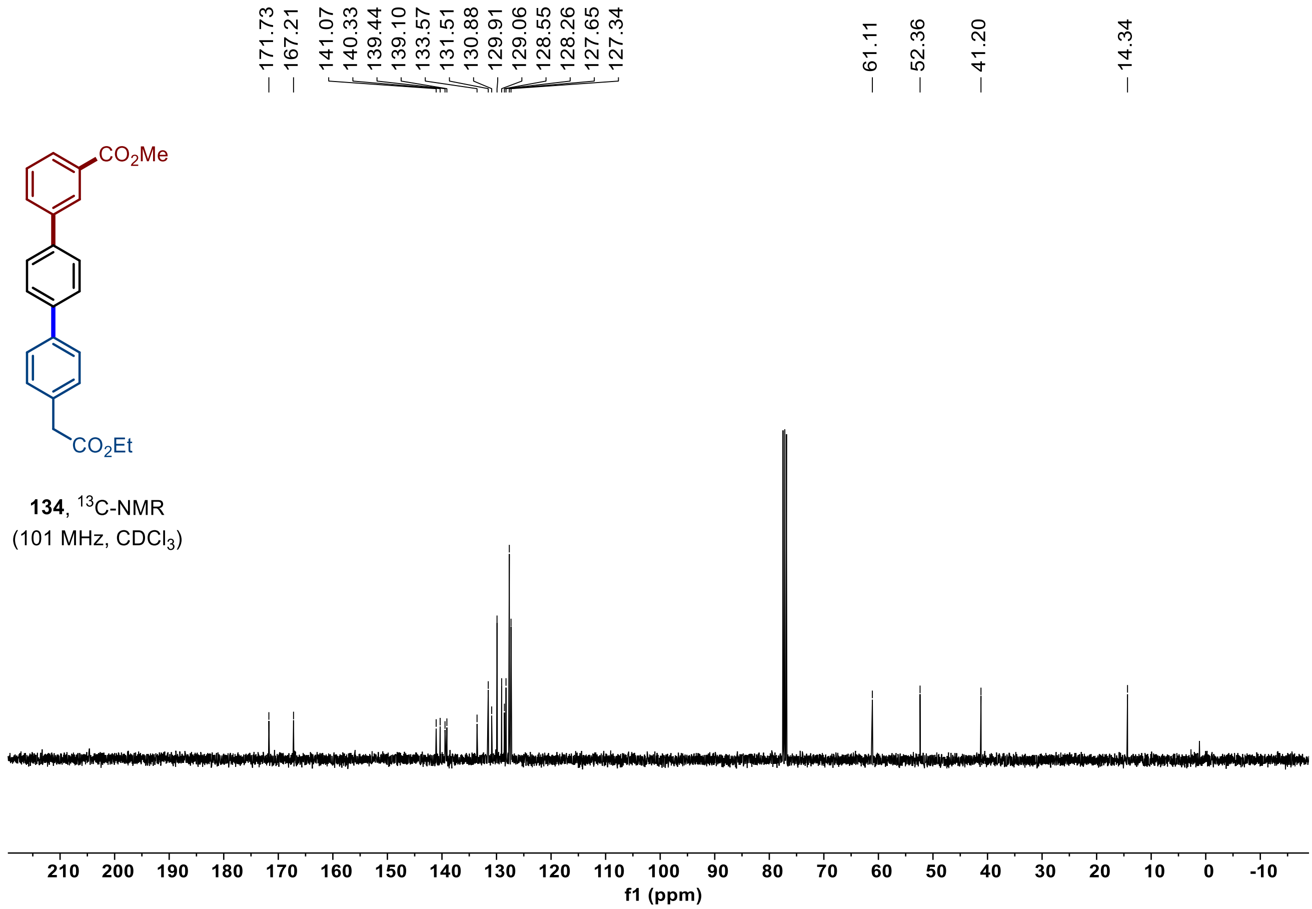

134, ^{13}C-NMR
(101 MHz, $CDCl_3$)

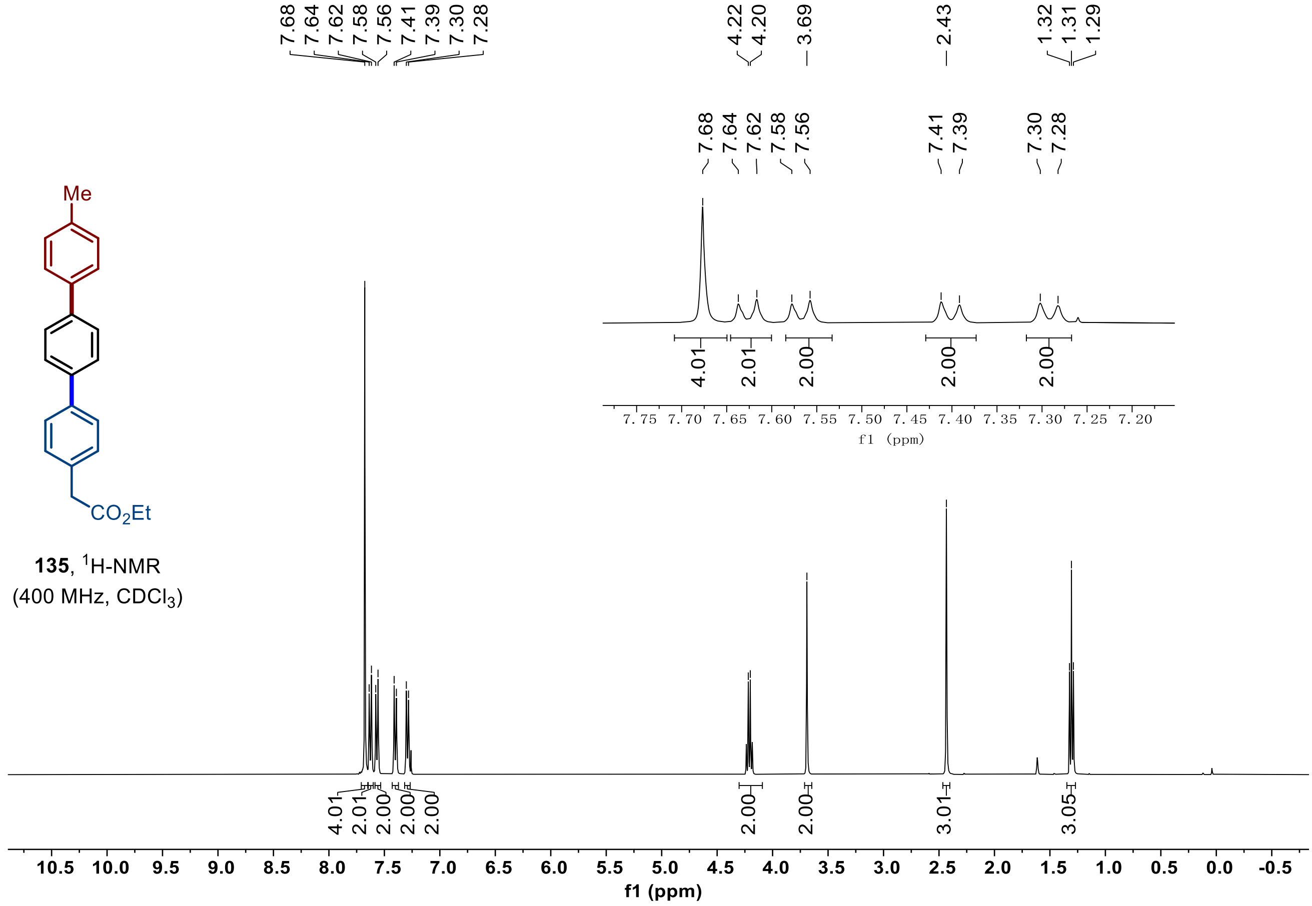

135, ^{1}H-NMR
(400 MHz, $CDCl_3$)

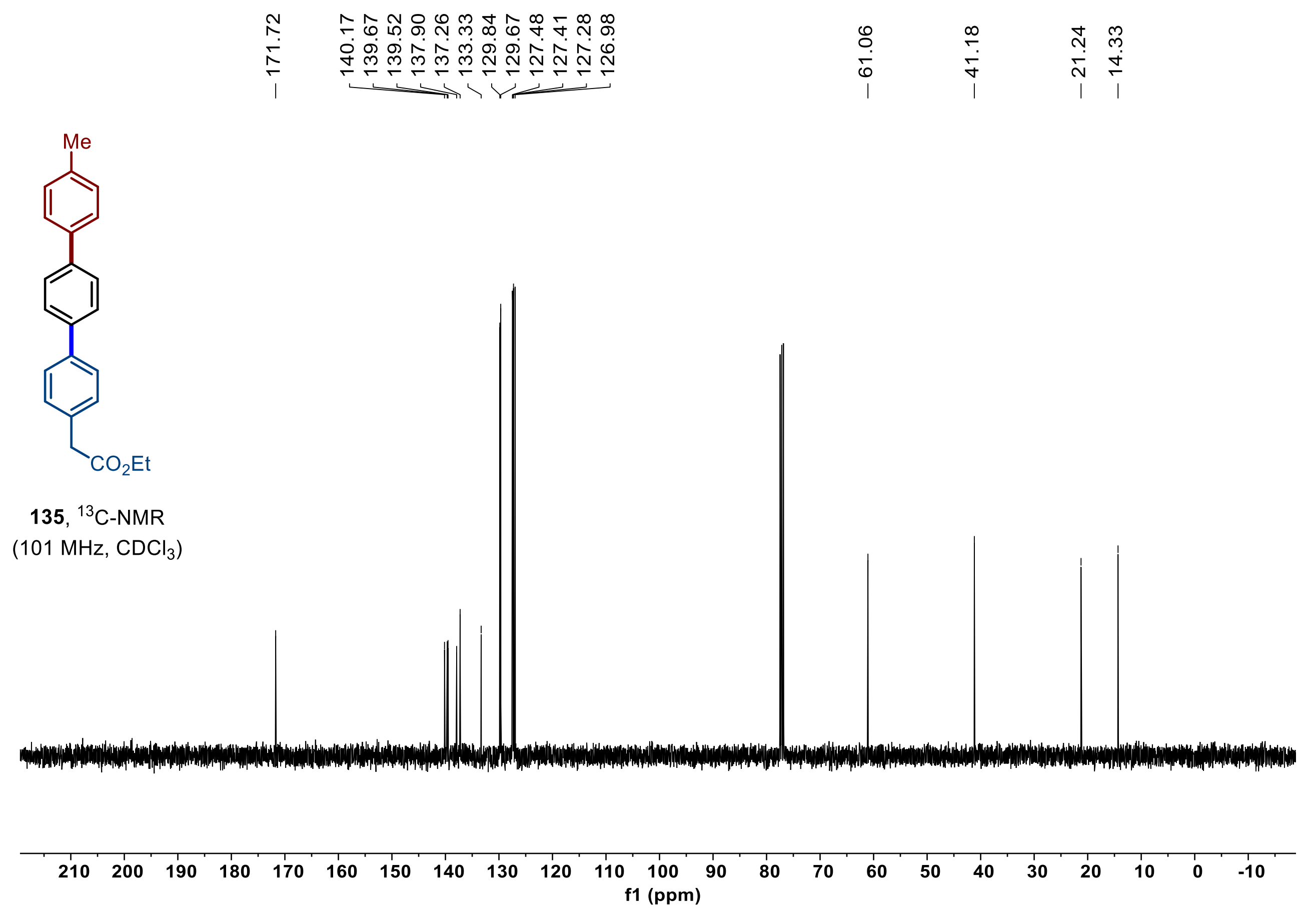

135, ^{13}C-NMR
(101 MHz, $CDCl_3$)

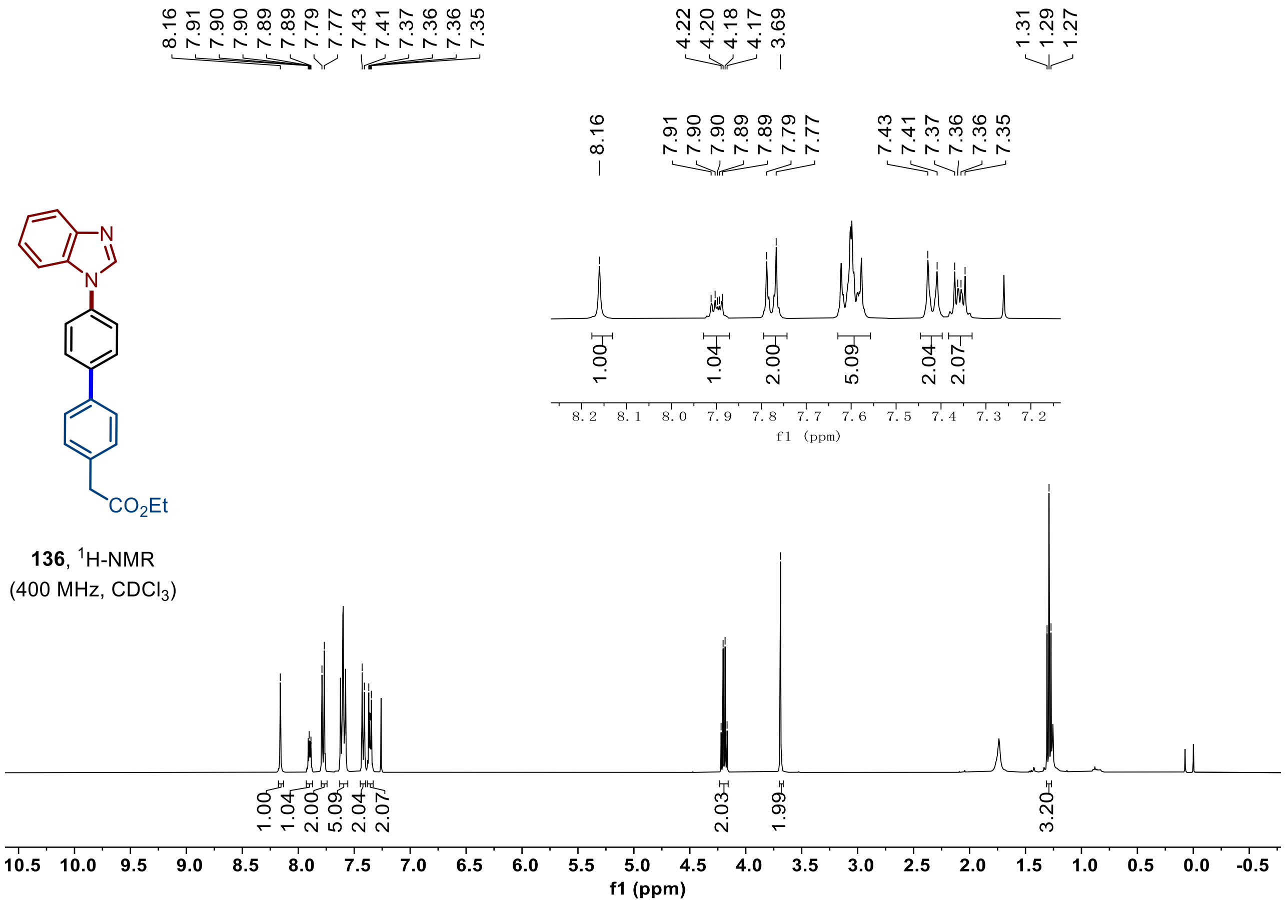

136, ^{1}H-NMR
(400 MHz, $CDCl_3$)

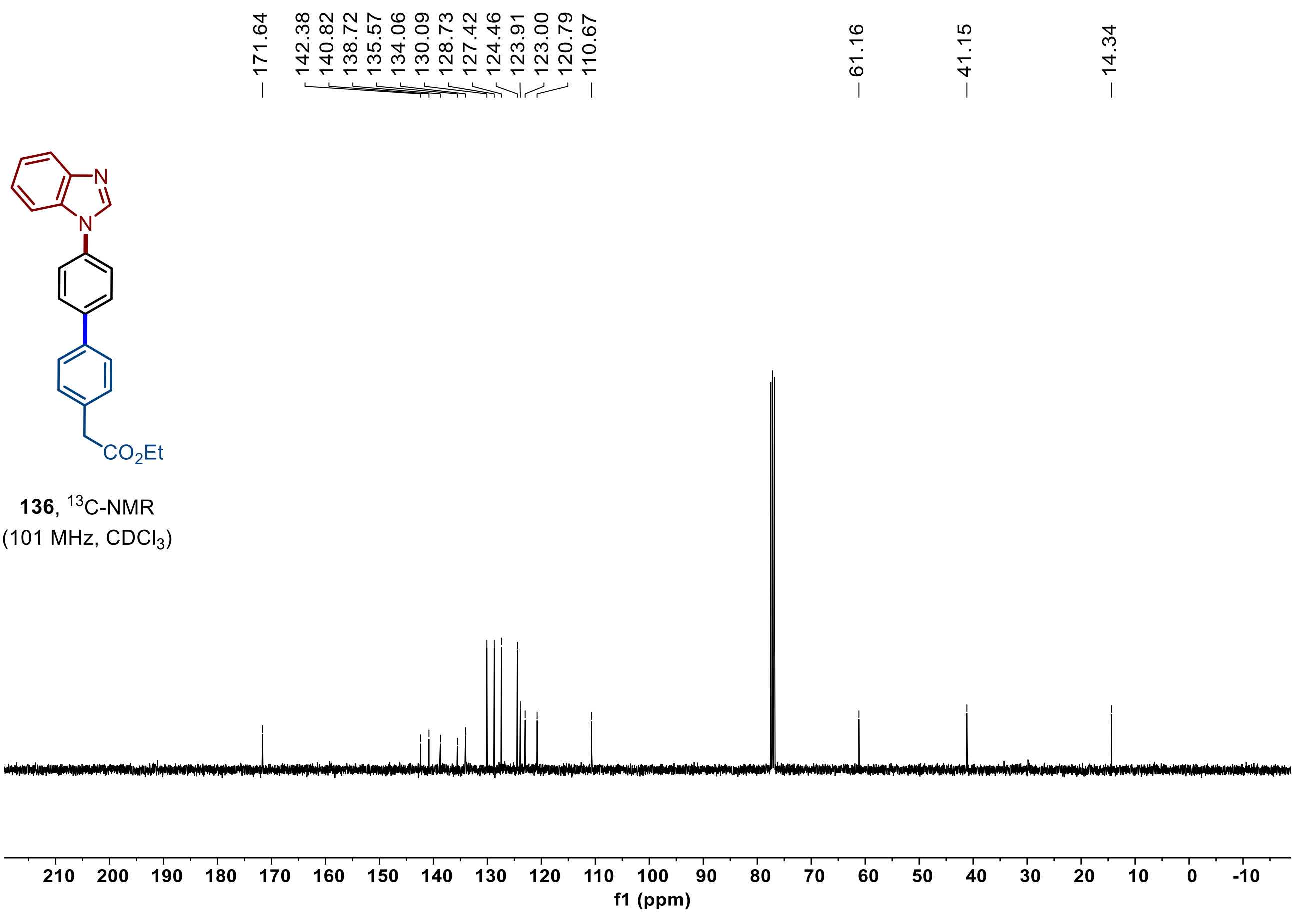

136, ^{13}C-NMR
(101 MHz, $CDCl_3$)

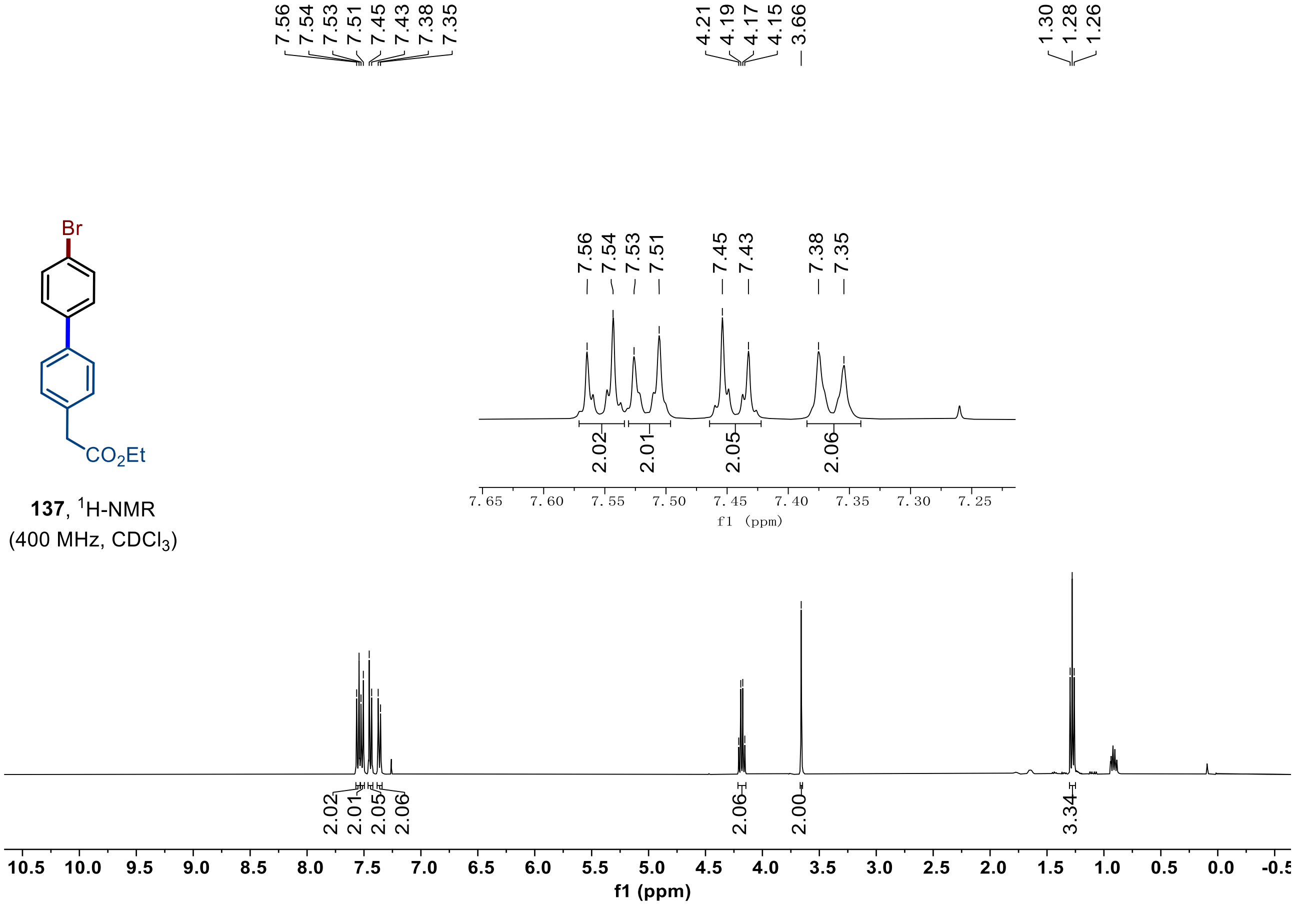

137, ^{1}H-NMR
(400 MHz, $CDCl_3$)

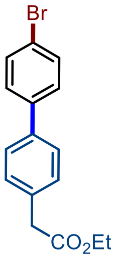

137, ^{13}C-NMR
(101 MHz, $CDCl_3$)

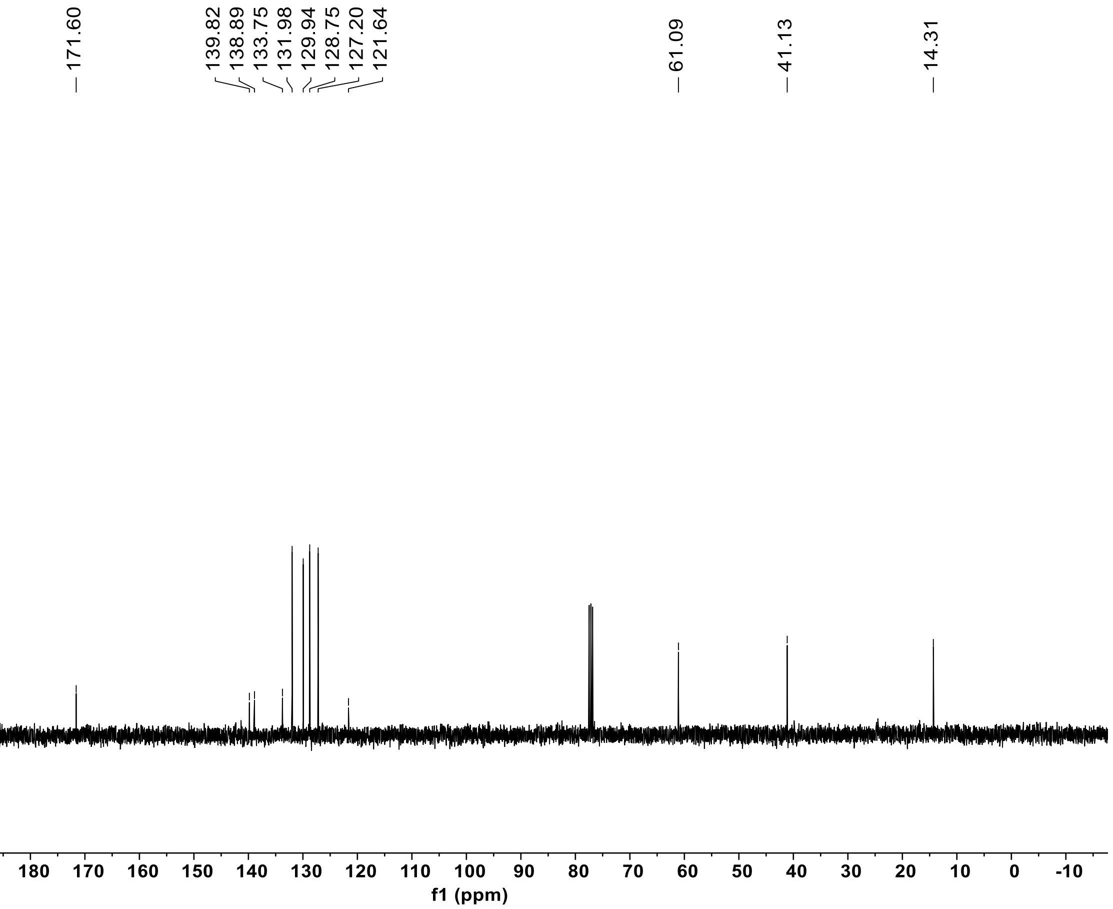

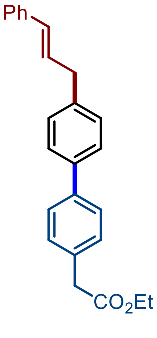

138, ^{1}H-NMR
(400 MHz, $CDCl_3$)

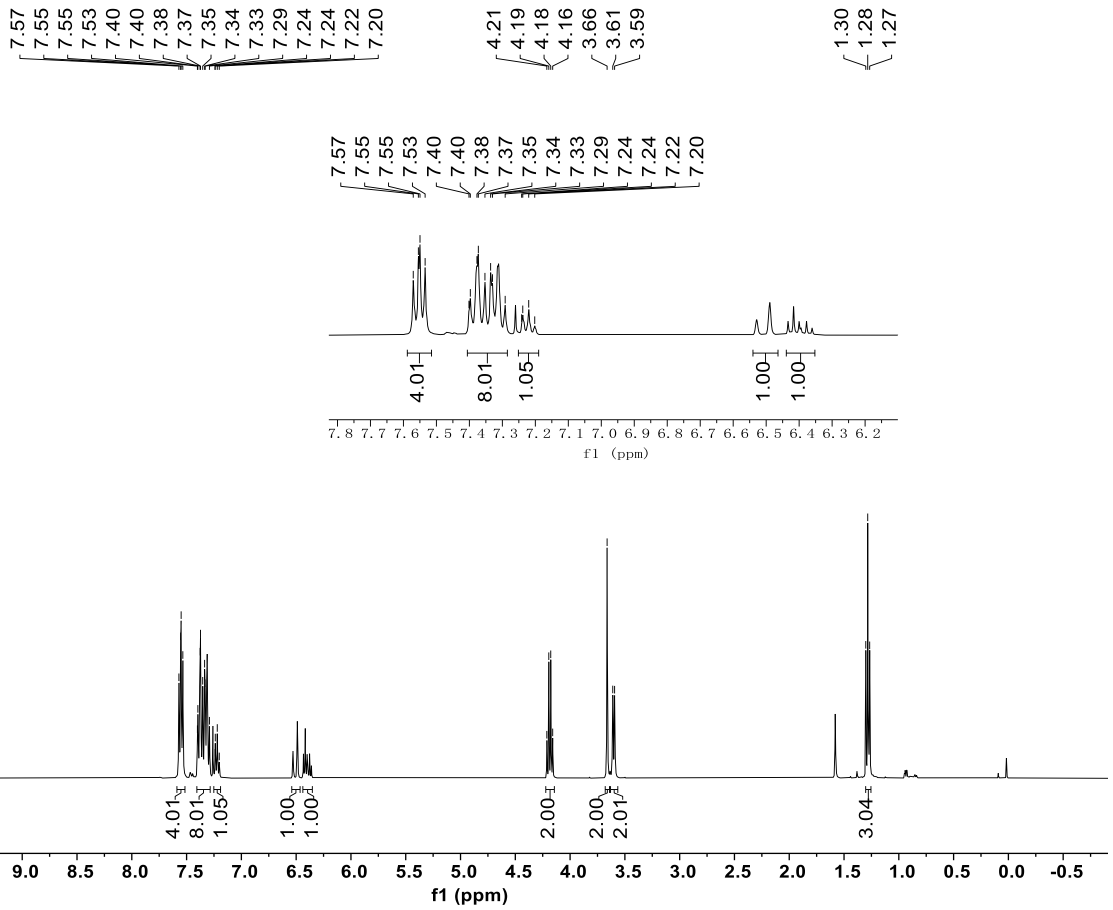

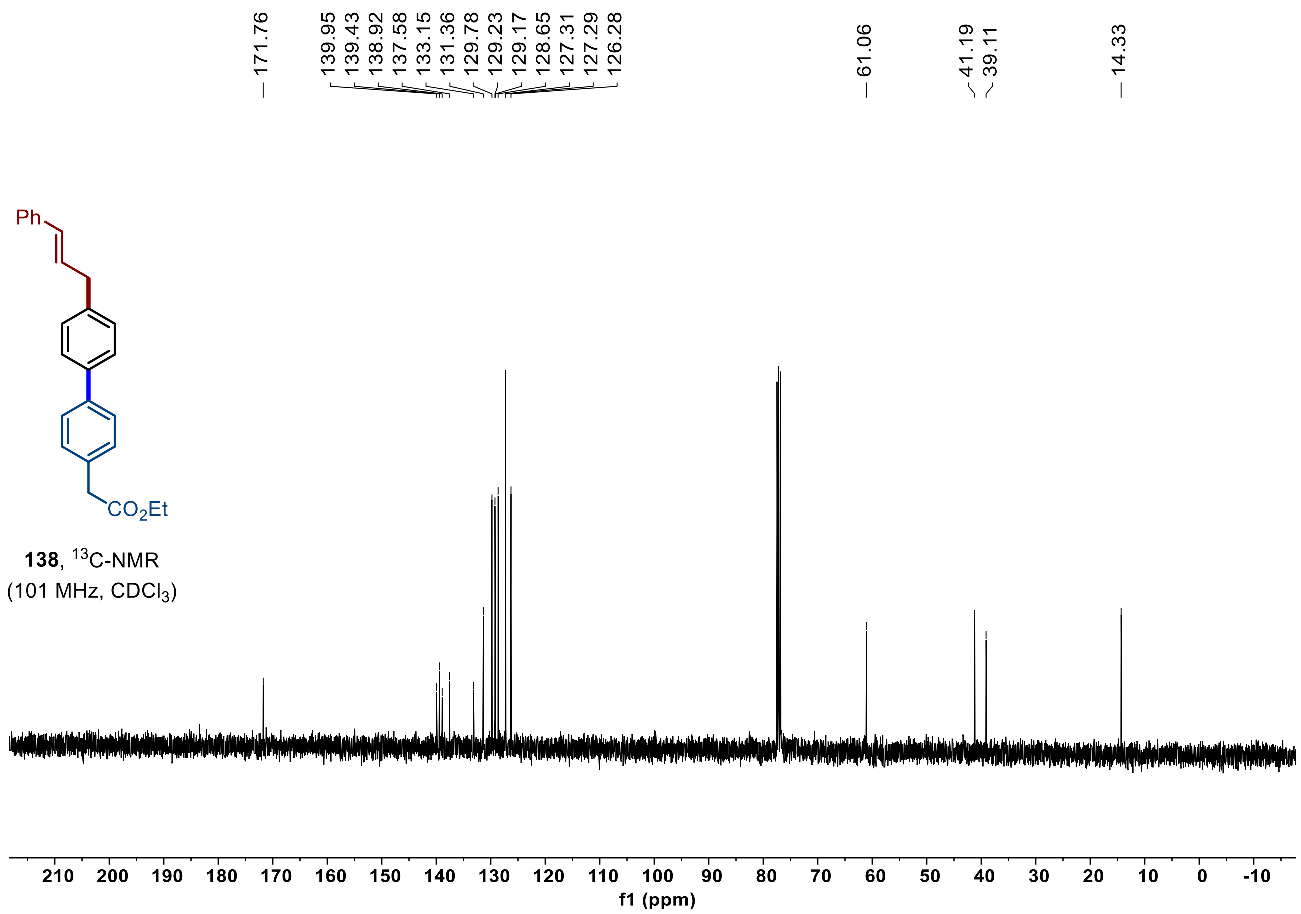
Ph
CO2Et
138, 13C-NMR
(101 MHz, CDCl3)
f1 (ppm)

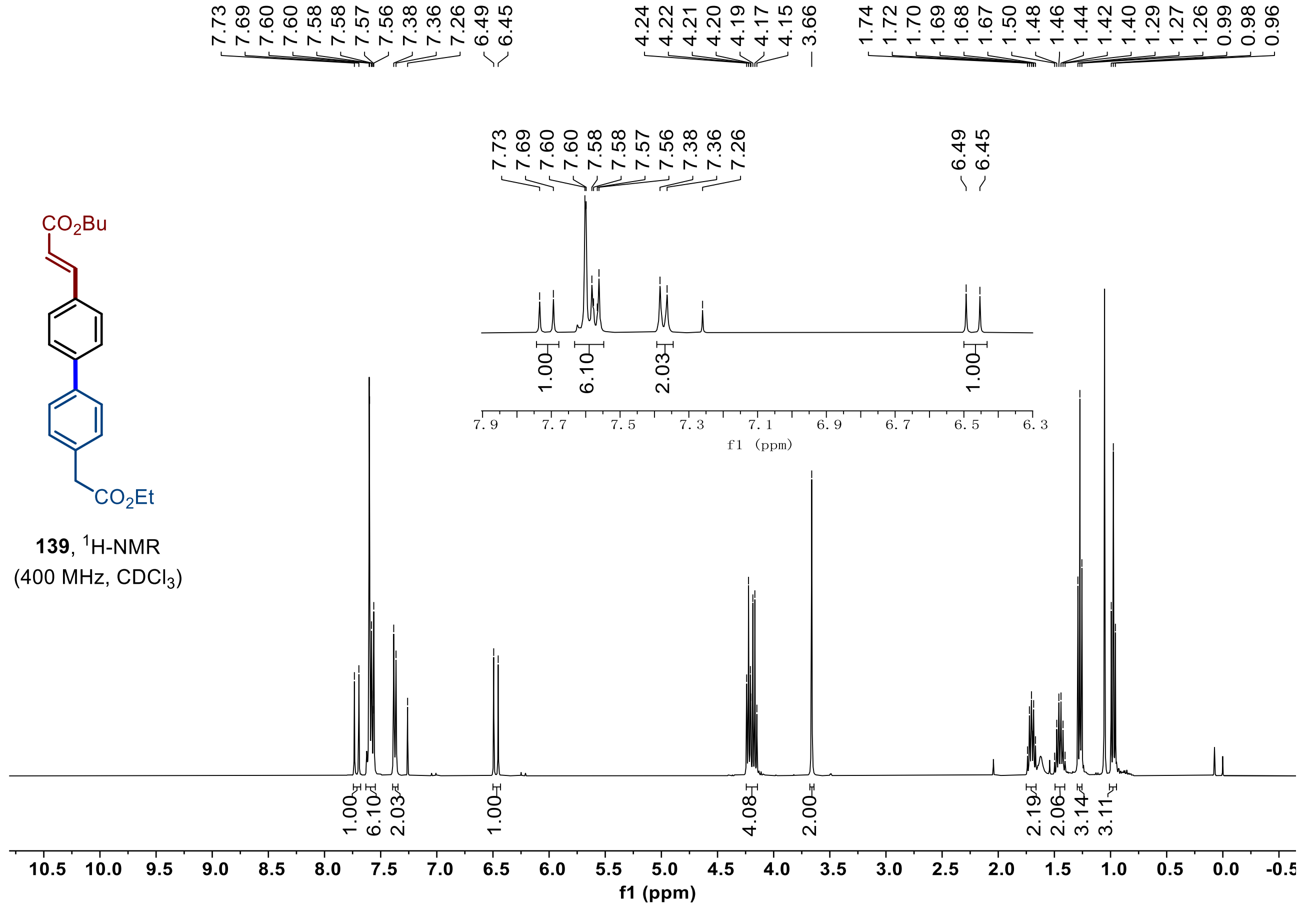
CO2Bu
CO2Et
139, 1H-NMR
(400 MHz, CDCl3)
f1 (ppm)

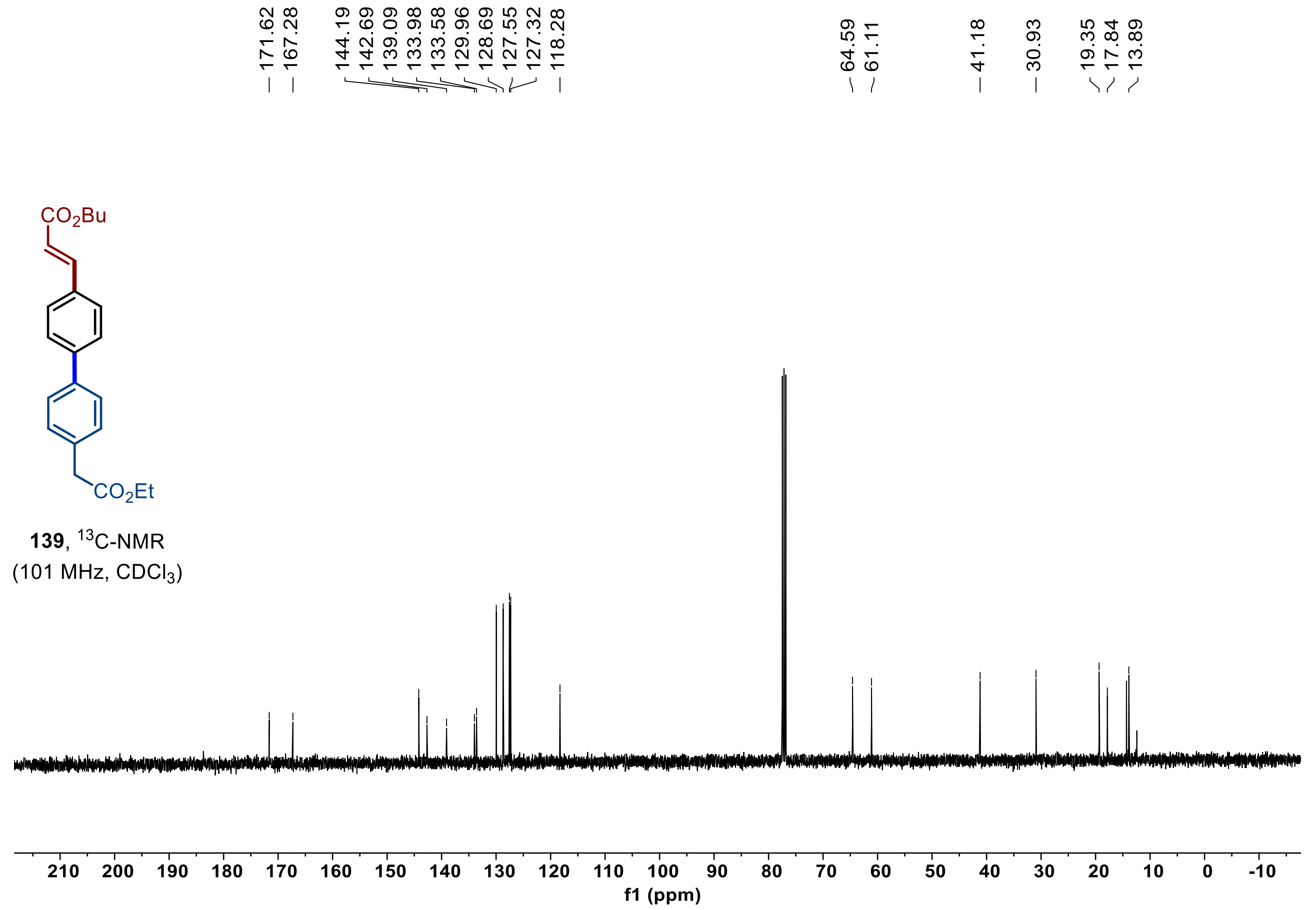

171.62
167.28
144.19
142.69
139.09
133.98
133.58
129.96
128.69
127.55
127.32
118.28
64.59
61.11
41.18
30.93
19.35
17.84
13.89
CO2Bu
CO2Et
139, 13C-NMR
(101 MHz, CDCl3)
210 200 190 180 170 160 150 140 130 120 110 100 90 80 70 60 50 40 30 20 10 0 -10
f1 (ppm)

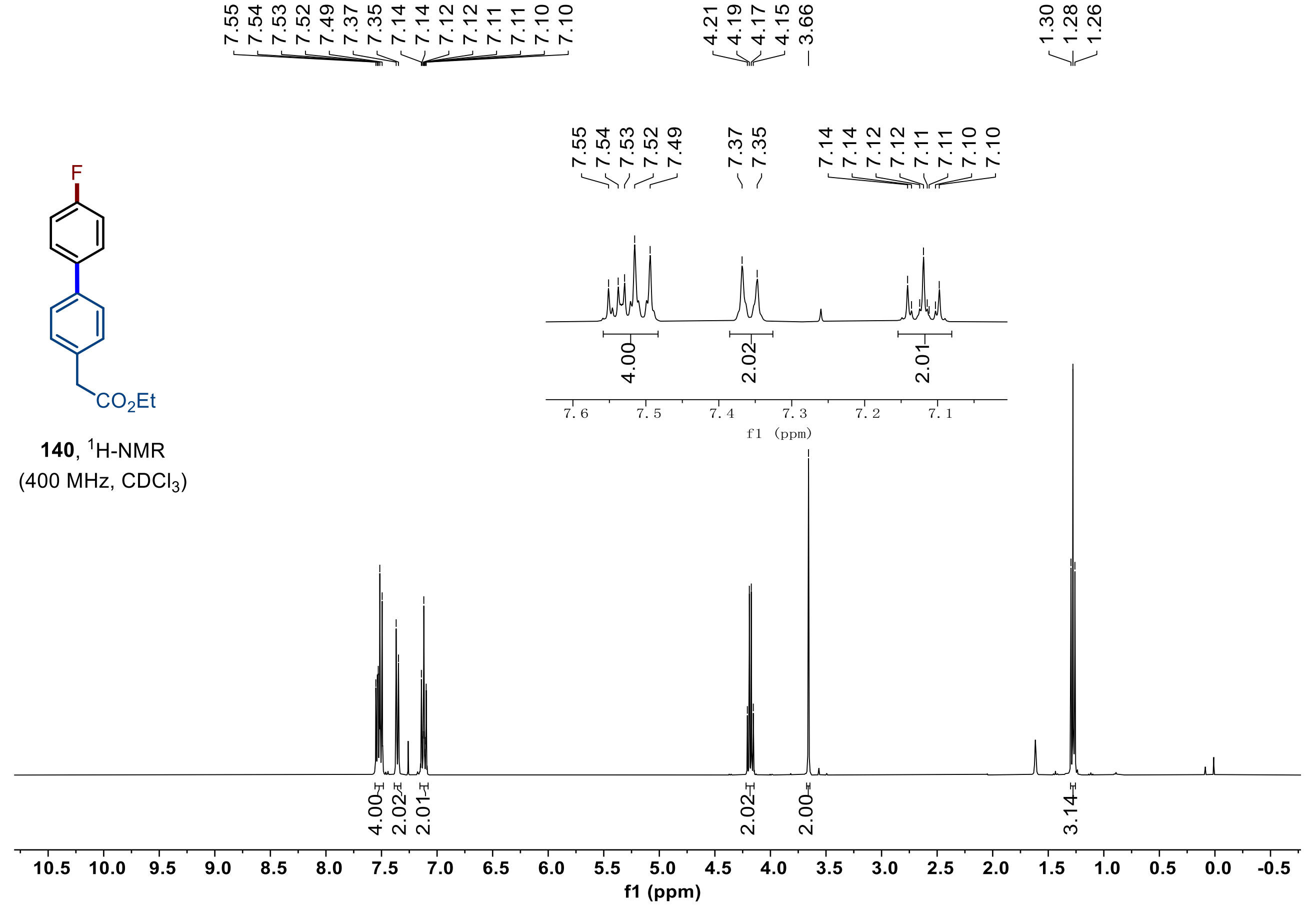

7.55
7.54
7.53
7.52
7.49
7.37
7.35
7.14
7.14
7.12
7.12
7.11
7.11
7.10
7.10
4.21
4.19
4.17
4.15
3.66
1.30
1.28
1.26
F
CO2Et
140, 1H-NMR
(400 MHz, CDCl3)
4.00
2.02
2.01
7.6 7.5 7.4 7.3 7.2 7.1
f1 (ppm)
4.00
2.02
2.01
2.02
2.00
3.14
10.5 10.0 9.5 9.0 8.5 8.0 7.5 7.0 6.5 6.0 5.5 5.0 4.5 4.0 3.5 3.0 2.5 2.0 1.5 1.0 0.5 0.0 -0.5
f1 (ppm)

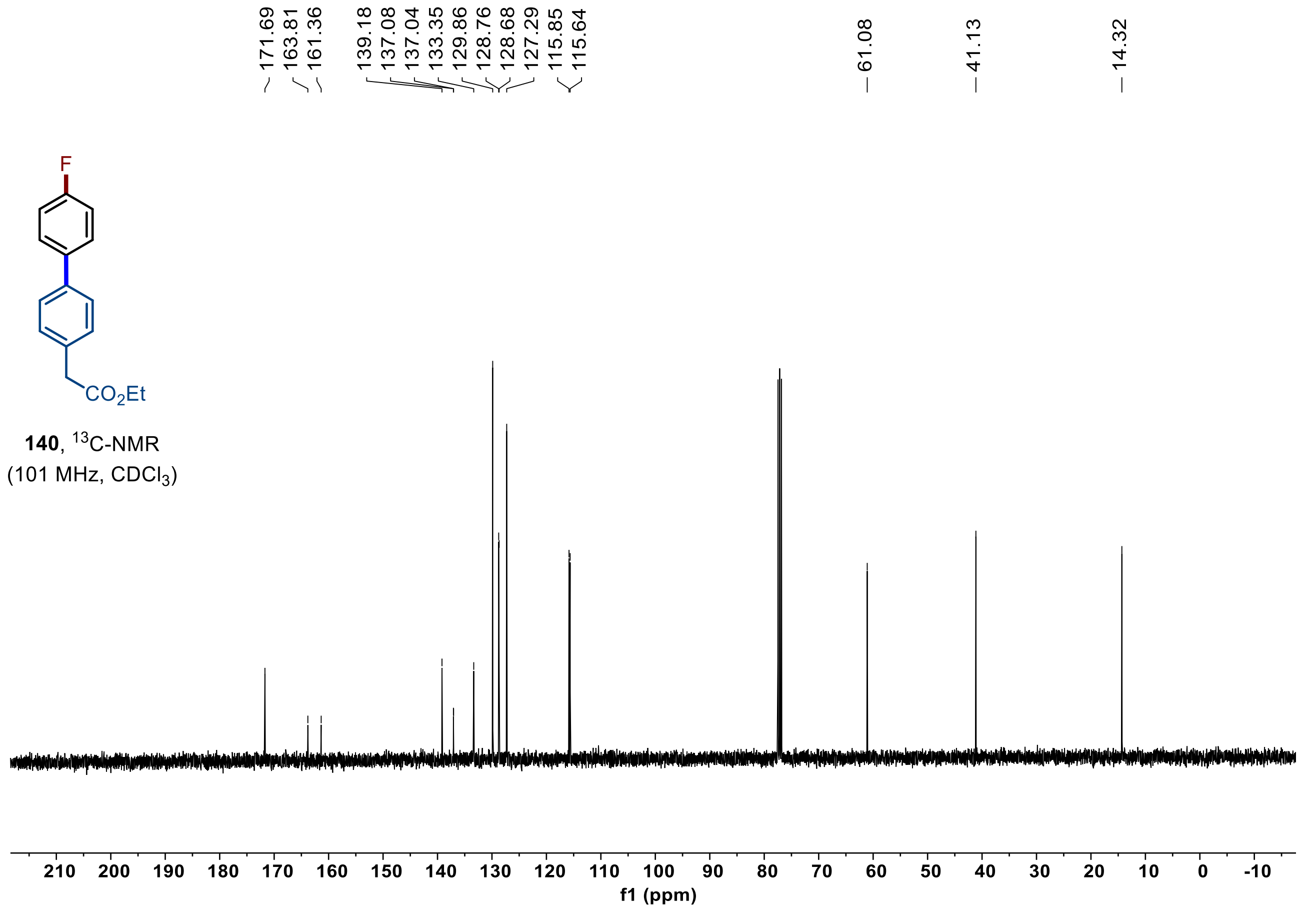
171.69
163.81
161.36
139.18
137.08
137.04
133.35
129.86
128.76
128.68
127.29
115.85
115.64
61.08
41.13
14.32
F
CO2Et
140, 13C-NMR
(101 MHz, CDCl3)
210 200 190 180 170 160 150 140 130 120 110 100 90 80 70 60 50 40 30 20 10 0 -10
f1 (ppm)

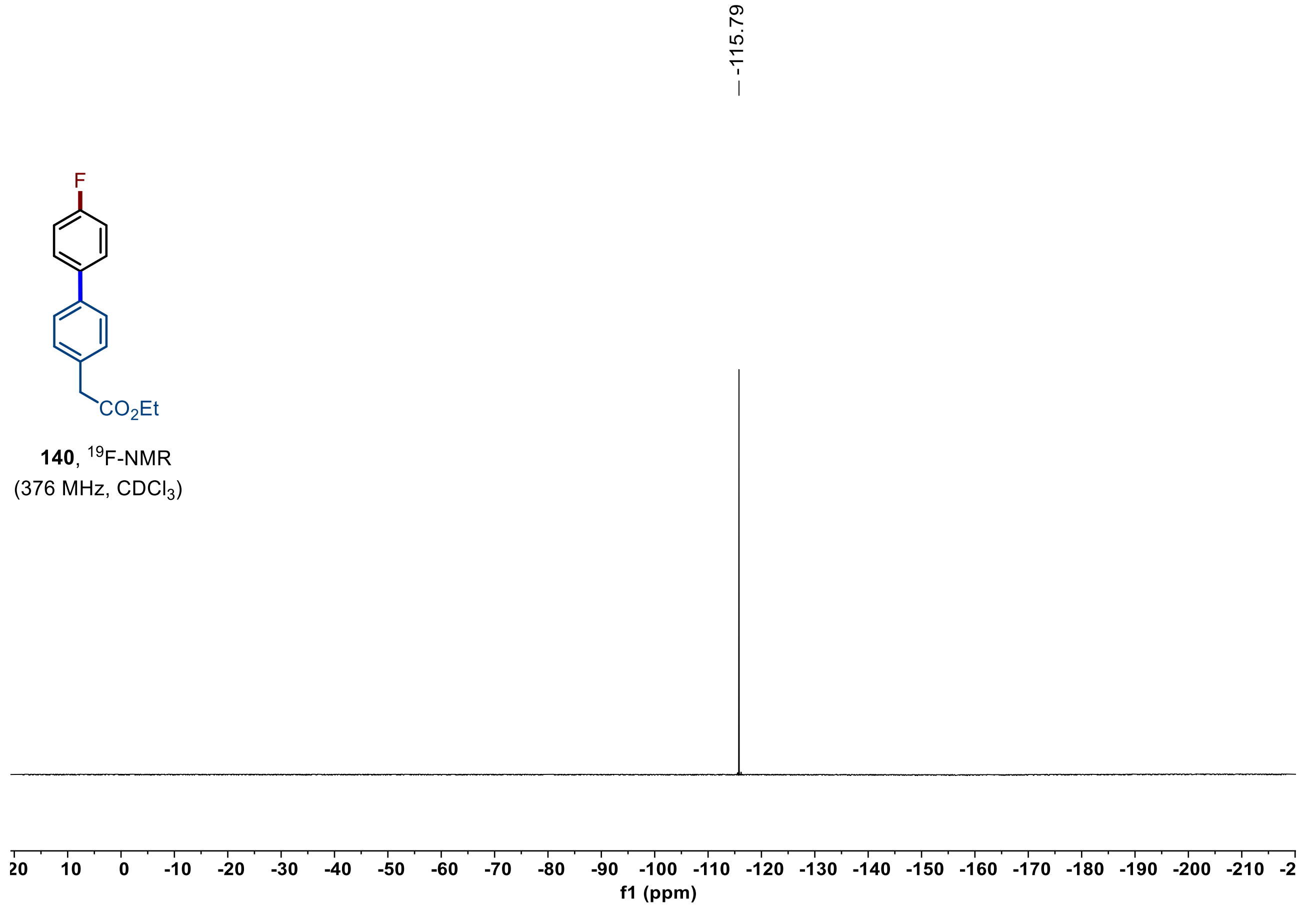
-115.79
F
CO2Et
140, 19F-NMR
(376 MHz, CDCl3)
20 10 0 -10 -20 -30 -40 -50 -60 -70 -80 -90 -100 -110 -120 -130 -140 -150 -160 -170 -180 -190 -200 -210 -2
f1 (ppm)

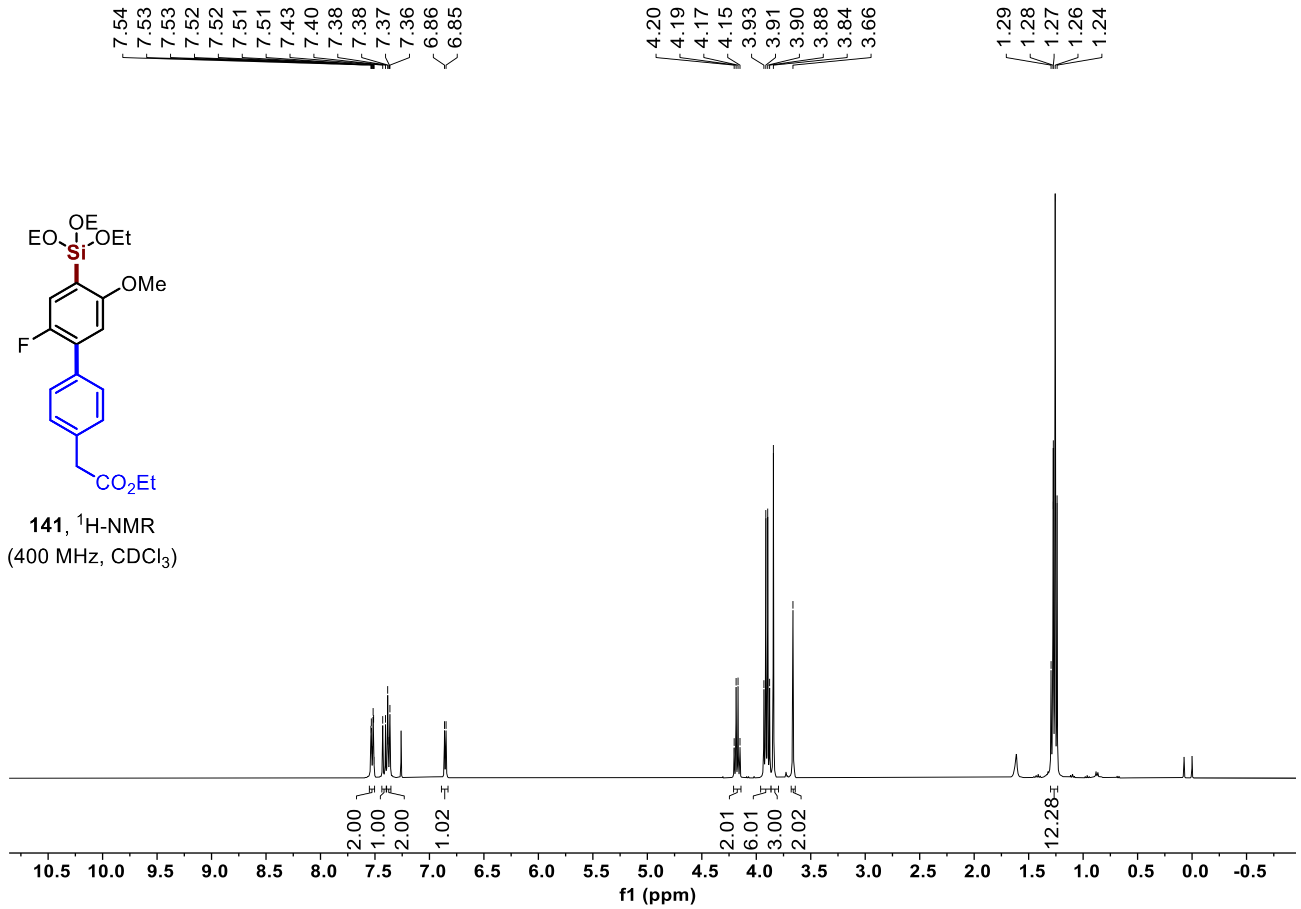

7.54
7.53
7.53
7.52
7.52
7.51
7.51
7.43
7.40
7.38
7.38
7.37
7.36
6.86
6.85
4.20
4.19
4.17
4.15
3.93
3.91
3.90
3.88
3.84
3.66
1.29
1.28
1.27
1.26
1.24
EO
OE
OEt
Si
OMe
F
CO2Et
141, 1H-NMR
(400 MHz, CDCl3)
2.00
1.00
2.00
1.02
2.01
6.01
3.00
2.02
12.28
10.5 10.0 9.5 9.0 8.5 8.0 7.5 7.0 6.5 6.0 5.5 5.0 4.5 4.0 3.5 3.0 2.5 2.0 1.5 1.0 0.5 0.0 -0.5
f1 (ppm)

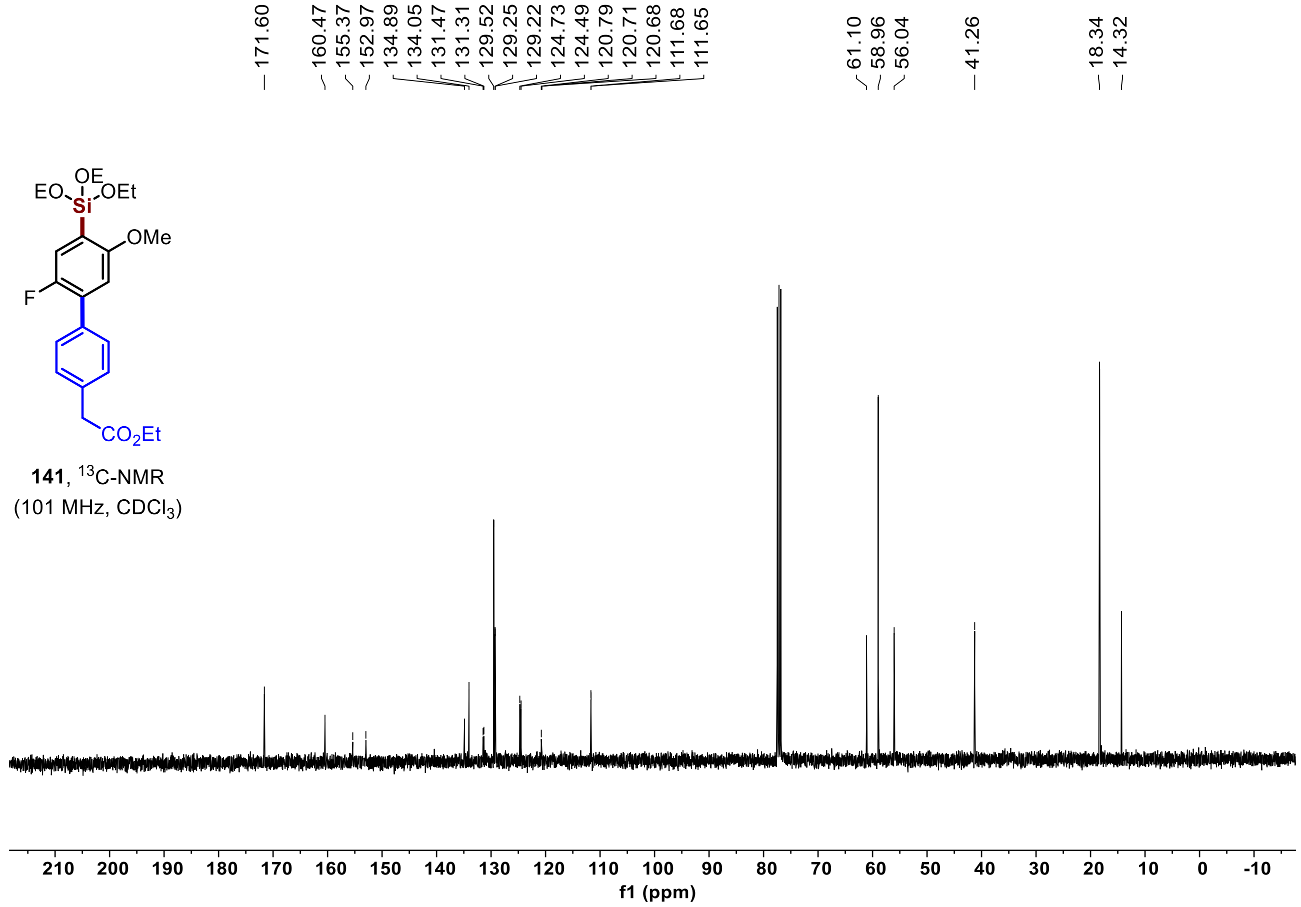

171.60
160.47
155.37
152.97
134.89
134.05
131.47
131.31
129.52
129.25
129.22
124.73
124.49
120.79
120.71
120.68
111.68
111.65
61.10
58.96
56.04
41.26
18.34
14.32
EO
OE
OEt
Si
OMe
F
CO2Et
141, 13C-NMR
(101 MHz, CDCl3)
210 200 190 180 170 160 150 140 130 120 110 100 90 80 70 60 50 40 30 20 10 0 -10
f1 (ppm)

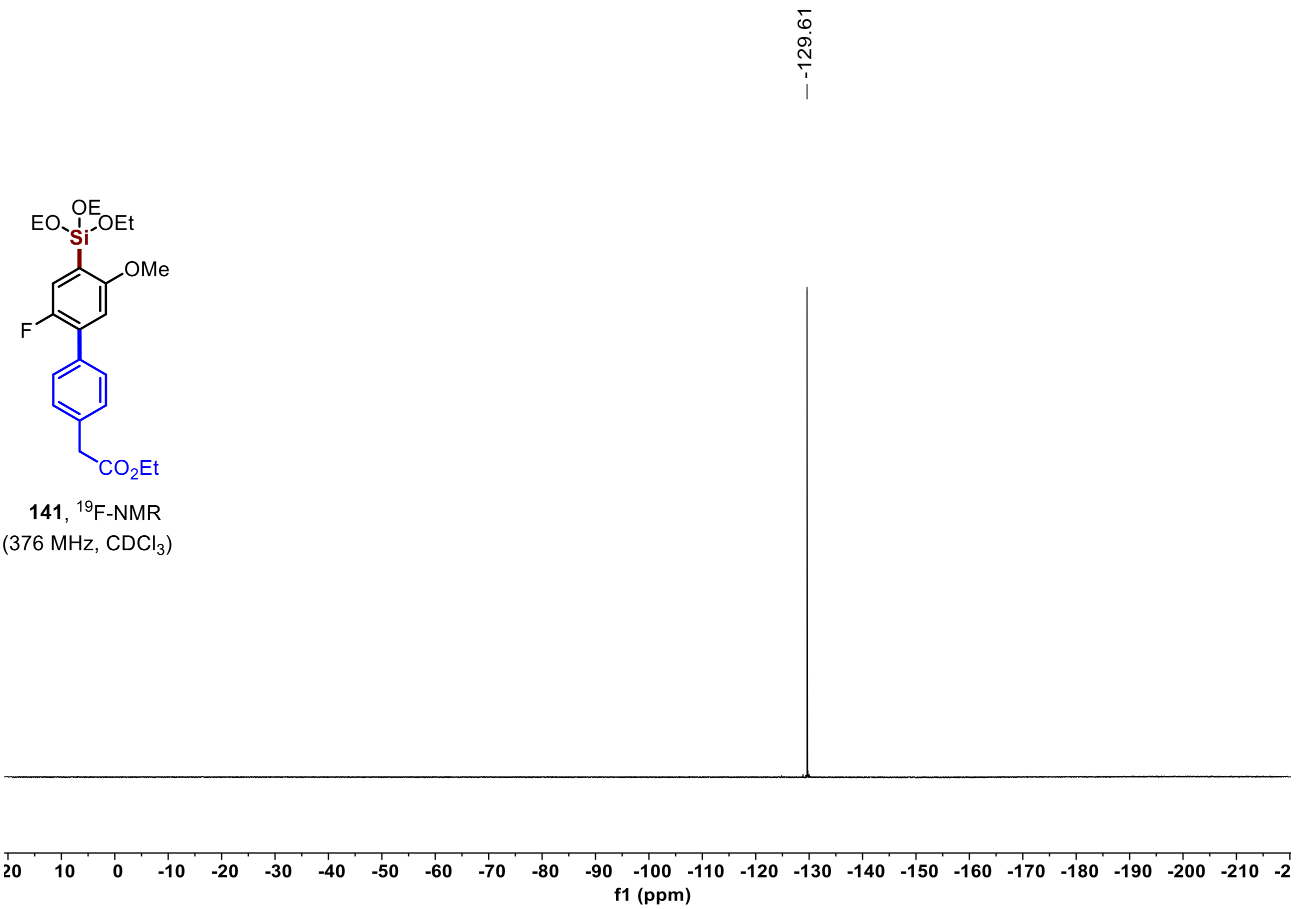

141, ^{19}F-NMR (376 MHz, $CDCl_3$)

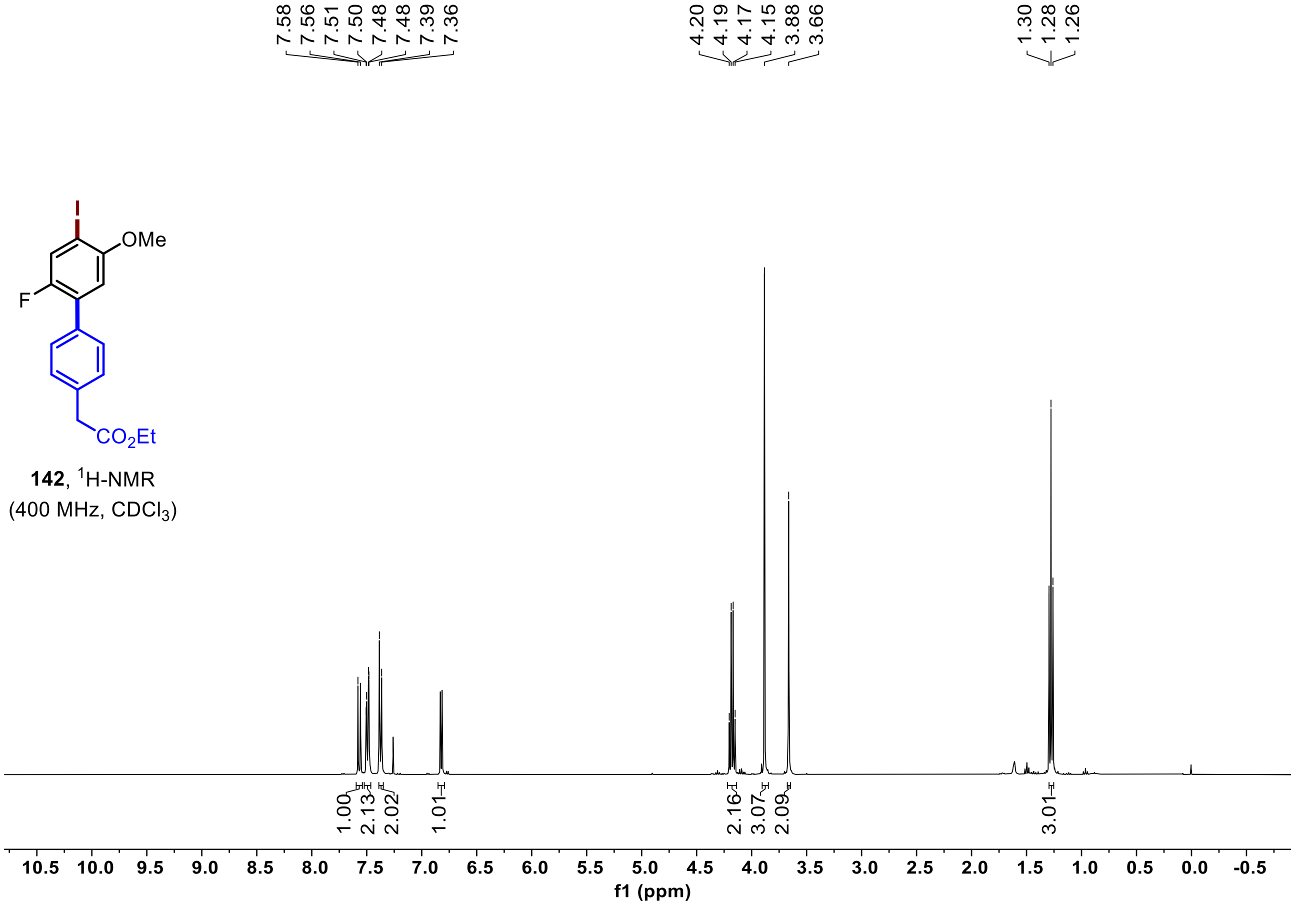

142, ^{1}H-NMR (400 MHz, $CDCl_3$)

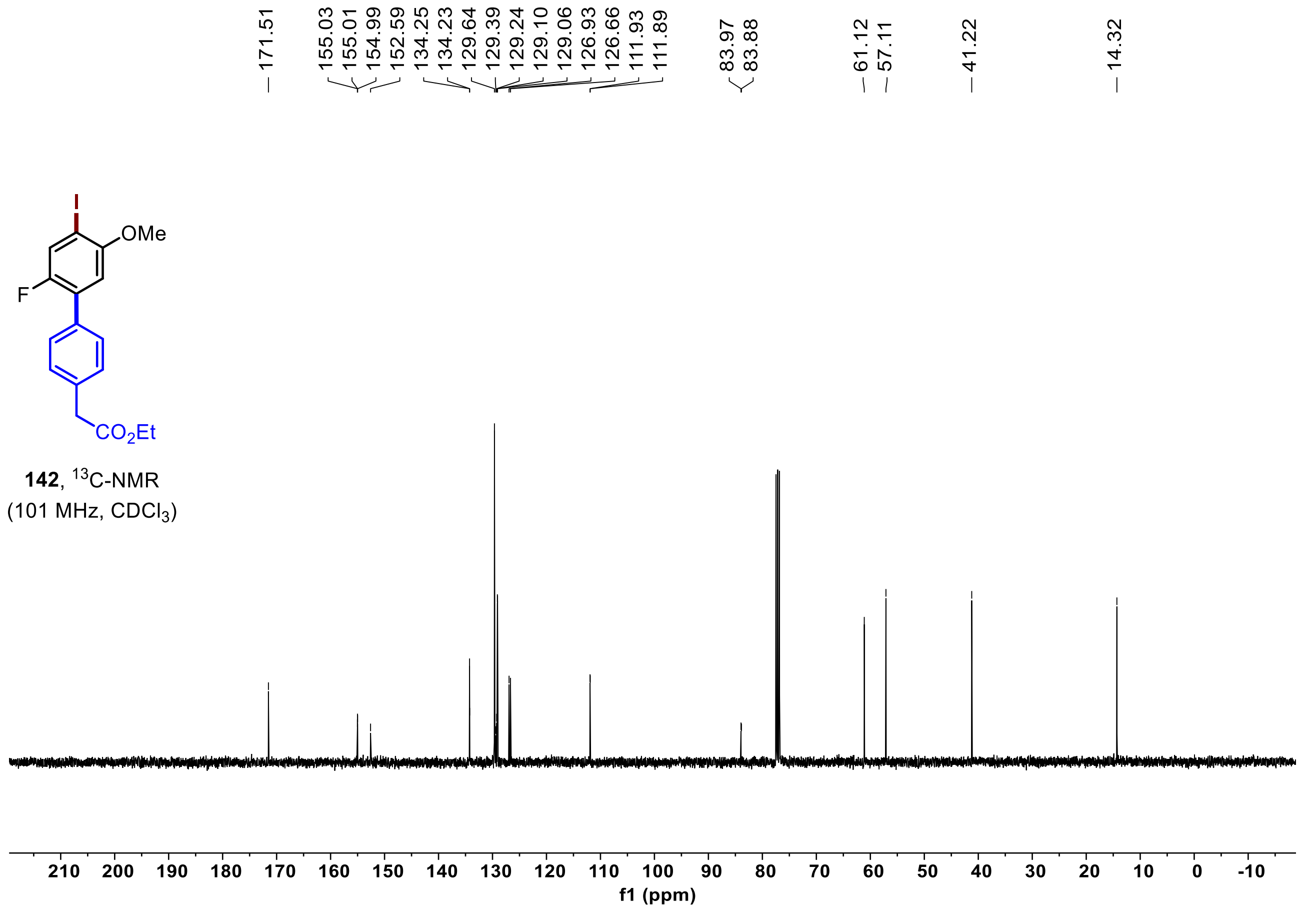

142, ^{13}C-NMR
(101 MHz, $CDCl_3$)

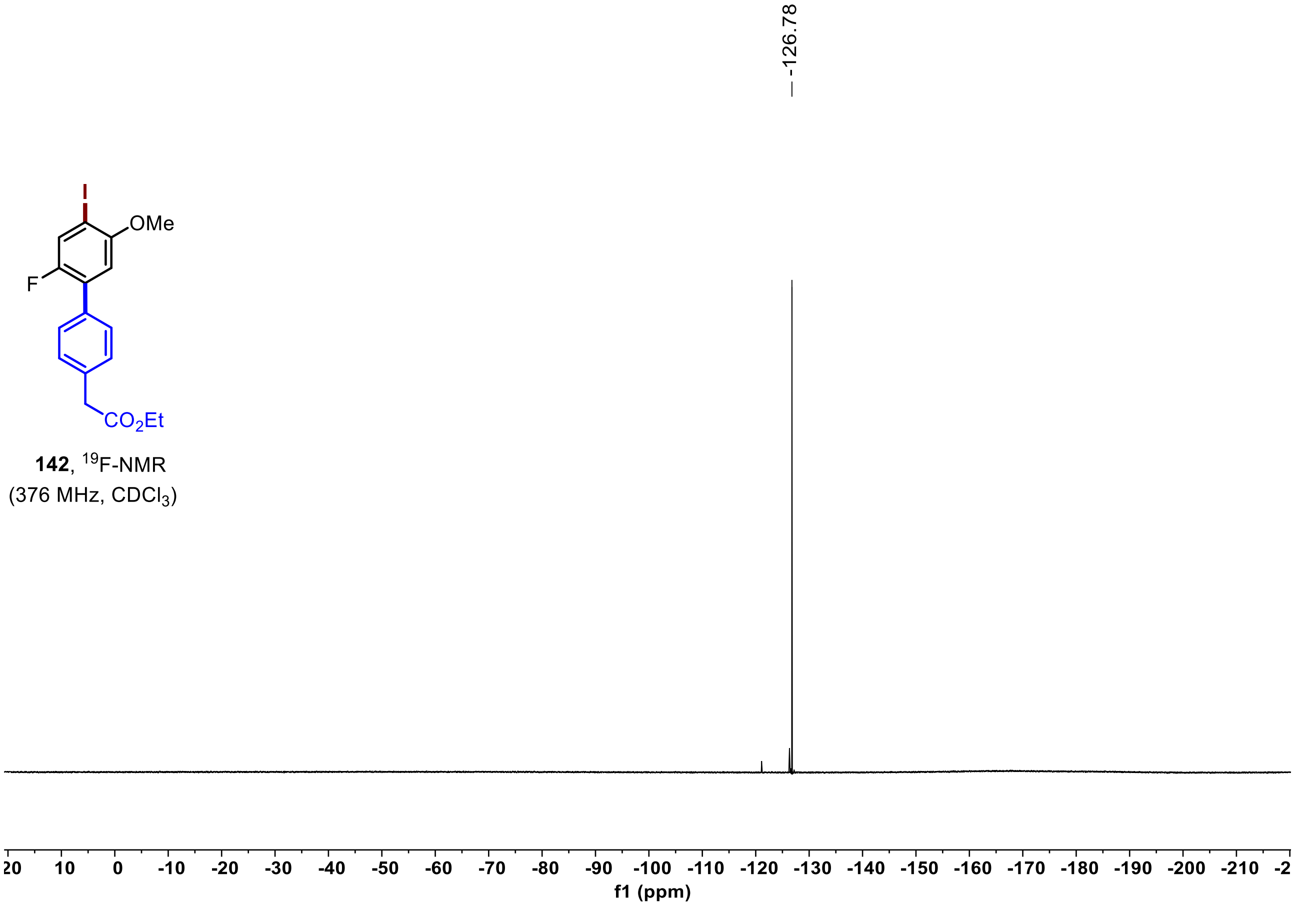

142, ^{19}F-NMR
(376 MHz, $CDCl_3$)

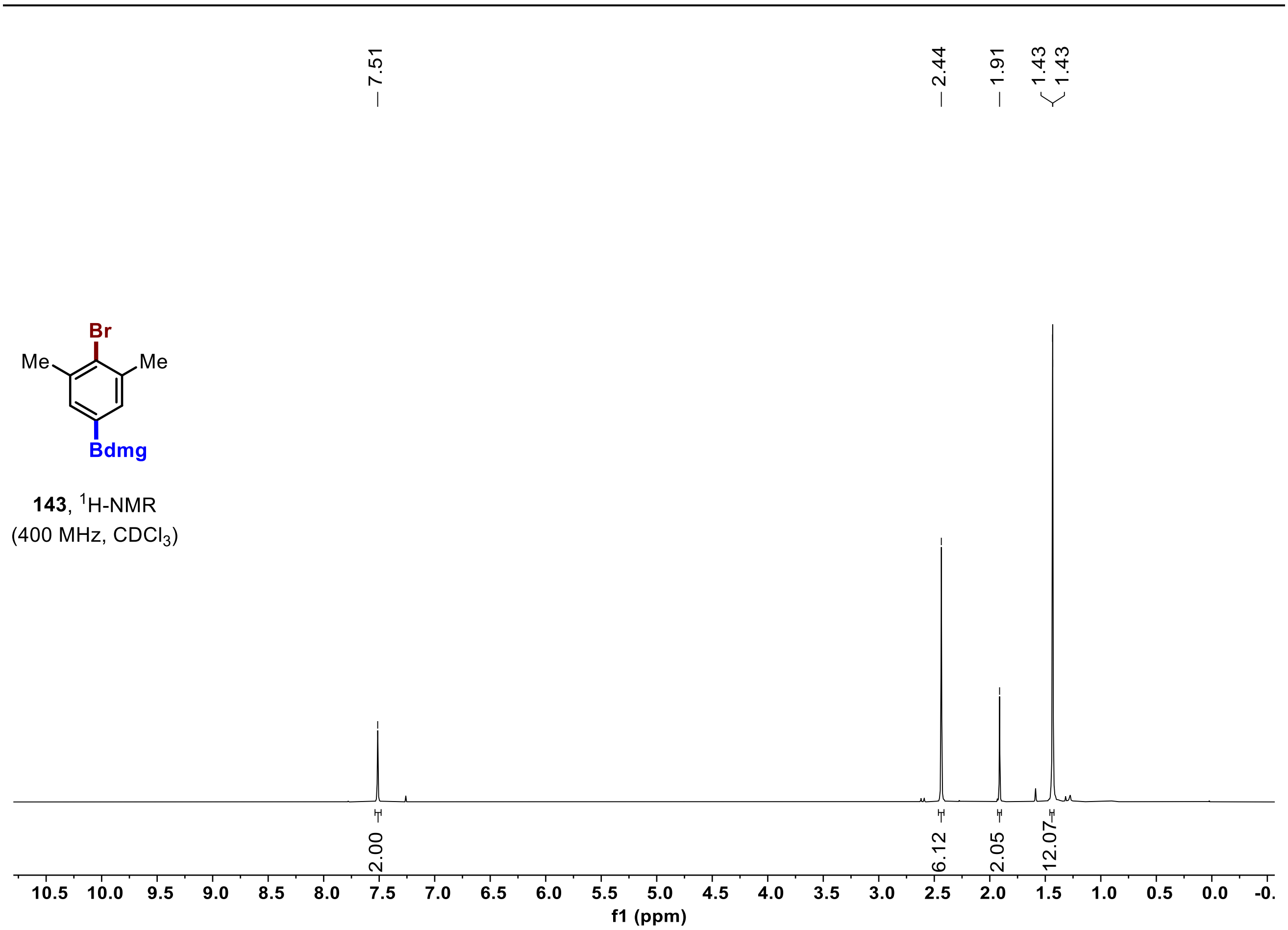

143, ^{1}H-NMR
(400 MHz, $CDCl_3$)

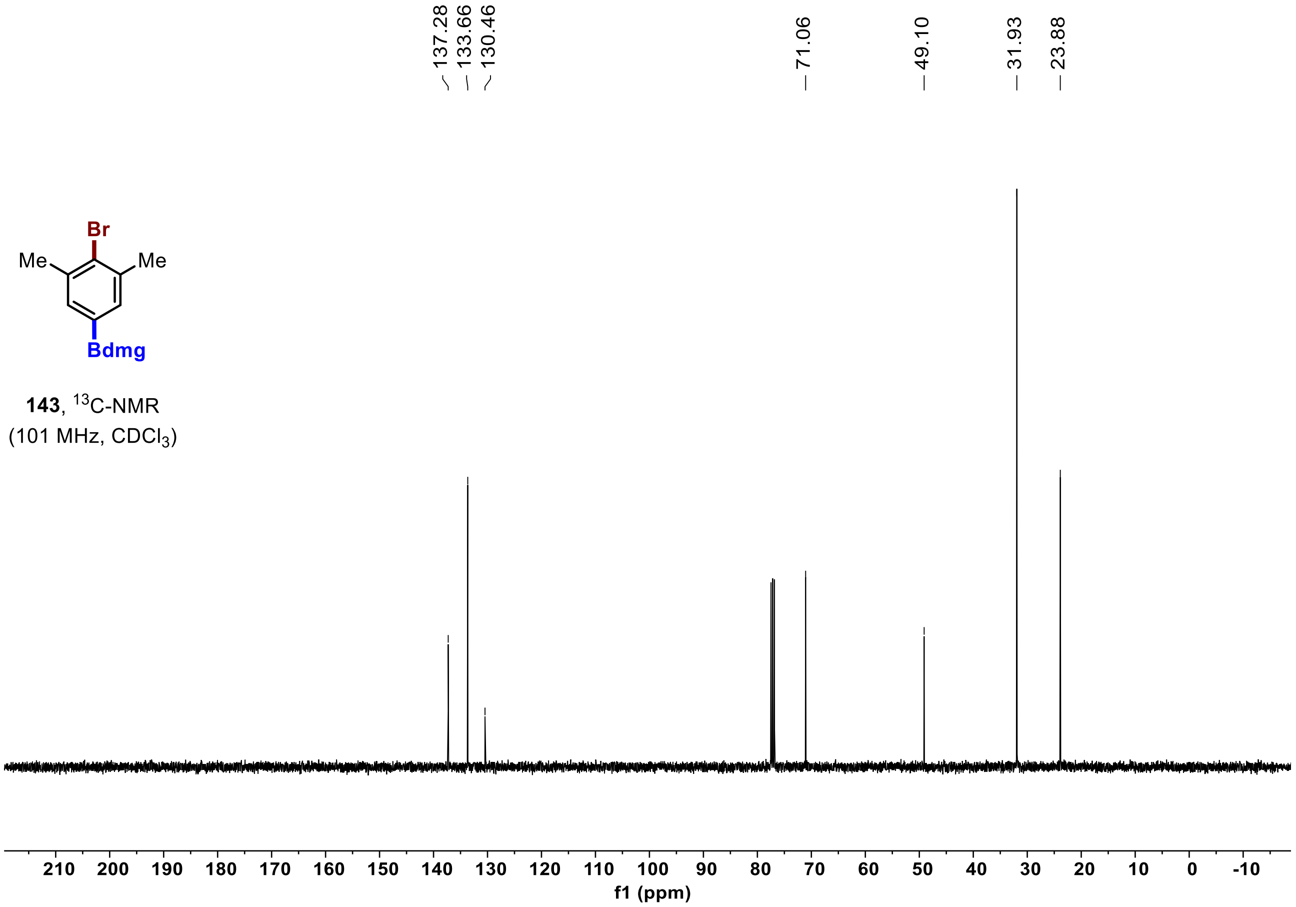

143, ^{13}C-NMR
(101 MHz, $CDCl_3$)

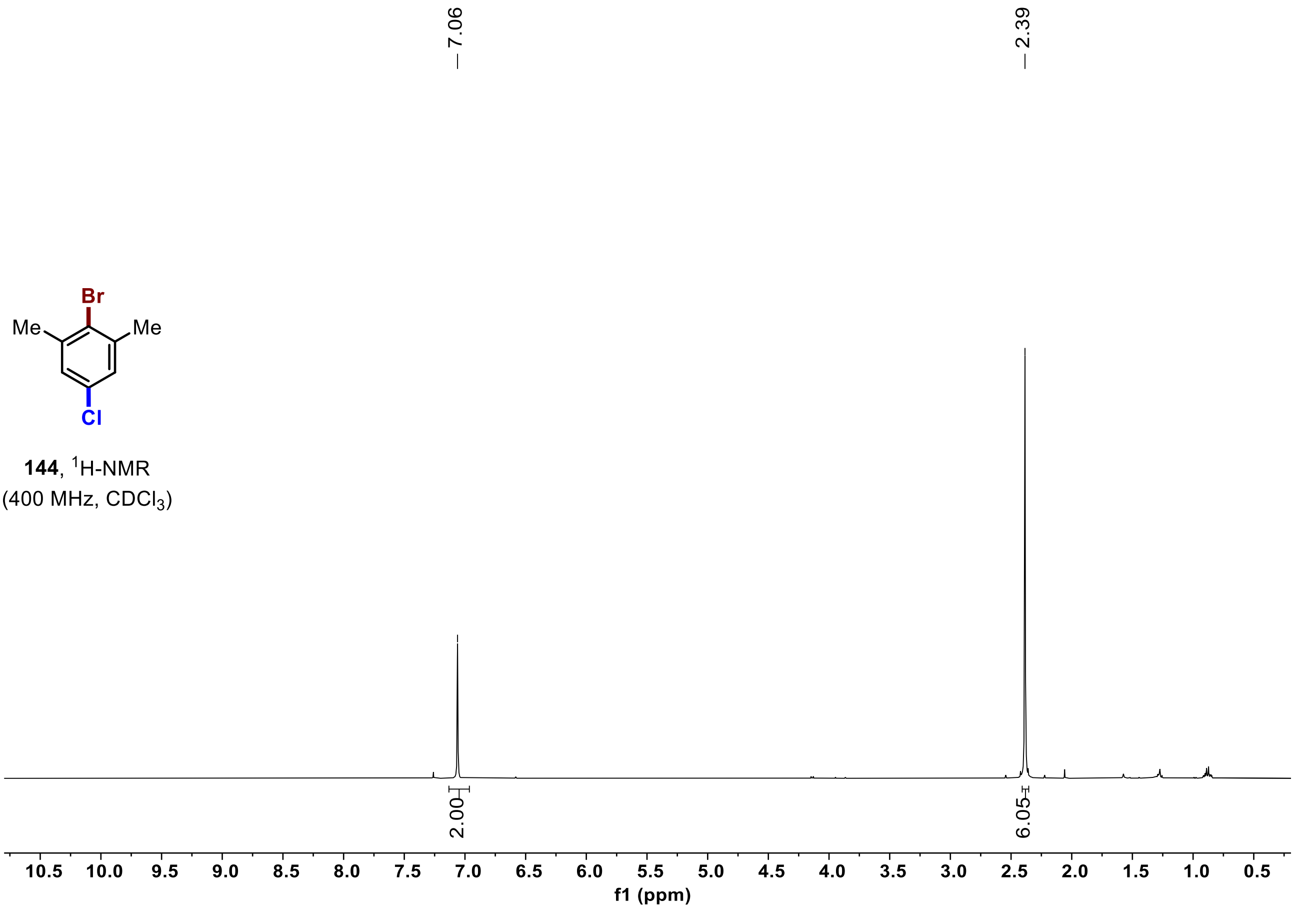

7.06
2.39
Br
Me
Me
Cl
144, ^{1}H-NMR
(400 MHz, $CDCl_3$)
2.00
6.05
10.5 10.0 9.5 9.0 8.5 8.0 7.5 7.0 6.5 6.0 5.5 5.0 4.5 4.0 3.5 3.0 2.5 2.0 1.5 1.0 0.5
f1 (ppm)

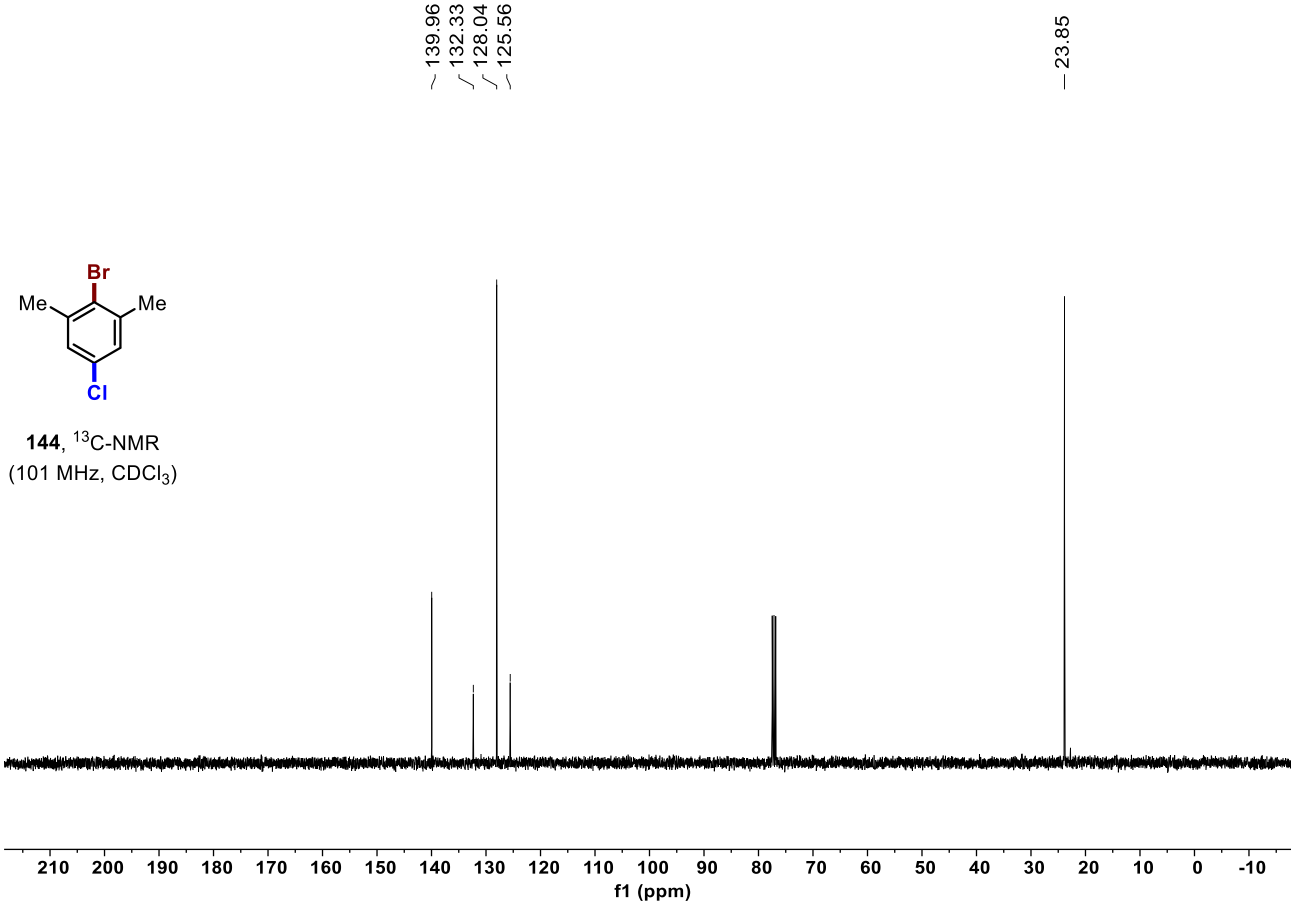

139.96
132.33
128.04
125.56
23.85
Br
Me
Me
Cl
144, ^{13}C-NMR
(101 MHz, $CDCl_3$)
210 200 190 180 170 160 150 140 130 120 110 100 90 80 70 60 50 40 30 20 10 0 -10
f1 (ppm)

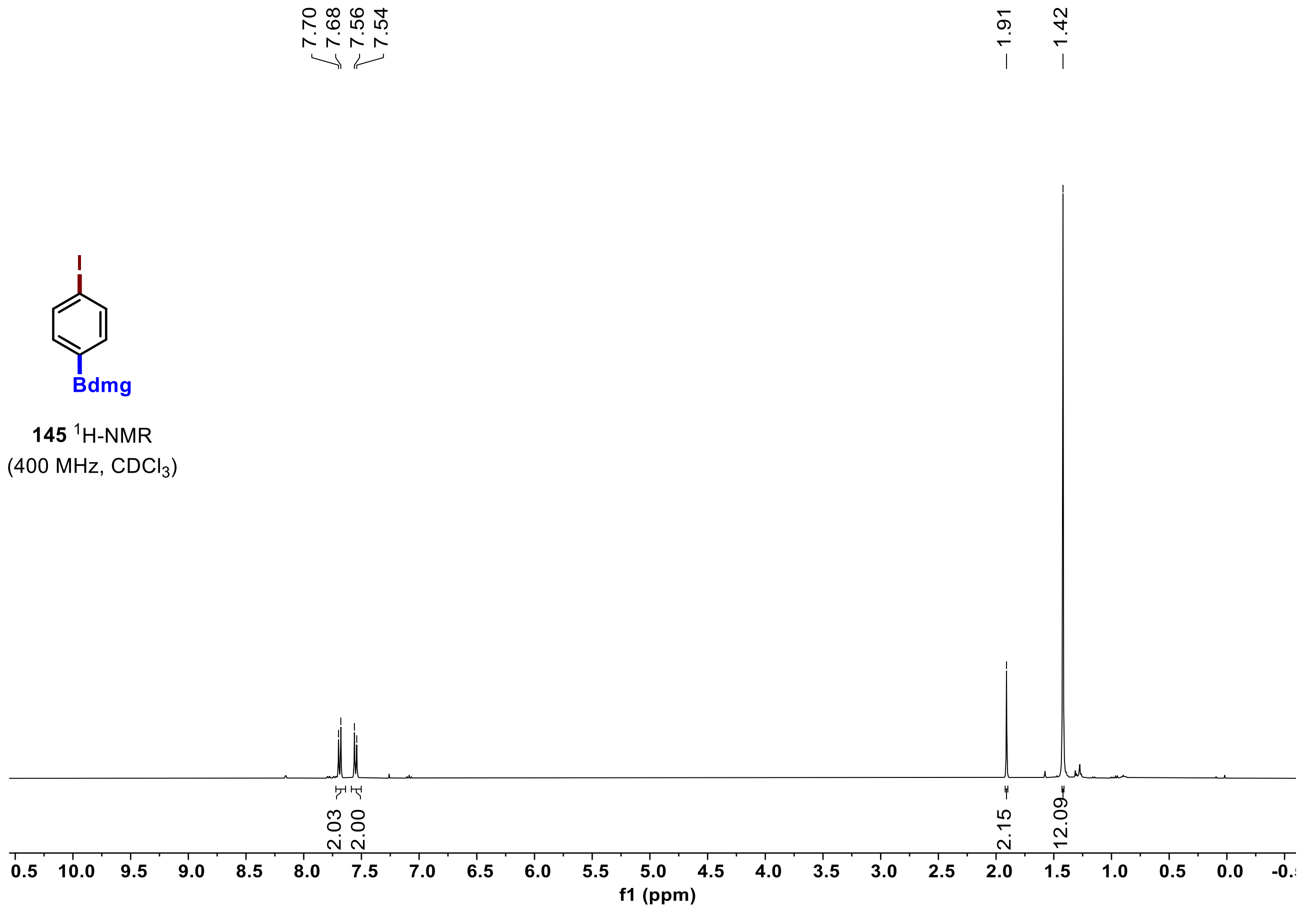

145 ^{1}H-NMR
(400 MHz, $CDCl_3$)

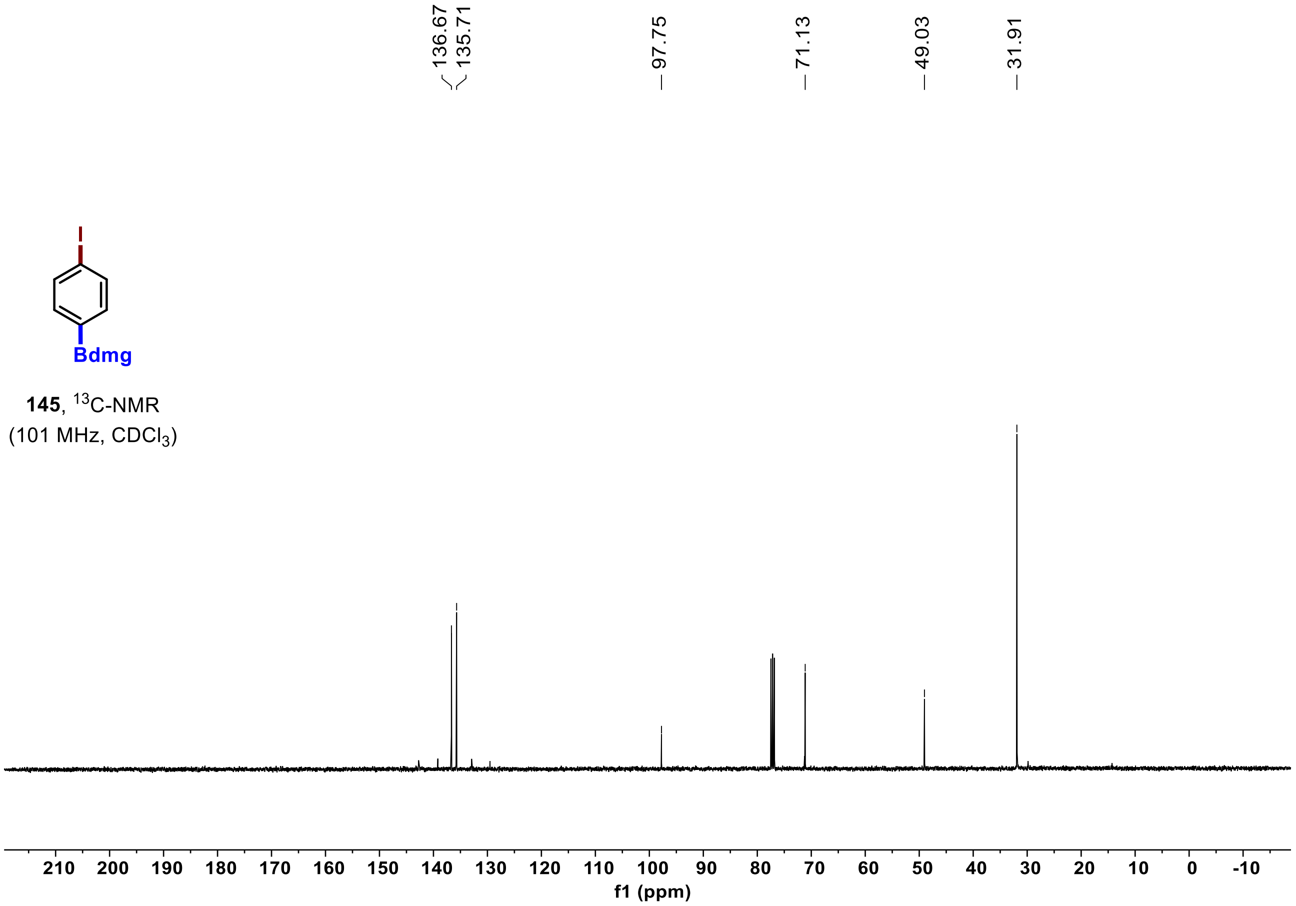

145, ^{13}C-NMR
(101 MHz, $CDCl_3$)

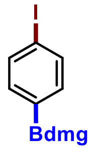

145, ^{11}B-NMR
(128 MHz, $CDCl_3$)

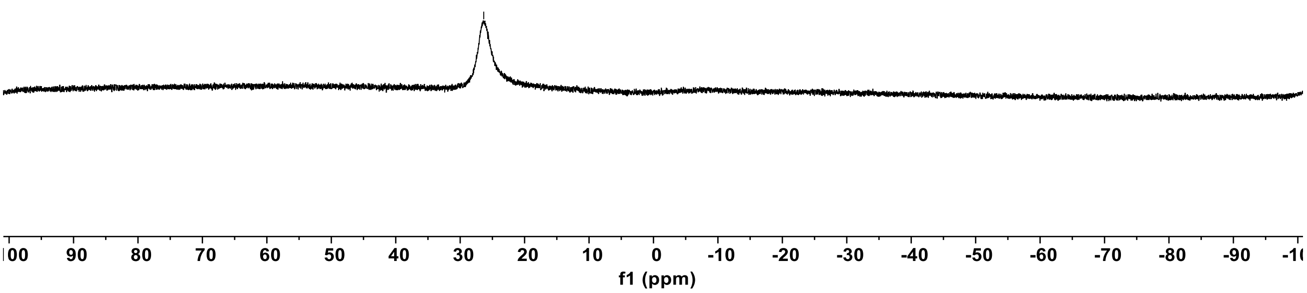

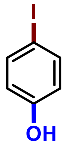

146, ^{1}H-NMR
(400 MHz, $CDCl_3$)

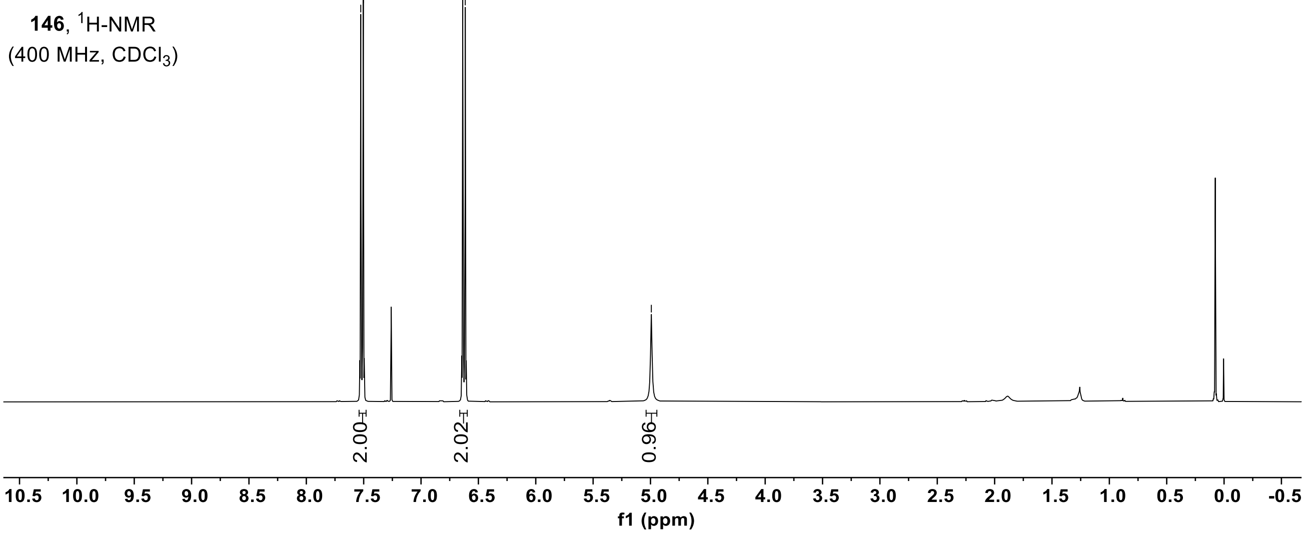

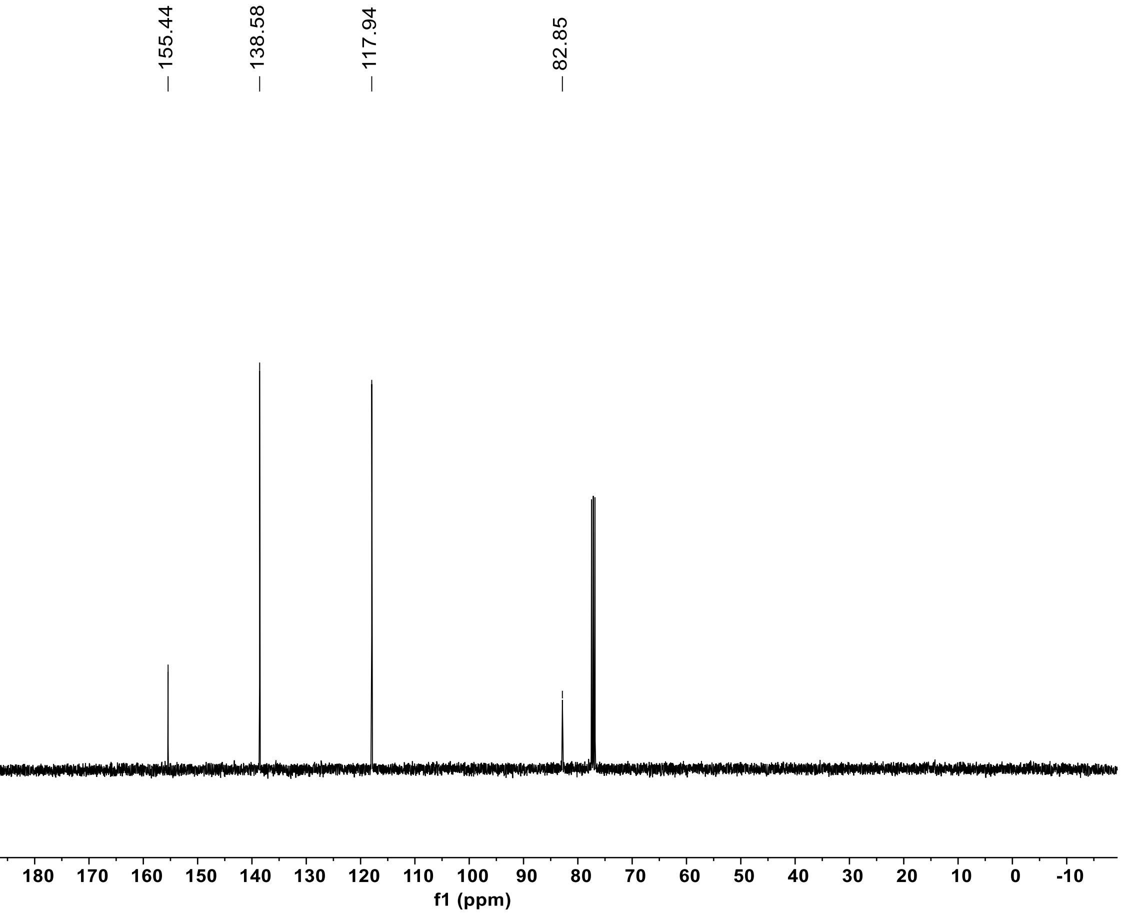

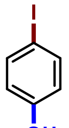

146, ^{13}C-NMR
(101 MHz, $CDCl_3$)

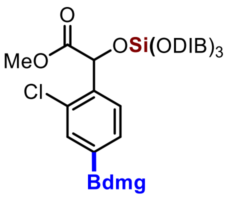

147, ^{1}H-NMR
(400 MHz, $CDCl_3$)

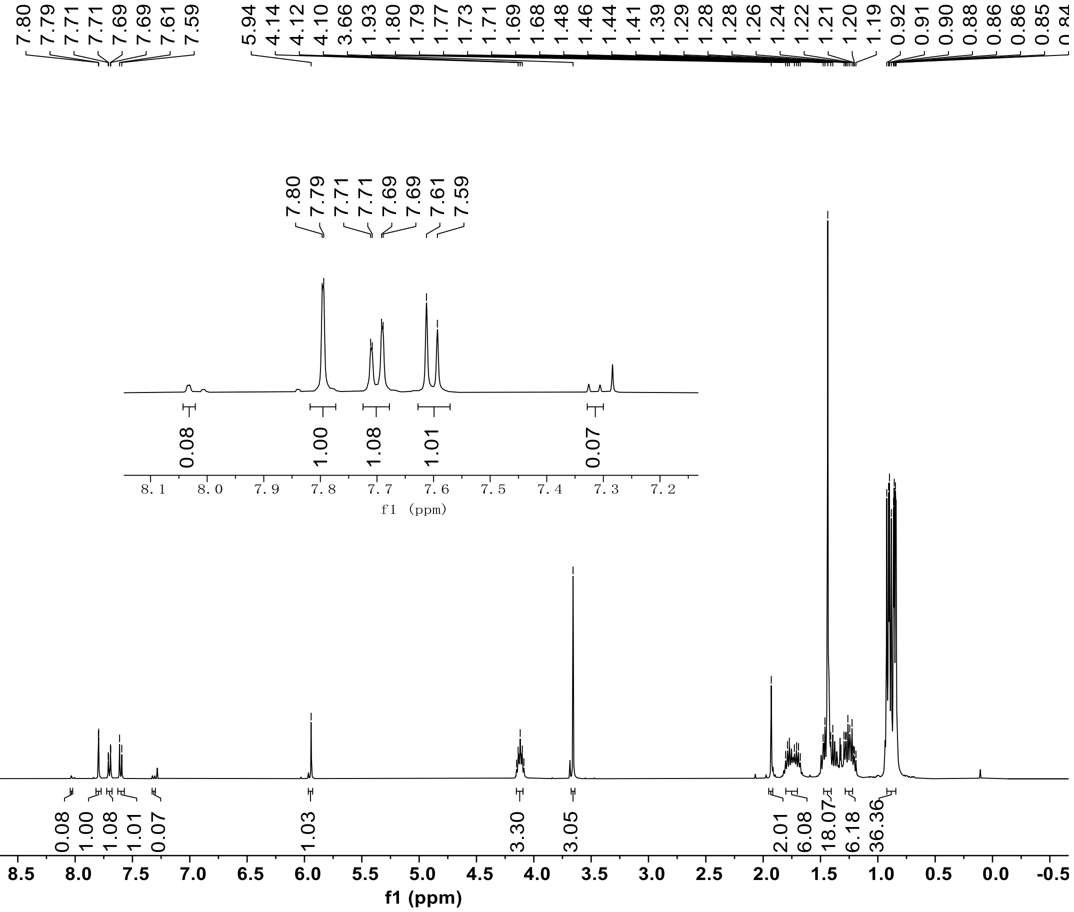

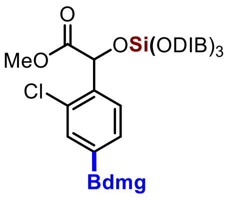

147, ^{13}C-NMR
(101 MHz, $CDCl_3$)

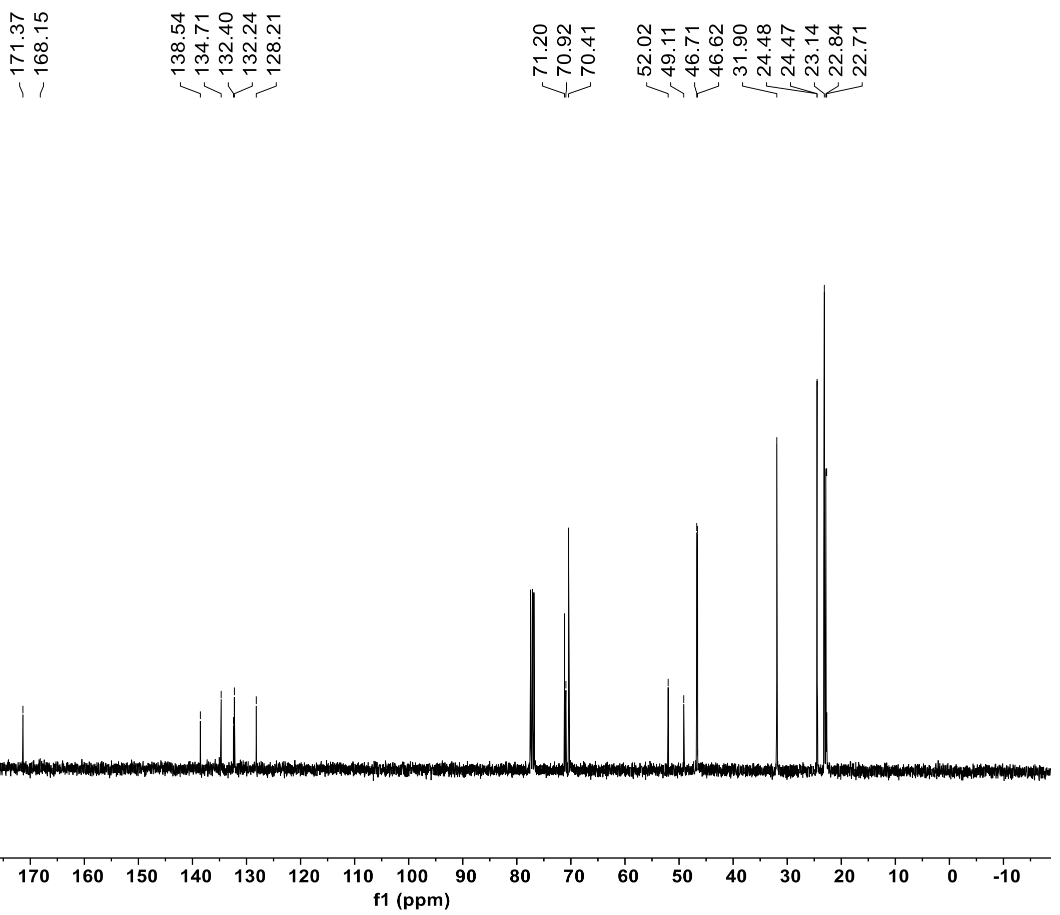

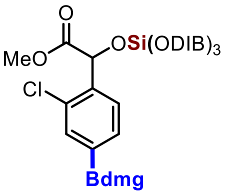

147, ^{11}B-NMR
(128 MHz, $CDCl_3$)

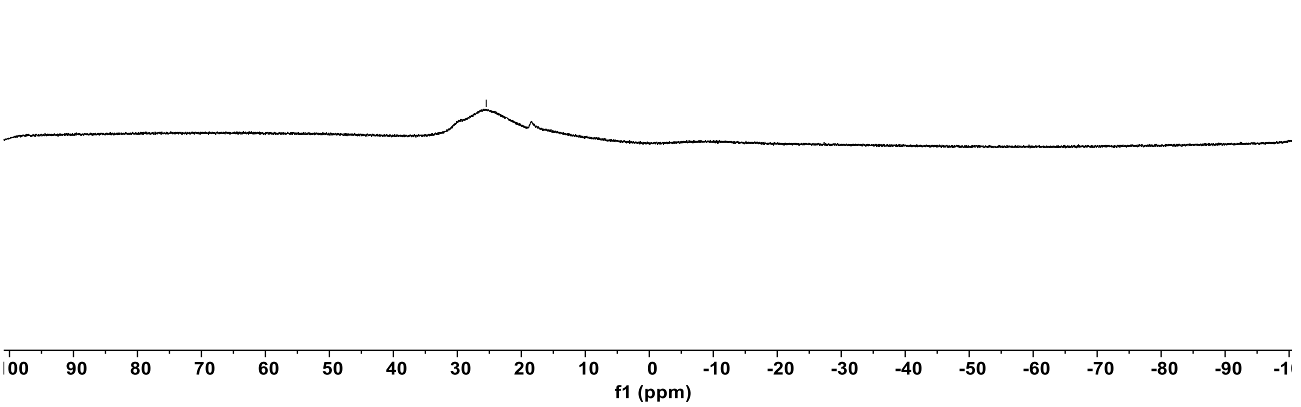

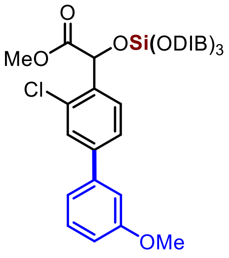

148, ^{1}H-NMR
(400 MHz, $CDCl_3$)

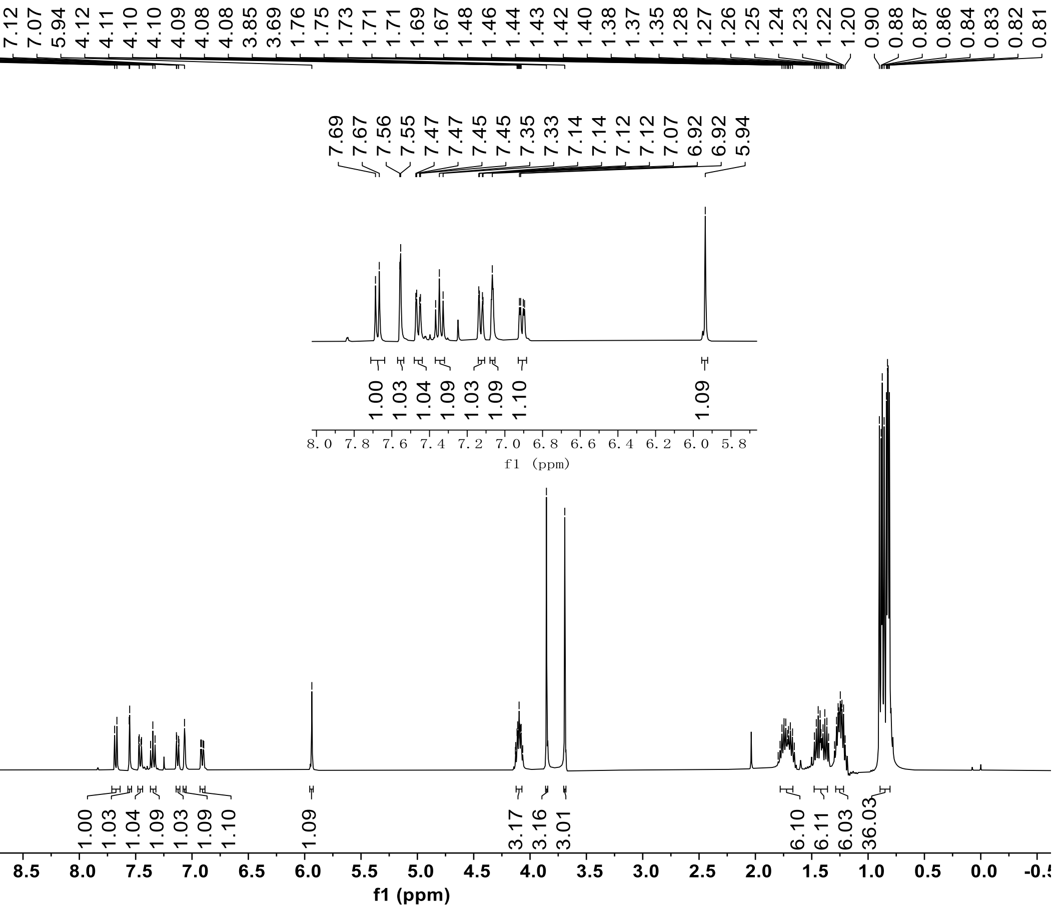

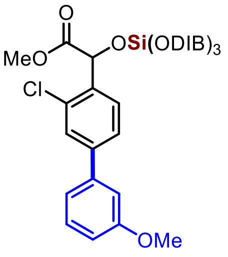

148, ^{13}C-NMR
(101 MHz, $CDCl_3$)

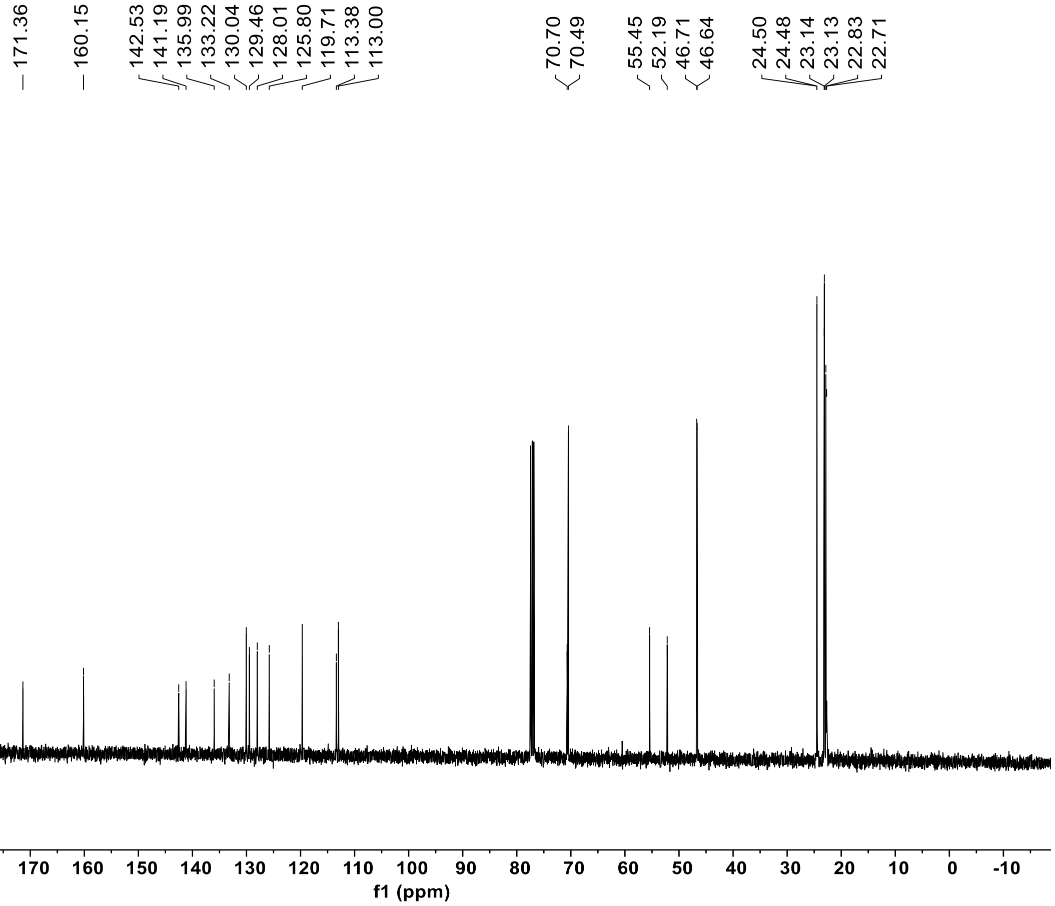

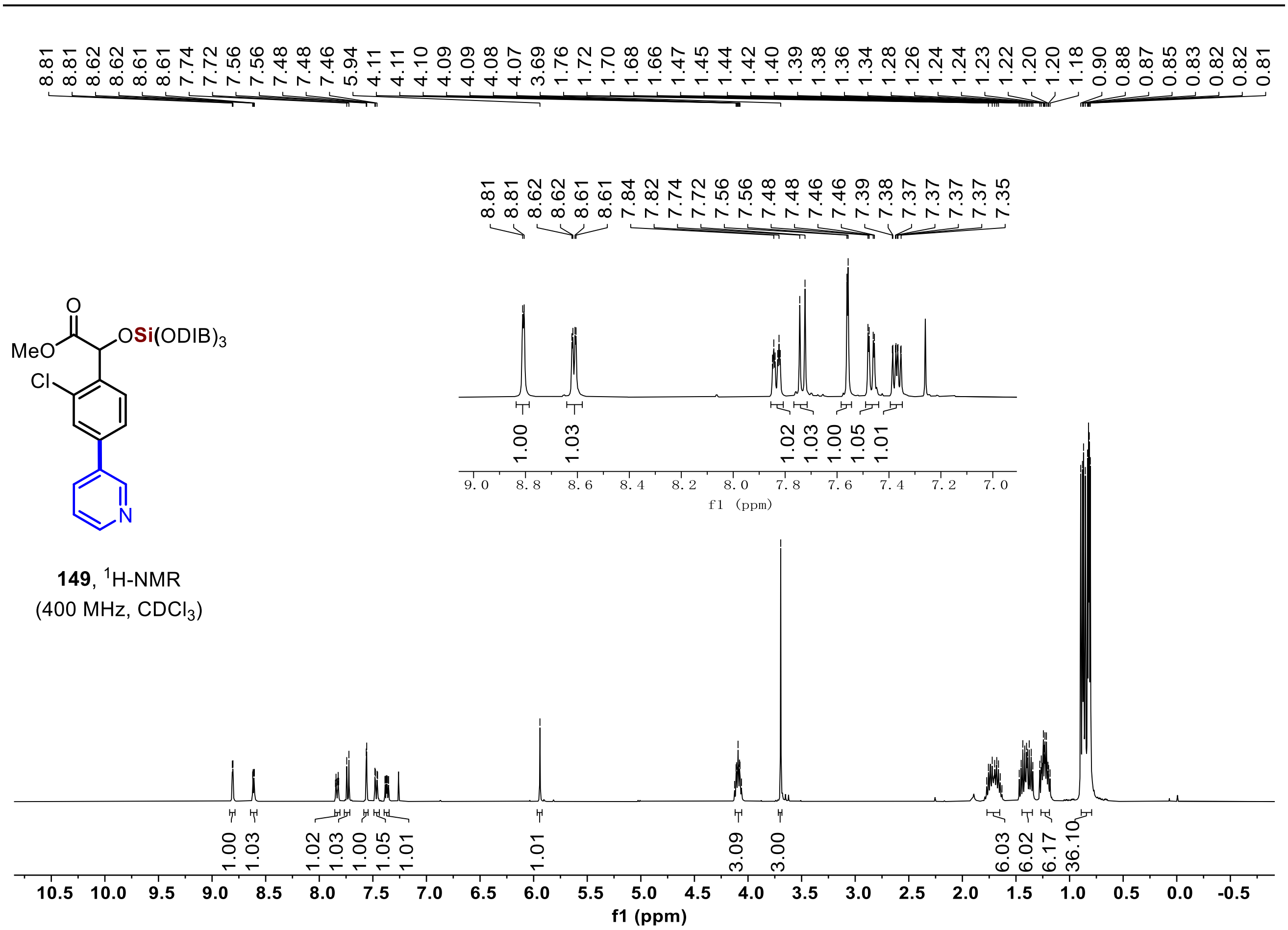

149, ^{1}H-NMR
(400 MHz, $CDCl_3$)

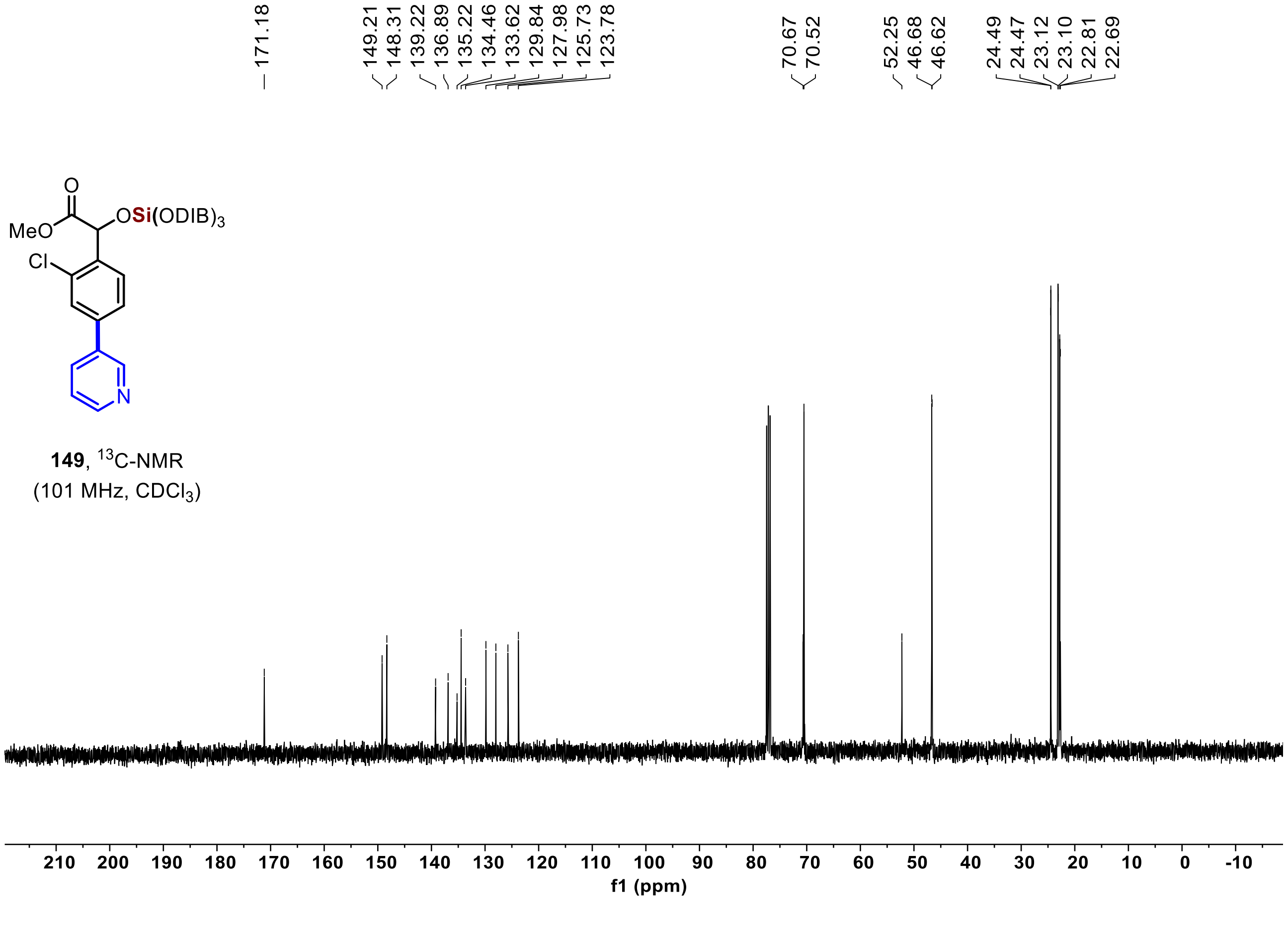

149, ^{13}C-NMR
(101 MHz, $CDCl_3$)

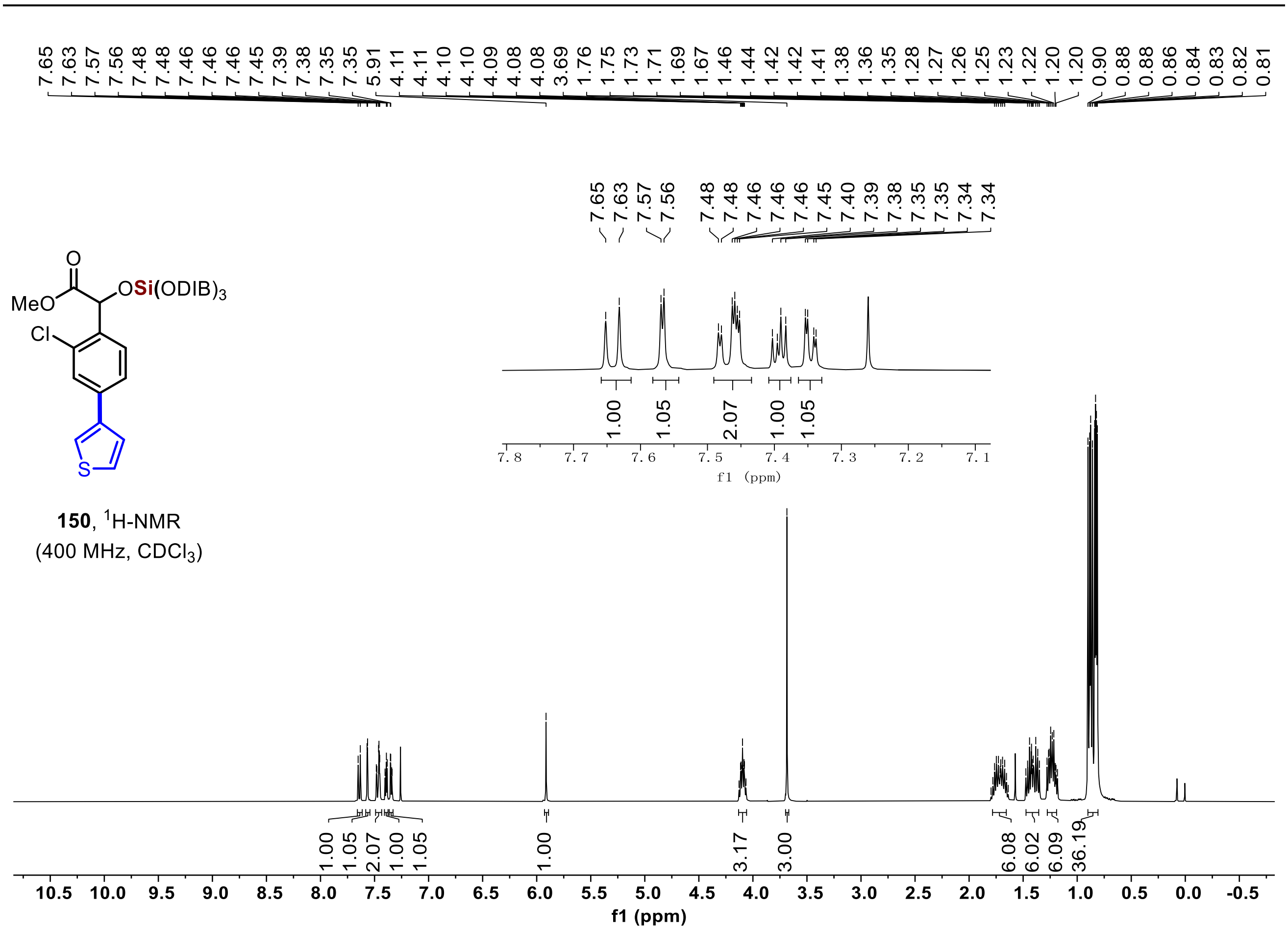
MeO
O
OSi(ODIB)3
Cl
S
150, 1H-NMR
(400 MHz, CDCl3)
f1 (ppm)

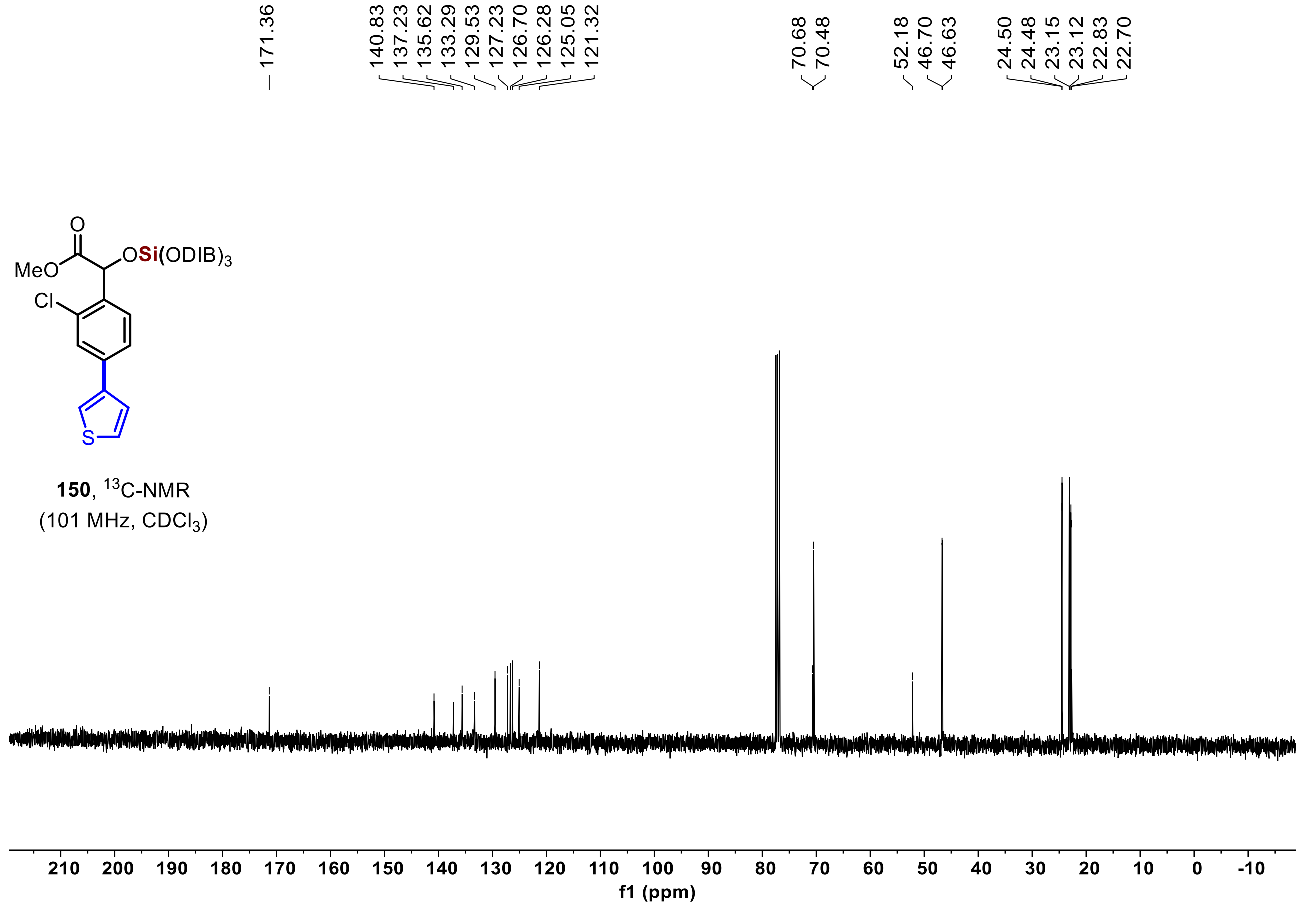
MeO
O
OSi(ODIB)3
Cl
S
150, 13C-NMR
(101 MHz, CDCl3)
171.36
140.83
137.23
135.62
133.29
129.53
127.23
126.70
126.28
125.05
121.32
70.68
70.48
52.18
46.70
46.63
24.50
24.48
23.15
23.12
22.83
22.70
f1 (ppm)

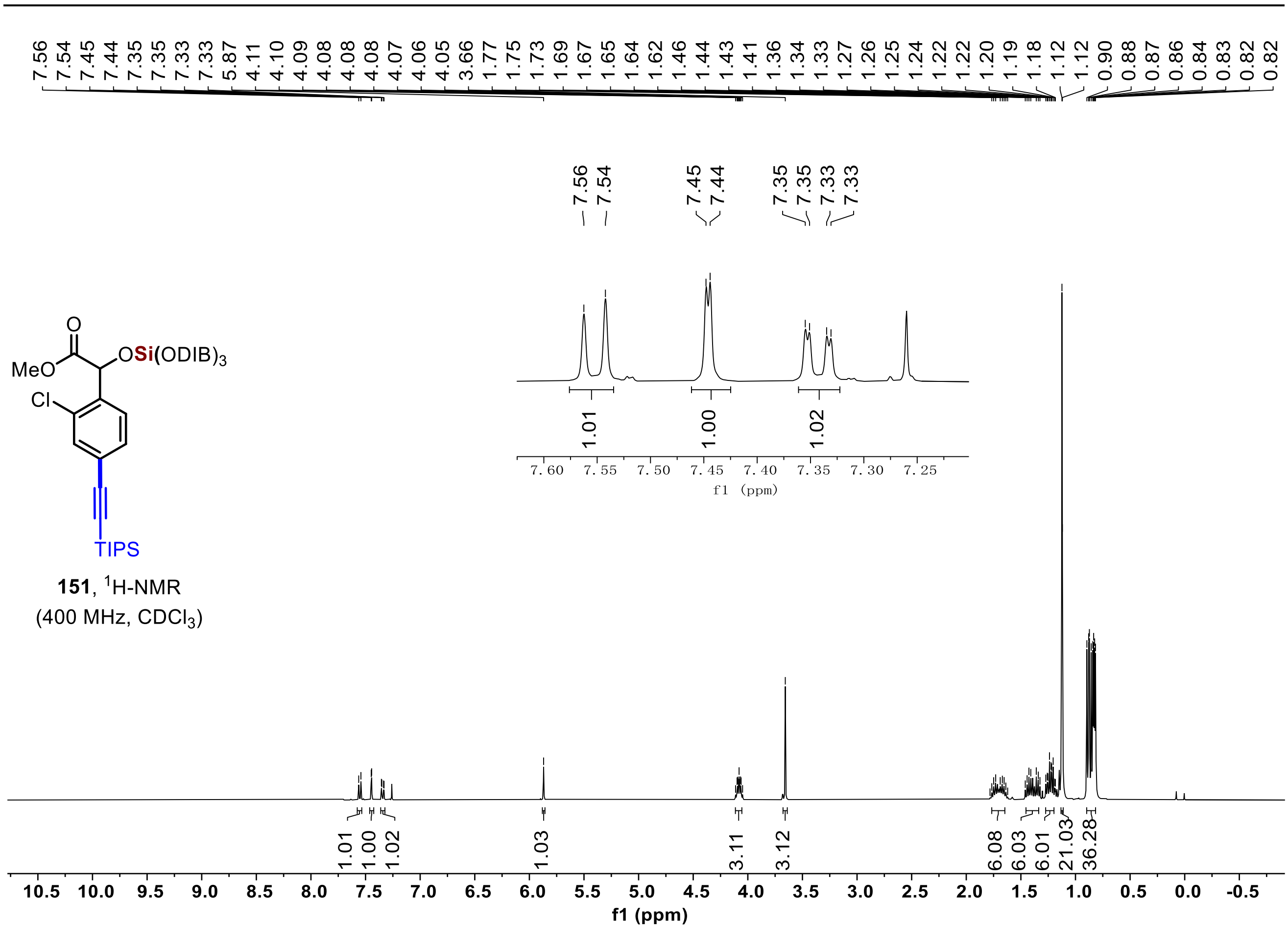
MeO
O
OSi(ODIB)3
Cl
TIPS
151, 1H-NMR
(400 MHz, CDCl3)
f1 (ppm)

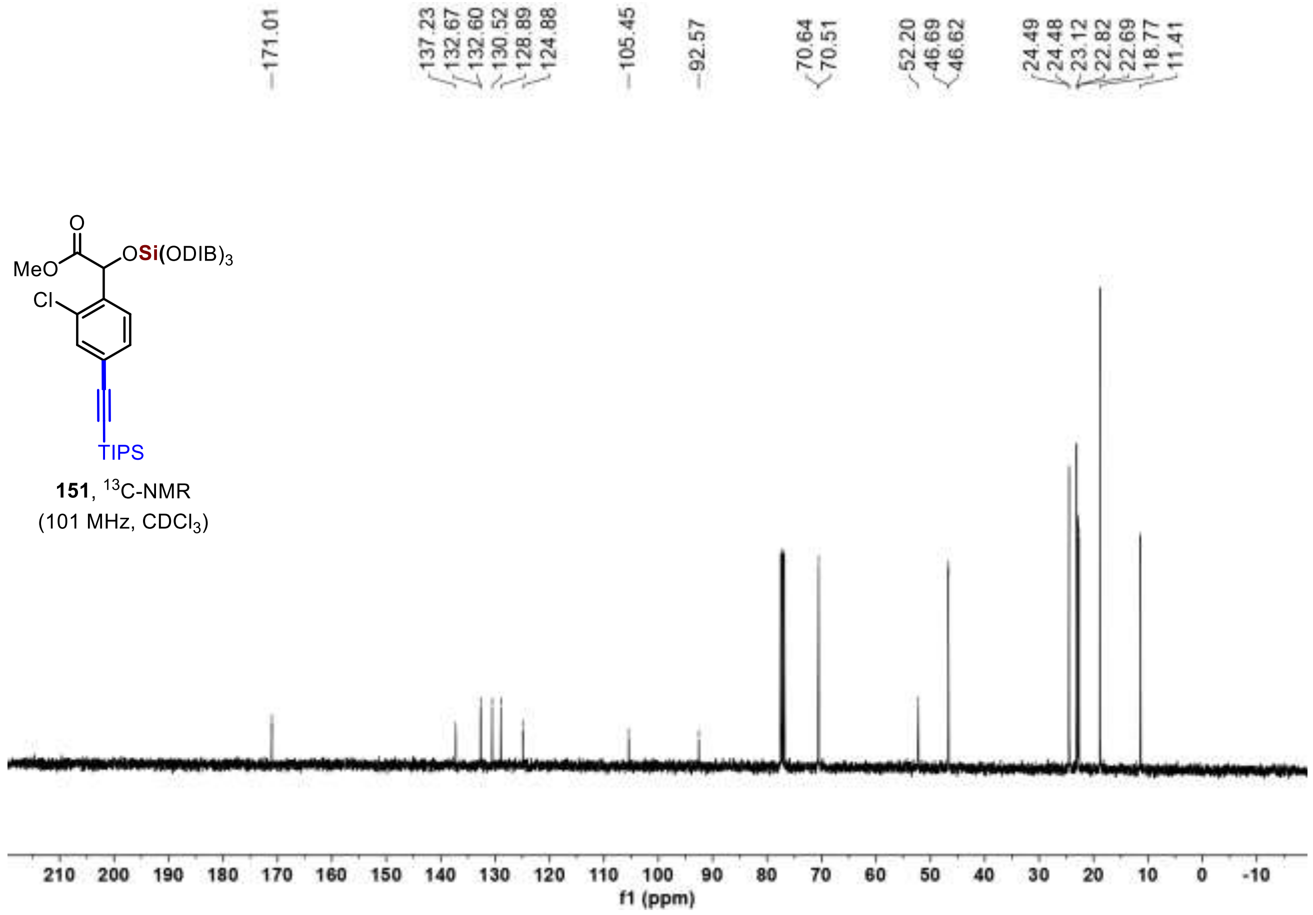
MeO
O
OSi(ODIB)3
Cl
TIPS
151, 13C-NMR
(101 MHz, CDCl3)
f1 (ppm)

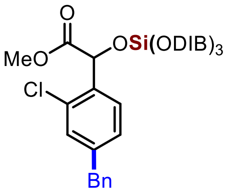

152, ^{1}H-NMR
(400 MHz, $CDCl_3$)

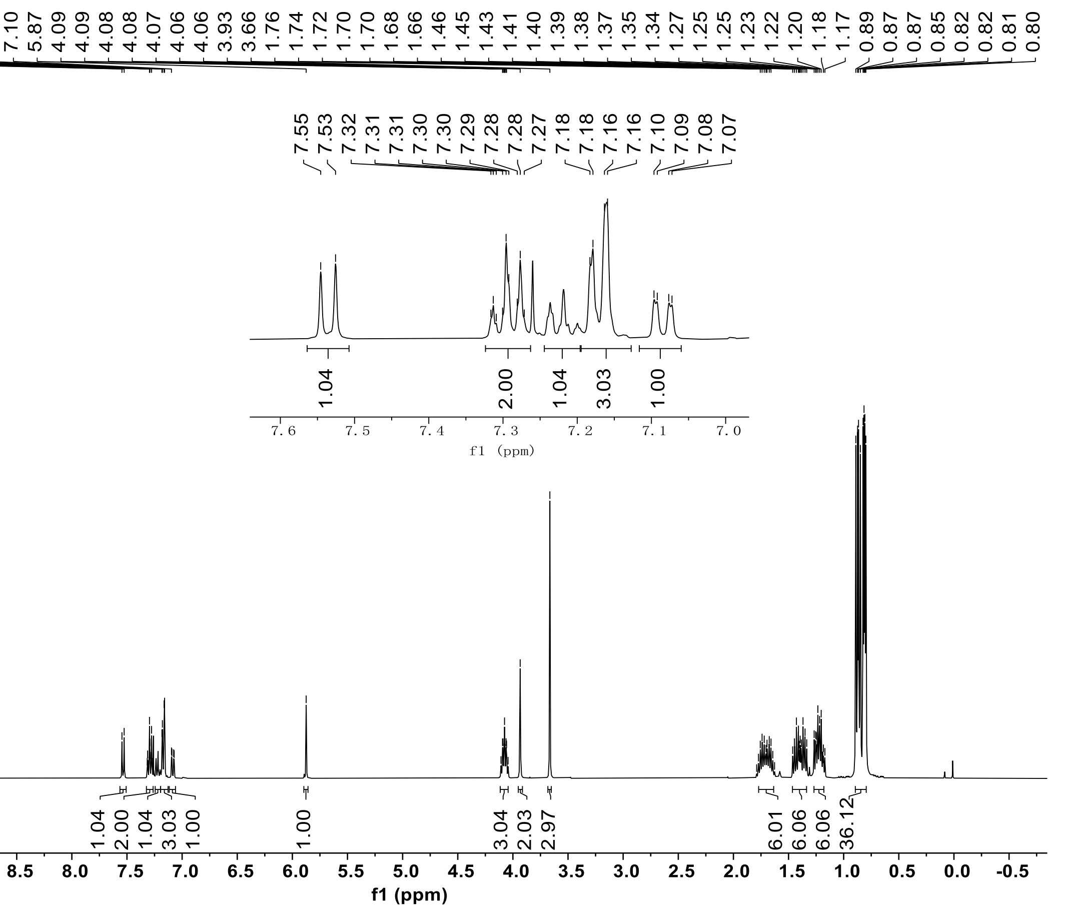

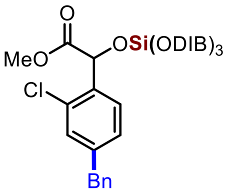

152, ^{13}C-NMR
(101 MHz, $CDCl_3$)

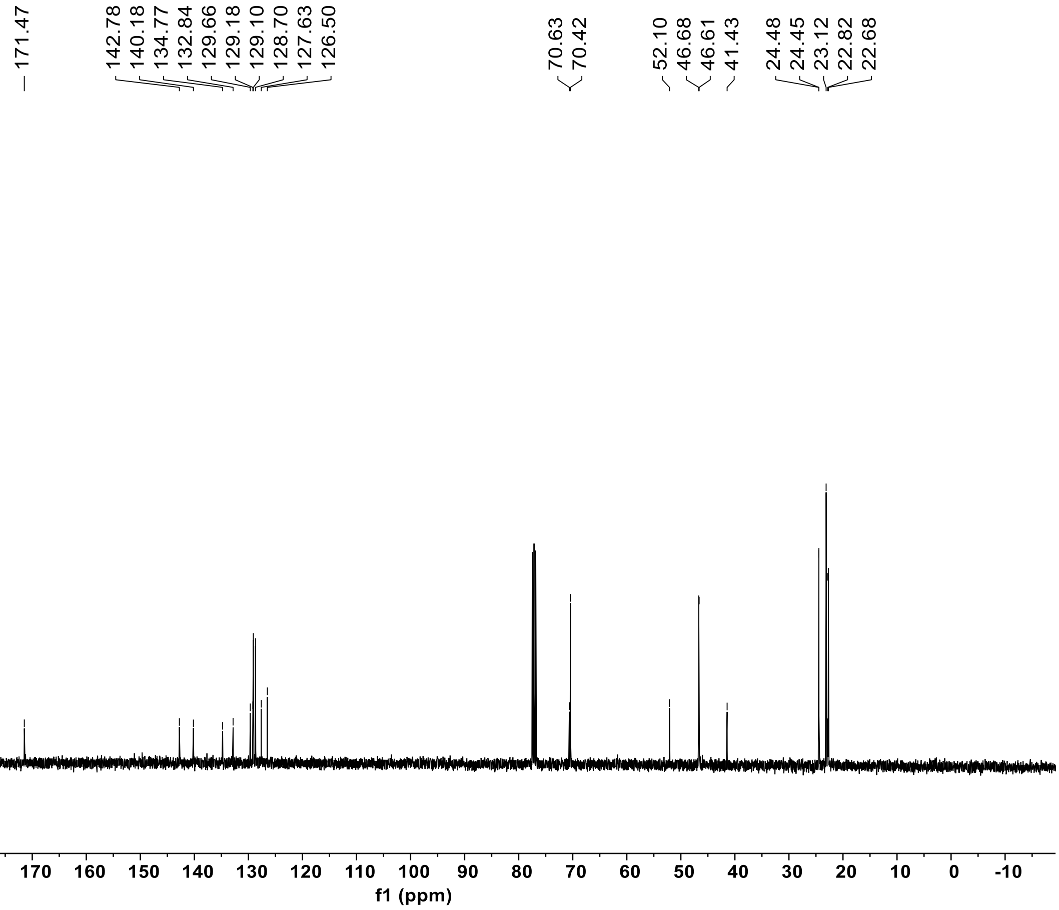

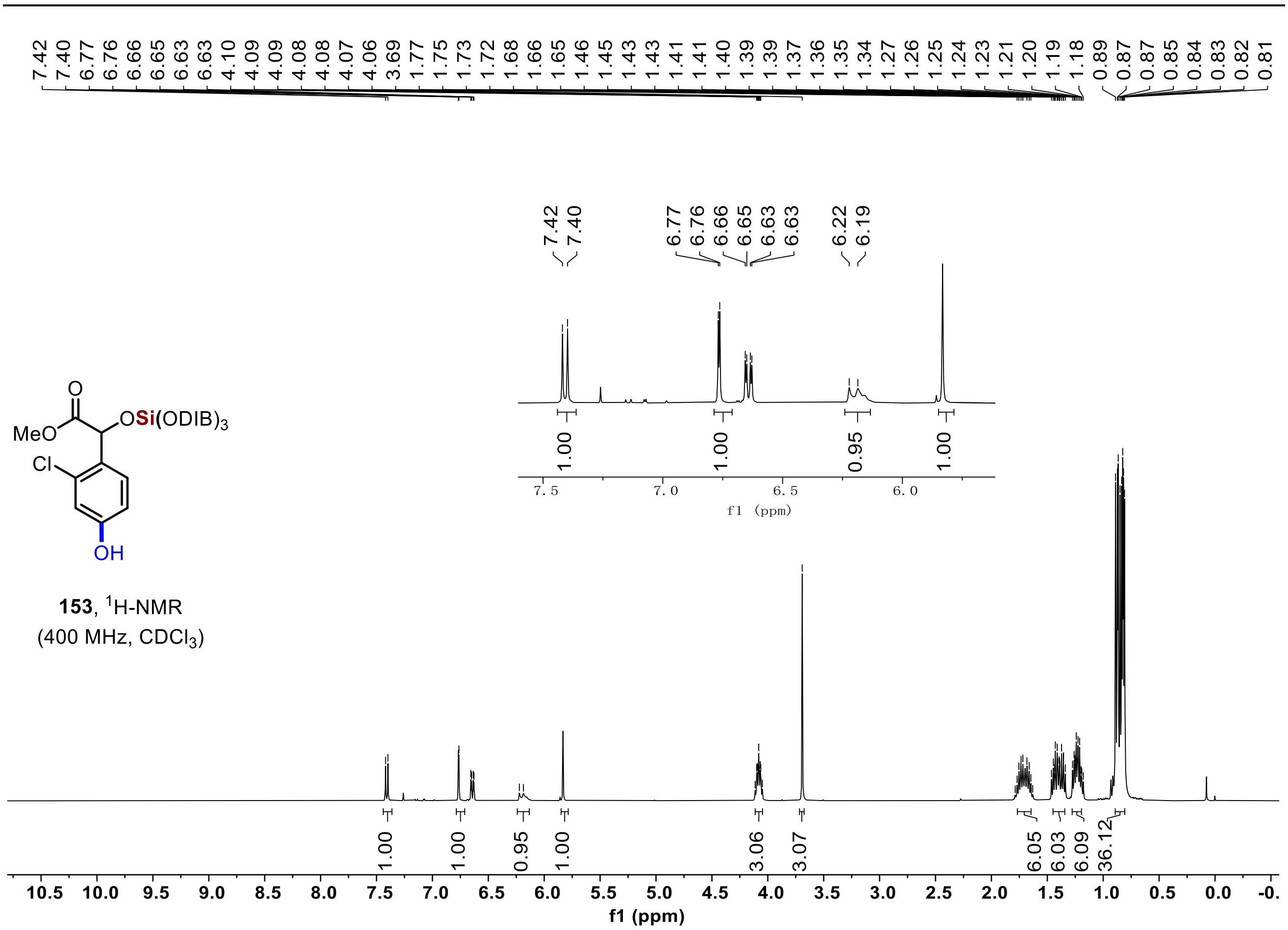

153, ^{1}H-NMR (400 MHz, $CDCl_3$)

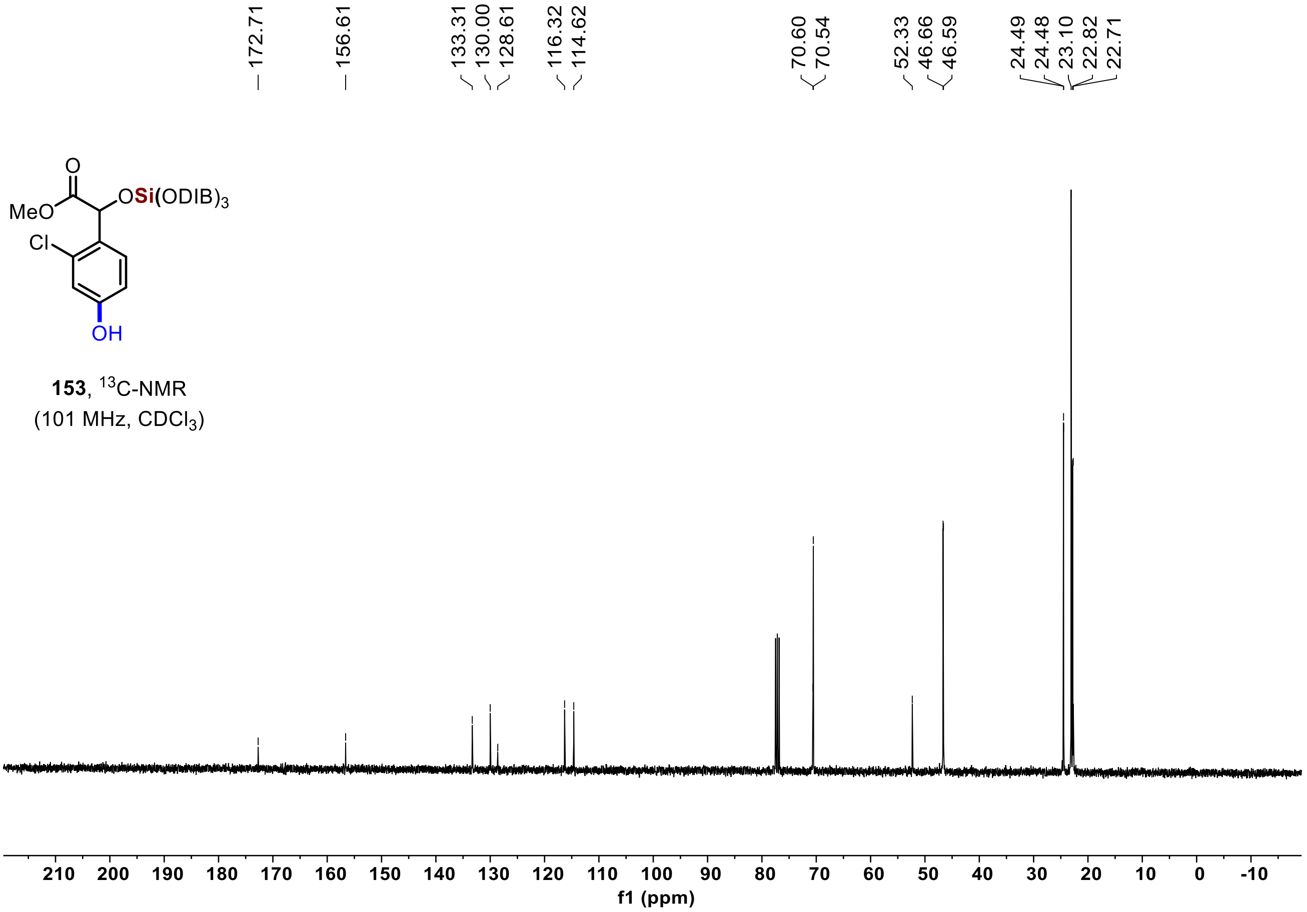

153, ^{13}C-NMR (101 MHz, $CDCl_3$)

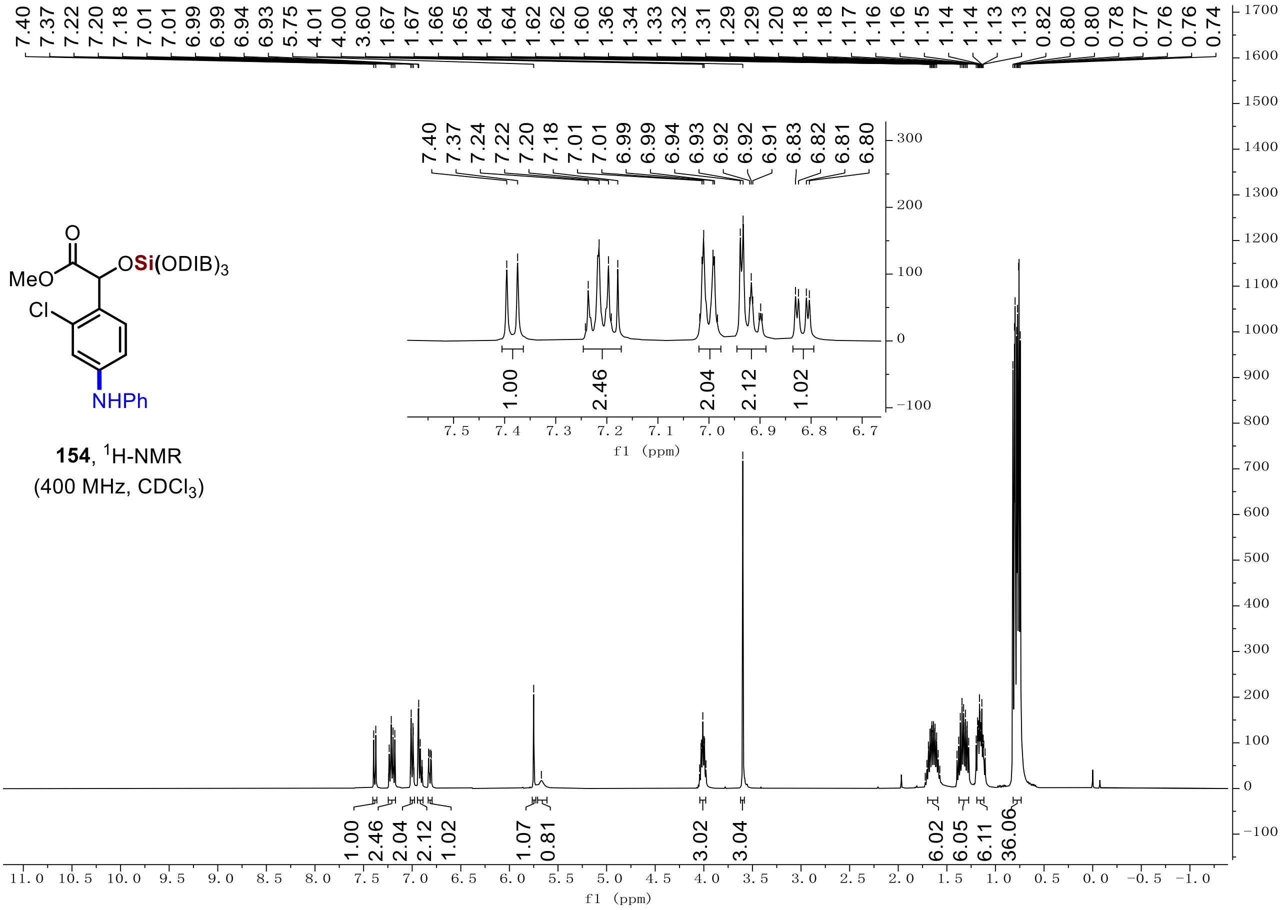
154, ^{1}H-NMR
(400 MHz, $CDCl_3$)
f1 (ppm)

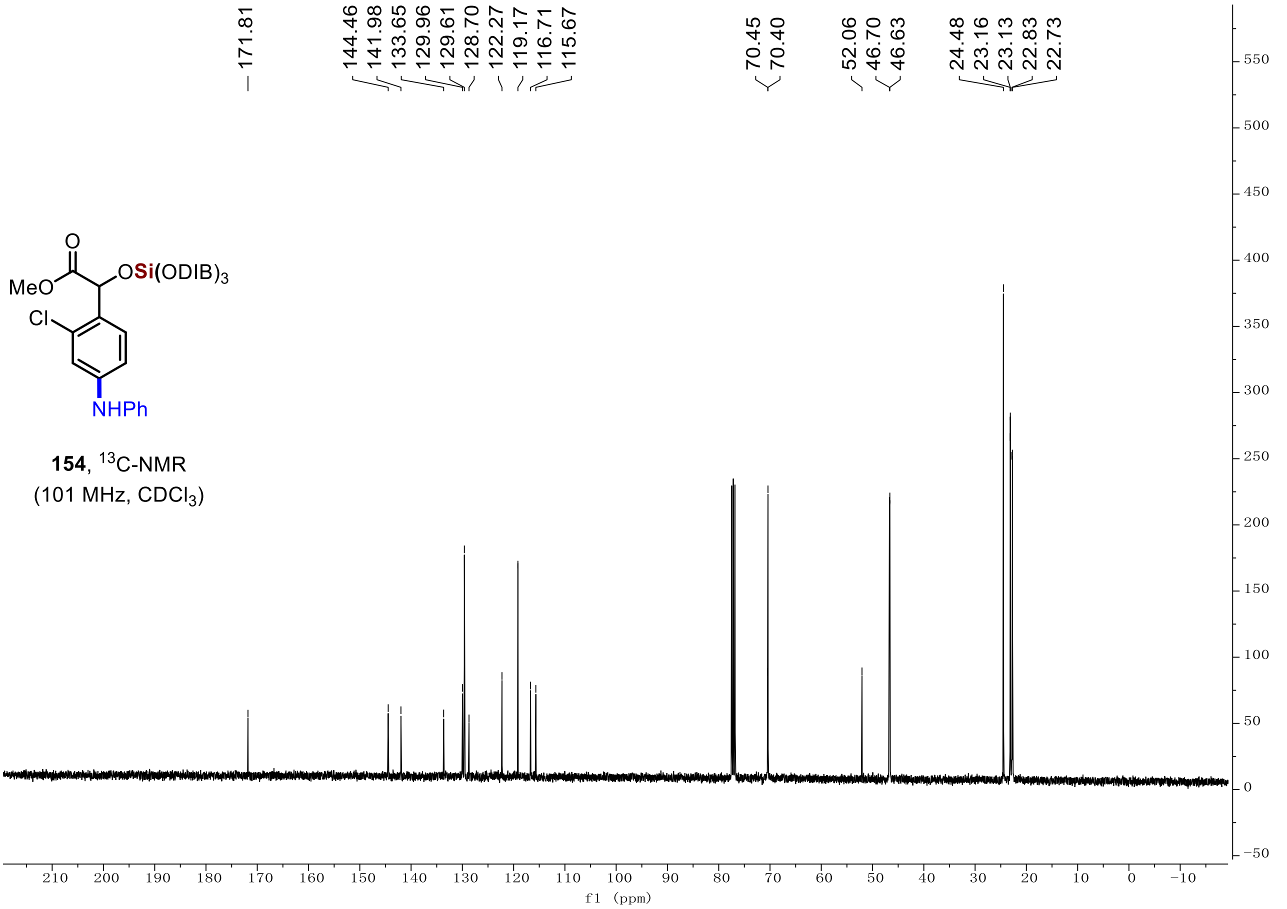
154, ^{13}C-NMR
(101 MHz, $CDCl_3$)
f1 (ppm)

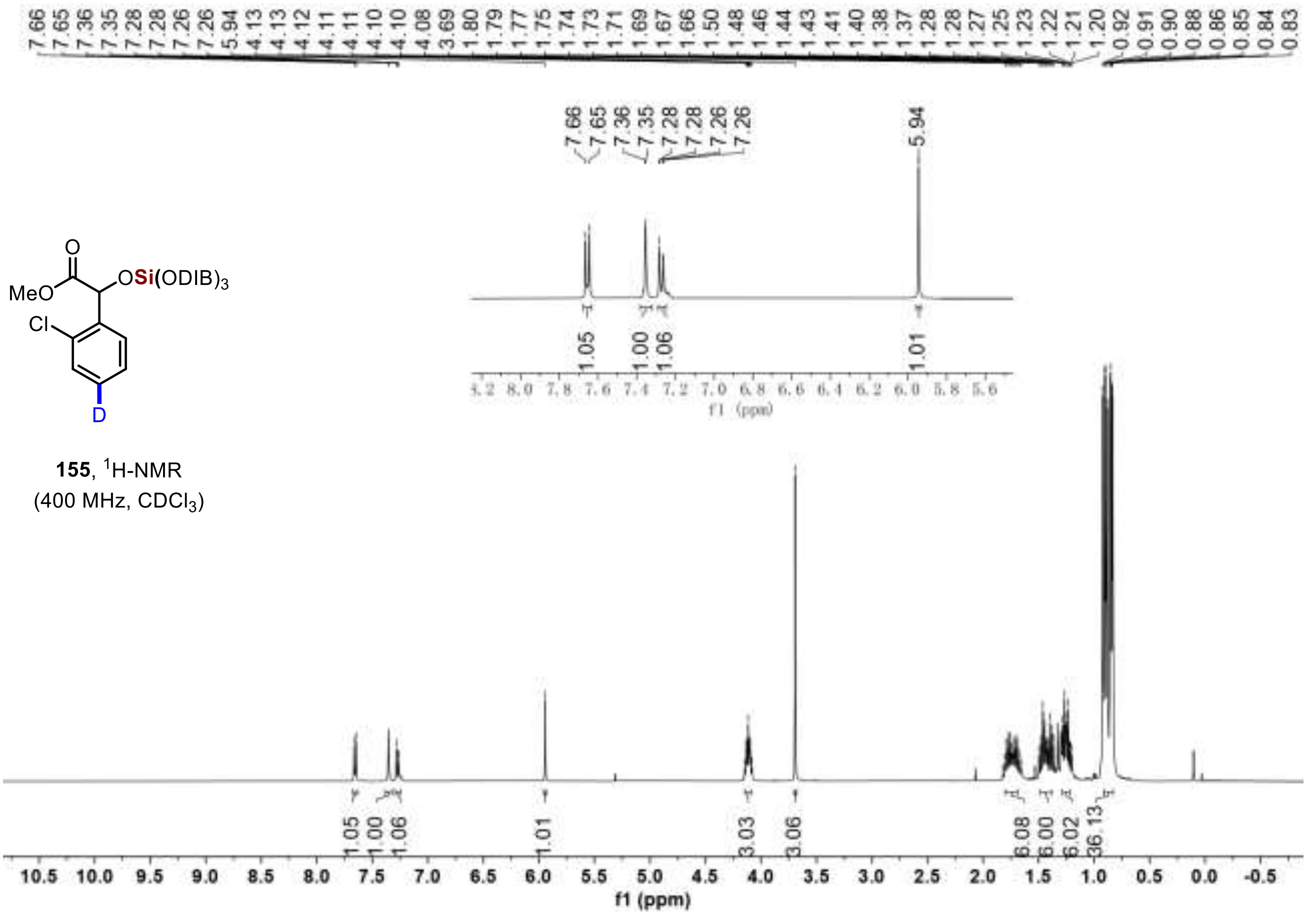

155, ^{1}H-NMR
(400 MHz, $CDCl_3$)

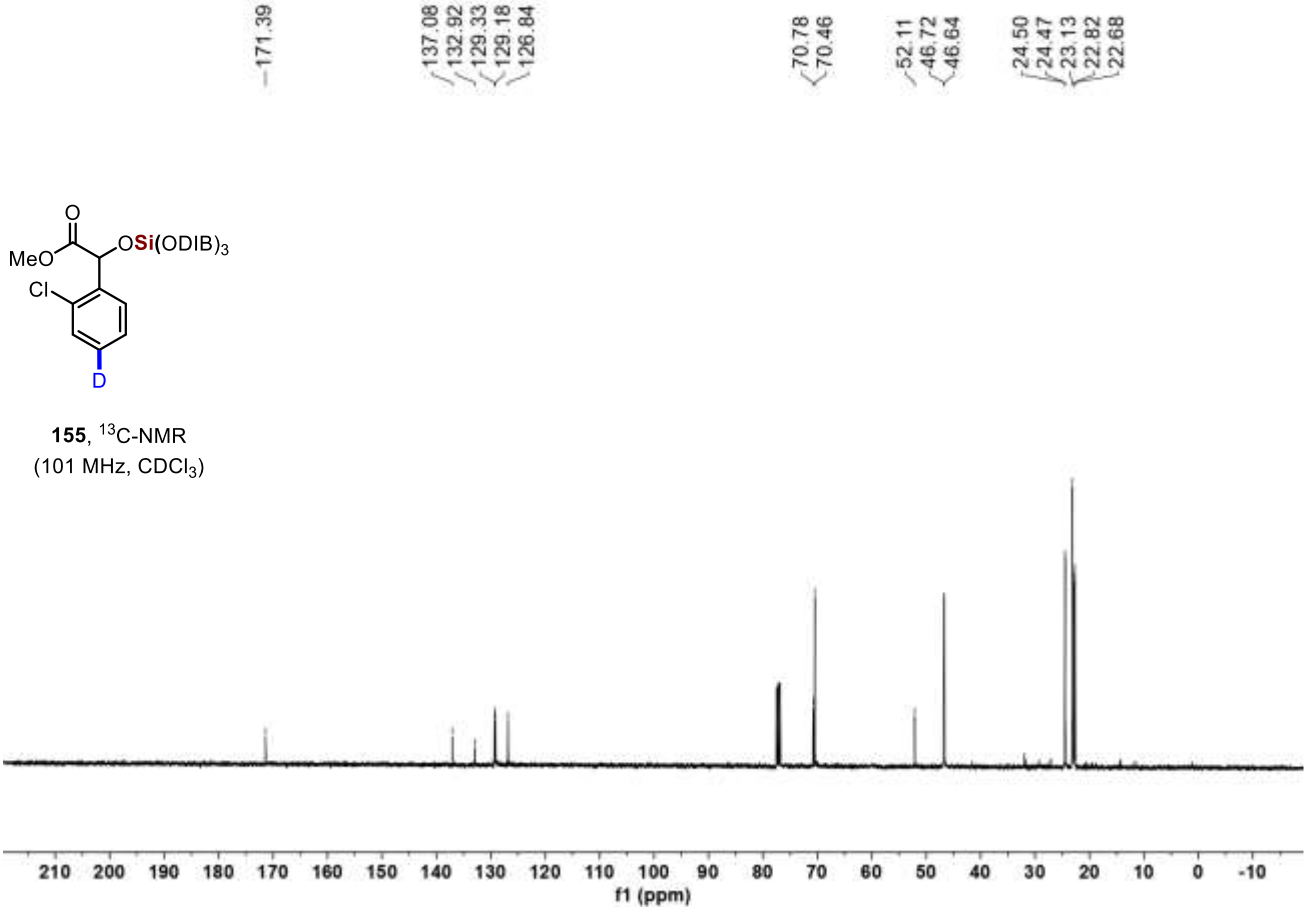

155, ^{13}C-NMR
(101 MHz, $CDCl_3$)

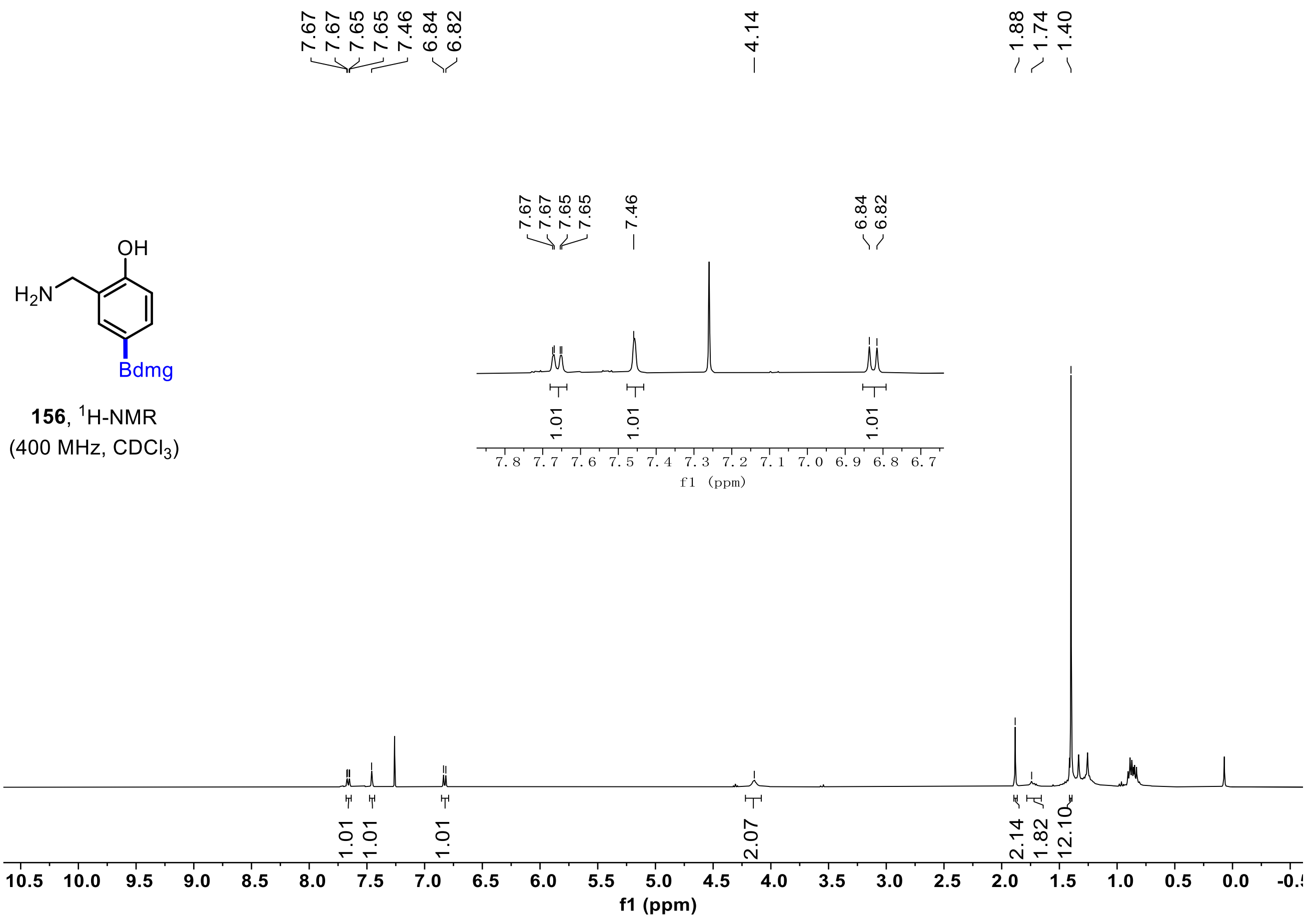
7.67 7.67 7.65 7.65 7.46 6.84 6.82
4.14
1.88 1.74 1.40
OH
H2N
Bdmg
156, 1H-NMR
(400 MHz, CDCl3)
1.01 1.01 1.01
f1 (ppm)
1.01 1.01 1.01 2.07 2.14 1.82 12.10
f1 (ppm)

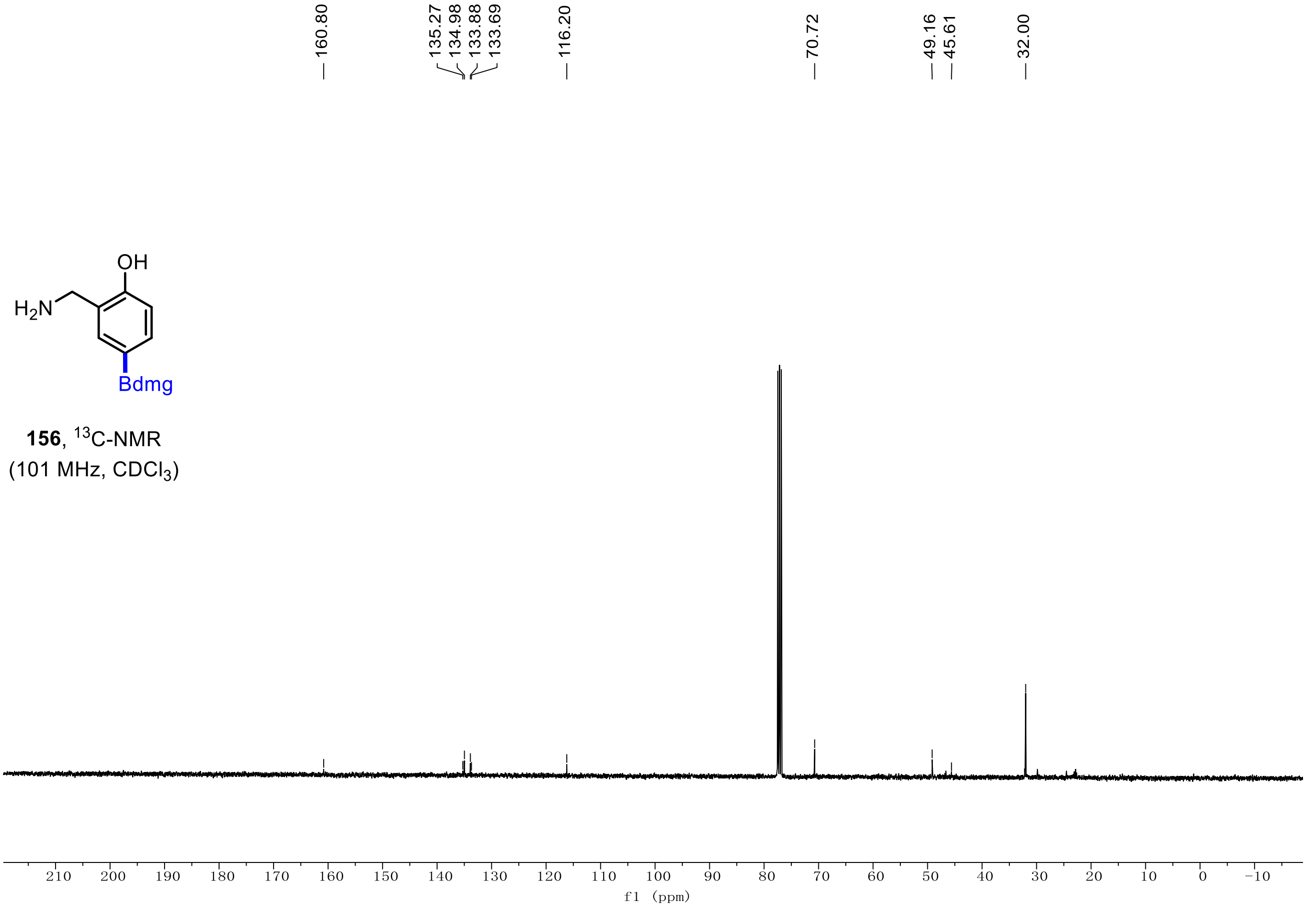
160.80
135.27 134.98 133.88 133.69
116.20
70.72
49.16 45.61
32.00
OH
H2N
Bdmg
156, 13C-NMR
(101 MHz, CDCl3)
f1 (ppm)

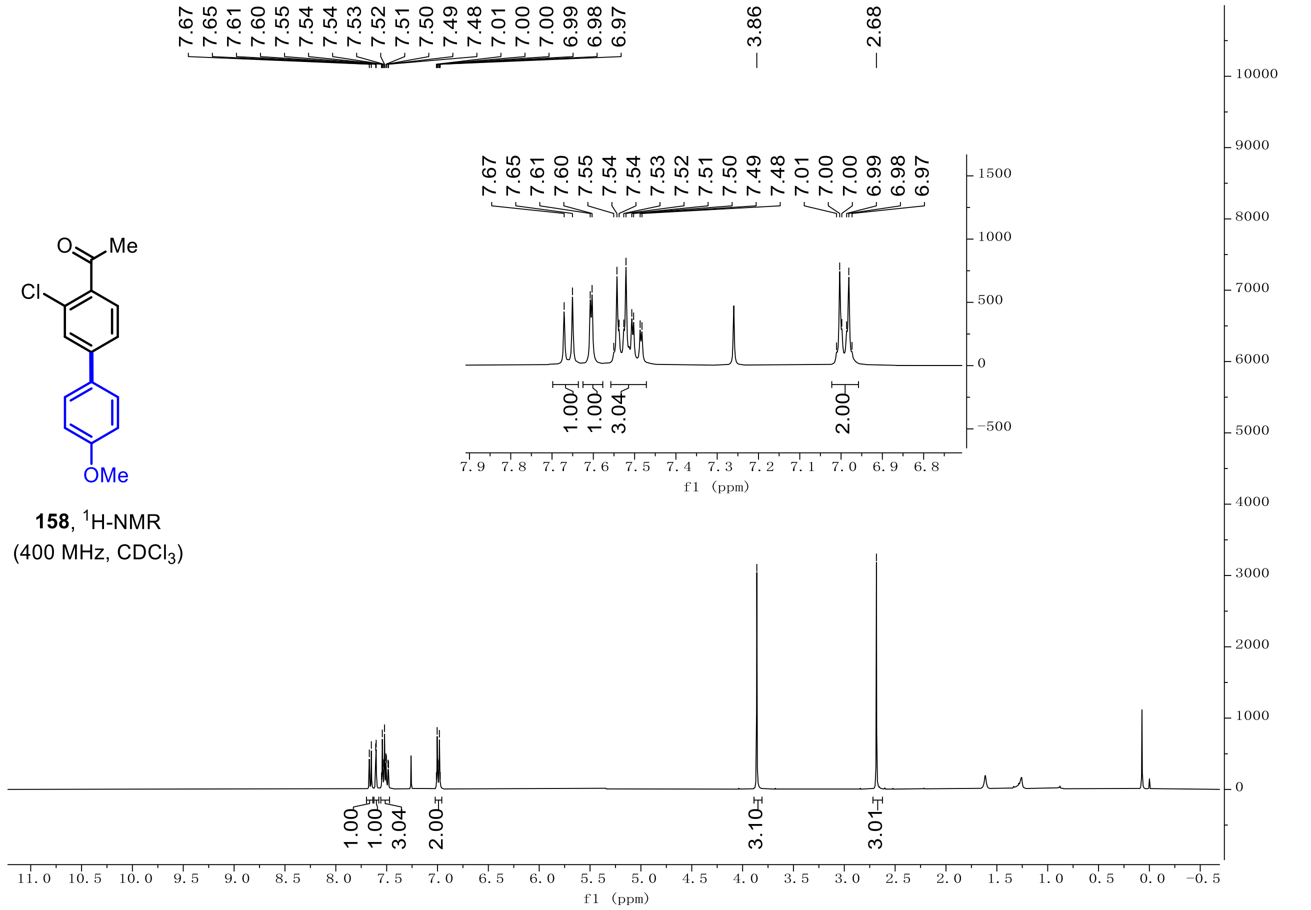

158, ^{1}H-NMR
(400 MHz, $CDCl_3$)

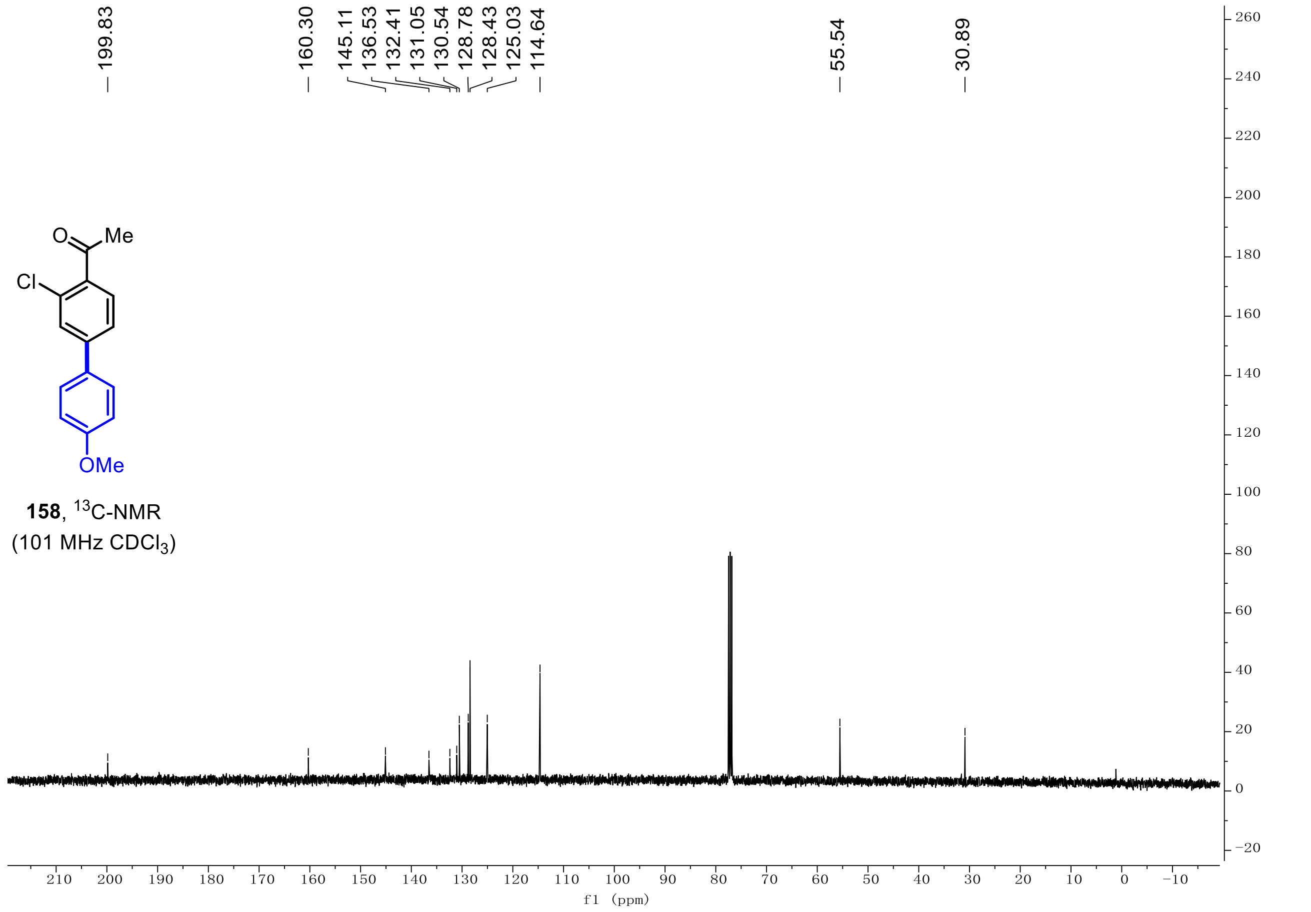

158, ^{13}C-NMR
(101 MHz $CDCl_3$)

11. Supplementary references

[1] (a) Kumar, P. & Kapur, M. Catalyst Control in Positional-Selective C–H Alkenylation of Isoxazoles and a Ruthenium-Mediated Assembly of Trisubstituted Pyrroles. *Org. Lett.* **21**, 2134-2138 (2019). (b) Li, S.; Shan, Q.-C.; Hu, L.-M.; Ma, X.- Q. & Hu, X.-H. Merging Alkenyl C–H Activation with the Ring-opening of 1,2- oxazetidines: Ruthenium-catalyzed Aminomethylation of Enamides. *Chem. Commun.* **56**, 7969-7972 (2020).

2. Mohr, J.; Porwal, D.; Chatterjee, I. & Oestreich, M. Extending the Scope of the B(C6F5)3-Catalyzed C-N Bond Reduction: Hydrogenation of Oxime Ethers and Hydrazones. *Chem. Eur. J.* **21**, 17583-17586 (2015).

3. McManus, J. B. & Nicewicz, D. A. Direct C–H Cyanation of Arenes via Organic Photoredox Catalysis. *J. Am. Chem. Soc.* **139**, 2880-2883 (2017).

4. Smith, A. B., III; Risatti, C. A.; Atasoylu, O.; Bennett, C. S.; Liu, J.; Cheng, H.; TenDyke, K.; Xu, Q., Design, Synthesis, and Biological Evaluation of Diminutive Forms of (+)-Spongistatin 1: Lessons Learned. *J. Am. Chem. Soc.* **133**, 14042-14053 (2011).

5. Kato, T. & Maruoka, K. Selective functionalization of benzylic C–H bonds of two different benzylic ethers by bowl-shaped N-hydroxyimide derivatives as efficient organoradical catalysts. *Chem. Commun* **58**, 1021-1024 (2022).

6. Zhang, Q.; Hitoshio, K.; Saito, H.; Shimokawa, J. & Yorimitsu, H. Copper- Catalyzed Electrophilic Amination of Alkoxyarylsilanes. *Eur. J. Org. Chem.* **2020**, 4018–4021 (2020).

7. Manoso, A. S.; Ahn, C.; Soheili, A.; Handy, C. J.; Correia, R.; Seganish, W. M. & DeShong, P. Improved Synthesis of Aryltrialkoxysilanes via Treatment of Aryl Grignard or Lithium Reagents with Tetraalkyl Orthosilicates. *J. Org. Chem.* **69**, 8305- 8314 (2004).

8. Lu, M.-Z.; Ding, X.; Shao, C.; Hu, Z.; Luo, H.; Zhi, S.; Hu, H.; Kan, Y. & Loh, T.- P. Direct Hiyama Cross-Coupling of (Hetero)arylsilanes with C(sp2)–H Bonds Enabled by Cobalt Catalysis. *Org. Lett.* **22**, 2663-2668 (2020).

9. Frohn, H. J.; Giesen, M.; Klose, A.; Lewin, A. & Bardin, V. V. A convenient preparation of pentafluorophenyl(fluoro)silanes: reactivity of pentafluorophenyltrifluorosilane. *J. Organomet. Chem.* **506**, 155-164 (1996).

10. Starikova, T. Y.; Surin, N. M.; Borshchev, O. V.; Pisarev, S. A.; Svidchenko, E. A.; Fedorov, Y. V. & Ponomarenko, S. A. A novel highly efficient nanostructured organosilicon luminophore with unusually fast photoluminescence. *J. Mater. Chem. C,* **4**, 4699-4708 (2016).

11. Cho, Y. S.; Kang, S.-H.; Han, J. S.; Yoo, B. R. & Jung, I. N. Novel Phosphonium Chloride-Catalyzed Dehydrohalogenative Si−C Coupling Reaction of Alkyl Halides with Trichlorosilane. *J. Am. Chem. Soc.* **123**, 5584-5585 (2001).

12. Chen, B.; Sun, H.-X.; Qin, J.-F. & Wang, B. DMF-mediated deprotection of bulky silyl esters under neutral and fluoride-free conditions. *Tetrahedron Lett.* **57**, 253-255 (2016).

13. Dong, X.; Kita, Y. & Oestreich, M. Kinetic Resolution of α-Hydroxy-Substituted Oxime Ethers by Enantioselective Cu−H-Catalyzed Si−O Coupling. *Angew. Chem. Int. Ed.* **57,** 10728-10731 (2018).

14. Ku, A. F. & Cuny, G. D. Synthetic Studies of 7-Oxygenated Aporphine Alkaloids: Preparation of (−)-Oliveroline, (−)-Nornuciferidine, and Derivatives. *Org. Lett.* **17**, 1134-1137 (2015).

15. Dong, X.; Kita, Y. & Oestreich, M. Kinetic Resolution of α-Hydroxy-Substituted Oxime Ethers by Enantioselective Cu−H-Catalyzed Si−O Coupling. *Angew. Chem. Int. Ed.* **57**, 10728-10731 (2018).

16. Yin, L.; Shan, W.; Jia, X.; Li, X. & Chan, A. S. C. Ru-catalyzed enantioselective preparation of methyl (R)-o-chloromandelate and its application in the synthesis of (S)-Clopidogrel. *J. Organomet. Chem.* **694***,* 2092-2095 (2009).

17. Suzuki, A. Cross-Coupling Reactions Of Organoboranes: An Easy Way To Construct C C Bonds (Nobel Lecture). *Angew. Chem. Int. Ed.* **50**, 6722-6737 (2011).

18. Cheng, C. & Hartwig, J. F., Rhodium-Catalyzed Intermolecular C–H Silylation of Arenes with High Steric Regiocontrol. *Science* **343**, 853-857 (2014).

19. Feng, X.; Pisula, W.; Zhi, L.; Takase, M. & Müllen, K. Controlling the Columnar Orientation of C3-Symmetric “Superbenzenes” through Alternating Polar/Apolar Substitutents. *Angew. Chem. Int. Ed.* **47**, 1703-1706 (2008).

20. Traficante, C. I.; Mata, E. G. & Delpiccolo, C. M. L. Very efficient and broad-inscope palladium-catalyzed Hiyama cross-coupling. The role of water and copper(i) salts. *RSC Adv.* **5**, 26796-26800 (2015).

21. Lam, P. Y. S.; Deudon, S.; Averill, K. M.; Li, R.; He, M. Y.; DeShong, P. & Clark, C. G. Copper-Promoted C−N Bond Cross-Coupling with Hypervalent Aryl Siloxanes and Room-Temperature N-Arylation with Aryl Iodide. *J. Am. Chem. Soc.* **122**, 7600- 7601 (2000).

22. Correia, R. & DeShong, P. Palladium-Catalyzed Arylation of Allylic Benzoates Using Hypervalent Siloxane Derivatives. *J. Org. Chem.* **66**, 7159-7165 (2001).

23. Koike, T.; Du, X.; Sanada, T.; Danda, Y. & Mori, A. Iridium-Catalyzed Mizoroki–Heck-Type Reaction of Organosilicon Reagents. *Angew. Chem. Int. Ed.* **42**, 89-92 (2003).

24. Tang, P. & Ritter, T., Silver-mediated fluorination of aryl silanes. *Tetrahedron* **67**, 4449-4454 (2011).

25. Suzuki, A. Cross-Coupling Reactions Of Organoboranes: An Easy Way To Construct C-C Bonds (Nobel Lecture). *Angew. Chem. Int. Ed.* **50**, 6722-6737 (2011).

26. Wang, Y.; Chang, W.; Qin, S.; Ang, H.; Ma, J.; Lu, S. & Liang, Y. Diversification of Aryl Sulfonyl Compounds through Ligand-Controlled meta- and para-C−H Borylation. *Angew. Chem. Int. Ed.* **61**, e202206797 (2022).

27. Ryota, O.; Kenji, Y. & Kenji, K. 2,7-Diborylanthracene as a Useful Building Block for Extended π-Conjugated Aromatics. *Chem. Lett.* **40**, 941-943 (2011).

28. Chaturvedi, J.; Haldar, C.; Bisht, R.; Pandey, G. & Chattopadhyay, B. Meta Selective C–H Borylation of Sterically Biased and Unbiased Substrates Directed by Electrostatic Interaction. *J. Am. Chem. Soc.* **143**, 7604-7611 (2021).

29. Cai, Y.; Yang, X.-T.; Zhang, S.-Q.; Li, F.; Li, Y.-Q.; Ruan, L.-X.; Hong, X. & Shi, S.-L. Copper-Catalyzed Enantioselective Markovnikov Protoboration of α-Olefins Enabled by a Buttressed N-Heterocyclic Carbene Ligand. *Angew. Chem. Int. Ed.* **57**, 1376-1380 (2018).

30. Vantourout, J. C.; Miras, H. N.; Isidro-Llobet, A.; Sproules, S. & Watson, A. J. B. Spectroscopic Studies of the Chan – Lam Amination: A Mechanism-Inspired Solution to Boronic Ester Reactivity. *J. Am. Chem. Soc.* **139**, 4769-4779 (2017).

31. Bisht, R.; Hoque, M. E. & Chattopadhyay, B. Amide Effects in C−H Activation: Noncovalent Interactions with L-Shaped Ligand for meta Borylation of Aromatic Amides. *Angew. Chem. Int. Ed.* **57**, 15762-15766 (2018).

32. Chang, W.; Chen, Y.; Lu, S.; Jiao, H.; Wang, Y.; Zheng, T.; Shi, Z.; Han, Y.; Lu, Y.; Wang, Y.; Pan, Y.; Yu, J.-Q.; Houk, K. N.; Liu, F. & Liang, Y. Computationally designed ligands enable tunable borylation of remote C – H bonds in arenes. *Chem* **8**, 1775-1788 (2022).

33. (a) Neese, F. The ORCA program system. *Wiley Interdiscip. Rev. Comput. Mol. Sci.*

2012, 2, 73. (b) Neese, F. Software update: the ORCA program system, version 4.0. *Wiley Interdiscip. Rev. Comput. Mol. Sci.* **8** (2018). (c) Neese, F. Software update: The ORCA program system Version 5.0. *WIREs Comput. Mol. Sci.* **2022**, e1606.

34. (a) Stephens, P. J.; Devlin,F. J.;Chabalowski,C. F.;Frisch,M.J. Ab Initio Calculation of Vibrational Absorptionand Circular Dichroism Spectra Using Density Functional Force Fields. *J. Phys. Chem.* **1994**, *98*, 11623. (b) Becke, A. D. A new mixing of Hartree−Fockand local density-functional theories. *J. Chem. Phys.* **1993**, *98*, 1372. (c) Becke, A.D. Density-functional thermochemistry. III. The role of exact exchange. *J. Chem. Phys.* **1993**, *98*, 5648.

35. (a) Grimme, S.; Antony, J.; Ehrlich, S.; Krieg, H. A consistent and accurate ab initio parametrization of density functional dispersion correction (DFT-D) for the 94 elements H-Pu. *J. Chem. Phys.* **132**, 154104 (2010). (b) Grimme, S.; Ehrlich, S.; Goerigk, L. Effect of the Damping Function in Dispersion Corrected Density Functional Theory. *J. Comput. Chem.* **32**, 1456 (2011).

36. Weigend, F.; Ahlrichs, R. Balanced basis sets of split valence, triple zeta valence and quadruple zeta valence quality for H to Rn: Design and assessment of accuracy. *Phys. Chem. Chem. Phys.* **7**, 3297 (2005).

37. Neese, F.; Wennmohs, F.; Hansen, A.; Becker, U. Efficient, Approximate and Parallel Hartree–Fock and Hybrid DFT Calculations. A 'Chain-of-Spheres' Algorithm for the Hartree–Fock Exchange. *Chem. Phys.* **356**, 98 (2009).

38. (a) Weigend, F. Accurate Coulomb-fitting basis sets for H to Rn. *Phys. Chem. Chem. Phys.* **8**, 1057 (2006). (b) Hellweg, A.; Hattig, C.; Hofener, S.; Klopper, W. Optimized accurate auxiliary basis sets for RI-MP2 and RI-CC2 calculations for the atoms Rb to Rn. *Theor. Chem. Acc.* **117**, 587 (2007).

39. CYLview20; Legault, C. Y. Université de Sherbrooke, 2020 (http://www.cylview.org)

40. Falivene, L.; Cao, Z.; Petta, A.; Serra, L.; Poater, A.; Oliva, R.; Scarano, V.; Cavallo, L. Towards the online computer-aided design of catalytic pockets. *Nat. Chem.* **11**, 872 (2019).
